# Use of Laplacian Heat Diffusion Algorithm to Infer Novel Genes With Functions Related to Uveitis

**DOI:** 10.3389/fgene.2018.00425

**Published:** 2018-10-08

**Authors:** Shiheng Lu, Ke Zhao, Xuefei Wang, Hui Liu, Xiamuxiya Ainiwaer, Yan Xu, Min Ye

**Affiliations:** ^1^Department of Ophthalmology, Shanghai Pudong Hospital, Fudan University Pudong Medical Center, Pudong, China; ^2^School of Life Sciences, Shanghai University, Shanghai, China

**Keywords:** uveitis, Laplacian heat diffusion, protein–protein interaction, disease gene, network construction

## Abstract

Uveitis is the inflammation of the uvea and is a serious eye disease that can cause blindness for middle-aged and young people. However, the pathogenesis of this disease has not been fully uncovered and thus renders difficulties in designing effective treatments. Completely identifying the genes related to this disease can help improve and accelerate the comprehension of uveitis. In this study, a new computational method was developed to infer potential related genes based on validated ones. We employed a large protein–protein interaction network reported in STRING, in which Laplacian heat diffusion algorithm was applied using validated genes as seed nodes. Except for the validated ones, all genes in the network were filtered by three tests, namely, permutation, association, and function tests, which evaluated the genes based on their specialties and associations to uveitis. Results indicated that 59 inferred genes were accessed, several of which were confirmed to be highly related to uveitis by literature review. In addition, the inferred genes were compared with those reported in a previous study, indicating that our reported genes are necessary supplements.

## Introduction

Uvea is a specific structure in the eyes and consists of the pigmented layer and the outer fibrous layer (Junqueira et al., 2013; Rekas et al., 2015). As one of the most common types of inflammation in uvea, uveitis is the third leading cause of blindness in all developed countries, generally affecting people aging 20–50 years (Junqueira et al., 2013). According to the statistics from the National Eye Institute in the United States, uveitis can be subtyped as anterior uveitis, intermediate uveitis, posterior uveitis, and panuveitis uveitis based on different pathogenic progressions and sites (Lim et al., 2016). Regardless of its subtype, the major pathogenesis of this disease is the over-activation of inflammatory cells *in situ* (Pan et al., 2014; Rosenbaum, 2015). Uvea contains most of the eye’s blood vessels, from which immune cells can enter the eye. Therefore, uvea gets inflamed easier than other eye tissue regions, revealing the histological causes of uveitis’ high morbidity rate.

The following five major clinical symptoms occur during the initiation and progression of uveitis: painful eye(s), bloodshot eye(s), sensitivity to light, and cloudy vision and floaters (Urban et al., 2014). In the early stage of such disease, the patient’s eye(s) can only manifest as redness and conjunctivitis with no visual defeats. With the quick progression of such disease, blindness and the five common complications, including glaucoma, cataracts, optic nerve damage, retinal detachment, and permanent vision loss, are usually identified in the patient population (Sen et al., 2015). For the detailed pathogenic cause of such disease, the specific cause of uveitis in most clinical cases cannot be clearly identified due to its complicated candidate pathogenesis. In general, the top five causes of uveitis at the histological and pathogenic level can be clustered into five subgroups as follows: (1) eye injury, surgery; (2) autoimmune disorder; (3) inflammatory disorder; (4) eye tissue specific infection; and (5) cancer (Kalinina Ayuso et al., 2014). All such causes can be summarized as exogenous environmental effects and endogenous genetic contributions. According to recent publications, genetic and infectious contributions have received increasing attention and are widely regarded as the top two major pathogenic factors. Infectious progressions and their related immune responses of various infections, including brucellosis (Akyol et al., 2015), herpesviruses (Bojanova et al., 2013), and leptospirosis (Loureiro et al., 2013), contribute to the progression of uveitis, thus reflecting the unique pathogenic contribution of exogenous factors for uveitis.

Apart from exogenous factors such as infections, genetic contributions are also a major pathogenesis of uveitis. Early in 2014, a functional gene named FOXO1 has been confirmed to participate in the pathogenesis of acute anterior uveitis, thus reflecting the specific endogenous role of such gene for uveitis (Yu et al., 2014). In 2015, another study on acute anterior uveitis confirmed that a specific immune associated gene, which is named C5 and encodes complement C5, contributes to its immune associated pathogenesis, thereby reflecting the complicated pathogenesis of such disease (Xu et al., 2015). Other complement associated genes and interleukin related genes have also been identified in different subtypes of uveitis, confirming the pathogenic genetic contribution of such disease (Yang et al., 2012). In 2015, a specific clinical trial (Ildefonso et al., 2015) on the gene therapy on uveitis revealed that the modification and recruitment of specific protein domains encoded by functional genes can reduce the ocular inflammatory response and relieve the symptoms. This finding indicated that genetic contributions may at least be one of the major pathogenic factors of uveitis.

Identifying the core pathogenic genetic factors and revealing the detailed pathogenic mechanisms based on experimental routines are difficult because of the organizational specificity (eye), relatively low incidence, and complicated pathogenesis of uveitis. In recent years, more and more computational methods (Tang et al., 2017; Zeng et al., 2017, 2018; Chen et al., 2018b; Pan et al., 2018; Wang et al., 2018) have been designed to investigate different diseases, thereby giving help to uncover pathogenic mechanisms of diseases. For uveitis, in 2017, uveitis-related genes were identified based on a computational method (Lu et al., 2017), which adopted the classic network algorithm named random walk with restart (RWR) algorithm (Kohler et al., 2008; Li and Li, 2012) to search novel genes in a protein–protein interaction (PPI) network. In the present study, we employed another network algorithm named Laplacian heat diffusion (LHD) to build a new computational method for inferring novel uveitis-related genes. LHD algorithm has different principles compared with RWR algorithm and thus may help us extract novel genes that cannot be identified by the method in Lu et al. (2017). In addition, the proposed method also adopted several tests to screen out the most related genes. Finally, 59 genes were accessed by our method, and only two of them were also reported in previous studies as validated pathogens (Lu et al., 2017). We conducted an extensive analysis on several of these genes to show the reliability of our method. The new findings reported in the present study may aid in revealing the detailed pathogenic mechanisms of uveitis.

## Materials and Methods

### Uveitis-Related Genes

We extracted uveitis related genes from literature indexed by PubMed^1^. In the search bar, we set “uveitis” and “genes” as the keywords, thus obtaining 744 published articles. Among these articles, 98 were review articles, in which several solid uveitis related genes were reported. A total of 121 genes were selected by manual reviewing. These genes are important for uveitis or specific uveitis symptoms and thus were called uveitis-related genes in this study. Proteins encoded by the 121 uveitis-related genes were obtained and further mapped onto their Ensembl IDs because we adopted the PPI network to infer novel uveitis related genes based on these genes. Finally, 113 Ensembl IDs were obtained. The 121 uveitis genes and the Ensembl IDs of their proteins are provided in **Supplementary Table S1**.

### Construction of PPI Network

PPI information is a useful material to study different protein- or gene-related problems (Hu et al., 2011a,b; Gao P. et al., 2012; Gao Y.F. et al., 2012; Chen et al., 2016a, 2018c; Huang et al., 2016; Zhang et al., 2016; Cai et al., 2017; Lu et al., 2017; Li et al., 2018a). Most studies using this information reported that two proteins that interact with each other always share similar functions. The proteins encoded by uveitis-related genes may have some common functions, which may also be shared by their interactive proteins. This procedure can further continue. If we start from the proteins encoded by uveitis-related genes and diffuse their status to their neighbors and neighbors’ neighbors, then some novel proteins that are strongly associated with proteins encoded by uveitis-related genes can be extracted. Their genes may be novel uveitis-related genes. We need a PPI network to complete these procedures. Here, we used the PPI network reported in STRING^2^ (version 10.0). Compared with PPI networks reported in other databases, such as DIP (Database of Interaction Proteins) database (Xenarios et al., 2000) and BioGRID (Stark et al., 2006), which are constructed using experimentally determined PPIs, the PPI network used in this study further contains functional associations between proteins. The PPIs in STRING were collected from the following sources: (1) genomic context predictions; (2) high-throughput lab experiments; (3) (conserved) co-expression; (4) automated text mining; and (5) previous knowledge in databases. Thus, they can widely measure the associations between proteins, providing more opportunities to infer novel uveitis-related genes. The file “9606.protein.links.v10.txt.gz” was retrieved from STRING to construct this PPI network. In this file, large numbers of human PPIs were included. Each PPI was assigned a score to indicate its strength. The constructed PPI network termed proteins, represented by Ensembl IDs, as nodes. Two nodes were adjacent if and only if their corresponding proteins can interact with each other. Furthermore, each edge in the PPI network was assigned a weight, which was defined as the score of its corresponding PPI. For easy description, the constructed PPI network was called as *N* in the following text.

### Method for Inferring Novel Uveitis-Related Genes

Inferring novel genes related to different diseases in network level has become quite popular (Barabasi et al., 2011). Several classic network algorithms, such as shortest path algorithm (Gormen et al., 1990; Gui et al., 2015; Chen et al., 2016a; Zhang et al., 2016; Cai et al., 2017, Chen et al., 2018a), and RWR algorithm (Chen et al., 2017a, 2018c; Li et al., 2017, 2018a; Yuan and Lu, 2017; Zhang et al., 2017), have been applied to develop different computational methods in this regard. A recent publication (Lu et al., 2017) proposed a RWR-based computational method to identify novel uveitis-related genes and reported several ones. Another classic network algorithm, LHD algorithm (Leiserson et al., 2015), was employed to construct a novel computational method to infer novel uveitis-related genes that were not reported in Lu et al. (2017).

#### LHD Algorithm

As a classic network diffusion algorithm, heat diffusion algorithm always starts from some nodes, called seed nodes, and transmits predefined heats on these nodes to other nodes in the network. A heat assigned to a node represents the strength of the associations between the node and seed nodes. Here, we adopted one kind of heat diffusion algorithm named LHD (Leiserson et al., 2015) to infer novel uveitis-related genes in PPI network *N*. The brief description of LHD algorithm was as below.

Given a PPI network *N*, let *A* be its adjacent matrix and *D* is a diagonal matrix storing the degree of each node. The graph Laplacian *L* was defined as *D*-*A*. According to the 113 Ensembl IDs, which were assigned to uveitis-related genes and used as seed nodes in LHD algorithm, an original heat distribution vector H_0_ can be constructed in a way that the components corresponding to seed nodes were set to 1/113 and others were set as zero. The heat distribution vector at time *t* can be accessed by

(1) $$H_{t}=H_{0}•\exp(-Lt)$$

where exp( ) is the matrix exponential. By setting a series of increasing values of *t*, we can obtain a series of heat distribution vectors. When two consecutive distribution vectors are quite similar, one vector is assigned as the output of LHD algorithm. In the output vector, each node, including seed nodes, received a heat value. We only extracted the heat values of non-seed nodes, which would be further used for selecting important genes.

#### Permutation Test

Each node received a heat value based on the LHD algorithm. However, this value may be affected by the structure of the PPI network *N*, i.e., some nodes may have high probabilities to receive high heat values regardless of which nodes are selected as seed nodes. Therefore, these nodes should not be selected as candidate genes of uveitis. To control this type of nodes, we performed a permutation test. We constructed 500 Ensembl ID sets, each comprising 113 Ensembl IDs that were randomly selected from the nodes in the PPI network *N*. For each set, the nodes were taken as seed nodes, which were inputted onto the LHD algorithm. After the 500 sets were tested, each node received several heat values. By comparing these values with the heat value obtained by using 113 Ensembl IDs of uveitis-related genes, we can compute a measurement, called zscore, to evaluate the reliability of the actual heat value. Zscore can defined as below:

(2) $$zscore\;(g)=\frac{h\;(g)-\text{μ }(g)}{\text{δ }(g)}$$

where h(*g*) is the heat value of gene *g* obtained by 113 Ensembl IDs of uveitis-related genes, and μ(*g*) and δ(*g*) are the mean and standard deviation, respectively, of the heat values obtained by 500 randomly produced sets. According to statistical theory, 1.96 is the threshold for selecting genes that significantly correlate with uveitis-related genes. Thus, we extracted Ensembl IDs with zscores no less than 1.96. These IDs would be further analyzed by the following tests.

#### Interaction Test

Large numbers of genes were discarded through the permutation test. For the remaining genes, some were strongly associated with uveitis-related genes, indicating their high relationships to uveitis. By contrast, others had few, even no associations with uveitis-related genes, implying that they were not related to uveitis and should be discarded. To indicate the association between the candidate genes passing the permutation test and the uveitis-related genes, we employed the PPI information mentioned in Section “Construction of PPI Network”. The score was also used to quantify the strength of the PPI. The score of the PPI of proteins *p*_1_ and *p*_2_ was denoted by *S*(*p*_1_, *p*_2_). For one candidate gene *g*, we assigned a measurement called maximum interaction score (MIS), which was defined as:

(3) MIS (g)=max{S(g, g′):g′ isauveitisrelatedgene}

A high MIS indicates that the gene was highly related to at least one uveitis-related gene. Accordingly, this specific gene may also share the functions shared by this uveitis-related gene and thus has a high probability to become a novel uveitis-related gene. According to the setting in STRING, 900 was the threshold of highest confidence and was also set as the threshold of MIS, i.e., candidate genes passing the permutation test were retained if their MISs were no less than 900.

#### Function Test

The last test measured the linkage between candidate genes and uveitis-related genes based on gene ontology (GO) terms and biological pathways. Each gene has special relationships with some GO terms or pathways. If one candidate gene exhibited GO terms or pathways that are similar to one uveitis related gene, then it may be highly related to this uveitis-related gene and thus shows a high probability of being a novel uveitis gene. To complete this test, we employed a scheme to indicate the relationship between a gene and a GO term (pathway). Here, we adopted the GO and KEGG enrichment theory (Chen et al., 2016b, 2017a,b, 2018c; Liu et al., 2016; Lu et al., 2017; Li et al., 2018a,b), which can transform the relationship between one gene and one GO term (KEGG pathway) into a number. A vector, denoted as *ES* (*g*), can be obtained by collecting the numbers of one gene *g* between all GO terms and KEGG pathways. We further used the direction cosine of two vectors *ES* (*g*) and *ES* (*g′*) to measure the linkage between g and g′ in terms of GO terms and KEGG pathways. The direction was defined as:

(4) $$C(g\text{, }g\prime )=\frac{ES(g)•ES(g\prime )}{\left||ES(g)||•||ES(g\prime )|\right|}$$

where *ES* (*g*) ∙ *ES* (*g′*) is the dot product of two vectors and ||*ES* (*g*)|| is the modulus of the vector *ES* (*g*). A high outcome of equation 4 suggested strong associations between two genes. We assigned the last measurement named maximum function score (MFS), which is similar to MIS, to the candidate gene *g*. MFS can be computed by:

(5) MFS (g)=max{C(g, g′)}:g′ isauveitisrelatedgene}

Then, 0.97 was set as the threshold of MFS to extract final candidate genes. For convenience, the final obtained genes were called inferred genes.

## Results

**FIGURE 1:**
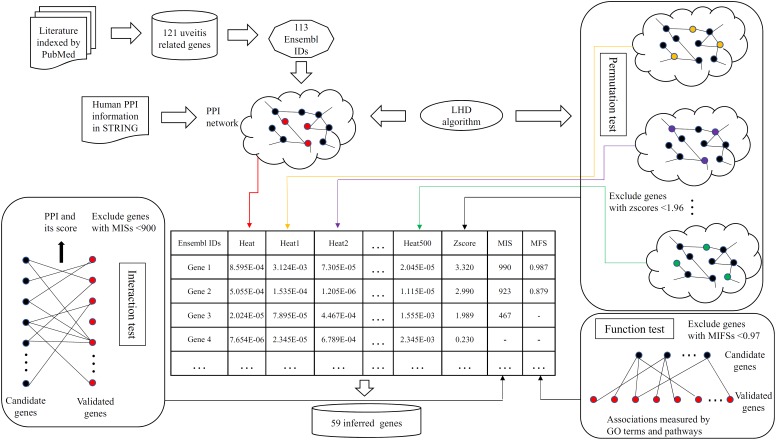
Flow chart showing the detailed procedures of the computational method for inferring novel uveitis-related genes. Ensembl IDs of uveitis-related genes were used as the seed nodes of Laplacian heat diffusion (LHD) algorithm, resulting in a heat value for each gene in the PPI network. In the permutation test, the LHD algorithm was executed 500 times with different seed nodes, yielding 500 heat values for each gene. Then, a zscore (cf. equation 2) of each gene was calculated and those with zscores less than 1.96 were discarded. The interaction test assessed each candidate gene by checking its associations to validated genes and excluded candidate genes with MISs (cf. equation 3) less than 900. Finally, the remaining genes were evaluated in the function test, which measured candidate genes by investigating their linkages based on gene ontology (GO) terms and biological pathways. Candidate genes with MFSs (cf. equation 5) less than 0.97 were discarded. Fifty-nine inferred genes were obtained.

In this study, we set up a computation method to infer novel uveitis-related genes based on validated ones retrieved from published literature. All procedures are shown in **Figure 1**. This section gave the detailed results of this method.

The Ensembl IDs for uveitis-related genes were picked up as seed nodes for LHD algorithm. Except for the uveitis-related ones, all genes were assigned heat values that are available in **Supplementary Table S2**. However, these values may be affected by the structure of the PPI network *N*. Genes receiving high heat values were not always highly related to uveitis. Accordingly, a permutation test was performed. Measurement zscores were computed and assigned to each candidate gene and are also available in **Supplementary Table S2**. According to Section “Permutation Test”, we selected genes with zscores no less than 1.96, accessing 1,287 candidate genes.

For the 1,287 candidate genes, we further filtered them by interaction test. The measurement MIS was calculated and assigned to each of these genes and is listed in **Supplementary Table S2**. We set 900 as its threshold, resulting in 391 candidate genes. In the function test, each of the 391 candidate genes was evaluated by MFS (see **Supplementary Table S2**). We set 0.97 as the threshold and finally obtained 59 genes (see first 59 genes in **Supplementary Table S2**). These genes were regarded to be highly related to uveitis and thus called inferred genes.

**FIGURE 2:**
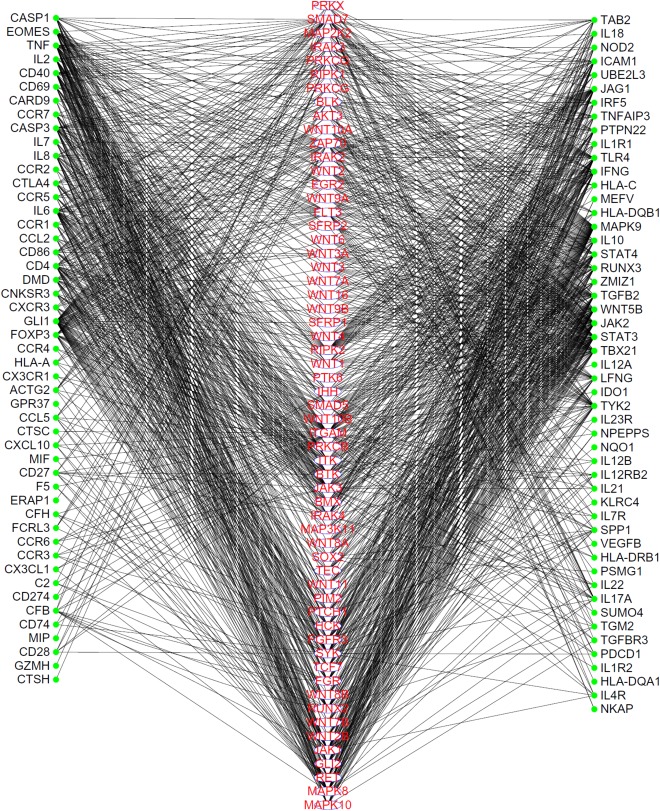
The linkages between inferred uveitis-related genes and validated ones, which were extracted from the PPI network in STRING. Genes in the middle column are inferred genes, while genes in the left or right columns are validated genes. Each inferred gene has strong associations to validated genes.

To show the high probabilities of inferred genes being novel uveitis-related genes, we extracted the linkages between 59 inferred genes and validated uveitis-related genes from the PPI network, as shown in **Figure 2**. It can be seen that each of these genes have strong associations with validated genes, proving that they can be novel uveitis-related genes.

## Discussion

In this study, we set up a new computational method for inferring novel uveitis-related genes. The method finally produced 59 inferred genes. This section first presents a comparison of the resulting genes with those reported in a previous study (Lu et al., 2017) and then gives an extensive analysis on several inferred genes.

### Comparison With Genes Reported in a Previous Study

**FIGURE 3:**
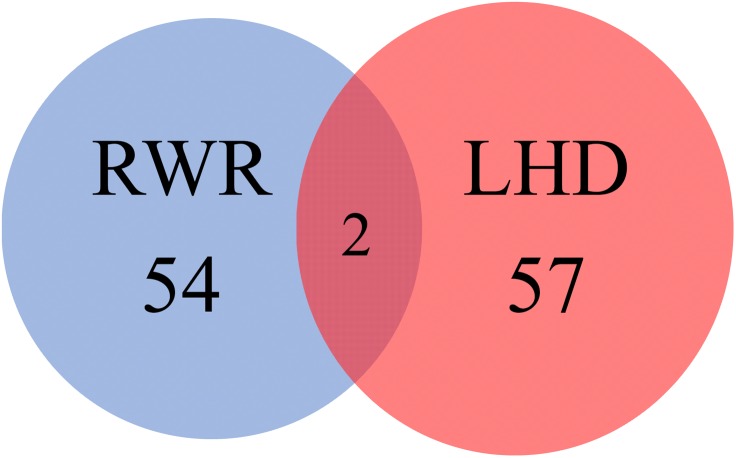
Venn diagram illustrating two gene sets, consisting of novel genes in a previous study and inferred genes reported in this study. The blue circle represents the gene set consisting of novel genes in a previous study, which adopted RWR algorithm to search novel genes; while the red circle represents the gene set containing the inferred genes reported in this study, which were identified by LHD algorithm.

A previous study (Lu et al., 2017) reported 56 novel genes that were related to uveitis and were accessed by using RWR algorithm and some screening tests. In the present study, 59 inferred uveitis-related genes were finally obtained. The Venn diagram on two gene sets, consisting of novel genes in Lu et al. (2017) and inferred genes in our study, is shown in **Figure 3**. We can see that only two genes, JAK1 (ENSP00000343204) and MAPK8 (ENSP00000353483), were identified by both methods. The Jaccard coefficient of these two sets was 1.77%, implying that the novel genes yielded by two methods were quite different. In addition, our inferred genes can be important supplements for the previous study if we can prove them to be highly related to uveitis. This result would be elaborated in the following subsection.

### Analysis of Inferred Genes

Our computational method identified 59 inferred genes and regarded them to be highly related to uveitis. To confirm this, we did the GO and KEGG enrichment on them using R program clusterProfiler for detailed functional annotation. The results are provided in **Supplementary Tables S3**, **S4**. It can be observed that the inferred genes are functionally enriched in some GO terms, such as frizzled binding (GO:0005109), G-protein coupled receptor binding (GO:0001664), non-membrane spanning protein tyrosine kinase activity (GO:0004715), and protein tyrosine kinase activity (GO:0004713). The enriched KEGG pathways included signaling pathways regulating pluripotency of stem cells (hsa04550), Wnt signaling pathway (hsa04310), and melanogenesis (hsa04916).

**FIGURE 4:**
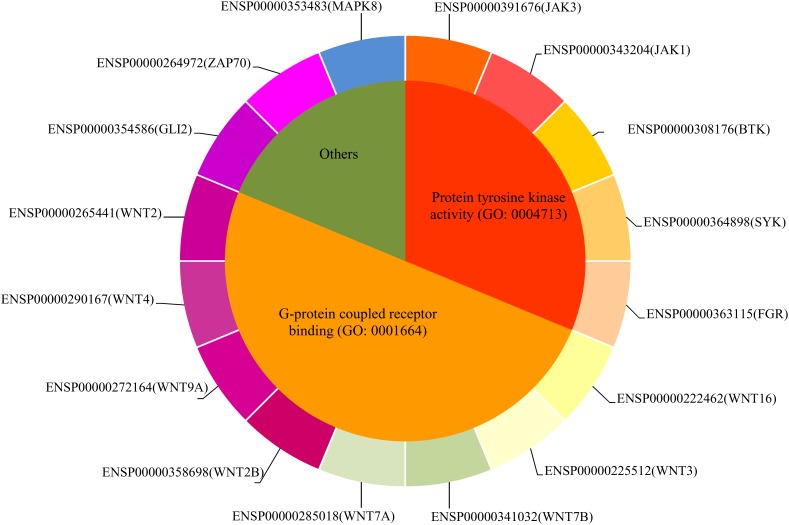
The distribution of sixteen important inferred genes.

According to recent publications, all inferred genes can be proved to be related to such disease or related pathogenic processes (**Supplementary Table S5**). Here, we selected important ones for detailed analyses, their detailed information is listed in **Table 1**. According to the enrichment analysis, we clustered these genes into three functional groups, as shown in **Figure 4**.

**Table 1:** Sixteen important inferred uveitis-related genes.

Ensembl ID	Gene symbol	Description	Heat	Zscore	MIS	MFS
ENSP00000391676	JAK3	Janus Kinase 3	8.595E-04	2.206	999	0.996
ENSP00000343204	JAK1	Janus Kinase 1	5.055E-04	2.999	999	0.995
ENSP00000308176	BTK	Bruton’s Tyrosine Kinase	5.248E-04	3.065	966	0.992
ENSP00000364898	SYK	Spleen Associated Tyrosine Kinase	5.178E-04	2.945	996	0.991
ENSP00000363115	FGR	FGR Proto-Oncogene, Src Family Tyrosine Kinase	5.289E-04	4.080	989	0.989
ENSP00000222462	WNT16	Wnt Family Member 16	2.024E-03	9.020	906	0.995
ENSP00000225512	WNT3	Wnt Family Member 3	1.584E-03	9.033	904	0.995
ENSP00000341032	WNT7B	Wnt Family Member 7B	1.572E-03	8.923	904	0.994
ENSP00000285018	WNT7A	Wnt Family Member 7A	1.626E-03	8.312	904	0.994
ENSP00000358698	WNT2B	Wnt Family Member 2B	1.648E-03	8.858	903	0.993
ENSP00000272164	WNT9A	Wnt Family Member 9A	1.874E-03	9.005	942	0.992
ENSP00000290167	WNT4	Wnt Family Member 4	1.109E-03	7.898	904	0.991
ENSP00000265441	WNT2	Wnt Family Member 2	1.361E-03	8.989	903	0.992
ENSP00000354586	GLI2	GLI Family Zinc Finger 2	7.961E-04	4.422	918	0.991
ENSP00000264972	ZAP70	Zeta Chain Of T Cell Receptor Associated Protein Kinase 70	5.018E-04	2.840	956	0.990
ENSP00000353483	MAPK8	Mitogen-Activated Protein Kinase 8	4.108E-03	6.884	993	0.985

#### Genes With Protein Tyrosine Kinase Activity (GO:0004713)

The first inferred gene is a functional immune cell specifically expressed gene JAK3 (ENSP00000391676) (Lee et al., 2012) and is pathologically related to autosomal severe combined immunodeficiency disease (Notarangelo et al., 2001; Bogaert et al., 2016). For its specific contribution on the pathogenesis of uveitis, a specific study (Liao et al., 2018) on ankylosing spondylitis confirmed that the expression levels of JAK1 and JAK3 are positively correlated with various autoimmune diseases, including enthesitis, ankylosing spondylitis and uveitis, thereby validating their potential pathogenic contributions.

Apart from JAK3, another gene named as JAK1 (ENSP00000343204) was also inferred to be a potential pathogenic uveitis-related gene by our method. Based on the publications mentioned above, such gene was also confirmed to participate in uveitis-related pathogenesis.

Apart from that, the next inferred gene was BTK (ENSP00000308176). BTK contributes to the regulation of B cell maturation and proliferation under physical or pathological conditions (Wu et al., 2014; Nagel et al., 2015). According to recent publications, BTK is pathologically connected to uveitis by interfering immune cell differentiation (Vargas et al., 2013) and autoimmune responses (Corneth et al., 2016).

The next gene was SYK (ENSP00000364898), which contributes to the regulation of cellular responses, including proliferation, differentiation, and phagocytosis in B, T and myeloid cells (Luger et al., 2013; Hauck et al., 2015; Wang et al., 2015). For its specific contribution on the pathogenesis of uveitis, SYK/CARD9 signaling axis participates in the pathogenesis of autoimmune eye diseases, including ocular inflammatory disorders, uveitis, and dry eye disease (Lee et al., 2016; Hagan et al., 2018).

Another study (Wang et al., 2008) reported that FGR (ENSP00000363115) may also participate in eye diseases, including age-related macular degeneration and uveitis.

#### Genes With G-Protein Coupled Receptor Binding (GO:0001664) Capacity

Apart from being proliferation regulatory genes in immune cells, JAK1 and JAK3, the next inferred gene, are also members of a functional WNT gene family WNT16 (ENSP00000222462). For encoding of a secreted signaling protein, such gene encodes a ligand for the members of frizzled family of seven transmembrane receptors (Wergedal et al., 2015; Ozeki et al., 2016). For its specific contribution on the pathogenic approach of uveitis, no direct evidence confirmed that WNT16 participates in uveitis-specific pathogenesis. However, two recent studies (Reischl et al., 2007; Nalesso et al., 2017) confirmed that WNT16, together with MNT5, participate in the pathogenesis of uveitis by interfering immune responses.

A homolog of WNT16, WNT3 (ENSP00000225512) was also predicted to participate in uveitis-specific pathogenesis. According to a recent publication on the genetic components of wnt/β-catenin signaling pathway (Nakatsu et al., 2011), this gene is similar to our predicted biomarker WNT3 and regulates the proliferation of eye cells, including urea cells (Nakatsu et al., 2011). The abnormal proliferation of urea cells, especially immune cells, may trigger the initiation and proliferation of uveitis. Therefore, speculating that WNT16 and WNT3 may be functionally related to uveitis is reasonable.

The next inferred gene named WNT7B (ENSP00000341032) is also a specific member of wnt/β-catenin signaling pathway. According to the same literature mentioned above, such gene participating in wnt/β-catenin signaling pathway may also contribute to the pathogenic progressions of uveitis due to its abnormal regulatory role on urea immune cell proliferation *in situ*. As a homolog of WNT7B, WNT7A (ENSP00000285018) acts as one of the potential pathogenic factors of uveitis by interfering immune cell proliferation and maturation. Similarly, WNT2B (ENSP00000358698), WNT9A (ENSP00000272164), WNT4 (ENSP00000290167), and WNT2 (ENSP00000265441) all participate in the pathogenesis of uveitis through the regulation of urea cells, thus validating their strong relationships with uveitis.

We screened out eight WNT signaling pathway components that may contribute to the pathogenesis of uveitis. According to recent publications, our inferred genes (WNT2, WNT16, WNT3, WNT7A, and WNT7B) are functionally related to the initiation and progression of uveitis due to their interference to the proliferation of urea and mixed immune cells.

#### Other Functional Inferred Uveitis-Related Genes

GLI2 (ENSP00000354586) was also regarded as a potential pathogenic gene of uveitis. Such gene has been widely reported to act as a transcriptional regulator by encoding a C2H2-type zinc finger protein (Eichberger et al., 2006; Jackson et al., 2015). For its specific contribution on uveitis, GLI2 regulates Notch-Gli2 axis and hedgehog signaling pathway; however, no direct reports confirmed its pathogenic role (Roessler et al., 2003; Ringuette et al., 2016). In the pathogenic conditions of urea tissues, the inflammatory environment is also regulated by such two pathways (Takezaki et al., 2011; Swiderska-Syn et al., 2013). Therefore, this gene can be regarded as a potential uveitis-related gene.

ZAP70 (ENSP00000264972) is also a functional inferred uveitis-related gene. According to a recent publication, ZAP70 is a potential biomarker instructing the onset of uveitis in mouse models (Kleinwort et al., 2016). Therefore, this gene can be inferred as a uveitis-related gene.

MAPK8 (ENSP00000353483) was inferred to be potential uveitis-related gene, participating in the specific pathogenesis of such disease. It is necessary to point out that this gene was also reported in one previous study (Lu et al., 2017). Early in 2014, a systematic study (Lisanti et al., 2014) on tumor stroma confirmed that MAPK8 contribute to the pathogenesis of a typical tumor complication, uveitis, corresponding with our prediction. In the same year, another independent study (Honke et al., 2014) validated that p38-MAPK8 participated in the specific IL-6 mediated inflammatory responses. Therefore, such identified gene MAPK8 may also be a specific uveitis associated gene, corresponding with previous studies and publications.

Literature-based analysis confirmed some of the inferred genes as participating in uveitis, thus validating that our results are reliable. The rest of the inferred genes were left for readers. Most of them are suggested to be related to uveitis.

## Conclusion

This study aims to infer novel uveitis-related genes. An efficient network algorithm, LHD algorithm, was adopted as the basic searching algorithm and was executed on a PPI network using validated uveitis-related genes as seed nodes. With the help of three screening tests, 59 functional genes were finally accessed. These novel inferred genes can be useful materials to uncover the pathogenesis of uveitis.

## Author Contributions

All authors contributed to the research and reviewed the manuscript. MY designed the study. SL, KZ, XW, and HL performed the experiments. SL, KZ, XA, and YX analyzed the results. SL and KZ wrote the manuscript.

## Conflict of Interest Statement

The authors declare that the research was conducted in the absence of any commercial or financial relationships that could be construed as a potential conflict of interest.

## Footnotes

1 http://www.ncbi.nlm.nih.gov/pubmed/

2 https://string-db.org
