# Supplementary material for: Use of Laplacian Heat Diffusion Algorithm to Infer Novel Genes With Functions Related to Uveitis
Source: Front Genet. 2018 Oct 8;9:425. doi: 10.3389/fgene.2018.00425 (PMC6186792; doi:10.3389/fgene.2018.00425)
Supplement: Supplementary file 1 [file Table_1.DOCX]

Supplementary Material

Use of Laplacian heat diffusion algorithm to infer novel genes with functions related to uveitis

Shiheng Lu, Ke Zhao, Xuefei Wang, Hui Liu, Xiamuxiya Ainiwaer, Yan Xu, Min Ye*

*** Correspondence:** Min Ye: gleye@163.com

**Supplementary Table 1.** Uveitis related genes and Ensembl IDs of their proteins.

| **Gene symbol** | **Ensembl ID** |
| --- | --- |
| C2 | ENSP00000299367 |
| CD4 | ENSP00000011653 |
| CFB | ENSP00000416561  ENSP00000410815 |
| CFH | ENSP00000356399 |
| CRP | ENSP00000255030 |
| DMD | ENSP00000354923 |
| F5 | ENSP00000356771 |
| IL2 | ENSP00000226730 |
| IL6 | ENSP00000258743 |
| IL7 | ENSP00000263851 |
| IL8 | ENSP00000306512 |
| MIF | ENSP00000215754 |
| MIP | ENSP00000257979 |
| TNF | ENSP00000398698 |
| ACTG2 | ENSP00000295137 |
| CARD15 | - |
| CARD9 | ENSP00000360797 |
| CASP1 | ENSP00000410076 |
| CASP3 | ENSP00000311032 |
| CCL2 | ENSP00000225831 |
| CCL5 | ENSP00000293272 |
| CCR1 | ENSP00000296140 |
| CCR2 | ENSP00000292301 |
| CCR3 | ENSP00000441600 |
| CCR4 | ENSP00000332659 |
| CCR5 | ENSP00000292303 |
| CCR6 | ENSP00000339393 |
| CCR7 | ENSP00000246657 |
| CD25 | - |
| CD27 | ENSP00000266557 |
| CD274 | ENSP00000370989 |
| CD28 | ENSP00000324890 |
| CD30 | - |
| CD40 | ENSP00000361359 |
| CD45 | - |
| CD57 | - |
| CD69 | ENSP00000228434 |
| CD74 | ENSP00000009530 |
| CD86 | ENSP00000332049 |
| CNKSR3 | ENSP00000356182 |
| CTLA4 | ENSP00000303939 |
| CTSC | ENSP00000227266 |
| CTSH | ENSP00000220166 |
| CX3CL1 | ENSP00000006053 |
| CX3CR1 | ENSP00000351059 |
| CXCL10 | ENSP00000305651 |
| CXCR3 | ENSP00000362795 |
| ECAD | - |
| EOMES | ENSP00000295743 |
| ERAP1 | ENSP00000296754 |
| ERAP2 | ENSP00000400376 |
| FCRL3 | ENSP00000357167 |
| FOXP3 | ENSP00000365380 |
| GIMAP2 | ENSP00000223293 |
| GIMAP4 | ENSP00000255945 |
| GLI1 | ENSP00000228682 |
| GPR35 | ENSP00000415890 |
| GPR37 | ENSP00000306449 |
| GPR65 | ENSP00000267549 |
| GZMH | ENSP00000216338 |
| HLA-A | ENSP00000366005 |
| HLA-B | ENSP00000399168 |
| HLA-C | ENSP00000365402 |
| ICAM1 | ENSP00000264832 |
| IDO1 | ENSP00000430505 |
| IFNG | ENSP00000229135 |
| IL10 | ENSP00000412237 |
| IL12A | ENSP00000303231 |
| IL12B | ENSP00000231228 |
| IL12RB2 | ENSP00000262345 |
| IL17A | ENSP00000344192 |
| IL18 | ENSP00000280357 |
| IL1R1 | ENSP00000233946 |
| IL1R2 | ENSP00000330959 |
| IL21 | ENSP00000264497 |
| IL22 | ENSP00000329384 |
| IL23 | - |
| IL23R | ENSP00000321345 |
| IL4R | ENSP00000170630 |
| IL7R | ENSP00000306157 |
| IRF5 | ENSP00000349770 |
| JAG1 | ENSP00000254958 |
| JAK2 | ENSP00000371067 |
| KLRC4 | ENSP00000310216 |
| LCE3B | ENSP00000335358 |
| LCE3C | ENSP00000334644 |
| LFNG | ENSP00000222725 |
| LNPEP | ENSP00000231368 |
| MAPK9 | ENSP00000389338 |
| MEFV | ENSP00000219596 |
| NKAP | ENSP00000360464 |
| NOD2 | ENSP00000300589 |
| NPEPPS | ENSP00000320324 |
| NQO1 | ENSP00000319788 |
| PDCD1 | ENSP00000335062 |
| PSMG1 | ENSP00000329915 |
| PTPN22 | ENSP00000352833 |
| RAB27A | - |
| RALGPS2 | ENSP00000356607 |
| RUNX3 | ENSP00000343477 |
| SPP1 | ENSP00000378517 |
| STAT3 | ENSP00000264657 |
| STAT4 | ENSP00000351255 |
| SUMO4 | ENSP00000318635 |
| TAB2 | ENSP00000286332 |
| TBX21 | ENSP00000177694 |
| TGFB2 | ENSP00000355896 |
| TGFBR3 | ENSP00000212355 |
| TGM2 | ENSP00000355330 |
| TLR4 | ENSP00000363089 |
| TNFAIP3 | ENSP00000237289 |
| TYK2 | ENSP00000264818 |
| UBAC2 | ENSP00000383911 |
| UBE2L3 | ENSP00000344259 |
| VEGFB | ENSP00000311127 |
| WNT5B | ENSP00000308887 |
| ZMIZ1 | ENSP00000334474 |
| HLA-DQA1 | ENSP00000339398 |
| HLA-DQB1 | ENSP00000382034 |
| HLA-DRB1 | ENSP00000353099 |
| PDCD1LG2 | - |
