# Supplementary material for: Use of Laplacian Heat Diffusion Algorithm to Infer Novel Genes With Functions Related to Uveitis
Source: Front Genet. 2018 Oct 8;9:425. doi: 10.3389/fgene.2018.00425 (PMC6186792; doi:10.3389/fgene.2018.00425)
Supplement: Supplementary file 3 [file Table_3.DOCX]

Supplementary Material

Use of Laplacian heat diffusion algorithm to infer novel genes with functions related to uveitis

Shiheng Lu, Ke Zhao, Xuefei Wang, Hui Liu, Xiamuxiya Ainiwaer, Yan Xu, Min Ye*

*** Correspondence:** Min Ye: gleye@163.com

**Supplementary Table 3.** Gene ontology enrichment analysis on 59 inferred uveitis-related genes.

| **ID** | **Description** | **GeneRatio** | **BgRatio** | **pvalue** | **p.adjust** | **qvalue** | **geneID** | **Count** |
| --- | --- | --- | --- | --- | --- | --- | --- | --- |
| GO:0005109 | frizzled binding | 18/59 | 36/17354 | 1.79E-36 | 2.43E-34 | 1.64E-34 | 51384/7473/7477/7476/7482/7472/7483/54361/7481/89780/7484/7471/80326/7475/7478/7480/7479/6422 | 18 |
| GO:0001664 | G-protein coupled receptor binding | 20/59 | 262/17354 | 3.01E-22 | 2.05E-20 | 1.38E-20 | 3716/51384/7473/7477/7476/7482/7472/7483/54361/7481/89780/7484/7471/80326/7475/7478/5727/7480/7479/6422 | 20 |
| GO:0004715 | non-membrane spanning protein tyrosine kinase activity | 13/59 | 51/17354 | 8.48E-22 | 3.84E-20 | 2.59E-20 | 3718/3716/695/6850/7535/2268/3055/3702/5753/640/8767/660/7006 | 13 |
| GO:0004713 | protein tyrosine kinase activity | 17/59 | 179/17354 | 1.49E-20 | 5.06E-19 | 3.41E-19 | 3718/3716/695/6850/7535/2268/3055/2322/3702/5605/5753/2261/640/8767/660/5979/7006 | 17 |
| GO:0004674 | protein serine/threonine kinase activity | 16/59 | 450/17354 | 1.27E-12 | 3.44E-11 | 2.32E-11 | 6850/10000/5599/5605/11040/5602/5579/51135/8767/4296/5588/5582/3656/5613/8737/11213 | 16 |
| GO:0048018 | receptor ligand activity | 14/59 | 456/17354 | 2.77E-10 | 6.28E-09 | 4.23E-09 | 7473/7477/7476/7472/7483/54361/89780/7484/7471/80326/7478/7480/6423/7479 | 14 |
| GO:0030545 | receptor regulator activity | 14/59 | 485/17354 | 6.19E-10 | 1.20E-08 | 8.10E-09 | 7473/7477/7476/7472/7483/54361/89780/7484/7471/80326/7478/7480/6423/7479 | 14 |
| GO:0005057 | signal transducer activity, downstream of receptor | 6/59 | 122/17354 | 3.55E-06 | 6.03E-05 | 4.06E-05 | 6850/5599/5602/4090/4092/4296 | 6 |
| GO:0004697 | protein kinase C activity | 3/59 | 16/17354 | 2.03E-05 | 0.000306085 | 0.000206 | 5579/5588/5582 | 3 |
| GO:0001784 | phosphotyrosine residue binding | 3/59 | 35/17354 | 0.000226111 | 0.003075104 | 0.002071 | 6850/2268/3055 | 3 |
| GO:0004712 | protein serine/threonine/tyrosine kinase activity | 3/59 | 42/17354 | 0.000389956 | 0.004821276 | 0.003247 | 5605/5602/5582 | 3 |
| GO:0045309 | protein phosphorylated amino acid binding | 3/59 | 44/17354 | 0.000447711 | 0.004881108 | 0.003287 | 6850/2268/3055 | 3 |
| GO:0005072 | transforming growth factor beta receptor, cytoplasmic mediator activity | 2/59 | 10/17354 | 0.000502467 | 0.004881108 | 0.003287 | 4090/4092 | 2 |
| GO:0039706 | co-receptor binding | 2/59 | 10/17354 | 0.000502467 | 0.004881108 | 0.003287 | 89780/7484 | 2 |
| GO:0009931 | calcium-dependent protein serine/threonine kinase activity | 2/59 | 11/17354 | 0.000612784 | 0.005555907 | 0.003741 | 5579/5582 | 2 |
| GO:0010857 | calcium-dependent protein kinase activity | 2/59 | 12/17354 | 0.000733734 | 0.006236736 | 0.0042 | 5579/5582 | 2 |
| GO:0004707 | MAP kinase activity | 2/59 | 14/17354 | 0.001007248 | 0.008057982 | 0.005426 | 5599/5602 | 2 |
| GO:0004708 | MAP kinase kinase activity | 2/59 | 15/17354 | 0.001159671 | 0.008761958 | 0.0059 | 5605/5602 | 2 |
| GO:0004714 | transmembrane receptor protein tyrosine kinase activity | 3/59 | 62/17354 | 0.001224239 | 0.00876297 | 0.005901 | 2322/2261/5979 | 3 |
| GO:0004702 | signal transducer, downstream of receptor, with serine/threonine kinase activity | 3/59 | 70/17354 | 0.001738169 | 0.011819549 | 0.007959 | 5599/5602/4296 | 3 |
| GO:0051219 | phosphoprotein binding | 3/59 | 77/17354 | 0.002283998 | 0.014151055 | 0.009529 | 6850/2268/3055 | 3 |
| GO:0042813 | Wnt-activated receptor activity | 2/59 | 21/17354 | 0.002289141 | 0.014151055 | 0.009529 | 6423/6422 | 2 |
| GO:0019199 | transmembrane receptor protein kinase activity | 3/59 | 79/17354 | 0.00245729 | 0.01453006 | 0.009784 | 2322/2261/5979 | 3 |
| GO:0031625 | ubiquitin protein ligase binding | 5/59 | 299/17354 | 0.003438714 | 0.019486049 | 0.013121 | 3716/4090/1959/4092/8737 | 5 |
| GO:0044389 | ubiquitin-like protein ligase binding | 5/59 | 313/17354 | 0.004176242 | 0.022718758 | 0.015298 | 3716/4090/1959/4092/8737 | 5 |
| GO:0017147 | Wnt-protein binding | 2/59 | 31/17354 | 0.004959492 | 0.025941959 | 0.017469 | 6423/6422 | 2 |
