# Supplementary material for: Use of Laplacian Heat Diffusion Algorithm to Infer Novel Genes With Functions Related to Uveitis
Source: Front Genet. 2018 Oct 8;9:425. doi: 10.3389/fgene.2018.00425 (PMC6186792; doi:10.3389/fgene.2018.00425)
Supplement: Supplementary file 4 [file Table_4.DOCX]

Supplementary Material

Use of Laplacian heat diffusion algorithm to infer novel genes with functions related to uveitis

Shiheng Lu, Ke Zhao, Xuefei Wang, Hui Liu, Xiamuxiya Ainiwaer, Yan Xu, Min Ye*

*** Correspondence:** Min Ye: gleye@163.com

**Supplementary Table 4.** KEGG enrichment analysis on 59 inferred uveitis-related genes.

| **ID** | **Description** | **GeneRatio** | **BgRatio** | **pvalue** | **p.adjust** | **qvalue** | **geneID** | **Count** |
| --- | --- | --- | --- | --- | --- | --- | --- | --- |
| hsa05217 | Basal cell carcinoma | 20/55 | 63/7441 | 5.17E-29 | 8.12E-27 | 3.05E-27 | 51384/7473/7477/7476/7482/7472/7483/54361/2736/7481/89780/7484/7471/80326/7475/6932/7478/5727/7480/7479 | 20 |
| hsa04550 | Signaling pathways regulating pluripotency of stem cells | 24/55 | 139/7441 | 6.42E-28 | 5.04E-26 | 1.89E-26 | 3718/3716/51384/7473/7477/7476/7482/7472/7483/54361/7481/89780/7484/7471/10000/5605/80326/7475/7478/7480/4090/2261/7479/6657 | 24 |
| hsa04310 | Wnt signaling pathway | 24/55 | 146/7441 | 2.26E-27 | 1.18E-25 | 4.44E-26 | 51384/7473/7477/7476/7482/7472/7483/54361/7481/89780/7484/7471/5599/80326/7475/6932/7478/5602/5579/7480/6423/7479/5582/6422 | 24 |
| hsa04916 | Melanogenesis | 21/55 | 101/7441 | 3.98E-26 | 1.56E-24 | 5.86E-25 | 51384/7473/7477/7476/7482/7472/7483/54361/7481/89780/7484/7471/5605/80326/7475/6932/7478/5579/7480/7479/5582 | 21 |
| hsa05225 | Hepatocellular carcinoma | 22/55 | 168/7441 | 1.03E-22 | 3.22E-21 | 1.21E-21 | 51384/7473/7477/7476/7482/7472/7483/54361/7481/89780/7484/7471/10000/5605/80326/7475/6932/7478/5579/7480/7479/5582 | 22 |
| hsa05205 | Proteoglycans in cancer | 23/55 | 201/7441 | 2.09E-22 | 5.46E-21 | 2.05E-21 | 51384/7473/7477/7476/7482/7472/7483/54361/7481/89780/7484/7471/10000/5605/80326/7475/7478/5727/5579/7480/7479/3549/5582 | 23 |
| hsa04150 | mTOR signaling pathway | 21/55 | 151/7441 | 3.23E-22 | 7.24E-21 | 2.72E-21 | 51384/7473/7477/7476/7482/7472/7483/54361/7481/89780/7484/7471/10000/5605/80326/7475/7478/5579/7480/7479/5582 | 21 |
| hsa04390 | Hippo signaling pathway | 21/55 | 154/7441 | 4.96E-22 | 9.73E-21 | 3.65E-21 | 51384/7473/7477/7476/7482/7472/7483/54361/2736/7481/89780/7484/7471/80326/7475/6932/7478/7480/4092/7479/6657 | 21 |
| hsa05224 | Breast cancer | 20/55 | 147/7441 | 6.17E-21 | 1.08E-19 | 4.04E-20 | 51384/7473/7477/7476/7482/7472/7483/54361/7481/89780/7484/7471/10000/5605/80326/7475/6932/7478/7480/7479 | 20 |
| hsa05226 | Gastric cancer | 20/55 | 149/7441 | 8.16E-21 | 1.28E-19 | 4.81E-20 | 51384/7473/7477/7476/7482/7472/7483/54361/7481/89780/7484/7471/10000/5605/80326/7475/6932/7478/7480/7479 | 20 |
| hsa04934 | Cushing syndrome | 19/55 | 154/7441 | 4.83E-19 | 6.90E-18 | 2.59E-18 | 51384/7473/7477/7476/7482/7472/7483/54361/7481/89780/7484/7471/5605/80326/7475/6932/7478/7480/7479 | 19 |
| hsa05166 | Human T-cell leukemia virus 1 infection | 22/55 | 255/7441 | 1.13E-18 | 1.48E-17 | 5.55E-18 | 3718/3716/51384/7473/7477/7476/7482/7472/7483/54361/7481/89780/7484/7471/10000/5599/80326/7475/7478/7480/1959/7479 | 22 |
| hsa05165 | Human papillomavirus infection | 21/55 | 339/7441 | 7.51E-15 | 9.07E-14 | 3.41E-14 | 3716/51384/7473/7477/7476/7482/7472/7483/54361/7481/89780/7484/7471/10000/5605/80326/7475/6932/7478/7480/7479 | 21 |
| hsa04722 | Neurotrophin signaling pathway | 8/55 | 119/7441 | 2.20E-06 | 2.46E-05 | 9.25E-06 | 10000/5599/5605/5602/51135/8767/3656/11213 | 8 |
| hsa05152 | Tuberculosis | 9/55 | 179/7441 | 5.41E-06 | 5.34E-05 | 2.01E-05 | 3716/6850/10000/5599/5602/51135/8767/3684/3656 | 9 |
| hsa04064 | NF-kappa B signaling pathway | 7/55 | 95/7441 | 5.45E-06 | 5.34E-05 | 2.01E-05 | 695/6850/7535/5579/51135/5588/8737 | 7 |
| hsa05221 | Acute myeloid leukemia | 6/55 | 66/7441 | 7.97E-06 | 7.36E-05 | 2.76E-05 | 2322/10000/5605/6932/11040/3684 | 6 |
| hsa05161 | Hepatitis B | 8/55 | 144/7441 | 9.14E-06 | 7.84E-05 | 2.95E-05 | 3716/10000/5599/5605/5602/5579/1959/5582 | 8 |
| hsa04664 | Fc epsilon RI signaling pathway | 6/55 | 68/7441 | 9.49E-06 | 7.84E-05 | 2.95E-05 | 695/6850/10000/5599/5605/5602 | 6 |
| hsa01521 | EGFR tyrosine kinase inhibitor resistance | 6/55 | 79/7441 | 2.27E-05 | 0.000178 | 6.68E-05 | 3716/10000/5605/5579/2261/5582 | 6 |
| hsa04012 | ErbB signaling pathway | 6/55 | 85/7441 | 3.44E-05 | 0.000258 | 9.67E-05 | 10000/5599/5605/5602/5579/5582 | 6 |
| hsa04380 | Osteoclast differentiation | 7/55 | 128/7441 | 3.86E-05 | 0.000276 | 0.000104 | 3716/695/6850/10000/5599/5602/7006 | 7 |
| hsa04014 | Ras signaling pathway | 9/55 | 232/7441 | 4.34E-05 | 0.000296 | 0.000111 | 7535/2322/10000/5599/5605/5602/5579/2261/5582 | 9 |
| hsa04010 | MAPK signaling pathway | 10/55 | 295/7441 | 4.93E-05 | 0.000323 | 0.000121 | 2322/10000/5599/5605/5602/5579/2261/51135/4296/5582 | 10 |
| hsa04658 | Th1 and Th2 cell differentiation | 6/55 | 92/7441 | 5.40E-05 | 0.000339 | 0.000127 | 3718/3716/7535/5599/5602/5588 | 6 |
| hsa05231 | Choline metabolism in cancer | 6/55 | 99/7441 | 8.15E-05 | 0.000492 | 0.000185 | 10000/5599/5605/5602/5579/5582 | 6 |
| hsa04660 | T cell receptor signaling pathway | 6/55 | 101/7441 | 9.12E-05 | 0.00053 | 0.000199 | 7535/3702/10000/5605/5588/7006 | 6 |
| hsa05169 | Epstein-Barr virus infection | 8/55 | 201/7441 | 0.000101 | 0.000566 | 0.000212 | 3718/3716/6850/2268/10000/5599/5602/8737 | 8 |
| hsa04620 | Toll-like receptor signaling pathway | 6/55 | 104/7441 | 0.000107 | 0.000566 | 0.000212 | 10000/5599/5605/5602/51135/8737 | 6 |
| hsa05230 | Central carbon metabolism in cancer | 5/55 | 65/7441 | 0.000108 | 0.000566 | 0.000212 | 2322/10000/5605/2261/5979 | 5 |
| hsa05223 | Non-small cell lung cancer | 5/55 | 66/7441 | 0.000116 | 0.000589 | 0.000221 | 3718/10000/5605/5579/5582 | 5 |
| hsa04659 | Th17 cell differentiation | 6/55 | 107/7441 | 0.000126 | 0.000617 | 0.000232 | 3718/3716/7535/5599/5602/5588 | 6 |
| hsa05170 | Human immunodeficiency virus 1 infection | 8/55 | 212/7441 | 0.000146 | 0.000696 | 0.000261 | 10000/5599/5605/5602/5579/51135/5582/8737 | 8 |
| hsa04662 | B cell receptor signaling pathway | 5/55 | 71/7441 | 0.000165 | 0.000761 | 0.000286 | 695/6850/10000/5605/5579 | 5 |
| hsa04071 | Sphingolipid signaling pathway | 6/55 | 118/7441 | 0.000215 | 0.000966 | 0.000363 | 10000/5599/5605/5602/5579/5582 | 6 |
| hsa05164 | Influenza A | 7/55 | 171/7441 | 0.00024 | 0.001046 | 0.000393 | 3716/10000/5599/5605/5602/5579/51135 | 7 |
| hsa05167 | Kaposi sarcoma-associated herpesvirus infection | 7/55 | 186/7441 | 0.000401 | 0.001674 | 0.000629 | 3716/6850/3055/10000/5599/5605/5602 | 7 |
| hsa05210 | Colorectal cancer | 5/55 | 86/7441 | 0.000405 | 0.001674 | 0.000629 | 10000/5599/5605/6932/5602 | 5 |
| hsa04666 | Fc gamma R-mediated phagocytosis | 5/55 | 91/7441 | 0.000526 | 0.002118 | 0.000795 | 6850/3055/10000/5579/5582 | 5 |
| hsa04750 | Inflammatory mediator regulation of TRP channels | 5/55 | 99/7441 | 0.000774 | 0.003037 | 0.00114 | 5599/5602/5579/5588/5582 | 5 |
| hsa04370 | VEGF signaling pathway | 4/55 | 59/7441 | 0.0009 | 0.003445 | 0.001293 | 10000/5605/5579/5582 | 4 |
| hsa04625 | C-type lectin receptor signaling pathway | 5/55 | 104/7441 | 0.000968 | 0.003617 | 0.001358 | 6850/10000/5599/5602/1959 | 5 |
| hsa04931 | Insulin resistance | 5/55 | 107/7441 | 0.0011 | 0.004016 | 0.001508 | 10000/5599/5602/5579/5588 | 5 |
| hsa05145 | Toxoplasmosis | 5/55 | 113/7441 | 0.001404 | 0.004903 | 0.001841 | 3716/10000/5599/5602/51135 | 5 |
| hsa04621 | NOD-like receptor signaling pathway | 6/55 | 168/7441 | 0.001405 | 0.004903 | 0.001841 | 3716/5599/5602/51135/8767/8737 | 6 |
| hsa04919 | Thyroid hormone signaling pathway | 5/55 | 116/7441 | 0.001578 | 0.005386 | 0.002022 | 54361/10000/5605/5579/5582 | 5 |
| hsa04920 | Adipocytokine signaling pathway | 4/55 | 69/7441 | 0.001618 | 0.005405 | 0.002029 | 10000/5599/5602/5588 | 4 |
| hsa04917 | Prolactin signaling pathway | 4/55 | 70/7441 | 0.001707 | 0.005582 | 0.002096 | 10000/5599/5605/5602 | 4 |
| hsa05214 | Glioma | 4/55 | 71/7441 | 0.001799 | 0.005763 | 0.002164 | 10000/5605/5579/5582 | 4 |
| hsa05140 | Leishmaniasis | 4/55 | 74/7441 | 0.002096 | 0.00658 | 0.00247 | 3716/5579/51135/3684 | 4 |
| hsa05212 | Pancreatic cancer | 4/55 | 75/7441 | 0.002202 | 0.006777 | 0.002545 | 3716/10000/5599/5602 | 4 |
| hsa05133 | Pertussis | 4/55 | 76/7441 | 0.002311 | 0.006978 | 0.00262 | 5599/5602/51135/3684 | 4 |
| hsa04140 | Autophagy - animal | 5/55 | 128/7441 | 0.002435 | 0.007095 | 0.002664 | 10000/5599/5605/5602/5588 | 5 |
| hsa05216 | Thyroid cancer | 3/55 | 37/7441 | 0.002486 | 0.007095 | 0.002664 | 5605/6932/5979 | 3 |
| hsa05340 | Primary immunodeficiency | 3/55 | 37/7441 | 0.002486 | 0.007095 | 0.002664 | 3718/695/7535 | 3 |
| hsa04062 | Chemokine signaling pathway | 6/55 | 189/7441 | 0.002556 | 0.007166 | 0.00269 | 3718/2268/3055/3702/10000/5579 | 6 |
| hsa04650 | Natural killer cell mediated cytotoxicity | 5/55 | 131/7441 | 0.002693 | 0.007167 | 0.002691 | 6850/7535/5605/5579/5582 | 5 |
| hsa04728 | Dopaminergic synapse | 5/55 | 131/7441 | 0.002693 | 0.007167 | 0.002691 | 10000/5599/5602/5579/5582 | 5 |
| hsa05160 | Hepatitis C | 5/55 | 131/7441 | 0.002693 | 0.007167 | 0.002691 | 3716/10000/5599/5602/8737 | 5 |
| hsa05162 | Measles | 5/55 | 132/7441 | 0.002784 | 0.007284 | 0.002735 | 3718/3716/10000/51135/5588 | 5 |
| hsa04210 | Apoptosis | 5/55 | 136/7441 | 0.003168 | 0.008153 | 0.003061 | 10000/5599/5605/5602/8737 | 5 |
| hsa04015 | Rap1 signaling pathway | 6/55 | 206/7441 | 0.003919 | 0.009925 | 0.003726 | 10000/5605/5579/2261/3684/5582 | 6 |
| hsa04912 | GnRH signaling pathway | 4/55 | 93/7441 | 0.004795 | 0.011949 | 0.004486 | 5599/5605/5602/5579 | 4 |
| hsa04340 | Hedgehog signaling pathway | 3/55 | 47/7441 | 0.004924 | 0.012079 | 0.004535 | 2736/5727/3549 | 3 |
| hsa01522 | Endocrine resistance | 4/55 | 98/7441 | 0.005773 | 0.013945 | 0.005236 | 10000/5599/5605/5602 | 4 |
| hsa04933 | AGE-RAGE signaling pathway in diabetic complications | 4/55 | 99/7441 | 0.005984 | 0.014096 | 0.005293 | 10000/5599/5602/5579 | 4 |
| hsa05163 | Human cytomegalovirus infection | 6/55 | 225/7441 | 0.006016 | 0.014096 | 0.005293 | 3716/10000/5605/5579/5582/8737 | 6 |
| hsa04066 | HIF-1 signaling pathway | 4/55 | 100/7441 | 0.006199 | 0.014313 | 0.005374 | 10000/5605/5579/5582 | 4 |
| hsa05142 | Chagas disease (American trypanosomiasis) | 4/55 | 102/7441 | 0.006646 | 0.014922 | 0.005603 | 10000/5599/5602/51135 | 4 |
| hsa04217 | Necroptosis | 5/55 | 162/7441 | 0.006653 | 0.014922 | 0.005603 | 3718/3716/5599/5602/8737 | 5 |
| hsa04668 | TNF signaling pathway | 4/55 | 110/7441 | 0.008645 | 0.019117 | 0.007178 | 10000/5599/5602/8737 | 4 |
| hsa05213 | Endometrial cancer | 3/55 | 58/7441 | 0.00885 | 0.019298 | 0.007246 | 10000/5605/6932 | 3 |
| hsa04670 | Leukocyte transendothelial migration | 4/55 | 112/7441 | 0.0092 | 0.019787 | 0.007429 | 3702/5579/3684/5582 | 4 |
| hsa04730 | Long-term depression | 3/55 | 60/7441 | 0.009714 | 0.020609 | 0.007738 | 5605/5579/5582 | 3 |
| hsa04270 | Vascular smooth muscle contraction | 4/55 | 121/7441 | 0.011986 | 0.024959 | 0.009371 | 5605/5579/5588/5582 | 4 |
| hsa05131 | Shigellosis | 3/55 | 65/7441 | 0.012082 | 0.024959 | 0.009371 | 5599/5602/8767 | 3 |
| hsa04720 | Long-term potentiation | 3/55 | 67/7441 | 0.013115 | 0.02674 | 0.01004 | 5605/5579/5582 | 3 |
| hsa04151 | PI3K-Akt signaling pathway | 7/55 | 354/7441 | 0.014738 | 0.029324 | 0.01101 | 3718/3716/6850/2322/10000/5605/2261 | 7 |
| hsa04622 | RIG-I-like receptor signaling pathway | 3/55 | 70/7441 | 0.014755 | 0.029324 | 0.01101 | 5599/5602/8737 | 3 |
| hsa04024 | cAMP signaling pathway | 5/55 | 198/7441 | 0.01503 | 0.029355 | 0.011022 | 10000/5599/5605/5602/5727 | 5 |
| hsa04926 | Relaxin signaling pathway | 4/55 | 130/7441 | 0.015265 | 0.029355 | 0.011022 | 10000/5599/5605/5602 | 4 |
| hsa04510 | Focal adhesion | 5/55 | 199/7441 | 0.015332 | 0.029355 | 0.011022 | 10000/5599/5602/5579/5582 | 5 |
| hsa04068 | FoxO signaling pathway | 4/55 | 132/7441 | 0.016063 | 0.030385 | 0.011408 | 10000/5599/5605/5602 | 4 |
| hsa04910 | Insulin signaling pathway | 4/55 | 137/7441 | 0.018173 | 0.033967 | 0.012753 | 10000/5599/5605/5602 | 4 |
| hsa05206 | MicroRNAs in cancer | 6/55 | 299/7441 | 0.022215 | 0.041033 | 0.015406 | 7473/89780/5605/5579/2261/5582 | 6 |
| hsa04723 | Retrograde endocannabinoid signaling | 4/55 | 148/7441 | 0.023404 | 0.042725 | 0.016042 | 5599/5602/5579/5582 | 4 |
| hsa04932 | Non-alcoholic fatty liver disease (NAFLD) | 4/55 | 149/7441 | 0.02392 | 0.043166 | 0.016207 | 10000/5599/5602/4296 | 4 |
| hsa04215 | Apoptosis - multiple species | 2/55 | 33/7441 | 0.024467 | 0.043651 | 0.016389 | 5599/5602 | 2 |
| hsa04540 | Gap junction | 3/55 | 88/7441 | 0.02696 | 0.047559 | 0.017856 | 5605/5579/5582 | 3 |
| hsa05143 | African trypanosomiasis | 2/55 | 35/7441 | 0.027314 | 0.047648 | 0.01789 | 5579/5582 | 2 |
