# Supplementary material for: Use of Laplacian Heat Diffusion Algorithm to Infer Novel Genes With Functions Related to Uveitis
Source: Front Genet. 2018 Oct 8;9:425. doi: 10.3389/fgene.2018.00425 (PMC6186792; doi:10.3389/fgene.2018.00425)
Supplement: Supplementary file 5 [file Table_5.DOCX]

Supplementary Material

Use of Laplacian heat diffusion algorithm to infer novel genes with functions related to uveitis

Shiheng Lu, Ke Zhao, Xuefei Wang, Hui Liu, Xiamuxiya Ainiwaer, Yan Xu, Min Ye*

*** Correspondence:** Min Ye: gleye@163.com

**Supplementary Table 5.** 59 inferred uveitis-related genes searched for literature support and the corresponding references.

| No. | Gene/Features | Reference |
| --- | --- | --- |
| 1 | JAK3 | (Liao et al., 2018) |
| 2 | JAK1 | (Liao et al., 2018) |
| 3 | WNT16 | (Reischl et al., 2007;Nalesso et al., 2017) |
| 4 | WNT3 | (Nakatsu et al., 2011) |
| 5 | WNT7B | (Nakatsu et al., 2011) |
| 6 | WNT7A | (Reischl et al., 2007;Nalesso et al., 2017) |
| 7 | WNT2B | (Reischl et al., 2007;Nalesso et al., 2017) |
| 8 | WNT2 | (Reischl et al., 2007;Nalesso et al., 2017) |
| 9 | WNT9A | (Nakatsu et al., 2011) |
| 10 | BTK | (Vargas et al., 2013;Corneth et al., 2016) |
| 11 | SYK | (Lee et al., 2016;Hagan et al., 2018) |
| 12 | WNT4 | (Reischl et al., 2007;Nalesso et al., 2017) |
| 13 | GLI2 | (Takezaki et al., 2011;Swiderska-Syn et al., 2013) |
| 14 | ZAP70 | (Kleinwort et al., 2016) |
| 15 | FGR | (Wang et al., 2008) |
| 16 | WNT11 | (Reischl et al., 2007;Nalesso et al., 2017) |
| 17 | WNT3A | (Reischl et al., 2007;Nalesso et al., 2017) |
| 18 | HCK | (Ernst et al., 2002) |
| 19 | WNT9B | (Reischl et al., 2007;Nalesso et al., 2017) |
| 20 | FLT3 | (Tang et al., 2007) |
| 21 | WNT1 | (Reischl et al., 2007;Nalesso et al., 2017) |
| 22 | ITK | (Bach et al., 2014) |
| 23 | AKT3 | (Hu et al., 2013) |
| 24 | MAPK8 | (Chen et al., 2015) |
| 25 | MAP2K2 | (Chen et al., 2015) |
| 26 | WNT10A | (Nakatsu et al., 2011) |
| 27 | WNT6 | (Nakatsu et al., 2011) |
| 28 | TCF7 | (Katoh, 2018) |
| 29 | PIM2 | (Du et al., 2017;Jimenez-Garcia et al., 2017) |
| 30 | WNT8A | (Reischl et al., 2007;Nalesso et al., 2017) |
| 31 | MAPK10 | (Reischl et al., 2007;Nalesso et al., 2017) |
| 32 | RUNX2 | (Balogh et al., 2016) |
| 33 | PTCH1 | (Braune et al., 2017) |
| 34 | PRKCB | (Patergnani et al., 2013) |
| 35 | PTK6 | (Levesque et al., 2017) |
| 36 | WNT10B | (Reischl et al., 2007;Nalesso et al., 2017) |
| 37 | SMAD5 | (Zhang et al., 2015) |
| 38 | FGFR3 | (Foth et al., 2018) |
| 39 | BLK | (Rangarajan et al., 2016) |
| 40 | EGR2 | (Li et al., 2012) |
| 41 | SMAD7 | (Zhang et al., 2015) |
| 42 | SFRP2 | (Eymard et al., 2017) |
| 43 | IRAK4 | (Chaudhary et al., 2015) |
| 44 | RIPK2 | (Tigno-Aranjuez et al., 2014) |
| 45 | MAP3K11 | (Duran Aguilar et al., 2017) |
| 46 | WNT8B | (Reischl et al., 2007;Nalesso et al., 2017) |
| 47 | BMX | (Stover et al., 2016) |
| 48 | RET | (Modrzejewska et al., 2016) |
| 49 | IHH | (Aschermann et al., 2016) |
| 50 | PRKCQ | (Chen et al., 2016) |
| 51 | ITGAM | (Macey et al., 2011) |
| 52 | TEC | (Manandhar et al., 2008) |
| 53 | PRKCG | (Lu et al., 2015) |
| 54 | SFRP1 | (Matzelle et al., 2012) |
| 55 | IRAK2 | (Chaudhary et al., 2015) |
| 56 | SOX2 | (Liu et al., 2013) |
| 57 | PRKX | (Ouyang et al., 2014) |
| 58 | RIPK1 | (Tigno-Aranjuez et al., 2014) |
| 59 | IRAK3 | (Chaudhary et al., 2015) |

**References**

Aschermann, S., Englbrecht, M., Bergua, A., Spriewald, B.M., Said-Nahal, R., Breban, M., Schett, G., and Rech, J. (2016). Presence of HLA-B27 is associated with changes of serum levels of mediators of the Wnt and hedgehog pathway. *Joint Bone Spine* 83**,** 43-46.

Bach, M.P., Hug, E., Werner, M., Holch, J., Sprissler, C., Pechloff, K., Zirlik, K., Zeiser, R., Dierks, C., Ruland, J., and Jumaa, H. (2014). Premature terminal differentiation protects from deregulated lymphocyte activation by ITK-Syk. *J Immunol* 192**,** 1024-1033.

Balogh, E., Toth, A., Tolnai, E., Bodo, T., Banyai, E., Szabo, D.J., Petrovski, G., and Jeney, V. (2016). Osteogenic differentiation of human lens epithelial cells might contribute to lens calcification. *Biochim Biophys Acta* 1862**,** 1724-1731.

Braune, J., Weyer, U., Matz-Soja, M., Hobusch, C., Kern, M., Kunath, A., Kloting, N., Kralisch, S., Bluher, M., Gebhardt, R., Zavros, Y., Bechmann, I., and Gericke, M. (2017). Hedgehog signalling in myeloid cells impacts on body weight, adipose tissue inflammation and glucose metabolism. *Diabetologia* 60**,** 889-899.

Chaudhary, D., Robinson, S., and Romero, D.L. (2015). Recent advances in the discovery of small molecule inhibitors of interleukin-1 receptor-associated kinase 4 (IRAK4) as a therapeutic target for inflammation and oncology disorders. *J Med Chem* 58**,** 96-110.

Chen, F., Xu, L., Zhao, T., Xiao, X., Pan, Y., and Hou, S. (2016). Genetic Variation in the REL Gene Increases Risk of Behcet's Disease in a Chinese Han Population but That of PRKCQ Does Not. *PLoS One* 11**,** e0147350.

Chen, P., Denniston, A., Hannes, S., Tucker, W., Wei, L., Liu, B., Xiao, T., Hirani, S., Li, Z., Jawad, S., Si, H., Lee, R.W., Sen, H.N., and Nussenblatt, R.B. (2015). Increased CD1c+ mDC1 with mature phenotype regulated by TNFalpha-p38 MAPK in autoimmune ocular inflammatory disease. *Clin Immunol* 158**,** 35-46.

Corneth, O.B.J., Klein Wolterink, R.G.J., and Hendriks, R.W. (2016). BTK Signaling in B Cell Differentiation and Autoimmunity. *Curr Top Microbiol Immunol* 393**,** 67-105.

Du, W., Chen, T., Ni, Y., Hou, X., Yu, Y., Zhou, Q., Wu, F., Tang, W., and Shi, G. (2017). Role of PIM2 in allergic asthma. *Mol Med Rep* 16**,** 7504-7512.

Duran Aguilar, M., Roman Ponce, S.I., Ruiz Lopez, F.J., Gonzalez Padilla, E., Vasquez Pelaez, C.G., Bagnato, A., and Strillacci, M.G. (2017). Genome-wide association study for milk somatic cell score in holstein cattle using copy number variation as markers. *J Anim Breed Genet* 134**,** 49-59.

Ernst, M., Inglese, M., Scholz, G.M., Harder, K.W., Clay, F.J., Bozinovski, S., Waring, P., Darwiche, R., Kay, T., Sly, P., Collins, R., Turner, D., Hibbs, M.L., Anderson, G.P., and Dunn, A.R. (2002). Constitutive activation of the SRC family kinase Hck results in spontaneous pulmonary inflammation and an enhanced innate immune response. *J Exp Med* 196**,** 589-604.

Eymard, F., Pigenet, A., Citadelle, D., Tordjman, J., Foucher, L., Rose, C., Flouzat Lachaniette, C.H., Rouault, C., Clement, K., Berenbaum, F., Chevalier, X., and Houard, X. (2017). Knee and hip intra-articular adipose tissues (IAATs) compared with autologous subcutaneous adipose tissue: a specific phenotype for a central player in osteoarthritis. *Ann Rheum Dis* 76**,** 1142-1148.

Foth, M., Ismail, N.F.B., Kung, J.S.C., Tomlinson, D., Knowles, M.A., Eriksson, P., Sjodahl, G., Salmond, J.M., Sansom, O.J., and Iwata, T. (2018). FGFR3 mutation increases bladder tumorigenesis by suppressing acute inflammation. *J Pathol*.

Hagan, S., Fyfe, M.C.T., Ofori-Frimpong, B., Oliver, K., Foster, M.R., Sirohi, S., Solanke, Y., Doughty, M., Rowley, A., Taylor, M., Webber, S., and Walshe, C.A. (2018). Narrow Spectrum Kinase Inhibitors Demonstrate Promise for the Treatment of Dry Eye Disease and Other Ocular Inflammatory Disorders. *Invest Ophthalmol Vis Sci* 59**,** 1443-1453.

Hu, X.F., Lu, H., Wang, J., Zhang, X.S., Zhang, X.L., Liu, X.H., Xu, Z.Z., Hu, J.M., and Lu, Q.J. (2013). [Screening of key genes and inflammatory signalling pathway involved in the pathogenesis of HLA-B27-associated acute anterior uveitis by gene expression microarray]. *Zhonghua Yan Ke Za Zhi* 49**,** 217-223.

Jimenez-Garcia, M.P., Lucena-Cacace, A., Robles-Frias, M.J., Ferrer, I., Narlik-Grassow, M., Blanco-Aparicio, C., and Carnero, A. (2017). Inflammation and stem markers association to PIM1/PIM2 kinase-induced tumors in breast and uterus. *Oncotarget* 8**,** 58872-58886.

Katoh, M. (2018). Multilayered prevention and treatment of chronic inflammation, organ fibrosis and cancer associated with canonical WNT/betacatenin signaling activation (Review). *Int J Mol Med* 42**,** 713-725.

Kleinwort, K.J., Amann, B., Hauck, S.M., Feederle, R., Sekundo, W., and Deeg, C.A. (2016). Immunological Characterization of Intraocular Lymphoid Follicles in a Spontaneous Recurrent Uveitis Model. *Invest Ophthalmol Vis Sci* 57**,** 4504-4511.

Lee, E.J., Brown, B.R., Vance, E.E., Snow, P.E., Silver, P.B., Heinrichs, D., Lin, X., Iwakura, Y., Wells, C.A., Caspi, R.R., and Rosenzweig, H.L. (2016). Mincle Activation and the Syk/Card9 Signaling Axis Are Central to the Development of Autoimmune Disease of the Eye. *J Immunol* 196**,** 3148-3158.

Levesque, N., Christensen, K.E., Van Der Kraak, L., Best, A.F., Deng, L., Caldwell, D., Macfarlane, A.J., Beauchemin, N., and Rozen, R. (2017). Murine MTHFD1-synthetase deficiency, a model for the human MTHFD1 R653Q polymorphism, decreases growth of colorectal tumors. *Mol Carcinog* 56**,** 1030-1040.

Li, S., Miao, T., Sebastian, M., Bhullar, P., Ghaffari, E., Liu, M., Symonds, A.L., and Wang, P. (2012). The transcription factors Egr2 and Egr3 are essential for the control of inflammation and antigen-induced proliferation of B and T cells. *Immunity* 37**,** 685-696.

Liao, H.T., Li, T.H., Chen, C.H., Chen, H.A., Chen, W.S., Lai, C.C., Chou, C.T., and Tsai, C.Y. (2018). Janus kinase-1 and 3 in ankylosing spondylitis. *J Formos Med Assoc*.

Liu, K., Jiang, M., Lu, Y., Chen, H., Sun, J., Wu, S., Ku, W.Y., Nakagawa, H., Kita, Y., Natsugoe, S., Peters, J.H., Rustgi, A., Onaitis, M.W., Kiernan, A., Chen, X., and Que, J. (2013). Sox2 cooperates with inflammation-mediated Stat3 activation in the malignant transformation of foregut basal progenitor cells. *Cell Stem Cell* 12**,** 304-315.

Lu, H., Zhu, L., Lian, L., Chen, M., Shi, D., and Wang, K. (2015). Genetic variations in the PRKCG gene and osteosarcoma risk in a Chinese population: a case-control study. *Tumour Biol* 36**,** 5241-5247.

Macey, M., Hagi-Pavli, E., Stewart, J., Wallace, G.R., Stanford, M., Shirlaw, P., and Fortune, F. (2011). Age, gender and disease-related platelet and neutrophil activation ex vivo in whole blood samples from patients with Behcet's disease. *Rheumatology (Oxford)* 50**,** 1849-1859.

Manandhar, A., Paudel, G., Rai, C.K., Rai, S.K., Gurung, R., and Ruit, S. (2008). Seasonal hyper acute pan uveitis--recent scenario in Nepal. *Nepal Med Coll J* 10**,** 196-198.

Matzelle, M.M., Gallant, M.A., Condon, K.W., Walsh, N.C., Manning, C.A., Stein, G.S., Lian, J.B., Burr, D.B., and Gravallese, E.M. (2012). Resolution of inflammation induces osteoblast function and regulates the Wnt signaling pathway. *Arthritis Rheum* 64**,** 1540-1550.

Modrzejewska, M., Patalan, J., Kulik, U., and Czeszynska, M.B. (2016). Ocular manifestation of congenital toxoplasmosis, clinical implication - case report. *Ginekol Pol* 87**,** 226-230.

Nakatsu, M.N., Ding, Z., Ng, M.Y., Truong, T.T., Yu, F., and Deng, S.X. (2011). Wnt/beta-catenin signaling regulates proliferation of human cornea epithelial stem/progenitor cells. *Invest Ophthalmol Vis Sci* 52**,** 4734-4741.

Nalesso, G., Thomas, B.L., Sherwood, J.C., Yu, J., Addimanda, O., Eldridge, S.E., Thorup, A.S., Dale, L., Schett, G., Zwerina, J., Eltawil, N., Pitzalis, C., and Dell'accio, F. (2017). WNT16 antagonises excessive canonical WNT activation and protects cartilage in osteoarthritis. *Ann Rheum Dis* 76**,** 218-226.

Ouyang, C., Nie, L., Gu, M., Wu, A., Han, X., Wang, X., Shao, J., and Xia, Z. (2014). Transforming growth factor (TGF)-beta-activated kinase 1 (TAK1) activation requires phosphorylation of serine 412 by protein kinase A catalytic subunit alpha (PKACalpha) and X-linked protein kinase (PRKX). *J Biol Chem* 289**,** 24226-24237.

Patergnani, S., Marchi, S., Rimessi, A., Bonora, M., Giorgi, C., Mehta, K.D., and Pinton, P. (2013). PRKCB/protein kinase C, beta and the mitochondrial axis as key regulators of autophagy. *Autophagy* 9**,** 1367-1385.

Rangarajan, P., Karthikeyan, A., and Dheen, S.T. (2016). Role of dietary phenols in mitigating microglia-mediated neuroinflammation. *Neuromolecular Med* 18**,** 453-464.

Reischl, J., Schwenke, S., Beekman, J.M., Mrowietz, U., Sturzebecher, S., and Heubach, J.F. (2007). Increased expression of Wnt5a in psoriatic plaques. *J Invest Dermatol* 127**,** 163-169.

Stover, K., Fukuyama, T., Young, A.T., Daniele, M.A., Oberley, R., Crapo, J.D., and Baumer, W. (2016). Topically applied manganese-porphyrins BMX-001 and BMX-010 display a significant anti-inflammatory response in a mouse model of allergic dermatitis. *Arch Dermatol Res* 308**,** 711-721.

Swiderska-Syn, M., Suzuki, A., Guy, C.D., Schwimmer, J.B., Abdelmalek, M.F., Lavine, J.E., and Diehl, A.M. (2013). Hedgehog pathway and pediatric nonalcoholic fatty liver disease. *Hepatology* 57**,** 1814-1825.

Takezaki, T., Hide, T., Takanaga, H., Nakamura, H., Kuratsu, J., and Kondo, T. (2011). Essential role of the Hedgehog signaling pathway in human glioma-initiating cells. *Cancer Sci* 102**,** 1306-1312.

Tang, J., Zhu, W., Silver, P.B., Su, S.B., Chan, C.C., and Caspi, R.R. (2007). Autoimmune uveitis elicited with antigen-pulsed dendritic cells has a distinct clinical signature and is driven by unique effector mechanisms: initial encounter with autoantigen defines disease phenotype. *J Immunol* 178**,** 5578-5587.

Tigno-Aranjuez, J.T., Benderitter, P., Rombouts, F., Deroose, F., Bai, X., Mattioli, B., Cominelli, F., Pizarro, T.T., Hoflack, J., and Abbott, D.W. (2014). In vivo inhibition of RIPK2 kinase alleviates inflammatory disease. *J Biol Chem* 289**,** 29651-29664.

Vargas, L., Hamasy, A., Nore, B.F., and Smith, C.I. (2013). Inhibitors of BTK and ITK: state of the new drugs for cancer, autoimmunity and inflammatory diseases. *Scand J Immunol* 78**,** 130-139.

Wang, J., Ohno-Matsui, K., Yoshida, T., Kojima, A., Shimada, N., Nakahama, K., Safranova, O., Iwata, N., Saido, T.C., Mochizuki, M., and Morita, I. (2008). Altered function of factor I caused by amyloid beta: implication for pathogenesis of age-related macular degeneration from Drusen. *J Immunol* 181**,** 712-720.

Zhang, X., Ai, F., Li, X., She, X., Li, N., Tang, A., Qin, Z., Ye, Q., Tian, L., Li, G., Shen, S., and Ma, J. (2015). Inflammation-induced S100A8 activates Id3 and promotes colorectal tumorigenesis. *Int J Cancer* 137**,** 2803-2814.
