# Supplementary material for: Use of Laplacian Heat Diffusion Algorithm to Infer Novel Genes With Functions Related to Uveitis
Source: Front Genet. 2018 Oct 8;9:425. doi: 10.3389/fgene.2018.00425 (PMC6186792; doi:10.3389/fgene.2018.00425)
Supplement: Supplementary file 2 [file Table_2.DOCX]

Supplementary Material

Use of Laplacian heat diffusion algorithm to infer novel genes with functions related to uveitis

Shiheng Lu, Ke Zhao, Xuefei Wang, Hui Liu, Xiamuxiya Ainiwaer, Yan Xu, Min Ye*

*** Correspondence:** Min Ye: [gleye@163.com](mailto:gleye@163.com)

**Supplementary Table 2.**  Measurements of genes obtained by the constructed computational method.

| **Index** | **Ensembl ID** | **Gene symbol** | **Heat** | **Zscore** | **MIS** | **MFS** |
| --- | --- | --- | --- | --- | --- | --- |
| 1 | ENSP00000391676 | JAK3 | 8.595E-04 | 2.206 | 999 | 0.996 |
| 2 | ENSP00000343204 | JAK1 | 5.055E-04 | 2.999 | 999 | 0.995 |
| 3 | ENSP00000222462 | WNT16 | 2.024E-03 | 9.020 | 906 | 0.995 |
| 4 | ENSP00000225512 | WNT3 | 1.584E-03 | 9.033 | 904 | 0.995 |
| 5 | ENSP00000341032 | WNT7B | 1.572E-03 | 8.923 | 904 | 0.994 |
| 6 | ENSP00000285018 | WNT7A | 1.626E-03 | 8.312 | 904 | 0.994 |
| 7 | ENSP00000358698 | WNT2B | 1.648E-03 | 8.858 | 903 | 0.993 |
| 8 | ENSP00000265441 | WNT2 | 1.361E-03 | 8.989 | 903 | 0.992 |
| 9 | ENSP00000272164 | WNT9A | 1.874E-03 | 9.005 | 942 | 0.992 |
| 10 | ENSP00000308176 | BTK | 5.248E-04 | 3.065 | 966 | 0.992 |
| 11 | ENSP00000364898 | SYK | 5.178E-04 | 2.945 | 996 | 0.991 |
| 12 | ENSP00000290167 | WNT4 | 1.109E-03 | 7.898 | 904 | 0.991 |
| 13 | ENSP00000354586 | GLI2 | 7.961E-04 | 4.422 | 918 | 0.991 |
| 14 | ENSP00000264972 | ZAP70 | 5.018E-04 | 2.840 | 956 | 0.990 |
| 15 | ENSP00000363115 | FGR | 5.289E-04 | 4.080 | 989 | 0.989 |
| 16 | ENSP00000325526 | WNT11 | 1.751E-03 | 4.140 | 907 | 0.989 |
| 17 | ENSP00000284523 | WNT3A | 8.297E-04 | 4.090 | 904 | 0.989 |
| 18 | ENSP00000365012 | HCK | 5.670E-04 | 3.808 | 995 | 0.989 |
| 19 | ENSP00000290015 | WNT9B | 2.192E-03 | 9.010 | 941 | 0.989 |
| 20 | ENSP00000241453 | FLT3 | 4.511E-04 | 3.256 | 921 | 0.988 |
| 21 | ENSP00000293549 | WNT1 | 7.405E-04 | 8.481 | 905 | 0.987 |
| 22 | ENSP00000398655 | ITK | 8.471E-04 | 5.230 | 970 | 0.986 |
| 23 | ENSP00000263826 | AKT3 | 4.051E-04 | 3.710 | 965 | 0.986 |
| 24 | ENSP00000353483 | MAPK8 | 4.108E-03 | 6.884 | 993 | 0.985 |
| 25 | ENSP00000262948 | MAP2K2 | 5.814E-04 | 2.373 | 911 | 0.984 |
| 26 | ENSP00000258411 | WNT10A | 2.919E-03 | 9.060 | 907 | 0.984 |
| 27 | ENSP00000233948 | WNT6 | 2.900E-03 | 9.948 | 917 | 0.983 |
| 28 | ENSP00000340347 | TCF7 | 1.373E-03 | 3.798 | 910 | 0.983 |
| 29 | ENSP00000365692 | PIM2 | 6.035E-04 | 4.137 | 935 | 0.982 |
| 30 | ENSP00000381739 | WNT8A | 3.087E-03 | 8.986 | 925 | 0.982 |
| 31 | ENSP00000352157 | MAPK10 | 6.225E-03 | 5.002 | 945 | 0.982 |
| 32 | ENSP00000360493 | RUNX2 | 9.723E-04 | 7.403 | 960 | 0.982 |
| 33 | ENSP00000332353 | PTCH1 | 8.522E-04 | 4.300 | 999 | 0.982 |
| 34 | ENSP00000305355 | PRKCB | 4.369E-04 | 3.473 | 905 | 0.980 |
| 35 | ENSP00000217185 | PTK6 | 6.583E-04 | 3.067 | 947 | 0.979 |
| 36 | ENSP00000301061 | WNT10B | 2.325E-03 | 4.384 | 908 | 0.979 |
| 37 | ENSP00000441954 | SMAD5 | 1.136E-03 | 2.029 | 948 | 0.979 |
| 38 | ENSP00000339824 | FGFR3 | 4.337E-04 | 2.554 | 980 | 0.978 |
| 39 | ENSP00000259089 | BLK | 5.923E-04 | 3.072 | 983 | 0.978 |
| 40 | ENSP00000242480 | EGR2 | 6.515E-04 | 3.210 | 927 | 0.977 |
| 41 | ENSP00000262158 | SMAD7 | 1.643E-03 | 4.073 | 965 | 0.977 |
| 42 | ENSP00000274063 | SFRP2 | 2.385E-03 | 8.915 | 919 | 0.977 |
| 43 | ENSP00000390651 | IRAK4 | 8.263E-04 | 4.998 | 999 | 0.976 |
| 44 | ENSP00000220751 | RIPK2 | 5.553E-04 | 3.129 | 999 | 0.976 |
| 45 | ENSP00000309597 | MAP3K11 | 6.530E-04 | 2.864 | 903 | 0.976 |
| 46 | ENSP00000340677 | WNT8B | 3.032E-03 | 8.963 | 926 | 0.976 |
| 47 | ENSP00000308774 | BMX | 6.292E-04 | 3.000 | 979 | 0.976 |
| 48 | ENSP00000347942 | RET | 3.096E-04 | 2.276 | 992 | 0.975 |
| 49 | ENSP00000295731 | IHH | 7.545E-04 | 8.582 | 985 | 0.975 |
| 50 | ENSP00000263125 | PRKCQ | 4.867E-04 | 2.718 | 958 | 0.975 |
| 51 | ENSP00000441691 | ITGAM | 9.577E-04 | 9.044 | 999 | 0.974 |
| 52 | ENSP00000370912 | TEC | 7.230E-04 | 4.096 | 985 | 0.974 |
| 53 | ENSP00000263431 | PRKCG | 4.442E-04 | 3.485 | 903 | 0.974 |
| 54 | ENSP00000220772 | SFRP1 | 1.627E-03 | 8.964 | 927 | 0.973 |
| 55 | ENSP00000256458 | IRAK2 | 6.215E-04 | 2.997 | 990 | 0.972 |
| 56 | ENSP00000323588 | SOX2 | 7.613E-04 | 3.634 | 957 | 0.972 |
| 57 | ENSP00000262848 | PRKX | 3.771E-04 | 2.841 | 950 | 0.972 |
| 58 | ENSP00000259808 | RIPK1 | 6.588E-04 | 2.398 | 996 | 0.971 |
| 59 | ENSP00000261233 | IRAK3 | 6.082E-04 | 3.008 | 962 | 0.970 |
| 60 | ENSP00000355785 | LEFTY2 | 2.553E-03 | 8.865 | 918 | 0.969 |
| 61 | ENSP00000422753 | DDX41 | 7.724E-04 | 2.364 | 989 | 0.969 |
| 62 | ENSP00000262493 | GNAO1 | 5.782E-04 | 3.015 | 913 | 0.968 |
| 63 | ENSP00000366488 | PRKACG | 4.410E-04 | 2.410 | 915 | 0.967 |
| 64 | ENSP00000359719 | PRKACB | 4.040E-04 | 3.025 | 950 | 0.967 |
| 65 | ENSP00000401399 | MYD88 | 9.111E-04 | 5.602 | 999 | 0.967 |
| 66 | ENSP00000309591 | PRKACA | 3.536E-04 | 2.593 | 950 | 0.967 |
| 67 | ENSP00000287934 | FZD1 | 2.467E-03 | 4.212 | 978 | 0.966 |
| 68 | ENSP00000360020 | DIRAS3 | 4.459E-04 | 3.294 | 927 | 0.966 |
| 69 | ENSP00000390500 | STK3 | 7.724E-04 | 3.166 | 991 | 0.963 |
| 70 | ENSP00000326432 | CCR8 | 1.438E-04 | 4.420 | 951 | 0.961 |
| 71 | ENSP00000263339 | IL1A | 1.930E-04 | 4.277 | 992 | 0.961 |
| 72 | ENSP00000361266 | PTCH2 | 1.936E-03 | 8.787 | 979 | 0.959 |
| 73 | ENSP00000272519 | RALB | 4.224E-04 | 2.676 | 934 | 0.958 |
| 74 | ENSP00000327025 | RORC | 2.797E-04 | 2.797 | 975 | 0.957 |
| 75 | ENSP00000303507 | BCR | 7.048E-04 | 3.618 | 983 | 0.957 |
| 76 | ENSP00000352121 | PIK3CG | 5.742E-04 | 4.906 | 915 | 0.957 |
| 77 | ENSP00000225474 | CSF3 | 1.389E-04 | 5.865 | 960 | 0.957 |
| 78 | ENSP00000347979 | FAS | 1.275E-03 | 3.769 | 919 | 0.955 |
| 79 | ENSP00000304414 | CXCR6 | 1.134E-04 | 4.797 | 949 | 0.955 |
| 80 | ENSP00000398632 | CD44 | 9.952E-04 | 2.166 | 998 | 0.954 |
| 81 | ENSP00000319635 | CXCR2 | 1.768E-04 | 5.496 | 999 | 0.954 |
| 82 | ENSP00000380066 | MAP4K1 | 9.940E-04 | 7.917 | 900 | 0.954 |
| 83 | ENSP00000379110 | CXCL1 | 2.699E-04 | 2.756 | 971 | 0.950 |
| 84 | ENSP00000379330 | NFATC2 | 2.751E-03 | 9.181 | 999 | 0.950 |
| 85 | ENSP00000329357 | SP1 | 6.840E-04 | 3.138 | 988 | 0.950 |
| 86 | ENSP00000268296 | ITGAX | 1.125E-03 | 4.655 | 988 | 0.950 |
| 87 | ENSP00000221452 | RELB | 2.745E-03 | 4.546 | 982 | 0.949 |
| 88 | ENSP00000230990 | HBEGF | 1.896E-03 | 9.274 | 947 | 0.949 |
| 89 | ENSP00000243786 | INHA | 3.339E-03 | 9.092 | 922 | 0.949 |
| 90 | ENSP00000239849 | TNFSF11 | 1.723E-03 | 9.284 | 968 | 0.949 |
| 91 | ENSP00000368169 | DVL1 | 7.461E-04 | 6.992 | 908 | 0.948 |
| 92 | ENSP00000260227 | MMP7 | 2.079E-03 | 9.220 | 994 | 0.948 |
| 93 | ENSP00000228280 | KITLG | 1.822E-03 | 4.410 | 938 | 0.947 |
| 94 | ENSP00000172229 | NGFR | 1.209E-03 | 4.357 | 933 | 0.945 |
| 95 | ENSP00000306884 | CXCL11 | 1.707E-04 | 6.492 | 999 | 0.944 |
| 96 | ENSP00000259607 | CCL21 | 1.167E-04 | 4.385 | 999 | 0.944 |
| 97 | ENSP00000295683 | CXCR1 | 1.755E-04 | 7.327 | 999 | 0.944 |
| 98 | ENSP00000376943 | BCL2L11 | 2.193E-03 | 8.278 | 993 | 0.943 |
| 99 | ENSP00000216341 | GZMB | 1.388E-03 | 9.576 | 996 | 0.943 |
| 100 | ENSP00000245414 | IRF1 | 1.457E-03 | 4.158 | 996 | 0.943 |
| 101 | ENSP00000216223 | IL2RB | 2.214E-03 | 4.731 | 999 | 0.943 |
| 102 | ENSP00000409346 | CISH | 1.085E-03 | 7.571 | 978 | 0.941 |
| 103 | ENSP00000370343 | IRF4 | 2.251E-04 | 2.853 | 920 | 0.941 |
| 104 | ENSP00000261349 | LRP6 | 2.964E-03 | 3.790 | 967 | 0.941 |
| 105 | ENSP00000164227 | BCL3 | 1.109E-03 | 4.389 | 951 | 0.938 |
| 106 | ENSP00000358327 | CASP7 | 2.125E-03 | 8.571 | 933 | 0.938 |
| 107 | ENSP00000362994 | TRAF1 | 2.446E-03 | 8.815 | 998 | 0.938 |
| 108 | ENSP00000261405 | VWF | 8.297E-04 | 3.743 | 980 | 0.937 |
| 109 | ENSP00000327850 | NFATC1 | 3.547E-03 | 9.137 | 987 | 0.937 |
| 110 | ENSP00000430684 | IKBKB | 9.681E-04 | 1.977 | 984 | 0.936 |
| 111 | ENSP00000375086 | CCL25 | 1.641E-04 | 5.388 | 983 | 0.936 |
| 112 | ENSP00000337432 | IL17F | 3.103E-04 | 7.891 | 947 | 0.935 |
| 113 | ENSP00000204615 | THPO | 1.179E-04 | 4.622 | 990 | 0.935 |
| 114 | ENSP00000360683 | PTPN1 | 1.184E-03 | 5.400 | 999 | 0.935 |
| 115 | ENSP00000403103 | IL12RB1 | 2.731E-04 | 4.766 | 990 | 0.934 |
| 116 | ENSP00000358525 | NGF | 1.200E-03 | 7.417 | 930 | 0.933 |
| 117 | ENSP00000226317 | CXCL6 | 1.878E-04 | 7.425 | 944 | 0.933 |
| 118 | ENSP00000354416 | CCL28 | 1.242E-04 | 4.450 | 963 | 0.932 |
| 119 | ENSP00000231454 | IL5 | 3.521E-03 | 9.546 | 991 | 0.931 |
| 120 | ENSP00000308165 | CD36 | 9.338E-04 | 8.227 | 929 | 0.929 |
| 121 | ENSP00000310127 | IRF3 | 1.998E-03 | 3.950 | 985 | 0.929 |
| 122 | ENSP00000222390 | HGF | 3.192E-03 | 9.237 | 925 | 0.928 |
| 123 | ENSP00000369293 | IL2RA | 2.387E-03 | 9.654 | 999 | 0.927 |
| 124 | ENSP00000427279 | CXCL2 | 1.897E-04 | 4.040 | 964 | 0.927 |
| 125 | ENSP00000358983 | NFKB2 | 1.004E-03 | 3.208 | 974 | 0.926 |
| 126 | ENSP00000289153 | PIK3CB | 7.008E-04 | 3.242 | 908 | 0.925 |
| 127 | ENSP00000330237 | CASP9 | 2.552E-03 | 4.264 | 999 | 0.925 |
| 128 | ENSP00000437940 | CD19 | 2.131E-04 | 2.388 | 909 | 0.925 |
| 129 | ENSP00000323901 | FZD2 | 3.563E-03 | 4.368 | 976 | 0.925 |
| 130 | ENSP00000357255 | BGLAP | 1.386E-03 | 8.371 | 909 | 0.925 |
| 131 | ENSP00000363318 | IL2RG | 3.289E-03 | 9.951 | 999 | 0.924 |
| 132 | ENSP00000264033 | CBL | 1.327E-03 | 3.948 | 999 | 0.923 |
| 133 | ENSP00000434034 | FZD4 | 1.219E-03 | 8.284 | 957 | 0.923 |
| 134 | ENSP00000247879 | TAS2R3 | 2.055E-04 | 6.727 | 900 | 0.923 |
| 135 | ENSP00000296027 | CXCL5 | 1.863E-04 | 9.500 | 971 | 0.922 |
| 136 | ENSP00000452780 | B2M | 4.168E-04 | 3.310 | 999 | 0.921 |
| 137 | ENSP00000215631 | GADD45B | 4.356E-03 | 4.581 | 927 | 0.921 |
| 138 | ENSP00000405095 | TAS2R39 | 8.877E-05 | 2.594 | 900 | 0.921 |
| 139 | ENSP00000340820 | MAPT | 2.263E-03 | 2.456 | 963 | 0.920 |
| 140 | ENSP00000249373 | SMO | 3.869E-03 | 9.050 | 992 | 0.920 |
| 141 | ENSP00000366563 | PIK3CD | 8.013E-04 | 4.771 | 912 | 0.919 |
| 142 | ENSP00000431719 | TAS2R43 | 8.903E-05 | 3.881 | 900 | 0.919 |
| 143 | ENSP00000247883 | TAS2R5 | 1.911E-04 | 2.624 | 900 | 0.918 |
| 144 | ENSP00000351671 | CCL20 | 2.510E-04 | 6.898 | 992 | 0.918 |
| 145 | ENSP00000376500 | TRAF3 | 3.214E-03 | 3.552 | 999 | 0.918 |
| 146 | ENSP00000350256 | CCR9 | 1.441E-04 | 5.904 | 930 | 0.918 |
| 147 | ENSP00000311857 | PTPN2 | 1.891E-03 | 4.264 | 982 | 0.916 |
| 148 | ENSP00000377836 | GPC3 | 2.274E-03 | 8.115 | 900 | 0.916 |
| 149 | ENSP00000296026 | CXCL3 | 1.822E-04 | 8.430 | 967 | 0.916 |
| 150 | ENSP00000211998 | VCL | 8.461E-04 | 3.022 | 912 | 0.915 |
| 151 | ENSP00000343535 | USP7 | 1.408E-03 | 3.333 | 922 | 0.915 |
| 152 | ENSP00000357470 | IL6R | 2.720E-04 | 9.347 | 999 | 0.914 |
| 153 | ENSP00000472929 | VAV1 | 9.700E-04 | 5.348 | 994 | 0.913 |
| 154 | ENSP00000410715 | SFRP4 | 1.118E-03 | 7.343 | 916 | 0.913 |
| 155 | ENSP00000329411 | IRF7 | 3.066E-03 | 3.977 | 932 | 0.913 |
| 156 | ENSP00000264563 | IL11 | 3.167E-04 | 10.322 | 991 | 0.912 |
| 157 | ENSP00000380150 | CARD11 | 5.453E-03 | 8.642 | 932 | 0.912 |
| 158 | ENSP00000347197 | C5AR1 | 1.648E-04 | 3.272 | 938 | 0.912 |
| 159 | ENSP00000431418 | NTRK1 | 9.443E-04 | 6.523 | 985 | 0.911 |
| 160 | ENSP00000367930 | OXER1 | 7.978E-05 | 2.695 | 900 | 0.911 |
| 161 | ENSP00000289473 | NCF1 | 3.036E-03 | 9.024 | 930 | 0.910 |
| 162 | ENSP00000419945 | ERVW-1 | 9.053E-04 | 5.928 | 949 | 0.910 |
| 163 | ENSP00000327890 | IL3RA | 2.138E-04 | 6.895 | 966 | 0.910 |
| 164 | ENSP00000342681 | CD5 | 1.917E-04 | 3.095 | 951 | 0.909 |
| 165 | ENSP00000246115 | S1PR4 | 1.222E-04 | 4.174 | 906 | 0.909 |
| 166 | ENSP00000249041 | GALR3 | 1.391E-04 | 5.436 | 901 | 0.908 |
| 167 | ENSP00000355370 | CNTF | 1.714E-04 | 6.259 | 988 | 0.908 |
| 168 | ENSP00000296545 | IL15 | 3.740E-04 | 8.145 | 937 | 0.908 |
| 169 | ENSP00000209728 | CDC6 | 7.947E-04 | 3.745 | 910 | 0.908 |
| 170 | ENSP00000329684 | GALR2 | 8.712E-05 | 2.134 | 901 | 0.907 |
| 171 | ENSP00000215781 | OSM | 2.575E-04 | 4.632 | 995 | 0.906 |
| 172 | ENSP00000319279 | MALT1 | 6.046E-03 | 4.943 | 969 | 0.906 |
| 173 | ENSP00000382423 | MAP3K1 | 9.538E-04 | 4.470 | 987 | 0.906 |
| 174 | ENSP00000315130 | CLU | 1.211E-03 | 4.267 | 912 | 0.906 |
| 175 | ENSP00000332812 | PTGDR2 | 1.354E-04 | 4.348 | 918 | 0.904 |
| 176 | ENSP00000256906 | HRH4 | 1.141E-04 | 4.771 | 904 | 0.902 |
| 177 | ENSP00000302234 | CCL11 | 1.974E-04 | 2.679 | 999 | 0.902 |
| 178 | ENSP00000330284 | NPBWR1 | 8.611E-05 | 3.391 | 901 | 0.900 |
| 179 | ENSP00000349252 | ITGAL | 1.968E-04 | 3.037 | 999 | 0.900 |
| 180 | ENSP00000363826 | FZD8 | 3.668E-03 | 3.227 | 976 | 0.899 |
| 181 | ENSP00000332766 | NPB | 7.728E-05 | 1.991 | 900 | 0.899 |
| 182 | ENSP00000339804 | TSLP | 2.441E-04 | 5.770 | 988 | 0.898 |
| 183 | ENSP00000255008 | SSTR4 | 1.046E-04 | 3.445 | 907 | 0.898 |
| 184 | ENSP00000357879 | PSMD4 | 1.251E-03 | 3.930 | 900 | 0.897 |
| 185 | ENSP00000316328 | CIITA | 1.822E-04 | 4.517 | 944 | 0.896 |
| 186 | ENSP00000307766 | HTR1E | 7.687E-05 | 2.598 | 903 | 0.896 |
| 187 | ENSP00000221132 | TNFRSF10A | 5.508E-03 | 4.458 | 956 | 0.896 |
| 188 | ENSP00000287907 | HTR5A | 1.144E-04 | 4.725 | 912 | 0.894 |
| 189 | ENSP00000240100 | DUSP4 | 1.320E-03 | 4.360 | 941 | 0.894 |
| 190 | ENSP00000297904 | FIGF | 1.238E-04 | 4.798 | 925 | 0.893 |
| 191 | ENSP00000358783 | NPBWR2 | 8.294E-05 | 2.780 | 901 | 0.893 |
| 192 | ENSP00000354207 | NTRK3 | 2.264E-03 | 8.691 | 933 | 0.893 |
| 193 | ENSP00000296028 | PPBP | 1.640E-04 | 3.148 | 973 | 0.891 |
| 194 | ENSP00000286201 | FZD7 | 4.980E-03 | 4.271 | 983 | 0.891 |
| 195 | ENSP00000240093 | FZD3 | 4.436E-03 | 9.012 | 958 | 0.890 |
| 196 | ENSP00000274520 | IL9 | 3.182E-04 | 8.357 | 909 | 0.890 |
| 197 | ENSP00000226279 | CD38 | 1.397E-04 | 7.785 | 983 | 0.889 |
| 198 | ENSP00000362924 | GSN | 1.076E-03 | 2.956 | 977 | 0.889 |
| 199 | ENSP00000302079 | C3AR1 | 1.672E-04 | 4.286 | 916 | 0.889 |
| 200 | ENSP00000266066 | SFRP5 | 5.088E-03 | 8.867 | 908 | 0.889 |
| 201 | ENSP00000299727 | GALR1 | 8.021E-05 | 2.459 | 901 | 0.888 |
| 202 | ENSP00000301908 | PNOC | 9.334E-05 | 3.437 | 900 | 0.888 |
| 203 | ENSP00000388910 | NFATC4 | 9.296E-03 | 9.001 | 938 | 0.888 |
| 204 | ENSP00000468348 | MAP2K6 | 1.514E-03 | 2.019 | 917 | 0.887 |
| 205 | ENSP00000254227 | NR0B2 | 2.001E-03 | 4.421 | 901 | 0.886 |
| 206 | ENSP00000271331 | PROK1 | 7.993E-05 | 2.198 | 900 | 0.886 |
| 207 | ENSP00000340191 | FPR2 | 9.292E-05 | 2.866 | 944 | 0.886 |
| 208 | ENSP00000361548 | MPL | 1.362E-04 | 3.697 | 997 | 0.885 |
| 209 | ENSP00000343957 | IFNAR2 | 2.696E-04 | 7.389 | 976 | 0.885 |
| 210 | ENSP00000354607 | FZD5 | 4.054E-03 | 8.957 | 973 | 0.884 |
| 211 | ENSP00000355156 | SUCNR1 | 7.096E-05 | 2.419 | 902 | 0.883 |
| 212 | ENSP00000247881 | TAS2R4 | 1.213E-04 | 1.978 | 900 | 0.882 |
| 213 | ENSP00000286186 | CASP10 | 3.845E-03 | 4.431 | 983 | 0.882 |
| 214 | ENSP00000320376 | P2RY13 | 8.710E-05 | 2.489 | 904 | 0.881 |
| 215 | ENSP00000321209 | ZNF274 | 1.621E-03 | 3.047 | 903 | 0.880 |
| 216 | ENSP00000298440 | OXGR1 | 9.410E-05 | 2.787 | 900 | 0.879 |
| 217 | ENSP00000327875 | LPAR5 | 7.245E-05 | 2.091 | 900 | 0.879 |
| 218 | ENSP00000366061 | NMS | 7.568E-05 | 3.446 | 900 | 0.879 |
| 219 | ENSP00000216629 | BDKRB1 | 9.022E-05 | 4.721 | 903 | 0.878 |
| 220 | ENSP00000391669 | BCAR1 | 1.107E-03 | 6.954 | 961 | 0.877 |
| 221 | ENSP00000255262 | NMUR2 | 7.621E-05 | 3.760 | 901 | 0.876 |
| 222 | ENSP00000267377 | SSTR1 | 9.178E-05 | 2.766 | 901 | 0.876 |
| 223 | ENSP00000276431 | TNFRSF10B | 3.325E-03 | 4.455 | 953 | 0.875 |
| 224 | ENSP00000305877 | NMUR1 | 6.732E-05 | 2.549 | 901 | 0.875 |
| 225 | ENSP00000356793 | XCL2 | 1.423E-04 | 3.804 | 900 | 0.875 |
| 226 | ENSP00000358501 | CD58 | 2.143E-04 | 2.364 | 942 | 0.875 |
| 227 | ENSP00000337383 | NLRP3 | 1.280E-04 | 2.239 | 987 | 0.875 |
| 228 | ENSP00000046794 | LCP2 | 4.440E-03 | 4.480 | 909 | 0.873 |
| 229 | ENSP00000272644 | GPR17 | 8.337E-05 | 3.927 | 903 | 0.873 |
| 230 | ENSP00000218758 | ACP5 | 2.837E-03 | 4.413 | 960 | 0.872 |
| 231 | ENSP00000296029 | PF4 | 2.008E-04 | 6.668 | 983 | 0.872 |
| 232 | ENSP00000355627 | AGT | 9.010E-04 | 7.848 | 977 | 0.871 |
| 233 | ENSP00000264218 | NMU | 7.650E-05 | 3.620 | 904 | 0.871 |
| 234 | ENSP00000228534 | IL23A | 3.337E-04 | 9.941 | 998 | 0.870 |
| 235 | ENSP00000273430 | AGTR1 | 1.690E-03 | 9.290 | 959 | 0.867 |
| 236 | ENSP00000302811 | MTNR1A | 2.648E-03 | 8.972 | 902 | 0.867 |
| 237 | ENSP00000338010 | IL21R | 2.595E-04 | 8.241 | 997 | 0.866 |
| 238 | ENSP00000009180 | CD9 | 7.225E-05 | 2.085 | 937 | 0.866 |
| 239 | ENSP00000291700 | S100B | 1.453E-03 | 9.019 | 900 | 0.865 |
| 240 | ENSP00000332816 | PTK2B | 1.665E-03 | 4.321 | 934 | 0.863 |
| 241 | ENSP00000259206 | IL1RN | 2.692E-04 | 13.077 | 999 | 0.862 |
| 242 | ENSP00000356515 | UTRN | 1.704E-03 | 8.524 | 962 | 0.862 |
| 243 | ENSP00000354952 | GAB2 | 5.694E-03 | 8.967 | 942 | 0.858 |
| 244 | ENSP00000370979 | CRLF2 | 1.847E-04 | 2.879 | 994 | 0.857 |
| 245 | ENSP00000367830 | PRKCZ | 2.421E-03 | 8.117 | 906 | 0.857 |
| 246 | ENSP00000364893 | ARHGEF7 | 2.422E-03 | 8.375 | 905 | 0.857 |
| 247 | ENSP00000257254 | APLNR | 7.744E-05 | 2.561 | 912 | 0.856 |
| 248 | ENSP00000349003 | PTGER3 | 2.187E-03 | 9.077 | 930 | 0.855 |
| 249 | ENSP00000381599 | MX1 | 6.984E-04 | 4.575 | 939 | 0.853 |
| 250 | ENSP00000301974 | PTAFR | 4.208E-03 | 4.538 | 955 | 0.853 |
| 251 | ENSP00000310405 | XCR1 | 9.539E-05 | 3.207 | 900 | 0.852 |
| 252 | ENSP00000339186 | GRAP2 | 2.587E-03 | 4.197 | 994 | 0.852 |
| 253 | ENSP00000307259 | P2RY12 | 8.403E-05 | 2.730 | 916 | 0.851 |
| 254 | ENSP00000241014 | MAPK8IP1 | 4.921E-03 | 8.819 | 998 | 0.851 |
| 255 | ENSP00000229030 | FZD10 | 4.336E-03 | 9.014 | 948 | 0.846 |
| 256 | ENSP00000160262 | ICAM3 | 2.192E-04 | 5.471 | 904 | 0.844 |
| 257 | ENSP00000265023 | KNG1 | 7.856E-04 | 4.372 | 951 | 0.843 |
| 258 | ENSP00000262958 | GNA15 | 5.674E-04 | 3.115 | 922 | 0.842 |
| 259 | ENSP00000274031 | SETD7 | 1.080E-03 | 2.550 | 925 | 0.842 |
| 260 | ENSP00000242338 | CNTFR | 1.607E-04 | 5.321 | 908 | 0.840 |
| 261 | ENSP00000409581 | ARRB1 | 9.761E-04 | 8.182 | 969 | 0.840 |
| 262 | ENSP00000265164 | CASP6 | 5.725E-03 | 4.359 | 994 | 0.839 |
| 263 | ENSP00000380855 | PDCD1LG2 | 2.502E-04 | 5.641 | 996 | 0.839 |
| 264 | ENSP00000360141 | GNAS | 9.685E-04 | 6.649 | 900 | 0.836 |
| 265 | ENSP00000363296 | GRM4 | 6.753E-05 | 2.302 | 902 | 0.833 |
| 266 | ENSP00000219255 | PARD6A | 1.991E-03 | 4.357 | 904 | 0.832 |
| 267 | ENSP00000270800 | IL22RA1 | 2.239E-04 | 2.856 | 999 | 0.832 |
| 268 | ENSP00000219235 | CCL22 | 4.754E-04 | 13.523 | 985 | 0.831 |
| 269 | ENSP00000262395 | TRAF4 | 2.568E-03 | 4.346 | 926 | 0.829 |
| 270 | ENSP00000274376 | RASA1 | 2.143E-03 | 4.287 | 902 | 0.826 |
| 271 | ENSP00000384675 | SOS1 | 1.031E-03 | 6.193 | 941 | 0.825 |
| 272 | ENSP00000356946 | FCGR3A | 2.910E-04 | 10.576 | 905 | 0.824 |
| 273 | ENSP00000231188 | GRM6 | 5.992E-05 | 2.687 | 902 | 0.819 |
| 274 | ENSP00000234347 | PRTN3 | 1.105E-04 | 4.923 | 909 | 0.812 |
| 275 | ENSP00000364860 | ROR2 | 7.864E-03 | 8.994 | 902 | 0.811 |
| 276 | ENSP00000233242 | APOB | 8.032E-04 | 3.746 | 913 | 0.811 |
| 277 | ENSP00000452746 | PSTPIP1 | 1.394E-03 | 6.520 | 995 | 0.809 |
| 278 | ENSP00000358511 | CNR1 | 7.725E-05 | 2.187 | 918 | 0.806 |
| 279 | ENSP00000219244 | CCL17 | 4.588E-04 | 15.830 | 971 | 0.801 |
| 280 | ENSP00000348069 | SREBF1 | 1.341E-03 | 7.105 | 953 | 0.799 |
| 281 | ENSP00000256452 | IL5RA | 2.803E-04 | 7.098 | 993 | 0.798 |
| 282 | ENSP00000363680 | EDA | 2.242E-03 | 8.388 | 913 | 0.796 |
| 283 | ENSP00000286548 | GNAQ | 9.962E-04 | 3.240 | 958 | 0.795 |
| 284 | ENSP00000364114 | HLA-DRB5 | 2.395E-04 | 2.265 | 921 | 0.790 |
| 285 | ENSP00000228862 | DUSP16 | 1.743E-03 | 8.313 | 905 | 0.789 |
| 286 | ENSP00000264260 | IL18RAP | 9.977E-03 | 9.331 | 981 | 0.789 |
| 287 | ENSP00000355866 | DUSP10 | 1.560E-03 | 7.906 | 972 | 0.788 |
| 288 | ENSP00000260049 | IL18BP | 3.841E-04 | 9.657 | 982 | 0.785 |
| 289 | ENSP00000300659 | NFATC3 | 1.474E-02 | 9.035 | 982 | 0.784 |
| 290 | ENSP00000282588 | ITGA1 | 1.515E-04 | 5.777 | 902 | 0.782 |
| 291 | ENSP00000299767 | HSP90B1 | 4.971E-04 | 3.238 | 968 | 0.782 |
| 292 | ENSP00000349365 | IL27 | 4.103E-04 | 6.109 | 937 | 0.780 |
| 293 | ENSP00000303452 | TRH | 1.736E-03 | 8.377 | 900 | 0.778 |
| 294 | ENSP00000338130 | KLRD1 | 5.175E-03 | 13.850 | 943 | 0.777 |
| 295 | ENSP00000256857 | GRP | 2.426E-03 | 9.032 | 901 | 0.777 |
| 296 | ENSP00000292513 | PTGER1 | 7.127E-05 | 2.949 | 900 | 0.776 |
| 297 | ENSP00000265094 | FBXW11 | 2.283E-03 | 4.110 | 916 | 0.775 |
| 298 | ENSP00000356792 | XCL1 | 1.315E-04 | 3.058 | 900 | 0.772 |
| 299 | ENSP00000328207 | TNFRSF18 | 4.627E-04 | 11.405 | 949 | 0.771 |
| 300 | ENSP00000288422 | TAB3 | 7.211E-03 | 4.385 | 989 | 0.768 |
| 301 | ENSP00000296980 | IL22RA2 | 1.895E-04 | 2.128 | 998 | 0.766 |
| 302 | ENSP00000278568 | PAK1 | 1.012E-03 | 3.164 | 907 | 0.764 |
| 303 | ENSP00000351049 | PAK4 | 1.014E-03 | 3.920 | 905 | 0.761 |
| 304 | ENSP00000233957 | IL18R1 | 8.105E-03 | 9.330 | 991 | 0.757 |
| 305 | ENSP00000378845 | LAT | 7.624E-03 | 9.096 | 900 | 0.757 |
| 306 | ENSP00000354566 | CD3E | 2.576E-04 | 2.552 | 937 | 0.753 |
| 307 | ENSP00000378118 | CCL8 | 2.004E-04 | 3.658 | 996 | 0.742 |
| 308 | ENSP00000261303 | PSMC1 | 1.413E-03 | 2.890 | 911 | 0.740 |
| 309 | ENSP00000252506 | GADD45G | 1.475E-04 | 2.612 | 901 | 0.733 |
| 310 | ENSP00000381412 | CAMK2A | 1.879E-03 | 4.559 | 901 | 0.730 |
| 311 | ENSP00000284818 | LY96 | 1.940E-04 | 2.997 | 999 | 0.729 |
| 312 | ENSP00000388566 | CASP4 | 5.839E-03 | 8.906 | 957 | 0.720 |
| 313 | ENSP00000412897 | IFNA4 | 1.206E-04 | 3.000 | 916 | 0.716 |
| 314 | ENSP00000225844 | CCL13 | 5.684E-04 | 16.200 | 984 | 0.716 |
| 315 | ENSP00000331831 | GAS6 | 1.881E-02 | 4.430 | 907 | 0.713 |
| 316 | ENSP00000371327 | KLRC2 | 2.086E-04 | 3.296 | 901 | 0.711 |
| 317 | ENSP00000314363 | MAP4K4 | 3.870E-03 | 4.394 | 955 | 0.705 |
| 318 | ENSP00000411940 | IFNA17 | 1.086E-04 | 2.513 | 920 | 0.699 |
| 319 | ENSP00000376849 | CASP5 | 6.790E-03 | 8.998 | 906 | 0.695 |
| 320 | ENSP00000319060 | CAMK2G | 2.095E-03 | 3.192 | 901 | 0.688 |
| 321 | ENSP00000279230 | PLCB3 | 2.025E-03 | 4.370 | 904 | 0.679 |
| 322 | ENSP00000260402 | PLCB2 | 1.743E-03 | 4.344 | 904 | 0.679 |
| 323 | ENSP00000362014 | DNM1 | 6.177E-04 | 4.517 | 910 | 0.678 |
| 324 | ENSP00000338185 | PLCB1 | 1.658E-03 | 3.856 | 904 | 0.677 |
| 325 | ENSP00000278407 | SERPING1 | 1.053E-04 | 4.163 | 912 | 0.674 |
| 326 | ENSP00000309871 | RACGAP1 | 1.324E-03 | 3.871 | 984 | 0.668 |
| 327 | ENSP00000361824 | SPTAN1 | 1.010E-03 | 5.745 | 975 | 0.665 |
| 328 | ENSP00000232003 | HRG | 1.200E-04 | 3.273 | 937 | 0.664 |
| 329 | ENSP00000216455 | PSMA3 | 1.820E-03 | 3.798 | 900 | 0.661 |
| 330 | ENSP00000369574 | IFNA21 | 1.568E-04 | 2.185 | 921 | 0.660 |
| 331 | ENSP00000339740 | CAMK2D | 2.217E-03 | 3.126 | 901 | 0.655 |
| 332 | ENSP00000348442 | PSMD12 | 4.494E-03 | 8.596 | 902 | 0.654 |
| 333 | ENSP00000366307 | THBD | 1.204E-04 | 3.344 | 926 | 0.650 |
| 334 | ENSP00000264870 | F13A1 | 1.070E-04 | 2.747 | 933 | 0.649 |
| 335 | ENSP00000352064 | KLRC1 | 3.048E-04 | 8.364 | 937 | 0.649 |
| 336 | ENSP00000217169 | BIRC7 | 6.441E-03 | 4.316 | 989 | 0.632 |
| 337 | ENSP00000239347 | IFNA7 | 3.152E-04 | 4.525 | 902 | 0.627 |
| 338 | ENSP00000369566 | IFNA10 | 1.781E-04 | 4.177 | 921 | 0.625 |
| 339 | ENSP00000369558 | IFNA6 | 1.603E-04 | 2.636 | 915 | 0.623 |
| 340 | ENSP00000297439 | DEFB1 | 8.819E-05 | 2.841 | 965 | 0.615 |
| 341 | ENSP00000309103 | BAD | 1.062E-02 | 8.707 | 907 | 0.602 |
| 342 | ENSP00000369553 | IFNA8 | 1.294E-04 | 2.663 | 918 | 0.598 |
| 343 | ENSP00000328173 | C1S | 1.384E-04 | 3.363 | 900 | 0.594 |
| 344 | ENSP00000369564 | IFNA16 | 1.139E-04 | 2.283 | 902 | 0.594 |
| 345 | ENSP00000389140 | DCC | 3.833E-03 | 8.659 | 963 | 0.591 |
| 346 | ENSP00000334329 | UNC5B | 8.984E-03 | 4.395 | 900 | 0.591 |
| 347 | ENSP00000313967 | C1QB | 1.610E-04 | 4.041 | 909 | 0.565 |
| 348 | ENSP00000384432 | ICOSLG | 2.321E-04 | 2.218 | 985 | 0.564 |
| 349 | ENSP00000294800 | FCGR3B | 2.916E-04 | 6.073 | 904 | 0.550 |
| 350 | ENSP00000264790 | MMRN1 | 5.522E-05 | 2.170 | 997 | 0.535 |
| 351 | ENSP00000336769 | KIR2DL1 | 3.418E-04 | 6.540 | 974 | 0.521 |
| 352 | ENSP00000430344 | PLCD1 | 1.121E-03 | 7.624 | 982 | 0.512 |
| 353 | ENSP00000221847 | EBI3 | 5.751E-04 | 6.752 | 911 | 0.500 |
| 354 | ENSP00000330572 | MAPK8IP2 | 6.880E-03 | 4.420 | 932 | 0.497 |
| 355 | ENSP00000356037 | C4BPA | 1.022E-04 | 2.323 | 918 | 0.463 |
| 356 | ENSP00000250894 | MAPK8IP3 | 3.730E-03 | 8.985 | 987 | 0.461 |
| 357 | ENSP00000272324 | REG3G | 2.635E-04 | 7.139 | 929 | 0.448 |
| 358 | ENSP00000226524 | PF4V1 | 9.063E-04 | 13.522 | 900 | 0.403 |
| 359 | ENSP00000342215 | KIR2DL3 | 1.141E-02 | 9.167 | 982 | 0.401 |
| 360 | ENSP00000356382 | F13B | 1.763E-04 | 4.353 | 929 | 0.364 |
| 361 | ENSP00000348278 | STK39 | 5.166E-03 | 8.689 | 900 | 0.347 |
| 362 | ENSP00000278187 | GAS2 | 1.889E-04 | 2.362 | 919 | 0.322 |
| 363 | ENSP00000312122 | SEC13 | 1.761E-03 | 3.602 | 902 | 0.277 |
| 364 | ENSP00000456039 | INMT-FAM188B | 7.296E-05 | 2.015 | 900 | 0.247 |
| 365 | ENSP00000225245 | CCL3 | 2.289E-04 | 11.140 | 999 | 0.000 |
| 366 | ENSP00000250151 | CCL4 | 2.101E-03 | 4.703 | 999 | 0.000 |
| 367 | ENSP00000274335 | PIK3R1 | 1.157E-03 | 3.370 | 999 | 0.000 |
| 368 | ENSP00000371432 | PRLR | 1.377E-04 | 1.965 | 999 | 0.000 |
| 369 | ENSP00000357103 | DARC | 9.600E-05 | 2.165 | 997 | 0.000 |
| 370 | ENSP00000303315 | JUNB | 6.277E-04 | 3.182 | 996 | 0.000 |
| 371 | ENSP00000315955 | FOXA2 | 5.829E-04 | 4.010 | 994 | 0.000 |
| 372 | ENSP00000293280 | CCL23 | 2.693E-04 | 9.326 | 988 | 0.000 |
| 373 | ENSP00000358622 | IKBKG | 1.074E-03 | 3.209 | 985 | 0.000 |
| 374 | ENSP00000269260 | ARRB2 | 1.106E-03 | 4.346 | 966 | 0.000 |
| 375 | ENSP00000273145 | CCBP2 | 2.733E-04 | 6.743 | 965 | 0.000 |
| 376 | ENSP00000340330 | KAT5 | 5.002E-04 | 2.892 | 961 | 0.000 |
| 377 | ENSP00000405533 | CCL4L1 | 1.628E-03 | 9.374 | 956 | 0.000 |
| 378 | ENSP00000305464 | APLN | 1.015E-04 | 5.070 | 949 | 0.000 |
| 379 | ENSP00000261991 | RPS6KA5 | 4.991E-04 | 3.074 | 947 | 0.000 |
| 380 | ENSP00000408984 | CCL3L1 | 1.373E-03 | 7.455 | 945 | 0.000 |
| 381 | ENSP00000310170 | FOSL1 | 7.120E-04 | 3.615 | 937 | 0.000 |
| 382 | ENSP00000316746 | - | 2.149E-04 | 2.804 | 937 | 0.000 |
| 383 | ENSP00000409197 | CCL14 | 3.564E-04 | 11.094 | 932 | 0.000 |
| 384 | ENSP00000280193 | VEGFC | 1.146E-04 | 2.965 | 925 | 0.000 |
| 385 | ENSP00000329212 | ATF7 | 1.083E-03 | 3.781 | 918 | 0.000 |
| 386 | ENSP00000259555 | IFNA5 | 2.453E-04 | 3.321 | 916 | 0.000 |
| 387 | ENSP00000004921 | CCL18 | 3.723E-04 | 2.029 | 912 | 0.000 |
| 388 | ENSP00000334105 | PLCB4 | 1.872E-03 | 4.180 | 904 | 0.000 |
| 389 | ENSP00000307713 | BDKRB2 | 7.138E-05 | 2.596 | 904 | 0.000 |
| 390 | ENSP00000243440 | BATF3 | 1.081E-03 | 8.928 | 900 | 0.000 |
| 391 | ENSP00000418447 | PPP2CA | 6.094E-04 | 3.364 | 900 | 0.000 |
| 392 | ENSP00000275525 | IGFBP1 | 1.865E-03 | 8.239 | 899 | - |
| 393 | ENSP00000316054 | DVL3 | 1.192E-03 | 3.247 | 898 | - |
| 394 | ENSP00000353472 | HLA-G | 5.491E-03 | 14.204 | 897 | - |
| 395 | ENSP00000342070 | CTSB | 1.087E-03 | 3.978 | 897 | - |
| 396 | ENSP00000428340 | ANGPT1 | 1.232E-03 | 2.120 | 897 | - |
| 397 | ENSP00000362181 | NCR2 | 2.972E-04 | 5.067 | 896 | - |
| 398 | ENSP00000297350 | TNFRSF11B | 2.469E-03 | 9.175 | 895 | - |
| 399 | ENSP00000339467 | RHOG | 3.883E-04 | 2.362 | 894 | - |
| 400 | ENSP00000299855 | MMP3 | 1.381E-03 | 9.557 | 891 | - |
| 401 | ENSP00000264554 | SHC2 | 2.752E-03 | 9.026 | 891 | - |
| 402 | ENSP00000376793 | SERPINA3 | 2.805E-03 | 9.158 | 889 | - |
| 403 | ENSP00000260302 | MMP13 | 4.250E-03 | 9.271 | 888 | - |
| 404 | ENSP00000263326 | IL37 | 4.090E-04 | 6.720 | 888 | - |
| 405 | ENSP00000309338 | CLCF1 | 1.690E-04 | 1.962 | 886 | - |
| 406 | ENSP00000226218 | SEBOX | 1.177E-03 | 8.483 | 885 | - |
| 407 | ENSP00000268704 | SPG7 | 1.044E-03 | 8.408 | 885 | - |
| 408 | ENSP00000369312 | IL15RA | 2.266E-04 | 3.530 | 883 | - |
| 409 | ENSP00000331736 | SELE | 1.406E-03 | 9.127 | 882 | - |
| 410 | ENSP00000329668 | SHC4 | 5.249E-03 | 8.938 | 882 | - |
| 411 | ENSP00000267843 | FGF7 | 1.261E-03 | 5.258 | 879 | - |
| 412 | ENSP00000286639 | BATF | 1.052E-03 | 8.984 | 878 | - |
| 413 | ENSP00000363868 | ABCA1 | 1.235E-03 | 7.706 | 878 | - |
| 414 | ENSP00000309968 | ADAM17 | 2.000E-03 | 4.508 | 878 | - |
| 415 | ENSP00000287295 | AIFM1 | 1.362E-03 | 4.132 | 878 | - |
| 416 | ENSP00000301653 | KRT16 | 6.090E-04 | 3.638 | 878 | - |
| 417 | ENSP00000331358 | GAST | 2.463E-03 | 7.414 | 877 | - |
| 418 | ENSP00000334458 | GATA4 | 4.953E-04 | 7.413 | 877 | - |
| 419 | ENSP00000327824 | IFNLR1 | 2.892E-04 | 7.385 | 877 | - |
| 420 | ENSP00000277480 | LCN2 | 9.426E-05 | 2.902 | 876 | - |
| 421 | ENSP00000327048 | MAF | 2.076E-03 | 9.187 | 874 | - |
| 422 | ENSP00000263621 | ELANE | 8.860E-04 | 8.883 | 874 | - |
| 423 | ENSP00000283147 | BMP6 | 1.892E-03 | 4.464 | 874 | - |
| 424 | ENSP00000348099 | PDLIM7 | 2.569E-03 | 7.989 | 873 | - |
| 425 | ENSP00000383672 | KIR3DS1 | 3.346E-04 | 9.369 | 872 | - |
| 426 | ENSP00000367605 | CCL3L3 | 8.155E-03 | 9.162 | 870 | - |
| 427 | ENSP00000312027 | CD7 | 1.390E-04 | 2.959 | 870 | - |
| 428 | ENSP00000261254 | CCND2 | 1.099E-03 | 2.034 | 870 | - |
| 429 | ENSP00000178640 | MAP2K5 | 5.781E-04 | 4.446 | 867 | - |
| 430 | ENSP00000457748 | RGSL1 | 1.276E-04 | 4.348 | 867 | - |
| 431 | ENSP00000263863 | GNLY | 3.629E-04 | 13.689 | 866 | - |
| 432 | ENSP00000366234 | IL31 | 1.589E-04 | 2.261 | 866 | - |
| 433 | ENSP00000314976 | IL20RA | 2.100E-04 | 2.184 | 866 | - |
| 434 | ENSP00000356016 | CR1 | 1.589E-04 | 2.191 | 864 | - |
| 435 | ENSP00000215637 | MADCAM1 | 2.650E-04 | 9.751 | 863 | - |
| 436 | ENSP00000263100 | A1BG | 9.771E-05 | 2.722 | 863 | - |
| 437 | ENSP00000279441 | MMP10 | 5.600E-03 | 9.151 | 860 | - |
| 438 | ENSP00000409007 | GDNF | 1.008E-03 | 7.964 | 860 | - |
| 439 | ENSP00000261693 | SCARB1 | 1.999E-03 | 4.279 | 860 | - |
| 440 | ENSP00000328133 | IL20RB | 1.422E-04 | 3.394 | 860 | - |
| 441 | ENSP00000354003 | GYPA | 9.819E-05 | 3.343 | 860 | - |
| 442 | ENSP00000370473 | IGFBP3 | 1.638E-03 | 5.891 | 859 | - |
| 443 | ENSP00000265077 | VCAN | 1.610E-03 | 4.362 | 859 | - |
| 444 | ENSP00000294304 | LRP5 | 1.588E-03 | 3.520 | 855 | - |
| 445 | ENSP00000318820 | TPO | 9.492E-05 | 2.382 | 855 | - |
| 446 | ENSP00000338548 | FGF1 | 1.399E-03 | 4.461 | 854 | - |
| 447 | ENSP00000364995 | SHC3 | 5.767E-03 | 8.963 | 852 | - |
| 448 | ENSP00000244336 | CEACAM8 | 1.036E-04 | 2.869 | 851 | - |
| 449 | ENSP00000373691 | DUOX2 | 6.458E-03 | 2.176 | 851 | - |
| 450 | ENSP00000265165 | LEF1 | 6.738E-04 | 3.709 | 850 | - |
| 451 | ENSP00000222598 | DLX5 | 2.122E-03 | 4.422 | 849 | - |
| 452 | ENSP00000361264 | RAPGEF1 | 6.317E-03 | 8.768 | 846 | - |
| 453 | ENSP00000391592 | PTPN6 | 7.015E-03 | 4.066 | 846 | - |
| 454 | ENSP00000286657 | ADAMTS3 | 8.750E-05 | 2.802 | 846 | - |
| 455 | ENSP00000255409 | CHI3L1 | 8.002E-05 | 2.104 | 842 | - |
| 456 | ENSP00000216923 | ZFP64 | 1.352E-03 | 3.296 | 841 | - |
| 457 | ENSP00000296417 | H2AFZ | 5.626E-04 | 2.826 | 840 | - |
| 458 | ENSP00000394794 | PTPN13 | 1.159E-03 | 7.602 | 839 | - |
| 459 | ENSP00000451511 | LY75 | 1.526E-04 | 2.879 | 839 | - |
| 460 | ENSP00000366244 | ARAF | 2.687E-03 | 8.710 | 838 | - |
| 461 | ENSP00000312002 | HAVCR2 | 2.439E-04 | 8.347 | 838 | - |
| 462 | ENSP00000329991 | IFNL1 | 2.312E-04 | 5.924 | 837 | - |
| 463 | ENSP00000379204 | BMP7 | 1.020E-03 | 6.017 | 836 | - |
| 464 | ENSP00000302924 | IL17D | 4.163E-04 | 5.856 | 836 | - |
| 465 | ENSP00000304501 | ADORA2B | 4.496E-03 | 4.375 | 836 | - |
| 466 | ENSP00000230895 | DAP | 6.390E-03 | 4.400 | 835 | - |
| 467 | ENSP00000282561 | GJA1 | 1.066E-03 | 4.224 | 835 | - |
| 468 | ENSP00000235329 | MFN2 | 1.152E-03 | 4.115 | 835 | - |
| 469 | ENSP00000355231 | BECN1 | 1.629E-03 | 7.457 | 834 | - |
| 470 | ENSP00000309116 | PTPN7 | 4.893E-03 | 4.362 | 834 | - |
| 471 | ENSP00000262629 | TYROBP | 1.201E-04 | 4.065 | 834 | - |
| 472 | ENSP00000294489 | PDPN | 1.131E-04 | 2.453 | 834 | - |
| 473 | ENSP00000384792 | WDR62 | 1.079E-02 | 8.335 | 833 | - |
| 474 | ENSP00000379065 | TRPS1 | 9.130E-04 | 7.106 | 833 | - |
| 475 | ENSP00000369889 | COL2A1 | 1.217E-03 | 8.743 | 831 | - |
| 476 | ENSP00000470004 | RPS19 | 1.017E-03 | 6.411 | 831 | - |
| 477 | ENSP00000393312 | FGFR1 | 6.749E-04 | 4.014 | 831 | - |
| 478 | ENSP00000296511 | ANXA5 | 2.453E-03 | 3.907 | 831 | - |
| 479 | ENSP00000353427 | NR4A1 | 1.415E-03 | 5.334 | 829 | - |
| 480 | ENSP00000244174 | IL9R | 2.053E-04 | 6.125 | 828 | - |
| 481 | ENSP00000356022 | SOD2 | 4.097E-04 | 2.550 | 828 | - |
| 482 | ENSP00000242208 | INHBA | 2.602E-03 | 9.109 | 827 | - |
| 483 | ENSP00000264664 | FGF10 | 1.753E-03 | 8.807 | 827 | - |
| 484 | ENSP00000255040 | APCS | 1.642E-04 | 3.103 | 827 | - |
| 485 | ENSP00000393099 | MAPKBP1 | 1.825E-03 | 7.205 | 826 | - |
| 486 | ENSP00000356063 | IL20 | 2.847E-04 | 5.744 | 826 | - |
| 487 | ENSP00000225698 | C1QBP | 9.316E-04 | 4.376 | 826 | - |
| 488 | ENSP00000353582 | NRP2 | 9.751E-05 | 2.375 | 826 | - |
| 489 | ENSP00000357153 | CD1D | 2.032E-04 | 4.995 | 825 | - |
| 490 | ENSP00000325525 | KIR3DL2 | 1.693E-02 | 19.371 | 824 | - |
| 491 | ENSP00000345281 | DUSP22 | 1.867E-03 | 4.509 | 824 | - |
| 492 | ENSP00000218099 | F9 | 9.712E-05 | 4.029 | 824 | - |
| 493 | ENSP00000357025 | CD48 | 5.061E-03 | 9.063 | 822 | - |
| 494 | ENSP00000264716 | FOSL2 | 8.449E-04 | 3.955 | 822 | - |
| 495 | ENSP00000363157 | TNFSF15 | 1.540E-04 | 3.928 | 821 | - |
| 496 | ENSP00000424038 | CASP12 | 1.322E-03 | 7.892 | 820 | - |
| 497 | ENSP00000317997 | DUOX1 | 6.234E-03 | 9.081 | 819 | - |
| 498 | ENSP00000312634 | PDLIM2 | 3.894E-03 | 4.206 | 819 | - |
| 499 | ENSP00000229134 | IL26 | 3.498E-04 | 9.189 | 818 | - |
| 500 | ENSP00000261623 | CYBA | 7.613E-03 | 4.451 | 817 | - |
| 501 | ENSP00000351497 | FCGR2B | 1.507E-04 | 3.567 | 817 | - |
| 502 | ENSP00000364597 | PADI4 | 1.706E-04 | 3.367 | 817 | - |
| 503 | ENSP00000358470 | VTCN1 | 2.154E-04 | 2.058 | 817 | - |
| 504 | ENSP00000295228 | INHBB | 3.474E-03 | 9.093 | 816 | - |
| 505 | ENSP00000295746 | KIAA1524 | 2.925E-02 | 4.429 | 815 | - |
| 506 | ENSP00000307875 | B3GAT1 | 1.402E-04 | 3.000 | 815 | - |
| 507 | ENSP00000278927 | ESAM | 1.052E-04 | 2.936 | 815 | - |
| 508 | ENSP00000336607 | P2RX4 | 8.262E-05 | 2.825 | 815 | - |
| 509 | ENSP00000320401 | UGT2B17 | 1.298E-04 | 2.192 | 815 | - |
| 510 | ENSP00000364858 | CARD16 | 9.201E-03 | 9.025 | 814 | - |
| 511 | ENSP00000356436 | PLA2G4A | 1.565E-03 | 8.629 | 814 | - |
| 512 | ENSP00000223271 | RARRES2 | 3.575E-04 | 7.550 | 814 | - |
| 513 | ENSP00000396538 | NFAT5 | 1.393E-03 | 5.863 | 814 | - |
| 514 | ENSP00000243077 | LRP1 | 2.389E-03 | 4.205 | 814 | - |
| 515 | ENSP00000345179 | APOD | 4.153E-03 | 8.996 | 811 | - |
| 516 | ENSP00000371790 | FGF9 | 1.961E-03 | 7.623 | 810 | - |
| 517 | ENSP00000309524 | CSHL1 | 1.866E-04 | 4.482 | 810 | - |
| 518 | ENSP00000302625 | AXIN2 | 1.431E-03 | 4.116 | 810 | - |
| 519 | ENSP00000334876 | GRK1 | 1.152E-04 | 2.716 | 809 | - |
| 520 | ENSP00000264952 | GRK7 | 6.217E-04 | 11.545 | 808 | - |
| 521 | ENSP00000433511 | GRK6 | 1.448E-04 | 2.198 | 808 | - |
| 522 | ENSP00000354687 | MT-ND1 | 2.047E-03 | 8.666 | 807 | - |
| 523 | ENSP00000259211 | IL36A | 7.365E-04 | 7.213 | 807 | - |
| 524 | ENSP00000308716 | INHBC | 5.928E-03 | 8.634 | 806 | - |
| 525 | ENSP00000356920 | DUSP12 | 1.391E-03 | 7.684 | 806 | - |
| 526 | ENSP00000333157 | GH2 | 1.168E-04 | 2.691 | 806 | - |
| 527 | ENSP00000375629 | LILRB2 | 4.513E-04 | 9.585 | 804 | - |
| 528 | ENSP00000215743 | MMP11 | 5.606E-03 | 9.081 | 804 | - |
| 529 | ENSP00000281172 | EPS8 | 3.166E-03 | 3.792 | 804 | - |
| 530 | ENSP00000292408 | FGFR4 | 5.879E-04 | 2.907 | 804 | - |
| 531 | ENSP00000319476 | ICOS | 6.787E-04 | 15.609 | 803 | - |
| 532 | ENSP00000277508 | PAEP | 8.162E-05 | 2.107 | 803 | - |
| 533 | ENSP00000316416 | CSH1 | 1.132E-04 | 2.323 | 802 | - |
| 534 | ENSP00000387356 | ACAN | 1.125E-04 | 2.148 | 802 | - |
| 535 | ENSP00000353660 | PEA15 | 6.166E-03 | 8.890 | 801 | - |
| 536 | ENSP00000376623 | CSH2 | 9.792E-05 | 2.749 | 801 | - |
| 537 | ENSP00000348308 | MMP23B | 2.298E-02 | 9.054 | 800 | - |
| 538 | ENSP00000398017 | CLDN9 | 1.494E-03 | 8.084 | 800 | - |
| 539 | ENSP00000333639 | IFNL2 | 1.773E-04 | 4.669 | 800 | - |
| 540 | ENSP00000282356 | CAMK4 | 8.664E-04 | 4.033 | 800 | - |
| 541 | ENSP00000250160 | WISP1 | 2.624E-04 | 3.455 | 800 | - |
| 542 | ENSP00000262039 | PIK3C3 | 7.942E-04 | 3.147 | 800 | - |
| 543 | ENSP00000409000 | IFNL3 | 1.265E-04 | 2.911 | 800 | - |
| 544 | ENSP00000437563 | NKG2-E | 4.638E-05 | 2.800 | 800 | - |
| 545 | ENSP00000240306 | DLX4 | 8.427E-05 | 2.704 | 800 | - |
| 546 | ENSP00000388223 | IFNAR2 | 1.660E-04 | 2.636 | 800 | - |
| 547 | ENSP00000276943 | IFNK | 2.508E-04 | 2.528 | 800 | - |
| 548 | ENSP00000394494 | IFNA13 | 1.090E-04 | 2.265 | 800 | - |
| 549 | ENSP00000377978 | CCL4L2 | 1.493E-02 | 9.116 | 792 | - |
| 550 | ENSP00000348611 | PHLPP2 | 4.457E-04 | 2.212 | 790 | - |
| 551 | ENSP00000285900 | GRIA1 | 1.658E-03 | 3.266 | 789 | - |
| 552 | ENSP00000306682 | PPM1D | 1.373E-03 | 3.798 | 788 | - |
| 553 | ENSP00000329539 | DUSP8 | 1.871E-03 | 8.278 | 783 | - |
| 554 | ENSP00000371554 | SOD3 | 2.291E-03 | 8.137 | 783 | - |
| 555 | ENSP00000372437 | TPTE2 | 7.173E-04 | 2.985 | 783 | - |
| 556 | ENSP00000353238 | SPN | 1.242E-04 | 2.522 | 782 | - |
| 557 | ENSP00000375608 | KIR3DL1 | 4.604E-04 | 6.579 | 777 | - |
| 558 | ENSP00000281834 | TNFSF4 | 2.040E-04 | 5.681 | 775 | - |
| 559 | ENSP00000282111 | TCF7L1 | 1.896E-03 | 4.316 | 773 | - |
| 560 | ENSP00000222330 | GSK3A | 3.940E-04 | 3.034 | 760 | - |
| 561 | ENSP00000364076 | HLA-DQA2 | 5.469E-04 | 9.164 | 755 | - |
| 562 | ENSP00000378332 | TGFB1I1 | 2.559E-03 | 8.596 | 755 | - |
| 563 | ENSP00000373574 | ACVRL1 | 1.147E-04 | 2.144 | 755 | - |
| 564 | ENSP00000386444 | SAG | 1.106E-03 | 4.798 | 754 | - |
| 565 | ENSP00000203629 | LAG3 | 4.952E-04 | 19.370 | 747 | - |
| 566 | ENSP00000323178 | KSR1 | 4.889E-03 | 9.924 | 747 | - |
| 567 | ENSP00000363976 | HLA-DMA | 3.276E-04 | 3.494 | 747 | - |
| 568 | ENSP00000253055 | MAP3K10 | 6.901E-04 | 4.435 | 746 | - |
| 569 | ENSP00000249075 | LIF | 1.270E-04 | 2.236 | 746 | - |
| 570 | ENSP00000357440 | HSF2 | 7.671E-04 | 4.897 | 745 | - |
| 571 | ENSP00000334869 | LCE1A | 2.513E-04 | 4.286 | 745 | - |
| 572 | ENSP00000357040 | VANGL2 | 3.397E-03 | 8.497 | 744 | - |
| 573 | ENSP00000321797 | FGF8 | 1.110E-03 | 8.113 | 744 | - |
| 574 | ENSP00000249071 | RAC2 | 2.994E-04 | 2.994 | 743 | - |
| 575 | ENSP00000264009 | HSF4 | 7.467E-04 | 8.185 | 742 | - |
| 576 | ENSP00000431284 | LPXN | 1.068E-03 | 7.618 | 742 | - |
| 577 | ENSP00000361202 | IRS4 | 4.074E-03 | 8.825 | 741 | - |
| 578 | ENSP00000216336 | CTSG | 6.769E-05 | 2.880 | 741 | - |
| 579 | ENSP00000159060 | NOX3 | 1.069E-04 | 3.894 | 740 | - |
| 580 | ENSP00000246792 | RRAS | 3.649E-04 | 2.903 | 735 | - |
| 581 | ENSP00000256196 | RRAS2 | 3.916E-04 | 2.790 | 735 | - |
| 582 | ENSP00000250092 | CD68 | 1.368E-03 | 9.582 | 734 | - |
| 583 | ENSP00000406738 | SARM1 | 1.277E-03 | 7.643 | 734 | - |
| 584 | ENSP00000335076 | EDARADD | 1.280E-04 | 2.481 | 732 | - |
| 585 | ENSP00000281708 | FBXW7 | 9.620E-04 | 2.145 | 732 | - |
| 586 | ENSP00000350359 | CREB5 | 8.835E-04 | 6.421 | 731 | - |
| 587 | ENSP00000406037 | KAT8 | 7.920E-04 | 5.738 | 731 | - |
| 588 | ENSP00000252945 | CYP2E1 | 2.386E-03 | 4.371 | 731 | - |
| 589 | ENSP00000314132 | BOK | 2.587E-03 | 3.951 | 730 | - |
| 590 | ENSP00000466174 | ATF7 | 1.201E-03 | 9.165 | 723 | - |
| 591 | ENSP00000301887 | BATF2 | 1.155E-03 | 8.674 | 723 | - |
| 592 | ENSP00000367494 | SPIRE2 | 1.734E-02 | 9.028 | 722 | - |
| 593 | ENSP00000387266 | SPIRE1 | 1.995E-02 | 9.013 | 722 | - |
| 594 | ENSP00000363779 | IKBKAP | 1.668E-03 | 4.485 | 722 | - |
| 595 | ENSP00000264316 | TXK | 6.198E-04 | 3.133 | 721 | - |
| 596 | ENSP00000316228 | CLEC4M | 1.556E-04 | 2.594 | 721 | - |
| 597 | ENSP00000356024 | CR2 | 1.474E-04 | 5.631 | 720 | - |
| 598 | ENSP00000328111 | IL25 | 3.306E-04 | 2.807 | 720 | - |
| 599 | ENSP00000408146 | HLA-DPB1 | 2.577E-04 | 2.793 | 720 | - |
| 600 | ENSP00000261681 | MPP5 | 1.459E-03 | 4.124 | 717 | - |
| 601 | ENSP00000395498 | PAX8 | 1.014E-03 | 8.578 | 708 | - |
| 602 | ENSP00000378485 | MATK | 5.888E-04 | 3.772 | 707 | - |
| 603 | ENSP00000399511 | TNIK | 1.125E-03 | 5.846 | 705 | - |
| 604 | ENSP00000299440 | RAG1 | 1.367E-04 | 2.276 | 705 | - |
| 605 | ENSP00000239243 | MSX2 | 1.851E-03 | 4.403 | 704 | - |
| 606 | ENSP00000215727 | SERPIND1 | 1.189E-04 | 3.412 | 704 | - |
| 607 | ENSP00000350844 | PAX5 | 1.627E-03 | 8.968 | 702 | - |
| 608 | ENSP00000284486 | FAM167A | 2.285E-04 | 2.078 | 700 | - |
| 609 | ENSP00000259271 | GAD2 | 9.585E-04 | 2.018 | 700 | - |
| 610 | ENSP00000311538 | ARR3 | 6.286E-03 | 4.373 | 698 | - |
| 611 | ENSP00000375777 | STRN4 | 6.437E-03 | 9.696 | 697 | - |
| 612 | ENSP00000345785 | FZD9 | 5.556E-03 | 8.956 | 697 | - |
| 613 | ENSP00000355510 | WDR64 | 6.427E-03 | 8.987 | 693 | - |
| 614 | ENSP00000359444 | HSFX1 | 9.774E-04 | 5.154 | 693 | - |
| 615 | ENSP00000306549 | HSFY2 | 9.598E-04 | 5.120 | 693 | - |
| 616 | ENSP00000431063 | HSFX2 | 9.236E-04 | 5.105 | 693 | - |
| 617 | ENSP00000303599 | HSFY1 | 9.232E-04 | 5.094 | 693 | - |
| 618 | ENSP00000264896 | SCARB2 | 4.221E-03 | 4.389 | 692 | - |
| 619 | ENSP00000341637 | NCR3LG1 | 2.880E-04 | 5.987 | 685 | - |
| 620 | ENSP00000231121 | HAND1 | 7.906E-05 | 2.587 | 685 | - |
| 621 | ENSP00000451320 | BCL2L2-PABPN1 | 9.969E-04 | 6.195 | 684 | - |
| 622 | ENSP00000325748 | STK25 | 7.377E-04 | 2.693 | 682 | - |
| 623 | ENSP00000352672 | PGLYRP4 | 2.973E-04 | 5.497 | 680 | - |
| 624 | ENSP00000290722 | PGLYRP3 | 4.949E-04 | 4.332 | 680 | - |
| 625 | ENSP00000337144 | AQP12A | 1.486E-04 | 3.073 | 680 | - |
| 626 | ENSP00000386870 | DCDC2B | 4.442E-02 | 9.049 | 677 | - |
| 627 | ENSP00000375986 | MAP3K4 | 4.580E-03 | 8.887 | 677 | - |
| 628 | ENSP00000228641 | MYF6 | 2.197E-03 | 9.091 | 676 | - |
| 629 | ENSP00000310263 | TNFRSF10D | 1.145E-02 | 4.426 | 675 | - |
| 630 | ENSP00000319756 | TENC1 | 1.093E-03 | 2.324 | 673 | - |
| 631 | ENSP00000312988 | NFKBIB | 1.279E-03 | 4.422 | 672 | - |
| 632 | ENSP00000337816 | MMP25 | 8.645E-03 | 9.865 | 670 | - |
| 633 | ENSP00000286614 | MMP16 | 8.220E-03 | 9.041 | 670 | - |
| 634 | ENSP00000246186 | MMP24 | 1.132E-02 | 9.038 | 670 | - |
| 635 | ENSP00000313437 | MMP19 | 7.552E-03 | 9.034 | 670 | - |
| 636 | ENSP00000353767 | MMP17 | 1.277E-02 | 9.012 | 670 | - |
| 637 | ENSP00000219271 | MMP15 | 8.558E-03 | 4.480 | 670 | - |
| 638 | ENSP00000248444 | VIL1 | 7.063E-05 | 2.727 | 669 | - |
| 639 | ENSP00000362413 | PGK1 | 8.621E-04 | 6.725 | 668 | - |
| 640 | ENSP00000396954 | ATF5 | 1.473E-03 | 5.157 | 668 | - |
| 641 | ENSP00000347427 | MINK1 | 3.296E-03 | 8.088 | 666 | - |
| 642 | ENSP00000236826 | MMP8 | 2.546E-03 | 4.564 | 663 | - |
| 643 | ENSP00000386378 | CD207 | 1.629E-04 | 2.476 | 662 | - |
| 644 | ENSP00000229402 | KLRB1 | 3.300E-04 | 16.376 | 659 | - |
| 645 | ENSP00000267953 | BCL2A1 | 1.540E-03 | 8.871 | 659 | - |
| 646 | ENSP00000311010 | ELF5 | 6.650E-03 | 8.788 | 659 | - |
| 647 | ENSP00000435835 | EHF | 1.285E-03 | 8.398 | 659 | - |
| 648 | ENSP00000352673 | ELF3 | 1.345E-03 | 7.087 | 659 | - |
| 649 | ENSP00000341843 | ETV7 | 8.883E-03 | 4.386 | 659 | - |
| 650 | ENSP00000311280 | ELF4 | 6.709E-03 | 4.316 | 659 | - |
| 651 | ENSP00000264202 | CAPZB | 2.651E-03 | 4.045 | 659 | - |
| 652 | ENSP00000239882 | ELF1 | 9.741E-03 | 3.949 | 659 | - |
| 653 | ENSP00000296280 | MASP1 | 1.198E-04 | 2.291 | 658 | - |
| 654 | ENSP00000353824 | PIM3 | 6.385E-04 | 3.975 | 656 | - |
| 655 | ENSP00000217188 | SRMS | 6.720E-04 | 3.053 | 652 | - |
| 656 | ENSP00000356150 | MDM4 | 3.675E-03 | 8.083 | 651 | - |
| 657 | ENSP00000262013 | SPAG9 | 3.122E-03 | 4.230 | 650 | - |
| 658 | ENSP00000383042 | KDM1A | 7.292E-04 | 4.239 | 648 | - |
| 659 | ENSP00000249887 | CCRL1 | 2.430E-04 | 2.534 | 648 | - |
| 660 | ENSP00000344666 | NF2 | 1.102E-03 | 3.090 | 647 | - |
| 661 | ENSP00000332455 | KPNA2 | 4.147E-03 | 8.813 | 646 | - |
| 662 | ENSP00000322706 | HMGCS1 | 2.576E-03 | 4.165 | 644 | - |
| 663 | ENSP00000357152 | CD1C | 2.226E-04 | 2.633 | 643 | - |
| 664 | ENSP00000369270 | RREB1 | 1.460E-03 | 4.202 | 642 | - |
| 665 | ENSP00000402084 | WEE1 | 9.103E-04 | 3.292 | 642 | - |
| 666 | ENSP00000375956 | MLLT4 | 2.398E-03 | 8.791 | 641 | - |
| 667 | ENSP00000427562 | ABL2 | 7.745E-04 | 4.647 | 640 | - |
| 668 | ENSP00000347665 | COL18A1 | 1.340E-04 | 4.147 | 639 | - |
| 669 | ENSP00000405176 | TWIST2 | 1.325E-03 | 8.870 | 638 | - |
| 670 | ENSP00000233668 | DOK1 | 1.321E-02 | 4.650 | 637 | - |
| 671 | ENSP00000259339 | TOR1B | 1.878E-04 | 3.287 | 636 | - |
| 672 | ENSP00000367248 | CD177 | 9.347E-05 | 2.932 | 635 | - |
| 673 | ENSP00000337691 | MAPK15 | 4.558E-04 | 2.756 | 634 | - |
| 674 | ENSP00000383234 | MAPK4 | 4.358E-04 | 2.276 | 634 | - |
| 675 | ENSP00000403495 | LTA | 9.734E-05 | 4.174 | 633 | - |
| 676 | ENSP00000282928 | ZIC1 | 9.497E-04 | 3.271 | 632 | - |
| 677 | ENSP00000349477 | KLRG1 | 2.516E-04 | 8.221 | 631 | - |
| 678 | ENSP00000311545 | EMR1 | 1.531E-04 | 3.760 | 631 | - |
| 679 | ENSP00000371505 | CLEC6A | 1.918E-04 | 6.231 | 623 | - |
| 680 | ENSP00000265983 | HPX | 2.934E-03 | 8.809 | 618 | - |
| 681 | ENSP00000309132 | BCL2L14 | 3.123E-03 | 8.757 | 614 | - |
| 682 | ENSP00000295113 | FRZB | 9.096E-04 | 8.703 | 614 | - |
| 683 | ENSP00000301264 | DAPK3 | 1.117E-03 | 4.093 | 614 | - |
| 684 | ENSP00000298510 | PRDX3 | 7.833E-04 | 3.134 | 613 | - |
| 685 | ENSP00000291744 | FCN2 | 1.085E-04 | 2.025 | 613 | - |
| 686 | ENSP00000260229 | MMP27 | 3.100E-02 | 9.106 | 609 | - |
| 687 | ENSP00000300762 | MMP26 | 1.936E-02 | 9.038 | 609 | - |
| 688 | ENSP00000357798 | MMP21 | 2.764E-02 | 9.037 | 609 | - |
| 689 | ENSP00000341762 | BSPH1 | 2.244E-02 | 9.020 | 609 | - |
| 690 | ENSP00000250144 | MMP28 | 1.507E-02 | 9.019 | 609 | - |
| 691 | ENSP00000260228 | MMP20 | 1.175E-02 | 8.961 | 609 | - |
| 692 | ENSP00000452347 | CTB-96E2.2 | 5.056E-03 | 8.958 | 609 | - |
| 693 | ENSP00000399679 | PRG4 | 1.743E-03 | 8.724 | 609 | - |
| 694 | ENSP00000470087 | AC006486.9 | 8.065E-04 | 5.196 | 609 | - |
| 695 | ENSP00000340660 | ELSPBP1 | 5.731E-03 | 4.129 | 609 | - |
| 696 | ENSP00000349238 | CCDC88B | 1.422E-03 | 8.398 | 607 | - |
| 697 | ENSP00000355778 | H3F3A | 1.353E-03 | 7.487 | 607 | - |
| 698 | ENSP00000363571 | MUSK | 7.720E-04 | 3.900 | 607 | - |
| 699 | ENSP00000333568 | PRKD1 | 2.078E-03 | 4.104 | 604 | - |
| 700 | ENSP00000291860 | KIR3DL3 | 5.586E-03 | 12.762 | 603 | - |
| 701 | ENSP00000221740 | CASP14 | 1.214E-02 | 4.492 | 603 | - |
| 702 | ENSP00000219700 | HMOX2 | 1.130E-03 | 3.986 | 603 | - |
| 703 | ENSP00000263895 | RND3 | 3.537E-04 | 2.833 | 603 | - |
| 704 | ENSP00000327246 | VIPR1 | 4.418E-03 | 9.067 | 602 | - |
| 705 | ENSP00000359073 | VAV3 | 4.367E-03 | 8.947 | 598 | - |
| 706 | ENSP00000247161 | ELK1 | 3.798E-02 | 9.035 | 591 | - |
| 707 | ENSP00000414334 | TK2 | 8.360E-04 | 2.294 | 591 | - |
| 708 | ENSP00000350032 | FRMD4A | 4.322E-03 | 8.761 | 590 | - |
| 709 | ENSP00000356311 | C1orf106 | 5.423E-03 | 7.846 | 590 | - |
| 710 | ENSP00000353654 | COL4A2 | 2.303E-03 | 7.671 | 590 | - |
| 711 | ENSP00000381549 | FRMD4B | 4.324E-03 | 7.057 | 590 | - |
| 712 | ENSP00000298527 | CLEC1B | 1.109E-04 | 2.999 | 590 | - |
| 713 | ENSP00000416445 | CCDC120 | 1.454E-03 | 2.703 | 590 | - |
| 714 | ENSP00000401437 | LDB3 | 9.863E-04 | 7.458 | 589 | - |
| 715 | ENSP00000377910 | DUSP14 | 2.121E-03 | 6.894 | 589 | - |
| 716 | ENSP00000288986 | NCK1 | 1.172E-03 | 4.059 | 589 | - |
| 717 | ENSP00000284637 | SH3RF1 | 1.969E-02 | 4.473 | 585 | - |
| 718 | ENSP00000357306 | RIT1 | 3.262E-04 | 2.882 | 585 | - |
| 719 | ENSP00000333919 | BTLA | 4.212E-04 | 8.775 | 583 | - |
| 720 | ENSP00000381253 | RGPD1 | 9.751E-04 | 7.547 | 583 | - |
| 721 | ENSP00000386588 | RGPD3 | 9.714E-04 | 6.930 | 583 | - |
| 722 | ENSP00000016946 | RGPD5 | 9.055E-04 | 3.741 | 583 | - |
| 723 | ENSP00000306637 | RGPD8 | 9.432E-04 | 3.509 | 583 | - |
| 724 | ENSP00000330842 | RGPD6 | 8.706E-04 | 3.306 | 583 | - |
| 725 | ENSP00000392762 | DCT | 1.266E-03 | 9.052 | 582 | - |
| 726 | ENSP00000344844 | HAVCR1 | 1.575E-04 | 2.347 | 582 | - |
| 727 | ENSP00000027335 | CDH17 | 5.126E-04 | 8.748 | 581 | - |
| 728 | ENSP00000265598 | LAMP3 | 2.267E-04 | 6.473 | 581 | - |
| 729 | ENSP00000340278 | PARK7 | 9.457E-04 | 3.252 | 581 | - |
| 730 | ENSP00000246062 | MKKS | 4.329E-04 | 2.864 | 581 | - |
| 731 | ENSP00000448689 | MAP3K12 | 1.907E-03 | 1.995 | 581 | - |
| 732 | ENSP00000314810 | EDDM3B | 3.430E-03 | 8.985 | 580 | - |
| 733 | ENSP00000364859 | CARD17 | 8.343E-03 | 8.891 | 580 | - |
| 734 | ENSP00000264357 | GRIA3 | 4.953E-03 | 8.686 | 580 | - |
| 735 | ENSP00000282499 | GRIA4 | 4.868E-03 | 8.639 | 580 | - |
| 736 | ENSP00000264426 | GRIA2 | 2.603E-03 | 8.361 | 580 | - |
| 737 | ENSP00000363387 | PDIK1L | 3.346E-03 | 8.710 | 576 | - |
| 738 | ENSP00000228741 | ELK3 | 2.262E-02 | 4.423 | 576 | - |
| 739 | ENSP00000365318 | NFKBIL1 | 2.048E-04 | 3.951 | 575 | - |
| 740 | ENSP00000314299 | CFHR1 | 1.709E-04 | 4.473 | 574 | - |
| 741 | ENSP00000344789 | ACACA | 3.560E-04 | 2.385 | 573 | - |
| 742 | ENSP00000353500 | CLEC4C | 2.165E-04 | 10.149 | 569 | - |
| 743 | ENSP00000356395 | CFHR3 | 2.219E-04 | 4.025 | 569 | - |
| 744 | ENSP00000315997 | LILRB1 | 3.572E-03 | 11.442 | 567 | - |
| 745 | ENSP00000221138 | PPP2CB | 3.980E-04 | 3.561 | 567 | - |
| 746 | ENSP00000355652 | RHOU | 3.631E-04 | 3.412 | 566 | - |
| 747 | ENSP00000299663 | CLEC4E | 3.149E-04 | 8.899 | 565 | - |
| 748 | ENSP00000355629 | COG2 | 3.690E-03 | 8.813 | 565 | - |
| 749 | ENSP00000369460 | ELAVL2 | 2.046E-03 | 6.950 | 565 | - |
| 750 | ENSP00000447173 | VDR | 9.003E-04 | 4.756 | 564 | - |
| 751 | ENSP00000263321 | TYR | 1.516E-03 | 4.408 | 564 | - |
| 752 | ENSP00000379566 | CCHCR1 | 1.138E-04 | 3.086 | 564 | - |
| 753 | ENSP00000308576 | RHOD | 3.553E-04 | 2.602 | 563 | - |
| 754 | ENSP00000368020 | KIF3A | 1.024E-03 | 6.989 | 562 | - |
| 755 | ENSP00000357393 | EFNA3 | 1.787E-04 | 3.530 | 562 | - |
| 756 | ENSP00000417404 | HFE | 1.278E-03 | 6.939 | 561 | - |
| 757 | ENSP00000301671 | GHDC | 1.033E-04 | 2.854 | 561 | - |
| 758 | ENSP00000335044 | ARID2 | 6.978E-04 | 2.818 | 560 | - |
| 759 | ENSP00000359866 | BMP5 | 2.913E-03 | 9.033 | 559 | - |
| 760 | ENSP00000427926 | RHOBTB2 | 6.475E-04 | 4.582 | 559 | - |
| 761 | ENSP00000466680 | RND2 | 6.653E-04 | 4.336 | 559 | - |
| 762 | ENSP00000308461 | RND1 | 3.668E-04 | 3.671 | 559 | - |
| 763 | ENSP00000371219 | RHOH | 3.756E-04 | 3.309 | 559 | - |
| 764 | ENSP00000369318 | RHOBTB3 | 3.895E-04 | 3.202 | 559 | - |
| 765 | ENSP00000220507 | RHOV | 3.912E-04 | 3.053 | 559 | - |
| 766 | ENSP00000338671 | RHOBTB1 | 3.849E-04 | 2.418 | 559 | - |
| 767 | ENSP00000239032 | PRLHR | 1.040E-04 | 2.037 | 557 | - |
| 768 | ENSP00000381895 | SMCO3 | 6.276E+01 | 1880.041 | 549 | - |
| 769 | ENSP00000318868 | SHMT1 | 9.773E-04 | 3.473 | 549 | - |
| 770 | ENSP00000384345 | GPR1 | 1.563E-04 | 2.341 | 547 | - |
| 771 | ENSP00000233154 | NCK2 | 3.142E-03 | 4.154 | 546 | - |
| 772 | ENSP00000462664 | POLI | 8.769E-04 | 3.017 | 543 | - |
| 773 | ENSP00000288943 | DUSP2 | 1.729E-03 | 4.249 | 542 | - |
| 774 | ENSP00000379500 | UEVLD | 3.993E-04 | 2.772 | 540 | - |
| 775 | ENSP00000289104 | MRAS | 4.209E-04 | 2.707 | 539 | - |
| 776 | ENSP00000262768 | TIMP2 | 2.534E-03 | 9.227 | 538 | - |
| 777 | ENSP00000075503 | STYK1 | 7.018E-04 | 3.093 | 538 | - |
| 778 | ENSP00000259633 | CD72 | 1.998E-04 | 4.967 | 537 | - |
| 779 | ENSP00000352162 | ELAVL3 | 2.055E-03 | 7.092 | 536 | - |
| 780 | ENSP00000382392 | NOP2 | 4.579E-03 | 8.525 | 534 | - |
| 781 | ENSP00000462196 | RP11-403P17.5 | 1.056E-03 | 8.347 | 530 | - |
| 782 | ENSP00000259212 | IL36RN | 5.304E-04 | 5.498 | 530 | - |
| 783 | ENSP00000346599 | STYX | 1.982E-03 | 4.790 | 530 | - |
| 784 | ENSP00000264093 | DGUOK | 7.435E-04 | 4.052 | 530 | - |
| 785 | ENSP00000269321 | ARHGDIA | 1.578E-03 | 3.853 | 530 | - |
| 786 | ENSP00000340609 | DUPD1 | 2.027E-03 | 8.185 | 528 | - |
| 787 | ENSP00000374309 | LAMA1 | 4.996E-03 | 8.978 | 525 | - |
| 788 | ENSP00000273550 | FTH1 | 8.235E-04 | 3.423 | 525 | - |
| 789 | ENSP00000008938 | PGLYRP1 | 2.136E-04 | 5.067 | 518 | - |
| 790 | ENSP00000319308 | RGS5 | 8.567E-05 | 3.540 | 514 | - |
| 791 | ENSP00000250378 | CMA1 | 8.768E-05 | 3.170 | 514 | - |
| 792 | ENSP00000233146 | MSH2 | 6.116E-04 | 2.549 | 514 | - |
| 793 | ENSP00000359013 | TNFRSF6B | 1.132E-04 | 2.455 | 514 | - |
| 794 | ENSP00000232496 | TUSC2 | 5.567E-02 | 9.034 | 512 | - |
| 795 | ENSP00000260950 | MSTN | 2.331E-03 | 4.364 | 512 | - |
| 796 | ENSP00000349709 | RAET1E | 1.184E-04 | 2.986 | 512 | - |
| 797 | ENSP00000454071 | LDLR | 9.723E-04 | 8.494 | 510 | - |
| 798 | ENSP00000342828 | NKX2-3 | 1.266E-04 | 3.964 | 508 | - |
| 799 | ENSP00000333952 | LCE5A | 1.520E-04 | 2.897 | 508 | - |
| 800 | ENSP00000291232 | TNFRSF13C | 1.422E-04 | 2.096 | 508 | - |
| 801 | ENSP00000326159 | KLK4 | 7.759E-05 | 2.205 | 507 | - |
| 802 | ENSP00000312702 | DEFB114 | 1.268E-03 | 6.373 | 506 | - |
| 803 | ENSP00000300515 | GOLGA6L9 | 1.566E-04 | 2.930 | 506 | - |
| 804 | ENSP00000367668 | MMEL1 | 1.119E-04 | 2.374 | 506 | - |
| 805 | ENSP00000349594 | ELAVL4 | 2.031E-03 | 7.504 | 505 | - |
| 806 | ENSP00000348912 | ADAM33 | 2.284E-04 | 5.416 | 505 | - |
| 807 | ENSP00000356489 | EPM2A | 8.073E-04 | 4.756 | 503 | - |
| 808 | ENSP00000317619 | LACC1 | 1.750E-04 | 4.120 | 503 | - |
| 809 | ENSP00000233997 | AZU1 | 8.395E-05 | 3.315 | 503 | - |
| 810 | ENSP00000366271 | KDM2B | 8.896E-04 | 6.579 | 501 | - |
| 811 | ENSP00000339136 | ERAS | 4.063E-04 | 2.538 | 501 | - |
| 812 | ENSP00000272134 | LEFTY1 | 3.354E-03 | 9.012 | 497 | - |
| 813 | ENSP00000271385 | DUSP27 | 2.403E-03 | 8.288 | 497 | - |
| 814 | ENSP00000256261 | DUSP26 | 1.966E-03 | 8.255 | 497 | - |
| 815 | ENSP00000248600 | STYXL1 | 1.995E-03 | 7.748 | 497 | - |
| 816 | ENSP00000452702 | DUSP13 | 1.154E-03 | 6.863 | 497 | - |
| 817 | ENSP00000312081 | SSH3 | 1.898E-03 | 3.815 | 497 | - |
| 818 | ENSP00000300574 | CRK | 1.074E-03 | 3.109 | 497 | - |
| 819 | ENSP00000220062 | RASL12 | 3.046E-04 | 2.861 | 493 | - |
| 820 | ENSP00000279488 | DUSP6 | 1.403E-03 | 3.189 | 491 | - |
| 821 | ENSP00000351473 | GIMAP5 | 3.224E-04 | 2.473 | 489 | - |
| 822 | ENSP00000370719 | ITSN1 | 1.055E-03 | 7.064 | 487 | - |
| 823 | ENSP00000284311 | GPR15 | 3.260E-04 | 5.024 | 485 | - |
| 824 | ENSP00000247219 | TBPL2 | 6.387E-04 | 3.167 | 484 | - |
| 825 | ENSP00000274625 | FGF18 | 9.740E-05 | 2.690 | 484 | - |
| 826 | ENSP00000426225 | RP11-542P2.1 | 1.637E-04 | 4.572 | 483 | - |
| 827 | ENSP00000347244 | ITSN2 | 1.571E-03 | 8.038 | 482 | - |
| 828 | ENSP00000228463 | SELPLG | 9.178E-05 | 2.941 | 482 | - |
| 829 | ENSP00000261304 | GALC | 1.091E-04 | 2.452 | 482 | - |
| 830 | ENSP00000250693 | ART1 | 3.225E-04 | 7.416 | 478 | - |
| 831 | ENSP00000456533 | MFRP | 1.354E-03 | 8.500 | 476 | - |
| 832 | ENSP00000391664 | MFRP | 1.225E-03 | 8.171 | 476 | - |
| 833 | ENSP00000363489 | GDF5 | 2.230E-03 | 8.984 | 474 | - |
| 834 | ENSP00000264487 | AREG | 2.989E-03 | 4.437 | 472 | - |
| 835 | ENSP00000424838 | DDX4 | 8.381E-04 | 3.391 | 472 | - |
| 836 | ENSP00000364589 | CUL4A | 9.977E-04 | 3.561 | 471 | - |
| 837 | ENSP00000287139 | NODAL | 3.005E-03 | 8.987 | 470 | - |
| 838 | ENSP00000258052 | SMPD2 | 3.820E-03 | 4.311 | 470 | - |
| 839 | ENSP00000222002 | SULT2A1 | 5.790E-05 | 1.972 | 470 | - |
| 840 | ENSP00000437409 | GRAPL | 1.161E-03 | 7.721 | 469 | - |
| 841 | ENSP00000353508 | MAP2 | 2.529E-03 | 8.594 | 468 | - |
| 842 | ENSP00000364543 | SKIV2L | 2.933E-03 | 8.152 | 465 | - |
| 843 | ENSP00000357178 | INSRR | 4.552E-04 | 3.687 | 464 | - |
| 844 | ENSP00000284154 | GRAP | 2.081E-03 | 4.034 | 463 | - |
| 845 | ENSP00000368759 | NEDD9 | 3.829E-03 | 8.646 | 461 | - |
| 846 | ENSP00000243924 | PI3 | 7.037E-04 | 8.967 | 460 | - |
| 847 | ENSP00000356308 | ULBP3 | 1.440E-04 | 3.456 | 459 | - |
| 848 | ENSP00000348089 | ERCC6 | 1.438E-03 | 7.413 | 457 | - |
| 849 | ENSP00000356320 | ULBP2 | 1.981E-04 | 4.655 | 457 | - |
| 850 | ENSP00000390637 | SLC14A1 | 1.481E-03 | 7.983 | 456 | - |
| 851 | ENSP00000253452 | COX4I1 | 2.356E-03 | 4.229 | 456 | - |
| 852 | ENSP00000295980 | IL17RE | 2.339E-04 | 4.168 | 450 | - |
| 853 | ENSP00000224337 | BLNK | 1.258E-04 | 2.269 | 449 | - |
| 854 | ENSP00000262464 | FBN2 | 5.219E-03 | 4.452 | 445 | - |
| 855 | ENSP00000282701 | BMP3 | 3.628E-03 | 9.065 | 443 | - |
| 856 | ENSP00000228438 | CLEC2B | 1.054E-04 | 4.512 | 440 | - |
| 857 | ENSP00000277903 | HABP2 | 1.519E-04 | 3.100 | 440 | - |
| 858 | ENSP00000201979 | REM1 | 2.604E-04 | 3.037 | 440 | - |
| 859 | ENSP00000349967 | CCRL2 | 2.812E-04 | 5.911 | 439 | - |
| 860 | ENSP00000216780 | PCK2 | 1.860E-03 | 4.200 | 439 | - |
| 861 | ENSP00000346155 | UCKL1 | 7.248E-04 | 2.887 | 439 | - |
| 862 | ENSP00000221496 | AMH | 1.555E-03 | 9.190 | 438 | - |
| 863 | ENSP00000291906 | PKN3 | 5.513E-04 | 3.811 | 437 | - |
| 864 | ENSP00000382004 | CTNND1 | 1.883E-03 | 7.929 | 434 | - |
| 865 | ENSP00000435412 | RPS6KA1 | 7.428E-04 | 4.284 | 431 | - |
| 866 | ENSP00000281821 | EPHA4 | 4.701E-04 | 3.010 | 426 | - |
| 867 | ENSP00000393198 | HES4 | 1.283E-03 | 7.175 | 424 | - |
| 868 | ENSP00000419970 | POR | 9.950E-04 | 5.259 | 423 | - |
| 869 | ENSP00000254066 | RARA | 9.367E-04 | 3.727 | 422 | - |
| 870 | ENSP00000268605 | NOL3 | 2.573E-03 | 9.072 | 420 | - |
| 871 | ENSP00000365817 | HLA-E | 1.525E-04 | 2.653 | 420 | - |
| 872 | ENSP00000251535 | ALOX12 | 1.084E-02 | 9.041 | 418 | - |
| 873 | ENSP00000356505 | NCF2 | 4.477E-03 | 8.588 | 418 | - |
| 874 | ENSP00000353656 | CDH4 | 5.526E-03 | 8.425 | 418 | - |
| 875 | ENSP00000468977 | FLT3LG | 1.142E-04 | 4.910 | 418 | - |
| 876 | ENSP00000240618 | KLRK1 | 1.635E-04 | 3.657 | 418 | - |
| 877 | ENSP00000299272 | SPIC | 1.838E-04 | 3.178 | 417 | - |
| 878 | ENSP00000262101 | MSR1 | 5.071E-03 | 9.158 | 416 | - |
| 879 | ENSP00000346437 | ATG7 | 1.914E-03 | 5.539 | 414 | - |
| 880 | ENSP00000306190 | SLAMF1 | 3.912E-03 | 4.566 | 414 | - |
| 881 | ENSP00000297268 | COL1A2 | 1.366E-03 | 4.283 | 414 | - |
| 882 | ENSP00000348074 | CLEC9A | 2.071E-04 | 4.186 | 414 | - |
| 883 | ENSP00000253754 | PDLIM4 | 1.360E-03 | 4.096 | 414 | - |
| 884 | ENSP00000329904 | IQCF2 | 7.954E-04 | 3.165 | 414 | - |
| 885 | ENSP00000263798 | TYRO3 | 3.614E-04 | 2.910 | 414 | - |
| 886 | ENSP00000275815 | EPHA1 | 4.605E-04 | 2.963 | 413 | - |
| 887 | ENSP00000337850 | KPTN | 3.119E-02 | 9.052 | 412 | - |
| 888 | ENSP00000402697 | CERS1 | 1.120E-03 | 6.614 | 412 | - |
| 889 | ENSP00000280362 | PTS | 3.206E-03 | 4.323 | 412 | - |
| 890 | ENSP00000343925 | ESR2 | 8.262E-04 | 3.315 | 410 | - |
| 891 | ENSP00000283228 | PTPRR | 2.571E-03 | 8.908 | 409 | - |
| 892 | ENSP00000350012 | ACSL3 | 2.572E-03 | 7.696 | 408 | - |
| 893 | ENSP00000352458 | PLGLB2 | 1.421E-04 | 4.872 | 408 | - |
| 894 | ENSP00000397297 | NTF3 | 1.194E-03 | 8.914 | 407 | - |
| 895 | ENSP00000271651 | CTSK | 1.054E-04 | 3.638 | 407 | - |
| 896 | ENSP00000329890 | DEFA5 | 1.290E-04 | 3.537 | 407 | - |
| 897 | ENSP00000271532 | FCRL4 | 1.662E-04 | 3.431 | 407 | - |
| 898 | ENSP00000357726 | S100A12 | 1.206E-04 | 2.443 | 407 | - |
| 899 | ENSP00000287020 | GDF6 | 3.668E-03 | 9.005 | 406 | - |
| 900 | ENSP00000366534 | FOXH1 | 1.024E-03 | 4.444 | 406 | - |
| 901 | ENSP00000278412 | SSRP1 | 1.113E-03 | 2.800 | 405 | - |
| 902 | ENSP00000354218 | PPP1R13L | 1.037E-03 | 7.014 | 404 | - |
| 903 | ENSP00000244137 | PEPD | 8.936E-04 | 4.318 | 404 | - |
| 904 | ENSP00000224605 | GDF10 | 4.360E-03 | 9.036 | 402 | - |
| 905 | ENSP00000272224 | GDF7 | 4.390E-03 | 9.030 | 402 | - |
| 906 | ENSP00000257868 | GDF11 | 2.800E-03 | 9.030 | 402 | - |
| 907 | ENSP00000295379 | BMP10 | 3.857E-03 | 9.028 | 402 | - |
| 908 | ENSP00000249598 | GDF2 | 5.736E-03 | 9.024 | 402 | - |
| 909 | ENSP00000327440 | BMP8A | 5.457E-03 | 9.003 | 402 | - |
| 910 | ENSP00000252677 | BMP15 | 3.809E-03 | 8.999 | 402 | - |
| 911 | ENSP00000296875 | GDF9 | 2.496E-03 | 8.995 | 402 | - |
| 912 | ENSP00000361915 | BMP8B | 3.951E-03 | 8.816 | 402 | - |
| 913 | ENSP00000382791 | GRIK1 | 3.478E-03 | 3.309 | 400 | - |
| 914 | ENSP00000276072 | TAF1 | 4.934E-04 | 3.172 | 400 | - |
| 915 | ENSP00000362183 | GRIK3 | 7.037E-03 | 8.949 | 398 | - |
| 916 | ENSP00000332296 | RARB | 3.950E-03 | 9.015 | 397 | - |
| 917 | ENSP00000377947 | RARG | 7.610E-03 | 8.978 | 397 | - |
| 918 | ENSP00000225396 | TADA2A | 8.412E-04 | 8.796 | 397 | - |
| 919 | ENSP00000309186 | SH3RF3 | 4.761E-03 | 4.278 | 397 | - |
| 920 | ENSP00000458537 | CTRL | 1.168E-03 | 2.026 | 396 | - |
| 921 | ENSP00000253673 | EMR3 | 1.601E-04 | 4.189 | 395 | - |
| 922 | ENSP00000350928 | GAD1 | 1.042E-03 | 7.741 | 394 | - |
| 923 | ENSP00000276925 | CDKN2B | 7.934E-04 | 4.201 | 394 | - |
| 924 | ENSP00000389870 | DLX3 | 1.237E-03 | 8.410 | 393 | - |
| 925 | ENSP00000363763 | EPHB2 | 4.231E-04 | 3.576 | 393 | - |
| 926 | ENSP00000261683 | ZBTB25 | 1.440E-03 | 2.956 | 392 | - |
| 927 | ENSP00000395253 | MUC19 | 1.441E-04 | 2.806 | 392 | - |
| 928 | ENSP00000263800 | LTK | 1.032E-04 | 2.785 | 392 | - |
| 929 | ENSP00000264051 | NGEF | 2.972E-03 | 8.895 | 387 | - |
| 930 | ENSP00000383396 | DEFB116 | 7.188E-05 | 4.904 | 386 | - |
| 931 | ENSP00000349156 | SIAH1 | 2.228E-03 | 4.563 | 386 | - |
| 932 | ENSP00000427015 | RPS6KA2 | 7.482E-04 | 6.401 | 383 | - |
| 933 | ENSP00000192314 | GAL3ST2 | 2.794E-04 | 3.041 | 381 | - |
| 934 | ENSP00000259205 | IL36G | 5.765E-04 | 10.108 | 380 | - |
| 935 | ENSP00000382544 | TSSK2 | 5.570E-04 | 3.606 | 380 | - |
| 936 | ENSP00000273857 | CORIN | 5.288E-03 | 8.975 | 379 | - |
| 937 | ENSP00000438378 | NRK | 1.577E-03 | 8.191 | 378 | - |
| 938 | ENSP00000366682 | IRG1 | 1.166E-04 | 3.281 | 378 | - |
| 939 | ENSP00000243222 | COL10A1 | 8.322E-03 | 9.049 | 375 | - |
| 940 | ENSP00000305503 | NUP62 | 2.879E-03 | 3.657 | 375 | - |
| 941 | ENSP00000364979 | COL4A1 | 2.031E-03 | 8.039 | 373 | - |
| 942 | ENSP00000386213 | MYO3B | 1.569E-03 | 4.443 | 373 | - |
| 943 | ENSP00000356448 | TPR | 1.735E-03 | 7.321 | 372 | - |
| 944 | ENSP00000304408 | COL3A1 | 1.893E-03 | 9.111 | 371 | - |
| 945 | ENSP00000265944 | MYO3A | 1.232E-03 | 3.095 | 371 | - |
| 946 | ENSP00000250559 | RAP1B | 3.848E-04 | 2.772 | 371 | - |
| 947 | ENSP00000302640 | UBTF | 1.620E-03 | 3.403 | 370 | - |
| 948 | ENSP00000245457 | PTGER2 | 1.348E-04 | 2.709 | 366 | - |
| 949 | ENSP00000375081 | TSSK1B | 6.066E-04 | 3.858 | 362 | - |
| 950 | ENSP00000307272 | RPTOR | 1.475E-03 | 4.093 | 360 | - |
| 951 | ENSP00000301149 | GPD1 | 1.960E-03 | 3.695 | 360 | - |
| 952 | ENSP00000363435 | ITPR3 | 2.043E-03 | 8.781 | 359 | - |
| 953 | ENSP00000354040 | GTPBP4 | 7.325E-04 | 6.188 | 358 | - |
| 954 | ENSP00000318944 | TRAF7 | 8.260E-04 | 3.709 | 357 | - |
| 955 | ENSP00000256797 | ERN2 | 6.843E-04 | 2.967 | 357 | - |
| 956 | ENSP00000326110 | C5orf30 | 2.376E-04 | 2.772 | 357 | - |
| 957 | ENSP00000299022 | LIPC | 7.714E-05 | 2.123 | 357 | - |
| 958 | ENSP00000262077 | NUP153 | 2.561E-03 | 3.775 | 355 | - |
| 959 | ENSP00000305714 | BMP1 | 8.718E-05 | 2.439 | 355 | - |
| 960 | ENSP00000301037 | SGK494 | 6.598E-04 | 8.420 | 354 | - |
| 961 | ENSP00000431165 | RP11-192H23.4 | 8.476E-04 | 8.410 | 354 | - |
| 962 | ENSP00000452401 | RP11-47I22.4 | 8.489E-04 | 8.394 | 354 | - |
| 963 | ENSP00000294964 | PKDCC | 5.862E-04 | 4.151 | 354 | - |
| 964 | ENSP00000355260 | PPP1R14C | 1.426E-04 | 3.042 | 351 | - |
| 965 | ENSP00000361725 | ENDOG | 3.657E-03 | 8.861 | 349 | - |
| 966 | ENSP00000369042 | IPO7 | 9.998E-04 | 7.003 | 349 | - |
| 967 | ENSP00000304447 | PTCRA | 3.680E-03 | 4.323 | 349 | - |
| 968 | ENSP00000321805 | RIT2 | 3.374E-04 | 2.335 | 349 | - |
| 969 | ENSP00000347717 | DHCR7 | 2.219E-03 | 7.907 | 347 | - |
| 970 | ENSP00000246949 | DNASE1 | 1.271E-04 | 4.770 | 347 | - |
| 971 | ENSP00000161559 | CEACAM1 | 1.352E-04 | 5.234 | 345 | - |
| 972 | ENSP00000264001 | CKLF | 1.452E-02 | 4.640 | 345 | - |
| 973 | ENSP00000346294 | S100A4 | 4.013E-03 | 8.992 | 343 | - |
| 974 | ENSP00000316845 | ARHGEF4 | 4.870E-03 | 8.851 | 343 | - |
| 975 | ENSP00000354573 | MACF1 | 1.233E-03 | 7.668 | 343 | - |
| 976 | ENSP00000321746 | PDLIM5 | 7.521E-04 | 7.237 | 343 | - |
| 977 | ENSP00000419325 | SUCLG2 | 8.384E-04 | 6.036 | 343 | - |
| 978 | ENSP00000311035 | CD200R1 | 2.775E-04 | 4.618 | 343 | - |
| 979 | ENSP00000443411 | FAM92B | 9.844E-05 | 2.518 | 343 | - |
| 980 | ENSP00000359345 | RPL5 | 5.927E-04 | 5.519 | 341 | - |
| 981 | ENSP00000216274 | RIPK3 | 7.169E-04 | 3.103 | 341 | - |
| 982 | ENSP00000266674 | LGR5 | 5.909E-04 | 2.905 | 341 | - |
| 983 | ENSP00000301522 | PRDX2 | 7.078E-04 | 2.756 | 341 | - |
| 984 | ENSP00000282538 | GADL1 | 4.081E-03 | 8.620 | 339 | - |
| 985 | ENSP00000267085 | CSAD | 3.851E-03 | 8.519 | 339 | - |
| 986 | ENSP00000352028 | SH3RF2 | 1.295E-02 | 8.975 | 337 | - |
| 987 | ENSP00000381237 | HM13 | 5.788E-04 | 2.900 | 337 | - |
| 988 | ENSP00000362481 | UPRT | 1.068E-03 | 3.897 | 333 | - |
| 989 | ENSP00000361120 | RALGDS | 3.825E-03 | 8.858 | 330 | - |
| 990 | ENSP00000259213 | IL36B | 3.925E-04 | 6.067 | 330 | - |
| 991 | ENSP00000335384 | ZPBP2 | 2.227E-04 | 2.226 | 330 | - |
| 992 | ENSP00000443189 | AC073610.5 | 8.111E-04 | 5.048 | 329 | - |
| 993 | ENSP00000254868 | CLEC10A | 7.839E-05 | 2.750 | 329 | - |
| 994 | ENSP00000233954 | IL1RL1 | 2.061E-04 | 5.531 | 328 | - |
| 995 | ENSP00000394085 | JMJD6 | 1.134E-03 | 6.089 | 327 | - |
| 996 | ENSP00000410481 | LTB | 9.337E-04 | 3.516 | 327 | - |
| 997 | ENSP00000357022 | SLAMF7 | 1.644E-04 | 2.380 | 327 | - |
| 998 | ENSP00000321348 | DIAPH2 | 2.293E-03 | 4.317 | 325 | - |
| 999 | ENSP00000297001 | C7orf72 | 4.073E-04 | 2.577 | 325 | - |
| 1000 | ENSP00000037502 | MYOC | 6.470E-03 | 9.072 | 323 | - |
| 1001 | ENSP00000408994 | DET1 | 1.606E-03 | 7.441 | 323 | - |
| 1002 | ENSP00000303999 | TMEM133 | 3.544E-04 | 3.960 | 323 | - |
| 1003 | ENSP00000244869 | EREG | 8.239E-05 | 2.913 | 323 | - |
| 1004 | ENSP00000299759 | RRAD | 3.285E-04 | 2.543 | 323 | - |
| 1005 | ENSP00000329584 | ODF3L1 | 5.724E-04 | 2.223 | 323 | - |
| 1006 | ENSP00000295408 | MERTK | 5.934E-04 | 2.920 | 322 | - |
| 1007 | ENSP00000360882 | COL5A1 | 2.820E-03 | 8.674 | 320 | - |
| 1008 | ENSP00000271628 | SF3B4 | 3.867E-03 | 8.920 | 319 | - |
| 1009 | ENSP00000340998 | NTN4 | 1.819E-02 | 9.003 | 317 | - |
| 1010 | ENSP00000342520 | IL17REL | 3.029E-04 | 8.225 | 315 | - |
| 1011 | ENSP00000361014 | MKNK1 | 1.036E-02 | 9.004 | 313 | - |
| 1012 | ENSP00000350425 | APOA4 | 4.547E-03 | 8.256 | 313 | - |
| 1013 | ENSP00000234701 | CLCA1 | 1.965E-04 | 5.490 | 313 | - |
| 1014 | ENSP00000303398 | MTBP | 2.862E-02 | 4.527 | 313 | - |
| 1015 | ENSP00000303366 | LMAN2 | 9.401E-03 | 4.266 | 313 | - |
| 1016 | ENSP00000253255 | PKDREJ | 2.079E-04 | 4.250 | 313 | - |
| 1017 | ENSP00000199764 | CEACAM6 | 1.364E-04 | 3.761 | 313 | - |
| 1018 | ENSP00000256412 | ADAMDEC1 | 3.395E-04 | 7.203 | 311 | - |
| 1019 | ENSP00000363676 | RPL11 | 1.045E-03 | 6.402 | 311 | - |
| 1020 | ENSP00000342011 | XRCC4 | 6.526E-03 | 3.703 | 311 | - |
| 1021 | ENSP00000295755 | RETNLB | 1.021E-04 | 1.972 | 311 | - |
| 1022 | ENSP00000251412 | TUBG2 | 8.877E-04 | 4.329 | 309 | - |
| 1023 | ENSP00000251413 | TUBG1 | 7.079E-04 | 3.771 | 309 | - |
| 1024 | ENSP00000301764 | DDB1 | 1.247E-03 | 3.566 | 309 | - |
| 1025 | ENSP00000271555 | MEF2D | 6.054E-04 | 2.771 | 307 | - |
| 1026 | ENSP00000286733 | NAAA | 1.808E-04 | 4.595 | 302 | - |
| 1027 | ENSP00000229332 | CLEC4A | 2.668E-04 | 4.481 | 302 | - |
| 1028 | ENSP00000301727 | E4F1 | 1.279E-03 | 3.172 | 302 | - |
| 1029 | ENSP00000387077 | LYPD6B | 4.757E-03 | 2.110 | 302 | - |
| 1030 | ENSP00000387966 | PGBD3 | 5.307E-03 | 8.668 | 301 | - |
| 1031 | ENSP00000402355 | CDRT15 | 1.342E-04 | 3.496 | 301 | - |
| 1032 | ENSP00000289429 | CD1A | 1.898E-04 | 2.756 | 301 | - |
| 1033 | ENSP00000291890 | NCR1 | 5.820E-04 | 2.858 | 300 | - |
| 1034 | ENSP00000463999 | AURKB | 6.979E-04 | 4.217 | 299 | - |
| 1035 | ENSP00000355583 | MLK4 | 7.663E-04 | 4.254 | 298 | - |
| 1036 | ENSP00000264381 | BCHE | 4.753E-03 | 9.057 | 295 | - |
| 1037 | ENSP00000265769 | ADAM28 | 8.634E-05 | 2.376 | 295 | - |
| 1038 | ENSP00000216127 | RASD2 | 3.866E-04 | 3.045 | 293 | - |
| 1039 | ENSP00000436691 | CARD18 | 1.410E-03 | 1.997 | 292 | - |
| 1040 | ENSP00000326324 | LRRC37A | 2.072E-01 | 9.065 | 290 | - |
| 1041 | ENSP00000325713 | LRRC37A3 | 4.156E-01 | 9.051 | 290 | - |
| 1042 | ENSP00000333071 | LRRC37A2 | 1.406E-01 | 9.037 | 290 | - |
| 1043 | ENSP00000302537 | MDM1 | 6.665E-03 | 8.994 | 290 | - |
| 1044 | ENSP00000292169 | S100A1 | 7.961E-05 | 1.970 | 290 | - |
| 1045 | ENSP00000300482 | TRPM2 | 8.098E-03 | 4.403 | 288 | - |
| 1046 | ENSP00000301905 | PBK | 5.079E-04 | 2.771 | 288 | - |
| 1047 | ENSP00000293826 | TNFSF12-TNFSF13 | 1.884E-04 | 2.015 | 287 | - |
| 1048 | ENSP00000360310 | SPO11 | 1.562E-03 | 8.634 | 286 | - |
| 1049 | ENSP00000384197 | PDZRN4 | 2.220E-03 | 8.590 | 286 | - |
| 1050 | ENSP00000263666 | PDZRN3 | 2.321E-03 | 8.161 | 286 | - |
| 1051 | ENSP00000430276 | MCF2 | 9.556E-04 | 5.692 | 286 | - |
| 1052 | ENSP00000316032 | NUP98 | 2.277E-03 | 3.722 | 286 | - |
| 1053 | ENSP00000423665 | CDK18 | 6.133E-04 | 4.238 | 285 | - |
| 1054 | ENSP00000260967 | CDK15 | 3.291E-04 | 2.835 | 285 | - |
| 1055 | ENSP00000261211 | CDK17 | 3.079E-04 | 2.741 | 285 | - |
| 1056 | ENSP00000265741 | CDK14 | 2.902E-04 | 2.575 | 285 | - |
| 1057 | ENSP00000416683 | IGFALS | 1.093E-03 | 4.791 | 281 | - |
| 1058 | ENSP00000321684 | MEOX1 | 5.390E-03 | 8.936 | 280 | - |
| 1059 | ENSP00000451263 | MAP3K9 | 8.868E-04 | 5.641 | 280 | - |
| 1060 | ENSP00000345853 | DUSP9 | 2.644E-03 | 5.194 | 280 | - |
| 1061 | ENSP00000302898 | AURKC | 1.088E-03 | 3.987 | 280 | - |
| 1062 | ENSP00000354916 | CDC14A | 5.292E-04 | 4.590 | 276 | - |
| 1063 | ENSP00000322775 | RHBDF2 | 9.649E-04 | 3.950 | 273 | - |
| 1064 | ENSP00000376684 | EPHB6 | 6.965E-04 | 3.908 | 273 | - |
| 1065 | ENSP00000289968 | ARHGAP17 | 7.614E-03 | 4.386 | 271 | - |
| 1066 | ENSP00000361027 | AMOT | 6.870E-03 | 8.974 | 270 | - |
| 1067 | ENSP00000371542 | SPATA13 | 2.477E-03 | 8.315 | 270 | - |
| 1068 | ENSP00000414982 | KLC1 | 7.704E-04 | 4.949 | 270 | - |
| 1069 | ENSP00000265026 | MAP3K13 | 1.358E-02 | 4.418 | 270 | - |
| 1070 | ENSP00000362221 | STK40 | 6.425E-04 | 4.108 | 270 | - |
| 1071 | ENSP00000350336 | DSCR8 | 2.236E-02 | 9.031 | 267 | - |
| 1072 | ENSP00000328640 | C11orf72 | 8.142E-04 | 8.673 | 267 | - |
| 1073 | ENSP00000286827 | TIAM1 | 3.086E-03 | 4.344 | 266 | - |
| 1074 | ENSP00000306253 | ITPR1 | 1.493E-03 | 3.181 | 266 | - |
| 1075 | ENSP00000385643 | - | 1.285E-04 | 2.246 | 266 | - |
| 1076 | ENSP00000341071 | ARHGEF3 | 1.964E-03 | 3.689 | 265 | - |
| 1077 | ENSP00000340125 | PPP1R17 | 9.852E-05 | 2.056 | 263 | - |
| 1078 | ENSP00000351342 | PTPN5 | 2.665E-03 | 8.731 | 262 | - |
| 1079 | ENSP00000360316 | DHCR24 | 1.404E-03 | 7.918 | 261 | - |
| 1080 | ENSP00000310722 | MOS | 7.151E-04 | 4.346 | 260 | - |
| 1081 | ENSP00000421922 | LRPAP1 | 1.110E-03 | 3.729 | 260 | - |
| 1082 | ENSP00000308351 | MLKL | 6.852E-04 | 2.885 | 259 | - |
| 1083 | ENSP00000196061 | PLOD1 | 3.938E-03 | 3.887 | 258 | - |
| 1084 | ENSP00000389427 | CEP44 | 2.033E-03 | 4.635 | 256 | - |
| 1085 | ENSP00000276440 | DOCK5 | 6.641E-03 | 4.388 | 256 | - |
| 1086 | ENSP00000308413 | RPS6KB2 | 5.387E-04 | 3.670 | 256 | - |
| 1087 | ENSP00000373518 | NOX5 | 8.604E-03 | 9.006 | 255 | - |
| 1088 | ENSP00000280333 | DOCK1 | 3.384E-03 | 4.316 | 255 | - |
| 1089 | ENSP00000352424 | TIAF1 | 2.738E-02 | 9.068 | 254 | - |
| 1090 | ENSP00000452854 | MEIS3 | 6.313E-04 | 7.219 | 253 | - |
| 1091 | ENSP00000252491 | APOC1 | 7.045E-03 | 9.104 | 252 | - |
| 1092 | ENSP00000450730 | RP11-934B9.3 | 9.492E-04 | 5.480 | 250 | - |
| 1093 | ENSP00000010404 | MGST1 | 5.321E-03 | 4.363 | 250 | - |
| 1094 | ENSP00000356130 | DSTYK | 6.881E-04 | 3.989 | 250 | - |
| 1095 | ENSP00000340608 | SGK2 | 5.326E-04 | 3.554 | 250 | - |
| 1096 | ENSP00000216264 | CERK | 5.415E-03 | 3.284 | 250 | - |
| 1097 | ENSP00000221466 | FCGRT | 1.150E-04 | 2.255 | 250 | - |
| 1098 | ENSP00000333917 | DUSP18 | 2.684E-03 | 8.915 | 249 | - |
| 1099 | ENSP00000343244 | DUSP21 | 3.274E-03 | 7.970 | 249 | - |
| 1100 | ENSP00000288976 | PTPDC1 | 7.120E-04 | 7.563 | 249 | - |
| 1101 | ENSP00000357087 | DUSP23 | 7.557E-04 | 6.488 | 249 | - |
| 1102 | ENSP00000364389 | CDC14B | 7.435E-04 | 5.259 | 249 | - |
| 1103 | ENSP00000335357 | CDKN3 | 6.688E-04 | 3.812 | 249 | - |
| 1104 | ENSP00000343463 | MAP3K2 | 6.000E-04 | 2.785 | 249 | - |
| 1105 | ENSP00000295256 | HPGDS | 4.408E-03 | 9.042 | 248 | - |
| 1106 | ENSP00000333896 | RPS6KA4 | 5.545E-04 | 3.247 | 247 | - |
| 1107 | ENSP00000332371 | COL7A1 | 7.697E-05 | 2.086 | 247 | - |
| 1108 | ENSP00000262211 | - | 4.530E-04 | 3.897 | 244 | - |
| 1109 | ENSP00000369756 | PTPRA | 3.744E-03 | 8.776 | 243 | - |
| 1110 | ENSP00000287814 | TIMP4 | 2.357E-04 | 5.405 | 242 | - |
| 1111 | ENSP00000231009 | GZMK | 1.482E-04 | 5.315 | 242 | - |
| 1112 | ENSP00000301068 | RHEBL1 | 4.080E-04 | 2.477 | 242 | - |
| 1113 | ENSP00000302105 | CRYGA | 1.826E-04 | 2.419 | 242 | - |
| 1114 | ENSP00000265825 | FSCN3 | 1.028E-04 | 2.150 | 242 | - |
| 1115 | ENSP00000344420 | KRTAP1-3 | 2.248E-04 | 2.137 | 242 | - |
| 1116 | ENSP00000274643 | MYLK4 | 1.127E-03 | 8.742 | 240 | - |
| 1117 | ENSP00000472121 | AL136376.1 | 1.133E-04 | 4.753 | 240 | - |
| 1118 | ENSP00000313572 | NIM1 | 6.631E-04 | 3.691 | 240 | - |
| 1119 | ENSP00000356386 | CFHR4 | 3.599E-04 | 3.024 | 240 | - |
| 1120 | ENSP00000355470 | CNST | 1.810E-04 | 2.042 | 240 | - |
| 1121 | ENSP00000350387 | HYAL2 | 1.643E-04 | 3.744 | 239 | - |
| 1122 | ENSP00000259951 | HLA-F | 1.742E-04 | 2.796 | 238 | - |
| 1123 | ENSP00000246672 | NR1D1 | 6.967E-03 | 4.329 | 234 | - |
| 1124 | ENSP00000270452 | LILRB4 | 5.645E-04 | 4.128 | 234 | - |
| 1125 | ENSP00000319192 | STK17A | 1.150E-03 | 4.117 | 234 | - |
| 1126 | ENSP00000263980 | SLC9A1 | 1.825E-03 | 4.314 | 233 | - |
| 1127 | ENSP00000362139 | EPHA10 | 7.179E-04 | 4.148 | 232 | - |
| 1128 | ENSP00000379514 | ADCYAP1R1 | 8.290E-03 | 8.513 | 231 | - |
| 1129 | ENSP00000432743 | TP53AIP1 | 1.827E-03 | 8.458 | 227 | - |
| 1130 | ENSP00000468280 | RP11-178C3.1 | 8.652E-04 | 7.706 | 227 | - |
| 1131 | ENSP00000403536 | GAMT | 1.135E-03 | 7.613 | 227 | - |
| 1132 | ENSP00000347933 | PLGLB1 | 3.624E-04 | 5.600 | 227 | - |
| 1133 | ENSP00000355924 | SMYD2 | 1.234E-03 | 4.304 | 227 | - |
| 1134 | ENSP00000301645 | CYP7A1 | 2.417E-03 | 4.260 | 227 | - |
| 1135 | ENSP00000216101 | RASL10A | 4.379E-04 | 3.059 | 227 | - |
| 1136 | ENSP00000229002 | RERGL | 4.427E-04 | 3.055 | 227 | - |
| 1137 | ENSP00000364919 | DIRAS2 | 4.063E-04 | 3.028 | 227 | - |
| 1138 | ENSP00000248706 | RASL11B | 4.116E-04 | 2.980 | 227 | - |
| 1139 | ENSP00000267396 | REM2 | 4.620E-04 | 2.956 | 227 | - |
| 1140 | ENSP00000262752 | RPS6KA6 | 5.304E-04 | 2.675 | 227 | - |
| 1141 | ENSP00000364749 | RSG1 | 4.999E-04 | 2.671 | 227 | - |
| 1142 | ENSP00000256953 | RERG | 4.076E-04 | 2.582 | 227 | - |
| 1143 | ENSP00000325836 | DIRAS1 | 4.468E-04 | 2.572 | 227 | - |
| 1144 | ENSP00000319096 | RAP2B | 4.338E-04 | 2.398 | 227 | - |
| 1145 | ENSP00000297596 | GEM | 4.264E-04 | 2.384 | 227 | - |
| 1146 | ENSP00000307684 | TADA3 | 2.239E-03 | 8.238 | 226 | - |
| 1147 | ENSP00000295213 | SPATA18 | 4.076E-04 | 4.609 | 226 | - |
| 1148 | ENSP00000310933 | MADD | 6.692E-03 | 4.333 | 226 | - |
| 1149 | ENSP00000252785 | SCO2 | 3.120E-03 | 4.222 | 226 | - |
| 1150 | ENSP00000362634 | TSSK3 | 6.160E-04 | 3.983 | 226 | - |
| 1151 | ENSP00000349359 | SASH3 | 3.476E-04 | 2.157 | 226 | - |
| 1152 | ENSP00000284770 | PDLIM3 | 3.828E-03 | 8.920 | 225 | - |
| 1153 | ENSP00000333982 | NDEL1 | 3.335E-03 | 3.969 | 225 | - |
| 1154 | ENSP00000412457 | HLA-DMB | 1.721E-04 | 2.449 | 225 | - |
| 1155 | ENSP00000303424 | PTGDR | 3.568E-03 | 8.888 | 223 | - |
| 1156 | ENSP00000377446 | SUCLG1 | 9.026E-04 | 6.303 | 223 | - |
| 1157 | ENSP00000339179 | TSSK4 | 6.298E-04 | 4.276 | 223 | - |
| 1158 | ENSP00000217893 | TAF9 | 2.312E-03 | 3.918 | 223 | - |
| 1159 | ENSP00000354168 | TSSK6 | 5.745E-04 | 3.893 | 223 | - |
| 1160 | ENSP00000270560 | TM4SF5 | 3.309E-02 | 9.033 | 221 | - |
| 1161 | ENSP00000365312 | ABI1 | 4.882E-03 | 8.777 | 221 | - |
| 1162 | ENSP00000263816 | LRP2 | 2.513E-03 | 8.832 | 218 | - |
| 1163 | ENSP00000360608 | GRIN1 | 2.196E-03 | 8.456 | 216 | - |
| 1164 | ENSP00000362230 | RP11-268J15.5 | 6.689E-04 | 5.894 | 216 | - |
| 1165 | ENSP00000301012 | MVD | 5.643E-03 | 4.382 | 216 | - |
| 1166 | ENSP00000302938 | WFDC13 | 3.625E-04 | 3.680 | 216 | - |
| 1167 | ENSP00000316193 | DNASE1L3 | 1.126E-04 | 2.964 | 216 | - |
| 1168 | ENSP00000354929 | NOTCH2NL | 4.664E-04 | 2.738 | 216 | - |
| 1169 | ENSP00000363840 | COL11A2 | 3.427E-03 | 8.954 | 215 | - |
| 1170 | ENSP00000344936 | PTTG1 | 3.604E-03 | 8.587 | 215 | - |
| 1171 | ENSP00000439065 | KLC1 | 8.584E-04 | 5.663 | 214 | - |
| 1172 | ENSP00000256652 | CD101 | 2.762E-04 | 2.401 | 214 | - |
| 1173 | ENSP00000340995 | TEX11 | 4.551E-03 | 8.674 | 213 | - |
| 1174 | ENSP00000319574 | SLC25A19 | 5.672E-03 | 4.333 | 212 | - |
| 1175 | ENSP00000293604 | C6orf136 | 3.123E-04 | 2.338 | 212 | - |
| 1176 | ENSP00000402861 | PLCL1 | 1.200E-03 | 7.967 | 211 | - |
| 1177 | ENSP00000224181 | C8G | 2.103E-04 | 3.407 | 211 | - |
| 1178 | ENSP00000314837 | KLC2 | 1.023E-03 | 3.004 | 211 | - |
| 1179 | ENSP00000289707 | SLAMF8 | 2.253E-04 | 2.731 | 211 | - |
| 1180 | ENSP00000359603 | COL24A1 | 5.239E-03 | 8.992 | 210 | - |
| 1181 | ENSP00000379475 | DDX39B | 4.416E-03 | 5.310 | 209 | - |
| 1182 | ENSP00000371475 | TP53BP1 | 1.072E-03 | 4.908 | 209 | - |
| 1183 | ENSP00000361189 | CDHR1 | 9.130E-03 | 9.023 | 208 | - |
| 1184 | ENSP00000360305 | PDLIM1 | 2.102E-03 | 4.320 | 208 | - |
| 1185 | ENSP00000310491 | ARHGAP1 | 2.934E-03 | 4.044 | 208 | - |
| 1186 | ENSP00000221954 | CEACAM4 | 4.324E-04 | 3.994 | 208 | - |
| 1187 | ENSP00000264748 | FGFRL1 | 7.025E-04 | 4.390 | 207 | - |
| 1188 | ENSP00000251630 | PDGFRL | 7.093E-04 | 4.316 | 207 | - |
| 1189 | ENSP00000155926 | TRIB2 | 5.854E-04 | 3.045 | 207 | - |
| 1190 | ENSP00000332163 | SPRR4 | 3.114E-04 | 4.880 | 206 | - |
| 1191 | ENSP00000263955 | STK17B | 1.080E-03 | 3.952 | 206 | - |
| 1192 | ENSP00000333823 | EFHC2 | 2.303E-02 | 9.037 | 205 | - |
| 1193 | ENSP00000325929 | LNX2 | 1.478E-02 | 4.419 | 205 | - |
| 1194 | ENSP00000360281 | C8B | 1.340E-04 | 2.711 | 203 | - |
| 1195 | ENSP00000356370 | CRB1 | 9.816E-05 | 2.104 | 203 | - |
| 1196 | ENSP00000365643 | DOCK9 | 7.677E-03 | 8.975 | 202 | - |
| 1197 | ENSP00000406022 | UNC5C | 1.357E-03 | 8.141 | 201 | - |
| 1198 | ENSP00000355237 | CDC42BPB | 7.296E-04 | 3.956 | 200 | - |
| 1199 | ENSP00000411459 | ARHGEF28 | 9.432E-04 | 4.516 | 199 | - |
| 1200 | ENSP00000270288 | WTIP | 5.436E-03 | 8.994 | 198 | - |
| 1201 | ENSP00000401310 | FRG2B | 6.742E-04 | 7.887 | 198 | - |
| 1202 | ENSP00000416387 | FBLIM1 | 1.357E-03 | 6.272 | 197 | - |
| 1203 | ENSP00000318999 | C11orf16 | 2.069E-04 | 2.346 | 197 | - |
| 1204 | ENSP00000264828 | COL5A3 | 6.736E-03 | 9.002 | 193 | - |
| 1205 | ENSP00000348385 | COL27A1 | 5.403E-03 | 8.966 | 193 | - |
| 1206 | ENSP00000354526 | L3MBTL3 | 9.150E-03 | 8.762 | 191 | - |
| 1207 | ENSP00000251363 | CERS4 | 2.995E-03 | 8.538 | 190 | - |
| 1208 | ENSP00000284382 | CERS3 | 2.759E-03 | 8.448 | 190 | - |
| 1209 | ENSP00000401932 | PRSS45 | 1.046E-03 | 8.389 | 190 | - |
| 1210 | ENSP00000271688 | CERS2 | 2.491E-03 | 4.106 | 190 | - |
| 1211 | ENSP00000295314 | TMOD4 | 8.231E-05 | 2.321 | 190 | - |
| 1212 | ENSP00000218316 | GPR50 | 4.868E-03 | 9.025 | 189 | - |
| 1213 | ENSP00000409612 | ACADM | 9.534E-04 | 5.119 | 188 | - |
| 1214 | ENSP00000229829 | HLA-DOA | 5.021E-04 | 3.633 | 188 | - |
| 1215 | ENSP00000259708 | KLC4 | 1.120E-03 | 3.197 | 188 | - |
| 1216 | ENSP00000381480 | SPAG11B | 1.389E-03 | 9.390 | 187 | - |
| 1217 | ENSP00000387426 | CYHR1 | 1.692E-02 | 9.034 | 187 | - |
| 1218 | ENSP00000260766 | PLCE1 | 2.170E-03 | 4.358 | 186 | - |
| 1219 | ENSP00000386911 | ARHGAP25 | 2.443E-04 | 2.408 | 186 | - |
| 1220 | ENSP00000378611 | ARHGAP24 | 2.276E-04 | 2.388 | 186 | - |
| 1221 | ENSP00000398979 | CYB561A3 | 1.189E-04 | 3.294 | 184 | - |
| 1222 | ENSP00000276616 | PSKH2 | 1.365E-03 | 8.762 | 182 | - |
| 1223 | ENSP00000301408 | CGB5 | 5.104E-04 | 6.954 | 181 | - |
| 1224 | ENSP00000352295 | CGB2 | 1.168E-03 | 6.450 | 181 | - |
| 1225 | ENSP00000381780 | ARHGEF33 | 4.955E-04 | 6.322 | 181 | - |
| 1226 | ENSP00000261891 | DAPK2 | 1.203E-03 | 8.756 | 180 | - |
| 1227 | ENSP00000383210 | NEK3 | 6.741E-03 | 8.999 | 179 | - |
| 1228 | ENSP00000357086 | FCRL6 | 3.138E-04 | 4.100 | 179 | - |
| 1229 | ENSP00000275358 | VWDE | 1.641E-04 | 2.091 | 179 | - |
| 1230 | ENSP00000263408 | C9 | 8.402E-04 | 9.166 | 177 | - |
| 1231 | ENSP00000405950 | PNCK | 9.718E-04 | 8.196 | 177 | - |
| 1232 | ENSP00000009105 | CAMK1G | 9.255E-04 | 4.232 | 177 | - |
| 1233 | ENSP00000320754 | STK33 | 1.215E-03 | 3.926 | 177 | - |
| 1234 | ENSP00000367747 | PLCH2 | 2.953E-03 | 8.850 | 175 | - |
| 1235 | ENSP00000345988 | PLCH1 | 2.148E-03 | 8.521 | 175 | - |
| 1236 | ENSP00000388631 | PLCD4 | 2.769E-03 | 8.135 | 175 | - |
| 1237 | ENSP00000409637 | PLCL2 | 1.177E-03 | 7.345 | 175 | - |
| 1238 | ENSP00000313731 | PLCD3 | 2.588E-03 | 4.152 | 175 | - |
| 1239 | ENSP00000378356 | KIF20A | 1.381E-03 | 6.469 | 174 | - |
| 1240 | ENSP00000329482 | CCSER1 | 1.795E-04 | 2.029 | 173 | - |
| 1241 | ENSP00000403208 | MAP10 | 1.824E-03 | 8.675 | 170 | - |
| 1242 | ENSP00000383630 | KIR3DL2 | 8.250E-03 | 12.759 | 169 | - |
| 1243 | ENSP00000399696 | AC037459.4 | 1.790E-03 | 8.732 | 169 | - |
| 1244 | ENSP00000307954 | FAM174A | 2.855E-04 | 2.630 | 167 | - |
| 1245 | ENSP00000368908 | LHFP | 1.542E-02 | 8.973 | 165 | - |
| 1246 | ENSP00000326500 | IL11RA | 3.260E-04 | 2.430 | 165 | - |
| 1247 | ENSP00000360718 | RAB3B | 4.517E-03 | 8.665 | 163 | - |
| 1248 | ENSP00000262713 | AJUBA | 3.782E-03 | 4.301 | 163 | - |
| 1249 | ENSP00000364119 | EIF2S2 | 2.300E-03 | 7.884 | 161 | - |
| 1250 | ENSP00000335618 | PITPNC1 | 4.119E-03 | 7.388 | 161 | - |
| 1251 | ENSP00000418994 | SYNPR | 1.699E-03 | 7.256 | 161 | - |
| 1252 | ENSP00000371800 | RNF215 | 2.550E-03 | 6.641 | 161 | - |
| 1253 | ENSP00000336801 | SLC25A26 | 1.823E-02 | 4.490 | 159 | - |
| 1254 | ENSP00000301665 | TMEM99 | 3.555E-04 | 2.491 | 159 | - |
| 1255 | ENSP00000321464 | C10orf67 | 3.173E-04 | 2.393 | 159 | - |
| 1256 | ENSP00000291386 | SSU72 | 5.290E-03 | 4.232 | 158 | - |
| 1257 | ENSP00000400592 | CASP16 | 2.983E-03 | 8.885 | 157 | - |
| 1258 | ENSP00000350547 | SEMA4F | 8.035E-04 | 3.693 | 157 | - |
| 1259 | ENSP00000345849 | SPAM1 | 1.182E-04 | 2.820 | 157 | - |
| 1260 | ENSP00000310255 | GPR152 | 1.582E-04 | 2.477 | 156 | - |
| 1261 | ENSP00000273317 | LIMD1 | 6.671E-03 | 4.253 | 154 | - |
| 1262 | ENSP00000328813 | KCNH8 | 1.241E-02 | 9.015 | 153 | - |
| 1263 | ENSP00000345374 | CXXC11 | 3.784E-04 | 3.134 | 153 | - |
| 1264 | ENSP00000377381 | UBTD2 | 8.032E-02 | 9.037 | 152 | - |
| 1265 | ENSP00000319991 | SLC16A6 | 1.604E-04 | 2.590 | 152 | - |
| 1266 | ENSP00000253778 | GFPT2 | 1.990E-03 | 4.248 | 150 | - |
| 1267 | ENSP00000462879 | LYRM9 | 8.608E-03 | 2.044 | 150 | - |
| 1268 | ENSP00000376522 | CEP112 | 1.298E-02 | 8.978 | 0 | - |
| 1269 | ENSP00000417674 | CYLC2 | 2.959E-03 | 8.847 | 0 | - |
| 1270 | ENSP00000453042 | LY6K | 2.621E-03 | 8.733 | 0 | - |
| 1271 | ENSP00000342840 | LYSMD4 | 2.696E-03 | 8.665 | 0 | - |
| 1272 | ENSP00000359507 | CUTC | 1.463E-03 | 7.764 | 0 | - |
| 1273 | ENSP00000348668 | GALNT6 | 6.436E-04 | 7.749 | 0 | - |
| 1274 | ENSP00000342570 | TREML4 | 9.826E-04 | 7.717 | 0 | - |
| 1275 | ENSP00000389913 | PLEKHM1 | 1.893E-03 | 7.050 | 0 | - |
| 1276 | ENSP00000360272 | SORBS1 | 1.616E-04 | 6.472 | 0 | - |
| 1277 | ENSP00000253247 | NOL11 | 4.795E-04 | 5.705 | 0 | - |
| 1278 | ENSP00000224600 | RBP3 | 7.121E-04 | 4.856 | 0 | - |
| 1279 | ENSP00000417806 | SMIM4 | 2.117E-04 | 4.844 | 0 | - |
| 1280 | ENSP00000299164 | ADAMTS15 | 3.634E-04 | 4.663 | 0 | - |
| 1281 | ENSP00000350592 | MAGEA12 | 2.432E-04 | 4.167 | 0 | - |
| 1282 | ENSP00000369729 | OR51A2 | 9.145E-05 | 3.912 | 0 | - |
| 1283 | ENSP00000273814 | DGKQ | 2.077E-04 | 3.109 | 0 | - |
| 1284 | ENSP00000412388 | C9orf172 | 1.329E-04 | 3.064 | 0 | - |
| 1285 | ENSP00000427975 | RANBP17 | 2.896E-04 | 2.874 | 0 | - |
| 1286 | ENSP00000364964 | C6orf25 | 1.970E-03 | 2.437 | 0 | - |
| 1287 | ENSP00000406925 | C4orf26 | 1.046E-04 | 2.379 | 0 | - |
| 1288 | ENSP00000313661 | HTR1D | 7.307E-05 | 1.958 | - | - |
| 1289 | ENSP00000296694 | SCGB3A2 | 1.780E-04 | 1.957 | - | - |
| 1290 | ENSP00000297436 | DEFA6 | 1.453E-04 | 1.953 | - | - |
| 1291 | ENSP00000431512 | HSF1 | 8.232E-04 | 1.953 | - | - |
| 1292 | ENSP00000337261 | ARHGEF1 | 1.555E-03 | 1.952 | - | - |
| 1293 | ENSP00000344220 | PDPK1 | 1.952E-03 | 1.945 | - | - |
| 1294 | ENSP00000320509 | BANK1 | 1.516E-04 | 1.945 | - | - |
| 1295 | ENSP00000404503 | BBC3 | 1.452E-03 | 1.945 | - | - |
| 1296 | ENSP00000388648 | CHEK1 | 9.250E-04 | 1.933 | - | - |
| 1297 | ENSP00000220809 | PLAT | 8.032E-05 | 1.931 | - | - |
| 1298 | ENSP00000359699 | DNASE2B | 1.619E-04 | 1.929 | - | - |
| 1299 | ENSP00000377783 | PROS1 | 8.387E-05 | 1.929 | - | - |
| 1300 | ENSP00000257359 | ADAMTS8 | 1.614E-04 | 1.925 | - | - |
| 1301 | ENSP00000249601 | ARHGAP22 | 1.268E-04 | 1.920 | - | - |
| 1302 | ENSP00000451040 | PGF | 1.361E-03 | 1.917 | - | - |
| 1303 | ENSP00000293851 | PRSS33 | 1.181E-04 | 1.915 | - | - |
| 1304 | ENSP00000468991 | MTRNR2L12 | 6.913E-05 | 1.899 | - | - |
| 1305 | ENSP00000312143 | TNS3 | 7.036E-04 | 1.897 | - | - |
| 1306 | ENSP00000206513 | CEBPE | 1.930E-04 | 1.893 | - | - |
| 1307 | ENSP00000295927 | PTX3 | 2.037E-04 | 1.891 | - | - |
| 1308 | ENSP00000384053 | CSF2RB | 1.495E-04 | 1.884 | - | - |
| 1309 | ENSP00000349954 | CGB | 8.451E-05 | 1.880 | - | - |
| 1310 | ENSP00000396259 | PAX2 | 7.951E-04 | 1.875 | - | - |
| 1311 | ENSP00000356385 | CFHR2 | 2.211E-04 | 1.873 | - | - |
| 1312 | ENSP00000383690 | MASP2 | 1.174E-04 | 1.862 | - | - |
| 1313 | ENSP00000358490 | CD2 | 2.127E-04 | 1.860 | - | - |
| 1314 | ENSP00000466933 | S1PR2 | 8.501E-05 | 1.859 | - | - |
| 1315 | ENSP00000401303 | SHC1 | 8.642E-04 | 1.856 | - | - |
| 1316 | ENSP00000240328 | TBX2 | 5.366E-05 | 1.854 | - | - |
| 1317 | ENSP00000316152 | SFTPC | 8.668E-05 | 1.854 | - | - |
| 1318 | ENSP00000386201 | TAS2R41 | 7.640E-05 | 1.852 | - | - |
| 1319 | ENSP00000211076 | TPSD1 | 1.101E-04 | 1.840 | - | - |
| 1320 | ENSP00000229335 | AICDA | 7.691E-05 | 1.839 | - | - |
| 1321 | ENSP00000343428 | GPR18 | 1.143E-04 | 1.838 | - | - |
| 1322 | ENSP00000414303 | BDNF | 7.260E-04 | 1.837 | - | - |
| 1323 | ENSP00000457706 | HSPB2-C11orf52 | 1.078E-03 | 1.837 | - | - |
| 1324 | ENSP00000369578 | IFNW1 | 1.246E-04 | 1.828 | - | - |
| 1325 | ENSP00000306579 | CERS6 | 2.892E-03 | 1.827 | - | - |
| 1326 | ENSP00000444972 | TCF7L2 | 8.639E-04 | 1.826 | - | - |
| 1327 | ENSP00000261652 | TNFRSF13B | 1.832E-04 | 1.825 | - | - |
| 1328 | ENSP00000297819 | SSMEM1 | 3.171E-04 | 1.809 | - | - |
| 1329 | ENSP00000375893 | GPR55 | 8.052E-05 | 1.808 | - | - |
| 1330 | ENSP00000384665 | LPAR2 | 7.498E-05 | 1.806 | - | - |
| 1331 | ENSP00000295633 | FSTL1 | 1.003E-04 | 1.802 | - | - |
| 1332 | ENSP00000247829 | TSPAN8 | 1.125E-04 | 1.798 | - | - |
| 1333 | ENSP00000359540 | CREG1 | 1.699E-04 | 1.798 | - | - |
| 1334 | ENSP00000428657 | NAIP | 1.161E-03 | 1.794 | - | - |
| 1335 | ENSP00000358963 | HTR1B | 6.413E-05 | 1.792 | - | - |
| 1336 | ENSP00000290216 | SCRN2 | 1.429E-04 | 1.792 | - | - |
| 1337 | ENSP00000310036 | CD34 | 1.235E-04 | 1.788 | - | - |
| 1338 | ENSP00000376848 | PRIMA1 | 1.378E-04 | 1.780 | - | - |
| 1339 | ENSP00000338345 | SNCA | 3.339E-04 | 1.775 | - | - |
| 1340 | ENSP00000378492 | GRM2 | 6.395E-05 | 1.775 | - | - |
| 1341 | ENSP00000337065 | CXCL14 | 1.281E-04 | 1.773 | - | - |
| 1342 | ENSP00000362195 | CSF3R | 1.522E-04 | 1.771 | - | - |
| 1343 | ENSP00000288466 | ZNF618 | 1.328E-02 | 1.771 | - | - |
| 1344 | ENSP00000332454 | RIPK4 | 2.624E-04 | 1.764 | - | - |
| 1345 | ENSP00000265016 | BST1 | 1.189E-04 | 1.758 | - | - |
| 1346 | ENSP00000303963 | CTRB2 | 9.163E-05 | 1.758 | - | - |
| 1347 | ENSP00000417281 | MDM2 | 8.802E-04 | 1.756 | - | - |
| 1348 | ENSP00000391826 | IGF2 | 9.062E-04 | 1.754 | - | - |
| 1349 | ENSP00000341141 | SIGLEC1 | 8.952E-05 | 1.751 | - | - |
| 1350 | ENSP00000259631 | CCL27 | 1.452E-04 | 1.750 | - | - |
| 1351 | ENSP00000359643 | LPAR3 | 7.450E-05 | 1.749 | - | - |
| 1352 | ENSP00000261978 | LTBP2 | 1.765E-04 | 1.747 | - | - |
| 1353 | ENSP00000332504 | CCR10 | 1.279E-04 | 1.743 | - | - |
| 1354 | ENSP00000336764 | OPRL1 | 7.276E-05 | 1.740 | - | - |
| 1355 | ENSP00000406162 | PRMT1 | 8.307E-04 | 1.736 | - | - |
| 1356 | ENSP00000329507 | CD300C | 1.105E-04 | 1.735 | - | - |
| 1357 | ENSP00000407685 | TNXB | 2.081E-04 | 1.734 | - | - |
| 1358 | ENSP00000363596 | CNR2 | 9.542E-05 | 1.731 | - | - |
| 1359 | ENSP00000222304 | HAMP | 1.155E-04 | 1.731 | - | - |
| 1360 | ENSP00000378130 | CFI | 7.582E-05 | 1.731 | - | - |
| 1361 | ENSP00000426909 | GNB2L1 | 7.416E-04 | 1.728 | - | - |
| 1362 | ENSP00000225371 | EPX | 6.593E-05 | 1.728 | - | - |
| 1363 | ENSP00000401802 | PSMC6 | 8.791E-04 | 1.725 | - | - |
| 1364 | ENSP00000380227 | ITGA4 | 1.434E-04 | 1.723 | - | - |
| 1365 | ENSP00000371170 | GAGE13 | 1.983E-04 | 1.721 | - | - |
| 1366 | ENSP00000363643 | P2RY4 | 7.692E-05 | 1.720 | - | - |
| 1367 | ENSP00000220597 | PAG1 | 1.461E-04 | 1.720 | - | - |
| 1368 | ENSP00000281703 | GLT1D1 | 1.490E-04 | 1.719 | - | - |
| 1369 | ENSP00000428220 | IRGM | 2.633E-04 | 1.717 | - | - |
| 1370 | ENSP00000356713 | IFNGR1 | 2.607E-04 | 1.715 | - | - |
| 1371 | ENSP00000409937 | ALS2CR11 | 4.814E-05 | 1.713 | - | - |
| 1372 | ENSP00000293275 | CCL16 | 6.887E-05 | 1.711 | - | - |
| 1373 | ENSP00000315477 | CD209 | 1.757E-04 | 1.704 | - | - |
| 1374 | ENSP00000297316 | SOX17 | 4.876E-05 | 1.703 | - | - |
| 1375 | ENSP00000414378 | EFNA4 | 1.386E-04 | 1.699 | - | - |
| 1376 | ENSP00000386810 | RGPD4 | 7.269E-04 | 1.698 | - | - |
| 1377 | ENSP00000369554 | IFNA2 | 1.610E-04 | 1.695 | - | - |
| 1378 | ENSP00000421689 | CSNK1A1 | 7.996E-04 | 1.690 | - | - |
| 1379 | ENSP00000346671 | FYN | 3.283E-04 | 1.689 | - | - |
| 1380 | ENSP00000365435 | TNFRSF1B | 2.227E-04 | 1.688 | - | - |
| 1381 | ENSP00000384894 | AQP12B | 1.117E-04 | 1.685 | - | - |
| 1382 | ENSP00000293889 | CCDC78 | 2.464E-04 | 1.684 | - | - |
| 1383 | ENSP00000242465 | SRGN | 1.254E-04 | 1.679 | - | - |
| 1384 | ENSP00000299665 | CLEC4D | 1.278E-04 | 1.669 | - | - |
| 1385 | ENSP00000279544 | KLRF1 | 2.301E-04 | 1.669 | - | - |
| 1386 | ENSP00000222271 | COMP | 5.902E-05 | 1.665 | - | - |
| 1387 | ENSP00000281806 | MCHR2 | 5.382E-05 | 1.662 | - | - |
| 1388 | ENSP00000454919 | RNF112 | 1.444E-04 | 1.656 | - | - |
| 1389 | ENSP00000316729 | RHOJ | 2.905E-04 | 1.654 | - | - |
| 1390 | ENSP00000278175 | ADM | 6.915E-05 | 1.652 | - | - |
| 1391 | ENSP00000350052 | POTEF | 1.985E-04 | 1.649 | - | - |
| 1392 | ENSP00000439425 | AC025287.1 | 4.451E-05 | 1.645 | - | - |
| 1393 | ENSP00000400088 | CDK3 | 6.037E-04 | 1.642 | - | - |
| 1394 | ENSP00000294413 | RHCE | 5.561E-05 | 1.642 | - | - |
| 1395 | ENSP00000264376 | CRYGD | 6.920E-05 | 1.640 | - | - |
| 1396 | ENSP00000428924 | LYN | 7.447E-04 | 1.639 | - | - |
| 1397 | ENSP00000305696 | RBFA | 7.019E-05 | 1.639 | - | - |
| 1398 | ENSP00000302630 | ONECUT1 | 6.639E-05 | 1.638 | - | - |
| 1399 | ENSP00000216911 | AURKA | 5.990E-04 | 1.637 | - | - |
| 1400 | ENSP00000447803 | DDIT3 | 1.063E-03 | 1.635 | - | - |
| 1401 | ENSP00000266068 | GMEB2 | 1.776E-04 | 1.634 | - | - |
| 1402 | ENSP00000263686 | SELP | 1.566E-04 | 1.632 | - | - |
| 1403 | ENSP00000360997 | ADAMTS13 | 1.605E-04 | 1.628 | - | - |
| 1404 | ENSP00000401445 | ERN1 | 7.123E-04 | 1.627 | - | - |
| 1405 | ENSP00000295550 | COL6A3 | 7.780E-05 | 1.626 | - | - |
| 1406 | ENSP00000387662 | GCG | 6.312E-04 | 1.621 | - | - |
| 1407 | ENSP00000284245 | C16orf74 | 2.004E-04 | 1.621 | - | - |
| 1408 | ENSP00000274979 | ANO7 | 1.180E-04 | 1.619 | - | - |
| 1409 | ENSP00000372126 | DEFA1 | 2.239E-04 | 1.619 | - | - |
| 1410 | ENSP00000200676 | CETP | 8.433E-05 | 1.618 | - | - |
| 1411 | ENSP00000425561 | EIF4E | 8.873E-04 | 1.618 | - | - |
| 1412 | ENSP00000257408 | KLB | 6.382E-05 | 1.614 | - | - |
| 1413 | ENSP00000410294 | FGFR2 | 6.661E-04 | 1.613 | - | - |
| 1414 | ENSP00000448165 | APAF1 | 7.739E-04 | 1.612 | - | - |
| 1415 | ENSP00000400175 | RHOA | 5.794E-04 | 1.609 | - | - |
| 1416 | ENSP00000217407 | LBP | 1.340E-04 | 1.609 | - | - |
| 1417 | ENSP00000360250 | ENTPD1 | 1.229E-04 | 1.609 | - | - |
| 1418 | ENSP00000290953 | AGRP | 8.051E-05 | 1.606 | - | - |
| 1419 | ENSP00000165524 | PRLH | 1.053E-04 | 1.605 | - | - |
| 1420 | ENSP00000226413 | GNRHR | 5.156E-05 | 1.604 | - | - |
| 1421 | ENSP00000355316 | GRM3 | 6.401E-05 | 1.604 | - | - |
| 1422 | ENSP00000318057 | EGR3 | 9.642E-04 | 1.603 | - | - |
| 1423 | ENSP00000244241 | IL17C | 1.407E-04 | 1.597 | - | - |
| 1424 | ENSP00000363708 | BMPR2 | 6.528E-05 | 1.591 | - | - |
| 1425 | ENSP00000304236 | CD14 | 1.636E-04 | 1.589 | - | - |
| 1426 | ENSP00000341794 | IL1F10 | 1.868E-04 | 1.586 | - | - |
| 1427 | ENSP00000402515 | SMG1 | 7.395E-04 | 1.586 | - | - |
| 1428 | ENSP00000390020 | HLA-DOB | 1.828E-04 | 1.582 | - | - |
| 1429 | ENSP00000264992 | ASTE1 | 9.935E-05 | 1.582 | - | - |
| 1430 | ENSP00000366248 | CORT | 9.920E-05 | 1.576 | - | - |
| 1431 | ENSP00000180166 | FGF20 | 5.662E-05 | 1.575 | - | - |
| 1432 | ENSP00000288098 | IL34 | 1.539E-04 | 1.574 | - | - |
| 1433 | ENSP00000373854 | ITGAD | 1.041E-04 | 1.571 | - | - |
| 1434 | ENSP00000249016 | MCHR1 | 5.456E-05 | 1.564 | - | - |
| 1435 | ENSP00000408695 | PRKCA | 6.465E-04 | 1.555 | - | - |
| 1436 | ENSP00000436682 | ARMS2 | 2.505E-04 | 1.554 | - | - |
| 1437 | ENSP00000274629 | KCNMB1 | 7.649E-05 | 1.553 | - | - |
| 1438 | ENSP00000262741 | PIK3R3 | 8.013E-05 | 1.547 | - | - |
| 1439 | ENSP00000237837 | FGF23 | 5.907E-05 | 1.543 | - | - |
| 1440 | ENSP00000226091 | EFNB3 | 6.042E-05 | 1.540 | - | - |
| 1441 | ENSP00000279058 | SPINT4 | 8.506E-05 | 1.538 | - | - |
| 1442 | ENSP00000357012 | CD244 | 1.635E-04 | 1.537 | - | - |
| 1443 | ENSP00000310241 | SLC22A13 | 9.317E-05 | 1.534 | - | - |
| 1444 | ENSP00000393566 | HLA-DPA1 | 1.002E-04 | 1.528 | - | - |
| 1445 | ENSP00000296121 | KIAA1143 | 1.544E-04 | 1.524 | - | - |
| 1446 | ENSP00000257863 | AMHR2 | 6.820E-05 | 1.520 | - | - |
| 1447 | ENSP00000408617 | HDAC9 | 6.193E-04 | 1.519 | - | - |
| 1448 | ENSP00000327724 | TAS2R60 | 1.191E-04 | 1.516 | - | - |
| 1449 | ENSP00000175238 | ADAM7 | 6.722E-05 | 1.515 | - | - |
| 1450 | ENSP00000358730 | ADORA3 | 1.031E-04 | 1.511 | - | - |
| 1451 | ENSP00000252229 | MICB | 7.768E-04 | 1.507 | - | - |
| 1452 | ENSP00000354782 | CD247 | 2.480E-04 | 1.507 | - | - |
| 1453 | ENSP00000419782 | CDK5 | 5.595E-04 | 1.505 | - | - |
| 1454 | ENSP00000264187 | NID1 | 6.700E-05 | 1.500 | - | - |
| 1455 | ENSP00000467176 | TNNT1 | 6.696E-04 | 1.500 | - | - |
| 1456 | ENSP00000357615 | FRK | 5.002E-04 | 1.493 | - | - |
| 1457 | ENSP00000334003 | INTU | 1.009E-04 | 1.490 | - | - |
| 1458 | ENSP00000222381 | PON1 | 4.975E-05 | 1.489 | - | - |
| 1459 | ENSP00000334122 | FGF3 | 8.238E-05 | 1.487 | - | - |
| 1460 | ENSP00000303149 | GPR27 | 8.570E-05 | 1.485 | - | - |
| 1461 | ENSP00000219551 | RHBDL1 | 4.334E-05 | 1.484 | - | - |
| 1462 | ENSP00000356346 | PTPRC | 6.321E-04 | 1.482 | - | - |
| 1463 | ENSP00000442349 | P2RX7 | 8.989E-05 | 1.480 | - | - |
| 1464 | ENSP00000419119 | OR10C1 | 2.825E-05 | 1.471 | - | - |
| 1465 | ENSP00000273183 | STAC | 2.139E-04 | 1.465 | - | - |
| 1466 | ENSP00000394954 | GSG1L | 6.307E-05 | 1.462 | - | - |
| 1467 | ENSP00000244565 | UNC5CL | 1.266E-04 | 1.462 | - | - |
| 1468 | ENSP00000290122 | CELA3A | 9.080E-05 | 1.460 | - | - |
| 1469 | ENSP00000341013 | AMTN | 1.597E-04 | 1.458 | - | - |
| 1470 | ENSP00000361125 | VEGFA | 5.424E-04 | 1.444 | - | - |
| 1471 | ENSP00000351484 | NAALADL1 | 1.417E-04 | 1.440 | - | - |
| 1472 | ENSP00000295886 | NKX6-1 | 6.008E-05 | 1.439 | - | - |
| 1473 | ENSP00000334050 | TAS2R42 | 1.122E-04 | 1.434 | - | - |
| 1474 | ENSP00000326018 | PRX | 1.203E-04 | 1.431 | - | - |
| 1475 | ENSP00000301920 | MMADHC | 1.526E-04 | 1.430 | - | - |
| 1476 | ENSP00000349436 | ADAM15 | 1.173E-04 | 1.430 | - | - |
| 1477 | ENSP00000472998 | SNRNP70 | 8.606E-04 | 1.429 | - | - |
| 1478 | ENSP00000225538 | P2RX1 | 5.275E-05 | 1.418 | - | - |
| 1479 | ENSP00000415481 | PROM1 | 9.254E-05 | 1.418 | - | - |
| 1480 | ENSP00000360255 | OPN5 | 7.446E-05 | 1.418 | - | - |
| 1481 | ENSP00000357778 | LCE3E | 1.021E-04 | 1.415 | - | - |
| 1482 | ENSP00000338157 | ZBTB16 | 8.041E-04 | 1.414 | - | - |
| 1483 | ENSP00000393262 | RBFOX3 | 9.255E-04 | 1.413 | - | - |
| 1484 | ENSP00000329127 | PRKCH | 5.318E-04 | 1.409 | - | - |
| 1485 | ENSP00000328472 | S1PR5 | 7.814E-05 | 1.408 | - | - |
| 1486 | ENSP00000343339 | P2RX2 | 7.782E-05 | 1.406 | - | - |
| 1487 | ENSP00000340477 | TMEM179 | 1.112E-04 | 1.401 | - | - |
| 1488 | ENSP00000225275 | MPO | 1.003E-04 | 1.400 | - | - |
| 1489 | ENSP00000298966 | SMCO4 | 1.692E-04 | 1.399 | - | - |
| 1490 | ENSP00000342336 | FAM180A | 1.012E-04 | 1.395 | - | - |
| 1491 | ENSP00000264072 | FCER2 | 8.731E-05 | 1.392 | - | - |
| 1492 | ENSP00000016913 | MS4A12 | 1.560E-04 | 1.387 | - | - |
| 1493 | ENSP00000333680 | NFAM1 | 1.617E-04 | 1.387 | - | - |
| 1494 | ENSP00000379140 | CXCL12 | 8.957E-04 | 1.385 | - | - |
| 1495 | ENSP00000263413 | C6 | 1.355E-04 | 1.384 | - | - |
| 1496 | ENSP00000365227 | AIF1 | 9.593E-05 | 1.383 | - | - |
| 1497 | ENSP00000349629 | LRBA | 1.079E-04 | 1.382 | - | - |
| 1498 | ENSP00000340644 | C15orf43 | 3.375E-04 | 1.382 | - | - |
| 1499 | ENSP00000230301 | C6orf118 | 1.299E-04 | 1.381 | - | - |
| 1500 | ENSP00000364595 | NINJ1 | 2.109E-04 | 1.375 | - | - |
| 1501 | ENSP00000303532 | DEFB4A | 9.595E-05 | 1.373 | - | - |
| 1502 | ENSP00000370297 | CER1 | 4.230E-05 | 1.370 | - | - |
| 1503 | ENSP00000260113 | PI15 | 8.161E-05 | 1.368 | - | - |
| 1504 | ENSP00000390431 | HLA-DQB2 | 1.831E-04 | 1.366 | - | - |
| 1505 | ENSP00000418960 | BRCA1 | 6.819E-04 | 1.362 | - | - |
| 1506 | ENSP00000335006 | LCE3A | 9.352E-05 | 1.360 | - | - |
| 1507 | ENSP00000302716 | MUC17 | 9.729E-05 | 1.358 | - | - |
| 1508 | ENSP00000378786 | HLA-DRA | 1.218E-04 | 1.356 | - | - |
| 1509 | ENSP00000282849 | ADAMTS18 | 1.272E-04 | 1.352 | - | - |
| 1510 | ENSP00000368632 | GATA3 | 5.608E-04 | 1.352 | - | - |
| 1511 | ENSP00000253796 | RAMP2 | 1.234E-04 | 1.347 | - | - |
| 1512 | ENSP00000274181 | ADAMTS16 | 1.217E-04 | 1.347 | - | - |
| 1513 | ENSP00000305200 | IL1RAPL1 | 9.550E-05 | 1.346 | - | - |
| 1514 | ENSP00000357731 | LOR | 8.852E-05 | 1.343 | - | - |
| 1515 | ENSP00000398526 | BBS1 | 7.791E-05 | 1.343 | - | - |
| 1516 | ENSP00000323280 | CD6 | 2.502E-04 | 1.342 | - | - |
| 1517 | ENSP00000239761 | MRC1 | 2.078E-04 | 1.341 | - | - |
| 1518 | ENSP00000246006 | CD93 | 6.873E-05 | 1.340 | - | - |
| 1519 | ENSP00000356708 | OLIG3 | 1.319E-04 | 1.339 | - | - |
| 1520 | ENSP00000349085 | MAGEA1 | 6.199E-05 | 1.338 | - | - |
| 1521 | ENSP00000318472 | NCAM1 | 8.448E-04 | 1.337 | - | - |
| 1522 | ENSP00000356172 | SOX13 | 7.624E-05 | 1.336 | - | - |
| 1523 | ENSP00000006724 | CEACAM7 | 1.305E-04 | 1.335 | - | - |
| 1524 | ENSP00000312652 | LEP | 4.697E-04 | 1.335 | - | - |
| 1525 | ENSP00000354360 | LAMC3 | 1.289E-04 | 1.335 | - | - |
| 1526 | ENSP00000264257 | IL1RL2 | 2.446E-04 | 1.333 | - | - |
| 1527 | ENSP00000356658 | GPR52 | 8.272E-05 | 1.332 | - | - |
| 1528 | ENSP00000398729 | PAPPA-AS1 | 7.326E-05 | 1.328 | - | - |
| 1529 | ENSP00000315011 | EDNRA | 1.486E-03 | 1.327 | - | - |
| 1530 | ENSP00000307445 | LTB4R | 5.034E-05 | 1.326 | - | - |
| 1531 | ENSP00000356671 | SERPINC1 | 1.252E-04 | 1.325 | - | - |
| 1532 | ENSP00000394932 | CDKN2A | 6.065E-04 | 1.323 | - | - |
| 1533 | ENSP00000370880 | MLANA | 1.323E-04 | 1.318 | - | - |
| 1534 | ENSP00000292199 | NLRX1 | 1.110E-04 | 1.318 | - | - |
| 1535 | ENSP00000361405 | MMP9 | 7.132E-04 | 1.318 | - | - |
| 1536 | ENSP00000329623 | BCL2 | 3.881E-04 | 1.317 | - | - |
| 1537 | ENSP00000334848 | C11orf74 | 2.090E-04 | 1.316 | - | - |
| 1538 | ENSP00000415941 | C4B | 1.898E-04 | 1.315 | - | - |
| 1539 | ENSP00000279575 | PRB4 | 1.185E-04 | 1.315 | - | - |
| 1540 | ENSP00000359635 | FGF13 | 7.724E-04 | 1.315 | - | - |
| 1541 | ENSP00000357668 | ADAM12 | 9.707E-05 | 1.310 | - | - |
| 1542 | ENSP00000354394 | STAT1 | 6.486E-04 | 1.308 | - | - |
| 1543 | ENSP00000307132 | CST5 | 1.042E-04 | 1.306 | - | - |
| 1544 | ENSP00000223795 | TNFSF8 | 4.324E-04 | 1.305 | - | - |
| 1545 | ENSP00000263025 | MAPK3 | 2.581E-04 | 1.304 | - | - |
| 1546 | ENSP00000356438 | PTGS2 | 1.107E-03 | 1.303 | - | - |
| 1547 | ENSP00000327763 | NLRP10 | 1.855E-04 | 1.303 | - | - |
| 1548 | ENSP00000381129 | GRK4 | 1.127E-04 | 1.302 | - | - |
| 1549 | ENSP00000364348 | CCDC180 | 1.131E-04 | 1.298 | - | - |
| 1550 | ENSP00000294312 | FGF19 | 6.045E-05 | 1.298 | - | - |
| 1551 | ENSP00000310832 | CTSF | 9.878E-05 | 1.297 | - | - |
| 1552 | ENSP00000473153 | ERVV-1 | 3.748E-05 | 1.297 | - | - |
| 1553 | ENSP00000325485 | CERS5 | 2.925E-03 | 1.296 | - | - |
| 1554 | ENSP00000231656 | CDX1 | 3.787E-05 | 1.291 | - | - |
| 1555 | ENSP00000430432 | HDAC2 | 6.122E-04 | 1.289 | - | - |
| 1556 | ENSP00000375066 | HCAR2 | 6.774E-05 | 1.289 | - | - |
| 1557 | ENSP00000308893 | SNED1 | 1.142E-04 | 1.289 | - | - |
| 1558 | ENSP00000323929 | A2M | 1.121E-04 | 1.287 | - | - |
| 1559 | ENSP00000382714 | IFITM2 | 1.140E-04 | 1.286 | - | - |
| 1560 | ENSP00000221891 | APLP1 | 5.635E-05 | 1.286 | - | - |
| 1561 | ENSP00000301039 | PROCA1 | 2.188E-04 | 1.284 | - | - |
| 1562 | ENSP00000349478 | HCAR1 | 7.115E-05 | 1.283 | - | - |
| 1563 | ENSP00000308279 | C9orf131 | 3.312E-04 | 1.276 | - | - |
| 1564 | ENSP00000361366 | SFTPD | 6.566E-05 | 1.275 | - | - |
| 1565 | ENSP00000375795 | IL24 | 1.534E-04 | 1.272 | - | - |
| 1566 | ENSP00000341031 | TPRG1 | 2.008E-04 | 1.271 | - | - |
| 1567 | ENSP00000332875 | C2orf70 | 1.528E-04 | 1.268 | - | - |
| 1568 | ENSP00000431445 | CD3G | 1.768E-04 | 1.265 | - | - |
| 1569 | ENSP00000356694 | FASLG | 1.495E-03 | 1.263 | - | - |
| 1570 | ENSP00000296946 | T | 3.894E-05 | 1.260 | - | - |
| 1571 | ENSP00000236979 | TNP1 | 1.019E-04 | 1.257 | - | - |
| 1572 | ENSP00000385834 | TF | 5.109E-04 | 1.257 | - | - |
| 1573 | ENSP00000225387 | CRYBA1 | 8.145E-05 | 1.255 | - | - |
| 1574 | ENSP00000385721 | GGT2 | 5.968E-04 | 1.254 | - | - |
| 1575 | ENSP00000219454 | WFDC1 | 7.111E-05 | 1.254 | - | - |
| 1576 | ENSP00000473193 | AC087645.1 | 3.056E-05 | 1.250 | - | - |
| 1577 | ENSP00000357769 | LCE2B | 9.354E-05 | 1.250 | - | - |
| 1578 | ENSP00000228644 | MYF5 | 3.538E-05 | 1.247 | - | - |
| 1579 | ENSP00000341208 | STAT5A | 9.018E-04 | 1.246 | - | - |
| 1580 | ENSP00000339730 | THBS4 | 9.456E-05 | 1.243 | - | - |
| 1581 | ENSP00000436042 | PCDHA9 | 9.887E-05 | 1.241 | - | - |
| 1582 | ENSP00000331111 | ZNRD1 | 5.688E-05 | 1.240 | - | - |
| 1583 | ENSP00000264246 | CD80 | 1.626E-03 | 1.237 | - | - |
| 1584 | ENSP00000261038 | DIRC2 | 8.402E-05 | 1.237 | - | - |
| 1585 | ENSP00000269593 | IGFBP4 | 6.842E-05 | 1.237 | - | - |
| 1586 | ENSP00000368683 | EDN1 | 7.534E-04 | 1.236 | - | - |
| 1587 | ENSP00000356033 | TAGAP | 1.079E-04 | 1.232 | - | - |
| 1588 | ENSP00000308361 | P2RY14 | 8.174E-05 | 1.231 | - | - |
| 1589 | ENSP00000306245 | FOS | 2.946E-04 | 1.230 | - | - |
| 1590 | ENSP00000399324 | FGF16 | 6.608E-05 | 1.229 | - | - |
| 1591 | ENSP00000370074 | SERPINB9 | 1.317E-04 | 1.227 | - | - |
| 1592 | ENSP00000379658 | ETV6 | 1.300E-03 | 1.226 | - | - |
| 1593 | ENSP00000341327 | SOCS4 | 1.141E-04 | 1.222 | - | - |
| 1594 | ENSP00000328818 | GPR132 | 4.895E-05 | 1.221 | - | - |
| 1595 | ENSP00000353465 | GSDMB | 1.516E-04 | 1.219 | - | - |
| 1596 | ENSP00000296870 | IL3 | 1.365E-03 | 1.219 | - | - |
| 1597 | ENSP00000283233 | CCDC148 | 1.272E-04 | 1.218 | - | - |
| 1598 | ENSP00000234071 | PROC | 8.655E-04 | 1.217 | - | - |
| 1599 | ENSP00000344115 | CDH5 | 8.786E-05 | 1.211 | - | - |
| 1600 | ENSP00000277541 | NOTCH1 | 2.552E-04 | 1.210 | - | - |
| 1601 | ENSP00000260356 | THBS1 | 8.786E-04 | 1.209 | - | - |
| 1602 | ENSP00000369581 | IFNB1 | 1.227E-03 | 1.209 | - | - |
| 1603 | ENSP00000266085 | TIMP3 | 1.855E-03 | 1.209 | - | - |
| 1604 | ENSP00000362935 | ITGB1BP2 | 8.466E-05 | 1.209 | - | - |
| 1605 | ENSP00000296871 | CSF2 | 8.849E-04 | 1.208 | - | - |
| 1606 | ENSP00000365280 | ID1 | 1.122E-04 | 1.208 | - | - |
| 1607 | ENSP00000297435 | DEFA4 | 6.714E-05 | 1.208 | - | - |
| 1608 | ENSP00000351602 | FUT4 | 9.681E-05 | 1.204 | - | - |
| 1609 | ENSP00000263056 | MAP3K8 | 7.125E-04 | 1.203 | - | - |
| 1610 | ENSP00000283635 | CD8A | 3.291E-04 | 1.200 | - | - |
| 1611 | ENSP00000248598 | FGL2 | 1.293E-04 | 1.199 | - | - |
| 1612 | ENSP00000367202 | RECK | 1.104E-04 | 1.199 | - | - |
| 1613 | ENSP00000231449 | IL4 | 8.824E-04 | 1.198 | - | - |
| 1614 | ENSP00000300134 | STAT6 | 1.176E-03 | 1.194 | - | - |
| 1615 | ENSP00000303153 | COL22A1 | 5.167E-05 | 1.193 | - | - |
| 1616 | ENSP00000252818 | JUND | 5.132E-04 | 1.193 | - | - |
| 1617 | ENSP00000354569 | - | 8.588E-05 | 1.193 | - | - |
| 1618 | ENSP00000246549 | FFAR2 | 5.492E-05 | 1.191 | - | - |
| 1619 | ENSP00000337825 | LCK | 3.805E-04 | 1.190 | - | - |
| 1620 | ENSP00000418018 | IFNE | 1.060E-04 | 1.190 | - | - |
| 1621 | ENSP00000265372 | CREM | 6.882E-04 | 1.189 | - | - |
| 1622 | ENSP00000357772 | LCE2C | 1.135E-04 | 1.189 | - | - |
| 1623 | ENSP00000353344 | ETS2 | 1.801E-03 | 1.186 | - | - |
| 1624 | ENSP00000238682 | TGFB3 | 1.890E-03 | 1.186 | - | - |
| 1625 | ENSP00000307821 | FADS6 | 1.038E-04 | 1.185 | - | - |
| 1626 | ENSP00000330382 | PDGFB | 1.199E-03 | 1.184 | - | - |
| 1627 | ENSP00000263341 | IL1B | 5.930E-04 | 1.183 | - | - |
| 1628 | ENSP00000417183 | DUSP7 | 1.271E-03 | 1.181 | - | - |
| 1629 | ENSP00000330341 | SOCS3 | 1.293E-03 | 1.180 | - | - |
| 1630 | ENSP00000329418 | SOCS1 | 1.874E-03 | 1.179 | - | - |
| 1631 | ENSP00000331462 | ZNF704 | 4.393E-03 | 1.178 | - | - |
| 1632 | ENSP00000260010 | TLR2 | 5.867E-04 | 1.177 | - | - |
| 1633 | ENSP00000227163 | SPI1 | 1.300E-03 | 1.174 | - | - |
| 1634 | ENSP00000345083 | MAP2K3 | 2.309E-03 | 1.172 | - | - |
| 1635 | ENSP00000431371 | VKORC1 | 3.395E-05 | 1.171 | - | - |
| 1636 | ENSP00000231061 | SPARC | 1.307E-03 | 1.171 | - | - |
| 1637 | ENSP00000297814 | KIF27 | 8.905E-05 | 1.170 | - | - |
| 1638 | ENSP00000286648 | DCK | 6.475E-04 | 1.170 | - | - |
| 1639 | ENSP00000246794 | PRRG2 | 2.292E-04 | 1.169 | - | - |
| 1640 | ENSP00000407763 | CCDC122 | 7.446E-05 | 1.168 | - | - |
| 1641 | ENSP00000263967 | PIK3CA | 4.297E-04 | 1.168 | - | - |
| 1642 | ENSP00000372025 | MBP | 1.103E-03 | 1.167 | - | - |
| 1643 | ENSP00000360916 | VAV2 | 2.977E-03 | 1.166 | - | - |
| 1644 | ENSP00000296130 | CLEC3B | 6.139E-05 | 1.166 | - | - |
| 1645 | ENSP00000363827 | HSPG2 | 1.357E-03 | 1.165 | - | - |
| 1646 | ENSP00000327251 | NOS2 | 7.396E-04 | 1.165 | - | - |
| 1647 | ENSP00000391088 | UNC80 | 7.236E-05 | 1.165 | - | - |
| 1648 | ENSP00000466140 | CGB1 | 4.975E-05 | 1.164 | - | - |
| 1649 | ENSP00000294728 | VCAM1 | 1.175E-03 | 1.163 | - | - |
| 1650 | ENSP00000259895 | GTF2H4 | 7.706E-05 | 1.162 | - | - |
| 1651 | ENSP00000363921 | PARD3 | 2.560E-03 | 1.161 | - | - |
| 1652 | ENSP00000422293 | FAM153C | 1.885E-04 | 1.160 | - | - |
| 1653 | ENSP00000358022 | MCL1 | 7.621E-04 | 1.160 | - | - |
| 1654 | ENSP00000288135 | KIT | 3.000E-04 | 1.160 | - | - |
| 1655 | ENSP00000356954 | CTGF | 1.182E-03 | 1.159 | - | - |
| 1656 | ENSP00000221930 | TGFB1 | 2.776E-04 | 1.159 | - | - |
| 1657 | ENSP00000219476 | TSC2 | 1.581E-03 | 1.158 | - | - |
| 1658 | ENSP00000351755 | LPAR1 | 6.158E-05 | 1.158 | - | - |
| 1659 | ENSP00000267101 | ERBB3 | 6.233E-04 | 1.158 | - | - |
| 1660 | ENSP00000373700 | ALK | 1.676E-03 | 1.156 | - | - |
| 1661 | ENSP00000252723 | EPO | 3.574E-03 | 1.156 | - | - |
| 1662 | ENSP00000357900 | CUZD1 | 1.049E-04 | 1.156 | - | - |
| 1663 | ENSP00000220764 | DECR1 | 2.562E-04 | 1.156 | - | - |
| 1664 | ENSP00000279804 | CTF1 | 6.668E-03 | 1.156 | - | - |
| 1665 | ENSP00000320957 | VGLL2 | 5.159E-05 | 1.155 | - | - |
| 1666 | ENSP00000347169 | NUMB | 1.588E-03 | 1.153 | - | - |
| 1667 | ENSP00000354612 | PTGS1 | 2.392E-03 | 1.153 | - | - |
| 1668 | ENSP00000248076 | F2RL3 | 6.411E-05 | 1.153 | - | - |
| 1669 | ENSP00000351605 | FZD6 | 4.791E-03 | 1.153 | - | - |
| 1670 | ENSP00000352852 | MDK | 2.420E-03 | 1.151 | - | - |
| 1671 | ENSP00000293761 | ALOX15 | 5.383E-05 | 1.149 | - | - |
| 1672 | ENSP00000337697 | DCX | 1.957E-03 | 1.148 | - | - |
| 1673 | ENSP00000216037 | XBP1 | 2.182E-03 | 1.147 | - | - |
| 1674 | ENSP00000251849 | RAF1 | 6.829E-04 | 1.147 | - | - |
| 1675 | ENSP00000228872 | CDKN1B | 6.930E-04 | 1.146 | - | - |
| 1676 | ENSP00000263918 | STRN | 6.684E-03 | 1.146 | - | - |
| 1677 | ENSP00000168712 | FGF4 | 1.374E-03 | 1.146 | - | - |
| 1678 | ENSP00000301633 | BIRC5 | 6.185E-04 | 1.145 | - | - |
| 1679 | ENSP00000219070 | MMP2 | 6.406E-04 | 1.145 | - | - |
| 1680 | ENSP00000368104 | BMP2 | 7.846E-04 | 1.145 | - | - |
| 1681 | ENSP00000269033 | SSH2 | 1.912E-03 | 1.145 | - | - |
| 1682 | ENSP00000409378 | CHRM4 | 6.632E-05 | 1.145 | - | - |
| 1683 | ENSP00000349790 | COL9A1 | 7.354E-05 | 1.145 | - | - |
| 1684 | ENSP00000357727 | S100A9 | 1.996E-04 | 1.144 | - | - |
| 1685 | ENSP00000221515 | RETN | 3.544E-03 | 1.143 | - | - |
| 1686 | ENSP00000226574 | NFKB1 | 2.309E-04 | 1.143 | - | - |
| 1687 | ENSP00000364731 | F7 | 1.989E-03 | 1.143 | - | - |
| 1688 | ENSP00000307860 | DIRC1 | 1.261E-04 | 1.142 | - | - |
| 1689 | ENSP00000259526 | NOV | 4.598E-05 | 1.142 | - | - |
| 1690 | ENSP00000366416 | EDNRB | 1.208E-03 | 1.142 | - | - |
| 1691 | ENSP00000247005 | GDF1 | 3.655E-03 | 1.141 | - | - |
| 1692 | ENSP00000280155 | ADRA2A | 7.998E-05 | 1.141 | - | - |
| 1693 | ENSP00000385361 | VPREB1 | 7.569E-05 | 1.140 | - | - |
| 1694 | ENSP00000346508 | PDGFA | 7.464E-03 | 1.139 | - | - |
| 1695 | ENSP00000173229 | NTN1 | 2.218E-03 | 1.139 | - | - |
| 1696 | ENSP00000331791 | TBX1 | 2.205E-03 | 1.139 | - | - |
| 1697 | ENSP00000389792 | DCDC1 | 1.316E-04 | 1.138 | - | - |
| 1698 | ENSP00000375767 | CARD8 | 1.227E-02 | 1.138 | - | - |
| 1699 | ENSP00000252486 | APOE | 3.892E-04 | 1.138 | - | - |
| 1700 | ENSP00000162749 | TNFRSF1A | 1.023E-03 | 1.138 | - | - |
| 1701 | ENSP00000369757 | RPS6 | 9.849E-04 | 1.138 | - | - |
| 1702 | ENSP00000350639 | MAPKAPK3 | 6.379E-03 | 1.137 | - | - |
| 1703 | ENSP00000347379 | OCLN | 2.313E-03 | 1.137 | - | - |
| 1704 | ENSP00000363804 | KLF4 | 6.352E-04 | 1.137 | - | - |
| 1705 | ENSP00000250495 | NEDD8 | 3.262E-04 | 1.136 | - | - |
| 1706 | ENSP00000352271 | XRCC2 | 3.872E-03 | 1.136 | - | - |
| 1707 | ENSP00000381066 | MAP2K7 | 5.248E-03 | 1.135 | - | - |
| 1708 | ENSP00000340409 | SMPD1 | 3.556E-03 | 1.135 | - | - |
| 1709 | ENSP00000286301 | CSF1R | 4.768E-04 | 1.135 | - | - |
| 1710 | ENSP00000362778 | PI16 | 1.072E-04 | 1.134 | - | - |
| 1711 | ENSP00000262053 | ATF1 | 1.726E-03 | 1.134 | - | - |
| 1712 | ENSP00000362057 | NOX1 | 3.122E-03 | 1.134 | - | - |
| 1713 | ENSP00000364943 | DDAH2 | 2.324E-02 | 1.133 | - | - |
| 1714 | ENSP00000223023 | WASL | 1.424E-03 | 1.133 | - | - |
| 1715 | ENSP00000264595 | AGA | 5.907E-05 | 1.133 | - | - |
| 1716 | ENSP00000215530 | FGF22 | 7.239E-05 | 1.133 | - | - |
| 1717 | ENSP00000266000 | DAXX | 3.003E-03 | 1.132 | - | - |
| 1718 | ENSP00000266646 | INHBE | 4.732E-03 | 1.132 | - | - |
| 1719 | ENSP00000337103 | CHAT | 1.596E-03 | 1.131 | - | - |
| 1720 | ENSP00000326407 | CLEC1A | 1.312E-04 | 1.130 | - | - |
| 1721 | ENSP00000326652 | FAM71F1 | 1.012E-04 | 1.130 | - | - |
| 1722 | ENSP00000300305 | RUNX1 | 8.490E-04 | 1.129 | - | - |
| 1723 | ENSP00000356070 | MAPKAPK2 | 6.018E-03 | 1.129 | - | - |
| 1724 | ENSP00000331902 | COL4A5 | 3.013E-03 | 1.128 | - | - |
| 1725 | ENSP00000331745 | GDF3 | 2.578E-03 | 1.128 | - | - |
| 1726 | ENSP00000218388 | TIMP1 | 8.038E-04 | 1.127 | - | - |
| 1727 | ENSP00000358997 | IRAK1 | 6.178E-04 | 1.126 | - | - |
| 1728 | ENSP00000346839 | FN1 | 1.501E-04 | 1.126 | - | - |
| 1729 | ENSP00000350071 | STRN3 | 6.566E-03 | 1.124 | - | - |
| 1730 | ENSP00000315768 | STAT2 | 1.885E-03 | 1.124 | - | - |
| 1731 | ENSP00000257068 | MTNR1B | 2.409E-03 | 1.124 | - | - |
| 1732 | ENSP00000296921 | TLX3 | 7.405E-05 | 1.124 | - | - |
| 1733 | ENSP00000223095 | SERPINE1 | 7.283E-04 | 1.124 | - | - |
| 1734 | ENSP00000356125 | NUAK2 | 8.905E-03 | 1.124 | - | - |
| 1735 | ENSP00000263388 | NOTCH3 | 5.998E-04 | 1.124 | - | - |
| 1736 | ENSP00000263665 | CNTN3 | 3.674E-03 | 1.123 | - | - |
| 1737 | ENSP00000307235 | EIF2AK3 | 1.237E-03 | 1.123 | - | - |
| 1738 | ENSP00000296795 | TLR3 | 9.795E-04 | 1.123 | - | - |
| 1739 | ENSP00000322788 | MMP1 | 1.343E-03 | 1.123 | - | - |
| 1740 | ENSP00000308541 | F2 | 3.561E-04 | 1.122 | - | - |
| 1741 | ENSP00000337212 | SERPINB7 | 1.404E-04 | 1.122 | - | - |
| 1742 | ENSP00000384554 | CLDN5 | 4.347E-03 | 1.122 | - | - |
| 1743 | ENSP00000387699 | CREB1 | 4.334E-04 | 1.122 | - | - |
| 1744 | ENSP00000303394 | HSPB3 | 1.781E-03 | 1.122 | - | - |
| 1745 | ENSP00000269571 | ERBB2 | 3.792E-04 | 1.121 | - | - |
| 1746 | ENSP00000360216 | DNTT | 1.058E-03 | 1.120 | - | - |
| 1747 | ENSP00000344215 | TP53INP1 | 8.398E-03 | 1.120 | - | - |
| 1748 | ENSP00000367545 | TP73 | 2.687E-03 | 1.120 | - | - |
| 1749 | ENSP00000347858 | XIAP | 1.286E-03 | 1.119 | - | - |
| 1750 | ENSP00000201586 | SULT2B1 | 4.355E-05 | 1.118 | - | - |
| 1751 | ENSP00000216117 | HMOX1 | 8.300E-04 | 1.118 | - | - |
| 1752 | ENSP00000385422 | GPR61 | 1.092E-04 | 1.118 | - | - |
| 1753 | ENSP00000302665 | IGF1 | 5.255E-04 | 1.118 | - | - |
| 1754 | ENSP00000364277 | FGD1 | 3.953E-03 | 1.118 | - | - |
| 1755 | ENSP00000229239 | GAPDH | 1.251E-04 | 1.118 | - | - |
| 1756 | ENSP00000204604 | CHRD | 4.057E-05 | 1.118 | - | - |
| 1757 | ENSP00000365016 | IRS2 | 2.301E-03 | 1.117 | - | - |
| 1758 | ENSP00000417517 | TLR9 | 9.482E-04 | 1.117 | - | - |
| 1759 | ENSP00000239938 | EGR1 | 3.852E-04 | 1.115 | - | - |
| 1760 | ENSP00000355759 | PARP1 | 1.172E-03 | 1.115 | - | - |
| 1761 | ENSP00000302100 | LGALS4 | 1.047E-03 | 1.115 | - | - |
| 1762 | ENSP00000369699 | TMEM27 | 6.445E-05 | 1.115 | - | - |
| 1763 | ENSP00000315713 | SSH1 | 2.080E-03 | 1.114 | - | - |
| 1764 | ENSP00000340944 | PTPN11 | 7.608E-04 | 1.114 | - | - |
| 1765 | ENSP00000338018 | HIF1A | 6.126E-04 | 1.113 | - | - |
| 1766 | ENSP00000373301 | SH3BP5 | 3.925E-03 | 1.113 | - | - |
| 1767 | ENSP00000369218 | RBM17 | 6.630E-03 | 1.113 | - | - |
| 1768 | ENSP00000344235 | DUSP28 | 2.535E-03 | 1.111 | - | - |
| 1769 | ENSP00000261799 | PDGFRB | 3.158E-04 | 1.110 | - | - |
| 1770 | ENSP00000320768 | RCAN1 | 2.777E-03 | 1.110 | - | - |
| 1771 | ENSP00000250405 | BCL2L2 | 2.896E-03 | 1.110 | - | - |
| 1772 | ENSP00000373864 | RAPGEF3 | 2.400E-03 | 1.110 | - | - |
| 1773 | ENSP00000456337 | FLJ27352 | 3.812E-05 | 1.110 | - | - |
| 1774 | ENSP00000245451 | BMP4 | 5.293E-04 | 1.109 | - | - |
| 1775 | ENSP00000261336 | PZP | 9.007E-05 | 1.109 | - | - |
| 1776 | ENSP00000225964 | COL1A1 | 1.087E-03 | 1.107 | - | - |
| 1777 | ENSP00000312455 | CFLAR | 1.469E-03 | 1.107 | - | - |
| 1778 | ENSP00000265354 | SRF | 4.238E-04 | 1.106 | - | - |
| 1779 | ENSP00000361544 | NUP62CL | 7.114E-03 | 1.106 | - | - |
| 1780 | ENSP00000284273 | UBASH3B | 2.335E-03 | 1.106 | - | - |
| 1781 | ENSP00000293328 | STAT5B | 8.410E-04 | 1.105 | - | - |
| 1782 | ENSP00000351273 | CASP8 | 1.083E-03 | 1.105 | - | - |
| 1783 | ENSP00000380252 | NFE2L2 | 3.604E-03 | 1.103 | - | - |
| 1784 | ENSP00000383303 | DSCAM | 6.940E-03 | 1.103 | - | - |
| 1785 | ENSP00000293973 | NTN3 | 7.163E-05 | 1.101 | - | - |
| 1786 | ENSP00000264634 | WNT5A | 9.366E-04 | 1.101 | - | - |
| 1787 | ENSP00000375892 | AKT2 | 4.006E-04 | 1.101 | - | - |
| 1788 | ENSP00000346160 | DUSP19 | 1.728E-03 | 1.100 | - | - |
| 1789 | ENSP00000314897 | ANGPT2 | 2.644E-03 | 1.100 | - | - |
| 1790 | ENSP00000250971 | INS | 1.540E-04 | 1.098 | - | - |
| 1791 | ENSP00000222139 | EPOR | 1.629E-03 | 1.097 | - | - |
| 1792 | ENSP00000282478 | DSPP | 9.232E-05 | 1.096 | - | - |
| 1793 | ENSP00000262160 | SMAD2 | 7.110E-04 | 1.094 | - | - |
| 1794 | ENSP00000262445 | MAP2K4 | 2.870E-03 | 1.094 | - | - |
| 1795 | ENSP00000308208 | MMP14 | 1.804E-03 | 1.093 | - | - |
| 1796 | ENSP00000265371 | NRP1 | 2.502E-03 | 1.093 | - | - |
| 1797 | ENSP00000432104 | EBLN2 | 1.559E-04 | 1.092 | - | - |
| 1798 | ENSP00000362082 | CCND3 | 1.990E-03 | 1.092 | - | - |
| 1799 | ENSP00000268389 | IGSF6 | 1.321E-04 | 1.091 | - | - |
| 1800 | ENSP00000216797 | NFKBIA | 6.150E-04 | 1.090 | - | - |
| 1801 | ENSP00000379258 | GLI3 | 4.901E-04 | 1.090 | - | - |
| 1802 | ENSP00000216160 | TAB1 | 1.227E-03 | 1.090 | - | - |
| 1803 | ENSP00000365745 | CTTN | 1.565E-03 | 1.089 | - | - |
| 1804 | ENSP00000386169 | C12orf61 | 3.499E-05 | 1.088 | - | - |
| 1805 | ENSP00000263369 | MIA | 7.722E-05 | 1.087 | - | - |
| 1806 | ENSP00000352516 | DNMT1 | 9.920E-04 | 1.087 | - | - |
| 1807 | ENSP00000346300 | CRKL | 2.057E-03 | 1.086 | - | - |
| 1808 | ENSP00000264498 | FGF2 | 4.454E-04 | 1.085 | - | - |
| 1809 | ENSP00000239223 | DUSP1 | 9.691E-04 | 1.085 | - | - |
| 1810 | ENSP00000264110 | ATF2 | 8.236E-04 | 1.085 | - | - |
| 1811 | ENSP00000321656 | CDC25C | 1.526E-03 | 1.085 | - | - |
| 1812 | ENSP00000254722 | SERPINF1 | 2.672E-03 | 1.084 | - | - |
| 1813 | ENSP00000303351 | ITGB1 | 1.096E-03 | 1.084 | - | - |
| 1814 | ENSP00000263464 | BIRC3 | 1.415E-03 | 1.083 | - | - |
| 1815 | ENSP00000228938 | MGP | 3.471E-05 | 1.083 | - | - |
| 1816 | ENSP00000265495 | ELF2 | 6.604E-03 | 1.083 | - | - |
| 1817 | ENSP00000303706 | CDC25A | 1.421E-03 | 1.082 | - | - |
| 1818 | ENSP00000337459 | NOS1 | 6.219E-04 | 1.081 | - | - |
| 1819 | ENSP00000309124 | OLR1 | 6.468E-03 | 1.080 | - | - |
| 1820 | ENSP00000304437 | KNDC1 | 1.058E-04 | 1.080 | - | - |
| 1821 | ENSP00000237014 | TTR | 6.324E-04 | 1.080 | - | - |
| 1822 | ENSP00000249749 | DLL4 | 3.428E-03 | 1.079 | - | - |
| 1823 | ENSP00000271015 | - | 3.221E-03 | 1.079 | - | - |
| 1824 | ENSP00000228307 | PXN | 8.555E-04 | 1.079 | - | - |
| 1825 | ENSP00000353362 | CACNA1A | 9.863E-04 | 1.079 | - | - |
| 1826 | ENSP00000355751 | THBS2 | 1.045E-04 | 1.079 | - | - |
| 1827 | ENSP00000263253 | EP300 | 3.210E-04 | 1.078 | - | - |
| 1828 | ENSP00000242261 | TWIST1 | 1.043E-03 | 1.078 | - | - |
| 1829 | ENSP00000375863 | HNRNPUL1 | 3.599E-03 | 1.078 | - | - |
| 1830 | ENSP00000227507 | CCND1 | 3.118E-04 | 1.078 | - | - |
| 1831 | ENSP00000256646 | NOTCH2 | 5.330E-04 | 1.078 | - | - |
| 1832 | ENSP00000297261 | SHH | 5.001E-04 | 1.077 | - | - |
| 1833 | ENSP00000315878 | MS4A6A | 1.186E-04 | 1.077 | - | - |
| 1834 | ENSP00000261845 | MAPK6 | 3.746E-04 | 1.077 | - | - |
| 1835 | ENSP00000248923 | GGT1 | 4.826E-04 | 1.076 | - | - |
| 1836 | ENSP00000261769 | CDH1 | 3.825E-04 | 1.076 | - | - |
| 1837 | ENSP00000376436 | ETS1 | 1.422E-03 | 1.076 | - | - |
| 1838 | ENSP00000364252 | PLA2G2A | 9.690E-05 | 1.075 | - | - |
| 1839 | ENSP00000280665 | DCP1B | 7.674E-03 | 1.075 | - | - |
| 1840 | ENSP00000267569 | JDP2 | 1.048E-03 | 1.074 | - | - |
| 1841 | ENSP00000347134 | NET1 | 2.243E-03 | 1.074 | - | - |
| 1842 | ENSP00000302564 | BCL2L1 | 4.942E-04 | 1.074 | - | - |
| 1843 | ENSP00000304895 | IRS1 | 9.000E-04 | 1.073 | - | - |
| 1844 | ENSP00000357097 | FCER1A | 1.150E-04 | 1.073 | - | - |
| 1845 | ENSP00000261937 | FLT4 | 5.477E-04 | 1.072 | - | - |
| 1846 | ENSP00000233735 | REG1A | 7.025E-05 | 1.072 | - | - |
| 1847 | ENSP00000269141 | CDH2 | 8.292E-04 | 1.072 | - | - |
| 1848 | ENSP00000397351 | GRID2IP | 5.048E-05 | 1.071 | - | - |
| 1849 | ENSP00000326366 | PSEN1 | 8.056E-04 | 1.071 | - | - |
| 1850 | ENSP00000360200 | INADL | 1.420E-03 | 1.069 | - | - |
| 1851 | ENSP00000252809 | GDF15 | 1.995E-03 | 1.068 | - | - |
| 1852 | ENSP00000234313 | PLEK | 6.590E-04 | 1.068 | - | - |
| 1853 | ENSP00000363377 | FOXO4 | 9.359E-04 | 1.068 | - | - |
| 1854 | ENSP00000242796 | WDR83 | 7.110E-03 | 1.068 | - | - |
| 1855 | ENSP00000359787 | IFI44L | 8.672E-05 | 1.068 | - | - |
| 1856 | ENSP00000369375 | TEK | 5.209E-04 | 1.067 | - | - |
| 1857 | ENSP00000227758 | BIRC2 | 1.529E-03 | 1.067 | - | - |
| 1858 | ENSP00000361112 | CHRDL1 | 5.947E-05 | 1.066 | - | - |
| 1859 | ENSP00000245960 | CDC25B | 2.191E-03 | 1.066 | - | - |
| 1860 | ENSP00000360290 | PRKAA2 | 1.134E-03 | 1.066 | - | - |
| 1861 | ENSP00000263317 | NOX4 | 3.733E-03 | 1.065 | - | - |
| 1862 | ENSP00000301411 | NTF4 | 5.919E-05 | 1.065 | - | - |
| 1863 | ENSP00000005340 | DVL2 | 1.086E-03 | 1.065 | - | - |
| 1864 | ENSP00000353408 | MSN | 1.034E-03 | 1.065 | - | - |
| 1865 | ENSP00000261464 | TRAF5 | 9.222E-03 | 1.064 | - | - |
| 1866 | ENSP00000242057 | AHR | 1.874E-03 | 1.064 | - | - |
| 1867 | ENSP00000354558 | MTOR | 3.551E-04 | 1.064 | - | - |
| 1868 | ENSP00000284981 | APP | 4.765E-04 | 1.064 | - | - |
| 1869 | ENSP00000300303 | NAT16 | 2.129E-04 | 1.063 | - | - |
| 1870 | ENSP00000365514 | ZIC2 | 1.084E-03 | 1.063 | - | - |
| 1871 | ENSP00000353393 | F8 | 6.664E-05 | 1.063 | - | - |
| 1872 | ENSP00000231509 | NR3C1 | 1.058E-03 | 1.063 | - | - |
| 1873 | ENSP00000202788 | - | 1.144E-02 | 1.061 | - | - |
| 1874 | ENSP00000343313 | ATG5 | 2.333E-03 | 1.061 | - | - |
| 1875 | ENSP00000341189 | PTK2 | 9.637E-04 | 1.060 | - | - |
| 1876 | ENSP00000265192 | PAIP2 | 3.838E-02 | 1.060 | - | - |
| 1877 | ENSP00000368880 | FOXO1 | 5.354E-04 | 1.060 | - | - |
| 1878 | ENSP00000263026 | EEF2K | 2.527E-02 | 1.060 | - | - |
| 1879 | ENSP00000311713 | OXSR1 | 9.113E-03 | 1.058 | - | - |
| 1880 | ENSP00000245919 | FOSB | 7.297E-04 | 1.058 | - | - |
| 1881 | ENSP00000232424 | HES1 | 3.977E-03 | 1.058 | - | - |
| 1882 | ENSP00000356832 | SGK1 | 3.956E-04 | 1.058 | - | - |
| 1883 | ENSP00000261454 | PROX1 | 9.865E-05 | 1.058 | - | - |
| 1884 | ENSP00000340519 | LRRC37B | 4.838E-02 | 1.058 | - | - |
| 1885 | ENSP00000263556 | P4HA1 | 3.999E-03 | 1.055 | - | - |
| 1886 | ENSP00000250896 | MKNK2 | 9.654E-03 | 1.054 | - | - |
| 1887 | ENSP00000305422 | CEBPB | 1.508E-03 | 1.054 | - | - |
| 1888 | ENSP00000256495 | BHLHE40 | 1.753E-03 | 1.054 | - | - |
| 1889 | ENSP00000286122 | BANP | 9.311E-05 | 1.054 | - | - |
| 1890 | ENSP00000215368 | EFNA2 | 5.888E-05 | 1.054 | - | - |
| 1891 | ENSP00000261908 | NEO1 | 5.324E-03 | 1.053 | - | - |
| 1892 | ENSP00000264731 | TP63 | 9.765E-04 | 1.052 | - | - |
| 1893 | ENSP00000171111 | KEAP1 | 2.011E-03 | 1.052 | - | - |
| 1894 | ENSP00000249396 | SIRT2 | 1.275E-03 | 1.052 | - | - |
| 1895 | ENSP00000162391 | FOXJ2 | 1.137E-03 | 1.052 | - | - |
| 1896 | ENSP00000297494 | NOS3 | 3.822E-04 | 1.052 | - | - |
| 1897 | ENSP00000198767 | RRN3 | 5.374E-03 | 1.051 | - | - |
| 1898 | ENSP00000410626 | PHRF1 | 1.433E-04 | 1.051 | - | - |
| 1899 | ENSP00000281537 | TJP1 | 7.951E-04 | 1.051 | - | - |
| 1900 | ENSP00000345731 | DLG1 | 7.921E-04 | 1.051 | - | - |
| 1901 | ENSP00000206249 | ESR1 | 2.183E-04 | 1.050 | - | - |
| 1902 | ENSP00000363768 | C1QC | 6.014E-05 | 1.050 | - | - |
| 1903 | ENSP00000252029 | TYMP | 4.136E-03 | 1.050 | - | - |
| 1904 | ENSP00000360154 | OCRL | 1.840E-03 | 1.050 | - | - |
| 1905 | ENSP00000318089 | LPP | 2.554E-03 | 1.049 | - | - |
| 1906 | ENSP00000225296 | DHX33 | 3.072E-03 | 1.049 | - | - |
| 1907 | ENSP00000337332 | SIRT6 | 2.347E-03 | 1.049 | - | - |
| 1908 | ENSP00000345728 | ATP7A | 1.528E-03 | 1.049 | - | - |
| 1909 | ENSP00000226004 | DUSP3 | 1.717E-03 | 1.049 | - | - |
| 1910 | ENSP00000215832 | MAPK1 | 1.876E-04 | 1.048 | - | - |
| 1911 | ENSP00000000442 | ESRRA | 4.095E-03 | 1.048 | - | - |
| 1912 | ENSP00000284000 | CEBPG | 5.220E-03 | 1.048 | - | - |
| 1913 | ENSP00000256637 | SORT1 | 5.654E-03 | 1.048 | - | - |
| 1914 | ENSP00000262942 | ARPC1A | 2.628E-03 | 1.048 | - | - |
| 1915 | ENSP00000287706 | NTAN1 | 6.933E-05 | 1.047 | - | - |
| 1916 | ENSP00000171887 | TNS1 | 3.910E-04 | 1.047 | - | - |
| 1917 | ENSP00000262367 | CREBBP | 4.509E-04 | 1.047 | - | - |
| 1918 | ENSP00000339004 | FOXG1 | 6.937E-04 | 1.047 | - | - |
| 1919 | ENSP00000312664 | CASP2 | 4.535E-03 | 1.045 | - | - |
| 1920 | ENSP00000368538 | TNFRSF4 | 2.171E-04 | 1.045 | - | - |
| 1921 | ENSP00000263574 | APLP2 | 3.216E-03 | 1.044 | - | - |
| 1922 | ENSP00000222726 | HOXA5 | 2.682E-03 | 1.044 | - | - |
| 1923 | ENSP00000324633 | DEFB103B | 5.013E-05 | 1.043 | - | - |
| 1924 | ENSP00000293276 | CCL15 | 3.320E-04 | 1.043 | - | - |
| 1925 | ENSP00000299421 | ILK | 7.873E-04 | 1.043 | - | - |
| 1926 | ENSP00000293288 | BAX | 7.823E-04 | 1.042 | - | - |
| 1927 | ENSP00000296755 | MAP1B | 3.045E-03 | 1.041 | - | - |
| 1928 | ENSP00000306124 | PRKCE | 4.612E-04 | 1.041 | - | - |
| 1929 | ENSP00000330633 | CNTN2 | 2.343E-03 | 1.040 | - | - |
| 1930 | ENSP00000370571 | TH | 8.352E-04 | 1.039 | - | - |
| 1931 | ENSP00000363998 | ITCH | 1.235E-03 | 1.039 | - | - |
| 1932 | ENSP00000288840 | SMAD6 | 2.903E-03 | 1.038 | - | - |
| 1933 | ENSP00000212015 | SIRT1 | 4.757E-04 | 1.038 | - | - |
| 1934 | ENSP00000250448 | FOXA1 | 6.324E-04 | 1.037 | - | - |
| 1935 | ENSP00000250003 | MYOD1 | 5.247E-04 | 1.036 | - | - |
| 1936 | ENSP00000301838 | FADD | 2.450E-03 | 1.036 | - | - |
| 1937 | ENSP00000244741 | CDKN1A | 4.225E-04 | 1.035 | - | - |
| 1938 | ENSP00000268957 | TOB1 | 4.658E-03 | 1.035 | - | - |
| 1939 | ENSP00000332973 | SMAD3 | 6.640E-04 | 1.035 | - | - |
| 1940 | ENSP00000278823 | MTA2 | 3.442E-03 | 1.035 | - | - |
| 1941 | ENSP00000381399 | SLC2A11 | 7.901E-05 | 1.034 | - | - |
| 1942 | ENSP00000358335 | MAP3K7 | 6.085E-04 | 1.034 | - | - |
| 1943 | ENSP00000221419 | HNRNPL | 3.391E-03 | 1.034 | - | - |
| 1944 | ENSP00000282541 | GPD1L | 2.159E-03 | 1.033 | - | - |
| 1945 | ENSP00000326119 | PMAIP1 | 1.202E-02 | 1.033 | - | - |
| 1946 | ENSP00000310670 | KIAA1967 | 8.904E-03 | 1.032 | - | - |
| 1947 | ENSP00000282397 | FLT1 | 3.384E-04 | 1.032 | - | - |
| 1948 | ENSP00000356953 | SDHC | 5.478E-04 | 1.031 | - | - |
| 1949 | ENSP00000359380 | SCD | 9.342E-04 | 1.031 | - | - |
| 1950 | ENSP00000324856 | STK11 | 4.785E-04 | 1.031 | - | - |
| 1951 | ENSP00000247668 | TRAF2 | 3.028E-03 | 1.030 | - | - |
| 1952 | ENSP00000227251 | CRYAB | 9.425E-05 | 1.030 | - | - |
| 1953 | ENSP00000370256 | FOXC1 | 8.715E-04 | 1.030 | - | - |
| 1954 | ENSP00000317272 | MET | 3.985E-04 | 1.030 | - | - |
| 1955 | ENSP00000352101 | ANXA8L1 | 6.167E-05 | 1.030 | - | - |
| 1956 | ENSP00000263734 | EPAS1 | 1.817E-03 | 1.030 | - | - |
| 1957 | ENSP00000324422 | ZYX | 2.228E-03 | 1.029 | - | - |
| 1958 | ENSP00000205557 | ABCC6 | 1.241E-03 | 1.027 | - | - |
| 1959 | ENSP00000302486 | MAP2K1 | 5.290E-04 | 1.027 | - | - |
| 1960 | ENSP00000300399 | BPIFC | 1.040E-04 | 1.026 | - | - |
| 1961 | ENSP00000340691 | EIF4EBP1 | 1.531E-03 | 1.026 | - | - |
| 1962 | ENSP00000283249 | ITGB6 | 7.410E-05 | 1.026 | - | - |
| 1963 | ENSP00000327255 | PPM1A | 1.415E-03 | 1.026 | - | - |
| 1964 | ENSP00000354280 | PRSS3 | 1.331E-04 | 1.025 | - | - |
| 1965 | ENSP00000355523 | RGS7 | 6.203E-05 | 1.025 | - | - |
| 1966 | ENSP00000320866 | CALR | 8.321E-04 | 1.025 | - | - |
| 1967 | ENSP00000372191 | SIRT3 | 1.724E-03 | 1.025 | - | - |
| 1968 | ENSP00000274487 | ADAMTS19 | 1.211E-04 | 1.024 | - | - |
| 1969 | ENSP00000368884 | RPS6KA3 | 4.352E-04 | 1.024 | - | - |
| 1970 | ENSP00000275493 | EGFR | 2.749E-04 | 1.024 | - | - |
| 1971 | ENSP00000360676 | KTI12 | 1.148E-04 | 1.024 | - | - |
| 1972 | ENSP00000320709 | ADIPOQ | 1.548E-03 | 1.023 | - | - |
| 1973 | ENSP00000422464 | PDGFC | 7.149E-05 | 1.023 | - | - |
| 1974 | ENSP00000248437 | TUBA4A | 6.990E-04 | 1.022 | - | - |
| 1975 | ENSP00000256078 | KRAS | 1.895E-04 | 1.022 | - | - |
| 1976 | ENSP00000296930 | NPM1 | 7.415E-04 | 1.022 | - | - |
| 1977 | ENSP00000348793 | AFF3 | 7.884E-05 | 1.022 | - | - |
| 1978 | ENSP00000314116 | TMEM107 | 9.866E-05 | 1.021 | - | - |
| 1979 | ENSP00000327758 | NKX2-5 | 5.228E-05 | 1.021 | - | - |
| 1980 | ENSP00000325690 | CARM1 | 1.834E-03 | 1.020 | - | - |
| 1981 | ENSP00000219169 | NUTF2 | 3.594E-03 | 1.020 | - | - |
| 1982 | ENSP00000364163 | NOTCH4 | 6.144E-04 | 1.019 | - | - |
| 1983 | ENSP00000338934 | EZR | 7.778E-04 | 1.019 | - | - |
| 1984 | ENSP00000278616 | ATM | 4.579E-04 | 1.019 | - | - |
| 1985 | ENSP00000421736 | CR1L | 3.935E-05 | 1.018 | - | - |
| 1986 | ENSP00000287641 | SST | 6.198E-05 | 1.018 | - | - |
| 1987 | ENSP00000236959 | ATIC | 1.256E-03 | 1.018 | - | - |
| 1988 | ENSP00000356213 | VIP | 6.012E-05 | 1.018 | - | - |
| 1989 | ENSP00000342830 | RDX | 1.283E-03 | 1.017 | - | - |
| 1990 | ENSP00000264867 | PPARGC1A | 1.303E-03 | 1.017 | - | - |
| 1991 | ENSP00000238628 | IFT43 | 3.820E-03 | 1.016 | - | - |
| 1992 | ENSP00000358596 | DUSP5 | 1.363E-03 | 1.015 | - | - |
| 1993 | ENSP00000225792 | DDX5 | 7.409E-04 | 1.015 | - | - |
| 1994 | ENSP00000256104 | FABP4 | 4.747E-05 | 1.014 | - | - |
| 1995 | ENSP00000361878 | CAP1 | 8.486E-04 | 1.014 | - | - |
| 1996 | ENSP00000371787 | CEMP1 | 1.324E-04 | 1.013 | - | - |
| 1997 | ENSP00000267163 | RB1 | 5.243E-04 | 1.013 | - | - |
| 1998 | ENSP00000360025 | GADD45A | 1.314E-03 | 1.013 | - | - |
| 1999 | ENSP00000381214 | RGPD2 | 8.589E-04 | 1.011 | - | - |
| 2000 | ENSP00000262320 | AXIN1 | 9.506E-04 | 1.009 | - | - |
| 2001 | ENSP00000224784 | ACTA2 | 2.266E-04 | 1.009 | - | - |
| 2002 | ENSP00000290573 | HK2 | 7.195E-04 | 1.009 | - | - |
| 2003 | ENSP00000347997 | UBQLN3 | 8.274E-05 | 1.008 | - | - |
| 2004 | ENSP00000270142 | SOD1 | 1.059E-03 | 1.008 | - | - |
| 2005 | ENSP00000355775 | MIXL1 | 6.033E-05 | 1.008 | - | - |
| 2006 | ENSP00000314499 | GAK | 2.951E-04 | 1.008 | - | - |
| 2007 | ENSP00000259818 | TUBB2B | 6.956E-04 | 1.007 | - | - |
| 2008 | ENSP00000291582 | AIRE | 5.323E-05 | 1.006 | - | - |
| 2009 | ENSP00000339527 | FOXO3 | 5.437E-04 | 1.005 | - | - |
| 2010 | ENSP00000348986 | INS-IGF2 | 9.953E-05 | 1.005 | - | - |
| 2011 | ENSP00000078429 | GNA11 | 4.898E-04 | 1.005 | - | - |
| 2012 | ENSP00000220003 | CSK | 5.269E-04 | 1.004 | - | - |
| 2013 | ENSP00000343040 | HMGB1 | 9.595E-04 | 1.004 | - | - |
| 2014 | ENSP00000302603 | ZNF556 | 2.441E-03 | 1.004 | - | - |
| 2015 | ENSP00000353874 | TLR9 | 8.771E-04 | 1.003 | - | - |
| 2016 | ENSP00000216225 | RBX1 | 1.062E-03 | 1.002 | - | - |
| 2017 | ENSP00000256178 | LYVE1 | 5.257E-05 | 1.002 | - | - |
| 2018 | ENSP00000407552 | GAS7 | 1.771E-04 | 1.002 | - | - |
| 2019 | ENSP00000285379 | CA2 | 4.473E-04 | 1.001 | - | - |
| 2020 | ENSP00000264668 | MTRR | 1.963E-03 | 1.000 | - | - |
| 2021 | ENSP00000366506 | HRH2 | 1.337E-04 | 0.999 | - | - |
| 2022 | ENSP00000249923 | COPB1 | 1.770E-03 | 0.999 | - | - |
| 2023 | ENSP00000339787 | ACSL4 | 2.348E-03 | 0.998 | - | - |
| 2024 | ENSP00000354522 | TOP1 | 8.472E-04 | 0.997 | - | - |
| 2025 | ENSP00000302046 | RNASE6 | 1.183E-04 | 0.997 | - | - |
| 2026 | ENSP00000356919 | ATF6 | 2.449E-03 | 0.997 | - | - |
| 2027 | ENSP00000351015 | NF1 | 5.599E-04 | 0.997 | - | - |
| 2028 | ENSP00000264708 | POMC | 5.126E-04 | 0.997 | - | - |
| 2029 | ENSP00000264071 | TUBB4A | 5.976E-04 | 0.996 | - | - |
| 2030 | ENSP00000320935 | SLC2A4 | 6.380E-04 | 0.995 | - | - |
| 2031 | ENSP00000364801 | HSPA1B | 5.218E-04 | 0.994 | - | - |
| 2032 | ENSP00000341821 | FPR3 | 8.536E-05 | 0.994 | - | - |
| 2033 | ENSP00000370034 | TLR7 | 2.202E-04 | 0.993 | - | - |
| 2034 | ENSP00000225577 | RPS6KB1 | 3.370E-04 | 0.992 | - | - |
| 2035 | ENSP00000261007 | CHRNA1 | 6.643E-05 | 0.992 | - | - |
| 2036 | ENSP00000314067 | PAK2 | 9.731E-04 | 0.992 | - | - |
| 2037 | ENSP00000258749 | AOAH | 9.354E-05 | 0.992 | - | - |
| 2038 | ENSP00000270202 | AKT1 | 1.737E-04 | 0.992 | - | - |
| 2039 | ENSP00000430487 | MATN2 | 1.613E-04 | 0.991 | - | - |
| 2040 | ENSP00000242839 | ATP7B | 1.485E-03 | 0.990 | - | - |
| 2041 | ENSP00000364246 | PLA2G2D | 4.154E-05 | 0.990 | - | - |
| 2042 | ENSP00000229922 | CAP2 | 8.957E-04 | 0.989 | - | - |
| 2043 | ENSP00000320089 | NKX2-6 | 7.790E-05 | 0.989 | - | - |
| 2044 | ENSP00000225688 | RASD1 | 3.277E-04 | 0.988 | - | - |
| 2045 | ENSP00000362608 | PIM1 | 5.244E-04 | 0.987 | - | - |
| 2046 | ENSP00000364805 | HSPA1L | 5.204E-04 | 0.986 | - | - |
| 2047 | ENSP00000328181 | NOG | 5.881E-05 | 0.985 | - | - |
| 2048 | ENSP00000331602 | PRKCD | 4.694E-04 | 0.985 | - | - |
| 2049 | ENSP00000303830 | INSR | 2.998E-04 | 0.985 | - | - |
| 2050 | ENSP00000282441 | YAP1 | 9.448E-04 | 0.983 | - | - |
| 2051 | ENSP00000356530 | RNASEL | 5.054E-04 | 0.983 | - | - |
| 2052 | ENSP00000248553 | HSPB1 | 1.028E-03 | 0.983 | - | - |
| 2053 | ENSP00000382659 | TRPV1 | 7.936E-05 | 0.983 | - | - |
| 2054 | ENSP00000472280 | EMR2 | 9.363E-05 | 0.981 | - | - |
| 2055 | ENSP00000276571 | CRH | 6.950E-05 | 0.981 | - | - |
| 2056 | ENSP00000346032 | ANXA2 | 1.428E-03 | 0.980 | - | - |
| 2057 | ENSP00000205143 | DLL3 | 5.505E-05 | 0.979 | - | - |
| 2058 | ENSP00000371785 | OLIG1 | 6.373E-05 | 0.978 | - | - |
| 2059 | ENSP00000351908 | MAP3K5 | 8.506E-04 | 0.978 | - | - |
| 2060 | ENSP00000261205 | SYT1 | 1.900E-03 | 0.978 | - | - |
| 2061 | ENSP00000324740 | YES1 | 4.310E-04 | 0.978 | - | - |
| 2062 | ENSP00000354822 | XAF1 | 1.571E-04 | 0.977 | - | - |
| 2063 | ENSP00000273320 | ZKSCAN7 | 1.085E-03 | 0.975 | - | - |
| 2064 | ENSP00000344563 | CLEC12B | 7.529E-05 | 0.974 | - | - |
| 2065 | ENSP00000245304 | RAP2A | 3.772E-04 | 0.973 | - | - |
| 2066 | ENSP00000262238 | YY1 | 4.979E-04 | 0.972 | - | - |
| 2067 | ENSP00000337853 | TRAF6 | 1.122E-03 | 0.972 | - | - |
| 2068 | ENSP00000326042 | TUBA3D | 7.401E-04 | 0.972 | - | - |
| 2069 | ENSP00000344352 | ATF3 | 6.105E-04 | 0.971 | - | - |
| 2070 | ENSP00000464034 | SOCS7 | 9.524E-05 | 0.971 | - | - |
| 2071 | ENSP00000363641 | TXN | 4.917E-04 | 0.971 | - | - |
| 2072 | ENSP00000321835 | ETV4 | 2.578E-03 | 0.970 | - | - |
| 2073 | ENSP00000005257 | RALA | 2.985E-04 | 0.970 | - | - |
| 2074 | ENSP00000341658 | DUSP15 | 2.098E-03 | 0.970 | - | - |
| 2075 | ENSP00000358212 | C10orf82 | 9.417E-06 | 0.968 | - | - |
| 2076 | ENSP00000253109 | ANGPTL6 | 1.102E-04 | 0.967 | - | - |
| 2077 | ENSP00000357789 | FLG | 1.284E-04 | 0.966 | - | - |
| 2078 | ENSP00000256682 | ARF3 | 6.248E-04 | 0.965 | - | - |
| 2079 | ENSP00000241052 | CAT | 2.109E-04 | 0.965 | - | - |
| 2080 | ENSP00000238738 | RHOQ | 3.649E-04 | 0.964 | - | - |
| 2081 | ENSP00000336790 | ATF4 | 1.627E-03 | 0.964 | - | - |
| 2082 | ENSP00000217233 | TRIB3 | 5.178E-04 | 0.962 | - | - |
| 2083 | ENSP00000241463 | RASL11A | 4.482E-04 | 0.961 | - | - |
| 2084 | ENSP00000366493 | CGB7 | 5.894E-05 | 0.960 | - | - |
| 2085 | ENSP00000268035 | IGF1R | 3.488E-04 | 0.960 | - | - |
| 2086 | ENSP00000297130 | MYOZ3 | 8.798E-05 | 0.959 | - | - |
| 2087 | ENSP00000362361 | CDK9 | 6.710E-04 | 0.959 | - | - |
| 2088 | ENSP00000370842 | IL33 | 1.566E-04 | 0.959 | - | - |
| 2089 | ENSP00000227256 | DBX1 | 5.523E-05 | 0.958 | - | - |
| 2090 | ENSP00000327647 | CRADD | 1.832E-04 | 0.958 | - | - |
| 2091 | ENSP00000290921 | CTBP1 | 1.377E-03 | 0.957 | - | - |
| 2092 | ENSP00000465500 | TNFRSF11A | 7.671E-05 | 0.957 | - | - |
| 2093 | ENSP00000262187 | RHEB | 3.616E-04 | 0.956 | - | - |
| 2094 | ENSP00000257904 | CDK4 | 2.205E-04 | 0.955 | - | - |
| 2095 | ENSP00000293218 | UNK | 8.940E-05 | 0.955 | - | - |
| 2096 | ENSP00000401338 | TMEM8A | 8.292E-05 | 0.955 | - | - |
| 2097 | ENSP00000330138 | SSTR3 | 9.708E-05 | 0.954 | - | - |
| 2098 | ENSP00000357733 | PRR9 | 3.090E-04 | 0.954 | - | - |
| 2099 | ENSP00000293813 | - | 5.651E-04 | 0.954 | - | - |
| 2100 | ENSP00000297145 | C7orf60 | 2.453E-04 | 0.952 | - | - |
| 2101 | ENSP00000328016 | TNFAIP8L3 | 8.270E-05 | 0.952 | - | - |
| 2102 | ENSP00000364361 | MLTK | 7.177E-04 | 0.951 | - | - |
| 2103 | ENSP00000352929 | CSNK1E | 6.773E-04 | 0.951 | - | - |
| 2104 | ENSP00000260753 | KIF20B | 1.496E-03 | 0.946 | - | - |
| 2105 | ENSP00000310219 | HSPA6 | 4.467E-04 | 0.945 | - | - |
| 2106 | ENSP00000296327 | SLC51A | 8.573E-05 | 0.944 | - | - |
| 2107 | ENSP00000265734 | CDK6 | 2.800E-04 | 0.943 | - | - |
| 2108 | ENSP00000247207 | HSPA2 | 4.188E-04 | 0.943 | - | - |
| 2109 | ENSP00000301071 | TUBA1A | 5.757E-04 | 0.942 | - | - |
| 2110 | ENSP00000233027 | NEK4 | 1.416E-04 | 0.942 | - | - |
| 2111 | ENSP00000382218 | ZSWIM7 | 1.594E-04 | 0.942 | - | - |
| 2112 | ENSP00000298125 | WDFY2 | 3.236E-04 | 0.940 | - | - |
| 2113 | ENSP00000262269 | MYH14 | 2.466E-04 | 0.939 | - | - |
| 2114 | ENSP00000256733 | SAA2 | 8.901E-05 | 0.939 | - | - |
| 2115 | ENSP00000276052 | CDK16 | 2.615E-04 | 0.938 | - | - |
| 2116 | ENSP00000268864 | RASL10B | 4.332E-04 | 0.937 | - | - |
| 2117 | ENSP00000357721 | S100A8 | 2.014E-04 | 0.937 | - | - |
| 2118 | ENSP00000256785 | CFHR5 | 1.965E-04 | 0.937 | - | - |
| 2119 | ENSP00000256442 | CCNB1 | 4.314E-04 | 0.936 | - | - |
| 2120 | ENSP00000227348 | CRTAM | 1.111E-04 | 0.935 | - | - |
| 2121 | ENSP00000364702 | NBPF9 | 4.134E-04 | 0.934 | - | - |
| 2122 | ENSP00000340943 | CRHR2 | 5.437E-05 | 0.933 | - | - |
| 2123 | ENSP00000308472 | EIF4EBP3 | 9.181E-05 | 0.933 | - | - |
| 2124 | ENSP00000330276 | PTPN21 | 4.797E-05 | 0.933 | - | - |
| 2125 | ENSP00000355870 | HLX | 1.033E-04 | 0.930 | - | - |
| 2126 | ENSP00000249440 | HOXD3 | 5.091E-05 | 0.929 | - | - |
| 2127 | ENSP00000369497 | BRCA2 | 1.085E-03 | 0.928 | - | - |
| 2128 | ENSP00000263754 | KAT2B | 3.451E-04 | 0.926 | - | - |
| 2129 | ENSP00000369695 | MLLT3 | 8.889E-05 | 0.925 | - | - |
| 2130 | ENSP00000364802 | HSPA1A | 4.313E-04 | 0.922 | - | - |
| 2131 | ENSP00000339001 | TUBB | 6.895E-04 | 0.921 | - | - |
| 2132 | ENSP00000248673 | ZFP36 | 3.137E-03 | 0.919 | - | - |
| 2133 | ENSP00000318195 | NCL | 1.820E-03 | 0.919 | - | - |
| 2134 | ENSP00000373698 | - | 1.034E-04 | 0.917 | - | - |
| 2135 | ENSP00000359891 | ZDHHC16 | 1.868E-04 | 0.917 | - | - |
| 2136 | ENSP00000378735 | DNAJC6 | 8.117E-04 | 0.917 | - | - |
| 2137 | ENSP00000267205 | RHOF | 3.772E-04 | 0.916 | - | - |
| 2138 | ENSP00000350878 | S1PR3 | 8.786E-05 | 0.915 | - | - |
| 2139 | ENSP00000341674 | ANXA8 | 6.515E-05 | 0.914 | - | - |
| 2140 | ENSP00000332995 | SETD8 | 6.173E-04 | 0.913 | - | - |
| 2141 | ENSP00000370115 | SERPINB1 | 9.447E-05 | 0.911 | - | - |
| 2142 | ENSP00000360266 | JUN | 3.691E-04 | 0.911 | - | - |
| 2143 | ENSP00000381486 | NOTO | 9.642E-05 | 0.911 | - | - |
| 2144 | ENSP00000227378 | HSPA8 | 2.748E-04 | 0.910 | - | - |
| 2145 | ENSP00000340874 | MEF2C | 4.932E-04 | 0.910 | - | - |
| 2146 | ENSP00000357380 | MUC1 | 1.186E-04 | 0.909 | - | - |
| 2147 | ENSP00000230354 | TBP | 2.989E-04 | 0.908 | - | - |
| 2148 | ENSP00000322898 | EBF1 | 5.437E-05 | 0.907 | - | - |
| 2149 | ENSP00000361892 | STK4 | 7.442E-04 | 0.906 | - | - |
| 2150 | ENSP00000346389 | MEF2A | 4.287E-04 | 0.906 | - | - |
| 2151 | ENSP00000263923 | KDR | 3.162E-04 | 0.905 | - | - |
| 2152 | ENSP00000365730 | STK24 | 7.107E-04 | 0.904 | - | - |
| 2153 | ENSP00000354777 | TBKBP1 | 1.038E-04 | 0.904 | - | - |
| 2154 | ENSP00000357036 | SLAMF6 | 1.147E-04 | 0.901 | - | - |
| 2155 | ENSP00000381921 | DSCR4 | 7.398E-05 | 0.900 | - | - |
| 2156 | ENSP00000274353 | BHMT | 7.701E-05 | 0.900 | - | - |
| 2157 | ENSP00000331242 | KREMEN1 | 7.937E-05 | 0.899 | - | - |
| 2158 | ENSP00000339845 | DROSHA | 5.979E-04 | 0.899 | - | - |
| 2159 | ENSP00000252453 | C19orf80 | 1.264E-04 | 0.899 | - | - |
| 2160 | ENSP00000394472 | BTBD18 | 5.771E-05 | 0.899 | - | - |
| 2161 | ENSP00000341289 | TUBB4B | 6.185E-04 | 0.899 | - | - |
| 2162 | ENSP00000342307 | FOXM1 | 5.348E-04 | 0.898 | - | - |
| 2163 | ENSP00000300184 | MS4A7 | 1.515E-04 | 0.897 | - | - |
| 2164 | ENSP00000272233 | RHOB | 3.052E-04 | 0.897 | - | - |
| 2165 | ENSP00000285735 | RHOC | 3.078E-04 | 0.897 | - | - |
| 2166 | ENSP00000343490 | LCTL | 5.955E-05 | 0.897 | - | - |
| 2167 | ENSP00000350512 | COPS5 | 1.134E-03 | 0.896 | - | - |
| 2168 | ENSP00000369703 | TUBB2A | 5.695E-04 | 0.896 | - | - |
| 2169 | ENSP00000353878 | BAK1 | 8.321E-04 | 0.895 | - | - |
| 2170 | ENSP00000386210 | TAS2R40 | 8.507E-05 | 0.891 | - | - |
| 2171 | ENSP00000308549 | ADORA1 | 6.921E-05 | 0.889 | - | - |
| 2172 | ENSP00000352400 | NUP214 | 2.336E-03 | 0.889 | - | - |
| 2173 | ENSP00000367013 | TNFRSF25 | 1.655E-04 | 0.887 | - | - |
| 2174 | ENSP00000469049 | TNFSF14 | 1.298E-04 | 0.887 | - | - |
| 2175 | ENSP00000205386 | LAMB4 | 7.511E-05 | 0.887 | - | - |
| 2176 | ENSP00000359114 | COL11A1 | 7.720E-05 | 0.885 | - | - |
| 2177 | ENSP00000347890 | DNM2 | 3.952E-04 | 0.883 | - | - |
| 2178 | ENSP00000252998 | RBBP8NL | 7.885E-05 | 0.881 | - | - |
| 2179 | ENSP00000310447 | GLS2 | 4.524E-05 | 0.881 | - | - |
| 2180 | ENSP00000459962 | SHPK | 7.643E-05 | 0.879 | - | - |
| 2181 | ENSP00000353679 | MME | 9.432E-05 | 0.878 | - | - |
| 2182 | ENSP00000331766 | CLECL1 | 7.312E-05 | 0.876 | - | - |
| 2183 | ENSP00000358595 | CGA | 1.031E-04 | 0.872 | - | - |
| 2184 | ENSP00000344549 | CARD14 | 9.462E-05 | 0.872 | - | - |
| 2185 | ENSP00000331741 | HOXB2 | 4.858E-05 | 0.871 | - | - |
| 2186 | ENSP00000328444 | KRTAP4-8 | 3.356E-04 | 0.871 | - | - |
| 2187 | ENSP00000313513 | ANKAR | 1.323E-04 | 0.871 | - | - |
| 2188 | ENSP00000334314 | EML1 | 7.068E-05 | 0.870 | - | - |
| 2189 | ENSP00000413496 | FGF12 | 1.255E-04 | 0.870 | - | - |
| 2190 | ENSP00000304283 | RAC3 | 2.312E-04 | 0.870 | - | - |
| 2191 | ENSP00000262811 | MAST3 | 6.166E-05 | 0.868 | - | - |
| 2192 | ENSP00000311113 | JUP | 7.948E-04 | 0.868 | - | - |
| 2193 | ENSP00000221992 | CEACAM5 | 6.158E-05 | 0.867 | - | - |
| 2194 | ENSP00000257905 | PPP1R1A | 7.319E-05 | 0.866 | - | - |
| 2195 | ENSP00000241124 | GJB6 | 3.955E-05 | 0.864 | - | - |
| 2196 | ENSP00000357362 | THBS3 | 9.028E-05 | 0.864 | - | - |
| 2197 | ENSP00000359489 | AFF2 | 5.984E-05 | 0.864 | - | - |
| 2198 | ENSP00000364310 | H2AFX | 6.948E-04 | 0.863 | - | - |
| 2199 | ENSP00000432034 | CCL15-CCL14 | 1.467E-04 | 0.862 | - | - |
| 2200 | ENSP00000257981 | KCNH3 | 6.419E-05 | 0.861 | - | - |
| 2201 | ENSP00000359663 | CD40LG | 8.180E-04 | 0.861 | - | - |
| 2202 | ENSP00000257189 | DSG3 | 7.610E-05 | 0.859 | - | - |
| 2203 | ENSP00000322924 | HTR1F | 6.853E-05 | 0.858 | - | - |
| 2204 | ENSP00000257555 | HNF1A | 6.494E-05 | 0.855 | - | - |
| 2205 | ENSP00000348918 | SAA1 | 1.347E-04 | 0.854 | - | - |
| 2206 | ENSP00000362304 | ADAMTS14 | 1.104E-04 | 0.854 | - | - |
| 2207 | ENSP00000357156 | CD5L | 5.507E-05 | 0.853 | - | - |
| 2208 | ENSP00000365081 | APOM | 8.141E-05 | 0.852 | - | - |
| 2209 | ENSP00000272102 | ARF1 | 4.734E-04 | 0.852 | - | - |
| 2210 | ENSP00000442477 | ASIC5 | 5.909E-05 | 0.852 | - | - |
| 2211 | ENSP00000351905 | TGFBR2 | 1.141E-04 | 0.851 | - | - |
| 2212 | ENSP00000302961 | HSPA4 | 3.102E-04 | 0.851 | - | - |
| 2213 | ENSP00000226355 | AFM | 8.465E-05 | 0.850 | - | - |
| 2214 | ENSP00000443432 | IDO2 | 3.553E-05 | 0.850 | - | - |
| 2215 | ENSP00000342560 | HRH3 | 7.832E-05 | 0.849 | - | - |
| 2216 | ENSP00000339992 | MYB | 3.221E-04 | 0.849 | - | - |
| 2217 | ENSP00000340274 | RAP2C | 4.348E-04 | 0.848 | - | - |
| 2218 | ENSP00000366729 | TNFRSF9 | 1.610E-04 | 0.847 | - | - |
| 2219 | ENSP00000301072 | TUBA1C | 6.287E-04 | 0.846 | - | - |
| 2220 | ENSP00000330070 | NPW | 7.879E-05 | 0.845 | - | - |
| 2221 | ENSP00000256039 | DYDC2 | 1.003E-04 | 0.844 | - | - |
| 2222 | ENSP00000350941 | SRC | 2.811E-04 | 0.843 | - | - |
| 2223 | ENSP00000445366 | DND1 | 1.677E-03 | 0.843 | - | - |
| 2224 | ENSP00000281043 | MYCN | 1.313E-03 | 0.842 | - | - |
| 2225 | ENSP00000240335 | TBX4 | 4.006E-05 | 0.841 | - | - |
| 2226 | ENSP00000386229 | RGS14 | 7.255E-05 | 0.840 | - | - |
| 2227 | ENSP00000358106 | PREP | 6.423E-05 | 0.840 | - | - |
| 2228 | ENSP00000336799 | TUBA1B | 5.539E-04 | 0.838 | - | - |
| 2229 | ENSP00000334879 | SOWAHB | 8.694E-05 | 0.837 | - | - |
| 2230 | ENSP00000373570 | TYRP1 | 6.833E-05 | 0.837 | - | - |
| 2231 | ENSP00000262995 | GAB1 | 8.990E-05 | 0.836 | - | - |
| 2232 | ENSP00000295718 | PTPRN | 6.437E-05 | 0.836 | - | - |
| 2233 | ENSP00000262017 | - | 7.548E-05 | 0.834 | - | - |
| 2234 | ENSP00000175756 | PTPN18 | 4.487E-05 | 0.831 | - | - |
| 2235 | ENSP00000283195 | RANBP2 | 2.902E-04 | 0.831 | - | - |
| 2236 | ENSP00000261340 | CLEC2D | 1.088E-04 | 0.828 | - | - |
| 2237 | ENSP00000324806 | GSK3B | 2.585E-04 | 0.826 | - | - |
| 2238 | ENSP00000354579 | CMTM3 | 1.026E-04 | 0.825 | - | - |
| 2239 | ENSP00000282096 | PDE3B | 5.511E-05 | 0.825 | - | - |
| 2240 | ENSP00000359552 | PKN2 | 5.722E-04 | 0.824 | - | - |
| 2241 | ENSP00000362734 | CRB2 | 6.518E-05 | 0.824 | - | - |
| 2242 | ENSP00000263071 | SCARF1 | 4.481E-05 | 0.822 | - | - |
| 2243 | ENSP00000314173 | SAMD12 | 7.528E-05 | 0.822 | - | - |
| 2244 | ENSP00000357150 | CD1B | 1.047E-04 | 0.821 | - | - |
| 2245 | ENSP00000232508 | CYB561D2 | 1.428E-04 | 0.821 | - | - |
| 2246 | ENSP00000455951 | KLRK1 | 1.234E-04 | 0.820 | - | - |
| 2247 | ENSP00000340988 | LDLRAD2 | 8.979E-05 | 0.819 | - | - |
| 2248 | ENSP00000410395 | - | 5.806E-05 | 0.819 | - | - |
| 2249 | ENSP00000346442 | LRMP | 6.621E-05 | 0.817 | - | - |
| 2250 | ENSP00000375429 | KRTAP3-2 | 3.798E-05 | 0.817 | - | - |
| 2251 | ENSP00000246104 | SCRT2 | 5.368E-05 | 0.817 | - | - |
| 2252 | ENSP00000330374 | GIMAP6 | 6.913E-04 | 0.812 | - | - |
| 2253 | ENSP00000313420 | PRKDC | 4.937E-04 | 0.809 | - | - |
| 2254 | ENSP00000331327 | WT1 | 4.660E-04 | 0.808 | - | - |
| 2255 | ENSP00000346148 | PRKAA1 | 1.089E-03 | 0.807 | - | - |
| 2256 | ENSP00000386184 | C5orf54 | 1.184E-04 | 0.807 | - | - |
| 2257 | ENSP00000416753 | MUC15 | 1.060E-04 | 0.806 | - | - |
| 2258 | ENSP00000366015 | ANGPTL7 | 6.551E-05 | 0.806 | - | - |
| 2259 | ENSP00000296585 | ITGA2 | 2.087E-04 | 0.802 | - | - |
| 2260 | ENSP00000314458 | CDC42 | 2.369E-04 | 0.801 | - | - |
| 2261 | ENSP00000325589 | PTPRCAP | 8.048E-05 | 0.800 | - | - |
| 2262 | ENSP00000219162 | MT4 | 2.719E-04 | 0.800 | - | - |
| 2263 | ENSP00000357123 | MNDA | 5.682E-05 | 0.799 | - | - |
| 2264 | ENSP00000363773 | C1QA | 1.659E-04 | 0.797 | - | - |
| 2265 | ENSP00000361021 | PTEN | 3.643E-04 | 0.795 | - | - |
| 2266 | ENSP00000383421 | RBM11 | 6.799E-05 | 0.795 | - | - |
| 2267 | ENSP00000321987 | TSPEAR | 5.349E-05 | 0.793 | - | - |
| 2268 | ENSP00000357112 | AIM2 | 1.025E-04 | 0.792 | - | - |
| 2269 | ENSP00000384273 | RELA | 5.783E-04 | 0.789 | - | - |
| 2270 | ENSP00000420037 | PIGP | 5.894E-05 | 0.787 | - | - |
| 2271 | ENSP00000302967 | HDAC3 | 3.208E-04 | 0.783 | - | - |
| 2272 | ENSP00000295108 | NEUROD1 | 3.984E-05 | 0.782 | - | - |
| 2273 | ENSP00000348316 | HN1 | 1.284E-04 | 0.780 | - | - |
| 2274 | ENSP00000357080 | VSIG8 | 1.096E-04 | 0.780 | - | - |
| 2275 | ENSP00000320951 | DEFB103A | 4.308E-05 | 0.779 | - | - |
| 2276 | ENSP00000353259 | CD300A | 1.131E-04 | 0.778 | - | - |
| 2277 | ENSP00000289248 | RHBDL2 | 5.169E-05 | 0.778 | - | - |
| 2278 | ENSP00000350402 | HHLA2 | 6.910E-05 | 0.778 | - | - |
| 2279 | ENSP00000357020 | LY9 | 9.534E-05 | 0.776 | - | - |
| 2280 | ENSP00000371875 | NKX3-2 | 4.555E-05 | 0.775 | - | - |
| 2281 | ENSP00000433290 | LTB4R2 | 4.892E-05 | 0.775 | - | - |
| 2282 | ENSP00000236850 | APOA1 | 6.927E-05 | 0.775 | - | - |
| 2283 | ENSP00000363689 | ID3 | 6.560E-05 | 0.775 | - | - |
| 2284 | ENSP00000278282 | SCGB1A1 | 6.956E-05 | 0.767 | - | - |
| 2285 | ENSP00000225893 | HNF1B | 6.559E-05 | 0.767 | - | - |
| 2286 | ENSP00000298110 | GPR101 | 5.756E-05 | 0.767 | - | - |
| 2287 | ENSP00000255192 | BHMT2 | 7.456E-05 | 0.767 | - | - |
| 2288 | ENSP00000234816 | ANGPTL1 | 7.734E-05 | 0.765 | - | - |
| 2289 | ENSP00000283946 | FBXO36 | 1.206E-04 | 0.765 | - | - |
| 2290 | ENSP00000401701 | PRSS42 | 4.176E-05 | 0.765 | - | - |
| 2291 | ENSP00000385006 | THAP4 | 2.099E-04 | 0.765 | - | - |
| 2292 | ENSP00000317674 | APOL1 | 4.424E-05 | 0.764 | - | - |
| 2293 | ENSP00000363822 | AR | 3.855E-04 | 0.763 | - | - |
| 2294 | ENSP00000341551 | SMAD4 | 7.017E-04 | 0.762 | - | - |
| 2295 | ENSP00000322801 | OR52N2 | 3.388E-05 | 0.762 | - | - |
| 2296 | ENSP00000378033 | KCNK4 | 9.964E-05 | 0.761 | - | - |
| 2297 | ENSP00000415151 | CBFB | 1.789E-03 | 0.759 | - | - |
| 2298 | ENSP00000370376 | DUT | 8.032E-04 | 0.759 | - | - |
| 2299 | ENSP00000341584 | SERPINB13 | 5.850E-05 | 0.758 | - | - |
| 2300 | ENSP00000382707 | IFITM3 | 9.842E-05 | 0.757 | - | - |
| 2301 | ENSP00000253727 | NR1H2 | 3.833E-05 | 0.757 | - | - |
| 2302 | ENSP00000457682 | RP11-458D21.5 | 2.339E-05 | 0.757 | - | - |
| 2303 | ENSP00000422554 | ADAMTS12 | 6.973E-05 | 0.756 | - | - |
| 2304 | ENSP00000414287 | MST1 | 9.094E-05 | 0.755 | - | - |
| 2305 | ENSP00000361423 | ABL1 | 3.445E-04 | 0.755 | - | - |
| 2306 | ENSP00000244314 | IRGC | 8.150E-05 | 0.754 | - | - |
| 2307 | ENSP00000265132 | AMBP | 4.819E-05 | 0.753 | - | - |
| 2308 | ENSP00000361009 | PREX1 | 9.787E-05 | 0.748 | - | - |
| 2309 | ENSP00000320396 | AL033381.1 | 6.209E-04 | 0.748 | - | - |
| 2310 | ENSP00000398971 | IFRD2 | 1.361E-04 | 0.746 | - | - |
| 2311 | ENSP00000329466 | SIRT7 | 8.981E-04 | 0.745 | - | - |
| 2312 | ENSP00000296318 | IL17RD | 8.077E-05 | 0.743 | - | - |
| 2313 | ENSP00000242462 | NEUROG3 | 4.233E-05 | 0.742 | - | - |
| 2314 | ENSP00000333363 | DNAH8 | 2.367E-04 | 0.742 | - | - |
| 2315 | ENSP00000348786 | RAP1A | 3.415E-04 | 0.741 | - | - |
| 2316 | ENSP00000338812 | C1QTNF6 | 1.305E-04 | 0.740 | - | - |
| 2317 | ENSP00000420686 | PLCXD2 | 8.433E-05 | 0.739 | - | - |
| 2318 | ENSP00000430024 | PCDHGA7 | 4.603E-05 | 0.738 | - | - |
| 2319 | ENSP00000359563 | CDR1 | 1.038E-04 | 0.737 | - | - |
| 2320 | ENSP00000348068 | SERPINA1 | 1.056E-04 | 0.737 | - | - |
| 2321 | ENSP00000376372 | PLD4 | 8.577E-05 | 0.735 | - | - |
| 2322 | ENSP00000355082 | PCDH18 | 5.401E-05 | 0.735 | - | - |
| 2323 | ENSP00000419512 | - | 1.622E-04 | 0.735 | - | - |
| 2324 | ENSP00000348463 | TMEM236 | 1.337E-04 | 0.732 | - | - |
| 2325 | ENSP00000355719 | PRSS38 | 8.156E-05 | 0.732 | - | - |
| 2326 | ENSP00000258829 | NKX2-8 | 5.739E-05 | 0.732 | - | - |
| 2327 | ENSP00000322609 | C1orf173 | 1.045E-04 | 0.732 | - | - |
| 2328 | ENSP00000397026 | GRIK2 | 1.015E-03 | 0.731 | - | - |
| 2329 | ENSP00000357768 | LCE2A | 9.538E-05 | 0.730 | - | - |
| 2330 | ENSP00000363317 | FRMPD2 | 5.908E-05 | 0.726 | - | - |
| 2331 | ENSP00000349805 | FAM212B | 7.764E-05 | 0.726 | - | - |
| 2332 | ENSP00000312150 | TRIB1 | 4.989E-04 | 0.725 | - | - |
| 2333 | ENSP00000344456 | CTNNB1 | 3.119E-04 | 0.724 | - | - |
| 2334 | ENSP00000309503 | YWHAZ | 2.762E-04 | 0.721 | - | - |
| 2335 | ENSP00000446280 | PCSK5 | 1.462E-04 | 0.721 | - | - |
| 2336 | ENSP00000342343 | MED22 | 5.566E-05 | 0.721 | - | - |
| 2337 | ENSP00000386069 | ADRA2C | 7.070E-05 | 0.718 | - | - |
| 2338 | ENSP00000303206 | REG1B | 8.207E-05 | 0.717 | - | - |
| 2339 | ENSP00000355865 | PARK2 | 3.910E-04 | 0.716 | - | - |
| 2340 | ENSP00000359096 | AMY1C | 4.198E-05 | 0.716 | - | - |
| 2341 | ENSP00000312672 | TMEM136 | 1.976E-04 | 0.715 | - | - |
| 2342 | ENSP00000414670 | LMOD3 | 4.152E-05 | 0.714 | - | - |
| 2343 | ENSP00000238508 | SERPINB10 | 1.037E-04 | 0.713 | - | - |
| 2344 | ENSP00000347916 | CLEC12A | 5.834E-05 | 0.713 | - | - |
| 2345 | ENSP00000287275 | GLYATL2 | 1.125E-04 | 0.713 | - | - |
| 2346 | ENSP00000382673 | - | 4.910E-05 | 0.710 | - | - |
| 2347 | ENSP00000405514 | PPP1R9A | 9.799E-05 | 0.709 | - | - |
| 2348 | ENSP00000384957 | F11 | 5.178E-05 | 0.708 | - | - |
| 2349 | ENSP00000298910 | LRRK2 | 2.504E-04 | 0.706 | - | - |
| 2350 | ENSP00000264824 | LYL1 | 8.984E-05 | 0.703 | - | - |
| 2351 | ENSP00000292596 | LTC4S | 1.308E-04 | 0.703 | - | - |
| 2352 | ENSP00000416935 | FOLR4 | 4.479E-05 | 0.701 | - | - |
| 2353 | ENSP00000316491 | FCAMR | 6.071E-05 | 0.700 | - | - |
| 2354 | ENSP00000386043 | LTBP1 | 1.038E-04 | 0.698 | - | - |
| 2355 | ENSP00000370844 | GPR12 | 6.173E-05 | 0.696 | - | - |
| 2356 | ENSP00000215939 | CRYBB1 | 7.346E-05 | 0.696 | - | - |
| 2357 | ENSP00000273371 | PLA1A | 5.673E-05 | 0.696 | - | - |
| 2358 | ENSP00000376421 | SDK2 | 7.706E-05 | 0.694 | - | - |
| 2359 | ENSP00000363031 | THEMIS2 | 6.026E-05 | 0.693 | - | - |
| 2360 | ENSP00000358918 | SUFU | 1.459E-04 | 0.689 | - | - |
| 2361 | ENSP00000421725 | GC | 6.819E-05 | 0.685 | - | - |
| 2362 | ENSP00000398890 | HLA-DMB | 1.340E-04 | 0.682 | - | - |
| 2363 | ENSP00000452126 | RGMA | 5.082E-05 | 0.680 | - | - |
| 2364 | ENSP00000356936 | FCRLA | 6.258E-05 | 0.679 | - | - |
| 2365 | ENSP00000376865 | PDGFD | 9.975E-05 | 0.677 | - | - |
| 2366 | ENSP00000344976 | IL1RAPL2 | 7.184E-05 | 0.676 | - | - |
| 2367 | ENSP00000267291 | RNF113B | 8.074E-05 | 0.676 | - | - |
| 2368 | ENSP00000161006 | PRSS22 | 6.418E-05 | 0.675 | - | - |
| 2369 | ENSP00000350348 | GRM7 | 6.283E-05 | 0.675 | - | - |
| 2370 | ENSP00000255380 | CHRM3 | 3.052E-05 | 0.672 | - | - |
| 2371 | ENSP00000359384 | OOEP | 9.918E-05 | 0.670 | - | - |
| 2372 | ENSP00000415517 | C6orf10 | 9.300E-05 | 0.670 | - | - |
| 2373 | ENSP00000347601 | C11orf92 | 1.584E-04 | 0.670 | - | - |
| 2374 | ENSP00000258443 | EDAR | 1.072E-04 | 0.669 | - | - |
| 2375 | ENSP00000360210 | C20orf85 | 1.092E-04 | 0.669 | - | - |
| 2376 | ENSP00000329403 | PCP4 | 8.676E-05 | 0.666 | - | - |
| 2377 | ENSP00000375829 | LAD1 | 7.096E-05 | 0.666 | - | - |
| 2378 | ENSP00000364257 | PLA2G2E | 6.324E-05 | 0.666 | - | - |
| 2379 | ENSP00000349960 | ACTB | 2.115E-04 | 0.665 | - | - |
| 2380 | ENSP00000294954 | LHCGR | 4.126E-05 | 0.661 | - | - |
| 2381 | ENSP00000343445 | SERPINB4 | 5.100E-05 | 0.661 | - | - |
| 2382 | ENSP00000216540 | SLC10A1 | 4.588E-05 | 0.660 | - | - |
| 2383 | ENSP00000391137 | ASB10 | 6.392E-05 | 0.660 | - | - |
| 2384 | ENSP00000262648 | KAL1 | 4.397E-05 | 0.660 | - | - |
| 2385 | ENSP00000312673 | GH1 | 6.473E-05 | 0.659 | - | - |
| 2386 | ENSP00000364133 | TGFBR1 | 1.405E-03 | 0.659 | - | - |
| 2387 | ENSP00000387261 | PSTPIP2 | 8.807E-05 | 0.658 | - | - |
| 2388 | ENSP00000282753 | GRM1 | 2.549E-05 | 0.657 | - | - |
| 2389 | ENSP00000372449 | RP11-408E5.4 | 6.286E-05 | 0.657 | - | - |
| 2390 | ENSP00000338343 | SGCD | 5.935E-05 | 0.655 | - | - |
| 2391 | ENSP00000355675 | GJC2 | 7.118E-05 | 0.655 | - | - |
| 2392 | ENSP00000366233 | GABBR1 | 4.597E-05 | 0.655 | - | - |
| 2393 | ENSP00000348888 | PIGR | 6.117E-05 | 0.655 | - | - |
| 2394 | ENSP00000393840 | BLOC1S3 | 4.784E-05 | 0.655 | - | - |
| 2395 | ENSP00000368438 | PCNA | 3.061E-04 | 0.652 | - | - |
| 2396 | ENSP00000283916 | TMPRSS11D | 4.789E-05 | 0.650 | - | - |
| 2397 | ENSP00000471914 | PIK3R2 | 6.666E-05 | 0.649 | - | - |
| 2398 | ENSP00000343577 | TPSAB1 | 4.841E-05 | 0.648 | - | - |
| 2399 | ENSP00000411827 | GPR137 | 1.238E-04 | 0.647 | - | - |
| 2400 | ENSP00000357981 | CTSS | 1.407E-04 | 0.646 | - | - |
| 2401 | ENSP00000310631 | MRGPRD | 7.459E-05 | 0.645 | - | - |
| 2402 | ENSP00000321077 | SIGLEC8 | 6.658E-05 | 0.645 | - | - |
| 2403 | ENSP00000357773 | LCE2D | 5.826E-05 | 0.644 | - | - |
| 2404 | ENSP00000379701 | HNF4G | 6.813E-05 | 0.643 | - | - |
| 2405 | ENSP00000362174 | - | 9.377E-05 | 0.640 | - | - |
| 2406 | ENSP00000364777 | MAGED4 | 9.003E-05 | 0.640 | - | - |
| 2407 | ENSP00000349547 | RASSF1 | 1.126E-04 | 0.639 | - | - |
| 2408 | ENSP00000242994 | NEUROD4 | 5.647E-05 | 0.638 | - | - |
| 2409 | ENSP00000307552 | NINJ2 | 7.491E-05 | 0.638 | - | - |
| 2410 | ENSP00000252891 | NUMBL | 4.286E-05 | 0.636 | - | - |
| 2411 | ENSP00000298841 | SERPINA4 | 4.257E-05 | 0.636 | - | - |
| 2412 | ENSP00000282406 | PLEKHH2 | 6.416E-05 | 0.636 | - | - |
| 2413 | ENSP00000319377 | NLRP12 | 4.023E-05 | 0.635 | - | - |
| 2414 | ENSP00000296125 | TGM4 | 6.085E-05 | 0.634 | - | - |
| 2415 | ENSP00000425809 | PGM3 | 1.127E-04 | 0.633 | - | - |
| 2416 | ENSP00000380053 | LEUTX | 3.824E-05 | 0.630 | - | - |
| 2417 | ENSP00000200457 | TRIP6 | 2.023E-03 | 0.629 | - | - |
| 2418 | ENSP00000367714 | HES5 | 6.987E-05 | 0.628 | - | - |
| 2419 | ENSP00000377409 | SFTPB | 5.390E-05 | 0.628 | - | - |
| 2420 | ENSP00000288167 | IL17RB | 6.711E-05 | 0.627 | - | - |
| 2421 | ENSP00000364956 | PLEKHM2 | 4.256E-05 | 0.626 | - | - |
| 2422 | ENSP00000335578 | ARGFX | 4.616E-05 | 0.626 | - | - |
| 2423 | ENSP00000309767 | NLRP6 | 5.038E-05 | 0.625 | - | - |
| 2424 | ENSP00000407546 | CHRNA7 | 1.037E-04 | 0.625 | - | - |
| 2425 | ENSP00000259254 | GYPC | 6.201E-05 | 0.624 | - | - |
| 2426 | ENSP00000462963 | TEFM | 1.332E-04 | 0.624 | - | - |
| 2427 | ENSP00000283256 | SCN2A | 3.319E-05 | 0.621 | - | - |
| 2428 | ENSP00000258400 | HTR2B | 3.608E-05 | 0.620 | - | - |
| 2429 | ENSP00000365858 | GATA1 | 9.769E-05 | 0.617 | - | - |
| 2430 | ENSP00000372221 | SERPINB5 | 1.169E-04 | 0.616 | - | - |
| 2431 | ENSP00000357066 | ARG1 | 1.056E-04 | 0.616 | - | - |
| 2432 | ENSP00000257267 | C5AR2 | 7.875E-05 | 0.616 | - | - |
| 2433 | ENSP00000325561 | TMC8 | 6.053E-05 | 0.615 | - | - |
| 2434 | ENSP00000309845 | HRAS | 1.749E-04 | 0.614 | - | - |
| 2435 | ENSP00000349768 | DCHS2 | 6.835E-05 | 0.613 | - | - |
| 2436 | ENSP00000198536 | PILRA | 6.310E-05 | 0.611 | - | - |
| 2437 | ENSP00000388666 | ICAM2 | 8.515E-05 | 0.611 | - | - |
| 2438 | ENSP00000295897 | ALB | 1.610E-04 | 0.611 | - | - |
| 2439 | ENSP00000339221 | SULT1A4 | 5.428E-05 | 0.610 | - | - |
| 2440 | ENSP00000301407 | CGB1 | 9.597E-05 | 0.609 | - | - |
| 2441 | ENSP00000472467 | CXCL17 | 6.031E-05 | 0.608 | - | - |
| 2442 | ENSP00000437781 | - | 2.908E-06 | 0.608 | - | - |
| 2443 | ENSP00000325402 | MYPOP | 1.601E-05 | 0.608 | - | - |
| 2444 | ENSP00000350447 | SGOL2 | 1.195E-04 | 0.608 | - | - |
| 2445 | ENSP00000324173 | HSPA5 | 2.940E-04 | 0.608 | - | - |
| 2446 | ENSP00000222792 | CHN2 | 3.205E-05 | 0.606 | - | - |
| 2447 | ENSP00000354478 | DLX1 | 4.648E-05 | 0.606 | - | - |
| 2448 | ENSP00000342935 | SDCBP2 | 6.697E-05 | 0.605 | - | - |
| 2449 | ENSP00000296847 | C6orf195 | 3.216E-04 | 0.605 | - | - |
| 2450 | ENSP00000222032 | CNFN | 7.743E-05 | 0.603 | - | - |
| 2451 | ENSP00000284674 | GPR26 | 7.322E-05 | 0.602 | - | - |
| 2452 | ENSP00000354769 | C1orf68 | 1.104E-04 | 0.601 | - | - |
| 2453 | ENSP00000456711 | ITGB3 | 6.294E-05 | 0.599 | - | - |
| 2454 | ENSP00000354910 | NRG2 | 6.299E-05 | 0.599 | - | - |
| 2455 | ENSP00000356251 | GPR37L1 | 5.595E-05 | 0.598 | - | - |
| 2456 | ENSP00000303276 | RNASE2 | 3.154E-05 | 0.597 | - | - |
| 2457 | ENSP00000339377 | NPY5R | 7.787E-05 | 0.597 | - | - |
| 2458 | ENSP00000347409 | KEL | 5.005E-05 | 0.596 | - | - |
| 2459 | ENSP00000357779 | CRCT1 | 7.334E-05 | 0.596 | - | - |
| 2460 | ENSP00000328069 | C6orf58 | 8.926E-05 | 0.595 | - | - |
| 2461 | ENSP00000364016 | PSMB8 | 8.843E-05 | 0.594 | - | - |
| 2462 | ENSP00000336832 | GJD3 | 6.655E-05 | 0.593 | - | - |
| 2463 | ENSP00000258526 | PLXNC1 | 7.076E-05 | 0.592 | - | - |
| 2464 | ENSP00000420194 | CASR | 7.885E-05 | 0.592 | - | - |
| 2465 | ENSP00000328973 | TSPO | 2.005E-04 | 0.592 | - | - |
| 2466 | ENSP00000252519 | ACE2 | 5.418E-05 | 0.592 | - | - |
| 2467 | ENSP00000340361 | ACVR2B | 6.221E-05 | 0.591 | - | - |
| 2468 | ENSP00000348461 | RAC1 | 2.535E-04 | 0.591 | - | - |
| 2469 | ENSP00000302251 | GBX2 | 3.754E-05 | 0.590 | - | - |
| 2470 | ENSP00000164024 | CELSR3 | 4.189E-05 | 0.590 | - | - |
| 2471 | ENSP00000355208 | TPTE | 6.279E-04 | 0.589 | - | - |
| 2472 | ENSP00000054668 | UTS2 | 3.508E-05 | 0.588 | - | - |
| 2473 | ENSP00000393275 | NANOS1 | 7.476E-05 | 0.587 | - | - |
| 2474 | ENSP00000386884 | CXCR4 | 1.144E-04 | 0.583 | - | - |
| 2475 | ENSP00000376445 | TIRAP | 1.737E-04 | 0.582 | - | - |
| 2476 | ENSP00000346805 | CRYBA4 | 5.109E-05 | 0.576 | - | - |
| 2477 | ENSP00000363330 | CD52 | 8.753E-05 | 0.575 | - | - |
| 2478 | ENSP00000317980 | IGSF9B | 5.360E-05 | 0.572 | - | - |
| 2479 | ENSP00000222543 | TFPI2 | 6.460E-05 | 0.572 | - | - |
| 2480 | ENSP00000304327 | COPRS | 1.957E-04 | 0.571 | - | - |
| 2481 | ENSP00000353331 | KIRREL2 | 5.080E-05 | 0.571 | - | - |
| 2482 | ENSP00000357711 | S100A7 | 8.689E-05 | 0.570 | - | - |
| 2483 | ENSP00000434466 | INTS4 | 8.921E-05 | 0.569 | - | - |
| 2484 | ENSP00000353203 | LCE1B | 6.919E-05 | 0.568 | - | - |
| 2485 | ENSP00000259365 | TMOD1 | 6.602E-05 | 0.568 | - | - |
| 2486 | ENSP00000259989 | FGFBP2 | 6.100E-05 | 0.568 | - | - |
| 2487 | ENSP00000369238 | ADAM32 | 4.315E-05 | 0.567 | - | - |
| 2488 | ENSP00000347767 | NEK5 | 7.208E-05 | 0.566 | - | - |
| 2489 | ENSP00000377502 | PHLDB2 | 1.047E-04 | 0.565 | - | - |
| 2490 | ENSP00000251642 | DHX58 | 6.761E-05 | 0.565 | - | - |
| 2491 | ENSP00000369274 | PHKA2 | 5.484E-05 | 0.564 | - | - |
| 2492 | ENSP00000281047 | MSGN1 | 5.051E-05 | 0.563 | - | - |
| 2493 | ENSP00000369344 | GPR150 | 9.114E-05 | 0.562 | - | - |
| 2494 | ENSP00000242152 | NPY | 4.708E-05 | 0.562 | - | - |
| 2495 | ENSP00000239165 | HOXB7 | 3.480E-05 | 0.560 | - | - |
| 2496 | ENSP00000345716 | NKAPL | 8.602E-05 | 0.559 | - | - |
| 2497 | ENSP00000387281 | ADRA2B | 7.318E-05 | 0.558 | - | - |
| 2498 | ENSP00000376786 | POU2AF1 | 6.108E-05 | 0.556 | - | - |
| 2499 | ENSP00000343366 | VSTM1 | 6.877E-06 | 0.553 | - | - |
| 2500 | ENSP00000377218 | IRF2 | 1.500E-04 | 0.551 | - | - |
| 2501 | ENSP00000378367 | NRG4 | 6.548E-05 | 0.551 | - | - |
| 2502 | ENSP00000303192 | RGL1 | 5.607E-05 | 0.551 | - | - |
| 2503 | ENSP00000345008 | FBLN5 | 8.442E-05 | 0.550 | - | - |
| 2504 | ENSP00000415183 | MUC2 | 7.188E-05 | 0.549 | - | - |
| 2505 | ENSP00000333592 | C21orf90 | 8.534E-05 | 0.549 | - | - |
| 2506 | ENSP00000275857 | NLGN4X | 1.008E-04 | 0.548 | - | - |
| 2507 | ENSP00000369442 | KL | 4.316E-05 | 0.546 | - | - |
| 2508 | ENSP00000364709 | F10 | 8.390E-05 | 0.545 | - | - |
| 2509 | ENSP00000384863 | XPO1 | 4.414E-04 | 0.545 | - | - |
| 2510 | ENSP00000272972 | ANKMY1 | 1.183E-04 | 0.544 | - | - |
| 2511 | ENSP00000322061 | C7 | 6.780E-05 | 0.544 | - | - |
| 2512 | ENSP00000237527 | GHRH | 2.885E-05 | 0.544 | - | - |
| 2513 | ENSP00000330484 | AMY1B | 4.064E-05 | 0.544 | - | - |
| 2514 | ENSP00000320318 | NEU4 | 8.037E-05 | 0.541 | - | - |
| 2515 | ENSP00000254122 | FSHB | 3.582E-05 | 0.541 | - | - |
| 2516 | ENSP00000227665 | APOA5 | 4.339E-05 | 0.540 | - | - |
| 2517 | ENSP00000357753 | IVL | 1.105E-04 | 0.540 | - | - |
| 2518 | ENSP00000369317 | KRT6A | 8.779E-05 | 0.540 | - | - |
| 2519 | ENSP00000397582 | AMY2A | 5.937E-05 | 0.539 | - | - |
| 2520 | ENSP00000344577 | APOL3 | 7.272E-05 | 0.538 | - | - |
| 2521 | ENSP00000249501 | HOXD10 | 3.976E-05 | 0.536 | - | - |
| 2522 | ENSP00000315383 | ASPRV1 | 6.063E-05 | 0.533 | - | - |
| 2523 | ENSP00000321026 | LURAP1L | 7.106E-05 | 0.532 | - | - |
| 2524 | ENSP00000328359 | DEFA3 | 4.192E-05 | 0.532 | - | - |
| 2525 | ENSP00000341442 | SNTN | 4.887E-05 | 0.532 | - | - |
| 2526 | ENSP00000324741 | C7orf43 | 9.068E-05 | 0.531 | - | - |
| 2527 | ENSP00000378577 | CLDN3 | 7.307E-05 | 0.531 | - | - |
| 2528 | ENSP00000362649 | HDAC1 | 2.979E-04 | 0.531 | - | - |
| 2529 | ENSP00000355988 | IRF6 | 7.607E-05 | 0.530 | - | - |
| 2530 | ENSP00000255082 | ACY3 | 5.198E-05 | 0.529 | - | - |
| 2531 | ENSP00000261883 | CILP | 5.255E-05 | 0.529 | - | - |
| 2532 | ENSP00000289753 | HTR6 | 4.059E-05 | 0.529 | - | - |
| 2533 | ENSP00000243911 | MC3R | 3.895E-05 | 0.529 | - | - |
| 2534 | ENSP00000348307 | SIRPA | 9.345E-05 | 0.527 | - | - |
| 2535 | ENSP00000362205 | TREM2 | 9.116E-05 | 0.526 | - | - |
| 2536 | ENSP00000378699 | CDK1 | 2.353E-04 | 0.526 | - | - |
| 2537 | ENSP00000411096 | ABCA13 | 4.471E-05 | 0.525 | - | - |
| 2538 | ENSP00000436714 | HCAR3 | 6.961E-05 | 0.525 | - | - |
| 2539 | ENSP00000443539 | - | 5.687E-05 | 0.525 | - | - |
| 2540 | ENSP00000377396 | FAM198B | 7.326E-05 | 0.525 | - | - |
| 2541 | ENSP00000429374 | RPS20 | 8.899E-04 | 0.524 | - | - |
| 2542 | ENSP00000253392 | EDA2R | 1.752E-04 | 0.524 | - | - |
| 2543 | ENSP00000403293 | FBXO43 | 6.302E-05 | 0.523 | - | - |
| 2544 | ENSP00000354294 | CTRB1 | 4.658E-05 | 0.523 | - | - |
| 2545 | ENSP00000393333 | TBXA2R | 4.347E-05 | 0.522 | - | - |
| 2546 | ENSP00000219022 | OLFM4 | 4.955E-05 | 0.521 | - | - |
| 2547 | ENSP00000377075 | RGL3 | 7.925E-05 | 0.519 | - | - |
| 2548 | ENSP00000005226 | USH1C | 3.592E-05 | 0.519 | - | - |
| 2549 | ENSP00000349274 | PPP1R26 | 4.514E-05 | 0.518 | - | - |
| 2550 | ENSP00000328777 | EFNA5 | 5.552E-05 | 0.516 | - | - |
| 2551 | ENSP00000309794 | TBC1D16 | 4.418E-05 | 0.515 | - | - |
| 2552 | ENSP00000328307 | FAM47B | 1.367E-04 | 0.515 | - | - |
| 2553 | ENSP00000362524 | ANGPTL2 | 6.032E-05 | 0.514 | - | - |
| 2554 | ENSP00000281923 | MGAT5 | 4.577E-05 | 0.511 | - | - |
| 2555 | ENSP00000416037 | TMPRSS4 | 5.729E-05 | 0.509 | - | - |
| 2556 | ENSP00000399588 | GAB3 | 3.216E-05 | 0.509 | - | - |
| 2557 | ENSP00000354335 | MPEG1 | 5.505E-05 | 0.508 | - | - |
| 2558 | ENSP00000363350 | PTPN20B | 2.683E-05 | 0.507 | - | - |
| 2559 | ENSP00000339168 | COLEC11 | 7.194E-05 | 0.507 | - | - |
| 2560 | ENSP00000249504 | HOXD11 | 3.608E-05 | 0.505 | - | - |
| 2561 | ENSP00000451700 | DUX4L7 | 4.341E-05 | 0.504 | - | - |
| 2562 | ENSP00000451798 | DUX4L3 | 4.341E-05 | 0.504 | - | - |
| 2563 | ENSP00000268097 | HEXA | 3.726E-05 | 0.504 | - | - |
| 2564 | ENSP00000249116 | APOBEC3A | 8.582E-05 | 0.502 | - | - |
| 2565 | ENSP00000354932 | TLR1 | 1.400E-04 | 0.500 | - | - |
| 2566 | ENSP00000451253 | DUX4L6 | 4.356E-05 | 0.500 | - | - |
| 2567 | ENSP00000323587 | ITLN1 | 5.712E-05 | 0.499 | - | - |
| 2568 | ENSP00000384434 | ZGLP1 | 4.398E-05 | 0.498 | - | - |
| 2569 | ENSP00000316938 | DNASE1L2 | 8.811E-05 | 0.497 | - | - |
| 2570 | ENSP00000362743 | C2orf72 | 6.719E-05 | 0.497 | - | - |
| 2571 | ENSP00000286479 | NAT2 | 4.481E-05 | 0.496 | - | - |
| 2572 | ENSP00000438833 | - | 1.024E-04 | 0.494 | - | - |
| 2573 | ENSP00000312273 | LGI4 | 1.147E-04 | 0.494 | - | - |
| 2574 | ENSP00000362613 | CDX4 | 4.605E-05 | 0.493 | - | - |
| 2575 | ENSP00000369071 | POSTN | 6.394E-05 | 0.493 | - | - |
| 2576 | ENSP00000262735 | PPARA | 4.742E-04 | 0.489 | - | - |
| 2577 | ENSP00000321005 | CD300LG | 5.338E-05 | 0.488 | - | - |
| 2578 | ENSP00000217386 | OXT | 3.678E-05 | 0.488 | - | - |
| 2579 | ENSP00000299847 | CHRFAM7A | 1.137E-04 | 0.488 | - | - |
| 2580 | ENSP00000348472 | PXK | 1.659E-04 | 0.487 | - | - |
| 2581 | ENSP00000218721 | MLNR | 4.168E-05 | 0.487 | - | - |
| 2582 | ENSP00000297161 | BMPER | 5.106E-05 | 0.486 | - | - |
| 2583 | ENSP00000309913 | TBX5 | 3.769E-05 | 0.486 | - | - |
| 2584 | ENSP00000472225 | PRED58 | 1.944E-05 | 0.486 | - | - |
| 2585 | ENSP00000355991 | TRAF3IP3 | 8.660E-05 | 0.486 | - | - |
| 2586 | ENSP00000382318 | C14orf23 | 2.814E-06 | 0.485 | - | - |
| 2587 | ENSP00000258302 | RGS8 | 4.567E-05 | 0.483 | - | - |
| 2588 | ENSP00000302150 | PRL | 7.771E-05 | 0.483 | - | - |
| 2589 | ENSP00000356000 | PLXNA2 | 3.935E-05 | 0.483 | - | - |
| 2590 | ENSP00000293829 | FGF11 | 7.412E-05 | 0.483 | - | - |
| 2591 | ENSP00000295878 | KIAA1407 | 1.103E-04 | 0.482 | - | - |
| 2592 | ENSP00000390478 | TREX1 | 8.174E-05 | 0.482 | - | - |
| 2593 | ENSP00000369960 | ADRA1A | 3.845E-05 | 0.482 | - | - |
| 2594 | ENSP00000297788 | CCDC136 | 8.251E-05 | 0.481 | - | - |
| 2595 | ENSP00000393847 | PLA2G10 | 4.923E-05 | 0.481 | - | - |
| 2596 | ENSP00000303540 | SCN1A | 3.918E-05 | 0.480 | - | - |
| 2597 | ENSP00000375259 | DKFZP667F0711 | 4.256E-05 | 0.479 | - | - |
| 2598 | ENSP00000223061 | PCOLCE | 7.588E-05 | 0.478 | - | - |
| 2599 | ENSP00000359446 | CPN1 | 5.785E-05 | 0.476 | - | - |
| 2600 | ENSP00000389585 | DUX4L2 | 4.369E-05 | 0.476 | - | - |
| 2601 | ENSP00000220616 | TG | 7.194E-05 | 0.476 | - | - |
| 2602 | ENSP00000287820 | PPARG | 4.323E-04 | 0.475 | - | - |
| 2603 | ENSP00000335153 | HSP90AA1 | 2.179E-04 | 0.474 | - | - |
| 2604 | ENSP00000342538 | STMN4 | 7.401E-05 | 0.473 | - | - |
| 2605 | ENSP00000417498 | MUC4 | 6.111E-05 | 0.473 | - | - |
| 2606 | ENSP00000357757 | LCE1C | 9.138E-05 | 0.472 | - | - |
| 2607 | ENSP00000370293 | HUS1B | 6.122E-05 | 0.471 | - | - |
| 2608 | ENSP00000263370 | ITPKC | 6.764E-05 | 0.471 | - | - |
| 2609 | ENSP00000292779 | CCDC116 | 1.869E-04 | 0.469 | - | - |
| 2610 | ENSP00000458149 | CAMP | 2.113E-04 | 0.467 | - | - |
| 2611 | ENSP00000306241 | - | 1.601E-04 | 0.467 | - | - |
| 2612 | ENSP00000358548 | NRAS | 2.674E-04 | 0.466 | - | - |
| 2613 | ENSP00000377934 | KIF7 | 9.947E-05 | 0.464 | - | - |
| 2614 | ENSP00000347322 | C6orf89 | 1.855E-04 | 0.462 | - | - |
| 2615 | ENSP00000305107 | GIMAP8 | 6.779E-05 | 0.461 | - | - |
| 2616 | ENSP00000315295 | KIAA1549L | 1.078E-04 | 0.460 | - | - |
| 2617 | ENSP00000302476 | HSPB2 | 6.018E-05 | 0.458 | - | - |
| 2618 | ENSP00000291670 | FTCD | 4.688E-05 | 0.457 | - | - |
| 2619 | ENSP00000396688 | C4A | 1.347E-04 | 0.456 | - | - |
| 2620 | ENSP00000269394 | ZNF750 | 8.156E-05 | 0.456 | - | - |
| 2621 | ENSP00000268459 | NKD1 | 5.113E-05 | 0.455 | - | - |
| 2622 | ENSP00000381298 | LONRF1 | 7.716E-05 | 0.454 | - | - |
| 2623 | ENSP00000283285 | CD96 | 1.078E-04 | 0.453 | - | - |
| 2624 | ENSP00000452398 | DUXA | 3.622E-05 | 0.452 | - | - |
| 2625 | ENSP00000338072 | AVPR2 | 3.814E-05 | 0.452 | - | - |
| 2626 | ENSP00000167106 | VASH1 | 7.148E-05 | 0.451 | - | - |
| 2627 | ENSP00000345344 | CTSL1 | 8.257E-05 | 0.450 | - | - |
| 2628 | ENSP00000411948 | SAPCD1 | 5.467E-05 | 0.448 | - | - |
| 2629 | ENSP00000296506 | SCRG1 | 1.002E-04 | 0.448 | - | - |
| 2630 | ENSP00000261994 | SERPINA10 | 4.733E-05 | 0.447 | - | - |
| 2631 | ENSP00000255476 | RFXAP | 5.227E-05 | 0.447 | - | - |
| 2632 | ENSP00000364978 | LY6G6C | 4.738E-05 | 0.445 | - | - |
| 2633 | ENSP00000299709 | SLC38A8 | 4.548E-05 | 0.445 | - | - |
| 2634 | ENSP00000305824 | KCNS3 | 4.072E-05 | 0.445 | - | - |
| 2635 | ENSP00000426741 | EFNA3 | 9.679E-05 | 0.444 | - | - |
| 2636 | ENSP00000264741 | ITGA9 | 4.066E-05 | 0.443 | - | - |
| 2637 | ENSP00000443399 | - | 5.264E-06 | 0.442 | - | - |
| 2638 | ENSP00000421799 | RP1-170O19.20 | 1.011E-04 | 0.441 | - | - |
| 2639 | ENSP00000360973 | AGTR2 | 4.530E-05 | 0.441 | - | - |
| 2640 | ENSP00000416341 | NELL2 | 5.986E-05 | 0.440 | - | - |
| 2641 | ENSP00000294671 | GBP7 | 1.187E-04 | 0.440 | - | - |
| 2642 | ENSP00000261374 | HS3ST2 | 4.558E-05 | 0.438 | - | - |
| 2643 | ENSP00000346298 | SLC6A20 | 4.459E-05 | 0.438 | - | - |
| 2644 | ENSP00000228841 | MYL2 | 2.273E-05 | 0.437 | - | - |
| 2645 | ENSP00000356429 | RGS1 | 1.124E-04 | 0.435 | - | - |
| 2646 | ENSP00000337128 | EDN3 | 3.792E-05 | 0.435 | - | - |
| 2647 | ENSP00000358763 | CHI3L2 | 1.291E-04 | 0.434 | - | - |
| 2648 | ENSP00000333685 | MAPK11 | 1.072E-04 | 0.433 | - | - |
| 2649 | ENSP00000350467 | NAT9 | 5.569E-05 | 0.430 | - | - |
| 2650 | ENSP00000346577 | AK5 | 4.444E-05 | 0.427 | - | - |
| 2651 | ENSP00000298251 | HEPACAM | 5.083E-05 | 0.424 | - | - |
| 2652 | ENSP00000253008 | PRDM12 | 7.738E-05 | 0.424 | - | - |
| 2653 | ENSP00000414687 | C11orf82 | 2.603E-05 | 0.424 | - | - |
| 2654 | ENSP00000314520 | KCNA2 | 3.512E-05 | 0.423 | - | - |
| 2655 | ENSP00000290294 | PRAC | 5.143E-05 | 0.423 | - | - |
| 2656 | ENSP00000387122 | CLEC16A | 2.469E-04 | 0.423 | - | - |
| 2657 | ENSP00000267015 | GPR84 | 5.968E-05 | 0.423 | - | - |
| 2658 | ENSP00000257248 | GIF | 3.706E-05 | 0.422 | - | - |
| 2659 | ENSP00000356189 | IPCEF1 | 6.341E-05 | 0.422 | - | - |
| 2660 | ENSP00000401678 | MYOCD | 8.311E-05 | 0.420 | - | - |
| 2661 | ENSP00000318270 | PROM2 | 4.800E-05 | 0.419 | - | - |
| 2662 | ENSP00000266077 | SLC2A4RG | 7.351E-05 | 0.419 | - | - |
| 2663 | ENSP00000365048 | TNFSF13B | 1.966E-04 | 0.417 | - | - |
| 2664 | ENSP00000269202 | MEP1B | 5.512E-05 | 0.416 | - | - |
| 2665 | ENSP00000336571 | CLDN2 | 4.467E-05 | 0.415 | - | - |
| 2666 | ENSP00000306490 | CHRM1 | 4.280E-05 | 0.415 | - | - |
| 2667 | ENSP00000319322 | OR4K13 | 2.966E-05 | 0.415 | - | - |
| 2668 | ENSP00000290894 | SHF | 5.695E-05 | 0.414 | - | - |
| 2669 | ENSP00000269582 | PNMT | 5.375E-05 | 0.414 | - | - |
| 2670 | ENSP00000335560 | ARHGEF25 | 3.615E-05 | 0.413 | - | - |
| 2671 | ENSP00000253799 | AOC2 | 3.117E-05 | 0.411 | - | - |
| 2672 | ENSP00000434279 | TMPRSS13 | 3.810E-05 | 0.410 | - | - |
| 2673 | ENSP00000302194 | ATP6V1G2 | 4.041E-05 | 0.410 | - | - |
| 2674 | ENSP00000453302 | ADAM8 | 4.587E-05 | 0.408 | - | - |
| 2675 | ENSP00000267853 | MYZAP | 6.027E-05 | 0.408 | - | - |
| 2676 | ENSP00000283921 | HOXA10 | 3.582E-05 | 0.407 | - | - |
| 2677 | ENSP00000371835 | DEFB126 | 1.227E-04 | 0.407 | - | - |
| 2678 | ENSP00000311153 | PILRB | 1.113E-04 | 0.405 | - | - |
| 2679 | ENSP00000273590 | TCTA | 4.440E-05 | 0.404 | - | - |
| 2680 | ENSP00000228887 | GPRC5D | 8.494E-05 | 0.404 | - | - |
| 2681 | ENSP00000359449 | TMEM185A | 6.125E-05 | 0.404 | - | - |
| 2682 | ENSP00000379571 | MRGPRX3 | 5.545E-05 | 0.404 | - | - |
| 2683 | ENSP00000417677 | ZBTB8OS | 9.469E-05 | 0.402 | - | - |
| 2684 | ENSP00000299481 | NLRP14 | 7.455E-05 | 0.401 | - | - |
| 2685 | ENSP00000296099 | UCN | 3.858E-05 | 0.401 | - | - |
| 2686 | ENSP00000317818 | PIANP | 9.979E-05 | 0.400 | - | - |
| 2687 | ENSP00000342156 | NCR3 | 9.033E-05 | 0.400 | - | - |
| 2688 | ENSP00000285273 | CA10 | 5.426E-05 | 0.399 | - | - |
| 2689 | ENSP00000369573 | OR51B4 | 3.734E-05 | 0.399 | - | - |
| 2690 | ENSP00000272847 | PTH2R | 3.906E-05 | 0.399 | - | - |
| 2691 | ENSP00000215917 | SRRD | 8.041E-05 | 0.398 | - | - |
| 2692 | ENSP00000334267 | C5orf38 | 3.693E-05 | 0.396 | - | - |
| 2693 | ENSP00000303686 | NTSR2 | 4.662E-05 | 0.395 | - | - |
| 2694 | ENSP00000375232 | KRTAP4-11 | 3.991E-05 | 0.394 | - | - |
| 2695 | ENSP00000381098 | GRIP1 | 7.332E-05 | 0.394 | - | - |
| 2696 | ENSP00000366797 | SLC22A12 | 5.869E-05 | 0.392 | - | - |
| 2697 | ENSP00000326244 | TMEM151A | 5.444E-05 | 0.392 | - | - |
| 2698 | ENSP00000429562 | UNC13A | 7.358E-05 | 0.392 | - | - |
| 2699 | ENSP00000358099 | RGS10 | 6.540E-05 | 0.391 | - | - |
| 2700 | ENSP00000360286 | RAE1 | 9.704E-05 | 0.391 | - | - |
| 2701 | ENSP00000362410 | MAFB | 7.296E-05 | 0.389 | - | - |
| 2702 | ENSP00000363993 | PSMB9 | 1.092E-04 | 0.389 | - | - |
| 2703 | ENSP00000358202 | - | 3.699E-05 | 0.389 | - | - |
| 2704 | ENSP00000221804 | CLC | 7.824E-05 | 0.387 | - | - |
| 2705 | ENSP00000371529 | C22orf42 | 4.516E-05 | 0.387 | - | - |
| 2706 | ENSP00000362299 | ENG | 6.661E-05 | 0.387 | - | - |
| 2707 | ENSP00000246553 | FFAR1 | 3.566E-05 | 0.387 | - | - |
| 2708 | ENSP00000433721 | C11orf54 | 1.220E-04 | 0.386 | - | - |
| 2709 | ENSP00000308405 | H2AFV | 5.664E-04 | 0.386 | - | - |
| 2710 | ENSP00000356906 | SH2D1B | 7.920E-05 | 0.384 | - | - |
| 2711 | ENSP00000266970 | CDK2 | 1.843E-04 | 0.383 | - | - |
| 2712 | ENSP00000266991 | DHH | 6.581E-05 | 0.383 | - | - |
| 2713 | ENSP00000367207 | MYC | 3.313E-04 | 0.382 | - | - |
| 2714 | ENSP00000378191 | AIMP1 | 8.360E-05 | 0.381 | - | - |
| 2715 | ENSP00000364270 | XPA | 1.459E-04 | 0.381 | - | - |
| 2716 | ENSP00000319744 | GPR4 | 4.040E-05 | 0.380 | - | - |
| 2717 | ENSP00000418767 | TREML2 | 4.354E-05 | 0.380 | - | - |
| 2718 | ENSP00000258807 | CIDEB | 1.046E-04 | 0.380 | - | - |
| 2719 | ENSP00000243673 | GPR83 | 4.151E-05 | 0.379 | - | - |
| 2720 | ENSP00000364720 | XAGE1E | 1.941E-06 | 0.377 | - | - |
| 2721 | ENSP00000230732 | POU4F3 | 4.045E-05 | 0.374 | - | - |
| 2722 | ENSP00000006777 | RHBDD2 | 8.102E-05 | 0.374 | - | - |
| 2723 | ENSP00000370508 | MTIF3 | 5.439E-05 | 0.372 | - | - |
| 2724 | ENSP00000356329 | RAET1G | 2.844E-05 | 0.372 | - | - |
| 2725 | ENSP00000345147 | NKX2-4 | 4.595E-05 | 0.372 | - | - |
| 2726 | ENSP00000261918 | SEMA7A | 4.319E-05 | 0.372 | - | - |
| 2727 | ENSP00000379739 | IPP | 6.667E-05 | 0.372 | - | - |
| 2728 | ENSP00000338067 | PKIG | 6.381E-05 | 0.371 | - | - |
| 2729 | ENSP00000314042 | MRGPRX4 | 5.170E-05 | 0.370 | - | - |
| 2730 | ENSP00000296589 | SLC45A2 | 4.132E-05 | 0.369 | - | - |
| 2731 | ENSP00000384742 | GAGE2E | 4.781E-05 | 0.368 | - | - |
| 2732 | ENSP00000311697 | FGF5 | 8.429E-05 | 0.367 | - | - |
| 2733 | ENSP00000356430 | RGS18 | 6.568E-05 | 0.367 | - | - |
| 2734 | ENSP00000330787 | NDUFB1 | 5.744E-05 | 0.367 | - | - |
| 2735 | ENSP00000368304 | MAGEB10 | 7.108E-05 | 0.367 | - | - |
| 2736 | ENSP00000334287 | KRTAP21-2 | 6.896E-05 | 0.367 | - | - |
| 2737 | ENSP00000332170 | P4HA3 | 7.526E-05 | 0.365 | - | - |
| 2738 | ENSP00000365301 | FGF14 | 9.489E-05 | 0.365 | - | - |
| 2739 | ENSP00000239144 | HOXB8 | 3.546E-05 | 0.364 | - | - |
| 2740 | ENSP00000348639 | TMPRSS11F | 4.511E-05 | 0.363 | - | - |
| 2741 | ENSP00000296350 | MFI2 | 5.926E-05 | 0.361 | - | - |
| 2742 | ENSP00000265362 | SEMA3A | 3.199E-05 | 0.361 | - | - |
| 2743 | ENSP00000349727 | DOK3 | 6.518E-05 | 0.360 | - | - |
| 2744 | ENSP00000325301 | CDPF1 | 2.328E-06 | 0.359 | - | - |
| 2745 | ENSP00000370697 | P2RY8 | 5.298E-05 | 0.358 | - | - |
| 2746 | ENSP00000274382 | LIX1 | 6.204E-05 | 0.358 | - | - |
| 2747 | ENSP00000301455 | ANGPTL4 | 4.267E-05 | 0.357 | - | - |
| 2748 | ENSP00000379138 | AFP | 9.489E-05 | 0.356 | - | - |
| 2749 | ENSP00000292377 | GPC2 | 5.992E-05 | 0.356 | - | - |
| 2750 | ENSP00000352414 | FGF17 | 5.345E-05 | 0.356 | - | - |
| 2751 | ENSP00000331062 | VSTM4 | 6.542E-05 | 0.356 | - | - |
| 2752 | ENSP00000361072 | TSPAN1 | 5.260E-05 | 0.354 | - | - |
| 2753 | ENSP00000335191 | TBX10 | 4.877E-05 | 0.353 | - | - |
| 2754 | ENSP00000364855 | MSH5 | 7.452E-05 | 0.353 | - | - |
| 2755 | ENSP00000231749 | ZMYND10 | 8.265E-05 | 0.353 | - | - |
| 2756 | ENSP00000348831 | NUDT10 | 4.953E-05 | 0.352 | - | - |
| 2757 | ENSP00000305973 | HOXC4 | 3.566E-05 | 0.351 | - | - |
| 2758 | ENSP00000290271 | STC1 | 3.460E-05 | 0.351 | - | - |
| 2759 | ENSP00000373169 | DRD3 | 5.481E-05 | 0.351 | - | - |
| 2760 | ENSP00000287042 | KCNS2 | 3.830E-05 | 0.350 | - | - |
| 2761 | ENSP00000353452 | MYLK | 5.863E-05 | 0.348 | - | - |
| 2762 | ENSP00000222674 | NPVF | 5.908E-05 | 0.348 | - | - |
| 2763 | ENSP00000352401 | RPE | 4.618E-05 | 0.348 | - | - |
| 2764 | ENSP00000340089 | TLR5 | 1.369E-04 | 0.348 | - | - |
| 2765 | ENSP00000262018 | SGCA | 3.127E-05 | 0.348 | - | - |
| 2766 | ENSP00000363124 | PHF20 | 8.660E-05 | 0.347 | - | - |
| 2767 | ENSP00000397073 | TRIM10 | 5.601E-05 | 0.346 | - | - |
| 2768 | ENSP00000217305 | PDYN | 6.839E-05 | 0.346 | - | - |
| 2769 | ENSP00000309493 | GPRC6A | 3.936E-05 | 0.346 | - | - |
| 2770 | ENSP00000418191 | CTNNA2 | 8.622E-05 | 0.346 | - | - |
| 2771 | ENSP00000231721 | SEMA3G | 6.093E-05 | 0.343 | - | - |
| 2772 | ENSP00000269299 | ASGR1 | 6.099E-05 | 0.343 | - | - |
| 2773 | ENSP00000273352 | GPR128 | 5.808E-05 | 0.341 | - | - |
| 2774 | ENSP00000386733 | PLEKHG2 | 6.046E-05 | 0.340 | - | - |
| 2775 | ENSP00000300954 | PCSK4 | 3.744E-05 | 0.339 | - | - |
| 2776 | ENSP00000323635 | FAM210A | 6.398E-05 | 0.339 | - | - |
| 2777 | ENSP00000326022 | FHL5 | 4.022E-05 | 0.338 | - | - |
| 2778 | ENSP00000366748 | AUP1 | 8.004E-05 | 0.337 | - | - |
| 2779 | ENSP00000221972 | CD79A | 6.963E-05 | 0.337 | - | - |
| 2780 | ENSP00000245983 | GNRH2 | 3.563E-05 | 0.336 | - | - |
| 2781 | ENSP00000318264 | ELMOD3 | 1.339E-04 | 0.335 | - | - |
| 2782 | ENSP00000295619 | PROK2 | 3.803E-05 | 0.335 | - | - |
| 2783 | ENSP00000310193 | TBC1D10C | 6.458E-05 | 0.333 | - | - |
| 2784 | ENSP00000216775 | CPNE6 | 5.228E-05 | 0.332 | - | - |
| 2785 | ENSP00000368982 | KATNB1 | 6.384E-05 | 0.332 | - | - |
| 2786 | ENSP00000363079 | MBL2 | 1.344E-04 | 0.331 | - | - |
| 2787 | ENSP00000354398 | CYTH1 | 7.340E-05 | 0.331 | - | - |
| 2788 | ENSP00000350570 | IFT20 | 1.192E-04 | 0.330 | - | - |
| 2789 | ENSP00000358407 | ADAM30 | 1.202E-04 | 0.330 | - | - |
| 2790 | ENSP00000265500 | NDUFC1 | 4.864E-05 | 0.329 | - | - |
| 2791 | ENSP00000372313 | MSLN | 3.990E-05 | 0.329 | - | - |
| 2792 | ENSP00000296953 | CREBRF | 9.332E-05 | 0.329 | - | - |
| 2793 | ENSP00000367454 | DFFB | 8.142E-05 | 0.329 | - | - |
| 2794 | ENSP00000364938 | TMEM82 | 5.465E-05 | 0.327 | - | - |
| 2795 | ENSP00000469538 | AC104057.1 | 1.506E-06 | 0.327 | - | - |
| 2796 | ENSP00000219593 | ZP2 | 3.601E-05 | 0.320 | - | - |
| 2797 | ENSP00000353820 | CYP2D6 | 7.227E-05 | 0.319 | - | - |
| 2798 | ENSP00000360871 | FCN1 | 8.031E-05 | 0.318 | - | - |
| 2799 | ENSP00000259938 | CLPS | 7.228E-05 | 0.317 | - | - |
| 2800 | ENSP00000247933 | IDUA | 5.049E-05 | 0.317 | - | - |
| 2801 | ENSP00000228606 | CYP27B1 | 7.101E-05 | 0.316 | - | - |
| 2802 | ENSP00000280979 | AKAP6 | 5.585E-05 | 0.315 | - | - |
| 2803 | ENSP00000451605 | MC1R | 6.876E-05 | 0.315 | - | - |
| 2804 | ENSP00000360215 | ZBP1 | 4.787E-05 | 0.313 | - | - |
| 2805 | ENSP00000340396 | GBP5 | 8.901E-05 | 0.313 | - | - |
| 2806 | ENSP00000355601 | EGLN1 | 9.606E-05 | 0.313 | - | - |
| 2807 | ENSP00000305766 | MRGPRX1 | 4.930E-05 | 0.312 | - | - |
| 2808 | ENSP00000460475 | NLRP1 | 1.419E-04 | 0.312 | - | - |
| 2809 | ENSP00000436926 | NFRKB | 9.333E-05 | 0.310 | - | - |
| 2810 | ENSP00000333496 | KCND2 | 3.402E-05 | 0.310 | - | - |
| 2811 | ENSP00000314910 | DKK3 | 7.468E-05 | 0.309 | - | - |
| 2812 | ENSP00000396896 | DNAJC4 | 3.986E-05 | 0.309 | - | - |
| 2813 | ENSP00000376423 | SPRR2A | 4.697E-05 | 0.309 | - | - |
| 2814 | ENSP00000218176 | KCND1 | 3.517E-05 | 0.309 | - | - |
| 2815 | ENSP00000325978 | LRRC31 | 8.034E-05 | 0.309 | - | - |
| 2816 | ENSP00000359783 | IFI44 | 9.982E-05 | 0.308 | - | - |
| 2817 | ENSP00000414904 | CTSO | 2.831E-05 | 0.308 | - | - |
| 2818 | ENSP00000357829 | METTL10 | 7.409E-05 | 0.306 | - | - |
| 2819 | ENSP00000284437 | C4orf19 | 6.027E-05 | 0.305 | - | - |
| 2820 | ENSP00000243349 | ACVR1C | 6.243E-05 | 0.305 | - | - |
| 2821 | ENSP00000363226 | C10orf53 | 1.829E-04 | 0.305 | - | - |
| 2822 | ENSP00000262150 | CDH19 | 6.277E-05 | 0.304 | - | - |
| 2823 | ENSP00000246080 | TCF15 | 3.389E-05 | 0.304 | - | - |
| 2824 | ENSP00000302327 | PYGO1 | 5.518E-05 | 0.303 | - | - |
| 2825 | ENSP00000344173 | GRM8 | 5.734E-05 | 0.302 | - | - |
| 2826 | ENSP00000349324 | TNFRSF10C | 5.715E-05 | 0.300 | - | - |
| 2827 | ENSP00000308535 | GOLT1A | 5.642E-05 | 0.300 | - | - |
| 2828 | ENSP00000306390 | PRSS27 | 5.914E-05 | 0.300 | - | - |
| 2829 | ENSP00000333097 | FIGLA | 2.903E-05 | 0.297 | - | - |
| 2830 | ENSP00000200453 | PPP1R15A | 5.986E-05 | 0.296 | - | - |
| 2831 | ENSP00000357594 | FAM26F | 5.609E-05 | 0.295 | - | - |
| 2832 | ENSP00000371532 | VLDLR | 6.977E-05 | 0.295 | - | - |
| 2833 | ENSP00000334187 | LCE1F | 4.532E-05 | 0.294 | - | - |
| 2834 | ENSP00000308620 | RAG2 | 1.655E-04 | 0.292 | - | - |
| 2835 | ENSP00000293662 | GRASP | 3.754E-05 | 0.292 | - | - |
| 2836 | ENSP00000260653 | SIX3 | 3.003E-05 | 0.290 | - | - |
| 2837 | ENSP00000373472 | ADAMTS7 | 5.259E-05 | 0.290 | - | - |
| 2838 | ENSP00000296145 | TDGF1 | 4.196E-05 | 0.289 | - | - |
| 2839 | ENSP00000230568 | LY86 | 6.015E-05 | 0.288 | - | - |
| 2840 | ENSP00000269881 | CALR3 | 3.070E-05 | 0.287 | - | - |
| 2841 | ENSP00000346227 | PKIB | 1.011E-04 | 0.287 | - | - |
| 2842 | ENSP00000364000 | COL5A2 | 6.225E-05 | 0.286 | - | - |
| 2843 | ENSP00000358071 | SEC23IP | 5.669E-05 | 0.286 | - | - |
| 2844 | ENSP00000341504 | DCAKD | 2.192E-03 | 0.286 | - | - |
| 2845 | ENSP00000264400 | RASGEF1B | 2.625E-05 | 0.285 | - | - |
| 2846 | ENSP00000342392 | MESP2 | 3.382E-05 | 0.285 | - | - |
| 2847 | ENSP00000338171 | SKAP1 | 6.578E-05 | 0.284 | - | - |
| 2848 | ENSP00000286574 | WIF1 | 7.806E-05 | 0.284 | - | - |
| 2849 | ENSP00000302274 | C1QTNF4 | 6.408E-05 | 0.283 | - | - |
| 2850 | ENSP00000341170 | PTN | 6.100E-05 | 0.282 | - | - |
| 2851 | ENSP00000374274 | SSC5D | 3.708E-05 | 0.280 | - | - |
| 2852 | ENSP00000248150 | GNG13 | 3.697E-05 | 0.280 | - | - |
| 2853 | ENSP00000351686 | PRDM10 | 1.955E-04 | 0.280 | - | - |
| 2854 | ENSP00000472293 | AC026407.1 | 2.665E-06 | 0.279 | - | - |
| 2855 | ENSP00000383394 | POM121L7 | 3.376E-05 | 0.279 | - | - |
| 2856 | ENSP00000225737 | AKAP10 | 4.223E-05 | 0.279 | - | - |
| 2857 | ENSP00000352561 | CALCR | 5.627E-05 | 0.277 | - | - |
| 2858 | ENSP00000364349 | ATF6B | 3.973E-05 | 0.276 | - | - |
| 2859 | ENSP00000382083 | COL25A1 | 3.929E-05 | 0.275 | - | - |
| 2860 | ENSP00000366237 | DFFA | 1.009E-04 | 0.274 | - | - |
| 2861 | ENSP00000329374 | SERPINA7 | 4.546E-05 | 0.273 | - | - |
| 2862 | ENSP00000296513 | ADAD1 | 9.090E-05 | 0.273 | - | - |
| 2863 | ENSP00000360045 | C1orf141 | 8.089E-05 | 0.273 | - | - |
| 2864 | ENSP00000360829 | GLT6D1 | 4.332E-05 | 0.272 | - | - |
| 2865 | ENSP00000460682 | CLEC3A | 5.147E-05 | 0.272 | - | - |
| 2866 | ENSP00000381566 | MFSD12 | 7.562E-05 | 0.271 | - | - |
| 2867 | ENSP00000218867 | SGCG | 2.299E-05 | 0.270 | - | - |
| 2868 | ENSP00000324532 | LAMA3 | 4.063E-05 | 0.270 | - | - |
| 2869 | ENSP00000221980 | ICAM5 | 5.507E-05 | 0.270 | - | - |
| 2870 | ENSP00000366641 | SLC2A5 | 5.564E-05 | 0.269 | - | - |
| 2871 | ENSP00000216139 | ACR | 2.217E-05 | 0.269 | - | - |
| 2872 | ENSP00000323978 | CRIPAK | 5.924E-05 | 0.269 | - | - |
| 2873 | ENSP00000359497 | GBP2 | 1.513E-04 | 0.268 | - | - |
| 2874 | ENSP00000397679 | ENDOU | 6.520E-05 | 0.267 | - | - |
| 2875 | ENSP00000296595 | TMEM161B | 5.310E-05 | 0.267 | - | - |
| 2876 | ENSP00000464149 | ACE | 8.384E-05 | 0.267 | - | - |
| 2877 | ENSP00000304767 | P2RY1 | 3.102E-05 | 0.266 | - | - |
| 2878 | ENSP00000365529 | GPR158 | 7.059E-05 | 0.266 | - | - |
| 2879 | ENSP00000385586 | HOXD12 | 3.915E-05 | 0.266 | - | - |
| 2880 | ENSP00000236147 | SELL | 1.672E-04 | 0.265 | - | - |
| 2881 | ENSP00000233809 | IGFBP2 | 5.755E-05 | 0.263 | - | - |
| 2882 | ENSP00000357776 | LCE3D | 1.290E-04 | 0.263 | - | - |
| 2883 | ENSP00000291934 | TMEM190 | 4.307E-05 | 0.263 | - | - |
| 2884 | ENSP00000374205 | TBR1 | 5.116E-05 | 0.263 | - | - |
| 2885 | ENSP00000328998 | TRAK1 | 8.984E-05 | 0.262 | - | - |
| 2886 | ENSP00000371420 | GPNMB | 6.419E-05 | 0.260 | - | - |
| 2887 | ENSP00000370639 | ASMT | 4.589E-05 | 0.258 | - | - |
| 2888 | ENSP00000416696 | KIAA1432 | 7.830E-05 | 0.258 | - | - |
| 2889 | ENSP00000308597 | DLEC1 | 5.721E-05 | 0.256 | - | - |
| 2890 | ENSP00000315949 | HOXD8 | 3.165E-05 | 0.255 | - | - |
| 2891 | ENSP00000357581 | NKX6-2 | 5.585E-05 | 0.255 | - | - |
| 2892 | ENSP00000378856 | LGALS9 | 9.973E-05 | 0.255 | - | - |
| 2893 | ENSP00000293780 | CHRNE | 4.562E-05 | 0.255 | - | - |
| 2894 | ENSP00000428325 | PYDC2 | 4.390E-05 | 0.254 | - | - |
| 2895 | ENSP00000352071 | CD163 | 1.906E-04 | 0.254 | - | - |
| 2896 | ENSP00000280097 | HNMT | 3.506E-05 | 0.254 | - | - |
| 2897 | ENSP00000318032 | RPRML | 5.012E-05 | 0.254 | - | - |
| 2898 | ENSP00000304945 | ROBO4 | 3.933E-05 | 0.253 | - | - |
| 2899 | ENSP00000428480 | DLX6 | 4.075E-05 | 0.253 | - | - |
| 2900 | ENSP00000362643 | PHKA1 | 6.826E-05 | 0.253 | - | - |
| 2901 | ENSP00000333779 | EVI2B | 1.108E-04 | 0.252 | - | - |
| 2902 | ENSP00000397181 | RGS4 | 5.956E-05 | 0.252 | - | - |
| 2903 | ENSP00000267803 | DUOXA1 | 3.081E-05 | 0.251 | - | - |
| 2904 | ENSP00000368544 | VIT | 4.348E-05 | 0.251 | - | - |
| 2905 | ENSP00000271324 | CD53 | 1.042E-04 | 0.251 | - | - |
| 2906 | ENSP00000383219 | KRTAP10-6 | 6.980E-05 | 0.249 | - | - |
| 2907 | ENSP00000356058 | FAIM3 | 8.332E-05 | 0.249 | - | - |
| 2908 | ENSP00000375622 | LAIR1 | 1.413E-04 | 0.248 | - | - |
| 2909 | ENSP00000259396 | ORM1 | 4.839E-05 | 0.247 | - | - |
| 2910 | ENSP00000220812 | DKK4 | 1.436E-04 | 0.247 | - | - |
| 2911 | ENSP00000392828 | GPSM1 | 6.965E-05 | 0.247 | - | - |
| 2912 | ENSP00000395902 | - | 3.922E-05 | 0.246 | - | - |
| 2913 | ENSP00000362776 | COL16A1 | 3.166E-05 | 0.246 | - | - |
| 2914 | ENSP00000360841 | OBP2A | 7.355E-05 | 0.246 | - | - |
| 2915 | ENSP00000313809 | AMBN | 7.802E-05 | 0.246 | - | - |
| 2916 | ENSP00000335544 | CCKBR | 3.458E-05 | 0.245 | - | - |
| 2917 | ENSP00000232854 | HEMK1 | 2.672E-05 | 0.245 | - | - |
| 2918 | ENSP00000351086 | RPS6KL1 | 4.920E-05 | 0.244 | - | - |
| 2919 | ENSP00000315006 | SLC28A2 | 3.556E-05 | 0.244 | - | - |
| 2920 | ENSP00000262659 | CCM2L | 9.941E-05 | 0.242 | - | - |
| 2921 | ENSP00000381567 | TMF1 | 6.215E-05 | 0.242 | - | - |
| 2922 | ENSP00000310565 | DSEL | 4.468E-05 | 0.241 | - | - |
| 2923 | ENSP00000358510 | FUNDC2 | 3.571E-05 | 0.241 | - | - |
| 2924 | ENSP00000336829 | FGG | 1.068E-04 | 0.241 | - | - |
| 2925 | ENSP00000366482 | FXN | 2.800E-03 | 0.240 | - | - |
| 2926 | ENSP00000418081 | CACNA2D2 | 9.177E-05 | 0.240 | - | - |
| 2927 | ENSP00000387091 | ZNF365 | 5.851E-05 | 0.238 | - | - |
| 2928 | ENSP00000325808 | LRRN4CL | 1.779E-04 | 0.237 | - | - |
| 2929 | ENSP00000234798 | TPSG1 | 3.399E-05 | 0.237 | - | - |
| 2930 | ENSP00000265071 | CDH6 | 5.132E-05 | 0.236 | - | - |
| 2931 | ENSP00000451030 | RGS6 | 6.047E-05 | 0.235 | - | - |
| 2932 | ENSP00000368517 | ECHDC3 | 4.645E-05 | 0.235 | - | - |
| 2933 | ENSP00000323328 | SIGLEC7 | 3.308E-05 | 0.234 | - | - |
| 2934 | ENSP00000284136 | SEMA3D | 4.460E-05 | 0.234 | - | - |
| 2935 | ENSP00000371376 | TLR6 | 1.295E-04 | 0.233 | - | - |
| 2936 | ENSP00000341640 | COL9A3 | 3.270E-05 | 0.233 | - | - |
| 2937 | ENSP00000368496 | B3GALT6 | 4.543E-05 | 0.232 | - | - |
| 2938 | ENSP00000331857 | NLRP9 | 7.316E-05 | 0.232 | - | - |
| 2939 | ENSP00000412957 | BIRC8 | 6.053E-05 | 0.232 | - | - |
| 2940 | ENSP00000366093 | RORB | 6.698E-05 | 0.231 | - | - |
| 2941 | ENSP00000327290 | ITGA11 | 3.818E-05 | 0.231 | - | - |
| 2942 | ENSP00000348384 | LAMB3 | 5.012E-05 | 0.231 | - | - |
| 2943 | ENSP00000254466 | GAS2L2 | 4.266E-05 | 0.231 | - | - |
| 2944 | ENSP00000473233 | SPIB | 9.065E-05 | 0.231 | - | - |
| 2945 | ENSP00000324570 | GYLTL1B | 7.263E-05 | 0.230 | - | - |
| 2946 | ENSP00000331698 | KCNJ15 | 3.461E-05 | 0.229 | - | - |
| 2947 | ENSP00000309953 | EFEMP2 | 5.311E-05 | 0.229 | - | - |
| 2948 | ENSP00000359100 | AMY1A | 4.520E-05 | 0.229 | - | - |
| 2949 | ENSP00000327545 | NPTXR | 5.017E-05 | 0.228 | - | - |
| 2950 | ENSP00000323155 | DAND5 | 3.909E-05 | 0.227 | - | - |
| 2951 | ENSP00000370443 | ADAMTS6 | 5.550E-05 | 0.227 | - | - |
| 2952 | ENSP00000302552 | GLOD5 | 8.828E-05 | 0.227 | - | - |
| 2953 | ENSP00000234739 | BCL9 | 4.468E-05 | 0.226 | - | - |
| 2954 | ENSP00000356366 | DENND1B | 7.187E-05 | 0.226 | - | - |
| 2955 | ENSP00000312987 | HNF4A | 6.287E-04 | 0.225 | - | - |
| 2956 | ENSP00000302289 | EMB | 5.022E-05 | 0.225 | - | - |
| 2957 | ENSP00000366246 | GPC6 | 4.217E-05 | 0.224 | - | - |
| 2958 | ENSP00000359410 | EPHX4 | 4.458E-05 | 0.224 | - | - |
| 2959 | ENSP00000289292 | SHROOM4 | 4.965E-05 | 0.224 | - | - |
| 2960 | ENSP00000347213 | ENTPD2 | 3.072E-05 | 0.224 | - | - |
| 2961 | ENSP00000303549 | GPR82 | 5.178E-05 | 0.223 | - | - |
| 2962 | ENSP00000355923 | PTPN14 | 4.395E-05 | 0.223 | - | - |
| 2963 | ENSP00000359404 | KIAA1107 | 3.853E-05 | 0.222 | - | - |
| 2964 | ENSP00000328672 | GPRIN3 | 5.760E-05 | 0.222 | - | - |
| 2965 | ENSP00000288040 | CLEC18A | 4.800E-05 | 0.221 | - | - |
| 2966 | ENSP00000368686 | E2F4 | 6.244E-05 | 0.221 | - | - |
| 2967 | ENSP00000306461 | SPRR1B | 3.731E-05 | 0.220 | - | - |
| 2968 | ENSP00000446916 | PRSS58 | 4.526E-05 | 0.219 | - | - |
| 2969 | ENSP00000436812 | MUC5B | 7.774E-05 | 0.219 | - | - |
| 2970 | ENSP00000262407 | ITGA2B | 8.314E-05 | 0.218 | - | - |
| 2971 | ENSP00000312946 | STAB1 | 4.303E-05 | 0.218 | - | - |
| 2972 | ENSP00000362799 | ACRC | 2.710E-05 | 0.217 | - | - |
| 2973 | ENSP00000277942 | NPFFR1 | 3.037E-05 | 0.216 | - | - |
| 2974 | ENSP00000394033 | KCNK2 | 4.289E-05 | 0.216 | - | - |
| 2975 | ENSP00000346874 | FAR1 | 6.330E-05 | 0.216 | - | - |
| 2976 | ENSP00000435591 | MUC5AC | 7.042E-05 | 0.216 | - | - |
| 2977 | ENSP00000376776 | DBH | 5.628E-05 | 0.216 | - | - |
| 2978 | ENSP00000360687 | PTGDS | 4.386E-05 | 0.216 | - | - |
| 2979 | ENSP00000408464 | DOCK8 | 6.538E-05 | 0.215 | - | - |
| 2980 | ENSP00000356840 | POU2F1 | 8.074E-05 | 0.215 | - | - |
| 2981 | ENSP00000314608 | KLHL11 | 7.683E-05 | 0.215 | - | - |
| 2982 | ENSP00000225805 | C17orf75 | 1.048E-04 | 0.214 | - | - |
| 2983 | ENSP00000345243 | SIGLEC10 | 4.555E-05 | 0.213 | - | - |
| 2984 | ENSP00000312029 | UCP2 | 5.679E-05 | 0.213 | - | - |
| 2985 | ENSP00000311746 | KLK10 | 4.633E-05 | 0.213 | - | - |
| 2986 | ENSP00000355944 | NSL1 | 5.828E-05 | 0.213 | - | - |
| 2987 | ENSP00000230588 | MEP1A | 4.928E-05 | 0.212 | - | - |
| 2988 | ENSP00000310622 | APOBEC4 | 9.283E-05 | 0.211 | - | - |
| 2989 | ENSP00000207157 | TBX15 | 3.848E-05 | 0.211 | - | - |
| 2990 | ENSP00000225648 | HOXB6 | 3.189E-05 | 0.211 | - | - |
| 2991 | ENSP00000355180 | COL6A1 | 4.913E-05 | 0.211 | - | - |
| 2992 | ENSP00000254605 | RRP8 | 2.425E-05 | 0.209 | - | - |
| 2993 | ENSP00000358677 | TBX18 | 3.064E-05 | 0.209 | - | - |
| 2994 | ENSP00000379964 | STK16 | 7.272E-05 | 0.208 | - | - |
| 2995 | ENSP00000347546 | BOC | 2.968E-05 | 0.208 | - | - |
| 2996 | ENSP00000332591 | NPY2R | 7.089E-05 | 0.208 | - | - |
| 2997 | ENSP00000270517 | ECSIT | 9.831E-05 | 0.207 | - | - |
| 2998 | ENSP00000354159 | NLRC4 | 1.047E-04 | 0.205 | - | - |
| 2999 | ENSP00000365884 | TRIM15 | 8.312E-05 | 0.205 | - | - |
| 3000 | ENSP00000291971 | NLRP8 | 1.037E-04 | 0.204 | - | - |
| 3001 | ENSP00000403932 | EFCAB3 | 5.795E-05 | 0.204 | - | - |
| 3002 | ENSP00000284288 | PANX3 | 5.779E-05 | 0.203 | - | - |
| 3003 | ENSP00000264651 | KRT24 | 7.082E-05 | 0.203 | - | - |
| 3004 | ENSP00000358491 | CMC4 | 4.017E-05 | 0.202 | - | - |
| 3005 | ENSP00000297375 | EN2 | 3.724E-05 | 0.202 | - | - |
| 3006 | ENSP00000420298 | CD200 | 4.860E-05 | 0.202 | - | - |
| 3007 | ENSP00000363189 | TMEM222 | 6.256E-05 | 0.200 | - | - |
| 3008 | ENSP00000356087 | IKBKE | 5.079E-05 | 0.200 | - | - |
| 3009 | ENSP00000411658 | ADCYAP1 | 8.870E-05 | 0.200 | - | - |
| 3010 | ENSP00000407262 | ZNF138 | 7.874E-05 | 0.200 | - | - |
| 3011 | ENSP00000225992 | PPY | 5.948E-05 | 0.200 | - | - |
| 3012 | ENSP00000362463 | GLO1 | 4.771E-05 | 0.199 | - | - |
| 3013 | ENSP00000215855 | CRYBB3 | 5.813E-05 | 0.198 | - | - |
| 3014 | ENSP00000371471 | RSAD2 | 8.334E-05 | 0.198 | - | - |
| 3015 | ENSP00000413163 | TMEM130 | 4.797E-05 | 0.198 | - | - |
| 3016 | ENSP00000438513 | DUX4L4 | 4.197E-05 | 0.198 | - | - |
| 3017 | ENSP00000407952 | NAMPTL | 2.936E-05 | 0.197 | - | - |
| 3018 | ENSP00000356581 | GPR126 | 6.424E-05 | 0.196 | - | - |
| 3019 | ENSP00000362746 | GPR21 | 4.077E-05 | 0.196 | - | - |
| 3020 | ENSP00000363459 | PTPN20A | 2.446E-05 | 0.196 | - | - |
| 3021 | ENSP00000260649 | SLC3A1 | 2.677E-05 | 0.196 | - | - |
| 3022 | ENSP00000363545 | ZDBF2 | 7.009E-05 | 0.196 | - | - |
| 3023 | ENSP00000354652 | NPY1R | 6.378E-05 | 0.196 | - | - |
| 3024 | ENSP00000322108 | C9orf84 | 9.759E-05 | 0.196 | - | - |
| 3025 | ENSP00000371682 | DCAF16 | 7.196E-05 | 0.196 | - | - |
| 3026 | ENSP00000445162 | GTF2H3 | 5.994E-05 | 0.195 | - | - |
| 3027 | ENSP00000385470 | TNFSF18 | 2.033E-04 | 0.194 | - | - |
| 3028 | ENSP00000301464 | IGFBP6 | 4.080E-05 | 0.193 | - | - |
| 3029 | ENSP00000364691 | CROCC | 1.242E-04 | 0.193 | - | - |
| 3030 | ENSP00000217381 | SNTA1 | 3.479E-05 | 0.193 | - | - |
| 3031 | ENSP00000330877 | TEX40 | 5.548E-05 | 0.192 | - | - |
| 3032 | ENSP00000261177 | TSPAN11 | 1.063E-04 | 0.192 | - | - |
| 3033 | ENSP00000367851 | CYBB | 3.893E-05 | 0.191 | - | - |
| 3034 | ENSP00000376397 | CD300LB | 3.360E-05 | 0.191 | - | - |
| 3035 | ENSP00000360761 | AGPAT2 | 5.279E-05 | 0.190 | - | - |
| 3036 | ENSP00000360899 | BEND5 | 5.204E-05 | 0.189 | - | - |
| 3037 | ENSP00000451304 | DUX4 | 4.141E-05 | 0.189 | - | - |
| 3038 | ENSP00000370003 | BNIP3L | 6.362E-05 | 0.189 | - | - |
| 3039 | ENSP00000346340 | COPS8 | 6.586E-05 | 0.189 | - | - |
| 3040 | ENSP00000285311 | DKK2 | 3.896E-05 | 0.189 | - | - |
| 3041 | ENSP00000364934 | CLIC1 | 3.618E-05 | 0.188 | - | - |
| 3042 | ENSP00000388684 | TMEM247 | 4.910E-05 | 0.188 | - | - |
| 3043 | ENSP00000297029 | SCIN | 2.882E-05 | 0.188 | - | - |
| 3044 | ENSP00000273610 | UCN2 | 7.838E-05 | 0.188 | - | - |
| 3045 | ENSP00000328230 | FFAR3 | 2.939E-05 | 0.187 | - | - |
| 3046 | ENSP00000374490 | DNAH17 | 7.400E-05 | 0.187 | - | - |
| 3047 | ENSP00000304355 | CCDC126 | 7.182E-05 | 0.187 | - | - |
| 3048 | ENSP00000339906 | PAX4 | 4.330E-05 | 0.187 | - | - |
| 3049 | ENSP00000262865 | BPI | 5.473E-05 | 0.187 | - | - |
| 3050 | ENSP00000351885 | PPP2R4 | 5.149E-05 | 0.186 | - | - |
| 3051 | ENSP00000401980 | MAVS | 5.895E-05 | 0.186 | - | - |
| 3052 | ENSP00000353542 | SPRR2D | 3.776E-05 | 0.185 | - | - |
| 3053 | ENSP00000281882 | CFC1B | 4.822E-05 | 0.185 | - | - |
| 3054 | ENSP00000349705 | TOP3B | 6.962E-05 | 0.185 | - | - |
| 3055 | ENSP00000315167 | ALOX12B | 6.319E-05 | 0.185 | - | - |
| 3056 | ENSP00000361569 | IER5L | 6.056E-05 | 0.184 | - | - |
| 3057 | ENSP00000359301 | MAGEA3 | 1.144E-04 | 0.184 | - | - |
| 3058 | ENSP00000384708 | FSHR | 6.198E-05 | 0.184 | - | - |
| 3059 | ENSP00000308219 | RGMB | 3.541E-05 | 0.182 | - | - |
| 3060 | ENSP00000368080 | CDKL4 | 3.413E-05 | 0.181 | - | - |
| 3061 | ENSP00000302724 | INSL5 | 4.636E-05 | 0.181 | - | - |
| 3062 | ENSP00000449795 | TRIAP1 | 5.212E-05 | 0.181 | - | - |
| 3063 | ENSP00000370966 | CRYZL1 | 5.640E-05 | 0.180 | - | - |
| 3064 | ENSP00000415900 | IL31RA | 3.069E-05 | 0.180 | - | - |
| 3065 | ENSP00000265922 | DBC1 | 3.955E-05 | 0.179 | - | - |
| 3066 | ENSP00000241261 | TNFSF10 | 1.323E-04 | 0.178 | - | - |
| 3067 | ENSP00000386341 | TICAM2 | 3.990E-05 | 0.176 | - | - |
| 3068 | ENSP00000304147 | MYEOV2 | 4.701E-05 | 0.175 | - | - |
| 3069 | ENSP00000307080 | EGLN2 | 7.557E-05 | 0.174 | - | - |
| 3070 | ENSP00000344829 | QPCT | 8.752E-05 | 0.174 | - | - |
| 3071 | ENSP00000226798 | FRG1 | 5.370E-05 | 0.174 | - | - |
| 3072 | ENSP00000283936 | KCNJ16 | 3.185E-05 | 0.173 | - | - |
| 3073 | ENSP00000268070 | ADAMTS17 | 6.972E-05 | 0.173 | - | - |
| 3074 | ENSP00000218436 | ITIH6 | 3.503E-05 | 0.173 | - | - |
| 3075 | ENSP00000346103 | GPX4 | 4.567E-05 | 0.172 | - | - |
| 3076 | ENSP00000356700 | C1orf105 | 4.880E-05 | 0.171 | - | - |
| 3077 | ENSP00000299529 | CRABP1 | 3.189E-05 | 0.171 | - | - |
| 3078 | ENSP00000215479 | AMELY | 4.041E-05 | 0.171 | - | - |
| 3079 | ENSP00000383225 | KRTAP10-4 | 2.919E-05 | 0.171 | - | - |
| 3080 | ENSP00000367743 | TSPAN7 | 5.126E-05 | 0.171 | - | - |
| 3081 | ENSP00000401450 | RPL28 | 7.854E-05 | 0.170 | - | - |
| 3082 | ENSP00000259216 | CFC1 | 3.641E-05 | 0.170 | - | - |
| 3083 | ENSP00000469960 | AL138815.2 | 3.845E-06 | 0.170 | - | - |
| 3084 | ENSP00000393719 | CLEC17A | 1.055E-04 | 0.169 | - | - |
| 3085 | ENSP00000309741 | SLCO4C1 | 4.798E-05 | 0.169 | - | - |
| 3086 | ENSP00000282549 | OTX1 | 3.780E-05 | 0.168 | - | - |
| 3087 | ENSP00000251582 | ADAMTS2 | 5.045E-05 | 0.168 | - | - |
| 3088 | ENSP00000366356 | - | 4.388E-05 | 0.168 | - | - |
| 3089 | ENSP00000252997 | GATA5 | 2.241E-05 | 0.167 | - | - |
| 3090 | ENSP00000396160 | FBRSL1 | 5.485E-05 | 0.166 | - | - |
| 3091 | ENSP00000352565 | HAND2 | 6.733E-05 | 0.166 | - | - |
| 3092 | ENSP00000334611 | TMPRSS11A | 3.711E-05 | 0.166 | - | - |
| 3093 | ENSP00000411010 | DNTTIP2 | 6.291E-05 | 0.166 | - | - |
| 3094 | ENSP00000352936 | SPINK5 | 6.495E-05 | 0.165 | - | - |
| 3095 | ENSP00000219207 | PLLP | 6.081E-05 | 0.164 | - | - |
| 3096 | ENSP00000229281 | C12orf57 | 7.944E-05 | 0.163 | - | - |
| 3097 | ENSP00000356162 | KISS1 | 3.223E-05 | 0.162 | - | - |
| 3098 | ENSP00000346550 | ANXA6 | 5.463E-05 | 0.162 | - | - |
| 3099 | ENSP00000323479 | B3GALNT1 | 3.487E-05 | 0.161 | - | - |
| 3100 | ENSP00000354623 | DFNB31 | 4.517E-05 | 0.161 | - | - |
| 3101 | ENSP00000281171 | PTPRO | 3.865E-05 | 0.160 | - | - |
| 3102 | ENSP00000344668 | KRIT1 | 3.742E-05 | 0.160 | - | - |
| 3103 | ENSP00000380929 | TCP10 | 7.169E-05 | 0.160 | - | - |
| 3104 | ENSP00000265022 | DGKG | 3.804E-05 | 0.159 | - | - |
| 3105 | ENSP00000341828 | CHIA | 6.139E-05 | 0.158 | - | - |
| 3106 | ENSP00000300557 | PRR15L | 5.526E-05 | 0.157 | - | - |
| 3107 | ENSP00000279396 | TMEM219 | 1.478E-04 | 0.157 | - | - |
| 3108 | ENSP00000367378 | GPR34 | 4.213E-05 | 0.157 | - | - |
| 3109 | ENSP00000351789 | PELI1 | 1.132E-04 | 0.157 | - | - |
| 3110 | ENSP00000240986 | GJA8 | 4.971E-05 | 0.156 | - | - |
| 3111 | ENSP00000306523 | INSM2 | 4.303E-05 | 0.155 | - | - |
| 3112 | ENSP00000384192 | RASGRP3 | 6.585E-05 | 0.154 | - | - |
| 3113 | ENSP00000343325 | PKN1 | 5.078E-04 | 0.154 | - | - |
| 3114 | ENSP00000259915 | POU5F1 | 3.586E-05 | 0.153 | - | - |
| 3115 | ENSP00000332737 | UNC5A | 3.454E-05 | 0.153 | - | - |
| 3116 | ENSP00000369473 | N4BP2L1 | 7.468E-05 | 0.152 | - | - |
| 3117 | ENSP00000389252 | NPNT | 3.173E-05 | 0.152 | - | - |
| 3118 | ENSP00000262901 | SIM1 | 4.800E-05 | 0.152 | - | - |
| 3119 | ENSP00000357175 | ETV3 | 4.076E-05 | 0.152 | - | - |
| 3120 | ENSP00000281317 | MMAA | 4.304E-05 | 0.151 | - | - |
| 3121 | ENSP00000347294 | EYA4 | 4.399E-05 | 0.151 | - | - |
| 3122 | ENSP00000325708 | CD37 | 9.466E-05 | 0.151 | - | - |
| 3123 | ENSP00000341698 | AKT1S1 | 4.519E-05 | 0.150 | - | - |
| 3124 | ENSP00000223368 | BCL7B | 5.540E-05 | 0.150 | - | - |
| 3125 | ENSP00000473047 | KLK11 | 4.645E-05 | 0.150 | - | - |
| 3126 | ENSP00000403649 | CGB8 | 3.961E-05 | 0.149 | - | - |
| 3127 | ENSP00000420514 | BAG1 | 6.050E-05 | 0.149 | - | - |
| 3128 | ENSP00000318029 | ODF3L2 | 8.142E-05 | 0.148 | - | - |
| 3129 | ENSP00000464036 | CDK11B | 7.010E-05 | 0.148 | - | - |
| 3130 | ENSP00000388741 | SPATA3 | 3.099E-05 | 0.147 | - | - |
| 3131 | ENSP00000359504 | GBP1 | 1.741E-04 | 0.146 | - | - |
| 3132 | ENSP00000351767 | COL20A1 | 5.123E-05 | 0.146 | - | - |
| 3133 | ENSP00000369351 | TET2 | 8.004E-05 | 0.145 | - | - |
| 3134 | ENSP00000308815 | CCL19 | 1.251E-04 | 0.144 | - | - |
| 3135 | ENSP00000195455 | C4orf6 | 6.462E-05 | 0.144 | - | - |
| 3136 | ENSP00000289989 | C8orf58 | 7.863E-05 | 0.144 | - | - |
| 3137 | ENSP00000257940 | ZC3H10 | 5.476E-05 | 0.143 | - | - |
| 3138 | ENSP00000360058 | FRAT2 | 2.921E-05 | 0.143 | - | - |
| 3139 | ENSP00000366926 | IGFL4 | 5.867E-05 | 0.143 | - | - |
| 3140 | ENSP00000389455 | DIRC3 | 3.990E-05 | 0.142 | - | - |
| 3141 | ENSP00000373420 | RUFY2 | 6.334E-05 | 0.142 | - | - |
| 3142 | ENSP00000330218 | SOX1 | 3.042E-05 | 0.142 | - | - |
| 3143 | ENSP00000315137 | SGPP2 | 5.177E-05 | 0.142 | - | - |
| 3144 | ENSP00000373828 | CCDC154 | 5.195E-05 | 0.142 | - | - |
| 3145 | ENSP00000341838 | TNNI3 | 2.911E-05 | 0.142 | - | - |
| 3146 | ENSP00000268164 | ST8SIA2 | 3.254E-05 | 0.141 | - | - |
| 3147 | ENSP00000351591 | NLGN3 | 1.068E-04 | 0.140 | - | - |
| 3148 | ENSP00000261921 | LOXL1 | 3.612E-05 | 0.139 | - | - |
| 3149 | ENSP00000366081 | SSX4B | 5.277E-07 | 0.139 | - | - |
| 3150 | ENSP00000238667 | FLVCR2 | 4.585E-05 | 0.138 | - | - |
| 3151 | ENSP00000301691 | SOST | 6.850E-05 | 0.138 | - | - |
| 3152 | ENSP00000392452 | FAM219A | 3.087E-05 | 0.138 | - | - |
| 3153 | ENSP00000374507 | CCDC88C | 5.578E-05 | 0.137 | - | - |
| 3154 | ENSP00000342848 | NOXA1 | 8.970E-05 | 0.137 | - | - |
| 3155 | ENSP00000386203 | C1orf229 | 2.330E-05 | 0.137 | - | - |
| 3156 | ENSP00000353094 | SDF4 | 3.998E-05 | 0.137 | - | - |
| 3157 | ENSP00000252674 | MLLT1 | 7.305E-05 | 0.137 | - | - |
| 3158 | ENSP00000331734 | DSCR6 | 1.066E-04 | 0.137 | - | - |
| 3159 | ENSP00000384551 | NXPH1 | 5.652E-05 | 0.135 | - | - |
| 3160 | ENSP00000384534 | INPP5J | 5.151E-05 | 0.135 | - | - |
| 3161 | ENSP00000291707 | SIGLEC12 | 4.768E-05 | 0.134 | - | - |
| 3162 | ENSP00000373136 | OR2H2 | 1.496E-06 | 0.133 | - | - |
| 3163 | ENSP00000366336 | OR2H1 | 1.496E-06 | 0.133 | - | - |
| 3164 | ENSP00000401335 | C16orf90 | 2.793E-05 | 0.132 | - | - |
| 3165 | ENSP00000357206 | NES | 4.564E-05 | 0.132 | - | - |
| 3166 | ENSP00000229955 | GPR63 | 4.780E-05 | 0.130 | - | - |
| 3167 | ENSP00000324277 | APOLD1 | 6.231E-05 | 0.130 | - | - |
| 3168 | ENSP00000221996 | CRX | 4.058E-05 | 0.130 | - | - |
| 3169 | ENSP00000308024 | PCSK1 | 3.599E-05 | 0.130 | - | - |
| 3170 | ENSP00000296575 | HHIP | 2.771E-05 | 0.129 | - | - |
| 3171 | ENSP00000357348 | HEY2 | 2.880E-05 | 0.129 | - | - |
| 3172 | ENSP00000417003 | PLEKHM3 | 5.471E-05 | 0.128 | - | - |
| 3173 | ENSP00000351503 | SPATS2L | 5.583E-05 | 0.128 | - | - |
| 3174 | ENSP00000471921 | SPIB | 1.275E-04 | 0.128 | - | - |
| 3175 | ENSP00000359245 | ABCA4 | 5.089E-05 | 0.127 | - | - |
| 3176 | ENSP00000327943 | SLC5A2 | 3.631E-05 | 0.127 | - | - |
| 3177 | ENSP00000376609 | GRK5 | 1.087E-04 | 0.127 | - | - |
| 3178 | ENSP00000466519 | ZNF229 | 8.982E-05 | 0.126 | - | - |
| 3179 | ENSP00000310814 | STARD6 | 1.055E-04 | 0.126 | - | - |
| 3180 | ENSP00000245907 | C3 | 1.199E-04 | 0.126 | - | - |
| 3181 | ENSP00000347948 | TNFRSF14 | 1.580E-04 | 0.125 | - | - |
| 3182 | ENSP00000308733 | GPR151 | 3.152E-05 | 0.125 | - | - |
| 3183 | ENSP00000362562 | TMEM54 | 3.961E-05 | 0.125 | - | - |
| 3184 | ENSP00000216177 | PNPLA5 | 5.536E-05 | 0.125 | - | - |
| 3185 | ENSP00000246801 | TSKS | 5.905E-05 | 0.125 | - | - |
| 3186 | ENSP00000344430 | IFITM10 | 5.878E-05 | 0.125 | - | - |
| 3187 | ENSP00000369979 | CPXM1 | 5.529E-05 | 0.124 | - | - |
| 3188 | ENSP00000323584 | TSHZ1 | 4.815E-05 | 0.124 | - | - |
| 3189 | ENSP00000360552 | TSHZ2 | 5.410E-05 | 0.124 | - | - |
| 3190 | ENSP00000354590 | SFT2D1 | 5.275E-05 | 0.123 | - | - |
| 3191 | ENSP00000348019 | SLC17A5 | 4.569E-05 | 0.122 | - | - |
| 3192 | ENSP00000375921 | PAX3 | 5.084E-05 | 0.122 | - | - |
| 3193 | ENSP00000396505 | AC142381.1 | 6.659E-07 | 0.122 | - | - |
| 3194 | ENSP00000417710 | DPPA2 | 3.874E-05 | 0.122 | - | - |
| 3195 | ENSP00000414964 | CCDC107 | 5.231E-05 | 0.121 | - | - |
| 3196 | ENSP00000261751 | CHRNB4 | 4.237E-05 | 0.121 | - | - |
| 3197 | ENSP00000436426 | PCDHA7 | 3.123E-05 | 0.120 | - | - |
| 3198 | ENSP00000384886 | IFI30 | 8.406E-05 | 0.120 | - | - |
| 3199 | ENSP00000369666 | FBXL19 | 5.461E-05 | 0.119 | - | - |
| 3200 | ENSP00000358165 | FCGR1A | 8.989E-05 | 0.119 | - | - |
| 3201 | ENSP00000295756 | TRAT1 | 8.600E-05 | 0.118 | - | - |
| 3202 | ENSP00000371693 | TNFRSF19 | 5.247E-05 | 0.118 | - | - |
| 3203 | ENSP00000322304 | PORCN | 4.033E-05 | 0.117 | - | - |
| 3204 | ENSP00000361668 | EDN2 | 2.839E-05 | 0.117 | - | - |
| 3205 | ENSP00000352704 | C20orf112 | 1.592E-04 | 0.116 | - | - |
| 3206 | ENSP00000349358 | C12orf29 | 6.402E-05 | 0.115 | - | - |
| 3207 | ENSP00000342087 | FHIT | 1.209E-04 | 0.115 | - | - |
| 3208 | ENSP00000359401 | C1orf146 | 5.167E-05 | 0.114 | - | - |
| 3209 | ENSP00000271348 | GJA5 | 2.354E-05 | 0.114 | - | - |
| 3210 | ENSP00000441462 | CSMD1 | 8.015E-05 | 0.114 | - | - |
| 3211 | ENSP00000363869 | VSIG4 | 5.167E-05 | 0.113 | - | - |
| 3212 | ENSP00000304811 | UGT2B7 | 3.640E-05 | 0.113 | - | - |
| 3213 | ENSP00000307971 | RFNG | 2.891E-05 | 0.112 | - | - |
| 3214 | ENSP00000450527 | HOPX | 6.272E-05 | 0.112 | - | - |
| 3215 | ENSP00000359085 | NTNG1 | 7.267E-05 | 0.112 | - | - |
| 3216 | ENSP00000340590 | ZCCHC16 | 5.045E-05 | 0.112 | - | - |
| 3217 | ENSP00000415430 | GTSE1 | 8.032E-05 | 0.111 | - | - |
| 3218 | ENSP00000282018 | CYSLTR2 | 4.146E-05 | 0.111 | - | - |
| 3219 | ENSP00000353128 | DCAF12L2 | 5.533E-05 | 0.110 | - | - |
| 3220 | ENSP00000464258 | VHLL | 2.135E-05 | 0.110 | - | - |
| 3221 | ENSP00000366032 | COMMD3 | 6.140E-05 | 0.110 | - | - |
| 3222 | ENSP00000360060 | FRAT1 | 1.207E-04 | 0.109 | - | - |
| 3223 | ENSP00000429344 | PNMA2 | 4.369E-05 | 0.109 | - | - |
| 3224 | ENSP00000263314 | P2RX3 | 2.878E-05 | 0.108 | - | - |
| 3225 | ENSP00000472933 | AC012493.2 | 3.937E-05 | 0.108 | - | - |
| 3226 | ENSP00000324672 | C16orf89 | 5.907E-05 | 0.107 | - | - |
| 3227 | ENSP00000344942 | UNC119B | 4.505E-05 | 0.107 | - | - |
| 3228 | ENSP00000381272 | CD200R1L | 4.491E-05 | 0.107 | - | - |
| 3229 | ENSP00000363512 | ALOX5 | 8.351E-05 | 0.107 | - | - |
| 3230 | ENSP00000259053 | CD302 | 5.077E-05 | 0.107 | - | - |
| 3231 | ENSP00000304915 | IL13 | 4.843E-04 | 0.107 | - | - |
| 3232 | ENSP00000275635 | LAT2 | 1.706E-04 | 0.106 | - | - |
| 3233 | ENSP00000303242 | ITGB2 | 1.261E-04 | 0.105 | - | - |
| 3234 | ENSP00000365233 | TPP2 | 6.762E-05 | 0.105 | - | - |
| 3235 | ENSP00000346879 | NKX2-1 | 4.860E-05 | 0.105 | - | - |
| 3236 | ENSP00000202625 | TGM6 | 5.164E-05 | 0.105 | - | - |
| 3237 | ENSP00000339390 | CDH26 | 4.556E-05 | 0.105 | - | - |
| 3238 | ENSP00000364482 | STK19 | 4.482E-05 | 0.105 | - | - |
| 3239 | ENSP00000261654 | GPR133 | 4.460E-05 | 0.104 | - | - |
| 3240 | ENSP00000423463 | LY75-CD302 | 3.294E-05 | 0.103 | - | - |
| 3241 | ENSP00000425845 | FYB | 7.752E-05 | 0.103 | - | - |
| 3242 | ENSP00000366402 | C9orf135 | 3.117E-05 | 0.103 | - | - |
| 3243 | ENSP00000260323 | UNC13C | 3.350E-05 | 0.103 | - | - |
| 3244 | ENSP00000398131 | GSPT1 | 1.719E-04 | 0.102 | - | - |
| 3245 | ENSP00000426070 | TRPC7 | 2.291E-05 | 0.102 | - | - |
| 3246 | ENSP00000296464 | HSPA4L | 6.847E-05 | 0.102 | - | - |
| 3247 | ENSP00000376317 | TAOK3 | 4.252E-05 | 0.102 | - | - |
| 3248 | ENSP00000258530 | APPL2 | 3.527E-05 | 0.101 | - | - |
| 3249 | ENSP00000389813 | ACAD10 | 6.253E-05 | 0.101 | - | - |
| 3250 | ENSP00000356545 | CACNA1E | 4.371E-05 | 0.101 | - | - |
| 3251 | ENSP00000342850 | SERPINA6 | 3.569E-05 | 0.101 | - | - |
| 3252 | ENSP00000004103 | TMEM176A | 9.949E-05 | 0.100 | - | - |
| 3253 | ENSP00000351132 | RHOT1 | 7.688E-05 | 0.100 | - | - |
| 3254 | ENSP00000321221 | SH2B1 | 3.723E-05 | 0.100 | - | - |
| 3255 | ENSP00000416330 | TGFBI | 4.723E-05 | 0.100 | - | - |
| 3256 | ENSP00000400010 | PTPRJ | 4.116E-05 | 0.098 | - | - |
| 3257 | ENSP00000364524 | PAX7 | 5.294E-05 | 0.098 | - | - |
| 3258 | ENSP00000379057 | SMCR7 | 5.317E-05 | 0.098 | - | - |
| 3259 | ENSP00000353198 | PYY | 7.302E-05 | 0.097 | - | - |
| 3260 | ENSP00000401435 | VPS53 | 5.487E-05 | 0.097 | - | - |
| 3261 | ENSP00000346088 | RPL22 | 4.534E-05 | 0.097 | - | - |
| 3262 | ENSP00000361276 | DYDC1 | 3.891E-05 | 0.097 | - | - |
| 3263 | ENSP00000347596 | EFEMP1 | 6.931E-05 | 0.096 | - | - |
| 3264 | ENSP00000370007 | TMSB4X | 6.405E-05 | 0.096 | - | - |
| 3265 | ENSP00000345133 | CDC42BPG | 5.365E-05 | 0.096 | - | - |
| 3266 | ENSP00000305941 | USH2A | 3.946E-05 | 0.096 | - | - |
| 3267 | ENSP00000470082 | DEDD2 | 6.148E-05 | 0.095 | - | - |
| 3268 | ENSP00000362810 | HCRTR1 | 5.927E-05 | 0.095 | - | - |
| 3269 | ENSP00000248933 | SEZ6L | 4.116E-05 | 0.095 | - | - |
| 3270 | ENSP00000394405 | FAM213B | 8.811E-05 | 0.095 | - | - |
| 3271 | ENSP00000358160 | HIST1H3H | 8.268E-05 | 0.094 | - | - |
| 3272 | ENSP00000297186 | RSPH10B2 | 4.300E-05 | 0.094 | - | - |
| 3273 | ENSP00000357643 | MKI67 | 8.338E-05 | 0.094 | - | - |
| 3274 | ENSP00000365596 | GPR183 | 9.831E-05 | 0.094 | - | - |
| 3275 | ENSP00000337014 | HFE2 | 4.696E-05 | 0.093 | - | - |
| 3276 | ENSP00000374592 | ZC3H12D | 3.159E-05 | 0.093 | - | - |
| 3277 | ENSP00000291041 | PSKH1 | 2.760E-05 | 0.093 | - | - |
| 3278 | ENSP00000438244 | KLRF2 | 4.117E-05 | 0.093 | - | - |
| 3279 | ENSP00000367952 | UCMA | 3.478E-05 | 0.092 | - | - |
| 3280 | ENSP00000269216 | GATA6 | 2.452E-05 | 0.092 | - | - |
| 3281 | ENSP00000359693 | FKBP1C | 5.096E-05 | 0.092 | - | - |
| 3282 | ENSP00000345395 | PAPLN | 4.512E-05 | 0.092 | - | - |
| 3283 | ENSP00000433352 | LMO7 | 8.525E-05 | 0.092 | - | - |
| 3284 | ENSP00000400157 | B3GNT9 | 3.805E-05 | 0.091 | - | - |
| 3285 | ENSP00000216180 | PNPLA3 | 3.226E-05 | 0.091 | - | - |
| 3286 | ENSP00000359490 | GBP4 | 1.155E-04 | 0.091 | - | - |
| 3287 | ENSP00000366955 | KRTAP4-2 | 4.164E-05 | 0.091 | - | - |
| 3288 | ENSP00000352839 | NPSR1 | 4.447E-05 | 0.091 | - | - |
| 3289 | ENSP00000259455 | GABBR2 | 2.865E-05 | 0.090 | - | - |
| 3290 | ENSP00000371364 | TMEM156 | 2.731E-05 | 0.089 | - | - |
| 3291 | ENSP00000359055 | - | 3.113E-05 | 0.089 | - | - |
| 3292 | ENSP00000221459 | LIN7B | 3.727E-05 | 0.089 | - | - |
| 3293 | ENSP00000383223 | KRTAP10-5 | 3.278E-05 | 0.089 | - | - |
| 3294 | ENSP00000276198 | HTR2C | 4.552E-05 | 0.089 | - | - |
| 3295 | ENSP00000315265 | DYNAP | 1.650E-04 | 0.088 | - | - |
| 3296 | ENSP00000385143 | UNC5D | 3.802E-05 | 0.088 | - | - |
| 3297 | ENSP00000361554 | TIE1 | 6.087E-05 | 0.088 | - | - |
| 3298 | ENSP00000362748 | TET1 | 4.756E-05 | 0.087 | - | - |
| 3299 | ENSP00000364683 | BAMBI | 4.117E-05 | 0.087 | - | - |
| 3300 | ENSP00000302707 | FPR1 | 8.666E-05 | 0.087 | - | - |
| 3301 | ENSP00000369299 | TRIM22 | 4.733E-05 | 0.086 | - | - |
| 3302 | ENSP00000371985 | KCNA1 | 6.652E-05 | 0.086 | - | - |
| 3303 | ENSP00000345571 | E2F1 | 5.928E-05 | 0.086 | - | - |
| 3304 | ENSP00000341346 | TEKT1 | 4.772E-05 | 0.085 | - | - |
| 3305 | ENSP00000333950 | FMN1 | 3.760E-05 | 0.085 | - | - |
| 3306 | ENSP00000365759 | DDR1 | 3.614E-05 | 0.085 | - | - |
| 3307 | ENSP00000388293 | TSTD1 | 3.292E-05 | 0.085 | - | - |
| 3308 | ENSP00000296777 | CARTPT | 6.668E-05 | 0.085 | - | - |
| 3309 | ENSP00000392189 | KRTAP9-3 | 1.852E-05 | 0.084 | - | - |
| 3310 | ENSP00000333003 | PRSS55 | 3.412E-05 | 0.084 | - | - |
| 3311 | ENSP00000263087 | ITGAE | 1.830E-04 | 0.084 | - | - |
| 3312 | ENSP00000385247 | CAPN14 | 3.879E-05 | 0.084 | - | - |
| 3313 | ENSP00000411593 | GLRA1 | 5.290E-05 | 0.084 | - | - |
| 3314 | ENSP00000312436 | NFE2 | 5.558E-05 | 0.083 | - | - |
| 3315 | ENSP00000227752 | IL10RA | 4.205E-04 | 0.082 | - | - |
| 3316 | ENSP00000348784 | IGBP1 | 1.317E-04 | 0.082 | - | - |
| 3317 | ENSP00000265634 | NPTX2 | 3.471E-05 | 0.082 | - | - |
| 3318 | ENSP00000269305 | TP53 | 1.717E-04 | 0.081 | - | - |
| 3319 | ENSP00000369959 | TMEM239 | 3.569E-05 | 0.080 | - | - |
| 3320 | ENSP00000370839 | SGCB | 4.035E-05 | 0.080 | - | - |
| 3321 | ENSP00000341867 | SH2D6 | 3.995E-05 | 0.079 | - | - |
| 3322 | ENSP00000257209 | FHOD3 | 3.752E-05 | 0.079 | - | - |
| 3323 | ENSP00000372750 | CHRM5 | 3.356E-05 | 0.079 | - | - |
| 3324 | ENSP00000243213 | IL13RA2 | 4.039E-04 | 0.078 | - | - |
| 3325 | ENSP00000441929 | MUC12 | 5.316E-05 | 0.078 | - | - |
| 3326 | ENSP00000358866 | FLNA | 5.853E-05 | 0.078 | - | - |
| 3327 | ENSP00000312326 | AOC3 | 6.746E-05 | 0.078 | - | - |
| 3328 | ENSP00000301656 | KRT27 | 5.148E-05 | 0.078 | - | - |
| 3329 | ENSP00000381740 | PARP3 | 5.347E-05 | 0.077 | - | - |
| 3330 | ENSP00000392353 | PSMG4 | 3.834E-05 | 0.077 | - | - |
| 3331 | ENSP00000368964 | MAP7D2 | 5.327E-05 | 0.076 | - | - |
| 3332 | ENSP00000304234 | PCDHA10 | 5.279E-05 | 0.076 | - | - |
| 3333 | ENSP00000263593 | SIAE | 2.706E-05 | 0.076 | - | - |
| 3334 | ENSP00000292174 | CXCR5 | 2.131E-04 | 0.076 | - | - |
| 3335 | ENSP00000369816 | SHBG | 6.042E-05 | 0.075 | - | - |
| 3336 | ENSP00000375080 | PPP2R3B | 4.240E-05 | 0.074 | - | - |
| 3337 | ENSP00000325527 | FBN1 | 1.194E-04 | 0.074 | - | - |
| 3338 | ENSP00000230582 | PRSS16 | 3.211E-05 | 0.074 | - | - |
| 3339 | ENSP00000262653 | CBFA2T2 | 2.833E-05 | 0.073 | - | - |
| 3340 | ENSP00000352336 | PLCG2 | 3.752E-05 | 0.073 | - | - |
| 3341 | ENSP00000381333 | CRHR1 | 5.197E-05 | 0.073 | - | - |
| 3342 | ENSP00000262932 | CNPY4 | 7.306E-05 | 0.073 | - | - |
| 3343 | ENSP00000324882 | IQUB | 3.825E-05 | 0.072 | - | - |
| 3344 | ENSP00000268638 | IRF8 | 1.723E-04 | 0.072 | - | - |
| 3345 | ENSP00000223215 | MEST | 3.809E-05 | 0.072 | - | - |
| 3346 | ENSP00000261707 | SLC6A4 | 2.764E-05 | 0.071 | - | - |
| 3347 | ENSP00000375557 | POU5F1B | 4.621E-05 | 0.070 | - | - |
| 3348 | ENSP00000357583 | INPP5A | 7.771E-05 | 0.070 | - | - |
| 3349 | ENSP00000383398 | DEFB115 | 2.932E-05 | 0.070 | - | - |
| 3350 | ENSP00000373600 | LRRK1 | 7.119E-04 | 0.070 | - | - |
| 3351 | ENSP00000394227 | CSF2RA | 1.718E-04 | 0.069 | - | - |
| 3352 | ENSP00000349616 | ADIPOR2 | 4.579E-05 | 0.069 | - | - |
| 3353 | ENSP00000323194 | PLXNA4 | 3.194E-05 | 0.069 | - | - |
| 3354 | ENSP00000362111 | TSPAN6 | 5.378E-05 | 0.069 | - | - |
| 3355 | ENSP00000300177 | GREM1 | 4.999E-05 | 0.068 | - | - |
| 3356 | ENSP00000254301 | LGALS3 | 7.945E-05 | 0.068 | - | - |
| 3357 | ENSP00000316675 | PVRIG | 5.033E-05 | 0.068 | - | - |
| 3358 | ENSP00000283713 | VILL | 3.179E-05 | 0.068 | - | - |
| 3359 | ENSP00000328495 | C21orf128 | 5.455E-05 | 0.067 | - | - |
| 3360 | ENSP00000349884 | GALP | 3.109E-05 | 0.067 | - | - |
| 3361 | ENSP00000375857 | LRRFIP1 | 5.406E-05 | 0.067 | - | - |
| 3362 | ENSP00000376123 | SH2D2A | 1.609E-05 | 0.066 | - | - |
| 3363 | ENSP00000309818 | TRHR | 2.671E-05 | 0.066 | - | - |
| 3364 | ENSP00000357060 | ATP1A4 | 4.782E-05 | 0.066 | - | - |
| 3365 | ENSP00000269720 | CTB-55O6.8 | 6.048E-05 | 0.066 | - | - |
| 3366 | ENSP00000279386 | TBX6 | 5.586E-05 | 0.066 | - | - |
| 3367 | ENSP00000222823 | NOD1 | 1.680E-04 | 0.066 | - | - |
| 3368 | ENSP00000452296 | POTEM | 3.378E-05 | 0.065 | - | - |
| 3369 | ENSP00000412886 | CAMTA2 | 6.740E-05 | 0.065 | - | - |
| 3370 | ENSP00000408979 | KIF5A | 6.400E-05 | 0.065 | - | - |
| 3371 | ENSP00000337040 | UNC119 | 4.833E-05 | 0.064 | - | - |
| 3372 | ENSP00000454861 | CTC-554D6.1 | 2.234E-05 | 0.064 | - | - |
| 3373 | ENSP00000305731 | CST1 | 3.075E-05 | 0.064 | - | - |
| 3374 | ENSP00000441875 | PHB2 | 9.864E-05 | 0.064 | - | - |
| 3375 | ENSP00000458791 | GPR139 | 1.277E-04 | 0.063 | - | - |
| 3376 | ENSP00000356779 | CCDC181 | 4.121E-05 | 0.062 | - | - |
| 3377 | ENSP00000354734 | WISP3 | 2.827E-05 | 0.062 | - | - |
| 3378 | ENSP00000262406 | RGS9 | 2.790E-04 | 0.062 | - | - |
| 3379 | ENSP00000339191 | CAV1 | 9.583E-05 | 0.062 | - | - |
| 3380 | ENSP00000368190 | NPHS1 | 3.734E-05 | 0.061 | - | - |
| 3381 | ENSP00000381768 | CDH23 | 4.514E-05 | 0.061 | - | - |
| 3382 | ENSP00000340292 | DLK1 | 8.578E-05 | 0.061 | - | - |
| 3383 | ENSP00000423134 | - | 2.591E-05 | 0.061 | - | - |
| 3384 | ENSP00000359526 | SLC25A28 | 6.847E-05 | 0.061 | - | - |
| 3385 | ENSP00000262041 | MEOX2 | 3.289E-05 | 0.061 | - | - |
| 3386 | ENSP00000386962 | CATSPERG | 3.173E-05 | 0.060 | - | - |
| 3387 | ENSP00000271450 | FCGR2A | 1.563E-04 | 0.060 | - | - |
| 3388 | ENSP00000373575 | C6orf163 | 4.660E-05 | 0.060 | - | - |
| 3389 | ENSP00000338369 | CELA3B | 7.941E-05 | 0.060 | - | - |
| 3390 | ENSP00000341610 | C15orf48 | 5.003E-05 | 0.060 | - | - |
| 3391 | ENSP00000372224 | HGFAC | 2.491E-05 | 0.059 | - | - |
| 3392 | ENSP00000438468 | HHAT | 3.879E-05 | 0.058 | - | - |
| 3393 | ENSP00000325508 | C11orf71 | 3.104E-05 | 0.058 | - | - |
| 3394 | ENSP00000408005 | SLC9A3R2 | 4.096E-05 | 0.058 | - | - |
| 3395 | ENSP00000363046 | IPMK | 6.490E-05 | 0.058 | - | - |
| 3396 | ENSP00000255226 | SLC14A2 | 3.279E-05 | 0.058 | - | - |
| 3397 | ENSP00000309148 | KLK6 | 3.139E-05 | 0.058 | - | - |
| 3398 | ENSP00000466775 | APOC2 | 5.382E-05 | 0.058 | - | - |
| 3399 | ENSP00000379441 | JMY | 5.351E-05 | 0.058 | - | - |
| 3400 | ENSP00000346196 | NDUFV3 | 4.873E-05 | 0.058 | - | - |
| 3401 | ENSP00000305065 | ZBBX | 6.833E-05 | 0.058 | - | - |
| 3402 | ENSP00000420959 | C2orf15 | 9.963E-05 | 0.058 | - | - |
| 3403 | ENSP00000365963 | PPP1R11 | 6.388E-05 | 0.057 | - | - |
| 3404 | ENSP00000334866 | KRTAP15-1 | 3.341E-05 | 0.057 | - | - |
| 3405 | ENSP00000378324 | DNAJA4 | 5.196E-05 | 0.057 | - | - |
| 3406 | ENSP00000428338 | SPSB2 | 2.639E-05 | 0.057 | - | - |
| 3407 | ENSP00000242776 | DDX39A | 6.360E-05 | 0.057 | - | - |
| 3408 | ENSP00000380247 | HRH1 | 6.018E-05 | 0.056 | - | - |
| 3409 | ENSP00000372170 | MSX1 | 9.979E-05 | 0.056 | - | - |
| 3410 | ENSP00000369899 | RRAGA | 4.876E-05 | 0.055 | - | - |
| 3411 | ENSP00000278882 | FRG1B | 4.300E-05 | 0.055 | - | - |
| 3412 | ENSP00000275216 | TAAR1 | 3.805E-05 | 0.055 | - | - |
| 3413 | ENSP00000356057 | TMEM181 | 5.123E-05 | 0.055 | - | - |
| 3414 | ENSP00000243167 | FAAH | 2.199E-05 | 0.055 | - | - |
| 3415 | ENSP00000275169 | GPR6 | 3.651E-05 | 0.055 | - | - |
| 3416 | ENSP00000335620 | GSTA1 | 3.199E-05 | 0.054 | - | - |
| 3417 | ENSP00000218894 | SUPT20H | 5.233E-05 | 0.054 | - | - |
| 3418 | ENSP00000411932 | LMOD2 | 3.428E-05 | 0.054 | - | - |
| 3419 | ENSP00000357122 | PYHIN1 | 5.758E-05 | 0.054 | - | - |
| 3420 | ENSP00000341803 | TCTEX1D4 | 2.983E-05 | 0.053 | - | - |
| 3421 | ENSP00000361761 | WFDC2 | 3.437E-05 | 0.052 | - | - |
| 3422 | ENSP00000363812 | RXRB | 3.033E-05 | 0.052 | - | - |
| 3423 | ENSP00000398736 | CYR61 | 8.942E-05 | 0.052 | - | - |
| 3424 | ENSP00000302846 | PTGER4 | 1.294E-04 | 0.052 | - | - |
| 3425 | ENSP00000222122 | DBP | 7.578E-05 | 0.052 | - | - |
| 3426 | ENSP00000354006 | TRPM6 | 4.191E-05 | 0.051 | - | - |
| 3427 | ENSP00000311733 | CMKLR1 | 4.127E-05 | 0.051 | - | - |
| 3428 | ENSP00000286760 | WHAMM | 4.061E-05 | 0.051 | - | - |
| 3429 | ENSP00000311496 | 10-Mar | 3.888E-04 | 0.051 | - | - |
| 3430 | ENSP00000395848 | FAM90A26 | 1.933E-05 | 0.050 | - | - |
| 3431 | ENSP00000378702 | BRD2 | 4.609E-05 | 0.050 | - | - |
| 3432 | ENSP00000452773 | C15orf57 | 4.756E-05 | 0.050 | - | - |
| 3433 | ENSP00000345708 | KCNJ11 | 7.231E-05 | 0.050 | - | - |
| 3434 | ENSP00000233813 | IGFBP5 | 8.086E-05 | 0.050 | - | - |
| 3435 | ENSP00000375053 | TMEM203 | 4.953E-05 | 0.050 | - | - |
| 3436 | ENSP00000359795 | GIPC2 | 3.234E-05 | 0.050 | - | - |
| 3437 | ENSP00000380494 | MYCL1 | 5.409E-05 | 0.050 | - | - |
| 3438 | ENSP00000472450 | BAGE5 | 2.798E-05 | 0.049 | - | - |
| 3439 | ENSP00000372155 | PSME1 | 5.552E-05 | 0.049 | - | - |
| 3440 | ENSP00000335285 | C1QL4 | 5.218E-05 | 0.049 | - | - |
| 3441 | ENSP00000302548 | HOXD4 | 3.108E-05 | 0.049 | - | - |
| 3442 | ENSP00000344071 | METTL24 | 7.797E-05 | 0.048 | - | - |
| 3443 | ENSP00000362409 | FAM129B | 8.582E-05 | 0.048 | - | - |
| 3444 | ENSP00000369858 | ALOX5AP | 7.195E-05 | 0.048 | - | - |
| 3445 | ENSP00000420826 | PRB1 | 8.021E-05 | 0.048 | - | - |
| 3446 | ENSP00000454411 | OST4 | 2.491E-05 | 0.048 | - | - |
| 3447 | ENSP00000431198 | RP11-585F1.10 | 2.545E-05 | 0.048 | - | - |
| 3448 | ENSP00000158166 | CAMKK1 | 2.682E-05 | 0.048 | - | - |
| 3449 | ENSP00000354458 | C8A | 3.189E-05 | 0.047 | - | - |
| 3450 | ENSP00000171757 | P2RY10 | 3.041E-05 | 0.047 | - | - |
| 3451 | ENSP00000362403 | TACR2 | 5.130E-05 | 0.046 | - | - |
| 3452 | ENSP00000349669 | C5orf48 | 4.066E-05 | 0.046 | - | - |
| 3453 | ENSP00000354774 | SPRYD7 | 7.247E-05 | 0.046 | - | - |
| 3454 | ENSP00000376177 | CALCRL | 3.648E-05 | 0.046 | - | - |
| 3455 | ENSP00000373702 | PP2D1 | 2.040E-05 | 0.045 | - | - |
| 3456 | ENSP00000454380 | FAM173A | 5.026E-05 | 0.045 | - | - |
| 3457 | ENSP00000381698 | SHARPIN | 6.539E-05 | 0.045 | - | - |
| 3458 | ENSP00000259456 | HEMGN | 5.072E-05 | 0.045 | - | - |
| 3459 | ENSP00000359864 | GPC4 | 4.781E-05 | 0.044 | - | - |
| 3460 | ENSP00000262545 | PCSK2 | 2.138E-05 | 0.044 | - | - |
| 3461 | ENSP00000378312 | EFCAB5 | 2.839E-05 | 0.044 | - | - |
| 3462 | ENSP00000288955 | GRHL3 | 2.919E-05 | 0.044 | - | - |
| 3463 | ENSP00000310111 | HSPB7 | 3.216E-05 | 0.043 | - | - |
| 3464 | ENSP00000296862 | GPR111 | 4.036E-05 | 0.043 | - | - |
| 3465 | ENSP00000418667 | RGAG4 | 3.588E-05 | 0.042 | - | - |
| 3466 | ENSP00000246112 | TLE6 | 6.407E-05 | 0.042 | - | - |
| 3467 | ENSP00000308976 | VWC2L | 3.793E-05 | 0.042 | - | - |
| 3468 | ENSP00000341368 | C6orf132 | 2.932E-05 | 0.042 | - | - |
| 3469 | ENSP00000390849 | ABHD5 | 7.280E-05 | 0.042 | - | - |
| 3470 | ENSP00000340864 | C1QTNF1 | 4.917E-05 | 0.042 | - | - |
| 3471 | ENSP00000297512 | ASIC3 | 2.701E-05 | 0.042 | - | - |
| 3472 | ENSP00000406490 | PMS1 | 5.339E-05 | 0.041 | - | - |
| 3473 | ENSP00000468308 | FAM20A | 5.745E-05 | 0.041 | - | - |
| 3474 | ENSP00000296051 | HPS3 | 3.216E-05 | 0.041 | - | - |
| 3475 | ENSP00000241808 | PRM2 | 3.123E-05 | 0.041 | - | - |
| 3476 | ENSP00000350005 | GIP | 6.373E-05 | 0.041 | - | - |
| 3477 | ENSP00000370125 | HMGN1 | 5.586E-05 | 0.040 | - | - |
| 3478 | ENSP00000465676 | PIAS2 | 7.142E-05 | 0.040 | - | - |
| 3479 | ENSP00000451792 | GALT | 1.021E-04 | 0.040 | - | - |
| 3480 | ENSP00000361141 | OPN4 | 2.828E-05 | 0.039 | - | - |
| 3481 | ENSP00000352425 | WASF1 | 4.881E-05 | 0.039 | - | - |
| 3482 | ENSP00000393596 | BIRC6 | 5.876E-05 | 0.038 | - | - |
| 3483 | ENSP00000215906 | ASPHD2 | 4.798E-05 | 0.038 | - | - |
| 3484 | ENSP00000217270 | PROKR2 | 6.331E-05 | 0.038 | - | - |
| 3485 | ENSP00000394049 | SLA | 4.042E-05 | 0.038 | - | - |
| 3486 | ENSP00000401566 | ECD | 5.999E-05 | 0.037 | - | - |
| 3487 | ENSP00000306407 | DBNDD1 | 5.539E-05 | 0.037 | - | - |
| 3488 | ENSP00000297307 | SLC35G3 | 8.469E-05 | 0.036 | - | - |
| 3489 | ENSP00000338799 | IL6ST | 2.125E-04 | 0.036 | - | - |
| 3490 | ENSP00000300055 | PLIN1 | 3.302E-05 | 0.035 | - | - |
| 3491 | ENSP00000419266 | EVX1 | 2.758E-05 | 0.035 | - | - |
| 3492 | ENSP00000219334 | SMPD3 | 4.006E-05 | 0.035 | - | - |
| 3493 | ENSP00000328708 | RXFP3 | 8.163E-05 | 0.034 | - | - |
| 3494 | ENSP00000365926 | RFK | 3.764E-05 | 0.034 | - | - |
| 3495 | ENSP00000387564 | HMCN2 | 2.940E-05 | 0.033 | - | - |
| 3496 | ENSP00000351318 | PLEKHG1 | 4.879E-05 | 0.033 | - | - |
| 3497 | ENSP00000290866 | ACE | 6.187E-05 | 0.032 | - | - |
| 3498 | ENSP00000264433 | FNIP2 | 3.523E-05 | 0.032 | - | - |
| 3499 | ENSP00000405676 | RP11-565P22.6 | 1.927E-05 | 0.032 | - | - |
| 3500 | ENSP00000229304 | APOBEC1 | 3.162E-05 | 0.032 | - | - |
| 3501 | ENSP00000384889 | HIC2 | 8.990E-05 | 0.032 | - | - |
| 3502 | ENSP00000257836 | PRRG4 | 5.090E-05 | 0.032 | - | - |
| 3503 | ENSP00000245810 | PSPN | 5.496E-05 | 0.032 | - | - |
| 3504 | ENSP00000322323 | FAM20C | 2.552E-05 | 0.031 | - | - |
| 3505 | ENSP00000264162 | LCT | 2.563E-05 | 0.031 | - | - |
| 3506 | ENSP00000400365 | LAMA2 | 4.638E-05 | 0.031 | - | - |
| 3507 | ENSP00000263577 | CDON | 2.389E-05 | 0.031 | - | - |
| 3508 | ENSP00000356791 | DPT | 6.055E-05 | 0.031 | - | - |
| 3509 | ENSP00000343115 | FAM222B | 5.269E-03 | 0.031 | - | - |
| 3510 | ENSP00000266534 | TMEM117 | 8.014E-05 | 0.031 | - | - |
| 3511 | ENSP00000349853 | - | 5.732E-05 | 0.030 | - | - |
| 3512 | ENSP00000356990 | KLHDC9 | 3.618E-05 | 0.029 | - | - |
| 3513 | ENSP00000246532 | IGFLR1 | 4.182E-05 | 0.029 | - | - |
| 3514 | ENSP00000300060 | ANPEP | 6.620E-05 | 0.029 | - | - |
| 3515 | ENSP00000391879 | TRIM26 | 6.178E-05 | 0.029 | - | - |
| 3516 | ENSP00000347919 | COA4 | 5.179E-05 | 0.028 | - | - |
| 3517 | ENSP00000318128 | BLOC1S4 | 3.704E-05 | 0.027 | - | - |
| 3518 | ENSP00000374237 | NXPE2 | 2.905E-05 | 0.027 | - | - |
| 3519 | ENSP00000367198 | LECT1 | 3.269E-05 | 0.026 | - | - |
| 3520 | ENSP00000247194 | L3HYPDH | 8.083E-05 | 0.026 | - | - |
| 3521 | ENSP00000408631 | CIDEC | 5.747E-05 | 0.026 | - | - |
| 3522 | ENSP00000254351 | SDC1 | 9.867E-05 | 0.026 | - | - |
| 3523 | ENSP00000358391 | FCGR1B | 4.114E-05 | 0.025 | - | - |
| 3524 | ENSP00000251203 | PBX4 | 4.244E-05 | 0.025 | - | - |
| 3525 | ENSP00000302413 | TPST1 | 4.030E-05 | 0.025 | - | - |
| 3526 | ENSP00000338228 | C5orf56 | 6.264E-05 | 0.025 | - | - |
| 3527 | ENSP00000246515 | SLURP1 | 2.820E-05 | 0.025 | - | - |
| 3528 | ENSP00000380271 | RAPGEF4 | 5.373E-05 | 0.024 | - | - |
| 3529 | ENSP00000329303 | - | 1.001E-04 | 0.024 | - | - |
| 3530 | ENSP00000384625 | NLK | 8.510E-03 | 0.024 | - | - |
| 3531 | ENSP00000383516 | HMX1 | 4.293E-05 | 0.023 | - | - |
| 3532 | ENSP00000354849 | MYBPC1 | 2.795E-05 | 0.023 | - | - |
| 3533 | ENSP00000321239 | RCHY1 | 1.305E-04 | 0.023 | - | - |
| 3534 | ENSP00000415823 | IGFL1 | 3.991E-05 | 0.023 | - | - |
| 3535 | ENSP00000356958 | NR1I3 | 4.366E-05 | 0.022 | - | - |
| 3536 | ENSP00000331544 | FBLN1 | 4.036E-05 | 0.022 | - | - |
| 3537 | ENSP00000323795 | TEX35 | 1.245E-04 | 0.022 | - | - |
| 3538 | ENSP00000332389 | BRICD5 | 5.025E-05 | 0.022 | - | - |
| 3539 | ENSP00000455906 | MUC22 | 1.421E-05 | 0.021 | - | - |
| 3540 | ENSP00000299766 | MC4R | 4.686E-05 | 0.021 | - | - |
| 3541 | ENSP00000392423 | RELN | 4.308E-05 | 0.021 | - | - |
| 3542 | ENSP00000345968 | PGLYRP2 | 3.703E-05 | 0.021 | - | - |
| 3543 | ENSP00000450742 | ZFYVE1 | 5.235E-05 | 0.021 | - | - |
| 3544 | ENSP00000329942 | CD300E | 3.341E-05 | 0.021 | - | - |
| 3545 | ENSP00000365806 | GNL1 | 5.235E-05 | 0.020 | - | - |
| 3546 | ENSP00000269919 | PGPEP1 | 6.710E-05 | 0.020 | - | - |
| 3547 | ENSP00000290200 | IL10RB | 2.411E-04 | 0.020 | - | - |
| 3548 | ENSP00000359899 | HCRTR2 | 5.109E-05 | 0.019 | - | - |
| 3549 | ENSP00000357130 | SPTA1 | 5.788E-05 | 0.019 | - | - |
| 3550 | ENSP00000272369 | MEIS1 | 2.586E-05 | 0.019 | - | - |
| 3551 | ENSP00000468772 | GPS2 | 5.432E-05 | 0.019 | - | - |
| 3552 | ENSP00000357283 | LMNA | 7.010E-05 | 0.018 | - | - |
| 3553 | ENSP00000284562 | GSTA5 | 3.213E-05 | 0.018 | - | - |
| 3554 | ENSP00000361355 | TMEM254 | 4.415E-05 | 0.018 | - | - |
| 3555 | ENSP00000408236 | CYTH2 | 4.776E-05 | 0.016 | - | - |
| 3556 | ENSP00000262352 | SLC1A1 | 2.232E-05 | 0.016 | - | - |
| 3557 | ENSP00000379651 | LILRA6 | 9.189E-06 | 0.016 | - | - |
| 3558 | ENSP00000355537 | ACTN2 | 5.903E-05 | 0.016 | - | - |
| 3559 | ENSP00000318753 | WFDC11 | 6.051E-05 | 0.015 | - | - |
| 3560 | ENSP00000362398 | LPAR4 | 2.919E-05 | 0.015 | - | - |
| 3561 | ENSP00000264956 | EVC | 3.416E-05 | 0.014 | - | - |
| 3562 | ENSP00000270139 | IFNAR1 | 2.675E-04 | 0.014 | - | - |
| 3563 | ENSP00000465432 | C19orf53 | 6.094E-05 | 0.014 | - | - |
| 3564 | ENSP00000363873 | OXLD1 | 1.996E-05 | 0.014 | - | - |
| 3565 | ENSP00000244513 | BTN1A1 | 2.634E-05 | 0.014 | - | - |
| 3566 | ENSP00000228918 | LTBR | 2.211E-04 | 0.013 | - | - |
| 3567 | ENSP00000352177 | ADAM29 | 5.459E-05 | 0.013 | - | - |
| 3568 | ENSP00000294485 | DRAXIN | 1.190E-04 | 0.013 | - | - |
| 3569 | ENSP00000359571 | CXorf66 | 3.645E-05 | 0.013 | - | - |
| 3570 | ENSP00000300187 | MS4A14 | 4.247E-05 | 0.013 | - | - |
| 3571 | ENSP00000447149 | NR1H4 | 4.412E-05 | 0.012 | - | - |
| 3572 | ENSP00000366267 | GPC5 | 6.840E-05 | 0.012 | - | - |
| 3573 | ENSP00000256119 | CA1 | 1.865E-05 | 0.012 | - | - |
| 3574 | ENSP00000347748 | TPRA1 | 3.880E-05 | 0.012 | - | - |
| 3575 | ENSP00000318480 | YME1L1 | 6.878E-05 | 0.011 | - | - |
| 3576 | ENSP00000318437 | FDCSP | 4.659E-05 | 0.011 | - | - |
| 3577 | ENSP00000355110 | SMOC1 | 5.504E-05 | 0.011 | - | - |
| 3578 | ENSP00000289575 | SLCO2B1 | 3.330E-05 | 0.011 | - | - |
| 3579 | ENSP00000428603 | PCDHGB6 | 2.102E-05 | 0.011 | - | - |
| 3580 | ENSP00000367123 | SLC3A2 | 5.830E-05 | 0.011 | - | - |
| 3581 | ENSP00000355119 | TGIF2LX | 4.661E-05 | 0.011 | - | - |
| 3582 | ENSP00000340913 | TRPC6 | 2.920E-05 | 0.010 | - | - |
| 3583 | ENSP00000339250 | DPPA3 | 4.741E-05 | 0.010 | - | - |
| 3584 | ENSP00000471397 | GBA3 | 3.857E-05 | 0.010 | - | - |
| 3585 | ENSP00000308107 | HPSE | 4.236E-05 | 0.010 | - | - |
| 3586 | ENSP00000375063 | NLRP5 | 5.979E-05 | 0.010 | - | - |
| 3587 | ENSP00000307519 | TMPRSS11E | 3.773E-05 | 0.009 | - | - |
| 3588 | ENSP00000365175 | PRRC2A | 5.106E-05 | 0.009 | - | - |
| 3589 | ENSP00000289373 | TMSB15A | 4.858E-05 | 0.009 | - | - |
| 3590 | ENSP00000303575 | NUDT9 | 3.425E-05 | 0.009 | - | - |
| 3591 | ENSP00000429018 | PCDHGB2 | 1.924E-05 | 0.008 | - | - |
| 3592 | ENSP00000343782 | ADRB3 | 4.108E-05 | 0.008 | - | - |
| 3593 | ENSP00000377948 | QRFPR | 3.191E-05 | 0.008 | - | - |
| 3594 | ENSP00000262593 | DOK5 | 4.819E-05 | 0.008 | - | - |
| 3595 | ENSP00000265417 | GPR116 | 3.782E-05 | 0.008 | - | - |
| 3596 | ENSP00000306697 | MRAP | 1.077E-04 | 0.008 | - | - |
| 3597 | ENSP00000377372 | GAP43 | 5.810E-05 | 0.007 | - | - |
| 3598 | ENSP00000343676 | GJA4 | 3.698E-05 | 0.007 | - | - |
| 3599 | ENSP00000362092 | RRAGC | 4.825E-05 | 0.007 | - | - |
| 3600 | ENSP00000296468 | MFSD8 | 4.625E-05 | 0.007 | - | - |
| 3601 | ENSP00000262424 | CRISPLD2 | 3.866E-05 | 0.007 | - | - |
| 3602 | ENSP00000253801 | G6PC | 4.405E-05 | 0.006 | - | - |
| 3603 | ENSP00000441685 | PDZD9 | 2.545E-06 | 0.006 | - | - |
| 3604 | ENSP00000415528 | CCDC81 | 9.024E-08 | 0.006 | - | - |
| 3605 | ENSP00000357631 | TCERG1L | 3.659E-05 | 0.006 | - | - |
| 3606 | ENSP00000409316 | OR4F29 | 8.000E-07 | 0.006 | - | - |
| 3607 | ENSP00000340811 | GJB5 | 3.749E-05 | 0.006 | - | - |
| 3608 | ENSP00000346829 | WLS | 3.811E-05 | 0.006 | - | - |
| 3609 | ENSP00000256103 | PMP2 | 3.811E-05 | 0.005 | - | - |
| 3610 | ENSP00000360360 | CT47B1 | 3.476E-05 | 0.005 | - | - |
| 3611 | ENSP00000394624 | OPRM1 | 1.069E-04 | 0.005 | - | - |
| 3612 | ENSP00000339428 | SOCS2 | 1.780E-04 | 0.005 | - | - |
| 3613 | ENSP00000460274 | PCDHGA9 | 1.935E-05 | 0.005 | - | - |
| 3614 | ENSP00000342082 | SLPI | 3.114E-04 | 0.004 | - | - |
| 3615 | ENSP00000312385 | EVX2 | 2.794E-05 | 0.004 | - | - |
| 3616 | ENSP00000377047 | PTPRZ1 | 4.099E-05 | 0.004 | - | - |
| 3617 | ENSP00000334186 | VWCE | 3.563E-05 | 0.004 | - | - |
| 3618 | ENSP00000263372 | KCNK6 | 3.966E-05 | 0.004 | - | - |
| 3619 | ENSP00000347988 | NDUFA5 | 4.298E-05 | 0.004 | - | - |
| 3620 | ENSP00000371824 | C9orf66 | 2.247E-05 | 0.003 | - | - |
| 3621 | ENSP00000395359 | CADM1 | 6.086E-05 | 0.003 | - | - |
| 3622 | ENSP00000349132 | SPATA31A3 | 6.416E-05 | 0.003 | - | - |
| 3623 | ENSP00000262207 | CRISPLD1 | 3.974E-05 | 0.003 | - | - |
| 3624 | ENSP00000351052 | ZC3HC1 | 8.287E-05 | 0.003 | - | - |
| 3625 | ENSP00000266542 | C1RL | 5.743E-05 | 0.003 | - | - |
| 3626 | ENSP00000419457 | NOBOX | 5.443E-05 | 0.003 | - | - |
| 3627 | ENSP00000342557 | IL4I1 | 3.825E-05 | 0.003 | - | - |
| 3628 | ENSP00000393541 | IFT27 | 3.794E-05 | 0.002 | - | - |
| 3629 | ENSP00000292427 | CYP11B1 | 3.313E-05 | 0.002 | - | - |
| 3630 | ENSP00000417659 | PPM1L | 3.705E-05 | 0.002 | - | - |
| 3631 | ENSP00000349893 | DLK2 | 3.409E-05 | 0.002 | - | - |
| 3632 | ENSP00000334330 | DEFB105A | 2.405E-05 | 0.002 | - | - |
| 3633 | ENSP00000364650 | LYZL1 | 3.773E-05 | 0.002 | - | - |
| 3634 | ENSP00000305056 | PCSK6 | 5.142E-05 | 0.002 | - | - |
| 3635 | ENSP00000405575 | CHMP3 | 4.628E-05 | 0.002 | - | - |
| 3636 | ENSP00000369895 | GEMIN8 | 4.390E-05 | 0.002 | - | - |
| 3637 | ENSP00000308925 | TLR10 | 6.970E-05 | 0.001 | - | - |
| 3638 | ENSP00000285381 | CA3 | 3.564E-05 | 0.001 | - | - |
| 3639 | ENSP00000363683 | MDS2 | 5.857E-05 | 0.001 | - | - |
| 3640 | ENSP00000453364 | HCG27 | 2.099E-05 | 0.001 | - | - |
| 3641 | ENSP00000379092 | BTC | 4.088E-05 | 0.001 | - | - |
| 3642 | ENSP00000384442 | CDK11A | 7.164E-05 | 0.001 | - | - |
| 3643 | ENSP00000259953 | NRM | 3.053E-05 | 0.000 | - | - |
| 3644 | ENSP00000355799 | GPR31 | 5.747E-05 | 0.000 | - | - |
| 3645 | ENSP00000391536 | AC021860.1 | 2.385E-05 | 0.000 | - | - |
| 3646 | ENSP00000262999 | UCP1 | 3.045E-05 | -0.001 | - | - |
| 3647 | ENSP00000364476 | RIBC1 | 2.691E-05 | -0.001 | - | - |
| 3648 | ENSP00000229794 | MAPK14 | 4.526E-05 | -0.001 | - | - |
| 3649 | ENSP00000419765 | BRD9 | 1.162E-04 | -0.001 | - | - |
| 3650 | ENSP00000407375 | GPX1 | 4.226E-05 | -0.001 | - | - |
| 3651 | ENSP00000263932 | TNFRSF8 | 2.165E-04 | -0.001 | - | - |
| 3652 | ENSP00000353157 | 2-Sep | 6.258E-05 | -0.002 | - | - |
| 3653 | ENSP00000381607 | GSTP1 | 2.075E-03 | -0.002 | - | - |
| 3654 | ENSP00000344223 | MBTPS1 | 8.482E-05 | -0.002 | - | - |
| 3655 | ENSP00000345393 | MAFF | 1.017E-04 | -0.002 | - | - |
| 3656 | ENSP00000240874 | KALRN | 2.766E-05 | -0.003 | - | - |
| 3657 | ENSP00000362401 | CYSLTR1 | 5.689E-05 | -0.003 | - | - |
| 3658 | ENSP00000409898 | NLRP11 | 6.887E-05 | -0.003 | - | - |
| 3659 | ENSP00000420727 | ZNF181 | 5.141E-05 | -0.003 | - | - |
| 3660 | ENSP00000366124 | CST3 | 1.271E-04 | -0.003 | - | - |
| 3661 | ENSP00000399970 | PPP2R2D | 4.330E-05 | -0.003 | - | - |
| 3662 | ENSP00000396918 | URGCP | 6.835E-05 | -0.003 | - | - |
| 3663 | ENSP00000465194 | IER3IP1 | 2.899E-05 | -0.004 | - | - |
| 3664 | ENSP00000219197 | CBLN1 | 3.990E-05 | -0.004 | - | - |
| 3665 | ENSP00000348107 | C1D | 4.136E-05 | -0.004 | - | - |
| 3666 | ENSP00000311005 | MAPK7 | 8.527E-05 | -0.004 | - | - |
| 3667 | ENSP00000332530 | RNLS | 4.657E-05 | -0.005 | - | - |
| 3668 | ENSP00000365775 | MTHFR | 5.191E-05 | -0.006 | - | - |
| 3669 | ENSP00000221978 | NKG7 | 8.544E-05 | -0.006 | - | - |
| 3670 | ENSP00000365924 | TRIM31 | 6.182E-05 | -0.007 | - | - |
| 3671 | ENSP00000337732 | RUNDC3B | 3.636E-05 | -0.007 | - | - |
| 3672 | ENSP00000295824 | EFHB | 2.912E-05 | -0.007 | - | - |
| 3673 | ENSP00000354707 | OR10Z1 | 2.050E-05 | -0.007 | - | - |
| 3674 | ENSP00000383900 | LIPK | 4.462E-05 | -0.008 | - | - |
| 3675 | ENSP00000313309 | FUZ | 2.537E-05 | -0.008 | - | - |
| 3676 | ENSP00000357736 | LELP1 | 2.870E-05 | -0.008 | - | - |
| 3677 | ENSP00000156499 | KLK14 | 3.466E-05 | -0.008 | - | - |
| 3678 | ENSP00000392812 | PIK3R5 | 6.752E-05 | -0.008 | - | - |
| 3679 | ENSP00000258042 | NMBR | 5.380E-05 | -0.008 | - | - |
| 3680 | ENSP00000382356 | COL28A1 | 1.650E-05 | -0.008 | - | - |
| 3681 | ENSP00000222248 | SLC5A5 | 1.779E-05 | -0.008 | - | - |
| 3682 | ENSP00000263645 | CD81 | 1.144E-04 | -0.009 | - | - |
| 3683 | ENSP00000300692 | CD3D | 1.972E-04 | -0.009 | - | - |
| 3684 | ENSP00000354901 | CXCL9 | 2.377E-04 | -0.009 | - | - |
| 3685 | ENSP00000276173 | RIPPLY1 | 2.864E-05 | -0.009 | - | - |
| 3686 | ENSP00000416797 | CAMSAP3 | 3.989E-05 | -0.009 | - | - |
| 3687 | ENSP00000371932 | TAS2R1 | 6.745E-05 | -0.009 | - | - |
| 3688 | ENSP00000280606 | PRSS53 | 4.446E-05 | -0.010 | - | - |
| 3689 | ENSP00000261023 | ITGAV | 1.181E-04 | -0.010 | - | - |
| 3690 | ENSP00000355157 | FCRL2 | 3.139E-05 | -0.010 | - | - |
| 3691 | ENSP00000264265 | LXN | 3.106E-05 | -0.011 | - | - |
| 3692 | ENSP00000299178 | AVPR1A | 2.832E-05 | -0.011 | - | - |
| 3693 | ENSP00000381711 | AL162389.1 | 2.323E-05 | -0.011 | - | - |
| 3694 | ENSP00000299505 | KIAA0355 | 1.351E-04 | -0.011 | - | - |
| 3695 | ENSP00000331411 | FBLN7 | 4.273E-05 | -0.012 | - | - |
| 3696 | ENSP00000419901 | L1TD1 | 5.072E-05 | -0.012 | - | - |
| 3697 | ENSP00000367965 | SOWAHA | 3.930E-05 | -0.012 | - | - |
| 3698 | ENSP00000388550 | RNF24 | 4.680E-05 | -0.012 | - | - |
| 3699 | ENSP00000378089 | NMB | 2.891E-05 | -0.012 | - | - |
| 3700 | ENSP00000349114 | C7orf76 | 4.316E-05 | -0.012 | - | - |
| 3701 | ENSP00000215838 | TCN2 | 3.103E-05 | -0.012 | - | - |
| 3702 | ENSP00000357794 | TCHH | 5.052E-05 | -0.013 | - | - |
| 3703 | ENSP00000224764 | - | 8.870E-05 | -0.013 | - | - |
| 3704 | ENSP00000380334 | NCF4 | 8.481E-05 | -0.013 | - | - |
| 3705 | ENSP00000217961 | STS | 2.246E-05 | -0.013 | - | - |
| 3706 | ENSP00000329454 | RCAN2 | 3.340E-05 | -0.014 | - | - |
| 3707 | ENSP00000415786 | SERPINE2 | 1.281E-04 | -0.014 | - | - |
| 3708 | ENSP00000431287 | METTL12 | 2.265E-05 | -0.014 | - | - |
| 3709 | ENSP00000299340 | CYYR1 | 4.719E-05 | -0.014 | - | - |
| 3710 | ENSP00000264977 | PPP2R3A | 3.054E-05 | -0.014 | - | - |
| 3711 | ENSP00000390423 | CATSPER4 | 1.865E-05 | -0.014 | - | - |
| 3712 | ENSP00000290575 | - | 1.704E-04 | -0.014 | - | - |
| 3713 | ENSP00000232014 | BCL6 | 4.984E-05 | -0.014 | - | - |
| 3714 | ENSP00000257215 | DAGLA | 3.078E-05 | -0.015 | - | - |
| 3715 | ENSP00000365663 | NPPA | 4.973E-05 | -0.015 | - | - |
| 3716 | ENSP00000351884 | SPANXC | 6.654E-05 | -0.015 | - | - |
| 3717 | ENSP00000403609 | - | 8.862E-07 | -0.015 | - | - |
| 3718 | ENSP00000257290 | PDGFRA | 4.105E-05 | -0.015 | - | - |
| 3719 | ENSP00000354130 | SOX10 | 4.405E-05 | -0.015 | - | - |
| 3720 | ENSP00000276414 | GNRH1 | 2.902E-05 | -0.016 | - | - |
| 3721 | ENSP00000457511 | CCDC179 | 2.554E-06 | -0.016 | - | - |
| 3722 | ENSP00000349016 | PEX14 | 5.246E-05 | -0.017 | - | - |
| 3723 | ENSP00000354511 | COMT | 4.352E-05 | -0.017 | - | - |
| 3724 | ENSP00000363395 | ANXA8L2 | 3.115E-05 | -0.017 | - | - |
| 3725 | ENSP00000085219 | CD22 | 1.566E-04 | -0.017 | - | - |
| 3726 | ENSP00000301452 | ACER1 | 3.549E-05 | -0.017 | - | - |
| 3727 | ENSP00000358541 | SIKE1 | 4.023E-05 | -0.017 | - | - |
| 3728 | ENSP00000359998 | GSTA4 | 2.247E-05 | -0.018 | - | - |
| 3729 | ENSP00000364822 | RP11-410N8.4 | 2.322E-06 | -0.018 | - | - |
| 3730 | ENSP00000326391 | BHLHA15 | 3.851E-05 | -0.018 | - | - |
| 3731 | ENSP00000298861 | AGBL2 | 1.395E-04 | -0.019 | - | - |
| 3732 | ENSP00000303775 | PROKR1 | 5.570E-05 | -0.019 | - | - |
| 3733 | ENSP00000329548 | TUSC5 | 8.268E-05 | -0.019 | - | - |
| 3734 | ENSP00000268271 | - | 4.205E-05 | -0.019 | - | - |
| 3735 | ENSP00000387946 | NR1H3 | 4.747E-05 | -0.019 | - | - |
| 3736 | ENSP00000253435 | TEX101 | 4.241E-05 | -0.020 | - | - |
| 3737 | ENSP00000272907 | NYAP2 | 9.183E-05 | -0.021 | - | - |
| 3738 | ENSP00000400312 | ULK3 | 4.133E-05 | -0.021 | - | - |
| 3739 | ENSP00000293525 | KRT86 | 5.529E-05 | -0.022 | - | - |
| 3740 | ENSP00000287585 | LHFPL4 | 1.800E-04 | -0.022 | - | - |
| 3741 | ENSP00000362817 | FABP3 | 3.099E-05 | -0.022 | - | - |
| 3742 | ENSP00000380193 | NRL | 7.373E-05 | -0.022 | - | - |
| 3743 | ENSP00000336762 | ANG | 4.486E-05 | -0.022 | - | - |
| 3744 | ENSP00000348564 | CDAN1 | 3.089E-05 | -0.022 | - | - |
| 3745 | ENSP00000345001 | CRTC1 | 2.970E-05 | -0.022 | - | - |
| 3746 | ENSP00000262418 | SLC4A1 | 2.685E-05 | -0.023 | - | - |
| 3747 | ENSP00000373300 | METTL6 | 5.869E-05 | -0.024 | - | - |
| 3748 | ENSP00000443246 | BCKDHA | 3.410E-05 | -0.024 | - | - |
| 3749 | ENSP00000419417 | C1orf52 | 3.160E-05 | -0.024 | - | - |
| 3750 | ENSP00000278780 | OVOL2 | 5.166E-05 | -0.024 | - | - |
| 3751 | ENSP00000362399 | STXBP1 | 6.169E-05 | -0.024 | - | - |
| 3752 | ENSP00000424598 | DEFB4B | 3.691E-06 | -0.025 | - | - |
| 3753 | ENSP00000309270 | CHST1 | 3.295E-05 | -0.025 | - | - |
| 3754 | ENSP00000412130 | MRVI1 | 3.233E-05 | -0.026 | - | - |
| 3755 | ENSP00000215909 | LGALS1 | 1.130E-04 | -0.026 | - | - |
| 3756 | ENSP00000410400 | CT47A11 | 1.049E-05 | -0.026 | - | - |
| 3757 | ENSP00000362001 | C20orf62 | 2.071E-05 | -0.027 | - | - |
| 3758 | ENSP00000260408 | ADAM10 | 1.004E-04 | -0.027 | - | - |
| 3759 | ENSP00000254325 | RFX1 | 2.236E-05 | -0.027 | - | - |
| 3760 | ENSP00000226193 | RCVRN | 2.028E-05 | -0.027 | - | - |
| 3761 | ENSP00000357158 | FCRL1 | 4.964E-05 | -0.027 | - | - |
| 3762 | ENSP00000374501 | UVSSA | 6.003E-05 | -0.027 | - | - |
| 3763 | ENSP00000385739 | CEACAM21 | 2.557E-05 | -0.027 | - | - |
| 3764 | ENSP00000345470 | CCDC38 | 1.106E-04 | -0.027 | - | - |
| 3765 | ENSP00000284617 | CCSAP | 3.798E-05 | -0.027 | - | - |
| 3766 | ENSP00000354859 | DRD2 | 6.820E-05 | -0.028 | - | - |
| 3767 | ENSP00000241256 | GHSR | 3.945E-05 | -0.028 | - | - |
| 3768 | ENSP00000233840 | NEU2 | 2.636E-05 | -0.028 | - | - |
| 3769 | ENSP00000381494 | KRTAP2-2 | 3.262E-05 | -0.028 | - | - |
| 3770 | ENSP00000227155 | CD82 | 8.621E-05 | -0.028 | - | - |
| 3771 | ENSP00000160740 | CIC | 5.852E-05 | -0.028 | - | - |
| 3772 | ENSP00000290472 | PLA2G4D | 3.144E-05 | -0.028 | - | - |
| 3773 | ENSP00000278200 | IMMP1L | 2.069E-05 | -0.029 | - | - |
| 3774 | ENSP00000415904 | OAZ3 | 2.946E-05 | -0.029 | - | - |
| 3775 | ENSP00000300433 | TMEM92 | 1.084E-04 | -0.029 | - | - |
| 3776 | ENSP00000334650 | KCNK18 | 3.476E-05 | -0.030 | - | - |
| 3777 | ENSP00000422168 | SH3BP2 | 4.268E-05 | -0.030 | - | - |
| 3778 | ENSP00000429273 | PCDHGB1 | 1.907E-05 | -0.030 | - | - |
| 3779 | ENSP00000349873 | HIST1H2AL | 1.062E-04 | -0.030 | - | - |
| 3780 | ENSP00000357234 | TSACC | 4.313E-05 | -0.030 | - | - |
| 3781 | ENSP00000221421 | LHB | 3.220E-05 | -0.031 | - | - |
| 3782 | ENSP00000324191 | KCNRG | 1.043E-04 | -0.031 | - | - |
| 3783 | ENSP00000371127 | GAGE2C | 1.982E-06 | -0.031 | - | - |
| 3784 | ENSP00000297423 | KIAA0146 | 2.129E-04 | -0.031 | - | - |
| 3785 | ENSP00000353731 | DPP4 | 9.298E-05 | -0.031 | - | - |
| 3786 | ENSP00000449999 | CLEC5A | 4.574E-05 | -0.031 | - | - |
| 3787 | ENSP00000338191 | SNTB2 | 2.910E-05 | -0.031 | - | - |
| 3788 | ENSP00000226230 | TMEM97 | 7.373E-05 | -0.031 | - | - |
| 3789 | ENSP00000168148 | SPP2 | 6.690E-05 | -0.031 | - | - |
| 3790 | ENSP00000334665 | FSCN2 | 4.544E-05 | -0.031 | - | - |
| 3791 | ENSP00000308782 | GP6 | 2.533E-05 | -0.031 | - | - |
| 3792 | ENSP00000259392 | SLC31A2 | 4.389E-05 | -0.032 | - | - |
| 3793 | ENSP00000332823 | NR2C2AP | 4.031E-05 | -0.032 | - | - |
| 3794 | ENSP00000345972 | C1orf168 | 4.130E-05 | -0.032 | - | - |
| 3795 | ENSP00000311528 | GPR162 | 6.116E-05 | -0.032 | - | - |
| 3796 | ENSP00000360269 | TACSTD2 | 2.352E-05 | -0.033 | - | - |
| 3797 | ENSP00000246081 | OTOR | 6.312E-05 | -0.033 | - | - |
| 3798 | ENSP00000268171 | FURIN | 6.523E-05 | -0.034 | - | - |
| 3799 | ENSP00000019103 | SCTR | 2.260E-05 | -0.034 | - | - |
| 3800 | ENSP00000376758 | PTRH2 | 5.111E-05 | -0.034 | - | - |
| 3801 | ENSP00000417581 | FAM199X | 6.192E-07 | -0.034 | - | - |
| 3802 | ENSP00000261797 | NDST1 | 2.780E-05 | -0.034 | - | - |
| 3803 | ENSP00000396774 | MUC20 | 6.973E-05 | -0.035 | - | - |
| 3804 | ENSP00000222902 | CCL24 | 8.814E-04 | -0.035 | - | - |
| 3805 | ENSP00000381115 | C11orf93 | 2.825E-05 | -0.035 | - | - |
| 3806 | ENSP00000366522 | ARHGAP39 | 2.562E-05 | -0.035 | - | - |
| 3807 | ENSP00000352584 | AKR1B10 | 3.720E-05 | -0.035 | - | - |
| 3808 | ENSP00000368966 | TRPC3 | 2.668E-05 | -0.035 | - | - |
| 3809 | ENSP00000379612 | TAX1BP1 | 5.689E-05 | -0.035 | - | - |
| 3810 | ENSP00000360124 | PGM1 | 5.365E-05 | -0.036 | - | - |
| 3811 | ENSP00000257770 | NT5E | 4.609E-05 | -0.036 | - | - |
| 3812 | ENSP00000337340 | MED19 | 4.586E-05 | -0.036 | - | - |
| 3813 | ENSP00000292599 | MAML1 | 3.590E-05 | -0.037 | - | - |
| 3814 | ENSP00000362576 | YARS | 4.920E-05 | -0.037 | - | - |
| 3815 | ENSP00000357342 | HCN3 | 3.087E-05 | -0.037 | - | - |
| 3816 | ENSP00000359512 | GBP3 | 5.067E-05 | -0.037 | - | - |
| 3817 | ENSP00000283905 | C7orf31 | 1.149E-04 | -0.037 | - | - |
| 3818 | ENSP00000341490 | ISM2 | 5.170E-05 | -0.038 | - | - |
| 3819 | ENSP00000353608 | DSC3 | 3.288E-05 | -0.038 | - | - |
| 3820 | ENSP00000365131 | BAG6 | 4.727E-05 | -0.038 | - | - |
| 3821 | ENSP00000252087 | PCDHGC5 | 4.475E-05 | -0.038 | - | - |
| 3822 | ENSP00000278968 | TAGLN | 7.772E-05 | -0.038 | - | - |
| 3823 | ENSP00000375717 | PTOV1 | 3.450E-05 | -0.038 | - | - |
| 3824 | ENSP00000301293 | SEMA6B | 3.284E-05 | -0.039 | - | - |
| 3825 | ENSP00000298047 | FAT3 | 4.360E-05 | -0.039 | - | - |
| 3826 | ENSP00000319705 | DUOXA2 | 3.210E-05 | -0.039 | - | - |
| 3827 | ENSP00000234091 | ID2 | 8.537E-05 | -0.039 | - | - |
| 3828 | ENSP00000414330 | RIMKLA | 2.449E-05 | -0.039 | - | - |
| 3829 | ENSP00000335185 | PRICKLE4 | 3.265E-05 | -0.039 | - | - |
| 3830 | ENSP00000378690 | CALN1 | 3.642E-05 | -0.039 | - | - |
| 3831 | ENSP00000357301 | RXFP4 | 1.402E-04 | -0.039 | - | - |
| 3832 | ENSP00000356056 | DYNLT1 | 6.066E-05 | -0.039 | - | - |
| 3833 | ENSP00000263409 | LIFR | 1.942E-04 | -0.039 | - | - |
| 3834 | ENSP00000357196 | PTPRK | 3.553E-05 | -0.040 | - | - |
| 3835 | ENSP00000355060 | ARHGEF10L | 4.806E-05 | -0.040 | - | - |
| 3836 | ENSP00000289272 | PCDHA13 | 4.192E-05 | -0.040 | - | - |
| 3837 | ENSP00000321106 | TAC1 | 5.488E-05 | -0.040 | - | - |
| 3838 | ENSP00000291554 | CRYAA | 3.042E-05 | -0.040 | - | - |
| 3839 | ENSP00000305416 | S1PR1 | 1.297E-04 | -0.040 | - | - |
| 3840 | ENSP00000348965 | DYNC1H1 | 4.735E-05 | -0.041 | - | - |
| 3841 | ENSP00000258385 | CHRND | 7.837E-05 | -0.041 | - | - |
| 3842 | ENSP00000307822 | NPFFR2 | 2.399E-05 | -0.041 | - | - |
| 3843 | ENSP00000218032 | TLR8 | 9.088E-05 | -0.041 | - | - |
| 3844 | ENSP00000264363 | NDST4 | 3.167E-05 | -0.041 | - | - |
| 3845 | ENSP00000371805 | FBXL18 | 5.297E-05 | -0.041 | - | - |
| 3846 | ENSP00000302935 | IL16 | 2.484E-04 | -0.041 | - | - |
| 3847 | ENSP00000362446 | ZNF79 | 5.637E-05 | -0.041 | - | - |
| 3848 | ENSP00000312286 | PLA2G1B | 3.842E-05 | -0.042 | - | - |
| 3849 | ENSP00000381499 | PAX1 | 3.070E-05 | -0.042 | - | - |
| 3850 | ENSP00000328631 | ARMCX2 | 1.546E-07 | -0.042 | - | - |
| 3851 | ENSP00000288602 | BRAF | 1.289E-04 | -0.042 | - | - |
| 3852 | ENSP00000317721 | PIPOX | 3.450E-05 | -0.042 | - | - |
| 3853 | ENSP00000356985 | DEDD | 4.624E-05 | -0.042 | - | - |
| 3854 | ENSP00000330612 | MRGPRG | 4.174E-05 | -0.042 | - | - |
| 3855 | ENSP00000332513 | SH3BGR | 2.740E-04 | -0.042 | - | - |
| 3856 | ENSP00000380184 | AGPAT6 | 4.274E-05 | -0.042 | - | - |
| 3857 | ENSP00000391101 | SLFN14 | 2.056E-06 | -0.042 | - | - |
| 3858 | ENSP00000447378 | MGAM | 1.076E-04 | -0.043 | - | - |
| 3859 | ENSP00000331678 | PKP3 | 3.035E-05 | -0.043 | - | - |
| 3860 | ENSP00000343027 | GNAI1 | 6.420E-05 | -0.043 | - | - |
| 3861 | ENSP00000290552 | IRX6 | 3.275E-05 | -0.043 | - | - |
| 3862 | ENSP00000262968 | TJP3 | 2.955E-05 | -0.043 | - | - |
| 3863 | ENSP00000367959 | HTR2A | 3.462E-05 | -0.043 | - | - |
| 3864 | ENSP00000359727 | BAG2 | 5.070E-05 | -0.043 | - | - |
| 3865 | ENSP00000330862 | OPCML | 2.574E-05 | -0.044 | - | - |
| 3866 | ENSP00000347170 | NEIL1 | 3.377E-05 | -0.044 | - | - |
| 3867 | ENSP00000262738 | CELSR1 | 3.645E-05 | -0.044 | - | - |
| 3868 | ENSP00000469046 | AC018445.1 | 1.345E-06 | -0.044 | - | - |
| 3869 | ENSP00000240652 | IAPP | 3.826E-05 | -0.044 | - | - |
| 3870 | ENSP00000379368 | ZNF35 | 6.615E-05 | -0.044 | - | - |
| 3871 | ENSP00000327513 | CSF1 | 1.818E-04 | -0.044 | - | - |
| 3872 | ENSP00000306565 | ISG20 | 7.138E-05 | -0.045 | - | - |
| 3873 | ENSP00000256010 | NTS | 2.963E-05 | -0.045 | - | - |
| 3874 | ENSP00000280684 | KCNA6 | 2.360E-05 | -0.045 | - | - |
| 3875 | ENSP00000326003 | PUS10 | 2.967E-05 | -0.045 | - | - |
| 3876 | ENSP00000270593 | ACPT | 3.644E-05 | -0.045 | - | - |
| 3877 | ENSP00000350332 | MYBPC2 | 2.649E-05 | -0.045 | - | - |
| 3878 | ENSP00000361207 | GHITM | 8.813E-05 | -0.046 | - | - |
| 3879 | ENSP00000436786 | MLL | 4.704E-05 | -0.046 | - | - |
| 3880 | ENSP00000259030 | RTP4 | 4.257E-05 | -0.046 | - | - |
| 3881 | ENSP00000357980 | HTRA1 | 7.512E-05 | -0.046 | - | - |
| 3882 | ENSP00000394484 | DCLK3 | 6.104E-05 | -0.047 | - | - |
| 3883 | ENSP00000360107 | EFHC1 | 4.654E-05 | -0.047 | - | - |
| 3884 | ENSP00000395590 | IFI35 | 8.566E-05 | -0.047 | - | - |
| 3885 | ENSP00000423673 | NDUFA13 | 5.823E-05 | -0.047 | - | - |
| 3886 | ENSP00000381240 | TRDN | 3.173E-05 | -0.047 | - | - |
| 3887 | ENSP00000353475 | CLDN7 | 3.980E-05 | -0.047 | - | - |
| 3888 | ENSP00000345873 | SLC26A3 | 3.007E-05 | -0.047 | - | - |
| 3889 | ENSP00000306776 | CCDC110 | 3.221E-05 | -0.047 | - | - |
| 3890 | ENSP00000420419 | JAM2 | 4.491E-05 | -0.047 | - | - |
| 3891 | ENSP00000416193 | P2RX6 | 4.110E-05 | -0.047 | - | - |
| 3892 | ENSP00000284240 | THY1 | 1.301E-04 | -0.047 | - | - |
| 3893 | ENSP00000444736 | TAS2R30 | 3.337E-07 | -0.048 | - | - |
| 3894 | ENSP00000441624 | TAS2R20 | 3.337E-07 | -0.048 | - | - |
| 3895 | ENSP00000333915 | CCDC42B | 6.060E-05 | -0.048 | - | - |
| 3896 | ENSP00000371234 | CYS1 | 1.426E-05 | -0.048 | - | - |
| 3897 | ENSP00000420267 | NR6A1 | 4.480E-05 | -0.048 | - | - |
| 3898 | ENSP00000355797 | TCP10L2 | 7.757E-07 | -0.048 | - | - |
| 3899 | ENSP00000371347 | ANGPT4 | 5.705E-05 | -0.048 | - | - |
| 3900 | ENSP00000402203 | CEACAM18 | 1.843E-06 | -0.048 | - | - |
| 3901 | ENSP00000261267 | LYZ | 5.546E-05 | -0.049 | - | - |
| 3902 | ENSP00000261800 | FAT2 | 4.107E-05 | -0.049 | - | - |
| 3903 | ENSP00000345023 | GSTO2 | 3.706E-05 | -0.049 | - | - |
| 3904 | ENSP00000308252 | HOXB3 | 3.378E-05 | -0.049 | - | - |
| 3905 | ENSP00000417895 | RASA4B | 3.521E-05 | -0.049 | - | - |
| 3906 | ENSP00000320936 | IL17RA | 3.876E-04 | -0.049 | - | - |
| 3907 | ENSP00000461862 | PCDHGB3 | 1.849E-05 | -0.049 | - | - |
| 3908 | ENSP00000430307 | PRSS46 | 3.181E-05 | -0.049 | - | - |
| 3909 | ENSP00000380858 | ST7-OT4 | 2.886E-05 | -0.049 | - | - |
| 3910 | ENSP00000265136 | COBL | 3.958E-05 | -0.049 | - | - |
| 3911 | ENSP00000305839 | GPRIN1 | 5.264E-05 | -0.049 | - | - |
| 3912 | ENSP00000316786 | HSD11B2 | 3.165E-05 | -0.049 | - | - |
| 3913 | ENSP00000247020 | SDF2 | 3.231E-05 | -0.049 | - | - |
| 3914 | ENSP00000386163 | TMEM14E | 3.458E-05 | -0.049 | - | - |
| 3915 | ENSP00000355760 | DACT2 | 2.886E-05 | -0.049 | - | - |
| 3916 | ENSP00000296499 | NDST3 | 2.727E-05 | -0.050 | - | - |
| 3917 | ENSP00000300098 | GPR182 | 2.667E-05 | -0.050 | - | - |
| 3918 | ENSP00000308908 | GPR148 | 1.886E-05 | -0.050 | - | - |
| 3919 | ENSP00000364512 | G6PC2 | 4.365E-05 | -0.050 | - | - |
| 3920 | ENSP00000414635 | LRRC34 | 4.966E-05 | -0.051 | - | - |
| 3921 | ENSP00000020945 | SNAI2 | 4.805E-05 | -0.051 | - | - |
| 3922 | ENSP00000347153 | SPATA31A7 | 4.160E-05 | -0.051 | - | - |
| 3923 | ENSP00000386488 | ZC3H8 | 6.076E-05 | -0.051 | - | - |
| 3924 | ENSP00000332690 | KRTAP6-1 | 3.234E-05 | -0.052 | - | - |
| 3925 | ENSP00000467123 | NLRP7 | 9.103E-06 | -0.052 | - | - |
| 3926 | ENSP00000331040 | OLIG2 | 2.464E-05 | -0.052 | - | - |
| 3927 | ENSP00000379895 | GATM | 4.929E-05 | -0.052 | - | - |
| 3928 | ENSP00000352900 | RXRG | 3.289E-05 | -0.052 | - | - |
| 3929 | ENSP00000343246 | HOXA1 | 2.781E-05 | -0.052 | - | - |
| 3930 | ENSP00000265239 | IQCG | 9.697E-05 | -0.052 | - | - |
| 3931 | ENSP00000367879 | XK | 4.298E-05 | -0.052 | - | - |
| 3932 | ENSP00000339681 | FAM78B | 1.554E-04 | -0.053 | - | - |
| 3933 | ENSP00000219599 | CRYM | 2.487E-05 | -0.053 | - | - |
| 3934 | ENSP00000364263 | RNF186 | 4.425E-05 | -0.053 | - | - |
| 3935 | ENSP00000450687 | RAX2 | 5.687E-05 | -0.053 | - | - |
| 3936 | ENSP00000351314 | PSMB10 | 4.346E-05 | -0.053 | - | - |
| 3937 | ENSP00000377941 | ACTN1 | 6.084E-05 | -0.053 | - | - |
| 3938 | ENSP00000249284 | TAS2R16 | 1.310E-04 | -0.054 | - | - |
| 3939 | ENSP00000357448 | PBXIP1 | 5.807E-05 | -0.054 | - | - |
| 3940 | ENSP00000266718 | LUM | 5.369E-05 | -0.054 | - | - |
| 3941 | ENSP00000415299 | FAM221B | 1.839E-05 | -0.054 | - | - |
| 3942 | ENSP00000380903 | ROBO3 | 3.614E-05 | -0.054 | - | - |
| 3943 | ENSP00000417190 | ATPAF2 | 2.002E-05 | -0.054 | - | - |
| 3944 | ENSP00000292330 | PPP1R35 | 4.566E-05 | -0.054 | - | - |
| 3945 | ENSP00000257497 | ANXA1 | 5.902E-05 | -0.054 | - | - |
| 3946 | ENSP00000276927 | IFNA1 | 1.446E-04 | -0.055 | - | - |
| 3947 | ENSP00000402962 | FKSG52 | 1.952E-05 | -0.055 | - | - |
| 3948 | ENSP00000361596 | NEURL2 | 6.695E-05 | -0.055 | - | - |
| 3949 | ENSP00000409370 | NLRP2 | 9.103E-06 | -0.055 | - | - |
| 3950 | ENSP00000385571 | HIPK2 | 7.042E-05 | -0.055 | - | - |
| 3951 | ENSP00000437523 | RP11-571M6.6 | 8.893E-06 | -0.055 | - | - |
| 3952 | ENSP00000234371 | KISS1R | 2.183E-05 | -0.055 | - | - |
| 3953 | ENSP00000362485 | GARNL3 | 5.898E-05 | -0.055 | - | - |
| 3954 | ENSP00000381150 | MBLAC1 | 1.862E-05 | -0.056 | - | - |
| 3955 | ENSP00000311862 | MS4A10 | 5.232E-05 | -0.056 | - | - |
| 3956 | ENSP00000284601 | PPP1R3A | 3.632E-05 | -0.056 | - | - |
| 3957 | ENSP00000249499 | HOXD9 | 3.411E-05 | -0.056 | - | - |
| 3958 | ENSP00000378426 | VKORC1 | 2.571E-05 | -0.056 | - | - |
| 3959 | ENSP00000463376 | CTC-479C5.12 | 2.454E-05 | -0.057 | - | - |
| 3960 | ENSP00000272852 | CPO | 3.894E-05 | -0.057 | - | - |
| 3961 | ENSP00000367391 | PGA4 | 1.596E-04 | -0.057 | - | - |
| 3962 | ENSP00000355031 | PER3 | 3.280E-05 | -0.058 | - | - |
| 3963 | ENSP00000348161 | GPR160 | 3.994E-05 | -0.058 | - | - |
| 3964 | ENSP00000360240 | GLYATL3 | 8.764E-06 | -0.058 | - | - |
| 3965 | ENSP00000352927 | SOGA2 | 4.101E-05 | -0.058 | - | - |
| 3966 | ENSP00000256958 | SLCO1B1 | 2.356E-05 | -0.058 | - | - |
| 3967 | ENSP00000465978 | RP11-477N12.3 | 7.142E-08 | -0.058 | - | - |
| 3968 | ENSP00000298295 | C10orf10 | 3.100E-05 | -0.058 | - | - |
| 3969 | ENSP00000286758 | CXCL13 | 6.729E-04 | -0.058 | - | - |
| 3970 | ENSP00000469019 | ASCL5 | 3.343E-06 | -0.059 | - | - |
| 3971 | ENSP00000392204 | KLLN | 4.477E-06 | -0.059 | - | - |
| 3972 | ENSP00000303325 | TACR3 | 3.637E-05 | -0.059 | - | - |
| 3973 | ENSP00000247470 | PYCARD | 1.415E-04 | -0.059 | - | - |
| 3974 | ENSP00000394757 | ZNF516 | 7.828E-05 | -0.059 | - | - |
| 3975 | ENSP00000265708 | ADAM2 | 1.986E-05 | -0.059 | - | - |
| 3976 | ENSP00000286380 | RAET1L | 3.563E-05 | -0.059 | - | - |
| 3977 | ENSP00000277526 | LCN9 | 5.852E-05 | -0.059 | - | - |
| 3978 | ENSP00000222800 | ABHD11 | 5.888E-05 | -0.060 | - | - |
| 3979 | ENSP00000363458 | LDLRAP1 | 6.872E-05 | -0.060 | - | - |
| 3980 | ENSP00000354691 | FCRL5 | 3.279E-05 | -0.060 | - | - |
| 3981 | ENSP00000263881 | MAP4K3 | 2.307E-05 | -0.060 | - | - |
| 3982 | ENSP00000349693 | - | 1.054E-04 | -0.061 | - | - |
| 3983 | ENSP00000315626 | FAM101A | 3.642E-05 | -0.061 | - | - |
| 3984 | ENSP00000221742 | SLC1A6 | 2.335E-05 | -0.061 | - | - |
| 3985 | ENSP00000412150 | CCDC121 | 1.858E-06 | -0.061 | - | - |
| 3986 | ENSP00000358747 | OVGP1 | 5.128E-05 | -0.061 | - | - |
| 3987 | ENSP00000349296 | TMEM71 | 6.992E-05 | -0.061 | - | - |
| 3988 | ENSP00000471817 | PRED62 | 1.577E-06 | -0.062 | - | - |
| 3989 | ENSP00000256585 | REG4 | 3.376E-05 | -0.062 | - | - |
| 3990 | ENSP00000216124 | ARSA | 3.985E-05 | -0.062 | - | - |
| 3991 | ENSP00000351118 | CDHR5 | 3.573E-05 | -0.062 | - | - |
| 3992 | ENSP00000366178 | CST9L | 2.493E-05 | -0.062 | - | - |
| 3993 | ENSP00000263708 | PTPN4 | 2.725E-05 | -0.063 | - | - |
| 3994 | ENSP00000341905 | RASAL3 | 3.278E-05 | -0.063 | - | - |
| 3995 | ENSP00000355609 | C1orf131 | 4.361E-05 | -0.063 | - | - |
| 3996 | ENSP00000357905 | DMBT1 | 7.480E-05 | -0.063 | - | - |
| 3997 | ENSP00000375109 | KRTAP13-3 | 1.693E-05 | -0.063 | - | - |
| 3998 | ENSP00000357914 | GABPB2 | 6.944E-05 | -0.063 | - | - |
| 3999 | ENSP00000470441 | AC012313.1 | 3.477E-08 | -0.063 | - | - |
| 4000 | ENSP00000442304 | FAM132B | 2.346E-05 | -0.063 | - | - |
| 4001 | ENSP00000445868 | BCL7A | 5.133E-05 | -0.064 | - | - |
| 4002 | ENSP00000324897 | UBE2I | 6.369E-05 | -0.064 | - | - |
| 4003 | ENSP00000289619 | PAGE5 | 3.611E-05 | -0.064 | - | - |
| 4004 | ENSP00000360696 | LCN12 | 1.001E-04 | -0.064 | - | - |
| 4005 | ENSP00000377980 | HEPACAM2 | 2.801E-05 | -0.064 | - | - |
| 4006 | ENSP00000364235 | RNF5 | 3.874E-05 | -0.064 | - | - |
| 4007 | ENSP00000257264 | TCN1 | 3.208E-05 | -0.064 | - | - |
| 4008 | ENSP00000353785 | TPPP | 6.746E-05 | -0.064 | - | - |
| 4009 | ENSP00000463817 | FAM27E3 | 2.649E-06 | -0.065 | - | - |
| 4010 | ENSP00000429834 | PCDHGA5 | 1.862E-05 | -0.065 | - | - |
| 4011 | ENSP00000368824 | CNKSR2 | 3.567E-05 | -0.065 | - | - |
| 4012 | ENSP00000361411 | ZCCHC24 | 4.027E-05 | -0.065 | - | - |
| 4013 | ENSP00000390761 | TBC1D3C | 2.086E-05 | -0.065 | - | - |
| 4014 | ENSP00000250825 | VCY | 1.559E-05 | -0.065 | - | - |
| 4015 | ENSP00000472724 | AC003043.1 | 1.790E-06 | -0.066 | - | - |
| 4016 | ENSP00000317891 | TNIP1 | 1.470E-04 | -0.066 | - | - |
| 4017 | ENSP00000390179 | ZNF527 | 5.487E-05 | -0.066 | - | - |
| 4018 | ENSP00000349593 | ZHX1-C8ORF76 | 5.072E-05 | -0.066 | - | - |
| 4019 | ENSP00000368266 | MAGEB4 | 3.586E-05 | -0.066 | - | - |
| 4020 | ENSP00000468678 | PLEKHH3 | 4.572E-05 | -0.066 | - | - |
| 4021 | ENSP00000301202 | LAIR2 | 3.050E-03 | -0.066 | - | - |
| 4022 | ENSP00000263915 | GRB14 | 4.883E-05 | -0.066 | - | - |
| 4023 | ENSP00000275560 | SRCRB4D | 4.622E-05 | -0.066 | - | - |
| 4024 | ENSP00000246020 | CSTL1 | 1.686E-04 | -0.066 | - | - |
| 4025 | ENSP00000369154 | SMAD9 | 4.931E-05 | -0.066 | - | - |
| 4026 | ENSP00000451995 | DUX4L5 | 3.700E-05 | -0.067 | - | - |
| 4027 | ENSP00000360147 | XPNPEP2 | 4.438E-05 | -0.067 | - | - |
| 4028 | ENSP00000382867 | MAGEB16 | 1.700E-05 | -0.067 | - | - |
| 4029 | ENSP00000427407 | SMIM20 | 3.046E-05 | -0.067 | - | - |
| 4030 | ENSP00000370710 | KDM4C | 3.439E-05 | -0.067 | - | - |
| 4031 | ENSP00000347714 | FCAR | 3.408E-05 | -0.068 | - | - |
| 4032 | ENSP00000273668 | EAF2 | 2.755E-05 | -0.068 | - | - |
| 4033 | ENSP00000436450 | TAS2R46 | 2.704E-06 | -0.068 | - | - |
| 4034 | ENSP00000377527 | PC | 5.262E-05 | -0.068 | - | - |
| 4035 | ENSP00000293330 | HCRT | 6.433E-05 | -0.068 | - | - |
| 4036 | ENSP00000309572 | TERT | 5.339E-05 | -0.068 | - | - |
| 4037 | ENSP00000373411 | NKIRAS1 | 3.409E-05 | -0.068 | - | - |
| 4038 | ENSP00000367891 | LGALS7 | 3.100E-05 | -0.068 | - | - |
| 4039 | ENSP00000381960 | OOSP1 | 1.660E-05 | -0.068 | - | - |
| 4040 | ENSP00000265361 | SEMA3C | 7.440E-05 | -0.068 | - | - |
| 4041 | ENSP00000354855 | NFE2L1 | 5.831E-05 | -0.068 | - | - |
| 4042 | ENSP00000257857 | CD63 | 9.123E-05 | -0.069 | - | - |
| 4043 | ENSP00000386787 | KIAA0922 | 8.893E-05 | -0.069 | - | - |
| 4044 | ENSP00000350075 | C15orf38 | 1.282E-04 | -0.069 | - | - |
| 4045 | ENSP00000371846 | DEAF1 | 5.237E-05 | -0.069 | - | - |
| 4046 | ENSP00000380671 | SMCO1 | 4.163E-07 | -0.069 | - | - |
| 4047 | ENSP00000349465 | PICK1 | 3.883E-05 | -0.069 | - | - |
| 4048 | ENSP00000263525 | TNR | 2.381E-05 | -0.069 | - | - |
| 4049 | ENSP00000362133 | C1orf122 | 9.091E-05 | -0.069 | - | - |
| 4050 | ENSP00000455948 | CLEC19A | 9.037E-06 | -0.069 | - | - |
| 4051 | ENSP00000364985 | LY6G6D | 1.709E-05 | -0.069 | - | - |
| 4052 | ENSP00000263640 | ACVR1 | 9.499E-05 | -0.070 | - | - |
| 4053 | ENSP00000384223 | ECE2 | 4.032E-05 | -0.070 | - | - |
| 4054 | ENSP00000404074 | ZSCAN9 | 3.983E-05 | -0.070 | - | - |
| 4055 | ENSP00000362435 | ZMYM6NB | 2.142E-05 | -0.070 | - | - |
| 4056 | ENSP00000376945 | FBXW9 | 4.715E-05 | -0.070 | - | - |
| 4057 | ENSP00000310801 | C20orf201 | 1.365E-05 | -0.070 | - | - |
| 4058 | ENSP00000303070 | SCGB1D1 | 2.898E-05 | -0.071 | - | - |
| 4059 | ENSP00000355969 | RD3 | 3.306E-05 | -0.071 | - | - |
| 4060 | ENSP00000304956 | MN1 | 3.081E-05 | -0.071 | - | - |
| 4061 | ENSP00000332139 | CFD | 1.368E-04 | -0.071 | - | - |
| 4062 | ENSP00000274024 | FABP2 | 3.621E-05 | -0.071 | - | - |
| 4063 | ENSP00000449253 | KCNC2 | 3.378E-05 | -0.071 | - | - |
| 4064 | ENSP00000243347 | TNFAIP6 | 7.733E-05 | -0.071 | - | - |
| 4065 | ENSP00000382434 | PLA2G4E | 6.124E-05 | -0.071 | - | - |
| 4066 | ENSP00000225842 | CCL1 | 6.389E-04 | -0.072 | - | - |
| 4067 | ENSP00000378642 | ZNF785 | 5.385E-05 | -0.072 | - | - |
| 4068 | ENSP00000347314 | SPG20 | 6.499E-05 | -0.072 | - | - |
| 4069 | ENSP00000303208 | PCSK9 | 5.175E-05 | -0.072 | - | - |
| 4070 | ENSP00000369456 | ITPA | 4.524E-05 | -0.072 | - | - |
| 4071 | ENSP00000327336 | BGN | 9.058E-05 | -0.072 | - | - |
| 4072 | ENSP00000394316 | BZW1 | 6.114E-05 | -0.072 | - | - |
| 4073 | ENSP00000304707 | SLN | 3.154E-05 | -0.072 | - | - |
| 4074 | ENSP00000411145 | FEZF1 | 4.597E-05 | -0.072 | - | - |
| 4075 | ENSP00000356411 | TROVE2 | 4.262E-05 | -0.072 | - | - |
| 4076 | ENSP00000346345 | C9orf163 | 2.895E-04 | -0.073 | - | - |
| 4077 | ENSP00000393511 | ABLIM2 | 8.130E-05 | -0.073 | - | - |
| 4078 | ENSP00000327968 | SNAI3 | 2.677E-05 | -0.073 | - | - |
| 4079 | ENSP00000365076 | C6orf47 | 6.913E-06 | -0.073 | - | - |
| 4080 | ENSP00000469270 | SBP1 | 5.550E-08 | -0.073 | - | - |
| 4081 | ENSP00000222982 | CYP3A5 | 3.396E-05 | -0.073 | - | - |
| 4082 | ENSP00000391397 | MT1G | 3.293E-05 | -0.073 | - | - |
| 4083 | ENSP00000404304 | ARMCX4 | 1.738E-06 | -0.073 | - | - |
| 4084 | ENSP00000364204 | PINK1 | 4.016E-05 | -0.074 | - | - |
| 4085 | ENSP00000321853 | SERPINF2 | 9.555E-05 | -0.074 | - | - |
| 4086 | ENSP00000242729 | SSPN | 3.503E-05 | -0.074 | - | - |
| 4087 | ENSP00000354090 | SIGLEC14 | 2.475E-05 | -0.074 | - | - |
| 4088 | ENSP00000223357 | AEBP1 | 3.529E-05 | -0.074 | - | - |
| 4089 | ENSP00000357113 | IFI16 | 8.054E-05 | -0.075 | - | - |
| 4090 | ENSP00000365503 | SHISA7 | 4.230E-05 | -0.075 | - | - |
| 4091 | ENSP00000266041 | ITIH4 | 1.017E-04 | -0.075 | - | - |
| 4092 | ENSP00000364034 | TAP2 | 5.815E-05 | -0.075 | - | - |
| 4093 | ENSP00000355787 | C6orf123 | 7.137E-06 | -0.075 | - | - |
| 4094 | ENSP00000263233 | SYP | 3.764E-05 | -0.075 | - | - |
| 4095 | ENSP00000365838 | DPRX | 1.041E-04 | -0.076 | - | - |
| 4096 | ENSP00000310008 | SWSAP1 | 4.347E-05 | -0.076 | - | - |
| 4097 | ENSP00000420295 | PDE6B | 4.867E-05 | -0.076 | - | - |
| 4098 | ENSP00000303174 | UGT1A6 | 3.646E-05 | -0.076 | - | - |
| 4099 | ENSP00000272937 | HES6 | 4.700E-05 | -0.076 | - | - |
| 4100 | ENSP00000216361 | COCH | 3.996E-05 | -0.076 | - | - |
| 4101 | ENSP00000369643 | GRPR | 2.945E-05 | -0.076 | - | - |
| 4102 | ENSP00000331664 | MGAT4C | 3.665E-05 | -0.076 | - | - |
| 4103 | ENSP00000344353 | LPAR6 | 2.829E-05 | -0.076 | - | - |
| 4104 | ENSP00000355084 | MSRB1 | 3.301E-05 | -0.076 | - | - |
| 4105 | ENSP00000367527 | DCLRE1C | 2.821E-05 | -0.076 | - | - |
| 4106 | ENSP00000331965 | TLCD2 | 1.745E-04 | -0.077 | - | - |
| 4107 | ENSP00000401477 | TMEM232 | 7.913E-05 | -0.077 | - | - |
| 4108 | ENSP00000242786 | CD97 | 1.642E-04 | -0.077 | - | - |
| 4109 | ENSP00000364467 | LYZL2 | 4.097E-05 | -0.077 | - | - |
| 4110 | ENSP00000472951 | AC138517.1 | 4.720E-07 | -0.077 | - | - |
| 4111 | ENSP00000352608 | RYR1 | 3.928E-05 | -0.077 | - | - |
| 4112 | ENSP00000361577 | C10orf11 | 6.133E-05 | -0.077 | - | - |
| 4113 | ENSP00000335320 | BCL9L | 2.985E-05 | -0.077 | - | - |
| 4114 | ENSP00000342033 | PDC | 6.358E-05 | -0.077 | - | - |
| 4115 | ENSP00000355245 | PAX9 | 4.100E-05 | -0.077 | - | - |
| 4116 | ENSP00000295206 | EN1 | 3.951E-05 | -0.077 | - | - |
| 4117 | ENSP00000402758 | PMEL | 1.427E-04 | -0.077 | - | - |
| 4118 | ENSP00000298171 | TSHR | 9.126E-05 | -0.078 | - | - |
| 4119 | ENSP00000167586 | KRT14 | 9.561E-05 | -0.078 | - | - |
| 4120 | ENSP00000364220 | VWA5B1 | 2.736E-05 | -0.078 | - | - |
| 4121 | ENSP00000308708 | TCP11 | 4.816E-05 | -0.078 | - | - |
| 4122 | ENSP00000366902 | ZBTB48 | 4.311E-05 | -0.078 | - | - |
| 4123 | ENSP00000327179 | CIRH1A | 3.987E-05 | -0.078 | - | - |
| 4124 | ENSP00000321445 | MSC | 2.708E-05 | -0.078 | - | - |
| 4125 | ENSP00000353887 | FAM153A | 1.414E-05 | -0.078 | - | - |
| 4126 | ENSP00000402152 | SLC12A3 | 3.662E-05 | -0.078 | - | - |
| 4127 | ENSP00000267845 | HDC | 2.797E-05 | -0.078 | - | - |
| 4128 | ENSP00000371397 | FAM43A | 4.383E-05 | -0.078 | - | - |
| 4129 | ENSP00000307041 | BNC1 | 6.903E-05 | -0.079 | - | - |
| 4130 | ENSP00000372910 | LY6G6E | 2.630E-07 | -0.079 | - | - |
| 4131 | ENSP00000349658 | XPNPEP3 | 2.773E-05 | -0.079 | - | - |
| 4132 | ENSP00000433816 | TSPAN19 | 1.502E-07 | -0.079 | - | - |
| 4133 | ENSP00000224237 | VIM | 4.135E-05 | -0.079 | - | - |
| 4134 | ENSP00000371724 | BPY2C | 1.387E-03 | -0.079 | - | - |
| 4135 | ENSP00000381045 | UBE3A | 9.257E-03 | -0.079 | - | - |
| 4136 | ENSP00000368737 | TMEM170B | 3.181E-06 | -0.079 | - | - |
| 4137 | ENSP00000455561 | ATP6C | 1.748E-05 | -0.079 | - | - |
| 4138 | ENSP00000383431 | GAB4 | 3.140E-05 | -0.079 | - | - |
| 4139 | ENSP00000351783 | FAM222A | 5.394E-05 | -0.079 | - | - |
| 4140 | ENSP00000384670 | KIAA1210 | 3.187E-05 | -0.080 | - | - |
| 4141 | ENSP00000375822 | ZNF222 | 4.936E-05 | -0.080 | - | - |
| 4142 | ENSP00000308938 | PLG | 1.257E-04 | -0.080 | - | - |
| 4143 | ENSP00000240123 | SORBS3 | 3.746E-05 | -0.080 | - | - |
| 4144 | ENSP00000354347 | GFPT1 | 3.056E-03 | -0.080 | - | - |
| 4145 | ENSP00000256861 | ITIH5 | 2.575E-05 | -0.080 | - | - |
| 4146 | ENSP00000274242 | RPL37 | 9.870E-05 | -0.080 | - | - |
| 4147 | ENSP00000264434 | C2orf42 | 1.376E-03 | -0.080 | - | - |
| 4148 | ENSP00000366853 | ZNF658 | 5.307E-05 | -0.080 | - | - |
| 4149 | ENSP00000366347 | NKX2-2 | 3.443E-05 | -0.080 | - | - |
| 4150 | ENSP00000294338 | PDZK1IP1 | 1.261E-04 | -0.080 | - | - |
| 4151 | ENSP00000303522 | TACR1 | 4.254E-05 | -0.080 | - | - |
| 4152 | ENSP00000345684 | NBPF10 | 4.668E-05 | -0.081 | - | - |
| 4153 | ENSP00000471857 | AC096677.1 | 4.788E-08 | -0.081 | - | - |
| 4154 | ENSP00000380352 | DDHD2 | 4.820E-05 | -0.081 | - | - |
| 4155 | ENSP00000401770 | C14orf2 | 1.082E-05 | -0.081 | - | - |
| 4156 | ENSP00000410910 | MUSTN1 | 5.056E-05 | -0.081 | - | - |
| 4157 | ENSP00000432545 | - | 2.344E-05 | -0.082 | - | - |
| 4158 | ENSP00000371595 | KRTAP5-6 | 2.479E-05 | -0.082 | - | - |
| 4159 | ENSP00000263735 | EPCAM | 8.414E-05 | -0.082 | - | - |
| 4160 | ENSP00000283309 | FRMD1 | 4.176E-05 | -0.082 | - | - |
| 4161 | ENSP00000349654 | NELL1 | 3.764E-05 | -0.082 | - | - |
| 4162 | ENSP00000351363 | MSMB | 8.271E-05 | -0.082 | - | - |
| 4163 | ENSP00000292069 | ZNF667 | 6.766E-05 | -0.082 | - | - |
| 4164 | ENSP00000408581 | TMEM132D | 2.934E-05 | -0.082 | - | - |
| 4165 | ENSP00000252338 | FAM155B | 1.888E-05 | -0.082 | - | - |
| 4166 | ENSP00000352119 | HIST1H2AG | 7.431E-05 | -0.082 | - | - |
| 4167 | ENSP00000265517 | MTTP | 3.502E-05 | -0.082 | - | - |
| 4168 | ENSP00000274532 | TIMD4 | 5.292E-05 | -0.083 | - | - |
| 4169 | ENSP00000355051 | TAF13 | 3.659E-05 | -0.083 | - | - |
| 4170 | ENSP00000246100 | FAM110A | 6.614E-05 | -0.083 | - | - |
| 4171 | ENSP00000313833 | LCN15 | 2.963E-05 | -0.083 | - | - |
| 4172 | ENSP00000351575 | BDP1 | 5.337E-05 | -0.083 | - | - |
| 4173 | ENSP00000365431 | TCF19 | 2.894E-05 | -0.083 | - | - |
| 4174 | ENSP00000298974 | MAGEA9 | 3.439E-05 | -0.083 | - | - |
| 4175 | ENSP00000451229 | TMEM253 | 1.851E-07 | -0.083 | - | - |
| 4176 | ENSP00000235933 | CD160 | 1.557E-04 | -0.083 | - | - |
| 4177 | ENSP00000216714 | APEX1 | 3.899E-05 | -0.083 | - | - |
| 4178 | ENSP00000346762 | SND1 | 5.308E-05 | -0.084 | - | - |
| 4179 | ENSP00000358162 | HIST2H4A | 4.434E-05 | -0.084 | - | - |
| 4180 | ENSP00000244007 | PLCG1 | 5.734E-05 | -0.084 | - | - |
| 4181 | ENSP00000335074 | GHRL | 4.350E-05 | -0.084 | - | - |
| 4182 | ENSP00000377298 | NUMA1 | 5.938E-05 | -0.084 | - | - |
| 4183 | ENSP00000311778 | ZMIZ2 | 4.444E-05 | -0.084 | - | - |
| 4184 | ENSP00000413479 | KRT3 | 4.464E-05 | -0.084 | - | - |
| 4185 | ENSP00000293922 | PTX4 | 6.097E-05 | -0.084 | - | - |
| 4186 | ENSP00000302324 | RNASE3 | 1.031E-04 | -0.085 | - | - |
| 4187 | ENSP00000351384 | - | 5.944E-06 | -0.085 | - | - |
| 4188 | ENSP00000369849 | MEDAG | 3.328E-05 | -0.085 | - | - |
| 4189 | ENSP00000309782 | MRGPRF | 3.116E-05 | -0.085 | - | - |
| 4190 | ENSP00000377370 | IGSF11 | 4.268E-05 | -0.085 | - | - |
| 4191 | ENSP00000433931 | AIM1L | 1.653E-05 | -0.085 | - | - |
| 4192 | ENSP00000369777 | ZNF768 | 5.390E-05 | -0.085 | - | - |
| 4193 | ENSP00000230882 | GHR | 6.434E-05 | -0.085 | - | - |
| 4194 | ENSP00000404432 | LIMS3L | 2.146E-05 | -0.085 | - | - |
| 4195 | ENSP00000358241 | ACP6 | 2.794E-05 | -0.085 | - | - |
| 4196 | ENSP00000218224 | PQBP1 | 4.200E-05 | -0.086 | - | - |
| 4197 | ENSP00000386394 | NIF3L1 | 1.392E-04 | -0.086 | - | - |
| 4198 | ENSP00000391249 | 9-Sep | 5.912E-05 | -0.086 | - | - |
| 4199 | ENSP00000274306 | GZMA | 2.368E-04 | -0.086 | - | - |
| 4200 | ENSP00000298296 | MAGEC3 | 3.193E-05 | -0.086 | - | - |
| 4201 | ENSP00000370253 | NKX3-1 | 3.704E-05 | -0.086 | - | - |
| 4202 | ENSP00000360635 | DPP7 | 3.633E-05 | -0.086 | - | - |
| 4203 | ENSP00000363972 | ZC4H2 | 2.182E-05 | -0.086 | - | - |
| 4204 | ENSP00000328083 | ARHGEF37 | 3.327E-05 | -0.086 | - | - |
| 4205 | ENSP00000170150 | BPIFB2 | 5.433E-05 | -0.086 | - | - |
| 4206 | ENSP00000370997 | C2orf50 | 2.394E-07 | -0.086 | - | - |
| 4207 | ENSP00000351395 | SCGB1D4 | 2.466E-05 | -0.087 | - | - |
| 4208 | ENSP00000358955 | KIAA1324 | 3.426E-05 | -0.087 | - | - |
| 4209 | ENSP00000262120 | TWSG1 | 1.463E-04 | -0.087 | - | - |
| 4210 | ENSP00000456026 | SMLR1 | 2.432E-07 | -0.087 | - | - |
| 4211 | ENSP00000341274 | AAK1 | 1.126E-07 | -0.087 | - | - |
| 4212 | ENSP00000325519 | AC020907.1 | 2.943E-05 | -0.087 | - | - |
| 4213 | ENSP00000435517 | ALG9 | 4.144E-05 | -0.087 | - | - |
| 4214 | ENSP00000275699 | ASB15 | 6.283E-05 | -0.087 | - | - |
| 4215 | ENSP00000458570 | PCDHGA4 | 1.858E-05 | -0.087 | - | - |
| 4216 | ENSP00000216024 | DMC1 | 1.565E-05 | -0.087 | - | - |
| 4217 | ENSP00000409950 | ANHX | 6.990E-08 | -0.087 | - | - |
| 4218 | ENSP00000329106 | BPY2 | 3.836E-05 | -0.088 | - | - |
| 4219 | ENSP00000357392 | EFNA1 | 6.730E-05 | -0.088 | - | - |
| 4220 | ENSP00000330774 | TMEM106A | 4.468E-05 | -0.088 | - | - |
| 4221 | ENSP00000381589 | PCDHGA11 | 2.404E-05 | -0.088 | - | - |
| 4222 | ENSP00000358919 | PSRC1 | 4.525E-05 | -0.088 | - | - |
| 4223 | ENSP00000370503 | CCM2 | 3.148E-05 | -0.088 | - | - |
| 4224 | ENSP00000299206 | POLL | 1.185E-04 | -0.088 | - | - |
| 4225 | ENSP00000367832 | CCL7 | 1.745E-04 | -0.088 | - | - |
| 4226 | ENSP00000403270 | AGAP9 | 8.194E-05 | -0.088 | - | - |
| 4227 | ENSP00000341550 | SLC24A5 | 2.661E-05 | -0.088 | - | - |
| 4228 | ENSP00000351466 | OR5AC2 | 2.574E-05 | -0.088 | - | - |
| 4229 | ENSP00000358045 | ECM1 | 5.058E-05 | -0.088 | - | - |
| 4230 | ENSP00000344432 | DOK7 | 4.500E-05 | -0.088 | - | - |
| 4231 | ENSP00000381034 | SOCS6 | 3.207E-05 | -0.089 | - | - |
| 4232 | ENSP00000369373 | TRIM5 | 6.856E-05 | -0.089 | - | - |
| 4233 | ENSP00000401535 | RP1-130H16.18 | 1.027E-05 | -0.089 | - | - |
| 4234 | ENSP00000370270 | ESRRB | 3.107E-05 | -0.089 | - | - |
| 4235 | ENSP00000358531 | SYCP1 | 2.802E-05 | -0.089 | - | - |
| 4236 | ENSP00000368362 | FABP9 | 4.032E-05 | -0.089 | - | - |
| 4237 | ENSP00000385706 | PSG2 | 1.539E-05 | -0.089 | - | - |
| 4238 | ENSP00000383520 | AL354822.1 | 4.457E-05 | -0.089 | - | - |
| 4239 | ENSP00000459439 | CTD-3148I10.1 | 4.842E-08 | -0.089 | - | - |
| 4240 | ENSP00000273596 | ABHD14A | 6.904E-05 | -0.089 | - | - |
| 4241 | ENSP00000205948 | APOH | 1.333E-04 | -0.089 | - | - |
| 4242 | ENSP00000364992 | LY6G6F | 2.065E-05 | -0.089 | - | - |
| 4243 | ENSP00000338648 | MS4A4A | 3.561E-05 | -0.090 | - | - |
| 4244 | ENSP00000367316 | ITGA8 | 3.498E-05 | -0.090 | - | - |
| 4245 | ENSP00000331418 | PTPRM | 2.642E-05 | -0.090 | - | - |
| 4246 | ENSP00000265724 | ABCB1 | 4.543E-05 | -0.090 | - | - |
| 4247 | ENSP00000358397 | FAM72B | 3.013E-05 | -0.090 | - | - |
| 4248 | ENSP00000358464 | SHOC2 | 4.717E-05 | -0.090 | - | - |
| 4249 | ENSP00000386960 | C10orf55 | 9.801E-06 | -0.090 | - | - |
| 4250 | ENSP00000416549 | AC109135.1 | 1.018E-09 | -0.090 | - | - |
| 4251 | ENSP00000231461 | ST8SIA4 | 2.335E-05 | -0.090 | - | - |
| 4252 | ENSP00000361669 | ESX1 | 5.484E-05 | -0.090 | - | - |
| 4253 | ENSP00000457230 | WWOX | 7.732E-04 | -0.091 | - | - |
| 4254 | ENSP00000342235 | ERBB4 | 9.951E-05 | -0.091 | - | - |
| 4255 | ENSP00000335158 | GPR142 | 2.877E-05 | -0.091 | - | - |
| 4256 | ENSP00000239461 | PRRX1 | 4.426E-05 | -0.091 | - | - |
| 4257 | ENSP00000322192 | PGA3 | 1.782E-04 | -0.091 | - | - |
| 4258 | ENSP00000353346 | LHFPL5 | 3.839E-05 | -0.091 | - | - |
| 4259 | ENSP00000345487 | QRFP | 2.381E-05 | -0.091 | - | - |
| 4260 | ENSP00000226382 | PHOX2B | 8.243E-05 | -0.091 | - | - |
| 4261 | ENSP00000344545 | SCGB1C1 | 5.033E-05 | -0.091 | - | - |
| 4262 | ENSP00000046087 | ZPBP | 2.902E-05 | -0.091 | - | - |
| 4263 | ENSP00000361400 | SFTPA2 | 2.695E-05 | -0.091 | - | - |
| 4264 | ENSP00000418803 | TIMMDC1 | 5.421E-05 | -0.091 | - | - |
| 4265 | ENSP00000306662 | ADRA1B | 6.401E-05 | -0.091 | - | - |
| 4266 | ENSP00000456635 | TEX38 | 4.816E-06 | -0.092 | - | - |
| 4267 | ENSP00000221485 | SLC17A7 | 2.071E-05 | -0.092 | - | - |
| 4268 | ENSP00000383639 | AC145212.1 | 4.457E-05 | -0.092 | - | - |
| 4269 | ENSP00000368450 | CD83 | 2.389E-04 | -0.092 | - | - |
| 4270 | ENSP00000355812 | FGFR1OP | 3.387E-05 | -0.092 | - | - |
| 4271 | ENSP00000354669 | FAM63A | 4.194E-05 | -0.092 | - | - |
| 4272 | ENSP00000386175 | OR2A1 | 1.940E-05 | -0.092 | - | - |
| 4273 | ENSP00000364107 | PAGE2 | 2.701E-05 | -0.092 | - | - |
| 4274 | ENSP00000359539 | GOT1 | 3.213E-05 | -0.092 | - | - |
| 4275 | ENSP00000369981 | SH3GL2 | 4.085E-05 | -0.092 | - | - |
| 4276 | ENSP00000364261 | OTUD3 | 5.251E-05 | -0.092 | - | - |
| 4277 | ENSP00000284984 | ADAMTS1 | 1.010E-04 | -0.092 | - | - |
| 4278 | ENSP00000471024 | AC093917.1 | 2.345E-05 | -0.092 | - | - |
| 4279 | ENSP00000291527 | TFF1 | 3.674E-05 | -0.092 | - | - |
| 4280 | ENSP00000308720 | PRSS1 | 1.291E-04 | -0.093 | - | - |
| 4281 | ENSP00000252660 | MAS1 | 3.435E-05 | -0.093 | - | - |
| 4282 | ENSP00000320084 | CD276 | 2.478E-04 | -0.093 | - | - |
| 4283 | ENSP00000398124 | E2F5 | 3.338E-05 | -0.093 | - | - |
| 4284 | ENSP00000343398 | TMPPE | 3.369E-06 | -0.093 | - | - |
| 4285 | ENSP00000355136 | SORBS1 | 6.803E-05 | -0.093 | - | - |
| 4286 | ENSP00000361508 | PLTP | 2.806E-05 | -0.093 | - | - |
| 4287 | ENSP00000236671 | CTSD | 5.065E-05 | -0.093 | - | - |
| 4288 | ENSP00000388311 | ZNF845 | 4.520E-05 | -0.093 | - | - |
| 4289 | ENSP00000385527 | MFSD2B | 2.149E-05 | -0.093 | - | - |
| 4290 | ENSP00000256592 | TSHB | 4.721E-05 | -0.093 | - | - |
| 4291 | ENSP00000325917 | PCSK7 | 6.389E-05 | -0.093 | - | - |
| 4292 | ENSP00000258106 | EMX1 | 4.617E-05 | -0.093 | - | - |
| 4293 | ENSP00000472120 | CATX-2 | 9.983E-06 | -0.093 | - | - |
| 4294 | ENSP00000368994 | ARHGAP44 | 3.452E-05 | -0.093 | - | - |
| 4295 | ENSP00000429190 | ENDOV | 3.225E-05 | -0.093 | - | - |
| 4296 | ENSP00000409722 | AL592183.1 | 3.271E-05 | -0.094 | - | - |
| 4297 | ENSP00000216106 | HMGXB4 | 3.897E-05 | -0.094 | - | - |
| 4298 | ENSP00000344749 | CCDC37 | 3.695E-05 | -0.094 | - | - |
| 4299 | ENSP00000370372 | NLN | 2.839E-05 | -0.094 | - | - |
| 4300 | ENSP00000274008 | SPATA5 | 5.475E-05 | -0.094 | - | - |
| 4301 | ENSP00000218075 | GLRA2 | 2.217E-05 | -0.094 | - | - |
| 4302 | ENSP00000293897 | SSTR5 | 8.776E-05 | -0.094 | - | - |
| 4303 | ENSP00000225823 | ASIC2 | 2.270E-05 | -0.094 | - | - |
| 4304 | ENSP00000042931 | BEST2 | 3.631E-05 | -0.094 | - | - |
| 4305 | ENSP00000361871 | WFDC12 | 2.069E-05 | -0.094 | - | - |
| 4306 | ENSP00000342222 | TRPV2 | 7.597E-05 | -0.094 | - | - |
| 4307 | ENSP00000295589 | CCKAR | 2.711E-05 | -0.094 | - | - |
| 4308 | ENSP00000365528 | PTPRH | 3.523E-05 | -0.094 | - | - |
| 4309 | ENSP00000378784 | PHF20L1 | 3.468E-05 | -0.094 | - | - |
| 4310 | ENSP00000363390 | TRIM63 | 5.184E-05 | -0.095 | - | - |
| 4311 | ENSP00000345751 | SCNN1B | 2.950E-05 | -0.095 | - | - |
| 4312 | ENSP00000358703 | RIPPLY2 | 2.988E-05 | -0.095 | - | - |
| 4313 | ENSP00000339952 | KSR2 | 1.553E-04 | -0.095 | - | - |
| 4314 | ENSP00000377545 | C1QTNF2 | 2.748E-05 | -0.095 | - | - |
| 4315 | ENSP00000354504 | ASTN2 | 6.083E-05 | -0.095 | - | - |
| 4316 | ENSP00000360538 | FFAR4 | 1.054E-04 | -0.095 | - | - |
| 4317 | ENSP00000280200 | CD226 | 1.502E-04 | -0.095 | - | - |
| 4318 | ENSP00000404833 | TAPBP | 1.036E-04 | -0.095 | - | - |
| 4319 | ENSP00000360757 | FAM69B | 2.449E-05 | -0.095 | - | - |
| 4320 | ENSP00000318374 | LENG8 | 1.272E-05 | -0.096 | - | - |
| 4321 | ENSP00000311398 | RNASE8 | 2.953E-05 | -0.096 | - | - |
| 4322 | ENSP00000326598 | PRSS50 | 2.483E-05 | -0.096 | - | - |
| 4323 | ENSP00000385276 | TENM3 | 4.027E-05 | -0.096 | - | - |
| 4324 | ENSP00000404403 | ZBTB22 | 3.719E-05 | -0.096 | - | - |
| 4325 | ENSP00000301921 | SOX7 | 2.451E-05 | -0.096 | - | - |
| 4326 | ENSP00000352111 | GOLGA8A | 6.883E-05 | -0.096 | - | - |
| 4327 | ENSP00000332773 | IGDCC3 | 3.098E-05 | -0.096 | - | - |
| 4328 | ENSP00000280057 | FAM124A | 5.884E-05 | -0.096 | - | - |
| 4329 | ENSP00000389414 | TBXAS1 | 4.673E-05 | -0.096 | - | - |
| 4330 | ENSP00000361834 | COL9A2 | 2.565E-05 | -0.096 | - | - |
| 4331 | ENSP00000231004 | LOX | 6.552E-05 | -0.096 | - | - |
| 4332 | ENSP00000348459 | ACMSD | 8.152E-05 | -0.097 | - | - |
| 4333 | ENSP00000333956 | SELV | 3.512E-05 | -0.097 | - | - |
| 4334 | ENSP00000306361 | FGA | 1.123E-04 | -0.097 | - | - |
| 4335 | ENSP00000298942 | PTER | 5.944E-05 | -0.097 | - | - |
| 4336 | ENSP00000215773 | DDT | 1.568E-04 | -0.097 | - | - |
| 4337 | ENSP00000363999 | MRPL50 | 4.234E-05 | -0.097 | - | - |
| 4338 | ENSP00000356230 | KLHL12 | 3.801E-05 | -0.097 | - | - |
| 4339 | ENSP00000352575 | INPP5D | 5.738E-05 | -0.097 | - | - |
| 4340 | ENSP00000273968 | PYURF | 2.972E-05 | -0.097 | - | - |
| 4341 | ENSP00000287777 | KLHL40 | 1.236E-04 | -0.097 | - | - |
| 4342 | ENSP00000375783 | DACT3 | 1.831E-05 | -0.097 | - | - |
| 4343 | ENSP00000289932 | SLC5A11 | 4.536E-05 | -0.097 | - | - |
| 4344 | ENSP00000321326 | F2R | 5.336E-05 | -0.097 | - | - |
| 4345 | ENSP00000377061 | PLXNA1 | 3.545E-05 | -0.097 | - | - |
| 4346 | ENSP00000357116 | SAMD3 | 3.449E-05 | -0.098 | - | - |
| 4347 | ENSP00000364697 | PROZ | 1.858E-05 | -0.098 | - | - |
| 4348 | ENSP00000364621 | SUSD3 | 3.954E-05 | -0.098 | - | - |
| 4349 | ENSP00000366036 | TBC1D8 | 3.596E-05 | -0.098 | - | - |
| 4350 | ENSP00000365394 | - | 1.529E-05 | -0.098 | - | - |
| 4351 | ENSP00000312741 | CAMKK2 | 6.383E-05 | -0.098 | - | - |
| 4352 | ENSP00000363431 | NPY4R | 3.327E-05 | -0.098 | - | - |
| 4353 | ENSP00000356687 | SLC9C2 | 2.388E-05 | -0.098 | - | - |
| 4354 | ENSP00000338624 | TASP1 | 4.042E-05 | -0.098 | - | - |
| 4355 | ENSP00000356908 | TAAR2 | 2.780E-05 | -0.098 | - | - |
| 4356 | ENSP00000403397 | ZDHHC17 | 1.156E-02 | -0.099 | - | - |
| 4357 | ENSP00000337949 | HTR7 | 5.351E-05 | -0.099 | - | - |
| 4358 | ENSP00000425864 | MEF2BNB | 4.079E-06 | -0.099 | - | - |
| 4359 | ENSP00000259371 | DAB2IP | 3.341E-05 | -0.099 | - | - |
| 4360 | ENSP00000386230 | C19orf73 | 8.852E-08 | -0.099 | - | - |
| 4361 | ENSP00000262262 | CD33 | 1.348E-04 | -0.099 | - | - |
| 4362 | ENSP00000261168 | ATF7IP | 2.481E-05 | -0.099 | - | - |
| 4363 | ENSP00000348936 | LEKR1 | 2.761E-05 | -0.099 | - | - |
| 4364 | ENSP00000258534 | DRAM1 | 3.283E-05 | -0.099 | - | - |
| 4365 | ENSP00000349305 | GOLGA4 | 3.857E-05 | -0.099 | - | - |
| 4366 | ENSP00000306381 | - | 4.209E-05 | -0.099 | - | - |
| 4367 | ENSP00000333697 | TMEM179B | 4.843E-05 | -0.099 | - | - |
| 4368 | ENSP00000368682 | PHEX | 5.535E-05 | -0.099 | - | - |
| 4369 | ENSP00000204961 | EFNB1 | 6.777E-05 | -0.099 | - | - |
| 4370 | ENSP00000334148 | WDR25 | 1.344E-05 | -0.099 | - | - |
| 4371 | ENSP00000370213 | CLCN4 | 4.563E-05 | -0.100 | - | - |
| 4372 | ENSP00000471878 | AC005606.1 | 1.429E-07 | -0.100 | - | - |
| 4373 | ENSP00000345681 | GATA2 | 6.820E-05 | -0.100 | - | - |
| 4374 | ENSP00000324270 | OXTR | 5.682E-05 | -0.100 | - | - |
| 4375 | ENSP00000271643 | ADAMTSL4 | 3.469E-05 | -0.100 | - | - |
| 4376 | ENSP00000346697 | BMF | 4.186E-05 | -0.100 | - | - |
| 4377 | ENSP00000345102 | C19orf35 | 3.656E-05 | -0.100 | - | - |
| 4378 | ENSP00000176183 | DRD4 | 5.408E-05 | -0.100 | - | - |
| 4379 | ENSP00000222747 | TSPAN12 | 3.139E-05 | -0.100 | - | - |
| 4380 | ENSP00000336630 | ADORA2A | 5.250E-05 | -0.100 | - | - |
| 4381 | ENSP00000451258 | RP11-293M10.1 | 5.385E-08 | -0.100 | - | - |
| 4382 | ENSP00000356481 | FAM129A | 5.290E-05 | -0.100 | - | - |
| 4383 | ENSP00000385478 | ISPD | 8.198E-06 | -0.100 | - | - |
| 4384 | ENSP00000370104 | GPS2 | 1.904E-05 | -0.100 | - | - |
| 4385 | ENSP00000270631 | PTH2 | 3.173E-05 | -0.100 | - | - |
| 4386 | ENSP00000270590 | GPR32 | 8.696E-05 | -0.101 | - | - |
| 4387 | ENSP00000438757 | PRR20C | 6.117E-08 | -0.101 | - | - |
| 4388 | ENSP00000415769 | ITIH3 | 7.298E-05 | -0.101 | - | - |
| 4389 | ENSP00000053243 | TNFRSF17 | 9.255E-05 | -0.101 | - | - |
| 4390 | ENSP00000359534 | SPANXN3 | 6.960E-07 | -0.101 | - | - |
| 4391 | ENSP00000342300 | NELFCD | 2.501E-04 | -0.101 | - | - |
| 4392 | ENSP00000375562 | RBM12B-AS1 | 2.050E-08 | -0.101 | - | - |
| 4393 | ENSP00000337797 | PIDD | 4.459E-05 | -0.101 | - | - |
| 4394 | ENSP00000357697 | S100A2 | 3.696E-05 | -0.101 | - | - |
| 4395 | ENSP00000383873 | TTC34 | 1.298E-08 | -0.101 | - | - |
| 4396 | ENSP00000278888 | MS4A2 | 1.723E-04 | -0.101 | - | - |
| 4397 | ENSP00000316496 | DCAF4L2 | 3.171E-05 | -0.102 | - | - |
| 4398 | ENSP00000262623 | ATP4A | 3.803E-05 | -0.102 | - | - |
| 4399 | ENSP00000307234 | PCDHB1 | 1.937E-05 | -0.102 | - | - |
| 4400 | ENSP00000344544 | IKZF3 | 7.860E-05 | -0.102 | - | - |
| 4401 | ENSP00000359206 | BTRC | 1.269E-04 | -0.102 | - | - |
| 4402 | ENSP00000420213 | FLNB | 4.197E-05 | -0.102 | - | - |
| 4403 | ENSP00000211122 | GSTA3 | 3.040E-05 | -0.102 | - | - |
| 4404 | ENSP00000263182 | BBOX1 | 3.946E-05 | -0.102 | - | - |
| 4405 | ENSP00000367490 | DEPDC4 | 3.064E-05 | -0.103 | - | - |
| 4406 | ENSP00000223642 | C5 | 1.713E-04 | -0.103 | - | - |
| 4407 | ENSP00000367309 | MAOB | 3.624E-05 | -0.103 | - | - |
| 4408 | ENSP00000352408 | BABAM1 | 5.655E-05 | -0.103 | - | - |
| 4409 | ENSP00000351589 | HIST1H2AI | 7.236E-05 | -0.103 | - | - |
| 4410 | ENSP00000234590 | ENO1 | 2.309E-05 | -0.103 | - | - |
| 4411 | ENSP00000330393 | LEPR | 3.052E-04 | -0.103 | - | - |
| 4412 | ENSP00000455169 | NETO2 | 8.396E-05 | -0.103 | - | - |
| 4413 | ENSP00000381611 | PCDHGA10 | 1.909E-05 | -0.103 | - | - |
| 4414 | ENSP00000361214 | NRG3 | 2.389E-05 | -0.103 | - | - |
| 4415 | ENSP00000471531 | PRED57 | 3.290E-07 | -0.103 | - | - |
| 4416 | ENSP00000364865 | RP11-65D24.2 | 3.466E-06 | -0.103 | - | - |
| 4417 | ENSP00000332225 | PMCH | 6.324E-05 | -0.103 | - | - |
| 4418 | ENSP00000351618 | PARD3B | 3.547E-05 | -0.103 | - | - |
| 4419 | ENSP00000328879 | TBL1Y | 2.475E-05 | -0.103 | - | - |
| 4420 | ENSP00000417871 | MSH5-SAPCD1 | 2.043E-05 | -0.103 | - | - |
| 4421 | ENSP00000288266 | APPL1 | 6.742E-05 | -0.104 | - | - |
| 4422 | ENSP00000331933 | WSCD2 | 2.296E-05 | -0.104 | - | - |
| 4423 | ENSP00000429399 | C1orf210 | 4.537E-07 | -0.104 | - | - |
| 4424 | ENSP00000318650 | GREM2 | 2.480E-05 | -0.104 | - | - |
| 4425 | ENSP00000337804 | SPAG17 | 3.590E-05 | -0.104 | - | - |
| 4426 | ENSP00000368332 | ARX | 5.584E-05 | -0.104 | - | - |
| 4427 | ENSP00000356733 | MROH9 | 1.438E-06 | -0.104 | - | - |
| 4428 | ENSP00000264463 | CDH10 | 2.615E-05 | -0.104 | - | - |
| 4429 | ENSP00000360252 | HOOK1 | 5.929E-05 | -0.104 | - | - |
| 4430 | ENSP00000221973 | LIM2 | 3.074E-05 | -0.105 | - | - |
| 4431 | ENSP00000327417 | GPR39 | 2.644E-05 | -0.105 | - | - |
| 4432 | ENSP00000451382 | - | 1.090E-05 | -0.105 | - | - |
| 4433 | ENSP00000261588 | KIAA0556 | 3.200E-05 | -0.105 | - | - |
| 4434 | ENSP00000395244 | TMEM229A | 1.533E-06 | -0.105 | - | - |
| 4435 | ENSP00000388825 | MLN | 2.884E-05 | -0.105 | - | - |
| 4436 | ENSP00000216075 | MIOX | 4.110E-05 | -0.105 | - | - |
| 4437 | ENSP00000265162 | ENPEP | 4.590E-05 | -0.105 | - | - |
| 4438 | ENSP00000258214 | CCDC102A | 3.966E-05 | -0.105 | - | - |
| 4439 | ENSP00000344012 | GAS2L1 | 2.279E-05 | -0.105 | - | - |
| 4440 | ENSP00000367263 | AHNAK | 6.116E-05 | -0.105 | - | - |
| 4441 | ENSP00000301219 | LILRA5 | 9.566E-06 | -0.105 | - | - |
| 4442 | ENSP00000257749 | BACH2 | 1.850E-04 | -0.105 | - | - |
| 4443 | ENSP00000341268 | TRADD | 1.600E-04 | -0.106 | - | - |
| 4444 | ENSP00000272520 | C1QL2 | 2.654E-05 | -0.106 | - | - |
| 4445 | ENSP00000316333 | CD55 | 8.792E-05 | -0.106 | - | - |
| 4446 | ENSP00000284440 | UCHL1 | 5.843E-05 | -0.106 | - | - |
| 4447 | ENSP00000386280 | APOL6 | 6.588E-05 | -0.106 | - | - |
| 4448 | ENSP00000296370 | S100P | 1.034E-04 | -0.106 | - | - |
| 4449 | ENSP00000274276 | OSMR | 1.651E-04 | -0.106 | - | - |
| 4450 | ENSP00000367926 | CXorf30 | 1.804E-07 | -0.106 | - | - |
| 4451 | ENSP00000392398 | GPX5 | 3.230E-05 | -0.106 | - | - |
| 4452 | ENSP00000268603 | CDH11 | 3.027E-05 | -0.106 | - | - |
| 4453 | ENSP00000426996 | - | 9.113E-08 | -0.106 | - | - |
| 4454 | ENSP00000252242 | KRT5 | 6.406E-05 | -0.106 | - | - |
| 4455 | ENSP00000238044 | C2orf40 | 3.530E-05 | -0.106 | - | - |
| 4456 | ENSP00000416897 | AC008394.1 | 4.903E-07 | -0.106 | - | - |
| 4457 | ENSP00000354826 | CALD1 | 3.730E-05 | -0.106 | - | - |
| 4458 | ENSP00000252825 | HRC | 3.657E-05 | -0.107 | - | - |
| 4459 | ENSP00000222718 | HOXA2 | 3.485E-05 | -0.107 | - | - |
| 4460 | ENSP00000349971 | CEACAM3 | 2.811E-05 | -0.107 | - | - |
| 4461 | ENSP00000299402 | APBB1 | 5.320E-05 | -0.107 | - | - |
| 4462 | ENSP00000278833 | ROM1 | 2.097E-05 | -0.107 | - | - |
| 4463 | ENSP00000376322 | HOXD13 | 3.171E-05 | -0.107 | - | - |
| 4464 | ENSP00000240687 | TAS2R7 | 1.260E-04 | -0.107 | - | - |
| 4465 | ENSP00000225941 | ABI3 | 2.239E-05 | -0.107 | - | - |
| 4466 | ENSP00000333194 | RGS19 | 7.058E-05 | -0.107 | - | - |
| 4467 | ENSP00000245323 | EFNB2 | 5.652E-05 | -0.107 | - | - |
| 4468 | ENSP00000410024 | PHGR1 | 5.940E-07 | -0.107 | - | - |
| 4469 | ENSP00000361917 | ARMCX1 | 2.241E-05 | -0.108 | - | - |
| 4470 | ENSP00000413861 | SIGLEC9 | 1.583E-05 | -0.108 | - | - |
| 4471 | ENSP00000363205 | NFS1 | 4.303E-05 | -0.108 | - | - |
| 4472 | ENSP00000397082 | SFTPA1 | 2.803E-05 | -0.108 | - | - |
| 4473 | ENSP00000278855 | PLAC1L | 4.986E-05 | -0.108 | - | - |
| 4474 | ENSP00000220562 | EXTL3 | 1.658E-05 | -0.108 | - | - |
| 4475 | ENSP00000272065 | ACP1 | 7.133E-05 | -0.108 | - | - |
| 4476 | ENSP00000418259 | ZFP57 | 4.775E-05 | -0.108 | - | - |
| 4477 | ENSP00000428966 | ZFP41 | 2.847E-05 | -0.108 | - | - |
| 4478 | ENSP00000383256 | SIAH3 | 1.853E-05 | -0.108 | - | - |
| 4479 | ENSP00000331368 | SERPINB8 | 8.988E-05 | -0.108 | - | - |
| 4480 | ENSP00000358092 | PRDM1 | 1.671E-04 | -0.108 | - | - |
| 4481 | ENSP00000436901 | DPF2 | 3.441E-05 | -0.108 | - | - |
| 4482 | ENSP00000371968 | AC074389.6 | 1.156E-02 | -0.108 | - | - |
| 4483 | ENSP00000359070 | STMN3 | 4.920E-05 | -0.108 | - | - |
| 4484 | ENSP00000284262 | JPH3 | 5.960E-05 | -0.108 | - | - |
| 4485 | ENSP00000399078 | OR10J1 | 3.878E-05 | -0.108 | - | - |
| 4486 | ENSP00000335229 | C17orf82 | 4.434E-05 | -0.109 | - | - |
| 4487 | ENSP00000382260 | DGKB | 3.028E-05 | -0.109 | - | - |
| 4488 | ENSP00000248594 | PTPN12 | 6.656E-05 | -0.109 | - | - |
| 4489 | ENSP00000248244 | TICAM1 | 2.093E-04 | -0.109 | - | - |
| 4490 | ENSP00000361852 | TMCO2 | 1.162E-05 | -0.109 | - | - |
| 4491 | ENSP00000254457 | LHX1 | 2.987E-05 | -0.109 | - | - |
| 4492 | ENSP00000393887 | AHSG | 2.603E-05 | -0.109 | - | - |
| 4493 | ENSP00000274853 | PPP1R18 | 3.703E-06 | -0.109 | - | - |
| 4494 | ENSP00000453076 | GPR176 | 3.073E-05 | -0.109 | - | - |
| 4495 | ENSP00000364784 | C6orf48 | 5.724E-08 | -0.110 | - | - |
| 4496 | ENSP00000271915 | KCNN3 | 1.031E-04 | -0.110 | - | - |
| 4497 | ENSP00000355140 | HOXB1 | 2.353E-05 | -0.110 | - | - |
| 4498 | ENSP00000457539 | APOBR | 2.697E-05 | -0.110 | - | - |
| 4499 | ENSP00000296474 | MST1R | 6.543E-05 | -0.110 | - | - |
| 4500 | ENSP00000369571 | IFNA14 | 1.560E-04 | -0.110 | - | - |
| 4501 | ENSP00000322295 | OR10AB1P | 6.220E-05 | -0.110 | - | - |
| 4502 | ENSP00000052754 | DCN | 2.882E-05 | -0.110 | - | - |
| 4503 | ENSP00000386207 | HMSD | 2.171E-05 | -0.110 | - | - |
| 4504 | ENSP00000256343 | CATSPERB | 2.839E-05 | -0.110 | - | - |
| 4505 | ENSP00000330475 | TMPRSS11B | 4.149E-05 | -0.110 | - | - |
| 4506 | ENSP00000324742 | IL32 | 2.249E-04 | -0.111 | - | - |
| 4507 | ENSP00000446137 | AL360154.1 | 5.254E-09 | -0.111 | - | - |
| 4508 | ENSP00000307879 | C11orf45 | 4.948E-05 | -0.111 | - | - |
| 4509 | ENSP00000262419 | KANSL1 | 1.372E-04 | -0.111 | - | - |
| 4510 | ENSP00000350911 | CTSE | 3.010E-05 | -0.111 | - | - |
| 4511 | ENSP00000365651 | NPPB | 3.557E-05 | -0.111 | - | - |
| 4512 | ENSP00000341524 | 6-Sep | 7.790E-05 | -0.111 | - | - |
| 4513 | ENSP00000264360 | PCDH10 | 2.879E-05 | -0.111 | - | - |
| 4514 | ENSP00000359532 | NTSR1 | 4.061E-05 | -0.111 | - | - |
| 4515 | ENSP00000361302 | POMT1 | 3.029E-05 | -0.111 | - | - |
| 4516 | ENSP00000258168 | BCMO1 | 2.613E-05 | -0.111 | - | - |
| 4517 | ENSP00000353224 | TFRC | 8.589E-05 | -0.111 | - | - |
| 4518 | ENSP00000377492 | HMMR | 4.372E-05 | -0.111 | - | - |
| 4519 | ENSP00000281382 | PIGF | 6.406E-05 | -0.111 | - | - |
| 4520 | ENSP00000359240 | LZTS2 | 6.715E-05 | -0.111 | - | - |
| 4521 | ENSP00000244061 | RNF114 | 2.696E-04 | -0.111 | - | - |
| 4522 | ENSP00000368727 | XDH | 3.984E-05 | -0.111 | - | - |
| 4523 | ENSP00000371644 | DEFB136 | 1.614E-05 | -0.111 | - | - |
| 4524 | ENSP00000336984 | HORMAD2 | 2.825E-05 | -0.111 | - | - |
| 4525 | ENSP00000321475 | KLHL38 | 1.069E-05 | -0.111 | - | - |
| 4526 | ENSP00000406381 | APOC4 | 1.279E-05 | -0.112 | - | - |
| 4527 | ENSP00000341481 | FAM25C | 7.435E-05 | -0.112 | - | - |
| 4528 | ENSP00000342790 | FAM25A | 7.435E-05 | -0.112 | - | - |
| 4529 | ENSP00000354829 | SGMS1 | 4.026E-05 | -0.112 | - | - |
| 4530 | ENSP00000368173 | DHRS13 | 1.874E-05 | -0.112 | - | - |
| 4531 | ENSP00000330825 | IFITM1 | 1.231E-04 | -0.112 | - | - |
| 4532 | ENSP00000309259 | ALAS1 | 1.382E-03 | -0.112 | - | - |
| 4533 | ENSP00000358966 | IMPG1 | 3.896E-05 | -0.112 | - | - |
| 4534 | ENSP00000392726 | POM121L2 | 7.827E-06 | -0.112 | - | - |
| 4535 | ENSP00000343171 | C2orf80 | 2.442E-05 | -0.112 | - | - |
| 4536 | ENSP00000356795 | TBX19 | 5.011E-05 | -0.112 | - | - |
| 4537 | ENSP00000343674 | ASTL | 3.235E-05 | -0.112 | - | - |
| 4538 | ENSP00000246012 | CST8 | 2.221E-05 | -0.112 | - | - |
| 4539 | ENSP00000329967 | TBK1 | 6.924E-05 | -0.112 | - | - |
| 4540 | ENSP00000368664 | RGCC | 2.646E-05 | -0.112 | - | - |
| 4541 | ENSP00000322640 | SPATA31E1 | 6.396E-07 | -0.112 | - | - |
| 4542 | ENSP00000407497 | SPINK8 | 3.197E-05 | -0.113 | - | - |
| 4543 | ENSP00000289749 | NBL1 | 5.608E-05 | -0.113 | - | - |
| 4544 | ENSP00000291574 | TRAPPC10 | 2.551E-05 | -0.113 | - | - |
| 4545 | ENSP00000401508 | NAPRT1 | 3.057E-05 | -0.113 | - | - |
| 4546 | ENSP00000307694 | KCNS1 | 2.217E-05 | -0.113 | - | - |
| 4547 | ENSP00000368119 | GALT | 3.888E-05 | -0.113 | - | - |
| 4548 | ENSP00000353893 | LGALS14 | 5.559E-05 | -0.113 | - | - |
| 4549 | ENSP00000334928 | PTPRB | 3.467E-05 | -0.113 | - | - |
| 4550 | ENSP00000411070 | LCE6A | 4.424E-06 | -0.113 | - | - |
| 4551 | ENSP00000014930 | HEBP1 | 1.735E-04 | -0.114 | - | - |
| 4552 | ENSP00000226299 | LAP3 | 4.107E-05 | -0.114 | - | - |
| 4553 | ENSP00000364578 | NELFE | 5.471E-05 | -0.114 | - | - |
| 4554 | ENSP00000263043 | ICK | 2.517E-05 | -0.114 | - | - |
| 4555 | ENSP00000357535 | ECHS1 | 1.066E-04 | -0.114 | - | - |
| 4556 | ENSP00000365851 | BMI1 | 8.860E-04 | -0.114 | - | - |
| 4557 | ENSP00000366997 | DIS3 | 7.099E-05 | -0.114 | - | - |
| 4558 | ENSP00000324944 | MBOAT1 | 3.374E-05 | -0.114 | - | - |
| 4559 | ENSP00000261740 | TRPV4 | 5.894E-05 | -0.114 | - | - |
| 4560 | ENSP00000356529 | RGS16 | 2.417E-05 | -0.114 | - | - |
| 4561 | ENSP00000305133 | SOCS5 | 1.670E-04 | -0.114 | - | - |
| 4562 | ENSP00000264036 | MCAM | 8.707E-05 | -0.114 | - | - |
| 4563 | ENSP00000383407 | AF165138.7 | 3.142E-06 | -0.114 | - | - |
| 4564 | ENSP00000364217 | AGER | 1.672E-05 | -0.114 | - | - |
| 4565 | ENSP00000200691 | MT3 | 5.419E-05 | -0.114 | - | - |
| 4566 | ENSP00000340210 | CD59 | 8.566E-05 | -0.114 | - | - |
| 4567 | ENSP00000251372 | LILRA1 | 1.340E-04 | -0.114 | - | - |
| 4568 | ENSP00000395549 | C22orf34 | 8.787E-06 | -0.114 | - | - |
| 4569 | ENSP00000254627 | OC90 | 3.377E-05 | -0.115 | - | - |
| 4570 | ENSP00000328090 | OTOP3 | 2.881E-05 | -0.115 | - | - |
| 4571 | ENSP00000316284 | OR2Z1 | 8.651E-06 | -0.115 | - | - |
| 4572 | ENSP00000367130 | HES3 | 1.496E-05 | -0.115 | - | - |
| 4573 | ENSP00000295872 | SPICE1 | 1.321E-04 | -0.115 | - | - |
| 4574 | ENSP00000230036 | GPLD1 | 2.817E-05 | -0.115 | - | - |
| 4575 | ENSP00000333735 | NPAP1 | 1.531E-05 | -0.115 | - | - |
| 4576 | ENSP00000422769 | DNAH10OS | 9.113E-08 | -0.115 | - | - |
| 4577 | ENSP00000345797 | KRBOX4 | 3.422E-05 | -0.115 | - | - |
| 4578 | ENSP00000353048 | CCT8L2 | 2.545E-05 | -0.115 | - | - |
| 4579 | ENSP00000297203 | C7orf62 | 6.474E-06 | -0.115 | - | - |
| 4580 | ENSP00000391944 | EEF1D | 4.660E-05 | -0.116 | - | - |
| 4581 | ENSP00000381302 | PPAPDC1A | 2.545E-05 | -0.116 | - | - |
| 4582 | ENSP00000442736 | PLXNB3 | 2.928E-05 | -0.116 | - | - |
| 4583 | ENSP00000464833 | HSH2D | 2.947E-07 | -0.116 | - | - |
| 4584 | ENSP00000357507 | C1orf43 | 3.700E-05 | -0.116 | - | - |
| 4585 | ENSP00000359485 | GBP6 | 5.744E-05 | -0.116 | - | - |
| 4586 | ENSP00000251102 | CNGB1 | 2.745E-05 | -0.116 | - | - |
| 4587 | ENSP00000356842 | GPA33 | 2.309E-05 | -0.116 | - | - |
| 4588 | ENSP00000298854 | RAPSN | 3.088E-05 | -0.116 | - | - |
| 4589 | ENSP00000450144 | CHURC1 | 1.283E-05 | -0.116 | - | - |
| 4590 | ENSP00000451451 | TNFSF12 | 5.887E-05 | -0.116 | - | - |
| 4591 | ENSP00000383517 | AC002321.2 | 6.507E-08 | -0.116 | - | - |
| 4592 | ENSP00000235180 | DLGAP3 | 2.767E-05 | -0.116 | - | - |
| 4593 | ENSP00000403459 | GRB7 | 3.444E-05 | -0.116 | - | - |
| 4594 | ENSP00000381010 | PRKRIP1 | 3.088E-05 | -0.116 | - | - |
| 4595 | ENSP00000362170 | NAIF1 | 2.479E-05 | -0.117 | - | - |
| 4596 | ENSP00000264122 | CBLB | 1.344E-04 | -0.117 | - | - |
| 4597 | ENSP00000381436 | VGLL3 | 2.511E-05 | -0.117 | - | - |
| 4598 | ENSP00000289877 | SLC45A1 | 2.042E-05 | -0.117 | - | - |
| 4599 | ENSP00000295082 | KCNF1 | 2.338E-05 | -0.117 | - | - |
| 4600 | ENSP00000416125 | ARHGEF38 | 1.094E-05 | -0.117 | - | - |
| 4601 | ENSP00000328928 | HOXB4 | 3.569E-05 | -0.117 | - | - |
| 4602 | ENSP00000295981 | IL17RC | 3.642E-04 | -0.117 | - | - |
| 4603 | ENSP00000432279 | CHMP1B | 7.791E-05 | -0.117 | - | - |
| 4604 | ENSP00000264245 | ARHGAP31 | 2.452E-05 | -0.117 | - | - |
| 4605 | ENSP00000304845 | UGT1A1 | 3.341E-05 | -0.117 | - | - |
| 4606 | ENSP00000321334 | LPA | 9.151E-05 | -0.117 | - | - |
| 4607 | ENSP00000369840 | TEX26 | 2.391E-05 | -0.118 | - | - |
| 4608 | ENSP00000297469 | GPER | 9.426E-05 | -0.118 | - | - |
| 4609 | ENSP00000265272 | JAKMIP2 | 5.511E-05 | -0.118 | - | - |
| 4610 | ENSP00000454261 | TMEM114 | 1.868E-05 | -0.118 | - | - |
| 4611 | ENSP00000316518 | OR9A2 | 1.496E-05 | -0.118 | - | - |
| 4612 | ENSP00000383682 | FAM72D | 2.829E-08 | -0.118 | - | - |
| 4613 | ENSP00000381703 | DEFB113 | 1.293E-06 | -0.118 | - | - |
| 4614 | ENSP00000300576 | GOLGA6C | 3.599E-05 | -0.118 | - | - |
| 4615 | ENSP00000365536 | WDR45 | 9.258E-06 | -0.118 | - | - |
| 4616 | ENSP00000263642 | IFIH1 | 1.186E-04 | -0.118 | - | - |
| 4617 | ENSP00000355895 | LYPLAL1 | 4.095E-05 | -0.118 | - | - |
| 4618 | ENSP00000286190 | ALS2CR12 | 5.021E-05 | -0.119 | - | - |
| 4619 | ENSP00000320828 | EPS8L2 | 3.101E-05 | -0.119 | - | - |
| 4620 | ENSP00000300131 | NAB2 | 7.602E-05 | -0.119 | - | - |
| 4621 | ENSP00000437635 | SLFN12L | 7.586E-06 | -0.119 | - | - |
| 4622 | ENSP00000331704 | XKR3 | 4.806E-05 | -0.119 | - | - |
| 4623 | ENSP00000346412 | ELP5 | 4.044E-05 | -0.119 | - | - |
| 4624 | ENSP00000360576 | NDOR1 | 6.113E-05 | -0.119 | - | - |
| 4625 | ENSP00000294973 | HAAO | 7.026E-05 | -0.119 | - | - |
| 4626 | ENSP00000343443 | ZNF107 | 7.070E-05 | -0.119 | - | - |
| 4627 | ENSP00000260257 | FDXACB1 | 4.626E-05 | -0.120 | - | - |
| 4628 | ENSP00000444856 | CUL2 | 1.030E-04 | -0.120 | - | - |
| 4629 | ENSP00000312262 | ADRBK1 | 1.272E-04 | -0.120 | - | - |
| 4630 | ENSP00000368752 | PRNP | 5.818E-05 | -0.120 | - | - |
| 4631 | ENSP00000321856 | CRELD1 | 4.266E-05 | -0.120 | - | - |
| 4632 | ENSP00000409555 | HERPUD1 | 3.336E-05 | -0.120 | - | - |
| 4633 | ENSP00000385328 | RNF43 | 3.047E-05 | -0.120 | - | - |
| 4634 | ENSP00000317842 | CES2 | 2.736E-05 | -0.120 | - | - |
| 4635 | ENSP00000448789 | OR9A4 | 5.582E-08 | -0.120 | - | - |
| 4636 | ENSP00000468389 | AC002398.9 | 1.643E-06 | -0.120 | - | - |
| 4637 | ENSP00000362570 | FNDC5 | 2.439E-05 | -0.120 | - | - |
| 4638 | ENSP00000378254 | EML3 | 4.222E-05 | -0.120 | - | - |
| 4639 | ENSP00000418491 | LCN10 | 8.144E-06 | -0.120 | - | - |
| 4640 | ENSP00000253496 | F12 | 5.442E-05 | -0.121 | - | - |
| 4641 | ENSP00000360944 | STIL | 2.936E-05 | -0.121 | - | - |
| 4642 | ENSP00000295269 | SLC9A4 | 7.318E-05 | -0.121 | - | - |
| 4643 | ENSP00000472130 | AL161784.1 | 6.384E-08 | -0.121 | - | - |
| 4644 | ENSP00000309757 | LPL | 4.618E-05 | -0.121 | - | - |
| 4645 | ENSP00000419446 | ADAM9 | 4.178E-05 | -0.121 | - | - |
| 4646 | ENSP00000360277 | - | 3.991E-05 | -0.121 | - | - |
| 4647 | ENSP00000268695 | GALNS | 2.733E-05 | -0.121 | - | - |
| 4648 | ENSP00000365807 | GNA14 | 3.905E-05 | -0.121 | - | - |
| 4649 | ENSP00000366509 | ZKSCAN4 | 3.301E-05 | -0.121 | - | - |
| 4650 | ENSP00000443339 | MTRNR2L3 | 2.097E-07 | -0.121 | - | - |
| 4651 | ENSP00000346316 | HIST1H4I | 3.478E-05 | -0.121 | - | - |
| 4652 | ENSP00000367185 | LYPD5 | 2.892E-05 | -0.121 | - | - |
| 4653 | ENSP00000450461 | CEP95 | 1.174E-05 | -0.121 | - | - |
| 4654 | ENSP00000402257 | RUNX1T1 | 5.542E-05 | -0.121 | - | - |
| 4655 | ENSP00000384524 | ETV2 | 2.518E-05 | -0.122 | - | - |
| 4656 | ENSP00000331532 | SPATA32 | 3.617E-06 | -0.122 | - | - |
| 4657 | ENSP00000319118 | GSX2 | 2.606E-05 | -0.122 | - | - |
| 4658 | ENSP00000453019 | C9orf69 | 2.009E-05 | -0.122 | - | - |
| 4659 | ENSP00000222573 | ITGB8 | 1.108E-04 | -0.122 | - | - |
| 4660 | ENSP00000465742 | RASGRP4 | 7.063E-05 | -0.122 | - | - |
| 4661 | ENSP00000328478 | CNGA2 | 1.983E-05 | -0.122 | - | - |
| 4662 | ENSP00000254488 | SLC6A11 | 2.638E-05 | -0.122 | - | - |
| 4663 | ENSP00000284481 | C8orf12 | 2.005E-05 | -0.122 | - | - |
| 4664 | ENSP00000386393 | ZNF630 | 3.045E-05 | -0.122 | - | - |
| 4665 | ENSP00000354675 | BHLHB9 | 4.245E-05 | -0.122 | - | - |
| 4666 | ENSP00000226284 | IBSP | 5.867E-05 | -0.122 | - | - |
| 4667 | ENSP00000355657 | HIST3H3 | 3.942E-05 | -0.122 | - | - |
| 4668 | ENSP00000420182 | IQCJ-SCHIP1 | 1.208E-04 | -0.122 | - | - |
| 4669 | ENSP00000352273 | DIO3 | 1.159E-05 | -0.123 | - | - |
| 4670 | ENSP00000357804 | THEM4 | 2.831E-05 | -0.123 | - | - |
| 4671 | ENSP00000435150 | KIAA0408 | 4.560E-05 | -0.123 | - | - |
| 4672 | ENSP00000252037 | FKBP6 | 1.774E-05 | -0.123 | - | - |
| 4673 | ENSP00000369986 | C21orf88 | 1.421E-06 | -0.123 | - | - |
| 4674 | ENSP00000259075 | TANK | 1.753E-04 | -0.123 | - | - |
| 4675 | ENSP00000272928 | CXCR7 | 1.276E-04 | -0.123 | - | - |
| 4676 | ENSP00000323740 | UCP3 | 2.577E-05 | -0.123 | - | - |
| 4677 | ENSP00000343819 | OTX2 | 4.595E-05 | -0.123 | - | - |
| 4678 | ENSP00000357759 | LCE1E | 2.415E-05 | -0.123 | - | - |
| 4679 | ENSP00000237316 | TCF21 | 2.339E-05 | -0.123 | - | - |
| 4680 | ENSP00000307199 | AHSP | 1.162E-05 | -0.123 | - | - |
| 4681 | ENSP00000335657 | CCK | 5.369E-05 | -0.123 | - | - |
| 4682 | ENSP00000309087 | PLAC8L1 | 5.629E-08 | -0.123 | - | - |
| 4683 | ENSP00000317912 | - | 5.714E-05 | -0.123 | - | - |
| 4684 | ENSP00000315098 | EDDM3A | 2.071E-05 | -0.123 | - | - |
| 4685 | ENSP00000320430 | S100Z | 1.994E-05 | -0.123 | - | - |
| 4686 | ENSP00000387298 | ERICH2 | 7.785E-07 | -0.123 | - | - |
| 4687 | ENSP00000302812 | SP7 | 5.392E-05 | -0.123 | - | - |
| 4688 | ENSP00000457849 | RP11-613M10.8 | 3.929E-06 | -0.123 | - | - |
| 4689 | ENSP00000007722 | ITGA3 | 8.213E-05 | -0.123 | - | - |
| 4690 | ENSP00000470965 | PCDP1 | 9.427E-07 | -0.123 | - | - |
| 4691 | ENSP00000271357 | GPR161 | 2.945E-05 | -0.123 | - | - |
| 4692 | ENSP00000354581 | GPX6 | 2.435E-05 | -0.124 | - | - |
| 4693 | ENSP00000331727 | KCNH7 | 2.490E-05 | -0.124 | - | - |
| 4694 | ENSP00000268058 | PML | 5.473E-05 | -0.124 | - | - |
| 4695 | ENSP00000461324 | SLC25A10 | 1.276E-05 | -0.124 | - | - |
| 4696 | ENSP00000256737 | ANO3 | 3.689E-05 | -0.124 | - | - |
| 4697 | ENSP00000272602 | CNGA3 | 2.349E-05 | -0.124 | - | - |
| 4698 | ENSP00000388001 | OAS1 | 7.574E-05 | -0.124 | - | - |
| 4699 | ENSP00000390427 | PPIL2 | 4.084E-05 | -0.124 | - | - |
| 4700 | ENSP00000368007 | TMEM240 | 2.125E-05 | -0.124 | - | - |
| 4701 | ENSP00000292574 | CCDC105 | 3.783E-05 | -0.124 | - | - |
| 4702 | ENSP00000086933 | GSC2 | 5.016E-05 | -0.124 | - | - |
| 4703 | ENSP00000316244 | HTR1A | 5.363E-05 | -0.124 | - | - |
| 4704 | ENSP00000366976 | KRTAP1-4 | 5.283E-05 | -0.124 | - | - |
| 4705 | ENSP00000254079 | PPP1R1B | 4.647E-05 | -0.124 | - | - |
| 4706 | ENSP00000345494 | PLSCR1 | 4.672E-05 | -0.124 | - | - |
| 4707 | ENSP00000388519 | - | 5.104E-07 | -0.124 | - | - |
| 4708 | ENSP00000285930 | AKR1B1 | 5.441E-05 | -0.124 | - | - |
| 4709 | ENSP00000368135 | LRRN4 | 4.041E-05 | -0.124 | - | - |
| 4710 | ENSP00000302021 | MUC7 | 4.299E-05 | -0.125 | - | - |
| 4711 | ENSP00000353915 | HTR4 | 2.846E-05 | -0.125 | - | - |
| 4712 | ENSP00000346791 | TTC3 | 3.115E-05 | -0.125 | - | - |
| 4713 | ENSP00000358170 | FAM204A | 4.949E-05 | -0.125 | - | - |
| 4714 | ENSP00000417246 | C10orf128 | 7.462E-07 | -0.125 | - | - |
| 4715 | ENSP00000234389 | GRIN3B | 2.354E-05 | -0.125 | - | - |
| 4716 | ENSP00000349022 | ARC | 1.396E-05 | -0.125 | - | - |
| 4717 | ENSP00000380087 | ZNF213 | 3.776E-05 | -0.125 | - | - |
| 4718 | ENSP00000423602 | DAPP1 | 2.475E-05 | -0.125 | - | - |
| 4719 | ENSP00000251377 | LILRA2 | 1.340E-04 | -0.125 | - | - |
| 4720 | ENSP00000432622 | CCDC163P | 1.589E-07 | -0.125 | - | - |
| 4721 | ENSP00000378050 | TRPT1 | 3.486E-05 | -0.125 | - | - |
| 4722 | ENSP00000358067 | C10orf85 | 1.412E-05 | -0.125 | - | - |
| 4723 | ENSP00000318585 | BACE1 | 8.390E-05 | -0.125 | - | - |
| 4724 | ENSP00000261037 | COL8A1 | 4.445E-05 | -0.125 | - | - |
| 4725 | ENSP00000326253 | RTBDN | 4.319E-05 | -0.125 | - | - |
| 4726 | ENSP00000366288 | SLITRK1 | 2.679E-05 | -0.126 | - | - |
| 4727 | ENSP00000355146 | GPRASP1 | 3.708E-05 | -0.126 | - | - |
| 4728 | ENSP00000361556 | C9orf50 | 1.319E-06 | -0.126 | - | - |
| 4729 | ENSP00000394653 | IQCF5 | 3.333E-08 | -0.126 | - | - |
| 4730 | ENSP00000348211 | SLC25A46 | 5.883E-05 | -0.126 | - | - |
| 4731 | ENSP00000430690 | TFF3 | 2.818E-05 | -0.126 | - | - |
| 4732 | ENSP00000293379 | ITGA5 | 6.658E-05 | -0.126 | - | - |
| 4733 | ENSP00000363516 | RCAN3 | 5.519E-05 | -0.126 | - | - |
| 4734 | ENSP00000305988 | ALCAM | 1.011E-04 | -0.126 | - | - |
| 4735 | ENSP00000324248 | PENK | 6.752E-05 | -0.126 | - | - |
| 4736 | ENSP00000310928 | PPARD | 5.483E-05 | -0.126 | - | - |
| 4737 | ENSP00000350314 | BCO2 | 2.613E-05 | -0.126 | - | - |
| 4738 | ENSP00000265572 | OPRK1 | 7.445E-05 | -0.126 | - | - |
| 4739 | ENSP00000329825 | SPATA31A6 | 6.396E-07 | -0.126 | - | - |
| 4740 | ENSP00000359819 | MOSPD1 | 4.632E-05 | -0.126 | - | - |
| 4741 | ENSP00000299213 | LARP6 | 2.696E-05 | -0.127 | - | - |
| 4742 | ENSP00000457330 | LIMS1 | 8.297E-06 | -0.127 | - | - |
| 4743 | ENSP00000295685 | ARPC2 | 6.678E-05 | -0.127 | - | - |
| 4744 | ENSP00000263379 | IL27RA | 3.830E-04 | -0.127 | - | - |
| 4745 | ENSP00000369682 | MRPS26 | 7.680E-06 | -0.127 | - | - |
| 4746 | ENSP00000244709 | TREM1 | 4.559E-04 | -0.127 | - | - |
| 4747 | ENSP00000332123 | PROSAPIP1 | 7.038E-05 | -0.127 | - | - |
| 4748 | ENSP00000403984 | DCDC2C | 1.499E-05 | -0.127 | - | - |
| 4749 | ENSP00000438895 | IZUMO3 | 9.051E-08 | -0.127 | - | - |
| 4750 | ENSP00000287936 | HMGCR | 4.631E-05 | -0.127 | - | - |
| 4751 | ENSP00000241416 | ACVR2A | 9.124E-05 | -0.127 | - | - |
| 4752 | ENSP00000385806 | KCNMA1 | 5.532E-05 | -0.127 | - | - |
| 4753 | ENSP00000381293 | NSF | 4.767E-05 | -0.127 | - | - |
| 4754 | ENSP00000386414 | RAD21L1 | 3.431E-05 | -0.127 | - | - |
| 4755 | ENSP00000358784 | KCNA3 | 4.336E-05 | -0.127 | - | - |
| 4756 | ENSP00000452581 | IFRG15 | 1.212E-06 | -0.128 | - | - |
| 4757 | ENSP00000262613 | SLC9A3R1 | 5.380E-05 | -0.128 | - | - |
| 4758 | ENSP00000378394 | PSAP | 5.568E-05 | -0.128 | - | - |
| 4759 | ENSP00000219150 | CORO1A | 3.933E-05 | -0.128 | - | - |
| 4760 | ENSP00000415034 | NBEAL2 | 3.027E-05 | -0.128 | - | - |
| 4761 | ENSP00000350018 | SH3BP1 | 3.413E-05 | -0.128 | - | - |
| 4762 | ENSP00000317578 | ADRBK2 | 5.022E-05 | -0.128 | - | - |
| 4763 | ENSP00000350719 | SYNE2 | 4.560E-05 | -0.128 | - | - |
| 4764 | ENSP00000417182 | DPCR1 | 3.150E-06 | -0.128 | - | - |
| 4765 | ENSP00000307142 | TEX37 | 2.325E-05 | -0.128 | - | - |
| 4766 | ENSP00000354223 | MPC1 | 2.637E-05 | -0.128 | - | - |
| 4767 | ENSP00000407818 | USP46 | 3.929E-05 | -0.128 | - | - |
| 4768 | ENSP00000219548 | STUB1 | 4.479E-05 | -0.128 | - | - |
| 4769 | ENSP00000331152 | MXI1 | 1.113E-04 | -0.128 | - | - |
| 4770 | ENSP00000369050 | CYP1A1 | 6.761E-05 | -0.129 | - | - |
| 4771 | ENSP00000446514 | TMEM235 | 4.008E-08 | -0.129 | - | - |
| 4772 | ENSP00000301336 | RILP | 3.435E-05 | -0.129 | - | - |
| 4773 | ENSP00000294816 | LMX1A | 2.834E-05 | -0.129 | - | - |
| 4774 | ENSP00000352671 | OSCAR | 2.157E-05 | -0.129 | - | - |
| 4775 | ENSP00000365931 | SP2 | 3.244E-05 | -0.129 | - | - |
| 4776 | ENSP00000245817 | TNFSF9 | 3.840E-04 | -0.129 | - | - |
| 4777 | ENSP00000348828 | ARHGEF26 | 2.495E-05 | -0.129 | - | - |
| 4778 | ENSP00000313875 | CD46 | 1.983E-04 | -0.129 | - | - |
| 4779 | ENSP00000308901 | MRPL45 | 3.251E-05 | -0.129 | - | - |
| 4780 | ENSP00000387380 | NECAB1 | 4.991E-05 | -0.129 | - | - |
| 4781 | ENSP00000230538 | LAMA4 | 6.593E-05 | -0.129 | - | - |
| 4782 | ENSP00000304930 | SOSTDC1 | 2.305E-05 | -0.129 | - | - |
| 4783 | ENSP00000251921 | NXPE1 | 4.137E-05 | -0.129 | - | - |
| 4784 | ENSP00000302569 | CLEC7A | 1.350E-04 | -0.129 | - | - |
| 4785 | ENSP00000346206 | TAP1 | 3.445E-05 | -0.129 | - | - |
| 4786 | ENSP00000267017 | NPFF | 5.397E-05 | -0.130 | - | - |
| 4787 | ENSP00000264568 | BMPR1B | 7.121E-05 | -0.130 | - | - |
| 4788 | ENSP00000325618 | PARP10 | 3.450E-05 | -0.130 | - | - |
| 4789 | ENSP00000369810 | VPS16 | 3.411E-05 | -0.130 | - | - |
| 4790 | ENSP00000381105 | NPS | 8.801E-05 | -0.130 | - | - |
| 4791 | ENSP00000296440 | PLXNB1 | 4.266E-05 | -0.130 | - | - |
| 4792 | ENSP00000419371 | AZI2 | 3.146E-05 | -0.130 | - | - |
| 4793 | ENSP00000308699 | REC8 | 2.703E-05 | -0.130 | - | - |
| 4794 | ENSP00000396749 | KANSL3 | 1.736E-05 | -0.130 | - | - |
| 4795 | ENSP00000318799 | BHLHE22 | 1.850E-05 | -0.130 | - | - |
| 4796 | ENSP00000398273 | SOX5 | 2.449E-05 | -0.130 | - | - |
| 4797 | ENSP00000347184 | HTT | 6.396E-05 | -0.130 | - | - |
| 4798 | ENSP00000215886 | LGALS2 | 1.612E-05 | -0.130 | - | - |
| 4799 | ENSP00000361483 | GPR107 | 2.464E-05 | -0.130 | - | - |
| 4800 | ENSP00000378851 | MAPK1IP1L | 3.025E-05 | -0.130 | - | - |
| 4801 | ENSP00000291759 | LILRA4 | 1.340E-04 | -0.130 | - | - |
| 4802 | ENSP00000311290 | PROP1 | 2.910E-05 | -0.130 | - | - |
| 4803 | ENSP00000245620 | LILRB3 | 1.340E-04 | -0.130 | - | - |
| 4804 | ENSP00000465021 | RP11-127I20.4 | 1.721E-07 | -0.130 | - | - |
| 4805 | ENSP00000297313 | RGS20 | 6.192E-05 | -0.130 | - | - |
| 4806 | ENSP00000356655 | ECT2L | 1.105E-05 | -0.130 | - | - |
| 4807 | ENSP00000407521 | NUTM2E | 4.997E-07 | -0.131 | - | - |
| 4808 | ENSP00000331871 | RHD | 3.467E-05 | -0.131 | - | - |
| 4809 | ENSP00000375660 | ZNF320 | 2.790E-05 | -0.131 | - | - |
| 4810 | ENSP00000290942 | TPPP3 | 9.541E-05 | -0.131 | - | - |
| 4811 | ENSP00000251973 | CARD10 | 4.571E-05 | -0.131 | - | - |
| 4812 | ENSP00000358310 | ITGA10 | 2.392E-05 | -0.131 | - | - |
| 4813 | ENSP00000367878 | C11orf49 | 7.933E-06 | -0.131 | - | - |
| 4814 | ENSP00000384640 | MGC10955 | 1.422E-07 | -0.131 | - | - |
| 4815 | ENSP00000385720 | ARHGAP35 | 3.438E-05 | -0.131 | - | - |
| 4816 | ENSP00000295834 | FABP1 | 2.538E-05 | -0.131 | - | - |
| 4817 | ENSP00000337838 | RTN4 | 5.195E-05 | -0.131 | - | - |
| 4818 | ENSP00000053867 | GRN | 5.309E-05 | -0.131 | - | - |
| 4819 | ENSP00000371923 | RP1L1 | 4.192E-05 | -0.131 | - | - |
| 4820 | ENSP00000352047 | GULP1 | 2.533E-05 | -0.131 | - | - |
| 4821 | ENSP00000320219 | ARSJ | 6.577E-06 | -0.131 | - | - |
| 4822 | ENSP00000261523 | RORA | 1.071E-04 | -0.131 | - | - |
| 4823 | ENSP00000234198 | DLX2 | 4.765E-05 | -0.132 | - | - |
| 4824 | ENSP00000338358 | SERPINB6 | 7.437E-05 | -0.132 | - | - |
| 4825 | ENSP00000442365 | EML2 | 8.459E-05 | -0.132 | - | - |
| 4826 | ENSP00000451998 | EML5 | 2.243E-05 | -0.132 | - | - |
| 4827 | ENSP00000385432 | SAR1B | 4.506E-05 | -0.132 | - | - |
| 4828 | ENSP00000014935 | DNASE1L1 | 7.638E-05 | -0.132 | - | - |
| 4829 | ENSP00000352272 | MYOZ1 | 2.738E-05 | -0.132 | - | - |
| 4830 | ENSP00000383894 | MATN3 | 3.716E-05 | -0.132 | - | - |
| 4831 | ENSP00000310003 | CNIH2 | 2.805E-05 | -0.132 | - | - |
| 4832 | ENSP00000343144 | PARD6G | 8.867E-05 | -0.132 | - | - |
| 4833 | ENSP00000200652 | SLC22A4 | 6.493E-05 | -0.132 | - | - |
| 4834 | ENSP00000340937 | COL17A1 | 8.519E-05 | -0.132 | - | - |
| 4835 | ENSP00000316137 | C22orf43 | 2.160E-07 | -0.132 | - | - |
| 4836 | ENSP00000333656 | SIGIRR | 2.548E-04 | -0.132 | - | - |
| 4837 | ENSP00000296438 | FBXW12 | 5.840E-06 | -0.132 | - | - |
| 4838 | ENSP00000226225 | TNFAIP1 | 6.715E-05 | -0.132 | - | - |
| 4839 | ENSP00000264039 | GPC1 | 5.901E-05 | -0.132 | - | - |
| 4840 | ENSP00000277120 | NTRK2 | 4.028E-05 | -0.133 | - | - |
| 4841 | ENSP00000360069 | ZNF831 | 3.430E-05 | -0.133 | - | - |
| 4842 | ENSP00000304331 | GSX1 | 2.921E-05 | -0.133 | - | - |
| 4843 | ENSP00000315557 | C2orf57 | 4.264E-06 | -0.133 | - | - |
| 4844 | ENSP00000296452 | BSN | 7.971E-05 | -0.133 | - | - |
| 4845 | ENSP00000361547 | PRRX2 | 3.350E-05 | -0.133 | - | - |
| 4846 | ENSP00000366525 | FTL | 4.547E-05 | -0.133 | - | - |
| 4847 | ENSP00000217999 | RHOXF1 | 3.691E-05 | -0.133 | - | - |
| 4848 | ENSP00000211287 | MAPK13 | 2.195E-05 | -0.133 | - | - |
| 4849 | ENSP00000365351 | ITGBL1 | 2.436E-05 | -0.133 | - | - |
| 4850 | ENSP00000472384 | MIA | 1.109E-05 | -0.133 | - | - |
| 4851 | ENSP00000437532 | C16orf93 | 9.855E-08 | -0.133 | - | - |
| 4852 | ENSP00000446205 | HES7 | 2.065E-05 | -0.133 | - | - |
| 4853 | ENSP00000309515 | PHYHD1 | 1.010E-04 | -0.133 | - | - |
| 4854 | ENSP00000359531 | GTF2B | 4.355E-05 | -0.133 | - | - |
| 4855 | ENSP00000322909 | FHL2 | 9.001E-05 | -0.133 | - | - |
| 4856 | ENSP00000249776 | KNSTRN | 5.629E-05 | -0.133 | - | - |
| 4857 | ENSP00000459028 | OR1D5 | 1.352E-06 | -0.133 | - | - |
| 4858 | ENSP00000367615 | APRT | 3.865E-05 | -0.134 | - | - |
| 4859 | ENSP00000236877 | SLC8A2 | 3.569E-05 | -0.134 | - | - |
| 4860 | ENSP00000217260 | RSPO4 | 3.893E-05 | -0.134 | - | - |
| 4861 | ENSP00000264167 | AGPS | 5.690E-05 | -0.134 | - | - |
| 4862 | ENSP00000351157 | TXNDC15 | 9.141E-05 | -0.134 | - | - |
| 4863 | ENSP00000298552 | TSC1 | 6.593E-05 | -0.134 | - | - |
| 4864 | ENSP00000375234 | KRTAP4-9 | 3.378E-06 | -0.134 | - | - |
| 4865 | ENSP00000304311 | REG3A | 1.477E-04 | -0.134 | - | - |
| 4866 | ENSP00000423933 | THUMPD2 | 2.262E-05 | -0.134 | - | - |
| 4867 | ENSP00000248935 | GSTT1 | 3.203E-05 | -0.134 | - | - |
| 4868 | ENSP00000385467 | C22orf46 | 3.206E-07 | -0.134 | - | - |
| 4869 | ENSP00000333298 | LAMP1 | 1.121E-04 | -0.134 | - | - |
| 4870 | ENSP00000279101 | CABLES2 | 3.911E-05 | -0.135 | - | - |
| 4871 | ENSP00000353796 | ANKRD53 | 2.733E-05 | -0.135 | - | - |
| 4872 | ENSP00000226578 | MANBA | 1.891E-05 | -0.135 | - | - |
| 4873 | ENSP00000418112 | PTPRG | 4.423E-05 | -0.135 | - | - |
| 4874 | ENSP00000358497 | RNGTT | 3.207E-05 | -0.135 | - | - |
| 4875 | ENSP00000318884 | FMN2 | 3.705E-05 | -0.135 | - | - |
| 4876 | ENSP00000269445 | DYM | 1.377E-04 | -0.135 | - | - |
| 4877 | ENSP00000360411 | LDLRAD1 | 2.368E-05 | -0.135 | - | - |
| 4878 | ENSP00000356133 | CLDN20 | 2.666E-05 | -0.135 | - | - |
| 4879 | ENSP00000307156 | LAMB2 | 5.294E-05 | -0.135 | - | - |
| 4880 | ENSP00000242067 | BBS9 | 4.212E-05 | -0.135 | - | - |
| 4881 | ENSP00000370391 | FAM9A | 2.367E-05 | -0.135 | - | - |
| 4882 | ENSP00000301729 | ECI1 | 3.900E-05 | -0.135 | - | - |
| 4883 | ENSP00000328423 | C15orf60 | 3.987E-05 | -0.135 | - | - |
| 4884 | ENSP00000282957 | CPB1 | 3.637E-05 | -0.135 | - | - |
| 4885 | ENSP00000324767 | CHDC2 | 7.522E-06 | -0.135 | - | - |
| 4886 | ENSP00000260442 | BCL2L10 | 1.626E-04 | -0.135 | - | - |
| 4887 | ENSP00000375683 | KLK7 | 2.986E-05 | -0.135 | - | - |
| 4888 | ENSP00000384651 | MTMR3 | 3.525E-05 | -0.135 | - | - |
| 4889 | ENSP00000215659 | MAPK12 | 2.193E-05 | -0.135 | - | - |
| 4890 | ENSP00000251390 | LILRA3 | 1.340E-04 | -0.135 | - | - |
| 4891 | ENSP00000298556 | HPRT1 | 6.257E-05 | -0.135 | - | - |
| 4892 | ENSP00000340526 | UTS2B | 2.818E-05 | -0.136 | - | - |
| 4893 | ENSP00000382590 | KRTAP5-4 | 1.487E-05 | -0.136 | - | - |
| 4894 | ENSP00000377900 | LIPF | 4.184E-05 | -0.136 | - | - |
| 4895 | ENSP00000306997 | MYOZ2 | 3.268E-05 | -0.136 | - | - |
| 4896 | ENSP00000357465 | TDRD10 | 1.719E-06 | -0.136 | - | - |
| 4897 | ENSP00000343023 | SP100 | 7.444E-05 | -0.136 | - | - |
| 4898 | ENSP00000407749 | FAM25G | 1.271E-07 | -0.136 | - | - |
| 4899 | ENSP00000413896 | FAM25B | 1.271E-07 | -0.136 | - | - |
| 4900 | ENSP00000257724 | MDFIC | 3.946E-05 | -0.136 | - | - |
| 4901 | ENSP00000350369 | MAFG | 6.656E-05 | -0.136 | - | - |
| 4902 | ENSP00000411552 | CDKN1C | 6.917E-05 | -0.136 | - | - |
| 4903 | ENSP00000419502 | FAM211A | 3.943E-06 | -0.136 | - | - |
| 4904 | ENSP00000464272 | FAM25E | 1.271E-07 | -0.136 | - | - |
| 4905 | ENSP00000385551 | ETV1 | 4.518E-05 | -0.136 | - | - |
| 4906 | ENSP00000345333 | ZNF69 | 2.234E-05 | -0.136 | - | - |
| 4907 | ENSP00000245185 | MT2A | 1.314E-04 | -0.137 | - | - |
| 4908 | ENSP00000260128 | SULF1 | 2.347E-05 | -0.137 | - | - |
| 4909 | ENSP00000339723 | CIR1 | 3.682E-05 | -0.137 | - | - |
| 4910 | ENSP00000278601 | C11orf52 | 2.758E-08 | -0.137 | - | - |
| 4911 | ENSP00000367193 | FBXO48 | 1.340E-07 | -0.137 | - | - |
| 4912 | ENSP00000302814 | OTP | 2.787E-05 | -0.137 | - | - |
| 4913 | ENSP00000380313 | MLST8 | 4.638E-05 | -0.137 | - | - |
| 4914 | ENSP00000266556 | TAPBPL | 1.677E-04 | -0.137 | - | - |
| 4915 | ENSP00000370088 | AMELX | 4.585E-05 | -0.137 | - | - |
| 4916 | ENSP00000312411 | PPM1E | 2.380E-05 | -0.137 | - | - |
| 4917 | ENSP00000228476 | DAO | 2.235E-05 | -0.137 | - | - |
| 4918 | ENSP00000367284 | FUNDC1 | 2.918E-05 | -0.137 | - | - |
| 4919 | ENSP00000469799 | AC091948.1 | 1.877E-07 | -0.137 | - | - |
| 4920 | ENSP00000472367 | HUG1 | 3.091E-07 | -0.137 | - | - |
| 4921 | ENSP00000250457 | EGLN3 | 6.363E-05 | -0.137 | - | - |
| 4922 | ENSP00000379704 | 3-Sep | 4.654E-05 | -0.137 | - | - |
| 4923 | ENSP00000261731 | LHX5 | 2.571E-05 | -0.137 | - | - |
| 4924 | ENSP00000452120 | ITGA7 | 2.785E-05 | -0.138 | - | - |
| 4925 | ENSP00000225174 | PPIF | 2.941E-05 | -0.138 | - | - |
| 4926 | ENSP00000292641 | SCGB3A1 | 3.974E-05 | -0.138 | - | - |
| 4927 | ENSP00000353292 | SCNN1A | 3.266E-05 | -0.138 | - | - |
| 4928 | ENSP00000328169 | JAG2 | 4.902E-05 | -0.138 | - | - |
| 4929 | ENSP00000377176 | SPATA7 | 2.689E-05 | -0.138 | - | - |
| 4930 | ENSP00000327459 | APOBEC3B | 2.660E-04 | -0.138 | - | - |
| 4931 | ENSP00000457904 | GOLGA8Q | 7.522E-06 | -0.138 | - | - |
| 4932 | ENSP00000314223 | GPR88 | 3.309E-05 | -0.138 | - | - |
| 4933 | ENSP00000306185 | ANTXR2 | 5.290E-05 | -0.138 | - | - |
| 4934 | ENSP00000396627 | - | 1.905E-05 | -0.138 | - | - |
| 4935 | ENSP00000310829 | TTC18 | 3.092E-05 | -0.138 | - | - |
| 4936 | ENSP00000349932 | PTPRS | 3.215E-05 | -0.138 | - | - |
| 4937 | ENSP00000319730 | PRSS8 | 2.079E-05 | -0.138 | - | - |
| 4938 | ENSP00000227471 | UNC93B1 | 1.404E-04 | -0.138 | - | - |
| 4939 | ENSP00000261047 | GUCA1C | 2.203E-05 | -0.138 | - | - |
| 4940 | ENSP00000352455 | MFAP5 | 4.546E-05 | -0.139 | - | - |
| 4941 | ENSP00000274793 | PLA2G7 | 7.971E-05 | -0.139 | - | - |
| 4942 | ENSP00000346886 | GABPA | 4.625E-05 | -0.139 | - | - |
| 4943 | ENSP00000370767 | GP2 | 4.427E-05 | -0.139 | - | - |
| 4944 | ENSP00000331214 | C5orf50 | 1.041E-05 | -0.139 | - | - |
| 4945 | ENSP00000378548 | GPR135 | 3.990E-05 | -0.139 | - | - |
| 4946 | ENSP00000424058 | SPDYE2L | 2.986E-08 | -0.139 | - | - |
| 4947 | ENSP00000366731 | SPATA31A4 | 1.938E-08 | -0.139 | - | - |
| 4948 | ENSP00000362807 | PEF1 | 2.836E-05 | -0.139 | - | - |
| 4949 | ENSP00000267082 | ITGB7 | 6.799E-05 | -0.139 | - | - |
| 4950 | ENSP00000354219 | TPM2 | 2.973E-05 | -0.139 | - | - |
| 4951 | ENSP00000420384 | CST7 | 2.811E-05 | -0.139 | - | - |
| 4952 | ENSP00000366352 | OR11A1 | 1.554E-04 | -0.139 | - | - |
| 4953 | ENSP00000235332 | MIIP | 1.311E-04 | -0.139 | - | - |
| 4954 | ENSP00000396045 | PLA2G4B | 3.363E-05 | -0.139 | - | - |
| 4955 | ENSP00000406957 | SPATA31A2 | 1.938E-08 | -0.139 | - | - |
| 4956 | ENSP00000352657 | ME3 | 2.623E-05 | -0.139 | - | - |
| 4957 | ENSP00000250937 | DOHH | 6.604E-05 | -0.139 | - | - |
| 4958 | ENSP00000356198 | CHIT1 | 4.876E-05 | -0.139 | - | - |
| 4959 | ENSP00000366847 | SPATA31A5 | 1.938E-08 | -0.139 | - | - |
| 4960 | ENSP00000383558 | GCGR | 2.777E-05 | -0.139 | - | - |
| 4961 | ENSP00000222305 | USF2 | 6.818E-05 | -0.139 | - | - |
| 4962 | ENSP00000463047 | RP11-111H3.1 | 9.354E-07 | -0.139 | - | - |
| 4963 | ENSP00000314807 | IL1RAP | 2.586E-04 | -0.139 | - | - |
| 4964 | ENSP00000305152 | PLP1 | 2.870E-05 | -0.140 | - | - |
| 4965 | ENSP00000306099 | FGB | 8.559E-05 | -0.140 | - | - |
| 4966 | ENSP00000354476 | SREBF2 | 4.253E-05 | -0.140 | - | - |
| 4967 | ENSP00000345512 | SEMA6A | 2.877E-05 | -0.140 | - | - |
| 4968 | ENSP00000360455 | RHOXF2B | 2.729E-05 | -0.140 | - | - |
| 4969 | ENSP00000295934 | HESX1 | 3.367E-05 | -0.140 | - | - |
| 4970 | ENSP00000265381 | APBA1 | 3.134E-05 | -0.140 | - | - |
| 4971 | ENSP00000401653 | DRGX | 3.000E-05 | -0.140 | - | - |
| 4972 | ENSP00000310431 | LMAN1L | 2.730E-05 | -0.140 | - | - |
| 4973 | ENSP00000369963 | C20orf141 | 2.692E-04 | -0.140 | - | - |
| 4974 | ENSP00000260743 | CALHM2 | 4.299E-05 | -0.140 | - | - |
| 4975 | ENSP00000387941 | IFFO2 | 2.047E-05 | -0.140 | - | - |
| 4976 | ENSP00000228468 | ASIC1 | 1.628E-05 | -0.140 | - | - |
| 4977 | ENSP00000368315 | MAGEB5 | 2.592E-05 | -0.140 | - | - |
| 4978 | ENSP00000345392 | ZNF2 | 2.462E-05 | -0.140 | - | - |
| 4979 | ENSP00000225402 | AATF | 3.054E-05 | -0.140 | - | - |
| 4980 | ENSP00000441828 | HPR | 3.466E-05 | -0.140 | - | - |
| 4981 | ENSP00000266088 | SLC5A1 | 1.833E-05 | -0.140 | - | - |
| 4982 | ENSP00000271588 | HMCN1 | 1.089E-04 | -0.140 | - | - |
| 4983 | ENSP00000375656 | ZNF321P | 2.887E-05 | -0.140 | - | - |
| 4984 | ENSP00000354609 | CNKSR1 | 3.816E-05 | -0.140 | - | - |
| 4985 | ENSP00000350874 | METTL9 | 3.532E-05 | -0.140 | - | - |
| 4986 | ENSP00000288350 | LCA5L | 8.103E-05 | -0.140 | - | - |
| 4987 | ENSP00000302599 | FUT9 | 1.810E-05 | -0.140 | - | - |
| 4988 | ENSP00000244458 | PACSIN1 | 3.102E-05 | -0.141 | - | - |
| 4989 | ENSP00000386499 | C16orf11 | 2.649E-07 | -0.141 | - | - |
| 4990 | ENSP00000334813 | RAX | 2.911E-05 | -0.141 | - | - |
| 4991 | ENSP00000269980 | BCKDHA | 4.566E-05 | -0.141 | - | - |
| 4992 | ENSP00000330523 | CTHRC1 | 4.006E-05 | -0.141 | - | - |
| 4993 | ENSP00000320038 | ARHGAP33 | 3.848E-05 | -0.141 | - | - |
| 4994 | ENSP00000294952 | PPP1R21 | 5.640E-05 | -0.141 | - | - |
| 4995 | ENSP00000395962 | MAGED4B | 1.435E-05 | -0.141 | - | - |
| 4996 | ENSP00000262803 | UPF1 | 1.217E-04 | -0.141 | - | - |
| 4997 | ENSP00000361850 | PLAU | 1.005E-04 | -0.141 | - | - |
| 4998 | ENSP00000277657 | CCDC7 | 1.897E-04 | -0.141 | - | - |
| 4999 | ENSP00000381339 | GNAT3 | 7.715E-05 | -0.141 | - | - |
| 5000 | ENSP00000374558 | KIAA0226L | 5.060E-05 | -0.141 | - | - |
| 5001 | ENSP00000304762 | SPACA5B | 4.140E-05 | -0.141 | - | - |
| 5002 | ENSP00000305847 | SPACA5 | 4.140E-05 | -0.141 | - | - |
| 5003 | ENSP00000301146 | CYP2A7 | 3.352E-05 | -0.142 | - | - |
| 5004 | ENSP00000374049 | VWA3A | 1.172E-05 | -0.142 | - | - |
| 5005 | ENSP00000363168 | AGAP6 | 2.248E-05 | -0.142 | - | - |
| 5006 | ENSP00000420381 | CALU | 5.357E-05 | -0.142 | - | - |
| 5007 | ENSP00000352138 | KIRREL | 4.296E-05 | -0.142 | - | - |
| 5008 | ENSP00000265732 | RBM48 | 6.292E-05 | -0.142 | - | - |
| 5009 | ENSP00000384408 | PARG | 2.320E-05 | -0.142 | - | - |
| 5010 | ENSP00000357660 | CLRN3 | 2.568E-05 | -0.142 | - | - |
| 5011 | ENSP00000457654 | RP11-77K12.1 | 6.508E-08 | -0.142 | - | - |
| 5012 | ENSP00000360730 | IL13RA1 | 2.302E-04 | -0.142 | - | - |
| 5013 | ENSP00000221462 | PPP1R37 | 5.450E-05 | -0.142 | - | - |
| 5014 | ENSP00000373477 | GPX3 | 4.238E-05 | -0.142 | - | - |
| 5015 | ENSP00000222219 | DNASE2 | 7.469E-05 | -0.142 | - | - |
| 5016 | ENSP00000251473 | DKFZP761J1410 | 3.296E-05 | -0.142 | - | - |
| 5017 | ENSP00000349041 | NRARP | 2.590E-05 | -0.142 | - | - |
| 5018 | ENSP00000440765 | C16orf13 | 4.589E-05 | -0.142 | - | - |
| 5019 | ENSP00000312856 | LMO3 | 2.782E-05 | -0.142 | - | - |
| 5020 | ENSP00000380467 | JMJD7 | 1.916E-05 | -0.142 | - | - |
| 5021 | ENSP00000361558 | NTMT1 | 2.980E-05 | -0.142 | - | - |
| 5022 | ENSP00000325663 | NFKBIZ | 3.659E-05 | -0.143 | - | - |
| 5023 | ENSP00000358807 | ALX3 | 3.260E-05 | -0.143 | - | - |
| 5024 | ENSP00000284202 | IMPACT | 5.839E-05 | -0.143 | - | - |
| 5025 | ENSP00000371872 | SLC35G5 | 2.725E-05 | -0.143 | - | - |
| 5026 | ENSP00000357304 | KIAA0907 | 3.498E-05 | -0.143 | - | - |
| 5027 | ENSP00000377854 | LHX6 | 2.534E-05 | -0.143 | - | - |
| 5028 | ENSP00000262903 | HACE1 | 2.329E-05 | -0.143 | - | - |
| 5029 | ENSP00000415452 | AGAP8 | 2.077E-05 | -0.143 | - | - |
| 5030 | ENSP00000348170 | HP | 8.756E-05 | -0.143 | - | - |
| 5031 | ENSP00000380177 | HIST1H2BN | 3.264E-05 | -0.143 | - | - |
| 5032 | ENSP00000375593 | ZSCAN5A | 4.300E-05 | -0.143 | - | - |
| 5033 | ENSP00000359498 | FMR1NB | 1.301E-07 | -0.143 | - | - |
| 5034 | ENSP00000403683 | NABP1 | 7.383E-05 | -0.143 | - | - |
| 5035 | ENSP00000396211 | WASF2 | 3.407E-05 | -0.143 | - | - |
| 5036 | ENSP00000390224 | C1orf132 | 1.225E-05 | -0.143 | - | - |
| 5037 | ENSP00000362457 | MAGEE2 | 1.740E-05 | -0.143 | - | - |
| 5038 | ENSP00000456163 | MIR3654 | 2.205E-05 | -0.143 | - | - |
| 5039 | ENSP00000295600 | MITF | 4.575E-05 | -0.143 | - | - |
| 5040 | ENSP00000312235 | MUC13 | 1.100E-04 | -0.143 | - | - |
| 5041 | ENSP00000245105 | SH3TC1 | 3.709E-05 | -0.143 | - | - |
| 5042 | ENSP00000359820 | FAM122C | 5.008E-05 | -0.143 | - | - |
| 5043 | ENSP00000300057 | MESP1 | 2.885E-05 | -0.143 | - | - |
| 5044 | ENSP00000238558 | GSC | 3.792E-05 | -0.143 | - | - |
| 5045 | ENSP00000357082 | C1orf204 | 1.023E-05 | -0.143 | - | - |
| 5046 | ENSP00000255499 | RNF128 | 1.570E-04 | -0.143 | - | - |
| 5047 | ENSP00000357625 | BNIP3 | 4.112E-05 | -0.143 | - | - |
| 5048 | ENSP00000455952 | RP13-279N23.2 | 1.316E-05 | -0.143 | - | - |
| 5049 | ENSP00000454437 | RP4-576H24.4 | 2.513E-06 | -0.143 | - | - |
| 5050 | ENSP00000346127 | PLEKHB1 | 2.206E-05 | -0.143 | - | - |
| 5051 | ENSP00000382026 | C4orf3 | 2.578E-07 | -0.143 | - | - |
| 5052 | ENSP00000388731 | SSFA2 | 9.457E-06 | -0.143 | - | - |
| 5053 | ENSP00000468236 | APOC4 | 1.008E-05 | -0.144 | - | - |
| 5054 | ENSP00000342216 | ABCA9 | 2.797E-05 | -0.144 | - | - |
| 5055 | ENSP00000300961 | JSRP1 | 3.230E-05 | -0.144 | - | - |
| 5056 | ENSP00000434385 | ADIG | 1.813E-05 | -0.144 | - | - |
| 5057 | ENSP00000298705 | PPP1R36 | 8.355E-06 | -0.144 | - | - |
| 5058 | ENSP00000339511 | CXorf27 | 2.033E-05 | -0.144 | - | - |
| 5059 | ENSP00000396301 | IK | 4.250E-05 | -0.144 | - | - |
| 5060 | ENSP00000367318 | RGP1 | 3.432E-05 | -0.144 | - | - |
| 5061 | ENSP00000347464 | GIT2 | 2.798E-05 | -0.144 | - | - |
| 5062 | ENSP00000300404 | B4GALNT2 | 2.970E-05 | -0.144 | - | - |
| 5063 | ENSP00000363081 | DKK1 | 7.400E-05 | -0.144 | - | - |
| 5064 | ENSP00000340568 | COX8C | 2.627E-05 | -0.144 | - | - |
| 5065 | ENSP00000340983 | LRRC25 | 2.238E-05 | -0.144 | - | - |
| 5066 | ENSP00000330720 | KRTAP11-1 | 1.543E-05 | -0.144 | - | - |
| 5067 | ENSP00000284987 | ADAMTS5 | 1.130E-04 | -0.144 | - | - |
| 5068 | ENSP00000367972 | ID4 | 3.011E-05 | -0.144 | - | - |
| 5069 | ENSP00000302648 | NRTN | 2.581E-05 | -0.144 | - | - |
| 5070 | ENSP00000293778 | CXCL16 | 1.221E-04 | -0.145 | - | - |
| 5071 | ENSP00000263846 | SYT7 | 2.306E-05 | -0.145 | - | - |
| 5072 | ENSP00000270279 | CBLC | 1.251E-04 | -0.145 | - | - |
| 5073 | ENSP00000297537 | GBX1 | 2.821E-05 | -0.145 | - | - |
| 5074 | ENSP00000272163 | LBR | 3.575E-05 | -0.145 | - | - |
| 5075 | ENSP00000210633 | SEMA4G | 3.337E-05 | -0.145 | - | - |
| 5076 | ENSP00000344967 | SPRY4 | 1.749E-05 | -0.145 | - | - |
| 5077 | ENSP00000354671 | MAST2 | 4.304E-05 | -0.145 | - | - |
| 5078 | ENSP00000315417 | ALX1 | 2.331E-05 | -0.145 | - | - |
| 5079 | ENSP00000360540 | CEP55 | 4.771E-05 | -0.145 | - | - |
| 5080 | ENSP00000371271 | PRH2 | 1.340E-06 | -0.145 | - | - |
| 5081 | ENSP00000371994 | NYNRIN | 4.486E-05 | -0.145 | - | - |
| 5082 | ENSP00000382957 | ZNF705D | 3.668E-05 | -0.145 | - | - |
| 5083 | ENSP00000313021 | MAK | 4.327E-05 | -0.145 | - | - |
| 5084 | ENSP00000345826 | PMEPA1 | 3.255E-05 | -0.145 | - | - |
| 5085 | ENSP00000435010 | DEFB134 | 3.562E-06 | -0.145 | - | - |
| 5086 | ENSP00000315757 | LCP1 | 4.559E-05 | -0.145 | - | - |
| 5087 | ENSP00000424007 | CTC-534A2.2 | 1.805E-07 | -0.146 | - | - |
| 5088 | ENSP00000216802 | PSME2 | 9.259E-05 | -0.146 | - | - |
| 5089 | ENSP00000362978 | EYA3 | 2.004E-05 | -0.146 | - | - |
| 5090 | ENSP00000350260 | FAM219B | 1.374E-01 | -0.146 | - | - |
| 5091 | ENSP00000286349 | TRIM42 | 4.500E-05 | -0.146 | - | - |
| 5092 | ENSP00000309402 | TRAM1L1 | 3.848E-05 | -0.146 | - | - |
| 5093 | ENSP00000324884 | HOXA3 | 2.778E-05 | -0.146 | - | - |
| 5094 | ENSP00000351209 | EPHA2 | 5.878E-05 | -0.146 | - | - |
| 5095 | ENSP00000378710 | FAM135B | 2.115E-05 | -0.146 | - | - |
| 5096 | ENSP00000368017 | ZFC3H1 | 4.192E-05 | -0.146 | - | - |
| 5097 | ENSP00000457962 | TGFBR3L | 9.771E-07 | -0.146 | - | - |
| 5098 | ENSP00000232501 | NPRL2 | 1.265E-04 | -0.146 | - | - |
| 5099 | ENSP00000344820 | KCNK7 | 2.973E-05 | -0.146 | - | - |
| 5100 | ENSP00000355718 | DLL1 | 7.175E-05 | -0.146 | - | - |
| 5101 | ENSP00000353030 | PTPRF | 3.438E-05 | -0.146 | - | - |
| 5102 | ENSP00000270328 | ADAMTS10 | 4.164E-05 | -0.146 | - | - |
| 5103 | ENSP00000293261 | TMEM143 | 4.706E-05 | -0.146 | - | - |
| 5104 | ENSP00000326272 | FOXL1 | 3.139E-05 | -0.146 | - | - |
| 5105 | ENSP00000368138 | TRIQK | 5.109E-05 | -0.146 | - | - |
| 5106 | ENSP00000369547 | S100G | 2.477E-05 | -0.146 | - | - |
| 5107 | ENSP00000391453 | PARVG | 3.804E-05 | -0.146 | - | - |
| 5108 | ENSP00000359368 | RPAP2 | 5.333E-05 | -0.146 | - | - |
| 5109 | ENSP00000399075 | C18orf42 | 1.067E-06 | -0.146 | - | - |
| 5110 | ENSP00000414517 | MAMLD1 | 9.390E-07 | -0.147 | - | - |
| 5111 | ENSP00000367065 | HES2 | 2.309E-05 | -0.147 | - | - |
| 5112 | ENSP00000319141 | CYBRD1 | 6.476E-05 | -0.147 | - | - |
| 5113 | ENSP00000358413 | ZDHHC6 | 2.639E-05 | -0.147 | - | - |
| 5114 | ENSP00000302176 | ASPSCR1 | 6.519E-05 | -0.147 | - | - |
| 5115 | ENSP00000362376 | C10orf35 | 3.808E-05 | -0.147 | - | - |
| 5116 | ENSP00000370420 | VCX3B | 2.865E-05 | -0.147 | - | - |
| 5117 | ENSP00000381198 | KIAA1468 | 1.728E-05 | -0.147 | - | - |
| 5118 | ENSP00000347684 | LMX1B | 3.112E-05 | -0.147 | - | - |
| 5119 | ENSP00000225728 | MED31 | 6.357E-05 | -0.147 | - | - |
| 5120 | ENSP00000295908 | PPM1K | 2.119E-05 | -0.147 | - | - |
| 5121 | ENSP00000358131 | OTUD7B | 4.099E-05 | -0.147 | - | - |
| 5122 | ENSP00000470473 | MROH5 | 1.323E-07 | -0.147 | - | - |
| 5123 | ENSP00000345405 | SPRED3 | 2.430E-05 | -0.147 | - | - |
| 5124 | ENSP00000262965 | TCF3 | 8.148E-05 | -0.147 | - | - |
| 5125 | ENSP00000349131 | RSPO3 | 4.670E-05 | -0.147 | - | - |
| 5126 | ENSP00000334448 | GNG2 | 6.366E-05 | -0.147 | - | - |
| 5127 | ENSP00000318077 | MC5R | 2.761E-05 | -0.147 | - | - |
| 5128 | ENSP00000428230 | C8orf17 | 2.964E-06 | -0.147 | - | - |
| 5129 | ENSP00000299502 | SERPINB2 | 1.113E-04 | -0.147 | - | - |
| 5130 | ENSP00000349244 | C10orf137 | 1.219E-05 | -0.147 | - | - |
| 5131 | ENSP00000367570 | PHF11 | 4.587E-05 | -0.147 | - | - |
| 5132 | ENSP00000264613 | CP | 4.876E-05 | -0.147 | - | - |
| 5133 | ENSP00000256935 | DOCK2 | 7.866E-05 | -0.148 | - | - |
| 5134 | ENSP00000244364 | DST | 4.120E-05 | -0.148 | - | - |
| 5135 | ENSP00000366326 | NEBL | 1.094E-05 | -0.148 | - | - |
| 5136 | ENSP00000251808 | GRHL2 | 2.272E-05 | -0.148 | - | - |
| 5137 | ENSP00000261796 | IL17B | 3.803E-04 | -0.148 | - | - |
| 5138 | ENSP00000349486 | SCRIB | 3.725E-05 | -0.148 | - | - |
| 5139 | ENSP00000360441 | RHOXF2 | 2.672E-05 | -0.148 | - | - |
| 5140 | ENSP00000310275 | BANF1 | 4.527E-05 | -0.148 | - | - |
| 5141 | ENSP00000288462 | C9orf43 | 1.825E-05 | -0.148 | - | - |
| 5142 | ENSP00000333203 | SERPINA5 | 4.321E-05 | -0.148 | - | - |
| 5143 | ENSP00000358320 | POLR3GL | 2.410E-05 | -0.148 | - | - |
| 5144 | ENSP00000324763 | WFIKKN1 | 3.505E-05 | -0.148 | - | - |
| 5145 | ENSP00000364643 | BPIFB3 | 3.349E-05 | -0.148 | - | - |
| 5146 | ENSP00000366679 | HIST1H2AH | 5.072E-05 | -0.148 | - | - |
| 5147 | ENSP00000323805 | PALM2 | 1.434E-05 | -0.148 | - | - |
| 5148 | ENSP00000375872 | ATG16L1 | 1.467E-04 | -0.148 | - | - |
| 5149 | ENSP00000235382 | RGS2 | 3.608E-05 | -0.148 | - | - |
| 5150 | ENSP00000268624 | ADAD2 | 9.127E-06 | -0.148 | - | - |
| 5151 | ENSP00000336927 | ALDOA | 4.839E-05 | -0.148 | - | - |
| 5152 | ENSP00000299882 | TMPRSS5 | 3.376E-05 | -0.148 | - | - |
| 5153 | ENSP00000296695 | SPINK1 | 2.059E-05 | -0.148 | - | - |
| 5154 | ENSP00000359573 | LMO4 | 5.575E-05 | -0.148 | - | - |
| 5155 | ENSP00000294638 | LHX8 | 3.194E-05 | -0.148 | - | - |
| 5156 | ENSP00000368623 | C1orf159 | 2.855E-06 | -0.148 | - | - |
| 5157 | ENSP00000265131 | TNC | 1.047E-04 | -0.148 | - | - |
| 5158 | ENSP00000384515 | PARVB | 3.343E-05 | -0.148 | - | - |
| 5159 | ENSP00000384224 | AP000525.1 | 1.698E-06 | -0.148 | - | - |
| 5160 | ENSP00000349678 | MYBPHL | 1.071E-05 | -0.149 | - | - |
| 5161 | ENSP00000359403 | CD99L2 | 2.875E-06 | -0.149 | - | - |
| 5162 | ENSP00000359424 | CHUK | 1.031E-04 | -0.149 | - | - |
| 5163 | ENSP00000356363 | C1orf53 | 3.208E-05 | -0.149 | - | - |
| 5164 | ENSP00000396593 | DYTN | 1.626E-05 | -0.149 | - | - |
| 5165 | ENSP00000275764 | STRA8 | 3.204E-05 | -0.149 | - | - |
| 5166 | ENSP00000362768 | RBL1 | 4.261E-05 | -0.149 | - | - |
| 5167 | ENSP00000356331 | NR5A2 | 2.865E-05 | -0.149 | - | - |
| 5168 | ENSP00000381717 | UBE2D2 | 6.166E-05 | -0.149 | - | - |
| 5169 | ENSP00000406485 | SMTNL1 | 3.913E-05 | -0.149 | - | - |
| 5170 | ENSP00000320447 | NR2C2 | 9.368E-05 | -0.149 | - | - |
| 5171 | ENSP00000328915 | PRAMEF7 | 5.183E-05 | -0.149 | - | - |
| 5172 | ENSP00000252034 | ELN | 4.314E-05 | -0.149 | - | - |
| 5173 | ENSP00000351697 | REV3L | 2.714E-05 | -0.149 | - | - |
| 5174 | ENSP00000382250 | MRPS6 | 6.656E-06 | -0.149 | - | - |
| 5175 | ENSP00000376213 | KIAA1683 | 1.833E-05 | -0.149 | - | - |
| 5176 | ENSP00000362465 | ZDHHC15 | 2.591E-05 | -0.149 | - | - |
| 5177 | ENSP00000226067 | HLF | 2.744E-05 | -0.149 | - | - |
| 5178 | ENSP00000318486 | CDK5R1 | 5.825E-05 | -0.149 | - | - |
| 5179 | ENSP00000348395 | NPEPL1 | 2.397E-05 | -0.149 | - | - |
| 5180 | ENSP00000380270 | C5orf28 | 6.211E-05 | -0.149 | - | - |
| 5181 | ENSP00000181383 | CPB2 | 7.095E-05 | -0.150 | - | - |
| 5182 | ENSP00000328720 | FOXK1 | 2.522E-05 | -0.150 | - | - |
| 5183 | ENSP00000356236 | SYT2 | 2.109E-05 | -0.150 | - | - |
| 5184 | ENSP00000262460 | GINS1 | 5.065E-05 | -0.150 | - | - |
| 5185 | ENSP00000310557 | KCNE3 | 1.248E-05 | -0.150 | - | - |
| 5186 | ENSP00000353794 | FBXL22 | 1.047E-05 | -0.150 | - | - |
| 5187 | ENSP00000375853 | RGS13 | 2.363E-05 | -0.150 | - | - |
| 5188 | ENSP00000283547 | TEX29 | 2.165E-05 | -0.150 | - | - |
| 5189 | ENSP00000360869 | IFIT1 | 5.152E-05 | -0.150 | - | - |
| 5190 | ENSP00000298902 | IFI27 | 1.102E-04 | -0.150 | - | - |
| 5191 | ENSP00000345282 | GPT2 | 1.488E-05 | -0.150 | - | - |
| 5192 | ENSP00000261980 | VSX2 | 2.167E-05 | -0.150 | - | - |
| 5193 | ENSP00000242576 | UNG | 3.630E-05 | -0.150 | - | - |
| 5194 | ENSP00000264025 | PVRL1 | 5.579E-05 | -0.150 | - | - |
| 5195 | ENSP00000228945 | ARHGDIB | 4.585E-05 | -0.150 | - | - |
| 5196 | ENSP00000268122 | RHCG | 2.648E-05 | -0.150 | - | - |
| 5197 | ENSP00000356969 | APOA2 | 2.253E-05 | -0.150 | - | - |
| 5198 | ENSP00000362849 | CTNNA3 | 3.248E-05 | -0.150 | - | - |
| 5199 | ENSP00000326627 | ASIC4 | 1.409E-05 | -0.150 | - | - |
| 5200 | ENSP00000414893 | C9orf53 | 6.137E-06 | -0.150 | - | - |
| 5201 | ENSP00000262630 | ZBTB32 | 3.765E-05 | -0.150 | - | - |
| 5202 | ENSP00000358466 | RAB39B | 2.744E-05 | -0.150 | - | - |
| 5203 | ENSP00000258403 | SLC19A3 | 3.695E-05 | -0.150 | - | - |
| 5204 | ENSP00000372654 | AQP4 | 2.725E-05 | -0.150 | - | - |
| 5205 | ENSP00000435509 | ZIC4 | 3.694E-05 | -0.150 | - | - |
| 5206 | ENSP00000377862 | FAT4 | 4.212E-05 | -0.150 | - | - |
| 5207 | ENSP00000359845 | CCDC160 | 1.798E-07 | -0.150 | - | - |
| 5208 | ENSP00000304065 | C2orf27B | 8.569E-08 | -0.150 | - | - |
| 5209 | ENSP00000405610 | S100A7L2 | 5.199E-06 | -0.150 | - | - |
| 5210 | ENSP00000320209 | CIDEA | 8.340E-05 | -0.150 | - | - |
| 5211 | ENSP00000377751 | SCOC | 1.661E-05 | -0.151 | - | - |
| 5212 | ENSP00000357973 | LACE1 | 8.304E-06 | -0.151 | - | - |
| 5213 | ENSP00000375682 | KLK8 | 3.086E-05 | -0.151 | - | - |
| 5214 | ENSP00000386186 | TIGD1 | 7.300E-06 | -0.151 | - | - |
| 5215 | ENSP00000347581 | AGK | 2.791E-05 | -0.151 | - | - |
| 5216 | ENSP00000318142 | FUT7 | 9.596E-05 | -0.151 | - | - |
| 5217 | ENSP00000328054 | CCDC144NL | 1.854E-05 | -0.151 | - | - |
| 5218 | ENSP00000455681 | RP11-48B14.2 | 7.935E-06 | -0.151 | - | - |
| 5219 | ENSP00000386557 | C19orf24 | 1.077E-06 | -0.151 | - | - |
| 5220 | ENSP00000322532 | PELI3 | 3.171E-04 | -0.151 | - | - |
| 5221 | ENSP00000386722 | FCHSD2 | 5.716E-05 | -0.151 | - | - |
| 5222 | ENSP00000265322 | PECR | 2.394E-05 | -0.151 | - | - |
| 5223 | ENSP00000332413 | BEST3 | 1.019E-05 | -0.152 | - | - |
| 5224 | ENSP00000350195 | MAP3K6 | 2.819E-05 | -0.152 | - | - |
| 5225 | ENSP00000382987 | ZNF705B | 3.667E-05 | -0.152 | - | - |
| 5226 | ENSP00000352627 | HIST1H2AM | 5.261E-05 | -0.152 | - | - |
| 5227 | ENSP00000366787 | CNTNAP3B | 2.192E-05 | -0.152 | - | - |
| 5228 | ENSP00000359119 | SRPK3 | 2.066E-05 | -0.152 | - | - |
| 5229 | ENSP00000418842 | NDUFA6 | 3.810E-05 | -0.152 | - | - |
| 5230 | ENSP00000070846 | PKP2 | 4.889E-05 | -0.152 | - | - |
| 5231 | ENSP00000333395 | ARSI | 8.781E-06 | -0.152 | - | - |
| 5232 | ENSP00000350132 | PLN | 2.829E-05 | -0.152 | - | - |
| 5233 | ENSP00000266771 | SLC15A4 | 8.366E-05 | -0.152 | - | - |
| 5234 | ENSP00000382327 | SDR39U1 | 4.021E-05 | -0.152 | - | - |
| 5235 | ENSP00000380453 | ERMN | 2.784E-05 | -0.152 | - | - |
| 5236 | ENSP00000439128 | BANF2 | 2.455E-05 | -0.152 | - | - |
| 5237 | ENSP00000295984 | PRRT3 | 1.318E-05 | -0.152 | - | - |
| 5238 | ENSP00000348842 | EML6 | 1.647E-05 | -0.152 | - | - |
| 5239 | ENSP00000368547 | FEZ2 | 1.960E-05 | -0.152 | - | - |
| 5240 | ENSP00000325120 | PGR | 6.752E-05 | -0.152 | - | - |
| 5241 | ENSP00000262776 | LGALS3BP | 8.909E-05 | -0.152 | - | - |
| 5242 | ENSP00000280187 | GPM6A | 1.007E-05 | -0.152 | - | - |
| 5243 | ENSP00000339161 | AIDA | 8.875E-06 | -0.152 | - | - |
| 5244 | ENSP00000386935 | UAP1L1 | 4.361E-05 | -0.152 | - | - |
| 5245 | ENSP00000227756 | GALNT18 | 3.124E-05 | -0.152 | - | - |
| 5246 | ENSP00000355050 | CTNNBL1 | 4.065E-05 | -0.152 | - | - |
| 5247 | ENSP00000366950 | KRTAP9-2 | 1.582E-05 | -0.152 | - | - |
| 5248 | ENSP00000307305 | DDIT4 | 8.171E-05 | -0.152 | - | - |
| 5249 | ENSP00000303212 | SEMA3E | 2.651E-05 | -0.152 | - | - |
| 5250 | ENSP00000388457 | CLDND1 | 4.358E-05 | -0.152 | - | - |
| 5251 | ENSP00000369136 | NWD1 | 7.309E-06 | -0.152 | - | - |
| 5252 | ENSP00000305852 | TM4SF4 | 2.491E-05 | -0.152 | - | - |
| 5253 | ENSP00000263268 | MREG | 6.999E-05 | -0.153 | - | - |
| 5254 | ENSP00000348550 | FRMD6 | 1.564E-05 | -0.153 | - | - |
| 5255 | ENSP00000431443 | KRTAP5-9 | 3.899E-05 | -0.153 | - | - |
| 5256 | ENSP00000231751 | LTF | 5.200E-05 | -0.153 | - | - |
| 5257 | ENSP00000331614 | IKZF1 | 9.127E-05 | -0.153 | - | - |
| 5258 | ENSP00000394352 | DIXDC1 | 2.977E-05 | -0.153 | - | - |
| 5259 | ENSP00000421180 | MAML3 | 8.006E-06 | -0.153 | - | - |
| 5260 | ENSP00000359640 | MCOLN2 | 3.290E-05 | -0.153 | - | - |
| 5261 | ENSP00000361855 | SEMG2 | 6.472E-05 | -0.153 | - | - |
| 5262 | ENSP00000257974 | KRT82 | 3.001E-05 | -0.153 | - | - |
| 5263 | ENSP00000470948 | LRLE1 | 8.007E-06 | -0.153 | - | - |
| 5264 | ENSP00000363208 | AGAP7 | 6.890E-06 | -0.153 | - | - |
| 5265 | ENSP00000309007 | THAP6 | 4.176E-05 | -0.153 | - | - |
| 5266 | ENSP00000393776 | FCHO2 | 3.136E-05 | -0.153 | - | - |
| 5267 | ENSP00000269051 | RHBDL3 | 2.355E-05 | -0.153 | - | - |
| 5268 | ENSP00000367787 | LIG3 | 4.318E-05 | -0.153 | - | - |
| 5269 | ENSP00000226247 | FOXN1 | 1.847E-05 | -0.153 | - | - |
| 5270 | ENSP00000406463 | DNAJC7 | 3.350E-05 | -0.153 | - | - |
| 5271 | ENSP00000424711 | CLRN2 | 5.618E-07 | -0.153 | - | - |
| 5272 | ENSP00000338796 | SSX2 | 9.259E-05 | -0.153 | - | - |
| 5273 | ENSP00000346986 | WAC | 1.595E-05 | -0.153 | - | - |
| 5274 | ENSP00000357750 | TRAF3IP2 | 2.737E-04 | -0.153 | - | - |
| 5275 | ENSP00000360688 | TXNDC12 | 2.522E-05 | -0.153 | - | - |
| 5276 | ENSP00000256509 | CHL1 | 4.141E-05 | -0.153 | - | - |
| 5277 | ENSP00000217423 | CST4 | 1.036E-04 | -0.153 | - | - |
| 5278 | ENSP00000339826 | PIK3AP1 | 1.182E-04 | -0.153 | - | - |
| 5279 | ENSP00000408527 | C2orf61 | 2.351E-07 | -0.153 | - | - |
| 5280 | ENSP00000343877 | PAQR5 | 2.791E-05 | -0.154 | - | - |
| 5281 | ENSP00000305343 | SOX14 | 1.649E-05 | -0.154 | - | - |
| 5282 | ENSP00000364212 | CDA | 7.374E-05 | -0.154 | - | - |
| 5283 | ENSP00000431083 | PCDHGA1 | 1.798E-05 | -0.154 | - | - |
| 5284 | ENSP00000250024 | E2F8 | 1.230E-05 | -0.154 | - | - |
| 5285 | ENSP00000265310 | TRPV5 | 2.147E-05 | -0.154 | - | - |
| 5286 | ENSP00000429084 | UBR5 | 6.482E-05 | -0.154 | - | - |
| 5287 | ENSP00000216968 | PROCR | 1.545E-04 | -0.154 | - | - |
| 5288 | ENSP00000368698 | HIVEP1 | 3.086E-05 | -0.154 | - | - |
| 5289 | ENSP00000296137 | FYCO1 | 8.420E-05 | -0.154 | - | - |
| 5290 | ENSP00000278070 | PPRC1 | 6.852E-05 | -0.154 | - | - |
| 5291 | ENSP00000367069 | ZFAT | 3.955E-05 | -0.154 | - | - |
| 5292 | ENSP00000336655 | STRADA | 1.508E-04 | -0.154 | - | - |
| 5293 | ENSP00000259512 | DERL1 | 5.167E-05 | -0.154 | - | - |
| 5294 | ENSP00000250351 | KLK12 | 2.688E-05 | -0.154 | - | - |
| 5295 | ENSP00000370299 | SHROOM2 | 2.761E-05 | -0.154 | - | - |
| 5296 | ENSP00000351608 | PRRT2 | 1.569E-05 | -0.154 | - | - |
| 5297 | ENSP00000371138 | FKBP1A | 6.175E-05 | -0.154 | - | - |
| 5298 | ENSP00000326477 | SETDB2 | 2.300E-05 | -0.154 | - | - |
| 5299 | ENSP00000372651 | C3orf72 | 2.728E-06 | -0.154 | - | - |
| 5300 | ENSP00000359378 | PRRG3 | 1.153E-05 | -0.155 | - | - |
| 5301 | ENSP00000370031 | TTC8 | 2.058E-05 | -0.155 | - | - |
| 5302 | ENSP00000389658 | AC079341.1 | 4.272E-06 | -0.155 | - | - |
| 5303 | ENSP00000260505 | TTLL7 | 1.087E-05 | -0.155 | - | - |
| 5304 | ENSP00000429243 | STMN2 | 3.687E-05 | -0.155 | - | - |
| 5305 | ENSP00000361875 | WFDC5 | 1.471E-05 | -0.155 | - | - |
| 5306 | ENSP00000249389 | OPN1SW | 9.402E-05 | -0.155 | - | - |
| 5307 | ENSP00000252898 | TNNI2 | 2.343E-05 | -0.155 | - | - |
| 5308 | ENSP00000252321 | KCNA5 | 2.516E-05 | -0.155 | - | - |
| 5309 | ENSP00000249042 | TST | 3.025E-05 | -0.155 | - | - |
| 5310 | ENSP00000371825 | DEFB127 | 1.270E-05 | -0.155 | - | - |
| 5311 | ENSP00000294339 | TAL1 | 1.434E-04 | -0.155 | - | - |
| 5312 | ENSP00000342780 | RP11-113D6.10 | 4.564E-06 | -0.155 | - | - |
| 5313 | ENSP00000262643 | CCNE1 | 1.059E-04 | -0.155 | - | - |
| 5314 | ENSP00000334463 | LYPD6 | 2.894E-05 | -0.155 | - | - |
| 5315 | ENSP00000376855 | DPP10 | 3.176E-05 | -0.155 | - | - |
| 5316 | ENSP00000378195 | GPR75 | 4.022E-05 | -0.155 | - | - |
| 5317 | ENSP00000358777 | ATP6AP1 | 2.842E-05 | -0.155 | - | - |
| 5318 | ENSP00000342313 | SAMD11 | 3.744E-05 | -0.155 | - | - |
| 5319 | ENSP00000337950 | LYNX1 | 1.561E-05 | -0.155 | - | - |
| 5320 | ENSP00000297848 | COL14A1 | 4.279E-05 | -0.155 | - | - |
| 5321 | ENSP00000366376 | OR2J1 | 4.211E-06 | -0.155 | - | - |
| 5322 | ENSP00000469669 | AC002985.3 | 1.788E-07 | -0.155 | - | - |
| 5323 | ENSP00000304401 | SLFNL1 | 7.959E-05 | -0.155 | - | - |
| 5324 | ENSP00000360821 | UBAC1 | 1.747E-05 | -0.155 | - | - |
| 5325 | ENSP00000358314 | LIX1L | 2.741E-05 | -0.155 | - | - |
| 5326 | ENSP00000265421 | POLB | 8.103E-05 | -0.155 | - | - |
| 5327 | ENSP00000431971 | SAMD1 | 5.811E-05 | -0.155 | - | - |
| 5328 | ENSP00000268674 | TIGD7 | 8.229E-06 | -0.155 | - | - |
| 5329 | ENSP00000314620 | MS4A1 | 1.188E-04 | -0.155 | - | - |
| 5330 | ENSP00000257915 | TFCP2 | 2.861E-05 | -0.155 | - | - |
| 5331 | ENSP00000341815 | SOX18 | 2.106E-05 | -0.156 | - | - |
| 5332 | ENSP00000382713 | UBE2QL1 | 1.499E-05 | -0.156 | - | - |
| 5333 | ENSP00000328203 | TRAIP | 1.279E-04 | -0.156 | - | - |
| 5334 | ENSP00000359940 | IGSF1 | 2.934E-05 | -0.156 | - | - |
| 5335 | ENSP00000376992 | BCL2L15 | 2.015E-05 | -0.156 | - | - |
| 5336 | ENSP00000331692 | SCRT1 | 2.459E-05 | -0.156 | - | - |
| 5337 | ENSP00000387219 | NFU1 | 2.342E-05 | -0.156 | - | - |
| 5338 | ENSP00000367157 | LRRC3C | 1.314E-05 | -0.156 | - | - |
| 5339 | ENSP00000362551 | ZBTB34 | 4.499E-05 | -0.156 | - | - |
| 5340 | ENSP00000266058 | SLIT1 | 1.896E-05 | -0.156 | - | - |
| 5341 | ENSP00000383020 | ZNF705G | 3.665E-05 | -0.156 | - | - |
| 5342 | ENSP00000365261 | LST1 | 3.793E-07 | -0.156 | - | - |
| 5343 | ENSP00000375748 | FUT2 | 1.911E-05 | -0.156 | - | - |
| 5344 | ENSP00000341787 | BTNL3 | 3.152E-05 | -0.156 | - | - |
| 5345 | ENSP00000301490 | NUDT8 | 2.207E-05 | -0.156 | - | - |
| 5346 | ENSP00000353793 | WDHD1 | 3.398E-05 | -0.156 | - | - |
| 5347 | ENSP00000400997 | ZNF780A | 1.959E-05 | -0.156 | - | - |
| 5348 | ENSP00000339958 | NISCH | 7.482E-05 | -0.156 | - | - |
| 5349 | ENSP00000415200 | SIGLEC5 | 1.336E-05 | -0.156 | - | - |
| 5350 | ENSP00000347810 | DEFB107B | 1.226E-06 | -0.156 | - | - |
| 5351 | ENSP00000387536 | PDK3 | 3.275E-05 | -0.156 | - | - |
| 5352 | ENSP00000355649 | SPHAR | 1.133E-05 | -0.156 | - | - |
| 5353 | ENSP00000291294 | PTGIR | 8.203E-05 | -0.157 | - | - |
| 5354 | ENSP00000379823 | COL4A3 | 2.934E-05 | -0.157 | - | - |
| 5355 | ENSP00000246069 | DSTN | 2.446E-05 | -0.157 | - | - |
| 5356 | ENSP00000300108 | TAC3 | 6.617E-05 | -0.157 | - | - |
| 5357 | ENSP00000350990 | TNKS1BP1 | 4.320E-05 | -0.157 | - | - |
| 5358 | ENSP00000438400 | CLDN24 | 4.332E-06 | -0.157 | - | - |
| 5359 | ENSP00000265870 | SLC25A16 | 6.110E-05 | -0.157 | - | - |
| 5360 | ENSP00000340305 | UBE2V1 | 6.460E-05 | -0.157 | - | - |
| 5361 | ENSP00000365411 | APBB1IP | 2.091E-05 | -0.157 | - | - |
| 5362 | ENSP00000391218 | C9orf173 | 1.796E-06 | -0.157 | - | - |
| 5363 | ENSP00000295400 | TGFA | 1.022E-04 | -0.157 | - | - |
| 5364 | ENSP00000292778 | YDJC | 3.231E-04 | -0.157 | - | - |
| 5365 | ENSP00000222254 | PIK3R2 | 8.957E-05 | -0.157 | - | - |
| 5366 | ENSP00000359042 | PRPF38B | 4.844E-05 | -0.157 | - | - |
| 5367 | ENSP00000342105 | DYRK2 | 3.333E-05 | -0.157 | - | - |
| 5368 | ENSP00000373073 | UROC1 | 2.373E-05 | -0.157 | - | - |
| 5369 | ENSP00000247153 | CFP | 9.614E-05 | -0.157 | - | - |
| 5370 | ENSP00000391409 | CDHR4 | 6.569E-06 | -0.157 | - | - |
| 5371 | ENSP00000333350 | JRKL | 1.937E-05 | -0.157 | - | - |
| 5372 | ENSP00000252593 | BST2 | 8.825E-05 | -0.157 | - | - |
| 5373 | ENSP00000322594 | RAB39A | 1.855E-04 | -0.157 | - | - |
| 5374 | ENSP00000290363 | C1orf51 | 1.677E-05 | -0.157 | - | - |
| 5375 | ENSP00000240050 | MTERFD3 | 1.555E-05 | -0.157 | - | - |
| 5376 | ENSP00000281523 | ZNF385D | 4.237E-05 | -0.157 | - | - |
| 5377 | ENSP00000252971 | MNX1 | 3.232E-05 | -0.157 | - | - |
| 5378 | ENSP00000339585 | ZNF449 | 6.371E-05 | -0.157 | - | - |
| 5379 | ENSP00000262031 | RBMS2 | 3.186E-05 | -0.157 | - | - |
| 5380 | ENSP00000345206 | RBPJ | 6.620E-05 | -0.158 | - | - |
| 5381 | ENSP00000339356 | KLRG2 | 1.415E-05 | -0.158 | - | - |
| 5382 | ENSP00000216492 | CHGA | 4.201E-05 | -0.158 | - | - |
| 5383 | ENSP00000350378 | GOLGA7 | 2.897E-05 | -0.158 | - | - |
| 5384 | ENSP00000262435 | SMURF2 | 4.121E-05 | -0.158 | - | - |
| 5385 | ENSP00000358718 | CCDC147 | 2.385E-06 | -0.158 | - | - |
| 5386 | ENSP00000347338 | ZNF607 | 1.712E-05 | -0.158 | - | - |
| 5387 | ENSP00000367177 | PCDH8 | 2.325E-05 | -0.158 | - | - |
| 5388 | ENSP00000209873 | AAAS | 1.198E-04 | -0.158 | - | - |
| 5389 | ENSP00000445063 | AC105052.1 | 1.267E-06 | -0.158 | - | - |
| 5390 | ENSP00000335106 | KATNA1 | 6.884E-05 | -0.158 | - | - |
| 5391 | ENSP00000373594 | C15orf27 | 5.968E-06 | -0.158 | - | - |
| 5392 | ENSP00000454561 | RP11-122A3.2 | 3.423E-07 | -0.158 | - | - |
| 5393 | ENSP00000331103 | IP6K2 | 9.370E-05 | -0.158 | - | - |
| 5394 | ENSP00000342144 | EVC2 | 2.497E-05 | -0.158 | - | - |
| 5395 | ENSP00000442795 | MYBPC3 | 1.547E-05 | -0.158 | - | - |
| 5396 | ENSP00000361748 | ZDHHC12 | 8.776E-06 | -0.158 | - | - |
| 5397 | ENSP00000310094 | TAOK2 | 1.125E-04 | -0.158 | - | - |
| 5398 | ENSP00000369213 | DDX58 | 1.055E-04 | -0.158 | - | - |
| 5399 | ENSP00000370222 | POMP | 3.438E-05 | -0.158 | - | - |
| 5400 | ENSP00000311997 | NEFH | 3.579E-05 | -0.158 | - | - |
| 5401 | ENSP00000335040 | FAM129C | 2.911E-05 | -0.158 | - | - |
| 5402 | ENSP00000468354 | ANKRD29 | 1.032E-05 | -0.158 | - | - |
| 5403 | ENSP00000369976 | EGFL6 | 6.448E-06 | -0.158 | - | - |
| 5404 | ENSP00000299736 | CENPV | 1.025E-04 | -0.158 | - | - |
| 5405 | ENSP00000301790 | HRASLS5 | 1.210E-05 | -0.158 | - | - |
| 5406 | ENSP00000299441 | DCHS1 | 6.319E-05 | -0.158 | - | - |
| 5407 | ENSP00000293190 | GRIN2C | 2.755E-05 | -0.158 | - | - |
| 5408 | ENSP00000282091 | PTH | 5.540E-05 | -0.158 | - | - |
| 5409 | ENSP00000342931 | POU1F1 | 2.562E-05 | -0.158 | - | - |
| 5410 | ENSP00000362360 | ZHX3 | 1.844E-05 | -0.159 | - | - |
| 5411 | ENSP00000371905 | ZDHHC20 | 6.257E-06 | -0.159 | - | - |
| 5412 | ENSP00000376127 | MAU2 | 2.437E-05 | -0.159 | - | - |
| 5413 | ENSP00000365105 | TTLL9 | 2.104E-05 | -0.159 | - | - |
| 5414 | ENSP00000369127 | DNAJA1 | 6.243E-05 | -0.159 | - | - |
| 5415 | ENSP00000360149 | ALG6 | 4.083E-05 | -0.159 | - | - |
| 5416 | ENSP00000259119 | SKIL | 5.513E-05 | -0.159 | - | - |
| 5417 | ENSP00000215742 | THAP7 | 2.184E-05 | -0.159 | - | - |
| 5418 | ENSP00000221283 | STXBP2 | 3.971E-05 | -0.159 | - | - |
| 5419 | ENSP00000289902 | FCER1G | 8.556E-05 | -0.159 | - | - |
| 5420 | ENSP00000282728 | HHEX | 3.724E-05 | -0.159 | - | - |
| 5421 | ENSP00000227667 | APOC3 | 5.635E-05 | -0.159 | - | - |
| 5422 | ENSP00000453573 | RP13-996F3.5 | 7.274E-06 | -0.159 | - | - |
| 5423 | ENSP00000331504 | FES | 4.402E-05 | -0.159 | - | - |
| 5424 | ENSP00000358727 | GSTO1 | 3.098E-05 | -0.159 | - | - |
| 5425 | ENSP00000405890 | PBX1 | 5.168E-05 | -0.159 | - | - |
| 5426 | ENSP00000258080 | HTRA2 | 3.904E-05 | -0.159 | - | - |
| 5427 | ENSP00000344413 | PSG5 | 2.280E-05 | -0.159 | - | - |
| 5428 | ENSP00000314508 | GBA | 5.799E-05 | -0.159 | - | - |
| 5429 | ENSP00000345079 | CSRP1 | 4.527E-05 | -0.159 | - | - |
| 5430 | ENSP00000217131 | CTSZ | 5.973E-05 | -0.159 | - | - |
| 5431 | ENSP00000448536 | C12orf75 | 4.652E-05 | -0.159 | - | - |
| 5432 | ENSP00000351407 | ARNT | 1.032E-04 | -0.159 | - | - |
| 5433 | ENSP00000399947 | ASAH2C | 2.417E-05 | -0.159 | - | - |
| 5434 | ENSP00000257527 | ADAM19 | 9.562E-05 | -0.159 | - | - |
| 5435 | ENSP00000245543 | ARMC7 | 4.823E-05 | -0.159 | - | - |
| 5436 | ENSP00000471094 | LRRC3DN | 4.607E-07 | -0.159 | - | - |
| 5437 | ENSP00000340279 | SSX2IP | 2.163E-04 | -0.159 | - | - |
| 5438 | ENSP00000227638 | PANX1 | 1.706E-04 | -0.160 | - | - |
| 5439 | ENSP00000237612 | ABCG2 | 3.695E-05 | -0.160 | - | - |
| 5440 | ENSP00000309771 | P2RY6 | 7.750E-05 | -0.160 | - | - |
| 5441 | ENSP00000257861 | AVIL | 6.761E-05 | -0.160 | - | - |
| 5442 | ENSP00000296449 | CCDC36 | 2.960E-05 | -0.160 | - | - |
| 5443 | ENSP00000349785 | MAP1LC3C | 4.603E-05 | -0.160 | - | - |
| 5444 | ENSP00000357901 | SCNM1 | 6.242E-06 | -0.160 | - | - |
| 5445 | ENSP00000331167 | C11orf35 | 2.245E-05 | -0.160 | - | - |
| 5446 | ENSP00000254801 | IGJ | 7.349E-05 | -0.160 | - | - |
| 5447 | ENSP00000276202 | DOCK11 | 8.588E-05 | -0.160 | - | - |
| 5448 | ENSP00000393963 | KIFC1 | 3.978E-05 | -0.160 | - | - |
| 5449 | ENSP00000303129 | VAT1L | 7.591E-05 | -0.160 | - | - |
| 5450 | ENSP00000329468 | ADAP2 | 2.294E-05 | -0.160 | - | - |
| 5451 | ENSP00000304593 | ZNHIT1 | 4.607E-05 | -0.160 | - | - |
| 5452 | ENSP00000317469 | BBS1 | 6.000E-05 | -0.160 | - | - |
| 5453 | ENSP00000264553 | GZMM | 5.266E-05 | -0.160 | - | - |
| 5454 | ENSP00000412324 | PPP2R5C | 3.597E-05 | -0.160 | - | - |
| 5455 | ENSP00000345107 | C2orf88 | 1.249E-05 | -0.160 | - | - |
| 5456 | ENSP00000405635 | CNEP1R1 | 1.045E-05 | -0.160 | - | - |
| 5457 | ENSP00000057513 | TNIP3 | 1.344E-04 | -0.160 | - | - |
| 5458 | ENSP00000308227 | HMGA1 | 4.800E-05 | -0.160 | - | - |
| 5459 | ENSP00000345719 | TOR1A | 1.162E-04 | -0.160 | - | - |
| 5460 | ENSP00000449500 | RNASEK | 3.708E-05 | -0.160 | - | - |
| 5461 | ENSP00000275015 | NFKBIE | 3.324E-05 | -0.160 | - | - |
| 5462 | ENSP00000373825 | DNAH2 | 2.004E-05 | -0.160 | - | - |
| 5463 | ENSP00000265986 | IDE | 4.403E-05 | -0.160 | - | - |
| 5464 | ENSP00000431872 | TPT1 | 2.144E-05 | -0.161 | - | - |
| 5465 | ENSP00000392410 | ZNF587B | 2.089E-05 | -0.161 | - | - |
| 5466 | ENSP00000311300 | CTSW | 1.700E-04 | -0.161 | - | - |
| 5467 | ENSP00000321498 | SLC37A3 | 2.697E-05 | -0.161 | - | - |
| 5468 | ENSP00000352846 | ZNF808 | 1.671E-05 | -0.161 | - | - |
| 5469 | ENSP00000364073 | EIF4G3 | 7.995E-05 | -0.161 | - | - |
| 5470 | ENSP00000263461 | WDR11 | 2.423E-04 | -0.161 | - | - |
| 5471 | ENSP00000270879 | FCN3 | 3.181E-05 | -0.161 | - | - |
| 5472 | ENSP00000395465 | NCOA4 | 5.663E-06 | -0.161 | - | - |
| 5473 | ENSP00000356135 | TMEM81 | 1.638E-06 | -0.161 | - | - |
| 5474 | ENSP00000350198 | SSTR2 | 8.368E-05 | -0.161 | - | - |
| 5475 | ENSP00000334002 | HAP1 | 9.583E-05 | -0.161 | - | - |
| 5476 | ENSP00000229319 | LDHB | 2.684E-05 | -0.161 | - | - |
| 5477 | ENSP00000347088 | LARGE | 6.721E-05 | -0.161 | - | - |
| 5478 | ENSP00000462730 | LMNB2 | 6.148E-05 | -0.161 | - | - |
| 5479 | ENSP00000366374 | OR2J3 | 4.211E-06 | -0.161 | - | - |
| 5480 | ENSP00000253408 | GFAP | 7.420E-05 | -0.161 | - | - |
| 5481 | ENSP00000233156 | TFPI | 5.062E-05 | -0.161 | - | - |
| 5482 | ENSP00000366372 | OR2J2 | 4.211E-06 | -0.161 | - | - |
| 5483 | ENSP00000234961 | OPRD1 | 6.951E-05 | -0.161 | - | - |
| 5484 | ENSP00000242728 | BHLHE41 | 4.410E-05 | -0.161 | - | - |
| 5485 | ENSP00000378418 | SP9 | 1.659E-05 | -0.161 | - | - |
| 5486 | ENSP00000249750 | ALDH1A2 | 2.416E-05 | -0.161 | - | - |
| 5487 | ENSP00000360181 | SH2D1A | 1.518E-04 | -0.161 | - | - |
| 5488 | ENSP00000265643 | GAL | 6.802E-05 | -0.161 | - | - |
| 5489 | ENSP00000347730 | ZNF536 | 3.704E-05 | -0.161 | - | - |
| 5490 | ENSP00000421917 | HMGXB3 | 2.114E-05 | -0.161 | - | - |
| 5491 | ENSP00000357356 | AL603926.1 | 1.395E-05 | -0.161 | - | - |
| 5492 | ENSP00000257566 | TBX3 | 4.454E-05 | -0.162 | - | - |
| 5493 | ENSP00000330307 | HIST1H2AK | 7.817E-05 | -0.162 | - | - |
| 5494 | ENSP00000277632 | FAM188A | 1.468E-04 | -0.162 | - | - |
| 5495 | ENSP00000409463 | ZNF740 | 2.346E-05 | -0.162 | - | - |
| 5496 | ENSP00000243346 | NMI | 1.825E-04 | -0.162 | - | - |
| 5497 | ENSP00000354694 | ZNF485 | 1.413E-05 | -0.162 | - | - |
| 5498 | ENSP00000340698 | GIPC1 | 7.788E-05 | -0.162 | - | - |
| 5499 | ENSP00000365735 | TLE4 | 4.396E-05 | -0.162 | - | - |
| 5500 | ENSP00000350937 | TES | 6.301E-05 | -0.162 | - | - |
| 5501 | ENSP00000340684 | MAOA | 3.220E-05 | -0.162 | - | - |
| 5502 | ENSP00000354487 | ZEB1 | 7.226E-05 | -0.162 | - | - |
| 5503 | ENSP00000347210 | ZNF736 | 1.430E-05 | -0.162 | - | - |
| 5504 | ENSP00000274093 | GLRA3 | 2.445E-05 | -0.162 | - | - |
| 5505 | ENSP00000222308 | FKBP8 | 2.619E-05 | -0.162 | - | - |
| 5506 | ENSP00000381726 | PRAME | 2.721E-05 | -0.162 | - | - |
| 5507 | ENSP00000339801 | IDS | 1.271E-05 | -0.162 | - | - |
| 5508 | ENSP00000337088 | MEN1 | 5.534E-05 | -0.162 | - | - |
| 5509 | ENSP00000311344 | PPP2R1B | 5.020E-05 | -0.162 | - | - |
| 5510 | ENSP00000296238 | CAMK2N2 | 2.907E-05 | -0.162 | - | - |
| 5511 | ENSP00000382804 | ODF3B | 7.893E-06 | -0.162 | - | - |
| 5512 | ENSP00000283752 | SERPINB3 | 6.563E-05 | -0.162 | - | - |
| 5513 | ENSP00000346348 | ZNF774 | 1.378E-05 | -0.162 | - | - |
| 5514 | ENSP00000262304 | PKD1 | 3.772E-05 | -0.162 | - | - |
| 5515 | ENSP00000215780 | GSTT2 | 2.949E-05 | -0.162 | - | - |
| 5516 | ENSP00000259806 | FOXF2 | 1.765E-05 | -0.162 | - | - |
| 5517 | ENSP00000339328 | PLAUR | 9.084E-05 | -0.162 | - | - |
| 5518 | ENSP00000364700 | OMD | 3.587E-05 | -0.162 | - | - |
| 5519 | ENSP00000350199 | AP1B1 | 1.587E-05 | -0.162 | - | - |
| 5520 | ENSP00000262895 | GRIK5 | 2.479E-05 | -0.162 | - | - |
| 5521 | ENSP00000281441 | TMEM45B | 3.697E-05 | -0.162 | - | - |
| 5522 | ENSP00000276689 | NDUFB9 | 5.041E-05 | -0.163 | - | - |
| 5523 | ENSP00000411372 | FAM200A | 4.871E-08 | -0.163 | - | - |
| 5524 | ENSP00000346151 | MAGI2 | 3.329E-05 | -0.163 | - | - |
| 5525 | ENSP00000265171 | EGF | 4.487E-05 | -0.163 | - | - |
| 5526 | ENSP00000296861 | TNFRSF21 | 1.193E-04 | -0.163 | - | - |
| 5527 | ENSP00000351391 | ZNF781 | 1.416E-05 | -0.163 | - | - |
| 5528 | ENSP00000272298 | CALM2 | 2.146E-05 | -0.163 | - | - |
| 5529 | ENSP00000455385 | C3orf83 | 1.768E-07 | -0.163 | - | - |
| 5530 | ENSP00000265727 | ADAM22 | 2.958E-05 | -0.163 | - | - |
| 5531 | ENSP00000264160 | R3HDM1 | 7.831E-06 | -0.163 | - | - |
| 5532 | ENSP00000254436 | TRIM21 | 4.405E-05 | -0.163 | - | - |
| 5533 | ENSP00000245552 | NT5C | 3.071E-05 | -0.163 | - | - |
| 5534 | ENSP00000453969 | C15orf52 | 2.950E-05 | -0.163 | - | - |
| 5535 | ENSP00000310244 | RASGRP1 | 1.011E-04 | -0.163 | - | - |
| 5536 | ENSP00000337461 | STPG1 | 8.361E-05 | -0.163 | - | - |
| 5537 | ENSP00000369647 | AVP | 5.799E-05 | -0.163 | - | - |
| 5538 | ENSP00000361524 | C9orf78 | 4.089E-05 | -0.163 | - | - |
| 5539 | ENSP00000258947 | CALCOCO2 | 6.189E-05 | -0.163 | - | - |
| 5540 | ENSP00000230449 | EXOC2 | 4.594E-05 | -0.163 | - | - |
| 5541 | ENSP00000450675 | LIMS3 | 9.055E-06 | -0.163 | - | - |
| 5542 | ENSP00000470580 | AC027228.1 | 1.361E-05 | -0.163 | - | - |
| 5543 | ENSP00000223051 | TFR2 | 8.357E-05 | -0.164 | - | - |
| 5544 | ENSP00000354912 | MAGEE1 | 2.786E-05 | -0.164 | - | - |
| 5545 | ENSP00000385347 | TAPT1 | 2.753E-05 | -0.164 | - | - |
| 5546 | ENSP00000398495 | DIABLO | 1.683E-03 | -0.164 | - | - |
| 5547 | ENSP00000352676 | ZRANB1 | 2.948E-05 | -0.164 | - | - |
| 5548 | ENSP00000368502 | SCGB2B2 | 1.244E-05 | -0.164 | - | - |
| 5549 | ENSP00000276420 | DOK2 | 7.976E-05 | -0.164 | - | - |
| 5550 | ENSP00000229708 | ULBP1 | 1.811E-04 | -0.164 | - | - |
| 5551 | ENSP00000259406 | RGS3 | 7.141E-05 | -0.164 | - | - |
| 5552 | ENSP00000366280 | LMAN2L | 1.614E-05 | -0.164 | - | - |
| 5553 | ENSP00000376583 | RIC8B | 3.342E-05 | -0.164 | - | - |
| 5554 | ENSP00000261479 | PSMA6 | 3.587E-05 | -0.164 | - | - |
| 5555 | ENSP00000410926 | LMLN | 1.174E-05 | -0.164 | - | - |
| 5556 | ENSP00000304290 | CHRNB1 | 1.469E-04 | -0.164 | - | - |
| 5557 | ENSP00000002829 | SEMA3F | 7.643E-05 | -0.164 | - | - |
| 5558 | ENSP00000345492 | SH2B3 | 1.303E-04 | -0.164 | - | - |
| 5559 | ENSP00000359910 | PSMA7 | 5.249E-05 | -0.164 | - | - |
| 5560 | ENSP00000307461 | KIAA1024 | 4.077E-05 | -0.164 | - | - |
| 5561 | ENSP00000366894 | KLF12 | 2.593E-05 | -0.164 | - | - |
| 5562 | ENSP00000355459 | ZNF670 | 1.341E-05 | -0.164 | - | - |
| 5563 | ENSP00000349161 | ZNF790 | 1.403E-05 | -0.164 | - | - |
| 5564 | ENSP00000417185 | VWA1 | 3.691E-05 | -0.164 | - | - |
| 5565 | ENSP00000297324 | C8orf48 | 2.444E-05 | -0.164 | - | - |
| 5566 | ENSP00000380073 | IRF9 | 1.527E-04 | -0.164 | - | - |
| 5567 | ENSP00000345151 | RFPL4AL1 | 9.751E-06 | -0.164 | - | - |
| 5568 | ENSP00000347251 | GPS1 | 3.253E-05 | -0.164 | - | - |
| 5569 | ENSP00000314914 | AMER3 | 6.070E-05 | -0.164 | - | - |
| 5570 | ENSP00000323184 | MYCBPAP | 4.184E-05 | -0.164 | - | - |
| 5571 | ENSP00000452844 | RP13-996F3.4 | 7.235E-06 | -0.164 | - | - |
| 5572 | ENSP00000357811 | TEX36 | 1.359E-06 | -0.164 | - | - |
| 5573 | ENSP00000353132 | DMBX1 | 2.435E-05 | -0.164 | - | - |
| 5574 | ENSP00000343000 | IL19 | 2.940E-04 | -0.164 | - | - |
| 5575 | ENSP00000290219 | IFNGR2 | 2.778E-04 | -0.164 | - | - |
| 5576 | ENSP00000358565 | SLC35A1 | 4.931E-06 | -0.164 | - | - |
| 5577 | ENSP00000264933 | PDCD6 | 4.676E-05 | -0.164 | - | - |
| 5578 | ENSP00000261714 | BLMH | 7.248E-05 | -0.164 | - | - |
| 5579 | ENSP00000261558 | AP5M1 | 3.238E-05 | -0.164 | - | - |
| 5580 | ENSP00000296677 | F2RL1 | 5.733E-05 | -0.164 | - | - |
| 5581 | ENSP00000350418 | ZNF98 | 1.366E-05 | -0.164 | - | - |
| 5582 | ENSP00000457881 | CPEB1 | 6.187E-05 | -0.164 | - | - |
| 5583 | ENSP00000209875 | CBX5 | 4.949E-05 | -0.164 | - | - |
| 5584 | ENSP00000394047 | CTD-3105H18.14 | 1.005E-05 | -0.165 | - | - |
| 5585 | ENSP00000265403 | UGT2B10 | 2.573E-05 | -0.165 | - | - |
| 5586 | ENSP00000372394 | TMEM129 | 3.535E-05 | -0.165 | - | - |
| 5587 | ENSP00000355517 | KMO | 2.827E-05 | -0.165 | - | - |
| 5588 | ENSP00000318615 | TRIM73 | 8.977E-05 | -0.165 | - | - |
| 5589 | ENSP00000357132 | OR10X1 | 1.582E-05 | -0.165 | - | - |
| 5590 | ENSP00000254667 | PTPRE | 4.665E-05 | -0.165 | - | - |
| 5591 | ENSP00000363001 | SMPDL3B | 4.080E-05 | -0.165 | - | - |
| 5592 | ENSP00000296641 | F2RL2 | 6.262E-05 | -0.165 | - | - |
| 5593 | ENSP00000365443 | ZSCAN5C | 1.191E-05 | -0.165 | - | - |
| 5594 | ENSP00000379458 | ZNF586 | 1.038E-05 | -0.165 | - | - |
| 5595 | ENSP00000389841 | ZNF852 | 1.017E-05 | -0.165 | - | - |
| 5596 | ENSP00000380603 | NCKAP5 | 3.211E-05 | -0.165 | - | - |
| 5597 | ENSP00000386791 | AP000322.54 | 1.025E-06 | -0.165 | - | - |
| 5598 | ENSP00000394248 | ZNF716 | 1.005E-05 | -0.165 | - | - |
| 5599 | ENSP00000331172 | CD8B | 3.807E-04 | -0.165 | - | - |
| 5600 | ENSP00000325738 | TNP2 | 2.243E-05 | -0.165 | - | - |
| 5601 | ENSP00000363836 | CCNY | 1.450E-04 | -0.165 | - | - |
| 5602 | ENSP00000260630 | CYP1B1 | 4.402E-05 | -0.165 | - | - |
| 5603 | ENSP00000470117 | LUZPP1 | 2.141E-07 | -0.165 | - | - |
| 5604 | ENSP00000397435 | TSC22D1 | 3.721E-05 | -0.165 | - | - |
| 5605 | ENSP00000303754 | PPID | 3.816E-05 | -0.165 | - | - |
| 5606 | ENSP00000370421 | PDX1 | 3.859E-05 | -0.165 | - | - |
| 5607 | ENSP00000348812 | PTPRD | 2.884E-05 | -0.165 | - | - |
| 5608 | ENSP00000219066 | NTHL1 | 3.462E-05 | -0.165 | - | - |
| 5609 | ENSP00000361768 | RIMS3 | 9.662E-06 | -0.165 | - | - |
| 5610 | ENSP00000392024 | ZNF844 | 1.062E-05 | -0.165 | - | - |
| 5611 | ENSP00000395629 | ZNF812 | 1.005E-05 | -0.165 | - | - |
| 5612 | ENSP00000297044 | CYTH3 | 4.842E-05 | -0.165 | - | - |
| 5613 | ENSP00000353058 | ZNF517 | 1.403E-05 | -0.165 | - | - |
| 5614 | ENSP00000272167 | EPHX1 | 5.824E-05 | -0.165 | - | - |
| 5615 | ENSP00000386110 | GAGE2D | 3.425E-07 | -0.165 | - | - |
| 5616 | ENSP00000221444 | KCNA7 | 2.289E-05 | -0.165 | - | - |
| 5617 | ENSP00000279593 | GRIN2B | 3.683E-05 | -0.165 | - | - |
| 5618 | ENSP00000229758 | FBXO5 | 8.855E-05 | -0.166 | - | - |
| 5619 | ENSP00000245479 | SOX9 | 5.183E-05 | -0.166 | - | - |
| 5620 | ENSP00000246912 | MLX | 5.482E-05 | -0.166 | - | - |
| 5621 | ENSP00000264501 | KIAA1109 | 7.605E-05 | -0.166 | - | - |
| 5622 | ENSP00000380480 | ZNF891 | 1.033E-05 | -0.166 | - | - |
| 5623 | ENSP00000302232 | ALKBH3 | 1.440E-05 | -0.166 | - | - |
| 5624 | ENSP00000300527 | COL6A2 | 5.985E-05 | -0.166 | - | - |
| 5625 | ENSP00000222693 | CAV2 | 5.476E-05 | -0.166 | - | - |
| 5626 | ENSP00000404583 | C5orf60 | 3.425E-07 | -0.166 | - | - |
| 5627 | ENSP00000381089 | SLC35E3 | 7.747E-06 | -0.166 | - | - |
| 5628 | ENSP00000413196 | FAM160A1 | 2.236E-07 | -0.166 | - | - |
| 5629 | ENSP00000370408 | CDX2 | 4.974E-05 | -0.166 | - | - |
| 5630 | ENSP00000324948 | SOX6 | 5.501E-05 | -0.166 | - | - |
| 5631 | ENSP00000374013 | PRB2 | 1.443E-05 | -0.166 | - | - |
| 5632 | ENSP00000262850 | RRAGB | 7.147E-05 | -0.166 | - | - |
| 5633 | ENSP00000380413 | AGAP3 | 6.670E-06 | -0.166 | - | - |
| 5634 | ENSP00000265404 | STAP1 | 4.146E-05 | -0.166 | - | - |
| 5635 | ENSP00000350295 | ZNF816 | 1.907E-05 | -0.166 | - | - |
| 5636 | ENSP00000386485 | APOPT1 | 2.272E-05 | -0.166 | - | - |
| 5637 | ENSP00000272190 | REN | 5.625E-05 | -0.166 | - | - |
| 5638 | ENSP00000247182 | SIX1 | 3.923E-05 | -0.166 | - | - |
| 5639 | ENSP00000252575 | NCAN | 2.016E-05 | -0.166 | - | - |
| 5640 | ENSP00000361467 | DLG5 | 8.162E-05 | -0.166 | - | - |
| 5641 | ENSP00000373254 | KRBOX1 | 1.051E-05 | -0.166 | - | - |
| 5642 | ENSP00000377833 | ANXA4 | 5.450E-05 | -0.166 | - | - |
| 5643 | ENSP00000445831 | HIST2H2BF | 2.872E-05 | -0.166 | - | - |
| 5644 | ENSP00000326267 | EFCAB11 | 2.643E-05 | -0.166 | - | - |
| 5645 | ENSP00000347329 | SEC31A | 4.523E-05 | -0.166 | - | - |
| 5646 | ENSP00000352233 | ZNF705A | 2.582E-05 | -0.166 | - | - |
| 5647 | ENSP00000334798 | KRT26 | 2.637E-05 | -0.166 | - | - |
| 5648 | ENSP00000319255 | CHORDC1 | 1.275E-04 | -0.166 | - | - |
| 5649 | ENSP00000283928 | JAZF1 | 4.612E-05 | -0.166 | - | - |
| 5650 | ENSP00000462172 | KIAA0040 | 2.939E-07 | -0.166 | - | - |
| 5651 | ENSP00000375237 | KRTAP2-3 | 6.372E-06 | -0.166 | - | - |
| 5652 | ENSP00000307134 | CTNND2 | 4.183E-05 | -0.166 | - | - |
| 5653 | ENSP00000354610 | AMY2B | 4.834E-05 | -0.166 | - | - |
| 5654 | ENSP00000369634 | PRPF40B | 1.988E-05 | -0.166 | - | - |
| 5655 | ENSP00000328335 | SMIM10 | 1.284E-01 | -0.166 | - | - |
| 5656 | ENSP00000363207 | AGAP5 | 7.752E-06 | -0.166 | - | - |
| 5657 | ENSP00000366898 | HIF3A | 3.797E-05 | -0.167 | - | - |
| 5658 | ENSP00000419362 | SHOX2 | 3.223E-05 | -0.167 | - | - |
| 5659 | ENSP00000372484 | RBMY1B | 6.603E-08 | -0.167 | - | - |
| 5660 | ENSP00000395733 | ZNF737 | 9.942E-06 | -0.167 | - | - |
| 5661 | ENSP00000261435 | N4BP2 | 7.596E-05 | -0.167 | - | - |
| 5662 | ENSP00000237858 | GLRX | 4.356E-05 | -0.167 | - | - |
| 5663 | ENSP00000379464 | ZNF134 | 1.029E-05 | -0.167 | - | - |
| 5664 | ENSP00000259457 | PSMB7 | 3.862E-05 | -0.167 | - | - |
| 5665 | ENSP00000372124 | RBMY1D | 6.603E-08 | -0.167 | - | - |
| 5666 | ENSP00000319984 | CHRM2 | 6.204E-05 | -0.167 | - | - |
| 5667 | ENSP00000371829 | BPY2B | 9.142E-03 | -0.167 | - | - |
| 5668 | ENSP00000228799 | ITFG2 | 8.250E-05 | -0.167 | - | - |
| 5669 | ENSP00000243389 | SLC36A1 | 5.196E-05 | -0.167 | - | - |
| 5670 | ENSP00000361818 | SDC4 | 8.272E-05 | -0.167 | - | - |
| 5671 | ENSP00000356674 | ZBTB37 | 1.361E-05 | -0.167 | - | - |
| 5672 | ENSP00000233607 | APC2 | 5.680E-05 | -0.167 | - | - |
| 5673 | ENSP00000430633 | 61E3.4 | 9.032E-06 | -0.167 | - | - |
| 5674 | ENSP00000447300 | RAB3IP | 4.192E-05 | -0.167 | - | - |
| 5675 | ENSP00000429601 | PCDHGA6 | 1.888E-05 | -0.167 | - | - |
| 5676 | ENSP00000356110 | NUCKS1 | 5.780E-05 | -0.167 | - | - |
| 5677 | ENSP00000366283 | SLITRK5 | 1.454E-05 | -0.167 | - | - |
| 5678 | ENSP00000368411 | SCNN1D | 2.022E-05 | -0.167 | - | - |
| 5679 | ENSP00000241651 | MYOG | 2.842E-05 | -0.167 | - | - |
| 5680 | ENSP00000298396 | SSX3 | 1.831E-04 | -0.167 | - | - |
| 5681 | ENSP00000345339 | ZSCAN31 | 1.495E-05 | -0.167 | - | - |
| 5682 | ENSP00000422846 | RNASET2 | 3.479E-05 | -0.167 | - | - |
| 5683 | ENSP00000362803 | PPIL1 | 2.683E-05 | -0.167 | - | - |
| 5684 | ENSP00000359802 | ZNF75D | 1.261E-05 | -0.167 | - | - |
| 5685 | ENSP00000356584 | TOR1AIP2 | 3.694E-05 | -0.167 | - | - |
| 5686 | ENSP00000318176 | PRKRA | 6.507E-05 | -0.167 | - | - |
| 5687 | ENSP00000334276 | UGT2B28 | 2.451E-05 | -0.167 | - | - |
| 5688 | ENSP00000349459 | SPINK14 | 1.785E-05 | -0.167 | - | - |
| 5689 | ENSP00000282470 | SPARCL1 | 6.289E-05 | -0.167 | - | - |
| 5690 | ENSP00000439594 | THEMIS | 4.001E-05 | -0.167 | - | - |
| 5691 | ENSP00000316740 | TTC29 | 2.352E-05 | -0.167 | - | - |
| 5692 | ENSP00000343745 | DICER1 | 5.224E-05 | -0.168 | - | - |
| 5693 | ENSP00000265689 | CHKA | 4.168E-05 | -0.168 | - | - |
| 5694 | ENSP00000376544 | CD79B | 1.581E-04 | -0.168 | - | - |
| 5695 | ENSP00000166139 | FSTL3 | 7.020E-05 | -0.168 | - | - |
| 5696 | ENSP00000230124 | FIG4 | 3.520E-05 | -0.168 | - | - |
| 5697 | ENSP00000291442 | NR2F6 | 5.522E-05 | -0.168 | - | - |
| 5698 | ENSP00000392985 | HTATIP2 | 2.705E-05 | -0.168 | - | - |
| 5699 | ENSP00000352836 | ZNF675 | 1.414E-05 | -0.168 | - | - |
| 5700 | ENSP00000356975 | ADAMTS4 | 1.057E-04 | -0.168 | - | - |
| 5701 | ENSP00000264717 | GCKR | 7.692E-05 | -0.168 | - | - |
| 5702 | ENSP00000262953 | TLE2 | 4.422E-05 | -0.168 | - | - |
| 5703 | ENSP00000248564 | GNG11 | 2.645E-05 | -0.168 | - | - |
| 5704 | ENSP00000370109 | PSIP1 | 5.825E-05 | -0.168 | - | - |
| 5705 | ENSP00000433998 | CKLF-CMTM1 | 1.764E-05 | -0.168 | - | - |
| 5706 | ENSP00000392513 | AGAP4 | 6.212E-06 | -0.168 | - | - |
| 5707 | ENSP00000332806 | EMILIN3 | 2.529E-05 | -0.168 | - | - |
| 5708 | ENSP00000322885 | DTX2 | 9.850E-05 | -0.168 | - | - |
| 5709 | ENSP00000264552 | UBE2S | 2.514E-05 | -0.168 | - | - |
| 5710 | ENSP00000301286 | PLIN4 | 1.575E-04 | -0.168 | - | - |
| 5711 | ENSP00000254654 | ILKAP | 2.951E-05 | -0.168 | - | - |
| 5712 | ENSP00000353646 | PLEKHG4 | 1.342E-05 | -0.168 | - | - |
| 5713 | ENSP00000216465 | GSTZ1 | 2.769E-05 | -0.168 | - | - |
| 5714 | ENSP00000402060 | PVR | 4.558E-05 | -0.168 | - | - |
| 5715 | ENSP00000384000 | ZNF681 | 1.033E-05 | -0.168 | - | - |
| 5716 | ENSP00000261416 | HEXB | 4.278E-05 | -0.168 | - | - |
| 5717 | ENSP00000222266 | PSENEN | 4.736E-05 | -0.168 | - | - |
| 5718 | ENSP00000260264 | POU2F3 | 5.332E-05 | -0.168 | - | - |
| 5719 | ENSP00000251968 | TSG101 | 3.693E-05 | -0.168 | - | - |
| 5720 | ENSP00000450635 | TMEM189 | 5.502E-05 | -0.168 | - | - |
| 5721 | ENSP00000301904 | SCARA3 | 4.652E-05 | -0.168 | - | - |
| 5722 | ENSP00000469038 | FLJ14816 | 8.900E-07 | -0.168 | - | - |
| 5723 | ENSP00000402151 | CTC-203F4.1 | 7.341E-06 | -0.168 | - | - |
| 5724 | ENSP00000413728 | C12orf71 | 1.828E-05 | -0.168 | - | - |
| 5725 | ENSP00000274056 | 1-Mar | 6.186E-05 | -0.168 | - | - |
| 5726 | ENSP00000311502 | HEG1 | 8.620E-05 | -0.168 | - | - |
| 5727 | ENSP00000351939 | ZNF574 | 1.363E-05 | -0.168 | - | - |
| 5728 | ENSP00000295522 | CLDN1 | 4.824E-05 | -0.168 | - | - |
| 5729 | ENSP00000362105 | UTP11L | 3.049E-05 | -0.168 | - | - |
| 5730 | ENSP00000362314 | EIF4EBP2 | 7.990E-05 | -0.169 | - | - |
| 5731 | ENSP00000398076 | ATP9B | 2.556E-05 | -0.169 | - | - |
| 5732 | ENSP00000356243 | UBE2T | 3.623E-05 | -0.169 | - | - |
| 5733 | ENSP00000273588 | AMT | 1.323E-05 | -0.169 | - | - |
| 5734 | ENSP00000366098 | YIF1A | 7.321E-06 | -0.169 | - | - |
| 5735 | ENSP00000316426 | MLLT6 | 6.754E-05 | -0.169 | - | - |
| 5736 | ENSP00000243108 | HOXC6 | 5.944E-05 | -0.169 | - | - |
| 5737 | ENSP00000261722 | AP3B2 | 3.763E-05 | -0.169 | - | - |
| 5738 | ENSP00000338020 | EIF4G1 | 8.226E-05 | -0.169 | - | - |
| 5739 | ENSP00000302895 | ARAP2 | 1.361E-05 | -0.169 | - | - |
| 5740 | ENSP00000463832 | RP11-41O4.1 | 9.358E-07 | -0.169 | - | - |
| 5741 | ENSP00000261716 | TAOK1 | 5.445E-05 | -0.169 | - | - |
| 5742 | ENSP00000248948 | VPREB3 | 4.568E-05 | -0.169 | - | - |
| 5743 | ENSP00000325312 | METAP2 | 3.658E-05 | -0.169 | - | - |
| 5744 | ENSP00000276914 | PLIN2 | 4.140E-05 | -0.169 | - | - |
| 5745 | ENSP00000363257 | ZDHHC18 | 1.017E-05 | -0.169 | - | - |
| 5746 | ENSP00000416240 | SEBOX | 2.088E-05 | -0.169 | - | - |
| 5747 | ENSP00000258774 | HUS1 | 6.025E-05 | -0.169 | - | - |
| 5748 | ENSP00000367300 | ZNF425 | 1.148E-05 | -0.169 | - | - |
| 5749 | ENSP00000267460 | PELI2 | 1.543E-04 | -0.169 | - | - |
| 5750 | ENSP00000334052 | LGMN | 5.845E-05 | -0.169 | - | - |
| 5751 | ENSP00000349929 | SDCCAG3 | 1.221E-04 | -0.169 | - | - |
| 5752 | ENSP00000265175 | SEC24B | 4.189E-05 | -0.169 | - | - |
| 5753 | ENSP00000233143 | TMSB10 | 1.278E-04 | -0.169 | - | - |
| 5754 | ENSP00000361803 | SMAP2 | 3.699E-05 | -0.169 | - | - |
| 5755 | ENSP00000243611 | C4BPB | 1.041E-04 | -0.169 | - | - |
| 5756 | ENSP00000317327 | UBASH3A | 1.190E-04 | -0.169 | - | - |
| 5757 | ENSP00000204566 | SPG21 | 4.962E-05 | -0.169 | - | - |
| 5758 | ENSP00000297533 | TMUB1 | 3.118E-04 | -0.169 | - | - |
| 5759 | ENSP00000241527 | MTERFD2 | 1.937E-04 | -0.169 | - | - |
| 5760 | ENSP00000385610 | MEX3C | 4.822E-05 | -0.169 | - | - |
| 5761 | ENSP00000371220 | LOH12CR2 | 1.824E-05 | -0.169 | - | - |
| 5762 | ENSP00000338235 | MTDH | 6.384E-05 | -0.169 | - | - |
| 5763 | ENSP00000332194 | HIST2H2AC | 4.618E-05 | -0.169 | - | - |
| 5764 | ENSP00000349882 | ZNF248 | 1.079E-05 | -0.169 | - | - |
| 5765 | ENSP00000307634 | AGAP1 | 8.791E-06 | -0.169 | - | - |
| 5766 | ENSP00000371729 | SACS | 1.116E-05 | -0.169 | - | - |
| 5767 | ENSP00000363019 | UBE2D1 | 5.072E-05 | -0.169 | - | - |
| 5768 | ENSP00000265729 | SRI | 3.288E-05 | -0.169 | - | - |
| 5769 | ENSP00000304736 | ELOVL6 | 3.619E-05 | -0.169 | - | - |
| 5770 | ENSP00000376110 | ZNF493 | 1.034E-05 | -0.169 | - | - |
| 5771 | ENSP00000364677 | ZBTB12 | 1.263E-05 | -0.169 | - | - |
| 5772 | ENSP00000341390 | NR3C2 | 3.362E-05 | -0.169 | - | - |
| 5773 | ENSP00000222399 | LAMB1 | 5.081E-05 | -0.169 | - | - |
| 5774 | ENSP00000262961 | ZFR2 | 3.054E-05 | -0.169 | - | - |
| 5775 | ENSP00000331746 | CALCA | 2.427E-05 | -0.169 | - | - |
| 5776 | ENSP00000269466 | ELAC1 | 8.268E-06 | -0.169 | - | - |
| 5777 | ENSP00000377547 | CCNJL | 1.613E-05 | -0.169 | - | - |
| 5778 | ENSP00000219905 | MGA | 7.638E-05 | -0.169 | - | - |
| 5779 | ENSP00000262065 | MMD | 4.620E-05 | -0.169 | - | - |
| 5780 | ENSP00000270586 | PSMB6 | 4.396E-05 | -0.169 | - | - |
| 5781 | ENSP00000344314 | OFD1 | 5.104E-05 | -0.169 | - | - |
| 5782 | ENSP00000279968 | TMEM218 | 1.930E-05 | -0.170 | - | - |
| 5783 | ENSP00000356257 | LMOD1 | 7.841E-05 | -0.170 | - | - |
| 5784 | ENSP00000354791 | DCTN1 | 2.792E-05 | -0.170 | - | - |
| 5785 | ENSP00000364561 | FAM120AOS | 1.374E-01 | -0.170 | - | - |
| 5786 | ENSP00000055682 | KIAA2022 | 5.129E-05 | -0.170 | - | - |
| 5787 | ENSP00000297063 | KIAA0895 | 1.374E-01 | -0.170 | - | - |
| 5788 | ENSP00000309096 | B3GNT1 | 9.325E-05 | -0.170 | - | - |
| 5789 | ENSP00000342513 | IFI6 | 1.579E-04 | -0.170 | - | - |
| 5790 | ENSP00000293230 | CYGB | 4.078E-05 | -0.170 | - | - |
| 5791 | ENSP00000323516 | UTS2R | 9.692E-05 | -0.170 | - | - |
| 5792 | ENSP00000312367 | CD84 | 4.550E-04 | -0.170 | - | - |
| 5793 | ENSP00000250018 | TPH1 | 3.090E-05 | -0.170 | - | - |
| 5794 | ENSP00000310305 | P2RY2 | 5.299E-05 | -0.170 | - | - |
| 5795 | ENSP00000228837 | FGF6 | 6.737E-05 | -0.170 | - | - |
| 5796 | ENSP00000287239 | KAT6B | 2.666E-05 | -0.170 | - | - |
| 5797 | ENSP00000354677 | GPX7 | 2.962E-05 | -0.170 | - | - |
| 5798 | ENSP00000270349 | SLC6A3 | 2.341E-05 | -0.170 | - | - |
| 5799 | ENSP00000246554 | COX6B1 | 4.807E-05 | -0.170 | - | - |
| 5800 | ENSP00000316842 | SIX5 | 2.232E-05 | -0.170 | - | - |
| 5801 | ENSP00000326247 | SAMD9L | 4.529E-05 | -0.170 | - | - |
| 5802 | ENSP00000385057 | APOBEC3G | 7.491E-05 | -0.170 | - | - |
| 5803 | ENSP00000347032 | PIP4K2C | 5.483E-05 | -0.170 | - | - |
| 5804 | ENSP00000227868 | PDHX | 2.381E-05 | -0.170 | - | - |
| 5805 | ENSP00000353732 | CLIP3 | 8.178E-06 | -0.170 | - | - |
| 5806 | ENSP00000219406 | PDIA2 | 2.106E-05 | -0.170 | - | - |
| 5807 | ENSP00000302216 | ATOH1 | 4.488E-05 | -0.170 | - | - |
| 5808 | ENSP00000401371 | TIA1 | 5.150E-05 | -0.170 | - | - |
| 5809 | ENSP00000364265 | FOXE1 | 3.032E-05 | -0.170 | - | - |
| 5810 | ENSP00000297373 | PHKG1 | 2.584E-05 | -0.170 | - | - |
| 5811 | ENSP00000339634 | KIR2DL4 | 3.155E-04 | -0.170 | - | - |
| 5812 | ENSP00000377520 | SYT12 | 8.583E-06 | -0.170 | - | - |
| 5813 | ENSP00000321732 | CTDNEP1 | 5.522E-05 | -0.170 | - | - |
| 5814 | ENSP00000229195 | CNOT2 | 3.307E-05 | -0.170 | - | - |
| 5815 | ENSP00000005558 | IFRD1 | 6.603E-05 | -0.170 | - | - |
| 5816 | ENSP00000296181 | ITGB5 | 4.576E-05 | -0.170 | - | - |
| 5817 | ENSP00000435926 | CELF1 | 5.557E-05 | -0.170 | - | - |
| 5818 | ENSP00000280346 | DLAT | 3.072E-05 | -0.170 | - | - |
| 5819 | ENSP00000326309 | DEFB124 | 1.113E-05 | -0.170 | - | - |
| 5820 | ENSP00000321203 | TNIP2 | 1.480E-04 | -0.171 | - | - |
| 5821 | ENSP00000217182 | EEF1A2 | 1.962E-05 | -0.171 | - | - |
| 5822 | ENSP00000359484 | SMAP1 | 6.431E-06 | -0.171 | - | - |
| 5823 | ENSP00000355317 | WDTC1 | 5.462E-05 | -0.171 | - | - |
| 5824 | ENSP00000346402 | RNH1 | 4.028E-05 | -0.171 | - | - |
| 5825 | ENSP00000361700 | GLRA4 | 2.139E-05 | -0.171 | - | - |
| 5826 | ENSP00000356552 | MR1 | 3.798E-04 | -0.171 | - | - |
| 5827 | ENSP00000347043 | ZNF649 | 1.273E-05 | -0.171 | - | - |
| 5828 | ENSP00000394863 | PPT1 | 3.792E-05 | -0.171 | - | - |
| 5829 | ENSP00000309474 | PSMD1 | 5.206E-05 | -0.171 | - | - |
| 5830 | ENSP00000326063 | TMEM39A | 1.036E-04 | -0.171 | - | - |
| 5831 | ENSP00000366157 | GPR180 | 4.734E-05 | -0.171 | - | - |
| 5832 | ENSP00000256897 | CCNH | 2.002E-05 | -0.171 | - | - |
| 5833 | ENSP00000346440 | TCF4 | 4.547E-05 | -0.171 | - | - |
| 5834 | ENSP00000216286 | NID2 | 2.826E-05 | -0.171 | - | - |
| 5835 | ENSP00000386921 | TBC1D14 | 4.625E-05 | -0.171 | - | - |
| 5836 | ENSP00000292035 | MED27 | 6.481E-05 | -0.171 | - | - |
| 5837 | ENSP00000365107 | MASTL | 3.960E-05 | -0.171 | - | - |
| 5838 | ENSP00000248114 | GFER | 2.798E-05 | -0.171 | - | - |
| 5839 | ENSP00000255688 | RARRES3 | 1.795E-04 | -0.171 | - | - |
| 5840 | ENSP00000338207 | LMO1 | 4.286E-05 | -0.171 | - | - |
| 5841 | ENSP00000338272 | HEY1 | 5.145E-05 | -0.171 | - | - |
| 5842 | ENSP00000369075 | CENPB | 3.836E-05 | -0.171 | - | - |
| 5843 | ENSP00000264883 | NUP54 | 6.654E-05 | -0.171 | - | - |
| 5844 | ENSP00000005587 | SKAP2 | 6.522E-05 | -0.171 | - | - |
| 5845 | ENSP00000433773 | ZNF585B | 3.659E-05 | -0.171 | - | - |
| 5846 | ENSP00000348944 | RSPO1 | 5.281E-05 | -0.171 | - | - |
| 5847 | ENSP00000227880 | SLC15A3 | 6.516E-05 | -0.171 | - | - |
| 5848 | ENSP00000311028 | RPS14 | 3.755E-05 | -0.171 | - | - |
| 5849 | ENSP00000269122 | CLTC | 2.508E-05 | -0.171 | - | - |
| 5850 | ENSP00000242607 | HVCN1 | 8.859E-05 | -0.172 | - | - |
| 5851 | ENSP00000455826 | GOLGA8T | 7.070E-06 | -0.172 | - | - |
| 5852 | ENSP00000273258 | ARL6IP5 | 6.831E-05 | -0.172 | - | - |
| 5853 | ENSP00000348632 | RBCK1 | 4.893E-05 | -0.172 | - | - |
| 5854 | ENSP00000355241 | C19orf45 | 4.169E-05 | -0.172 | - | - |
| 5855 | ENSP00000311572 | PTGR1 | 6.574E-05 | -0.172 | - | - |
| 5856 | ENSP00000360798 | EPS15 | 6.898E-05 | -0.172 | - | - |
| 5857 | ENSP00000253669 | HAUS8 | 3.332E-04 | -0.172 | - | - |
| 5858 | ENSP00000356348 | PCMT1 | 2.364E-05 | -0.172 | - | - |
| 5859 | ENSP00000292476 | CPSF4 | 3.376E-05 | -0.172 | - | - |
| 5860 | ENSP00000264709 | DNMT3A | 3.400E-05 | -0.172 | - | - |
| 5861 | ENSP00000347198 | SRGAP1 | 9.663E-05 | -0.172 | - | - |
| 5862 | ENSP00000310561 | ARHGAP32 | 8.400E-05 | -0.172 | - | - |
| 5863 | ENSP00000380444 | MYO9B | 4.677E-05 | -0.172 | - | - |
| 5864 | ENSP00000314080 | HIC1 | 1.018E-04 | -0.172 | - | - |
| 5865 | ENSP00000273853 | CENPC1 | 5.336E-05 | -0.172 | - | - |
| 5866 | ENSP00000266744 | ASCL1 | 3.814E-05 | -0.172 | - | - |
| 5867 | ENSP00000349689 | ZDHHC9 | 8.887E-06 | -0.172 | - | - |
| 5868 | ENSP00000246841 | FLRT1 | 1.010E-05 | -0.172 | - | - |
| 5869 | ENSP00000367608 | CA9 | 6.781E-05 | -0.172 | - | - |
| 5870 | ENSP00000290691 | RGL4 | 5.141E-05 | -0.172 | - | - |
| 5871 | ENSP00000264377 | ADAM23 | 7.187E-05 | -0.172 | - | - |
| 5872 | ENSP00000373298 | COLQ | 4.059E-05 | -0.172 | - | - |
| 5873 | ENSP00000336528 | NR1I2 | 4.673E-05 | -0.172 | - | - |
| 5874 | ENSP00000294409 | GMEB1 | 1.517E-04 | -0.172 | - | - |
| 5875 | ENSP00000359567 | SOX3 | 3.530E-05 | -0.172 | - | - |
| 5876 | ENSP00000370303 | ZDHHC21 | 8.621E-06 | -0.172 | - | - |
| 5877 | ENSP00000344503 | TMEM201 | 1.481E-04 | -0.172 | - | - |
| 5878 | ENSP00000338769 | ASAP3 | 1.342E-05 | -0.172 | - | - |
| 5879 | ENSP00000252999 | LAMA5 | 5.477E-05 | -0.172 | - | - |
| 5880 | ENSP00000315602 | CHRNA3 | 2.685E-05 | -0.172 | - | - |
| 5881 | ENSP00000239151 | HOXB5 | 4.822E-05 | -0.172 | - | - |
| 5882 | ENSP00000260643 | PREB | 4.287E-05 | -0.172 | - | - |
| 5883 | ENSP00000365962 | TBC1D25 | 8.748E-06 | -0.172 | - | - |
| 5884 | ENSP00000348577 | RANGAP1 | 3.803E-05 | -0.172 | - | - |
| 5885 | ENSP00000316948 | CLK4 | 1.197E-05 | -0.172 | - | - |
| 5886 | ENSP00000336817 | LUZP2 | 3.315E-05 | -0.172 | - | - |
| 5887 | ENSP00000222572 | PON2 | 9.554E-05 | -0.172 | - | - |
| 5888 | ENSP00000318775 | ANAPC4 | 5.135E-05 | -0.172 | - | - |
| 5889 | ENSP00000235372 | PRDM2 | 5.308E-05 | -0.172 | - | - |
| 5890 | ENSP00000292401 | AZGP1 | 6.311E-05 | -0.172 | - | - |
| 5891 | ENSP00000235310 | MAD2L2 | 6.254E-05 | -0.173 | - | - |
| 5892 | ENSP00000359340 | NKAIN4 | 5.182E-05 | -0.173 | - | - |
| 5893 | ENSP00000292896 | HBE1 | 3.348E-05 | -0.173 | - | - |
| 5894 | ENSP00000337463 | AGPAT1 | 4.020E-05 | -0.173 | - | - |
| 5895 | ENSP00000254661 | RAMP1 | 3.734E-05 | -0.173 | - | - |
| 5896 | ENSP00000303211 | ACHE | 3.339E-05 | -0.173 | - | - |
| 5897 | ENSP00000359892 | SLC44A5 | 2.732E-05 | -0.173 | - | - |
| 5898 | ENSP00000350681 | ELK4 | 1.044E-04 | -0.173 | - | - |
| 5899 | ENSP00000285947 | SETD9 | 9.549E-05 | -0.173 | - | - |
| 5900 | ENSP00000261180 | TRHDE | 3.347E-05 | -0.173 | - | - |
| 5901 | ENSP00000262608 | CECR2 | 2.795E-05 | -0.173 | - | - |
| 5902 | ENSP00000355396 | LRIG2 | 7.973E-06 | -0.173 | - | - |
| 5903 | ENSP00000251472 | MAST1 | 6.364E-05 | -0.173 | - | - |
| 5904 | ENSP00000380840 | ZNF709 | 1.049E-05 | -0.173 | - | - |
| 5905 | ENSP00000280551 | SEC24D | 3.909E-05 | -0.173 | - | - |
| 5906 | ENSP00000312134 | PRG2 | 1.778E-04 | -0.173 | - | - |
| 5907 | ENSP00000230361 | GUCA1B | 1.620E-05 | -0.173 | - | - |
| 5908 | ENSP00000406478 | LILRB5 | 3.136E-06 | -0.173 | - | - |
| 5909 | ENSP00000312778 | IFT80 | 7.769E-06 | -0.173 | - | - |
| 5910 | ENSP00000277570 | PROSER2 | 5.957E-05 | -0.173 | - | - |
| 5911 | ENSP00000358867 | GNAI3 | 6.048E-05 | -0.173 | - | - |
| 5912 | ENSP00000376250 | CCDC50 | 1.995E-05 | -0.173 | - | - |
| 5913 | ENSP00000222382 | CYP3A43 | 1.897E-05 | -0.173 | - | - |
| 5914 | ENSP00000371559 | TSSC1 | 4.268E-05 | -0.173 | - | - |
| 5915 | ENSP00000326371 | FOXC2 | 2.596E-05 | -0.173 | - | - |
| 5916 | ENSP00000359239 | HAUS7 | 3.367E-05 | -0.173 | - | - |
| 5917 | ENSP00000356999 | USF1 | 5.564E-05 | -0.173 | - | - |
| 5918 | ENSP00000351490 | MAX | 5.502E-05 | -0.173 | - | - |
| 5919 | ENSP00000275428 | GGCT | 6.128E-05 | -0.173 | - | - |
| 5920 | ENSP00000233838 | GGCX | 1.176E-04 | -0.173 | - | - |
| 5921 | ENSP00000343129 | TRIM29 | 4.041E-05 | -0.173 | - | - |
| 5922 | ENSP00000379168 | ZNF287 | 1.578E-05 | -0.173 | - | - |
| 5923 | ENSP00000298596 | STOX1 | 1.535E-04 | -0.173 | - | - |
| 5924 | ENSP00000378077 | PCDHGA2 | 1.759E-05 | -0.173 | - | - |
| 5925 | ENSP00000379157 | CAST | 9.084E-05 | -0.173 | - | - |
| 5926 | ENSP00000264144 | LAMC2 | 6.139E-05 | -0.173 | - | - |
| 5927 | ENSP00000368253 | NR0B1 | 3.512E-05 | -0.173 | - | - |
| 5928 | ENSP00000345629 | MAP3K15 | 2.429E-05 | -0.173 | - | - |
| 5929 | ENSP00000358223 | PNLIP | 2.710E-05 | -0.173 | - | - |
| 5930 | ENSP00000346414 | LRRIQ3 | 1.615E-05 | -0.173 | - | - |
| 5931 | ENSP00000252483 | PVRL2 | 1.032E-04 | -0.173 | - | - |
| 5932 | ENSP00000369798 | UCN3 | 1.057E-05 | -0.174 | - | - |
| 5933 | ENSP00000264012 | CDH3 | 5.557E-05 | -0.174 | - | - |
| 5934 | ENSP00000254691 | CARD6 | 4.796E-04 | -0.174 | - | - |
| 5935 | ENSP00000056217 | ARHGEF5 | 3.788E-05 | -0.174 | - | - |
| 5936 | ENSP00000295324 | CDC42EP3 | 1.054E-04 | -0.174 | - | - |
| 5937 | ENSP00000354158 | ERC1 | 5.412E-05 | -0.174 | - | - |
| 5938 | ENSP00000383382 | GP1BB | 1.858E-05 | -0.174 | - | - |
| 5939 | ENSP00000262219 | ANXA13 | 5.859E-05 | -0.174 | - | - |
| 5940 | ENSP00000282030 | SETBP1 | 1.470E-05 | -0.174 | - | - |
| 5941 | ENSP00000379451 | ZNF418 | 1.541E-05 | -0.174 | - | - |
| 5942 | ENSP00000355361 | CD47 | 1.005E-04 | -0.174 | - | - |
| 5943 | ENSP00000230671 | SLC6A7 | 6.640E-05 | -0.174 | - | - |
| 5944 | ENSP00000321999 | PTH1R | 4.039E-05 | -0.174 | - | - |
| 5945 | ENSP00000358770 | SLK | 1.030E-04 | -0.174 | - | - |
| 5946 | ENSP00000385269 | ELAVL1 | 2.821E-01 | -0.174 | - | - |
| 5947 | ENSP00000384770 | PSG4 | 1.705E-05 | -0.174 | - | - |
| 5948 | ENSP00000354238 | ENPP1 | 2.964E-05 | -0.174 | - | - |
| 5949 | ENSP00000270357 | RNPEPL1 | 3.948E-05 | -0.174 | - | - |
| 5950 | ENSP00000346659 | OCA2 | 2.032E-05 | -0.174 | - | - |
| 5951 | ENSP00000357986 | PLEKHA1 | 2.043E-04 | -0.174 | - | - |
| 5952 | ENSP00000373827 | CCDC150 | 1.355E-05 | -0.174 | - | - |
| 5953 | ENSP00000351883 | ZSCAN5B | 1.388E-05 | -0.174 | - | - |
| 5954 | ENSP00000257600 | DTX1 | 6.459E-05 | -0.174 | - | - |
| 5955 | ENSP00000317271 | MCFD2 | 1.020E-04 | -0.174 | - | - |
| 5956 | ENSP00000358696 | PLXNA3 | 2.907E-05 | -0.174 | - | - |
| 5957 | ENSP00000252244 | KRT1 | 4.063E-05 | -0.174 | - | - |
| 5958 | ENSP00000371144 | GAGE2B | 4.877E-06 | -0.174 | - | - |
| 5959 | ENSP00000358323 | TXNIP | 7.093E-05 | -0.174 | - | - |
| 5960 | ENSP00000262940 | RASA4 | 5.739E-05 | -0.174 | - | - |
| 5961 | ENSP00000371117 | GAGE12E | 4.877E-06 | -0.174 | - | - |
| 5962 | ENSP00000359018 | AKNAD1 | 5.751E-06 | -0.174 | - | - |
| 5963 | ENSP00000273347 | NXPE3 | 2.833E-05 | -0.174 | - | - |
| 5964 | ENSP00000347896 | MBD6 | 4.543E-05 | -0.174 | - | - |
| 5965 | ENSP00000352172 | VSIG10 | 6.196E-02 | -0.174 | - | - |
| 5966 | ENSP00000362116 | PGC | 3.068E-05 | -0.174 | - | - |
| 5967 | ENSP00000338050 | DTX3 | 3.071E-05 | -0.174 | - | - |
| 5968 | ENSP00000356623 | CITED2 | 1.227E-04 | -0.174 | - | - |
| 5969 | ENSP00000281453 | MLF1IP | 6.518E-05 | -0.174 | - | - |
| 5970 | ENSP00000382697 | ROCK1 | 5.513E-05 | -0.174 | - | - |
| 5971 | ENSP00000303634 | LRP8 | 5.850E-05 | -0.174 | - | - |
| 5972 | ENSP00000246041 | AP5S1 | 7.742E-05 | -0.174 | - | - |
| 5973 | ENSP00000244745 | SOX4 | 6.357E-05 | -0.174 | - | - |
| 5974 | ENSP00000371927 | GPR78 | 1.605E-05 | -0.174 | - | - |
| 5975 | ENSP00000365682 | TLE1 | 4.492E-05 | -0.174 | - | - |
| 5976 | ENSP00000297146 | GPR85 | 2.897E-05 | -0.174 | - | - |
| 5977 | ENSP00000205061 | GLG1 | 6.117E-05 | -0.174 | - | - |
| 5978 | ENSP00000328232 | TCEB3C | 3.907E-05 | -0.175 | - | - |
| 5979 | ENSP00000273482 | TRPC1 | 4.399E-05 | -0.175 | - | - |
| 5980 | ENSP00000359793 | PTGFR | 2.920E-05 | -0.175 | - | - |
| 5981 | ENSP00000359674 | C10orf62 | 9.478E-06 | -0.175 | - | - |
| 5982 | ENSP00000374455 | SQSTM1 | 5.982E-05 | -0.175 | - | - |
| 5983 | ENSP00000418210 | ZMYND8 | 4.860E-05 | -0.175 | - | - |
| 5984 | ENSP00000330606 | HS3ST4 | 2.661E-05 | -0.175 | - | - |
| 5985 | ENSP00000349541 | PTRF | 1.034E-04 | -0.175 | - | - |
| 5986 | ENSP00000265715 | SLC26A4 | 3.584E-05 | -0.175 | - | - |
| 5987 | ENSP00000262366 | GLIS2 | 3.575E-05 | -0.175 | - | - |
| 5988 | ENSP00000362690 | NR5A1 | 4.899E-05 | -0.175 | - | - |
| 5989 | ENSP00000361598 | ZSWIM1 | 8.465E-03 | -0.175 | - | - |
| 5990 | ENSP00000367096 | PCDH9 | 6.009E-05 | -0.175 | - | - |
| 5991 | ENSP00000370990 | SHOX | 3.214E-05 | -0.175 | - | - |
| 5992 | ENSP00000388107 | UBA52 | 5.526E-05 | -0.175 | - | - |
| 5993 | ENSP00000295025 | REL | 7.637E-04 | -0.175 | - | - |
| 5994 | ENSP00000257604 | TRAFD1 | 1.530E-04 | -0.175 | - | - |
| 5995 | ENSP00000340328 | NYX | 1.417E-05 | -0.175 | - | - |
| 5996 | ENSP00000246533 | CAPNS1 | 3.813E-05 | -0.175 | - | - |
| 5997 | ENSP00000371606 | KRTAP5-1 | 1.102E-05 | -0.175 | - | - |
| 5998 | ENSP00000442656 | ACVR1B | 5.480E-05 | -0.175 | - | - |
| 5999 | ENSP00000226840 | AADAT | 5.794E-05 | -0.175 | - | - |
| 6000 | ENSP00000328405 | DGKA | 4.861E-05 | -0.175 | - | - |
| 6001 | ENSP00000379213 | PTHLH | 5.439E-05 | -0.175 | - | - |
| 6002 | ENSP00000164139 | PYGM | 2.139E-05 | -0.175 | - | - |
| 6003 | ENSP00000013807 | ERCC1 | 3.141E-05 | -0.175 | - | - |
| 6004 | ENSP00000351327 | AKAP4 | 2.035E-05 | -0.175 | - | - |
| 6005 | ENSP00000345530 | NEDD4 | 4.339E-05 | -0.175 | - | - |
| 6006 | ENSP00000267485 | C14orf37 | 3.823E-05 | -0.175 | - | - |
| 6007 | ENSP00000393876 | ZNF655 | 3.836E-05 | -0.175 | - | - |
| 6008 | ENSP00000348145 | C14orf178 | 1.073E-04 | -0.175 | - | - |
| 6009 | ENSP00000349275 | NRG1 | 4.478E-05 | -0.175 | - | - |
| 6010 | ENSP00000262055 | LETMD1 | 4.994E-04 | -0.175 | - | - |
| 6011 | ENSP00000298690 | RNASE7 | 1.380E-04 | -0.175 | - | - |
| 6012 | ENSP00000332326 | TNFAIP2 | 3.631E-05 | -0.175 | - | - |
| 6013 | ENSP00000351040 | FAM217B | 1.374E-01 | -0.175 | - | - |
| 6014 | ENSP00000404860 | SCHIP1 | 1.540E-05 | -0.175 | - | - |
| 6015 | ENSP00000239940 | PFN2 | 2.406E-05 | -0.175 | - | - |
| 6016 | ENSP00000272732 | SCRN3 | 1.485E-04 | -0.175 | - | - |
| 6017 | ENSP00000340200 | GLYAT | 4.889E-05 | -0.175 | - | - |
| 6018 | ENSP00000310568 | KCNK10 | 4.622E-05 | -0.175 | - | - |
| 6019 | ENSP00000369419 | STEAP4 | 1.319E-05 | -0.175 | - | - |
| 6020 | ENSP00000368667 | DDX53 | 5.932E-05 | -0.175 | - | - |
| 6021 | ENSP00000262626 | HPN | 4.447E-05 | -0.175 | - | - |
| 6022 | ENSP00000384015 | SUN1 | 1.102E-05 | -0.175 | - | - |
| 6023 | ENSP00000459775 | ANKFY1 | 4.833E-05 | -0.175 | - | - |
| 6024 | ENSP00000371572 | C1QTNF9B | 1.324E-05 | -0.176 | - | - |
| 6025 | ENSP00000370588 | CD99 | 2.051E-05 | -0.176 | - | - |
| 6026 | ENSP00000371497 | C1QTNF3 | 1.828E-05 | -0.176 | - | - |
| 6027 | ENSP00000336775 | SYNM | 3.785E-05 | -0.176 | - | - |
| 6028 | ENSP00000355979 | SERTAD4 | 2.277E-04 | -0.176 | - | - |
| 6029 | ENSP00000308236 | COMMD1 | 7.620E-05 | -0.176 | - | - |
| 6030 | ENSP00000263576 | DDX25 | 3.793E-05 | -0.176 | - | - |
| 6031 | ENSP00000308937 | PMP22 | 4.703E-05 | -0.176 | - | - |
| 6032 | ENSP00000398003 | U82695.9 | 7.677E-06 | -0.176 | - | - |
| 6033 | ENSP00000355340 | APOBEC3C | 1.618E-04 | -0.176 | - | - |
| 6034 | ENSP00000307260 | TBL2 | 9.975E-06 | -0.176 | - | - |
| 6035 | ENSP00000397157 | PERP | 1.743E-04 | -0.176 | - | - |
| 6036 | ENSP00000345752 | MTMR2 | 4.521E-05 | -0.176 | - | - |
| 6037 | ENSP00000391372 | SH3GL3 | 2.766E-05 | -0.176 | - | - |
| 6038 | ENSP00000328511 | KCNA4 | 2.730E-05 | -0.176 | - | - |
| 6039 | ENSP00000216862 | CYP24A1 | 4.617E-05 | -0.176 | - | - |
| 6040 | ENSP00000234179 | PRKD3 | 4.748E-05 | -0.176 | - | - |
| 6041 | ENSP00000305426 | TUB | 5.225E-05 | -0.176 | - | - |
| 6042 | ENSP00000219746 | TOX3 | 2.236E-05 | -0.176 | - | - |
| 6043 | ENSP00000222157 | FGF21 | 5.262E-05 | -0.176 | - | - |
| 6044 | ENSP00000397178 | ZNF300 | 1.030E-05 | -0.176 | - | - |
| 6045 | ENSP00000306279 | UMOD | 3.554E-05 | -0.176 | - | - |
| 6046 | ENSP00000377374 | ERGIC1 | 4.479E-05 | -0.176 | - | - |
| 6047 | ENSP00000310729 | HERPUD2 | 2.097E-04 | -0.176 | - | - |
| 6048 | ENSP00000332744 | ALX4 | 4.551E-05 | -0.176 | - | - |
| 6049 | ENSP00000300289 | PDIA3 | 2.769E-05 | -0.176 | - | - |
| 6050 | ENSP00000376822 | STEAP3 | 3.962E-05 | -0.176 | - | - |
| 6051 | ENSP00000247866 | NDUFB2 | 2.409E-05 | -0.176 | - | - |
| 6052 | ENSP00000253193 | LRP3 | 3.654E-05 | -0.176 | - | - |
| 6053 | ENSP00000231572 | RARS | 2.460E-05 | -0.176 | - | - |
| 6054 | ENSP00000217740 | RNF125 | 7.314E-05 | -0.176 | - | - |
| 6055 | ENSP00000383866 | SZRD1 | 3.249E-04 | -0.176 | - | - |
| 6056 | ENSP00000244050 | SNAI1 | 4.634E-05 | -0.176 | - | - |
| 6057 | ENSP00000270509 | FBN3 | 1.040E-04 | -0.176 | - | - |
| 6058 | ENSP00000349098 | ZNF74 | 1.356E-05 | -0.176 | - | - |
| 6059 | ENSP00000418287 | CIAO1 | 1.275E-05 | -0.176 | - | - |
| 6060 | ENSP00000266579 | SLC38A4 | 1.039E-05 | -0.176 | - | - |
| 6061 | ENSP00000421592 | ALYREF | 5.132E-05 | -0.176 | - | - |
| 6062 | ENSP00000259874 | IER3 | 1.263E-04 | -0.176 | - | - |
| 6063 | ENSP00000228510 | MVK | 5.252E-05 | -0.176 | - | - |
| 6064 | ENSP00000355249 | E2F2 | 2.811E-05 | -0.176 | - | - |
| 6065 | ENSP00000317087 | 8-Mar | 5.782E-05 | -0.176 | - | - |
| 6066 | ENSP00000367355 | ARHGEF35 | 3.645E-05 | -0.176 | - | - |
| 6067 | ENSP00000247270 | EVI2A | 1.172E-04 | -0.176 | - | - |
| 6068 | ENSP00000369702 | LHFPL2 | 1.859E-05 | -0.176 | - | - |
| 6069 | ENSP00000324101 | CD151 | 6.510E-05 | -0.176 | - | - |
| 6070 | ENSP00000384026 | HMGA2 | 5.343E-05 | -0.176 | - | - |
| 6071 | ENSP00000367808 | C1orf86 | 9.766E-05 | -0.176 | - | - |
| 6072 | ENSP00000348234 | TAT | 2.953E-05 | -0.176 | - | - |
| 6073 | ENSP00000296839 | FOXQ1 | 1.284E-05 | -0.176 | - | - |
| 6074 | ENSP00000304336 | PYDC1 | 2.554E-05 | -0.176 | - | - |
| 6075 | ENSP00000357799 | S100A10 | 5.749E-05 | -0.176 | - | - |
| 6076 | ENSP00000263946 | PKP1 | 3.564E-05 | -0.177 | - | - |
| 6077 | ENSP00000319140 | PSG11 | 1.456E-05 | -0.177 | - | - |
| 6078 | ENSP00000278379 | SLC1A2 | 3.465E-05 | -0.177 | - | - |
| 6079 | ENSP00000303580 | NKIRAS2 | 7.615E-05 | -0.177 | - | - |
| 6080 | ENSP00000262244 | MOB3B | 7.810E-05 | -0.177 | - | - |
| 6081 | ENSP00000244766 | NRN1 | 3.247E-05 | -0.177 | - | - |
| 6082 | ENSP00000362979 | TGIF2 | 4.901E-05 | -0.177 | - | - |
| 6083 | ENSP00000372547 | SRY | 5.892E-05 | -0.177 | - | - |
| 6084 | ENSP00000238609 | IFI27L2 | 8.596E-05 | -0.177 | - | - |
| 6085 | ENSP00000262178 | VIPR2 | 2.332E-05 | -0.177 | - | - |
| 6086 | ENSP00000457957 | MEF2BNB-MEF2B | 4.302E-06 | -0.177 | - | - |
| 6087 | ENSP00000372649 | PRR23A | 2.098E-05 | -0.177 | - | - |
| 6088 | ENSP00000320563 | WDFY4 | 5.768E-05 | -0.177 | - | - |
| 6089 | ENSP00000380450 | NOXO1 | 1.876E-05 | -0.177 | - | - |
| 6090 | ENSP00000377721 | NR2F2 | 4.889E-05 | -0.177 | - | - |
| 6091 | ENSP00000348933 | CCDC25 | 3.814E-05 | -0.177 | - | - |
| 6092 | ENSP00000357047 | MED23 | 5.947E-05 | -0.177 | - | - |
| 6093 | ENSP00000281321 | POU4F2 | 1.922E-05 | -0.177 | - | - |
| 6094 | ENSP00000259351 | RALGPS1 | 4.045E-05 | -0.177 | - | - |
| 6095 | ENSP00000375598 | ZNF628 | 1.259E-05 | -0.177 | - | - |
| 6096 | ENSP00000356630 | HECA | 8.336E-05 | -0.177 | - | - |
| 6097 | ENSP00000315112 | RNF31 | 8.625E-05 | -0.177 | - | - |
| 6098 | ENSP00000338814 | BAG5 | 6.328E-05 | -0.177 | - | - |
| 6099 | ENSP00000323895 | OR10D3 | 6.544E-05 | -0.177 | - | - |
| 6100 | ENSP00000375820 | ZNF233 | 1.030E-05 | -0.177 | - | - |
| 6101 | ENSP00000283290 | ATG3 | 3.821E-05 | -0.177 | - | - |
| 6102 | ENSP00000317382 | SLC36A4 | 5.799E-05 | -0.177 | - | - |
| 6103 | ENSP00000432561 | HTN3 | 3.541E-05 | -0.177 | - | - |
| 6104 | ENSP00000222329 | ERF | 7.740E-05 | -0.177 | - | - |
| 6105 | ENSP00000242285 | CLTA | 3.994E-05 | -0.177 | - | - |
| 6106 | ENSP00000373274 | ZNF860 | 1.031E-05 | -0.177 | - | - |
| 6107 | ENSP00000379372 | GTF2A2 | 4.510E-05 | -0.177 | - | - |
| 6108 | ENSP00000320081 | C3orf58 | 7.877E-06 | -0.177 | - | - |
| 6109 | ENSP00000361463 | AL391421.1 | 2.017E-05 | -0.177 | - | - |
| 6110 | ENSP00000351530 | ZXDA | 1.271E-05 | -0.177 | - | - |
| 6111 | ENSP00000254900 | BRD8 | 1.843E-04 | -0.177 | - | - |
| 6112 | ENSP00000295571 | EOGT | 1.183E-04 | -0.177 | - | - |
| 6113 | ENSP00000265085 | CPEB4 | 4.028E-05 | -0.177 | - | - |
| 6114 | ENSP00000358599 | SMIM11P1 | 3.285E-06 | -0.177 | - | - |
| 6115 | ENSP00000363524 | KIF4A | 3.952E-05 | -0.177 | - | - |
| 6116 | ENSP00000259470 | CTSL2 | 5.579E-05 | -0.177 | - | - |
| 6117 | ENSP00000220509 | VPS18 | 3.853E-05 | -0.177 | - | - |
| 6118 | ENSP00000257622 | POM121 | 8.152E-05 | -0.177 | - | - |
| 6119 | ENSP00000339881 | MIOS | 5.219E-05 | -0.177 | - | - |
| 6120 | ENSP00000289316 | HIST1H2BD | 2.680E-05 | -0.177 | - | - |
| 6121 | ENSP00000301327 | MFSD3 | 4.233E-05 | -0.177 | - | - |
| 6122 | ENSP00000279463 | CNTN5 | 4.418E-05 | -0.177 | - | - |
| 6123 | ENSP00000302913 | SH3D19 | 7.013E-05 | -0.178 | - | - |
| 6124 | ENSP00000385927 | RMDN1 | 3.696E-05 | -0.178 | - | - |
| 6125 | ENSP00000344758 | ECM2 | 5.596E-05 | -0.178 | - | - |
| 6126 | ENSP00000247226 | PLEKHG3 | 2.530E-04 | -0.178 | - | - |
| 6127 | ENSP00000364382 | AAED1 | 1.261E-04 | -0.178 | - | - |
| 6128 | ENSP00000257572 | HRK | 1.707E-04 | -0.178 | - | - |
| 6129 | ENSP00000273153 | CSRNP1 | 5.360E-05 | -0.178 | - | - |
| 6130 | ENSP00000354270 | FAM107A | 5.031E-05 | -0.178 | - | - |
| 6131 | ENSP00000282382 | TMED7-TICAM2 | 8.890E-05 | -0.178 | - | - |
| 6132 | ENSP00000263610 | BARHL1 | 5.532E-05 | -0.178 | - | - |
| 6133 | ENSP00000304467 | THOP1 | 4.655E-05 | -0.178 | - | - |
| 6134 | ENSP00000305372 | ADRB2 | 8.536E-05 | -0.178 | - | - |
| 6135 | ENSP00000367755 | CAMKMT | 8.322E-05 | -0.178 | - | - |
| 6136 | ENSP00000354415 | MICU1 | 2.444E-05 | -0.178 | - | - |
| 6137 | ENSP00000333640 | EYA2 | 6.343E-05 | -0.178 | - | - |
| 6138 | ENSP00000317636 | RECQL5 | 3.922E-05 | -0.178 | - | - |
| 6139 | ENSP00000357079 | CCDC19 | 9.532E-05 | -0.178 | - | - |
| 6140 | ENSP00000261368 | SNCAIP | 2.963E-05 | -0.178 | - | - |
| 6141 | ENSP00000221166 | NEFM | 4.656E-05 | -0.178 | - | - |
| 6142 | ENSP00000371073 | PLCXD1 | 4.921E-05 | -0.178 | - | - |
| 6143 | ENSP00000339381 | RGS12 | 4.795E-05 | -0.178 | - | - |
| 6144 | ENSP00000354111 | DNAJC5 | 1.891E-05 | -0.178 | - | - |
| 6145 | ENSP00000358605 | SMNDC1 | 5.248E-05 | -0.178 | - | - |
| 6146 | ENSP00000262340 | RPE65 | 3.998E-05 | -0.178 | - | - |
| 6147 | ENSP00000207870 | XYLB | 5.124E-05 | -0.178 | - | - |
| 6148 | ENSP00000267984 | MESDC1 | 7.379E-05 | -0.178 | - | - |
| 6149 | ENSP00000309376 | CD164 | 1.346E-04 | -0.178 | - | - |
| 6150 | ENSP00000328875 | TRAK2 | 1.050E-04 | -0.178 | - | - |
| 6151 | ENSP00000354680 | ZNF266 | 1.313E-05 | -0.178 | - | - |
| 6152 | ENSP00000259229 | CCDC115 | 4.988E-04 | -0.178 | - | - |
| 6153 | ENSP00000308479 | GPR171 | 4.381E-05 | -0.178 | - | - |
| 6154 | ENSP00000265195 | SIL1 | 3.470E-05 | -0.178 | - | - |
| 6155 | ENSP00000033079 | FAM13B | 9.943E-05 | -0.178 | - | - |
| 6156 | ENSP00000467889 | ZNF607 | 1.360E-05 | -0.178 | - | - |
| 6157 | ENSP00000320246 | SLC9A9 | 1.626E-05 | -0.178 | - | - |
| 6158 | ENSP00000386139 | C5orf55 | 9.100E-06 | -0.178 | - | - |
| 6159 | ENSP00000314806 | FOXR1 | 1.617E-05 | -0.178 | - | - |
| 6160 | ENSP00000317580 | NEUROG1 | 3.405E-05 | -0.178 | - | - |
| 6161 | ENSP00000368589 | GFOD1 | 1.726E-05 | -0.178 | - | - |
| 6162 | ENSP00000359804 | FUBP1 | 4.223E-05 | -0.178 | - | - |
| 6163 | ENSP00000040584 | HOXC8 | 3.973E-05 | -0.178 | - | - |
| 6164 | ENSP00000264689 | UFSP2 | 1.137E-04 | -0.178 | - | - |
| 6165 | ENSP00000456648 | TUBGCP4 | 2.542E-05 | -0.178 | - | - |
| 6166 | ENSP00000332151 | DSE | 3.519E-05 | -0.178 | - | - |
| 6167 | ENSP00000376048 | MAG | 5.215E-05 | -0.178 | - | - |
| 6168 | ENSP00000259486 | ENPP2 | 3.359E-05 | -0.178 | - | - |
| 6169 | ENSP00000263671 | CHRDL2 | 2.609E-05 | -0.178 | - | - |
| 6170 | ENSP00000305529 | SIRPG | 9.518E-05 | -0.178 | - | - |
| 6171 | ENSP00000296657 | ANKRD33B | 3.373E-05 | -0.178 | - | - |
| 6172 | ENSP00000357881 | IKZF5 | 1.325E-05 | -0.178 | - | - |
| 6173 | ENSP00000262904 | E2F3 | 2.983E-05 | -0.178 | - | - |
| 6174 | ENSP00000406229 | FAT1 | 5.026E-05 | -0.178 | - | - |
| 6175 | ENSP00000371388 | SRXN1 | 1.817E-05 | -0.178 | - | - |
| 6176 | ENSP00000245908 | SH2D3A | 1.495E-04 | -0.178 | - | - |
| 6177 | ENSP00000371278 | CDC37L1 | 2.105E-05 | -0.178 | - | - |
| 6178 | ENSP00000253329 | PPIL4 | 3.076E-05 | -0.179 | - | - |
| 6179 | ENSP00000266673 | TMEM19 | 2.392E-04 | -0.179 | - | - |
| 6180 | ENSP00000378349 | RCN2 | 1.172E-05 | -0.179 | - | - |
| 6181 | ENSP00000352319 | ARSF | 6.876E-06 | -0.179 | - | - |
| 6182 | ENSP00000401597 | DTHD1 | 2.205E-06 | -0.179 | - | - |
| 6183 | ENSP00000331288 | TMEM173 | 5.169E-05 | -0.179 | - | - |
| 6184 | ENSP00000376010 | ZNF566 | 1.374E-05 | -0.179 | - | - |
| 6185 | ENSP00000262888 | KCNN4 | 3.743E-05 | -0.179 | - | - |
| 6186 | ENSP00000437910 | MTRNR2L5 | 7.096E-06 | -0.179 | - | - |
| 6187 | ENSP00000350894 | SERPINH1 | 4.817E-05 | -0.179 | - | - |
| 6188 | ENSP00000349773 | CDC42SE1 | 3.848E-05 | -0.179 | - | - |
| 6189 | ENSP00000258428 | REV1 | 3.150E-05 | -0.179 | - | - |
| 6190 | ENSP00000371307 | PPAPDC2 | 7.421E-06 | -0.179 | - | - |
| 6191 | ENSP00000339912 | FLRT3 | 1.299E-05 | -0.179 | - | - |
| 6192 | ENSP00000232219 | RBP1 | 4.736E-05 | -0.179 | - | - |
| 6193 | ENSP00000000412 | M6PR | 3.647E-05 | -0.179 | - | - |
| 6194 | ENSP00000353093 | TCEAL8 | 2.451E-04 | -0.179 | - | - |
| 6195 | ENSP00000362217 | DACH2 | 4.883E-05 | -0.179 | - | - |
| 6196 | ENSP00000352264 | CD2AP | 3.221E-05 | -0.179 | - | - |
| 6197 | ENSP00000289746 | CDH15 | 5.446E-05 | -0.179 | - | - |
| 6198 | ENSP00000272430 | RTKN | 5.444E-05 | -0.179 | - | - |
| 6199 | ENSP00000295666 | IGFBP7 | 4.901E-05 | -0.179 | - | - |
| 6200 | ENSP00000339813 | TPST2 | 7.126E-05 | -0.179 | - | - |
| 6201 | ENSP00000313571 | LGALS7B | 3.359E-05 | -0.179 | - | - |
| 6202 | ENSP00000254250 | THAP1 | 3.481E-05 | -0.179 | - | - |
| 6203 | ENSP00000318977 | GEN1 | 2.605E-05 | -0.179 | - | - |
| 6204 | ENSP00000364895 | ZBTB17 | 1.840E-05 | -0.179 | - | - |
| 6205 | ENSP00000430620 | HEPH | 3.322E-05 | -0.179 | - | - |
| 6206 | ENSP00000263733 | FAM20B | 1.301E-04 | -0.179 | - | - |
| 6207 | ENSP00000338457 | HPS4 | 3.579E-05 | -0.179 | - | - |
| 6208 | ENSP00000304169 | PITX2 | 2.656E-05 | -0.179 | - | - |
| 6209 | ENSP00000348538 | RBM38 | 9.266E-05 | -0.179 | - | - |
| 6210 | ENSP00000370812 | ESM1 | 2.498E-05 | -0.179 | - | - |
| 6211 | ENSP00000374409 | PKP4 | 2.653E-05 | -0.179 | - | - |
| 6212 | ENSP00000351280 | ZNF429 | 2.192E-05 | -0.179 | - | - |
| 6213 | ENSP00000340083 | KRCC1 | 6.463E-04 | -0.179 | - | - |
| 6214 | ENSP00000307340 | SPRR1A | 7.646E-05 | -0.179 | - | - |
| 6215 | ENSP00000282493 | PDZD2 | 2.249E-05 | -0.179 | - | - |
| 6216 | ENSP00000276344 | MAGEA4 | 8.107E-05 | -0.179 | - | - |
| 6217 | ENSP00000263207 | ARVCF | 3.320E-05 | -0.179 | - | - |
| 6218 | ENSP00000370889 | DENND1C | 9.708E-05 | -0.179 | - | - |
| 6219 | ENSP00000331912 | GABRG3 | 9.246E-06 | -0.179 | - | - |
| 6220 | ENSP00000343464 | HELT | 3.248E-05 | -0.179 | - | - |
| 6221 | ENSP00000406482 | THSD7A | 4.781E-05 | -0.179 | - | - |
| 6222 | ENSP00000400836 | ZBTB8B | 1.099E-05 | -0.179 | - | - |
| 6223 | ENSP00000354305 | ZSCAN12 | 1.157E-05 | -0.180 | - | - |
| 6224 | ENSP00000346065 | TDRD6 | 5.080E-05 | -0.180 | - | - |
| 6225 | ENSP00000246190 | NECAB3 | 1.315E-04 | -0.180 | - | - |
| 6226 | ENSP00000334696 | KRTAP19-7 | 2.360E-05 | -0.180 | - | - |
| 6227 | ENSP00000354911 | TIFA | 9.441E-05 | -0.180 | - | - |
| 6228 | ENSP00000339007 | GRB2 | 5.066E-04 | -0.180 | - | - |
| 6229 | ENSP00000314946 | RPRM | 8.269E-05 | -0.180 | - | - |
| 6230 | ENSP00000375482 | KRTAP6-3 | 1.272E-05 | -0.180 | - | - |
| 6231 | ENSP00000355809 | ENAH | 4.144E-05 | -0.180 | - | - |
| 6232 | ENSP00000044462 | PSMA4 | 2.919E-05 | -0.180 | - | - |
| 6233 | ENSP00000324648 | CYP2B6 | 3.961E-05 | -0.180 | - | - |
| 6234 | ENSP00000269389 | SECTM1 | 1.488E-04 | -0.180 | - | - |
| 6235 | ENSP00000263209 | DGCR8 | 3.377E-05 | -0.180 | - | - |
| 6236 | ENSP00000367453 | ACBD7 | 3.046E-05 | -0.180 | - | - |
| 6237 | ENSP00000345292 | NDRG3 | 6.032E-05 | -0.180 | - | - |
| 6238 | ENSP00000269554 | PIP4K2B | 3.445E-05 | -0.180 | - | - |
| 6239 | ENSP00000281092 | FER | 2.779E-05 | -0.180 | - | - |
| 6240 | ENSP00000398342 | PPP1R14D | 1.802E-05 | -0.180 | - | - |
| 6241 | ENSP00000261837 | GNB5 | 3.841E-05 | -0.180 | - | - |
| 6242 | ENSP00000303554 | PTPN9 | 4.911E-05 | -0.180 | - | - |
| 6243 | ENSP00000290401 | NPTN | 5.208E-05 | -0.180 | - | - |
| 6244 | ENSP00000327975 | DHRS7C | 2.211E-05 | -0.180 | - | - |
| 6245 | ENSP00000302120 | TRIM8 | 5.954E-05 | -0.180 | - | - |
| 6246 | ENSP00000251588 | NARFL | 3.144E-05 | -0.180 | - | - |
| 6247 | ENSP00000366697 | ANKRD20A1 | 2.921E-05 | -0.180 | - | - |
| 6248 | ENSP00000288502 | C9orf91 | 5.154E-03 | -0.180 | - | - |
| 6249 | ENSP00000370532 | GTF3A | 2.909E-05 | -0.180 | - | - |
| 6250 | ENSP00000332215 | PSG3 | 2.390E-05 | -0.180 | - | - |
| 6251 | ENSP00000359215 | TLX1 | 3.259E-05 | -0.180 | - | - |
| 6252 | ENSP00000352841 | CEP57L1 | 2.178E-04 | -0.180 | - | - |
| 6253 | ENSP00000253968 | BARX1 | 6.285E-05 | -0.180 | - | - |
| 6254 | ENSP00000323568 | SLC2A2 | 2.462E-05 | -0.180 | - | - |
| 6255 | ENSP00000355124 | KRT19 | 3.970E-05 | -0.180 | - | - |
| 6256 | ENSP00000368401 | PAX6 | 4.753E-05 | -0.180 | - | - |
| 6257 | ENSP00000174618 | MNT | 2.057E-05 | -0.180 | - | - |
| 6258 | ENSP00000259803 | GCM1 | 7.379E-05 | -0.180 | - | - |
| 6259 | ENSP00000356898 | DDR2 | 8.147E-05 | -0.180 | - | - |
| 6260 | ENSP00000353575 | EIF2B3 | 2.180E-05 | -0.180 | - | - |
| 6261 | ENSP00000371061 | C5orf51 | 6.959E-04 | -0.180 | - | - |
| 6262 | ENSP00000342109 | SERPINA12 | 7.854E-05 | -0.180 | - | - |
| 6263 | ENSP00000345997 | DMPK | 6.710E-05 | -0.180 | - | - |
| 6264 | ENSP00000303356 | DCXR | 3.480E-05 | -0.180 | - | - |
| 6265 | ENSP00000302189 | NHLH1 | 3.594E-05 | -0.180 | - | - |
| 6266 | ENSP00000311905 | LTBP4 | 1.317E-04 | -0.180 | - | - |
| 6267 | ENSP00000318822 | BID | 9.072E-05 | -0.180 | - | - |
| 6268 | ENSP00000360913 | SPATA6 | 7.820E-05 | -0.180 | - | - |
| 6269 | ENSP00000202556 | PPP1R13B | 7.093E-05 | -0.180 | - | - |
| 6270 | ENSP00000264658 | FBXL20 | 5.849E-05 | -0.180 | - | - |
| 6271 | ENSP00000419718 | OFCC1 | 2.563E-06 | -0.180 | - | - |
| 6272 | ENSP00000352513 | MPZL1 | 2.021E-04 | -0.180 | - | - |
| 6273 | ENSP00000381672 | TP53TG3 | 3.437E-03 | -0.180 | - | - |
| 6274 | ENSP00000072644 | YIPF1 | 7.482E-05 | -0.180 | - | - |
| 6275 | ENSP00000289953 | WFDC8 | 1.240E-04 | -0.180 | - | - |
| 6276 | ENSP00000290399 | SIM2 | 5.836E-05 | -0.180 | - | - |
| 6277 | ENSP00000363136 | GPR3 | 6.427E-05 | -0.180 | - | - |
| 6278 | ENSP00000368798 | MBTPS2 | 3.066E-05 | -0.180 | - | - |
| 6279 | ENSP00000371789 | ZCCHC3 | 3.330E-03 | -0.180 | - | - |
| 6280 | ENSP00000367196 | GLIPR2 | 6.616E-05 | -0.180 | - | - |
| 6281 | ENSP00000269228 | NPC1 | 4.195E-05 | -0.180 | - | - |
| 6282 | ENSP00000362068 | TAF8 | 7.632E-06 | -0.180 | - | - |
| 6283 | ENSP00000353971 | TTI2 | 1.157E-04 | -0.181 | - | - |
| 6284 | ENSP00000261590 | DSG2 | 5.090E-05 | -0.181 | - | - |
| 6285 | ENSP00000332602 | CTAG1B | 1.835E-04 | -0.181 | - | - |
| 6286 | ENSP00000266754 | GAS2L3 | 7.227E-05 | -0.181 | - | - |
| 6287 | ENSP00000303508 | FRMD3 | 1.276E-05 | -0.181 | - | - |
| 6288 | ENSP00000340935 | DMP1 | 5.510E-05 | -0.181 | - | - |
| 6289 | ENSP00000330200 | BTNL9 | 2.974E-05 | -0.181 | - | - |
| 6290 | ENSP00000314099 | CA5B | 7.434E-05 | -0.181 | - | - |
| 6291 | ENSP00000315351 | D2HGDH | 4.765E-05 | -0.181 | - | - |
| 6292 | ENSP00000355319 | KIAA1737 | 1.101E-03 | -0.181 | - | - |
| 6293 | ENSP00000346981 | TMEM207 | 1.428E-03 | -0.181 | - | - |
| 6294 | ENSP00000233336 | TTL | 2.426E-05 | -0.181 | - | - |
| 6295 | ENSP00000380116 | C19orf55 | 4.963E-04 | -0.181 | - | - |
| 6296 | ENSP00000274341 | HAPLN1 | 4.992E-05 | -0.181 | - | - |
| 6297 | ENSP00000204679 | GNPTG | 5.564E-05 | -0.181 | - | - |
| 6298 | ENSP00000259748 | FRS3 | 1.416E-04 | -0.181 | - | - |
| 6299 | ENSP00000264169 | ORC4 | 6.987E-05 | -0.181 | - | - |
| 6300 | ENSP00000327608 | ADAMTSL5 | 1.370E-04 | -0.181 | - | - |
| 6301 | ENSP00000440920 | C14orf132 | 9.887E-07 | -0.181 | - | - |
| 6302 | ENSP00000384563 | OR6B2 | 2.414E-05 | -0.181 | - | - |
| 6303 | ENSP00000429200 | SLC25A37 | 6.325E-05 | -0.181 | - | - |
| 6304 | ENSP00000381204 | TMEM123 | 2.831E-04 | -0.181 | - | - |
| 6305 | ENSP00000344277 | DOK4 | 7.113E-05 | -0.181 | - | - |
| 6306 | ENSP00000408411 | LAMP2 | 4.811E-05 | -0.181 | - | - |
| 6307 | ENSP00000366317 | C10orf114 | 1.441E-03 | -0.181 | - | - |
| 6308 | ENSP00000377665 | GAPVD1 | 6.919E-05 | -0.181 | - | - |
| 6309 | ENSP00000299633 | HDGFRP3 | 1.974E-04 | -0.181 | - | - |
| 6310 | ENSP00000311399 | DSCR3 | 5.219E-05 | -0.181 | - | - |
| 6311 | ENSP00000369431 | CAAP1 | 1.774E-04 | -0.181 | - | - |
| 6312 | ENSP00000233627 | NDUFS7 | 3.122E-05 | -0.181 | - | - |
| 6313 | ENSP00000355089 | CELF4 | 4.006E-05 | -0.181 | - | - |
| 6314 | ENSP00000229903 | SAYSD1 | 1.050E-03 | -0.181 | - | - |
| 6315 | ENSP00000309649 | CA5A | 6.945E-05 | -0.181 | - | - |
| 6316 | ENSP00000282223 | SPOCK1 | 3.107E-05 | -0.181 | - | - |
| 6317 | ENSP00000267970 | TSPAN3 | 5.875E-05 | -0.181 | - | - |
| 6318 | ENSP00000333394 | FAM104B | 1.621E-03 | -0.181 | - | - |
| 6319 | ENSP00000363520 | NIPAL3 | 1.019E-04 | -0.181 | - | - |
| 6320 | ENSP00000253407 | C1QL1 | 2.032E-04 | -0.181 | - | - |
| 6321 | ENSP00000334130 | C11orf30 | 7.855E-05 | -0.181 | - | - |
| 6322 | ENSP00000339157 | CPM | 5.031E-05 | -0.181 | - | - |
| 6323 | ENSP00000262316 | RHBDF1 | 3.911E-05 | -0.181 | - | - |
| 6324 | ENSP00000442163 | AL590867.1 | 3.063E-04 | -0.181 | - | - |
| 6325 | ENSP00000219281 | USB1 | 1.063E-04 | -0.181 | - | - |
| 6326 | ENSP00000369217 | ARIH1 | 4.807E-05 | -0.181 | - | - |
| 6327 | ENSP00000386896 | ITGA6 | 7.339E-05 | -0.181 | - | - |
| 6328 | ENSP00000363441 | SYT15 | 2.593E-04 | -0.181 | - | - |
| 6329 | ENSP00000312304 | TPMT | 1.173E-04 | -0.181 | - | - |
| 6330 | ENSP00000382231 | SMIM11 | 1.501E-04 | -0.181 | - | - |
| 6331 | ENSP00000442633 | - | 1.322E-05 | -0.181 | - | - |
| 6332 | ENSP00000327959 | TGIF1 | 3.600E-05 | -0.181 | - | - |
| 6333 | ENSP00000377461 | ZNF559 | 1.253E-05 | -0.181 | - | - |
| 6334 | ENSP00000286364 | RASA2 | 5.656E-05 | -0.181 | - | - |
| 6335 | ENSP00000350716 | TUSC1 | 7.562E-05 | -0.181 | - | - |
| 6336 | ENSP00000343505 | TNFSF13 | 2.584E-04 | -0.181 | - | - |
| 6337 | ENSP00000358458 | FAM46C | 3.005E-05 | -0.181 | - | - |
| 6338 | ENSP00000262477 | RABEP1 | 3.223E-05 | -0.181 | - | - |
| 6339 | ENSP00000363416 | AUNIP | 1.264E-04 | -0.181 | - | - |
| 6340 | ENSP00000064724 | CLDN11 | 2.016E-05 | -0.181 | - | - |
| 6341 | ENSP00000325146 | COL12A1 | 6.536E-05 | -0.181 | - | - |
| 6342 | ENSP00000372720 | TMEM241 | 2.046E-04 | -0.181 | - | - |
| 6343 | ENSP00000310800 | VANGL1 | 6.163E-05 | -0.181 | - | - |
| 6344 | ENSP00000350094 | QRICH1 | 2.444E-05 | -0.181 | - | - |
| 6345 | ENSP00000328938 | AFMID | 5.880E-05 | -0.181 | - | - |
| 6346 | ENSP00000249861 | THAP10 | 1.427E-04 | -0.181 | - | - |
| 6347 | ENSP00000360104 | LEPROT | 5.190E-05 | -0.181 | - | - |
| 6348 | ENSP00000216115 | BIK | 1.122E-04 | -0.181 | - | - |
| 6349 | ENSP00000361001 | EFCAB14 | 7.923E-05 | -0.181 | - | - |
| 6350 | ENSP00000304410 | C2orf68 | 1.048E-04 | -0.181 | - | - |
| 6351 | ENSP00000273075 | DNPEP | 4.767E-05 | -0.181 | - | - |
| 6352 | ENSP00000363556 | ZNF32 | 1.135E-05 | -0.181 | - | - |
| 6353 | ENSP00000261318 | C12orf49 | 4.580E-04 | -0.181 | - | - |
| 6354 | ENSP00000262510 | NLRC5 | 1.167E-04 | -0.181 | - | - |
| 6355 | ENSP00000293831 | EIF4A1 | 6.125E-05 | -0.181 | - | - |
| 6356 | ENSP00000322457 | SIAH2 | 4.664E-05 | -0.181 | - | - |
| 6357 | ENSP00000357459 | ADAR | 5.582E-05 | -0.181 | - | - |
| 6358 | ENSP00000368405 | GDPGP1 | 2.193E-04 | -0.181 | - | - |
| 6359 | ENSP00000468720 | ZNF253 | 1.373E-05 | -0.181 | - | - |
| 6360 | ENSP00000256544 | KATNBL1 | 4.118E-04 | -0.181 | - | - |
| 6361 | ENSP00000255198 | ZBED3 | 1.268E-04 | -0.181 | - | - |
| 6362 | ENSP00000364054 | SPIN3 | 5.676E-05 | -0.181 | - | - |
| 6363 | ENSP00000356849 | POGK | 8.520E-05 | -0.181 | - | - |
| 6364 | ENSP00000348714 | AREL1 | 3.343E-04 | -0.181 | - | - |
| 6365 | ENSP00000357842 | TUFT1 | 1.986E-04 | -0.181 | - | - |
| 6366 | ENSP00000332721 | TANGO2 | 1.068E-04 | -0.181 | - | - |
| 6367 | ENSP00000244601 | HIST1H2BG | 2.326E-05 | -0.181 | - | - |
| 6368 | ENSP00000262547 | DZANK1 | 9.220E-05 | -0.181 | - | - |
| 6369 | ENSP00000368805 | GCM2 | 4.463E-05 | -0.181 | - | - |
| 6370 | ENSP00000291526 | TFF2 | 3.492E-05 | -0.181 | - | - |
| 6371 | ENSP00000391641 | ZNF780B | 1.875E-05 | -0.181 | - | - |
| 6372 | ENSP00000368396 | LYRM9 | 1.052E-03 | -0.181 | - | - |
| 6373 | ENSP00000297323 | ADCY1 | 2.731E-05 | -0.181 | - | - |
| 6374 | ENSP00000351650 | NUDT3 | 5.425E-05 | -0.181 | - | - |
| 6375 | ENSP00000357199 | RRNAD1 | 9.094E-05 | -0.181 | - | - |
| 6376 | ENSP00000306425 | CHCHD7 | 4.522E-05 | -0.181 | - | - |
| 6377 | ENSP00000308533 | GEMIN2 | 4.533E-05 | -0.181 | - | - |
| 6378 | ENSP00000331305 | TOB2 | 7.122E-05 | -0.181 | - | - |
| 6379 | ENSP00000310459 | AKIP1 | 1.396E-04 | -0.182 | - | - |
| 6380 | ENSP00000369636 | PXDC1 | 7.073E-05 | -0.182 | - | - |
| 6381 | ENSP00000380747 | TMEM209 | 5.436E-05 | -0.182 | - | - |
| 6382 | ENSP00000315915 | ANXA2R | 8.184E-05 | -0.182 | - | - |
| 6383 | ENSP00000344218 | C1orf162 | 1.998E-04 | -0.182 | - | - |
| 6384 | ENSP00000325660 | CNTN1 | 3.169E-05 | -0.182 | - | - |
| 6385 | ENSP00000318602 | PRTFDC1 | 3.503E-05 | -0.182 | - | - |
| 6386 | ENSP00000327344 | IGIP | 1.604E-04 | -0.182 | - | - |
| 6387 | ENSP00000266987 | TARBP2 | 3.700E-05 | -0.182 | - | - |
| 6388 | ENSP00000231668 | BNIP1 | 7.775E-05 | -0.182 | - | - |
| 6389 | ENSP00000281623 | FBXO4 | 5.164E-05 | -0.182 | - | - |
| 6390 | ENSP00000371221 | MTMR6 | 7.879E-05 | -0.182 | - | - |
| 6391 | ENSP00000367265 | CKAP4 | 8.539E-05 | -0.182 | - | - |
| 6392 | ENSP00000323889 | TRIM25 | 7.142E-05 | -0.182 | - | - |
| 6393 | ENSP00000362103 | POU3F1 | 2.100E-05 | -0.182 | - | - |
| 6394 | ENSP00000384291 | LRP5L | 2.206E-05 | -0.182 | - | - |
| 6395 | ENSP00000359664 | CTBS | 9.196E-05 | -0.182 | - | - |
| 6396 | ENSP00000366243 | TMEM2 | 5.821E-05 | -0.182 | - | - |
| 6397 | ENSP00000298585 | ZMYND19 | 1.091E-04 | -0.182 | - | - |
| 6398 | ENSP00000220531 | BLOC1S6 | 4.733E-05 | -0.182 | - | - |
| 6399 | ENSP00000340954 | CNBD2 | 3.784E-05 | -0.182 | - | - |
| 6400 | ENSP00000264718 | GPN1 | 3.601E-05 | -0.182 | - | - |
| 6401 | ENSP00000384573 | DAZ1 | 1.695E-04 | -0.182 | - | - |
| 6402 | ENSP00000348596 | SDAD1 | 3.769E-05 | -0.182 | - | - |
| 6403 | ENSP00000420270 | UHMK1 | 4.123E-05 | -0.182 | - | - |
| 6404 | ENSP00000257895 | RDH5 | 1.801E-05 | -0.182 | - | - |
| 6405 | ENSP00000378935 | PPHLN1 | 6.877E-05 | -0.182 | - | - |
| 6406 | ENSP00000345789 | MUM1 | 9.645E-05 | -0.182 | - | - |
| 6407 | ENSP00000261593 | RNF138 | 9.358E-05 | -0.182 | - | - |
| 6408 | ENSP00000349087 | TMEM259 | 5.864E-05 | -0.182 | - | - |
| 6409 | ENSP00000349687 | GM2A | 6.894E-05 | -0.182 | - | - |
| 6410 | ENSP00000353998 | VKORC1L1 | 9.981E-05 | -0.182 | - | - |
| 6411 | ENSP00000364613 | C9orf89 | 3.303E-06 | -0.182 | - | - |
| 6412 | ENSP00000336931 | PNRC1 | 5.541E-05 | -0.182 | - | - |
| 6413 | ENSP00000370439 | FLJ27365 | 8.908E-05 | -0.182 | - | - |
| 6414 | ENSP00000371419 | PARP4 | 4.783E-05 | -0.182 | - | - |
| 6415 | ENSP00000233638 | TLX2 | 3.714E-05 | -0.182 | - | - |
| 6416 | ENSP00000261461 | PPP2R5A | 3.096E-05 | -0.182 | - | - |
| 6417 | ENSP00000344616 | ZAR1L | 1.331E-04 | -0.182 | - | - |
| 6418 | ENSP00000352928 | DDA1 | 8.510E-05 | -0.182 | - | - |
| 6419 | ENSP00000361185 | LRIT2 | 7.867E-06 | -0.182 | - | - |
| 6420 | ENSP00000301295 | NLRP4 | 1.157E-04 | -0.182 | - | - |
| 6421 | ENSP00000300091 | C18orf54 | 4.226E-05 | -0.182 | - | - |
| 6422 | ENSP00000372724 | LY6G5C | 4.870E-06 | -0.182 | - | - |
| 6423 | ENSP00000362707 | MANBAL | 8.159E-05 | -0.182 | - | - |
| 6424 | ENSP00000217909 | SLC25A43 | 7.098E-05 | -0.182 | - | - |
| 6425 | ENSP00000322376 | - | 6.269E-05 | -0.182 | - | - |
| 6426 | ENSP00000318607 | LDLRAD3 | 7.790E-05 | -0.182 | - | - |
| 6427 | ENSP00000340494 | ZNF395 | 5.106E-05 | -0.182 | - | - |
| 6428 | ENSP00000359171 | HIAT1 | 5.609E-05 | -0.182 | - | - |
| 6429 | ENSP00000268720 | CPNE7 | 7.079E-05 | -0.182 | - | - |
| 6430 | ENSP00000265029 | FETUB | 8.235E-05 | -0.182 | - | - |
| 6431 | ENSP00000355306 | C1orf174 | 8.444E-05 | -0.182 | - | - |
| 6432 | ENSP00000379669 | MPV17L | 4.957E-05 | -0.182 | - | - |
| 6433 | ENSP00000261692 | CDK2AP1 | 5.283E-05 | -0.182 | - | - |
| 6434 | ENSP00000240851 | TFG | 5.908E-05 | -0.182 | - | - |
| 6435 | ENSP00000203664 | OTUB2 | 6.469E-05 | -0.182 | - | - |
| 6436 | ENSP00000375881 | ALPP | 3.590E-05 | -0.182 | - | - |
| 6437 | ENSP00000339659 | TRIM2 | 6.644E-05 | -0.182 | - | - |
| 6438 | ENSP00000379294 | FAM172A | 6.342E-05 | -0.182 | - | - |
| 6439 | ENSP00000285071 | FLCN | 5.353E-05 | -0.182 | - | - |
| 6440 | ENSP00000298530 | TMEM52B | 9.005E-05 | -0.182 | - | - |
| 6441 | ENSP00000316891 | LIMCH1 | 4.217E-05 | -0.182 | - | - |
| 6442 | ENSP00000349723 | BCL11B | 2.856E-05 | -0.182 | - | - |
| 6443 | ENSP00000261017 | ABI2 | 3.218E-05 | -0.182 | - | - |
| 6444 | ENSP00000454370 | SMKR1 | 4.787E-07 | -0.182 | - | - |
| 6445 | ENSP00000328325 | PTTG1IP | 9.684E-05 | -0.182 | - | - |
| 6446 | ENSP00000311196 | NPAS4 | 3.519E-05 | -0.182 | - | - |
| 6447 | ENSP00000293404 | NAGS | 1.030E-05 | -0.182 | - | - |
| 6448 | ENSP00000358147 | CACUL1 | 2.662E-04 | -0.182 | - | - |
| 6449 | ENSP00000370340 | NFIB | 5.305E-05 | -0.182 | - | - |
| 6450 | ENSP00000317903 | R3HDM2 | 1.208E-04 | -0.182 | - | - |
| 6451 | ENSP00000307706 | MT1E | 8.418E-05 | -0.182 | - | - |
| 6452 | ENSP00000353706 | CDC42SE2 | 7.959E-05 | -0.182 | - | - |
| 6453 | ENSP00000406598 | C4orf27 | 2.973E-05 | -0.182 | - | - |
| 6454 | ENSP00000373476 | MEX3D | 4.257E-05 | -0.182 | - | - |
| 6455 | ENSP00000359364 | YTHDF1 | 5.200E-05 | -0.182 | - | - |
| 6456 | ENSP00000350215 | SEMA5B | 6.857E-05 | -0.182 | - | - |
| 6457 | ENSP00000378996 | NREP | 9.269E-05 | -0.182 | - | - |
| 6458 | ENSP00000367301 | NDP | 4.184E-05 | -0.182 | - | - |
| 6459 | ENSP00000345528 | SLC35F6 | 5.249E-05 | -0.182 | - | - |
| 6460 | ENSP00000312615 | TMEM134 | 6.191E-05 | -0.182 | - | - |
| 6461 | ENSP00000368881 | KIN | 2.782E-05 | -0.182 | - | - |
| 6462 | ENSP00000347560 | PLEKHA5 | 5.805E-05 | -0.182 | - | - |
| 6463 | ENSP00000299957 | CASC4 | 8.228E-05 | -0.182 | - | - |
| 6464 | ENSP00000342012 | DMKN | 5.841E-05 | -0.182 | - | - |
| 6465 | ENSP00000376071 | FAM117B | 5.943E-05 | -0.182 | - | - |
| 6466 | ENSP00000370055 | UBL3 | 8.308E-05 | -0.182 | - | - |
| 6467 | ENSP00000349575 | MOB3A | 8.178E-05 | -0.182 | - | - |
| 6468 | ENSP00000419485 | GCSAM | 1.278E-05 | -0.182 | - | - |
| 6469 | ENSP00000330075 | OGFOD3 | 1.034E-04 | -0.182 | - | - |
| 6470 | ENSP00000269491 | SERPINB12 | 1.166E-04 | -0.182 | - | - |
| 6471 | ENSP00000341652 | TBCCD1 | 9.222E-05 | -0.182 | - | - |
| 6472 | ENSP00000290101 | RAP1GAP | 4.848E-05 | -0.182 | - | - |
| 6473 | ENSP00000272117 | ITPKB | 4.344E-05 | -0.182 | - | - |
| 6474 | ENSP00000182527 | TRAM2 | 4.510E-05 | -0.182 | - | - |
| 6475 | ENSP00000402914 | RP11-685N3.1 | 1.432E-06 | -0.182 | - | - |
| 6476 | ENSP00000254190 | CHSY1 | 3.689E-05 | -0.182 | - | - |
| 6477 | ENSP00000381216 | KHSRP | 6.815E-05 | -0.182 | - | - |
| 6478 | ENSP00000296522 | HPGD | 2.830E-05 | -0.182 | - | - |
| 6479 | ENSP00000356641 | RFWD2 | 4.468E-05 | -0.182 | - | - |
| 6480 | ENSP00000377428 | WDR52 | 4.764E-05 | -0.182 | - | - |
| 6481 | ENSP00000359827 | FAM73A | 7.137E-05 | -0.182 | - | - |
| 6482 | ENSP00000286692 | DRAM2 | 6.605E-05 | -0.182 | - | - |
| 6483 | ENSP00000253719 | NAPSA | 3.965E-05 | -0.182 | - | - |
| 6484 | ENSP00000261407 | LPCAT3 | 6.995E-05 | -0.182 | - | - |
| 6485 | ENSP00000343871 | TMTC4 | 5.301E-05 | -0.182 | - | - |
| 6486 | ENSP00000367359 | C6orf62 | 1.081E-04 | -0.182 | - | - |
| 6487 | ENSP00000471653 | AC018692.2 | 3.500E-06 | -0.182 | - | - |
| 6488 | ENSP00000362942 | PHACTR4 | 5.151E-05 | -0.182 | - | - |
| 6489 | ENSP00000392257 | PPIL6 | 2.508E-05 | -0.182 | - | - |
| 6490 | ENSP00000364219 | CAMK2N1 | 3.215E-05 | -0.182 | - | - |
| 6491 | ENSP00000381823 | SEC24A | 3.916E-05 | -0.182 | - | - |
| 6492 | ENSP00000369380 | SCML1 | 4.560E-05 | -0.182 | - | - |
| 6493 | ENSP00000360231 | NFIA | 4.189E-05 | -0.182 | - | - |
| 6494 | ENSP00000263512 | SLC10A3 | 5.269E-05 | -0.182 | - | - |
| 6495 | ENSP00000262302 | NUBP2 | 2.622E-05 | -0.182 | - | - |
| 6496 | ENSP00000349437 | IGF2R | 5.217E-05 | -0.182 | - | - |
| 6497 | ENSP00000359222 | TMEM56 | 3.837E-05 | -0.182 | - | - |
| 6498 | ENSP00000384179 | ZFPM2 | 2.795E-05 | -0.182 | - | - |
| 6499 | ENSP00000385045 | MTA3 | 3.817E-05 | -0.182 | - | - |
| 6500 | ENSP00000264852 | SIDT1 | 5.561E-05 | -0.182 | - | - |
| 6501 | ENSP00000295777 | SERPINI1 | 4.239E-05 | -0.182 | - | - |
| 6502 | ENSP00000341988 | SPATA31D1 | 1.284E-01 | -0.182 | - | - |
| 6503 | ENSP00000341333 | PRAMEF14 | 1.284E-01 | -0.182 | - | - |
| 6504 | ENSP00000359052 | FAM102B | 5.118E-05 | -0.182 | - | - |
| 6505 | ENSP00000355536 | MTR | 3.607E-05 | -0.183 | - | - |
| 6506 | ENSP00000363852 | SLC44A1 | 4.096E-05 | -0.183 | - | - |
| 6507 | ENSP00000312185 | ELMO1 | 4.570E-05 | -0.183 | - | - |
| 6508 | ENSP00000371200 | ARRDC5 | 1.673E-05 | -0.183 | - | - |
| 6509 | ENSP00000344818 | UBC | 2.589E-01 | -0.183 | - | - |
| 6510 | ENSP00000200557 | ADAM11 | 3.224E-05 | -0.183 | - | - |
| 6511 | ENSP00000371362 | NADKD1 | 5.707E-05 | -0.183 | - | - |
| 6512 | ENSP00000019317 | RALBP1 | 3.586E-05 | -0.183 | - | - |
| 6513 | ENSP00000257336 | BIVM | 1.205E-04 | -0.183 | - | - |
| 6514 | ENSP00000347444 | TDRD7 | 4.046E-05 | -0.183 | - | - |
| 6515 | ENSP00000250340 | CLEC11A | 2.854E-04 | -0.183 | - | - |
| 6516 | ENSP00000358203 | NBPF20 | 3.796E-05 | -0.183 | - | - |
| 6517 | ENSP00000263205 | MED15 | 3.921E-05 | -0.183 | - | - |
| 6518 | ENSP00000362574 | S100PBP | 5.004E-05 | -0.183 | - | - |
| 6519 | ENSP00000388864 | ZNF419 | 1.309E-05 | -0.183 | - | - |
| 6520 | ENSP00000358251 | FAM160B1 | 5.888E-05 | -0.183 | - | - |
| 6521 | ENSP00000262186 | KCNH2 | 2.826E-05 | -0.183 | - | - |
| 6522 | ENSP00000382091 | ALKBH5 | 6.340E-05 | -0.183 | - | - |
| 6523 | ENSP00000284031 | DDAH1 | 2.765E-05 | -0.183 | - | - |
| 6524 | ENSP00000325421 | PARL | 3.827E-05 | -0.183 | - | - |
| 6525 | ENSP00000290583 | CELF3 | 3.923E-05 | -0.183 | - | - |
| 6526 | ENSP00000376076 | SUMO1 | 5.249E-04 | -0.183 | - | - |
| 6527 | ENSP00000428824 | GPBAR1 | 2.889E-05 | -0.183 | - | - |
| 6528 | ENSP00000270066 | SMG9 | 5.523E-05 | -0.183 | - | - |
| 6529 | ENSP00000362638 | MARCKSL1 | 6.840E-05 | -0.183 | - | - |
| 6530 | ENSP00000290795 | GPBP1L1 | 9.247E-05 | -0.183 | - | - |
| 6531 | ENSP00000351141 | WTAP | 3.316E-05 | -0.183 | - | - |
| 6532 | ENSP00000239730 | - | 5.434E-05 | -0.183 | - | - |
| 6533 | ENSP00000353770 | RRM2 | 4.844E-05 | -0.183 | - | - |
| 6534 | ENSP00000293308 | KRT8 | 3.120E-05 | -0.183 | - | - |
| 6535 | ENSP00000371634 | IGF2BP2 | 3.892E-05 | -0.183 | - | - |
| 6536 | ENSP00000299964 | NNMT | 4.788E-05 | -0.183 | - | - |
| 6537 | ENSP00000373474 | METTL14 | 6.242E-05 | -0.183 | - | - |
| 6538 | ENSP00000369625 | FAM50B | 3.478E-05 | -0.183 | - | - |
| 6539 | ENSP00000349783 | RC3H2 | 4.108E-05 | -0.183 | - | - |
| 6540 | ENSP00000260433 | CYP19A1 | 4.354E-05 | -0.183 | - | - |
| 6541 | ENSP00000350009 | USP33 | 3.887E-05 | -0.183 | - | - |
| 6542 | ENSP00000265602 | AHI1 | 2.461E-05 | -0.183 | - | - |
| 6543 | ENSP00000361765 | TCEAL5 | 1.183E-05 | -0.183 | - | - |
| 6544 | ENSP00000364190 | PBX2 | 4.162E-05 | -0.183 | - | - |
| 6545 | ENSP00000290246 | ELMO2 | 4.295E-05 | -0.183 | - | - |
| 6546 | ENSP00000322652 | SNX16 | 5.525E-05 | -0.183 | - | - |
| 6547 | ENSP00000359212 | LBX1 | 2.926E-05 | -0.183 | - | - |
| 6548 | ENSP00000313933 | GLTSCR1L | 4.578E-05 | -0.183 | - | - |
| 6549 | ENSP00000364493 | HIATL1 | 5.143E-05 | -0.183 | - | - |
| 6550 | ENSP00000343619 | HOXA9 | 3.373E-05 | -0.183 | - | - |
| 6551 | ENSP00000367893 | SEPHS1 | 4.128E-05 | -0.183 | - | - |
| 6552 | ENSP00000321845 | SEC24C | 4.101E-05 | -0.183 | - | - |
| 6553 | ENSP00000360776 | B4GALT5 | 4.207E-05 | -0.183 | - | - |
| 6554 | ENSP00000415222 | MS4A4E | 9.327E-06 | -0.183 | - | - |
| 6555 | ENSP00000225603 | CBX1 | 4.278E-05 | -0.183 | - | - |
| 6556 | ENSP00000265997 | CPEB3 | 3.293E-05 | -0.183 | - | - |
| 6557 | ENSP00000299314 | GNPTAB | 2.620E-05 | -0.183 | - | - |
| 6558 | ENSP00000287202 | CELF6 | 4.053E-05 | -0.183 | - | - |
| 6559 | ENSP00000361383 | AIF1L | 5.451E-05 | -0.183 | - | - |
| 6560 | ENSP00000306561 | OGG1 | 3.367E-05 | -0.183 | - | - |
| 6561 | ENSP00000237455 | RNF103 | 4.772E-05 | -0.183 | - | - |
| 6562 | ENSP00000377942 | RSPRY1 | 6.535E-05 | -0.183 | - | - |
| 6563 | ENSP00000364028 | ECE1 | 4.983E-05 | -0.183 | - | - |
| 6564 | ENSP00000305244 | IRX1 | 2.681E-05 | -0.183 | - | - |
| 6565 | ENSP00000298130 | SPTSSA | 3.597E-05 | -0.183 | - | - |
| 6566 | ENSP00000305742 | LRTOMT | 2.830E-05 | -0.183 | - | - |
| 6567 | ENSP00000353564 | EXOC8 | 3.629E-05 | -0.183 | - | - |
| 6568 | ENSP00000362711 | TXLNA | 3.939E-05 | -0.183 | - | - |
| 6569 | ENSP00000297273 | CASD1 | 4.563E-05 | -0.183 | - | - |
| 6570 | ENSP00000265981 | RNF141 | 7.330E-05 | -0.183 | - | - |
| 6571 | ENSP00000262067 | TSPAN13 | 3.168E-05 | -0.183 | - | - |
| 6572 | ENSP00000372459 | CTIF | 4.931E-05 | -0.183 | - | - |
| 6573 | ENSP00000373360 | FAM131A | 2.965E-05 | -0.183 | - | - |
| 6574 | ENSP00000368503 | C4orf46 | 9.372E-05 | -0.183 | - | - |
| 6575 | ENSP00000299353 | C10ORF32 | 1.145E-04 | -0.183 | - | - |
| 6576 | ENSP00000472412 | AC187652.1 | 3.912E-07 | -0.183 | - | - |
| 6577 | ENSP00000356265 | NAV1 | 3.734E-05 | -0.183 | - | - |
| 6578 | ENSP00000300061 | SCNN1G | 2.928E-05 | -0.183 | - | - |
| 6579 | ENSP00000376800 | MTPN | 3.520E-05 | -0.183 | - | - |
| 6580 | ENSP00000359292 | FAM178A | 4.225E-05 | -0.183 | - | - |
| 6581 | ENSP00000256594 | GSTM3 | 6.781E-05 | -0.183 | - | - |
| 6582 | ENSP00000360320 | C1orf177 | 2.477E-05 | -0.183 | - | - |
| 6583 | ENSP00000006015 | HOXA11 | 3.844E-05 | -0.183 | - | - |
| 6584 | ENSP00000357429 | FABP7 | 2.073E-05 | -0.183 | - | - |
| 6585 | ENSP00000326519 | PAGR1 | 3.709E-05 | -0.183 | - | - |
| 6586 | ENSP00000365756 | KIAA2013 | 4.552E-05 | -0.183 | - | - |
| 6587 | ENSP00000264908 | ANXA3 | 4.427E-05 | -0.183 | - | - |
| 6588 | ENSP00000365522 | C9orf64 | 4.302E-05 | -0.183 | - | - |
| 6589 | ENSP00000356737 | GORAB | 5.665E-05 | -0.183 | - | - |
| 6590 | ENSP00000354995 | ABCG1 | 4.988E-05 | -0.183 | - | - |
| 6591 | ENSP00000374145 | CHRNG | 2.323E-05 | -0.183 | - | - |
| 6592 | ENSP00000296849 | NKD2 | 9.383E-05 | -0.183 | - | - |
| 6593 | ENSP00000361795 | DBNDD2 | 4.722E-06 | -0.183 | - | - |
| 6594 | ENSP00000361927 | HNRNPH2 | 2.459E-05 | -0.183 | - | - |
| 6595 | ENSP00000381104 | TM9SF4 | 3.454E-05 | -0.183 | - | - |
| 6596 | ENSP00000370345 | PAN3 | 3.965E-05 | -0.183 | - | - |
| 6597 | ENSP00000377995 | MT1X | 4.888E-05 | -0.183 | - | - |
| 6598 | ENSP00000343199 | TC2N | 4.356E-05 | -0.183 | - | - |
| 6599 | ENSP00000262662 | CDKN2C | 2.281E-05 | -0.183 | - | - |
| 6600 | ENSP00000336587 | CDR2L | 4.373E-05 | -0.183 | - | - |
| 6601 | ENSP00000311224 | ACOT1 | 3.959E-05 | -0.183 | - | - |
| 6602 | ENSP00000295767 | CHCHD4 | 5.854E-05 | -0.183 | - | - |
| 6603 | ENSP00000263974 | TAF12 | 2.442E-05 | -0.183 | - | - |
| 6604 | ENSP00000256644 | LAMTOR5 | 7.557E-05 | -0.183 | - | - |
| 6605 | ENSP00000355204 | FAM110B | 3.797E-05 | -0.184 | - | - |
| 6606 | ENSP00000301873 | LTBP3 | 7.179E-05 | -0.184 | - | - |
| 6607 | ENSP00000302108 | PANK1 | 3.304E-05 | -0.184 | - | - |
| 6608 | ENSP00000217428 | SPINT3 | 1.682E-05 | -0.184 | - | - |
| 6609 | ENSP00000361625 | DOLPP1 | 2.902E-05 | -0.184 | - | - |
| 6610 | ENSP00000360184 | TM9SF3 | 4.091E-05 | -0.184 | - | - |
| 6611 | ENSP00000215071 | PSMD8 | 2.636E-05 | -0.184 | - | - |
| 6612 | ENSP00000380872 | PGBD4 | 2.840E-05 | -0.184 | - | - |
| 6613 | ENSP00000360372 | CYP2C19 | 2.493E-05 | -0.184 | - | - |
| 6614 | ENSP00000380054 | EAF1 | 3.358E-05 | -0.184 | - | - |
| 6615 | ENSP00000359339 | MB21D1 | 3.814E-05 | -0.184 | - | - |
| 6616 | ENSP00000326411 | CWF19L1 | 7.052E-05 | -0.184 | - | - |
| 6617 | ENSP00000271636 | CGN | 4.449E-05 | -0.184 | - | - |
| 6618 | ENSP00000331720 | C16orf72 | 5.467E-05 | -0.184 | - | - |
| 6619 | ENSP00000428331 | SFTA3 | 4.746E-05 | -0.184 | - | - |
| 6620 | ENSP00000355731 | CDC42BPA | 2.946E-05 | -0.184 | - | - |
| 6621 | ENSP00000379654 | KIAA0430 | 3.370E-05 | -0.184 | - | - |
| 6622 | ENSP00000357810 | LINGO4 | 7.963E-06 | -0.184 | - | - |
| 6623 | ENSP00000369402 | IFT74 | 3.458E-05 | -0.184 | - | - |
| 6624 | ENSP00000231790 | MLH1 | 2.328E-05 | -0.184 | - | - |
| 6625 | ENSP00000355114 | DCAF12 | 3.052E-05 | -0.184 | - | - |
| 6626 | ENSP00000366384 | ZNF311 | 1.340E-05 | -0.184 | - | - |
| 6627 | ENSP00000246421 | SLC35E2 | 4.075E-05 | -0.184 | - | - |
| 6628 | ENSP00000330737 | NDUFA12 | 4.436E-05 | -0.184 | - | - |
| 6629 | ENSP00000315247 | FAM71C | 5.980E-06 | -0.184 | - | - |
| 6630 | ENSP00000315106 | GPR137C | 3.034E-05 | -0.184 | - | - |
| 6631 | ENSP00000316240 | DNAJC14 | 2.795E-05 | -0.184 | - | - |
| 6632 | ENSP00000364946 | MKX | 3.356E-05 | -0.184 | - | - |
| 6633 | ENSP00000364430 | SP5 | 1.932E-05 | -0.184 | - | - |
| 6634 | ENSP00000359356 | EVI5 | 3.627E-05 | -0.184 | - | - |
| 6635 | ENSP00000419378 | CLIC3 | 2.554E-05 | -0.184 | - | - |
| 6636 | ENSP00000228865 | CREBL2 | 3.037E-05 | -0.184 | - | - |
| 6637 | ENSP00000350757 | GMFB | 3.563E-05 | -0.184 | - | - |
| 6638 | ENSP00000315615 | AKAP5 | 5.273E-05 | -0.184 | - | - |
| 6639 | ENSP00000211314 | TMEM14A | 1.073E-04 | -0.184 | - | - |
| 6640 | ENSP00000434241 | KCNC3 | 2.036E-05 | -0.184 | - | - |
| 6641 | ENSP00000350261 | SAMD4A | 3.723E-05 | -0.184 | - | - |
| 6642 | ENSP00000243052 | PDE1B | 1.236E-05 | -0.184 | - | - |
| 6643 | ENSP00000262960 | DPP9 | 7.461E-05 | -0.184 | - | - |
| 6644 | ENSP00000277540 | WDR85 | 8.565E-05 | -0.184 | - | - |
| 6645 | ENSP00000394390 | KCTD15 | 4.342E-05 | -0.184 | - | - |
| 6646 | ENSP00000305288 | NLGN2 | 4.227E-05 | -0.184 | - | - |
| 6647 | ENSP00000303158 | CCDC8 | 1.778E-04 | -0.184 | - | - |
| 6648 | ENSP00000341992 | NANOS3 | 2.789E-05 | -0.184 | - | - |
| 6649 | ENSP00000296412 | ADH5 | 2.952E-05 | -0.184 | - | - |
| 6650 | ENSP00000357622 | CRTC2 | 3.763E-05 | -0.184 | - | - |
| 6651 | ENSP00000370460 | CWC27 | 2.468E-05 | -0.184 | - | - |
| 6652 | ENSP00000314879 | ALOXE3 | 5.740E-05 | -0.184 | - | - |
| 6653 | ENSP00000361162 | TOE1 | 3.998E-05 | -0.184 | - | - |
| 6654 | ENSP00000341882 | CNTN6 | 2.562E-05 | -0.184 | - | - |
| 6655 | ENSP00000384312 | TRIOBP | 2.333E-05 | -0.184 | - | - |
| 6656 | ENSP00000025008 | RB1CC1 | 2.960E-05 | -0.184 | - | - |
| 6657 | ENSP00000263776 | ACCS | 6.251E-06 | -0.184 | - | - |
| 6658 | ENSP00000359474 | BARHL2 | 4.442E-05 | -0.184 | - | - |
| 6659 | ENSP00000361245 | HECTD3 | 3.004E-05 | -0.184 | - | - |
| 6660 | ENSP00000352655 | SLC39A10 | 2.705E-05 | -0.184 | - | - |
| 6661 | ENSP00000290765 | GSTT2B | 2.483E-05 | -0.184 | - | - |
| 6662 | ENSP00000383909 | DMRTA2 | 2.733E-05 | -0.184 | - | - |
| 6663 | ENSP00000292140 | PHLDB3 | 1.818E-04 | -0.184 | - | - |
| 6664 | ENSP00000244930 | SCGB2A1 | 7.993E-05 | -0.184 | - | - |
| 6665 | ENSP00000356602 | VTA1 | 3.446E-05 | -0.184 | - | - |
| 6666 | ENSP00000357836 | SNX27 | 3.268E-05 | -0.184 | - | - |
| 6667 | ENSP00000238156 | CCDC92 | 1.192E-04 | -0.184 | - | - |
| 6668 | ENSP00000250831 | RBMY1J | 6.968E-06 | -0.184 | - | - |
| 6669 | ENSP00000359160 | TRMT13 | 6.760E-05 | -0.184 | - | - |
| 6670 | ENSP00000381428 | CGGBP1 | 5.806E-05 | -0.184 | - | - |
| 6671 | ENSP00000295640 | RNPEP | 2.992E-05 | -0.184 | - | - |
| 6672 | ENSP00000363824 | TMEM38B | 4.011E-05 | -0.184 | - | - |
| 6673 | ENSP00000368666 | PTCHD1 | 1.311E-05 | -0.184 | - | - |
| 6674 | ENSP00000206380 | TMEM101 | 3.613E-05 | -0.184 | - | - |
| 6675 | ENSP00000279281 | VPS51 | 4.748E-05 | -0.184 | - | - |
| 6676 | ENSP00000289361 | BTN3A1 | 1.286E-04 | -0.184 | - | - |
| 6677 | ENSP00000290418 | CCDC142 | 3.986E-05 | -0.184 | - | - |
| 6678 | ENSP00000303423 | FNTA | 5.039E-05 | -0.184 | - | - |
| 6679 | ENSP00000297156 | CAMLG | 9.749E-05 | -0.184 | - | - |
| 6680 | ENSP00000297239 | SYTL3 | 7.092E-05 | -0.184 | - | - |
| 6681 | ENSP00000355556 | GNG4 | 2.673E-05 | -0.184 | - | - |
| 6682 | ENSP00000316699 | TTC7A | 1.143E-04 | -0.184 | - | - |
| 6683 | ENSP00000394380 | ZNF625 | 1.032E-05 | -0.184 | - | - |
| 6684 | ENSP00000284476 | DISP1 | 6.840E-05 | -0.184 | - | - |
| 6685 | ENSP00000366466 | CTNNBIP1 | 3.004E-05 | -0.184 | - | - |
| 6686 | ENSP00000354633 | TAF11 | 2.125E-05 | -0.184 | - | - |
| 6687 | ENSP00000297632 | TMEM65 | 2.906E-05 | -0.184 | - | - |
| 6688 | ENSP00000333122 | NR4A3 | 2.874E-05 | -0.184 | - | - |
| 6689 | ENSP00000266497 | PIK3C2G | 1.325E-05 | -0.184 | - | - |
| 6690 | ENSP00000355141 | SEPN1 | 2.274E-05 | -0.184 | - | - |
| 6691 | ENSP00000360379 | FAM209A | 1.117E-06 | -0.184 | - | - |
| 6692 | ENSP00000345024 | GLI4 | 1.244E-05 | -0.184 | - | - |
| 6693 | ENSP00000354869 | GATSL1 | 1.240E-05 | -0.184 | - | - |
| 6694 | ENSP00000005178 | PDK4 | 2.640E-05 | -0.184 | - | - |
| 6695 | ENSP00000349076 | ATN1 | 3.059E-05 | -0.184 | - | - |
| 6696 | ENSP00000259608 | SIT1 | 2.439E-04 | -0.184 | - | - |
| 6697 | ENSP00000312767 | CTD-2373H9.6 | 2.136E-05 | -0.184 | - | - |
| 6698 | ENSP00000290705 | MT1A | 8.211E-05 | -0.184 | - | - |
| 6699 | ENSP00000327191 | CCT6B | 3.755E-05 | -0.184 | - | - |
| 6700 | ENSP00000357389 | SLC50A1 | 4.571E-05 | -0.184 | - | - |
| 6701 | ENSP00000302578 | FAM84B | 3.483E-05 | -0.184 | - | - |
| 6702 | ENSP00000275162 | CLVS2 | 3.470E-05 | -0.185 | - | - |
| 6703 | ENSP00000378887 | DHRS7B | 1.617E-05 | -0.185 | - | - |
| 6704 | ENSP00000425840 | RNF150 | 3.597E-05 | -0.185 | - | - |
| 6705 | ENSP00000364766 | XAGE1B | 1.976E-06 | -0.185 | - | - |
| 6706 | ENSP00000305974 | SLCO1A2 | 2.464E-05 | -0.185 | - | - |
| 6707 | ENSP00000290524 | RFX5 | 4.001E-05 | -0.185 | - | - |
| 6708 | ENSP00000263243 | ZC3H7B | 1.276E-04 | -0.185 | - | - |
| 6709 | ENSP00000292807 | AP2M1 | 2.457E-05 | -0.185 | - | - |
| 6710 | ENSP00000348050 | MXRA7 | 3.918E-05 | -0.185 | - | - |
| 6711 | ENSP00000337452 | NIPA1 | 1.066E-05 | -0.185 | - | - |
| 6712 | ENSP00000375904 | LGALS16 | 2.740E-06 | -0.185 | - | - |
| 6713 | ENSP00000354033 | PCGF2 | 2.677E-05 | -0.185 | - | - |
| 6714 | ENSP00000362637 | BLCAP | 3.509E-05 | -0.185 | - | - |
| 6715 | ENSP00000308928 | CYLD | 6.541E-05 | -0.185 | - | - |
| 6716 | ENSP00000279147 | ORAOV1 | 3.432E-05 | -0.185 | - | - |
| 6717 | ENSP00000221399 | TULP2 | 3.117E-05 | -0.185 | - | - |
| 6718 | ENSP00000085068 | ISOC2 | 7.945E-05 | -0.185 | - | - |
| 6719 | ENSP00000364140 | COL15A1 | 3.936E-05 | -0.185 | - | - |
| 6720 | ENSP00000216373 | SOS2 | 2.252E-05 | -0.185 | - | - |
| 6721 | ENSP00000351811 | EDC4 | 3.018E-05 | -0.185 | - | - |
| 6722 | ENSP00000171214 | RDH8 | 2.459E-05 | -0.185 | - | - |
| 6723 | ENSP00000217159 | SLCO4A1 | 5.366E-05 | -0.185 | - | - |
| 6724 | ENSP00000419712 | TMEM8C | 1.020E-06 | -0.185 | - | - |
| 6725 | ENSP00000261783 | ARG2 | 3.876E-05 | -0.185 | - | - |
| 6726 | ENSP00000362460 | GJB3 | 3.425E-05 | -0.185 | - | - |
| 6727 | ENSP00000376615 | NT5DC3 | 3.531E-05 | -0.185 | - | - |
| 6728 | ENSP00000346959 | SLC38A6 | 2.167E-05 | -0.185 | - | - |
| 6729 | ENSP00000254584 | ARFIP2 | 3.392E-05 | -0.185 | - | - |
| 6730 | ENSP00000363694 | EPB41L4B | 4.635E-05 | -0.185 | - | - |
| 6731 | ENSP00000362918 | YTHDF2 | 5.057E-05 | -0.185 | - | - |
| 6732 | ENSP00000314444 | WDR35 | 4.499E-05 | -0.185 | - | - |
| 6733 | ENSP00000374357 | ARNTL | 5.242E-05 | -0.185 | - | - |
| 6734 | ENSP00000357683 | CHTOP | 4.836E-05 | -0.185 | - | - |
| 6735 | ENSP00000349959 | RICTOR | 5.915E-05 | -0.185 | - | - |
| 6736 | ENSP00000354850 | MGEA5 | 3.914E-05 | -0.185 | - | - |
| 6737 | ENSP00000380254 | AC007390.5 | 2.486E-06 | -0.185 | - | - |
| 6738 | ENSP00000223029 | AIMP2 | 4.724E-05 | -0.185 | - | - |
| 6739 | ENSP00000358363 | PDE4DIP | 2.876E-05 | -0.185 | - | - |
| 6740 | ENSP00000170564 | GPATCH1 | 8.637E-05 | -0.185 | - | - |
| 6741 | ENSP00000377470 | CNP | 6.542E-06 | -0.185 | - | - |
| 6742 | ENSP00000298355 | TRIM9 | 5.568E-05 | -0.185 | - | - |
| 6743 | ENSP00000368341 | RBM24 | 3.226E-05 | -0.185 | - | - |
| 6744 | ENSP00000356468 | IVNS1ABP | 2.238E-05 | -0.185 | - | - |
| 6745 | ENSP00000216452 | PIGH | 9.094E-05 | -0.185 | - | - |
| 6746 | ENSP00000361522 | TJAP1 | 7.168E-05 | -0.185 | - | - |
| 6747 | ENSP00000379051 | PURB | 3.350E-05 | -0.185 | - | - |
| 6748 | ENSP00000345175 | PODNL1 | 9.097E-06 | -0.185 | - | - |
| 6749 | ENSP00000265052 | MGLL | 2.295E-05 | -0.185 | - | - |
| 6750 | ENSP00000222803 | FKBP14 | 1.561E-05 | -0.185 | - | - |
| 6751 | ENSP00000264080 | GPR108 | 8.033E-05 | -0.185 | - | - |
| 6752 | ENSP00000258390 | DOCK10 | 6.495E-05 | -0.185 | - | - |
| 6753 | ENSP00000325822 | CYP11B2 | 2.163E-05 | -0.185 | - | - |
| 6754 | ENSP00000299427 | TPP1 | 5.378E-05 | -0.185 | - | - |
| 6755 | ENSP00000324693 | GRHL1 | 5.234E-05 | -0.185 | - | - |
| 6756 | ENSP00000292853 | FBXO27 | 2.290E-05 | -0.185 | - | - |
| 6757 | ENSP00000376652 | EVL | 1.319E-05 | -0.185 | - | - |
| 6758 | ENSP00000333769 | BSG | 4.787E-05 | -0.185 | - | - |
| 6759 | ENSP00000355623 | C1orf198 | 5.356E-05 | -0.185 | - | - |
| 6760 | ENSP00000263257 | NOVA2 | 2.650E-05 | -0.185 | - | - |
| 6761 | ENSP00000262506 | CSNK2A2 | 1.959E-05 | -0.185 | - | - |
| 6762 | ENSP00000295049 | RFTN2 | 3.501E-05 | -0.185 | - | - |
| 6763 | ENSP00000365147 | EFHD2 | 2.286E-05 | -0.185 | - | - |
| 6764 | ENSP00000271889 | CREB3L4 | 5.251E-05 | -0.185 | - | - |
| 6765 | ENSP00000297689 | NFIL3 | 6.801E-05 | -0.185 | - | - |
| 6766 | ENSP00000325919 | PSMG2 | 7.033E-05 | -0.185 | - | - |
| 6767 | ENSP00000375975 | TMCO1 | 4.155E-05 | -0.185 | - | - |
| 6768 | ENSP00000349796 | ZNF23 | 1.795E-05 | -0.185 | - | - |
| 6769 | ENSP00000215904 | PDXP | 2.022E-05 | -0.185 | - | - |
| 6770 | ENSP00000337632 | SARNP | 5.952E-05 | -0.185 | - | - |
| 6771 | ENSP00000307280 | MYL1 | 2.441E-05 | -0.185 | - | - |
| 6772 | ENSP00000376162 | HOMER3 | 2.999E-05 | -0.185 | - | - |
| 6773 | ENSP00000056233 | NFE2L3 | 5.110E-05 | -0.185 | - | - |
| 6774 | ENSP00000265018 | FAM184B | 1.566E-04 | -0.185 | - | - |
| 6775 | ENSP00000282570 | GMCL1 | 4.109E-05 | -0.185 | - | - |
| 6776 | ENSP00000360222 | TM2D1 | 2.961E-05 | -0.185 | - | - |
| 6777 | ENSP00000341657 | THYN1 | 8.773E-05 | -0.185 | - | - |
| 6778 | ENSP00000346139 | TBC1D20 | 2.480E-05 | -0.185 | - | - |
| 6779 | ENSP00000347041 | FMOD | 2.400E-05 | -0.185 | - | - |
| 6780 | ENSP00000216085 | RHBDD3 | 5.909E-05 | -0.185 | - | - |
| 6781 | ENSP00000316861 | GPM6B | 2.893E-05 | -0.185 | - | - |
| 6782 | ENSP00000368952 | STOML3 | 1.500E-05 | -0.185 | - | - |
| 6783 | ENSP00000299798 | SLC9A5 | 1.894E-05 | -0.185 | - | - |
| 6784 | ENSP00000268231 | 12-Sep | 4.987E-05 | -0.185 | - | - |
| 6785 | ENSP00000234420 | MSH6 | 2.216E-05 | -0.185 | - | - |
| 6786 | ENSP00000230859 | PAPD7 | 1.634E-05 | -0.185 | - | - |
| 6787 | ENSP00000300571 | GPRC5B | 6.021E-05 | -0.185 | - | - |
| 6788 | ENSP00000304604 | MAGI3 | 3.146E-05 | -0.185 | - | - |
| 6789 | ENSP00000271657 | PI4KB | 8.245E-06 | -0.185 | - | - |
| 6790 | ENSP00000206262 | RGS17 | 4.581E-05 | -0.185 | - | - |
| 6791 | ENSP00000279206 | NUDT22 | 3.843E-04 | -0.185 | - | - |
| 6792 | ENSP00000229307 | NANOG | 4.777E-05 | -0.186 | - | - |
| 6793 | ENSP00000281129 | CEP128 | 1.723E-04 | -0.186 | - | - |
| 6794 | ENSP00000316329 | SCD5 | 2.890E-05 | -0.186 | - | - |
| 6795 | ENSP00000451560 | TUBB3 | 5.798E-05 | -0.186 | - | - |
| 6796 | ENSP00000351524 | HELZ | 2.432E-05 | -0.186 | - | - |
| 6797 | ENSP00000307859 | SLC22A18 | 3.106E-05 | -0.186 | - | - |
| 6798 | ENSP00000272322 | VPS54 | 6.060E-06 | -0.186 | - | - |
| 6799 | ENSP00000359074 | L1CAM | 2.006E-05 | -0.186 | - | - |
| 6800 | ENSP00000362010 | ANXA7 | 4.629E-05 | -0.186 | - | - |
| 6801 | ENSP00000261879 | APH1B | 5.374E-05 | -0.186 | - | - |
| 6802 | ENSP00000382595 | PAICS | 3.158E-05 | -0.186 | - | - |
| 6803 | ENSP00000400803 | LRRC69 | 7.461E-06 | -0.186 | - | - |
| 6804 | ENSP00000364519 | TFDP1 | 3.519E-05 | -0.186 | - | - |
| 6805 | ENSP00000464743 | FAM215A | 4.619E-05 | -0.186 | - | - |
| 6806 | ENSP00000317128 | PLXND1 | 4.465E-05 | -0.186 | - | - |
| 6807 | ENSP00000342114 | ICAM4 | 3.914E-05 | -0.186 | - | - |
| 6808 | ENSP00000295951 | SLMAP | 2.846E-05 | -0.186 | - | - |
| 6809 | ENSP00000078445 | CREB3L3 | 2.219E-05 | -0.186 | - | - |
| 6810 | ENSP00000309548 | PDP2 | 2.172E-05 | -0.186 | - | - |
| 6811 | ENSP00000252669 | ACSBG2 | 3.767E-05 | -0.186 | - | - |
| 6812 | ENSP00000217964 | TBL1X | 2.715E-05 | -0.186 | - | - |
| 6813 | ENSP00000373648 | KCNQ3 | 2.118E-05 | -0.186 | - | - |
| 6814 | ENSP00000292823 | PCYT1A | 3.038E-05 | -0.186 | - | - |
| 6815 | ENSP00000391121 | RDH13 | 5.018E-06 | -0.186 | - | - |
| 6816 | ENSP00000357982 | NR2E1 | 4.092E-05 | -0.186 | - | - |
| 6817 | ENSP00000376139 | WDR45B | 3.499E-05 | -0.186 | - | - |
| 6818 | ENSP00000313171 | ATAD5 | 5.073E-05 | -0.186 | - | - |
| 6819 | ENSP00000225519 | SHPK | 2.359E-05 | -0.186 | - | - |
| 6820 | ENSP00000265967 | HPS5 | 7.053E-05 | -0.186 | - | - |
| 6821 | ENSP00000310935 | FKBP2 | 2.534E-05 | -0.186 | - | - |
| 6822 | ENSP00000333938 | NEXN | 4.560E-05 | -0.186 | - | - |
| 6823 | ENSP00000399441 | LRRC70 | 8.970E-06 | -0.186 | - | - |
| 6824 | ENSP00000369339 | C9orf72 | 3.960E-05 | -0.186 | - | - |
| 6825 | ENSP00000357624 | MARCKS | 4.913E-05 | -0.186 | - | - |
| 6826 | ENSP00000440207 | TBC1D30 | 2.595E-05 | -0.186 | - | - |
| 6827 | ENSP00000254051 | TNS4 | 1.111E-04 | -0.186 | - | - |
| 6828 | ENSP00000297347 | MED30 | 5.334E-05 | -0.186 | - | - |
| 6829 | ENSP00000334538 | SREK1 | 2.980E-05 | -0.186 | - | - |
| 6830 | ENSP00000358081 | BAG3 | 8.849E-05 | -0.186 | - | - |
| 6831 | ENSP00000261024 | SLC40A1 | 7.259E-05 | -0.186 | - | - |
| 6832 | ENSP00000317257 | CPNE1 | 5.157E-05 | -0.186 | - | - |
| 6833 | ENSP00000367471 | BTBD3 | 2.131E-05 | -0.186 | - | - |
| 6834 | ENSP00000357069 | PIGM | 2.116E-05 | -0.186 | - | - |
| 6835 | ENSP00000270233 | BCAM | 5.599E-05 | -0.186 | - | - |
| 6836 | ENSP00000325506 | CLVS1 | 3.410E-05 | -0.186 | - | - |
| 6837 | ENSP00000359518 | MRGBP | 1.662E-05 | -0.186 | - | - |
| 6838 | ENSP00000251871 | MED17 | 3.988E-05 | -0.186 | - | - |
| 6839 | ENSP00000238561 | ADCK1 | 2.932E-05 | -0.186 | - | - |
| 6840 | ENSP00000274192 | SRD5A1 | 2.263E-05 | -0.186 | - | - |
| 6841 | ENSP00000287156 | UBE2L6 | 3.136E-05 | -0.186 | - | - |
| 6842 | ENSP00000316527 | ZNF609 | 4.771E-04 | -0.186 | - | - |
| 6843 | ENSP00000312224 | DTD2 | 1.898E-05 | -0.186 | - | - |
| 6844 | ENSP00000307701 | TSPAN5 | 7.017E-05 | -0.186 | - | - |
| 6845 | ENSP00000281938 | HSPB8 | 4.905E-05 | -0.186 | - | - |
| 6846 | ENSP00000351333 | ARHGAP19 | 3.111E-05 | -0.186 | - | - |
| 6847 | ENSP00000321260 | COX8A | 4.277E-05 | -0.186 | - | - |
| 6848 | ENSP00000268383 | CDR2 | 4.586E-05 | -0.186 | - | - |
| 6849 | ENSP00000264094 | LOXL3 | 3.566E-05 | -0.186 | - | - |
| 6850 | ENSP00000369871 | HAUS6 | 2.525E-05 | -0.186 | - | - |
| 6851 | ENSP00000298687 | NDRG2 | 2.507E-05 | -0.186 | - | - |
| 6852 | ENSP00000313350 | RNASEH1 | 1.682E-05 | -0.186 | - | - |
| 6853 | ENSP00000383270 | SMIM2 | 6.034E-06 | -0.186 | - | - |
| 6854 | ENSP00000261534 | POMT2 | 4.271E-05 | -0.186 | - | - |
| 6855 | ENSP00000302918 | C11orf40 | 1.234E-04 | -0.186 | - | - |
| 6856 | ENSP00000365159 | ISCA1 | 1.532E-05 | -0.186 | - | - |
| 6857 | ENSP00000355621 | TTC13 | 1.567E-05 | -0.186 | - | - |
| 6858 | ENSP00000376306 | CIT | 2.137E-05 | -0.186 | - | - |
| 6859 | ENSP00000369853 | C2CD2 | 2.577E-05 | -0.186 | - | - |
| 6860 | ENSP00000350278 | SLC30A7 | 2.399E-05 | -0.186 | - | - |
| 6861 | ENSP00000222567 | TWISTNB | 1.397E-05 | -0.186 | - | - |
| 6862 | ENSP00000164133 | PPP2R5B | 2.382E-05 | -0.186 | - | - |
| 6863 | ENSP00000356789 | ATP1B1 | 2.770E-05 | -0.186 | - | - |
| 6864 | ENSP00000384823 | FBXO11 | 3.010E-05 | -0.186 | - | - |
| 6865 | ENSP00000250263 | ERI1 | 4.896E-05 | -0.186 | - | - |
| 6866 | ENSP00000264938 | SLC9A3 | 2.191E-05 | -0.186 | - | - |
| 6867 | ENSP00000359685 | PTP4A1 | 3.322E-05 | -0.186 | - | - |
| 6868 | ENSP00000360569 | SCP2 | 2.551E-05 | -0.186 | - | - |
| 6869 | ENSP00000366225 | NAPB | 1.949E-05 | -0.186 | - | - |
| 6870 | ENSP00000325546 | PLAG1 | 3.938E-05 | -0.186 | - | - |
| 6871 | ENSP00000391596 | AMDHD2 | 2.296E-05 | -0.186 | - | - |
| 6872 | ENSP00000264157 | CCNT2 | 1.874E-05 | -0.186 | - | - |
| 6873 | ENSP00000345985 | LRFN2 | 1.650E-05 | -0.186 | - | - |
| 6874 | ENSP00000261658 | BFAR | 7.123E-05 | -0.186 | - | - |
| 6875 | ENSP00000297770 | CPA6 | 5.286E-05 | -0.186 | - | - |
| 6876 | ENSP00000359258 | GCLM | 3.711E-05 | -0.186 | - | - |
| 6877 | ENSP00000337946 | PACRG | 5.487E-05 | -0.186 | - | - |
| 6878 | ENSP00000296015 | TTC14 | 8.905E-05 | -0.186 | - | - |
| 6879 | ENSP00000381588 | TMPRSS2 | 3.877E-05 | -0.186 | - | - |
| 6880 | ENSP00000368314 | NRCAM | 2.719E-05 | -0.186 | - | - |
| 6881 | ENSP00000251020 | SALL1 | 3.162E-05 | -0.186 | - | - |
| 6882 | ENSP00000361397 | NUTMG | 2.931E-06 | -0.186 | - | - |
| 6883 | ENSP00000262109 | ERICH1 | 3.110E-02 | -0.186 | - | - |
| 6884 | ENSP00000354361 | RFWD3 | 3.756E-05 | -0.186 | - | - |
| 6885 | ENSP00000264690 | KLKB1 | 5.822E-05 | -0.186 | - | - |
| 6886 | ENSP00000289528 | ZFAND2B | 4.767E-05 | -0.186 | - | - |
| 6887 | ENSP00000352918 | ARPC5 | 2.249E-05 | -0.186 | - | - |
| 6888 | ENSP00000339726 | OR14I1 | 8.628E-06 | -0.186 | - | - |
| 6889 | ENSP00000366702 | ERRFI1 | 2.725E-05 | -0.186 | - | - |
| 6890 | ENSP00000256759 | FST | 6.126E-05 | -0.187 | - | - |
| 6891 | ENSP00000231021 | CDH9 | 3.808E-05 | -0.187 | - | - |
| 6892 | ENSP00000371230 | AK3 | 8.992E-05 | -0.187 | - | - |
| 6893 | ENSP00000358794 | SLC16A4 | 3.567E-05 | -0.187 | - | - |
| 6894 | ENSP00000260746 | ARL3 | 6.491E-05 | -0.187 | - | - |
| 6895 | ENSP00000309689 | LRCH4 | 8.895E-06 | -0.187 | - | - |
| 6896 | ENSP00000296358 | OTOP1 | 2.825E-05 | -0.187 | - | - |
| 6897 | ENSP00000343891 | NLRP13 | 3.132E-04 | -0.187 | - | - |
| 6898 | ENSP00000306106 | JAGN1 | 1.075E-04 | -0.187 | - | - |
| 6899 | ENSP00000316482 | KCTD14 | 2.992E-04 | -0.187 | - | - |
| 6900 | ENSP00000411734 | SRXN1 | 3.530E-06 | -0.187 | - | - |
| 6901 | ENSP00000306129 | DRD5 | 2.350E-05 | -0.187 | - | - |
| 6902 | ENSP00000267522 | WDR89 | 5.425E-05 | -0.187 | - | - |
| 6903 | ENSP00000340402 | SLC15A5 | 6.484E-05 | -0.187 | - | - |
| 6904 | ENSP00000263635 | TANC1 | 2.243E-05 | -0.187 | - | - |
| 6905 | ENSP00000340510 | PPL | 8.901E-05 | -0.187 | - | - |
| 6906 | ENSP00000362207 | CHST3 | 2.252E-05 | -0.187 | - | - |
| 6907 | ENSP00000301244 | SPINT2 | 5.300E-05 | -0.187 | - | - |
| 6908 | ENSP00000258201 | FHOD1 | 5.827E-05 | -0.187 | - | - |
| 6909 | ENSP00000055077 | RFC2 | 1.261E-05 | -0.187 | - | - |
| 6910 | ENSP00000450114 | C14orf79 | 1.431E-06 | -0.187 | - | - |
| 6911 | ENSP00000323076 | NDUFAF3 | 4.111E-05 | -0.187 | - | - |
| 6912 | ENSP00000262426 | FOXF1 | 2.159E-05 | -0.187 | - | - |
| 6913 | ENSP00000364883 | AUH | 1.409E-05 | -0.187 | - | - |
| 6914 | ENSP00000356859 | MGST3 | 3.806E-05 | -0.187 | - | - |
| 6915 | ENSP00000326381 | EIF4A2 | 5.213E-05 | -0.187 | - | - |
| 6916 | ENSP00000352497 | SLC25A44 | 5.012E-05 | -0.187 | - | - |
| 6917 | ENSP00000054950 | RCN1 | 2.344E-05 | -0.187 | - | - |
| 6918 | ENSP00000219313 | PSMD7 | 2.803E-05 | -0.187 | - | - |
| 6919 | ENSP00000348848 | WDR45 | 6.938E-06 | -0.187 | - | - |
| 6920 | ENSP00000344779 | RHBDD1 | 8.184E-05 | -0.187 | - | - |
| 6921 | ENSP00000270310 | FXYD7 | 9.850E-05 | -0.187 | - | - |
| 6922 | ENSP00000344479 | NR4A2 | 4.922E-05 | -0.187 | - | - |
| 6923 | ENSP00000249883 | AMOTL2 | 5.905E-05 | -0.187 | - | - |
| 6924 | ENSP00000332274 | PTP4A3 | 2.889E-05 | -0.187 | - | - |
| 6925 | ENSP00000330875 | FANCF | 5.426E-05 | -0.187 | - | - |
| 6926 | ENSP00000382511 | ZNF195 | 1.005E-05 | -0.187 | - | - |
| 6927 | ENSP00000354497 | FAN1 | 3.164E-05 | -0.187 | - | - |
| 6928 | ENSP00000336127 | TTC7B | 4.433E-05 | -0.187 | - | - |
| 6929 | ENSP00000322191 | DEFB104B | 1.255E-06 | -0.187 | - | - |
| 6930 | ENSP00000216044 | GTPBP1 | 4.107E-05 | -0.187 | - | - |
| 6931 | ENSP00000363414 | PAQR7 | 2.928E-05 | -0.187 | - | - |
| 6932 | ENSP00000347755 | ZNF140 | 1.376E-05 | -0.187 | - | - |
| 6933 | ENSP00000410041 | SKIDA1 | 1.298E-05 | -0.187 | - | - |
| 6934 | ENSP00000374529 | CCNK | 1.957E-05 | -0.187 | - | - |
| 6935 | ENSP00000409126 | ANO6 | 2.102E-05 | -0.187 | - | - |
| 6936 | ENSP00000272139 | C1orf35 | 1.408E-04 | -0.187 | - | - |
| 6937 | ENSP00000061240 | TLL1 | 4.688E-05 | -0.187 | - | - |
| 6938 | ENSP00000300035 | KIAA0101 | 1.199E-04 | -0.187 | - | - |
| 6939 | ENSP00000410545 | ZNF814 | 9.620E-06 | -0.187 | - | - |
| 6940 | ENSP00000349313 | NHEJ1 | 2.898E-05 | -0.187 | - | - |
| 6941 | ENSP00000424757 | NEK1 | 4.746E-05 | -0.187 | - | - |
| 6942 | ENSP00000331748 | RAB12 | 3.068E-05 | -0.187 | - | - |
| 6943 | ENSP00000219409 | ARHGDIG | 2.856E-05 | -0.187 | - | - |
| 6944 | ENSP00000309501 | KIAA1239 | 8.243E-06 | -0.187 | - | - |
| 6945 | ENSP00000360626 | KCNG1 | 2.511E-05 | -0.187 | - | - |
| 6946 | ENSP00000332861 | ZNF530 | 4.264E-05 | -0.187 | - | - |
| 6947 | ENSP00000377390 | PPP6R3 | 2.210E-05 | -0.187 | - | - |
| 6948 | ENSP00000258418 | CAB39 | 3.023E-05 | -0.187 | - | - |
| 6949 | ENSP00000355343 | UBAP2L | 3.416E-05 | -0.187 | - | - |
| 6950 | ENSP00000358862 | PCGF6 | 1.774E-05 | -0.187 | - | - |
| 6951 | ENSP00000380351 | ZNF682 | 1.412E-05 | -0.187 | - | - |
| 6952 | ENSP00000369989 | KATNAL1 | 2.070E-05 | -0.187 | - | - |
| 6953 | ENSP00000391404 | C4orf51 | 3.011E-06 | -0.187 | - | - |
| 6954 | ENSP00000262879 | RALGAPB | 5.176E-05 | -0.187 | - | - |
| 6955 | ENSP00000216294 | SNAPC1 | 6.638E-05 | -0.187 | - | - |
| 6956 | ENSP00000234256 | SLC1A4 | 2.747E-05 | -0.187 | - | - |
| 6957 | ENSP00000231198 | THG1L | 9.018E-05 | -0.187 | - | - |
| 6958 | ENSP00000262982 | CSE1L | 3.303E-05 | -0.187 | - | - |
| 6959 | ENSP00000358283 | UFL1 | 3.152E-05 | -0.187 | - | - |
| 6960 | ENSP00000266022 | RBM6 | 2.095E-04 | -0.187 | - | - |
| 6961 | ENSP00000273130 | DYNC1LI1 | 4.706E-05 | -0.187 | - | - |
| 6962 | ENSP00000336894 | NAB1 | 5.496E-05 | -0.187 | - | - |
| 6963 | ENSP00000181839 | CDK13 | 2.033E-05 | -0.187 | - | - |
| 6964 | ENSP00000260570 | IFT172 | 2.565E-05 | -0.187 | - | - |
| 6965 | ENSP00000303427 | PDS5A | 3.457E-05 | -0.187 | - | - |
| 6966 | ENSP00000369055 | B4GALT1 | 2.568E-05 | -0.187 | - | - |
| 6967 | ENSP00000266458 | GABARAPL1 | 2.858E-05 | -0.187 | - | - |
| 6968 | ENSP00000295924 | TIPARP | 8.505E-05 | -0.187 | - | - |
| 6969 | ENSP00000207549 | UNC13D | 4.247E-05 | -0.187 | - | - |
| 6970 | ENSP00000276297 | DLC1 | 3.156E-05 | -0.187 | - | - |
| 6971 | ENSP00000363040 | MEGF9 | 3.083E-05 | -0.187 | - | - |
| 6972 | ENSP00000365343 | PRAMEF3 | 1.233E-05 | -0.187 | - | - |
| 6973 | ENSP00000417963 | PPP2R5D | 4.138E-05 | -0.188 | - | - |
| 6974 | ENSP00000261900 | CCNT1 | 2.473E-05 | -0.188 | - | - |
| 6975 | ENSP00000348510 | GTF3C1 | 5.627E-05 | -0.188 | - | - |
| 6976 | ENSP00000334941 | PTPRU | 3.135E-05 | -0.188 | - | - |
| 6977 | ENSP00000294618 | DOCK6 | 4.240E-05 | -0.188 | - | - |
| 6978 | ENSP00000328768 | PRR23B | 1.284E-01 | -0.188 | - | - |
| 6979 | ENSP00000306070 | NUDT6 | 5.879E-05 | -0.188 | - | - |
| 6980 | ENSP00000170168 | REXO1 | 9.194E-06 | -0.188 | - | - |
| 6981 | ENSP00000364986 | AGMAT | 1.676E-05 | -0.188 | - | - |
| 6982 | ENSP00000303476 | TLN2 | 3.170E-05 | -0.188 | - | - |
| 6983 | ENSP00000385083 | KDSR | 2.384E-05 | -0.188 | - | - |
| 6984 | ENSP00000354837 | ARHGEF2 | 3.421E-05 | -0.188 | - | - |
| 6985 | ENSP00000379042 | ERO1L | 2.407E-05 | -0.188 | - | - |
| 6986 | ENSP00000281437 | BARX2 | 8.350E-05 | -0.188 | - | - |
| 6987 | ENSP00000268314 | ARMC5 | 1.816E-04 | -0.188 | - | - |
| 6988 | ENSP00000263598 | LCN1 | 4.960E-05 | -0.188 | - | - |
| 6989 | ENSP00000299424 | TAF10 | 1.218E-05 | -0.188 | - | - |
| 6990 | ENSP00000362590 | TBC1D22B | 2.024E-05 | -0.188 | - | - |
| 6991 | ENSP00000379836 | SLC30A5 | 2.352E-05 | -0.188 | - | - |
| 6992 | ENSP00000323687 | UBE2O | 3.785E-05 | -0.188 | - | - |
| 6993 | ENSP00000278886 | NINL | 3.007E-05 | -0.188 | - | - |
| 6994 | ENSP00000178638 | CA12 | 4.394E-05 | -0.188 | - | - |
| 6995 | ENSP00000380310 | ZNF676 | 9.882E-06 | -0.188 | - | - |
| 6996 | ENSP00000290130 | MIS18A | 3.386E-05 | -0.188 | - | - |
| 6997 | ENSP00000364685 | MFAP2 | 2.437E-05 | -0.188 | - | - |
| 6998 | ENSP00000295641 | STK11IP | 1.152E-04 | -0.188 | - | - |
| 6999 | ENSP00000327650 | VPS33B | 3.550E-05 | -0.188 | - | - |
| 7000 | ENSP00000376150 | SLC16A3 | 6.024E-06 | -0.188 | - | - |
| 7001 | ENSP00000367408 | CASK | 5.787E-05 | -0.188 | - | - |
| 7002 | ENSP00000347712 | C2CD4A | 1.082E-05 | -0.188 | - | - |
| 7003 | ENSP00000316990 | TRAPPC5 | 3.500E-05 | -0.188 | - | - |
| 7004 | ENSP00000359890 | MBNL3 | 2.358E-05 | -0.188 | - | - |
| 7005 | ENSP00000244096 | MAGEA10 | 6.855E-05 | -0.188 | - | - |
| 7006 | ENSP00000366410 | NMNAT1 | 3.826E-05 | -0.188 | - | - |
| 7007 | ENSP00000343706 | MAGEH1 | 4.447E-05 | -0.188 | - | - |
| 7008 | ENSP00000323036 | ACPP | 4.227E-05 | -0.188 | - | - |
| 7009 | ENSP00000361229 | TSPAN14 | 3.650E-05 | -0.188 | - | - |
| 7010 | ENSP00000276416 | BIN3 | 1.426E-04 | -0.188 | - | - |
| 7011 | ENSP00000385479 | HIST2H3A | 2.488E-05 | -0.188 | - | - |
| 7012 | ENSP00000351250 | TRIM33 | 5.034E-05 | -0.188 | - | - |
| 7013 | ENSP00000418735 | ADAMTS9 | 3.155E-05 | -0.188 | - | - |
| 7014 | ENSP00000306100 | PAM | 4.470E-05 | -0.188 | - | - |
| 7015 | ENSP00000276185 | FRMPD3 | 3.124E-05 | -0.188 | - | - |
| 7016 | ENSP00000241125 | GJA3 | 3.659E-05 | -0.188 | - | - |
| 7017 | ENSP00000419628 | NUDT5 | 3.032E-05 | -0.188 | - | - |
| 7018 | ENSP00000295902 | PRICKLE2 | 2.865E-05 | -0.188 | - | - |
| 7019 | ENSP00000255324 | RNF17 | 4.276E-05 | -0.188 | - | - |
| 7020 | ENSP00000322249 | ORAI3 | 4.743E-05 | -0.188 | - | - |
| 7021 | ENSP00000251757 | PHC1 | 4.196E-05 | -0.188 | - | - |
| 7022 | ENSP00000234170 | CEBPZ | 2.319E-05 | -0.188 | - | - |
| 7023 | ENSP00000300917 | SMG8 | 5.652E-05 | -0.188 | - | - |
| 7024 | ENSP00000355217 | SNX6 | 2.605E-05 | -0.188 | - | - |
| 7025 | ENSP00000252934 | ATXN10 | 6.638E-05 | -0.188 | - | - |
| 7026 | ENSP00000351684 | CAMSAP2 | 2.142E-05 | -0.188 | - | - |
| 7027 | ENSP00000271638 | S100A11 | 5.559E-05 | -0.188 | - | - |
| 7028 | ENSP00000306522 | CAMTA1 | 4.229E-05 | -0.188 | - | - |
| 7029 | ENSP00000306138 | GRM5 | 2.782E-05 | -0.188 | - | - |
| 7030 | ENSP00000329867 | PPME1 | 6.276E-05 | -0.188 | - | - |
| 7031 | ENSP00000278612 | NPAT | 2.778E-05 | -0.188 | - | - |
| 7032 | ENSP00000262483 | PITPNM3 | 1.219E-05 | -0.188 | - | - |
| 7033 | ENSP00000364448 | UPF3A | 1.587E-05 | -0.188 | - | - |
| 7034 | ENSP00000367299 | VPS36 | 1.850E-05 | -0.188 | - | - |
| 7035 | ENSP00000328426 | FAM208B | 1.775E-04 | -0.188 | - | - |
| 7036 | ENSP00000326514 | EXOC1 | 3.436E-05 | -0.188 | - | - |
| 7037 | ENSP00000344307 | FOCAD | 8.015E-05 | -0.188 | - | - |
| 7038 | ENSP00000329213 | SIVA1 | 5.656E-05 | -0.188 | - | - |
| 7039 | ENSP00000319250 | GPR62 | 2.222E-05 | -0.188 | - | - |
| 7040 | ENSP00000351482 | ZNF707 | 1.249E-05 | -0.188 | - | - |
| 7041 | ENSP00000257192 | DSG1 | 4.267E-05 | -0.188 | - | - |
| 7042 | ENSP00000301659 | GSDMA | 2.021E-04 | -0.188 | - | - |
| 7043 | ENSP00000321370 | ZBED2 | 8.630E-05 | -0.188 | - | - |
| 7044 | ENSP00000314396 | PRMT10 | 1.125E-04 | -0.188 | - | - |
| 7045 | ENSP00000379144 | TNRC6A | 2.895E-05 | -0.188 | - | - |
| 7046 | ENSP00000216099 | APOBEC3D | 8.704E-05 | -0.188 | - | - |
| 7047 | ENSP00000260645 | ABCG5 | 3.570E-05 | -0.188 | - | - |
| 7048 | ENSP00000279022 | MYL9 | 2.204E-05 | -0.188 | - | - |
| 7049 | ENSP00000357880 | AMD1 | 3.574E-05 | -0.188 | - | - |
| 7050 | ENSP00000264935 | CEP72 | 5.049E-05 | -0.188 | - | - |
| 7051 | ENSP00000373853 | LRRC4B | 8.624E-06 | -0.188 | - | - |
| 7052 | ENSP00000348886 | STOML2 | 1.421E-05 | -0.188 | - | - |
| 7053 | ENSP00000383168 | PCBP3 | 1.846E-05 | -0.188 | - | - |
| 7054 | ENSP00000384490 | CBX6 | 1.779E-05 | -0.188 | - | - |
| 7055 | ENSP00000331682 | KLHL22 | 3.621E-05 | -0.188 | - | - |
| 7056 | ENSP00000347433 | APCDD1 | 3.534E-05 | -0.188 | - | - |
| 7057 | ENSP00000345479 | ZNF587 | 1.494E-05 | -0.188 | - | - |
| 7058 | ENSP00000297164 | RELL2 | 2.448E-05 | -0.188 | - | - |
| 7059 | ENSP00000460371 | OCLM | 1.680E-05 | -0.188 | - | - |
| 7060 | ENSP00000374332 | TSN | 1.865E-05 | -0.188 | - | - |
| 7061 | ENSP00000295124 | CCDC138 | 2.888E-04 | -0.188 | - | - |
| 7062 | ENSP00000286234 | DEPTOR | 5.387E-05 | -0.188 | - | - |
| 7063 | ENSP00000363529 | FAM83C | 1.183E-05 | -0.188 | - | - |
| 7064 | ENSP00000256997 | ACP2 | 4.760E-05 | -0.188 | - | - |
| 7065 | ENSP00000472919 | ERVV-2 | 4.997E-06 | -0.188 | - | - |
| 7066 | ENSP00000311405 | ADCY6 | 3.301E-05 | -0.188 | - | - |
| 7067 | ENSP00000298139 | WRN | 2.227E-05 | -0.188 | - | - |
| 7068 | ENSP00000261244 | KIAA0586 | 1.042E-04 | -0.188 | - | - |
| 7069 | ENSP00000352527 | KCNK5 | 3.066E-05 | -0.188 | - | - |
| 7070 | ENSP00000368189 | ARID3C | 2.724E-05 | -0.188 | - | - |
| 7071 | ENSP00000331397 | FAM43B | 1.870E-04 | -0.189 | - | - |
| 7072 | ENSP00000265379 | COL6A5 | 7.334E-05 | -0.189 | - | - |
| 7073 | ENSP00000290902 | SPON2 | 5.603E-05 | -0.189 | - | - |
| 7074 | ENSP00000289081 | FANCC | 2.757E-05 | -0.189 | - | - |
| 7075 | ENSP00000318697 | TUBB6 | 2.486E-05 | -0.189 | - | - |
| 7076 | ENSP00000252252 | KRT6B | 3.113E-05 | -0.189 | - | - |
| 7077 | ENSP00000370803 | CCP110 | 2.210E-05 | -0.189 | - | - |
| 7078 | ENSP00000381891 | ERG | 2.976E-05 | -0.189 | - | - |
| 7079 | ENSP00000247001 | SUGP1 | 3.838E-05 | -0.189 | - | - |
| 7080 | ENSP00000222728 | HOXA6 | 4.663E-05 | -0.189 | - | - |
| 7081 | ENSP00000267859 | BNIP2 | 7.128E-05 | -0.189 | - | - |
| 7082 | ENSP00000380174 | ZSCAN29 | 1.026E-05 | -0.189 | - | - |
| 7083 | ENSP00000352802 | WIPF1 | 4.963E-05 | -0.189 | - | - |
| 7084 | ENSP00000276659 | RSPO2 | 3.773E-05 | -0.189 | - | - |
| 7085 | ENSP00000252655 | RSPH3 | 9.600E-05 | -0.189 | - | - |
| 7086 | ENSP00000234454 | SPR | 7.707E-05 | -0.189 | - | - |
| 7087 | ENSP00000362115 | INPP5B | 3.022E-05 | -0.189 | - | - |
| 7088 | ENSP00000358547 | MPP1 | 1.953E-05 | -0.189 | - | - |
| 7089 | ENSP00000360034 | SERBP1 | 4.494E-05 | -0.189 | - | - |
| 7090 | ENSP00000353698 | CNPPD1 | 6.734E-06 | -0.189 | - | - |
| 7091 | ENSP00000222753 | HOXA13 | 3.984E-05 | -0.189 | - | - |
| 7092 | ENSP00000382815 | KIAA2026 | 1.100E-05 | -0.189 | - | - |
| 7093 | ENSP00000370119 | SMN2 | 3.545E-05 | -0.189 | - | - |
| 7094 | ENSP00000304854 | NUDCD2 | 3.338E-05 | -0.189 | - | - |
| 7095 | ENSP00000361784 | ZNF684 | 1.156E-05 | -0.189 | - | - |
| 7096 | ENSP00000333994 | HBB | 3.987E-05 | -0.189 | - | - |
| 7097 | ENSP00000369860 | MOSPD2 | 3.177E-05 | -0.189 | - | - |
| 7098 | ENSP00000348708 | UPF2 | 2.356E-05 | -0.189 | - | - |
| 7099 | ENSP00000298352 | NGB | 3.064E-05 | -0.189 | - | - |
| 7100 | ENSP00000396402 | ZNF793 | 9.315E-06 | -0.189 | - | - |
| 7101 | ENSP00000176763 | STK10 | 1.115E-04 | -0.189 | - | - |
| 7102 | ENSP00000341243 | RBPJL | 4.812E-05 | -0.189 | - | - |
| 7103 | ENSP00000408792 | SP8 | 2.244E-05 | -0.189 | - | - |
| 7104 | ENSP00000354964 | ZNF318 | 7.479E-05 | -0.189 | - | - |
| 7105 | ENSP00000368414 | BCAP29 | 2.029E-05 | -0.189 | - | - |
| 7106 | ENSP00000361672 | FOXO6 | 1.240E-05 | -0.189 | - | - |
| 7107 | ENSP00000350616 | DDC | 2.346E-05 | -0.189 | - | - |
| 7108 | ENSP00000316454 | PACS1 | 5.714E-05 | -0.189 | - | - |
| 7109 | ENSP00000350773 | ZNF329 | 1.192E-05 | -0.189 | - | - |
| 7110 | ENSP00000432016 | ANKHD1-EIF4EBP3 | 3.679E-05 | -0.189 | - | - |
| 7111 | ENSP00000332134 | PRAMEF1 | 3.886E-04 | -0.189 | - | - |
| 7112 | ENSP00000260682 | CYP2C9 | 4.085E-05 | -0.189 | - | - |
| 7113 | ENSP00000314414 | AP2B1 | 3.379E-05 | -0.189 | - | - |
| 7114 | ENSP00000302051 | ECEL1 | 4.105E-05 | -0.189 | - | - |
| 7115 | ENSP00000253024 | TRIM28 | 4.183E-05 | -0.189 | - | - |
| 7116 | ENSP00000265963 | GTF2H1 | 3.378E-05 | -0.189 | - | - |
| 7117 | ENSP00000258381 | SP110 | 5.526E-05 | -0.189 | - | - |
| 7118 | ENSP00000394086 | ZNF84 | 1.302E-05 | -0.189 | - | - |
| 7119 | ENSP00000264031 | UPK2 | 1.214E-04 | -0.189 | - | - |
| 7120 | ENSP00000349576 | DCTD | 1.879E-05 | -0.189 | - | - |
| 7121 | ENSP00000360500 | DMRTB1 | 2.394E-05 | -0.189 | - | - |
| 7122 | ENSP00000310260 | INTS6 | 3.764E-05 | -0.189 | - | - |
| 7123 | ENSP00000376921 | NTNG2 | 2.971E-05 | -0.189 | - | - |
| 7124 | ENSP00000252951 | HBZ | 3.187E-05 | -0.189 | - | - |
| 7125 | ENSP00000305632 | HOMER2 | 2.985E-05 | -0.189 | - | - |
| 7126 | ENSP00000264714 | PPM1G | 2.358E-05 | -0.189 | - | - |
| 7127 | ENSP00000411099 | TLK1 | 3.607E-05 | -0.189 | - | - |
| 7128 | ENSP00000315474 | GIMAP7 | 2.519E-04 | -0.189 | - | - |
| 7129 | ENSP00000233202 | SLC11A1 | 6.945E-05 | -0.189 | - | - |
| 7130 | ENSP00000264914 | ARSB | 3.288E-05 | -0.189 | - | - |
| 7131 | ENSP00000246785 | BCL2L12 | 6.586E-05 | -0.189 | - | - |
| 7132 | ENSP00000369820 | PIGA | 2.896E-05 | -0.189 | - | - |
| 7133 | ENSP00000325002 | COPG1 | 4.040E-05 | -0.189 | - | - |
| 7134 | ENSP00000220058 | MTFMT | 2.203E-05 | -0.189 | - | - |
| 7135 | ENSP00000002165 | FUCA2 | 5.630E-05 | -0.189 | - | - |
| 7136 | ENSP00000379689 | ZNF765 | 9.894E-06 | -0.189 | - | - |
| 7137 | ENSP00000353073 | CLN3 | 3.646E-05 | -0.189 | - | - |
| 7138 | ENSP00000347045 | ZNF43 | 1.186E-05 | -0.189 | - | - |
| 7139 | ENSP00000320672 | COX6B2 | 3.050E-05 | -0.189 | - | - |
| 7140 | ENSP00000219431 | MPG | 2.860E-05 | -0.189 | - | - |
| 7141 | ENSP00000297142 | NEUROD6 | 5.420E-05 | -0.189 | - | - |
| 7142 | ENSP00000376930 | TMCC1 | 7.121E-06 | -0.189 | - | - |
| 7143 | ENSP00000377504 | ZNF414 | 1.231E-05 | -0.189 | - | - |
| 7144 | ENSP00000263867 | CAPG | 3.909E-05 | -0.189 | - | - |
| 7145 | ENSP00000361373 | KLF17 | 1.219E-05 | -0.189 | - | - |
| 7146 | ENSP00000335281 | DEFB105B | 9.309E-06 | -0.189 | - | - |
| 7147 | ENSP00000263119 | CABIN1 | 4.445E-05 | -0.189 | - | - |
| 7148 | ENSP00000380156 | RPL32 | 2.309E-05 | -0.189 | - | - |
| 7149 | ENSP00000334872 | MT1F | 8.224E-05 | -0.189 | - | - |
| 7150 | ENSP00000458866 | CTB-133G6.1 | 8.703E-07 | -0.189 | - | - |
| 7151 | ENSP00000348206 | SLMO2 | 3.885E-05 | -0.189 | - | - |
| 7152 | ENSP00000257118 | PHC2 | 3.678E-05 | -0.189 | - | - |
| 7153 | ENSP00000257789 | ORC3 | 4.888E-05 | -0.189 | - | - |
| 7154 | ENSP00000373153 | PTPLB | 2.336E-05 | -0.189 | - | - |
| 7155 | ENSP00000264605 | MLPH | 3.329E-05 | -0.189 | - | - |
| 7156 | ENSP00000471714 | AC068620.1 | 9.106E-06 | -0.189 | - | - |
| 7157 | ENSP00000256999 | FOLH1 | 2.825E-05 | -0.189 | - | - |
| 7158 | ENSP00000351631 | FAM76B | 2.272E-05 | -0.189 | - | - |
| 7159 | ENSP00000318406 | KIAA0319L | 4.973E-05 | -0.190 | - | - |
| 7160 | ENSP00000360939 | CMPK1 | 1.264E-05 | -0.190 | - | - |
| 7161 | ENSP00000354632 | MT-ATP6 | 2.685E-05 | -0.190 | - | - |
| 7162 | ENSP00000344446 | FGD6 | 4.874E-05 | -0.190 | - | - |
| 7163 | ENSP00000318883 | BCL2L13 | 9.127E-05 | -0.190 | - | - |
| 7164 | ENSP00000252137 | DGCR14 | 3.143E-05 | -0.190 | - | - |
| 7165 | ENSP00000155840 | KCNQ1 | 1.812E-05 | -0.190 | - | - |
| 7166 | ENSP00000289448 | HMHB1 | 1.805E-05 | -0.190 | - | - |
| 7167 | ENSP00000351632 | FAM3D | 1.794E-05 | -0.190 | - | - |
| 7168 | ENSP00000216807 | BRMS1L | 4.618E-05 | -0.190 | - | - |
| 7169 | ENSP00000319254 | GIPC3 | 4.572E-05 | -0.190 | - | - |
| 7170 | ENSP00000262843 | MID2 | 5.761E-05 | -0.190 | - | - |
| 7171 | ENSP00000255175 | SERINC3 | 1.660E-05 | -0.190 | - | - |
| 7172 | ENSP00000307252 | SMOX | 3.923E-05 | -0.190 | - | - |
| 7173 | ENSP00000331342 | RAB11FIP1 | 5.014E-05 | -0.190 | - | - |
| 7174 | ENSP00000354947 | CAPZA2 | 3.276E-05 | -0.190 | - | - |
| 7175 | ENSP00000283628 | UBP1 | 3.570E-05 | -0.190 | - | - |
| 7176 | ENSP00000270747 | ARHGEF19 | 2.774E-05 | -0.190 | - | - |
| 7177 | ENSP00000288199 | RNF111 | 3.302E-05 | -0.190 | - | - |
| 7178 | ENSP00000263578 | FOXRED1 | 4.597E-05 | -0.190 | - | - |
| 7179 | ENSP00000326737 | TNFRSF12A | 9.757E-05 | -0.190 | - | - |
| 7180 | ENSP00000223528 | FKTN | 2.504E-05 | -0.190 | - | - |
| 7181 | ENSP00000361943 | HEYL | 2.157E-05 | -0.190 | - | - |
| 7182 | ENSP00000263368 | BLVRB | 4.146E-05 | -0.190 | - | - |
| 7183 | ENSP00000268793 | DPEP3 | 3.022E-05 | -0.190 | - | - |
| 7184 | ENSP00000304139 | RBMXL2 | 4.829E-05 | -0.190 | - | - |
| 7185 | ENSP00000348108 | KHDRBS3 | 2.069E-05 | -0.190 | - | - |
| 7186 | ENSP00000314484 | MEIOB | 3.634E-05 | -0.190 | - | - |
| 7187 | ENSP00000298282 | PKNOX2 | 2.863E-05 | -0.190 | - | - |
| 7188 | ENSP00000378792 | ZNF169 | 1.150E-05 | -0.190 | - | - |
| 7189 | ENSP00000384160 | RBM33 | 7.366E-05 | -0.190 | - | - |
| 7190 | ENSP00000399664 | ZFP69B | 1.001E-05 | -0.190 | - | - |
| 7191 | ENSP00000312066 | SRP68 | 3.754E-05 | -0.190 | - | - |
| 7192 | ENSP00000241305 | CPXM2 | 2.525E-05 | -0.190 | - | - |
| 7193 | ENSP00000245903 | CD70 | 4.633E-04 | -0.190 | - | - |
| 7194 | ENSP00000356562 | XPR1 | 2.369E-05 | -0.190 | - | - |
| 7195 | ENSP00000261789 | TM9SF1 | 8.259E-05 | -0.190 | - | - |
| 7196 | ENSP00000362013 | TRERF1 | 4.065E-05 | -0.190 | - | - |
| 7197 | ENSP00000380414 | DEK | 3.430E-05 | -0.190 | - | - |
| 7198 | ENSP00000391806 | TAP2 | 1.229E-05 | -0.190 | - | - |
| 7199 | ENSP00000290776 | CPNE2 | 5.090E-05 | -0.190 | - | - |
| 7200 | ENSP00000071281 | FHL1 | 5.511E-05 | -0.190 | - | - |
| 7201 | ENSP00000320434 | CTNNAL1 | 3.909E-05 | -0.190 | - | - |
| 7202 | ENSP00000233557 | NRBP1 | 6.008E-05 | -0.190 | - | - |
| 7203 | ENSP00000250244 | AP1M2 | 2.629E-05 | -0.190 | - | - |
| 7204 | ENSP00000382021 | LCMT1 | 2.942E-05 | -0.190 | - | - |
| 7205 | ENSP00000291547 | PKNOX1 | 2.704E-05 | -0.190 | - | - |
| 7206 | ENSP00000328287 | RNF123 | 3.851E-05 | -0.190 | - | - |
| 7207 | ENSP00000350028 | MOV10 | 2.040E-05 | -0.190 | - | - |
| 7208 | ENSP00000352606 | HAPLN3 | 2.590E-05 | -0.190 | - | - |
| 7209 | ENSP00000287701 | HMBOX1 | 4.079E-05 | -0.190 | - | - |
| 7210 | ENSP00000264982 | CEP70 | 6.118E-05 | -0.190 | - | - |
| 7211 | ENSP00000359606 | ZNHIT6 | 4.380E-05 | -0.190 | - | - |
| 7212 | ENSP00000345809 | ZNF197 | 1.285E-05 | -0.190 | - | - |
| 7213 | ENSP00000282369 | TRIM36 | 4.242E-05 | -0.190 | - | - |
| 7214 | ENSP00000326342 | ELMOD2 | 6.226E-05 | -0.190 | - | - |
| 7215 | ENSP00000303887 | DCLK2 | 1.438E-05 | -0.190 | - | - |
| 7216 | ENSP00000221403 | DHDH | 1.735E-05 | -0.190 | - | - |
| 7217 | ENSP00000262146 | MTFR1 | 8.418E-05 | -0.190 | - | - |
| 7218 | ENSP00000258062 | REPS1 | 5.828E-05 | -0.190 | - | - |
| 7219 | ENSP00000294016 | ADCY9 | 4.298E-05 | -0.190 | - | - |
| 7220 | ENSP00000252854 | OLFM1 | 2.727E-05 | -0.190 | - | - |
| 7221 | ENSP00000262753 | POF1B | 5.628E-05 | -0.190 | - | - |
| 7222 | ENSP00000364929 | ING1 | 3.416E-05 | -0.190 | - | - |
| 7223 | ENSP00000353957 | ZNF567 | 1.371E-05 | -0.190 | - | - |
| 7224 | ENSP00000339207 | FAM122B | 4.687E-05 | -0.190 | - | - |
| 7225 | ENSP00000348527 | CENPL | 3.459E-05 | -0.190 | - | - |
| 7226 | ENSP00000225777 | SYNGR2 | 8.019E-05 | -0.190 | - | - |
| 7227 | ENSP00000264638 | CNTNAP1 | 2.767E-05 | -0.190 | - | - |
| 7228 | ENSP00000449002 | OR11H12 | 4.038E-06 | -0.190 | - | - |
| 7229 | ENSP00000302886 | PA2G4 | 2.874E-05 | -0.190 | - | - |
| 7230 | ENSP00000299238 | HPS6 | 8.644E-05 | -0.190 | - | - |
| 7231 | ENSP00000253457 | EMC8 | 6.693E-05 | -0.190 | - | - |
| 7232 | ENSP00000271308 | PSMA5 | 2.553E-05 | -0.190 | - | - |
| 7233 | ENSP00000351552 | GATAD2A | 3.360E-05 | -0.190 | - | - |
| 7234 | ENSP00000357051 | PEX19 | 2.079E-05 | -0.190 | - | - |
| 7235 | ENSP00000358155 | HIST2H2AA4 | 1.767E-05 | -0.190 | - | - |
| 7236 | ENSP00000352956 | RBM23 | 1.869E-05 | -0.190 | - | - |
| 7237 | ENSP00000364902 | POFUT1 | 3.307E-05 | -0.190 | - | - |
| 7238 | ENSP00000260598 | KHK | 4.675E-05 | -0.190 | - | - |
| 7239 | ENSP00000346509 | H2AFB2 | 1.319E-05 | -0.190 | - | - |
| 7240 | ENSP00000370867 | TGM3 | 2.560E-05 | -0.190 | - | - |
| 7241 | ENSP00000303570 | ANKMY2 | 5.074E-05 | -0.190 | - | - |
| 7242 | ENSP00000257860 | PRPH | 4.937E-05 | -0.190 | - | - |
| 7243 | ENSP00000261503 | SPECC1 | 2.606E-05 | -0.190 | - | - |
| 7244 | ENSP00000310901 | CSRP2 | 6.796E-05 | -0.190 | - | - |
| 7245 | ENSP00000265394 | CPVL | 3.349E-05 | -0.190 | - | - |
| 7246 | ENSP00000242210 | NT5C3A | 4.092E-05 | -0.190 | - | - |
| 7247 | ENSP00000219919 | AQP9 | 1.927E-05 | -0.190 | - | - |
| 7248 | ENSP00000331376 | SERHL2 | 5.012E-05 | -0.190 | - | - |
| 7249 | ENSP00000335615 | BTBD7 | 4.145E-05 | -0.190 | - | - |
| 7250 | ENSP00000271843 | JTB | 4.224E-05 | -0.191 | - | - |
| 7251 | ENSP00000388421 | ZNF487 | 1.358E-05 | -0.191 | - | - |
| 7252 | ENSP00000310338 | KIAA1551 | 7.173E-05 | -0.191 | - | - |
| 7253 | ENSP00000362751 | RABGAP1 | 2.074E-05 | -0.191 | - | - |
| 7254 | ENSP00000387310 | NOTUM | 4.241E-05 | -0.191 | - | - |
| 7255 | ENSP00000258243 | URB2 | 5.459E-05 | -0.191 | - | - |
| 7256 | ENSP00000338989 | TRIM16 | 5.179E-05 | -0.191 | - | - |
| 7257 | ENSP00000265107 | WDR70 | 5.421E-05 | -0.191 | - | - |
| 7258 | ENSP00000362308 | SH3BGRL | 3.450E-05 | -0.191 | - | - |
| 7259 | ENSP00000011473 | SYPL1 | 1.357E-04 | -0.191 | - | - |
| 7260 | ENSP00000361777 | SET | 4.622E-05 | -0.191 | - | - |
| 7261 | ENSP00000369371 | EQTN | 1.259E-05 | -0.191 | - | - |
| 7262 | ENSP00000263563 | PALD1 | 1.092E-04 | -0.191 | - | - |
| 7263 | ENSP00000335029 | RASA3 | 3.665E-05 | -0.191 | - | - |
| 7264 | ENSP00000361597 | SPATA25 | 2.185E-06 | -0.191 | - | - |
| 7265 | ENSP00000315564 | LGALS9B | 6.507E-05 | -0.191 | - | - |
| 7266 | ENSP00000273368 | TAGLN3 | 2.340E-05 | -0.191 | - | - |
| 7267 | ENSP00000246957 | TRAP1 | 1.673E-05 | -0.191 | - | - |
| 7268 | ENSP00000379931 | UBE2E2 | 4.561E-05 | -0.191 | - | - |
| 7269 | ENSP00000382779 | SEH1L | 3.325E-05 | -0.191 | - | - |
| 7270 | ENSP00000296955 | DCBLD1 | 7.841E-05 | -0.191 | - | - |
| 7271 | ENSP00000306844 | SEMA4C | 3.837E-05 | -0.191 | - | - |
| 7272 | ENSP00000359976 | CTH | 2.586E-05 | -0.191 | - | - |
| 7273 | ENSP00000307513 | MRC2 | 7.135E-05 | -0.191 | - | - |
| 7274 | ENSP00000384048 | PAXIP1 | 2.190E-05 | -0.191 | - | - |
| 7275 | ENSP00000312550 | SEZ6L2 | 7.394E-05 | -0.191 | - | - |
| 7276 | ENSP00000363135 | C6orf106 | 1.896E-05 | -0.191 | - | - |
| 7277 | ENSP00000301732 | ABCA3 | 3.188E-05 | -0.191 | - | - |
| 7278 | ENSP00000306881 | SEC23A | 3.607E-05 | -0.191 | - | - |
| 7279 | ENSP00000260970 | PPIG | 2.860E-05 | -0.191 | - | - |
| 7280 | ENSP00000274507 | LECT2 | 9.283E-05 | -0.191 | - | - |
| 7281 | ENSP00000233121 | MAPRE3 | 2.192E-05 | -0.191 | - | - |
| 7282 | ENSP00000379607 | KNCN | 2.112E-05 | -0.191 | - | - |
| 7283 | ENSP00000170447 | MKRN2 | 6.556E-05 | -0.191 | - | - |
| 7284 | ENSP00000365494 | PRICKLE3 | 2.526E-05 | -0.191 | - | - |
| 7285 | ENSP00000378504 | NXF3 | 2.288E-05 | -0.191 | - | - |
| 7286 | ENSP00000256443 | CDK7 | 1.650E-05 | -0.191 | - | - |
| 7287 | ENSP00000256389 | ADAM20 | 3.698E-05 | -0.191 | - | - |
| 7288 | ENSP00000263688 | SUCO | 3.279E-05 | -0.191 | - | - |
| 7289 | ENSP00000340925 | NADK | 3.390E-05 | -0.191 | - | - |
| 7290 | ENSP00000360922 | STAU1 | 3.284E-05 | -0.191 | - | - |
| 7291 | ENSP00000216214 | FAM118A | 9.958E-05 | -0.191 | - | - |
| 7292 | ENSP00000345060 | GPR179 | 2.186E-05 | -0.191 | - | - |
| 7293 | ENSP00000237536 | SOGA1 | 7.691E-05 | -0.191 | - | - |
| 7294 | ENSP00000215770 | DDTL | 8.406E-05 | -0.191 | - | - |
| 7295 | ENSP00000468271 | ZFP112 | 9.166E-06 | -0.191 | - | - |
| 7296 | ENSP00000182377 | FAR2 | 5.673E-05 | -0.191 | - | - |
| 7297 | ENSP00000209884 | KLHL20 | 4.051E-05 | -0.191 | - | - |
| 7298 | ENSP00000355613 | TRIM67 | 2.636E-05 | -0.191 | - | - |
| 7299 | ENSP00000345848 | ANP32B | 2.902E-05 | -0.191 | - | - |
| 7300 | ENSP00000238112 | CPSF3 | 2.201E-05 | -0.191 | - | - |
| 7301 | ENSP00000217109 | CSTF1 | 3.461E-05 | -0.191 | - | - |
| 7302 | ENSP00000346378 | APBB3 | 2.795E-05 | -0.191 | - | - |
| 7303 | ENSP00000011691 | SS18L2 | 8.031E-05 | -0.191 | - | - |
| 7304 | ENSP00000303769 | AKNA | 7.208E-05 | -0.191 | - | - |
| 7305 | ENSP00000318916 | MARCO | 2.271E-05 | -0.191 | - | - |
| 7306 | ENSP00000387911 | PARN | 7.054E-05 | -0.191 | - | - |
| 7307 | ENSP00000291281 | PRKD2 | 3.596E-05 | -0.191 | - | - |
| 7308 | ENSP00000241337 | GSTM2 | 4.652E-05 | -0.191 | - | - |
| 7309 | ENSP00000362166 | MEAF6 | 2.624E-05 | -0.191 | - | - |
| 7310 | ENSP00000313140 | EXD2 | 4.156E-05 | -0.191 | - | - |
| 7311 | ENSP00000366828 | POLR3F | 1.233E-05 | -0.191 | - | - |
| 7312 | ENSP00000217289 | FERMT1 | 3.385E-05 | -0.191 | - | - |
| 7313 | ENSP00000273063 | SLC4A3 | 2.628E-05 | -0.191 | - | - |
| 7314 | ENSP00000355777 | ACBD3 | 3.063E-05 | -0.191 | - | - |
| 7315 | ENSP00000313391 | DAB2 | 3.335E-05 | -0.191 | - | - |
| 7316 | ENSP00000368523 | DNAJC15 | 1.562E-05 | -0.191 | - | - |
| 7317 | ENSP00000242159 | HOXA7 | 2.712E-05 | -0.191 | - | - |
| 7318 | ENSP00000369519 | MTAP | 2.238E-05 | -0.191 | - | - |
| 7319 | ENSP00000296456 | APEH | 2.769E-05 | -0.191 | - | - |
| 7320 | ENSP00000254810 | H3F3B | 1.813E-05 | -0.191 | - | - |
| 7321 | ENSP00000308452 | KRT17 | 1.083E-04 | -0.191 | - | - |
| 7322 | ENSP00000216185 | TXN2 | 1.504E-05 | -0.191 | - | - |
| 7323 | ENSP00000186436 | TMEM131 | 3.783E-05 | -0.191 | - | - |
| 7324 | ENSP00000169298 | ST6GAL1 | 2.450E-05 | -0.191 | - | - |
| 7325 | ENSP00000328729 | 15-Sep | 4.400E-05 | -0.191 | - | - |
| 7326 | ENSP00000363095 | TRIM32 | 1.081E-05 | -0.191 | - | - |
| 7327 | ENSP00000301015 | PIEZO1 | 4.839E-05 | -0.191 | - | - |
| 7328 | ENSP00000244360 | RNF39 | 5.590E-05 | -0.191 | - | - |
| 7329 | ENSP00000419005 | DIEXF | 3.045E-05 | -0.191 | - | - |
| 7330 | ENSP00000379183 | PANX2 | 7.623E-06 | -0.191 | - | - |
| 7331 | ENSP00000239666 | PDZD11 | 9.329E-05 | -0.191 | - | - |
| 7332 | ENSP00000225726 | CCDC47 | 5.060E-05 | -0.191 | - | - |
| 7333 | ENSP00000366612 | CBWD5 | 2.960E-05 | -0.191 | - | - |
| 7334 | ENSP00000300456 | SLC27A4 | 3.434E-05 | -0.191 | - | - |
| 7335 | ENSP00000272286 | ABCG8 | 3.269E-05 | -0.191 | - | - |
| 7336 | ENSP00000457715 | ZNF865 | 1.136E-05 | -0.191 | - | - |
| 7337 | ENSP00000299638 | POLR2M | 4.119E-05 | -0.191 | - | - |
| 7338 | ENSP00000234195 | RMDN2 | 1.118E-04 | -0.191 | - | - |
| 7339 | ENSP00000229277 | ENO2 | 1.777E-05 | -0.191 | - | - |
| 7340 | ENSP00000335091 | ZNRF1 | 6.818E-05 | -0.191 | - | - |
| 7341 | ENSP00000262290 | LPO | 3.182E-05 | -0.191 | - | - |
| 7342 | ENSP00000405965 | SUMO2 | 1.119E-02 | -0.191 | - | - |
| 7343 | ENSP00000296679 | WDR41 | 1.394E-04 | -0.191 | - | - |
| 7344 | ENSP00000332359 | TCEAL2 | 9.024E-05 | -0.191 | - | - |
| 7345 | ENSP00000295131 | ZSWIM2 | 3.430E-05 | -0.192 | - | - |
| 7346 | ENSP00000258449 | TGFBRAP1 | 3.532E-05 | -0.192 | - | - |
| 7347 | ENSP00000264734 | CLDN16 | 1.483E-05 | -0.192 | - | - |
| 7348 | ENSP00000258484 | EPC2 | 3.364E-05 | -0.192 | - | - |
| 7349 | ENSP00000276218 | GPR119 | 4.296E-05 | -0.192 | - | - |
| 7350 | ENSP00000319918 | ITFG1 | 6.402E-05 | -0.192 | - | - |
| 7351 | ENSP00000326649 | HPS1 | 4.944E-05 | -0.192 | - | - |
| 7352 | ENSP00000307843 | EGFL7 | 4.111E-05 | -0.192 | - | - |
| 7353 | ENSP00000262878 | SAMHD1 | 4.619E-05 | -0.192 | - | - |
| 7354 | ENSP00000314792 | BOLL | 2.497E-05 | -0.192 | - | - |
| 7355 | ENSP00000369681 | USP3 | 9.183E-06 | -0.192 | - | - |
| 7356 | ENSP00000315111 | C8orf42 | 4.004E-05 | -0.192 | - | - |
| 7357 | ENSP00000355963 | LPGAT1 | 2.020E-05 | -0.192 | - | - |
| 7358 | ENSP00000270776 | PGD | 2.670E-05 | -0.192 | - | - |
| 7359 | ENSP00000334801 | DHRS4L2 | 3.336E-05 | -0.192 | - | - |
| 7360 | ENSP00000268099 | SCAMP2 | 4.280E-05 | -0.192 | - | - |
| 7361 | ENSP00000269439 | RNF165 | 4.913E-05 | -0.192 | - | - |
| 7362 | ENSP00000306117 | DDX19A | 2.339E-05 | -0.192 | - | - |
| 7363 | ENSP00000305263 | KRT28 | 4.891E-04 | -0.192 | - | - |
| 7364 | ENSP00000296257 | SENP2 | 4.730E-05 | -0.192 | - | - |
| 7365 | ENSP00000351035 | HSD17B11 | 1.297E-05 | -0.192 | - | - |
| 7366 | ENSP00000225328 | P2RX5 | 7.554E-05 | -0.192 | - | - |
| 7367 | ENSP00000303997 | GTPBP2 | 5.255E-05 | -0.192 | - | - |
| 7368 | ENSP00000349145 | MYO16 | 9.492E-06 | -0.192 | - | - |
| 7369 | ENSP00000279227 | FERMT3 | 3.946E-05 | -0.192 | - | - |
| 7370 | ENSP00000322170 | PFN4 | 2.543E-05 | -0.192 | - | - |
| 7371 | ENSP00000296003 | MTMR14 | 5.581E-05 | -0.192 | - | - |
| 7372 | ENSP00000316881 | LEPREL1 | 4.042E-05 | -0.192 | - | - |
| 7373 | ENSP00000360488 | FRA10AC1 | 2.439E-05 | -0.192 | - | - |
| 7374 | ENSP00000240327 | SPOP | 2.855E-05 | -0.192 | - | - |
| 7375 | ENSP00000278980 | COMMD7 | 4.980E-05 | -0.192 | - | - |
| 7376 | ENSP00000337396 | CCNB1IP1 | 4.275E-05 | -0.192 | - | - |
| 7377 | ENSP00000325958 | PTPMT1 | 5.540E-05 | -0.192 | - | - |
| 7378 | ENSP00000337053 | SEL1L | 5.341E-05 | -0.192 | - | - |
| 7379 | ENSP00000334037 | EMC10 | 6.149E-05 | -0.192 | - | - |
| 7380 | ENSP00000437878 | ZNF595 | 9.304E-06 | -0.192 | - | - |
| 7381 | ENSP00000326819 | FANCB | 4.805E-05 | -0.192 | - | - |
| 7382 | ENSP00000263094 | ABCA7 | 2.707E-05 | -0.192 | - | - |
| 7383 | ENSP00000310332 | FBXO45 | 3.353E-05 | -0.192 | - | - |
| 7384 | ENSP00000291568 | CSTB | 3.126E-05 | -0.192 | - | - |
| 7385 | ENSP00000311221 | ZMAT3 | 4.106E-05 | -0.192 | - | - |
| 7386 | ENSP00000306473 | SHCBP1 | 4.131E-05 | -0.192 | - | - |
| 7387 | ENSP00000390661 | CLEC2L | 8.893E-06 | -0.192 | - | - |
| 7388 | ENSP00000396080 | NUTM2D | 2.001E-07 | -0.192 | - | - |
| 7389 | ENSP00000268676 | DEF8 | 8.850E-05 | -0.192 | - | - |
| 7390 | ENSP00000307423 | MGAT2 | 2.666E-05 | -0.192 | - | - |
| 7391 | ENSP00000462745 | DCAF8L2 | 1.622E-05 | -0.192 | - | - |
| 7392 | ENSP00000293745 | KRT72 | 4.867E-05 | -0.192 | - | - |
| 7393 | ENSP00000350576 | ZNF441 | 1.265E-05 | -0.192 | - | - |
| 7394 | ENSP00000332293 | ASCL2 | 2.322E-05 | -0.192 | - | - |
| 7395 | ENSP00000337733 | KLK5 | 3.481E-05 | -0.192 | - | - |
| 7396 | ENSP00000298310 | NEMF | 3.664E-05 | -0.192 | - | - |
| 7397 | ENSP00000358674 | UBL4A | 1.070E-04 | -0.192 | - | - |
| 7398 | ENSP00000283141 | SYCP2L | 1.921E-05 | -0.192 | - | - |
| 7399 | ENSP00000363433 | GPRIN2 | 2.126E-05 | -0.192 | - | - |
| 7400 | ENSP00000349696 | CEPT1 | 2.142E-05 | -0.192 | - | - |
| 7401 | ENSP00000296658 | CMBL | 4.620E-05 | -0.192 | - | - |
| 7402 | ENSP00000305664 | TRABD | 4.280E-05 | -0.192 | - | - |
| 7403 | ENSP00000273920 | ENOPH1 | 5.030E-05 | -0.192 | - | - |
| 7404 | ENSP00000274170 | CDH18 | 3.062E-05 | -0.192 | - | - |
| 7405 | ENSP00000309576 | LGALS8 | 4.366E-05 | -0.192 | - | - |
| 7406 | ENSP00000264389 | COPS4 | 3.623E-05 | -0.192 | - | - |
| 7407 | ENSP00000308330 | MYEOV | 6.920E-05 | -0.192 | - | - |
| 7408 | ENSP00000319977 | NDRG1 | 3.241E-05 | -0.192 | - | - |
| 7409 | ENSP00000233893 | HSPE1 | 2.075E-05 | -0.192 | - | - |
| 7410 | ENSP00000240651 | PYROXD1 | 9.061E-05 | -0.192 | - | - |
| 7411 | ENSP00000263795 | WDR76 | 3.849E-05 | -0.192 | - | - |
| 7412 | ENSP00000221671 | C19orf44 | 3.043E-03 | -0.192 | - | - |
| 7413 | ENSP00000285879 | MAGEC1 | 2.507E-05 | -0.192 | - | - |
| 7414 | ENSP00000239926 | MYOT | 3.698E-05 | -0.192 | - | - |
| 7415 | ENSP00000304422 | KREMEN2 | 2.723E-05 | -0.192 | - | - |
| 7416 | ENSP00000336627 | SLITRK4 | 5.275E-05 | -0.192 | - | - |
| 7417 | ENSP00000337761 | RAB27A | 3.341E-05 | -0.192 | - | - |
| 7418 | ENSP00000216129 | TTLL12 | 4.608E-05 | -0.192 | - | - |
| 7419 | ENSP00000216951 | GSS | 3.540E-05 | -0.192 | - | - |
| 7420 | ENSP00000216733 | EFS | 5.609E-05 | -0.192 | - | - |
| 7421 | ENSP00000314036 | AGPAT4 | 2.435E-05 | -0.192 | - | - |
| 7422 | ENSP00000335371 | TRAPPC11 | 3.940E-05 | -0.192 | - | - |
| 7423 | ENSP00000357425 | SMPDL3A | 2.876E-05 | -0.192 | - | - |
| 7424 | ENSP00000012049 | QPCTL | 4.941E-05 | -0.192 | - | - |
| 7425 | ENSP00000268679 | CBFA2T3 | 3.606E-05 | -0.192 | - | - |
| 7426 | ENSP00000265968 | CSRP3 | 2.573E-05 | -0.192 | - | - |
| 7427 | ENSP00000335004 | TRABD2A | 4.157E-04 | -0.192 | - | - |
| 7428 | ENSP00000291009 | PIP | 4.116E-05 | -0.192 | - | - |
| 7429 | ENSP00000247306 | CTAG2 | 8.234E-03 | -0.192 | - | - |
| 7430 | ENSP00000263088 | PLD2 | 2.155E-05 | -0.192 | - | - |
| 7431 | ENSP00000258884 | ABHD17C | 3.913E-05 | -0.192 | - | - |
| 7432 | ENSP00000244573 | HIST1H1A | 3.209E-05 | -0.192 | - | - |
| 7433 | ENSP00000352678 | SUPV3L1 | 2.512E-05 | -0.192 | - | - |
| 7434 | ENSP00000235347 | PRAMEF10 | 1.205E-03 | -0.192 | - | - |
| 7435 | ENSP00000272771 | TMEFF2 | 3.246E-05 | -0.192 | - | - |
| 7436 | ENSP00000327994 | TRIM50 | 8.236E-05 | -0.192 | - | - |
| 7437 | ENSP00000296252 | LIPH | 4.971E-05 | -0.192 | - | - |
| 7438 | ENSP00000253332 | AKAP12 | 2.966E-05 | -0.192 | - | - |
| 7439 | ENSP00000402301 | LAPTM4B | 5.886E-04 | -0.192 | - | - |
| 7440 | ENSP00000257013 | FAM127A | 3.607E-05 | -0.192 | - | - |
| 7441 | ENSP00000333487 | MAGEA2B | 2.420E-05 | -0.192 | - | - |
| 7442 | ENSP00000353444 | RAB5B | 2.111E-05 | -0.192 | - | - |
| 7443 | ENSP00000219481 | DECR2 | 3.214E-05 | -0.192 | - | - |
| 7444 | ENSP00000355929 | ANGEL2 | 1.655E-05 | -0.192 | - | - |
| 7445 | ENSP00000377280 | FBXO8 | 1.248E-05 | -0.192 | - | - |
| 7446 | ENSP00000347836 | UBE2H | 4.134E-05 | -0.192 | - | - |
| 7447 | ENSP00000239367 | LRP11 | 1.282E-04 | -0.192 | - | - |
| 7448 | ENSP00000349955 | RPRD1A | 2.433E-05 | -0.192 | - | - |
| 7449 | ENSP00000315614 | C8orf47 | 4.811E-03 | -0.192 | - | - |
| 7450 | ENSP00000283645 | TUBGCP5 | 3.520E-05 | -0.192 | - | - |
| 7451 | ENSP00000261167 | WBP11 | 7.057E-05 | -0.192 | - | - |
| 7452 | ENSP00000330587 | MT1H | 7.135E-05 | -0.192 | - | - |
| 7453 | ENSP00000330730 | COA5 | 2.202E-05 | -0.193 | - | - |
| 7454 | ENSP00000228825 | ARPC3 | 2.611E-05 | -0.193 | - | - |
| 7455 | ENSP00000362127 | MTF1 | 1.686E-05 | -0.193 | - | - |
| 7456 | ENSP00000309439 | HOXB9 | 2.814E-05 | -0.193 | - | - |
| 7457 | ENSP00000325827 | SEC16A | 3.970E-05 | -0.193 | - | - |
| 7458 | ENSP00000233575 | SNX17 | 5.583E-05 | -0.193 | - | - |
| 7459 | ENSP00000355568 | IRF2BP2 | 2.887E-05 | -0.193 | - | - |
| 7460 | ENSP00000242577 | DYNLL1 | 2.070E-05 | -0.193 | - | - |
| 7461 | ENSP00000310573 | KRT25 | 1.579E-04 | -0.193 | - | - |
| 7462 | ENSP00000343645 | SULT1A3 | 2.780E-05 | -0.193 | - | - |
| 7463 | ENSP00000273375 | RABL3 | 6.561E-05 | -0.193 | - | - |
| 7464 | ENSP00000240189 | PRAMEF2 | 3.807E-03 | -0.193 | - | - |
| 7465 | ENSP00000360682 | C9orf142 | 1.183E-05 | -0.193 | - | - |
| 7466 | ENSP00000432472 | BRK1 | 3.669E-05 | -0.193 | - | - |
| 7467 | ENSP00000288228 | FAM81A | 3.727E-05 | -0.193 | - | - |
| 7468 | ENSP00000365397 | DHRS3 | 2.644E-05 | -0.193 | - | - |
| 7469 | ENSP00000302393 | LDHAL6B | 2.271E-05 | -0.193 | - | - |
| 7470 | ENSP00000340093 | NAPEPLD | 3.820E-05 | -0.193 | - | - |
| 7471 | ENSP00000256593 | GSTM5 | 3.740E-05 | -0.193 | - | - |
| 7472 | ENSP00000354775 | PJA2 | 2.176E-05 | -0.193 | - | - |
| 7473 | ENSP00000270172 | DNMT3L | 4.797E-05 | -0.193 | - | - |
| 7474 | ENSP00000310406 | RIN1 | 3.983E-05 | -0.193 | - | - |
| 7475 | ENSP00000286788 | CCT8 | 2.518E-05 | -0.193 | - | - |
| 7476 | ENSP00000358297 | RNF115 | 2.219E-05 | -0.193 | - | - |
| 7477 | ENSP00000309907 | NBPF14 | 1.981E-04 | -0.193 | - | - |
| 7478 | ENSP00000255608 | BTBD2 | 3.642E-05 | -0.193 | - | - |
| 7479 | ENSP00000362244 | MAP7D1 | 4.283E-05 | -0.193 | - | - |
| 7480 | ENSP00000005260 | BAIAP2L1 | 5.757E-05 | -0.193 | - | - |
| 7481 | ENSP00000286298 | SLC26A2 | 2.486E-05 | -0.193 | - | - |
| 7482 | ENSP00000365266 | PRAMEF17 | 1.183E-05 | -0.193 | - | - |
| 7483 | ENSP00000020673 | PSD | 2.558E-05 | -0.193 | - | - |
| 7484 | ENSP00000394705 | AL353791.1 | 1.183E-05 | -0.193 | - | - |
| 7485 | ENSP00000343298 | LRRC41 | 3.027E-05 | -0.193 | - | - |
| 7486 | ENSP00000262633 | RBM42 | 5.886E-05 | -0.193 | - | - |
| 7487 | ENSP00000221130 | GSR | 3.642E-05 | -0.193 | - | - |
| 7488 | ENSP00000296783 | PAPD4 | 2.514E-05 | -0.193 | - | - |
| 7489 | ENSP00000379133 | APIP | 1.966E-05 | -0.193 | - | - |
| 7490 | ENSP00000358033 | PDSS2 | 1.775E-05 | -0.193 | - | - |
| 7491 | ENSP00000349393 | LIG4 | 3.476E-05 | -0.193 | - | - |
| 7492 | ENSP00000282074 | SPC25 | 3.555E-05 | -0.193 | - | - |
| 7493 | ENSP00000295704 | RNF25 | 5.416E-05 | -0.193 | - | - |
| 7494 | ENSP00000371798 | FSCN1 | 2.763E-05 | -0.193 | - | - |
| 7495 | ENSP00000463013 | OAZ2 | 9.619E-06 | -0.193 | - | - |
| 7496 | ENSP00000307525 | NMD3 | 2.782E-05 | -0.193 | - | - |
| 7497 | ENSP00000349931 | PRAMEF8 | 3.661E-06 | -0.193 | - | - |
| 7498 | ENSP00000337512 | FTSJD1 | 2.838E-05 | -0.193 | - | - |
| 7499 | ENSP00000365576 | UBQLN1 | 1.100E-03 | -0.193 | - | - |
| 7500 | ENSP00000268802 | NOB1 | 2.175E-05 | -0.193 | - | - |
| 7501 | ENSP00000179259 | C12orf5 | 3.121E-05 | -0.193 | - | - |
| 7502 | ENSP00000275766 | ZC3HAV1L | 1.383E-03 | -0.193 | - | - |
| 7503 | ENSP00000316476 | DEGS1 | 3.388E-05 | -0.193 | - | - |
| 7504 | ENSP00000274764 | HIST1H2BA | 2.110E-05 | -0.193 | - | - |
| 7505 | ENSP00000354878 | KIF21A | 1.992E-05 | -0.193 | - | - |
| 7506 | ENSP00000431179 | KIAA0754 | 1.183E-05 | -0.193 | - | - |
| 7507 | ENSP00000256339 | UNC79 | 3.694E-05 | -0.193 | - | - |
| 7508 | ENSP00000395538 | TNRC18 | 3.223E-05 | -0.193 | - | - |
| 7509 | ENSP00000249806 | CLN6 | 4.944E-05 | -0.193 | - | - |
| 7510 | ENSP00000365289 | PRAMEF16 | 1.183E-05 | -0.193 | - | - |
| 7511 | ENSP00000358372 | NBPF8 | 1.183E-05 | -0.193 | - | - |
| 7512 | ENSP00000318113 | CLDN22 | 2.314E-05 | -0.193 | - | - |
| 7513 | ENSP00000344393 | FCF1 | 1.437E-05 | -0.193 | - | - |
| 7514 | ENSP00000004982 | HSPB6 | 3.581E-05 | -0.193 | - | - |
| 7515 | ENSP00000278836 | MYRF | 3.048E-05 | -0.193 | - | - |
| 7516 | ENSP00000313851 | PDS5B | 2.825E-05 | -0.193 | - | - |
| 7517 | ENSP00000321737 | MROH1 | 7.199E-05 | -0.193 | - | - |
| 7518 | ENSP00000297532 | FASTK | 1.314E-04 | -0.193 | - | - |
| 7519 | ENSP00000293845 | CCDC42 | 9.979E-05 | -0.193 | - | - |
| 7520 | ENSP00000258098 | RAB11FIP5 | 3.753E-05 | -0.193 | - | - |
| 7521 | ENSP00000216181 | MYH9 | 2.064E-05 | -0.193 | - | - |
| 7522 | ENSP00000362298 | SGPL1 | 1.702E-05 | -0.193 | - | - |
| 7523 | ENSP00000294244 | C11orf84 | 2.755E-03 | -0.193 | - | - |
| 7524 | ENSP00000288757 | C12orf43 | 7.856E-05 | -0.193 | - | - |
| 7525 | ENSP00000358228 | NBPF24 | 1.183E-05 | -0.193 | - | - |
| 7526 | ENSP00000329033 | SLC25A18 | 2.146E-05 | -0.193 | - | - |
| 7527 | ENSP00000358831 | ELOVL4 | 3.199E-05 | -0.193 | - | - |
| 7528 | ENSP00000340742 | DOCK7 | 3.592E-05 | -0.193 | - | - |
| 7529 | ENSP00000316737 | LCE1D | 2.459E-05 | -0.193 | - | - |
| 7530 | ENSP00000421868 | CCDC169-SOHLH2 | 1.183E-05 | -0.193 | - | - |
| 7531 | ENSP00000274605 | N4BP3 | 2.026E-04 | -0.193 | - | - |
| 7532 | ENSP00000365302 | PRAMEF13 | 1.183E-05 | -0.193 | - | - |
| 7533 | ENSP00000403130 | TMEM233 | 1.183E-05 | -0.193 | - | - |
| 7534 | ENSP00000230056 | GMNN | 3.535E-05 | -0.193 | - | - |
| 7535 | ENSP00000409009 | FAM195B | 1.183E-05 | -0.193 | - | - |
| 7536 | ENSP00000333001 | RBM8A | 1.789E-05 | -0.193 | - | - |
| 7537 | ENSP00000314518 | LYSMD3 | 8.129E-05 | -0.193 | - | - |
| 7538 | ENSP00000295709 | STK36 | 3.086E-05 | -0.193 | - | - |
| 7539 | ENSP00000303490 | C5orf34 | 1.270E-04 | -0.193 | - | - |
| 7540 | ENSP00000322730 | ITM2C | 3.387E-05 | -0.193 | - | - |
| 7541 | ENSP00000331363 | C19orf68 | 2.680E-04 | -0.193 | - | - |
| 7542 | ENSP00000342710 | KRT77 | 3.079E-05 | -0.193 | - | - |
| 7543 | ENSP00000309751 | SLC16A13 | 3.649E-05 | -0.193 | - | - |
| 7544 | ENSP00000391465 | GAGE12I | 1.428E-05 | -0.193 | - | - |
| 7545 | ENSP00000295658 | TMEM169 | 7.451E-04 | -0.193 | - | - |
| 7546 | ENSP00000456213 | RP4-734P14.4 | 9.212E-06 | -0.193 | - | - |
| 7547 | ENSP00000394936 | ORM2 | 2.148E-05 | -0.193 | - | - |
| 7548 | ENSP00000375810 | KLC3 | 3.648E-05 | -0.193 | - | - |
| 7549 | ENSP00000265523 | BLVRA | 3.007E-05 | -0.193 | - | - |
| 7550 | ENSP00000337889 | MXD4 | 4.584E-05 | -0.193 | - | - |
| 7551 | ENSP00000360843 | FAF1 | 4.029E-05 | -0.193 | - | - |
| 7552 | ENSP00000354206 | ZNF219 | 1.122E-05 | -0.193 | - | - |
| 7553 | ENSP00000344674 | DHRS2 | 2.998E-05 | -0.193 | - | - |
| 7554 | ENSP00000473243 | ZNF788 | 9.114E-06 | -0.193 | - | - |
| 7555 | ENSP00000216489 | ALKBH1 | 4.794E-05 | -0.193 | - | - |
| 7556 | ENSP00000363018 | RPL10A | 3.489E-05 | -0.193 | - | - |
| 7557 | ENSP00000250974 | ABHD17A | 3.555E-05 | -0.193 | - | - |
| 7558 | ENSP00000320232 | CCDC168 | 1.057E-03 | -0.193 | - | - |
| 7559 | ENSP00000245796 | PSD4 | 2.888E-05 | -0.193 | - | - |
| 7560 | ENSP00000245206 | GOT2 | 2.401E-05 | -0.193 | - | - |
| 7561 | ENSP00000263674 | ARHGEF17 | 3.933E-05 | -0.193 | - | - |
| 7562 | ENSP00000323967 | SMARCE1 | 3.228E-05 | -0.193 | - | - |
| 7563 | ENSP00000342075 | SIMC1 | 2.806E-04 | -0.193 | - | - |
| 7564 | ENSP00000287727 | ZFYVE9 | 3.789E-05 | -0.193 | - | - |
| 7565 | ENSP00000455659 | AC002310.13 | 9.212E-06 | -0.193 | - | - |
| 7566 | ENSP00000339374 | LGALS12 | 1.140E-04 | -0.193 | - | - |
| 7567 | ENSP00000323065 | GADD45GIP1 | 3.960E-05 | -0.193 | - | - |
| 7568 | ENSP00000380293 | ZNF99 | 1.056E-05 | -0.193 | - | - |
| 7569 | ENSP00000315371 | ANO5 | 5.211E-05 | -0.193 | - | - |
| 7570 | ENSP00000231357 | IRX4 | 2.221E-05 | -0.193 | - | - |
| 7571 | ENSP00000295079 | C2orf47 | 1.419E-04 | -0.193 | - | - |
| 7572 | ENSP00000355580 | KCNK1 | 3.657E-05 | -0.193 | - | - |
| 7573 | ENSP00000294258 | ZFPL1 | 3.040E-05 | -0.193 | - | - |
| 7574 | ENSP00000246914 | WNK4 | 3.049E-05 | -0.193 | - | - |
| 7575 | ENSP00000306320 | C14orf142 | 3.454E-04 | -0.193 | - | - |
| 7576 | ENSP00000265343 | AFF4 | 2.992E-05 | -0.193 | - | - |
| 7577 | ENSP00000340237 | SH3BP4 | 3.350E-05 | -0.193 | - | - |
| 7578 | ENSP00000337513 | SRA1 | 3.158E-05 | -0.193 | - | - |
| 7579 | ENSP00000472160 | AC003006.7 | 9.114E-06 | -0.193 | - | - |
| 7580 | ENSP00000473043 | CTD-2192J16.20 | 9.114E-06 | -0.193 | - | - |
| 7581 | ENSP00000311427 | SNX33 | 3.452E-05 | -0.193 | - | - |
| 7582 | ENSP00000187397 | ARPP21 | 2.305E-05 | -0.193 | - | - |
| 7583 | ENSP00000251343 | KHNYN | 2.143E-04 | -0.193 | - | - |
| 7584 | ENSP00000374185 | ZNF841 | 1.163E-05 | -0.194 | - | - |
| 7585 | ENSP00000244751 | CPNE5 | 3.404E-05 | -0.194 | - | - |
| 7586 | ENSP00000328364 | MAFA | 8.751E-05 | -0.194 | - | - |
| 7587 | ENSP00000300650 | RNF214 | 2.524E-04 | -0.194 | - | - |
| 7588 | ENSP00000292327 | MYL3 | 2.022E-05 | -0.194 | - | - |
| 7589 | ENSP00000413660 | ZNF492 | 1.132E-05 | -0.194 | - | - |
| 7590 | ENSP00000258105 | MRPL53 | 9.686E-05 | -0.194 | - | - |
| 7591 | ENSP00000319831 | DEF6 | 7.051E-05 | -0.194 | - | - |
| 7592 | ENSP00000336800 | KLHL25 | 4.660E-05 | -0.194 | - | - |
| 7593 | ENSP00000289269 | PCDHAC2 | 1.701E-04 | -0.194 | - | - |
| 7594 | ENSP00000357177 | ARHGEF11 | 2.421E-05 | -0.194 | - | - |
| 7595 | ENSP00000305692 | GAA | 4.382E-05 | -0.194 | - | - |
| 7596 | ENSP00000014914 | GPRC5A | 5.525E-05 | -0.194 | - | - |
| 7597 | ENSP00000215531 | C19orf77 | 2.354E-04 | -0.194 | - | - |
| 7598 | ENSP00000322977 | SSBP2 | 3.188E-05 | -0.194 | - | - |
| 7599 | ENSP00000312506 | CSPG4 | 3.965E-05 | -0.194 | - | - |
| 7600 | ENSP00000471607 | CTD-3105H18.18 | 9.114E-06 | -0.194 | - | - |
| 7601 | ENSP00000466387 | CTC-398G3.6 | 9.188E-06 | -0.194 | - | - |
| 7602 | ENSP00000287908 | STEAP2 | 4.146E-05 | -0.194 | - | - |
| 7603 | ENSP00000323199 | C18orf32 | 6.256E-04 | -0.194 | - | - |
| 7604 | ENSP00000471613 | CTD-3105H18.16 | 9.114E-06 | -0.194 | - | - |
| 7605 | ENSP00000318075 | PLEKHJ1 | 1.535E-04 | -0.194 | - | - |
| 7606 | ENSP00000264229 | KIAA1211 | 5.577E-04 | -0.194 | - | - |
| 7607 | ENSP00000340578 | RASSF6 | 4.584E-05 | -0.194 | - | - |
| 7608 | ENSP00000381857 | MROH6 | 1.183E-05 | -0.194 | - | - |
| 7609 | ENSP00000356590 | AXDND1 | 2.645E-07 | -0.194 | - | - |
| 7610 | ENSP00000310057 | FAM53A | 1.855E-04 | -0.194 | - | - |
| 7611 | ENSP00000339324 | SMIM15 | 2.524E-04 | -0.194 | - | - |
| 7612 | ENSP00000281282 | CGNL1 | 3.219E-05 | -0.194 | - | - |
| 7613 | ENSP00000347710 | OPHN1 | 2.340E-05 | -0.194 | - | - |
| 7614 | ENSP00000353864 | PAK3 | 2.328E-05 | -0.194 | - | - |
| 7615 | ENSP00000338641 | MUM1L1 | 2.105E-04 | -0.194 | - | - |
| 7616 | ENSP00000347211 | PRAMEF6 | 1.183E-05 | -0.194 | - | - |
| 7617 | ENSP00000375238 | KRTAP2-1 | 2.223E-05 | -0.194 | - | - |
| 7618 | ENSP00000298097 | FBXO33 | 6.253E-05 | -0.194 | - | - |
| 7619 | ENSP00000330408 | IST1 | 4.523E-05 | -0.194 | - | - |
| 7620 | ENSP00000321691 | PDDC1 | 1.582E-04 | -0.194 | - | - |
| 7621 | ENSP00000471593 | ZNF728 | 9.114E-06 | -0.194 | - | - |
| 7622 | ENSP00000472894 | ZNF547 | 9.114E-06 | -0.194 | - | - |
| 7623 | ENSP00000335041 | C2orf76 | 1.812E-04 | -0.194 | - | - |
| 7624 | ENSP00000441995 | AC073343.1 | 9.311E-06 | -0.194 | - | - |
| 7625 | ENSP00000347005 | C7orf55-LUC7L2 | 1.428E-05 | -0.194 | - | - |
| 7626 | ENSP00000299191 | C16orf78 | 8.048E-03 | -0.194 | - | - |
| 7627 | ENSP00000334849 | TMEM104 | 4.361E-05 | -0.194 | - | - |
| 7628 | ENSP00000375730 | RPL13A | 3.768E-05 | -0.194 | - | - |
| 7629 | ENSP00000341565 | FAM111B | 6.871E-05 | -0.194 | - | - |
| 7630 | ENSP00000342136 | CREB3 | 4.866E-05 | -0.194 | - | - |
| 7631 | ENSP00000336666 | AP1S1 | 3.439E-05 | -0.194 | - | - |
| 7632 | ENSP00000384018 | NCOR2 | 4.390E-05 | -0.194 | - | - |
| 7633 | ENSP00000319814 | PCK1 | 3.368E-05 | -0.194 | - | - |
| 7634 | ENSP00000303550 | CYTL1 | 2.688E-05 | -0.194 | - | - |
| 7635 | ENSP00000277458 | ASB6 | 7.176E-05 | -0.194 | - | - |
| 7636 | ENSP00000249344 | STRIP2 | 3.188E-05 | -0.194 | - | - |
| 7637 | ENSP00000280083 | CTAGE5 | 1.416E-04 | -0.194 | - | - |
| 7638 | ENSP00000301242 | PPP1R14A | 2.623E-05 | -0.194 | - | - |
| 7639 | ENSP00000284669 | KLHL41 | 3.457E-05 | -0.194 | - | - |
| 7640 | ENSP00000370616 | ZBED1 | 2.529E-05 | -0.194 | - | - |
| 7641 | ENSP00000219794 | BCKDK | 2.796E-05 | -0.194 | - | - |
| 7642 | ENSP00000382794 | C14orf164 | 3.198E-05 | -0.194 | - | - |
| 7643 | ENSP00000372959 | LRRC30 | 7.550E-06 | -0.194 | - | - |
| 7644 | ENSP00000242719 | RNF11 | 3.415E-05 | -0.194 | - | - |
| 7645 | ENSP00000301021 | TRAPPC2L | 4.162E-05 | -0.194 | - | - |
| 7646 | ENSP00000238831 | YIPF4 | 6.304E-05 | -0.194 | - | - |
| 7647 | ENSP00000352645 | TYW1 | 5.653E-05 | -0.194 | - | - |
| 7648 | ENSP00000346450 | H2AFB1 | 1.255E-05 | -0.194 | - | - |
| 7649 | ENSP00000342938 | UPK3BL | 3.831E-04 | -0.194 | - | - |
| 7650 | ENSP00000261813 | PFDN1 | 3.686E-05 | -0.194 | - | - |
| 7651 | ENSP00000240587 | TSHZ3 | 5.176E-05 | -0.194 | - | - |
| 7652 | ENSP00000332204 | SEMA4B | 3.832E-05 | -0.194 | - | - |
| 7653 | ENSP00000344546 | ARID1B | 3.056E-05 | -0.194 | - | - |
| 7654 | ENSP00000447931 | ZNF878 | 9.293E-06 | -0.194 | - | - |
| 7655 | ENSP00000298784 | FAM35A | 9.703E-05 | -0.194 | - | - |
| 7656 | ENSP00000321029 | EXD1 | 8.630E-05 | -0.194 | - | - |
| 7657 | ENSP00000458124 | CTD-2006C1.10 | 9.212E-06 | -0.194 | - | - |
| 7658 | ENSP00000241051 | DEPDC7 | 4.339E-05 | -0.194 | - | - |
| 7659 | ENSP00000279477 | SIRPB1 | 9.707E-05 | -0.194 | - | - |
| 7660 | ENSP00000383059 | ARGLU1 | 2.261E-05 | -0.194 | - | - |
| 7661 | ENSP00000312158 | BTN2A1 | 6.963E-05 | -0.194 | - | - |
| 7662 | ENSP00000337701 | PNPLA2 | 3.081E-05 | -0.194 | - | - |
| 7663 | ENSP00000294064 | NEU3 | 4.779E-05 | -0.194 | - | - |
| 7664 | ENSP00000264126 | GPSM2 | 2.705E-05 | -0.194 | - | - |
| 7665 | ENSP00000265462 | PRDX5 | 2.986E-05 | -0.194 | - | - |
| 7666 | ENSP00000291532 | TMPRSS3 | 2.765E-05 | -0.194 | - | - |
| 7667 | ENSP00000331845 | PRR14L | 8.878E-05 | -0.194 | - | - |
| 7668 | ENSP00000262709 | TOX4 | 3.599E-05 | -0.194 | - | - |
| 7669 | ENSP00000301776 | ASRGL1 | 3.706E-05 | -0.194 | - | - |
| 7670 | ENSP00000301046 | LALBA | 5.027E-05 | -0.194 | - | - |
| 7671 | ENSP00000319006 | CCDC71 | 6.584E-05 | -0.194 | - | - |
| 7672 | ENSP00000249700 | TMOD2 | 6.142E-05 | -0.194 | - | - |
| 7673 | ENSP00000368924 | TFAP2A | 3.846E-05 | -0.194 | - | - |
| 7674 | ENSP00000421652 | RP11-1396O13.13 | 9.568E-06 | -0.194 | - | - |
| 7675 | ENSP00000277746 | NRBF2 | 5.522E-05 | -0.194 | - | - |
| 7676 | ENSP00000330488 | CXorf38 | 3.930E-05 | -0.194 | - | - |
| 7677 | ENSP00000340668 | TMEM248 | 7.841E-05 | -0.194 | - | - |
| 7678 | ENSP00000302833 | GIMAP1 | 9.468E-05 | -0.194 | - | - |
| 7679 | ENSP00000348706 | HIST1H2BH | 3.118E-05 | -0.194 | - | - |
| 7680 | ENSP00000286031 | C1orf112 | 2.215E-04 | -0.194 | - | - |
| 7681 | ENSP00000473166 | CTD-2561J22.3 | 9.114E-06 | -0.194 | - | - |
| 7682 | ENSP00000251900 | SCML2 | 2.641E-05 | -0.194 | - | - |
| 7683 | ENSP00000296271 | RHO | 4.377E-05 | -0.194 | - | - |
| 7684 | ENSP00000305071 | RFXANK | 2.156E-05 | -0.194 | - | - |
| 7685 | ENSP00000270645 | RCN3 | 7.686E-05 | -0.194 | - | - |
| 7686 | ENSP00000296600 | ZNF474 | 3.542E-05 | -0.194 | - | - |
| 7687 | ENSP00000389709 | RNF212 | 1.890E-05 | -0.194 | - | - |
| 7688 | ENSP00000253063 | SESN2 | 3.316E-05 | -0.194 | - | - |
| 7689 | ENSP00000361433 | EXOSC2 | 1.185E-05 | -0.194 | - | - |
| 7690 | ENSP00000244926 | SCGB1D2 | 8.492E-05 | -0.194 | - | - |
| 7691 | ENSP00000362328 | KCNK17 | 2.559E-05 | -0.194 | - | - |
| 7692 | ENSP00000377170 | ANKRD39 | 3.766E-05 | -0.194 | - | - |
| 7693 | ENSP00000419057 | RP11-159G9.5 | 9.513E-06 | -0.194 | - | - |
| 7694 | ENSP00000339637 | BRAT1 | 8.495E-05 | -0.194 | - | - |
| 7695 | ENSP00000281243 | QDPR | 3.360E-05 | -0.194 | - | - |
| 7696 | ENSP00000292524 | LRRC14 | 1.217E-04 | -0.194 | - | - |
| 7697 | ENSP00000376410 | INF2 | 2.804E-05 | -0.194 | - | - |
| 7698 | ENSP00000255224 | SYT4 | 2.692E-05 | -0.194 | - | - |
| 7699 | ENSP00000260187 | USP2 | 2.709E-05 | -0.194 | - | - |
| 7700 | ENSP00000362063 | CSTF2 | 2.668E-05 | -0.194 | - | - |
| 7701 | ENSP00000353246 | MAK16 | 1.364E-05 | -0.194 | - | - |
| 7702 | ENSP00000355922 | CENPF | 3.732E-05 | -0.194 | - | - |
| 7703 | ENSP00000386869 | TET3 | 2.496E-05 | -0.194 | - | - |
| 7704 | ENSP00000299872 | NGFRAP1 | 5.476E-05 | -0.194 | - | - |
| 7705 | ENSP00000219168 | LYRM1 | 2.684E-05 | -0.194 | - | - |
| 7706 | ENSP00000228811 | PRR4 | 1.094E-04 | -0.194 | - | - |
| 7707 | ENSP00000309561 | C19orf70 | 7.093E-05 | -0.194 | - | - |
| 7708 | ENSP00000230658 | ISL1 | 3.043E-05 | -0.194 | - | - |
| 7709 | ENSP00000255631 | HSPBP1 | 2.750E-05 | -0.194 | - | - |
| 7710 | ENSP00000420854 | EFCAB12 | 3.760E-07 | -0.194 | - | - |
| 7711 | ENSP00000447987 | ZNF727 | 9.293E-06 | -0.194 | - | - |
| 7712 | ENSP00000318185 | LRRC42 | 8.093E-05 | -0.194 | - | - |
| 7713 | ENSP00000411132 | ZNF619 | 9.619E-06 | -0.194 | - | - |
| 7714 | ENSP00000338288 | RNASE11 | 1.342E-04 | -0.194 | - | - |
| 7715 | ENSP00000197268 | KIAA1467 | 8.977E-05 | -0.194 | - | - |
| 7716 | ENSP00000419397 | ZNF782 | 9.592E-06 | -0.194 | - | - |
| 7717 | ENSP00000297431 | ORC5 | 2.441E-05 | -0.194 | - | - |
| 7718 | ENSP00000303408 | HIST1H2BO | 2.102E-05 | -0.194 | - | - |
| 7719 | ENSP00000326706 | PLEKHO2 | 7.658E-05 | -0.194 | - | - |
| 7720 | ENSP00000472277 | ZNF548 | 9.114E-06 | -0.194 | - | - |
| 7721 | ENSP00000259199 | CBWD2 | 2.054E-05 | -0.194 | - | - |
| 7722 | ENSP00000268699 | GAS8 | 3.015E-05 | -0.194 | - | - |
| 7723 | ENSP00000231173 | PCDHB15 | 8.802E-05 | -0.194 | - | - |
| 7724 | ENSP00000269053 | SPACA3 | 5.608E-05 | -0.194 | - | - |
| 7725 | ENSP00000385597 | IQCE | 1.797E-05 | -0.194 | - | - |
| 7726 | ENSP00000358452 | H2AFB3 | 1.213E-05 | -0.194 | - | - |
| 7727 | ENSP00000261868 | EIF3J | 2.960E-05 | -0.194 | - | - |
| 7728 | ENSP00000252826 | TRPM4 | 2.670E-05 | -0.194 | - | - |
| 7729 | ENSP00000414887 | ZNF879 | 9.608E-06 | -0.194 | - | - |
| 7730 | ENSP00000312679 | TESPA1 | 6.340E-05 | -0.194 | - | - |
| 7731 | ENSP00000354453 | ZFP2 | 2.026E-05 | -0.194 | - | - |
| 7732 | ENSP00000239449 | PCDHB14 | 8.588E-05 | -0.194 | - | - |
| 7733 | ENSP00000284322 | ABI3BP | 5.645E-05 | -0.194 | - | - |
| 7734 | ENSP00000247977 | FBXL12 | 7.583E-05 | -0.194 | - | - |
| 7735 | ENSP00000300976 | KLHL26 | 6.861E-05 | -0.194 | - | - |
| 7736 | ENSP00000254043 | KRT15 | 2.129E-05 | -0.195 | - | - |
| 7737 | ENSP00000417335 | ROBO2 | 2.962E-05 | -0.195 | - | - |
| 7738 | ENSP00000334958 | RLTPR | 4.743E-05 | -0.195 | - | - |
| 7739 | ENSP00000296043 | SHROOM3 | 3.610E-05 | -0.195 | - | - |
| 7740 | ENSP00000309189 | SPERT | 3.982E-04 | -0.195 | - | - |
| 7741 | ENSP00000315568 | AVL9 | 3.349E-05 | -0.195 | - | - |
| 7742 | ENSP00000332449 | CRIP1 | 5.039E-05 | -0.195 | - | - |
| 7743 | ENSP00000205194 | NAT14 | 5.883E-05 | -0.195 | - | - |
| 7744 | ENSP00000323696 | CRTAP | 4.985E-05 | -0.195 | - | - |
| 7745 | ENSP00000249014 | CDC42EP1 | 1.281E-04 | -0.195 | - | - |
| 7746 | ENSP00000263256 | DESI1 | 2.064E-04 | -0.195 | - | - |
| 7747 | ENSP00000316029 | TLN1 | 3.989E-05 | -0.195 | - | - |
| 7748 | ENSP00000293883 | WDR24 | 1.864E-05 | -0.195 | - | - |
| 7749 | ENSP00000376792 | VTI1A | 5.614E-06 | -0.195 | - | - |
| 7750 | ENSP00000465960 | ZNF585B | 9.205E-06 | -0.195 | - | - |
| 7751 | ENSP00000270792 | SH3BGRL3 | 2.274E-05 | -0.195 | - | - |
| 7752 | ENSP00000321674 | 4-Sep | 5.615E-05 | -0.195 | - | - |
| 7753 | ENSP00000460441 | 1-Sep | 3.260E-05 | -0.195 | - | - |
| 7754 | ENSP00000287859 | C1orf27 | 6.986E-05 | -0.195 | - | - |
| 7755 | ENSP00000308727 | SUSD5 | 3.277E-05 | -0.195 | - | - |
| 7756 | ENSP00000382030 | RAD51AP2 | 5.584E-05 | -0.195 | - | - |
| 7757 | ENSP00000288087 | MDP1 | 1.410E-04 | -0.195 | - | - |
| 7758 | ENSP00000360157 | FOXD3 | 2.767E-05 | -0.195 | - | - |
| 7759 | ENSP00000302077 | SLFN12 | 1.550E-04 | -0.195 | - | - |
| 7760 | ENSP00000322956 | MFAP3 | 6.951E-05 | -0.195 | - | - |
| 7761 | ENSP00000258770 | TBRG4 | 5.107E-05 | -0.195 | - | - |
| 7762 | ENSP00000310117 | PPP1R14B | 5.738E-05 | -0.195 | - | - |
| 7763 | ENSP00000297205 | STEAP1 | 7.550E-05 | -0.195 | - | - |
| 7764 | ENSP00000264834 | KLF1 | 4.105E-04 | -0.195 | - | - |
| 7765 | ENSP00000371607 | MIPEP | 2.769E-05 | -0.195 | - | - |
| 7766 | ENSP00000301396 | PELP1 | 3.980E-05 | -0.195 | - | - |
| 7767 | ENSP00000340761 | BRI3BP | 6.073E-05 | -0.195 | - | - |
| 7768 | ENSP00000427514 | CEBPA | 8.651E-05 | -0.195 | - | - |
| 7769 | ENSP00000268595 | CMTM2 | 3.055E-05 | -0.195 | - | - |
| 7770 | ENSP00000312402 | SLFN11 | 7.020E-05 | -0.195 | - | - |
| 7771 | ENSP00000262293 | PRR11 | 2.230E-05 | -0.195 | - | - |
| 7772 | ENSP00000354481 | FAM5B | 3.319E-05 | -0.195 | - | - |
| 7773 | ENSP00000380552 | TSPAN4 | 5.005E-05 | -0.195 | - | - |
| 7774 | ENSP00000276585 | MRPS28 | 4.197E-05 | -0.195 | - | - |
| 7775 | ENSP00000305161 | TRIM56 | 5.118E-05 | -0.195 | - | - |
| 7776 | ENSP00000295962 | ABHD6 | 2.790E-05 | -0.195 | - | - |
| 7777 | ENSP00000270061 | SSBP4 | 3.260E-05 | -0.195 | - | - |
| 7778 | ENSP00000309230 | PEAK1 | 5.660E-05 | -0.195 | - | - |
| 7779 | ENSP00000278426 | SLC43A1 | 7.622E-05 | -0.195 | - | - |
| 7780 | ENSP00000251312 | DHRS11 | 2.341E-05 | -0.195 | - | - |
| 7781 | ENSP00000460571 | RP1-4G17.5 | 5.086E-06 | -0.195 | - | - |
| 7782 | ENSP00000339532 | THUMPD3 | 3.347E-05 | -0.195 | - | - |
| 7783 | ENSP00000266025 | TMEM115 | 1.872E-04 | -0.195 | - | - |
| 7784 | ENSP00000360672 | PARD6B | 2.404E-05 | -0.195 | - | - |
| 7785 | ENSP00000176195 | SCT | 4.332E-05 | -0.195 | - | - |
| 7786 | ENSP00000222968 | PDAP1 | 5.609E-05 | -0.195 | - | - |
| 7787 | ENSP00000314407 | CA8 | 2.928E-05 | -0.195 | - | - |
| 7788 | ENSP00000304903 | CD2BP2 | 3.020E-05 | -0.195 | - | - |
| 7789 | ENSP00000312070 | PCDHGC3 | 4.916E-05 | -0.195 | - | - |
| 7790 | ENSP00000217195 | C20orf27 | 3.992E-05 | -0.195 | - | - |
| 7791 | ENSP00000342499 | TSTD2 | 9.492E-05 | -0.195 | - | - |
| 7792 | ENSP00000283303 | GPR115 | 5.447E-05 | -0.195 | - | - |
| 7793 | ENSP00000219315 | SETD6 | 5.139E-05 | -0.195 | - | - |
| 7794 | ENSP00000362300 | AGO1 | 3.842E-05 | -0.195 | - | - |
| 7795 | ENSP00000253413 | ATP6V1E1 | 2.155E-05 | -0.195 | - | - |
| 7796 | ENSP00000302936 | DYNLRB2 | 2.858E-05 | -0.195 | - | - |
| 7797 | ENSP00000371897 | ARHGAP5 | 2.412E-05 | -0.195 | - | - |
| 7798 | ENSP00000264866 | TBC1D19 | 9.325E-05 | -0.195 | - | - |
| 7799 | ENSP00000409652 | ZNF766 | 9.641E-06 | -0.195 | - | - |
| 7800 | ENSP00000271883 | GON4L | 2.824E-05 | -0.195 | - | - |
| 7801 | ENSP00000328216 | ORAI1 | 3.330E-05 | -0.195 | - | - |
| 7802 | ENSP00000156626 | ST6GALNAC1 | 2.234E-05 | -0.195 | - | - |
| 7803 | ENSP00000303987 | TMEM223 | 8.239E-05 | -0.195 | - | - |
| 7804 | ENSP00000407509 | PNPLA6 | 2.628E-04 | -0.195 | - | - |
| 7805 | ENSP00000343847 | NEK10 | 3.600E-05 | -0.195 | - | - |
| 7806 | ENSP00000278071 | ITPRIP | 3.555E-05 | -0.195 | - | - |
| 7807 | ENSP00000307321 | HOXC10 | 3.127E-05 | -0.195 | - | - |
| 7808 | ENSP00000317175 | PLEKHD1 | 6.143E-05 | -0.195 | - | - |
| 7809 | ENSP00000265342 | FSTL4 | 7.272E-05 | -0.195 | - | - |
| 7810 | ENSP00000241436 | POLK | 2.949E-05 | -0.195 | - | - |
| 7811 | ENSP00000330060 | CNOT10 | 4.703E-05 | -0.195 | - | - |
| 7812 | ENSP00000289805 | SPATA2L | 5.195E-05 | -0.195 | - | - |
| 7813 | ENSP00000311469 | GSTM1 | 8.355E-05 | -0.195 | - | - |
| 7814 | ENSP00000339529 | POP1 | 1.887E-05 | -0.195 | - | - |
| 7815 | ENSP00000300087 | DCTN5 | 3.783E-05 | -0.195 | - | - |
| 7816 | ENSP00000304360 | SMYD4 | 8.098E-06 | -0.195 | - | - |
| 7817 | ENSP00000254950 | VPS4A | 3.517E-05 | -0.195 | - | - |
| 7818 | ENSP00000451575 | ASB2 | 2.111E-04 | -0.195 | - | - |
| 7819 | ENSP00000222005 | CDC37 | 2.732E-04 | -0.195 | - | - |
| 7820 | ENSP00000316053 | DNAJB8 | 3.520E-05 | -0.195 | - | - |
| 7821 | ENSP00000301939 | TMEM256 | 6.432E-05 | -0.195 | - | - |
| 7822 | ENSP00000290974 | ZFYVE28 | 3.802E-05 | -0.195 | - | - |
| 7823 | ENSP00000216446 | PLEK2 | 7.981E-05 | -0.195 | - | - |
| 7824 | ENSP00000260605 | DYNC2LI1 | 3.136E-05 | -0.195 | - | - |
| 7825 | ENSP00000206544 | SLC22A17 | 4.806E-05 | -0.195 | - | - |
| 7826 | ENSP00000369162 | RIOK1 | 1.539E-05 | -0.195 | - | - |
| 7827 | ENSP00000313046 | BAI1 | 5.494E-05 | -0.195 | - | - |
| 7828 | ENSP00000338706 | MID1IP1 | 5.437E-05 | -0.195 | - | - |
| 7829 | ENSP00000301717 | SPSB3 | 5.887E-05 | -0.195 | - | - |
| 7830 | ENSP00000254816 | TRIM47 | 9.529E-05 | -0.195 | - | - |
| 7831 | ENSP00000386773 | AC013269.5 | 6.695E-06 | -0.195 | - | - |
| 7832 | ENSP00000355279 | CNOT7 | 1.652E-05 | -0.195 | - | - |
| 7833 | ENSP00000255174 | C20orf111 | 1.261E-04 | -0.195 | - | - |
| 7834 | ENSP00000343990 | TRIM14 | 5.600E-05 | -0.195 | - | - |
| 7835 | ENSP00000272895 | ABCA12 | 2.767E-05 | -0.195 | - | - |
| 7836 | ENSP00000261250 | C12orf4 | 1.044E-04 | -0.195 | - | - |
| 7837 | ENSP00000270502 | C19orf52 | 7.339E-05 | -0.195 | - | - |
| 7838 | ENSP00000283206 | TMEM87B | 2.749E-05 | -0.195 | - | - |
| 7839 | ENSP00000231134 | PCDHB5 | 5.905E-05 | -0.195 | - | - |
| 7840 | ENSP00000327075 | CD300LF | 2.964E-05 | -0.195 | - | - |
| 7841 | ENSP00000166534 | P4HA2 | 3.101E-05 | -0.195 | - | - |
| 7842 | ENSP00000348769 | ARIH2 | 1.560E-05 | -0.195 | - | - |
| 7843 | ENSP00000265565 | SCAP | 3.075E-05 | -0.195 | - | - |
| 7844 | ENSP00000246802 | GLTSCR2 | 2.252E-05 | -0.195 | - | - |
| 7845 | ENSP00000344801 | SLC24A2 | 1.917E-05 | -0.195 | - | - |
| 7846 | ENSP00000329360 | CCDC137 | 5.290E-05 | -0.195 | - | - |
| 7847 | ENSP00000341324 | SRFBP1 | 6.343E-05 | -0.195 | - | - |
| 7848 | ENSP00000313226 | CTXN1 | 8.967E-05 | -0.195 | - | - |
| 7849 | ENSP00000332549 | GRIN2A | 4.022E-05 | -0.195 | - | - |
| 7850 | ENSP00000310088 | PTMS | 3.088E-05 | -0.195 | - | - |
| 7851 | ENSP00000264108 | HAT1 | 2.361E-05 | -0.195 | - | - |
| 7852 | ENSP00000370891 | STK35 | 2.522E-05 | -0.195 | - | - |
| 7853 | ENSP00000260372 | HAUS2 | 2.962E-05 | -0.195 | - | - |
| 7854 | ENSP00000267012 | BIN2 | 7.184E-05 | -0.195 | - | - |
| 7855 | ENSP00000276603 | TERF1 | 6.473E-05 | -0.195 | - | - |
| 7856 | ENSP00000388920 | RWDD4 | 1.946E-05 | -0.195 | - | - |
| 7857 | ENSP00000368511 | CXorf58 | 2.603E-05 | -0.195 | - | - |
| 7858 | ENSP00000338190 | - | 4.248E-05 | -0.195 | - | - |
| 7859 | ENSP00000292090 | TLCD1 | 8.964E-05 | -0.195 | - | - |
| 7860 | ENSP00000329920 | C19orf59 | 2.860E-05 | -0.195 | - | - |
| 7861 | ENSP00000316681 | KIAA1731 | 8.527E-05 | -0.195 | - | - |
| 7862 | ENSP00000383820 | MIER1 | 2.659E-05 | -0.195 | - | - |
| 7863 | ENSP00000324404 | TCHP | 5.066E-05 | -0.195 | - | - |
| 7864 | ENSP00000251166 | CORO7 | 6.143E-05 | -0.195 | - | - |
| 7865 | ENSP00000384880 | FAM49B | 2.456E-05 | -0.195 | - | - |
| 7866 | ENSP00000331258 | SGK196 | 3.903E-05 | -0.195 | - | - |
| 7867 | ENSP00000275034 | PHIP | 4.473E-05 | -0.195 | - | - |
| 7868 | ENSP00000286067 | C10orf12 | 1.433E-04 | -0.195 | - | - |
| 7869 | ENSP00000272425 | NAT8 | 1.700E-05 | -0.195 | - | - |
| 7870 | ENSP00000282884 | RASSF8 | 5.949E-05 | -0.195 | - | - |
| 7871 | ENSP00000233712 | EVA1A | 7.746E-05 | -0.195 | - | - |
| 7872 | ENSP00000272902 | SUMF1 | 2.460E-05 | -0.195 | - | - |
| 7873 | ENSP00000348901 | PLD3 | 7.459E-06 | -0.195 | - | - |
| 7874 | ENSP00000310861 | KRT2 | 3.600E-05 | -0.195 | - | - |
| 7875 | ENSP00000304544 | COMMD5 | 5.499E-05 | -0.195 | - | - |
| 7876 | ENSP00000274458 | COMMD10 | 6.210E-05 | -0.195 | - | - |
| 7877 | ENSP00000338742 | SULT1A2 | 3.540E-05 | -0.195 | - | - |
| 7878 | ENSP00000242784 | C19orf43 | 8.134E-05 | -0.195 | - | - |
| 7879 | ENSP00000265978 | FAM160A2 | 9.208E-05 | -0.195 | - | - |
| 7880 | ENSP00000316782 | NBPF3 | 4.330E-05 | -0.195 | - | - |
| 7881 | ENSP00000261439 | TBC1D1 | 2.836E-05 | -0.195 | - | - |
| 7882 | ENSP00000455300 | RP1-27O5.3 | 9.197E-06 | -0.195 | - | - |
| 7883 | ENSP00000359506 | FMR1 | 1.968E-05 | -0.195 | - | - |
| 7884 | ENSP00000339353 | CPSF1 | 2.796E-05 | -0.195 | - | - |
| 7885 | ENSP00000254742 | TMEM128 | 1.061E-04 | -0.195 | - | - |
| 7886 | ENSP00000313759 | CTC1 | 6.820E-05 | -0.195 | - | - |
| 7887 | ENSP00000362335 | SAR1A | 2.106E-05 | -0.195 | - | - |
| 7888 | ENSP00000194152 | PCDHB4 | 6.370E-05 | -0.195 | - | - |
| 7889 | ENSP00000221114 | DCTN6 | 3.429E-05 | -0.195 | - | - |
| 7890 | ENSP00000365160 | NUDT11 | 4.730E-05 | -0.195 | - | - |
| 7891 | ENSP00000268717 | COPS3 | 3.959E-05 | -0.195 | - | - |
| 7892 | ENSP00000058691 | HEBP2 | 5.620E-05 | -0.195 | - | - |
| 7893 | ENSP00000263681 | POLD3 | 3.642E-05 | -0.195 | - | - |
| 7894 | ENSP00000216038 | C22orf28 | 3.938E-05 | -0.196 | - | - |
| 7895 | ENSP00000344655 | CCDC41 | 5.302E-05 | -0.196 | - | - |
| 7896 | ENSP00000298375 | AKR1E2 | 2.349E-05 | -0.196 | - | - |
| 7897 | ENSP00000223336 | COA1 | 1.304E-04 | -0.196 | - | - |
| 7898 | ENSP00000372272 | KRTAP19-8 | 3.748E-07 | -0.196 | - | - |
| 7899 | ENSP00000354742 | IPO9 | 2.849E-05 | -0.196 | - | - |
| 7900 | ENSP00000339626 | SLC35E4 | 2.287E-05 | -0.196 | - | - |
| 7901 | ENSP00000322469 | C3orf38 | 1.378E-04 | -0.196 | - | - |
| 7902 | ENSP00000248121 | SYNGR3 | 4.396E-05 | -0.196 | - | - |
| 7903 | ENSP00000348838 | UBE2C | 1.842E-05 | -0.196 | - | - |
| 7904 | ENSP00000267615 | ITPK1 | 4.385E-05 | -0.196 | - | - |
| 7905 | ENSP00000370447 | VCX | 2.356E-06 | -0.196 | - | - |
| 7906 | ENSP00000231130 | PCDHB3 | 6.867E-05 | -0.196 | - | - |
| 7907 | ENSP00000262809 | ELL | 3.420E-04 | -0.196 | - | - |
| 7908 | ENSP00000311360 | RAD9A | 2.435E-05 | -0.196 | - | - |
| 7909 | ENSP00000339835 | H3F3C | 2.306E-05 | -0.196 | - | - |
| 7910 | ENSP00000253461 | C16orf95 | 6.773E-05 | -0.196 | - | - |
| 7911 | ENSP00000315630 | SWAP70 | 5.107E-05 | -0.196 | - | - |
| 7912 | ENSP00000297613 | RPP25L | 5.898E-05 | -0.196 | - | - |
| 7913 | ENSP00000341743 | ENSA | 2.947E-05 | -0.196 | - | - |
| 7914 | ENSP00000320359 | RABL5 | 8.512E-05 | -0.196 | - | - |
| 7915 | ENSP00000368079 | CYP4V2 | 2.515E-05 | -0.196 | - | - |
| 7916 | ENSP00000262102 | MTUS1 | 4.055E-05 | -0.196 | - | - |
| 7917 | ENSP00000258083 | PRADC1 | 8.008E-05 | -0.196 | - | - |
| 7918 | ENSP00000376611 | TDG | 2.832E-05 | -0.196 | - | - |
| 7919 | ENSP00000294507 | LAPTM5 | 4.275E-05 | -0.196 | - | - |
| 7920 | ENSP00000328036 | DGAT2L6 | 3.320E-05 | -0.196 | - | - |
| 7921 | ENSP00000222374 | CADM4 | 3.936E-05 | -0.196 | - | - |
| 7922 | ENSP00000308820 | RIC3 | 4.332E-05 | -0.196 | - | - |
| 7923 | ENSP00000349428 | PTBP1 | 3.130E-05 | -0.196 | - | - |
| 7924 | ENSP00000267622 | TRIP11 | 5.158E-05 | -0.196 | - | - |
| 7925 | ENSP00000352438 | PCBP2 | 2.116E-05 | -0.196 | - | - |
| 7926 | ENSP00000257901 | KRT85 | 3.186E-05 | -0.196 | - | - |
| 7927 | ENSP00000288071 | DDX19B | 2.243E-05 | -0.196 | - | - |
| 7928 | ENSP00000326095 | RNF166 | 3.325E-05 | -0.196 | - | - |
| 7929 | ENSP00000261712 | PSMD11 | 2.728E-05 | -0.196 | - | - |
| 7930 | ENSP00000216420 | CGRRF1 | 6.989E-05 | -0.196 | - | - |
| 7931 | ENSP00000338013 | KIAA1984 | 3.500E-05 | -0.196 | - | - |
| 7932 | ENSP00000268138 | TICRR | 5.358E-05 | -0.196 | - | - |
| 7933 | ENSP00000301678 | ITFG3 | 7.302E-05 | -0.196 | - | - |
| 7934 | ENSP00000276704 | C8orf76 | 6.959E-05 | -0.196 | - | - |
| 7935 | ENSP00000417257 | FNDC3A | 3.911E-04 | -0.196 | - | - |
| 7936 | ENSP00000011619 | RANBP9 | 3.207E-05 | -0.196 | - | - |
| 7937 | ENSP00000337313 | ZCCHC8 | 2.703E-05 | -0.196 | - | - |
| 7938 | ENSP00000354346 | MATR3 | 2.028E-05 | -0.196 | - | - |
| 7939 | ENSP00000256151 | CCDC59 | 5.635E-05 | -0.196 | - | - |
| 7940 | ENSP00000299977 | SLFN5 | 5.528E-05 | -0.196 | - | - |
| 7941 | ENSP00000302397 | ATP1A3 | 2.165E-05 | -0.196 | - | - |
| 7942 | ENSP00000206474 | HAUS4 | 5.602E-05 | -0.196 | - | - |
| 7943 | ENSP00000313670 | ALS2CL | 3.917E-05 | -0.196 | - | - |
| 7944 | ENSP00000365465 | CDSN | 6.440E-06 | -0.196 | - | - |
| 7945 | ENSP00000299198 | CKB | 3.107E-05 | -0.196 | - | - |
| 7946 | ENSP00000301420 | KLK1 | 3.619E-05 | -0.196 | - | - |
| 7947 | ENSP00000369018 | GPR56 | 2.557E-05 | -0.196 | - | - |
| 7948 | ENSP00000290349 | CBR1 | 2.656E-05 | -0.196 | - | - |
| 7949 | ENSP00000307014 | KRT73 | 3.845E-05 | -0.196 | - | - |
| 7950 | ENSP00000314971 | TAF1D | 4.388E-05 | -0.196 | - | - |
| 7951 | ENSP00000248958 | SDF2L1 | 3.923E-05 | -0.196 | - | - |
| 7952 | ENSP00000263713 | EPB41L5 | 4.730E-05 | -0.196 | - | - |
| 7953 | ENSP00000230431 | DNPH1 | 2.227E-05 | -0.196 | - | - |
| 7954 | ENSP00000322579 | PHF23 | 4.335E-05 | -0.196 | - | - |
| 7955 | ENSP00000309233 | POMZP3 | 2.953E-04 | -0.196 | - | - |
| 7956 | ENSP00000251547 | FBXO44 | 3.031E-05 | -0.196 | - | - |
| 7957 | ENSP00000247815 | HELB | 2.736E-05 | -0.196 | - | - |
| 7958 | ENSP00000381992 | 7-Sep | 3.234E-05 | -0.196 | - | - |
| 7959 | ENSP00000387875 | KIF4B | 3.029E-05 | -0.196 | - | - |
| 7960 | ENSP00000244519 | BTN3A3 | 4.780E-05 | -0.196 | - | - |
| 7961 | ENSP00000265036 | DEPDC1B | 2.593E-05 | -0.196 | - | - |
| 7962 | ENSP00000246635 | KRT13 | 2.724E-05 | -0.196 | - | - |
| 7963 | ENSP00000386143 | HEPN1 | 3.382E-06 | -0.196 | - | - |
| 7964 | ENSP00000329102 | LIN9 | 3.395E-05 | -0.196 | - | - |
| 7965 | ENSP00000224862 | FBXL15 | 4.012E-05 | -0.196 | - | - |
| 7966 | ENSP00000266682 | SLC6A15 | 3.013E-05 | -0.196 | - | - |
| 7967 | ENSP00000184956 | HEATR6 | 7.220E-05 | -0.196 | - | - |
| 7968 | ENSP00000169551 | TIMM21 | 4.048E-05 | -0.196 | - | - |
| 7969 | ENSP00000304669 | CTNNA1 | 3.582E-05 | -0.196 | - | - |
| 7970 | ENSP00000371472 | NIN | 2.359E-05 | -0.196 | - | - |
| 7971 | ENSP00000463741 | ZNF19 | 1.073E-05 | -0.196 | - | - |
| 7972 | ENSP00000325414 | SERTAD3 | 1.184E-04 | -0.196 | - | - |
| 7973 | ENSP00000411032 | ZNF284 | 9.588E-06 | -0.196 | - | - |
| 7974 | ENSP00000265295 | SPDL1 | 3.072E-05 | -0.196 | - | - |
| 7975 | ENSP00000244217 | MCEE | 2.502E-05 | -0.196 | - | - |
| 7976 | ENSP00000397002 | TEX22 | 4.961E-06 | -0.196 | - | - |
| 7977 | ENSP00000330753 | BRWD1 | 3.038E-05 | -0.196 | - | - |
| 7978 | ENSP00000295500 | GPR155 | 2.108E-05 | -0.196 | - | - |
| 7979 | ENSP00000249910 | MBD4 | 3.224E-05 | -0.196 | - | - |
| 7980 | ENSP00000288014 | THTPA | 3.759E-05 | -0.196 | - | - |
| 7981 | ENSP00000325681 | CCZ1 | 3.861E-05 | -0.196 | - | - |
| 7982 | ENSP00000292852 | FBXO17 | 2.793E-05 | -0.196 | - | - |
| 7983 | ENSP00000341280 | JARID2 | 2.191E-05 | -0.196 | - | - |
| 7984 | ENSP00000296292 | RFT1 | 7.022E-05 | -0.196 | - | - |
| 7985 | ENSP00000347232 | BLM | 2.717E-05 | -0.196 | - | - |
| 7986 | ENSP00000327487 | TSEN54 | 3.604E-05 | -0.196 | - | - |
| 7987 | ENSP00000256186 | MICALCL | 6.416E-05 | -0.196 | - | - |
| 7988 | ENSP00000252771 | FCHO1 | 2.267E-05 | -0.196 | - | - |
| 7989 | ENSP00000333193 | GJC1 | 3.575E-05 | -0.196 | - | - |
| 7990 | ENSP00000245541 | GGA3 | 1.960E-05 | -0.196 | - | - |
| 7991 | ENSP00000298894 | MOAP1 | 5.970E-05 | -0.196 | - | - |
| 7992 | ENSP00000248901 | CYTH4 | 2.221E-05 | -0.196 | - | - |
| 7993 | ENSP00000265806 | R3HCC1 | 5.553E-05 | -0.196 | - | - |
| 7994 | ENSP00000296603 | LMBRD2 | 5.474E-05 | -0.196 | - | - |
| 7995 | ENSP00000308921 | ZCCHC12 | 4.818E-05 | -0.196 | - | - |
| 7996 | ENSP00000321544 | KBTBD11 | 4.373E-05 | -0.196 | - | - |
| 7997 | ENSP00000338348 | UBE2G2 | 5.393E-05 | -0.196 | - | - |
| 7998 | ENSP00000274030 | USP53 | 4.570E-05 | -0.196 | - | - |
| 7999 | ENSP00000302586 | KBTBD2 | 4.052E-05 | -0.196 | - | - |
| 8000 | ENSP00000309336 | HOXC5 | 2.883E-05 | -0.196 | - | - |
| 8001 | ENSP00000244661 | HIST1H3B | 1.861E-05 | -0.196 | - | - |
| 8002 | ENSP00000376472 | STT3A | 2.897E-05 | -0.196 | - | - |
| 8003 | ENSP00000358799 | RBM15 | 2.833E-05 | -0.196 | - | - |
| 8004 | ENSP00000229251 | COPS7A | 2.902E-05 | -0.196 | - | - |
| 8005 | ENSP00000337991 | SPTY2D1 | 3.319E-05 | -0.196 | - | - |
| 8006 | ENSP00000267935 | COMMD4 | 5.144E-05 | -0.196 | - | - |
| 8007 | ENSP00000343273 | KLHL7 | 2.901E-05 | -0.196 | - | - |
| 8008 | ENSP00000340703 | SPRR2B | 2.561E-05 | -0.196 | - | - |
| 8009 | ENSP00000217455 | ACOT8 | 2.000E-05 | -0.196 | - | - |
| 8010 | ENSP00000312017 | FAM57A | 3.901E-05 | -0.196 | - | - |
| 8011 | ENSP00000341957 | TP53BP2 | 4.714E-05 | -0.196 | - | - |
| 8012 | ENSP00000245564 | MSTO1 | 3.314E-05 | -0.196 | - | - |
| 8013 | ENSP00000341543 | METTL23 | 3.888E-05 | -0.196 | - | - |
| 8014 | ENSP00000321330 | RNF144A | 3.171E-05 | -0.196 | - | - |
| 8015 | ENSP00000373191 | LNP1 | 1.129E-05 | -0.196 | - | - |
| 8016 | ENSP00000351100 | WDR55 | 1.404E-05 | -0.196 | - | - |
| 8017 | ENSP00000276654 | LRP12 | 5.221E-05 | -0.196 | - | - |
| 8018 | ENSP00000381785 | HSDL2 | 3.330E-05 | -0.196 | - | - |
| 8019 | ENSP00000260118 | GGH | 3.060E-05 | -0.196 | - | - |
| 8020 | ENSP00000344609 | BTG3 | 3.340E-05 | -0.196 | - | - |
| 8021 | ENSP00000299413 | TRIM44 | 4.037E-05 | -0.196 | - | - |
| 8022 | ENSP00000320176 | HCLS1 | 2.851E-05 | -0.196 | - | - |
| 8023 | ENSP00000345599 | SCXA | 6.461E-05 | -0.196 | - | - |
| 8024 | ENSP00000263663 | TAF1B | 5.878E-05 | -0.196 | - | - |
| 8025 | ENSP00000316465 | C18orf56 | 1.319E-05 | -0.196 | - | - |
| 8026 | ENSP00000320251 | C3orf17 | 4.044E-05 | -0.196 | - | - |
| 8027 | ENSP00000302647 | RALGAPA1 | 2.795E-05 | -0.196 | - | - |
| 8028 | ENSP00000221922 | CCDC9 | 4.494E-05 | -0.196 | - | - |
| 8029 | ENSP00000259477 | ARPC5L | 2.808E-05 | -0.196 | - | - |
| 8030 | ENSP00000365946 | RBM3 | 1.456E-05 | -0.196 | - | - |
| 8031 | ENSP00000305892 | TMEM208 | 5.525E-05 | -0.196 | - | - |
| 8032 | ENSP00000265807 | SH2D4A | 4.262E-05 | -0.196 | - | - |
| 8033 | ENSP00000364739 | XAGE1C | 1.998E-06 | -0.196 | - | - |
| 8034 | ENSP00000350170 | FXR1 | 2.281E-05 | -0.196 | - | - |
| 8035 | ENSP00000229633 | HINT3 | 9.241E-05 | -0.196 | - | - |
| 8036 | ENSP00000342667 | RNF19A | 5.458E-05 | -0.196 | - | - |
| 8037 | ENSP00000315693 | PAM16 | 2.878E-05 | -0.196 | - | - |
| 8038 | ENSP00000337773 | NQO2 | 2.420E-05 | -0.196 | - | - |
| 8039 | ENSP00000338990 | C12orf44 | 2.687E-05 | -0.196 | - | - |
| 8040 | ENSP00000366365 | OR14J1 | 1.684E-06 | -0.196 | - | - |
| 8041 | ENSP00000365198 | KAZN | 3.007E-05 | -0.196 | - | - |
| 8042 | ENSP00000263401 | COMMD9 | 6.911E-05 | -0.196 | - | - |
| 8043 | ENSP00000358217 | PRDM13 | 1.401E-05 | -0.196 | - | - |
| 8044 | ENSP00000344254 | FXYD5 | 2.790E-05 | -0.196 | - | - |
| 8045 | ENSP00000302562 | HSPBAP1 | 3.958E-05 | -0.196 | - | - |
| 8046 | ENSP00000296129 | CDCP1 | 3.488E-05 | -0.196 | - | - |
| 8047 | ENSP00000313422 | ARL6IP4 | 6.902E-05 | -0.196 | - | - |
| 8048 | ENSP00000362306 | AGO4 | 1.948E-05 | -0.196 | - | - |
| 8049 | ENSP00000261778 | TANGO6 | 3.904E-05 | -0.196 | - | - |
| 8050 | ENSP00000346050 | RPS3A | 3.437E-05 | -0.196 | - | - |
| 8051 | ENSP00000281871 | MZT2B | 5.319E-05 | -0.196 | - | - |
| 8052 | ENSP00000283027 | NUBP1 | 2.298E-05 | -0.196 | - | - |
| 8053 | ENSP00000261888 | PARP16 | 5.084E-05 | -0.196 | - | - |
| 8054 | ENSP00000324810 | MVB12A | 2.937E-05 | -0.196 | - | - |
| 8055 | ENSP00000380921 | SH3KBP1 | 2.817E-05 | -0.196 | - | - |
| 8056 | ENSP00000306396 | RNF187 | 5.378E-05 | -0.196 | - | - |
| 8057 | ENSP00000280800 | PLBD2 | 4.317E-05 | -0.196 | - | - |
| 8058 | ENSP00000359321 | MTF2 | 2.220E-05 | -0.196 | - | - |
| 8059 | ENSP00000300605 | HDHD2 | 3.103E-05 | -0.196 | - | - |
| 8060 | ENSP00000311500 | MZT2A | 4.708E-05 | -0.197 | - | - |
| 8061 | ENSP00000362047 | XKRX | 1.774E-05 | -0.197 | - | - |
| 8062 | ENSP00000290039 | CACHD1 | 2.748E-05 | -0.197 | - | - |
| 8063 | ENSP00000337044 | C5orf24 | 3.388E-05 | -0.197 | - | - |
| 8064 | ENSP00000359483 | LRRC8C | 8.743E-06 | -0.197 | - | - |
| 8065 | ENSP00000251289 | WDR18 | 2.986E-05 | -0.197 | - | - |
| 8066 | ENSP00000293925 | CRAMP1L | 5.676E-05 | -0.197 | - | - |
| 8067 | ENSP00000256460 | CAMK1 | 1.833E-05 | -0.197 | - | - |
| 8068 | ENSP00000325612 | MED16 | 2.947E-05 | -0.197 | - | - |
| 8069 | ENSP00000203630 | MLF2 | 3.184E-05 | -0.197 | - | - |
| 8070 | ENSP00000257548 | USP30 | 2.409E-05 | -0.197 | - | - |
| 8071 | ENSP00000322238 | C14orf119 | 4.102E-05 | -0.197 | - | - |
| 8072 | ENSP00000266643 | 9-Mar | 2.666E-05 | -0.197 | - | - |
| 8073 | ENSP00000295321 | IWS1 | 3.253E-05 | -0.197 | - | - |
| 8074 | ENSP00000355094 | QKI | 1.736E-05 | -0.197 | - | - |
| 8075 | ENSP00000256538 | ATPBD4 | 5.294E-05 | -0.197 | - | - |
| 8076 | ENSP00000471898 | AC099780.1 | 3.344E-06 | -0.197 | - | - |
| 8077 | ENSP00000361066 | NCOA3 | 3.144E-05 | -0.197 | - | - |
| 8078 | ENSP00000335595 | TCP11L1 | 4.793E-05 | -0.197 | - | - |
| 8079 | ENSP00000194155 | PCDHB2 | 5.838E-05 | -0.197 | - | - |
| 8080 | ENSP00000338838 | CHID1 | 4.970E-05 | -0.197 | - | - |
| 8081 | ENSP00000295727 | FEV | 2.572E-05 | -0.197 | - | - |
| 8082 | ENSP00000305769 | SMAD1 | 6.481E-05 | -0.197 | - | - |
| 8083 | ENSP00000340969 | TEX264 | 3.689E-05 | -0.197 | - | - |
| 8084 | ENSP00000319412 | ARMC10 | 5.826E-05 | -0.197 | - | - |
| 8085 | ENSP00000216602 | ZFYVE21 | 5.522E-05 | -0.197 | - | - |
| 8086 | ENSP00000278937 | MPZL2 | 3.314E-05 | -0.197 | - | - |
| 8087 | ENSP00000342604 | FAM184A | 4.327E-05 | -0.197 | - | - |
| 8088 | ENSP00000332757 | FUT10 | 2.765E-05 | -0.197 | - | - |
| 8089 | ENSP00000362556 | ZBTB43 | 5.117E-05 | -0.197 | - | - |
| 8090 | ENSP00000261721 | BTBD1 | 3.655E-05 | -0.197 | - | - |
| 8091 | ENSP00000356433 | UST | 2.276E-05 | -0.197 | - | - |
| 8092 | ENSP00000279907 | UHRF1BP1L | 6.382E-05 | -0.197 | - | - |
| 8093 | ENSP00000266736 | AMDHD1 | 3.824E-05 | -0.197 | - | - |
| 8094 | ENSP00000462106 | SMIM21 | 8.676E-07 | -0.197 | - | - |
| 8095 | ENSP00000248098 | HN1L | 6.741E-05 | -0.197 | - | - |
| 8096 | ENSP00000281631 | PARP8 | 5.797E-05 | -0.197 | - | - |
| 8097 | ENSP00000333539 | MIPOL1 | 3.629E-05 | -0.197 | - | - |
| 8098 | ENSP00000299952 | MARVELD3 | 5.935E-05 | -0.197 | - | - |
| 8099 | ENSP00000287675 | EXOG | 2.400E-05 | -0.197 | - | - |
| 8100 | ENSP00000308193 | RNASEH2C | 5.389E-05 | -0.197 | - | - |
| 8101 | ENSP00000266263 | MTFP1 | 2.780E-05 | -0.197 | - | - |
| 8102 | ENSP00000367422 | DLEU1 | 5.861E-06 | -0.197 | - | - |
| 8103 | ENSP00000256015 | BTG1 | 5.193E-05 | -0.197 | - | - |
| 8104 | ENSP00000389951 | CELF2 | 6.116E-05 | -0.197 | - | - |
| 8105 | ENSP00000470691 | ZSCAN5D | 9.017E-06 | -0.197 | - | - |
| 8106 | ENSP00000321826 | STXBP5 | 1.983E-05 | -0.197 | - | - |
| 8107 | ENSP00000404251 | VGLL4 | 3.479E-06 | -0.197 | - | - |
| 8108 | ENSP00000278618 | AASDHPPT | 3.096E-05 | -0.197 | - | - |
| 8109 | ENSP00000256379 | MED6 | 2.619E-05 | -0.197 | - | - |
| 8110 | ENSP00000237172 | FILIP1 | 2.979E-05 | -0.197 | - | - |
| 8111 | ENSP00000338777 | AP3S2 | 2.990E-05 | -0.197 | - | - |
| 8112 | ENSP00000220931 | NCALD | 1.597E-05 | -0.197 | - | - |
| 8113 | ENSP00000333275 | NR2C1 | 3.238E-05 | -0.197 | - | - |
| 8114 | ENSP00000315386 | KDELC2 | 3.366E-05 | -0.197 | - | - |
| 8115 | ENSP00000376965 | ATXN3 | 4.032E-05 | -0.197 | - | - |
| 8116 | ENSP00000366899 | ZNF133 | 1.128E-05 | -0.197 | - | - |
| 8117 | ENSP00000258991 | TEX2 | 2.683E-05 | -0.197 | - | - |
| 8118 | ENSP00000263269 | GRIN2D | 2.437E-05 | -0.197 | - | - |
| 8119 | ENSP00000309899 | NARF | 2.860E-05 | -0.197 | - | - |
| 8120 | ENSP00000344468 | SDC3 | 2.593E-05 | -0.197 | - | - |
| 8121 | ENSP00000290310 | KCNE2 | 1.590E-05 | -0.197 | - | - |
| 8122 | ENSP00000335055 | WASF3 | 3.128E-05 | -0.197 | - | - |
| 8123 | ENSP00000326379 | VWA9 | 5.144E-05 | -0.197 | - | - |
| 8124 | ENSP00000006526 | WDR54 | 4.399E-05 | -0.197 | - | - |
| 8125 | ENSP00000269499 | ZCCHC2 | 4.405E-05 | -0.197 | - | - |
| 8126 | ENSP00000263991 | EHBP1 | 2.232E-05 | -0.197 | - | - |
| 8127 | ENSP00000246105 | DEFB129 | 2.990E-05 | -0.197 | - | - |
| 8128 | ENSP00000306614 | PPIH | 2.405E-05 | -0.197 | - | - |
| 8129 | ENSP00000264926 | RAD18 | 2.935E-05 | -0.197 | - | - |
| 8130 | ENSP00000278903 | EI24 | 4.448E-05 | -0.197 | - | - |
| 8131 | ENSP00000302756 | FOXD4L1 | 8.850E-06 | -0.197 | - | - |
| 8132 | ENSP00000164305 | PIGB | 3.895E-05 | -0.197 | - | - |
| 8133 | ENSP00000309166 | RBM4 | 3.525E-05 | -0.197 | - | - |
| 8134 | ENSP00000185150 | ERLEC1 | 2.037E-05 | -0.197 | - | - |
| 8135 | ENSP00000220420 | TGM5 | 3.848E-05 | -0.197 | - | - |
| 8136 | ENSP00000290536 | M1AP | 3.279E-05 | -0.197 | - | - |
| 8137 | ENSP00000219139 | C16orf70 | 4.864E-05 | -0.197 | - | - |
| 8138 | ENSP00000355529 | ZP4 | 2.695E-05 | -0.197 | - | - |
| 8139 | ENSP00000287996 | IPPK | 3.796E-05 | -0.197 | - | - |
| 8140 | ENSP00000259006 | LIMD2 | 4.290E-05 | -0.197 | - | - |
| 8141 | ENSP00000367315 | LRRC10B | 7.089E-06 | -0.197 | - | - |
| 8142 | ENSP00000261187 | SLC16A7 | 4.284E-05 | -0.197 | - | - |
| 8143 | ENSP00000317404 | CHST13 | 2.893E-05 | -0.197 | - | - |
| 8144 | ENSP00000332148 | DZIP1L | 3.773E-05 | -0.197 | - | - |
| 8145 | ENSP00000244537 | HIST1H4G | 2.021E-05 | -0.197 | - | - |
| 8146 | ENSP00000264344 | FAM13A | 4.682E-05 | -0.197 | - | - |
| 8147 | ENSP00000339587 | DFNA5 | 3.631E-05 | -0.197 | - | - |
| 8148 | ENSP00000299821 | NCAPH2 | 2.523E-05 | -0.197 | - | - |
| 8149 | ENSP00000310803 | SFT2D3 | 2.766E-05 | -0.197 | - | - |
| 8150 | ENSP00000354649 | MAGT1 | 1.916E-05 | -0.197 | - | - |
| 8151 | ENSP00000298694 | ARHGEF40 | 2.990E-05 | -0.197 | - | - |
| 8152 | ENSP00000290291 | OGFR | 2.295E-05 | -0.197 | - | - |
| 8153 | ENSP00000409107 | ZBTB42 | 1.030E-05 | -0.197 | - | - |
| 8154 | ENSP00000295119 | NUP35 | 3.280E-05 | -0.197 | - | - |
| 8155 | ENSP00000343223 | TCL1B | 3.551E-05 | -0.197 | - | - |
| 8156 | ENSP00000329715 | DRG1 | 2.919E-05 | -0.197 | - | - |
| 8157 | ENSP00000367629 | ARHGEF16 | 2.546E-05 | -0.197 | - | - |
| 8158 | ENSP00000317144 | PIBF1 | 3.075E-05 | -0.197 | - | - |
| 8159 | ENSP00000284898 | L3MBTL4 | 7.539E-06 | -0.197 | - | - |
| 8160 | ENSP00000281273 | QTRTD1 | 2.851E-05 | -0.197 | - | - |
| 8161 | ENSP00000288873 | KRTCAP3 | 5.414E-05 | -0.197 | - | - |
| 8162 | ENSP00000265723 | ABCB4 | 2.691E-05 | -0.197 | - | - |
| 8163 | ENSP00000272342 | ETAA1 | 6.806E-05 | -0.197 | - | - |
| 8164 | ENSP00000297135 | COG5 | 2.703E-05 | -0.197 | - | - |
| 8165 | ENSP00000315212 | RNF4 | 4.479E-05 | -0.197 | - | - |
| 8166 | ENSP00000312663 | CHRNA9 | 2.622E-05 | -0.197 | - | - |
| 8167 | ENSP00000282633 | FAM21A | 6.234E-05 | -0.197 | - | - |
| 8168 | ENSP00000295171 | CCDC74A | 3.398E-05 | -0.197 | - | - |
| 8169 | ENSP00000299267 | GABRB3 | 2.648E-05 | -0.197 | - | - |
| 8170 | ENSP00000308137 | FOLR1 | 3.260E-05 | -0.197 | - | - |
| 8171 | ENSP00000278483 | C11orf73 | 3.409E-05 | -0.197 | - | - |
| 8172 | ENSP00000261313 | PEBP1 | 2.704E-05 | -0.197 | - | - |
| 8173 | ENSP00000325594 | RUFY1 | 3.043E-05 | -0.197 | - | - |
| 8174 | ENSP00000250617 | ARHGEF6 | 2.543E-05 | -0.197 | - | - |
| 8175 | ENSP00000343126 | ARID3B | 2.484E-05 | -0.197 | - | - |
| 8176 | ENSP00000296741 | ENPP6 | 2.602E-05 | -0.197 | - | - |
| 8177 | ENSP00000452706 | GOLGA6L9 | 5.216E-06 | -0.197 | - | - |
| 8178 | ENSP00000260702 | LOXL4 | 5.556E-05 | -0.197 | - | - |
| 8179 | ENSP00000293443 | FAM171A2 | 3.946E-05 | -0.197 | - | - |
| 8180 | ENSP00000272395 | TRIM43 | 3.673E-05 | -0.197 | - | - |
| 8181 | ENSP00000307265 | IRF2BP1 | 4.793E-05 | -0.197 | - | - |
| 8182 | ENSP00000222574 | HBP1 | 3.893E-05 | -0.197 | - | - |
| 8183 | ENSP00000293777 | MED11 | 3.212E-05 | -0.197 | - | - |
| 8184 | ENSP00000247712 | MPV17L2 | 4.818E-05 | -0.197 | - | - |
| 8185 | ENSP00000372035 | LATS2 | 6.330E-05 | -0.197 | - | - |
| 8186 | ENSP00000314544 | CCZ1B | 2.829E-05 | -0.197 | - | - |
| 8187 | ENSP00000317542 | WASH4P | 3.684E-05 | -0.197 | - | - |
| 8188 | ENSP00000327468 | KLHDC8B | 2.906E-05 | -0.197 | - | - |
| 8189 | ENSP00000342805 | FAIM | 2.892E-05 | -0.197 | - | - |
| 8190 | ENSP00000306015 | SYNPO2 | 2.462E-05 | -0.197 | - | - |
| 8191 | ENSP00000305069 | TMEM192 | 3.566E-05 | -0.197 | - | - |
| 8192 | ENSP00000302037 | MFF | 3.740E-05 | -0.197 | - | - |
| 8193 | ENSP00000292494 | LY6E | 2.857E-05 | -0.197 | - | - |
| 8194 | ENSP00000307292 | PRKCDBP | 4.928E-05 | -0.197 | - | - |
| 8195 | ENSP00000320898 | RNF168 | 4.523E-05 | -0.197 | - | - |
| 8196 | ENSP00000265742 | ANKIB1 | 3.340E-05 | -0.197 | - | - |
| 8197 | ENSP00000343021 | ALKBH2 | 1.853E-05 | -0.197 | - | - |
| 8198 | ENSP00000293373 | NCKAP1L | 2.496E-05 | -0.197 | - | - |
| 8199 | ENSP00000252245 | KRT75 | 2.664E-05 | -0.197 | - | - |
| 8200 | ENSP00000434939 | CREB3L1 | 1.881E-05 | -0.197 | - | - |
| 8201 | ENSP00000257198 | DSC1 | 3.206E-05 | -0.197 | - | - |
| 8202 | ENSP00000255784 | CCDC134 | 3.500E-05 | -0.197 | - | - |
| 8203 | ENSP00000238823 | FAM98A | 4.727E-05 | -0.197 | - | - |
| 8204 | ENSP00000301011 | ZC3H18 | 3.837E-05 | -0.197 | - | - |
| 8205 | ENSP00000328088 | PAWR | 3.418E-05 | -0.197 | - | - |
| 8206 | ENSP00000292566 | ALKBH4 | 4.247E-05 | -0.197 | - | - |
| 8207 | ENSP00000329243 | KRT7 | 2.750E-05 | -0.197 | - | - |
| 8208 | ENSP00000346478 | ADAMTSL2 | 2.622E-05 | -0.197 | - | - |
| 8209 | ENSP00000372322 | ZMYM2 | 2.584E-05 | -0.198 | - | - |
| 8210 | ENSP00000260210 | BUD13 | 2.149E-05 | -0.198 | - | - |
| 8211 | ENSP00000315387 | TMEM102 | 4.974E-05 | -0.198 | - | - |
| 8212 | ENSP00000005386 | RPAP3 | 2.783E-05 | -0.198 | - | - |
| 8213 | ENSP00000297258 | FABP5 | 2.789E-05 | -0.198 | - | - |
| 8214 | ENSP00000317431 | ENTHD1 | 2.520E-05 | -0.198 | - | - |
| 8215 | ENSP00000378578 | AFF1 | 2.247E-05 | -0.198 | - | - |
| 8216 | ENSP00000254806 | WBP2 | 9.218E-05 | -0.198 | - | - |
| 8217 | ENSP00000252725 | ARPC1B | 3.575E-05 | -0.198 | - | - |
| 8218 | ENSP00000310356 | RMI2 | 2.788E-05 | -0.198 | - | - |
| 8219 | ENSP00000253047 | TMEM160 | 3.093E-05 | -0.198 | - | - |
| 8220 | ENSP00000215582 | C19orf21 | 4.171E-05 | -0.198 | - | - |
| 8221 | ENSP00000199706 | MRPL28 | 2.671E-05 | -0.198 | - | - |
| 8222 | ENSP00000280154 | PDCD4 | 1.012E-04 | -0.198 | - | - |
| 8223 | ENSP00000348799 | ATF7IP2 | 5.893E-05 | -0.198 | - | - |
| 8224 | ENSP00000321988 | SULT1A1 | 2.258E-05 | -0.198 | - | - |
| 8225 | ENSP00000386171 | ESRRG | 2.543E-05 | -0.198 | - | - |
| 8226 | ENSP00000266087 | FBXO7 | 1.890E-05 | -0.198 | - | - |
| 8227 | ENSP00000226760 | WFS1 | 2.549E-05 | -0.198 | - | - |
| 8228 | ENSP00000290155 | C21orf59 | 3.608E-05 | -0.198 | - | - |
| 8229 | ENSP00000329097 | PHF6 | 2.417E-05 | -0.198 | - | - |
| 8230 | ENSP00000307887 | MXRA8 | 4.597E-05 | -0.198 | - | - |
| 8231 | ENSP00000342023 | FBXO38 | 3.278E-05 | -0.198 | - | - |
| 8232 | ENSP00000379847 | ZNF143 | 3.273E-05 | -0.198 | - | - |
| 8233 | ENSP00000366095 | MOG | 5.254E-06 | -0.198 | - | - |
| 8234 | ENSP00000239446 | PCDHB10 | 4.420E-05 | -0.198 | - | - |
| 8235 | ENSP00000310375 | MLC1 | 2.412E-05 | -0.198 | - | - |
| 8236 | ENSP00000310726 | MRP63 | 2.475E-05 | -0.198 | - | - |
| 8237 | ENSP00000320688 | SLC25A36 | 3.166E-05 | -0.198 | - | - |
| 8238 | ENSP00000366306 | SPRY2 | 6.454E-05 | -0.198 | - | - |
| 8239 | ENSP00000264059 | EFHD1 | 2.496E-05 | -0.198 | - | - |
| 8240 | ENSP00000327506 | C16orf54 | 6.025E-05 | -0.198 | - | - |
| 8241 | ENSP00000239690 | NUDCD1 | 3.481E-05 | -0.198 | - | - |
| 8242 | ENSP00000358903 | CYP17A1 | 2.783E-05 | -0.198 | - | - |
| 8243 | ENSP00000248420 | CACTIN | 3.947E-05 | -0.198 | - | - |
| 8244 | ENSP00000325423 | INPP1 | 2.978E-05 | -0.198 | - | - |
| 8245 | ENSP00000343966 | PSPC1 | 2.456E-05 | -0.198 | - | - |
| 8246 | ENSP00000287097 | CD109 | 3.871E-05 | -0.198 | - | - |
| 8247 | ENSP00000230792 | NUDT12 | 2.418E-05 | -0.198 | - | - |
| 8248 | ENSP00000442373 | ZDHHC11B | 7.468E-06 | -0.198 | - | - |
| 8249 | ENSP00000271277 | CTTNBP2NL | 4.078E-05 | -0.198 | - | - |
| 8250 | ENSP00000216513 | SIX4 | 3.268E-05 | -0.198 | - | - |
| 8251 | ENSP00000312318 | SSSCA1 | 2.104E-05 | -0.198 | - | - |
| 8252 | ENSP00000337675 | EBAG9 | 3.479E-05 | -0.198 | - | - |
| 8253 | ENSP00000024061 | SLC45A4 | 3.700E-05 | -0.198 | - | - |
| 8254 | ENSP00000350265 | ENPP3 | 2.483E-05 | -0.198 | - | - |
| 8255 | ENSP00000334216 | ATP4B | 2.200E-05 | -0.198 | - | - |
| 8256 | ENSP00000221856 | FSD1 | 3.768E-05 | -0.198 | - | - |
| 8257 | ENSP00000282276 | MARS2 | 2.020E-05 | -0.198 | - | - |
| 8258 | ENSP00000337688 | SEC62 | 2.446E-05 | -0.198 | - | - |
| 8259 | ENSP00000294129 | NCKIPSD | 2.782E-05 | -0.198 | - | - |
| 8260 | ENSP00000337353 | TDP1 | 2.463E-05 | -0.198 | - | - |
| 8261 | ENSP00000260247 | DCUN1D5 | 5.128E-05 | -0.198 | - | - |
| 8262 | ENSP00000203556 | GMIP | 3.503E-05 | -0.198 | - | - |
| 8263 | ENSP00000345230 | TPM4 | 2.708E-05 | -0.198 | - | - |
| 8264 | ENSP00000295148 | C2orf44 | 6.627E-05 | -0.198 | - | - |
| 8265 | ENSP00000225525 | TAX1BP3 | 3.175E-05 | -0.198 | - | - |
| 8266 | ENSP00000357452 | PMVK | 2.284E-05 | -0.198 | - | - |
| 8267 | ENSP00000323645 | TTC39C | 3.821E-05 | -0.198 | - | - |
| 8268 | ENSP00000216194 | ADSL | 3.378E-05 | -0.198 | - | - |
| 8269 | ENSP00000273308 | CNPY2 | 2.768E-05 | -0.198 | - | - |
| 8270 | ENSP00000356191 | OPTC | 9.651E-06 | -0.198 | - | - |
| 8271 | ENSP00000319713 | AGTRAP | 3.522E-05 | -0.198 | - | - |
| 8272 | ENSP00000255674 | RTTN | 3.519E-05 | -0.198 | - | - |
| 8273 | ENSP00000261191 | ASUN | 4.125E-05 | -0.198 | - | - |
| 8274 | ENSP00000319343 | CPT1C | 1.598E-05 | -0.198 | - | - |
| 8275 | ENSP00000008391 | TFAP2D | 4.138E-05 | -0.198 | - | - |
| 8276 | ENSP00000256474 | VHL | 8.417E-05 | -0.198 | - | - |
| 8277 | ENSP00000336923 | ARHGAP10 | 2.571E-05 | -0.198 | - | - |
| 8278 | ENSP00000326604 | LMBR1 | 3.904E-05 | -0.198 | - | - |
| 8279 | ENSP00000272797 | KLHL23 | 3.281E-05 | -0.198 | - | - |
| 8280 | ENSP00000356989 | PFDN2 | 1.732E-05 | -0.198 | - | - |
| 8281 | ENSP00000264596 | NEIL3 | 3.389E-05 | -0.198 | - | - |
| 8282 | ENSP00000369100 | PFKFB3 | 1.639E-05 | -0.198 | - | - |
| 8283 | ENSP00000299088 | C14orf93 | 6.767E-05 | -0.198 | - | - |
| 8284 | ENSP00000271526 | PRCC | 2.976E-05 | -0.198 | - | - |
| 8285 | ENSP00000361635 | VDAC2 | 1.652E-05 | -0.198 | - | - |
| 8286 | ENSP00000005286 | TMEM132A | 3.358E-05 | -0.198 | - | - |
| 8287 | ENSP00000318852 | BPNT1 | 4.012E-05 | -0.198 | - | - |
| 8288 | ENSP00000251595 | HBA2 | 2.318E-05 | -0.198 | - | - |
| 8289 | ENSP00000328358 | KRT79 | 3.591E-05 | -0.198 | - | - |
| 8290 | ENSP00000332979 | BACE2 | 2.560E-05 | -0.198 | - | - |
| 8291 | ENSP00000417864 | ANP32A | 3.109E-05 | -0.198 | - | - |
| 8292 | ENSP00000419692 | RXRA | 3.558E-05 | -0.198 | - | - |
| 8293 | ENSP00000259056 | GALNT5 | 3.270E-05 | -0.198 | - | - |
| 8294 | ENSP00000329219 | FMNL1 | 2.794E-05 | -0.198 | - | - |
| 8295 | ENSP00000280987 | FAM177A1 | 3.392E-05 | -0.198 | - | - |
| 8296 | ENSP00000330361 | C8orf33 | 2.492E-05 | -0.198 | - | - |
| 8297 | ENSP00000380033 | DDX17 | 3.949E-05 | -0.198 | - | - |
| 8298 | ENSP00000333283 | C17orf70 | 3.371E-05 | -0.198 | - | - |
| 8299 | ENSP00000271332 | CELSR2 | 3.522E-05 | -0.198 | - | - |
| 8300 | ENSP00000265433 | NBN | 2.245E-05 | -0.198 | - | - |
| 8301 | ENSP00000298316 | ARF6 | 3.543E-05 | -0.198 | - | - |
| 8302 | ENSP00000194214 | HSPB11 | 2.717E-05 | -0.198 | - | - |
| 8303 | ENSP00000239830 | CCDC77 | 4.695E-05 | -0.198 | - | - |
| 8304 | ENSP00000231948 | CRBN | 2.388E-05 | -0.198 | - | - |
| 8305 | ENSP00000298546 | C9orf9 | 2.872E-05 | -0.198 | - | - |
| 8306 | ENSP00000311609 | SPSB4 | 2.847E-05 | -0.198 | - | - |
| 8307 | ENSP00000217420 | SLC32A1 | 2.441E-05 | -0.198 | - | - |
| 8308 | ENSP00000285979 | CYP2C18 | 1.675E-05 | -0.198 | - | - |
| 8309 | ENSP00000265627 | PON3 | 2.535E-05 | -0.198 | - | - |
| 8310 | ENSP00000369434 | ANKRD16 | 3.712E-05 | -0.198 | - | - |
| 8311 | ENSP00000376534 | BAZ2B | 1.492E-05 | -0.198 | - | - |
| 8312 | ENSP00000329918 | MEX3B | 2.355E-05 | -0.198 | - | - |
| 8313 | ENSP00000299565 | CHRNA5 | 2.493E-05 | -0.198 | - | - |
| 8314 | ENSP00000311318 | ATG4D | 3.265E-05 | -0.198 | - | - |
| 8315 | ENSP00000254770 | LANCL2 | 3.051E-05 | -0.198 | - | - |
| 8316 | ENSP00000270176 | SCYL1 | 3.705E-05 | -0.198 | - | - |
| 8317 | ENSP00000162044 | TMEM161A | 4.705E-05 | -0.198 | - | - |
| 8318 | ENSP00000452871 | TLE3 | 4.051E-05 | -0.198 | - | - |
| 8319 | ENSP00000286070 | RBM45 | 5.058E-05 | -0.198 | - | - |
| 8320 | ENSP00000346015 | RPL27A | 4.859E-05 | -0.198 | - | - |
| 8321 | ENSP00000300030 | FAM96A | 4.065E-05 | -0.198 | - | - |
| 8322 | ENSP00000256366 | SYNJ2BP | 4.961E-05 | -0.198 | - | - |
| 8323 | ENSP00000319009 | TMEM45A | 2.911E-05 | -0.198 | - | - |
| 8324 | ENSP00000428541 | SMIM12 | 6.656E-06 | -0.198 | - | - |
| 8325 | ENSP00000283426 | PLEKHG4B | 2.421E-05 | -0.198 | - | - |
| 8326 | ENSP00000295030 | PEX13 | 3.526E-05 | -0.198 | - | - |
| 8327 | ENSP00000262887 | XRCC1 | 2.837E-05 | -0.198 | - | - |
| 8328 | ENSP00000396052 | BRMS1 | 2.453E-05 | -0.198 | - | - |
| 8329 | ENSP00000295304 | CHAC2 | 3.418E-05 | -0.198 | - | - |
| 8330 | ENSP00000268766 | NEK8 | 2.668E-05 | -0.198 | - | - |
| 8331 | ENSP00000350263 | PIGN | 1.789E-05 | -0.198 | - | - |
| 8332 | ENSP00000344547 | PTMA | 1.026E-03 | -0.198 | - | - |
| 8333 | ENSP00000264151 | OSGEPL1 | 5.553E-06 | -0.198 | - | - |
| 8334 | ENSP00000335579 | PPP4R1L | 2.575E-05 | -0.198 | - | - |
| 8335 | ENSP00000238379 | HDHD3 | 2.720E-05 | -0.198 | - | - |
| 8336 | ENSP00000341213 | C20orf24 | 2.111E-05 | -0.198 | - | - |
| 8337 | ENSP00000291416 | TRIM62 | 3.636E-05 | -0.198 | - | - |
| 8338 | ENSP00000330074 | HIST1H1B | 3.175E-05 | -0.198 | - | - |
| 8339 | ENSP00000265437 | ST7 | 3.448E-05 | -0.198 | - | - |
| 8340 | ENSP00000255613 | SUV420H2 | 2.780E-05 | -0.198 | - | - |
| 8341 | ENSP00000261488 | ENOX1 | 4.857E-05 | -0.198 | - | - |
| 8342 | ENSP00000299886 | COG1 | 2.441E-05 | -0.198 | - | - |
| 8343 | ENSP00000217456 | APMAP | 3.926E-05 | -0.198 | - | - |
| 8344 | ENSP00000362820 | SRSF3 | 1.625E-05 | -0.198 | - | - |
| 8345 | ENSP00000311513 | RSF1 | 2.449E-05 | -0.198 | - | - |
| 8346 | ENSP00000323348 | ZNF645 | 3.036E-05 | -0.198 | - | - |
| 8347 | ENSP00000276893 | UHRF2 | 2.723E-05 | -0.198 | - | - |
| 8348 | ENSP00000252622 | LSM7 | 2.189E-05 | -0.198 | - | - |
| 8349 | ENSP00000359221 | RWDD3 | 3.963E-05 | -0.198 | - | - |
| 8350 | ENSP00000331574 | PDE1A | 2.637E-05 | -0.198 | - | - |
| 8351 | ENSP00000316109 | AGGF1 | 3.200E-05 | -0.198 | - | - |
| 8352 | ENSP00000344903 | MAFK | 2.521E-05 | -0.198 | - | - |
| 8353 | ENSP00000321184 | IGSF3 | 4.096E-05 | -0.198 | - | - |
| 8354 | ENSP00000296380 | EXO5 | 5.245E-05 | -0.198 | - | - |
| 8355 | ENSP00000354842 | TOX | 2.927E-05 | -0.198 | - | - |
| 8356 | ENSP00000238018 | GDA | 2.219E-05 | -0.198 | - | - |
| 8357 | ENSP00000302783 | TRAPPC1 | 2.716E-05 | -0.198 | - | - |
| 8358 | ENSP00000298472 | SLC18A2 | 2.439E-05 | -0.199 | - | - |
| 8359 | ENSP00000289547 | NPC1L1 | 2.465E-05 | -0.199 | - | - |
| 8360 | ENSP00000262441 | GLP2R | 2.293E-05 | -0.199 | - | - |
| 8361 | ENSP00000267430 | FANCM | 1.990E-05 | -0.199 | - | - |
| 8362 | ENSP00000361740 | WBP5 | 5.593E-05 | -0.199 | - | - |
| 8363 | ENSP00000309749 | APOBEC3F | 3.214E-05 | -0.199 | - | - |
| 8364 | ENSP00000281513 | NBAS | 3.017E-05 | -0.199 | - | - |
| 8365 | ENSP00000299705 | TMED3 | 1.813E-05 | -0.199 | - | - |
| 8366 | ENSP00000254878 | HRSP12 | 1.962E-05 | -0.199 | - | - |
| 8367 | ENSP00000219400 | CMC2 | 2.118E-05 | -0.199 | - | - |
| 8368 | ENSP00000231137 | PCDHB7 | 2.722E-05 | -0.199 | - | - |
| 8369 | ENSP00000358089 | TIAL1 | 2.740E-05 | -0.199 | - | - |
| 8370 | ENSP00000262193 | PSMB1 | 2.092E-05 | -0.199 | - | - |
| 8371 | ENSP00000357887 | VPS72 | 1.420E-05 | -0.199 | - | - |
| 8372 | ENSP00000296333 | PIGX | 3.023E-05 | -0.199 | - | - |
| 8373 | ENSP00000303511 | PEX6 | 2.098E-05 | -0.199 | - | - |
| 8374 | ENSP00000264198 | MUL1 | 3.921E-05 | -0.199 | - | - |
| 8375 | ENSP00000348578 | G3BP1 | 1.906E-05 | -0.199 | - | - |
| 8376 | ENSP00000312999 | GNAI2 | 3.248E-05 | -0.199 | - | - |
| 8377 | ENSP00000354414 | ZNF627 | 1.342E-05 | -0.199 | - | - |
| 8378 | ENSP00000307765 | C17orf62 | 3.861E-05 | -0.199 | - | - |
| 8379 | ENSP00000329662 | H1FX | 2.439E-05 | -0.199 | - | - |
| 8380 | ENSP00000316021 | PLSCR3 | 2.110E-05 | -0.199 | - | - |
| 8381 | ENSP00000383115 | COLEC12 | 3.134E-05 | -0.199 | - | - |
| 8382 | ENSP00000341267 | HMGN3 | 5.298E-05 | -0.199 | - | - |
| 8383 | ENSP00000308032 | CYP2S1 | 5.222E-05 | -0.199 | - | - |
| 8384 | ENSP00000383411 | SAMSN1 | 2.397E-05 | -0.199 | - | - |
| 8385 | ENSP00000334223 | SLC36A2 | 2.243E-05 | -0.199 | - | - |
| 8386 | ENSP00000254579 | DNHD1 | 2.497E-05 | -0.199 | - | - |
| 8387 | ENSP00000417509 | FAM208A | 9.915E-06 | -0.199 | - | - |
| 8388 | ENSP00000259154 | KCTD3 | 1.598E-05 | -0.199 | - | - |
| 8389 | ENSP00000379908 | ICA1 | 3.697E-05 | -0.199 | - | - |
| 8390 | ENSP00000263384 | FAM32A | 2.927E-05 | -0.199 | - | - |
| 8391 | ENSP00000269373 | FN3KRP | 2.932E-05 | -0.199 | - | - |
| 8392 | ENSP00000433361 | TMEM9B | 1.654E-05 | -0.199 | - | - |
| 8393 | ENSP00000291900 | ZER1 | 2.957E-05 | -0.199 | - | - |
| 8394 | ENSP00000369531 | UBQLNL | 3.867E-05 | -0.199 | - | - |
| 8395 | ENSP00000316664 | IGSF8 | 2.254E-05 | -0.199 | - | - |
| 8396 | ENSP00000264917 | PDE8B | 2.419E-05 | -0.199 | - | - |
| 8397 | ENSP00000323780 | IP6K1 | 2.764E-05 | -0.199 | - | - |
| 8398 | ENSP00000240617 | PLBD1 | 3.968E-05 | -0.199 | - | - |
| 8399 | ENSP00000317039 | RMI1 | 3.862E-05 | -0.199 | - | - |
| 8400 | ENSP00000255108 | DPH2 | 2.300E-05 | -0.199 | - | - |
| 8401 | ENSP00000262731 | PRR5-ARHGAP8 | 2.613E-05 | -0.199 | - | - |
| 8402 | ENSP00000235835 | AKR7A2 | 2.578E-05 | -0.199 | - | - |
| 8403 | ENSP00000326767 | MED25 | 1.987E-05 | -0.199 | - | - |
| 8404 | ENSP00000005905 | - | 2.773E-05 | -0.199 | - | - |
| 8405 | ENSP00000247461 | CANX | 3.663E-05 | -0.199 | - | - |
| 8406 | ENSP00000244227 | SNRNP27 | 3.143E-05 | -0.199 | - | - |
| 8407 | ENSP00000358154 | HIST2H3C | 2.806E-05 | -0.199 | - | - |
| 8408 | ENSP00000265000 | GALNT7 | 2.606E-05 | -0.199 | - | - |
| 8409 | ENSP00000416015 | MICAL3 | 4.859E-05 | -0.199 | - | - |
| 8410 | ENSP00000074304 | INPP4A | 2.692E-05 | -0.199 | - | - |
| 8411 | ENSP00000306887 | TMEM126A | 2.456E-05 | -0.199 | - | - |
| 8412 | ENSP00000218147 | BCORL1 | 3.314E-05 | -0.199 | - | - |
| 8413 | ENSP00000305524 | TSEN34 | 2.164E-05 | -0.199 | - | - |
| 8414 | ENSP00000293273 | RDM1 | 3.444E-05 | -0.199 | - | - |
| 8415 | ENSP00000334364 | DEFB106B | 5.302E-06 | -0.199 | - | - |
| 8416 | ENSP00000338673 | CDK10 | 2.624E-05 | -0.199 | - | - |
| 8417 | ENSP00000040738 | BOD1L1 | 3.004E-05 | -0.199 | - | - |
| 8418 | ENSP00000260526 | ARHGAP29 | 2.169E-05 | -0.199 | - | - |
| 8419 | ENSP00000321070 | ME2 | 3.156E-05 | -0.199 | - | - |
| 8420 | ENSP00000246747 | ARL2 | 3.621E-05 | -0.199 | - | - |
| 8421 | ENSP00000263092 | METTL16 | 3.559E-05 | -0.199 | - | - |
| 8422 | ENSP00000262384 | N4BP1 | 5.879E-05 | -0.199 | - | - |
| 8423 | ENSP00000305926 | OTUD7A | 3.045E-05 | -0.199 | - | - |
| 8424 | ENSP00000304192 | PCNX | 3.276E-05 | -0.199 | - | - |
| 8425 | ENSP00000266971 | SUOX | 2.189E-05 | -0.199 | - | - |
| 8426 | ENSP00000264720 | GTF3C2 | 3.151E-05 | -0.199 | - | - |
| 8427 | ENSP00000285279 | VOPP1 | 3.178E-05 | -0.199 | - | - |
| 8428 | ENSP00000263033 | SYTL4 | 3.429E-05 | -0.199 | - | - |
| 8429 | ENSP00000373657 | C17orf85 | 2.561E-05 | -0.199 | - | - |
| 8430 | ENSP00000351682 | CNDP1 | 3.041E-05 | -0.199 | - | - |
| 8431 | ENSP00000262265 | PIH1D1 | 1.921E-05 | -0.199 | - | - |
| 8432 | ENSP00000333456 | RAI2 | 4.028E-05 | -0.199 | - | - |
| 8433 | ENSP00000258417 | ARMC9 | 3.804E-05 | -0.199 | - | - |
| 8434 | ENSP00000215957 | MICALL1 | 2.371E-05 | -0.199 | - | - |
| 8435 | ENSP00000368766 | ADRA1D | 5.653E-05 | -0.199 | - | - |
| 8436 | ENSP00000229563 | TMEM14C | 3.973E-05 | -0.199 | - | - |
| 8437 | ENSP00000333666 | ADI1 | 3.601E-05 | -0.199 | - | - |
| 8438 | ENSP00000387081 | AC140481.2 | 6.695E-06 | -0.199 | - | - |
| 8439 | ENSP00000368005 | CCNI2 | 1.824E-05 | -0.199 | - | - |
| 8440 | ENSP00000344504 | H1F0 | 2.940E-05 | -0.199 | - | - |
| 8441 | ENSP00000276055 | CHST7 | 2.964E-05 | -0.199 | - | - |
| 8442 | ENSP00000284061 | DGKE | 2.316E-05 | -0.199 | - | - |
| 8443 | ENSP00000315680 | PEX7 | 2.781E-05 | -0.199 | - | - |
| 8444 | ENSP00000295117 | CCDC104 | 2.297E-05 | -0.199 | - | - |
| 8445 | ENSP00000232603 | MORC1 | 2.383E-05 | -0.199 | - | - |
| 8446 | ENSP00000343818 | CDK5RAP2 | 2.625E-05 | -0.199 | - | - |
| 8447 | ENSP00000243706 | HAUS3 | 2.248E-05 | -0.199 | - | - |
| 8448 | ENSP00000023064 | SLC7A9 | 2.568E-05 | -0.199 | - | - |
| 8449 | ENSP00000351957 | PDE3A | 2.484E-05 | -0.199 | - | - |
| 8450 | ENSP00000379999 | TIMM17B | 2.175E-05 | -0.199 | - | - |
| 8451 | ENSP00000285398 | ERCC3 | 2.197E-05 | -0.199 | - | - |
| 8452 | ENSP00000261944 | CDHR2 | 2.518E-05 | -0.199 | - | - |
| 8453 | ENSP00000349168 | HNRNPH1 | 2.239E-05 | -0.199 | - | - |
| 8454 | ENSP00000318902 | FOXP1 | 3.013E-05 | -0.199 | - | - |
| 8455 | ENSP00000334808 | USP16 | 2.832E-05 | -0.199 | - | - |
| 8456 | ENSP00000310620 | SNX32 | 4.180E-05 | -0.199 | - | - |
| 8457 | ENSP00000348043 | LRRC20 | 3.889E-05 | -0.199 | - | - |
| 8458 | ENSP00000288111 | DHRS1 | 2.779E-05 | -0.199 | - | - |
| 8459 | ENSP00000327214 | ATP13A2 | 3.229E-05 | -0.199 | - | - |
| 8460 | ENSP00000340797 | TRIM3 | 1.741E-05 | -0.199 | - | - |
| 8461 | ENSP00000295736 | SLC4A7 | 2.186E-05 | -0.199 | - | - |
| 8462 | ENSP00000294904 | RBMS1 | 3.521E-05 | -0.199 | - | - |
| 8463 | ENSP00000359299 | CCDC18 | 2.705E-05 | -0.199 | - | - |
| 8464 | ENSP00000248984 | C22orf24 | 3.096E-05 | -0.199 | - | - |
| 8465 | ENSP00000320508 | RNF185 | 1.914E-05 | -0.199 | - | - |
| 8466 | ENSP00000342098 | SPINT1 | 2.450E-05 | -0.199 | - | - |
| 8467 | ENSP00000292385 | DBN1 | 2.891E-05 | -0.199 | - | - |
| 8468 | ENSP00000331211 | RBM43 | 2.437E-05 | -0.199 | - | - |
| 8469 | ENSP00000311977 | DUS3L | 2.563E-05 | -0.199 | - | - |
| 8470 | ENSP00000334382 | HOMER1 | 1.884E-05 | -0.199 | - | - |
| 8471 | ENSP00000288912 | WDR66 | 1.638E-05 | -0.199 | - | - |
| 8472 | ENSP00000248929 | SGSM3 | 1.803E-05 | -0.199 | - | - |
| 8473 | ENSP00000342502 | PYCR2 | 2.539E-05 | -0.199 | - | - |
| 8474 | ENSP00000273861 | SLC10A4 | 2.876E-05 | -0.199 | - | - |
| 8475 | ENSP00000355566 | TOMM20 | 1.147E-05 | -0.199 | - | - |
| 8476 | ENSP00000199280 | AQP2 | 1.458E-05 | -0.199 | - | - |
| 8477 | ENSP00000374552 | RNF216 | 3.797E-05 | -0.199 | - | - |
| 8478 | ENSP00000265382 | PIP5K1B | 1.752E-05 | -0.199 | - | - |
| 8479 | ENSP00000274712 | ZMAT2 | 2.725E-05 | -0.199 | - | - |
| 8480 | ENSP00000319169 | PRMT5 | 9.379E-05 | -0.199 | - | - |
| 8481 | ENSP00000323099 | ATRIP | 2.061E-05 | -0.199 | - | - |
| 8482 | ENSP00000285419 | TMEM55A | 2.773E-05 | -0.199 | - | - |
| 8483 | ENSP00000269346 | TTYH2 | 2.052E-05 | -0.199 | - | - |
| 8484 | ENSP00000307181 | FAM103A1 | 3.106E-05 | -0.199 | - | - |
| 8485 | ENSP00000215739 | LZTR1 | 2.314E-05 | -0.199 | - | - |
| 8486 | ENSP00000227520 | CCDC86 | 2.640E-05 | -0.199 | - | - |
| 8487 | ENSP00000282058 | HAUS1 | 1.483E-05 | -0.199 | - | - |
| 8488 | ENSP00000246535 | PDCD2L | 2.689E-05 | -0.199 | - | - |
| 8489 | ENSP00000344572 | FIBP | 4.461E-05 | -0.199 | - | - |
| 8490 | ENSP00000062104 | NNAT | 2.441E-05 | -0.199 | - | - |
| 8491 | ENSP00000336616 | RASSF3 | 3.156E-05 | -0.199 | - | - |
| 8492 | ENSP00000300086 | TERF2IP | 3.177E-05 | -0.199 | - | - |
| 8493 | ENSP00000272217 | ARL8A | 2.683E-05 | -0.199 | - | - |
| 8494 | ENSP00000346236 | DDX46 | 1.979E-05 | -0.199 | - | - |
| 8495 | ENSP00000354441 | TMEM184B | 3.190E-05 | -0.199 | - | - |
| 8496 | ENSP00000305653 | LETM1 | 2.232E-05 | -0.199 | - | - |
| 8497 | ENSP00000264694 | SNX25 | 4.232E-05 | -0.199 | - | - |
| 8498 | ENSP00000338389 | MRPL35 | 1.998E-05 | -0.199 | - | - |
| 8499 | ENSP00000367528 | AARD | 1.546E-05 | -0.199 | - | - |
| 8500 | ENSP00000334319 | PCLO | 2.040E-05 | -0.199 | - | - |
| 8501 | ENSP00000318086 | MAP7D3 | 2.498E-05 | -0.199 | - | - |
| 8502 | ENSP00000308695 | DPY19L1 | 2.336E-05 | -0.199 | - | - |
| 8503 | ENSP00000263925 | LNX1 | 2.961E-05 | -0.199 | - | - |
| 8504 | ENSP00000246662 | KRT9 | 2.515E-05 | -0.199 | - | - |
| 8505 | ENSP00000303920 | MZB1 | 3.329E-05 | -0.200 | - | - |
| 8506 | ENSP00000329093 | TPH2 | 2.256E-05 | -0.200 | - | - |
| 8507 | ENSP00000281156 | KHDRBS2 | 1.118E-04 | -0.200 | - | - |
| 8508 | ENSP00000343635 | TLDC1 | 3.874E-05 | -0.200 | - | - |
| 8509 | ENSP00000297195 | SLC29A4 | 1.804E-05 | -0.200 | - | - |
| 8510 | ENSP00000321271 | PXMP2 | 2.424E-05 | -0.200 | - | - |
| 8511 | ENSP00000446100 | CMIP | 2.008E-05 | -0.200 | - | - |
| 8512 | ENSP00000329002 | HAX1 | 3.820E-05 | -0.200 | - | - |
| 8513 | ENSP00000260662 | CENPO | 1.741E-05 | -0.200 | - | - |
| 8514 | ENSP00000364582 | RCC2 | 1.712E-05 | -0.200 | - | - |
| 8515 | ENSP00000262210 | CSPP1 | 2.817E-05 | -0.200 | - | - |
| 8516 | ENSP00000263697 | DNAJC8 | 3.040E-05 | -0.200 | - | - |
| 8517 | ENSP00000290913 | CHCHD6 | 2.018E-05 | -0.200 | - | - |
| 8518 | ENSP00000313601 | CC2D1A | 2.028E-05 | -0.200 | - | - |
| 8519 | ENSP00000296149 | ELP6 | 3.252E-05 | -0.200 | - | - |
| 8520 | ENSP00000011684 | PLEKHG6 | 2.179E-05 | -0.200 | - | - |
| 8521 | ENSP00000236273 | SYF2 | 2.288E-05 | -0.200 | - | - |
| 8522 | ENSP00000335203 | ATPIF1 | 1.726E-05 | -0.200 | - | - |
| 8523 | ENSP00000360905 | AGBL4 | 2.283E-05 | -0.200 | - | - |
| 8524 | ENSP00000327691 | - | 1.920E-05 | -0.200 | - | - |
| 8525 | ENSP00000326070 | SLC41A3 | 2.640E-05 | -0.200 | - | - |
| 8526 | ENSP00000251250 | DTWD1 | 1.118E-04 | -0.200 | - | - |
| 8527 | ENSP00000274569 | PCYOX1L | 2.754E-05 | -0.200 | - | - |
| 8528 | ENSP00000263997 | SLC7A6OS | 1.767E-05 | -0.200 | - | - |
| 8529 | ENSP00000313050 | GBAS | 2.355E-05 | -0.200 | - | - |
| 8530 | ENSP00000322020 | SLC25A22 | 2.271E-05 | -0.200 | - | - |
| 8531 | ENSP00000252456 | CNN1 | 1.895E-05 | -0.200 | - | - |
| 8532 | ENSP00000257867 | LACRT | 2.250E-05 | -0.200 | - | - |
| 8533 | ENSP00000388902 | - | 2.315E-05 | -0.200 | - | - |
| 8534 | ENSP00000256429 | MBD2 | 3.121E-05 | -0.200 | - | - |
| 8535 | ENSP00000286353 | ACPL2 | 3.334E-05 | -0.200 | - | - |
| 8536 | ENSP00000367446 | EXT1 | 2.440E-05 | -0.200 | - | - |
| 8537 | ENSP00000335557 | SLX4IP | 3.172E-05 | -0.200 | - | - |
| 8538 | ENSP00000313059 | WNK1 | 2.821E-05 | -0.200 | - | - |
| 8539 | ENSP00000265651 | FBXO3 | 2.953E-05 | -0.200 | - | - |
| 8540 | ENSP00000233969 | SLC9A2 | 1.723E-05 | -0.200 | - | - |
| 8541 | ENSP00000380920 | C19orf38 | 6.516E-07 | -0.200 | - | - |
| 8542 | ENSP00000272424 | TPRKB | 2.450E-05 | -0.200 | - | - |
| 8543 | ENSP00000251074 | NUP37 | 3.079E-05 | -0.200 | - | - |
| 8544 | ENSP00000327541 | BLZF1 | 2.212E-05 | -0.200 | - | - |
| 8545 | ENSP00000242591 | IFT81 | 2.500E-05 | -0.200 | - | - |
| 8546 | ENSP00000268876 | UNC45B | 1.910E-05 | -0.200 | - | - |
| 8547 | ENSP00000343314 | FXYD1 | 7.765E-06 | -0.200 | - | - |
| 8548 | ENSP00000250101 | TXNDC17 | 2.913E-05 | -0.200 | - | - |
| 8549 | ENSP00000366901 | FAM216A | 2.684E-05 | -0.200 | - | - |
| 8550 | ENSP00000293894 | SOX8 | 2.072E-05 | -0.200 | - | - |
| 8551 | ENSP00000352185 | PHF2 | 9.549E-06 | -0.200 | - | - |
| 8552 | ENSP00000223321 | PSMA2 | 1.732E-05 | -0.200 | - | - |
| 8553 | ENSP00000390750 | SMIM19 | 1.212E-05 | -0.200 | - | - |
| 8554 | ENSP00000257951 | KRT84 | 2.052E-05 | -0.200 | - | - |
| 8555 | ENSP00000293255 | CABP5 | 1.877E-05 | -0.200 | - | - |
| 8556 | ENSP00000296215 | SNIP1 | 2.395E-05 | -0.200 | - | - |
| 8557 | ENSP00000236980 | FASTKD2 | 3.147E-05 | -0.200 | - | - |
| 8558 | ENSP00000269397 | CBX4 | 2.718E-05 | -0.200 | - | - |
| 8559 | ENSP00000264042 | FARP2 | 2.966E-05 | -0.200 | - | - |
| 8560 | ENSP00000273666 | STXBP5L | 2.041E-05 | -0.200 | - | - |
| 8561 | ENSP00000305502 | ASCC2 | 2.492E-05 | -0.200 | - | - |
| 8562 | ENSP00000268712 | NCOR1 | 4.521E-05 | -0.200 | - | - |
| 8563 | ENSP00000395505 | PATE3 | 2.084E-05 | -0.200 | - | - |
| 8564 | ENSP00000321424 | SLC34A1 | 2.643E-05 | -0.200 | - | - |
| 8565 | ENSP00000217121 | TPD52L2 | 2.895E-05 | -0.200 | - | - |
| 8566 | ENSP00000252455 | PRKCSH | 2.539E-05 | -0.200 | - | - |
| 8567 | ENSP00000158771 | DERL2 | 2.796E-05 | -0.200 | - | - |
| 8568 | ENSP00000214869 | TMED1 | 2.811E-05 | -0.200 | - | - |
| 8569 | ENSP00000217652 | MYL12A | 1.543E-05 | -0.200 | - | - |
| 8570 | ENSP00000301284 | HDGFRP2 | 2.588E-05 | -0.200 | - | - |
| 8571 | ENSP00000333821 | MC2R | 2.262E-05 | -0.200 | - | - |
| 8572 | ENSP00000316854 | ATOX1 | 2.447E-05 | -0.200 | - | - |
| 8573 | ENSP00000283429 | NMRAL1 | 2.243E-05 | -0.200 | - | - |
| 8574 | ENSP00000250111 | ATP1B2 | 1.811E-05 | -0.200 | - | - |
| 8575 | ENSP00000370648 | RDH14 | 7.464E-06 | -0.200 | - | - |
| 8576 | ENSP00000261735 | ERP29 | 1.854E-05 | -0.200 | - | - |
| 8577 | ENSP00000363397 | UGCG | 2.786E-05 | -0.200 | - | - |
| 8578 | ENSP00000317579 | C16orf58 | 1.715E-05 | -0.200 | - | - |
| 8579 | ENSP00000338606 | SMUG1 | 3.083E-05 | -0.200 | - | - |
| 8580 | ENSP00000225426 | PSMB3 | 2.657E-05 | -0.200 | - | - |
| 8581 | ENSP00000379880 | LRRC3B | 1.741E-05 | -0.200 | - | - |
| 8582 | ENSP00000256441 | MRPS36 | 2.073E-05 | -0.200 | - | - |
| 8583 | ENSP00000398619 | KRTAP25-1 | 1.929E-07 | -0.200 | - | - |
| 8584 | ENSP00000286317 | MED7 | 2.122E-05 | -0.200 | - | - |
| 8585 | ENSP00000264735 | HRASLS | 4.216E-05 | -0.200 | - | - |
| 8586 | ENSP00000445446 | C3orf33 | 4.397E-06 | -0.200 | - | - |
| 8587 | ENSP00000288078 | FUK | 2.261E-05 | -0.200 | - | - |
| 8588 | ENSP00000286371 | ATP1B3 | 1.630E-05 | -0.200 | - | - |
| 8589 | ENSP00000332103 | LSMD1 | 3.029E-05 | -0.200 | - | - |
| 8590 | ENSP00000265872 | CCAR1 | 2.183E-05 | -0.200 | - | - |
| 8591 | ENSP00000362728 | KPNA6 | 1.865E-05 | -0.200 | - | - |
| 8592 | ENSP00000327323 | THNSL2 | 1.877E-05 | -0.200 | - | - |
| 8593 | ENSP00000264313 | SLAIN2 | 5.207E-05 | -0.200 | - | - |
| 8594 | ENSP00000278919 | FEZ1 | 3.413E-05 | -0.200 | - | - |
| 8595 | ENSP00000285021 | XPC | 2.461E-05 | -0.200 | - | - |
| 8596 | ENSP00000283243 | PLA2R1 | 2.805E-05 | -0.200 | - | - |
| 8597 | ENSP00000266732 | TMPO | 2.650E-05 | -0.200 | - | - |
| 8598 | ENSP00000341117 | SDSL | 2.553E-05 | -0.200 | - | - |
| 8599 | ENSP00000342181 | SRP72 | 2.460E-05 | -0.200 | - | - |
| 8600 | ENSP00000262365 | FAM86B2 | 2.082E-05 | -0.200 | - | - |
| 8601 | ENSP00000199708 | HBQ1 | 1.843E-05 | -0.200 | - | - |
| 8602 | ENSP00000369897 | CARS | 3.774E-05 | -0.200 | - | - |
| 8603 | ENSP00000309644 | BOD1 | 3.448E-05 | -0.200 | - | - |
| 8604 | ENSP00000325266 | TTC9C | 2.206E-05 | -0.200 | - | - |
| 8605 | ENSP00000327145 | FLNC | 2.539E-05 | -0.200 | - | - |
| 8606 | ENSP00000234453 | PLEKHA3 | 6.650E-05 | -0.200 | - | - |
| 8607 | ENSP00000354718 | AKAP13 | 2.774E-05 | -0.200 | - | - |
| 8608 | ENSP00000025301 | AKAP11 | 3.643E-05 | -0.200 | - | - |
| 8609 | ENSP00000365898 | FOXB2 | 1.327E-05 | -0.200 | - | - |
| 8610 | ENSP00000360158 | PKHD1 | 2.396E-05 | -0.200 | - | - |
| 8611 | ENSP00000308310 | TRIM40 | 3.681E-05 | -0.200 | - | - |
| 8612 | ENSP00000261866 | SPG11 | 2.066E-05 | -0.200 | - | - |
| 8613 | ENSP00000271764 | EIF2D | 5.993E-05 | -0.200 | - | - |
| 8614 | ENSP00000287078 | TYSND1 | 1.696E-05 | -0.200 | - | - |
| 8615 | ENSP00000282041 | EPG5 | 2.758E-05 | -0.200 | - | - |
| 8616 | ENSP00000292246 | ANO10 | 1.394E-05 | -0.200 | - | - |
| 8617 | ENSP00000405222 | DCAF8L1 | 1.147E-06 | -0.200 | - | - |
| 8618 | ENSP00000240343 | DYNLL2 | 2.136E-05 | -0.200 | - | - |
| 8619 | ENSP00000296824 | CCDC127 | 4.808E-05 | -0.200 | - | - |
| 8620 | ENSP00000418001 | GK5 | 2.757E-05 | -0.200 | - | - |
| 8621 | ENSP00000342295 | ANKRD55 | 2.164E-05 | -0.200 | - | - |
| 8622 | ENSP00000315118 | ADCK4 | 1.882E-05 | -0.200 | - | - |
| 8623 | ENSP00000037869 | FAM136A | 2.711E-05 | -0.200 | - | - |
| 8624 | ENSP00000206595 | G2E3 | 4.162E-05 | -0.200 | - | - |
| 8625 | ENSP00000313034 | FBXW5 | 2.534E-05 | -0.200 | - | - |
| 8626 | ENSP00000313885 | KIAA0195 | 1.955E-05 | -0.200 | - | - |
| 8627 | ENSP00000269221 | C18orf8 | 7.599E-05 | -0.200 | - | - |
| 8628 | ENSP00000318313 | AMBRA1 | 2.177E-05 | -0.200 | - | - |
| 8629 | ENSP00000318131 | PNMAL1 | 2.730E-05 | -0.200 | - | - |
| 8630 | ENSP00000267202 | VPS37B | 2.071E-05 | -0.200 | - | - |
| 8631 | ENSP00000251089 | ANGEL1 | 2.838E-05 | -0.200 | - | - |
| 8632 | ENSP00000326924 | SCAPER | 2.948E-05 | -0.200 | - | - |
| 8633 | ENSP00000397881 | PET117 | 7.925E-06 | -0.200 | - | - |
| 8634 | ENSP00000228705 | PPM1H | 2.240E-05 | -0.200 | - | - |
| 8635 | ENSP00000320623 | RMND5B | 2.639E-05 | -0.200 | - | - |
| 8636 | ENSP00000306105 | CEP89 | 3.052E-05 | -0.200 | - | - |
| 8637 | ENSP00000300006 | C16orf45 | 3.302E-05 | -0.200 | - | - |
| 8638 | ENSP00000336724 | TBC1D22A | 2.298E-05 | -0.200 | - | - |
| 8639 | ENSP00000252530 | FAM98C | 2.538E-05 | -0.200 | - | - |
| 8640 | ENSP00000304133 | SCG2 | 3.279E-05 | -0.200 | - | - |
| 8641 | ENSP00000270474 | PDE4A | 1.879E-05 | -0.200 | - | - |
| 8642 | ENSP00000054650 | THAP3 | 3.870E-05 | -0.201 | - | - |
| 8643 | ENSP00000231524 | TRIM23 | 2.138E-05 | -0.201 | - | - |
| 8644 | ENSP00000330289 | TRAPPC6B | 1.621E-05 | -0.201 | - | - |
| 8645 | ENSP00000283632 | RMND5A | 3.941E-05 | -0.201 | - | - |
| 8646 | ENSP00000352955 | FAM3A | 2.916E-05 | -0.201 | - | - |
| 8647 | ENSP00000267023 | NABP2 | 2.036E-05 | -0.201 | - | - |
| 8648 | ENSP00000299259 | COPS2 | 2.214E-05 | -0.201 | - | - |
| 8649 | ENSP00000277010 | SIGMAR1 | 2.185E-05 | -0.201 | - | - |
| 8650 | ENSP00000262126 | ANKRD12 | 2.979E-05 | -0.201 | - | - |
| 8651 | ENSP00000258711 | CHST12 | 2.323E-05 | -0.201 | - | - |
| 8652 | ENSP00000237596 | PKD2 | 1.789E-05 | -0.201 | - | - |
| 8653 | ENSP00000383096 | RFPL2 | 2.231E-05 | -0.201 | - | - |
| 8654 | ENSP00000322343 | CDK20 | 1.965E-05 | -0.201 | - | - |
| 8655 | ENSP00000223366 | GCK | 1.552E-05 | -0.201 | - | - |
| 8656 | ENSP00000274498 | ARHGAP26 | 2.317E-05 | -0.201 | - | - |
| 8657 | ENSP00000297562 | AP5Z1 | 2.166E-05 | -0.201 | - | - |
| 8658 | ENSP00000233616 | MOGS | 2.128E-05 | -0.201 | - | - |
| 8659 | ENSP00000335605 | CMTM7 | 3.352E-05 | -0.201 | - | - |
| 8660 | ENSP00000337998 | NCKAP5L | 2.308E-04 | -0.201 | - | - |
| 8661 | ENSP00000311121 | PSMA8 | 2.470E-05 | -0.201 | - | - |
| 8662 | ENSP00000327716 | WRB | 2.473E-05 | -0.201 | - | - |
| 8663 | ENSP00000301765 | VPS37C | 2.082E-05 | -0.201 | - | - |
| 8664 | ENSP00000287008 | PCDH1 | 3.172E-05 | -0.201 | - | - |
| 8665 | ENSP00000308717 | MRPL48 | 1.878E-05 | -0.201 | - | - |
| 8666 | ENSP00000261499 | B9D1 | 2.741E-05 | -0.201 | - | - |
| 8667 | ENSP00000346964 | EGFLAM | 2.435E-05 | -0.201 | - | - |
| 8668 | ENSP00000344155 | ABCA2 | 2.930E-05 | -0.201 | - | - |
| 8669 | ENSP00000223127 | PLOD3 | 2.664E-05 | -0.201 | - | - |
| 8670 | ENSP00000340297 | ARHGEF10 | 1.930E-05 | -0.201 | - | - |
| 8671 | ENSP00000271375 | SFT2D2 | 1.617E-05 | -0.201 | - | - |
| 8672 | ENSP00000297056 | DAGLB | 2.325E-05 | -0.201 | - | - |
| 8673 | ENSP00000336914 | FGD3 | 2.326E-05 | -0.201 | - | - |
| 8674 | ENSP00000319678 | ST5 | 2.605E-05 | -0.201 | - | - |
| 8675 | ENSP00000271417 | ILDR2 | 2.871E-05 | -0.201 | - | - |
| 8676 | ENSP00000303992 | TMEM43 | 3.360E-05 | -0.201 | - | - |
| 8677 | ENSP00000286621 | ADK | 2.016E-05 | -0.201 | - | - |
| 8678 | ENSP00000321546 | LOH12CR1 | 2.641E-05 | -0.201 | - | - |
| 8679 | ENSP00000313581 | KLK2 | 3.254E-05 | -0.201 | - | - |
| 8680 | ENSP00000337289 | CENPQ | 2.422E-05 | -0.201 | - | - |
| 8681 | ENSP00000268711 | MED9 | 2.603E-05 | -0.201 | - | - |
| 8682 | ENSP00000246117 | NCLN | 2.644E-05 | -0.201 | - | - |
| 8683 | ENSP00000405202 | SPANXB2 | 2.189E-05 | -0.201 | - | - |
| 8684 | ENSP00000465655 | NFIC | 2.403E-05 | -0.201 | - | - |
| 8685 | ENSP00000241502 | FYTTD1 | 2.861E-05 | -0.201 | - | - |
| 8686 | ENSP00000362441 | ATRX | 2.980E-05 | -0.201 | - | - |
| 8687 | ENSP00000310785 | MRPS22 | 2.816E-05 | -0.201 | - | - |
| 8688 | ENSP00000253048 | ZC3H4 | 2.845E-05 | -0.201 | - | - |
| 8689 | ENSP00000258416 | EIF4E2 | 2.215E-05 | -0.201 | - | - |
| 8690 | ENSP00000254928 | ERAL1 | 1.981E-05 | -0.201 | - | - |
| 8691 | ENSP00000355747 | PSEN2 | 4.312E-05 | -0.201 | - | - |
| 8692 | ENSP00000237822 | C2orf43 | 3.178E-05 | -0.201 | - | - |
| 8693 | ENSP00000222120 | RAB3D | 2.615E-05 | -0.201 | - | - |
| 8694 | ENSP00000304376 | RAB24 | 2.685E-05 | -0.201 | - | - |
| 8695 | ENSP00000357288 | LAMTOR2 | 3.061E-05 | -0.201 | - | - |
| 8696 | ENSP00000266604 | LLPH | 2.008E-05 | -0.201 | - | - |
| 8697 | ENSP00000278742 | ST14 | 2.139E-05 | -0.201 | - | - |
| 8698 | ENSP00000219172 | CENPT | 2.034E-05 | -0.201 | - | - |
| 8699 | ENSP00000417711 | FAM205A | 2.833E-07 | -0.201 | - | - |
| 8700 | ENSP00000270708 | WRAP73 | 2.756E-05 | -0.201 | - | - |
| 8701 | ENSP00000359382 | PASD1 | 3.428E-05 | -0.201 | - | - |
| 8702 | ENSP00000336888 | SLC44A2 | 2.752E-05 | -0.201 | - | - |
| 8703 | ENSP00000301030 | ANKRD11 | 4.073E-05 | -0.201 | - | - |
| 8704 | ENSP00000339057 | GPRASP2 | 1.685E-05 | -0.201 | - | - |
| 8705 | ENSP00000358719 | ME1 | 2.358E-05 | -0.201 | - | - |
| 8706 | ENSP00000325290 | GSG2 | 2.492E-05 | -0.201 | - | - |
| 8707 | ENSP00000262861 | SIPA1L2 | 7.060E-05 | -0.201 | - | - |
| 8708 | ENSP00000365899 | VSX1 | 2.209E-05 | -0.201 | - | - |
| 8709 | ENSP00000258398 | TTLL4 | 2.002E-05 | -0.201 | - | - |
| 8710 | ENSP00000263202 | UFD1L | 2.435E-05 | -0.201 | - | - |
| 8711 | ENSP00000383933 | FAM19A5 | 9.789E-06 | -0.201 | - | - |
| 8712 | ENSP00000216832 | PNN | 2.888E-05 | -0.201 | - | - |
| 8713 | ENSP00000354541 | NLGN1 | 1.443E-05 | -0.201 | - | - |
| 8714 | ENSP00000307214 | LCMT2 | 2.244E-05 | -0.201 | - | - |
| 8715 | ENSP00000207437 | MYL6B | 1.758E-05 | -0.201 | - | - |
| 8716 | ENSP00000238341 | CNTRL | 2.255E-05 | -0.201 | - | - |
| 8717 | ENSP00000368927 | EIF1AX | 9.846E-06 | -0.201 | - | - |
| 8718 | ENSP00000396445 | OSGIN2 | 1.064E-05 | -0.201 | - | - |
| 8719 | ENSP00000248566 | SHFM1 | 2.386E-05 | -0.201 | - | - |
| 8720 | ENSP00000307833 | FBXO22 | 2.131E-05 | -0.201 | - | - |
| 8721 | ENSP00000431538 | MPZ | 2.293E-05 | -0.201 | - | - |
| 8722 | ENSP00000353458 | BAZ1A | 1.395E-05 | -0.201 | - | - |
| 8723 | ENSP00000221232 | CNOT3 | 1.852E-05 | -0.201 | - | - |
| 8724 | ENSP00000332013 | ZGPAT | 2.791E-05 | -0.201 | - | - |
| 8725 | ENSP00000222307 | KXD1 | 2.819E-05 | -0.201 | - | - |
| 8726 | ENSP00000307746 | ZNF608 | 2.525E-05 | -0.201 | - | - |
| 8727 | ENSP00000303758 | LUZP1 | 2.442E-05 | -0.201 | - | - |
| 8728 | ENSP00000342071 | LIPT1 | 2.488E-05 | -0.201 | - | - |
| 8729 | ENSP00000308466 | PBLD | 2.479E-05 | -0.201 | - | - |
| 8730 | ENSP00000220853 | EMC2 | 2.145E-05 | -0.201 | - | - |
| 8731 | ENSP00000271234 | FNBP1L | 2.327E-05 | -0.201 | - | - |
| 8732 | ENSP00000321347 | STRBP | 1.880E-05 | -0.201 | - | - |
| 8733 | ENSP00000271227 | SLC44A3 | 2.421E-05 | -0.201 | - | - |
| 8734 | ENSP00000326219 | DHRS4 | 2.368E-05 | -0.201 | - | - |
| 8735 | ENSP00000364041 | SPIN2A | 2.266E-05 | -0.201 | - | - |
| 8736 | ENSP00000356825 | ADCY10 | 4.670E-05 | -0.201 | - | - |
| 8737 | ENSP00000267889 | DISP2 | 3.226E-05 | -0.201 | - | - |
| 8738 | ENSP00000354553 | C1orf85 | 8.096E-06 | -0.201 | - | - |
| 8739 | ENSP00000341963 | RSC1A1 | 2.766E-05 | -0.201 | - | - |
| 8740 | ENSP00000311095 | CYP4A11 | 3.005E-05 | -0.201 | - | - |
| 8741 | ENSP00000263773 | FNBP4 | 2.416E-05 | -0.201 | - | - |
| 8742 | ENSP00000282251 | CWF19L2 | 2.950E-05 | -0.201 | - | - |
| 8743 | ENSP00000225972 | LRRC59 | 1.907E-05 | -0.201 | - | - |
| 8744 | ENSP00000344299 | PTAR1 | 2.567E-05 | -0.201 | - | - |
| 8745 | ENSP00000338352 | NUDT4 | 6.576E-05 | -0.201 | - | - |
| 8746 | ENSP00000348722 | PUS7 | 1.471E-05 | -0.201 | - | - |
| 8747 | ENSP00000222284 | TMEM147 | 2.236E-05 | -0.201 | - | - |
| 8748 | ENSP00000381490 | LARP4 | 1.515E-05 | -0.201 | - | - |
| 8749 | ENSP00000262300 | PKMYT1 | 2.981E-05 | -0.201 | - | - |
| 8750 | ENSP00000339382 | PIGO | 1.337E-05 | -0.201 | - | - |
| 8751 | ENSP00000357909 | SEMA6C | 2.299E-05 | -0.201 | - | - |
| 8752 | ENSP00000261655 | RIMBP2 | 1.593E-05 | -0.201 | - | - |
| 8753 | ENSP00000249066 | APOL2 | 2.339E-05 | -0.201 | - | - |
| 8754 | ENSP00000257696 | HILPDA | 2.730E-05 | -0.201 | - | - |
| 8755 | ENSP00000296597 | NDUFAF2 | 1.968E-05 | -0.201 | - | - |
| 8756 | ENSP00000304783 | SLC26A5 | 2.018E-05 | -0.201 | - | - |
| 8757 | ENSP00000300291 | NUDT21 | 2.805E-05 | -0.201 | - | - |
| 8758 | ENSP00000304286 | FOXI1 | 1.196E-05 | -0.201 | - | - |
| 8759 | ENSP00000310796 | THAP2 | 3.902E-05 | -0.201 | - | - |
| 8760 | ENSP00000249636 | PIAS1 | 5.891E-05 | -0.201 | - | - |
| 8761 | ENSP00000295453 | ALPPL2 | 1.991E-05 | -0.201 | - | - |
| 8762 | ENSP00000300249 | MAPRE2 | 2.103E-05 | -0.201 | - | - |
| 8763 | ENSP00000307705 | HIST1H1E | 1.785E-05 | -0.201 | - | - |
| 8764 | ENSP00000217426 | AHCY | 1.672E-05 | -0.201 | - | - |
| 8765 | ENSP00000369965 | BTF3 | 6.921E-06 | -0.201 | - | - |
| 8766 | ENSP00000365906 | ZNF615 | 4.926E-05 | -0.201 | - | - |
| 8767 | ENSP00000248272 | GAN | 2.214E-05 | -0.201 | - | - |
| 8768 | ENSP00000338885 | C2CD2L | 2.175E-05 | -0.201 | - | - |
| 8769 | ENSP00000303058 | CEP120 | 3.195E-05 | -0.201 | - | - |
| 8770 | ENSP00000263126 | AKR1C4 | 2.256E-05 | -0.201 | - | - |
| 8771 | ENSP00000228136 | C11orf58 | 3.491E-05 | -0.201 | - | - |
| 8772 | ENSP00000402153 | IQCJ | 3.500E-06 | -0.201 | - | - |
| 8773 | ENSP00000272643 | THSD7B | 2.877E-05 | -0.201 | - | - |
| 8774 | ENSP00000372023 | CHEK2 | 5.988E-05 | -0.201 | - | - |
| 8775 | ENSP00000261600 | THOC1 | 2.305E-05 | -0.201 | - | - |
| 8776 | ENSP00000261741 | RBM19 | 1.773E-05 | -0.201 | - | - |
| 8777 | ENSP00000227135 | SPA17 | 1.826E-05 | -0.201 | - | - |
| 8778 | ENSP00000307240 | KRT74 | 3.975E-05 | -0.201 | - | - |
| 8779 | ENSP00000309532 | TP53I11 | 2.217E-05 | -0.201 | - | - |
| 8780 | ENSP00000310110 | TMEM255A | 1.049E-05 | -0.201 | - | - |
| 8781 | ENSP00000401947 | ZNF223 | 9.398E-06 | -0.201 | - | - |
| 8782 | ENSP00000267540 | STON2 | 2.111E-05 | -0.201 | - | - |
| 8783 | ENSP00000243344 | TTC21B | 1.992E-05 | -0.201 | - | - |
| 8784 | ENSP00000243918 | SYS1 | 2.150E-05 | -0.201 | - | - |
| 8785 | ENSP00000359290 | DR1 | 5.667E-05 | -0.201 | - | - |
| 8786 | ENSP00000257262 | TMEM258 | 2.531E-05 | -0.201 | - | - |
| 8787 | ENSP00000372276 | KRTAP20-3 | 3.748E-07 | -0.201 | - | - |
| 8788 | ENSP00000353104 | DHDDS | 2.459E-05 | -0.201 | - | - |
| 8789 | ENSP00000238618 | ACYP1 | 3.099E-05 | -0.201 | - | - |
| 8790 | ENSP00000342952 | ADCY2 | 4.160E-05 | -0.201 | - | - |
| 8791 | ENSP00000367727 | PANK4 | 3.095E-05 | -0.202 | - | - |
| 8792 | ENSP00000323339 | C2CD3 | 1.490E-05 | -0.202 | - | - |
| 8793 | ENSP00000368976 | KIFC3 | 1.318E-05 | -0.202 | - | - |
| 8794 | ENSP00000317780 | COX5A | 2.982E-05 | -0.202 | - | - |
| 8795 | ENSP00000304858 | ORMDL3 | 1.221E-04 | -0.202 | - | - |
| 8796 | ENSP00000307046 | SDC2 | 7.060E-05 | -0.202 | - | - |
| 8797 | ENSP00000256545 | EMC7 | 3.239E-05 | -0.202 | - | - |
| 8798 | ENSP00000337159 | ZFYVE16 | 1.845E-05 | -0.202 | - | - |
| 8799 | ENSP00000352257 | XRCC6 | 3.404E-05 | -0.202 | - | - |
| 8800 | ENSP00000263150 | WDR37 | 2.593E-05 | -0.202 | - | - |
| 8801 | ENSP00000040663 | MRI1 | 1.756E-05 | -0.202 | - | - |
| 8802 | ENSP00000328062 | KIAA1033 | 1.794E-05 | -0.202 | - | - |
| 8803 | ENSP00000354341 | MEPE | 4.173E-05 | -0.202 | - | - |
| 8804 | ENSP00000313159 | FBXO34 | 2.737E-05 | -0.202 | - | - |
| 8805 | ENSP00000002596 | HS3ST1 | 1.955E-05 | -0.202 | - | - |
| 8806 | ENSP00000285599 | MAN2B2 | 1.828E-05 | -0.202 | - | - |
| 8807 | ENSP00000329040 | FAM174B | 2.038E-05 | -0.202 | - | - |
| 8808 | ENSP00000280325 | C11orf53 | 3.829E-05 | -0.202 | - | - |
| 8809 | ENSP00000219240 | DHODH | 1.416E-05 | -0.202 | - | - |
| 8810 | ENSP00000311493 | STON1-GTF2A1L | 1.749E-05 | -0.202 | - | - |
| 8811 | ENSP00000240488 | MND1 | 2.069E-05 | -0.202 | - | - |
| 8812 | ENSP00000326603 | SMCHD1 | 2.514E-05 | -0.202 | - | - |
| 8813 | ENSP00000279036 | PIGT | 1.782E-05 | -0.202 | - | - |
| 8814 | ENSP00000290208 | MRPL10 | 1.753E-05 | -0.202 | - | - |
| 8815 | ENSP00000454241 | ZNF747 | 1.110E-05 | -0.202 | - | - |
| 8816 | ENSP00000355304 | MOK | 9.256E-06 | -0.202 | - | - |
| 8817 | ENSP00000317333 | NEUROG2 | 3.327E-05 | -0.202 | - | - |
| 8818 | ENSP00000336752 | FIP1L1 | 3.637E-05 | -0.202 | - | - |
| 8819 | ENSP00000237500 | MYL12B | 1.568E-05 | -0.202 | - | - |
| 8820 | ENSP00000366693 | FOXD4L6 | 9.482E-06 | -0.202 | - | - |
| 8821 | ENSP00000415027 | C16orf96 | 6.100E-07 | -0.202 | - | - |
| 8822 | ENSP00000341489 | SYDE1 | 2.583E-05 | -0.202 | - | - |
| 8823 | ENSP00000252992 | CEP85 | 2.784E-05 | -0.202 | - | - |
| 8824 | ENSP00000261609 | HERC2 | 2.263E-05 | -0.202 | - | - |
| 8825 | ENSP00000159087 | ANO8 | 1.883E-05 | -0.202 | - | - |
| 8826 | ENSP00000292176 | ZBTB7B | 3.421E-05 | -0.202 | - | - |
| 8827 | ENSP00000262580 | GSDMD | 2.781E-05 | -0.202 | - | - |
| 8828 | ENSP00000266126 | EIF2B2 | 1.127E-05 | -0.202 | - | - |
| 8829 | ENSP00000445626 | EGR4 | 3.559E-05 | -0.202 | - | - |
| 8830 | ENSP00000184266 | NDUFB4 | 1.871E-05 | -0.202 | - | - |
| 8831 | ENSP00000262033 | PTGES3 | 2.366E-05 | -0.202 | - | - |
| 8832 | ENSP00000258874 | MTHFS | 1.782E-05 | -0.202 | - | - |
| 8833 | ENSP00000332656 | ENPP7 | 1.778E-05 | -0.202 | - | - |
| 8834 | ENSP00000443194 | NAT1 | 2.313E-05 | -0.202 | - | - |
| 8835 | ENSP00000331843 | NPIP | 1.461E-05 | -0.202 | - | - |
| 8836 | ENSP00000023939 | RTFDC1 | 3.473E-05 | -0.202 | - | - |
| 8837 | ENSP00000247026 | NSRP1 | 3.112E-05 | -0.202 | - | - |
| 8838 | ENSP00000312606 | AKR1A1 | 2.522E-05 | -0.202 | - | - |
| 8839 | ENSP00000357323 | YY1AP1 | 2.462E-05 | -0.202 | - | - |
| 8840 | ENSP00000225927 | NAGLU | 1.955E-05 | -0.202 | - | - |
| 8841 | ENSP00000319992 | TMEM11 | 1.727E-05 | -0.202 | - | - |
| 8842 | ENSP00000344566 | C21orf2 | 1.892E-05 | -0.202 | - | - |
| 8843 | ENSP00000233630 | PCGF1 | 2.129E-05 | -0.202 | - | - |
| 8844 | ENSP00000311453 | PDE8A | 2.004E-05 | -0.202 | - | - |
| 8845 | ENSP00000261308 | PPWD1 | 1.984E-05 | -0.202 | - | - |
| 8846 | ENSP00000258383 | MRPL44 | 1.675E-05 | -0.202 | - | - |
| 8847 | ENSP00000259050 | 7-Mar | 1.982E-05 | -0.202 | - | - |
| 8848 | ENSP00000261811 | CYSTM1 | 2.139E-05 | -0.202 | - | - |
| 8849 | ENSP00000256255 | TMEM66 | 2.889E-05 | -0.202 | - | - |
| 8850 | ENSP00000341848 | GOLGB1 | 1.699E-05 | -0.202 | - | - |
| 8851 | ENSP00000343742 | BROX | 1.814E-05 | -0.202 | - | - |
| 8852 | ENSP00000332679 | CYP2A13 | 3.567E-05 | -0.202 | - | - |
| 8853 | ENSP00000219837 | KNOP1 | 3.174E-05 | -0.202 | - | - |
| 8854 | ENSP00000324074 | ANP32E | 2.392E-05 | -0.202 | - | - |
| 8855 | ENSP00000313121 | SNX14 | 2.788E-05 | -0.202 | - | - |
| 8856 | ENSP00000266517 | ETNK1 | 2.479E-05 | -0.202 | - | - |
| 8857 | ENSP00000235345 | SLC35D1 | 1.975E-05 | -0.202 | - | - |
| 8858 | ENSP00000215862 | MORC2 | 2.136E-05 | -0.202 | - | - |
| 8859 | ENSP00000312395 | MRPS21 | 1.683E-05 | -0.202 | - | - |
| 8860 | ENSP00000354592 | ZNF384 | 2.308E-05 | -0.202 | - | - |
| 8861 | ENSP00000293371 | DCD | 2.696E-05 | -0.202 | - | - |
| 8862 | ENSP00000262094 | RAB27B | 1.306E-05 | -0.202 | - | - |
| 8863 | ENSP00000300026 | PPIB | 2.572E-05 | -0.202 | - | - |
| 8864 | ENSP00000259253 | UGGT1 | 2.456E-05 | -0.202 | - | - |
| 8865 | ENSP00000267568 | PTGR2 | 2.307E-05 | -0.202 | - | - |
| 8866 | ENSP00000194130 | SLC13A1 | 1.732E-05 | -0.202 | - | - |
| 8867 | ENSP00000352738 | G3BP2 | 1.866E-05 | -0.202 | - | - |
| 8868 | ENSP00000301785 | HNRNPUL2 | 1.636E-05 | -0.202 | - | - |
| 8869 | ENSP00000324318 | TRAPPC12 | 1.800E-05 | -0.202 | - | - |
| 8870 | ENSP00000361608 | ABCC10 | 1.273E-05 | -0.202 | - | - |
| 8871 | ENSP00000295225 | KCNIP3 | 1.640E-05 | -0.202 | - | - |
| 8872 | ENSP00000221476 | CKM | 6.817E-05 | -0.202 | - | - |
| 8873 | ENSP00000284292 | NRGN | 1.631E-05 | -0.202 | - | - |
| 8874 | ENSP00000320924 | WIPF2 | 1.993E-05 | -0.202 | - | - |
| 8875 | ENSP00000288774 | PEX10 | 3.491E-05 | -0.202 | - | - |
| 8876 | ENSP00000314151 | KLK3 | 3.514E-05 | -0.202 | - | - |
| 8877 | ENSP00000249269 | PMPCB | 1.826E-05 | -0.202 | - | - |
| 8878 | ENSP00000304353 | POP7 | 1.729E-05 | -0.202 | - | - |
| 8879 | ENSP00000310129 | PSMD2 | 2.534E-05 | -0.202 | - | - |
| 8880 | ENSP00000276533 | GINS4 | 1.783E-05 | -0.202 | - | - |
| 8881 | ENSP00000373485 | TSNAXIP1 | 1.051E-05 | -0.202 | - | - |
| 8882 | ENSP00000263774 | NDUFS3 | 1.948E-05 | -0.202 | - | - |
| 8883 | ENSP00000299563 | RNF169 | 3.511E-05 | -0.202 | - | - |
| 8884 | ENSP00000298292 | DNAAF2 | 1.578E-05 | -0.202 | - | - |
| 8885 | ENSP00000216187 | FOXRED2 | 1.669E-05 | -0.202 | - | - |
| 8886 | ENSP00000323837 | SLC4A1AP | 2.451E-05 | -0.202 | - | - |
| 8887 | ENSP00000252050 | CUL9 | 4.608E-05 | -0.202 | - | - |
| 8888 | ENSP00000325785 | SPECC1L | 1.663E-05 | -0.202 | - | - |
| 8889 | ENSP00000302114 | PRELID1 | 2.259E-05 | -0.202 | - | - |
| 8890 | ENSP00000272238 | ATP6V1C2 | 1.155E-05 | -0.202 | - | - |
| 8891 | ENSP00000305958 | STIP1 | 2.201E-05 | -0.202 | - | - |
| 8892 | ENSP00000262394 | WSB1 | 2.021E-05 | -0.202 | - | - |
| 8893 | ENSP00000264156 | MCM6 | 1.610E-05 | -0.202 | - | - |
| 8894 | ENSP00000243326 | RIF1 | 2.165E-05 | -0.202 | - | - |
| 8895 | ENSP00000312697 | DMAP1 | 2.615E-05 | -0.202 | - | - |
| 8896 | ENSP00000378503 | ZBTB9 | 1.744E-05 | -0.202 | - | - |
| 8897 | ENSP00000342007 | CYP1A2 | 3.382E-05 | -0.202 | - | - |
| 8898 | ENSP00000350720 | SMARCA4 | 4.582E-05 | -0.202 | - | - |
| 8899 | ENSP00000306261 | KRT78 | 2.172E-05 | -0.202 | - | - |
| 8900 | ENSP00000269127 | C17orf64 | 1.709E-05 | -0.202 | - | - |
| 8901 | ENSP00000365019 | SPIN1 | 2.096E-05 | -0.202 | - | - |
| 8902 | ENSP00000299335 | COX11 | 1.811E-05 | -0.202 | - | - |
| 8903 | ENSP00000254663 | SCLY | 3.031E-05 | -0.202 | - | - |
| 8904 | ENSP00000227525 | TMEM109 | 2.303E-05 | -0.202 | - | - |
| 8905 | ENSP00000261844 | FAM214A | 3.297E-05 | -0.202 | - | - |
| 8906 | ENSP00000259698 | FAM65B | 1.783E-05 | -0.202 | - | - |
| 8907 | ENSP00000349679 | DYNLRB1 | 2.082E-05 | -0.202 | - | - |
| 8908 | ENSP00000262577 | ZC3H3 | 1.887E-05 | -0.202 | - | - |
| 8909 | ENSP00000218439 | MAGED2 | 1.989E-05 | -0.202 | - | - |
| 8910 | ENSP00000002125 | NDUFAF7 | 1.543E-05 | -0.202 | - | - |
| 8911 | ENSP00000358965 | C1orf194 | 3.921E-06 | -0.202 | - | - |
| 8912 | ENSP00000220496 | DNAJC17 | 1.862E-05 | -0.202 | - | - |
| 8913 | ENSP00000297668 | CNTNAP3 | 1.891E-05 | -0.202 | - | - |
| 8914 | ENSP00000221480 | PEX11G | 3.206E-05 | -0.202 | - | - |
| 8915 | ENSP00000341364 | TMEM230 | 2.557E-05 | -0.202 | - | - |
| 8916 | ENSP00000220763 | CPQ | 2.058E-05 | -0.202 | - | - |
| 8917 | ENSP00000310842 | FANCI | 1.979E-05 | -0.202 | - | - |
| 8918 | ENSP00000246174 | ARMCX5 | 1.910E-05 | -0.202 | - | - |
| 8919 | ENSP00000261475 | PPP2R3C | 1.798E-05 | -0.202 | - | - |
| 8920 | ENSP00000269886 | SH3GL1 | 2.122E-05 | -0.202 | - | - |
| 8921 | ENSP00000342015 | COX19 | 1.842E-05 | -0.203 | - | - |
| 8922 | ENSP00000222482 | CPA4 | 1.910E-05 | -0.203 | - | - |
| 8923 | ENSP00000256190 | SBF2 | 2.077E-05 | -0.203 | - | - |
| 8924 | ENSP00000362850 | DSN1 | 2.502E-05 | -0.203 | - | - |
| 8925 | ENSP00000333277 | HIST2H3D | 2.088E-05 | -0.203 | - | - |
| 8926 | ENSP00000342905 | ADNP | 2.817E-05 | -0.203 | - | - |
| 8927 | ENSP00000324392 | RNF213 | 3.230E-05 | -0.203 | - | - |
| 8928 | ENSP00000261819 | ANAPC5 | 2.497E-05 | -0.203 | - | - |
| 8929 | ENSP00000418813 | SELK | 8.149E-05 | -0.203 | - | - |
| 8930 | ENSP00000308080 | GTF2IRD2B | 7.387E-05 | -0.203 | - | - |
| 8931 | ENSP00000264779 | GPBP1 | 2.301E-05 | -0.203 | - | - |
| 8932 | ENSP00000368648 | SLC30A6 | 5.656E-06 | -0.203 | - | - |
| 8933 | ENSP00000295713 | SRGAP2 | 1.889E-05 | -0.203 | - | - |
| 8934 | ENSP00000304843 | - | 2.029E-05 | -0.203 | - | - |
| 8935 | ENSP00000223136 | FIS1 | 2.191E-05 | -0.203 | - | - |
| 8936 | ENSP00000220514 | OIP5 | 1.792E-05 | -0.203 | - | - |
| 8937 | ENSP00000333401 | MRPL40 | 1.266E-05 | -0.203 | - | - |
| 8938 | ENSP00000243903 | ACTR5 | 2.186E-05 | -0.203 | - | - |
| 8939 | ENSP00000228027 | DGAT2 | 1.998E-05 | -0.203 | - | - |
| 8940 | ENSP00000277165 | FAM120A | 2.016E-05 | -0.203 | - | - |
| 8941 | ENSP00000220822 | GDAP1 | 1.884E-05 | -0.203 | - | - |
| 8942 | ENSP00000366028 | KLK9 | 2.325E-05 | -0.203 | - | - |
| 8943 | ENSP00000256447 | CD180 | 4.128E-05 | -0.203 | - | - |
| 8944 | ENSP00000278174 | BTBD10 | 2.117E-05 | -0.203 | - | - |
| 8945 | ENSP00000353735 | TOPORS | 3.089E-05 | -0.203 | - | - |
| 8946 | ENSP00000265537 | LARS2 | 2.035E-05 | -0.203 | - | - |
| 8947 | ENSP00000311368 | POLD4 | 2.419E-05 | -0.203 | - | - |
| 8948 | ENSP00000265748 | ANLN | 2.100E-05 | -0.203 | - | - |
| 8949 | ENSP00000324729 | SAV1 | 4.191E-05 | -0.203 | - | - |
| 8950 | ENSP00000340505 | SENP8 | 4.744E-05 | -0.203 | - | - |
| 8951 | ENSP00000307077 | METTL18 | 1.966E-05 | -0.203 | - | - |
| 8952 | ENSP00000229340 | RAB35 | 2.461E-05 | -0.203 | - | - |
| 8953 | ENSP00000331983 | LRRC16A | 2.024E-05 | -0.203 | - | - |
| 8954 | ENSP00000262482 | RNF167 | 1.737E-05 | -0.203 | - | - |
| 8955 | ENSP00000310686 | FBXW8 | 2.170E-05 | -0.203 | - | - |
| 8956 | ENSP00000244711 | MEA1 | 1.822E-05 | -0.203 | - | - |
| 8957 | ENSP00000296411 | METAP1 | 1.976E-05 | -0.203 | - | - |
| 8958 | ENSP00000406318 | ZNF880 | 9.437E-06 | -0.203 | - | - |
| 8959 | ENSP00000341342 | KLHL6 | 1.789E-05 | -0.203 | - | - |
| 8960 | ENSP00000278353 | HSD17B12 | 2.206E-05 | -0.203 | - | - |
| 8961 | ENSP00000267584 | AK7 | 2.022E-05 | -0.203 | - | - |
| 8962 | ENSP00000364243 | PLA2G2F | 2.583E-05 | -0.203 | - | - |
| 8963 | ENSP00000309163 | RCE1 | 1.571E-05 | -0.203 | - | - |
| 8964 | ENSP00000251776 | ROPN1B | 5.165E-05 | -0.203 | - | - |
| 8965 | ENSP00000263036 | OPTN | 4.349E-05 | -0.203 | - | - |
| 8966 | ENSP00000256194 | MICAL2 | 1.896E-05 | -0.203 | - | - |
| 8967 | ENSP00000254108 | FUS | 3.433E-05 | -0.203 | - | - |
| 8968 | ENSP00000228495 | KCTD10 | 1.411E-05 | -0.203 | - | - |
| 8969 | ENSP00000262507 | COQ9 | 1.762E-05 | -0.203 | - | - |
| 8970 | ENSP00000305442 | COG7 | 2.169E-05 | -0.203 | - | - |
| 8971 | ENSP00000261700 | C14orf166 | 2.285E-05 | -0.203 | - | - |
| 8972 | ENSP00000217446 | PIGU | 2.141E-05 | -0.203 | - | - |
| 8973 | ENSP00000261776 | VAC14 | 1.878E-05 | -0.203 | - | - |
| 8974 | ENSP00000324491 | CHMP7 | 2.112E-05 | -0.203 | - | - |
| 8975 | ENSP00000470209 | ZNF257 | 9.039E-06 | -0.203 | - | - |
| 8976 | ENSP00000316532 | TMEM132E | 2.015E-05 | -0.203 | - | - |
| 8977 | ENSP00000326858 | NT5DC1 | 2.595E-05 | -0.203 | - | - |
| 8978 | ENSP00000306627 | SLC9C1 | 1.841E-05 | -0.203 | - | - |
| 8979 | ENSP00000353089 | SRSF2 | 2.854E-05 | -0.203 | - | - |
| 8980 | ENSP00000345096 | IMPDH1 | 2.559E-05 | -0.203 | - | - |
| 8981 | ENSP00000268379 | UQCRC2 | 2.282E-05 | -0.203 | - | - |
| 8982 | ENSP00000377617 | MTHFD2 | 1.294E-05 | -0.203 | - | - |
| 8983 | ENSP00000375229 | - | 3.719E-05 | -0.203 | - | - |
| 8984 | ENSP00000244534 | HIST1H1D | 1.572E-05 | -0.203 | - | - |
| 8985 | ENSP00000321427 | KCNH5 | 1.802E-05 | -0.203 | - | - |
| 8986 | ENSP00000251993 | KIAA0930 | 2.513E-05 | -0.203 | - | - |
| 8987 | ENSP00000235932 | RAB7L1 | 1.801E-05 | -0.203 | - | - |
| 8988 | ENSP00000265271 | RBM27 | 2.038E-05 | -0.203 | - | - |
| 8989 | ENSP00000243056 | HOXC13 | 1.610E-05 | -0.203 | - | - |
| 8990 | ENSP00000298351 | TMEM63C | 1.881E-05 | -0.203 | - | - |
| 8991 | ENSP00000240691 | TAS2R9 | 9.248E-05 | -0.203 | - | - |
| 8992 | ENSP00000330945 | TMED9 | 1.717E-05 | -0.203 | - | - |
| 8993 | ENSP00000335246 | ENTPD5 | 1.192E-05 | -0.203 | - | - |
| 8994 | ENSP00000293406 | LSM12 | 1.960E-05 | -0.203 | - | - |
| 8995 | ENSP00000295702 | SSR2 | 1.412E-05 | -0.203 | - | - |
| 8996 | ENSP00000303482 | REEP4 | 1.810E-05 | -0.203 | - | - |
| 8997 | ENSP00000282032 | ARL14EP | 2.248E-05 | -0.203 | - | - |
| 8998 | ENSP00000307479 | ARNT2 | 2.201E-05 | -0.203 | - | - |
| 8999 | ENSP00000266214 | SCARF2 | 1.493E-05 | -0.203 | - | - |
| 9000 | ENSP00000222553 | NAMPT | 7.566E-05 | -0.203 | - | - |
| 9001 | ENSP00000303222 | MTCH2 | 1.489E-05 | -0.203 | - | - |
| 9002 | ENSP00000317332 | SNX18 | 3.260E-05 | -0.203 | - | - |
| 9003 | ENSP00000328596 | SIX6 | 2.466E-05 | -0.203 | - | - |
| 9004 | ENSP00000228284 | SART3 | 1.551E-05 | -0.203 | - | - |
| 9005 | ENSP00000333947 | CHST15 | 2.726E-05 | -0.203 | - | - |
| 9006 | ENSP00000294454 | SLC25A34 | 2.072E-05 | -0.203 | - | - |
| 9007 | ENSP00000360664 | BTF3L4 | 1.306E-05 | -0.203 | - | - |
| 9008 | ENSP00000261245 | MNAT1 | 1.921E-05 | -0.203 | - | - |
| 9009 | ENSP00000265641 | CPT1A | 1.782E-05 | -0.203 | - | - |
| 9010 | ENSP00000318900 | SOBP | 2.672E-05 | -0.203 | - | - |
| 9011 | ENSP00000308540 | TBC1D3 | 1.345E-05 | -0.203 | - | - |
| 9012 | ENSP00000296577 | ABCE1 | 1.988E-05 | -0.203 | - | - |
| 9013 | ENSP00000217372 | CDK5RAP1 | 1.686E-05 | -0.203 | - | - |
| 9014 | ENSP00000297283 | PGAM2 | 1.829E-05 | -0.203 | - | - |
| 9015 | ENSP00000357516 | TPM3 | 2.831E-05 | -0.203 | - | - |
| 9016 | ENSP00000275053 | MMS22L | 1.477E-05 | -0.203 | - | - |
| 9017 | ENSP00000296486 | AGXT2L1 | 2.253E-05 | -0.203 | - | - |
| 9018 | ENSP00000281543 | GUF1 | 1.714E-05 | -0.203 | - | - |
| 9019 | ENSP00000382840 | DDX3X | 3.403E-05 | -0.203 | - | - |
| 9020 | ENSP00000363348 | MNF1 | 1.968E-05 | -0.203 | - | - |
| 9021 | ENSP00000316596 | SLC38A9 | 2.239E-05 | -0.203 | - | - |
| 9022 | ENSP00000281017 | TMEM18 | 2.145E-05 | -0.203 | - | - |
| 9023 | ENSP00000262374 | ALG1 | 1.471E-05 | -0.203 | - | - |
| 9024 | ENSP00000262817 | TMEM59L | 2.301E-05 | -0.203 | - | - |
| 9025 | ENSP00000323782 | CCDC43 | 2.998E-05 | -0.203 | - | - |
| 9026 | ENSP00000287600 | PDE6D | 2.275E-05 | -0.203 | - | - |
| 9027 | ENSP00000295888 | WDFY3 | 3.516E-05 | -0.203 | - | - |
| 9028 | ENSP00000381009 | KRTAP10-9 | 3.042E-05 | -0.203 | - | - |
| 9029 | ENSP00000252250 | KRT6C | 1.722E-05 | -0.203 | - | - |
| 9030 | ENSP00000007390 | TSR3 | 2.237E-05 | -0.203 | - | - |
| 9031 | ENSP00000315702 | MOB4 | 2.147E-05 | -0.203 | - | - |
| 9032 | ENSP00000420405 | FSBP | 2.203E-05 | -0.203 | - | - |
| 9033 | ENSP00000238256 | FKBP15 | 2.131E-05 | -0.203 | - | - |
| 9034 | ENSP00000324302 | STXBP6 | 2.606E-05 | -0.203 | - | - |
| 9035 | ENSP00000039989 | TTC17 | 2.194E-05 | -0.203 | - | - |
| 9036 | ENSP00000306344 | TMEM135 | 1.723E-05 | -0.203 | - | - |
| 9037 | ENSP00000361868 | ZMAT1 | 5.318E-05 | -0.203 | - | - |
| 9038 | ENSP00000311768 | ZNF706 | 1.929E-05 | -0.203 | - | - |
| 9039 | ENSP00000262644 | IMPAD1 | 1.742E-05 | -0.203 | - | - |
| 9040 | ENSP00000400036 | PLA2G4C | 2.048E-05 | -0.203 | - | - |
| 9041 | ENSP00000377252 | MON2 | 3.116E-05 | -0.204 | - | - |
| 9042 | ENSP00000217971 | PGRMC1 | 2.565E-05 | -0.204 | - | - |
| 9043 | ENSP00000202017 | PDRG1 | 2.798E-05 | -0.204 | - | - |
| 9044 | ENSP00000279392 | HIRIP3 | 2.555E-05 | -0.204 | - | - |
| 9045 | ENSP00000331540 | ZNF570 | 3.484E-05 | -0.204 | - | - |
| 9046 | ENSP00000347178 | HSPB9 | 2.368E-05 | -0.204 | - | - |
| 9047 | ENSP00000471000 | ZNF616 | 8.902E-06 | -0.204 | - | - |
| 9048 | ENSP00000266581 | AMIGO2 | 2.125E-05 | -0.204 | - | - |
| 9049 | ENSP00000257552 | MSI1 | 2.361E-05 | -0.204 | - | - |
| 9050 | ENSP00000431202 | ZNF345 | 9.305E-06 | -0.204 | - | - |
| 9051 | ENSP00000359024 | NOLC1 | 1.765E-05 | -0.204 | - | - |
| 9052 | ENSP00000297440 | HEATR2 | 2.020E-05 | -0.204 | - | - |
| 9053 | ENSP00000159111 | KDM4B | 1.851E-05 | -0.204 | - | - |
| 9054 | ENSP00000231487 | SKP1 | 2.355E-05 | -0.204 | - | - |
| 9055 | ENSP00000338864 | RASGRP2 | 2.124E-05 | -0.204 | - | - |
| 9056 | ENSP00000341692 | DAP3 | 1.345E-05 | -0.204 | - | - |
| 9057 | ENSP00000245312 | SLC10A2 | 1.653E-05 | -0.204 | - | - |
| 9058 | ENSP00000295121 | WDR92 | 1.303E-05 | -0.204 | - | - |
| 9059 | ENSP00000263780 | CHMP2B | 1.632E-05 | -0.204 | - | - |
| 9060 | ENSP00000262319 | TELO2 | 1.553E-05 | -0.204 | - | - |
| 9061 | ENSP00000416707 | XKR6 | 3.886E-05 | -0.204 | - | - |
| 9062 | ENSP00000382895 | RPGRIP1 | 2.387E-05 | -0.204 | - | - |
| 9063 | ENSP00000263277 | EHD2 | 1.524E-05 | -0.204 | - | - |
| 9064 | ENSP00000252445 | ELOF1 | 3.309E-05 | -0.204 | - | - |
| 9065 | ENSP00000294360 | C1orf123 | 1.144E-05 | -0.204 | - | - |
| 9066 | ENSP00000269187 | SLC39A6 | 1.525E-05 | -0.204 | - | - |
| 9067 | ENSP00000223114 | MOGAT3 | 2.004E-05 | -0.204 | - | - |
| 9068 | ENSP00000313921 | MSRA | 1.360E-05 | -0.204 | - | - |
| 9069 | ENSP00000260045 | PRKRIR | 3.035E-05 | -0.204 | - | - |
| 9070 | ENSP00000320184 | MRPS23 | 1.181E-05 | -0.204 | - | - |
| 9071 | ENSP00000365991 | DNAJC3 | 2.582E-05 | -0.204 | - | - |
| 9072 | ENSP00000339245 | YTHDC1 | 3.919E-05 | -0.204 | - | - |
| 9073 | ENSP00000299299 | PCBD1 | 1.759E-05 | -0.204 | - | - |
| 9074 | ENSP00000303908 | TWF2 | 1.838E-05 | -0.204 | - | - |
| 9075 | ENSP00000309430 | PIGS | 1.752E-05 | -0.204 | - | - |
| 9076 | ENSP00000405032 | GSG1 | 6.055E-06 | -0.204 | - | - |
| 9077 | ENSP00000312458 | ARV1 | 1.486E-05 | -0.204 | - | - |
| 9078 | ENSP00000259873 | MRPS18B | 3.090E-05 | -0.204 | - | - |
| 9079 | ENSP00000385722 | INTS1 | 3.948E-05 | -0.204 | - | - |
| 9080 | ENSP00000279146 | AIP | 2.379E-05 | -0.204 | - | - |
| 9081 | ENSP00000331087 | TSSC4 | 6.700E-05 | -0.204 | - | - |
| 9082 | ENSP00000265471 | B3GAT3 | 1.669E-05 | -0.204 | - | - |
| 9083 | ENSP00000355323 | TEX28 | 8.548E-06 | -0.204 | - | - |
| 9084 | ENSP00000318604 | MAF1 | 2.288E-05 | -0.204 | - | - |
| 9085 | ENSP00000301962 | USP42 | 1.988E-05 | -0.204 | - | - |
| 9086 | ENSP00000272198 | PPFIA4 | 2.655E-05 | -0.204 | - | - |
| 9087 | ENSP00000301607 | EVPL | 5.375E-05 | -0.204 | - | - |
| 9088 | ENSP00000300452 | COQ4 | 2.956E-05 | -0.204 | - | - |
| 9089 | ENSP00000262812 | COPE | 1.898E-05 | -0.204 | - | - |
| 9090 | ENSP00000237937 | ZFAND5 | 2.059E-05 | -0.204 | - | - |
| 9091 | ENSP00000330219 | SYN3 | 1.722E-05 | -0.204 | - | - |
| 9092 | ENSP00000349748 | SFPQ | 2.177E-05 | -0.204 | - | - |
| 9093 | ENSP00000326031 | PPP1CA | 2.131E-05 | -0.204 | - | - |
| 9094 | ENSP00000341684 | CCDC15 | 6.019E-05 | -0.204 | - | - |
| 9095 | ENSP00000373066 | TXNRD3NB | 9.772E-06 | -0.204 | - | - |
| 9096 | ENSP00000324463 | YLPM1 | 2.504E-05 | -0.204 | - | - |
| 9097 | ENSP00000227495 | ST3GAL4 | 1.334E-05 | -0.204 | - | - |
| 9098 | ENSP00000287218 | ZFAND3 | 2.550E-05 | -0.204 | - | - |
| 9099 | ENSP00000261726 | CUX2 | 1.796E-05 | -0.204 | - | - |
| 9100 | ENSP00000258169 | MPHOSPH6 | 1.528E-05 | -0.204 | - | - |
| 9101 | ENSP00000217246 | MACROD2 | 1.898E-05 | -0.204 | - | - |
| 9102 | ENSP00000326671 | SLC47A2 | 1.276E-05 | -0.204 | - | - |
| 9103 | ENSP00000288670 | FMNL2 | 1.993E-05 | -0.204 | - | - |
| 9104 | ENSP00000329858 | TRMT12 | 1.580E-05 | -0.204 | - | - |
| 9105 | ENSP00000413152 | FKBP7 | 2.047E-05 | -0.204 | - | - |
| 9106 | ENSP00000419494 | RYBP | 2.817E-05 | -0.204 | - | - |
| 9107 | ENSP00000379678 | PGM5 | 2.398E-05 | -0.204 | - | - |
| 9108 | ENSP00000272610 | FAHD2B | 2.373E-05 | -0.204 | - | - |
| 9109 | ENSP00000309772 | CPED1 | 1.878E-05 | -0.204 | - | - |
| 9110 | ENSP00000387982 | WDR73 | 3.260E-05 | -0.204 | - | - |
| 9111 | ENSP00000226432 | CWH43 | 2.054E-05 | -0.204 | - | - |
| 9112 | ENSP00000249442 | MTX2 | 1.514E-05 | -0.204 | - | - |
| 9113 | ENSP00000356591 | SOAT1 | 2.602E-05 | -0.204 | - | - |
| 9114 | ENSP00000306670 | NUDT16L1 | 3.343E-05 | -0.204 | - | - |
| 9115 | ENSP00000265866 | HNRNPH3 | 2.123E-05 | -0.204 | - | - |
| 9116 | ENSP00000262432 | METTL2B | 3.451E-05 | -0.204 | - | - |
| 9117 | ENSP00000331201 | HGS | 2.979E-05 | -0.204 | - | - |
| 9118 | ENSP00000216121 | NIPSNAP1 | 1.773E-05 | -0.204 | - | - |
| 9119 | ENSP00000324549 | CYFIP1 | 2.870E-05 | -0.204 | - | - |
| 9120 | ENSP00000217429 | FAM83D | 1.646E-05 | -0.204 | - | - |
| 9121 | ENSP00000381932 | DYRK1A | 4.199E-05 | -0.204 | - | - |
| 9122 | ENSP00000261749 | ZFAND6 | 2.389E-05 | -0.204 | - | - |
| 9123 | ENSP00000300147 | ELFN2 | 1.422E-05 | -0.204 | - | - |
| 9124 | ENSP00000470770 | AC004466.1 | 6.915E-07 | -0.204 | - | - |
| 9125 | ENSP00000343764 | TTN | 2.393E-05 | -0.204 | - | - |
| 9126 | ENSP00000072869 | ADCK2 | 1.467E-05 | -0.204 | - | - |
| 9127 | ENSP00000454404 | TMEM170A | 1.649E-06 | -0.204 | - | - |
| 9128 | ENSP00000257700 | RINT1 | 1.244E-05 | -0.204 | - | - |
| 9129 | ENSP00000297954 | WNK2 | 1.956E-05 | -0.204 | - | - |
| 9130 | ENSP00000251038 | ZC3H14 | 1.916E-05 | -0.204 | - | - |
| 9131 | ENSP00000330918 | PGP | 3.788E-05 | -0.204 | - | - |
| 9132 | ENSP00000266014 | GLT8D1 | 2.168E-05 | -0.204 | - | - |
| 9133 | ENSP00000345341 | PTGES2 | 2.949E-05 | -0.204 | - | - |
| 9134 | ENSP00000395007 | ZNF746 | 2.501E-05 | -0.204 | - | - |
| 9135 | ENSP00000270583 | KLHDC4 | 1.229E-05 | -0.204 | - | - |
| 9136 | ENSP00000322316 | COQ7 | 1.586E-05 | -0.204 | - | - |
| 9137 | ENSP00000369530 | ALOX15B | 2.465E-05 | -0.204 | - | - |
| 9138 | ENSP00000280700 | NGLY1 | 2.237E-05 | -0.204 | - | - |
| 9139 | ENSP00000332646 | MFSD10 | 1.836E-05 | -0.204 | - | - |
| 9140 | ENSP00000261249 | TRMT5 | 1.056E-05 | -0.204 | - | - |
| 9141 | ENSP00000329554 | HIST1H3I | 2.058E-05 | -0.204 | - | - |
| 9142 | ENSP00000362372 | BRWD3 | 7.689E-06 | -0.204 | - | - |
| 9143 | ENSP00000221859 | POLR2I | 1.567E-05 | -0.204 | - | - |
| 9144 | ENSP00000267415 | TINF2 | 2.235E-05 | -0.204 | - | - |
| 9145 | ENSP00000399259 | FCHSD1 | 1.456E-05 | -0.204 | - | - |
| 9146 | ENSP00000262375 | DNAJA3 | 2.287E-05 | -0.204 | - | - |
| 9147 | ENSP00000336994 | CLPTM1 | 1.392E-05 | -0.204 | - | - |
| 9148 | ENSP00000346603 | ZBTB45 | 1.671E-05 | -0.204 | - | - |
| 9149 | ENSP00000358293 | C10orf118 | 4.765E-06 | -0.204 | - | - |
| 9150 | ENSP00000249007 | RFPL3 | 2.954E-05 | -0.204 | - | - |
| 9151 | ENSP00000261196 | SLCO1B3 | 2.314E-05 | -0.204 | - | - |
| 9152 | ENSP00000174653 | AP3M2 | 2.159E-05 | -0.204 | - | - |
| 9153 | ENSP00000322421 | HBA1 | 2.281E-05 | -0.204 | - | - |
| 9154 | ENSP00000282466 | IGSF10 | 1.870E-05 | -0.204 | - | - |
| 9155 | ENSP00000314619 | ZFAND2A | 2.522E-05 | -0.204 | - | - |
| 9156 | ENSP00000230321 | MDFI | 1.329E-05 | -0.204 | - | - |
| 9157 | ENSP00000248450 | AAMP | 1.730E-05 | -0.204 | - | - |
| 9158 | ENSP00000312309 | TNK1 | 2.779E-05 | -0.204 | - | - |
| 9159 | ENSP00000347847 | MKL1 | 2.240E-05 | -0.204 | - | - |
| 9160 | ENSP00000322568 | SOX11 | 2.190E-05 | -0.204 | - | - |
| 9161 | ENSP00000300255 | EVA1C | 1.868E-05 | -0.204 | - | - |
| 9162 | ENSP00000297282 | SLC13A4 | 1.821E-05 | -0.204 | - | - |
| 9163 | ENSP00000304710 | TMC7 | 1.503E-05 | -0.205 | - | - |
| 9164 | ENSP00000377233 | ARAP1 | 1.770E-05 | -0.205 | - | - |
| 9165 | ENSP00000355011 | ILF2 | 1.480E-05 | -0.205 | - | - |
| 9166 | ENSP00000227451 | DTX4 | 3.634E-05 | -0.205 | - | - |
| 9167 | ENSP00000238651 | ACOT2 | 1.462E-05 | -0.205 | - | - |
| 9168 | ENSP00000283254 | SCN3A | 1.736E-05 | -0.205 | - | - |
| 9169 | ENSP00000248342 | EIF3K | 1.682E-05 | -0.205 | - | - |
| 9170 | ENSP00000261755 | FAH | 2.342E-05 | -0.205 | - | - |
| 9171 | ENSP00000300213 | CCNDBP1 | 1.922E-05 | -0.205 | - | - |
| 9172 | ENSP00000358460 | CLIC2 | 2.670E-05 | -0.205 | - | - |
| 9173 | ENSP00000246166 | FNTB | 1.512E-05 | -0.205 | - | - |
| 9174 | ENSP00000313854 | CLPTM1L | 1.725E-05 | -0.205 | - | - |
| 9175 | ENSP00000350580 | HIST1H2BB | 2.520E-05 | -0.205 | - | - |
| 9176 | ENSP00000328773 | HEXIM1 | 1.391E-05 | -0.205 | - | - |
| 9177 | ENSP00000305699 | HOOK3 | 1.875E-05 | -0.205 | - | - |
| 9178 | ENSP00000252595 | SLC27A1 | 3.348E-05 | -0.205 | - | - |
| 9179 | ENSP00000358140 | EIF3A | 2.130E-05 | -0.205 | - | - |
| 9180 | ENSP00000261884 | TRIP4 | 1.495E-05 | -0.205 | - | - |
| 9181 | ENSP00000367406 | TAF2 | 1.389E-05 | -0.205 | - | - |
| 9182 | ENSP00000233596 | REEP6 | 1.721E-05 | -0.205 | - | - |
| 9183 | ENSP00000366208 | CST11 | 2.359E-05 | -0.205 | - | - |
| 9184 | ENSP00000267113 | ESYT1 | 1.902E-05 | -0.205 | - | - |
| 9185 | ENSP00000299134 | SVOP | 1.461E-05 | -0.205 | - | - |
| 9186 | ENSP00000334145 | F3 | 1.014E-04 | -0.205 | - | - |
| 9187 | ENSP00000319231 | PHLDA2 | 4.680E-05 | -0.205 | - | - |
| 9188 | ENSP00000210187 | RAB26 | 1.754E-05 | -0.205 | - | - |
| 9189 | ENSP00000396441 | GPATCH4 | 1.608E-05 | -0.205 | - | - |
| 9190 | ENSP00000373715 | DCP2 | 1.420E-05 | -0.205 | - | - |
| 9191 | ENSP00000267103 | C12orf10 | 1.657E-05 | -0.205 | - | - |
| 9192 | ENSP00000285298 | MRPS17 | 2.329E-05 | -0.205 | - | - |
| 9193 | ENSP00000335463 | CASC5 | 1.555E-05 | -0.205 | - | - |
| 9194 | ENSP00000233535 | SLC30A3 | 1.989E-05 | -0.205 | - | - |
| 9195 | ENSP00000283558 | ATP11A | 2.055E-05 | -0.205 | - | - |
| 9196 | ENSP00000332287 | SYNGR1 | 1.854E-05 | -0.205 | - | - |
| 9197 | ENSP00000279247 | CAPN1 | 2.016E-05 | -0.205 | - | - |
| 9198 | ENSP00000243776 | CHPF | 1.551E-05 | -0.205 | - | - |
| 9199 | ENSP00000413418 | ZFHX2 | 8.370E-06 | -0.205 | - | - |
| 9200 | ENSP00000266483 | ALG10 | 1.444E-05 | -0.205 | - | - |
| 9201 | ENSP00000286398 | SMC2 | 1.787E-05 | -0.205 | - | - |
| 9202 | ENSP00000260282 | FXYD6 | 1.670E-05 | -0.205 | - | - |
| 9203 | ENSP00000472801 | AC007204.1 | 9.035E-06 | -0.205 | - | - |
| 9204 | ENSP00000253861 | EXOC4 | 1.394E-05 | -0.205 | - | - |
| 9205 | ENSP00000254730 | EEFSEC | 1.191E-05 | -0.205 | - | - |
| 9206 | ENSP00000304891 | ATP6V1E2 | 1.285E-05 | -0.205 | - | - |
| 9207 | ENSP00000327072 | TAF1A | 1.800E-05 | -0.205 | - | - |
| 9208 | ENSP00000283351 | TRAPPC8 | 1.184E-05 | -0.205 | - | - |
| 9209 | ENSP00000291577 | C21orf33 | 1.769E-05 | -0.205 | - | - |
| 9210 | ENSP00000338728 | CCDC88A | 2.783E-05 | -0.205 | - | - |
| 9211 | ENSP00000366814 | FOXD4L2 | 1.071E-05 | -0.205 | - | - |
| 9212 | ENSP00000253462 | GINS2 | 1.358E-05 | -0.205 | - | - |
| 9213 | ENSP00000357484 | GOPC | 3.371E-05 | -0.205 | - | - |
| 9214 | ENSP00000382584 | KRTAP5-5 | 2.751E-06 | -0.205 | - | - |
| 9215 | ENSP00000340463 | S100A14 | 1.341E-05 | -0.205 | - | - |
| 9216 | ENSP00000316950 | MYEF2 | 2.189E-05 | -0.205 | - | - |
| 9217 | ENSP00000341179 | AC002472.13 | 2.352E-05 | -0.205 | - | - |
| 9218 | ENSP00000341737 | NECAP1 | 1.612E-05 | -0.205 | - | - |
| 9219 | ENSP00000215812 | SEC14L3 | 1.435E-05 | -0.205 | - | - |
| 9220 | ENSP00000221957 | PLIN3 | 1.920E-05 | -0.205 | - | - |
| 9221 | ENSP00000312675 | TRIM72 | 2.553E-05 | -0.205 | - | - |
| 9222 | ENSP00000334128 | CYP27C1 | 2.278E-05 | -0.205 | - | - |
| 9223 | ENSP00000262173 | RNMT | 1.470E-05 | -0.205 | - | - |
| 9224 | ENSP00000350633 | SERTAD1 | 5.879E-05 | -0.205 | - | - |
| 9225 | ENSP00000251287 | HCN2 | 1.356E-05 | -0.205 | - | - |
| 9226 | ENSP00000296632 | STARD4 | 2.046E-05 | -0.205 | - | - |
| 9227 | ENSP00000309052 | CATSPER1 | 1.246E-05 | -0.205 | - | - |
| 9228 | ENSP00000262585 | DENND3 | 2.163E-05 | -0.205 | - | - |
| 9229 | ENSP00000328228 | CDCA2 | 1.418E-05 | -0.205 | - | - |
| 9230 | ENSP00000268835 | PRPSAP2 | 1.734E-05 | -0.205 | - | - |
| 9231 | ENSP00000216962 | PYGB | 1.474E-05 | -0.205 | - | - |
| 9232 | ENSP00000372326 | FECH | 2.829E-05 | -0.205 | - | - |
| 9233 | ENSP00000218548 | ATP12A | 2.888E-05 | -0.205 | - | - |
| 9234 | ENSP00000301956 | ACSM1 | 2.575E-05 | -0.205 | - | - |
| 9235 | ENSP00000320130 | DYNC1I1 | 2.136E-05 | -0.205 | - | - |
| 9236 | ENSP00000281038 | NARS2 | 1.291E-05 | -0.205 | - | - |
| 9237 | ENSP00000005995 | PRSS21 | 4.668E-05 | -0.205 | - | - |
| 9238 | ENSP00000210444 | NANS | 1.601E-05 | -0.205 | - | - |
| 9239 | ENSP00000307342 | HCN1 | 1.371E-05 | -0.205 | - | - |
| 9240 | ENSP00000298159 | CFL2 | 1.573E-05 | -0.205 | - | - |
| 9241 | ENSP00000428756 | STAU2 | 3.111E-05 | -0.205 | - | - |
| 9242 | ENSP00000369395 | LRRC19 | 9.552E-06 | -0.205 | - | - |
| 9243 | ENSP00000314420 | PER1 | 1.526E-05 | -0.205 | - | - |
| 9244 | ENSP00000271373 | MPC2 | 2.306E-05 | -0.205 | - | - |
| 9245 | ENSP00000387006 | CWC22 | 3.297E-05 | -0.205 | - | - |
| 9246 | ENSP00000363500 | CLIC4 | 3.055E-05 | -0.205 | - | - |
| 9247 | ENSP00000254695 | RAP1GAP2 | 1.657E-05 | -0.205 | - | - |
| 9248 | ENSP00000255390 | SCO1 | 4.291E-05 | -0.205 | - | - |
| 9249 | ENSP00000327431 | HBG1 | 1.120E-05 | -0.205 | - | - |
| 9250 | ENSP00000380602 | CNTN4 | 1.521E-05 | -0.205 | - | - |
| 9251 | ENSP00000236957 | EEF1B2 | 1.554E-05 | -0.205 | - | - |
| 9252 | ENSP00000258646 | RCBTB1 | 2.628E-05 | -0.205 | - | - |
| 9253 | ENSP00000308583 | APITD1 | 1.428E-05 | -0.205 | - | - |
| 9254 | ENSP00000342858 | FERMT2 | 2.247E-05 | -0.205 | - | - |
| 9255 | ENSP00000263897 | USE1 | 2.956E-05 | -0.205 | - | - |
| 9256 | ENSP00000317895 | RPTN | 1.507E-05 | -0.205 | - | - |
| 9257 | ENSP00000362144 | MCU | 4.202E-05 | -0.205 | - | - |
| 9258 | ENSP00000290541 | PSMB4 | 2.773E-05 | -0.205 | - | - |
| 9259 | ENSP00000413411 | ZNF724P | 9.421E-06 | -0.205 | - | - |
| 9260 | ENSP00000327704 | PSMF1 | 2.981E-05 | -0.205 | - | - |
| 9261 | ENSP00000301141 | CYP2A6 | 3.066E-05 | -0.205 | - | - |
| 9262 | ENSP00000304185 | GTSF1 | 2.323E-05 | -0.205 | - | - |
| 9263 | ENSP00000223129 | RPA3 | 1.377E-05 | -0.205 | - | - |
| 9264 | ENSP00000265070 | GOLPH3 | 3.151E-05 | -0.205 | - | - |
| 9265 | ENSP00000349298 | MYLIP | 2.979E-05 | -0.205 | - | - |
| 9266 | ENSP00000344989 | DALRD3 | 1.847E-05 | -0.205 | - | - |
| 9267 | ENSP00000248378 | EMC6 | 1.890E-05 | -0.205 | - | - |
| 9268 | ENSP00000238721 | TP53I3 | 1.624E-05 | -0.206 | - | - |
| 9269 | ENSP00000395461 | BTBD19 | 1.213E-05 | -0.206 | - | - |
| 9270 | ENSP00000288985 | ERCC6L2 | 1.396E-05 | -0.206 | - | - |
| 9271 | ENSP00000237380 | MED28 | 1.221E-04 | -0.206 | - | - |
| 9272 | ENSP00000337926 | SUGP2 | 2.358E-05 | -0.206 | - | - |
| 9273 | ENSP00000333433 | SKA2 | 1.411E-05 | -0.206 | - | - |
| 9274 | ENSP00000329452 | SLC25A21 | 1.578E-05 | -0.206 | - | - |
| 9275 | ENSP00000366630 | FOXD4L4 | 1.071E-05 | -0.206 | - | - |
| 9276 | ENSP00000261247 | JKAMP | 2.839E-05 | -0.206 | - | - |
| 9277 | ENSP00000247170 | DAAM1 | 1.640E-05 | -0.206 | - | - |
| 9278 | ENSP00000337194 | PRPF4B | 1.303E-05 | -0.206 | - | - |
| 9279 | ENSP00000319778 | AURKAIP1 | 2.793E-05 | -0.206 | - | - |
| 9280 | ENSP00000297293 | LMTK2 | 2.311E-05 | -0.206 | - | - |
| 9281 | ENSP00000320493 | TRIP10 | 2.445E-05 | -0.206 | - | - |
| 9282 | ENSP00000397163 | ZNF286A | 8.667E-06 | -0.206 | - | - |
| 9283 | ENSP00000378350 | CDC23 | 2.137E-05 | -0.206 | - | - |
| 9284 | ENSP00000262890 | PAFAH1B3 | 2.145E-05 | -0.206 | - | - |
| 9285 | ENSP00000399518 | SATB1 | 2.939E-05 | -0.206 | - | - |
| 9286 | ENSP00000304689 | THAP11 | 2.866E-05 | -0.206 | - | - |
| 9287 | ENSP00000355884 | MARK1 | 2.253E-05 | -0.206 | - | - |
| 9288 | ENSP00000268719 | GID4 | 1.892E-05 | -0.206 | - | - |
| 9289 | ENSP00000282412 | PPM1B | 1.972E-05 | -0.206 | - | - |
| 9290 | ENSP00000263956 | GTF3C3 | 1.685E-05 | -0.206 | - | - |
| 9291 | ENSP00000200181 | ITGB4 | 7.810E-05 | -0.206 | - | - |
| 9292 | ENSP00000305810 | STX18 | 1.387E-05 | -0.206 | - | - |
| 9293 | ENSP00000309690 | DMXL1 | 3.589E-05 | -0.206 | - | - |
| 9294 | ENSP00000342262 | NUP43 | 2.111E-05 | -0.206 | - | - |
| 9295 | ENSP00000360597 | LRRC26 | 9.828E-06 | -0.206 | - | - |
| 9296 | ENSP00000285083 | OXNAD1 | 1.300E-05 | -0.206 | - | - |
| 9297 | ENSP00000262498 | C16orf80 | 5.682E-05 | -0.206 | - | - |
| 9298 | ENSP00000370021 | CNTLN | 4.954E-05 | -0.206 | - | - |
| 9299 | ENSP00000286800 | BACH1 | 8.230E-05 | -0.206 | - | - |
| 9300 | ENSP00000265080 | RASGRF2 | 2.481E-05 | -0.206 | - | - |
| 9301 | ENSP00000330694 | NDNL2 | 2.869E-05 | -0.206 | - | - |
| 9302 | ENSP00000233379 | FAHD2A | 2.090E-05 | -0.206 | - | - |
| 9303 | ENSP00000296424 | BDH2 | 2.342E-05 | -0.206 | - | - |
| 9304 | ENSP00000348216 | TRIM4 | 7.422E-06 | -0.206 | - | - |
| 9305 | ENSP00000244043 | PTGIS | 3.215E-05 | -0.206 | - | - |
| 9306 | ENSP00000218340 | RP2 | 2.462E-05 | -0.206 | - | - |
| 9307 | ENSP00000328070 | GLTPD2 | 8.097E-06 | -0.206 | - | - |
| 9308 | ENSP00000267229 | RBM26 | 1.616E-05 | -0.206 | - | - |
| 9309 | ENSP00000358374 | SCAF11 | 2.751E-05 | -0.206 | - | - |
| 9310 | ENSP00000325017 | NCAPD2 | 1.661E-05 | -0.206 | - | - |
| 9311 | ENSP00000342962 | SERINC1 | 5.057E-05 | -0.206 | - | - |
| 9312 | ENSP00000234142 | GREB1 | 2.507E-05 | -0.206 | - | - |
| 9313 | ENSP00000333813 | ALG12 | 1.766E-05 | -0.206 | - | - |
| 9314 | ENSP00000292539 | PPP1R16A | 1.876E-05 | -0.206 | - | - |
| 9315 | ENSP00000261637 | UTP20 | 1.066E-05 | -0.206 | - | - |
| 9316 | ENSP00000264057 | DGKD | 2.508E-05 | -0.206 | - | - |
| 9317 | ENSP00000370330 | ERBB2IP | 2.493E-05 | -0.206 | - | - |
| 9318 | ENSP00000261674 | SFSWAP | 2.630E-05 | -0.206 | - | - |
| 9319 | ENSP00000296564 | KIAA0947 | 4.371E-05 | -0.206 | - | - |
| 9320 | ENSP00000263904 | STAM2 | 2.958E-05 | -0.206 | - | - |
| 9321 | ENSP00000322845 | ZMYM3 | 1.673E-05 | -0.206 | - | - |
| 9322 | ENSP00000285968 | NUP205 | 1.678E-05 | -0.206 | - | - |
| 9323 | ENSP00000299626 | ALG8 | 1.669E-05 | -0.206 | - | - |
| 9324 | ENSP00000355046 | MT-ND2 | 2.215E-05 | -0.206 | - | - |
| 9325 | ENSP00000347649 | LEPREL4 | 2.669E-05 | -0.206 | - | - |
| 9326 | ENSP00000202831 | SLC24A6 | 3.183E-05 | -0.206 | - | - |
| 9327 | ENSP00000376215 | UBE2E3 | 4.392E-05 | -0.206 | - | - |
| 9328 | ENSP00000326227 | GANC | 1.244E-05 | -0.206 | - | - |
| 9329 | ENSP00000250498 | DAD1 | 2.330E-05 | -0.206 | - | - |
| 9330 | ENSP00000322915 | ZMYM4 | 2.369E-05 | -0.206 | - | - |
| 9331 | ENSP00000331065 | PPP1R27 | 1.697E-05 | -0.206 | - | - |
| 9332 | ENSP00000387124 | C2orf78 | 6.396E-06 | -0.206 | - | - |
| 9333 | ENSP00000280605 | TKTL2 | 1.863E-05 | -0.206 | - | - |
| 9334 | ENSP00000339389 | OTUD6A | 2.067E-05 | -0.206 | - | - |
| 9335 | ENSP00000337451 | EPHA3 | 3.186E-05 | -0.206 | - | - |
| 9336 | ENSP00000351042 | ZNF100 | 1.641E-05 | -0.206 | - | - |
| 9337 | ENSP00000285805 | TRIM74 | 2.942E-05 | -0.206 | - | - |
| 9338 | ENSP00000298937 | ELP4 | 1.608E-05 | -0.206 | - | - |
| 9339 | ENSP00000470969 | AC004076.9 | 8.917E-06 | -0.206 | - | - |
| 9340 | ENSP00000337439 | DACT1 | 3.101E-05 | -0.206 | - | - |
| 9341 | ENSP00000266079 | PRPF6 | 1.656E-05 | -0.206 | - | - |
| 9342 | ENSP00000252015 | TRPC4AP | 1.588E-05 | -0.206 | - | - |
| 9343 | ENSP00000309262 | USP48 | 2.857E-05 | -0.206 | - | - |
| 9344 | ENSP00000292211 | UBE2Q1 | 3.255E-05 | -0.206 | - | - |
| 9345 | ENSP00000242108 | EEPD1 | 1.590E-05 | -0.206 | - | - |
| 9346 | ENSP00000295033 | TRIM17 | 2.165E-05 | -0.206 | - | - |
| 9347 | ENSP00000317905 | C17orf96 | 3.297E-05 | -0.206 | - | - |
| 9348 | ENSP00000311888 | MGAT1 | 1.070E-05 | -0.206 | - | - |
| 9349 | ENSP00000251496 | NCAPG | 2.009E-05 | -0.206 | - | - |
| 9350 | ENSP00000309142 | PDE12 | 1.783E-05 | -0.206 | - | - |
| 9351 | ENSP00000349467 | CALM1 | 3.544E-05 | -0.206 | - | - |
| 9352 | ENSP00000338165 | TMEM39B | 3.223E-05 | -0.206 | - | - |
| 9353 | ENSP00000380941 | C21orf67 | 1.182E-05 | -0.206 | - | - |
| 9354 | ENSP00000367064 | CUBN | 2.434E-05 | -0.206 | - | - |
| 9355 | ENSP00000301634 | TK1 | 2.026E-05 | -0.206 | - | - |
| 9356 | ENSP00000314004 | ANAPC2 | 1.569E-05 | -0.206 | - | - |
| 9357 | ENSP00000373726 | FAM159B | 6.030E-06 | -0.206 | - | - |
| 9358 | ENSP00000341214 | HIST1H1T | 1.472E-05 | -0.206 | - | - |
| 9359 | ENSP00000408526 | IMPA1 | 1.778E-05 | -0.206 | - | - |
| 9360 | ENSP00000339479 | NT5C2 | 2.673E-05 | -0.206 | - | - |
| 9361 | ENSP00000348753 | SPRED2 | 9.227E-05 | -0.206 | - | - |
| 9362 | ENSP00000362107 | FHL3 | 3.455E-05 | -0.206 | - | - |
| 9363 | ENSP00000225504 | SUPT4H1 | 1.546E-05 | -0.206 | - | - |
| 9364 | ENSP00000199389 | EIF2AK1 | 1.716E-05 | -0.206 | - | - |
| 9365 | ENSP00000305654 | TVP23B | 1.980E-05 | -0.206 | - | - |
| 9366 | ENSP00000013070 | UBR7 | 3.788E-05 | -0.206 | - | - |
| 9367 | ENSP00000268797 | GFOD2 | 1.244E-05 | -0.206 | - | - |
| 9368 | ENSP00000216479 | AHSA1 | 1.569E-05 | -0.206 | - | - |
| 9369 | ENSP00000368066 | HAO1 | 2.203E-05 | -0.206 | - | - |
| 9370 | ENSP00000265276 | GPAM | 1.615E-05 | -0.206 | - | - |
| 9371 | ENSP00000303088 | POLR3D | 1.275E-05 | -0.206 | - | - |
| 9372 | ENSP00000348822 | SEMA4D | 6.186E-05 | -0.206 | - | - |
| 9373 | ENSP00000280704 | LDHC | 1.855E-05 | -0.206 | - | - |
| 9374 | ENSP00000281187 | VPS26B | 1.214E-05 | -0.206 | - | - |
| 9375 | ENSP00000247843 | YEATS4 | 1.641E-05 | -0.206 | - | - |
| 9376 | ENSP00000307599 | SCN11A | 2.001E-05 | -0.206 | - | - |
| 9377 | ENSP00000312837 | RAB11FIP4 | 5.349E-05 | -0.206 | - | - |
| 9378 | ENSP00000315035 | PPP1R42 | 1.344E-05 | -0.206 | - | - |
| 9379 | ENSP00000234296 | ORC2 | 1.640E-05 | -0.206 | - | - |
| 9380 | ENSP00000245046 | EMC3 | 1.461E-05 | -0.206 | - | - |
| 9381 | ENSP00000364645 | ZNF484 | 1.231E-05 | -0.206 | - | - |
| 9382 | ENSP00000332604 | EIF3C | 1.941E-05 | -0.206 | - | - |
| 9383 | ENSP00000337500 | PCGF5 | 1.228E-05 | -0.206 | - | - |
| 9384 | ENSP00000312625 | HP1BP3 | 1.254E-05 | -0.206 | - | - |
| 9385 | ENSP00000216190 | EIF3D | 1.306E-05 | -0.206 | - | - |
| 9386 | ENSP00000300403 | TPX2 | 2.591E-05 | -0.206 | - | - |
| 9387 | ENSP00000253023 | UBE2M | 2.407E-05 | -0.206 | - | - |
| 9388 | ENSP00000081029 | MRPS35 | 1.425E-05 | -0.207 | - | - |
| 9389 | ENSP00000351248 | C16orf47 | 4.412E-06 | -0.207 | - | - |
| 9390 | ENSP00000006101 | COPZ2 | 1.321E-05 | -0.207 | - | - |
| 9391 | ENSP00000330930 | SGK223 | 3.060E-05 | -0.207 | - | - |
| 9392 | ENSP00000254035 | CKMT2 | 2.001E-05 | -0.207 | - | - |
| 9393 | ENSP00000300283 | CKMT1B | 1.179E-05 | -0.207 | - | - |
| 9394 | ENSP00000313432 | GRHPR | 1.065E-05 | -0.207 | - | - |
| 9395 | ENSP00000380998 | CHMP1A | 1.126E-04 | -0.207 | - | - |
| 9396 | ENSP00000320413 | RAB23 | 1.517E-05 | -0.207 | - | - |
| 9397 | ENSP00000339769 | SMEK2 | 2.019E-05 | -0.207 | - | - |
| 9398 | ENSP00000461425 | ZNF66 | 9.414E-06 | -0.207 | - | - |
| 9399 | ENSP00000255389 | PEMT | 1.460E-05 | -0.207 | - | - |
| 9400 | ENSP00000267750 | EMC4 | 1.818E-05 | -0.207 | - | - |
| 9401 | ENSP00000219097 | ORC6 | 1.522E-05 | -0.207 | - | - |
| 9402 | ENSP00000287022 | UQCRB | 1.733E-05 | -0.207 | - | - |
| 9403 | ENSP00000194530 | STRADB | 8.064E-05 | -0.207 | - | - |
| 9404 | ENSP00000263969 | MFN1 | 1.798E-05 | -0.207 | - | - |
| 9405 | ENSP00000296277 | RPL39L | 1.572E-05 | -0.207 | - | - |
| 9406 | ENSP00000261758 | MESDC2 | 2.448E-05 | -0.207 | - | - |
| 9407 | ENSP00000265333 | VDAC1 | 1.843E-05 | -0.207 | - | - |
| 9408 | ENSP00000315659 | DNMBP | 3.437E-05 | -0.207 | - | - |
| 9409 | ENSP00000326491 | ABHD1 | 1.385E-05 | -0.207 | - | - |
| 9410 | ENSP00000398211 | TTC21A | 2.508E-05 | -0.207 | - | - |
| 9411 | ENSP00000332034 | PROSER1 | 2.231E-05 | -0.207 | - | - |
| 9412 | ENSP00000312370 | AIFM2 | 1.313E-05 | -0.207 | - | - |
| 9413 | ENSP00000262637 | OVOL3 | 4.639E-05 | -0.207 | - | - |
| 9414 | ENSP00000265447 | ANXA11 | 7.646E-05 | -0.207 | - | - |
| 9415 | ENSP00000203407 | UQCRC1 | 1.417E-05 | -0.207 | - | - |
| 9416 | ENSP00000361845 | ZMPSTE24 | 1.188E-05 | -0.207 | - | - |
| 9417 | ENSP00000248248 | MON1B | 1.424E-05 | -0.207 | - | - |
| 9418 | ENSP00000219054 | ACSM2A | 1.678E-05 | -0.207 | - | - |
| 9419 | ENSP00000315397 | MRPS31 | 3.936E-05 | -0.207 | - | - |
| 9420 | ENSP00000265038 | ERCC8 | 1.492E-05 | -0.207 | - | - |
| 9421 | ENSP00000337561 | SLC43A3 | 1.100E-05 | -0.207 | - | - |
| 9422 | ENSP00000471077 | ZNF417 | 9.368E-06 | -0.207 | - | - |
| 9423 | ENSP00000217133 | TUBB1 | 1.244E-05 | -0.207 | - | - |
| 9424 | ENSP00000280614 | CCRN4L | 1.736E-05 | -0.207 | - | - |
| 9425 | ENSP00000315654 | KCNG2 | 1.995E-05 | -0.207 | - | - |
| 9426 | ENSP00000231498 | NUP155 | 1.605E-05 | -0.207 | - | - |
| 9427 | ENSP00000310120 | ALG10B | 1.308E-05 | -0.207 | - | - |
| 9428 | ENSP00000305230 | SRP9 | 3.935E-05 | -0.207 | - | - |
| 9429 | ENSP00000263985 | PET112 | 1.229E-05 | -0.207 | - | - |
| 9430 | ENSP00000310873 | COQ2 | 1.220E-05 | -0.207 | - | - |
| 9431 | ENSP00000396320 | SCN4A | 2.821E-05 | -0.207 | - | - |
| 9432 | ENSP00000375416 | DKFZP666M039 | 1.132E-05 | -0.207 | - | - |
| 9433 | ENSP00000314343 | MED29 | 1.412E-05 | -0.207 | - | - |
| 9434 | ENSP00000318629 | VPS37A | 1.492E-05 | -0.207 | - | - |
| 9435 | ENSP00000260056 | RAB30 | 1.579E-05 | -0.207 | - | - |
| 9436 | ENSP00000302719 | KCNAB3 | 1.312E-05 | -0.207 | - | - |
| 9437 | ENSP00000354451 | IQGAP3 | 4.660E-05 | -0.207 | - | - |
| 9438 | ENSP00000294119 | UBXN1 | 3.547E-05 | -0.207 | - | - |
| 9439 | ENSP00000429266 | ZNF829 | 1.024E-05 | -0.207 | - | - |
| 9440 | ENSP00000297625 | KIAA1161 | 1.177E-05 | -0.207 | - | - |
| 9441 | ENSP00000315064 | MAGEF1 | 2.514E-05 | -0.207 | - | - |
| 9442 | ENSP00000455099 | TMEM88B | 5.044E-07 | -0.207 | - | - |
| 9443 | ENSP00000339495 | NELFB | 1.306E-05 | -0.207 | - | - |
| 9444 | ENSP00000301280 | CHAF1A | 1.657E-05 | -0.207 | - | - |
| 9445 | ENSP00000362727 | DENND1A | 3.173E-05 | -0.207 | - | - |
| 9446 | ENSP00000377262 | SRPK2 | 1.432E-03 | -0.207 | - | - |
| 9447 | ENSP00000259632 | DCTN3 | 4.769E-05 | -0.207 | - | - |
| 9448 | ENSP00000274496 | YIPF5 | 1.358E-05 | -0.207 | - | - |
| 9449 | ENSP00000266505 | PLCZ1 | 1.358E-05 | -0.207 | - | - |
| 9450 | ENSP00000284320 | TOMM70A | 1.136E-05 | -0.207 | - | - |
| 9451 | ENSP00000377700 | TBC1D23 | 1.754E-05 | -0.207 | - | - |
| 9452 | ENSP00000215980 | CENPM | 1.289E-05 | -0.207 | - | - |
| 9453 | ENSP00000379394 | NAV2 | 5.087E-06 | -0.207 | - | - |
| 9454 | ENSP00000234626 | CDC7 | 2.603E-05 | -0.207 | - | - |
| 9455 | ENSP00000341351 | DCAF5 | 2.275E-05 | -0.207 | - | - |
| 9456 | ENSP00000265857 | GET4 | 1.752E-05 | -0.207 | - | - |
| 9457 | ENSP00000318821 | GCC1 | 1.183E-05 | -0.207 | - | - |
| 9458 | ENSP00000299853 | POLR3E | 1.521E-05 | -0.207 | - | - |
| 9459 | ENSP00000339503 | NUDT1 | 1.398E-05 | -0.207 | - | - |
| 9460 | ENSP00000361180 | GTF3C5 | 2.815E-05 | -0.207 | - | - |
| 9461 | ENSP00000260116 | TTPA | 1.367E-05 | -0.207 | - | - |
| 9462 | ENSP00000341805 | PHF10 | 1.419E-05 | -0.207 | - | - |
| 9463 | ENSP00000297151 | SLU7 | 1.737E-05 | -0.207 | - | - |
| 9464 | ENSP00000312311 | MRPL46 | 1.095E-05 | -0.207 | - | - |
| 9465 | ENSP00000218348 | USP11 | 1.937E-05 | -0.207 | - | - |
| 9466 | ENSP00000273062 | CTDSP1 | 1.769E-05 | -0.207 | - | - |
| 9467 | ENSP00000263346 | TCF25 | 1.575E-05 | -0.207 | - | - |
| 9468 | ENSP00000304292 | ANKRD27 | 2.003E-05 | -0.207 | - | - |
| 9469 | ENSP00000323927 | PLXDC1 | 2.265E-05 | -0.207 | - | - |
| 9470 | ENSP00000343610 | SYNRG | 1.295E-05 | -0.207 | - | - |
| 9471 | ENSP00000260270 | FDX1 | 1.114E-05 | -0.207 | - | - |
| 9472 | ENSP00000471905 | ZNF17 | 1.334E-05 | -0.207 | - | - |
| 9473 | ENSP00000323074 | RAI1 | 2.708E-05 | -0.207 | - | - |
| 9474 | ENSP00000216367 | POLE2 | 1.547E-05 | -0.207 | - | - |
| 9475 | ENSP00000345824 | BZRAP1 | 2.509E-05 | -0.207 | - | - |
| 9476 | ENSP00000262715 | TEP1 | 1.288E-05 | -0.207 | - | - |
| 9477 | ENSP00000313500 | C17orf53 | 1.409E-05 | -0.207 | - | - |
| 9478 | ENSP00000297564 | COX6C | 1.447E-05 | -0.207 | - | - |
| 9479 | ENSP00000328598 | HOXD1 | 2.032E-05 | -0.207 | - | - |
| 9480 | ENSP00000376148 | ARMC6 | 2.109E-05 | -0.207 | - | - |
| 9481 | ENSP00000278422 | TMX2 | 3.686E-05 | -0.207 | - | - |
| 9482 | ENSP00000263270 | AP2S1 | 2.639E-05 | -0.207 | - | - |
| 9483 | ENSP00000253571 | RLIM | 2.629E-05 | -0.208 | - | - |
| 9484 | ENSP00000305204 | CKAP2L | 1.175E-05 | -0.208 | - | - |
| 9485 | ENSP00000225576 | TVP23C | 1.668E-05 | -0.208 | - | - |
| 9486 | ENSP00000296557 | ARFIP1 | 2.061E-05 | -0.208 | - | - |
| 9487 | ENSP00000233714 | LANCL1 | 1.741E-05 | -0.208 | - | - |
| 9488 | ENSP00000317955 | EEA1 | 2.308E-05 | -0.208 | - | - |
| 9489 | ENSP00000254630 | PTCD3 | 1.502E-05 | -0.208 | - | - |
| 9490 | ENSP00000338927 | ZNF385A | 2.014E-05 | -0.208 | - | - |
| 9491 | ENSP00000416293 | SLC2A1 | 5.745E-05 | -0.208 | - | - |
| 9492 | ENSP00000276033 | SLC16A2 | 1.532E-05 | -0.208 | - | - |
| 9493 | ENSP00000298717 | METTL3 | 1.540E-05 | -0.208 | - | - |
| 9494 | ENSP00000360806 | KCNB1 | 2.769E-05 | -0.208 | - | - |
| 9495 | ENSP00000311083 | CKS1B | 1.516E-05 | -0.208 | - | - |
| 9496 | ENSP00000413625 | FNBP1 | 3.353E-05 | -0.208 | - | - |
| 9497 | ENSP00000417653 | DBNL | 2.594E-05 | -0.208 | - | - |
| 9498 | ENSP00000274963 | FGD2 | 1.264E-05 | -0.208 | - | - |
| 9499 | ENSP00000307850 | DRAP1 | 1.529E-05 | -0.208 | - | - |
| 9500 | ENSP00000301738 | KCTD5 | 1.347E-05 | -0.208 | - | - |
| 9501 | ENSP00000252936 | TUBGCP2 | 1.170E-05 | -0.208 | - | - |
| 9502 | ENSP00000228843 | RAD51AP1 | 1.491E-05 | -0.208 | - | - |
| 9503 | ENSP00000290231 | NCOA5 | 1.383E-05 | -0.208 | - | - |
| 9504 | ENSP00000256578 | AMPD2 | 1.467E-05 | -0.208 | - | - |
| 9505 | ENSP00000215730 | SNAP29 | 1.469E-05 | -0.208 | - | - |
| 9506 | ENSP00000392095 | ZNF146 | 9.594E-06 | -0.208 | - | - |
| 9507 | ENSP00000233944 | PRKAG3 | 8.614E-06 | -0.208 | - | - |
| 9508 | ENSP00000354125 | EIF3B | 1.037E-05 | -0.208 | - | - |
| 9509 | ENSP00000307197 | DNAJB7 | 1.601E-05 | -0.208 | - | - |
| 9510 | ENSP00000411198 | KRTAP4-16P | 1.846E-06 | -0.208 | - | - |
| 9511 | ENSP00000300056 | PEX11A | 1.724E-05 | -0.208 | - | - |
| 9512 | ENSP00000335655 | FMNL3 | 2.269E-05 | -0.208 | - | - |
| 9513 | ENSP00000269159 | IMPA2 | 2.104E-05 | -0.208 | - | - |
| 9514 | ENSP00000298772 | TRIM13 | 2.324E-05 | -0.208 | - | - |
| 9515 | ENSP00000262127 | CEP76 | 1.708E-05 | -0.208 | - | - |
| 9516 | ENSP00000325564 | SYCN | 2.393E-05 | -0.208 | - | - |
| 9517 | ENSP00000218089 | STAG2 | 1.257E-05 | -0.208 | - | - |
| 9518 | ENSP00000346342 | RFPL1 | 9.592E-06 | -0.208 | - | - |
| 9519 | ENSP00000307939 | GCC2 | 1.548E-05 | -0.208 | - | - |
| 9520 | ENSP00000305595 | B3GNT2 | 1.062E-05 | -0.208 | - | - |
| 9521 | ENSP00000275517 | CDCA5 | 1.199E-05 | -0.208 | - | - |
| 9522 | ENSP00000264773 | KCNN2 | 1.418E-05 | -0.208 | - | - |
| 9523 | ENSP00000293970 | TBC1D24 | 1.231E-05 | -0.208 | - | - |
| 9524 | ENSP00000308344 | DCAF7 | 1.879E-05 | -0.208 | - | - |
| 9525 | ENSP00000292147 | ETHE1 | 1.169E-05 | -0.208 | - | - |
| 9526 | ENSP00000290551 | BTG2 | 9.876E-05 | -0.208 | - | - |
| 9527 | ENSP00000351706 | GK2 | 2.025E-05 | -0.208 | - | - |
| 9528 | ENSP00000312814 | KCTD2 | 1.151E-05 | -0.208 | - | - |
| 9529 | ENSP00000425048 | DTWD2 | 2.580E-05 | -0.208 | - | - |
| 9530 | ENSP00000325074 | PPP2R2A | 4.103E-05 | -0.208 | - | - |
| 9531 | ENSP00000284995 | TSEN2 | 2.003E-05 | -0.208 | - | - |
| 9532 | ENSP00000339122 | VIPAS39 | 1.469E-05 | -0.208 | - | - |
| 9533 | ENSP00000294702 | GFI1 | 3.848E-05 | -0.208 | - | - |
| 9534 | ENSP00000306356 | ENC1 | 1.647E-05 | -0.208 | - | - |
| 9535 | ENSP00000303434 | USP38 | 1.451E-05 | -0.208 | - | - |
| 9536 | ENSP00000224073 | EDF1 | 1.261E-05 | -0.208 | - | - |
| 9537 | ENSP00000250056 | FAM64A | 1.260E-05 | -0.208 | - | - |
| 9538 | ENSP00000262990 | ZNF330 | 1.514E-05 | -0.208 | - | - |
| 9539 | ENSP00000296543 | NAA15 | 1.632E-05 | -0.208 | - | - |
| 9540 | ENSP00000248071 | KLF2 | 5.403E-05 | -0.208 | - | - |
| 9541 | ENSP00000362717 | LHX2 | 4.570E-05 | -0.208 | - | - |
| 9542 | ENSP00000351727 | CCER1 | 6.097E-06 | -0.208 | - | - |
| 9543 | ENSP00000262134 | LPCAT2 | 2.126E-05 | -0.208 | - | - |
| 9544 | ENSP00000327104 | DDHD1 | 1.690E-05 | -0.208 | - | - |
| 9545 | ENSP00000216366 | AP4S1 | 1.189E-05 | -0.208 | - | - |
| 9546 | ENSP00000251527 | ESYT2 | 2.035E-05 | -0.208 | - | - |
| 9547 | ENSP00000246024 | TMX4 | 1.783E-05 | -0.208 | - | - |
| 9548 | ENSP00000192788 | UHRF1BP1 | 1.744E-04 | -0.208 | - | - |
| 9549 | ENSP00000258399 | USP37 | 2.358E-05 | -0.208 | - | - |
| 9550 | ENSP00000361811 | TP53TG5 | 4.916E-06 | -0.208 | - | - |
| 9551 | ENSP00000381605 | PCDHGA8 | 1.785E-05 | -0.208 | - | - |
| 9552 | ENSP00000368872 | INSC | 2.494E-05 | -0.208 | - | - |
| 9553 | ENSP00000309031 | VCPIP1 | 1.968E-05 | -0.208 | - | - |
| 9554 | ENSP00000341538 | SEC61G | 1.288E-05 | -0.208 | - | - |
| 9555 | ENSP00000282516 | NIPBL | 1.957E-05 | -0.208 | - | - |
| 9556 | ENSP00000362036 | MRPS16 | 1.427E-05 | -0.208 | - | - |
| 9557 | ENSP00000246868 | SBDS | 2.387E-05 | -0.208 | - | - |
| 9558 | ENSP00000237283 | ADAT2 | 1.840E-05 | -0.208 | - | - |
| 9559 | ENSP00000218364 | HTATSF1 | 1.585E-05 | -0.208 | - | - |
| 9560 | ENSP00000256383 | EIF2S1 | 4.511E-05 | -0.208 | - | - |
| 9561 | ENSP00000260665 | LRPPRC | 2.121E-05 | -0.208 | - | - |
| 9562 | ENSP00000238788 | TMEM214 | 1.448E-05 | -0.208 | - | - |
| 9563 | ENSP00000284073 | MSI2 | 2.543E-05 | -0.208 | - | - |
| 9564 | ENSP00000335565 | KRTAP10-8 | 2.617E-05 | -0.208 | - | - |
| 9565 | ENSP00000468311 | - | 2.623E-06 | -0.208 | - | - |
| 9566 | ENSP00000313752 | SSNA1 | 1.506E-05 | -0.208 | - | - |
| 9567 | ENSP00000306477 | BTD | 1.083E-05 | -0.208 | - | - |
| 9568 | ENSP00000321971 | RHOT2 | 1.699E-05 | -0.208 | - | - |
| 9569 | ENSP00000337358 | CECR5 | 1.633E-05 | -0.208 | - | - |
| 9570 | ENSP00000336940 | KIAA1958 | 1.684E-05 | -0.208 | - | - |
| 9571 | ENSP00000343435 | YIF1B | 1.073E-05 | -0.208 | - | - |
| 9572 | ENSP00000252542 | SAFB2 | 1.529E-05 | -0.208 | - | - |
| 9573 | ENSP00000310006 | NR1D2 | 3.546E-05 | -0.208 | - | - |
| 9574 | ENSP00000354681 | OPA1 | 1.599E-05 | -0.208 | - | - |
| 9575 | ENSP00000313674 | AAR2 | 1.502E-05 | -0.208 | - | - |
| 9576 | ENSP00000380785 | HOOK2 | 2.945E-05 | -0.208 | - | - |
| 9577 | ENSP00000330199 | SLC6A17 | 1.630E-05 | -0.208 | - | - |
| 9578 | ENSP00000345064 | PRICKLE1 | 2.972E-05 | -0.208 | - | - |
| 9579 | ENSP00000308383 | LSP1 | 3.610E-05 | -0.208 | - | - |
| 9580 | ENSP00000301463 | SPRYD3 | 3.622E-05 | -0.208 | - | - |
| 9581 | ENSP00000261514 | CLCN3 | 1.538E-05 | -0.208 | - | - |
| 9582 | ENSP00000327821 | XPOT | 1.413E-05 | -0.208 | - | - |
| 9583 | ENSP00000290429 | MCAT | 1.650E-05 | -0.208 | - | - |
| 9584 | ENSP00000400142 | EWSR1 | 3.649E-05 | -0.208 | - | - |
| 9585 | ENSP00000370669 | SLC8A3 | 1.753E-05 | -0.208 | - | - |
| 9586 | ENSP00000352358 | TRPV6 | 2.199E-05 | -0.208 | - | - |
| 9587 | ENSP00000269601 | TXNL4A | 1.237E-05 | -0.208 | - | - |
| 9588 | ENSP00000267978 | MAN2C1 | 1.752E-05 | -0.208 | - | - |
| 9589 | ENSP00000237530 | RPN2 | 1.310E-05 | -0.208 | - | - |
| 9590 | ENSP00000313169 | NPHP1 | 1.816E-05 | -0.208 | - | - |
| 9591 | ENSP00000311219 | TRIM59 | 2.631E-05 | -0.208 | - | - |
| 9592 | ENSP00000358038 | BEND3 | 1.944E-05 | -0.208 | - | - |
| 9593 | ENSP00000262607 | CECR1 | 1.282E-05 | -0.208 | - | - |
| 9594 | ENSP00000363275 | LRRC18 | 1.234E-05 | -0.208 | - | - |
| 9595 | ENSP00000307143 | DONSON | 1.456E-05 | -0.208 | - | - |
| 9596 | ENSP00000339960 | KCNJ5 | 2.636E-05 | -0.208 | - | - |
| 9597 | ENSP00000295802 | RETSAT | 1.532E-05 | -0.208 | - | - |
| 9598 | ENSP00000261208 | HAL | 3.167E-05 | -0.208 | - | - |
| 9599 | ENSP00000338766 | NPHP3 | 1.170E-05 | -0.208 | - | - |
| 9600 | ENSP00000332284 | TRIM69 | 1.938E-05 | -0.208 | - | - |
| 9601 | ENSP00000316377 | DLGAP1 | 1.331E-05 | -0.208 | - | - |
| 9602 | ENSP00000337393 | UXT | 2.304E-05 | -0.208 | - | - |
| 9603 | ENSP00000305995 | PGK2 | 4.554E-05 | -0.208 | - | - |
| 9604 | ENSP00000324573 | FLII | 2.418E-05 | -0.208 | - | - |
| 9605 | ENSP00000238616 | NEK9 | 2.196E-05 | -0.208 | - | - |
| 9606 | ENSP00000374152 | SMYD5 | 8.262E-06 | -0.209 | - | - |
| 9607 | ENSP00000234488 | CLCN6 | 1.137E-05 | -0.209 | - | - |
| 9608 | ENSP00000258349 | RC3H1 | 1.407E-04 | -0.209 | - | - |
| 9609 | ENSP00000243662 | RAB38 | 1.215E-05 | -0.209 | - | - |
| 9610 | ENSP00000317141 | KCTD12 | 1.785E-05 | -0.209 | - | - |
| 9611 | ENSP00000262315 | CHTF18 | 1.284E-05 | -0.209 | - | - |
| 9612 | ENSP00000340796 | ZNF326 | 1.651E-05 | -0.209 | - | - |
| 9613 | ENSP00000285679 | USP25 | 2.242E-05 | -0.209 | - | - |
| 9614 | ENSP00000335082 | FAM91A1 | 2.971E-05 | -0.209 | - | - |
| 9615 | ENSP00000442411 | C21orf119 | 1.372E-06 | -0.209 | - | - |
| 9616 | ENSP00000351108 | HNRNPAB | 1.254E-05 | -0.209 | - | - |
| 9617 | ENSP00000379369 | FOXB1 | 1.396E-05 | -0.209 | - | - |
| 9618 | ENSP00000222644 | MPP6 | 2.136E-05 | -0.209 | - | - |
| 9619 | ENSP00000323377 | EXOC3 | 1.219E-05 | -0.209 | - | - |
| 9620 | ENSP00000405268 | MBD1 | 1.096E-05 | -0.209 | - | - |
| 9621 | ENSP00000310966 | CD3EAP | 2.734E-05 | -0.209 | - | - |
| 9622 | ENSP00000288699 | DPYSL5 | 1.086E-05 | -0.209 | - | - |
| 9623 | ENSP00000314048 | ANO2 | 1.867E-05 | -0.209 | - | - |
| 9624 | ENSP00000351190 | ITIH2 | 2.536E-05 | -0.209 | - | - |
| 9625 | ENSP00000313377 | PANK2 | 1.533E-05 | -0.209 | - | - |
| 9626 | ENSP00000264711 | DNAJC27 | 9.958E-06 | -0.209 | - | - |
| 9627 | ENSP00000260061 | LRRC32 | 3.088E-05 | -0.209 | - | - |
| 9628 | ENSP00000230048 | ACOT13 | 1.653E-05 | -0.209 | - | - |
| 9629 | ENSP00000419361 | ADCY5 | 4.729E-05 | -0.209 | - | - |
| 9630 | ENSP00000216330 | FKBP3 | 1.264E-05 | -0.209 | - | - |
| 9631 | ENSP00000380178 | UBE2G1 | 2.291E-05 | -0.209 | - | - |
| 9632 | ENSP00000261867 | SLC30A4 | 1.227E-05 | -0.209 | - | - |
| 9633 | ENSP00000387019 | UXS1 | 4.507E-05 | -0.209 | - | - |
| 9634 | ENSP00000262962 | CCDC94 | 1.177E-05 | -0.209 | - | - |
| 9635 | ENSP00000318016 | KIAA0196 | 1.404E-05 | -0.209 | - | - |
| 9636 | ENSP00000297988 | AQP7 | 1.434E-05 | -0.209 | - | - |
| 9637 | ENSP00000256031 | ATP13A3 | 1.941E-05 | -0.209 | - | - |
| 9638 | ENSP00000308546 | MEPCE | 1.626E-05 | -0.209 | - | - |
| 9639 | ENSP00000244496 | RRP36 | 1.425E-05 | -0.209 | - | - |
| 9640 | ENSP00000386146 | C6orf226 | 1.154E-07 | -0.209 | - | - |
| 9641 | ENSP00000306999 | ESCO2 | 1.655E-05 | -0.209 | - | - |
| 9642 | ENSP00000265686 | TCIRG1 | 1.478E-05 | -0.209 | - | - |
| 9643 | ENSP00000262177 | DNAJB6 | 1.496E-05 | -0.209 | - | - |
| 9644 | ENSP00000318066 | ENPP4 | 1.863E-05 | -0.209 | - | - |
| 9645 | ENSP00000342032 | BPGM | 2.004E-05 | -0.209 | - | - |
| 9646 | ENSP00000276062 | NDUFB11 | 1.644E-05 | -0.209 | - | - |
| 9647 | ENSP00000353538 | LRRCC1 | 7.479E-06 | -0.209 | - | - |
| 9648 | ENSP00000264047 | SLC5A7 | 1.381E-05 | -0.209 | - | - |
| 9649 | ENSP00000233025 | SPCS1 | 9.095E-06 | -0.209 | - | - |
| 9650 | ENSP00000263187 | MSH4 | 2.311E-05 | -0.209 | - | - |
| 9651 | ENSP00000370410 | MPDZ | 2.882E-05 | -0.209 | - | - |
| 9652 | ENSP00000295899 | THOC7 | 3.048E-05 | -0.209 | - | - |
| 9653 | ENSP00000358423 | RRAGD | 2.952E-05 | -0.209 | - | - |
| 9654 | ENSP00000295065 | MEMO1 | 1.306E-05 | -0.209 | - | - |
| 9655 | ENSP00000379839 | GNE | 2.744E-05 | -0.209 | - | - |
| 9656 | ENSP00000363242 | PGBD3 | 1.922E-05 | -0.209 | - | - |
| 9657 | ENSP00000279242 | MRPL49 | 1.896E-05 | -0.209 | - | - |
| 9658 | ENSP00000310440 | CHMP2A | 2.074E-05 | -0.209 | - | - |
| 9659 | ENSP00000385746 | SMS | 1.932E-05 | -0.209 | - | - |
| 9660 | ENSP00000327009 | CLIP4 | 1.593E-05 | -0.209 | - | - |
| 9661 | ENSP00000251643 | KRT12 | 3.034E-05 | -0.209 | - | - |
| 9662 | ENSP00000253686 | MRPS25 | 1.064E-05 | -0.209 | - | - |
| 9663 | ENSP00000320543 | EPN2 | 1.065E-05 | -0.209 | - | - |
| 9664 | ENSP00000263073 | SMG6 | 1.279E-05 | -0.209 | - | - |
| 9665 | ENSP00000300738 | RRM1 | 4.317E-05 | -0.209 | - | - |
| 9666 | ENSP00000295926 | CCNL1 | 1.118E-05 | -0.209 | - | - |
| 9667 | ENSP00000274695 | CDKAL1 | 4.524E-05 | -0.209 | - | - |
| 9668 | ENSP00000331827 | TNFAIP8L1 | 1.428E-05 | -0.209 | - | - |
| 9669 | ENSP00000312981 | USP39 | 1.235E-05 | -0.209 | - | - |
| 9670 | ENSP00000410890 | ZNF783 | 9.552E-06 | -0.209 | - | - |
| 9671 | ENSP00000263246 | PACSIN2 | 5.919E-05 | -0.209 | - | - |
| 9672 | ENSP00000260810 | TOPBP1 | 1.745E-05 | -0.209 | - | - |
| 9673 | ENSP00000469018 | CTD-3138B18.4 | 9.626E-06 | -0.209 | - | - |
| 9674 | ENSP00000289352 | HIST1H4H | 1.589E-05 | -0.209 | - | - |
| 9675 | ENSP00000324127 | PSD3 | 1.305E-05 | -0.209 | - | - |
| 9676 | ENSP00000279263 | TM7SF2 | 1.665E-05 | -0.209 | - | - |
| 9677 | ENSP00000306968 | CDCA7 | 1.224E-05 | -0.209 | - | - |
| 9678 | ENSP00000217173 | UBOX5 | 2.549E-05 | -0.209 | - | - |
| 9679 | ENSP00000222969 | BUD31 | 1.524E-05 | -0.209 | - | - |
| 9680 | ENSP00000268124 | POLG | 1.554E-05 | -0.209 | - | - |
| 9681 | ENSP00000232458 | ECT2 | 1.966E-05 | -0.209 | - | - |
| 9682 | ENSP00000343081 | PUS7L | 1.108E-05 | -0.209 | - | - |
| 9683 | ENSP00000385461 | TFDP3 | 1.904E-05 | -0.209 | - | - |
| 9684 | ENSP00000328340 | RNF135 | 2.354E-05 | -0.209 | - | - |
| 9685 | ENSP00000310621 | HHATL | 1.726E-05 | -0.209 | - | - |
| 9686 | ENSP00000229238 | MRPL51 | 1.065E-05 | -0.209 | - | - |
| 9687 | ENSP00000339340 | KCTD21 | 2.829E-05 | -0.209 | - | - |
| 9688 | ENSP00000263354 | NAPA | 1.145E-05 | -0.209 | - | - |
| 9689 | ENSP00000371084 | ATP8A1 | 4.359E-05 | -0.209 | - | - |
| 9690 | ENSP00000303077 | GOT1L1 | 1.680E-05 | -0.209 | - | - |
| 9691 | ENSP00000265593 | CLCN2 | 1.288E-05 | -0.209 | - | - |
| 9692 | ENSP00000333181 | 11-Mar | 1.868E-05 | -0.209 | - | - |
| 9693 | ENSP00000295454 | GABRB1 | 2.191E-05 | -0.209 | - | - |
| 9694 | ENSP00000381282 | VIMP | 2.890E-05 | -0.209 | - | - |
| 9695 | ENSP00000270538 | TIMM44 | 3.359E-05 | -0.209 | - | - |
| 9696 | ENSP00000262544 | SEC23B | 1.717E-05 | -0.209 | - | - |
| 9697 | ENSP00000334100 | EXOC7 | 1.195E-05 | -0.209 | - | - |
| 9698 | ENSP00000265428 | WWP1 | 2.571E-05 | -0.209 | - | - |
| 9699 | ENSP00000330216 | TRIM49B | 1.950E-05 | -0.210 | - | - |
| 9700 | ENSP00000379616 | MYH11 | 3.596E-05 | -0.210 | - | - |
| 9701 | ENSP00000221418 | ECH1 | 1.491E-05 | -0.210 | - | - |
| 9702 | ENSP00000440698 | DBI | 2.454E-05 | -0.210 | - | - |
| 9703 | ENSP00000347451 | LINGO1 | 1.377E-05 | -0.210 | - | - |
| 9704 | ENSP00000302978 | SPRY3 | 2.896E-05 | -0.210 | - | - |
| 9705 | ENSP00000338487 | MGAT4B | 2.067E-05 | -0.210 | - | - |
| 9706 | ENSP00000230565 | ENPP5 | 1.237E-05 | -0.210 | - | - |
| 9707 | ENSP00000319622 | SULT1C2 | 1.288E-05 | -0.210 | - | - |
| 9708 | ENSP00000245157 | BBS2 | 4.489E-05 | -0.210 | - | - |
| 9709 | ENSP00000262043 | PHF3 | 1.138E-05 | -0.210 | - | - |
| 9710 | ENSP00000296473 | MON1A | 1.138E-05 | -0.210 | - | - |
| 9711 | ENSP00000310590 | USP36 | 2.361E-05 | -0.210 | - | - |
| 9712 | ENSP00000333836 | FIGN | 2.841E-05 | -0.210 | - | - |
| 9713 | ENSP00000248846 | TUBGCP6 | 1.459E-05 | -0.210 | - | - |
| 9714 | ENSP00000349356 | FIGNL1 | 4.715E-05 | -0.210 | - | - |
| 9715 | ENSP00000385122 | SYCE3 | 1.170E-05 | -0.210 | - | - |
| 9716 | ENSP00000326170 | RFFL | 1.408E-05 | -0.210 | - | - |
| 9717 | ENSP00000338283 | NPAS2 | 3.351E-05 | -0.210 | - | - |
| 9718 | ENSP00000309542 | PGA5 | 1.969E-05 | -0.210 | - | - |
| 9719 | ENSP00000262306 | TCEB2 | 1.922E-05 | -0.210 | - | - |
| 9720 | ENSP00000256319 | C14orf1 | 1.287E-05 | -0.210 | - | - |
| 9721 | ENSP00000315775 | COG4 | 1.061E-05 | -0.210 | - | - |
| 9722 | ENSP00000389381 | YY2 | 2.090E-05 | -0.210 | - | - |
| 9723 | ENSP00000244221 | PAIP2B | 1.238E-05 | -0.210 | - | - |
| 9724 | ENSP00000298832 | TTLL5 | 1.409E-05 | -0.210 | - | - |
| 9725 | ENSP00000362508 | UGT1A4 | 1.334E-05 | -0.210 | - | - |
| 9726 | ENSP00000316598 | GTPBP6 | 1.584E-05 | -0.210 | - | - |
| 9727 | ENSP00000334280 | TACC2 | 1.590E-05 | -0.210 | - | - |
| 9728 | ENSP00000333657 | MX2 | 3.443E-05 | -0.210 | - | - |
| 9729 | ENSP00000312671 | EHBP1L1 | 2.258E-05 | -0.210 | - | - |
| 9730 | ENSP00000245680 | SLC35F5 | 1.060E-05 | -0.210 | - | - |
| 9731 | ENSP00000387088 | HNRPLL | 1.041E-05 | -0.210 | - | - |
| 9732 | ENSP00000265594 | MCCC1 | 9.493E-06 | -0.210 | - | - |
| 9733 | ENSP00000267499 | ADAM21 | 3.211E-05 | -0.210 | - | - |
| 9734 | ENSP00000366637 | FOXD4L5 | 9.453E-06 | -0.210 | - | - |
| 9735 | ENSP00000265465 | POLA2 | 1.155E-05 | -0.210 | - | - |
| 9736 | ENSP00000313385 | RELL1 | 4.449E-05 | -0.210 | - | - |
| 9737 | ENSP00000292782 | DCUN1D1 | 1.404E-05 | -0.210 | - | - |
| 9738 | ENSP00000334962 | TMPRSS6 | 2.219E-05 | -0.210 | - | - |
| 9739 | ENSP00000292363 | RNF126 | 2.408E-05 | -0.210 | - | - |
| 9740 | ENSP00000353739 | PCMTD1 | 1.284E-05 | -0.210 | - | - |
| 9741 | ENSP00000303709 | UBE2E1 | 2.814E-05 | -0.210 | - | - |
| 9742 | ENSP00000264161 | DARS | 1.593E-05 | -0.210 | - | - |
| 9743 | ENSP00000287878 | PRKAG2 | 2.943E-05 | -0.210 | - | - |
| 9744 | ENSP00000233623 | TTC31 | 1.525E-05 | -0.210 | - | - |
| 9745 | ENSP00000321703 | TACC1 | 1.390E-05 | -0.210 | - | - |
| 9746 | ENSP00000228740 | LTA4H | 5.842E-05 | -0.210 | - | - |
| 9747 | ENSP00000358815 | NEURL1B | 4.549E-05 | -0.210 | - | - |
| 9748 | ENSP00000267884 | SRP14 | 2.645E-05 | -0.210 | - | - |
| 9749 | ENSP00000326841 | SPATS2 | 7.192E-05 | -0.210 | - | - |
| 9750 | ENSP00000325905 | SRSF7 | 1.828E-05 | -0.210 | - | - |
| 9751 | ENSP00000370128 | SLC7A1 | 2.060E-05 | -0.210 | - | - |
| 9752 | ENSP00000306894 | ETV5 | 4.712E-05 | -0.210 | - | - |
| 9753 | ENSP00000386076 | GUCD1 | 7.211E-06 | -0.210 | - | - |
| 9754 | ENSP00000336725 | DDX3Y | 2.667E-05 | -0.210 | - | - |
| 9755 | ENSP00000260867 | TIMM23 | 1.083E-05 | -0.210 | - | - |
| 9756 | ENSP00000295598 | ATP1A1 | 1.987E-05 | -0.210 | - | - |
| 9757 | ENSP00000354686 | ZNF652 | 1.462E-05 | -0.210 | - | - |
| 9758 | ENSP00000245932 | VASP | 3.973E-05 | -0.210 | - | - |
| 9759 | ENSP00000341045 | UGT2B15 | 1.992E-05 | -0.210 | - | - |
| 9760 | ENSP00000311401 | DENND6A | 2.909E-05 | -0.210 | - | - |
| 9761 | ENSP00000405699 | ZNF837 | 9.435E-06 | -0.210 | - | - |
| 9762 | ENSP00000315950 | RNF32 | 2.433E-05 | -0.210 | - | - |
| 9763 | ENSP00000281828 | FARSB | 1.518E-05 | -0.210 | - | - |
| 9764 | ENSP00000321744 | HIST1H2BC | 2.027E-05 | -0.210 | - | - |
| 9765 | ENSP00000225729 | DRG2 | 1.256E-05 | -0.210 | - | - |
| 9766 | ENSP00000361043 | RAD54L | 1.569E-05 | -0.210 | - | - |
| 9767 | ENSP00000341961 | FOXD4L3 | 9.448E-06 | -0.210 | - | - |
| 9768 | ENSP00000299166 | NDUFB8 | 2.550E-05 | -0.210 | - | - |
| 9769 | ENSP00000371070 | ATP8A2 | 4.474E-05 | -0.210 | - | - |
| 9770 | ENSP00000216442 | ATP6V1D | 1.205E-05 | -0.210 | - | - |
| 9771 | ENSP00000266070 | DIDO1 | 1.437E-05 | -0.210 | - | - |
| 9772 | ENSP00000242872 | CENPK | 1.228E-05 | -0.210 | - | - |
| 9773 | ENSP00000326884 | ARFRP1 | 1.810E-05 | -0.210 | - | - |
| 9774 | ENSP00000253108 | EIF3G | 1.395E-05 | -0.210 | - | - |
| 9775 | ENSP00000450085 | RNASEK-C17orf49 | 6.636E-06 | -0.210 | - | - |
| 9776 | ENSP00000300900 | CA4 | 2.471E-05 | -0.210 | - | - |
| 9777 | ENSP00000257910 | TSPAN31 | 5.434E-05 | -0.210 | - | - |
| 9778 | ENSP00000378338 | GIT1 | 3.750E-05 | -0.210 | - | - |
| 9779 | ENSP00000222008 | RABAC1 | 1.233E-05 | -0.210 | - | - |
| 9780 | ENSP00000410769 | CNBP | 1.552E-05 | -0.210 | - | - |
| 9781 | ENSP00000246551 | HCST | 2.334E-04 | -0.210 | - | - |
| 9782 | ENSP00000327738 | TRIML1 | 1.903E-05 | -0.210 | - | - |
| 9783 | ENSP00000352835 | MB | 2.929E-05 | -0.210 | - | - |
| 9784 | ENSP00000365014 | NXNL2 | 1.270E-05 | -0.210 | - | - |
| 9785 | ENSP00000319240 | MAGOHB | 2.097E-05 | -0.210 | - | - |
| 9786 | ENSP00000353701 | DPP3 | 1.741E-05 | -0.210 | - | - |
| 9787 | ENSP00000377055 | PRCP | 2.663E-05 | -0.210 | - | - |
| 9788 | ENSP00000304102 | COPS6 | 2.972E-05 | -0.210 | - | - |
| 9789 | ENSP00000239944 | SERP1 | 1.237E-05 | -0.210 | - | - |
| 9790 | ENSP00000323659 | KDM3A | 1.828E-05 | -0.210 | - | - |
| 9791 | ENSP00000247665 | PHPT1 | 1.254E-05 | -0.210 | - | - |
| 9792 | ENSP00000268125 | RLBP1 | 1.224E-05 | -0.210 | - | - |
| 9793 | ENSP00000459789 | RCOR1 | 1.985E-05 | -0.210 | - | - |
| 9794 | ENSP00000327675 | LRRC14B | 2.900E-05 | -0.210 | - | - |
| 9795 | ENSP00000378506 | MSANTD3 | 1.344E-04 | -0.210 | - | - |
| 9796 | ENSP00000216500 | DHRS7 | 1.194E-05 | -0.210 | - | - |
| 9797 | ENSP00000289119 | ABHD3 | 1.048E-05 | -0.211 | - | - |
| 9798 | ENSP00000309415 | CLTB | 1.205E-05 | -0.211 | - | - |
| 9799 | ENSP00000274547 | GABRB2 | 1.908E-05 | -0.211 | - | - |
| 9800 | ENSP00000322524 | DCTPP1 | 6.235E-05 | -0.211 | - | - |
| 9801 | ENSP00000412922 | URM1 | 1.931E-05 | -0.211 | - | - |
| 9802 | ENSP00000426964 | YJEFN3 | 1.020E-05 | -0.211 | - | - |
| 9803 | ENSP00000327604 | TRIM49 | 1.827E-05 | -0.211 | - | - |
| 9804 | ENSP00000332118 | EPHB3 | 3.050E-05 | -0.211 | - | - |
| 9805 | ENSP00000321821 | CYP4F12 | 1.911E-05 | -0.211 | - | - |
| 9806 | ENSP00000264637 | THRA | 2.743E-05 | -0.211 | - | - |
| 9807 | ENSP00000415011 | SLC30A8 | 4.084E-05 | -0.211 | - | - |
| 9808 | ENSP00000360671 | SLC25A5 | 2.267E-05 | -0.211 | - | - |
| 9809 | ENSP00000362618 | PABPC1L2A | 1.107E-05 | -0.211 | - | - |
| 9810 | ENSP00000332969 | TRIM64B | 1.590E-05 | -0.211 | - | - |
| 9811 | ENSP00000258499 | USP44 | 1.138E-05 | -0.211 | - | - |
| 9812 | ENSP00000248633 | PEX1 | 1.295E-05 | -0.211 | - | - |
| 9813 | ENSP00000366843 | ATXN2 | 5.571E-05 | -0.211 | - | - |
| 9814 | ENSP00000264318 | GABRA4 | 1.147E-05 | -0.211 | - | - |
| 9815 | ENSP00000324205 | CHMP4A | 1.913E-05 | -0.211 | - | - |
| 9816 | ENSP00000372278 | KRTAP20-4 | 3.748E-07 | -0.211 | - | - |
| 9817 | ENSP00000337466 | WFDC10B | 7.547E-06 | -0.211 | - | - |
| 9818 | ENSP00000342812 | USP9Y | 2.280E-05 | -0.211 | - | - |
| 9819 | ENSP00000380762 | TEN1 | 1.708E-05 | -0.211 | - | - |
| 9820 | ENSP00000366915 | KLF5 | 2.929E-05 | -0.211 | - | - |
| 9821 | ENSP00000263093 | SLC27A5 | 1.182E-05 | -0.211 | - | - |
| 9822 | ENSP00000358778 | LRIF1 | 2.790E-05 | -0.211 | - | - |
| 9823 | ENSP00000054666 | VAMP3 | 2.672E-05 | -0.211 | - | - |
| 9824 | ENSP00000221566 | SGTA | 1.687E-05 | -0.211 | - | - |
| 9825 | ENSP00000369538 | GDI2 | 1.386E-05 | -0.211 | - | - |
| 9826 | ENSP00000262764 | PGS1 | 1.552E-05 | -0.211 | - | - |
| 9827 | ENSP00000301458 | CD320 | 3.488E-05 | -0.211 | - | - |
| 9828 | ENSP00000258123 | USP15 | 2.177E-05 | -0.211 | - | - |
| 9829 | ENSP00000299543 | CTDP1 | 1.317E-05 | -0.211 | - | - |
| 9830 | ENSP00000267119 | KRT71 | 3.244E-05 | -0.211 | - | - |
| 9831 | ENSP00000262570 | CHCHD3 | 1.009E-05 | -0.211 | - | - |
| 9832 | ENSP00000312435 | DAG1 | 4.816E-05 | -0.211 | - | - |
| 9833 | ENSP00000296869 | ACSL6 | 1.738E-05 | -0.211 | - | - |
| 9834 | ENSP00000206765 | TGM1 | 4.236E-05 | -0.211 | - | - |
| 9835 | ENSP00000298085 | SLC7A3 | 1.604E-05 | -0.211 | - | - |
| 9836 | ENSP00000259037 | NDUFB5 | 1.068E-05 | -0.211 | - | - |
| 9837 | ENSP00000335651 | FSD2 | 1.340E-05 | -0.211 | - | - |
| 9838 | ENSP00000280612 | SLC7A11 | 2.106E-05 | -0.211 | - | - |
| 9839 | ENSP00000252699 | ACTN4 | 3.804E-05 | -0.211 | - | - |
| 9840 | ENSP00000233468 | SF3B14 | 1.361E-05 | -0.211 | - | - |
| 9841 | ENSP00000322180 | DSCC1 | 9.308E-06 | -0.211 | - | - |
| 9842 | ENSP00000444788 | TDO2 | 3.058E-05 | -0.211 | - | - |
| 9843 | ENSP00000273541 | ISY1 | 1.162E-05 | -0.211 | - | - |
| 9844 | ENSP00000247655 | COX7C | 1.163E-05 | -0.211 | - | - |
| 9845 | ENSP00000346120 | DDX21 | 3.408E-05 | -0.211 | - | - |
| 9846 | ENSP00000358765 | TPBG | 1.501E-05 | -0.211 | - | - |
| 9847 | ENSP00000359353 | GABRE | 5.985E-06 | -0.211 | - | - |
| 9848 | ENSP00000355599 | TSNAX | 2.723E-05 | -0.211 | - | - |
| 9849 | ENSP00000351790 | MYPN | 2.387E-05 | -0.211 | - | - |
| 9850 | ENSP00000312356 | TRMT10C | 7.838E-06 | -0.211 | - | - |
| 9851 | ENSP00000220966 | PYCRL | 1.151E-05 | -0.211 | - | - |
| 9852 | ENSP00000245407 | SLC22A5 | 9.805E-05 | -0.211 | - | - |
| 9853 | ENSP00000310688 | ABCD2 | 1.354E-05 | -0.211 | - | - |
| 9854 | ENSP00000338397 | - | 1.604E-05 | -0.211 | - | - |
| 9855 | ENSP00000414906 | DCTN4 | 2.220E-05 | -0.211 | - | - |
| 9856 | ENSP00000318355 | AQP10 | 1.342E-05 | -0.211 | - | - |
| 9857 | ENSP00000327077 | PCM1 | 1.542E-05 | -0.211 | - | - |
| 9858 | ENSP00000310749 | HPCAL1 | 1.302E-05 | -0.211 | - | - |
| 9859 | ENSP00000407724 | CSDE1 | 1.159E-05 | -0.211 | - | - |
| 9860 | ENSP00000261708 | UTP6 | 1.462E-05 | -0.211 | - | - |
| 9861 | ENSP00000385099 | ZNF792 | 1.118E-05 | -0.211 | - | - |
| 9862 | ENSP00000340466 | GANAB | 1.252E-05 | -0.211 | - | - |
| 9863 | ENSP00000246071 | SNRPB2 | 1.446E-05 | -0.211 | - | - |
| 9864 | ENSP00000358795 | NEURL | 3.687E-05 | -0.211 | - | - |
| 9865 | ENSP00000257745 | MLL5 | 1.988E-05 | -0.211 | - | - |
| 9866 | ENSP00000232375 | PFKFB4 | 9.855E-06 | -0.211 | - | - |
| 9867 | ENSP00000400258 | DLGAP2 | 8.605E-06 | -0.211 | - | - |
| 9868 | ENSP00000320646 | ACSF3 | 1.088E-05 | -0.211 | - | - |
| 9869 | ENSP00000160827 | KIF22 | 1.082E-05 | -0.211 | - | - |
| 9870 | ENSP00000367637 | MRS2 | 3.449E-05 | -0.211 | - | - |
| 9871 | ENSP00000229268 | USP5 | 1.775E-05 | -0.211 | - | - |
| 9872 | ENSP00000280886 | DIP2C | 1.296E-05 | -0.211 | - | - |
| 9873 | ENSP00000365057 | GPANK1 | 4.626E-06 | -0.211 | - | - |
| 9874 | ENSP00000277865 | GLUD1 | 3.943E-05 | -0.211 | - | - |
| 9875 | ENSP00000341361 | RNF13 | 1.893E-05 | -0.211 | - | - |
| 9876 | ENSP00000344106 | RTN3 | 1.303E-05 | -0.211 | - | - |
| 9877 | ENSP00000381064 | INTS10 | 2.200E-05 | -0.211 | - | - |
| 9878 | ENSP00000356357 | LHX9 | 4.350E-05 | -0.211 | - | - |
| 9879 | ENSP00000402915 | C2orf74 | 3.406E-07 | -0.211 | - | - |
| 9880 | ENSP00000301475 | ZNF558 | 2.714E-05 | -0.211 | - | - |
| 9881 | ENSP00000299162 | FOXN4 | 9.148E-06 | -0.211 | - | - |
| 9882 | ENSP00000264930 | SLC12A7 | 1.058E-05 | -0.211 | - | - |
| 9883 | ENSP00000369009 | CXorf23 | 2.540E-05 | -0.211 | - | - |
| 9884 | ENSP00000258742 | NUPL2 | 1.412E-05 | -0.211 | - | - |
| 9885 | ENSP00000337510 | FBXO40 | 2.321E-05 | -0.211 | - | - |
| 9886 | ENSP00000281830 | KCNE4 | 8.225E-06 | -0.211 | - | - |
| 9887 | ENSP00000312599 | TMEM70 | 1.320E-05 | -0.211 | - | - |
| 9888 | ENSP00000267996 | TPM1 | 3.638E-05 | -0.212 | - | - |
| 9889 | ENSP00000368552 | SIRT5 | 3.712E-05 | -0.212 | - | - |
| 9890 | ENSP00000311202 | KCTD13 | 1.905E-05 | -0.212 | - | - |
| 9891 | ENSP00000310448 | SART1 | 1.675E-05 | -0.212 | - | - |
| 9892 | ENSP00000376176 | RAN | 2.494E-05 | -0.212 | - | - |
| 9893 | ENSP00000265512 | ADH4 | 1.317E-05 | -0.212 | - | - |
| 9894 | ENSP00000338481 | EPB41L2 | 1.692E-05 | -0.212 | - | - |
| 9895 | ENSP00000347055 | MYL4 | 2.061E-05 | -0.212 | - | - |
| 9896 | ENSP00000348020 | MLH3 | 1.173E-05 | -0.212 | - | - |
| 9897 | ENSP00000383986 | CDCA7L | 4.751E-05 | -0.212 | - | - |
| 9898 | ENSP00000240922 | NAA50 | 1.776E-05 | -0.212 | - | - |
| 9899 | ENSP00000362621 | PABPC1L2B | 1.107E-05 | -0.212 | - | - |
| 9900 | ENSP00000333253 | ZC3H11A | 1.690E-05 | -0.212 | - | - |
| 9901 | ENSP00000382241 | QSER1 | 1.531E-05 | -0.212 | - | - |
| 9902 | ENSP00000243997 | ATP5E | 9.700E-06 | -0.212 | - | - |
| 9903 | ENSP00000326699 | CLGN | 1.131E-05 | -0.212 | - | - |
| 9904 | ENSP00000245838 | THOC2 | 1.648E-05 | -0.212 | - | - |
| 9905 | ENSP00000310723 | DDX23 | 1.214E-05 | -0.212 | - | - |
| 9906 | ENSP00000200135 | ZW10 | 1.079E-05 | -0.212 | - | - |
| 9907 | ENSP00000221265 | PAF1 | 1.261E-05 | -0.212 | - | - |
| 9908 | ENSP00000258145 | GNS | 2.435E-05 | -0.212 | - | - |
| 9909 | ENSP00000251127 | NALCN | 8.440E-06 | -0.212 | - | - |
| 9910 | ENSP00000380349 | CAPN3 | 3.367E-05 | -0.212 | - | - |
| 9911 | ENSP00000359990 | LRRC40 | 7.498E-06 | -0.212 | - | - |
| 9912 | ENSP00000362609 | ZBTB8A | 1.218E-05 | -0.212 | - | - |
| 9913 | ENSP00000309431 | RP11-683L23.1 | 1.556E-05 | -0.212 | - | - |
| 9914 | ENSP00000040877 | TARBP1 | 8.997E-06 | -0.212 | - | - |
| 9915 | ENSP00000285046 | FGD5 | 2.407E-05 | -0.212 | - | - |
| 9916 | ENSP00000372105 | RBMY1E | 5.562E-06 | -0.212 | - | - |
| 9917 | ENSP00000323050 | RBBP8 | 1.999E-05 | -0.212 | - | - |
| 9918 | ENSP00000392678 | AKIRIN1 | 5.774E-06 | -0.212 | - | - |
| 9919 | ENSP00000307666 | CCDC132 | 3.633E-05 | -0.212 | - | - |
| 9920 | ENSP00000320291 | OSBPL1A | 2.689E-05 | -0.212 | - | - |
| 9921 | ENSP00000275820 | NOM1 | 1.086E-05 | -0.212 | - | - |
| 9922 | ENSP00000298681 | SLC39A2 | 1.889E-05 | -0.212 | - | - |
| 9923 | ENSP00000321320 | FAM203A | 7.498E-06 | -0.212 | - | - |
| 9924 | ENSP00000350848 | HMGN5 | 1.265E-05 | -0.212 | - | - |
| 9925 | ENSP00000156471 | AQR | 1.269E-05 | -0.212 | - | - |
| 9926 | ENSP00000362968 | CLPSL1 | 1.204E-05 | -0.212 | - | - |
| 9927 | ENSP00000311429 | ZWILCH | 1.165E-05 | -0.212 | - | - |
| 9928 | ENSP00000267973 | WDR61 | 1.120E-05 | -0.212 | - | - |
| 9929 | ENSP00000404042 | TMED4 | 2.627E-05 | -0.212 | - | - |
| 9930 | ENSP00000311320 | LYG1 | 1.293E-05 | -0.212 | - | - |
| 9931 | ENSP00000357565 | ZUFSP | 1.236E-05 | -0.212 | - | - |
| 9932 | ENSP00000312995 | CLSPN | 1.440E-05 | -0.212 | - | - |
| 9933 | ENSP00000261745 | NAA25 | 1.111E-04 | -0.212 | - | - |
| 9934 | ENSP00000261396 | NUP133 | 1.285E-05 | -0.212 | - | - |
| 9935 | ENSP00000244230 | MPHOSPH10 | 1.188E-05 | -0.212 | - | - |
| 9936 | ENSP00000404127 | ZNF709 | 1.000E-05 | -0.212 | - | - |
| 9937 | ENSP00000257694 | PNPLA8 | 1.613E-05 | -0.212 | - | - |
| 9938 | ENSP00000327070 | MDH2 | 2.057E-05 | -0.212 | - | - |
| 9939 | ENSP00000287482 | SASS6 | 2.324E-05 | -0.212 | - | - |
| 9940 | ENSP00000296684 | NDUFS4 | 1.035E-05 | -0.212 | - | - |
| 9941 | ENSP00000455607 | PHKG2 | 2.976E-05 | -0.212 | - | - |
| 9942 | ENSP00000261881 | TIPIN | 1.214E-05 | -0.212 | - | - |
| 9943 | ENSP00000303145 | TMED10 | 1.646E-05 | -0.212 | - | - |
| 9944 | ENSP00000448220 | KRT4 | 2.151E-05 | -0.212 | - | - |
| 9945 | ENSP00000440586 | USO1 | 3.707E-05 | -0.212 | - | - |
| 9946 | ENSP00000391116 | NBPF12 | 1.015E-05 | -0.212 | - | - |
| 9947 | ENSP00000225665 | SLC25A11 | 1.179E-05 | -0.212 | - | - |
| 9948 | ENSP00000294189 | RPL29 | 1.170E-05 | -0.212 | - | - |
| 9949 | ENSP00000264448 | ALMS1 | 9.915E-06 | -0.212 | - | - |
| 9950 | ENSP00000275233 | SHPRH | 1.498E-05 | -0.212 | - | - |
| 9951 | ENSP00000349494 | ZNF254 | 1.071E-05 | -0.212 | - | - |
| 9952 | ENSP00000259008 | BRIP1 | 1.230E-05 | -0.212 | - | - |
| 9953 | ENSP00000215591 | POLRMT | 1.228E-05 | -0.212 | - | - |
| 9954 | ENSP00000302239 | USP8 | 2.250E-05 | -0.212 | - | - |
| 9955 | ENSP00000295930 | RSRC1 | 1.434E-05 | -0.212 | - | - |
| 9956 | ENSP00000016171 | COX15 | 9.857E-06 | -0.212 | - | - |
| 9957 | ENSP00000258975 | TACO1 | 1.105E-05 | -0.212 | - | - |
| 9958 | ENSP00000237449 | DNAH6 | 1.116E-05 | -0.212 | - | - |
| 9959 | ENSP00000326550 | TACC3 | 1.288E-05 | -0.212 | - | - |
| 9960 | ENSP00000308948 | PTCD2 | 1.245E-05 | -0.212 | - | - |
| 9961 | ENSP00000253303 | RGN | 1.341E-05 | -0.212 | - | - |
| 9962 | ENSP00000375844 | CAPN10 | 3.145E-05 | -0.212 | - | - |
| 9963 | ENSP00000354621 | SMURF1 | 5.192E-05 | -0.212 | - | - |
| 9964 | ENSP00000284811 | TCEB1 | 1.999E-05 | -0.212 | - | - |
| 9965 | ENSP00000422344 | ZNF550 | 1.260E-05 | -0.212 | - | - |
| 9966 | ENSP00000265388 | TNPO3 | 1.090E-04 | -0.212 | - | - |
| 9967 | ENSP00000245222 | SPHK2 | 6.050E-05 | -0.212 | - | - |
| 9968 | ENSP00000253188 | SLC7A10 | 1.103E-05 | -0.212 | - | - |
| 9969 | ENSP00000225724 | GOSR1 | 1.247E-05 | -0.212 | - | - |
| 9970 | ENSP00000202816 | ESF1 | 8.842E-06 | -0.212 | - | - |
| 9971 | ENSP00000219473 | USP10 | 1.260E-05 | -0.212 | - | - |
| 9972 | ENSP00000262839 | TRPC5 | 3.031E-05 | -0.212 | - | - |
| 9973 | ENSP00000305459 | COG8 | 9.932E-06 | -0.212 | - | - |
| 9974 | ENSP00000270570 | SLC47A1 | 9.208E-06 | -0.212 | - | - |
| 9975 | ENSP00000318914 | PNMA1 | 2.527E-05 | -0.213 | - | - |
| 9976 | ENSP00000243457 | KCNJ2 | 1.863E-05 | -0.213 | - | - |
| 9977 | ENSP00000395340 | FAM185A | 2.019E-07 | -0.213 | - | - |
| 9978 | ENSP00000283033 | TXNDC11 | 1.880E-05 | -0.213 | - | - |
| 9979 | ENSP00000400932 | SLC35G1 | 4.754E-06 | -0.213 | - | - |
| 9980 | ENSP00000310520 | ERCC4 | 1.655E-05 | -0.213 | - | - |
| 9981 | ENSP00000322582 | C4orf17 | 2.036E-05 | -0.213 | - | - |
| 9982 | ENSP00000365169 | C9orf153 | 1.136E-05 | -0.213 | - | - |
| 9983 | ENSP00000254759 | COQ3 | 7.396E-06 | -0.213 | - | - |
| 9984 | ENSP00000396936 | C17orf72 | 1.460E-06 | -0.213 | - | - |
| 9985 | ENSP00000356375 | ZBTB41 | 3.251E-05 | -0.213 | - | - |
| 9986 | ENSP00000263278 | HSD17B14 | 2.292E-05 | -0.213 | - | - |
| 9987 | ENSP00000444408 | SLC1A5 | 2.888E-05 | -0.213 | - | - |
| 9988 | ENSP00000219345 | PLA2G15 | 8.645E-06 | -0.213 | - | - |
| 9989 | ENSP00000332288 | TRIM61 | 2.195E-05 | -0.213 | - | - |
| 9990 | ENSP00000335628 | SPDYA | 7.885E-06 | -0.213 | - | - |
| 9991 | ENSP00000292095 | BACE1 | 5.234E-05 | -0.213 | - | - |
| 9992 | ENSP00000355402 | ISLR2 | 1.082E-05 | -0.213 | - | - |
| 9993 | ENSP00000260197 | SORL1 | 2.337E-05 | -0.213 | - | - |
| 9994 | ENSP00000360120 | ROR1 | 2.474E-05 | -0.213 | - | - |
| 9995 | ENSP00000407653 | PRR22 | 5.087E-07 | -0.213 | - | - |
| 9996 | ENSP00000265564 | EXOSC7 | 1.203E-05 | -0.213 | - | - |
| 9997 | ENSP00000297338 | RAD21 | 2.510E-05 | -0.213 | - | - |
| 9998 | ENSP00000379054 | ZNF488 | 1.058E-05 | -0.213 | - | - |
| 9999 | ENSP00000262966 | MPND | 1.654E-05 | -0.213 | - | - |
| 10000 | ENSP00000297508 | MICALL2 | 1.497E-05 | -0.213 | - | - |
| 10001 | ENSP00000317379 | GLS | 5.440E-05 | -0.213 | - | - |
| 10002 | ENSP00000356480 | RNF2 | 5.594E-05 | -0.213 | - | - |
| 10003 | ENSP00000333934 | TRMT11 | 8.615E-06 | -0.213 | - | - |
| 10004 | ENSP00000281950 | GEMIN6 | 9.116E-05 | -0.213 | - | - |
| 10005 | ENSP00000297991 | AQP3 | 3.114E-05 | -0.213 | - | - |
| 10006 | ENSP00000265965 | SERGEF | 1.311E-05 | -0.213 | - | - |
| 10007 | ENSP00000326572 | ANKRD62 | 1.220E-05 | -0.213 | - | - |
| 10008 | ENSP00000289004 | HPD | 9.562E-05 | -0.213 | - | - |
| 10009 | ENSP00000341422 | P4HTM | 2.841E-05 | -0.213 | - | - |
| 10010 | ENSP00000215574 | CDC34 | 1.289E-05 | -0.213 | - | - |
| 10011 | ENSP00000334246 | CYP4Z1 | 1.267E-05 | -0.213 | - | - |
| 10012 | ENSP00000407154 | HSD11B1L | 6.958E-06 | -0.213 | - | - |
| 10013 | ENSP00000250066 | USP6 | 1.172E-05 | -0.213 | - | - |
| 10014 | ENSP00000260818 | DNAJC13 | 1.114E-05 | -0.213 | - | - |
| 10015 | ENSP00000365693 | MSRB2 | 2.288E-05 | -0.213 | - | - |
| 10016 | ENSP00000312107 | UBXN2A | 1.475E-05 | -0.213 | - | - |
| 10017 | ENSP00000381504 | PRPS2 | 2.552E-05 | -0.213 | - | - |
| 10018 | ENSP00000358967 | OPN1LW | 2.168E-05 | -0.213 | - | - |
| 10019 | ENSP00000263579 | DCPS | 2.044E-05 | -0.213 | - | - |
| 10020 | ENSP00000358814 | AHCYL1 | 1.949E-05 | -0.213 | - | - |
| 10021 | ENSP00000273067 | 4-Mar | 1.743E-05 | -0.213 | - | - |
| 10022 | ENSP00000313995 | KLHDC3 | 9.139E-06 | -0.213 | - | - |
| 10023 | ENSP00000282344 | USP12 | 1.813E-05 | -0.213 | - | - |
| 10024 | ENSP00000333551 | PROSC | 1.182E-05 | -0.213 | - | - |
| 10025 | ENSP00000466799 | HSD17B1 | 1.066E-05 | -0.213 | - | - |
| 10026 | ENSP00000231706 | SLC7A14 | 1.091E-05 | -0.213 | - | - |
| 10027 | ENSP00000340823 | GTF2F2 | 1.683E-05 | -0.213 | - | - |
| 10028 | ENSP00000265056 | MCM2 | 1.025E-05 | -0.213 | - | - |
| 10029 | ENSP00000316114 | CCDC101 | 1.227E-05 | -0.213 | - | - |
| 10030 | ENSP00000355308 | PTPLA | 1.741E-05 | -0.213 | - | - |
| 10031 | ENSP00000230771 | HARS2 | 1.144E-05 | -0.213 | - | - |
| 10032 | ENSP00000303015 | ZRSR2 | 2.314E-05 | -0.213 | - | - |
| 10033 | ENSP00000323580 | IFT88 | 1.799E-05 | -0.213 | - | - |
| 10034 | ENSP00000279027 | SLC13A3 | 1.737E-05 | -0.213 | - | - |
| 10035 | ENSP00000298317 | RPUSD4 | 9.579E-06 | -0.213 | - | - |
| 10036 | ENSP00000384376 | TDRD15 | 1.874E-05 | -0.213 | - | - |
| 10037 | ENSP00000344659 | GLB1L2 | 1.498E-05 | -0.213 | - | - |
| 10038 | ENSP00000307889 | RPL13 | 1.528E-05 | -0.213 | - | - |
| 10039 | ENSP00000340737 | INTS12 | 1.563E-05 | -0.213 | - | - |
| 10040 | ENSP00000327427 | ZFP41 | 3.122E-05 | -0.213 | - | - |
| 10041 | ENSP00000355656 | HIST3H2A | 1.460E-05 | -0.213 | - | - |
| 10042 | ENSP00000307183 | ING2 | 1.774E-05 | -0.213 | - | - |
| 10043 | ENSP00000341680 | DTNBP1 | 1.612E-05 | -0.213 | - | - |
| 10044 | ENSP00000250237 | QTRT1 | 1.005E-05 | -0.213 | - | - |
| 10045 | ENSP00000240423 | NCAPH | 1.021E-05 | -0.213 | - | - |
| 10046 | ENSP00000361626 | YBX1 | 2.986E-05 | -0.213 | - | - |
| 10047 | ENSP00000222511 | GTPBP10 | 1.073E-05 | -0.213 | - | - |
| 10048 | ENSP00000222402 | UBE2D4 | 1.769E-05 | -0.213 | - | - |
| 10049 | ENSP00000237889 | NDUFB3 | 1.232E-05 | -0.213 | - | - |
| 10050 | ENSP00000343526 | USP1 | 1.915E-05 | -0.213 | - | - |
| 10051 | ENSP00000337125 | SMEK1 | 1.413E-05 | -0.213 | - | - |
| 10052 | ENSP00000338057 | MRPL30 | 1.035E-05 | -0.213 | - | - |
| 10053 | ENSP00000333188 | FOXL2 | 2.452E-05 | -0.213 | - | - |
| 10054 | ENSP00000297405 | CSMD3 | 1.313E-05 | -0.213 | - | - |
| 10055 | ENSP00000222547 | BET1 | 1.020E-05 | -0.213 | - | - |
| 10056 | ENSP00000298281 | PCF11 | 1.106E-05 | -0.214 | - | - |
| 10057 | ENSP00000310208 | SLC5A6 | 1.065E-05 | -0.214 | - | - |
| 10058 | ENSP00000312250 | ADPGK | 4.814E-05 | -0.214 | - | - |
| 10059 | ENSP00000263373 | SPTBN4 | 2.267E-05 | -0.214 | - | - |
| 10060 | ENSP00000358158 | HIST2H2AA3 | 1.136E-05 | -0.214 | - | - |
| 10061 | ENSP00000288828 | WIPI2 | 1.077E-05 | -0.214 | - | - |
| 10062 | ENSP00000356105 | SLC41A1 | 2.160E-05 | -0.214 | - | - |
| 10063 | ENSP00000365686 | SLC15A1 | 3.030E-05 | -0.214 | - | - |
| 10064 | ENSP00000216840 | RABGGTA | 1.989E-05 | -0.214 | - | - |
| 10065 | ENSP00000410852 | TMEM132C | 1.031E-05 | -0.214 | - | - |
| 10066 | ENSP00000393835 | ZNF506 | 1.076E-05 | -0.214 | - | - |
| 10067 | ENSP00000254508 | NUP210 | 3.256E-05 | -0.214 | - | - |
| 10068 | ENSP00000356064 | TULP4 | 3.241E-05 | -0.214 | - | - |
| 10069 | ENSP00000333537 | SALL2 | 2.515E-05 | -0.214 | - | - |
| 10070 | ENSP00000335614 | PPFIA3 | 1.710E-05 | -0.214 | - | - |
| 10071 | ENSP00000329266 | GPIHBP1 | 2.064E-05 | -0.214 | - | - |
| 10072 | ENSP00000472469 | RPS28 | 1.740E-05 | -0.214 | - | - |
| 10073 | ENSP00000355166 | LRRC10 | 8.963E-06 | -0.214 | - | - |
| 10074 | ENSP00000374292 | CCDC57 | 1.247E-05 | -0.214 | - | - |
| 10075 | ENSP00000339844 | MRPL43 | 1.406E-05 | -0.214 | - | - |
| 10076 | ENSP00000300747 | TRIM68 | 1.732E-05 | -0.214 | - | - |
| 10077 | ENSP00000326869 | ORMDL1 | 2.570E-05 | -0.214 | - | - |
| 10078 | ENSP00000256658 | AP4B1 | 1.171E-05 | -0.214 | - | - |
| 10079 | ENSP00000359095 | PRMT6 | 1.339E-05 | -0.214 | - | - |
| 10080 | ENSP00000322242 | RNF10 | 3.198E-05 | -0.214 | - | - |
| 10081 | ENSP00000268896 | PCTP | 1.618E-05 | -0.214 | - | - |
| 10082 | ENSP00000315925 | GMPPA | 3.696E-05 | -0.214 | - | - |
| 10083 | ENSP00000265044 | SSR3 | 9.961E-06 | -0.214 | - | - |
| 10084 | ENSP00000420608 | EMC1 | 2.939E-05 | -0.214 | - | - |
| 10085 | ENSP00000317439 | CNPY1 | 2.549E-05 | -0.214 | - | - |
| 10086 | ENSP00000311245 | USMG5 | 1.258E-05 | -0.214 | - | - |
| 10087 | ENSP00000329697 | PPAP2C | 2.141E-05 | -0.214 | - | - |
| 10088 | ENSP00000274811 | RNF44 | 1.970E-05 | -0.214 | - | - |
| 10089 | ENSP00000247003 | DDX49 | 1.241E-05 | -0.214 | - | - |
| 10090 | ENSP00000262947 | C19orf10 | 5.546E-05 | -0.214 | - | - |
| 10091 | ENSP00000259870 | C6orf15 | 7.556E-06 | -0.214 | - | - |
| 10092 | ENSP00000336831 | PLS1 | 2.189E-05 | -0.214 | - | - |
| 10093 | ENSP00000328236 | KNTC1 | 4.169E-05 | -0.214 | - | - |
| 10094 | ENSP00000366273 | ZNF157 | 1.099E-05 | -0.214 | - | - |
| 10095 | ENSP00000221561 | AES | 4.085E-05 | -0.214 | - | - |
| 10096 | ENSP00000238146 | DDX55 | 1.164E-05 | -0.214 | - | - |
| 10097 | ENSP00000302728 | GUSB | 3.191E-05 | -0.214 | - | - |
| 10098 | ENSP00000381793 | GRB10 | 7.590E-05 | -0.214 | - | - |
| 10099 | ENSP00000326305 | SLC25A20 | 2.927E-05 | -0.214 | - | - |
| 10100 | ENSP00000359818 | MMS19 | 3.162E-05 | -0.214 | - | - |
| 10101 | ENSP00000219343 | SLC7A6 | 1.205E-05 | -0.214 | - | - |
| 10102 | ENSP00000289865 | USP21 | 2.165E-05 | -0.214 | - | - |
| 10103 | ENSP00000258772 | DDX56 | 1.159E-05 | -0.214 | - | - |
| 10104 | ENSP00000302830 | MAEA | 2.369E-05 | -0.214 | - | - |
| 10105 | ENSP00000390784 | B3GALT4 | 2.238E-05 | -0.214 | - | - |
| 10106 | ENSP00000324956 | RNF14 | 3.084E-05 | -0.214 | - | - |
| 10107 | ENSP00000252711 | NDUFA10 | 9.772E-06 | -0.214 | - | - |
| 10108 | ENSP00000358716 | DDX20 | 1.872E-05 | -0.214 | - | - |
| 10109 | ENSP00000242375 | AKR1D1 | 1.045E-05 | -0.214 | - | - |
| 10110 | ENSP00000339720 | NDUFA4 | 1.381E-05 | -0.214 | - | - |
| 10111 | ENSP00000312649 | PPARGC1B | 2.309E-05 | -0.214 | - | - |
| 10112 | ENSP00000295066 | DPY30 | 1.079E-05 | -0.214 | - | - |
| 10113 | ENSP00000376910 | LRRIQ1 | 8.992E-06 | -0.214 | - | - |
| 10114 | ENSP00000333380 | EGFL8 | 9.397E-06 | -0.214 | - | - |
| 10115 | ENSP00000395706 | CCDC14 | 6.427E-06 | -0.214 | - | - |
| 10116 | ENSP00000252136 | TRMT2A | 9.168E-06 | -0.214 | - | - |
| 10117 | ENSP00000296581 | LSM6 | 1.378E-05 | -0.214 | - | - |
| 10118 | ENSP00000263726 | LHX4 | 2.160E-05 | -0.214 | - | - |
| 10119 | ENSP00000225171 | DNAJC12 | 9.494E-06 | -0.214 | - | - |
| 10120 | ENSP00000342121 | RNF6 | 2.442E-05 | -0.214 | - | - |
| 10121 | ENSP00000226253 | ALDOC | 1.188E-05 | -0.214 | - | - |
| 10122 | ENSP00000263966 | USP13 | 1.647E-05 | -0.214 | - | - |
| 10123 | ENSP00000258962 | SRSF1 | 2.662E-05 | -0.214 | - | - |
| 10124 | ENSP00000350333 | SAP130 | 2.633E-05 | -0.214 | - | - |
| 10125 | ENSP00000215793 | SF3A1 | 1.211E-05 | -0.214 | - | - |
| 10126 | ENSP00000309457 | VPS41 | 1.398E-05 | -0.214 | - | - |
| 10127 | ENSP00000226319 | PHF17 | 1.938E-05 | -0.214 | - | - |
| 10128 | ENSP00000255764 | MED10 | 3.065E-05 | -0.214 | - | - |
| 10129 | ENSP00000320567 | MRPS33 | 6.194E-06 | -0.214 | - | - |
| 10130 | ENSP00000315370 | C5orf46 | 1.087E-05 | -0.214 | - | - |
| 10131 | ENSP00000276708 | GSDMC | 2.981E-05 | -0.214 | - | - |
| 10132 | ENSP00000305913 | COL8A2 | 2.793E-05 | -0.214 | - | - |
| 10133 | ENSP00000282007 | ZC3H13 | 1.081E-05 | -0.214 | - | - |
| 10134 | ENSP00000007516 | NDUFAB1 | 1.804E-05 | -0.214 | - | - |
| 10135 | ENSP00000322142 | ING5 | 1.321E-05 | -0.214 | - | - |
| 10136 | ENSP00000256854 | NARS | 8.411E-06 | -0.214 | - | - |
| 10137 | ENSP00000252543 | RPL36 | 1.041E-05 | -0.214 | - | - |
| 10138 | ENSP00000305721 | IBTK | 2.160E-05 | -0.214 | - | - |
| 10139 | ENSP00000399938 | FLYWCH1 | 6.029E-06 | -0.214 | - | - |
| 10140 | ENSP00000360966 | SLC2A6 | 2.353E-05 | -0.214 | - | - |
| 10141 | ENSP00000254231 | LIN28A | 2.287E-05 | -0.214 | - | - |
| 10142 | ENSP00000392411 | SLC13A2 | 2.133E-05 | -0.214 | - | - |
| 10143 | ENSP00000219782 | MAZ | 2.575E-05 | -0.214 | - | - |
| 10144 | ENSP00000259469 | RPL35 | 1.126E-05 | -0.214 | - | - |
| 10145 | ENSP00000008527 | CRY1 | 4.156E-05 | -0.214 | - | - |
| 10146 | ENSP00000340176 | RBPMS | 4.286E-05 | -0.214 | - | - |
| 10147 | ENSP00000221167 | - | 1.003E-05 | -0.214 | - | - |
| 10148 | ENSP00000312244 | MSL3 | 1.352E-05 | -0.214 | - | - |
| 10149 | ENSP00000294168 | TAF6L | 1.256E-05 | -0.214 | - | - |
| 10150 | ENSP00000304994 | DACH1 | 7.036E-05 | -0.214 | - | - |
| 10151 | ENSP00000366934 | NOL9 | 7.034E-06 | -0.215 | - | - |
| 10152 | ENSP00000300175 | SCG5 | 2.361E-05 | -0.215 | - | - |
| 10153 | ENSP00000318867 | CYP8B1 | 1.284E-05 | -0.215 | - | - |
| 10154 | ENSP00000357708 | S100A6 | 2.638E-05 | -0.215 | - | - |
| 10155 | ENSP00000339850 | DPYSL4 | 8.803E-06 | -0.215 | - | - |
| 10156 | ENSP00000383199 | NEDD4L | 3.518E-05 | -0.215 | - | - |
| 10157 | ENSP00000234160 | GORASP2 | 1.631E-05 | -0.215 | - | - |
| 10158 | ENSP00000318115 | TIMM50 | 1.499E-05 | -0.215 | - | - |
| 10159 | ENSP00000261517 | VPS13C | 1.155E-05 | -0.215 | - | - |
| 10160 | ENSP00000281141 | CDC123 | 2.533E-05 | -0.215 | - | - |
| 10161 | ENSP00000362592 | RBBP4 | 2.989E-05 | -0.215 | - | - |
| 10162 | ENSP00000264663 | NNT | 1.353E-05 | -0.215 | - | - |
| 10163 | ENSP00000260956 | SSB | 3.271E-05 | -0.215 | - | - |
| 10164 | ENSP00000338572 | ZNF212 | 1.053E-05 | -0.215 | - | - |
| 10165 | ENSP00000406885 | TMEM41A | 1.554E-05 | -0.215 | - | - |
| 10166 | ENSP00000381304 | ZFP90 | 1.582E-05 | -0.215 | - | - |
| 10167 | ENSP00000309710 | SLC39A1 | 1.839E-05 | -0.215 | - | - |
| 10168 | ENSP00000292314 | CCDC12 | 9.265E-06 | -0.215 | - | - |
| 10169 | ENSP00000309836 | CCDC13 | 5.154E-06 | -0.215 | - | - |
| 10170 | ENSP00000381590 | ATXN7 | 3.906E-05 | -0.215 | - | - |
| 10171 | ENSP00000360560 | TCTE1 | 1.464E-05 | -0.215 | - | - |
| 10172 | ENSP00000338387 | HLCS | 2.923E-05 | -0.215 | - | - |
| 10173 | ENSP00000275603 | CCT6A | 2.036E-05 | -0.215 | - | - |
| 10174 | ENSP00000438590 | RHNO1 | 4.559E-06 | -0.215 | - | - |
| 10175 | ENSP00000350896 | EPHB4 | 3.076E-05 | -0.215 | - | - |
| 10176 | ENSP00000284509 | ATP8B4 | 1.816E-05 | -0.215 | - | - |
| 10177 | ENSP00000377873 | ZNF358 | 1.252E-05 | -0.215 | - | - |
| 10178 | ENSP00000348673 | ZNF770 | 1.882E-05 | -0.215 | - | - |
| 10179 | ENSP00000340009 | ALG1L | 1.271E-05 | -0.215 | - | - |
| 10180 | ENSP00000313258 | ZNF541 | 1.550E-05 | -0.215 | - | - |
| 10181 | ENSP00000345555 | ENO4 | 2.184E-05 | -0.215 | - | - |
| 10182 | ENSP00000348349 | MYO9A | 1.934E-05 | -0.215 | - | - |
| 10183 | ENSP00000225567 | GOSR2 | 9.662E-06 | -0.215 | - | - |
| 10184 | ENSP00000334840 | PNRC2 | 1.964E-05 | -0.215 | - | - |
| 10185 | ENSP00000264903 | CNOT6L | 4.438E-05 | -0.215 | - | - |
| 10186 | ENSP00000344411 | TAS1R3 | 1.482E-05 | -0.215 | - | - |
| 10187 | ENSP00000222990 | SNX8 | 9.812E-06 | -0.215 | - | - |
| 10188 | ENSP00000316772 | HMHA1 | 1.556E-05 | -0.215 | - | - |
| 10189 | ENSP00000303710 | C10orf68 | 9.182E-06 | -0.215 | - | - |
| 10190 | ENSP00000388942 | SCRN1 | 2.734E-05 | -0.215 | - | - |
| 10191 | ENSP00000268129 | ABHD2 | 1.015E-05 | -0.215 | - | - |
| 10192 | ENSP00000265838 | ACAT1 | 1.602E-05 | -0.215 | - | - |
| 10193 | ENSP00000309830 | RPL38 | 1.549E-05 | -0.215 | - | - |
| 10194 | ENSP00000240304 | LUC7L3 | 1.072E-05 | -0.215 | - | - |
| 10195 | ENSP00000268933 | EPN3 | 9.194E-06 | -0.215 | - | - |
| 10196 | ENSP00000330721 | HDDC3 | 1.322E-05 | -0.215 | - | - |
| 10197 | ENSP00000332887 | UQCR10 | 1.412E-05 | -0.215 | - | - |
| 10198 | ENSP00000305919 | HTRA4 | 1.902E-05 | -0.215 | - | - |
| 10199 | ENSP00000347906 | PRMT2 | 1.115E-05 | -0.215 | - | - |
| 10200 | ENSP00000442308 | NKRF | 3.103E-05 | -0.215 | - | - |
| 10201 | ENSP00000373783 | LOXL2 | 1.909E-05 | -0.215 | - | - |
| 10202 | ENSP00000349017 | ABCC11 | 1.336E-05 | -0.215 | - | - |
| 10203 | ENSP00000374265 | GPX2 | 2.935E-05 | -0.215 | - | - |
| 10204 | ENSP00000269576 | KRT10 | 5.269E-05 | -0.215 | - | - |
| 10205 | ENSP00000313699 | HEPHL1 | 1.828E-05 | -0.215 | - | - |
| 10206 | ENSP00000457299 | PSMA1 | 3.722E-05 | -0.215 | - | - |
| 10207 | ENSP00000362931 | SRPK1 | 1.455E-03 | -0.215 | - | - |
| 10208 | ENSP00000273047 | RAB5A | 3.100E-05 | -0.215 | - | - |
| 10209 | ENSP00000324870 | SPINK6 | 2.420E-05 | -0.215 | - | - |
| 10210 | ENSP00000349147 | CNNM1 | 1.286E-05 | -0.215 | - | - |
| 10211 | ENSP00000263038 | PHYH | 5.066E-05 | -0.215 | - | - |
| 10212 | ENSP00000347754 | HTR3A | 2.314E-05 | -0.215 | - | - |
| 10213 | ENSP00000359675 | GNG5 | 2.643E-05 | -0.215 | - | - |
| 10214 | ENSP00000372410 | RNASE13 | 3.950E-07 | -0.215 | - | - |
| 10215 | ENSP00000299218 | - | 1.652E-05 | -0.215 | - | - |
| 10216 | ENSP00000400223 | FJX1 | 2.031E-05 | -0.215 | - | - |
| 10217 | ENSP00000386284 | ALAD | 2.242E-05 | -0.215 | - | - |
| 10218 | ENSP00000277517 | IDI2 | 2.846E-05 | -0.215 | - | - |
| 10219 | ENSP00000287322 | BAG4 | 8.379E-05 | -0.215 | - | - |
| 10220 | ENSP00000344055 | AP3D1 | 1.379E-05 | -0.215 | - | - |
| 10221 | ENSP00000321636 | TOP3A | 2.309E-05 | -0.215 | - | - |
| 10222 | ENSP00000261326 | MOCOS | 9.818E-06 | -0.215 | - | - |
| 10223 | ENSP00000322270 | LPHN2 | 2.291E-05 | -0.215 | - | - |
| 10224 | ENSP00000354794 | ZNF521 | 2.331E-05 | -0.215 | - | - |
| 10225 | ENSP00000339095 | RPS7 | 9.794E-06 | -0.215 | - | - |
| 10226 | ENSP00000251775 | SNX4 | 1.023E-05 | -0.215 | - | - |
| 10227 | ENSP00000306772 | LDB2 | 1.756E-05 | -0.215 | - | - |
| 10228 | ENSP00000262027 | MARS | 1.823E-05 | -0.215 | - | - |
| 10229 | ENSP00000471569 | - | 1.622E-05 | -0.215 | - | - |
| 10230 | ENSP00000323300 | SPAG5 | 1.134E-05 | -0.215 | - | - |
| 10231 | ENSP00000302867 | OR7G3 | 4.556E-06 | -0.215 | - | - |
| 10232 | ENSP00000323867 | PRKAG1 | 3.111E-05 | -0.215 | - | - |
| 10233 | ENSP00000262866 | SLA2 | 8.084E-05 | -0.215 | - | - |
| 10234 | ENSP00000224356 | CYP26A1 | 2.552E-05 | -0.215 | - | - |
| 10235 | ENSP00000335321 | SF3B1 | 1.629E-05 | -0.215 | - | - |
| 10236 | ENSP00000256649 | TRIM45 | 1.900E-04 | -0.215 | - | - |
| 10237 | ENSP00000283882 | CFDP1 | 1.679E-05 | -0.215 | - | - |
| 10238 | ENSP00000320337 | PLA2G16 | 2.295E-05 | -0.215 | - | - |
| 10239 | ENSP00000220592 | AGO2 | 2.130E-05 | -0.215 | - | - |
| 10240 | ENSP00000307607 | SDR16C5 | 1.032E-05 | -0.216 | - | - |
| 10241 | ENSP00000233055 | WDFY1 | 1.550E-05 | -0.216 | - | - |
| 10242 | ENSP00000262056 | EIF4B | 3.677E-05 | -0.216 | - | - |
| 10243 | ENSP00000229824 | RAB44 | 9.024E-06 | -0.216 | - | - |
| 10244 | ENSP00000457704 | ZNF10 | 1.188E-05 | -0.216 | - | - |
| 10245 | ENSP00000322450 | NDUFV1 | 1.195E-05 | -0.216 | - | - |
| 10246 | ENSP00000331210 | BCAN | 2.207E-05 | -0.216 | - | - |
| 10247 | ENSP00000373487 | KRT18 | 2.140E-05 | -0.216 | - | - |
| 10248 | ENSP00000358309 | EPHA7 | 3.021E-05 | -0.216 | - | - |
| 10249 | ENSP00000352920 | ZMYM1 | 1.874E-05 | -0.216 | - | - |
| 10250 | ENSP00000261441 | RSBN1 | 8.535E-05 | -0.216 | - | - |
| 10251 | ENSP00000257245 | TIMM10 | 7.962E-06 | -0.216 | - | - |
| 10252 | ENSP00000336868 | CENPA | 2.302E-05 | -0.216 | - | - |
| 10253 | ENSP00000294785 | NCSTN | 4.453E-05 | -0.216 | - | - |
| 10254 | ENSP00000369927 | AKR1C3 | 1.924E-05 | -0.216 | - | - |
| 10255 | ENSP00000383402 | PPP4R1 | 1.501E-05 | -0.216 | - | - |
| 10256 | ENSP00000377582 | KRTAP4-12 | 3.658E-06 | -0.216 | - | - |
| 10257 | ENSP00000320797 | TUBD1 | 2.163E-05 | -0.216 | - | - |
| 10258 | ENSP00000260324 | SQRDL | 1.063E-05 | -0.216 | - | - |
| 10259 | ENSP00000284694 | C10orf90 | 2.962E-05 | -0.216 | - | - |
| 10260 | ENSP00000350856 | C8orf86 | 3.304E-05 | -0.216 | - | - |
| 10261 | ENSP00000348215 | SH3PXD2A | 2.140E-05 | -0.216 | - | - |
| 10262 | ENSP00000243563 | SNRPA | 2.991E-05 | -0.216 | - | - |
| 10263 | ENSP00000343212 | MAP3K7CL | 2.112E-05 | -0.216 | - | - |
| 10264 | ENSP00000226444 | SULT1E1 | 1.845E-05 | -0.216 | - | - |
| 10265 | ENSP00000339109 | ANAPC1 | 1.268E-05 | -0.216 | - | - |
| 10266 | ENSP00000343412 | BRE | 2.000E-05 | -0.216 | - | - |
| 10267 | ENSP00000316357 | USP9X | 2.319E-05 | -0.216 | - | - |
| 10268 | ENSP00000379098 | CAMK2B | 3.686E-05 | -0.216 | - | - |
| 10269 | ENSP00000253788 | RPL27 | 8.594E-06 | -0.216 | - | - |
| 10270 | ENSP00000320604 | FAXDC2 | 1.366E-05 | -0.216 | - | - |
| 10271 | ENSP00000330658 | PAPPA | 7.048E-05 | -0.216 | - | - |
| 10272 | ENSP00000340523 | NMNAT3 | 2.660E-05 | -0.216 | - | - |
| 10273 | ENSP00000339566 | HIST1H1C | 2.007E-05 | -0.216 | - | - |
| 10274 | ENSP00000026218 | PIGQ | 1.430E-05 | -0.216 | - | - |
| 10275 | ENSP00000287667 | NOMO1 | 2.021E-05 | -0.216 | - | - |
| 10276 | ENSP00000311677 | PPP1R8 | 2.900E-05 | -0.216 | - | - |
| 10277 | ENSP00000264538 | IFT57 | 4.097E-05 | -0.216 | - | - |
| 10278 | ENSP00000314736 | HYDIN | 1.441E-05 | -0.216 | - | - |
| 10279 | ENSP00000361290 | COL4A6 | 2.820E-05 | -0.216 | - | - |
| 10280 | ENSP00000274000 | AP1AR | 2.212E-04 | -0.216 | - | - |
| 10281 | ENSP00000265881 | REXO2 | 1.312E-05 | -0.216 | - | - |
| 10282 | ENSP00000421848 | TNFAIP8 | 1.206E-05 | -0.216 | - | - |
| 10283 | ENSP00000329380 | GP1BA | 2.981E-05 | -0.216 | - | - |
| 10284 | ENSP00000279068 | LSM14B | 1.210E-05 | -0.216 | - | - |
| 10285 | ENSP00000254835 | ANKRD20A3 | 1.231E-05 | -0.216 | - | - |
| 10286 | ENSP00000268182 | IQGAP1 | 2.658E-05 | -0.216 | - | - |
| 10287 | ENSP00000258455 | MRPS9 | 1.970E-05 | -0.216 | - | - |
| 10288 | ENSP00000294008 | SLX4 | 1.300E-05 | -0.216 | - | - |
| 10289 | ENSP00000403936 | - | 3.423E-05 | -0.216 | - | - |
| 10290 | ENSP00000009041 | STARD3NL | 2.877E-04 | -0.216 | - | - |
| 10291 | ENSP00000366875 | SPATA31A1 | 1.188E-06 | -0.216 | - | - |
| 10292 | ENSP00000210313 | PSMD5 | 6.709E-05 | -0.216 | - | - |
| 10293 | ENSP00000296490 | WDR82 | 9.858E-06 | -0.216 | - | - |
| 10294 | ENSP00000306080 | RNMTL1 | 1.010E-05 | -0.216 | - | - |
| 10295 | ENSP00000337724 | ZNF202 | 2.689E-05 | -0.216 | - | - |
| 10296 | ENSP00000325819 | NR2F1 | 4.199E-05 | -0.216 | - | - |
| 10297 | ENSP00000331907 | DHX34 | 1.565E-05 | -0.216 | - | - |
| 10298 | ENSP00000262061 | COPZ1 | 1.047E-05 | -0.216 | - | - |
| 10299 | ENSP00000297578 | SLC25A32 | 7.538E-05 | -0.216 | - | - |
| 10300 | ENSP00000350630 | TLL2 | 2.044E-05 | -0.216 | - | - |
| 10301 | ENSP00000284951 | SEL1L2 | 1.739E-05 | -0.216 | - | - |
| 10302 | ENSP00000339299 | TRIO | 2.171E-05 | -0.216 | - | - |
| 10303 | ENSP00000352821 | ZDHHC14 | 2.346E-05 | -0.216 | - | - |
| 10304 | ENSP00000363965 | ALPL | 2.559E-05 | -0.216 | - | - |
| 10305 | ENSP00000243045 | ORMDL2 | 4.834E-05 | -0.216 | - | - |
| 10306 | ENSP00000341940 | CAV3 | 2.263E-05 | -0.216 | - | - |
| 10307 | ENSP00000253925 | PPFIA1 | 1.007E-05 | -0.216 | - | - |
| 10308 | ENSP00000348827 | THRB | 3.550E-05 | -0.216 | - | - |
| 10309 | ENSP00000320917 | KRI1 | 6.814E-06 | -0.216 | - | - |
| 10310 | ENSP00000288937 | MRPL17 | 8.813E-06 | -0.216 | - | - |
| 10311 | ENSP00000237853 | ELL2 | 1.901E-05 | -0.216 | - | - |
| 10312 | ENSP00000333329 | USP17L2 | 1.577E-05 | -0.216 | - | - |
| 10313 | ENSP00000445175 | LDHA | 3.495E-05 | -0.216 | - | - |
| 10314 | ENSP00000299824 | PPP1R16B | 1.560E-05 | -0.216 | - | - |
| 10315 | ENSP00000305494 | SPATA5L1 | 1.489E-05 | -0.216 | - | - |
| 10316 | ENSP00000268042 | ARRDC4 | 1.930E-05 | -0.216 | - | - |
| 10317 | ENSP00000217515 | TXNL1 | 1.361E-05 | -0.216 | - | - |
| 10318 | ENSP00000223073 | RBM28 | 8.020E-06 | -0.216 | - | - |
| 10319 | ENSP00000313953 | TTC27 | 3.501E-05 | -0.216 | - | - |
| 10320 | ENSP00000372290 | FAM193A | 1.369E-05 | -0.216 | - | - |
| 10321 | ENSP00000297534 | C7orf55 | 6.791E-05 | -0.216 | - | - |
| 10322 | ENSP00000340736 | GYG1 | 1.835E-05 | -0.216 | - | - |
| 10323 | ENSP00000308000 | TUT1 | 2.305E-05 | -0.216 | - | - |
| 10324 | ENSP00000365439 | HNRNPK | 2.453E-05 | -0.216 | - | - |
| 10325 | ENSP00000309640 | XXYLT1 | 3.388E-04 | -0.216 | - | - |
| 10326 | ENSP00000222214 | GCDH | 9.410E-06 | -0.216 | - | - |
| 10327 | ENSP00000351113 | FRYL | 3.540E-05 | -0.216 | - | - |
| 10328 | ENSP00000296084 | RYK | 7.466E-05 | -0.216 | - | - |
| 10329 | ENSP00000277575 | USP6NL | 1.391E-05 | -0.217 | - | - |
| 10330 | ENSP00000302160 | LSM3 | 1.132E-05 | -0.217 | - | - |
| 10331 | ENSP00000262209 | TRPA1 | 1.243E-05 | -0.217 | - | - |
| 10332 | ENSP00000332152 | TRIM52 | 2.585E-05 | -0.217 | - | - |
| 10333 | ENSP00000334800 | ZSCAN25 | 1.161E-05 | -0.217 | - | - |
| 10334 | ENSP00000260102 | MRPL15 | 8.949E-06 | -0.217 | - | - |
| 10335 | ENSP00000280871 | SLC2A13 | 3.022E-05 | -0.217 | - | - |
| 10336 | ENSP00000324560 | ULK1 | 4.870E-05 | -0.217 | - | - |
| 10337 | ENSP00000261875 | PTPLAD1 | 1.478E-05 | -0.217 | - | - |
| 10338 | ENSP00000316203 | SEC14L2 | 7.458E-06 | -0.217 | - | - |
| 10339 | ENSP00000222584 | SP4 | 2.450E-05 | -0.217 | - | - |
| 10340 | ENSP00000412267 | CXXC4 | 1.349E-05 | -0.217 | - | - |
| 10341 | ENSP00000368140 | CRLS1 | 2.262E-05 | -0.217 | - | - |
| 10342 | ENSP00000355026 | ARHGEF15 | 2.631E-05 | -0.217 | - | - |
| 10343 | ENSP00000303585 | CLIP1 | 1.710E-05 | -0.217 | - | - |
| 10344 | ENSP00000246194 | RALY | 1.246E-05 | -0.217 | - | - |
| 10345 | ENSP00000328808 | - | 1.456E-05 | -0.217 | - | - |
| 10346 | ENSP00000417354 | GOLIM4 | 7.852E-06 | -0.217 | - | - |
| 10347 | ENSP00000302478 | POLR1D | 1.832E-05 | -0.217 | - | - |
| 10348 | ENSP00000238714 | PAPOLG | 1.562E-05 | -0.217 | - | - |
| 10349 | ENSP00000202773 | RPL6 | 8.521E-06 | -0.217 | - | - |
| 10350 | ENSP00000346046 | RPS17L | 2.103E-05 | -0.217 | - | - |
| 10351 | ENSP00000377080 | ZFP1 | 1.222E-05 | -0.217 | - | - |
| 10352 | ENSP00000274255 | SKP2 | 3.545E-05 | -0.217 | - | - |
| 10353 | ENSP00000372136 | DEFA1B | 6.536E-06 | -0.217 | - | - |
| 10354 | ENSP00000273859 | ATP10D | 1.029E-05 | -0.217 | - | - |
| 10355 | ENSP00000004531 | SLC7A2 | 8.830E-06 | -0.217 | - | - |
| 10356 | ENSP00000199814 | RBM22 | 1.261E-05 | -0.217 | - | - |
| 10357 | ENSP00000299443 | POTED | 1.270E-05 | -0.217 | - | - |
| 10358 | ENSP00000262551 | OGN | 1.823E-05 | -0.217 | - | - |
| 10359 | ENSP00000453854 | SECISBP2L | 7.652E-06 | -0.217 | - | - |
| 10360 | ENSP00000299328 | TAZ | 2.040E-05 | -0.217 | - | - |
| 10361 | ENSP00000428281 | LEPROTL1 | 9.598E-06 | -0.217 | - | - |
| 10362 | ENSP00000337623 | RNASEH2B | 1.665E-05 | -0.217 | - | - |
| 10363 | ENSP00000265140 | ANKRD32 | 1.365E-05 | -0.217 | - | - |
| 10364 | ENSP00000421655 | TCOF1 | 2.380E-05 | -0.217 | - | - |
| 10365 | ENSP00000370083 | SMN1 | 4.294E-05 | -0.217 | - | - |
| 10366 | ENSP00000353652 | ZNF528 | 1.630E-05 | -0.217 | - | - |
| 10367 | ENSP00000275730 | SLC12A9 | 1.043E-05 | -0.217 | - | - |
| 10368 | ENSP00000296525 | ASB5 | 1.382E-05 | -0.217 | - | - |
| 10369 | ENSP00000407925 | RIMBP3B | 2.762E-06 | -0.217 | - | - |
| 10370 | ENSP00000359668 | VGLL1 | 1.808E-05 | -0.217 | - | - |
| 10371 | ENSP00000254181 | USP29 | 1.880E-05 | -0.217 | - | - |
| 10372 | ENSP00000436604 | GALNT4 | 1.095E-05 | -0.217 | - | - |
| 10373 | ENSP00000310623 | ISCU | 1.677E-05 | -0.217 | - | - |
| 10374 | ENSP00000342385 | PTGES | 9.136E-05 | -0.217 | - | - |
| 10375 | ENSP00000420487 | ABHD14A-ACY1 | 2.332E-05 | -0.217 | - | - |
| 10376 | ENSP00000329448 | NUTM1 | 3.045E-05 | -0.217 | - | - |
| 10377 | ENSP00000247178 | ATG14 | 5.171E-05 | -0.217 | - | - |
| 10378 | ENSP00000415998 | SLC48A1 | 2.996E-05 | -0.217 | - | - |
| 10379 | ENSP00000323288 | RPUSD2 | 9.072E-06 | -0.217 | - | - |
| 10380 | ENSP00000307900 | GLUL | 2.319E-05 | -0.217 | - | - |
| 10381 | ENSP00000307853 | MUS81 | 1.898E-05 | -0.217 | - | - |
| 10382 | ENSP00000354032 | BSCL2 | 1.767E-05 | -0.217 | - | - |
| 10383 | ENSP00000289952 | SLC39A14 | 1.013E-04 | -0.217 | - | - |
| 10384 | ENSP00000298229 | INPPL1 | 2.829E-05 | -0.217 | - | - |
| 10385 | ENSP00000340610 | POTEH | 1.066E-05 | -0.217 | - | - |
| 10386 | ENSP00000221307 | CYP4F3 | 2.322E-05 | -0.217 | - | - |
| 10387 | ENSP00000339834 | SNRNP48 | 2.023E-04 | -0.217 | - | - |
| 10388 | ENSP00000266376 | CACNA1C | 1.643E-05 | -0.217 | - | - |
| 10389 | ENSP00000263390 | MED26 | 1.961E-05 | -0.217 | - | - |
| 10390 | ENSP00000265849 | PMS2 | 1.567E-05 | -0.217 | - | - |
| 10391 | ENSP00000258424 | COX5B | 9.376E-06 | -0.217 | - | - |
| 10392 | ENSP00000007264 | RPUSD1 | 1.044E-05 | -0.217 | - | - |
| 10393 | ENSP00000308117 | CD248 | 2.464E-05 | -0.217 | - | - |
| 10394 | ENSP00000269214 | ESCO1 | 1.767E-05 | -0.217 | - | - |
| 10395 | ENSP00000341082 | MRPL20 | 5.661E-06 | -0.217 | - | - |
| 10396 | ENSP00000386846 | AC106876.2 | 6.397E-06 | -0.217 | - | - |
| 10397 | ENSP00000264005 | LCAT | 1.874E-05 | -0.217 | - | - |
| 10398 | ENSP00000412788 | PTBP2 | 2.196E-05 | -0.217 | - | - |
| 10399 | ENSP00000310040 | EIF3F | 1.682E-05 | -0.217 | - | - |
| 10400 | ENSP00000373565 | FAM83H | 2.511E-05 | -0.217 | - | - |
| 10401 | ENSP00000356903 | UAP1 | 1.992E-05 | -0.217 | - | - |
| 10402 | ENSP00000276079 | NONO | 4.968E-05 | -0.217 | - | - |
| 10403 | ENSP00000235090 | WDR77 | 1.189E-05 | -0.217 | - | - |
| 10404 | ENSP00000419952 | KCNAB1 | 1.319E-05 | -0.218 | - | - |
| 10405 | ENSP00000355187 | LRRTM3 | 9.269E-06 | -0.218 | - | - |
| 10406 | ENSP00000307674 | CANT1 | 2.554E-05 | -0.218 | - | - |
| 10407 | ENSP00000345029 | FAM47A | 8.854E-06 | -0.218 | - | - |
| 10408 | ENSP00000283357 | FAM81B | 3.138E-05 | -0.218 | - | - |
| 10409 | ENSP00000317985 | ROCK2 | 4.878E-05 | -0.218 | - | - |
| 10410 | ENSP00000295095 | ARHGAP15 | 1.169E-05 | -0.218 | - | - |
| 10411 | ENSP00000318966 | PINX1 | 8.742E-06 | -0.218 | - | - |
| 10412 | ENSP00000343885 | PPA2 | 2.134E-05 | -0.218 | - | - |
| 10413 | ENSP00000346921 | AK2 | 2.526E-05 | -0.218 | - | - |
| 10414 | ENSP00000336543 | SNURF | 9.895E-06 | -0.218 | - | - |
| 10415 | ENSP00000222247 | RPL18A | 1.125E-05 | -0.218 | - | - |
| 10416 | ENSP00000362390 | TBX22 | 2.071E-05 | -0.218 | - | - |
| 10417 | ENSP00000332643 | NDN | 2.221E-05 | -0.218 | - | - |
| 10418 | ENSP00000064571 | CBLN4 | 1.364E-05 | -0.218 | - | - |
| 10419 | ENSP00000311962 | GGA2 | 1.545E-05 | -0.218 | - | - |
| 10420 | ENSP00000343765 | TRIM60 | 1.578E-05 | -0.218 | - | - |
| 10421 | ENSP00000297785 | ALDH1A1 | 1.866E-05 | -0.218 | - | - |
| 10422 | ENSP00000264954 | GRPEL1 | 1.711E-05 | -0.218 | - | - |
| 10423 | ENSP00000173898 | TRO | 3.663E-05 | -0.218 | - | - |
| 10424 | ENSP00000304713 | MSANTD4 | 2.326E-05 | -0.218 | - | - |
| 10425 | ENSP00000323853 | OR8K5 | 4.700E-06 | -0.218 | - | - |
| 10426 | ENSP00000350785 | DAPK1 | 2.746E-05 | -0.218 | - | - |
| 10427 | ENSP00000312709 | TAF7 | 1.144E-05 | -0.218 | - | - |
| 10428 | ENSP00000324804 | PPP2R1A | 4.761E-05 | -0.218 | - | - |
| 10429 | ENSP00000291565 | PDXK | 5.670E-05 | -0.218 | - | - |
| 10430 | ENSP00000305221 | UGT2B4 | 1.814E-05 | -0.218 | - | - |
| 10431 | ENSP00000265138 | ARRDC3 | 1.841E-05 | -0.218 | - | - |
| 10432 | ENSP00000302111 | MGMT | 3.276E-05 | -0.218 | - | - |
| 10433 | ENSP00000252011 | DHX35 | 1.124E-05 | -0.218 | - | - |
| 10434 | ENSP00000256496 | ARL8B | 1.004E-04 | -0.218 | - | - |
| 10435 | ENSP00000328858 | PYCR1 | 4.003E-05 | -0.218 | - | - |
| 10436 | ENSP00000362975 | FAM13C | 1.584E-05 | -0.218 | - | - |
| 10437 | ENSP00000314441 | METTL1 | 3.544E-05 | -0.218 | - | - |
| 10438 | ENSP00000350297 | ASAP1 | 1.993E-05 | -0.218 | - | - |
| 10439 | ENSP00000262450 | CHD5 | 1.127E-05 | -0.218 | - | - |
| 10440 | ENSP00000344871 | MYO1F | 1.630E-05 | -0.218 | - | - |
| 10441 | ENSP00000292475 | ATP5J2 | 7.994E-06 | -0.218 | - | - |
| 10442 | ENSP00000365687 | PTF1A | 2.357E-05 | -0.218 | - | - |
| 10443 | ENSP00000287038 | RPL30 | 1.023E-05 | -0.218 | - | - |
| 10444 | ENSP00000340299 | ZDHHC1 | 6.780E-05 | -0.218 | - | - |
| 10445 | ENSP00000361186 | TP53RK | 4.356E-05 | -0.218 | - | - |
| 10446 | ENSP00000409279 | OTUD4 | 6.917E-06 | -0.218 | - | - |
| 10447 | ENSP00000216392 | PYGL | 1.823E-05 | -0.218 | - | - |
| 10448 | ENSP00000387928 | MAGIX | 1.824E-05 | -0.218 | - | - |
| 10449 | ENSP00000326581 | PEG3 | 3.059E-05 | -0.218 | - | - |
| 10450 | ENSP00000316609 | SWI5 | 2.733E-05 | -0.218 | - | - |
| 10451 | ENSP00000280098 | SPOPL | 2.700E-05 | -0.218 | - | - |
| 10452 | ENSP00000366999 | HIST1H3D | 2.384E-05 | -0.218 | - | - |
| 10453 | ENSP00000332716 | CCDC28A | 2.730E-04 | -0.218 | - | - |
| 10454 | ENSP00000295463 | ALPI | 2.939E-05 | -0.218 | - | - |
| 10455 | ENSP00000256398 | ELP3 | 1.089E-05 | -0.218 | - | - |
| 10456 | ENSP00000305648 | RAB40A | 8.416E-06 | -0.218 | - | - |
| 10457 | ENSP00000322419 | RPLP2 | 1.775E-05 | -0.218 | - | - |
| 10458 | ENSP00000267199 | VPS33A | 9.596E-06 | -0.218 | - | - |
| 10459 | ENSP00000392909 | FLJ00104 | 2.885E-06 | -0.218 | - | - |
| 10460 | ENSP00000311489 | SPTBN2 | 2.892E-05 | -0.218 | - | - |
| 10461 | ENSP00000296102 | MRPL33 | 1.035E-05 | -0.218 | - | - |
| 10462 | ENSP00000337915 | CYP3A4 | 2.503E-05 | -0.218 | - | - |
| 10463 | ENSP00000258761 | BZW2 | 7.739E-05 | -0.218 | - | - |
| 10464 | ENSP00000342886 | HIST1H2BJ | 2.018E-05 | -0.218 | - | - |
| 10465 | ENSP00000274938 | SCUBE3 | 2.249E-05 | -0.218 | - | - |
| 10466 | ENSP00000375726 | NOSIP | 4.971E-05 | -0.218 | - | - |
| 10467 | ENSP00000263672 | SPCS2 | 1.083E-05 | -0.218 | - | - |
| 10468 | ENSP00000311273 | DNAH7 | 7.424E-06 | -0.218 | - | - |
| 10469 | ENSP00000352444 | ZNF33B | 1.165E-05 | -0.218 | - | - |
| 10470 | ENSP00000344431 | DNAJB13 | 1.720E-05 | -0.218 | - | - |
| 10471 | ENSP00000272995 | COPS7B | 9.330E-05 | -0.218 | - | - |
| 10472 | ENSP00000256433 | IER3IP1 | 8.241E-05 | -0.218 | - | - |
| 10473 | ENSP00000304051 | RNF139 | 3.404E-05 | -0.218 | - | - |
| 10474 | ENSP00000317159 | CYC1 | 9.264E-06 | -0.218 | - | - |
| 10475 | ENSP00000340900 | MIA3 | 1.238E-05 | -0.218 | - | - |
| 10476 | ENSP00000301945 | ANTXR1 | 1.106E-04 | -0.218 | - | - |
| 10477 | ENSP00000305785 | DMRT2 | 1.981E-05 | -0.219 | - | - |
| 10478 | ENSP00000274773 | TRIM7 | 1.249E-05 | -0.219 | - | - |
| 10479 | ENSP00000274813 | MUT | 2.053E-05 | -0.219 | - | - |
| 10480 | ENSP00000316628 | RNF152 | 3.764E-05 | -0.219 | - | - |
| 10481 | ENSP00000263921 | CHIC2 | 4.900E-05 | -0.219 | - | - |
| 10482 | ENSP00000257181 | PRPF38A | 4.264E-05 | -0.219 | - | - |
| 10483 | ENSP00000252102 | NDUFA2 | 1.086E-05 | -0.219 | - | - |
| 10484 | ENSP00000262971 | PIAS4 | 5.240E-05 | -0.219 | - | - |
| 10485 | ENSP00000285407 | KLF10 | 2.656E-05 | -0.219 | - | - |
| 10486 | ENSP00000290943 | ANKRD18B | 1.170E-05 | -0.219 | - | - |
| 10487 | ENSP00000323511 | PNKP | 1.854E-05 | -0.219 | - | - |
| 10488 | ENSP00000350686 | ANKLE2 | 2.912E-05 | -0.219 | - | - |
| 10489 | ENSP00000361721 | CITED4 | 1.454E-05 | -0.219 | - | - |
| 10490 | ENSP00000366863 | TBC1D4 | 3.126E-05 | -0.219 | - | - |
| 10491 | ENSP00000297990 | NOL6 | 8.731E-06 | -0.219 | - | - |
| 10492 | ENSP00000262461 | SLC12A2 | 1.781E-05 | -0.219 | - | - |
| 10493 | ENSP00000269703 | CYP4F22 | 1.953E-05 | -0.219 | - | - |
| 10494 | ENSP00000300584 | TBC1D2B | 1.205E-05 | -0.219 | - | - |
| 10495 | ENSP00000378286 | ZBTB3 | 1.546E-05 | -0.219 | - | - |
| 10496 | ENSP00000256707 | KIDINS220 | 1.280E-05 | -0.219 | - | - |
| 10497 | ENSP00000265560 | USP4 | 1.536E-05 | -0.219 | - | - |
| 10498 | ENSP00000351153 | PARPBP | 1.986E-05 | -0.219 | - | - |
| 10499 | ENSP00000329419 | COPB2 | 1.094E-05 | -0.219 | - | - |
| 10500 | ENSP00000284551 | TRIM11 | 1.265E-05 | -0.219 | - | - |
| 10501 | ENSP00000332247 | ATP6V0A2 | 3.869E-05 | -0.219 | - | - |
| 10502 | ENSP00000273359 | ABHD10 | 7.275E-05 | -0.219 | - | - |
| 10503 | ENSP00000251170 | SEC14L5 | 1.177E-05 | -0.219 | - | - |
| 10504 | ENSP00000434508 | RP11-849H4.2 | 1.466E-05 | -0.219 | - | - |
| 10505 | ENSP00000206020 | SPAG7 | 3.540E-05 | -0.219 | - | - |
| 10506 | ENSP00000261520 | NARG2 | 1.149E-04 | -0.219 | - | - |
| 10507 | ENSP00000342344 | CCNYL1 | 1.783E-05 | -0.219 | - | - |
| 10508 | ENSP00000397693 | ZNF28 | 8.743E-06 | -0.219 | - | - |
| 10509 | ENSP00000374125 | SIGLEC15 | 9.471E-06 | -0.219 | - | - |
| 10510 | ENSP00000304229 | HINT1 | 2.330E-05 | -0.219 | - | - |
| 10511 | ENSP00000378601 | C8orf31 | 6.164E-06 | -0.219 | - | - |
| 10512 | ENSP00000254803 | UTP3 | 9.195E-06 | -0.219 | - | - |
| 10513 | ENSP00000362283 | PTPRT | 2.404E-05 | -0.219 | - | - |
| 10514 | ENSP00000261296 | TGDS | 3.040E-05 | -0.219 | - | - |
| 10515 | ENSP00000265224 | ANKRD7 | 1.146E-05 | -0.219 | - | - |
| 10516 | ENSP00000225298 | UTP18 | 9.761E-06 | -0.219 | - | - |
| 10517 | ENSP00000310071 | ANAPC10 | 1.272E-05 | -0.219 | - | - |
| 10518 | ENSP00000318197 | TUBA3E | 1.559E-05 | -0.219 | - | - |
| 10519 | ENSP00000450244 | RASAL1 | 2.130E-05 | -0.219 | - | - |
| 10520 | ENSP00000276594 | PRDM14 | 2.347E-05 | -0.219 | - | - |
| 10521 | ENSP00000297792 | KDM1B | 1.556E-05 | -0.219 | - | - |
| 10522 | ENSP00000289779 | F11R | 5.286E-05 | -0.219 | - | - |
| 10523 | ENSP00000443985 | CPEB2 | 7.977E-06 | -0.219 | - | - |
| 10524 | ENSP00000316176 | UBE2N | 5.945E-05 | -0.219 | - | - |
| 10525 | ENSP00000007414 | OSBPL7 | 1.321E-05 | -0.219 | - | - |
| 10526 | ENSP00000316590 | AL445665.1 | 1.204E-05 | -0.219 | - | - |
| 10527 | ENSP00000223641 | SEC61B | 7.189E-06 | -0.219 | - | - |
| 10528 | ENSP00000361882 | ODF2 | 2.567E-05 | -0.219 | - | - |
| 10529 | ENSP00000314655 | TSC22D3 | 8.129E-05 | -0.219 | - | - |
| 10530 | ENSP00000245539 | MRPS7 | 1.125E-05 | -0.219 | - | - |
| 10531 | ENSP00000265245 | LSG1 | 9.054E-06 | -0.219 | - | - |
| 10532 | ENSP00000258341 | LAMC1 | 7.136E-05 | -0.219 | - | - |
| 10533 | ENSP00000247970 | PIN1 | 2.389E-05 | -0.219 | - | - |
| 10534 | ENSP00000260385 | RMDN3 | 1.580E-04 | -0.219 | - | - |
| 10535 | ENSP00000342626 | EYA1 | 5.372E-05 | -0.219 | - | - |
| 10536 | ENSP00000435188 | RP11-831H9.11 | 2.548E-06 | -0.219 | - | - |
| 10537 | ENSP00000260130 | SDCBP | 5.729E-05 | -0.219 | - | - |
| 10538 | ENSP00000318861 | SF3B2 | 1.354E-05 | -0.219 | - | - |
| 10539 | ENSP00000265335 | RAD50 | 2.841E-05 | -0.219 | - | - |
| 10540 | ENSP00000375093 | TAS2R31 | 2.099E-05 | -0.219 | - | - |
| 10541 | ENSP00000257934 | ESPL1 | 1.098E-05 | -0.219 | - | - |
| 10542 | ENSP00000356143 | LRRN2 | 1.170E-05 | -0.219 | - | - |
| 10543 | ENSP00000340507 | TRIM24 | 3.158E-05 | -0.219 | - | - |
| 10544 | ENSP00000348984 | SON | 2.090E-05 | -0.219 | - | - |
| 10545 | ENSP00000317473 | RABGGTB | 1.198E-05 | -0.219 | - | - |
| 10546 | ENSP00000266269 | PATZ1 | 2.355E-05 | -0.219 | - | - |
| 10547 | ENSP00000300737 | STIM1 | 4.524E-05 | -0.219 | - | - |
| 10548 | ENSP00000378260 | ALG13 | 3.094E-05 | -0.219 | - | - |
| 10549 | ENSP00000391835 | - | 2.885E-06 | -0.219 | - | - |
| 10550 | ENSP00000230099 | TRIM38 | 2.899E-04 | -0.219 | - | - |
| 10551 | ENSP00000293872 | LUC7L | 8.218E-06 | -0.219 | - | - |
| 10552 | ENSP00000276373 | SLC18A1 | 2.839E-05 | -0.219 | - | - |
| 10553 | ENSP00000262919 | ATRN | 1.928E-05 | -0.219 | - | - |
| 10554 | ENSP00000218006 | GUCY2F | 7.610E-06 | -0.220 | - | - |
| 10555 | ENSP00000356549 | IER5 | 2.299E-05 | -0.220 | - | - |
| 10556 | ENSP00000318227 | DCAF8 | 1.929E-05 | -0.220 | - | - |
| 10557 | ENSP00000341785 | ADIPOR1 | 6.575E-05 | -0.220 | - | - |
| 10558 | ENSP00000266095 | PISD | 1.078E-05 | -0.220 | - | - |
| 10559 | ENSP00000328269 | HMG20B | 1.528E-05 | -0.220 | - | - |
| 10560 | ENSP00000330836 | GLRX3 | 1.063E-05 | -0.220 | - | - |
| 10561 | ENSP00000333255 | VMA21 | 6.020E-05 | -0.220 | - | - |
| 10562 | ENSP00000338727 | LRRFIP2 | 1.571E-04 | -0.220 | - | - |
| 10563 | ENSP00000267425 | NOP9 | 9.733E-06 | -0.220 | - | - |
| 10564 | ENSP00000374260 | CCDC149 | 2.537E-06 | -0.220 | - | - |
| 10565 | ENSP00000392495 | TRAPPC2 | 1.701E-05 | -0.220 | - | - |
| 10566 | ENSP00000296802 | NSA2 | 1.074E-05 | -0.220 | - | - |
| 10567 | ENSP00000288050 | PDPR | 2.744E-05 | -0.220 | - | - |
| 10568 | ENSP00000361280 | - | 2.720E-05 | -0.220 | - | - |
| 10569 | ENSP00000320869 | TRIM41 | 1.300E-05 | -0.220 | - | - |
| 10570 | ENSP00000216200 | PVALB | 1.108E-05 | -0.220 | - | - |
| 10571 | ENSP00000196371 | OXCT1 | 1.161E-05 | -0.220 | - | - |
| 10572 | ENSP00000264230 | NOA1 | 7.902E-06 | -0.220 | - | - |
| 10573 | ENSP00000305480 | FEN1 | 1.984E-05 | -0.220 | - | - |
| 10574 | ENSP00000358224 | PPIAL4C | 2.304E-05 | -0.220 | - | - |
| 10575 | ENSP00000306003 | ATP5I | 6.452E-06 | -0.220 | - | - |
| 10576 | ENSP00000265087 | STC2 | 4.556E-05 | -0.220 | - | - |
| 10577 | ENSP00000449751 | FKBP11 | 1.327E-05 | -0.220 | - | - |
| 10578 | ENSP00000311825 | CTBP2 | 2.402E-05 | -0.220 | - | - |
| 10579 | ENSP00000284878 | CXADR | 6.649E-05 | -0.220 | - | - |
| 10580 | ENSP00000463159 | C19orf47 | 2.663E-06 | -0.220 | - | - |
| 10581 | ENSP00000325285 | MTMR4 | 5.516E-05 | -0.220 | - | - |
| 10582 | ENSP00000261366 | LMNB1 | 2.879E-05 | -0.220 | - | - |
| 10583 | ENSP00000307298 | FEM1B | 1.439E-05 | -0.220 | - | - |
| 10584 | ENSP00000248139 | RAB40C | 9.075E-06 | -0.220 | - | - |
| 10585 | ENSP00000295491 | MRPS18C | 4.981E-06 | -0.220 | - | - |
| 10586 | ENSP00000309181 | CCNE2 | 3.544E-05 | -0.220 | - | - |
| 10587 | ENSP00000384716 | ZDHHC8 | 1.719E-05 | -0.220 | - | - |
| 10588 | ENSP00000431759 | C12ORF55 | 1.708E-05 | -0.220 | - | - |
| 10589 | ENSP00000271452 | NUF2 | 1.062E-05 | -0.220 | - | - |
| 10590 | ENSP00000382513 | ZNF594 | 8.773E-06 | -0.220 | - | - |
| 10591 | ENSP00000314733 | TOLLIP | 1.298E-04 | -0.220 | - | - |
| 10592 | ENSP00000372361 | ZMYM5 | 1.095E-05 | -0.220 | - | - |
| 10593 | ENSP00000323714 | EIF4E1B | 1.797E-05 | -0.220 | - | - |
| 10594 | ENSP00000343479 | NBR1 | 1.862E-05 | -0.220 | - | - |
| 10595 | ENSP00000316779 | BIN1 | 6.296E-05 | -0.220 | - | - |
| 10596 | ENSP00000369996 | ATXN3L | 9.663E-06 | -0.220 | - | - |
| 10597 | ENSP00000252115 | POLDIP3 | 1.843E-05 | -0.220 | - | - |
| 10598 | ENSP00000322570 | POLE | 1.600E-05 | -0.220 | - | - |
| 10599 | ENSP00000267938 | UBE2Q2 | 6.766E-05 | -0.220 | - | - |
| 10600 | ENSP00000312397 | KLHL3 | 4.970E-05 | -0.220 | - | - |
| 10601 | ENSP00000295797 | PRKCI | 4.292E-05 | -0.220 | - | - |
| 10602 | ENSP00000382982 | TUBA3C | 2.873E-05 | -0.220 | - | - |
| 10603 | ENSP00000252487 | TOMM40 | 1.121E-05 | -0.220 | - | - |
| 10604 | ENSP00000276651 | DPYS | 8.835E-06 | -0.220 | - | - |
| 10605 | ENSP00000281928 | MED13L | 1.761E-05 | -0.220 | - | - |
| 10606 | ENSP00000343118 | LAMTOR4 | 2.765E-05 | -0.220 | - | - |
| 10607 | ENSP00000261835 | CYP46A1 | 2.154E-05 | -0.220 | - | - |
| 10608 | ENSP00000420211 | RGL2 | 8.359E-06 | -0.220 | - | - |
| 10609 | ENSP00000275227 | SLC18B1 | 3.106E-04 | -0.220 | - | - |
| 10610 | ENSP00000366519 | NSUN6 | 1.040E-05 | -0.220 | - | - |
| 10611 | ENSP00000302768 | PAIP1 | 9.903E-05 | -0.220 | - | - |
| 10612 | ENSP00000262291 | VMP1 | 4.235E-05 | -0.220 | - | - |
| 10613 | ENSP00000225614 | GALK1 | 1.725E-05 | -0.220 | - | - |
| 10614 | ENSP00000353720 | CES1 | 2.429E-05 | -0.220 | - | - |
| 10615 | ENSP00000352639 | CELA2A | 2.575E-05 | -0.220 | - | - |
| 10616 | ENSP00000266671 | PHLDA1 | 6.311E-05 | -0.220 | - | - |
| 10617 | ENSP00000313869 | GORASP1 | 1.434E-05 | -0.220 | - | - |
| 10618 | ENSP00000225655 | PFN1 | 2.782E-05 | -0.220 | - | - |
| 10619 | ENSP00000295317 | RNF149 | 6.906E-05 | -0.220 | - | - |
| 10620 | ENSP00000328182 | CLN8 | 2.144E-05 | -0.220 | - | - |
| 10621 | ENSP00000356234 | KDM5B | 1.863E-05 | -0.220 | - | - |
| 10622 | ENSP00000363344 | ZFP37 | 1.236E-05 | -0.220 | - | - |
| 10623 | ENSP00000252329 | PSMG3 | 7.233E-05 | -0.220 | - | - |
| 10624 | ENSP00000239891 | ALG5 | 1.964E-05 | -0.220 | - | - |
| 10625 | ENSP00000297229 | USP49 | 8.830E-06 | -0.220 | - | - |
| 10626 | ENSP00000365253 | LRRC38 | 2.109E-05 | -0.220 | - | - |
| 10627 | ENSP00000311984 | CARKD | 1.467E-05 | -0.220 | - | - |
| 10628 | ENSP00000218249 | RAB40AL | 1.109E-05 | -0.220 | - | - |
| 10629 | ENSP00000296504 | SAP30 | 1.670E-05 | -0.220 | - | - |
| 10630 | ENSP00000261537 | MIB1 | 2.125E-05 | -0.220 | - | - |
| 10631 | ENSP00000326579 | HCCS | 4.651E-05 | -0.220 | - | - |
| 10632 | ENSP00000371813 | DEFB132 | 1.296E-05 | -0.220 | - | - |
| 10633 | ENSP00000289788 | ZSCAN23 | 2.477E-05 | -0.220 | - | - |
| 10634 | ENSP00000252512 | XPO7 | 2.702E-05 | -0.220 | - | - |
| 10635 | ENSP00000226951 | CLNK | 3.384E-05 | -0.220 | - | - |
| 10636 | ENSP00000253814 | NDFIP1 | 1.501E-05 | -0.220 | - | - |
| 10637 | ENSP00000446680 | HOXC11 | 1.095E-05 | -0.221 | - | - |
| 10638 | ENSP00000304941 | TCTN2 | 1.576E-04 | -0.221 | - | - |
| 10639 | ENSP00000330671 | DNAH11 | 1.191E-05 | -0.221 | - | - |
| 10640 | ENSP00000258787 | MYO1G | 2.303E-05 | -0.221 | - | - |
| 10641 | ENSP00000373167 | TIGIT | 2.785E-05 | -0.221 | - | - |
| 10642 | ENSP00000294724 | AGL | 1.289E-05 | -0.221 | - | - |
| 10643 | ENSP00000258415 | CYP27A1 | 1.955E-05 | -0.221 | - | - |
| 10644 | ENSP00000375248 | BHLHA9 | 7.525E-06 | -0.221 | - | - |
| 10645 | ENSP00000365370 | HNRNPCL1 | 5.185E-05 | -0.221 | - | - |
| 10646 | ENSP00000431391 | API5 | 2.507E-05 | -0.221 | - | - |
| 10647 | ENSP00000351539 | ZBTB18 | 1.586E-05 | -0.221 | - | - |
| 10648 | ENSP00000323879 | ZNRF2 | 9.494E-05 | -0.221 | - | - |
| 10649 | ENSP00000356540 | STX11 | 1.931E-05 | -0.221 | - | - |
| 10650 | ENSP00000272427 | EXOC6B | 1.322E-05 | -0.221 | - | - |
| 10651 | ENSP00000312618 | ACAD9 | 1.694E-05 | -0.221 | - | - |
| 10652 | ENSP00000258729 | IGF2BP3 | 4.011E-05 | -0.221 | - | - |
| 10653 | ENSP00000254846 | KDM6B | 1.448E-05 | -0.221 | - | - |
| 10654 | ENSP00000339047 | LRRC61 | 4.214E-05 | -0.221 | - | - |
| 10655 | ENSP00000224140 | SETX | 1.980E-05 | -0.221 | - | - |
| 10656 | ENSP00000297591 | KIAA1429 | 1.078E-05 | -0.221 | - | - |
| 10657 | ENSP00000264451 | SLC30A9 | 5.884E-05 | -0.221 | - | - |
| 10658 | ENSP00000314901 | LRRN1 | 3.266E-05 | -0.221 | - | - |
| 10659 | ENSP00000320566 | ING3 | 4.156E-05 | -0.221 | - | - |
| 10660 | ENSP00000289382 | CNOT11 | 1.102E-04 | -0.221 | - | - |
| 10661 | ENSP00000229328 | PRKAB1 | 2.520E-05 | -0.221 | - | - |
| 10662 | ENSP00000202967 | SIRT4 | 3.376E-05 | -0.221 | - | - |
| 10663 | ENSP00000263382 | ASF1B | 1.023E-05 | -0.221 | - | - |
| 10664 | ENSP00000340454 | RAP1GDS1 | 1.637E-05 | -0.221 | - | - |
| 10665 | ENSP00000350049 | HOMEZ | 1.994E-05 | -0.221 | - | - |
| 10666 | ENSP00000266304 | TEF | 1.676E-05 | -0.221 | - | - |
| 10667 | ENSP00000417147 | SMIM7 | 6.852E-06 | -0.221 | - | - |
| 10668 | ENSP00000294383 | USP24 | 1.970E-05 | -0.221 | - | - |
| 10669 | ENSP00000347648 | ZNF512 | 1.050E-05 | -0.221 | - | - |
| 10670 | ENSP00000260762 | EXOC6 | 1.153E-05 | -0.221 | - | - |
| 10671 | ENSP00000375582 | ZNF552 | 1.116E-05 | -0.221 | - | - |
| 10672 | ENSP00000205214 | AASDH | 1.845E-05 | -0.221 | - | - |
| 10673 | ENSP00000354663 | ZNF438 | 1.124E-05 | -0.221 | - | - |
| 10674 | ENSP00000370543 | SLC5A3 | 2.708E-05 | -0.221 | - | - |
| 10675 | ENSP00000322542 | GTF2I | 3.131E-05 | -0.221 | - | - |
| 10676 | ENSP00000292125 | PSG6 | 3.078E-05 | -0.221 | - | - |
| 10677 | ENSP00000263212 | PPM1F | 2.796E-05 | -0.221 | - | - |
| 10678 | ENSP00000364798 | ZBTB40 | 2.898E-05 | -0.221 | - | - |
| 10679 | ENSP00000344087 | AUTS2 | 1.104E-05 | -0.221 | - | - |
| 10680 | ENSP00000294353 | ZYG11B | 5.014E-05 | -0.221 | - | - |
| 10681 | ENSP00000300176 | AGFG2 | 1.232E-05 | -0.221 | - | - |
| 10682 | ENSP00000358802 | KCNC4 | 2.108E-05 | -0.221 | - | - |
| 10683 | ENSP00000398824 | PHF21A | 3.537E-05 | -0.221 | - | - |
| 10684 | ENSP00000306822 | AVEN | 7.410E-05 | -0.221 | - | - |
| 10685 | ENSP00000362334 | PSMB2 | 2.773E-05 | -0.221 | - | - |
| 10686 | ENSP00000409514 | ZNF717 | 9.881E-06 | -0.221 | - | - |
| 10687 | ENSP00000344401 | LIN28B | 1.509E-05 | -0.221 | - | - |
| 10688 | ENSP00000415203 | PIGG | 2.766E-05 | -0.221 | - | - |
| 10689 | ENSP00000302088 | RER1 | 1.179E-05 | -0.221 | - | - |
| 10690 | ENSP00000274311 | PELO | 5.486E-05 | -0.221 | - | - |
| 10691 | ENSP00000290759 | ISL2 | 3.410E-05 | -0.221 | - | - |
| 10692 | ENSP00000224652 | ATE1 | 2.132E-05 | -0.221 | - | - |
| 10693 | ENSP00000265960 | MAPKAP1 | 4.406E-05 | -0.221 | - | - |
| 10694 | ENSP00000340427 | ERMP1 | 6.110E-05 | -0.221 | - | - |
| 10695 | ENSP00000376823 | C7orf49 | 1.941E-05 | -0.221 | - | - |
| 10696 | ENSP00000001008 | FKBP4 | 2.158E-05 | -0.221 | - | - |
| 10697 | ENSP00000249299 | NAA38 | 7.531E-06 | -0.221 | - | - |
| 10698 | ENSP00000352702 | SLC6A12 | 9.257E-06 | -0.221 | - | - |
| 10699 | ENSP00000386653 | PCDH20 | 8.216E-06 | -0.221 | - | - |
| 10700 | ENSP00000421566 | XPNPEP1 | 2.585E-05 | -0.221 | - | - |
| 10701 | ENSP00000451261 | UBE2J1 | 2.948E-05 | -0.221 | - | - |
| 10702 | ENSP00000229416 | GCLC | 5.416E-05 | -0.221 | - | - |
| 10703 | ENSP00000354919 | VEPH1 | 2.924E-05 | -0.221 | - | - |
| 10704 | ENSP00000360262 | FGGY | 2.795E-05 | -0.221 | - | - |
| 10705 | ENSP00000368684 | RASSF2 | 1.750E-05 | -0.221 | - | - |
| 10706 | ENSP00000274137 | NDUFS6 | 7.487E-06 | -0.221 | - | - |
| 10707 | ENSP00000262462 | SLC27A6 | 4.406E-05 | -0.221 | - | - |
| 10708 | ENSP00000362527 | ZNF362 | 1.662E-05 | -0.221 | - | - |
| 10709 | ENSP00000223122 | C1GALT1 | 1.297E-05 | -0.221 | - | - |
| 10710 | ENSP00000389399 | TMCO3 | 1.792E-05 | -0.221 | - | - |
| 10711 | ENSP00000374071 | ADAMTS20 | 2.169E-05 | -0.221 | - | - |
| 10712 | ENSP00000262133 | RBL2 | 2.851E-05 | -0.221 | - | - |
| 10713 | ENSP00000340596 | UBE3B | 2.226E-05 | -0.221 | - | - |
| 10714 | ENSP00000275016 | CYP39A1 | 1.736E-05 | -0.221 | - | - |
| 10715 | ENSP00000320849 | C12orf68 | 2.083E-05 | -0.221 | - | - |
| 10716 | ENSP00000342793 | PLD1 | 1.446E-05 | -0.221 | - | - |
| 10717 | ENSP00000337168 | EPB41L1 | 5.556E-05 | -0.221 | - | - |
| 10718 | ENSP00000181796 | FAM107B | 3.472E-04 | -0.221 | - | - |
| 10719 | ENSP00000239027 | HELLS | 1.049E-05 | -0.222 | - | - |
| 10720 | ENSP00000223190 | NRF1 | 6.767E-05 | -0.222 | - | - |
| 10721 | ENSP00000262457 | INVS | 1.203E-05 | -0.222 | - | - |
| 10722 | ENSP00000339208 | DPP8 | 7.039E-05 | -0.222 | - | - |
| 10723 | ENSP00000260926 | SATB2 | 3.514E-05 | -0.222 | - | - |
| 10724 | ENSP00000418748 | ESRP2 | 8.823E-06 | -0.222 | - | - |
| 10725 | ENSP00000248041 | CYP4F11 | 1.900E-05 | -0.222 | - | - |
| 10726 | ENSP00000253079 | CCDC62 | 2.966E-05 | -0.222 | - | - |
| 10727 | ENSP00000336866 | RBBP9 | 1.102E-04 | -0.222 | - | - |
| 10728 | ENSP00000321606 | CRMP1 | 1.156E-05 | -0.222 | - | - |
| 10729 | ENSP00000325817 | CYFIP2 | 2.292E-05 | -0.222 | - | - |
| 10730 | ENSP00000166345 | TRIP13 | 8.170E-06 | -0.222 | - | - |
| 10731 | ENSP00000270722 | PRDM16 | 1.895E-05 | -0.222 | - | - |
| 10732 | ENSP00000302843 | DNAJC18 | 7.990E-06 | -0.222 | - | - |
| 10733 | ENSP00000296388 | LEPRE1 | 3.906E-05 | -0.222 | - | - |
| 10734 | ENSP00000380237 | ZNF691 | 1.112E-05 | -0.222 | - | - |
| 10735 | ENSP00000376889 | NME2 | 3.922E-05 | -0.222 | - | - |
| 10736 | ENSP00000356599 | TOR3A | 1.167E-05 | -0.222 | - | - |
| 10737 | ENSP00000344683 | CDK5RAP3 | 3.343E-05 | -0.222 | - | - |
| 10738 | ENSP00000377867 | MST4 | 2.243E-05 | -0.222 | - | - |
| 10739 | ENSP00000335538 | DEFB131 | 1.447E-05 | -0.222 | - | - |
| 10740 | ENSP00000405289 | ZNF778 | 1.314E-05 | -0.222 | - | - |
| 10741 | ENSP00000013034 | NME1 | 2.174E-05 | -0.222 | - | - |
| 10742 | ENSP00000263097 | CNN2 | 3.772E-05 | -0.222 | - | - |
| 10743 | ENSP00000382000 | CDRT15L2 | 5.438E-06 | -0.222 | - | - |
| 10744 | ENSP00000300093 | PLK1 | 4.950E-05 | -0.222 | - | - |
| 10745 | ENSP00000306906 | RNF181 | 1.835E-05 | -0.222 | - | - |
| 10746 | ENSP00000320893 | ASB8 | 1.237E-05 | -0.222 | - | - |
| 10747 | ENSP00000290846 | TRMU | 8.531E-06 | -0.222 | - | - |
| 10748 | ENSP00000327124 | SMCR7L | 1.697E-05 | -0.222 | - | - |
| 10749 | ENSP00000259467 | PDCL | 7.363E-05 | -0.222 | - | - |
| 10750 | ENSP00000253794 | VPS25 | 4.503E-05 | -0.222 | - | - |
| 10751 | ENSP00000331057 | TCF12 | 3.557E-05 | -0.222 | - | - |
| 10752 | ENSP00000312442 | AP1G2 | 9.683E-06 | -0.222 | - | - |
| 10753 | ENSP00000366330 | KLF9 | 1.926E-05 | -0.222 | - | - |
| 10754 | ENSP00000364023 | ZXDB | 1.623E-05 | -0.222 | - | - |
| 10755 | ENSP00000318318 | MPI | 2.695E-05 | -0.222 | - | - |
| 10756 | ENSP00000342755 | RNF41 | 7.173E-05 | -0.222 | - | - |
| 10757 | ENSP00000376135 | ZNF605 | 9.149E-06 | -0.222 | - | - |
| 10758 | ENSP00000288139 | CACNA1D | 1.239E-05 | -0.222 | - | - |
| 10759 | ENSP00000280706 | LDHAL6A | 2.672E-05 | -0.222 | - | - |
| 10760 | ENSP00000313079 | SHISA2 | 1.820E-05 | -0.222 | - | - |
| 10761 | ENSP00000244289 | LIPE | 1.696E-05 | -0.222 | - | - |
| 10762 | ENSP00000262198 | ADNP2 | 5.294E-05 | -0.222 | - | - |
| 10763 | ENSP00000298743 | GAS1 | 5.875E-05 | -0.222 | - | - |
| 10764 | ENSP00000339399 | CRYZ | 1.175E-05 | -0.222 | - | - |
| 10765 | ENSP00000283943 | TRIP12 | 2.045E-05 | -0.222 | - | - |
| 10766 | ENSP00000370750 | RDH11 | 1.110E-05 | -0.222 | - | - |
| 10767 | ENSP00000230050 | RPS12 | 6.292E-06 | -0.222 | - | - |
| 10768 | ENSP00000264409 | AGPAT9 | 3.483E-05 | -0.222 | - | - |
| 10769 | ENSP00000334294 | TCFL5 | 2.020E-05 | -0.222 | - | - |
| 10770 | ENSP00000302501 | ZEB2 | 4.040E-05 | -0.222 | - | - |
| 10771 | ENSP00000366511 | AC025278.1 | 4.894E-06 | -0.222 | - | - |
| 10772 | ENSP00000233057 | EIF2AK2 | 8.653E-05 | -0.222 | - | - |
| 10773 | ENSP00000230640 | SKIV2L2 | 1.455E-05 | -0.222 | - | - |
| 10774 | ENSP00000333376 | USP45 | 5.109E-05 | -0.222 | - | - |
| 10775 | ENSP00000283268 | FEZF2 | 2.256E-05 | -0.222 | - | - |
| 10776 | ENSP00000276201 | UPF3B | 9.559E-06 | -0.222 | - | - |
| 10777 | ENSP00000328187 | FBXO21 | 6.249E-05 | -0.222 | - | - |
| 10778 | ENSP00000328968 | SCN5A | 1.769E-05 | -0.222 | - | - |
| 10779 | ENSP00000219252 | POLR2C | 9.576E-06 | -0.222 | - | - |
| 10780 | ENSP00000263849 | ZC2HC1A | 1.720E-04 | -0.222 | - | - |
| 10781 | ENSP00000253577 | ABCB7 | 1.765E-05 | -0.222 | - | - |
| 10782 | ENSP00000265909 | SNX19 | 9.093E-05 | -0.222 | - | - |
| 10783 | ENSP00000340138 | DLEU2L | 1.453E-05 | -0.222 | - | - |
| 10784 | ENSP00000220325 | EHD4 | 3.599E-05 | -0.222 | - | - |
| 10785 | ENSP00000307491 | WDR48 | 1.744E-05 | -0.222 | - | - |
| 10786 | ENSP00000332791 | KLHL15 | 2.298E-05 | -0.222 | - | - |
| 10787 | ENSP00000319914 | C8orf4 | 5.687E-05 | -0.222 | - | - |
| 10788 | ENSP00000229395 | FGFR1OP2 | 5.976E-05 | -0.222 | - | - |
| 10789 | ENSP00000217244 | CSNK2A1 | 1.642E-05 | -0.222 | - | - |
| 10790 | ENSP00000274217 | FAM105A | 1.833E-04 | -0.222 | - | - |
| 10791 | ENSP00000444350 | LHFPL3 | 2.743E-05 | -0.222 | - | - |
| 10792 | ENSP00000295770 | STT3B | 2.547E-05 | -0.222 | - | - |
| 10793 | ENSP00000285199 | USP43 | 1.990E-05 | -0.222 | - | - |
| 10794 | ENSP00000303279 | IARS2 | 2.442E-05 | -0.222 | - | - |
| 10795 | ENSP00000222250 | ARRDC2 | 1.203E-05 | -0.222 | - | - |
| 10796 | ENSP00000417424 | ZNF480 | 1.406E-05 | -0.222 | - | - |
| 10797 | ENSP00000216237 | L3MBTL2 | 2.309E-05 | -0.222 | - | - |
| 10798 | ENSP00000238892 | CRIPT | 4.682E-05 | -0.222 | - | - |
| 10799 | ENSP00000258198 | DYNC1LI2 | 7.161E-05 | -0.222 | - | - |
| 10800 | ENSP00000299518 | IDH3A | 1.046E-05 | -0.222 | - | - |
| 10801 | ENSP00000358739 | RWDD2A | 5.162E-06 | -0.222 | - | - |
| 10802 | ENSP00000264775 | PPAP2A | 1.971E-05 | -0.222 | - | - |
| 10803 | ENSP00000257287 | CEP135 | 1.799E-05 | -0.222 | - | - |
| 10804 | ENSP00000261497 | USP22 | 8.334E-06 | -0.223 | - | - |
| 10805 | ENSP00000264607 | ASB1 | 1.268E-05 | -0.223 | - | - |
| 10806 | ENSP00000313199 | HNRNPD | 3.305E-05 | -0.223 | - | - |
| 10807 | ENSP00000265073 | SUB1 | 3.818E-05 | -0.223 | - | - |
| 10808 | ENSP00000258281 | TAF5L | 4.820E-05 | -0.223 | - | - |
| 10809 | ENSP00000266037 | DOCK3 | 2.197E-05 | -0.223 | - | - |
| 10810 | ENSP00000344322 | SLC23A2 | 1.262E-05 | -0.223 | - | - |
| 10811 | ENSP00000302543 | CXXC5 | 2.019E-05 | -0.223 | - | - |
| 10812 | ENSP00000253004 | ASS1 | 1.291E-05 | -0.223 | - | - |
| 10813 | ENSP00000225430 | RPL19 | 2.660E-05 | -0.223 | - | - |
| 10814 | ENSP00000342322 | TM7SF3 | 1.022E-04 | -0.223 | - | - |
| 10815 | ENSP00000284690 | DHX32 | 8.662E-06 | -0.223 | - | - |
| 10816 | ENSP00000340896 | ASH2L | 1.316E-05 | -0.223 | - | - |
| 10817 | ENSP00000369126 | CSNK1A1L | 3.127E-05 | -0.223 | - | - |
| 10818 | ENSP00000352540 | EMP2 | 2.462E-05 | -0.223 | - | - |
| 10819 | ENSP00000285243 | ANKRD40 | 8.291E-05 | -0.223 | - | - |
| 10820 | ENSP00000351244 | VN1R2 | 9.750E-06 | -0.223 | - | - |
| 10821 | ENSP00000264712 | KIF3C | 1.260E-05 | -0.223 | - | - |
| 10822 | ENSP00000318147 | EDEM3 | 3.590E-05 | -0.223 | - | - |
| 10823 | ENSP00000288532 | COQ5 | 1.675E-05 | -0.223 | - | - |
| 10824 | ENSP00000263702 | MECR | 8.617E-06 | -0.223 | - | - |
| 10825 | ENSP00000413964 | LSM14A | 8.918E-06 | -0.223 | - | - |
| 10826 | ENSP00000300835 | PRR14 | 7.665E-05 | -0.223 | - | - |
| 10827 | ENSP00000301281 | UBXN6 | 3.536E-05 | -0.223 | - | - |
| 10828 | ENSP00000314606 | SGSH | 1.636E-05 | -0.223 | - | - |
| 10829 | ENSP00000309629 | CFL1 | 1.610E-05 | -0.223 | - | - |
| 10830 | ENSP00000331897 | IDH2 | 1.615E-05 | -0.223 | - | - |
| 10831 | ENSP00000261402 | NUAK1 | 8.416E-05 | -0.223 | - | - |
| 10832 | ENSP00000265350 | MED20 | 7.274E-05 | -0.223 | - | - |
| 10833 | ENSP00000284268 | ANKH | 5.232E-05 | -0.223 | - | - |
| 10834 | ENSP00000273480 | RNF7 | 1.516E-05 | -0.223 | - | - |
| 10835 | ENSP00000295987 | SYN1 | 2.234E-05 | -0.223 | - | - |
| 10836 | ENSP00000204726 | GOLGA3 | 1.120E-04 | -0.223 | - | - |
| 10837 | ENSP00000254657 | PER2 | 5.219E-05 | -0.223 | - | - |
| 10838 | ENSP00000263273 | NUCB1 | 3.986E-05 | -0.223 | - | - |
| 10839 | ENSP00000354676 | OSTC | 4.623E-05 | -0.223 | - | - |
| 10840 | ENSP00000309198 | UBE3C | 4.531E-05 | -0.223 | - | - |
| 10841 | ENSP00000247191 | DLGAP5 | 1.515E-05 | -0.223 | - | - |
| 10842 | ENSP00000251241 | DHX40 | 6.169E-06 | -0.223 | - | - |
| 10843 | ENSP00000215565 | NDUFB7 | 8.248E-06 | -0.223 | - | - |
| 10844 | ENSP00000303057 | PPIC | 5.025E-05 | -0.223 | - | - |
| 10845 | ENSP00000300952 | MIDN | 1.050E-04 | -0.223 | - | - |
| 10846 | ENSP00000356211 | TMEM183A | 1.803E-05 | -0.223 | - | - |
| 10847 | ENSP00000340296 | C10orf129 | 9.344E-06 | -0.223 | - | - |
| 10848 | ENSP00000246151 | PITHD1 | 9.043E-05 | -0.223 | - | - |
| 10849 | ENSP00000264649 | ATP6V0A1 | 6.337E-06 | -0.223 | - | - |
| 10850 | ENSP00000343690 | DPYSL3 | 9.010E-06 | -0.223 | - | - |
| 10851 | ENSP00000266529 | ZCRB1 | 1.480E-04 | -0.223 | - | - |
| 10852 | ENSP00000356425 | UCHL5 | 2.013E-05 | -0.223 | - | - |
| 10853 | ENSP00000287598 | BUB1B | 1.548E-05 | -0.223 | - | - |
| 10854 | ENSP00000340465 | SLC20A2 | 2.086E-05 | -0.223 | - | - |
| 10855 | ENSP00000369887 | SLTM | 2.667E-05 | -0.223 | - | - |
| 10856 | ENSP00000290341 | IGF2BP1 | 3.451E-05 | -0.223 | - | - |
| 10857 | ENSP00000318804 | CRLF3 | 8.737E-05 | -0.223 | - | - |
| 10858 | ENSP00000405926 | TMED7 | 2.047E-05 | -0.223 | - | - |
| 10859 | ENSP00000398704 | SHOX2 | 3.627E-05 | -0.223 | - | - |
| 10860 | ENSP00000261302 | FOXN3 | 1.118E-05 | -0.223 | - | - |
| 10861 | ENSP00000195654 | DOPEY1 | 1.261E-05 | -0.223 | - | - |
| 10862 | ENSP00000217958 | PSMD10 | 1.358E-05 | -0.223 | - | - |
| 10863 | ENSP00000314505 | MCM9 | 8.841E-06 | -0.223 | - | - |
| 10864 | ENSP00000357858 | BUB3 | 2.377E-05 | -0.223 | - | - |
| 10865 | ENSP00000454153 | C5orf63 | 4.597E-06 | -0.223 | - | - |
| 10866 | ENSP00000337445 | ELAC2 | 1.174E-05 | -0.223 | - | - |
| 10867 | ENSP00000360652 | CXorf56 | 3.076E-05 | -0.223 | - | - |
| 10868 | ENSP00000341344 | GGA1 | 1.718E-05 | -0.223 | - | - |
| 10869 | ENSP00000268854 | DDX52 | 7.250E-06 | -0.223 | - | - |
| 10870 | ENSP00000356966 | TOMM40L | 3.269E-05 | -0.223 | - | - |
| 10871 | ENSP00000262294 | TRIM37 | 3.162E-05 | -0.223 | - | - |
| 10872 | ENSP00000272748 | KIAA1715 | 9.195E-05 | -0.223 | - | - |
| 10873 | ENSP00000369349 | KRT81 | 1.836E-05 | -0.223 | - | - |
| 10874 | ENSP00000353854 | C15orf39 | 1.758E-05 | -0.223 | - | - |
| 10875 | ENSP00000339404 | ASCC1 | 9.124E-05 | -0.223 | - | - |
| 10876 | ENSP00000173527 | ISOC1 | 7.439E-05 | -0.223 | - | - |
| 10877 | ENSP00000391457 | INO80C | 6.642E-06 | -0.223 | - | - |
| 10878 | ENSP00000225740 | ALDH3A1 | 1.091E-05 | -0.223 | - | - |
| 10879 | ENSP00000392330 | BCAP31 | 2.636E-05 | -0.223 | - | - |
| 10880 | ENSP00000340329 | CAPRIN1 | 5.792E-05 | -0.223 | - | - |
| 10881 | ENSP00000240185 | TARDBP | 3.530E-05 | -0.223 | - | - |
| 10882 | ENSP00000402551 | OTUB1 | 2.190E-05 | -0.223 | - | - |
| 10883 | ENSP00000275230 | SLC2A12 | 1.700E-05 | -0.223 | - | - |
| 10884 | ENSP00000227474 | PUS3 | 5.758E-06 | -0.223 | - | - |
| 10885 | ENSP00000343785 | SPRY1 | 4.104E-05 | -0.223 | - | - |
| 10886 | ENSP00000356520 | DHX9 | 2.126E-05 | -0.223 | - | - |
| 10887 | ENSP00000354958 | FAM189B | 2.415E-05 | -0.223 | - | - |
| 10888 | ENSP00000238994 | PPP1R3C | 3.678E-05 | -0.223 | - | - |
| 10889 | ENSP00000358851 | GSTM4 | 2.582E-05 | -0.223 | - | - |
| 10890 | ENSP00000357650 | SLC27A3 | 4.132E-05 | -0.223 | - | - |
| 10891 | ENSP00000259939 | RNF144B | 8.906E-05 | -0.223 | - | - |
| 10892 | ENSP00000311596 | ZNF699 | 2.310E-05 | -0.223 | - | - |
| 10893 | ENSP00000408697 | NUGGC | 1.221E-05 | -0.223 | - | - |
| 10894 | ENSP00000223324 | MRPL32 | 3.051E-06 | -0.223 | - | - |
| 10895 | ENSP00000231238 | TTC1 | 1.636E-05 | -0.223 | - | - |
| 10896 | ENSP00000312734 | RAB8B | 1.526E-05 | -0.223 | - | - |
| 10897 | ENSP00000262139 | WIPI1 | 1.339E-05 | -0.223 | - | - |
| 10898 | ENSP00000353491 | ZNF460 | 1.502E-05 | -0.223 | - | - |
| 10899 | ENSP00000349503 | ZBTB14 | 1.313E-05 | -0.223 | - | - |
| 10900 | ENSP00000301329 | GLOD4 | 1.270E-04 | -0.223 | - | - |
| 10901 | ENSP00000237281 | FBXO30 | 3.223E-04 | -0.223 | - | - |
| 10902 | ENSP00000343103 | PRMT7 | 1.150E-05 | -0.223 | - | - |
| 10903 | ENSP00000376765 | PIAS3 | 3.762E-05 | -0.223 | - | - |
| 10904 | ENSP00000369857 | FAM175A | 1.238E-05 | -0.223 | - | - |
| 10905 | ENSP00000425679 | - | 5.947E-06 | -0.223 | - | - |
| 10906 | ENSP00000344724 | TOX2 | 1.145E-05 | -0.223 | - | - |
| 10907 | ENSP00000343002 | B3GALTL | 6.941E-05 | -0.223 | - | - |
| 10908 | ENSP00000359398 | BLOC1S2 | 2.415E-05 | -0.223 | - | - |
| 10909 | ENSP00000344488 | JPH1 | 2.554E-05 | -0.223 | - | - |
| 10910 | ENSP00000324605 | ZNF660 | 2.119E-05 | -0.224 | - | - |
| 10911 | ENSP00000300146 | PATL1 | 1.834E-05 | -0.224 | - | - |
| 10912 | ENSP00000229214 | KRR1 | 8.575E-06 | -0.224 | - | - |
| 10913 | ENSP00000410768 | FAM135A | 2.717E-05 | -0.224 | - | - |
| 10914 | ENSP00000375809 | ERCC2 | 2.800E-05 | -0.224 | - | - |
| 10915 | ENSP00000318690 | GCFC2 | 1.475E-05 | -0.224 | - | - |
| 10916 | ENSP00000430333 | SLIT3 | 4.833E-05 | -0.224 | - | - |
| 10917 | ENSP00000363064 | ZNF76 | 1.689E-05 | -0.224 | - | - |
| 10918 | ENSP00000323858 | DDX54 | 1.479E-05 | -0.224 | - | - |
| 10919 | ENSP00000447679 | PRR24 | 8.371E-07 | -0.224 | - | - |
| 10920 | ENSP00000263083 | DPH1 | 1.796E-05 | -0.224 | - | - |
| 10921 | ENSP00000156825 | MBD3 | 8.702E-05 | -0.224 | - | - |
| 10922 | ENSP00000278317 | TNNT3 | 1.766E-05 | -0.224 | - | - |
| 10923 | ENSP00000351644 | GTPBP3 | 1.820E-05 | -0.224 | - | - |
| 10924 | ENSP00000285896 | CNOT8 | 3.330E-05 | -0.224 | - | - |
| 10925 | ENSP00000228506 | MLEC | 9.104E-05 | -0.224 | - | - |
| 10926 | ENSP00000443989 | PPIAL4B | 2.465E-05 | -0.224 | - | - |
| 10927 | ENSP00000282486 | MBNL1 | 2.685E-05 | -0.224 | - | - |
| 10928 | ENSP00000425493 | CAGE1 | 2.042E-05 | -0.224 | - | - |
| 10929 | ENSP00000333948 | RPS19BP1 | 5.689E-05 | -0.224 | - | - |
| 10930 | ENSP00000263934 | KIF1B | 1.820E-05 | -0.224 | - | - |
| 10931 | ENSP00000198765 | CPNE3 | 5.019E-05 | -0.224 | - | - |
| 10932 | ENSP00000362344 | FPGS | 2.294E-05 | -0.224 | - | - |
| 10933 | ENSP00000265339 | UBE2B | 1.441E-05 | -0.224 | - | - |
| 10934 | ENSP00000320658 | YARS2 | 2.162E-05 | -0.224 | - | - |
| 10935 | ENSP00000347046 | PDE5A | 1.718E-05 | -0.224 | - | - |
| 10936 | ENSP00000261858 | GLCE | 5.000E-05 | -0.224 | - | - |
| 10937 | ENSP00000261733 | ALDH2 | 1.302E-05 | -0.224 | - | - |
| 10938 | ENSP00000221086 | MTMR9 | 5.065E-05 | -0.224 | - | - |
| 10939 | ENSP00000353581 | HIST1H3E | 2.251E-05 | -0.224 | - | - |
| 10940 | ENSP00000330432 | FAM83F | 1.026E-04 | -0.224 | - | - |
| 10941 | ENSP00000349508 | CHD4 | 2.129E-05 | -0.224 | - | - |
| 10942 | ENSP00000256497 | EDEM1 | 3.463E-05 | -0.224 | - | - |
| 10943 | ENSP00000263694 | SNRNP40 | 2.723E-05 | -0.224 | - | - |
| 10944 | ENSP00000359978 | HHLA3 | 1.088E-05 | -0.224 | - | - |
| 10945 | ENSP00000401363 | DAZ4 | 1.257E-05 | -0.224 | - | - |
| 10946 | ENSP00000377225 | FAM50A | 8.248E-06 | -0.224 | - | - |
| 10947 | ENSP00000262428 | COTL1 | 7.423E-05 | -0.224 | - | - |
| 10948 | ENSP00000280557 | DENR | 3.570E-05 | -0.224 | - | - |
| 10949 | ENSP00000379503 | PDP1 | 2.135E-05 | -0.224 | - | - |
| 10950 | ENSP00000262370 | MGRN1 | 7.453E-05 | -0.224 | - | - |
| 10951 | ENSP00000221486 | RNASEH2A | 7.369E-06 | -0.224 | - | - |
| 10952 | ENSP00000257575 | RNFT2 | 7.922E-06 | -0.224 | - | - |
| 10953 | ENSP00000222345 | SIPA1L3 | 5.356E-05 | -0.224 | - | - |
| 10954 | ENSP00000251810 | RRM2B | 1.570E-05 | -0.224 | - | - |
| 10955 | ENSP00000342267 | SLC25A15 | 5.251E-05 | -0.224 | - | - |
| 10956 | ENSP00000233047 | TMEM159 | 4.424E-04 | -0.224 | - | - |
| 10957 | ENSP00000304364 | C1GALT1C1 | 1.238E-05 | -0.224 | - | - |
| 10958 | ENSP00000316339 | HAO2 | 1.985E-05 | -0.224 | - | - |
| 10959 | ENSP00000267197 | SETD1B | 8.315E-06 | -0.224 | - | - |
| 10960 | ENSP00000337335 | SLC22A8 | 1.351E-05 | -0.224 | - | - |
| 10961 | ENSP00000284503 | NEIL2 | 8.905E-05 | -0.224 | - | - |
| 10962 | ENSP00000300209 | METTL21B | 8.328E-05 | -0.224 | - | - |
| 10963 | ENSP00000393571 | SLC35F2 | 1.925E-05 | -0.224 | - | - |
| 10964 | ENSP00000371003 | ZMYND11 | 1.645E-05 | -0.224 | - | - |
| 10965 | ENSP00000334472 | FOXE3 | 1.612E-05 | -0.224 | - | - |
| 10966 | ENSP00000260803 | DBR1 | 3.713E-05 | -0.224 | - | - |
| 10967 | ENSP00000312678 | MID1 | 7.659E-05 | -0.224 | - | - |
| 10968 | ENSP00000304586 | ANKS3 | 1.003E-05 | -0.224 | - | - |
| 10969 | ENSP00000285697 | C16orf87 | 1.616E-04 | -0.224 | - | - |
| 10970 | ENSP00000012134 | HIVEP2 | 1.902E-05 | -0.224 | - | - |
| 10971 | ENSP00000270257 | GEMIN7 | 6.206E-05 | -0.224 | - | - |
| 10972 | ENSP00000309831 | SNUPN | 5.710E-05 | -0.224 | - | - |
| 10973 | ENSP00000450980 | DIO2 | 8.051E-06 | -0.224 | - | - |
| 10974 | ENSP00000367001 | DPP6 | 9.526E-06 | -0.224 | - | - |
| 10975 | ENSP00000342137 | NCCRP1 | 3.994E-04 | -0.224 | - | - |
| 10976 | ENSP00000225719 | CPD | 7.257E-05 | -0.224 | - | - |
| 10977 | ENSP00000258648 | MED4 | 4.084E-05 | -0.224 | - | - |
| 10978 | ENSP00000310596 | LSM1 | 1.407E-05 | -0.224 | - | - |
| 10979 | ENSP00000376178 | STX2 | 1.948E-05 | -0.224 | - | - |
| 10980 | ENSP00000317224 | SAMD4B | 4.873E-05 | -0.224 | - | - |
| 10981 | ENSP00000221200 | KCTD9 | 5.247E-05 | -0.224 | - | - |
| 10982 | ENSP00000429824 | CLINT1 | 2.409E-05 | -0.224 | - | - |
| 10983 | ENSP00000296161 | DTX3L | 1.120E-04 | -0.224 | - | - |
| 10984 | ENSP00000323527 | MKLN1 | 8.556E-05 | -0.224 | - | - |
| 10985 | ENSP00000438875 | NOVA1 | 1.324E-05 | -0.224 | - | - |
| 10986 | ENSP00000354376 | RAB25 | 3.291E-05 | -0.224 | - | - |
| 10987 | ENSP00000295645 | PDCL2 | 2.520E-05 | -0.224 | - | - |
| 10988 | ENSP00000319501 | UGDH | 6.107E-05 | -0.224 | - | - |
| 10989 | ENSP00000398401 | FAM133B | 7.831E-06 | -0.224 | - | - |
| 10990 | ENSP00000380897 | TRPM1 | 1.913E-05 | -0.224 | - | - |
| 10991 | ENSP00000427687 | WDR1 | 3.060E-05 | -0.224 | - | - |
| 10992 | ENSP00000351777 | VCP | 2.756E-05 | -0.224 | - | - |
| 10993 | ENSP00000255416 | MYBPH | 5.431E-05 | -0.224 | - | - |
| 10994 | ENSP00000331369 | BOLA3 | 1.151E-05 | -0.224 | - | - |
| 10995 | ENSP00000333633 | MTA1 | 4.195E-05 | -0.224 | - | - |
| 10996 | ENSP00000298492 | FAM175B | 1.286E-04 | -0.224 | - | - |
| 10997 | ENSP00000306983 | YEATS2 | 1.326E-05 | -0.224 | - | - |
| 10998 | ENSP00000245255 | PIWIL1 | 2.512E-05 | -0.224 | - | - |
| 10999 | ENSP00000328103 | EIF4ENIF1 | 4.921E-05 | -0.224 | - | - |
| 11000 | ENSP00000336747 | HIP1 | 1.387E-05 | -0.224 | - | - |
| 11001 | ENSP00000261880 | AAGAB | 1.650E-04 | -0.224 | - | - |
| 11002 | ENSP00000366819 | UCHL3 | 2.906E-05 | -0.224 | - | - |
| 11003 | ENSP00000263549 | PARP12 | 9.830E-05 | -0.224 | - | - |
| 11004 | ENSP00000356444 | SAMD5 | 2.080E-05 | -0.224 | - | - |
| 11005 | ENSP00000261465 | HSD11B1 | 2.098E-05 | -0.224 | - | - |
| 11006 | ENSP00000328494 | KIF21B | 2.398E-05 | -0.224 | - | - |
| 11007 | ENSP00000264399 | PRKG2 | 1.813E-05 | -0.224 | - | - |
| 11008 | ENSP00000329117 | AMER1 | 5.665E-05 | -0.224 | - | - |
| 11009 | ENSP00000201031 | TFAP2C | 5.016E-05 | -0.224 | - | - |
| 11010 | ENSP00000327116 | EHD3 | 1.553E-05 | -0.224 | - | - |
| 11011 | ENSP00000333920 | TTF1 | 1.635E-05 | -0.224 | - | - |
| 11012 | ENSP00000320171 | PKM | 2.377E-05 | -0.224 | - | - |
| 11013 | ENSP00000240731 | ZNF211 | 2.442E-05 | -0.224 | - | - |
| 11014 | ENSP00000254719 | RPA1 | 1.114E-05 | -0.224 | - | - |
| 11015 | ENSP00000338533 | TTC33 | 2.957E-04 | -0.224 | - | - |
| 11016 | ENSP00000408598 | PABPN1L | 6.369E-06 | -0.224 | - | - |
| 11017 | ENSP00000253856 | ATP6V0A4 | 6.425E-06 | -0.224 | - | - |
| 11018 | ENSP00000295959 | RPP14 | 4.890E-04 | -0.224 | - | - |
| 11019 | ENSP00000254624 | EFR3A | 1.176E-04 | -0.224 | - | - |
| 11020 | ENSP00000266397 | ERP27 | 8.500E-06 | -0.225 | - | - |
| 11021 | ENSP00000334229 | C2CD5 | 7.970E-05 | -0.225 | - | - |
| 11022 | ENSP00000249822 | ARPP19 | 5.829E-05 | -0.225 | - | - |
| 11023 | ENSP00000310471 | RBM4B | 1.161E-04 | -0.225 | - | - |
| 11024 | ENSP00000340594 | SYDE2 | 2.185E-05 | -0.225 | - | - |
| 11025 | ENSP00000324204 | C7orf41 | 2.634E-04 | -0.225 | - | - |
| 11026 | ENSP00000273783 | EIF2B5 | 1.364E-05 | -0.225 | - | - |
| 11027 | ENSP00000286307 | LSM11 | 6.351E-05 | -0.225 | - | - |
| 11028 | ENSP00000339238 | KRTAP24-1 | 5.622E-06 | -0.225 | - | - |
| 11029 | ENSP00000376914 | ATP2C1 | 2.280E-05 | -0.225 | - | - |
| 11030 | ENSP00000414359 | PSMA1 | 3.606E-05 | -0.225 | - | - |
| 11031 | ENSP00000278193 | LIN7C | 3.761E-05 | -0.225 | - | - |
| 11032 | ENSP00000326879 | C5orf22 | 3.331E-04 | -0.225 | - | - |
| 11033 | ENSP00000261386 | GDE1 | 1.870E-05 | -0.225 | - | - |
| 11034 | ENSP00000261772 | AARS | 1.373E-05 | -0.225 | - | - |
| 11035 | ENSP00000455444 | TCF24 | 7.544E-06 | -0.225 | - | - |
| 11036 | ENSP00000310182 | C4orf32 | 2.141E-04 | -0.225 | - | - |
| 11037 | ENSP00000299498 | CYB5R2 | 1.649E-05 | -0.225 | - | - |
| 11038 | ENSP00000010299 | FAM76A | 1.011E-04 | -0.225 | - | - |
| 11039 | ENSP00000353953 | PAQR8 | 2.778E-05 | -0.225 | - | - |
| 11040 | ENSP00000334642 | PDZD8 | 7.165E-05 | -0.225 | - | - |
| 11041 | ENSP00000332931 | SLC8A1 | 9.971E-06 | -0.225 | - | - |
| 11042 | ENSP00000291688 | MCM3AP | 1.538E-05 | -0.225 | - | - |
| 11043 | ENSP00000205636 | CMTM6 | 6.534E-05 | -0.225 | - | - |
| 11044 | ENSP00000307449 | NECAB2 | 2.299E-05 | -0.225 | - | - |
| 11045 | ENSP00000317310 | CABP1 | 2.203E-05 | -0.225 | - | - |
| 11046 | ENSP00000307798 | FAM90A1 | 3.792E-05 | -0.225 | - | - |
| 11047 | ENSP00000280904 | DSC2 | 3.535E-05 | -0.225 | - | - |
| 11048 | ENSP00000307549 | NPTX1 | 1.062E-04 | -0.225 | - | - |
| 11049 | ENSP00000300441 | ACSF2 | 1.024E-05 | -0.225 | - | - |
| 11050 | ENSP00000309175 | EIF1AD | 6.100E-05 | -0.225 | - | - |
| 11051 | ENSP00000260952 | ASNSD1 | 1.694E-05 | -0.225 | - | - |
| 11052 | ENSP00000256689 | SLC38A2 | 3.253E-05 | -0.225 | - | - |
| 11053 | ENSP00000342148 | TMEM167B | 1.490E-04 | -0.225 | - | - |
| 11054 | ENSP00000363092 | PRKG1 | 3.883E-05 | -0.225 | - | - |
| 11055 | ENSP00000343867 | MRRF | 8.392E-06 | -0.225 | - | - |
| 11056 | ENSP00000264808 | PRDM5 | 2.268E-05 | -0.225 | - | - |
| 11057 | ENSP00000321573 | DCBLD2 | 6.340E-05 | -0.225 | - | - |
| 11058 | ENSP00000206542 | OSGEP | 1.590E-05 | -0.225 | - | - |
| 11059 | ENSP00000337386 | ZFP36L1 | 8.783E-05 | -0.225 | - | - |
| 11060 | ENSP00000341597 | FAM114A2 | 2.198E-04 | -0.225 | - | - |
| 11061 | ENSP00000250124 | MPDU1 | 3.181E-05 | -0.225 | - | - |
| 11062 | ENSP00000315182 | SHQ1 | 5.030E-05 | -0.225 | - | - |
| 11063 | ENSP00000296666 | PRRC1 | 9.102E-05 | -0.225 | - | - |
| 11064 | ENSP00000264312 | OCIAD1 | 6.726E-05 | -0.225 | - | - |
| 11065 | ENSP00000329170 | POU3F2 | 3.662E-05 | -0.225 | - | - |
| 11066 | ENSP00000319726 | FRMD8 | 1.259E-04 | -0.225 | - | - |
| 11067 | ENSP00000215057 | MZF1 | 1.943E-05 | -0.225 | - | - |
| 11068 | ENSP00000295958 | SMIM14 | 1.902E-04 | -0.225 | - | - |
| 11069 | ENSP00000329933 | PHF12 | 1.243E-05 | -0.225 | - | - |
| 11070 | ENSP00000227322 | ZNF259 | 2.475E-05 | -0.225 | - | - |
| 11071 | ENSP00000335201 | BCDIN3D | 4.320E-05 | -0.225 | - | - |
| 11072 | ENSP00000327268 | NDUFV2 | 7.884E-06 | -0.225 | - | - |
| 11073 | ENSP00000319281 | BASP1 | 1.715E-04 | -0.225 | - | - |
| 11074 | ENSP00000375133 | ZRSR1 | 1.047E-05 | -0.225 | - | - |
| 11075 | ENSP00000309432 | FAM134C | 8.035E-05 | -0.225 | - | - |
| 11076 | ENSP00000317177 | PLD6 | 2.247E-05 | -0.225 | - | - |
| 11077 | ENSP00000225573 | PNPO | 4.479E-05 | -0.225 | - | - |
| 11078 | ENSP00000337226 | CDCA4 | 7.896E-05 | -0.225 | - | - |
| 11079 | ENSP00000331787 | METTL7A | 3.807E-05 | -0.225 | - | - |
| 11080 | ENSP00000301335 | SLC43A2 | 5.929E-05 | -0.225 | - | - |
| 11081 | ENSP00000301096 | ZNF83 | 2.251E-05 | -0.225 | - | - |
| 11082 | ENSP00000360876 | IFIT3 | 3.757E-05 | -0.225 | - | - |
| 11083 | ENSP00000258439 | TMEM127 | 8.733E-05 | -0.225 | - | - |
| 11084 | ENSP00000299157 | IKBIP | 6.977E-05 | -0.225 | - | - |
| 11085 | ENSP00000278671 | LAMTOR1 | 7.975E-05 | -0.225 | - | - |
| 11086 | ENSP00000424183 | LAMTOR3 | 1.287E-05 | -0.225 | - | - |
| 11087 | ENSP00000322926 | FARP1 | 6.726E-05 | -0.225 | - | - |
| 11088 | ENSP00000375478 | KRTAP10-3 | 2.878E-05 | -0.225 | - | - |
| 11089 | ENSP00000234310 | PPP3R1 | 1.687E-05 | -0.225 | - | - |
| 11090 | ENSP00000249064 | CCDC117 | 4.920E-05 | -0.225 | - | - |
| 11091 | ENSP00000380315 | ZNF208 | 1.045E-05 | -0.225 | - | - |
| 11092 | ENSP00000255006 | RIN2 | 8.185E-05 | -0.225 | - | - |
| 11093 | ENSP00000257829 | NAT10 | 7.590E-06 | -0.225 | - | - |
| 11094 | ENSP00000306220 | MMGT1 | 5.563E-05 | -0.225 | - | - |
| 11095 | ENSP00000282892 | MED21 | 3.637E-05 | -0.225 | - | - |
| 11096 | ENSP00000257787 | AKIRIN2 | 5.910E-05 | -0.225 | - | - |
| 11097 | ENSP00000441365 | MARVELD1 | 1.690E-05 | -0.225 | - | - |
| 11098 | ENSP00000296255 | RPN1 | 3.177E-05 | -0.225 | - | - |
| 11099 | ENSP00000289431 | SPATA2 | 7.719E-05 | -0.225 | - | - |
| 11100 | ENSP00000284274 | FAM105B | 1.170E-04 | -0.225 | - | - |
| 11101 | ENSP00000270861 | PLK4 | 1.380E-05 | -0.225 | - | - |
| 11102 | ENSP00000299163 | HIF1AN | 4.487E-05 | -0.225 | - | - |
| 11103 | ENSP00000331106 | PEX26 | 1.446E-04 | -0.225 | - | - |
| 11104 | ENSP00000292672 | CELF5 | 6.244E-05 | -0.225 | - | - |
| 11105 | ENSP00000260648 | PREPL | 4.755E-05 | -0.225 | - | - |
| 11106 | ENSP00000309509 | PLAC8 | 5.450E-05 | -0.225 | - | - |
| 11107 | ENSP00000448059 | PAH | 2.288E-05 | -0.225 | - | - |
| 11108 | ENSP00000319979 | B3GNTL1 | 1.682E-05 | -0.225 | - | - |
| 11109 | ENSP00000389277 | TMBIM6 | 3.210E-05 | -0.225 | - | - |
| 11110 | ENSP00000240285 | RDH10 | 3.938E-05 | -0.225 | - | - |
| 11111 | ENSP00000453403 | NUSAP1 | 9.001E-06 | -0.225 | - | - |
| 11112 | ENSP00000333724 | OR6X1 | 5.448E-06 | -0.225 | - | - |
| 11113 | ENSP00000302227 | CCBL1 | 1.836E-05 | -0.225 | - | - |
| 11114 | ENSP00000175091 | LAPTM4A | 4.583E-05 | -0.225 | - | - |
| 11115 | ENSP00000238875 | LGALSL | 5.969E-05 | -0.225 | - | - |
| 11116 | ENSP00000238936 | TMEM180 | 1.318E-04 | -0.225 | - | - |
| 11117 | ENSP00000273064 | RQCD1 | 3.369E-05 | -0.225 | - | - |
| 11118 | ENSP00000253458 | GSE1 | 5.262E-05 | -0.225 | - | - |
| 11119 | ENSP00000239231 | PANK3 | 6.343E-05 | -0.225 | - | - |
| 11120 | ENSP00000321537 | LLGL1 | 2.912E-05 | -0.225 | - | - |
| 11121 | ENSP00000326128 | LARP4B | 3.081E-05 | -0.225 | - | - |
| 11122 | ENSP00000287143 | PRG3 | 2.369E-05 | -0.225 | - | - |
| 11123 | ENSP00000273986 | CISD2 | 3.493E-05 | -0.225 | - | - |
| 11124 | ENSP00000320043 | LYPLA1 | 4.526E-05 | -0.225 | - | - |
| 11125 | ENSP00000450685 | C14orf64 | 4.555E-06 | -0.225 | - | - |
| 11126 | ENSP00000276569 | ARMC1 | 8.007E-05 | -0.225 | - | - |
| 11127 | ENSP00000366527 | ZSCAN16 | 1.548E-05 | -0.225 | - | - |
| 11128 | ENSP00000365075 | CELA2B | 2.376E-05 | -0.225 | - | - |
| 11129 | ENSP00000265843 | EXPH5 | 8.585E-05 | -0.225 | - | - |
| 11130 | ENSP00000271620 | PRUNE | 6.376E-05 | -0.225 | - | - |
| 11131 | ENSP00000307411 | MLLT10 | 3.266E-05 | -0.225 | - | - |
| 11132 | ENSP00000300215 | EPB42 | 1.211E-05 | -0.225 | - | - |
| 11133 | ENSP00000261893 | LACTB | 3.445E-05 | -0.225 | - | - |
| 11134 | ENSP00000337222 | MYADM | 5.783E-05 | -0.225 | - | - |
| 11135 | ENSP00000204549 | PDCD7 | 8.734E-05 | -0.225 | - | - |
| 11136 | ENSP00000326933 | SERTAD2 | 1.253E-04 | -0.225 | - | - |
| 11137 | ENSP00000381928 | MGARP | 1.430E-05 | -0.225 | - | - |
| 11138 | ENSP00000344562 | CORO6 | 2.090E-05 | -0.225 | - | - |
| 11139 | ENSP00000303909 | ABR | 4.004E-05 | -0.225 | - | - |
| 11140 | ENSP00000219204 | ARL2BP | 1.199E-04 | -0.225 | - | - |
| 11141 | ENSP00000261739 | ANKRD13A | 6.607E-05 | -0.225 | - | - |
| 11142 | ENSP00000234301 | COX7A2L | 8.786E-05 | -0.225 | - | - |
| 11143 | ENSP00000325369 | AP3S1 | 4.029E-05 | -0.225 | - | - |
| 11144 | ENSP00000306788 | ARL6IP1 | 6.186E-05 | -0.225 | - | - |
| 11145 | ENSP00000333799 | WBSCR16 | 1.853E-05 | -0.225 | - | - |
| 11146 | ENSP00000263360 | EED | 1.869E-05 | -0.225 | - | - |
| 11147 | ENSP00000242351 | ZC3HAV1 | 6.844E-05 | -0.225 | - | - |
| 11148 | ENSP00000304277 | TM4SF1 | 5.013E-05 | -0.225 | - | - |
| 11149 | ENSP00000358884 | CYB561D1 | 4.829E-06 | -0.225 | - | - |
| 11150 | ENSP00000307854 | PCMTD2 | 8.212E-06 | -0.226 | - | - |
| 11151 | ENSP00000322991 | BCAT2 | 2.230E-05 | -0.226 | - | - |
| 11152 | ENSP00000322373 | PLEKHF2 | 3.809E-05 | -0.226 | - | - |
| 11153 | ENSP00000216254 | ACO2 | 1.749E-05 | -0.226 | - | - |
| 11154 | ENSP00000280258 | PRSS23 | 4.866E-05 | -0.226 | - | - |
| 11155 | ENSP00000385019 | CACNA1I | 2.033E-05 | -0.226 | - | - |
| 11156 | ENSP00000292879 | U2AF1L4 | 4.040E-05 | -0.226 | - | - |
| 11157 | ENSP00000300231 | MAP1A | 4.828E-05 | -0.226 | - | - |
| 11158 | ENSP00000272638 | UBXN4 | 4.270E-05 | -0.226 | - | - |
| 11159 | ENSP00000218004 | NXT2 | 8.219E-05 | -0.226 | - | - |
| 11160 | ENSP00000288680 | SPPL3 | 5.185E-05 | -0.226 | - | - |
| 11161 | ENSP00000376886 | NME1-NME2 | 2.087E-05 | -0.226 | - | - |
| 11162 | ENSP00000215555 | 2-Mar | 3.958E-05 | -0.226 | - | - |
| 11163 | ENSP00000301364 | TSR1 | 1.298E-05 | -0.226 | - | - |
| 11164 | ENSP00000287916 | CLDN12 | 6.598E-05 | -0.226 | - | - |
| 11165 | ENSP00000273963 | KLHL8 | 3.875E-05 | -0.226 | - | - |
| 11166 | ENSP00000229769 | FANCE | 7.463E-05 | -0.226 | - | - |
| 11167 | ENSP00000339115 | ESPNL | 1.599E-05 | -0.226 | - | - |
| 11168 | ENSP00000346694 | HNRNPA2B1 | 1.457E-05 | -0.226 | - | - |
| 11169 | ENSP00000301480 | ZNF560 | 2.412E-05 | -0.226 | - | - |
| 11170 | ENSP00000193322 | OSTM1 | 3.995E-05 | -0.226 | - | - |
| 11171 | ENSP00000234831 | TMEM59 | 6.184E-05 | -0.226 | - | - |
| 11172 | ENSP00000301585 | ICT1 | 1.150E-05 | -0.226 | - | - |
| 11173 | ENSP00000291598 | ZNF583 | 2.205E-05 | -0.226 | - | - |
| 11174 | ENSP00000307241 | PDHB | 1.180E-05 | -0.226 | - | - |
| 11175 | ENSP00000231512 | C5orf15 | 5.486E-05 | -0.226 | - | - |
| 11176 | ENSP00000286794 | NAA11 | 1.346E-05 | -0.226 | - | - |
| 11177 | ENSP00000260363 | KIF23 | 1.093E-05 | -0.226 | - | - |
| 11178 | ENSP00000296503 | HMGB2 | 2.436E-05 | -0.226 | - | - |
| 11179 | ENSP00000254029 | WDR44 | 5.360E-05 | -0.226 | - | - |
| 11180 | ENSP00000282388 | ZFP36L2 | 2.894E-05 | -0.226 | - | - |
| 11181 | ENSP00000453144 | APBA2 | 3.813E-05 | -0.226 | - | - |
| 11182 | ENSP00000318177 | FUBP3 | 3.207E-05 | -0.226 | - | - |
| 11183 | ENSP00000403476 | SH3D21 | 1.956E-05 | -0.226 | - | - |
| 11184 | ENSP00000316007 | SCAMP4 | 7.832E-05 | -0.226 | - | - |
| 11185 | ENSP00000399392 | C3orf52 | 8.927E-06 | -0.226 | - | - |
| 11186 | ENSP00000308622 | GLE1 | 3.615E-05 | -0.226 | - | - |
| 11187 | ENSP00000257430 | APC | 3.927E-05 | -0.226 | - | - |
| 11188 | ENSP00000255759 | FOPNL | 5.758E-05 | -0.226 | - | - |
| 11189 | ENSP00000343838 | UGT1A10 | 1.633E-05 | -0.226 | - | - |
| 11190 | ENSP00000331019 | RPS27L | 1.131E-05 | -0.226 | - | - |
| 11191 | ENSP00000306123 | RPAP1 | 4.363E-05 | -0.226 | - | - |
| 11192 | ENSP00000433757 | TTC12 | 2.682E-05 | -0.226 | - | - |
| 11193 | ENSP00000425421 | SATL1 | 1.319E-05 | -0.226 | - | - |
| 11194 | ENSP00000293362 | PSME3 | 3.384E-05 | -0.226 | - | - |
| 11195 | ENSP00000340836 | OSTF1 | 2.507E-05 | -0.226 | - | - |
| 11196 | ENSP00000243298 | RAB9B | 8.632E-06 | -0.226 | - | - |
| 11197 | ENSP00000263791 | EIF2AK4 | 1.999E-05 | -0.226 | - | - |
| 11198 | ENSP00000251864 | TDRD1 | 2.329E-05 | -0.226 | - | - |
| 11199 | ENSP00000260585 | EPT1 | 2.982E-05 | -0.226 | - | - |
| 11200 | ENSP00000328789 | AP1S2 | 3.344E-05 | -0.226 | - | - |
| 11201 | ENSP00000264993 | CDV3 | 7.123E-05 | -0.226 | - | - |
| 11202 | ENSP00000320815 | GDPD4 | 1.618E-05 | -0.226 | - | - |
| 11203 | ENSP00000255305 | XPO4 | 5.611E-05 | -0.226 | - | - |
| 11204 | ENSP00000285518 | AGPAT5 | 1.889E-05 | -0.226 | - | - |
| 11205 | ENSP00000245787 | INSIG2 | 1.367E-05 | -0.226 | - | - |
| 11206 | ENSP00000269195 | GALNT1 | 3.112E-05 | -0.226 | - | - |
| 11207 | ENSP00000400958 | PHTF2 | 6.139E-06 | -0.226 | - | - |
| 11208 | ENSP00000262992 | INPP4B | 4.075E-05 | -0.226 | - | - |
| 11209 | ENSP00000309610 | METTL2A | 5.093E-05 | -0.226 | - | - |
| 11210 | ENSP00000296220 | OSBPL11 | 2.266E-05 | -0.226 | - | - |
| 11211 | ENSP00000242810 | KLHL24 | 5.041E-05 | -0.226 | - | - |
| 11212 | ENSP00000236137 | SLC19A2 | 2.980E-05 | -0.226 | - | - |
| 11213 | ENSP00000372335 | NELFA | 7.185E-06 | -0.226 | - | - |
| 11214 | ENSP00000407353 | IFLTD1 | 8.835E-06 | -0.226 | - | - |
| 11215 | ENSP00000259746 | TMEM63B | 6.199E-05 | -0.226 | - | - |
| 11216 | ENSP00000464803 | RP11-795F19.5 | 2.130E-07 | -0.226 | - | - |
| 11217 | ENSP00000331608 | IRX3 | 2.268E-05 | -0.226 | - | - |
| 11218 | ENSP00000264447 | ZNF638 | 3.763E-05 | -0.226 | - | - |
| 11219 | ENSP00000340118 | LITAF | 4.620E-05 | -0.226 | - | - |
| 11220 | ENSP00000318298 | FAM46A | 5.474E-05 | -0.226 | - | - |
| 11221 | ENSP00000321706 | GEMIN4 | 5.754E-05 | -0.226 | - | - |
| 11222 | ENSP00000218343 | PHF16 | 3.642E-05 | -0.226 | - | - |
| 11223 | ENSP00000439918 | RBM7 | 2.492E-05 | -0.226 | - | - |
| 11224 | ENSP00000371886 | JMJD7-PLA2G4B | 2.260E-05 | -0.226 | - | - |
| 11225 | ENSP00000458954 | NUP88 | 1.065E-05 | -0.226 | - | - |
| 11226 | ENSP00000240619 | TAS2R10 | 2.634E-05 | -0.226 | - | - |
| 11227 | ENSP00000043402 | RTN4R | 1.824E-05 | -0.226 | - | - |
| 11228 | ENSP00000315674 | NOP14 | 8.388E-06 | -0.226 | - | - |
| 11229 | ENSP00000262844 | AMMECR1 | 3.774E-05 | -0.226 | - | - |
| 11230 | ENSP00000296135 | LZTFL1 | 5.542E-05 | -0.226 | - | - |
| 11231 | ENSP00000258960 | NMT1 | 1.656E-05 | -0.226 | - | - |
| 11232 | ENSP00000278826 | TMEM138 | 5.126E-05 | -0.226 | - | - |
| 11233 | ENSP00000263650 | OSBPL5 | 9.668E-06 | -0.226 | - | - |
| 11234 | ENSP00000258796 | TTYH3 | 5.224E-05 | -0.226 | - | - |
| 11235 | ENSP00000264645 | CASC3 | 3.411E-05 | -0.226 | - | - |
| 11236 | ENSP00000318631 | HSD17B6 | 5.233E-05 | -0.226 | - | - |
| 11237 | ENSP00000323264 | MARVELD2 | 3.862E-05 | -0.226 | - | - |
| 11238 | ENSP00000305059 | MAP1LC3B2 | 1.761E-05 | -0.226 | - | - |
| 11239 | ENSP00000389103 | RPL23A | 1.039E-05 | -0.226 | - | - |
| 11240 | ENSP00000266544 | NDUFA9 | 3.090E-05 | -0.226 | - | - |
| 11241 | ENSP00000275780 | TLK2 | 3.429E-05 | -0.226 | - | - |
| 11242 | ENSP00000221700 | CYP4F2 | 1.502E-05 | -0.226 | - | - |
| 11243 | ENSP00000217086 | SALL4 | 4.138E-05 | -0.226 | - | - |
| 11244 | ENSP00000319482 | DCUN1D3 | 5.953E-05 | -0.226 | - | - |
| 11245 | ENSP00000310721 | CYP7B1 | 1.053E-05 | -0.226 | - | - |
| 11246 | ENSP00000330221 | SPSB1 | 3.193E-05 | -0.226 | - | - |
| 11247 | ENSP00000292180 | FLAD1 | 1.516E-05 | -0.226 | - | - |
| 11248 | ENSP00000308772 | ANKS4B | 1.622E-05 | -0.226 | - | - |
| 11249 | ENSP00000257909 | TROAP | 4.479E-05 | -0.226 | - | - |
| 11250 | ENSP00000380999 | ZNF846 | 1.520E-05 | -0.226 | - | - |
| 11251 | ENSP00000328178 | PPP1R2 | 3.714E-05 | -0.226 | - | - |
| 11252 | ENSP00000300128 | TMEM194A | 4.967E-05 | -0.226 | - | - |
| 11253 | ENSP00000346255 | PPTC7 | 2.386E-05 | -0.226 | - | - |
| 11254 | ENSP00000011898 | TSPAN9 | 3.727E-05 | -0.226 | - | - |
| 11255 | ENSP00000291552 | U2AF1 | 2.761E-05 | -0.226 | - | - |
| 11256 | ENSP00000314480 | UNCX | 3.064E-05 | -0.226 | - | - |
| 11257 | ENSP00000291842 | SHKBP1 | 3.389E-05 | -0.226 | - | - |
| 11258 | ENSP00000314724 | PPFIBP1 | 5.039E-05 | -0.226 | - | - |
| 11259 | ENSP00000365407 | ZNF549 | 1.318E-05 | -0.226 | - | - |
| 11260 | ENSP00000275732 | GIGYF1 | 6.249E-05 | -0.226 | - | - |
| 11261 | ENSP00000299381 | ANAPC16 | 5.140E-05 | -0.226 | - | - |
| 11262 | ENSP00000334625 | DCUN1D4 | 2.387E-05 | -0.226 | - | - |
| 11263 | ENSP00000309117 | RBFOX1 | 2.306E-05 | -0.226 | - | - |
| 11264 | ENSP00000264027 | SC5D | 2.191E-05 | -0.226 | - | - |
| 11265 | ENSP00000301215 | ZNF526 | 2.192E-05 | -0.226 | - | - |
| 11266 | ENSP00000264254 | PDCL3 | 2.899E-05 | -0.226 | - | - |
| 11267 | ENSP00000467931 | ZNF461 | 1.076E-05 | -0.226 | - | - |
| 11268 | ENSP00000216218 | ST13 | 3.285E-05 | -0.226 | - | - |
| 11269 | ENSP00000338523 | FNDC3B | 4.288E-05 | -0.226 | - | - |
| 11270 | ENSP00000328251 | MINA | 2.706E-05 | -0.226 | - | - |
| 11271 | ENSP00000327436 | SETD3 | 4.410E-05 | -0.226 | - | - |
| 11272 | ENSP00000273854 | EPHA5 | 2.012E-05 | -0.226 | - | - |
| 11273 | ENSP00000337443 | NXN | 3.564E-05 | -0.226 | - | - |
| 11274 | ENSP00000315476 | EXOSC4 | 2.267E-05 | -0.226 | - | - |
| 11275 | ENSP00000233099 | HEATR5B | 3.181E-05 | -0.226 | - | - |
| 11276 | ENSP00000247225 | SGPP1 | 7.271E-05 | -0.226 | - | - |
| 11277 | ENSP00000320239 | TRPM7 | 3.945E-05 | -0.226 | - | - |
| 11278 | ENSP00000265062 | RAB7A | 2.360E-05 | -0.226 | - | - |
| 11279 | ENSP00000276461 | ERLIN2 | 3.087E-05 | -0.227 | - | - |
| 11280 | ENSP00000261263 | RAB21 | 4.374E-05 | -0.227 | - | - |
| 11281 | ENSP00000307786 | CYCS | 3.668E-05 | -0.227 | - | - |
| 11282 | ENSP00000282326 | ZSCAN1 | 1.968E-05 | -0.227 | - | - |
| 11283 | ENSP00000216039 | JOSD1 | 3.498E-05 | -0.227 | - | - |
| 11284 | ENSP00000283006 | CENPH | 4.176E-05 | -0.227 | - | - |
| 11285 | ENSP00000258821 | TTC5 | 4.113E-05 | -0.227 | - | - |
| 11286 | ENSP00000359128 | NPM3 | 2.173E-05 | -0.227 | - | - |
| 11287 | ENSP00000262605 | TTPAL | 2.460E-05 | -0.227 | - | - |
| 11288 | ENSP00000379383 | ENAM | 2.695E-05 | -0.227 | - | - |
| 11289 | ENSP00000242257 | FTSJ2 | 7.425E-06 | -0.227 | - | - |
| 11290 | ENSP00000261622 | SLC7A5 | 2.640E-05 | -0.227 | - | - |
| 11291 | ENSP00000297524 | SLC7A13 | 2.886E-05 | -0.227 | - | - |
| 11292 | ENSP00000260327 | CTDSPL2 | 3.188E-05 | -0.227 | - | - |
| 11293 | ENSP00000286175 | PPIL3 | 2.895E-05 | -0.227 | - | - |
| 11294 | ENSP00000261207 | PPP1R12A | 2.095E-05 | -0.227 | - | - |
| 11295 | ENSP00000274054 | NAF1 | 3.590E-05 | -0.227 | - | - |
| 11296 | ENSP00000384286 | C17orf51 | 5.438E-06 | -0.227 | - | - |
| 11297 | ENSP00000415978 | - | 3.030E-06 | -0.227 | - | - |
| 11298 | ENSP00000215567 | TECR | 1.284E-05 | -0.227 | - | - |
| 11299 | ENSP00000242104 | OCM | 1.080E-05 | -0.227 | - | - |
| 11300 | ENSP00000310309 | ABLIM3 | 3.154E-05 | -0.227 | - | - |
| 11301 | ENSP00000296098 | TRIM54 | 4.501E-05 | -0.227 | - | - |
| 11302 | ENSP00000387435 | ARTN | 1.532E-05 | -0.227 | - | - |
| 11303 | ENSP00000320236 | TIMM22 | 2.676E-05 | -0.227 | - | - |
| 11304 | ENSP00000299001 | PIWIL4 | 2.221E-05 | -0.227 | - | - |
| 11305 | ENSP00000330032 | UPP1 | 1.799E-05 | -0.227 | - | - |
| 11306 | ENSP00000395995 | CCDC141 | 1.935E-05 | -0.227 | - | - |
| 11307 | ENSP00000322175 | AK4 | 2.468E-05 | -0.227 | - | - |
| 11308 | ENSP00000433459 | CREBZF | 7.718E-06 | -0.227 | - | - |
| 11309 | ENSP00000281030 | THRSP | 2.171E-05 | -0.227 | - | - |
| 11310 | ENSP00000315410 | OSBPL3 | 1.992E-05 | -0.227 | - | - |
| 11311 | ENSP00000309606 | ZNF408 | 2.238E-05 | -0.227 | - | - |
| 11312 | ENSP00000335333 | PIP5K1C | 1.531E-05 | -0.227 | - | - |
| 11313 | ENSP00000340132 | ZNF383 | 2.212E-05 | -0.227 | - | - |
| 11314 | ENSP00000333358 | RNASE10 | 7.785E-06 | -0.227 | - | - |
| 11315 | ENSP00000219091 | ZNF205 | 1.829E-05 | -0.227 | - | - |
| 11316 | ENSP00000288221 | ERC2 | 3.102E-05 | -0.227 | - | - |
| 11317 | ENSP00000301454 | SLC25A23 | 4.310E-05 | -0.227 | - | - |
| 11318 | ENSP00000323880 | FOXJ1 | 1.678E-05 | -0.227 | - | - |
| 11319 | ENSP00000308258 | PTDSS2 | 1.532E-05 | -0.227 | - | - |
| 11320 | ENSP00000301761 | SDHAF2 | 5.319E-05 | -0.227 | - | - |
| 11321 | ENSP00000331927 | ZNF789 | 1.929E-05 | -0.227 | - | - |
| 11322 | ENSP00000238081 | YWHAQ | 1.968E-05 | -0.227 | - | - |
| 11323 | ENSP00000229266 | CHPT1 | 2.776E-05 | -0.227 | - | - |
| 11324 | ENSP00000307567 | QARS | 1.759E-05 | -0.227 | - | - |
| 11325 | ENSP00000234677 | SARS | 1.292E-05 | -0.227 | - | - |
| 11326 | ENSP00000262288 | SCPEP1 | 3.846E-05 | -0.227 | - | - |
| 11327 | ENSP00000324203 | WRAP53 | 1.152E-05 | -0.227 | - | - |
| 11328 | ENSP00000285737 | LONP2 | 2.770E-05 | -0.227 | - | - |
| 11329 | ENSP00000320940 | NCOA1 | 3.924E-05 | -0.227 | - | - |
| 11330 | ENSP00000328547 | DNMT3B | 4.444E-05 | -0.227 | - | - |
| 11331 | ENSP00000310697 | BRSK2 | 2.265E-05 | -0.227 | - | - |
| 11332 | ENSP00000369150 | C15orf59 | 2.724E-06 | -0.227 | - | - |
| 11333 | ENSP00000332444 | CSTF2T | 1.249E-05 | -0.227 | - | - |
| 11334 | ENSP00000269701 | AKAP8 | 1.874E-04 | -0.227 | - | - |
| 11335 | ENSP00000280756 | C12orf23 | 8.729E-05 | -0.227 | - | - |
| 11336 | ENSP00000254799 | GRSF1 | 3.627E-05 | -0.227 | - | - |
| 11337 | ENSP00000443168 | STH | 2.567E-05 | -0.227 | - | - |
| 11338 | ENSP00000361048 | LURAP1 | 8.225E-06 | -0.227 | - | - |
| 11339 | ENSP00000253031 | YIPF2 | 4.065E-05 | -0.227 | - | - |
| 11340 | ENSP00000251047 | LMAN1 | 2.154E-05 | -0.227 | - | - |
| 11341 | ENSP00000262213 | TRAM1 | 2.295E-05 | -0.227 | - | - |
| 11342 | ENSP00000369915 | NAP1L4 | 1.558E-05 | -0.227 | - | - |
| 11343 | ENSP00000265081 | MSH3 | 1.655E-05 | -0.227 | - | - |
| 11344 | ENSP00000324274 | ZCCHC10 | 2.760E-05 | -0.227 | - | - |
| 11345 | ENSP00000422131 | FAM193B | 9.404E-06 | -0.227 | - | - |
| 11346 | ENSP00000376432 | KCNJ1 | 2.596E-05 | -0.227 | - | - |
| 11347 | ENSP00000315263 | GLTP | 3.990E-05 | -0.227 | - | - |
| 11348 | ENSP00000428646 | FBXO41 | 1.806E-05 | -0.227 | - | - |
| 11349 | ENSP00000263102 | CCDC6 | 7.643E-05 | -0.227 | - | - |
| 11350 | ENSP00000275072 | PM20D2 | 6.269E-05 | -0.227 | - | - |
| 11351 | ENSP00000356548 | LTV1 | 8.211E-06 | -0.227 | - | - |
| 11352 | ENSP00000264893 | 11-Sep | 2.883E-05 | -0.227 | - | - |
| 11353 | ENSP00000254998 | NXT1 | 4.658E-05 | -0.227 | - | - |
| 11354 | ENSP00000220669 | ZFAND1 | 3.084E-05 | -0.227 | - | - |
| 11355 | ENSP00000379602 | VAMP1 | 1.773E-05 | -0.227 | - | - |
| 11356 | ENSP00000301019 | CDT1 | 1.347E-05 | -0.227 | - | - |
| 11357 | ENSP00000410083 | VEZT | 5.266E-06 | -0.227 | - | - |
| 11358 | ENSP00000263960 | COQ10B | 3.404E-05 | -0.227 | - | - |
| 11359 | ENSP00000196551 | RPS5 | 6.625E-06 | -0.227 | - | - |
| 11360 | ENSP00000250916 | KLF16 | 1.973E-05 | -0.227 | - | - |
| 11361 | ENSP00000264436 | ADD2 | 1.167E-05 | -0.227 | - | - |
| 11362 | ENSP00000281701 | NVL | 1.080E-05 | -0.227 | - | - |
| 11363 | ENSP00000320076 | USH1G | 1.683E-05 | -0.227 | - | - |
| 11364 | ENSP00000296792 | UTP15 | 7.916E-06 | -0.227 | - | - |
| 11365 | ENSP00000267512 | RAB15 | 7.320E-06 | -0.227 | - | - |
| 11366 | ENSP00000328854 | NOC4L | 7.025E-06 | -0.227 | - | - |
| 11367 | ENSP00000347507 | MYH7 | 2.446E-05 | -0.227 | - | - |
| 11368 | ENSP00000225388 | NUFIP2 | 5.438E-05 | -0.227 | - | - |
| 11369 | ENSP00000221233 | EXOSC5 | 1.701E-05 | -0.227 | - | - |
| 11370 | ENSP00000329140 | CREB3L2 | 2.615E-05 | -0.227 | - | - |
| 11371 | ENSP00000232978 | NKTR | 2.891E-05 | -0.227 | - | - |
| 11372 | ENSP00000219439 | HSDL1 | 1.235E-05 | -0.227 | - | - |
| 11373 | ENSP00000337736 | AKAP1 | 2.805E-05 | -0.227 | - | - |
| 11374 | ENSP00000255194 | AP3B1 | 1.337E-05 | -0.227 | - | - |
| 11375 | ENSP00000413035 | RBFOX2 | 1.342E-05 | -0.227 | - | - |
| 11376 | ENSP00000261413 | MRPS27 | 4.496E-05 | -0.227 | - | - |
| 11377 | ENSP00000344424 | KIAA0247 | 5.266E-05 | -0.227 | - | - |
| 11378 | ENSP00000312001 | LRRN3 | 4.022E-05 | -0.227 | - | - |
| 11379 | ENSP00000366413 | POU4F1 | 2.355E-05 | -0.228 | - | - |
| 11380 | ENSP00000426234 | SEPHS2 | 1.924E-05 | -0.228 | - | - |
| 11381 | ENSP00000251119 | ZFYVE26 | 1.547E-05 | -0.228 | - | - |
| 11382 | ENSP00000359310 | CSAG1 | 7.064E-04 | -0.228 | - | - |
| 11383 | ENSP00000295971 | RBM47 | 4.507E-05 | -0.228 | - | - |
| 11384 | ENSP00000330320 | SLC9A7 | 1.475E-05 | -0.228 | - | - |
| 11385 | ENSP00000287380 | WDR67 | 5.804E-05 | -0.228 | - | - |
| 11386 | ENSP00000313600 | ATP10B | 9.240E-06 | -0.228 | - | - |
| 11387 | ENSP00000439444 | PPIAL4D | 2.296E-05 | -0.228 | - | - |
| 11388 | ENSP00000305918 | BRD3 | 2.328E-05 | -0.228 | - | - |
| 11389 | ENSP00000218652 | NDFIP2 | 3.281E-05 | -0.228 | - | - |
| 11390 | ENSP00000412315 | GSKIP | 2.049E-05 | -0.228 | - | - |
| 11391 | ENSP00000309565 | UQCRH | 1.862E-05 | -0.228 | - | - |
| 11392 | ENSP00000291458 | CCDC58 | 5.027E-05 | -0.228 | - | - |
| 11393 | ENSP00000361104 | OBP2B | 2.380E-06 | -0.228 | - | - |
| 11394 | ENSP00000300069 | RBPMS2 | 3.419E-05 | -0.228 | - | - |
| 11395 | ENSP00000267064 | SMARCC2 | 2.491E-05 | -0.228 | - | - |
| 11396 | ENSP00000268483 | TXNL4B | 2.277E-05 | -0.228 | - | - |
| 11397 | ENSP00000274134 | ROPN1L | 2.633E-05 | -0.228 | - | - |
| 11398 | ENSP00000316543 | RAPH1 | 3.361E-05 | -0.228 | - | - |
| 11399 | ENSP00000265069 | ZFR | 5.021E-05 | -0.228 | - | - |
| 11400 | ENSP00000340281 | EIF4G2 | 5.759E-05 | -0.228 | - | - |
| 11401 | ENSP00000259667 | HINT2 | 1.128E-05 | -0.228 | - | - |
| 11402 | ENSP00000436376 | OMP | 2.809E-05 | -0.228 | - | - |
| 11403 | ENSP00000367030 | TRDMT1 | 1.674E-05 | -0.228 | - | - |
| 11404 | ENSP00000360475 | ARRDC1 | 1.872E-05 | -0.228 | - | - |
| 11405 | ENSP00000266594 | ANP32D | 1.144E-04 | -0.228 | - | - |
| 11406 | ENSP00000268184 | CRTC3 | 2.760E-05 | -0.228 | - | - |
| 11407 | ENSP00000258873 | ACSBG1 | 1.803E-05 | -0.228 | - | - |
| 11408 | ENSP00000250454 | EAPP | 6.443E-05 | -0.228 | - | - |
| 11409 | ENSP00000296859 | RAPGEF6 | 8.893E-05 | -0.228 | - | - |
| 11410 | ENSP00000326534 | VPS39 | 8.923E-06 | -0.228 | - | - |
| 11411 | ENSP00000355637 | ABCB10 | 1.784E-05 | -0.228 | - | - |
| 11412 | ENSP00000307078 | KIF5B | 2.255E-05 | -0.228 | - | - |
| 11413 | ENSP00000311135 | DHX37 | 6.217E-06 | -0.228 | - | - |
| 11414 | ENSP00000336740 | LIMK1 | 1.777E-05 | -0.228 | - | - |
| 11415 | ENSP00000309790 | MIS18BP1 | 5.298E-05 | -0.228 | - | - |
| 11416 | ENSP00000310015 | ADAT1 | 5.043E-05 | -0.228 | - | - |
| 11417 | ENSP00000284629 | ASZ1 | 8.539E-06 | -0.228 | - | - |
| 11418 | ENSP00000263181 | KIF18A | 8.186E-06 | -0.228 | - | - |
| 11419 | ENSP00000343552 | ICMT | 2.000E-05 | -0.228 | - | - |
| 11420 | ENSP00000298068 | HECTD2 | 2.271E-05 | -0.228 | - | - |
| 11421 | ENSP00000331268 | ANKRD45 | 2.486E-05 | -0.228 | - | - |
| 11422 | ENSP00000257017 | RAB33A | 8.263E-06 | -0.228 | - | - |
| 11423 | ENSP00000260985 | IDH1 | 1.945E-05 | -0.228 | - | - |
| 11424 | ENSP00000471625 | AC003005.4 | 1.250E-05 | -0.228 | - | - |
| 11425 | ENSP00000263805 | ZNF106 | 2.608E-05 | -0.228 | - | - |
| 11426 | ENSP00000332576 | OSBP2 | 1.727E-05 | -0.228 | - | - |
| 11427 | ENSP00000283131 | SMARCA5 | 1.327E-05 | -0.228 | - | - |
| 11428 | ENSP00000265854 | MAD1L1 | 1.530E-05 | -0.228 | - | - |
| 11429 | ENSP00000377892 | SRSF5 | 1.783E-05 | -0.228 | - | - |
| 11430 | ENSP00000297157 | RP9 | 1.826E-05 | -0.228 | - | - |
| 11431 | ENSP00000427211 | APBB2 | 1.913E-05 | -0.228 | - | - |
| 11432 | ENSP00000339449 | WDR16 | 2.051E-05 | -0.228 | - | - |
| 11433 | ENSP00000343282 | HIST1H4D | 1.824E-05 | -0.228 | - | - |
| 11434 | ENSP00000349401 | ZNF708 | 1.309E-05 | -0.228 | - | - |
| 11435 | ENSP00000316649 | OSBPL2 | 3.121E-05 | -0.228 | - | - |
| 11436 | ENSP00000382342 | ABCC1 | 2.276E-05 | -0.228 | - | - |
| 11437 | ENSP00000290650 | UBR1 | 1.306E-05 | -0.228 | - | - |
| 11438 | ENSP00000215587 | POLR2E | 7.596E-06 | -0.228 | - | - |
| 11439 | ENSP00000364912 | SPEN | 2.379E-05 | -0.228 | - | - |
| 11440 | ENSP00000403975 | ZNF668 | 1.117E-05 | -0.228 | - | - |
| 11441 | ENSP00000235349 | PRAMEF4 | 6.665E-05 | -0.228 | - | - |
| 11442 | ENSP00000075120 | SLC2A3 | 2.247E-05 | -0.228 | - | - |
| 11443 | ENSP00000292579 | ZNF250 | 2.338E-05 | -0.228 | - | - |
| 11444 | ENSP00000259239 | IMP4 | 5.585E-06 | -0.228 | - | - |
| 11445 | ENSP00000292433 | IER2 | 2.332E-04 | -0.228 | - | - |
| 11446 | ENSP00000324628 | NAPG | 2.787E-05 | -0.228 | - | - |
| 11447 | ENSP00000353904 | CSNK1G3 | 1.521E-05 | -0.228 | - | - |
| 11448 | ENSP00000283415 | LPCAT1 | 2.189E-05 | -0.228 | - | - |
| 11449 | ENSP00000266688 | PTPRQ | 1.956E-05 | -0.228 | - | - |
| 11450 | ENSP00000338258 | CDC42EP4 | 6.797E-05 | -0.228 | - | - |
| 11451 | ENSP00000320378 | SLC7A8 | 1.305E-05 | -0.228 | - | - |
| 11452 | ENSP00000263817 | ABCB11 | 1.849E-05 | -0.228 | - | - |
| 11453 | ENSP00000238855 | AFTPH | 2.637E-05 | -0.228 | - | - |
| 11454 | ENSP00000301587 | ATP5H | 7.578E-06 | -0.228 | - | - |
| 11455 | ENSP00000314491 | SRRT | 3.868E-05 | -0.228 | - | - |
| 11456 | ENSP00000283179 | HNRNPU | 2.947E-05 | -0.228 | - | - |
| 11457 | ENSP00000245544 | NUP85 | 1.522E-05 | -0.228 | - | - |
| 11458 | ENSP00000358590 | ZNF292 | 1.146E-05 | -0.228 | - | - |
| 11459 | ENSP00000267287 | MBNL2 | 2.589E-05 | -0.228 | - | - |
| 11460 | ENSP00000383515 | AC002321.1 | 1.490E-06 | -0.228 | - | - |
| 11461 | ENSP00000285873 | GEMIN5 | 5.632E-05 | -0.228 | - | - |
| 11462 | ENSP00000390475 | CXXC1 | 1.892E-05 | -0.228 | - | - |
| 11463 | ENSP00000375220 | CTAGE1 | 2.124E-05 | -0.228 | - | - |
| 11464 | ENSP00000308318 | PPP1R3B | 2.320E-05 | -0.228 | - | - |
| 11465 | ENSP00000272647 | AMMECR1L | 4.154E-05 | -0.228 | - | - |
| 11466 | ENSP00000268607 | MAP1LC3B | 3.063E-05 | -0.228 | - | - |
| 11467 | ENSP00000263753 | SGOL1 | 2.662E-05 | -0.228 | - | - |
| 11468 | ENSP00000254480 | SMARCC1 | 2.424E-05 | -0.229 | - | - |
| 11469 | ENSP00000243253 | SEC61A1 | 1.320E-05 | -0.229 | - | - |
| 11470 | ENSP00000266069 | GID8 | 4.837E-05 | -0.229 | - | - |
| 11471 | ENSP00000268261 | PMM2 | 1.533E-05 | -0.229 | - | - |
| 11472 | ENSP00000465578 | ZNF529 | 1.577E-05 | -0.229 | - | - |
| 11473 | ENSP00000229329 | CMAS | 1.238E-05 | -0.229 | - | - |
| 11474 | ENSP00000322341 | PCBP4 | 1.880E-05 | -0.229 | - | - |
| 11475 | ENSP00000303942 | GP9 | 2.617E-05 | -0.229 | - | - |
| 11476 | ENSP00000281031 | NDUFC2 | 1.948E-05 | -0.229 | - | - |
| 11477 | ENSP00000264431 | RAPGEF2 | 5.065E-05 | -0.229 | - | - |
| 11478 | ENSP00000335083 | PPP2R2C | 2.233E-05 | -0.229 | - | - |
| 11479 | ENSP00000269383 | TRIM65 | 2.189E-04 | -0.229 | - | - |
| 11480 | ENSP00000216034 | TOMM22 | 1.994E-05 | -0.229 | - | - |
| 11481 | ENSP00000293471 | ZNF613 | 2.249E-05 | -0.229 | - | - |
| 11482 | ENSP00000457522 | STX16-NPEPL1 | 4.821E-06 | -0.229 | - | - |
| 11483 | ENSP00000354900 | GJB1 | 2.690E-05 | -0.229 | - | - |
| 11484 | ENSP00000261842 | AP4E1 | 1.780E-05 | -0.229 | - | - |
| 11485 | ENSP00000278829 | FADS3 | 3.732E-05 | -0.229 | - | - |
| 11486 | ENSP00000470694 | MGC4294 | 3.578E-07 | -0.229 | - | - |
| 11487 | ENSP00000293414 | ASB16 | 1.115E-05 | -0.229 | - | - |
| 11488 | ENSP00000273179 | CTDSPL | 2.462E-05 | -0.229 | - | - |
| 11489 | ENSP00000037243 | GABARAPL2 | 2.278E-05 | -0.229 | - | - |
| 11490 | ENSP00000269856 | FEM1A | 1.704E-05 | -0.229 | - | - |
| 11491 | ENSP00000282003 | RNF219 | 1.786E-04 | -0.229 | - | - |
| 11492 | ENSP00000261182 | NAP1L1 | 1.875E-05 | -0.229 | - | - |
| 11493 | ENSP00000337056 | CDKN2D | 1.693E-05 | -0.229 | - | - |
| 11494 | ENSP00000278935 | CEP164 | 1.543E-05 | -0.229 | - | - |
| 11495 | ENSP00000255858 | SEC14L4 | 1.848E-05 | -0.229 | - | - |
| 11496 | ENSP00000239878 | UFM1 | 5.949E-05 | -0.229 | - | - |
| 11497 | ENSP00000310649 | BRSK1 | 2.333E-05 | -0.229 | - | - |
| 11498 | ENSP00000341874 | RIOK3 | 2.368E-05 | -0.229 | - | - |
| 11499 | ENSP00000397911 | SP140L | 1.945E-05 | -0.229 | - | - |
| 11500 | ENSP00000338030 | STARD7 | 1.582E-05 | -0.229 | - | - |
| 11501 | ENSP00000272348 | SNRPG | 1.134E-05 | -0.229 | - | - |
| 11502 | ENSP00000225873 | PEX12 | 3.248E-05 | -0.229 | - | - |
| 11503 | ENSP00000306528 | DESI2 | 2.164E-05 | -0.229 | - | - |
| 11504 | ENSP00000308741 | CLOCK | 4.085E-05 | -0.229 | - | - |
| 11505 | ENSP00000316042 | HNRNPA0 | 4.767E-05 | -0.229 | - | - |
| 11506 | ENSP00000003084 | CFTR | 1.884E-05 | -0.229 | - | - |
| 11507 | ENSP00000253339 | LATS1 | 1.340E-05 | -0.229 | - | - |
| 11508 | ENSP00000354251 | NCKAP1 | 2.367E-05 | -0.229 | - | - |
| 11509 | ENSP00000335808 | FAM192A | 9.038E-05 | -0.229 | - | - |
| 11510 | ENSP00000311847 | CARHSP1 | 1.102E-05 | -0.229 | - | - |
| 11511 | ENSP00000328671 | WDR4 | 1.358E-05 | -0.229 | - | - |
| 11512 | ENSP00000469964 | AC007461.1 | 5.684E-07 | -0.229 | - | - |
| 11513 | ENSP00000265713 | KAT6A | 2.059E-05 | -0.229 | - | - |
| 11514 | ENSP00000203001 | TRMT6 | 7.301E-06 | -0.229 | - | - |
| 11515 | ENSP00000264758 | ADD1 | 3.024E-05 | -0.229 | - | - |
| 11516 | ENSP00000353863 | ZNF148 | 1.965E-05 | -0.229 | - | - |
| 11517 | ENSP00000338788 | ZC3H15 | 9.415E-06 | -0.229 | - | - |
| 11518 | ENSP00000263377 | BRD4 | 2.284E-05 | -0.229 | - | - |
| 11519 | ENSP00000387170 | GIGYF2 | 9.971E-06 | -0.229 | - | - |
| 11520 | ENSP00000003302 | USP28 | 3.221E-05 | -0.229 | - | - |
| 11521 | ENSP00000332756 | ATP11C | 2.321E-05 | -0.229 | - | - |
| 11522 | ENSP00000278856 | WDR74 | 6.750E-06 | -0.229 | - | - |
| 11523 | ENSP00000285420 | OTUD6B | 2.031E-05 | -0.229 | - | - |
| 11524 | ENSP00000272091 | SDE2 | 2.474E-05 | -0.229 | - | - |
| 11525 | ENSP00000361310 | POLH | 1.998E-05 | -0.229 | - | - |
| 11526 | ENSP00000269740 | SLC39A3 | 1.048E-05 | -0.229 | - | - |
| 11527 | ENSP00000364699 | SDHD | 2.358E-05 | -0.229 | - | - |
| 11528 | ENSP00000383333 | MORC3 | 7.093E-06 | -0.229 | - | - |
| 11529 | ENSP00000264639 | PSMD3 | 3.200E-05 | -0.229 | - | - |
| 11530 | ENSP00000317300 | LPCAT4 | 2.561E-05 | -0.229 | - | - |
| 11531 | ENSP00000262225 | TMED2 | 2.228E-05 | -0.229 | - | - |
| 11532 | ENSP00000232905 | EIF1B | 2.512E-05 | -0.229 | - | - |
| 11533 | ENSP00000313756 | GPR75-ASB3 | 1.875E-05 | -0.229 | - | - |
| 11534 | ENSP00000265395 | HIBADH | 2.106E-05 | -0.229 | - | - |
| 11535 | ENSP00000250113 | FXR2 | 2.481E-05 | -0.229 | - | - |
| 11536 | ENSP00000252599 | COLGALT1 | 2.377E-04 | -0.229 | - | - |
| 11537 | ENSP00000293981 | FLYWCH2 | 1.047E-04 | -0.229 | - | - |
| 11538 | ENSP00000325296 | PKD2L1 | 2.308E-05 | -0.229 | - | - |
| 11539 | ENSP00000265028 | DNAJB11 | 1.967E-05 | -0.229 | - | - |
| 11540 | ENSP00000448051 | DAZAP2 | 9.329E-06 | -0.229 | - | - |
| 11541 | ENSP00000310668 | NUP93 | 3.239E-05 | -0.230 | - | - |
| 11542 | ENSP00000263620 | ARID3A | 3.202E-05 | -0.230 | - | - |
| 11543 | ENSP00000326563 | KDM3B | 8.761E-05 | -0.230 | - | - |
| 11544 | ENSP00000364502 | TMEM255B | 1.350E-05 | -0.230 | - | - |
| 11545 | ENSP00000288368 | PREX2 | 1.797E-05 | -0.230 | - | - |
| 11546 | ENSP00000293760 | LEMD2 | 3.252E-05 | -0.230 | - | - |
| 11547 | ENSP00000229390 | SRSF9 | 1.916E-05 | -0.230 | - | - |
| 11548 | ENSP00000238647 | IRF2BPL | 8.136E-05 | -0.230 | - | - |
| 11549 | ENSP00000328524 | DENND5A | 2.728E-05 | -0.230 | - | - |
| 11550 | ENSP00000362424 | TRAF3IP1 | 2.572E-05 | -0.230 | - | - |
| 11551 | ENSP00000261353 | YPEL5 | 1.750E-05 | -0.230 | - | - |
| 11552 | ENSP00000023897 | GABRA1 | 1.154E-05 | -0.230 | - | - |
| 11553 | ENSP00000466214 | PDCD5 | 1.437E-05 | -0.230 | - | - |
| 11554 | ENSP00000301459 | RCOR2 | 2.169E-05 | -0.230 | - | - |
| 11555 | ENSP00000321997 | LARP1B | 1.453E-05 | -0.230 | - | - |
| 11556 | ENSP00000254854 | GUCY2D | 6.746E-06 | -0.230 | - | - |
| 11557 | ENSP00000308753 | TMOD3 | 5.359E-05 | -0.230 | - | - |
| 11558 | ENSP00000297857 | ZHX1 | 8.692E-05 | -0.230 | - | - |
| 11559 | ENSP00000397843 | TMA7 | 1.827E-05 | -0.230 | - | - |
| 11560 | ENSP00000367655 | PKHD1L1 | 5.354E-06 | -0.230 | - | - |
| 11561 | ENSP00000394842 | GCA | 3.077E-05 | -0.230 | - | - |
| 11562 | ENSP00000254521 | HSD17B7 | 1.323E-05 | -0.230 | - | - |
| 11563 | ENSP00000240316 | COIL | 2.702E-05 | -0.230 | - | - |
| 11564 | ENSP00000216277 | PAPOLA | 1.934E-05 | -0.230 | - | - |
| 11565 | ENSP00000297183 | ANKHD1 | 1.338E-05 | -0.230 | - | - |
| 11566 | ENSP00000329748 | CPNE8 | 3.850E-05 | -0.230 | - | - |
| 11567 | ENSP00000421985 | FNIP1 | 2.572E-05 | -0.230 | - | - |
| 11568 | ENSP00000370377 | PITRM1 | 2.503E-05 | -0.230 | - | - |
| 11569 | ENSP00000263228 | UBE2R2 | 1.118E-05 | -0.230 | - | - |
| 11570 | ENSP00000399475 | JMJD8 | 3.747E-06 | -0.230 | - | - |
| 11571 | ENSP00000262455 | ERP44 | 1.259E-05 | -0.230 | - | - |
| 11572 | ENSP00000296518 | GUCY1A3 | 2.436E-05 | -0.230 | - | - |
| 11573 | ENSP00000338082 | HBG2 | 2.642E-05 | -0.230 | - | - |
| 11574 | ENSP00000336591 | PPP2R2B | 3.624E-05 | -0.230 | - | - |
| 11575 | ENSP00000351310 | COL6A6 | 2.852E-05 | -0.230 | - | - |
| 11576 | ENSP00000347733 | TRRAP | 2.454E-05 | -0.230 | - | - |
| 11577 | ENSP00000305790 | SF3B3 | 1.133E-05 | -0.230 | - | - |
| 11578 | ENSP00000304769 | ZNF467 | 2.043E-05 | -0.230 | - | - |
| 11579 | ENSP00000440638 | TMEM216 | 1.140E-05 | -0.230 | - | - |
| 11580 | ENSP00000364240 | UBXN10 | 3.072E-06 | -0.230 | - | - |
| 11581 | ENSP00000267436 | L2HGDH | 1.456E-05 | -0.230 | - | - |
| 11582 | ENSP00000392147 | PPP6C | 1.295E-05 | -0.230 | - | - |
| 11583 | ENSP00000373215 | CADPS | 1.388E-05 | -0.230 | - | - |
| 11584 | ENSP00000353942 | CCDC135 | 2.496E-05 | -0.230 | - | - |
| 11585 | ENSP00000248070 | EPS15L1 | 1.984E-05 | -0.230 | - | - |
| 11586 | ENSP00000319974 | BBX | 4.911E-05 | -0.230 | - | - |
| 11587 | ENSP00000329189 | SETD4 | 1.033E-05 | -0.230 | - | - |
| 11588 | ENSP00000245816 | CLPP | 1.921E-05 | -0.230 | - | - |
| 11589 | ENSP00000299192 | HEATR3 | 7.320E-05 | -0.230 | - | - |
| 11590 | ENSP00000253039 | EIF2S3 | 2.396E-05 | -0.230 | - | - |
| 11591 | ENSP00000261965 | TUBGCP3 | 2.357E-05 | -0.230 | - | - |
| 11592 | ENSP00000265605 | ALDH8A1 | 9.096E-06 | -0.230 | - | - |
| 11593 | ENSP00000325203 | OR6T1 | 1.214E-05 | -0.230 | - | - |
| 11594 | ENSP00000347532 | KIAA1598 | 1.844E-05 | -0.230 | - | - |
| 11595 | ENSP00000293677 | RAVER1 | 4.633E-05 | -0.230 | - | - |
| 11596 | ENSP00000291572 | AGPAT3 | 1.030E-05 | -0.230 | - | - |
| 11597 | ENSP00000447057 | C12orf45 | 3.982E-06 | -0.230 | - | - |
| 11598 | ENSP00000432172 | CCDC17 | 8.332E-06 | -0.230 | - | - |
| 11599 | ENSP00000300935 | RAB8A | 1.970E-05 | -0.230 | - | - |
| 11600 | ENSP00000304370 | CDC40 | 1.554E-05 | -0.230 | - | - |
| 11601 | ENSP00000334785 | TESC | 1.588E-05 | -0.230 | - | - |
| 11602 | ENSP00000339090 | RBMX2 | 3.181E-05 | -0.230 | - | - |
| 11603 | ENSP00000215375 | ATP5D | 1.260E-05 | -0.230 | - | - |
| 11604 | ENSP00000326238 | CAPZA3 | 9.688E-06 | -0.230 | - | - |
| 11605 | ENSP00000285093 | ACAA2 | 1.152E-05 | -0.230 | - | - |
| 11606 | ENSP00000263736 | SRBD1 | 2.320E-05 | -0.230 | - | - |
| 11607 | ENSP00000357637 | EBF3 | 1.895E-05 | -0.230 | - | - |
| 11608 | ENSP00000361562 | CTSA | 3.885E-05 | -0.230 | - | - |
| 11609 | ENSP00000267328 | RAB20 | 9.608E-06 | -0.230 | - | - |
| 11610 | ENSP00000332706 | PURA | 3.876E-05 | -0.230 | - | - |
| 11611 | ENSP00000256257 | RNF122 | 3.096E-05 | -0.230 | - | - |
| 11612 | ENSP00000320821 | KIF1C | 1.206E-05 | -0.230 | - | - |
| 11613 | ENSP00000343629 | PRRC2C | 2.943E-05 | -0.230 | - | - |
| 11614 | ENSP00000344166 | TMEM189-UBE2V1 | 3.457E-05 | -0.230 | - | - |
| 11615 | ENSP00000226796 | GAR1 | 1.174E-05 | -0.230 | - | - |
| 11616 | ENSP00000325376 | HNRNPM | 3.160E-05 | -0.230 | - | - |
| 11617 | ENSP00000420465 | RNF182 | 2.168E-05 | -0.230 | - | - |
| 11618 | ENSP00000338703 | UGP2 | 1.883E-05 | -0.230 | - | - |
| 11619 | ENSP00000342411 | NRN1L | 1.075E-05 | -0.230 | - | - |
| 11620 | ENSP00000288022 | PDF | 2.039E-05 | -0.230 | - | - |
| 11621 | ENSP00000215941 | ANKRD54 | 1.073E-05 | -0.230 | - | - |
| 11622 | ENSP00000269095 | MPP2 | 1.783E-05 | -0.231 | - | - |
| 11623 | ENSP00000204517 | TFAP4 | 3.297E-05 | -0.231 | - | - |
| 11624 | ENSP00000306163 | ANKRD2 | 1.251E-05 | -0.231 | - | - |
| 11625 | ENSP00000308548 | ZNHIT2 | 1.457E-05 | -0.231 | - | - |
| 11626 | ENSP00000250784 | RPS4Y1 | 1.082E-05 | -0.231 | - | - |
| 11627 | ENSP00000352248 | ANXA10 | 1.066E-05 | -0.231 | - | - |
| 11628 | ENSP00000287647 | FANCD2 | 3.297E-05 | -0.231 | - | - |
| 11629 | ENSP00000343001 | UBE4B | 3.620E-05 | -0.231 | - | - |
| 11630 | ENSP00000407107 | HHLA1 | 2.712E-05 | -0.231 | - | - |
| 11631 | ENSP00000265990 | BTAF1 | 2.485E-05 | -0.231 | - | - |
| 11632 | ENSP00000175506 | ASNS | 2.138E-05 | -0.231 | - | - |
| 11633 | ENSP00000244763 | SSR1 | 3.323E-05 | -0.231 | - | - |
| 11634 | ENSP00000327916 | ACSM5 | 8.604E-06 | -0.231 | - | - |
| 11635 | ENSP00000335493 | FOXD2 | 9.232E-06 | -0.231 | - | - |
| 11636 | ENSP00000270625 | RPS11 | 8.507E-06 | -0.231 | - | - |
| 11637 | ENSP00000003100 | CYP51A1 | 2.775E-05 | -0.231 | - | - |
| 11638 | ENSP00000321449 | RRP7A | 8.747E-06 | -0.231 | - | - |
| 11639 | ENSP00000290354 | CBR3 | 1.624E-05 | -0.231 | - | - |
| 11640 | ENSP00000304198 | MBD3L1 | 1.892E-05 | -0.231 | - | - |
| 11641 | ENSP00000274140 | 6-Mar | 2.823E-05 | -0.231 | - | - |
| 11642 | ENSP00000293201 | MYO15B | 3.861E-05 | -0.231 | - | - |
| 11643 | ENSP00000326531 | THOC6 | 6.223E-05 | -0.231 | - | - |
| 11644 | ENSP00000333775 | XAGE2 | 1.150E-05 | -0.231 | - | - |
| 11645 | ENSP00000240499 | ZNF141 | 2.227E-05 | -0.231 | - | - |
| 11646 | ENSP00000459566 | ZNF75A | 8.451E-06 | -0.231 | - | - |
| 11647 | ENSP00000233078 | DAZAP1 | 2.958E-05 | -0.231 | - | - |
| 11648 | ENSP00000340879 | RAD1 | 2.245E-05 | -0.231 | - | - |
| 11649 | ENSP00000228318 | SLC25A3 | 1.611E-05 | -0.231 | - | - |
| 11650 | ENSP00000312042 | HDLBP | 1.934E-05 | -0.231 | - | - |
| 11651 | ENSP00000260129 | TGS1 | 1.405E-05 | -0.231 | - | - |
| 11652 | ENSP00000351593 | SMTN | 2.681E-05 | -0.231 | - | - |
| 11653 | ENSP00000321259 | TALDO1 | 1.230E-05 | -0.231 | - | - |
| 11654 | ENSP00000429803 | ZNF260 | 1.497E-05 | -0.231 | - | - |
| 11655 | ENSP00000221855 | TBCB | 3.153E-05 | -0.231 | - | - |
| 11656 | ENSP00000383226 | KRTAP10-1 | 1.457E-05 | -0.231 | - | - |
| 11657 | ENSP00000272930 | UBE2F | 6.580E-05 | -0.231 | - | - |
| 11658 | ENSP00000391311 | 5-Sep | 4.124E-05 | -0.231 | - | - |
| 11659 | ENSP00000362123 | PLA2G12B | 1.459E-05 | -0.231 | - | - |
| 11660 | ENSP00000252211 | ZKSCAN3 | 2.255E-05 | -0.231 | - | - |
| 11661 | ENSP00000252603 | PGLS | 9.205E-06 | -0.231 | - | - |
| 11662 | ENSP00000244520 | SNRPC | 1.762E-05 | -0.231 | - | - |
| 11663 | ENSP00000286523 | ELMSAN1 | 4.917E-05 | -0.231 | - | - |
| 11664 | ENSP00000260600 | ADCY3 | 1.773E-05 | -0.231 | - | - |
| 11665 | ENSP00000337839 | ZCCHC7 | 3.846E-05 | -0.231 | - | - |
| 11666 | ENSP00000284116 | GDPD1 | 1.412E-05 | -0.231 | - | - |
| 11667 | ENSP00000410734 | ZNF562 | 1.062E-05 | -0.231 | - | - |
| 11668 | ENSP00000326261 | SRRM1 | 2.915E-05 | -0.231 | - | - |
| 11669 | ENSP00000341285 | HNRNPA1L2 | 2.582E-05 | -0.231 | - | - |
| 11670 | ENSP00000427463 | SPCS3 | 1.007E-05 | -0.231 | - | - |
| 11671 | ENSP00000303525 | CBR4 | 1.471E-05 | -0.231 | - | - |
| 11672 | ENSP00000282903 | PLOD2 | 1.727E-05 | -0.231 | - | - |
| 11673 | ENSP00000367869 | - | 4.876E-05 | -0.231 | - | - |
| 11674 | ENSP00000341483 | RANBP3 | 3.604E-05 | -0.231 | - | - |
| 11675 | ENSP00000234111 | ODC1 | 2.170E-05 | -0.231 | - | - |
| 11676 | ENSP00000338629 | RERE | 4.028E-05 | -0.231 | - | - |
| 11677 | ENSP00000262525 | ZNF629 | 2.029E-05 | -0.231 | - | - |
| 11678 | ENSP00000232888 | RRP9 | 5.435E-06 | -0.231 | - | - |
| 11679 | ENSP00000350541 | AQPEP | 2.293E-05 | -0.231 | - | - |
| 11680 | ENSP00000282841 | GGPS1 | 1.418E-05 | -0.231 | - | - |
| 11681 | ENSP00000250863 | DAZL | 1.420E-05 | -0.231 | - | - |
| 11682 | ENSP00000274457 | FEM1C | 1.462E-05 | -0.231 | - | - |
| 11683 | ENSP00000355377 | AKR7A3 | 2.006E-05 | -0.231 | - | - |
| 11684 | ENSP00000417573 | YIPF6 | 2.156E-05 | -0.231 | - | - |
| 11685 | ENSP00000310205 | NRIP3 | 2.679E-05 | -0.231 | - | - |
| 11686 | ENSP00000380876 | RGS11 | 1.669E-05 | -0.231 | - | - |
| 11687 | ENSP00000430237 | RNF130 | 1.738E-05 | -0.231 | - | - |
| 11688 | ENSP00000310551 | LCLAT1 | 1.484E-05 | -0.231 | - | - |
| 11689 | ENSP00000366604 | SF1 | 2.533E-05 | -0.231 | - | - |
| 11690 | ENSP00000373952 | FANCA | 3.027E-05 | -0.231 | - | - |
| 11691 | ENSP00000348634 | MYH6 | 2.757E-05 | -0.231 | - | - |
| 11692 | ENSP00000384296 | ACY1 | 2.870E-05 | -0.231 | - | - |
| 11693 | ENSP00000323678 | ZADH2 | 3.128E-05 | -0.231 | - | - |
| 11694 | ENSP00000314193 | WDR75 | 8.326E-06 | -0.231 | - | - |
| 11695 | ENSP00000196489 | ZNF416 | 1.944E-05 | -0.231 | - | - |
| 11696 | ENSP00000282059 | C18orf25 | 6.645E-05 | -0.231 | - | - |
| 11697 | ENSP00000393292 | TOR1AIP1 | 8.818E-06 | -0.231 | - | - |
| 11698 | ENSP00000297071 | TRA2A | 4.331E-05 | -0.231 | - | - |
| 11699 | ENSP00000220584 | FDFT1 | 1.126E-05 | -0.231 | - | - |
| 11700 | ENSP00000320309 | FARSA | 1.522E-05 | -0.231 | - | - |
| 11701 | ENSP00000297540 | PHAX | 3.287E-05 | -0.231 | - | - |
| 11702 | ENSP00000389128 | LRRC15 | 1.313E-05 | -0.231 | - | - |
| 11703 | ENSP00000001146 | CYP26B1 | 3.204E-05 | -0.231 | - | - |
| 11704 | ENSP00000335592 | GABRA5 | 1.407E-05 | -0.231 | - | - |
| 11705 | ENSP00000224807 | SFXN3 | 5.171E-05 | -0.231 | - | - |
| 11706 | ENSP00000301180 | DIP2B | 3.017E-05 | -0.231 | - | - |
| 11707 | ENSP00000262710 | ACIN1 | 4.192E-05 | -0.231 | - | - |
| 11708 | ENSP00000324020 | KIF15 | 7.888E-06 | -0.231 | - | - |
| 11709 | ENSP00000320516 | EHD1 | 1.573E-05 | -0.231 | - | - |
| 11710 | ENSP00000367828 | ITM2B | 2.969E-05 | -0.232 | - | - |
| 11711 | ENSP00000457423 | ZNF625 | 9.465E-06 | -0.232 | - | - |
| 11712 | ENSP00000389760 | GATS | 1.201E-05 | -0.232 | - | - |
| 11713 | ENSP00000342021 | ZNF788 | 1.618E-05 | -0.232 | - | - |
| 11714 | ENSP00000262383 | ZNF423 | 1.940E-05 | -0.232 | - | - |
| 11715 | ENSP00000414516 | SS18 | 9.989E-06 | -0.232 | - | - |
| 11716 | ENSP00000373772 | RNF20 | 1.362E-05 | -0.232 | - | - |
| 11717 | ENSP00000320503 | EDC3 | 3.173E-05 | -0.232 | - | - |
| 11718 | ENSP00000375095 | TAS2R13 | 2.241E-05 | -0.232 | - | - |
| 11719 | ENSP00000270649 | ZNF614 | 1.881E-05 | -0.232 | - | - |
| 11720 | ENSP00000257818 | LMO2 | 4.827E-05 | -0.232 | - | - |
| 11721 | ENSP00000309334 | RPL15 | 8.285E-06 | -0.232 | - | - |
| 11722 | ENSP00000298248 | CRYL1 | 2.003E-05 | -0.232 | - | - |
| 11723 | ENSP00000007699 | YBX2 | 2.168E-05 | -0.232 | - | - |
| 11724 | ENSP00000298307 | KLHDC2 | 9.325E-06 | -0.232 | - | - |
| 11725 | ENSP00000378360 | DAK | 1.949E-05 | -0.232 | - | - |
| 11726 | ENSP00000246229 | PLAGL2 | 1.934E-05 | -0.232 | - | - |
| 11727 | ENSP00000386200 | FOXP2 | 3.601E-05 | -0.232 | - | - |
| 11728 | ENSP00000374069 | PTPRN2 | 2.451E-05 | -0.232 | - | - |
| 11729 | ENSP00000253382 | ACSS2 | 7.356E-06 | -0.232 | - | - |
| 11730 | ENSP00000303777 | TEX13B | 1.553E-05 | -0.232 | - | - |
| 11731 | ENSP00000256951 | EMP1 | 2.452E-05 | -0.232 | - | - |
| 11732 | ENSP00000461460 | GOSR2 | 1.810E-05 | -0.232 | - | - |
| 11733 | ENSP00000364794 | IARS | 2.787E-05 | -0.232 | - | - |
| 11734 | ENSP00000321951 | FAIM2 | 1.223E-05 | -0.232 | - | - |
| 11735 | ENSP00000216259 | PMM1 | 1.484E-05 | -0.232 | - | - |
| 11736 | ENSP00000358151 | HIST2H2BE | 2.339E-05 | -0.232 | - | - |
| 11737 | ENSP00000313829 | KHDRBS1 | 2.948E-05 | -0.232 | - | - |
| 11738 | ENSP00000196482 | ZNF324 | 1.998E-05 | -0.232 | - | - |
| 11739 | ENSP00000298875 | CPSF2 | 2.238E-05 | -0.232 | - | - |
| 11740 | ENSP00000359837 | ZZZ3 | 1.136E-05 | -0.232 | - | - |
| 11741 | ENSP00000327538 | ZNF534 | 2.248E-05 | -0.232 | - | - |
| 11742 | ENSP00000320885 | SPAST | 3.111E-05 | -0.232 | - | - |
| 11743 | ENSP00000276123 | ZNF711 | 2.240E-05 | -0.232 | - | - |
| 11744 | ENSP00000340914 | SYT3 | 2.755E-05 | -0.232 | - | - |
| 11745 | ENSP00000365550 | GKAP1 | 2.078E-05 | -0.232 | - | - |
| 11746 | ENSP00000265970 | PIK3C2A | 1.865E-05 | -0.232 | - | - |
| 11747 | ENSP00000281455 | ACSL1 | 1.967E-05 | -0.232 | - | - |
| 11748 | ENSP00000216410 | GNPNAT1 | 1.217E-05 | -0.232 | - | - |
| 11749 | ENSP00000249356 | DNAJB9 | 1.087E-05 | -0.232 | - | - |
| 11750 | ENSP00000318423 | LINS | 3.367E-05 | -0.232 | - | - |
| 11751 | ENSP00000244040 | RAB22A | 1.327E-05 | -0.232 | - | - |
| 11752 | ENSP00000466121 | C19orf40 | 2.800E-05 | -0.232 | - | - |
| 11753 | ENSP00000283109 | RIOK2 | 9.936E-06 | -0.232 | - | - |
| 11754 | ENSP00000290649 | AMFR | 2.120E-05 | -0.232 | - | - |
| 11755 | ENSP00000391067 | ZNF302 | 1.013E-05 | -0.232 | - | - |
| 11756 | ENSP00000289416 | ACSM3 | 1.069E-05 | -0.232 | - | - |
| 11757 | ENSP00000368645 | POLR2J3 | 1.311E-05 | -0.232 | - | - |
| 11758 | ENSP00000287713 | NMNAT2 | 1.053E-05 | -0.232 | - | - |
| 11759 | ENSP00000371394 | SYT8 | 2.142E-05 | -0.232 | - | - |
| 11760 | ENSP00000359478 | ABCC2 | 2.180E-05 | -0.232 | - | - |
| 11761 | ENSP00000406220 | SLC13A5 | 1.982E-05 | -0.232 | - | - |
| 11762 | ENSP00000225969 | MRPL27 | 3.809E-06 | -0.232 | - | - |
| 11763 | ENSP00000244020 | SRSF6 | 1.217E-05 | -0.232 | - | - |
| 11764 | ENSP00000361949 | PABPC4 | 1.569E-05 | -0.232 | - | - |
| 11765 | ENSP00000394791 | SENP1 | 3.057E-05 | -0.232 | - | - |
| 11766 | ENSP00000264670 | NSUN2 | 8.211E-06 | -0.232 | - | - |
| 11767 | ENSP00000273261 | LRIG1 | 2.609E-05 | -0.232 | - | - |
| 11768 | ENSP00000265097 | THOC3 | 3.988E-05 | -0.232 | - | - |
| 11769 | ENSP00000337405 | PCID2 | 1.029E-05 | -0.232 | - | - |
| 11770 | ENSP00000304151 | BOP1 | 8.981E-06 | -0.232 | - | - |
| 11771 | ENSP00000319851 | CHDH | 1.930E-05 | -0.232 | - | - |
| 11772 | ENSP00000223364 | MYL7 | 1.598E-05 | -0.232 | - | - |
| 11773 | ENSP00000341382 | SMC4 | 1.688E-05 | -0.232 | - | - |
| 11774 | ENSP00000338573 | GNL3L | 3.103E-05 | -0.232 | - | - |
| 11775 | ENSP00000258494 | ALDH1L2 | 1.167E-05 | -0.232 | - | - |
| 11776 | ENSP00000300651 | MED1 | 3.175E-05 | -0.232 | - | - |
| 11777 | ENSP00000456548 | ELFN1 | 2.378E-06 | -0.232 | - | - |
| 11778 | ENSP00000258739 | KDELR2 | 1.831E-05 | -0.232 | - | - |
| 11779 | ENSP00000261195 | GYS2 | 1.004E-05 | -0.232 | - | - |
| 11780 | ENSP00000270451 | ZNF581 | 2.004E-05 | -0.232 | - | - |
| 11781 | ENSP00000420723 | KRTAP5-8 | 2.467E-06 | -0.232 | - | - |
| 11782 | ENSP00000373572 | FOXI2 | 1.110E-05 | -0.232 | - | - |
| 11783 | ENSP00000267842 | SLC27A2 | 1.195E-05 | -0.232 | - | - |
| 11784 | ENSP00000408288 | PPP1R3E | 1.266E-05 | -0.232 | - | - |
| 11785 | ENSP00000380129 | PPIP5K1 | 2.571E-05 | -0.232 | - | - |
| 11786 | ENSP00000263035 | DHTKD1 | 1.062E-05 | -0.232 | - | - |
| 11787 | ENSP00000260731 | KIF11 | 1.888E-05 | -0.232 | - | - |
| 11788 | ENSP00000341662 | AGMO | 1.579E-05 | -0.232 | - | - |
| 11789 | ENSP00000327213 | NRIP1 | 2.813E-05 | -0.232 | - | - |
| 11790 | ENSP00000300408 | PHB | 9.870E-06 | -0.232 | - | - |
| 11791 | ENSP00000356410 | GLRX2 | 1.486E-05 | -0.232 | - | - |
| 11792 | ENSP00000341947 | LIN54 | 2.457E-05 | -0.232 | - | - |
| 11793 | ENSP00000311747 | RBM14 | 2.524E-05 | -0.233 | - | - |
| 11794 | ENSP00000357927 | BNIPL | 1.653E-05 | -0.233 | - | - |
| 11795 | ENSP00000334040 | RSBN1L | 4.004E-05 | -0.233 | - | - |
| 11796 | ENSP00000285949 | CYP26C1 | 2.105E-05 | -0.233 | - | - |
| 11797 | ENSP00000259021 | KAT7 | 1.064E-05 | -0.233 | - | - |
| 11798 | ENSP00000366275 | CNNM4 | 1.560E-05 | -0.233 | - | - |
| 11799 | ENSP00000249005 | A4GALT | 2.662E-05 | -0.233 | - | - |
| 11800 | ENSP00000329558 | GRPEL2 | 2.037E-05 | -0.233 | - | - |
| 11801 | ENSP00000272317 | RPS27A | 2.137E-05 | -0.233 | - | - |
| 11802 | ENSP00000260443 | RSL24D1 | 1.143E-05 | -0.233 | - | - |
| 11803 | ENSP00000313490 | PFAS | 1.826E-05 | -0.233 | - | - |
| 11804 | ENSP00000268489 | ZFHX3 | 3.155E-05 | -0.233 | - | - |
| 11805 | ENSP00000418603 | POLR2J2 | 1.463E-05 | -0.233 | - | - |
| 11806 | ENSP00000304592 | FASN | 3.308E-05 | -0.233 | - | - |
| 11807 | ENSP00000264710 | RAB10 | 1.361E-05 | -0.233 | - | - |
| 11808 | ENSP00000257177 | ZCCHC11 | 2.689E-05 | -0.233 | - | - |
| 11809 | ENSP00000259791 | HIST1H2AB | 2.126E-05 | -0.233 | - | - |
| 11810 | ENSP00000305975 | KRTAP1-1 | 1.728E-05 | -0.233 | - | - |
| 11811 | ENSP00000446714 | MYL6 | 2.073E-05 | -0.233 | - | - |
| 11812 | ENSP00000402835 | - | 1.075E-05 | -0.233 | - | - |
| 11813 | ENSP00000298767 | WAPAL | 2.294E-05 | -0.233 | - | - |
| 11814 | ENSP00000468862 | ZNF714 | 1.061E-05 | -0.233 | - | - |
| 11815 | ENSP00000268616 | ZCCHC14 | 1.523E-05 | -0.233 | - | - |
| 11816 | ENSP00000283875 | GTF2E1 | 2.040E-05 | -0.233 | - | - |
| 11817 | ENSP00000247986 | KIF17 | 1.348E-05 | -0.233 | - | - |
| 11818 | ENSP00000470752 | AL117190.3 | 2.106E-06 | -0.233 | - | - |
| 11819 | ENSP00000298838 | PACSIN3 | 3.921E-05 | -0.233 | - | - |
| 11820 | ENSP00000358456 | F8A1 | 1.717E-05 | -0.233 | - | - |
| 11821 | ENSP00000080059 | HDAC7 | 1.289E-05 | -0.233 | - | - |
| 11822 | ENSP00000329757 | ATP6V0C | 9.963E-06 | -0.233 | - | - |
| 11823 | ENSP00000264895 | FRAS1 | 1.086E-05 | -0.233 | - | - |
| 11824 | ENSP00000309539 | DPYSL2 | 1.623E-05 | -0.233 | - | - |
| 11825 | ENSP00000286918 | ANKRD9 | 9.340E-06 | -0.233 | - | - |
| 11826 | ENSP00000269391 | RNF157 | 2.603E-05 | -0.233 | - | - |
| 11827 | ENSP00000313811 | USP20 | 1.375E-05 | -0.233 | - | - |
| 11828 | ENSP00000380598 | BLOC1S5 | 1.507E-05 | -0.233 | - | - |
| 11829 | ENSP00000230085 | SNX3 | 2.027E-05 | -0.233 | - | - |
| 11830 | ENSP00000233710 | ACADL | 1.198E-05 | -0.233 | - | - |
| 11831 | ENSP00000354620 | FOXJ3 | 1.075E-05 | -0.233 | - | - |
| 11832 | ENSP00000317904 | GYS1 | 1.670E-05 | -0.233 | - | - |
| 11833 | ENSP00000297837 | ANKS6 | 7.680E-06 | -0.233 | - | - |
| 11834 | ENSP00000333837 | MRPL12 | 7.337E-06 | -0.233 | - | - |
| 11835 | ENSP00000232607 | UMPS | 1.179E-05 | -0.233 | - | - |
| 11836 | ENSP00000296420 | EMCN | 2.323E-05 | -0.233 | - | - |
| 11837 | ENSP00000385751 | MARK2 | 3.624E-05 | -0.233 | - | - |
| 11838 | ENSP00000272542 | SLC20A1 | 2.848E-05 | -0.233 | - | - |
| 11839 | ENSP00000292431 | NACC1 | 1.769E-05 | -0.233 | - | - |
| 11840 | ENSP00000335416 | TMEM68 | 1.625E-05 | -0.233 | - | - |
| 11841 | ENSP00000310301 | SP3 | 2.295E-05 | -0.233 | - | - |
| 11842 | ENSP00000316905 | SFXN1 | 2.697E-05 | -0.233 | - | - |
| 11843 | ENSP00000264515 | RBBP5 | 1.950E-05 | -0.233 | - | - |
| 11844 | ENSP00000315743 | CCNG2 | 2.286E-05 | -0.233 | - | - |
| 11845 | ENSP00000267116 | ANKRD52 | 1.117E-05 | -0.233 | - | - |
| 11846 | ENSP00000394249 | FLCN | 8.250E-06 | -0.233 | - | - |
| 11847 | ENSP00000257347 | CARS2 | 5.344E-06 | -0.233 | - | - |
| 11848 | ENSP00000314556 | UACA | 1.663E-05 | -0.234 | - | - |
| 11849 | ENSP00000230340 | BYSL | 7.272E-06 | -0.234 | - | - |
| 11850 | ENSP00000219069 | ZNF263 | 1.766E-05 | -0.234 | - | - |
| 11851 | ENSP00000379387 | ZNF445 | 1.559E-05 | -0.234 | - | - |
| 11852 | ENSP00000348996 | TSGA13 | 4.085E-06 | -0.234 | - | - |
| 11853 | ENSP00000346142 | DPAGT1 | 1.902E-05 | -0.234 | - | - |
| 11854 | ENSP00000295448 | GNPDA2 | 1.110E-05 | -0.234 | - | - |
| 11855 | ENSP00000419425 | PPIA | 4.646E-05 | -0.234 | - | - |
| 11856 | ENSP00000292123 | SAFB | 2.998E-05 | -0.234 | - | - |
| 11857 | ENSP00000263864 | VAMP8 | 1.865E-05 | -0.234 | - | - |
| 11858 | ENSP00000332668 | TSKU | 3.049E-05 | -0.234 | - | - |
| 11859 | ENSP00000263168 | CAPZA1 | 1.971E-05 | -0.234 | - | - |
| 11860 | ENSP00000285850 | SLC7A7 | 1.259E-05 | -0.234 | - | - |
| 11861 | ENSP00000366173 | CBY3 | 1.207E-05 | -0.234 | - | - |
| 11862 | ENSP00000376952 | EME1 | 1.133E-05 | -0.234 | - | - |
| 11863 | ENSP00000333946 | COQ6 | 9.191E-06 | -0.234 | - | - |
| 11864 | ENSP00000326967 | ZNF121 | 1.958E-05 | -0.234 | - | - |
| 11865 | ENSP00000313007 | PABPC1 | 4.442E-05 | -0.234 | - | - |
| 11866 | ENSP00000365891 | WAS | 1.889E-05 | -0.234 | - | - |
| 11867 | ENSP00000293771 | ZNF653 | 2.037E-05 | -0.234 | - | - |
| 11868 | ENSP00000307540 | CST2 | 2.491E-05 | -0.234 | - | - |
| 11869 | ENSP00000188790 | FAP | 2.986E-05 | -0.234 | - | - |
| 11870 | ENSP00000215794 | USP18 | 4.041E-05 | -0.234 | - | - |
| 11871 | ENSP00000292530 | ZNF333 | 1.715E-05 | -0.234 | - | - |
| 11872 | ENSP00000372286 | KRTAP27-1 | 6.167E-07 | -0.234 | - | - |
| 11873 | ENSP00000190983 | WISP2 | 3.571E-05 | -0.234 | - | - |
| 11874 | ENSP00000311492 | ISX | 2.729E-05 | -0.234 | - | - |
| 11875 | ENSP00000337907 | MPP7 | 1.757E-05 | -0.234 | - | - |
| 11876 | ENSP00000315774 | NDUFS8 | 8.568E-06 | -0.234 | - | - |
| 11877 | ENSP00000325313 | MAP1S | 4.432E-05 | -0.234 | - | - |
| 11878 | ENSP00000364649 | SDHB | 2.942E-05 | -0.234 | - | - |
| 11879 | ENSP00000358478 | TTF2 | 2.216E-05 | -0.234 | - | - |
| 11880 | ENSP00000375977 | XRCC5 | 2.322E-05 | -0.234 | - | - |
| 11881 | ENSP00000403304 | TSPY1 | 1.796E-05 | -0.234 | - | - |
| 11882 | ENSP00000344786 | NFASC | 2.645E-05 | -0.234 | - | - |
| 11883 | ENSP00000295050 | SPRTN | 9.309E-06 | -0.234 | - | - |
| 11884 | ENSP00000264220 | PPAT | 8.727E-06 | -0.234 | - | - |
| 11885 | ENSP00000339435 | SRR | 1.194E-05 | -0.234 | - | - |
| 11886 | ENSP00000267294 | ZIC5 | 1.944E-05 | -0.234 | - | - |
| 11887 | ENSP00000389630 | DPY19L4 | 7.912E-06 | -0.234 | - | - |
| 11888 | ENSP00000251269 | ZNF221 | 2.108E-05 | -0.234 | - | - |
| 11889 | ENSP00000220429 | GABPB1 | 2.315E-05 | -0.234 | - | - |
| 11890 | ENSP00000300417 | LRSAM1 | 2.296E-05 | -0.234 | - | - |
| 11891 | ENSP00000244426 | FBXO9 | 2.048E-05 | -0.234 | - | - |
| 11892 | ENSP00000267339 | ANKRD10 | 1.251E-05 | -0.234 | - | - |
| 11893 | ENSP00000230122 | ZBTB24 | 1.659E-05 | -0.234 | - | - |
| 11894 | ENSP00000264029 | TREH | 2.396E-05 | -0.234 | - | - |
| 11895 | ENSP00000397947 | PLEKHA8 | 3.740E-05 | -0.234 | - | - |
| 11896 | ENSP00000422591 | SLIT2 | 4.674E-05 | -0.234 | - | - |
| 11897 | ENSP00000261560 | ZNF430 | 2.045E-05 | -0.234 | - | - |
| 11898 | ENSP00000309812 | ZNF80 | 1.957E-05 | -0.234 | - | - |
| 11899 | ENSP00000260563 | RTCA | 6.288E-06 | -0.234 | - | - |
| 11900 | ENSP00000357409 | DCST2 | 1.228E-05 | -0.234 | - | - |
| 11901 | ENSP00000347495 | WARS | 1.637E-05 | -0.234 | - | - |
| 11902 | ENSP00000324056 | ZNF48 | 1.612E-05 | -0.234 | - | - |
| 11903 | ENSP00000288177 | ZNF19 | 1.729E-05 | -0.234 | - | - |
| 11904 | ENSP00000252979 | ZNF337 | 1.819E-05 | -0.234 | - | - |
| 11905 | ENSP00000329793 | ZNF85 | 1.813E-05 | -0.234 | - | - |
| 11906 | ENSP00000451080 | ERH | 3.218E-05 | -0.234 | - | - |
| 11907 | ENSP00000316589 | RANBP10 | 1.111E-05 | -0.234 | - | - |
| 11908 | ENSP00000323439 | SMC6 | 1.523E-05 | -0.234 | - | - |
| 11909 | ENSP00000316121 | CDCA8 | 2.762E-05 | -0.234 | - | - |
| 11910 | ENSP00000381081 | RNASE4 | 5.411E-06 | -0.234 | - | - |
| 11911 | ENSP00000266735 | SNRPF | 1.676E-05 | -0.234 | - | - |
| 11912 | ENSP00000295470 | HNRPDL | 2.903E-05 | -0.234 | - | - |
| 11913 | ENSP00000240055 | NFYB | 1.065E-05 | -0.234 | - | - |
| 11914 | ENSP00000432353 | PBOV1 | 1.166E-05 | -0.234 | - | - |
| 11915 | ENSP00000375859 | TMEM91 | 1.235E-05 | -0.234 | - | - |
| 11916 | ENSP00000380523 | CAPSL | 2.967E-05 | -0.234 | - | - |
| 11917 | ENSP00000254321 | ZNF700 | 1.849E-05 | -0.234 | - | - |
| 11918 | ENSP00000205402 | DLD | 7.084E-06 | -0.234 | - | - |
| 11919 | ENSP00000375899 | SP140 | 2.872E-05 | -0.234 | - | - |
| 11920 | ENSP00000410360 | KIAA0125 | 5.179E-06 | -0.234 | - | - |
| 11921 | ENSP00000306397 | UQCRFS1 | 1.074E-05 | -0.234 | - | - |
| 11922 | ENSP00000283646 | RPIA | 1.753E-05 | -0.234 | - | - |
| 11923 | ENSP00000399709 | BEST1 | 2.601E-05 | -0.234 | - | - |
| 11924 | ENSP00000241600 | MRPS2 | 8.665E-06 | -0.234 | - | - |
| 11925 | ENSP00000415662 | C17orf105 | 5.328E-06 | -0.234 | - | - |
| 11926 | ENSP00000286355 | ADCY8 | 2.184E-05 | -0.234 | - | - |
| 11927 | ENSP00000300870 | ZNF267 | 1.843E-05 | -0.234 | - | - |
| 11928 | ENSP00000262746 | PRDX1 | 2.056E-05 | -0.234 | - | - |
| 11929 | ENSP00000229854 | MCM3 | 1.117E-05 | -0.234 | - | - |
| 11930 | ENSP00000255746 | ZNF679 | 2.039E-05 | -0.234 | - | - |
| 11931 | ENSP00000304565 | - | 1.473E-05 | -0.234 | - | - |
| 11932 | ENSP00000301399 | ZNF577 | 1.656E-05 | -0.234 | - | - |
| 11933 | ENSP00000331487 | NPLOC4 | 2.837E-05 | -0.234 | - | - |
| 11934 | ENSP00000264170 | KYNU | 3.408E-05 | -0.234 | - | - |
| 11935 | ENSP00000386458 | PRPF40A | 3.168E-05 | -0.234 | - | - |
| 11936 | ENSP00000402065 | POLR2J2 | 1.342E-05 | -0.234 | - | - |
| 11937 | ENSP00000304670 | RNFT1 | 7.032E-06 | -0.234 | - | - |
| 11938 | ENSP00000290299 | ATP5O | 1.460E-05 | -0.234 | - | - |
| 11939 | ENSP00000328245 | ZNF71 | 1.778E-05 | -0.234 | - | - |
| 11940 | ENSP00000274867 | DAAM2 | 1.944E-05 | -0.234 | - | - |
| 11941 | ENSP00000394734 | CLK1 | 2.030E-05 | -0.234 | - | - |
| 11942 | ENSP00000318297 | RUVBL1 | 2.874E-05 | -0.235 | - | - |
| 11943 | ENSP00000374135 | LRP1B | 2.568E-05 | -0.235 | - | - |
| 11944 | ENSP00000228251 | YBX3 | 1.456E-05 | -0.235 | - | - |
| 11945 | ENSP00000394623 | NUTM2B | 7.230E-06 | -0.235 | - | - |
| 11946 | ENSP00000264864 | PI4K2B | 1.156E-05 | -0.235 | - | - |
| 11947 | ENSP00000262215 | ARFGEF1 | 1.485E-05 | -0.235 | - | - |
| 11948 | ENSP00000418719 | ZNF273 | 1.025E-05 | -0.235 | - | - |
| 11949 | ENSP00000410466 | ZNF90 | 9.609E-06 | -0.235 | - | - |
| 11950 | ENSP00000363054 | STX12 | 9.178E-06 | -0.235 | - | - |
| 11951 | ENSP00000339957 | USP47 | 1.949E-05 | -0.235 | - | - |
| 11952 | ENSP00000337555 | ZNF548 | 1.760E-05 | -0.235 | - | - |
| 11953 | ENSP00000283441 | ZDHHC11 | 2.487E-05 | -0.235 | - | - |
| 11954 | ENSP00000261597 | NDC80 | 1.892E-05 | -0.235 | - | - |
| 11955 | ENSP00000260508 | CCBL2 | 1.209E-05 | -0.235 | - | - |
| 11956 | ENSP00000323046 | EXOSC3 | 1.587E-05 | -0.235 | - | - |
| 11957 | ENSP00000013125 | MAP4K5 | 1.180E-05 | -0.235 | - | - |
| 11958 | ENSP00000223459 | ZNF688 | 2.089E-05 | -0.235 | - | - |
| 11959 | ENSP00000053468 | MRPS10 | 8.031E-06 | -0.235 | - | - |
| 11960 | ENSP00000317790 | SPTBN5 | 2.328E-05 | -0.235 | - | - |
| 11961 | ENSP00000263383 | ILVBL | 1.232E-05 | -0.235 | - | - |
| 11962 | ENSP00000309141 | 3-Mar | 2.340E-05 | -0.235 | - | - |
| 11963 | ENSP00000467024 | RAD23A | 6.376E-05 | -0.235 | - | - |
| 11964 | ENSP00000215829 | SNRPD3 | 1.481E-05 | -0.235 | - | - |
| 11965 | ENSP00000318154 | MRFAP1L1 | 6.082E-05 | -0.235 | - | - |
| 11966 | ENSP00000378313 | RAB3IL1 | 2.691E-05 | -0.235 | - | - |
| 11967 | ENSP00000341911 | PALM | 5.157E-05 | -0.235 | - | - |
| 11968 | ENSP00000418910 | SELT | 4.852E-06 | -0.235 | - | - |
| 11969 | ENSP00000326888 | LIMS2 | 1.531E-05 | -0.235 | - | - |
| 11970 | ENSP00000388548 | CITED1 | 1.716E-05 | -0.235 | - | - |
| 11971 | ENSP00000326813 | YOD1 | 1.911E-05 | -0.235 | - | - |
| 11972 | ENSP00000274364 | IQGAP2 | 2.434E-05 | -0.235 | - | - |
| 11973 | ENSP00000337354 | LIPA | 2.519E-05 | -0.235 | - | - |
| 11974 | ENSP00000334501 | DMRTC1B | 3.805E-06 | -0.235 | - | - |
| 11975 | ENSP00000364839 | ASXL1 | 1.945E-05 | -0.235 | - | - |
| 11976 | ENSP00000263559 | VPS26A | 1.740E-05 | -0.235 | - | - |
| 11977 | ENSP00000302456 | KLF13 | 1.901E-05 | -0.235 | - | - |
| 11978 | ENSP00000301730 | RNPS1 | 1.583E-05 | -0.235 | - | - |
| 11979 | ENSP00000155093 | ZFY | 1.624E-05 | -0.235 | - | - |
| 11980 | ENSP00000263274 | LIG1 | 8.507E-06 | -0.235 | - | - |
| 11981 | ENSP00000362946 | RAB14 | 2.264E-05 | -0.235 | - | - |
| 11982 | ENSP00000250535 | CDO1 | 1.287E-05 | -0.235 | - | - |
| 11983 | ENSP00000221327 | ZNF180 | 2.039E-05 | -0.235 | - | - |
| 11984 | ENSP00000194118 | KIAA0141 | 1.905E-05 | -0.235 | - | - |
| 11985 | ENSP00000375736 | HIST3H2BB | 2.771E-05 | -0.235 | - | - |
| 11986 | ENSP00000470409 | AP003062.1 | 1.127E-07 | -0.235 | - | - |
| 11987 | ENSP00000272645 | POLR2D | 1.796E-05 | -0.235 | - | - |
| 11988 | ENSP00000369962 | IGSF5 | 2.165E-05 | -0.235 | - | - |
| 11989 | ENSP00000320949 | CNOT1 | 3.320E-05 | -0.235 | - | - |
| 11990 | ENSP00000333019 | ADSSL1 | 1.055E-05 | -0.235 | - | - |
| 11991 | ENSP00000319305 | ZNF572 | 1.885E-05 | -0.235 | - | - |
| 11992 | ENSP00000308275 | MRPL38 | 1.507E-05 | -0.235 | - | - |
| 11993 | ENSP00000261507 | MSMO1 | 2.127E-05 | -0.235 | - | - |
| 11994 | ENSP00000282869 | ZNF117 | 1.828E-05 | -0.235 | - | - |
| 11995 | ENSP00000386165 | CEBPD | 6.215E-05 | -0.235 | - | - |
| 11996 | ENSP00000309555 | HCFC1 | 1.945E-05 | -0.235 | - | - |
| 11997 | ENSP00000294172 | NXF1 | 1.947E-05 | -0.235 | - | - |
| 11998 | ENSP00000384370 | STEAP1B | 6.386E-06 | -0.235 | - | - |
| 11999 | ENSP00000386520 | RAD54L2 | 1.958E-05 | -0.235 | - | - |
| 12000 | ENSP00000242804 | ZNF442 | 1.901E-05 | -0.235 | - | - |
| 12001 | ENSP00000318352 | MRFAP1 | 5.715E-05 | -0.235 | - | - |
| 12002 | ENSP00000384848 | PRR5 | 2.641E-05 | -0.235 | - | - |
| 12003 | ENSP00000396308 | DHFR | 4.336E-05 | -0.235 | - | - |
| 12004 | ENSP00000386170 | TBX20 | 1.920E-05 | -0.235 | - | - |
| 12005 | ENSP00000265846 | ADAP1 | 2.286E-05 | -0.235 | - | - |
| 12006 | ENSP00000395369 | NBPF16 | 1.021E-06 | -0.235 | - | - |
| 12007 | ENSP00000300896 | USP32 | 2.596E-05 | -0.235 | - | - |
| 12008 | ENSP00000216554 | EIF5 | 1.674E-05 | -0.235 | - | - |
| 12009 | ENSP00000251607 | TRNT1 | 9.779E-06 | -0.235 | - | - |
| 12010 | ENSP00000215570 | TIMM13 | 1.318E-05 | -0.235 | - | - |
| 12011 | ENSP00000384979 | LINGO3 | 9.393E-06 | -0.235 | - | - |
| 12012 | ENSP00000381097 | EPHB1 | 3.281E-05 | -0.235 | - | - |
| 12013 | ENSP00000263063 | MTPAP | 2.540E-05 | -0.235 | - | - |
| 12014 | ENSP00000309092 | GMPPB | 9.023E-06 | -0.235 | - | - |
| 12015 | ENSP00000247584 | HIVEP3 | 1.893E-05 | -0.235 | - | - |
| 12016 | ENSP00000243644 | ZNF350 | 1.960E-05 | -0.235 | - | - |
| 12017 | ENSP00000287538 | ZIC3 | 1.823E-05 | -0.235 | - | - |
| 12018 | ENSP00000359777 | CT45A1 | 9.721E-06 | -0.235 | - | - |
| 12019 | ENSP00000321519 | C19orf18 | 2.289E-05 | -0.235 | - | - |
| 12020 | ENSP00000263710 | CLASP1 | 1.182E-05 | -0.235 | - | - |
| 12021 | ENSP00000248211 | ZNF10 | 1.848E-05 | -0.235 | - | - |
| 12022 | ENSP00000332198 | NOP10 | 7.553E-06 | -0.235 | - | - |
| 12023 | ENSP00000264193 | CPOX | 1.972E-05 | -0.235 | - | - |
| 12024 | ENSP00000319664 | NUDC | 1.288E-05 | -0.236 | - | - |
| 12025 | ENSP00000343741 | ATR | 2.828E-05 | -0.236 | - | - |
| 12026 | ENSP00000379702 | ZNF665 | 1.173E-05 | -0.236 | - | - |
| 12027 | ENSP00000265562 | PTPN23 | 1.506E-05 | -0.236 | - | - |
| 12028 | ENSP00000320347 | ZFP3 | 1.531E-05 | -0.236 | - | - |
| 12029 | ENSP00000333802 | ZNF599 | 1.662E-05 | -0.236 | - | - |
| 12030 | ENSP00000338477 | HNRNPF | 2.287E-05 | -0.236 | - | - |
| 12031 | ENSP00000317902 | CEP57 | 1.947E-05 | -0.236 | - | - |
| 12032 | ENSP00000352035 | KCNQ2 | 2.277E-05 | -0.236 | - | - |
| 12033 | ENSP00000272227 | PDIA6 | 1.115E-05 | -0.236 | - | - |
| 12034 | ENSP00000342832 | YPEL1 | 2.401E-05 | -0.236 | - | - |
| 12035 | ENSP00000383851 | KIAA1522 | 2.088E-05 | -0.236 | - | - |
| 12036 | ENSP00000308369 | LEMD3 | 2.532E-05 | -0.236 | - | - |
| 12037 | ENSP00000322229 | FADS1 | 1.670E-05 | -0.236 | - | - |
| 12038 | ENSP00000440403 | ZNF678 | 9.295E-06 | -0.236 | - | - |
| 12039 | ENSP00000314030 | DNAJA2 | 2.273E-05 | -0.236 | - | - |
| 12040 | ENSP00000346483 | DDRGK1 | 1.270E-05 | -0.236 | - | - |
| 12041 | ENSP00000252840 | ZNF557 | 1.804E-05 | -0.236 | - | - |
| 12042 | ENSP00000315299 | CALML3 | 1.721E-05 | -0.236 | - | - |
| 12043 | ENSP00000322716 | GLYR1 | 2.064E-05 | -0.236 | - | - |
| 12044 | ENSP00000305193 | HS1BP3 | 6.213E-05 | -0.236 | - | - |
| 12045 | ENSP00000446132 | NAV3 | 6.809E-06 | -0.236 | - | - |
| 12046 | ENSP00000319781 | RAB43 | 7.411E-06 | -0.236 | - | - |
| 12047 | ENSP00000341938 | SUMF2 | 3.461E-05 | -0.236 | - | - |
| 12048 | ENSP00000221801 | FBL | 1.498E-05 | -0.236 | - | - |
| 12049 | ENSP00000255695 | HRASLS2 | 4.002E-05 | -0.236 | - | - |
| 12050 | ENSP00000292928 | ZNF382 | 1.615E-05 | -0.236 | - | - |
| 12051 | ENSP00000278840 | FADS2 | 2.006E-05 | -0.236 | - | - |
| 12052 | ENSP00000343943 | PPOX | 3.485E-05 | -0.236 | - | - |
| 12053 | ENSP00000306760 | LRRC45 | 8.735E-05 | -0.236 | - | - |
| 12054 | ENSP00000282282 | ZNF547 | 1.902E-05 | -0.236 | - | - |
| 12055 | ENSP00000247087 | AHDC1 | 1.017E-04 | -0.236 | - | - |
| 12056 | ENSP00000369320 | RS1 | 2.041E-05 | -0.236 | - | - |
| 12057 | ENSP00000269349 | EIF4A3 | 2.501E-05 | -0.236 | - | - |
| 12058 | ENSP00000269829 | ZNF544 | 1.715E-05 | -0.236 | - | - |
| 12059 | ENSP00000324124 | POLR2L | 1.028E-05 | -0.236 | - | - |
| 12060 | ENSP00000262188 | SMARCD3 | 1.539E-05 | -0.236 | - | - |
| 12061 | ENSP00000337972 | GDPD5 | 1.395E-05 | -0.236 | - | - |
| 12062 | ENSP00000376024 | SNX9 | 3.095E-05 | -0.236 | - | - |
| 12063 | ENSP00000455911 | ZFP91-CNTF | 1.167E-05 | -0.236 | - | - |
| 12064 | ENSP00000316338 | BAIAP2 | 3.465E-05 | -0.236 | - | - |
| 12065 | ENSP00000265351 | XPO5 | 1.604E-05 | -0.236 | - | - |
| 12066 | ENSP00000278772 | ZNF343 | 1.865E-05 | -0.236 | - | - |
| 12067 | ENSP00000324792 | NFATC2IP | 2.467E-05 | -0.236 | - | - |
| 12068 | ENSP00000309429 | ZDHHC24 | 3.668E-05 | -0.236 | - | - |
| 12069 | ENSP00000335304 | DLST | 1.009E-05 | -0.236 | - | - |
| 12070 | ENSP00000359285 | CHRNA4 | 2.511E-05 | -0.236 | - | - |
| 12071 | ENSP00000223428 | ZNF510 | 1.925E-05 | -0.236 | - | - |
| 12072 | ENSP00000380781 | NFIX | 1.942E-05 | -0.236 | - | - |
| 12073 | ENSP00000356567 | ACBD6 | 7.007E-06 | -0.236 | - | - |
| 12074 | ENSP00000372093 | MRPL36 | 2.661E-05 | -0.236 | - | - |
| 12075 | ENSP00000329365 | - | 1.648E-05 | -0.236 | - | - |
| 12076 | ENSP00000260184 | DDX60L | 1.084E-05 | -0.236 | - | - |
| 12077 | ENSP00000422431 | TMEM158 | 2.585E-05 | -0.236 | - | - |
| 12078 | ENSP00000078527 | PIGV | 1.469E-05 | -0.236 | - | - |
| 12079 | ENSP00000216252 | PHF5A | 1.704E-05 | -0.236 | - | - |
| 12080 | ENSP00000294179 | STX5 | 6.738E-06 | -0.236 | - | - |
| 12081 | ENSP00000333800 | MRGPRX2 | 2.164E-05 | -0.236 | - | - |
| 12082 | ENSP00000322265 | ZNF620 | 1.667E-05 | -0.236 | - | - |
| 12083 | ENSP00000360891 | IFIT2 | 3.776E-05 | -0.236 | - | - |
| 12084 | ENSP00000419475 | COMMD2 | 6.025E-06 | -0.237 | - | - |
| 12085 | ENSP00000261427 | UBE2K | 1.875E-05 | -0.237 | - | - |
| 12086 | ENSP00000296509 | MAD2L1 | 1.616E-05 | -0.237 | - | - |
| 12087 | ENSP00000219368 | FA2H | 1.038E-05 | -0.237 | - | - |
| 12088 | ENSP00000305556 | PCBP1 | 1.917E-05 | -0.237 | - | - |
| 12089 | ENSP00000330141 | SLC16A5 | 1.331E-05 | -0.237 | - | - |
| 12090 | ENSP00000349208 | PIWIL2 | 2.176E-05 | -0.237 | - | - |
| 12091 | ENSP00000307508 | CALB2 | 1.800E-05 | -0.237 | - | - |
| 12092 | ENSP00000402239 | CCDC159 | 7.987E-06 | -0.237 | - | - |
| 12093 | ENSP00000304127 | KCNG3 | 2.001E-05 | -0.237 | - | - |
| 12094 | ENSP00000255448 | DCLK1 | 1.061E-05 | -0.237 | - | - |
| 12095 | ENSP00000393725 | GFRA1 | 2.094E-05 | -0.237 | - | - |
| 12096 | ENSP00000384183 | GATSL3 | 7.958E-06 | -0.237 | - | - |
| 12097 | ENSP00000311319 | ZNF417 | 1.751E-05 | -0.237 | - | - |
| 12098 | ENSP00000275423 | RBAK | 1.751E-05 | -0.237 | - | - |
| 12099 | ENSP00000331640 | TMEM186 | 6.081E-05 | -0.237 | - | - |
| 12100 | ENSP00000280701 | OXSM | 1.023E-05 | -0.237 | - | - |
| 12101 | ENSP00000408058 | METTL11B | 2.020E-05 | -0.237 | - | - |
| 12102 | ENSP00000341236 | ZNF695 | 1.855E-05 | -0.237 | - | - |
| 12103 | ENSP00000257627 | OCM2 | 1.023E-05 | -0.237 | - | - |
| 12104 | ENSP00000344741 | INSIG1 | 2.703E-05 | -0.237 | - | - |
| 12105 | ENSP00000322899 | ZNF843 | 1.665E-05 | -0.237 | - | - |
| 12106 | ENSP00000427550 | CEP192 | 7.970E-06 | -0.237 | - | - |
| 12107 | ENSP00000221665 | FIZ1 | 1.845E-05 | -0.237 | - | - |
| 12108 | ENSP00000297185 | HSPA9 | 2.499E-05 | -0.237 | - | - |
| 12109 | ENSP00000253363 | RBM39 | 1.418E-05 | -0.237 | - | - |
| 12110 | ENSP00000285238 | ABCC3 | 2.097E-05 | -0.237 | - | - |
| 12111 | ENSP00000339256 | NIPAL2 | 9.436E-06 | -0.237 | - | - |
| 12112 | ENSP00000309330 | ZNF680 | 1.696E-05 | -0.237 | - | - |
| 12113 | ENSP00000340688 | LPHN1 | 1.761E-05 | -0.237 | - | - |
| 12114 | ENSP00000361170 | MUTYH | 2.064E-05 | -0.237 | - | - |
| 12115 | ENSP00000422188 | TTC23L | 4.404E-06 | -0.237 | - | - |
| 12116 | ENSP00000418397 | IVD | 1.709E-05 | -0.237 | - | - |
| 12117 | ENSP00000222673 | OGDH | 9.581E-06 | -0.237 | - | - |
| 12118 | ENSP00000340841 | ZNF621 | 1.570E-05 | -0.237 | - | - |
| 12119 | ENSP00000313443 | ZNF491 | 1.685E-05 | -0.237 | - | - |
| 12120 | ENSP00000366618 | HIST1H2BL | 2.784E-05 | -0.237 | - | - |
| 12121 | ENSP00000335042 | ZNF486 | 1.640E-05 | -0.237 | - | - |
| 12122 | ENSP00000362110 | SF3A3 | 8.230E-06 | -0.237 | - | - |
| 12123 | ENSP00000242209 | FKBP9 | 1.307E-05 | -0.237 | - | - |
| 12124 | ENSP00000228437 | PRDM4 | 1.759E-05 | -0.237 | - | - |
| 12125 | ENSP00000268655 | ZNF174 | 1.838E-05 | -0.237 | - | - |
| 12126 | ENSP00000306869 | ZNF565 | 1.713E-05 | -0.237 | - | - |
| 12127 | ENSP00000261942 | FAF2 | 3.119E-05 | -0.237 | - | - |
| 12128 | ENSP00000285393 | ATP6V0D2 | 8.794E-06 | -0.237 | - | - |
| 12129 | ENSP00000396857 | ZNF697 | 1.165E-05 | -0.237 | - | - |
| 12130 | ENSP00000343701 | KPNA1 | 2.256E-05 | -0.237 | - | - |
| 12131 | ENSP00000320050 | ZNF580 | 1.536E-05 | -0.237 | - | - |
| 12132 | ENSP00000319591 | KCND3 | 2.161E-05 | -0.237 | - | - |
| 12133 | ENSP00000247930 | ZNF777 | 1.771E-05 | -0.237 | - | - |
| 12134 | ENSP00000264499 | BBS7 | 2.512E-05 | -0.237 | - | - |
| 12135 | ENSP00000393845 | PPIAL4G | 2.223E-05 | -0.237 | - | - |
| 12136 | ENSP00000308430 | CYB5B | 1.464E-05 | -0.237 | - | - |
| 12137 | ENSP00000281456 | SLC25A4 | 9.592E-06 | -0.237 | - | - |
| 12138 | ENSP00000464976 | ZNF850 | 8.993E-06 | -0.237 | - | - |
| 12139 | ENSP00000341165 | ZNF772 | 1.530E-05 | -0.237 | - | - |
| 12140 | ENSP00000270014 | ZNF155 | 1.770E-05 | -0.237 | - | - |
| 12141 | ENSP00000429808 | PCDHA3 | 7.219E-06 | -0.237 | - | - |
| 12142 | ENSP00000358820 | EPS8L3 | 3.144E-05 | -0.237 | - | - |
| 12143 | ENSP00000260947 | BARD1 | 1.725E-05 | -0.237 | - | - |
| 12144 | ENSP00000265316 | ABCB6 | 1.102E-05 | -0.237 | - | - |
| 12145 | ENSP00000367835 | PRPF18 | 1.172E-05 | -0.237 | - | - |
| 12146 | ENSP00000250416 | PARP2 | 4.357E-05 | -0.237 | - | - |
| 12147 | ENSP00000321812 | ZNF776 | 1.669E-05 | -0.237 | - | - |
| 12148 | ENSP00000261401 | CORO1C | 1.644E-05 | -0.237 | - | - |
| 12149 | ENSP00000414037 | AL592170.1 | 1.566E-06 | -0.237 | - | - |
| 12150 | ENSP00000306351 | ZNF182 | 1.595E-05 | -0.237 | - | - |
| 12151 | ENSP00000315870 | ZNF575 | 1.680E-05 | -0.237 | - | - |
| 12152 | ENSP00000221722 | ZIM2 | 1.691E-05 | -0.237 | - | - |
| 12153 | ENSP00000216071 | C22orf31 | 5.035E-05 | -0.237 | - | - |
| 12154 | ENSP00000293842 | RPL26 | 1.143E-05 | -0.237 | - | - |
| 12155 | ENSP00000258301 | STX6 | 2.171E-05 | -0.237 | - | - |
| 12156 | ENSP00000308895 | SLC19A1 | 1.667E-05 | -0.237 | - | - |
| 12157 | ENSP00000333980 | ZNF749 | 1.612E-05 | -0.237 | - | - |
| 12158 | ENSP00000252463 | ZSCAN10 | 1.887E-05 | -0.237 | - | - |
| 12159 | ENSP00000343930 | KLHL17 | 2.471E-05 | -0.237 | - | - |
| 12160 | ENSP00000265100 | RPL26L1 | 1.330E-05 | -0.237 | - | - |
| 12161 | ENSP00000255129 | ZNF334 | 1.749E-05 | -0.237 | - | - |
| 12162 | ENSP00000390600 | SCN10A | 2.190E-05 | -0.238 | - | - |
| 12163 | ENSP00000309714 | SH3PXD2B | 2.025E-05 | -0.238 | - | - |
| 12164 | ENSP00000349977 | MVP | 1.426E-05 | -0.238 | - | - |
| 12165 | ENSP00000244571 | AARS2 | 8.772E-06 | -0.238 | - | - |
| 12166 | ENSP00000328521 | CRIP2 | 2.814E-05 | -0.238 | - | - |
| 12167 | ENSP00000366135 | EXOSC10 | 1.432E-05 | -0.238 | - | - |
| 12168 | ENSP00000382675 | ENHO | 2.218E-06 | -0.238 | - | - |
| 12169 | ENSP00000215956 | NHP2L1 | 4.594E-06 | -0.238 | - | - |
| 12170 | ENSP00000263847 | OSBP | 1.586E-05 | -0.238 | - | - |
| 12171 | ENSP00000299575 | ATMIN | 1.699E-05 | -0.238 | - | - |
| 12172 | ENSP00000317686 | ZFP42 | 2.052E-05 | -0.238 | - | - |
| 12173 | ENSP00000222388 | ABCF2 | 7.342E-06 | -0.238 | - | - |
| 12174 | ENSP00000325776 | SERTM1 | 7.242E-06 | -0.238 | - | - |
| 12175 | ENSP00000261015 | WDR12 | 8.098E-06 | -0.238 | - | - |
| 12176 | ENSP00000223054 | MOSPD3 | 2.481E-05 | -0.238 | - | - |
| 12177 | ENSP00000269848 | PFKL | 1.501E-05 | -0.238 | - | - |
| 12178 | ENSP00000293670 | KRT83 | 2.890E-05 | -0.238 | - | - |
| 12179 | ENSP00000320485 | ARID1A | 2.884E-05 | -0.238 | - | - |
| 12180 | ENSP00000291295 | CALM3 | 1.316E-05 | -0.238 | - | - |
| 12181 | ENSP00000328690 | DDX24 | 6.405E-06 | -0.238 | - | - |
| 12182 | ENSP00000384881 | ABCB5 | 1.754E-05 | -0.238 | - | - |
| 12183 | ENSP00000308332 | UTP23 | 5.874E-06 | -0.238 | - | - |
| 12184 | ENSP00000330812 | SDR42E2 | 1.188E-05 | -0.238 | - | - |
| 12185 | ENSP00000327453 | ACSM2B | 1.072E-05 | -0.238 | - | - |
| 12186 | ENSP00000226105 | RANGRF | 3.685E-05 | -0.238 | - | - |
| 12187 | ENSP00000290949 | ATP6V0D1 | 9.325E-06 | -0.238 | - | - |
| 12188 | ENSP00000362888 | ADO | 2.164E-05 | -0.238 | - | - |
| 12189 | ENSP00000229270 | TPI1 | 1.135E-05 | -0.238 | - | - |
| 12190 | ENSP00000335632 | CHP1 | 1.757E-05 | -0.238 | - | - |
| 12191 | ENSP00000293805 | BCL6B | 2.411E-05 | -0.238 | - | - |
| 12192 | ENSP00000386166 | PRR21 | 5.356E-06 | -0.238 | - | - |
| 12193 | ENSP00000362352 | H2AFY2 | 1.339E-05 | -0.238 | - | - |
| 12194 | ENSP00000301740 | SRRM2 | 3.581E-05 | -0.238 | - | - |
| 12195 | ENSP00000317125 | ZNF726 | 1.585E-05 | -0.238 | - | - |
| 12196 | ENSP00000333660 | ZNF571 | 1.555E-05 | -0.238 | - | - |
| 12197 | ENSP00000228289 | ZNF268 | 1.528E-05 | -0.238 | - | - |
| 12198 | ENSP00000253237 | GRWD1 | 5.978E-06 | -0.238 | - | - |
| 12199 | ENSP00000469337 | LRRC63 | 6.884E-06 | -0.238 | - | - |
| 12200 | ENSP00000196548 | ZNF8 | 1.794E-05 | -0.238 | - | - |
| 12201 | ENSP00000390630 | RIMBP3C | 1.983E-06 | -0.238 | - | - |
| 12202 | ENSP00000359634 | GOLGA7B | 1.161E-05 | -0.238 | - | - |
| 12203 | ENSP00000364110 | PAGE2B | 7.921E-06 | -0.238 | - | - |
| 12204 | ENSP00000279873 | ARID5B | 4.022E-05 | -0.238 | - | - |
| 12205 | ENSP00000320295 | TUBB3 | 2.307E-05 | -0.238 | - | - |
| 12206 | ENSP00000398516 | L3MBTL1 | 1.506E-05 | -0.238 | - | - |
| 12207 | ENSP00000386541 | PSMD14 | 3.747E-05 | -0.238 | - | - |
| 12208 | ENSP00000396755 | CNTD2 | 1.727E-05 | -0.238 | - | - |
| 12209 | ENSP00000358518 | F8A2 | 1.823E-05 | -0.238 | - | - |
| 12210 | ENSP00000310335 | REP15 | 2.917E-05 | -0.238 | - | - |
| 12211 | ENSP00000309653 | GLIS1 | 1.851E-05 | -0.238 | - | - |
| 12212 | ENSP00000354920 | METTL13 | 1.299E-05 | -0.238 | - | - |
| 12213 | ENSP00000215115 | BCL7C | 2.212E-05 | -0.238 | - | - |
| 12214 | ENSP00000305465 | PDILT | 9.865E-06 | -0.238 | - | - |
| 12215 | ENSP00000335147 | ANKRD37 | 2.090E-05 | -0.238 | - | - |
| 12216 | ENSP00000290158 | KPNB1 | 1.866E-05 | -0.238 | - | - |
| 12217 | ENSP00000290810 | NAE1 | 2.517E-05 | -0.238 | - | - |
| 12218 | ENSP00000296233 | KLF15 | 1.667E-05 | -0.238 | - | - |
| 12219 | ENSP00000369325 | CDKL5 | 6.313E-06 | -0.238 | - | - |
| 12220 | ENSP00000342481 | ATP9A | 2.507E-05 | -0.238 | - | - |
| 12221 | ENSP00000250076 | ZNF232 | 1.780E-05 | -0.238 | - | - |
| 12222 | ENSP00000274026 | CCNA2 | 2.818E-05 | -0.238 | - | - |
| 12223 | ENSP00000229379 | COX6A1 | 7.239E-06 | -0.238 | - | - |
| 12224 | ENSP00000365005 | MAP3K19 | 1.625E-05 | -0.238 | - | - |
| 12225 | ENSP00000372122 | - | 3.087E-06 | -0.238 | - | - |
| 12226 | ENSP00000243643 | ZNF415 | 1.697E-05 | -0.238 | - | - |
| 12227 | ENSP00000383737 | DNAH14 | 1.818E-05 | -0.238 | - | - |
| 12228 | ENSP00000264028 | ARCN1 | 1.017E-05 | -0.238 | - | - |
| 12229 | ENSP00000258412 | TMBIM1 | 1.300E-05 | -0.238 | - | - |
| 12230 | ENSP00000359707 | SAMD13 | 5.273E-06 | -0.238 | - | - |
| 12231 | ENSP00000316924 | ACSS1 | 8.416E-06 | -0.238 | - | - |
| 12232 | ENSP00000352252 | HIST1H3J | 2.263E-05 | -0.239 | - | - |
| 12233 | ENSP00000334140 | KLHDC10 | 1.406E-05 | -0.239 | - | - |
| 12234 | ENSP00000253693 | CAPN7 | 8.705E-06 | -0.239 | - | - |
| 12235 | ENSP00000262554 | SPTLC1 | 1.163E-05 | -0.239 | - | - |
| 12236 | ENSP00000351769 | DUS2L | 9.453E-06 | -0.239 | - | - |
| 12237 | ENSP00000333595 | ZNF285 | 1.570E-05 | -0.239 | - | - |
| 12238 | ENSP00000353098 | MCMBP | 7.914E-06 | -0.239 | - | - |
| 12239 | ENSP00000276692 | TATDN1 | 1.946E-05 | -0.239 | - | - |
| 12240 | ENSP00000262144 | WDR59 | 1.901E-05 | -0.239 | - | - |
| 12241 | ENSP00000263121 | SMARCB1 | 1.779E-05 | -0.239 | - | - |
| 12242 | ENSP00000398798 | ZNF142 | 1.286E-05 | -0.239 | - | - |
| 12243 | ENSP00000315644 | TYMS | 2.277E-05 | -0.239 | - | - |
| 12244 | ENSP00000234396 | ATP6V1B1 | 9.780E-06 | -0.239 | - | - |
| 12245 | ENSP00000229447 | IYD | 1.925E-05 | -0.239 | - | - |
| 12246 | ENSP00000264474 | CSTA | 3.746E-05 | -0.239 | - | - |
| 12247 | ENSP00000291634 | FAM207A | 5.193E-05 | -0.239 | - | - |
| 12248 | ENSP00000269143 | AFG3L2 | 1.225E-05 | -0.239 | - | - |
| 12249 | ENSP00000336552 | PKIA | 1.350E-05 | -0.239 | - | - |
| 12250 | ENSP00000391774 | CPSF6 | 1.146E-05 | -0.239 | - | - |
| 12251 | ENSP00000292614 | POLR2J | 1.203E-05 | -0.239 | - | - |
| 12252 | ENSP00000335287 | ANKRD46 | 1.099E-05 | -0.239 | - | - |
| 12253 | ENSP00000348602 | AMPH | 2.344E-05 | -0.239 | - | - |
| 12254 | ENSP00000219789 | CDIPT | 1.282E-05 | -0.239 | - | - |
| 12255 | ENSP00000232974 | ZBTB47 | 1.741E-05 | -0.239 | - | - |
| 12256 | ENSP00000370968 | MYBBP1A | 1.706E-05 | -0.239 | - | - |
| 12257 | ENSP00000304782 | CES3 | 1.231E-05 | -0.239 | - | - |
| 12258 | ENSP00000296223 | POLR2H | 1.011E-05 | -0.239 | - | - |
| 12259 | ENSP00000372347 | WHSC1 | 2.004E-05 | -0.239 | - | - |
| 12260 | ENSP00000356621 | RASAL2 | 1.983E-05 | -0.239 | - | - |
| 12261 | ENSP00000319829 | ZNF687 | 1.709E-05 | -0.239 | - | - |
| 12262 | ENSP00000321963 | ZSCAN4 | 1.577E-05 | -0.239 | - | - |
| 12263 | ENSP00000339314 | ZNF70 | 1.562E-05 | -0.239 | - | - |
| 12264 | ENSP00000268154 | ZNF710 | 1.646E-05 | -0.239 | - | - |
| 12265 | ENSP00000325425 | MAT2B | 3.099E-05 | -0.239 | - | - |
| 12266 | ENSP00000307940 | EEF2 | 2.535E-05 | -0.239 | - | - |
| 12267 | ENSP00000292841 | ZNF585A | 1.591E-05 | -0.239 | - | - |
| 12268 | ENSP00000344129 | ZNF648 | 1.711E-05 | -0.239 | - | - |
| 12269 | ENSP00000275300 | SLC22A3 | 1.482E-05 | -0.239 | - | - |
| 12270 | ENSP00000338774 | BCL11A | 2.977E-05 | -0.239 | - | - |
| 12271 | ENSP00000329738 | ZSCAN30 | 1.600E-05 | -0.239 | - | - |
| 12272 | ENSP00000335437 | ZNF20 | 1.606E-05 | -0.239 | - | - |
| 12273 | ENSP00000304985 | ZFX | 1.677E-05 | -0.239 | - | - |
| 12274 | ENSP00000327984 | TSPY8 | 1.441E-05 | -0.239 | - | - |
| 12275 | ENSP00000398481 | CADPS2 | 9.735E-06 | -0.239 | - | - |
| 12276 | ENSP00000333208 | C2orf82 | 2.777E-05 | -0.239 | - | - |
| 12277 | ENSP00000266743 | SYCP3 | 1.376E-05 | -0.239 | - | - |
| 12278 | ENSP00000315214 | RP11-429E11.3 | 2.731E-05 | -0.239 | - | - |
| 12279 | ENSP00000324636 | VRK3 | 2.325E-05 | -0.239 | - | - |
| 12280 | ENSP00000219611 | SOLH | 8.415E-06 | -0.239 | - | - |
| 12281 | ENSP00000321049 | ZNF227 | 1.680E-05 | -0.239 | - | - |
| 12282 | ENSP00000367428 | MS4A13 | 2.479E-05 | -0.239 | - | - |
| 12283 | ENSP00000359925 | LRRC1 | 1.402E-05 | -0.239 | - | - |
| 12284 | ENSP00000221494 | SF3A2 | 1.404E-05 | -0.239 | - | - |
| 12285 | ENSP00000262032 | IKZF4 | 1.605E-05 | -0.239 | - | - |
| 12286 | ENSP00000261170 | GUCY2C | 8.049E-06 | -0.239 | - | - |
| 12287 | ENSP00000331310 | MORF4L1 | 1.719E-05 | -0.239 | - | - |
| 12288 | ENSP00000340004 | ZNF564 | 1.556E-05 | -0.239 | - | - |
| 12289 | ENSP00000313408 | TMC6 | 3.936E-05 | -0.239 | - | - |
| 12290 | ENSP00000330485 | ZDHHC23 | 2.158E-05 | -0.239 | - | - |
| 12291 | ENSP00000255084 | ALDH3B2 | 7.870E-06 | -0.239 | - | - |
| 12292 | ENSP00000282292 | ZNF773 | 1.427E-05 | -0.239 | - | - |
| 12293 | ENSP00000346892 | SERF1A | 4.621E-05 | -0.239 | - | - |
| 12294 | ENSP00000462337 | VEZF1 | 1.626E-05 | -0.239 | - | - |
| 12295 | ENSP00000337641 | PPP2R5E | 5.589E-05 | -0.239 | - | - |
| 12296 | ENSP00000361800 | WDR34 | 1.896E-05 | -0.239 | - | - |
| 12297 | ENSP00000320096 | ZNF784 | 1.696E-05 | -0.240 | - | - |
| 12298 | ENSP00000341528 | ZNF34 | 1.593E-05 | -0.240 | - | - |
| 12299 | ENSP00000302502 | ZSCAN32 | 1.620E-05 | -0.240 | - | - |
| 12300 | ENSP00000336856 | HMG20A | 2.224E-05 | -0.240 | - | - |
| 12301 | ENSP00000310472 | ZNF624 | 1.584E-05 | -0.240 | - | - |
| 12302 | ENSP00000355173 | ATG9A | 1.334E-05 | -0.240 | - | - |
| 12303 | ENSP00000301093 | ZNF701 | 1.381E-05 | -0.240 | - | - |
| 12304 | ENSP00000262429 | ATP2C2 | 1.120E-05 | -0.240 | - | - |
| 12305 | ENSP00000381273 | CRYBB2 | 1.685E-05 | -0.240 | - | - |
| 12306 | ENSP00000311343 | WDR49 | 2.101E-05 | -0.240 | - | - |
| 12307 | ENSP00000286091 | PDIA4 | 1.820E-05 | -0.240 | - | - |
| 12308 | ENSP00000357068 | KCNJ10 | 1.997E-05 | -0.240 | - | - |
| 12309 | ENSP00000324064 | ZNF354C | 2.159E-05 | -0.240 | - | - |
| 12310 | ENSP00000264279 | NOP58 | 5.177E-06 | -0.240 | - | - |
| 12311 | ENSP00000256079 | IPO8 | 1.014E-05 | -0.240 | - | - |
| 12312 | ENSP00000339145 | RRP1B | 9.942E-06 | -0.240 | - | - |
| 12313 | ENSP00000296326 | ZDHHC19 | 1.825E-05 | -0.240 | - | - |
| 12314 | ENSP00000380041 | INCA1 | 1.398E-05 | -0.240 | - | - |
| 12315 | ENSP00000262894 | ZNF225 | 1.697E-05 | -0.240 | - | - |
| 12316 | ENSP00000321679 | ANKRD23 | 1.140E-05 | -0.240 | - | - |
| 12317 | ENSP00000410758 | LSM5 | 1.558E-05 | -0.240 | - | - |
| 12318 | ENSP00000287394 | ATAD2 | 1.357E-05 | -0.240 | - | - |
| 12319 | ENSP00000247956 | ZNF317 | 1.707E-05 | -0.240 | - | - |
| 12320 | ENSP00000254166 | ZNF132 | 1.621E-05 | -0.240 | - | - |
| 12321 | ENSP00000211936 | ZNF184 | 1.454E-05 | -0.240 | - | - |
| 12322 | ENSP00000342374 | SNRPD2 | 1.999E-05 | -0.240 | - | - |
| 12323 | ENSP00000306866 | GABARAP | 1.352E-05 | -0.240 | - | - |
| 12324 | ENSP00000309570 | KLF7 | 1.653E-05 | -0.240 | - | - |
| 12325 | ENSP00000291182 | ZNF235 | 2.020E-05 | -0.240 | - | - |
| 12326 | ENSP00000260361 | NDUFAF1 | 2.162E-05 | -0.240 | - | - |
| 12327 | ENSP00000402460 | CLSTN2 | 4.352E-05 | -0.240 | - | - |
| 12328 | ENSP00000356918 | STX7 | 1.220E-05 | -0.240 | - | - |
| 12329 | ENSP00000327801 | P4HB | 2.974E-05 | -0.240 | - | - |
| 12330 | ENSP00000258886 | IREB2 | 1.757E-05 | -0.240 | - | - |
| 12331 | ENSP00000374467 | ABCC8 | 2.865E-05 | -0.240 | - | - |
| 12332 | ENSP00000299138 | VPS35 | 1.335E-05 | -0.240 | - | - |
| 12333 | ENSP00000261200 | ABCC9 | 1.695E-05 | -0.240 | - | - |
| 12334 | ENSP00000351717 | ABCB8 | 1.404E-05 | -0.240 | - | - |
| 12335 | ENSP00000413929 | RCOR3 | 9.640E-06 | -0.240 | - | - |
| 12336 | ENSP00000325548 | CNDP2 | 3.293E-05 | -0.240 | - | - |
| 12337 | ENSP00000315554 | SPEM1 | 7.728E-06 | -0.240 | - | - |
| 12338 | ENSP00000265148 | CENPE | 1.064E-05 | -0.240 | - | - |
| 12339 | ENSP00000268668 | NDUFB10 | 6.993E-06 | -0.240 | - | - |
| 12340 | ENSP00000322957 | PAK7 | 2.681E-05 | -0.240 | - | - |
| 12341 | ENSP00000325136 | HADHB | 2.022E-05 | -0.240 | - | - |
| 12342 | ENSP00000281741 | TXNDC16 | 7.462E-06 | -0.240 | - | - |
| 12343 | ENSP00000320627 | ZNF7 | 1.726E-05 | -0.240 | - | - |
| 12344 | ENSP00000306752 | BOLA2B | 1.121E-05 | -0.240 | - | - |
| 12345 | ENSP00000313582 | ZNF436 | 2.409E-05 | -0.240 | - | - |
| 12346 | ENSP00000366908 | - | 1.479E-05 | -0.240 | - | - |
| 12347 | ENSP00000327467 | SMDT1 | 3.789E-05 | -0.240 | - | - |
| 12348 | ENSP00000362773 | PIN4 | 8.641E-06 | -0.240 | - | - |
| 12349 | ENSP00000308610 | GPD2 | 1.461E-05 | -0.240 | - | - |
| 12350 | ENSP00000254940 | NIP7 | 6.348E-06 | -0.240 | - | - |
| 12351 | ENSP00000398597 | EXOSC6 | 9.313E-06 | -0.240 | - | - |
| 12352 | ENSP00000347504 | ITGB1BP1 | 1.894E-05 | -0.240 | - | - |
| 12353 | ENSP00000276390 | ATP6V1B2 | 1.114E-05 | -0.240 | - | - |
| 12354 | ENSP00000381636 | PCDHA11 | 1.362E-05 | -0.240 | - | - |
| 12355 | ENSP00000306920 | GLB1 | 2.211E-05 | -0.240 | - | - |
| 12356 | ENSP00000438346 | REEP1 | 5.878E-06 | -0.240 | - | - |
| 12357 | ENSP00000275605 | PSPH | 1.001E-05 | -0.240 | - | - |
| 12358 | ENSP00000354061 | SCYL2 | 1.598E-05 | -0.240 | - | - |
| 12359 | ENSP00000243914 | CTCFL | 1.684E-05 | -0.240 | - | - |
| 12360 | ENSP00000329932 | LGALS9C | 2.342E-05 | -0.240 | - | - |
| 12361 | ENSP00000254765 | POPDC3 | 2.824E-05 | -0.241 | - | - |
| 12362 | ENSP00000367498 | LRRC47 | 1.641E-05 | -0.241 | - | - |
| 12363 | ENSP00000228850 | AKAP3 | 1.015E-05 | -0.241 | - | - |
| 12364 | ENSP00000299608 | TMX3 | 1.562E-05 | -0.241 | - | - |
| 12365 | ENSP00000344162 | ZNF136 | 1.488E-05 | -0.241 | - | - |
| 12366 | ENSP00000265896 | SQLE | 9.861E-06 | -0.241 | - | - |
| 12367 | ENSP00000349640 | MCRS1 | 2.486E-05 | -0.241 | - | - |
| 12368 | ENSP00000308944 | HLTF | 2.143E-05 | -0.241 | - | - |
| 12369 | ENSP00000344791 | ZNF600 | 1.617E-05 | -0.241 | - | - |
| 12370 | ENSP00000301972 | MYRIP | 2.559E-05 | -0.241 | - | - |
| 12371 | ENSP00000273283 | ITIH1 | 2.145E-05 | -0.241 | - | - |
| 12372 | ENSP00000377112 | SLC12A8 | 1.897E-05 | -0.241 | - | - |
| 12373 | ENSP00000295208 | ZNF514 | 1.627E-05 | -0.241 | - | - |
| 12374 | ENSP00000282308 | ZNF256 | 1.567E-05 | -0.241 | - | - |
| 12375 | ENSP00000315700 | CHAF1B | 1.174E-05 | -0.241 | - | - |
| 12376 | ENSP00000272223 | OSR1 | 1.800E-05 | -0.241 | - | - |
| 12377 | ENSP00000259569 | RANBP6 | 1.409E-05 | -0.241 | - | - |
| 12378 | ENSP00000305675 | SDPR | 1.928E-05 | -0.241 | - | - |
| 12379 | ENSP00000291715 | CLDND2 | 3.386E-05 | -0.241 | - | - |
| 12380 | ENSP00000334853 | ZNF555 | 1.509E-05 | -0.241 | - | - |
| 12381 | ENSP00000262584 | RPL8 | 6.430E-06 | -0.241 | - | - |
| 12382 | ENSP00000262518 | SRCAP | 1.026E-05 | -0.241 | - | - |
| 12383 | ENSP00000428316 | SERPINE3 | 7.272E-06 | -0.241 | - | - |
| 12384 | ENSP00000308179 | WDR3 | 8.523E-06 | -0.241 | - | - |
| 12385 | ENSP00000336687 | CBX3 | 2.153E-05 | -0.241 | - | - |
| 12386 | ENSP00000315664 | ZNF18 | 1.439E-05 | -0.241 | - | - |
| 12387 | ENSP00000253003 | ADRM1 | 2.495E-05 | -0.241 | - | - |
| 12388 | ENSP00000266719 | KERA | 1.462E-05 | -0.241 | - | - |
| 12389 | ENSP00000300413 | SNRPD1 | 1.892E-05 | -0.241 | - | - |
| 12390 | ENSP00000331313 | GNB1L | 2.418E-05 | -0.241 | - | - |
| 12391 | ENSP00000304429 | SNX7 | 9.000E-06 | -0.241 | - | - |
| 12392 | ENSP00000305804 | ZNF131 | 1.928E-05 | -0.241 | - | - |
| 12393 | ENSP00000358994 | MYO6 | 2.404E-05 | -0.241 | - | - |
| 12394 | ENSP00000334188 | PFDN5 | 1.236E-05 | -0.241 | - | - |
| 12395 | ENSP00000288207 | CCNB2 | 2.286E-05 | -0.241 | - | - |
| 12396 | ENSP00000321848 | ZNF671 | 1.487E-05 | -0.241 | - | - |
| 12397 | ENSP00000310033 | ZNF596 | 1.564E-05 | -0.241 | - | - |
| 12398 | ENSP00000332256 | ALDH1A3 | 1.842E-05 | -0.241 | - | - |
| 12399 | ENSP00000322427 | ZNF611 | 1.463E-05 | -0.241 | - | - |
| 12400 | ENSP00000431265 | ZFP82 | 1.409E-05 | -0.241 | - | - |
| 12401 | ENSP00000363747 | ZNF33A | 1.798E-05 | -0.241 | - | - |
| 12402 | ENSP00000216036 | RTDR1 | 1.429E-05 | -0.241 | - | - |
| 12403 | ENSP00000407130 | ZNF410 | 1.643E-05 | -0.241 | - | - |
| 12404 | ENSP00000314649 | ALDH5A1 | 1.343E-05 | -0.241 | - | - |
| 12405 | ENSP00000359768 | KIAA1586 | 1.129E-05 | -0.241 | - | - |
| 12406 | ENSP00000311873 | EXO1 | 1.326E-05 | -0.241 | - | - |
| 12407 | ENSP00000325863 | MRE11A | 1.754E-05 | -0.241 | - | - |
| 12408 | ENSP00000335388 | KIR3DX1 | 2.027E-05 | -0.241 | - | - |
| 12409 | ENSP00000220849 | EIF3E | 2.545E-05 | -0.241 | - | - |
| 12410 | ENSP00000223500 | CHMP5 | 6.161E-06 | -0.241 | - | - |
| 12411 | ENSP00000266503 | ARNTL2 | 3.007E-05 | -0.241 | - | - |
| 12412 | ENSP00000258538 | SLC41A2 | 1.918E-05 | -0.241 | - | - |
| 12413 | ENSP00000294543 | TMCO4 | 6.537E-06 | -0.241 | - | - |
| 12414 | ENSP00000341562 | B4GALNT1 | 1.813E-05 | -0.241 | - | - |
| 12415 | ENSP00000275189 | - | 2.435E-05 | -0.241 | - | - |
| 12416 | ENSP00000355465 | AHCTF1 | 1.649E-05 | -0.241 | - | - |
| 12417 | ENSP00000264380 | PIKFYVE | 2.163E-05 | -0.241 | - | - |
| 12418 | ENSP00000383365 | TXNRD2 | 3.092E-05 | -0.242 | - | - |
| 12419 | ENSP00000317614 | ZNF518B | 1.510E-05 | -0.242 | - | - |
| 12420 | ENSP00000333776 | ZNF479 | 1.496E-05 | -0.242 | - | - |
| 12421 | ENSP00000315505 | HKR1 | 1.555E-05 | -0.242 | - | - |
| 12422 | ENSP00000290597 | ALDH4A1 | 9.576E-06 | -0.242 | - | - |
| 12423 | ENSP00000324598 | ZNF540 | 1.656E-05 | -0.242 | - | - |
| 12424 | ENSP00000352456 | SLC33A1 | 2.288E-05 | -0.242 | - | - |
| 12425 | ENSP00000308578 | ZNF431 | 1.535E-05 | -0.242 | - | - |
| 12426 | ENSP00000297012 | HIST1H2AA | 1.954E-05 | -0.242 | - | - |
| 12427 | ENSP00000245934 | SYMPK | 1.844E-05 | -0.242 | - | - |
| 12428 | ENSP00000283025 | TEKT5 | 2.161E-05 | -0.242 | - | - |
| 12429 | ENSP00000268482 | DHX38 | 8.282E-06 | -0.242 | - | - |
| 12430 | ENSP00000357402 | NKAIN2 | 1.474E-05 | -0.242 | - | - |
| 12431 | ENSP00000301995 | SCAND1 | 1.485E-05 | -0.242 | - | - |
| 12432 | ENSP00000216445 | C14orf105 | 4.228E-05 | -0.242 | - | - |
| 12433 | ENSP00000253270 | SLC35D2 | 2.122E-05 | -0.242 | - | - |
| 12434 | ENSP00000319690 | HNRNPC | 2.217E-05 | -0.242 | - | - |
| 12435 | ENSP00000341826 | HNRNPA1 | 2.926E-05 | -0.242 | - | - |
| 12436 | ENSP00000340799 | SP6 | 3.730E-05 | -0.242 | - | - |
| 12437 | ENSP00000301788 | POLR2G | 9.860E-06 | -0.242 | - | - |
| 12438 | ENSP00000307697 | ACOX2 | 8.026E-06 | -0.242 | - | - |
| 12439 | ENSP00000384892 | RIMS2 | 3.275E-05 | -0.242 | - | - |
| 12440 | ENSP00000262030 | ATP5B | 1.168E-05 | -0.242 | - | - |
| 12441 | ENSP00000420093 | - | 3.273E-06 | -0.242 | - | - |
| 12442 | ENSP00000326052 | COX14 | 3.833E-05 | -0.242 | - | - |
| 12443 | ENSP00000319104 | SUPT6H | 9.617E-06 | -0.242 | - | - |
| 12444 | ENSP00000287169 | ZDHHC5 | 1.673E-05 | -0.242 | - | - |
| 12445 | ENSP00000329471 | KDELR1 | 1.176E-05 | -0.242 | - | - |
| 12446 | ENSP00000261574 | IPO5 | 1.306E-05 | -0.242 | - | - |
| 12447 | ENSP00000282296 | ZNF551 | 1.636E-05 | -0.242 | - | - |
| 12448 | ENSP00000349175 | FAM180B | 7.069E-06 | -0.242 | - | - |
| 12449 | ENSP00000318986 | NSUN3 | 8.494E-06 | -0.242 | - | - |
| 12450 | ENSP00000254976 | SNAP25 | 1.438E-05 | -0.242 | - | - |
| 12451 | ENSP00000372482 | SNRNP25 | 7.192E-06 | -0.242 | - | - |
| 12452 | ENSP00000300504 | TBC1D21 | 1.493E-05 | -0.242 | - | - |
| 12453 | ENSP00000353699 | WDR72 | 2.142E-05 | -0.242 | - | - |
| 12454 | ENSP00000417686 | SLBP | 9.892E-06 | -0.242 | - | - |
| 12455 | ENSP00000331626 | ZKSCAN2 | 1.721E-05 | -0.242 | - | - |
| 12456 | ENSP00000221315 | ZNF432 | 1.610E-05 | -0.242 | - | - |
| 12457 | ENSP00000305077 | ZNF439 | 1.328E-05 | -0.242 | - | - |
| 12458 | ENSP00000217402 | CHMP4B | 8.170E-06 | -0.242 | - | - |
| 12459 | ENSP00000314380 | GRINA | 9.508E-06 | -0.242 | - | - |
| 12460 | ENSP00000362629 | DMRTC1 | 2.532E-05 | -0.242 | - | - |
| 12461 | ENSP00000284049 | CHD1 | 1.459E-05 | -0.242 | - | - |
| 12462 | ENSP00000383923 | LIPN | 2.556E-05 | -0.242 | - | - |
| 12463 | ENSP00000383692 | APITD1-CORT | 4.855E-06 | -0.242 | - | - |
| 12464 | ENSP00000337368 | ZNF224 | 1.434E-05 | -0.242 | - | - |
| 12465 | ENSP00000356146 | SCAF8 | 8.660E-06 | -0.242 | - | - |
| 12466 | ENSP00000299194 | LRTM2 | 1.828E-05 | -0.242 | - | - |
| 12467 | ENSP00000405987 | LRRD1 | 7.344E-06 | -0.242 | - | - |
| 12468 | ENSP00000364370 | C20orf144 | 1.324E-05 | -0.242 | - | - |
| 12469 | ENSP00000358095 | C1orf54 | 9.743E-06 | -0.242 | - | - |
| 12470 | ENSP00000332750 | ZKSCAN8 | 1.525E-05 | -0.242 | - | - |
| 12471 | ENSP00000342818 | ZNF669 | 1.526E-05 | -0.242 | - | - |
| 12472 | ENSP00000329264 | ZNF662 | 1.510E-05 | -0.242 | - | - |
| 12473 | ENSP00000265112 | TARS | 8.636E-06 | -0.242 | - | - |
| 12474 | ENSP00000294288 | CABP2 | 1.182E-05 | -0.242 | - | - |
| 12475 | ENSP00000267814 | SORD | 2.103E-05 | -0.242 | - | - |
| 12476 | ENSP00000302620 | AGXT | 1.653E-05 | -0.242 | - | - |
| 12477 | ENSP00000307858 | ZBTB4 | 1.651E-05 | -0.242 | - | - |
| 12478 | ENSP00000323945 | ZNF771 | 1.547E-05 | -0.242 | - | - |
| 12479 | ENSP00000261438 | KLF3 | 1.577E-05 | -0.242 | - | - |
| 12480 | ENSP00000313423 | GFRA4 | 3.039E-05 | -0.242 | - | - |
| 12481 | ENSP00000358454 | F8A3 | 1.616E-05 | -0.243 | - | - |
| 12482 | ENSP00000376808 | CUL5 | 1.638E-05 | -0.243 | - | - |
| 12483 | ENSP00000341730 | RPL10 | 6.119E-06 | -0.243 | - | - |
| 12484 | ENSP00000338290 | GZF1 | 1.621E-05 | -0.243 | - | - |
| 12485 | ENSP00000336741 | DHX15 | 1.410E-05 | -0.243 | - | - |
| 12486 | ENSP00000324842 | AACS | 1.059E-05 | -0.243 | - | - |
| 12487 | ENSP00000293350 | ALDH16A1 | 8.995E-06 | -0.243 | - | - |
| 12488 | ENSP00000330054 | EEF1A1 | 4.547E-05 | -0.243 | - | - |
| 12489 | ENSP00000344782 | GFI1B | 1.892E-05 | -0.243 | - | - |
| 12490 | ENSP00000343515 | TCEA2 | 9.888E-06 | -0.243 | - | - |
| 12491 | ENSP00000334738 | PITPNB | 9.984E-06 | -0.243 | - | - |
| 12492 | ENSP00000344331 | FAM69C | 4.433E-06 | -0.243 | - | - |
| 12493 | ENSP00000272133 | CNIH3 | 1.898E-05 | -0.243 | - | - |
| 12494 | ENSP00000264345 | HERC3 | 2.640E-05 | -0.243 | - | - |
| 12495 | ENSP00000383938 | CRELD2 | 1.505E-05 | -0.243 | - | - |
| 12496 | ENSP00000332790 | HIST2H2AB | 1.691E-05 | -0.243 | - | - |
| 12497 | ENSP00000355001 | POU3F3 | 1.785E-05 | -0.243 | - | - |
| 12498 | ENSP00000246529 | LRFN3 | 1.477E-05 | -0.243 | - | - |
| 12499 | ENSP00000302770 | ZNF296 | 1.459E-05 | -0.243 | - | - |
| 12500 | ENSP00000360497 | RNF113A | 2.514E-05 | -0.243 | - | - |
| 12501 | ENSP00000367802 | TAF1C | 1.897E-05 | -0.243 | - | - |
| 12502 | ENSP00000362588 | PBX3 | 2.818E-05 | -0.243 | - | - |
| 12503 | ENSP00000366058 | SYNDIG1 | 7.184E-06 | -0.243 | - | - |
| 12504 | ENSP00000423600 | MTX3 | 2.570E-05 | -0.243 | - | - |
| 12505 | ENSP00000371126 | NUTM2A | 1.165E-05 | -0.243 | - | - |
| 12506 | ENSP00000313603 | ABCF1 | 8.518E-06 | -0.243 | - | - |
| 12507 | ENSP00000339767 | ZNF433 | 1.702E-05 | -0.243 | - | - |
| 12508 | ENSP00000301073 | ZNF524 | 1.550E-05 | -0.243 | - | - |
| 12509 | ENSP00000419126 | C10orf88 | 3.540E-06 | -0.243 | - | - |
| 12510 | ENSP00000363667 | PTPN3 | 2.302E-05 | -0.243 | - | - |
| 12511 | ENSP00000239440 | ARAP3 | 1.861E-05 | -0.243 | - | - |
| 12512 | ENSP00000219478 | ZNF500 | 1.492E-05 | -0.243 | - | - |
| 12513 | ENSP00000301547 | ZNF443 | 1.425E-05 | -0.243 | - | - |
| 12514 | ENSP00000459216 | ZNF578 | 9.189E-06 | -0.243 | - | - |
| 12515 | ENSP00000416959 | TRA2B | 9.468E-06 | -0.243 | - | - |
| 12516 | ENSP00000219302 | NME3 | 8.282E-06 | -0.243 | - | - |
| 12517 | ENSP00000299293 | FRS2 | 2.230E-05 | -0.243 | - | - |
| 12518 | ENSP00000327197 | SENP5 | 2.587E-05 | -0.243 | - | - |
| 12519 | ENSP00000369440 | TRIM6 | 2.231E-05 | -0.243 | - | - |
| 12520 | ENSP00000325941 | RIC8A | 1.747E-05 | -0.243 | - | - |
| 12521 | ENSP00000229971 | FBXL4 | 7.279E-06 | -0.243 | - | - |
| 12522 | ENSP00000226207 | MYH1 | 1.319E-05 | -0.243 | - | - |
| 12523 | ENSP00000338019 | DNAJB2 | 1.743E-05 | -0.243 | - | - |
| 12524 | ENSP00000158762 | ACAP1 | 1.328E-05 | -0.243 | - | - |
| 12525 | ENSP00000223210 | ZNF862 | 1.406E-05 | -0.243 | - | - |
| 12526 | ENSP00000338785 | STARD13 | 2.841E-05 | -0.243 | - | - |
| 12527 | ENSP00000365145 | FOXS1 | 8.995E-06 | -0.243 | - | - |
| 12528 | ENSP00000336712 | TNPO1 | 1.831E-05 | -0.243 | - | - |
| 12529 | ENSP00000324441 | ZNF610 | 1.482E-05 | -0.243 | - | - |
| 12530 | ENSP00000430269 | EPHX2 | 2.961E-05 | -0.243 | - | - |
| 12531 | ENSP00000377583 | KRTAP2-4 | 3.772E-06 | -0.243 | - | - |
| 12532 | ENSP00000391504 | CCDC173 | 3.613E-07 | -0.243 | - | - |
| 12533 | ENSP00000472005 | HBCBP | 1.364E-06 | -0.243 | - | - |
| 12534 | ENSP00000432799 | MANF | 2.883E-05 | -0.243 | - | - |
| 12535 | ENSP00000298699 | C12orf50 | 3.173E-05 | -0.243 | - | - |
| 12536 | ENSP00000304908 | TXNDC2 | 9.554E-06 | -0.243 | - | - |
| 12537 | ENSP00000311957 | ZNF738 | 1.643E-05 | -0.243 | - | - |
| 12538 | ENSP00000337081 | ZNF112 | 1.561E-05 | -0.243 | - | - |
| 12539 | ENSP00000338807 | ZBTB49 | 1.441E-05 | -0.243 | - | - |
| 12540 | ENSP00000279249 | CDC42EP2 | 4.438E-05 | -0.243 | - | - |
| 12541 | ENSP00000416290 | C7orf25 | 1.432E-05 | -0.243 | - | - |
| 12542 | ENSP00000364180 | GPSM3 | 6.010E-06 | -0.243 | - | - |
| 12543 | ENSP00000435096 | RPS25 | 1.039E-05 | -0.243 | - | - |
| 12544 | ENSP00000362870 | MATN1 | 1.718E-05 | -0.243 | - | - |
| 12545 | ENSP00000328551 | RAB28 | 7.398E-06 | -0.243 | - | - |
| 12546 | ENSP00000278590 | ZC3H12C | 3.021E-05 | -0.244 | - | - |
| 12547 | ENSP00000306991 | HAS2 | 3.380E-05 | -0.244 | - | - |
| 12548 | ENSP00000388872 | CLUH | 2.250E-05 | -0.244 | - | - |
| 12549 | ENSP00000429969 | SCUBE2 | 2.437E-05 | -0.244 | - | - |
| 12550 | ENSP00000358894 | CNNM2 | 1.324E-05 | -0.244 | - | - |
| 12551 | ENSP00000398064 | HIGD1A | 8.967E-06 | -0.244 | - | - |
| 12552 | ENSP00000253233 | C12orf65 | 7.526E-06 | -0.244 | - | - |
| 12553 | ENSP00000325448 | KARS | 1.993E-05 | -0.244 | - | - |
| 12554 | ENSP00000292450 | ZSCAN21 | 1.632E-05 | -0.244 | - | - |
| 12555 | ENSP00000353025 | FAM3C | 2.481E-05 | -0.244 | - | - |
| 12556 | ENSP00000366962 | HIST1H2BI | 2.510E-05 | -0.244 | - | - |
| 12557 | ENSP00000364260 | C9orf156 | 1.404E-05 | -0.244 | - | - |
| 12558 | ENSP00000325326 | ZNF335 | 1.741E-05 | -0.244 | - | - |
| 12559 | ENSP00000357494 | ROS1 | 2.576E-05 | -0.244 | - | - |
| 12560 | ENSP00000349430 | HIST1H2BK | 1.925E-05 | -0.244 | - | - |
| 12561 | ENSP00000385795 | PHF14 | 4.951E-06 | -0.244 | - | - |
| 12562 | ENSP00000335636 | NAA20 | 1.443E-05 | -0.244 | - | - |
| 12563 | ENSP00000299237 | ZNF319 | 1.566E-05 | -0.244 | - | - |
| 12564 | ENSP00000348337 | C13orf35 | 6.086E-06 | -0.244 | - | - |
| 12565 | ENSP00000306223 | SNRPN | 1.630E-05 | -0.244 | - | - |
| 12566 | ENSP00000220913 | CHRAC1 | 9.718E-06 | -0.244 | - | - |
| 12567 | ENSP00000263381 | WIZ | 1.487E-05 | -0.244 | - | - |
| 12568 | ENSP00000264065 | DNAJC10 | 1.002E-05 | -0.244 | - | - |
| 12569 | ENSP00000221543 | TBC1D17 | 1.418E-05 | -0.244 | - | - |
| 12570 | ENSP00000233084 | DDX1 | 1.019E-05 | -0.244 | - | - |
| 12571 | ENSP00000216144 | CABP7 | 8.255E-06 | -0.244 | - | - |
| 12572 | ENSP00000407425 | - | 2.864E-07 | -0.244 | - | - |
| 12573 | ENSP00000361666 | SLC22A7 | 1.188E-05 | -0.244 | - | - |
| 12574 | ENSP00000365844 | TRIM39 | 2.398E-05 | -0.244 | - | - |
| 12575 | ENSP00000369755 | C2CD4B | 1.138E-05 | -0.244 | - | - |
| 12576 | ENSP00000261596 | LPIN2 | 1.398E-05 | -0.244 | - | - |
| 12577 | ENSP00000326630 | ZFPM1 | 1.893E-05 | -0.244 | - | - |
| 12578 | ENSP00000217073 | PABPC1L | 1.682E-05 | -0.244 | - | - |
| 12579 | ENSP00000334499 | PTBP3 | 2.684E-05 | -0.244 | - | - |
| 12580 | ENSP00000343709 | SNX10 | 5.131E-05 | -0.244 | - | - |
| 12581 | ENSP00000344308 | ZNF341 | 1.552E-05 | -0.244 | - | - |
| 12582 | ENSP00000314153 | ZBTB33 | 1.443E-05 | -0.244 | - | - |
| 12583 | ENSP00000430073 | CNBD1 | 4.356E-07 | -0.244 | - | - |
| 12584 | ENSP00000322872 | ZKSCAN5 | 1.435E-05 | -0.244 | - | - |
| 12585 | ENSP00000340989 | SFN | 2.678E-05 | -0.244 | - | - |
| 12586 | ENSP00000335144 | TPGS2 | 1.712E-05 | -0.244 | - | - |
| 12587 | ENSP00000374328 | FAXC | 1.936E-05 | -0.244 | - | - |
| 12588 | ENSP00000295887 | CDS1 | 8.673E-06 | -0.244 | - | - |
| 12589 | ENSP00000318898 | ZNF114 | 1.447E-05 | -0.244 | - | - |
| 12590 | ENSP00000264454 | SEC22C | 1.881E-05 | -0.244 | - | - |
| 12591 | ENSP00000472039 | LSP1 | 1.778E-05 | -0.244 | - | - |
| 12592 | ENSP00000311876 | GNPDA1 | 1.179E-05 | -0.244 | - | - |
| 12593 | ENSP00000377823 | NDRG4 | 2.010E-05 | -0.244 | - | - |
| 12594 | ENSP00000431905 | FAM71D | 2.494E-06 | -0.245 | - | - |
| 12595 | ENSP00000327764 | C22orf26 | 2.827E-05 | -0.245 | - | - |
| 12596 | ENSP00000405812 | SLC4A8 | 2.186E-05 | -0.245 | - | - |
| 12597 | ENSP00000232975 | TNNC1 | 8.630E-06 | -0.245 | - | - |
| 12598 | ENSP00000325038 | ZNF836 | 1.411E-05 | -0.245 | - | - |
| 12599 | ENSP00000319597 | SNAPC5 | 2.083E-05 | -0.245 | - | - |
| 12600 | ENSP00000391594 | ERV3-1 | 7.848E-06 | -0.245 | - | - |
| 12601 | ENSP00000267273 | METTL21C | 1.061E-05 | -0.245 | - | - |
| 12602 | ENSP00000337473 | ZNF324B | 1.474E-05 | -0.245 | - | - |
| 12603 | ENSP00000359609 | LMBRD1 | 2.295E-05 | -0.245 | - | - |
| 12604 | ENSP00000333223 | ZNF470 | 2.235E-05 | -0.245 | - | - |
| 12605 | ENSP00000264192 | CYTIP | 2.777E-05 | -0.245 | - | - |
| 12606 | ENSP00000278314 | ZNF214 | 1.637E-05 | -0.245 | - | - |
| 12607 | ENSP00000319417 | SKA3 | 1.434E-05 | -0.245 | - | - |
| 12608 | ENSP00000365811 | SPAG6 | 3.084E-05 | -0.245 | - | - |
| 12609 | ENSP00000401362 | C15orf41 | 1.877E-05 | -0.245 | - | - |
| 12610 | ENSP00000461728 | RP11-212D19.4 | 2.977E-05 | -0.245 | - | - |
| 12611 | ENSP00000401721 | PAN2 | 9.713E-06 | -0.245 | - | - |
| 12612 | ENSP00000216727 | PABPN1 | 2.238E-05 | -0.245 | - | - |
| 12613 | ENSP00000269834 | ZIM3 | 1.730E-05 | -0.245 | - | - |
| 12614 | ENSP00000261667 | KPNA3 | 1.569E-05 | -0.245 | - | - |
| 12615 | ENSP00000340667 | C11orf96 | 1.532E-05 | -0.245 | - | - |
| 12616 | ENSP00000321507 | AZIN1 | 1.306E-05 | -0.245 | - | - |
| 12617 | ENSP00000253099 | MRPL4 | 5.997E-06 | -0.245 | - | - |
| 12618 | ENSP00000268150 | MFGE8 | 5.829E-05 | -0.245 | - | - |
| 12619 | ENSP00000311183 | ZNF497 | 1.639E-05 | -0.245 | - | - |
| 12620 | ENSP00000254322 | DNAJB1 | 2.929E-05 | -0.245 | - | - |
| 12621 | ENSP00000353007 | SULF2 | 2.047E-05 | -0.245 | - | - |
| 12622 | ENSP00000308897 | MRPL11 | 7.755E-06 | -0.245 | - | - |
| 12623 | ENSP00000222270 | WBP7 | 1.330E-05 | -0.245 | - | - |
| 12624 | ENSP00000256720 | LPIN1 | 9.971E-06 | -0.245 | - | - |
| 12625 | ENSP00000294635 | LRRC53 | 1.293E-05 | -0.245 | - | - |
| 12626 | ENSP00000318646 | RPS15A | 1.088E-05 | -0.245 | - | - |
| 12627 | ENSP00000334394 | ZNF677 | 1.711E-05 | -0.245 | - | - |
| 12628 | ENSP00000380557 | AKAP8L | 1.324E-05 | -0.245 | - | - |
| 12629 | ENSP00000342002 | ZNF93 | 2.014E-05 | -0.245 | - | - |
| 12630 | ENSP00000307863 | U2AF2 | 1.527E-05 | -0.245 | - | - |
| 12631 | ENSP00000312141 | ZNF654 | 1.374E-05 | -0.245 | - | - |
| 12632 | ENSP00000254942 | TERF2 | 2.047E-05 | -0.245 | - | - |
| 12633 | ENSP00000345259 | EPB41 | 1.851E-05 | -0.245 | - | - |
| 12634 | ENSP00000210060 | DHPS | 1.334E-05 | -0.245 | - | - |
| 12635 | ENSP00000308368 | C12orf76 | 4.905E-05 | -0.245 | - | - |
| 12636 | ENSP00000253144 | ZNF331 | 1.573E-05 | -0.245 | - | - |
| 12637 | ENSP00000330343 | SUMO3 | 1.728E-05 | -0.245 | - | - |
| 12638 | ENSP00000371398 | GLIS3 | 1.484E-05 | -0.245 | - | - |
| 12639 | ENSP00000338173 | PUM2 | 1.522E-05 | -0.245 | - | - |
| 12640 | ENSP00000331471 | NXF2 | 1.559E-05 | -0.246 | - | - |
| 12641 | ENSP00000333725 | ZNF26 | 1.366E-05 | -0.246 | - | - |
| 12642 | ENSP00000349143 | BTN2A2 | 2.040E-05 | -0.246 | - | - |
| 12643 | ENSP00000251636 | DHX29 | 6.625E-06 | -0.246 | - | - |
| 12644 | ENSP00000313158 | ZNF366 | 1.990E-05 | -0.246 | - | - |
| 12645 | ENSP00000324287 | ACAP2 | 1.558E-05 | -0.246 | - | - |
| 12646 | ENSP00000405718 | PNMA6A | 1.765E-05 | -0.246 | - | - |
| 12647 | ENSP00000370801 | MBD3L4 | 7.374E-06 | -0.246 | - | - |
| 12648 | ENSP00000312631 | INSM1 | 2.051E-05 | -0.246 | - | - |
| 12649 | ENSP00000339764 | PRDM8 | 1.992E-05 | -0.246 | - | - |
| 12650 | ENSP00000362282 | MOCS1 | 1.709E-05 | -0.246 | - | - |
| 12651 | ENSP00000329698 | C12orf56 | 2.547E-05 | -0.246 | - | - |
| 12652 | ENSP00000328835 | TOP1MT | 2.049E-05 | -0.246 | - | - |
| 12653 | ENSP00000322791 | KIF1A | 2.063E-05 | -0.246 | - | - |
| 12654 | ENSP00000243896 | SLC35C2 | 1.341E-05 | -0.246 | - | - |
| 12655 | ENSP00000224950 | OBFC1 | 1.156E-05 | -0.246 | - | - |
| 12656 | ENSP00000401859 | YAF2 | 2.210E-05 | -0.246 | - | - |
| 12657 | ENSP00000367440 | TDP2 | 3.219E-05 | -0.246 | - | - |
| 12658 | ENSP00000294053 | CLPB | 1.357E-05 | -0.246 | - | - |
| 12659 | ENSP00000429986 | ENY2 | 1.646E-05 | -0.246 | - | - |
| 12660 | ENSP00000351732 | TMC2 | 6.130E-06 | -0.246 | - | - |
| 12661 | ENSP00000358813 | TTK | 2.366E-05 | -0.246 | - | - |
| 12662 | ENSP00000358737 | ATP5F1 | 7.464E-06 | -0.246 | - | - |
| 12663 | ENSP00000367449 | C10orf111 | 7.650E-06 | -0.246 | - | - |
| 12664 | ENSP00000387471 | FAM96B | 1.801E-05 | -0.246 | - | - |
| 12665 | ENSP00000386218 | C16orf3 | 3.008E-06 | -0.246 | - | - |
| 12666 | ENSP00000366829 | RBM10 | 2.487E-05 | -0.246 | - | - |
| 12667 | ENSP00000338770 | ZNF420 | 1.409E-05 | -0.246 | - | - |
| 12668 | ENSP00000354645 | GPATCH3 | 2.613E-05 | -0.246 | - | - |
| 12669 | ENSP00000292432 | HK3 | 1.278E-05 | -0.246 | - | - |
| 12670 | ENSP00000316940 | TMEM31 | 3.060E-05 | -0.246 | - | - |
| 12671 | ENSP00000264951 | XRN1 | 1.422E-05 | -0.246 | - | - |
| 12672 | ENSP00000298283 | RPL10L | 5.333E-06 | -0.246 | - | - |
| 12673 | ENSP00000374372 | SPTB | 2.117E-05 | -0.246 | - | - |
| 12674 | ENSP00000434258 | - | 1.043E-05 | -0.246 | - | - |
| 12675 | ENSP00000287652 | TATDN2 | 9.910E-06 | -0.246 | - | - |
| 12676 | ENSP00000348163 | PLS3 | 2.074E-05 | -0.246 | - | - |
| 12677 | ENSP00000247271 | OMG | 1.414E-05 | -0.246 | - | - |
| 12678 | ENSP00000306606 | ADH1B | 1.955E-05 | -0.246 | - | - |
| 12679 | ENSP00000339016 | ATAD1 | 1.414E-05 | -0.246 | - | - |
| 12680 | ENSP00000364089 | PAGE3 | 8.314E-06 | -0.246 | - | - |
| 12681 | ENSP00000357873 | ACADSB | 1.078E-05 | -0.246 | - | - |
| 12682 | ENSP00000317817 | SH2D3C | 2.504E-05 | -0.246 | - | - |
| 12683 | ENSP00000394178 | TMEM182 | 5.624E-06 | -0.246 | - | - |
| 12684 | ENSP00000305005 | PSG8 | 2.924E-05 | -0.246 | - | - |
| 12685 | ENSP00000161863 | YTHDC2 | 5.350E-06 | -0.246 | - | - |
| 12686 | ENSP00000268220 | SEC11A | 8.581E-06 | -0.246 | - | - |
| 12687 | ENSP00000319464 | CPN2 | 1.422E-05 | -0.246 | - | - |
| 12688 | ENSP00000316578 | SUZ12 | 2.760E-05 | -0.246 | - | - |
| 12689 | ENSP00000377969 | GTF2F1 | 1.997E-05 | -0.246 | - | - |
| 12690 | ENSP00000243501 | PLA2G12A | 3.042E-05 | -0.246 | - | - |
| 12691 | ENSP00000374234 | TMEM72 | 7.002E-06 | -0.246 | - | - |
| 12692 | ENSP00000469886 | PINLYP | 9.039E-07 | -0.246 | - | - |
| 12693 | ENSP00000421191 | IGSF22 | 9.573E-06 | -0.246 | - | - |
| 12694 | ENSP00000296266 | IFT122 | 1.475E-05 | -0.246 | - | - |
| 12695 | ENSP00000364289 | NCBP1 | 2.883E-05 | -0.246 | - | - |
| 12696 | ENSP00000292562 | ZNF251 | 1.944E-05 | -0.246 | - | - |
| 12697 | ENSP00000300648 | GCN1L1 | 9.788E-06 | -0.246 | - | - |
| 12698 | ENSP00000359385 | GLMN | 1.928E-05 | -0.246 | - | - |
| 12699 | ENSP00000311665 | PAAF1 | 2.031E-05 | -0.246 | - | - |
| 12700 | ENSP00000282572 | CCNO | 1.419E-05 | -0.246 | - | - |
| 12701 | ENSP00000243938 | WFDC3 | 1.251E-05 | -0.246 | - | - |
| 12702 | ENSP00000371345 | METTL22 | 2.586E-05 | -0.246 | - | - |
| 12703 | ENSP00000035383 | LRRC7 | 1.211E-05 | -0.246 | - | - |
| 12704 | ENSP00000377789 | SYNPO | 1.308E-05 | -0.246 | - | - |
| 12705 | ENSP00000306756 | ZNF584 | 1.293E-05 | -0.246 | - | - |
| 12706 | ENSP00000323856 | PLEC | 2.248E-05 | -0.246 | - | - |
| 12707 | ENSP00000325677 | RNF40 | 1.645E-05 | -0.246 | - | - |
| 12708 | ENSP00000398637 | ARL13A | 5.996E-06 | -0.246 | - | - |
| 12709 | ENSP00000216241 | CHADL | 1.343E-05 | -0.246 | - | - |
| 12710 | ENSP00000393324 | EIF4E3 | 7.497E-06 | -0.246 | - | - |
| 12711 | ENSP00000246548 | UBA2 | 1.648E-05 | -0.247 | - | - |
| 12712 | ENSP00000337363 | ZNF394 | 1.621E-05 | -0.247 | - | - |
| 12713 | ENSP00000296674 | RPS23 | 1.332E-05 | -0.247 | - | - |
| 12714 | ENSP00000361199 | C10orf99 | 1.371E-05 | -0.247 | - | - |
| 12715 | ENSP00000310226 | RAB1B | 8.738E-06 | -0.247 | - | - |
| 12716 | ENSP00000403524 | BRAP | 1.153E-05 | -0.247 | - | - |
| 12717 | ENSP00000007969 | LRRC23 | 1.171E-05 | -0.247 | - | - |
| 12718 | ENSP00000341094 | HIST1H2AD | 1.967E-05 | -0.247 | - | - |
| 12719 | ENSP00000322887 | CIRBP | 2.106E-05 | -0.247 | - | - |
| 12720 | ENSP00000341138 | EPB41L3 | 2.578E-05 | -0.247 | - | - |
| 12721 | ENSP00000225983 | HDAC5 | 1.753E-05 | -0.247 | - | - |
| 12722 | ENSP00000383901 | LIPM | 3.003E-05 | -0.247 | - | - |
| 12723 | ENSP00000341466 | KIF18B | 9.038E-06 | -0.247 | - | - |
| 12724 | ENSP00000299687 | ZNF407 | 1.154E-05 | -0.247 | - | - |
| 12725 | ENSP00000395449 | RECQL | 2.105E-05 | -0.247 | - | - |
| 12726 | ENSP00000328625 | LRRC33 | 2.013E-05 | -0.247 | - | - |
| 12727 | ENSP00000327143 | ZNF354B | 1.497E-05 | -0.247 | - | - |
| 12728 | ENSP00000380960 | C17orf67 | 2.491E-05 | -0.247 | - | - |
| 12729 | ENSP00000296091 | ZNF502 | 1.512E-05 | -0.247 | - | - |
| 12730 | ENSP00000343190 | PPCDC | 1.146E-05 | -0.247 | - | - |
| 12731 | ENSP00000349415 | RAB18 | 9.663E-06 | -0.247 | - | - |
| 12732 | ENSP00000333266 | UBA7 | 2.734E-05 | -0.247 | - | - |
| 12733 | ENSP00000303889 | ZNF30 | 1.363E-05 | -0.247 | - | - |
| 12734 | ENSP00000265801 | LZTS1 | 2.746E-05 | -0.247 | - | - |
| 12735 | ENSP00000373627 | 14-Sep | 1.670E-05 | -0.247 | - | - |
| 12736 | ENSP00000395929 | NSD1 | 3.914E-05 | -0.247 | - | - |
| 12737 | ENSP00000305976 | SIK2 | 1.643E-05 | -0.247 | - | - |
| 12738 | ENSP00000248975 | YWHAH | 1.481E-05 | -0.247 | - | - |
| 12739 | ENSP00000399985 | FLI1 | 2.122E-05 | -0.247 | - | - |
| 12740 | ENSP00000340211 | CORO1B | 1.047E-05 | -0.247 | - | - |
| 12741 | ENSP00000265113 | SLC1A3 | 3.516E-05 | -0.247 | - | - |
| 12742 | ENSP00000288490 | DGKI | 2.157E-05 | -0.247 | - | - |
| 12743 | ENSP00000437968 | HEATR5A | 1.746E-05 | -0.247 | - | - |
| 12744 | ENSP00000255465 | CCNA1 | 1.982E-05 | -0.247 | - | - |
| 12745 | ENSP00000386212 | PSMB11 | 1.696E-05 | -0.247 | - | - |
| 12746 | ENSP00000364814 | FAM131C | 5.210E-06 | -0.247 | - | - |
| 12747 | ENSP00000412292 | DEPDC1 | 1.279E-05 | -0.247 | - | - |
| 12748 | ENSP00000344929 | BPIFB6 | 2.085E-05 | -0.247 | - | - |
| 12749 | ENSP00000264010 | CTCF | 2.006E-05 | -0.247 | - | - |
| 12750 | ENSP00000355251 | ZNF511 | 1.059E-05 | -0.247 | - | - |
| 12751 | ENSP00000472934 | FKSG61 | 4.941E-07 | -0.247 | - | - |
| 12752 | ENSP00000298299 | ZNF22 | 1.512E-05 | -0.247 | - | - |
| 12753 | ENSP00000250173 | LRRC6 | 9.634E-06 | -0.247 | - | - |
| 12754 | ENSP00000261172 | EPYC | 1.241E-05 | -0.247 | - | - |
| 12755 | ENSP00000285848 | OXA1L | 1.011E-05 | -0.248 | - | - |
| 12756 | ENSP00000334008 | PARVA | 6.145E-05 | -0.248 | - | - |
| 12757 | ENSP00000368984 | EXOSC9 | 1.079E-05 | -0.248 | - | - |
| 12758 | ENSP00000215095 | STX1B | 1.204E-05 | -0.248 | - | - |
| 12759 | ENSP00000423567 | CCDC153 | 7.483E-06 | -0.248 | - | - |
| 12760 | ENSP00000295901 | PSMD6 | 1.606E-05 | -0.248 | - | - |
| 12761 | ENSP00000380718 | CDYL | 1.546E-05 | -0.248 | - | - |
| 12762 | ENSP00000259727 | GMPR | 1.373E-05 | -0.248 | - | - |
| 12763 | ENSP00000453129 | GALK2 | 1.908E-05 | -0.248 | - | - |
| 12764 | ENSP00000343924 | PRELP | 1.922E-05 | -0.248 | - | - |
| 12765 | ENSP00000306410 | ARMC4 | 1.488E-05 | -0.248 | - | - |
| 12766 | ENSP00000299927 | ZNF592 | 1.659E-05 | -0.248 | - | - |
| 12767 | ENSP00000293725 | ZNF563 | 1.516E-05 | -0.248 | - | - |
| 12768 | ENSP00000245503 | MYH2 | 1.294E-05 | -0.248 | - | - |
| 12769 | ENSP00000199320 | DIMT1 | 5.020E-06 | -0.248 | - | - |
| 12770 | ENSP00000261951 | CNOT6 | 2.172E-05 | -0.248 | - | - |
| 12771 | ENSP00000349923 | LAGE3 | 1.142E-05 | -0.248 | - | - |
| 12772 | ENSP00000249842 | ISLR | 1.172E-05 | -0.248 | - | - |
| 12773 | ENSP00000255381 | MYH4 | 1.370E-05 | -0.248 | - | - |
| 12774 | ENSP00000376568 | MLF1 | 1.613E-05 | -0.248 | - | - |
| 12775 | ENSP00000334952 | GPR125 | 2.838E-05 | -0.248 | - | - |
| 12776 | ENSP00000295266 | PDHA2 | 1.218E-05 | -0.248 | - | - |
| 12777 | ENSP00000363480 | DLG3 | 2.776E-05 | -0.248 | - | - |
| 12778 | ENSP00000317714 | STX4 | 1.512E-05 | -0.248 | - | - |
| 12779 | ENSP00000311336 | ATP8B3 | 1.341E-05 | -0.248 | - | - |
| 12780 | ENSP00000368102 | CCDC3 | 1.223E-05 | -0.248 | - | - |
| 12781 | ENSP00000326806 | NCBP2 | 1.116E-05 | -0.248 | - | - |
| 12782 | ENSP00000442793 | BEAN1 | 1.388E-05 | -0.248 | - | - |
| 12783 | ENSP00000371294 | CTNS | 2.195E-05 | -0.248 | - | - |
| 12784 | ENSP00000229314 | GOLT1B | 1.024E-04 | -0.248 | - | - |
| 12785 | ENSP00000350098 | POP5 | 1.014E-05 | -0.248 | - | - |
| 12786 | ENSP00000391723 | PUM1 | 1.340E-05 | -0.248 | - | - |
| 12787 | ENSP00000382834 | NPRL3 | 2.156E-05 | -0.248 | - | - |
| 12788 | ENSP00000297579 | DCAF13 | 9.643E-06 | -0.248 | - | - |
| 12789 | ENSP00000399968 | NCOA2 | 3.972E-05 | -0.248 | - | - |
| 12790 | ENSP00000320188 | ZNF579 | 1.463E-05 | -0.248 | - | - |
| 12791 | ENSP00000294517 | ADC | 1.719E-05 | -0.248 | - | - |
| 12792 | ENSP00000261332 | ZNF24 | 1.748E-05 | -0.248 | - | - |
| 12793 | ENSP00000318222 | ZDHHC22 | 1.138E-05 | -0.248 | - | - |
| 12794 | ENSP00000426120 | GRAMD3 | 1.396E-05 | -0.248 | - | - |
| 12795 | ENSP00000386461 | MYO7B | 1.551E-05 | -0.248 | - | - |
| 12796 | ENSP00000360824 | C1orf185 | 3.799E-06 | -0.248 | - | - |
| 12797 | ENSP00000270459 | ZNF787 | 1.323E-05 | -0.248 | - | - |
| 12798 | ENSP00000341681 | ZDHHC7 | 1.844E-05 | -0.248 | - | - |
| 12799 | ENSP00000319286 | GP5 | 2.182E-05 | -0.248 | - | - |
| 12800 | ENSP00000252505 | ALLC | 1.775E-05 | -0.248 | - | - |
| 12801 | ENSP00000323670 | ZBTB7A | 2.456E-05 | -0.248 | - | - |
| 12802 | ENSP00000341151 | ZNF81 | 1.316E-05 | -0.248 | - | - |
| 12803 | ENSP00000387278 | MPP4 | 1.644E-05 | -0.248 | - | - |
| 12804 | ENSP00000222286 | GAPDHS | 1.400E-05 | -0.248 | - | - |
| 12805 | ENSP00000294066 | MAP4K2 | 9.104E-06 | -0.248 | - | - |
| 12806 | ENSP00000425107 | ATG12 | 2.707E-05 | -0.248 | - | - |
| 12807 | ENSP00000217130 | ZNF512B | 1.254E-05 | -0.248 | - | - |
| 12808 | ENSP00000262096 | ZDHHC2 | 1.363E-05 | -0.248 | - | - |
| 12809 | ENSP00000222115 | HAS1 | 1.738E-05 | -0.248 | - | - |
| 12810 | ENSP00000320663 | EML4 | 3.229E-05 | -0.249 | - | - |
| 12811 | ENSP00000440832 | CACFD1 | 3.574E-06 | -0.249 | - | - |
| 12812 | ENSP00000420294 | PHC3 | 2.037E-05 | -0.249 | - | - |
| 12813 | ENSP00000414750 | SKOR2 | 2.026E-05 | -0.249 | - | - |
| 12814 | ENSP00000442688 | MFSD5 | 1.535E-05 | -0.249 | - | - |
| 12815 | ENSP00000328732 | ZBTB7C | 1.440E-05 | -0.249 | - | - |
| 12816 | ENSP00000261434 | LIAS | 1.937E-05 | -0.249 | - | - |
| 12817 | ENSP00000316457 | C20orf197 | 2.597E-05 | -0.249 | - | - |
| 12818 | ENSP00000365272 | DLG2 | 2.776E-05 | -0.249 | - | - |
| 12819 | ENSP00000363322 | CDC26 | 1.207E-05 | -0.249 | - | - |
| 12820 | ENSP00000343581 | ZFP30 | 1.433E-05 | -0.249 | - | - |
| 12821 | ENSP00000295373 | DHX57 | 9.642E-06 | -0.249 | - | - |
| 12822 | ENSP00000395723 | ZNF200 | 1.801E-05 | -0.249 | - | - |
| 12823 | ENSP00000225916 | KAT2A | 8.777E-06 | -0.249 | - | - |
| 12824 | ENSP00000321389 | HIST1H2AC | 1.502E-05 | -0.249 | - | - |
| 12825 | ENSP00000356155 | PIK3C2B | 1.394E-05 | -0.249 | - | - |
| 12826 | ENSP00000314214 | VAMP2 | 2.261E-05 | -0.249 | - | - |
| 12827 | ENSP00000366542 | ZNF165 | 1.301E-05 | -0.249 | - | - |
| 12828 | ENSP00000359686 | GPR112 | 2.595E-05 | -0.249 | - | - |
| 12829 | ENSP00000456894 | GOLGA8H | 7.854E-06 | -0.249 | - | - |
| 12830 | ENSP00000343746 | CORO2A | 1.093E-05 | -0.249 | - | - |
| 12831 | ENSP00000271640 | SETDB1 | 1.634E-05 | -0.249 | - | - |
| 12832 | ENSP00000337759 | DOM3Z | 2.017E-05 | -0.249 | - | - |
| 12833 | ENSP00000325091 | LRCH2 | 1.056E-05 | -0.249 | - | - |
| 12834 | ENSP00000270223 | DMWD | 1.719E-05 | -0.249 | - | - |
| 12835 | ENSP00000346733 | ACAP3 | 2.551E-05 | -0.249 | - | - |
| 12836 | ENSP00000356723 | FMO4 | 8.296E-06 | -0.249 | - | - |
| 12837 | ENSP00000361918 | PPIE | 2.971E-05 | -0.249 | - | - |
| 12838 | ENSP00000330101 | KRT76 | 1.118E-05 | -0.249 | - | - |
| 12839 | ENSP00000320679 | STX19 | 9.274E-06 | -0.249 | - | - |
| 12840 | ENSP00000255641 | CSNK1G2 | 1.430E-05 | -0.249 | - | - |
| 12841 | ENSP00000284727 | ATP5G3 | 6.333E-06 | -0.249 | - | - |
| 12842 | ENSP00000307023 | KLF11 | 2.292E-05 | -0.249 | - | - |
| 12843 | ENSP00000342188 | LRRIQ4 | 1.001E-05 | -0.249 | - | - |
| 12844 | ENSP00000300161 | YWHAB | 2.735E-05 | -0.249 | - | - |
| 12845 | ENSP00000283122 | CETN3 | 1.128E-05 | -0.249 | - | - |
| 12846 | ENSP00000230419 | PTK7 | 1.318E-05 | -0.249 | - | - |
| 12847 | ENSP00000303373 | HIST1H2AE | 1.634E-05 | -0.249 | - | - |
| 12848 | ENSP00000456401 | GOLGA8J | 7.436E-06 | -0.249 | - | - |
| 12849 | ENSP00000423159 | GOLGA8O | 7.661E-06 | -0.249 | - | - |
| 12850 | ENSP00000332433 | ZSCAN22 | 1.692E-05 | -0.249 | - | - |
| 12851 | ENSP00000407182 | TNPO2 | 1.110E-05 | -0.249 | - | - |
| 12852 | ENSP00000390084 | SHISA6 | 9.196E-06 | -0.249 | - | - |
| 12853 | ENSP00000291592 | LRRC3 | 1.773E-05 | -0.249 | - | - |
| 12854 | ENSP00000368813 | KLHL34 | 2.307E-05 | -0.249 | - | - |
| 12855 | ENSP00000397453 | UBE2W | 1.728E-05 | -0.249 | - | - |
| 12856 | ENSP00000326817 | LRRC57 | 9.518E-06 | -0.249 | - | - |
| 12857 | ENSP00000039007 | OTC | 1.036E-05 | -0.249 | - | - |
| 12858 | ENSP00000398454 | GOLGA8N | 7.661E-06 | -0.249 | - | - |
| 12859 | ENSP00000227349 | C11orf63 | 3.645E-04 | -0.249 | - | - |
| 12860 | ENSP00000270001 | ZFP14 | 1.328E-05 | -0.249 | - | - |
| 12861 | ENSP00000450811 | - | 3.449E-05 | -0.249 | - | - |
| 12862 | ENSP00000332407 | SDR42E1 | 1.069E-05 | -0.249 | - | - |
| 12863 | ENSP00000349722 | UBE2D3 | 5.860E-05 | -0.249 | - | - |
| 12864 | ENSP00000357597 | TSPYL1 | 1.438E-05 | -0.249 | - | - |
| 12865 | ENSP00000222812 | STX1A | 1.177E-05 | -0.249 | - | - |
| 12866 | ENSP00000332111 | EFCAB13 | 1.242E-05 | -0.249 | - | - |
| 12867 | ENSP00000226209 | MYH3 | 1.245E-05 | -0.249 | - | - |
| 12868 | ENSP00000317691 | RPP25 | 1.159E-05 | -0.249 | - | - |
| 12869 | ENSP00000262726 | EFCAB6 | 8.550E-06 | -0.249 | - | - |
| 12870 | ENSP00000302222 | ZNF25 | 1.384E-05 | -0.249 | - | - |
| 12871 | ENSP00000374014 | TM6SF2 | 1.612E-05 | -0.249 | - | - |
| 12872 | ENSP00000419153 | ZBTB20 | 1.512E-05 | -0.249 | - | - |
| 12873 | ENSP00000432786 | KDM2A | 2.018E-05 | -0.249 | - | - |
| 12874 | ENSP00000315791 | CSTF3 | 1.275E-05 | -0.249 | - | - |
| 12875 | ENSP00000219479 | NME4 | 7.368E-06 | -0.249 | - | - |
| 12876 | ENSP00000259605 | RNF38 | 4.984E-05 | -0.250 | - | - |
| 12877 | ENSP00000361289 | UCK1 | 2.252E-05 | -0.250 | - | - |
| 12878 | ENSP00000248668 | LRFN1 | 9.169E-06 | -0.250 | - | - |
| 12879 | ENSP00000263239 | DDX18 | 8.159E-06 | -0.250 | - | - |
| 12880 | ENSP00000349396 | ABCD4 | 2.707E-05 | -0.250 | - | - |
| 12881 | ENSP00000229179 | NUP107 | 6.049E-06 | -0.250 | - | - |
| 12882 | ENSP00000248643 | MTERF | 2.586E-05 | -0.250 | - | - |
| 12883 | ENSP00000337008 | ZNF644 | 1.283E-05 | -0.250 | - | - |
| 12884 | ENSP00000362824 | OGT | 2.715E-05 | -0.250 | - | - |
| 12885 | ENSP00000370242 | WWC3 | 2.941E-05 | -0.250 | - | - |
| 12886 | ENSP00000338860 | ZNF444 | 1.813E-05 | -0.250 | - | - |
| 12887 | ENSP00000270225 | SAE1 | 1.518E-05 | -0.250 | - | - |
| 12888 | ENSP00000356988 | NIT1 | 2.058E-05 | -0.250 | - | - |
| 12889 | ENSP00000258317 | NPL | 1.422E-05 | -0.250 | - | - |
| 12890 | ENSP00000296682 | PRDM9 | 1.276E-05 | -0.250 | - | - |
| 12891 | ENSP00000270115 | LRRC56 | 1.042E-05 | -0.250 | - | - |
| 12892 | ENSP00000322159 | ATG4C | 1.719E-05 | -0.250 | - | - |
| 12893 | ENSP00000216484 | SPTLC2 | 1.194E-05 | -0.250 | - | - |
| 12894 | ENSP00000339933 | PKLR | 1.886E-05 | -0.250 | - | - |
| 12895 | ENSP00000334373 | KPNA4 | 2.122E-05 | -0.250 | - | - |
| 12896 | ENSP00000272444 | DUSP11 | 5.795E-05 | -0.250 | - | - |
| 12897 | ENSP00000222145 | RASIP1 | 1.088E-05 | -0.250 | - | - |
| 12898 | ENSP00000301263 | LY6D | 2.144E-05 | -0.250 | - | - |
| 12899 | ENSP00000239906 | FAM53C | 4.347E-05 | -0.250 | - | - |
| 12900 | ENSP00000309477 | ACO1 | 1.594E-05 | -0.250 | - | - |
| 12901 | ENSP00000300151 | MRPL16 | 4.878E-06 | -0.250 | - | - |
| 12902 | ENSP00000314077 | TSPY2 | 1.308E-05 | -0.250 | - | - |
| 12903 | ENSP00000321388 | ASB4 | 9.456E-06 | -0.250 | - | - |
| 12904 | ENSP00000367360 | BLACE | 8.601E-06 | -0.250 | - | - |
| 12905 | ENSP00000276282 | MFHAS1 | 1.048E-05 | -0.250 | - | - |
| 12906 | ENSP00000312126 | ADCY4 | 2.969E-05 | -0.250 | - | - |
| 12907 | ENSP00000318094 | SCMH1 | 2.209E-05 | -0.250 | - | - |
| 12908 | ENSP00000264424 | GUCY1B3 | 1.080E-05 | -0.250 | - | - |
| 12909 | ENSP00000366923 | IGFBPL1 | 7.126E-06 | -0.250 | - | - |
| 12910 | ENSP00000464167 | CTD-2510F5.6 | 7.138E-07 | -0.250 | - | - |
| 12911 | ENSP00000304350 | PRPF8 | 1.212E-05 | -0.250 | - | - |
| 12912 | ENSP00000302485 | CSDC2 | 1.264E-05 | -0.250 | - | - |
| 12913 | ENSP00000375067 | BCAS3 | 2.636E-05 | -0.250 | - | - |
| 12914 | ENSP00000381949 | COL13A1 | 1.587E-05 | -0.250 | - | - |
| 12915 | ENSP00000313454 | UBA6 | 3.465E-05 | -0.250 | - | - |
| 12916 | ENSP00000299084 | SPRED1 | 5.140E-05 | -0.250 | - | - |
| 12917 | ENSP00000270617 | ZNF473 | 1.345E-05 | -0.250 | - | - |
| 12918 | ENSP00000216774 | SRP54 | 6.337E-06 | -0.250 | - | - |
| 12919 | ENSP00000253115 | ZNF426 | 2.040E-05 | -0.251 | - | - |
| 12920 | ENSP00000284719 | OLA1 | 7.964E-06 | -0.251 | - | - |
| 12921 | ENSP00000292055 | SIK3 | 1.702E-05 | -0.251 | - | - |
| 12922 | ENSP00000350267 | BRPF3 | 1.009E-05 | -0.251 | - | - |
| 12923 | ENSP00000360166 | CXorf64 | 5.144E-06 | -0.251 | - | - |
| 12924 | ENSP00000287461 | ZNF689 | 1.689E-05 | -0.251 | - | - |
| 12925 | ENSP00000292616 | LRWD1 | 1.119E-05 | -0.251 | - | - |
| 12926 | ENSP00000372982 | TMEM200C | 2.260E-05 | -0.251 | - | - |
| 12927 | ENSP00000302855 | ZNF280A | 1.767E-05 | -0.251 | - | - |
| 12928 | ENSP00000282286 | ZNF304 | 1.304E-05 | -0.251 | - | - |
| 12929 | ENSP00000303696 | ZNF57 | 1.574E-05 | -0.251 | - | - |
| 12930 | ENSP00000294818 | LRRC52 | 1.130E-05 | -0.251 | - | - |
| 12931 | ENSP00000304553 | MPLKIP | 1.183E-05 | -0.251 | - | - |
| 12932 | ENSP00000263657 | PNO1 | 5.343E-06 | -0.251 | - | - |
| 12933 | ENSP00000234038 | PPP1R7 | 1.157E-05 | -0.251 | - | - |
| 12934 | ENSP00000335307 | DEFB106A | 1.294E-05 | -0.251 | - | - |
| 12935 | ENSP00000454253 | CLN3 | 1.716E-05 | -0.251 | - | - |
| 12936 | ENSP00000376824 | IFI27L1 | 1.773E-06 | -0.251 | - | - |
| 12937 | ENSP00000401946 | TNRC6B | 8.114E-06 | -0.251 | - | - |
| 12938 | ENSP00000264183 | ARID4B | 8.022E-06 | -0.251 | - | - |
| 12939 | ENSP00000311816 | REST | 2.077E-05 | -0.251 | - | - |
| 12940 | ENSP00000369391 | RPP40 | 1.140E-05 | -0.251 | - | - |
| 12941 | ENSP00000444196 | RIBC2 | 2.176E-05 | -0.251 | - | - |
| 12942 | ENSP00000410833 | GBE1 | 2.845E-05 | -0.251 | - | - |
| 12943 | ENSP00000359525 | UBE2NL | 3.210E-05 | -0.251 | - | - |
| 12944 | ENSP00000256722 | CMPK2 | 1.166E-05 | -0.251 | - | - |
| 12945 | ENSP00000269844 | PRDM15 | 1.504E-05 | -0.251 | - | - |
| 12946 | ENSP00000260404 | PAK6 | 1.871E-05 | -0.251 | - | - |
| 12947 | ENSP00000229595 | ASF1A | 1.869E-05 | -0.251 | - | - |
| 12948 | ENSP00000395473 | MAN2B1 | 3.111E-05 | -0.251 | - | - |
| 12949 | ENSP00000311030 | ABCC12 | 1.658E-05 | -0.251 | - | - |
| 12950 | ENSP00000298288 | LRR1 | 1.400E-05 | -0.251 | - | - |
| 12951 | ENSP00000462054 | ZFP92 | 1.286E-05 | -0.251 | - | - |
| 12952 | ENSP00000264335 | YWHAE | 1.627E-05 | -0.251 | - | - |
| 12953 | ENSP00000363193 | MED12 | 1.808E-05 | -0.251 | - | - |
| 12954 | ENSP00000320650 | DNAJC25 | 5.461E-06 | -0.251 | - | - |
| 12955 | ENSP00000315136 | APBA3 | 2.006E-05 | -0.251 | - | - |
| 12956 | ENSP00000327796 | DCAF4L1 | 1.030E-05 | -0.251 | - | - |
| 12957 | ENSP00000264018 | DDX6 | 8.880E-06 | -0.251 | - | - |
| 12958 | ENSP00000287025 | MTERFD1 | 8.076E-06 | -0.251 | - | - |
| 12959 | ENSP00000434142 | TPD52L1 | 8.132E-06 | -0.251 | - | - |
| 12960 | ENSP00000157812 | PSMC4 | 1.525E-05 | -0.251 | - | - |
| 12961 | ENSP00000319222 | ZNF720 | 1.444E-05 | -0.251 | - | - |
| 12962 | ENSP00000306548 | MRPL13 | 5.529E-06 | -0.251 | - | - |
| 12963 | ENSP00000371341 | TNK2 | 2.029E-05 | -0.251 | - | - |
| 12964 | ENSP00000348298 | LCOR | 1.557E-05 | -0.251 | - | - |
| 12965 | ENSP00000331879 | PRMT3 | 1.371E-05 | -0.251 | - | - |
| 12966 | ENSP00000304440 | HAS3 | 2.981E-05 | -0.251 | - | - |
| 12967 | ENSP00000326249 | ZNF454 | 1.326E-05 | -0.251 | - | - |
| 12968 | ENSP00000420195 | SRSF10 | 1.451E-05 | -0.251 | - | - |
| 12969 | ENSP00000325240 | LASP1 | 1.701E-05 | -0.251 | - | - |
| 12970 | ENSP00000302896 | RPS9 | 6.686E-06 | -0.251 | - | - |
| 12971 | ENSP00000420418 | ZNF398 | 1.065E-05 | -0.251 | - | - |
| 12972 | ENSP00000350275 | HIST1H3A | 1.931E-05 | -0.251 | - | - |
| 12973 | ENSP00000308012 | PABPC5 | 1.379E-05 | -0.251 | - | - |
| 12974 | ENSP00000337852 | ZNF576 | 2.140E-05 | -0.252 | - | - |
| 12975 | ENSP00000359991 | PGAM1 | 2.341E-05 | -0.252 | - | - |
| 12976 | ENSP00000344909 | PTP4A2 | 2.603E-05 | -0.252 | - | - |
| 12977 | ENSP00000265827 | ZNF800 | 2.135E-05 | -0.252 | - | - |
| 12978 | ENSP00000313691 | FTMT | 1.058E-05 | -0.252 | - | - |
| 12979 | ENSP00000304308 | ZNF217 | 1.995E-05 | -0.252 | - | - |
| 12980 | ENSP00000327315 | TIAM2 | 2.246E-05 | -0.252 | - | - |
| 12981 | ENSP00000301310 | ZNF582 | 1.425E-05 | -0.252 | - | - |
| 12982 | ENSP00000418897 | ZNF322 | 9.642E-06 | -0.252 | - | - |
| 12983 | ENSP00000381840 | AEBP2 | 1.484E-05 | -0.252 | - | - |
| 12984 | ENSP00000396937 | PSMD13 | 2.104E-05 | -0.252 | - | - |
| 12985 | ENSP00000244769 | ATXN1 | 2.824E-05 | -0.252 | - | - |
| 12986 | ENSP00000307774 | ZNF239 | 1.419E-05 | -0.252 | - | - |
| 12987 | ENSP00000272418 | MRPS5 | 7.931E-06 | -0.252 | - | - |
| 12988 | ENSP00000451866 | SMIM13 | 1.679E-06 | -0.252 | - | - |
| 12989 | ENSP00000258969 | CHAD | 1.118E-05 | -0.252 | - | - |
| 12990 | ENSP00000325875 | HSP90AB1 | 3.169E-05 | -0.252 | - | - |
| 12991 | ENSP00000328694 | MOB2 | 1.111E-05 | -0.252 | - | - |
| 12992 | ENSP00000252799 | ZNF747 | 2.184E-05 | -0.252 | - | - |
| 12993 | ENSP00000347119 | H2BFM | 1.854E-05 | -0.252 | - | - |
| 12994 | ENSP00000342019 | ZNF189 | 1.720E-05 | -0.252 | - | - |
| 12995 | ENSP00000339918 | XRRA1 | 1.004E-05 | -0.252 | - | - |
| 12996 | ENSP00000327314 | ZNF283 | 1.214E-05 | -0.252 | - | - |
| 12997 | ENSP00000264159 | ZRANB3 | 1.384E-05 | -0.252 | - | - |
| 12998 | ENSP00000338413 | UBA1 | 3.676E-05 | -0.252 | - | - |
| 12999 | ENSP00000379684 | ZNF813 | 1.030E-05 | -0.252 | - | - |
| 13000 | ENSP00000303915 | ZNF561 | 2.061E-05 | -0.252 | - | - |
| 13001 | ENSP00000328023 | SRPR | 1.030E-05 | -0.252 | - | - |
| 13002 | ENSP00000314023 | SIDT2 | 7.605E-06 | -0.252 | - | - |
| 13003 | ENSP00000311521 | ZNF490 | 1.635E-05 | -0.252 | - | - |
| 13004 | ENSP00000354728 | MT-ND4L | 8.163E-06 | -0.252 | - | - |
| 13005 | ENSP00000319053 | ZNF77 | 1.790E-05 | -0.252 | - | - |
| 13006 | ENSP00000355996 | G0S2 | 2.183E-05 | -0.252 | - | - |
| 13007 | ENSP00000283684 | ATP8B1 | 4.017E-05 | -0.252 | - | - |
| 13008 | ENSP00000354813 | MT-ND5 | 2.105E-05 | -0.252 | - | - |
| 13009 | ENSP00000264234 | UPK1B | 1.331E-05 | -0.252 | - | - |
| 13010 | ENSP00000437812 | FAM106A | 5.283E-06 | -0.252 | - | - |
| 13011 | ENSP00000371643 | DEFB135 | 3.704E-06 | -0.252 | - | - |
| 13012 | ENSP00000348419 | ZNF44 | 2.261E-05 | -0.252 | - | - |
| 13013 | ENSP00000293405 | IZUMO2 | 1.036E-05 | -0.252 | - | - |
| 13014 | ENSP00000340171 | ZNF573 | 2.874E-05 | -0.252 | - | - |
| 13015 | ENSP00000264255 | TXNDC9 | 7.328E-06 | -0.252 | - | - |
| 13016 | ENSP00000298630 | STK32C | 1.634E-05 | -0.252 | - | - |
| 13017 | ENSP00000307604 | ZBTB5 | 1.434E-05 | -0.252 | - | - |
| 13018 | ENSP00000415774 | ZNF732 | 1.129E-05 | -0.252 | - | - |
| 13019 | ENSP00000205890 | MYO15A | 9.410E-06 | -0.252 | - | - |
| 13020 | ENSP00000296328 | UBXN7 | 1.298E-05 | -0.252 | - | - |
| 13021 | ENSP00000379891 | AP1S3 | 2.865E-05 | -0.252 | - | - |
| 13022 | ENSP00000388953 | C21orf37 | 9.323E-06 | -0.252 | - | - |
| 13023 | ENSP00000321132 | ZNF554 | 1.971E-05 | -0.252 | - | - |
| 13024 | ENSP00000400878 | ZNF234 | 1.066E-05 | -0.252 | - | - |
| 13025 | ENSP00000261183 | OSBPL8 | 9.911E-06 | -0.252 | - | - |
| 13026 | ENSP00000346725 | PES1 | 2.555E-05 | -0.252 | - | - |
| 13027 | ENSP00000327652 | - | 1.988E-05 | -0.252 | - | - |
| 13028 | ENSP00000469474 | CCDC177 | 4.002E-07 | -0.252 | - | - |
| 13029 | ENSP00000217939 | MXRA5 | 1.034E-05 | -0.252 | - | - |
| 13030 | ENSP00000237696 | RARRES1 | 3.699E-05 | -0.253 | - | - |
| 13031 | ENSP00000409964 | KIAA1430 | 8.968E-06 | -0.253 | - | - |
| 13032 | ENSP00000428878 | ZNF721 | 1.461E-05 | -0.253 | - | - |
| 13033 | ENSP00000334563 | MYO18B | 2.393E-05 | -0.253 | - | - |
| 13034 | ENSP00000373399 | PIP5KL1 | 1.914E-05 | -0.253 | - | - |
| 13035 | ENSP00000237654 | CCNI | 1.787E-05 | -0.253 | - | - |
| 13036 | ENSP00000406407 | TSPY10 | 1.784E-05 | -0.253 | - | - |
| 13037 | ENSP00000331044 | ENKUR | 3.955E-05 | -0.253 | - | - |
| 13038 | ENSP00000336719 | ZNF226 | 1.426E-05 | -0.253 | - | - |
| 13039 | ENSP00000245663 | ZBTB46 | 1.904E-05 | -0.253 | - | - |
| 13040 | ENSP00000252996 | TAF4 | 3.782E-05 | -0.253 | - | - |
| 13041 | ENSP00000229243 | ACRBP | 9.412E-06 | -0.253 | - | - |
| 13042 | ENSP00000361499 | YIPF3 | 1.871E-05 | -0.253 | - | - |
| 13043 | ENSP00000321195 | ATP11B | 2.296E-05 | -0.253 | - | - |
| 13044 | ENSP00000257765 | KHDC1 | 1.402E-05 | -0.253 | - | - |
| 13045 | ENSP00000232217 | RBP2 | 2.673E-05 | -0.253 | - | - |
| 13046 | ENSP00000166244 | EPHA8 | 1.441E-05 | -0.253 | - | - |
| 13047 | ENSP00000377576 | KRTAP9-9 | 1.272E-06 | -0.253 | - | - |
| 13048 | ENSP00000263095 | ZNF264 | 2.269E-05 | -0.253 | - | - |
| 13049 | ENSP00000276449 | STAR | 2.611E-05 | -0.253 | - | - |
| 13050 | ENSP00000370150 | WRNIP1 | 1.577E-05 | -0.253 | - | - |
| 13051 | ENSP00000437133 | RP11-719K4.1 | 1.015E-05 | -0.253 | - | - |
| 13052 | ENSP00000268661 | RPL3L | 5.493E-06 | -0.253 | - | - |
| 13053 | ENSP00000258654 | COG3 | 1.257E-05 | -0.253 | - | - |
| 13054 | ENSP00000340749 | ZNF124 | 1.462E-05 | -0.253 | - | - |
| 13055 | ENSP00000378368 | FLOT2 | 1.411E-05 | -0.253 | - | - |
| 13056 | ENSP00000366084 | ABCC4 | 1.862E-05 | -0.253 | - | - |
| 13057 | ENSP00000389649 | ATP5J | 2.751E-05 | -0.253 | - | - |
| 13058 | ENSP00000371297 | SNPH | 1.133E-05 | -0.253 | - | - |
| 13059 | ENSP00000357384 | DPM3 | 8.236E-06 | -0.253 | - | - |
| 13060 | ENSP00000274680 | FARS2 | 6.480E-06 | -0.253 | - | - |
| 13061 | ENSP00000336721 | LARP1 | 1.353E-05 | -0.253 | - | - |
| 13062 | ENSP00000278319 | ZNF215 | 1.437E-05 | -0.253 | - | - |
| 13063 | ENSP00000265260 | PCNP | 6.647E-05 | -0.253 | - | - |
| 13064 | ENSP00000276590 | LACTB2 | 7.495E-06 | -0.253 | - | - |
| 13065 | ENSP00000341247 | GSPT2 | 1.981E-05 | -0.253 | - | - |
| 13066 | ENSP00000401277 | CBLL1 | 1.782E-05 | -0.253 | - | - |
| 13067 | ENSP00000400591 | SNRPE | 2.373E-05 | -0.253 | - | - |
| 13068 | ENSP00000418401 | SRPRB | 9.756E-06 | -0.253 | - | - |
| 13069 | ENSP00000269973 | ZNF45 | 2.141E-05 | -0.253 | - | - |
| 13070 | ENSP00000328515 | ZNF696 | 1.656E-05 | -0.253 | - | - |
| 13071 | ENSP00000299596 | TMEM41B | 4.073E-05 | -0.253 | - | - |
| 13072 | ENSP00000352208 | MYOF | 1.583E-05 | -0.253 | - | - |
| 13073 | ENSP00000364847 | MAGED1 | 1.749E-05 | -0.253 | - | - |
| 13074 | ENSP00000360183 | STX16 | 5.118E-06 | -0.253 | - | - |
| 13075 | ENSP00000386655 | RP1-164F3.9 | 8.752E-06 | -0.253 | - | - |
| 13076 | ENSP00000331577 | ZNF397 | 1.332E-05 | -0.253 | - | - |
| 13077 | ENSP00000361857 | RLF | 1.130E-05 | -0.253 | - | - |
| 13078 | ENSP00000373343 | CPNE9 | 2.039E-05 | -0.253 | - | - |
| 13079 | ENSP00000270162 | SIK1 | 2.305E-05 | -0.253 | - | - |
| 13080 | ENSP00000311449 | RAB6A | 3.073E-05 | -0.253 | - | - |
| 13081 | ENSP00000249647 | SNAP23 | 2.402E-05 | -0.253 | - | - |
| 13082 | ENSP00000262640 | VAMP7 | 1.348E-05 | -0.253 | - | - |
| 13083 | ENSP00000327589 | GLUD2 | 1.029E-05 | -0.253 | - | - |
| 13084 | ENSP00000381468 | SPINK13 | 8.225E-06 | -0.253 | - | - |
| 13085 | ENSP00000262085 | ZNF282 | 2.750E-05 | -0.253 | - | - |
| 13086 | ENSP00000296702 | TCERG1 | 3.977E-05 | -0.253 | - | - |
| 13087 | ENSP00000348394 | NCDN | 3.164E-05 | -0.253 | - | - |
| 13088 | ENSP00000371155 | NUPL1 | 1.947E-05 | -0.254 | - | - |
| 13089 | ENSP00000370614 | DPF3 | 9.369E-06 | -0.254 | - | - |
| 13090 | ENSP00000315173 | ZNF41 | 1.245E-05 | -0.254 | - | - |
| 13091 | ENSP00000293217 | ACOX1 | 1.466E-05 | -0.254 | - | - |
| 13092 | ENSP00000353624 | HIST1H4E | 2.043E-05 | -0.254 | - | - |
| 13093 | ENSP00000251453 | RPS16 | 5.962E-06 | -0.254 | - | - |
| 13094 | ENSP00000344193 | RNASE1 | 1.637E-05 | -0.254 | - | - |
| 13095 | ENSP00000325634 | ZNF639 | 1.429E-05 | -0.254 | - | - |
| 13096 | ENSP00000357861 | SELENBP1 | 2.418E-05 | -0.254 | - | - |
| 13097 | ENSP00000367817 | PODXL | 2.521E-05 | -0.254 | - | - |
| 13098 | ENSP00000332595 | ZNF92 | 1.338E-05 | -0.254 | - | - |
| 13099 | ENSP00000306496 | RAB33B | 8.300E-06 | -0.254 | - | - |
| 13100 | ENSP00000083182 | APPBP2 | 8.970E-06 | -0.254 | - | - |
| 13101 | ENSP00000430242 | SBSN | 1.734E-05 | -0.254 | - | - |
| 13102 | ENSP00000364815 | VARS | 2.789E-05 | -0.254 | - | - |
| 13103 | ENSP00000363787 | RING1 | 3.215E-05 | -0.254 | - | - |
| 13104 | ENSP00000319474 | WSB2 | 1.033E-05 | -0.254 | - | - |
| 13105 | ENSP00000382688 | KDM5A | 4.559E-05 | -0.254 | - | - |
| 13106 | ENSP00000383578 | MGC39584 | 6.540E-06 | -0.254 | - | - |
| 13107 | ENSP00000364976 | CKS2 | 1.513E-05 | -0.254 | - | - |
| 13108 | ENSP00000334668 | SAMD7 | 2.058E-05 | -0.254 | - | - |
| 13109 | ENSP00000438095 | ELOVL5 | 2.351E-05 | -0.254 | - | - |
| 13110 | ENSP00000337618 | NIPA2 | 1.546E-05 | -0.254 | - | - |
| 13111 | ENSP00000260008 | FHDC1 | 2.217E-05 | -0.254 | - | - |
| 13112 | ENSP00000313681 | SPHK1 | 5.493E-05 | -0.254 | - | - |
| 13113 | ENSP00000389026 | CRISP3 | 2.148E-05 | -0.254 | - | - |
| 13114 | ENSP00000264995 | MRPL3 | 5.088E-06 | -0.254 | - | - |
| 13115 | ENSP00000273077 | PNKD | 5.940E-06 | -0.254 | - | - |
| 13116 | ENSP00000411397 | MARK3 | 2.089E-05 | -0.254 | - | - |
| 13117 | ENSP00000384211 | PSME4 | 1.280E-05 | -0.254 | - | - |
| 13118 | ENSP00000410198 | ZSCAN2 | 1.541E-05 | -0.254 | - | - |
| 13119 | ENSP00000256996 | DDB2 | 1.351E-05 | -0.254 | - | - |
| 13120 | ENSP00000223369 | YKT6 | 7.529E-06 | -0.254 | - | - |
| 13121 | ENSP00000308315 | PODN | 9.923E-06 | -0.254 | - | - |
| 13122 | ENSP00000308496 | ARL10 | 1.250E-05 | -0.254 | - | - |
| 13123 | ENSP00000361007 | LIPJ | 2.824E-05 | -0.254 | - | - |
| 13124 | ENSP00000304923 | LRRC28 | 9.151E-06 | -0.254 | - | - |
| 13125 | ENSP00000303147 | MAT2A | 1.141E-05 | -0.254 | - | - |
| 13126 | ENSP00000336750 | SUPT7L | 7.888E-06 | -0.254 | - | - |
| 13127 | ENSP00000333326 | TUBA8 | 1.255E-05 | -0.254 | - | - |
| 13128 | ENSP00000339823 | ZNF546 | 1.385E-05 | -0.254 | - | - |
| 13129 | ENSP00000454014 | SHISA9 | 1.025E-05 | -0.254 | - | - |
| 13130 | ENSP00000267102 | LMBR1L | 2.554E-05 | -0.254 | - | - |
| 13131 | ENSP00000280560 | ABCB9 | 2.051E-05 | -0.254 | - | - |
| 13132 | ENSP00000264674 | MECOM | 1.955E-05 | -0.254 | - | - |
| 13133 | ENSP00000354886 | SLC25A47 | 1.608E-05 | -0.254 | - | - |
| 13134 | ENSP00000398880 | CDK12 | 1.425E-05 | -0.254 | - | - |
| 13135 | ENSP00000262873 | MYH7B | 1.370E-05 | -0.254 | - | - |
| 13136 | ENSP00000439146 | PPIAL4A | 2.353E-05 | -0.254 | - | - |
| 13137 | ENSP00000346729 | ZNF589 | 1.215E-05 | -0.254 | - | - |
| 13138 | ENSP00000420168 | GSTA2 | 2.167E-05 | -0.254 | - | - |
| 13139 | ENSP00000286713 | STOM | 1.043E-05 | -0.254 | - | - |
| 13140 | ENSP00000351894 | NCOA6 | 3.056E-05 | -0.254 | - | - |
| 13141 | ENSP00000361705 | DNTTIP1 | 1.610E-05 | -0.254 | - | - |
| 13142 | ENSP00000261839 | MYO5C | 1.259E-05 | -0.254 | - | - |
| 13143 | ENSP00000359630 | BAI3 | 2.440E-05 | -0.255 | - | - |
| 13144 | ENSP00000373047 | OR12D2 | 8.563E-07 | -0.255 | - | - |
| 13145 | ENSP00000366331 | MAS1L | 1.531E-06 | -0.255 | - | - |
| 13146 | ENSP00000352420 | LDLRAD4 | 6.809E-06 | -0.255 | - | - |
| 13147 | ENSP00000230461 | TMEM30A | 2.246E-05 | -0.255 | - | - |
| 13148 | ENSP00000248996 | GNAZ | 1.377E-05 | -0.255 | - | - |
| 13149 | ENSP00000441459 | SDIM1 | 3.433E-06 | -0.255 | - | - |
| 13150 | ENSP00000286835 | SCAF4 | 3.090E-05 | -0.255 | - | - |
| 13151 | ENSP00000295006 | CAPN2 | 1.596E-05 | -0.255 | - | - |
| 13152 | ENSP00000390331 | MRPS17 | 1.425E-05 | -0.255 | - | - |
| 13153 | ENSP00000354554 | MT-CYB | 2.938E-05 | -0.255 | - | - |
| 13154 | ENSP00000288309 | KCNJ6 | 1.670E-05 | -0.255 | - | - |
| 13155 | ENSP00000374354 | EXOSC8 | 2.440E-05 | -0.255 | - | - |
| 13156 | ENSP00000281419 | ASAP2 | 2.378E-05 | -0.255 | - | - |
| 13157 | ENSP00000330509 | DEXI | 2.857E-05 | -0.255 | - | - |
| 13158 | ENSP00000380734 | FAM98B | 1.836E-05 | -0.255 | - | - |
| 13159 | ENSP00000356560 | KIAA1614 | 1.418E-05 | -0.255 | - | - |
| 13160 | ENSP00000277225 | ZNF462 | 1.272E-05 | -0.255 | - | - |
| 13161 | ENSP00000365678 | OTUD1 | 3.099E-05 | -0.255 | - | - |
| 13162 | ENSP00000360958 | CYP4A22 | 1.826E-05 | -0.255 | - | - |
| 13163 | ENSP00000273146 | FAM198A | 4.011E-05 | -0.255 | - | - |
| 13164 | ENSP00000373948 | C7orf63 | 7.461E-06 | -0.255 | - | - |
| 13165 | ENSP00000437142 | RPP14 | 1.175E-05 | -0.255 | - | - |
| 13166 | ENSP00000404121 | ILF3 | 2.851E-05 | -0.255 | - | - |
| 13167 | ENSP00000375430 | KRTAP3-1 | 1.800E-05 | -0.255 | - | - |
| 13168 | ENSP00000301744 | ZNF597 | 1.302E-05 | -0.255 | - | - |
| 13169 | ENSP00000370151 | RAD17 | 1.893E-05 | -0.255 | - | - |
| 13170 | ENSP00000382856 | MTRNR2L4 | 3.219E-06 | -0.255 | - | - |
| 13171 | ENSP00000249044 | APOL5 | 3.236E-05 | -0.255 | - | - |
| 13172 | ENSP00000244051 | MOCS3 | 1.177E-05 | -0.255 | - | - |
| 13173 | ENSP00000259324 | LRRC8A | 1.679E-05 | -0.255 | - | - |
| 13174 | ENSP00000366404 | TRIM27 | 2.655E-05 | -0.255 | - | - |
| 13175 | ENSP00000252172 | MYH13 | 9.139E-06 | -0.255 | - | - |
| 13176 | ENSP00000298119 | LRFN5 | 9.454E-06 | -0.255 | - | - |
| 13177 | ENSP00000228928 | OAS3 | 1.076E-05 | -0.255 | - | - |
| 13178 | ENSP00000318351 | BCKDHB | 1.644E-05 | -0.255 | - | - |
| 13179 | ENSP00000267502 | RDH12 | 9.853E-06 | -0.255 | - | - |
| 13180 | ENSP00000341159 | CERKL | 1.754E-05 | -0.255 | - | - |
| 13181 | ENSP00000467209 | RP11-125O5.2 | 2.939E-07 | -0.255 | - | - |
| 13182 | ENSP00000300850 | ZNF646 | 1.443E-05 | -0.255 | - | - |
| 13183 | ENSP00000238789 | ATAD2B | 9.541E-06 | -0.256 | - | - |
| 13184 | ENSP00000216122 | MCM5 | 8.003E-06 | -0.256 | - | - |
| 13185 | ENSP00000369677 | EMILIN1 | 1.660E-05 | -0.256 | - | - |
| 13186 | ENSP00000371983 | AFAP1 | 7.713E-06 | -0.256 | - | - |
| 13187 | ENSP00000300619 | ZNF91 | 1.744E-05 | -0.256 | - | - |
| 13188 | ENSP00000343557 | ZCCHC17 | 9.619E-06 | -0.256 | - | - |
| 13189 | ENSP00000264669 | FASTKD3 | 3.621E-05 | -0.256 | - | - |
| 13190 | ENSP00000305255 | STX8 | 1.236E-05 | -0.256 | - | - |
| 13191 | ENSP00000356278 | PHLDA3 | 2.042E-05 | -0.256 | - | - |
| 13192 | ENSP00000222224 | LENG1 | 2.263E-05 | -0.256 | - | - |
| 13193 | ENSP00000276816 | ZNF16 | 1.406E-05 | -0.256 | - | - |
| 13194 | ENSP00000467018 | ZNF223 | 8.803E-06 | -0.256 | - | - |
| 13195 | ENSP00000373937 | PRTG | 2.034E-05 | -0.256 | - | - |
| 13196 | ENSP00000350549 | HMX3 | 1.617E-05 | -0.256 | - | - |
| 13197 | ENSP00000344226 | RASSF7 | 2.376E-05 | -0.256 | - | - |
| 13198 | ENSP00000269097 | G6PC3 | 2.113E-05 | -0.256 | - | - |
| 13199 | ENSP00000271732 | GOLPH3L | 2.388E-05 | -0.256 | - | - |
| 13200 | ENSP00000199447 | NME8 | 6.004E-06 | -0.256 | - | - |
| 13201 | ENSP00000300101 | ZBTB39 | 1.468E-05 | -0.256 | - | - |
| 13202 | ENSP00000298428 | SEC61A2 | 1.704E-05 | -0.256 | - | - |
| 13203 | ENSP00000313881 | MKRN3 | 2.407E-05 | -0.256 | - | - |
| 13204 | ENSP00000462883 | CCDC79 | 2.545E-06 | -0.256 | - | - |
| 13205 | ENSP00000300258 | TCP10L | 2.286E-05 | -0.256 | - | - |
| 13206 | ENSP00000351422 | C19orf60 | 3.104E-06 | -0.256 | - | - |
| 13207 | ENSP00000294600 | CCDC27 | 4.922E-05 | -0.256 | - | - |
| 13208 | ENSP00000315662 | IQSEC3 | 1.856E-05 | -0.256 | - | - |
| 13209 | ENSP00000369592 | CCDC85C | 5.030E-06 | -0.256 | - | - |
| 13210 | ENSP00000304502 | SIX2 | 1.872E-05 | -0.256 | - | - |
| 13211 | ENSP00000326870 | LRRC48 | 1.098E-05 | -0.256 | - | - |
| 13212 | ENSP00000293879 | WDR90 | 7.945E-06 | -0.256 | - | - |
| 13213 | ENSP00000422581 | TRIML2 | 2.317E-05 | -0.256 | - | - |
| 13214 | ENSP00000301178 | AXL | 2.198E-05 | -0.256 | - | - |
| 13215 | ENSP00000414034 | PRR26 | 1.513E-06 | -0.256 | - | - |
| 13216 | ENSP00000451211 | NEDD1 | 2.075E-05 | -0.256 | - | - |
| 13217 | ENSP00000281154 | SLC25A31 | 1.085E-05 | -0.256 | - | - |
| 13218 | ENSP00000297565 | OSR2 | 1.749E-05 | -0.256 | - | - |
| 13219 | ENSP00000412999 | ZNF805 | 9.239E-06 | -0.256 | - | - |
| 13220 | ENSP00000343054 | RBM5 | 2.105E-05 | -0.256 | - | - |
| 13221 | ENSP00000294740 | ZNF281 | 2.084E-05 | -0.256 | - | - |
| 13222 | ENSP00000322276 | VNN2 | 2.120E-05 | -0.256 | - | - |
| 13223 | ENSP00000377801 | RCCD1 | 1.497E-05 | -0.256 | - | - |
| 13224 | ENSP00000358301 | ADRB1 | 2.608E-05 | -0.256 | - | - |
| 13225 | ENSP00000367102 | SLC22A6 | 1.116E-05 | -0.256 | - | - |
| 13226 | ENSP00000391498 | KCNK16 | 2.548E-05 | -0.256 | - | - |
| 13227 | ENSP00000445829 | SLC39A11 | 7.695E-06 | -0.256 | - | - |
| 13228 | ENSP00000441927 | SESN3 | 8.559E-06 | -0.256 | - | - |
| 13229 | ENSP00000450607 | TIMELESS | 2.660E-05 | -0.256 | - | - |
| 13230 | ENSP00000378957 | MFAP4 | 2.799E-05 | -0.256 | - | - |
| 13231 | ENSP00000371321 | RFC1 | 9.557E-06 | -0.256 | - | - |
| 13232 | ENSP00000344635 | CCNG1 | 3.607E-05 | -0.256 | - | - |
| 13233 | ENSP00000235521 | WARS2 | 7.880E-06 | -0.256 | - | - |
| 13234 | ENSP00000339521 | RSU1 | 1.192E-05 | -0.256 | - | - |
| 13235 | ENSP00000419923 | KLF6 | 3.372E-05 | -0.256 | - | - |
| 13236 | ENSP00000380514 | HAGH | 2.034E-05 | -0.256 | - | - |
| 13237 | ENSP00000369600 | FRY | 1.992E-05 | -0.256 | - | - |
| 13238 | ENSP00000412566 | SNRPB | 1.915E-05 | -0.257 | - | - |
| 13239 | ENSP00000455744 | DKFZP686D09174 | 1.054E-05 | -0.257 | - | - |
| 13240 | ENSP00000324025 | CGREF1 | 1.083E-05 | -0.257 | - | - |
| 13241 | ENSP00000362578 | RNF8 | 2.804E-05 | -0.257 | - | - |
| 13242 | ENSP00000299667 | ZNF3 | 1.737E-05 | -0.257 | - | - |
| 13243 | ENSP00000317872 | RBBP6 | 1.405E-05 | -0.257 | - | - |
| 13244 | ENSP00000417063 | SPATA6L | 4.828E-06 | -0.257 | - | - |
| 13245 | ENSP00000262189 | MLL3 | 1.469E-05 | -0.257 | - | - |
| 13246 | ENSP00000348302 | C3orf27 | 9.621E-06 | -0.257 | - | - |
| 13247 | ENSP00000275275 | PNLDC1 | 3.499E-05 | -0.257 | - | - |
| 13248 | ENSP00000350136 | RIMKLB | 2.183E-05 | -0.257 | - | - |
| 13249 | ENSP00000252797 | ZNF764 | 1.901E-05 | -0.257 | - | - |
| 13250 | ENSP00000351047 | ICA1L | 7.934E-06 | -0.257 | - | - |
| 13251 | ENSP00000412361 | SIGLEC11 | 1.091E-05 | -0.257 | - | - |
| 13252 | ENSP00000334051 | GNAL | 8.410E-06 | -0.257 | - | - |
| 13253 | ENSP00000261647 | TTC19 | 1.033E-05 | -0.257 | - | - |
| 13254 | ENSP00000368477 | SERP2 | 1.992E-05 | -0.257 | - | - |
| 13255 | ENSP00000304802 | DTYMK | 1.236E-05 | -0.257 | - | - |
| 13256 | ENSP00000333993 | KRTAP17-1 | 2.744E-06 | -0.257 | - | - |
| 13257 | ENSP00000367356 | FAM171A1 | 1.168E-05 | -0.257 | - | - |
| 13258 | ENSP00000223398 | CLIP2 | 1.876E-05 | -0.257 | - | - |
| 13259 | ENSP00000365130 | ZCCHC6 | 1.595E-05 | -0.257 | - | - |
| 13260 | ENSP00000366746 | STAM | 1.717E-05 | -0.257 | - | - |
| 13261 | ENSP00000325018 | ZNF569 | 2.255E-05 | -0.257 | - | - |
| 13262 | ENSP00000323313 | PDIA5 | 7.730E-06 | -0.257 | - | - |
| 13263 | ENSP00000321735 | SLC16A8 | 2.006E-05 | -0.257 | - | - |
| 13264 | ENSP00000367992 | ESD | 2.818E-05 | -0.257 | - | - |
| 13265 | ENSP00000351410 | PRKAR1A | 1.954E-05 | -0.257 | - | - |
| 13266 | ENSP00000385741 | APOBEC3H | 1.482E-05 | -0.257 | - | - |
| 13267 | ENSP00000268053 | CYP11A1 | 2.503E-05 | -0.257 | - | - |
| 13268 | ENSP00000262259 | ZNF175 | 1.363E-05 | -0.257 | - | - |
| 13269 | ENSP00000305373 | ZNF440 | 1.392E-05 | -0.257 | - | - |
| 13270 | ENSP00000466104 | C17orf61-PLSCR3 | 7.401E-07 | -0.257 | - | - |
| 13271 | ENSP00000424737 | C4orf21 | 6.884E-06 | -0.257 | - | - |
| 13272 | ENSP00000420269 | ADH7 | 1.322E-05 | -0.257 | - | - |
| 13273 | ENSP00000432816 | ACRV1 | 1.795E-05 | -0.257 | - | - |
| 13274 | ENSP00000358715 | SORCS3 | 8.978E-06 | -0.257 | - | - |
| 13275 | ENSP00000425166 | C1orf216 | 8.650E-06 | -0.257 | - | - |
| 13276 | ENSP00000420914 | HSD17B4 | 1.744E-05 | -0.257 | - | - |
| 13277 | ENSP00000249363 | LRRC4 | 1.010E-05 | -0.257 | - | - |
| 13278 | ENSP00000308759 | ZBTB21 | 1.226E-05 | -0.258 | - | - |
| 13279 | ENSP00000360613 | UBE2A | 2.236E-05 | -0.258 | - | - |
| 13280 | ENSP00000302621 | LRG1 | 1.371E-05 | -0.258 | - | - |
| 13281 | ENSP00000360525 | MAGOH | 2.360E-05 | -0.258 | - | - |
| 13282 | ENSP00000255977 | MKRN1 | 1.749E-05 | -0.258 | - | - |
| 13283 | ENSP00000352413 | TMC3 | 1.075E-05 | -0.258 | - | - |
| 13284 | ENSP00000312535 | LRFN4 | 9.714E-06 | -0.258 | - | - |
| 13285 | ENSP00000343617 | ZNF606 | 1.360E-05 | -0.258 | - | - |
| 13286 | ENSP00000301200 | CDC42EP5 | 1.490E-05 | -0.258 | - | - |
| 13287 | ENSP00000444747 | ZNF835 | 9.064E-06 | -0.258 | - | - |
| 13288 | ENSP00000297679 | HSD3B7 | 1.180E-05 | -0.258 | - | - |
| 13289 | ENSP00000288235 | MYO1E | 1.251E-05 | -0.258 | - | - |
| 13290 | ENSP00000313504 | PHKB | 1.133E-05 | -0.258 | - | - |
| 13291 | ENSP00000267807 | ZNF280D | 1.181E-05 | -0.258 | - | - |
| 13292 | ENSP00000361755 | WFDC6 | 1.856E-05 | -0.258 | - | - |
| 13293 | ENSP00000360190 | DEFB110 | 7.008E-06 | -0.258 | - | - |
| 13294 | ENSP00000436455 | NUCB2 | 1.324E-05 | -0.258 | - | - |
| 13295 | ENSP00000336702 | EIF5A | 1.564E-05 | -0.258 | - | - |
| 13296 | ENSP00000372955 | C3orf37 | 7.668E-06 | -0.258 | - | - |
| 13297 | ENSP00000370713 | ADARB2 | 9.869E-06 | -0.258 | - | - |
| 13298 | ENSP00000353826 | LONP1 | 1.373E-05 | -0.258 | - | - |
| 13299 | ENSP00000438465 | PGAM5 | 2.394E-05 | -0.258 | - | - |
| 13300 | ENSP00000263857 | POLR1A | 1.184E-05 | -0.258 | - | - |
| 13301 | ENSP00000334061 | HDAC6 | 1.793E-05 | -0.258 | - | - |
| 13302 | ENSP00000471539 | AC091150.1 | 2.040E-07 | -0.258 | - | - |
| 13303 | ENSP00000255992 | KRBA1 | 2.249E-05 | -0.258 | - | - |
| 13304 | ENSP00000380495 | DDX51 | 2.341E-05 | -0.258 | - | - |
| 13305 | ENSP00000358786 | KCNA10 | 2.195E-05 | -0.258 | - | - |
| 13306 | ENSP00000417078 | DHX36 | 1.482E-05 | -0.258 | - | - |
| 13307 | ENSP00000295628 | LRRC58 | 9.212E-06 | -0.258 | - | - |
| 13308 | ENSP00000302790 | XPO6 | 1.601E-05 | -0.258 | - | - |
| 13309 | ENSP00000263519 | ATP2B3 | 7.280E-06 | -0.258 | - | - |
| 13310 | ENSP00000352603 | AP4M1 | 2.318E-05 | -0.258 | - | - |
| 13311 | ENSP00000236192 | VAMP4 | 8.456E-06 | -0.258 | - | - |
| 13312 | ENSP00000180173 | MTMR7 | 2.200E-05 | -0.258 | - | - |
| 13313 | ENSP00000363970 | MAP1LC3A | 3.915E-05 | -0.258 | - | - |
| 13314 | ENSP00000420140 | FAM71F2 | 8.758E-07 | -0.258 | - | - |
| 13315 | ENSP00000327583 | RANBP1 | 1.691E-05 | -0.258 | - | - |
| 13316 | ENSP00000000233 | ARF5 | 9.319E-06 | -0.258 | - | - |
| 13317 | ENSP00000329287 | SNN | 1.863E-05 | -0.258 | - | - |
| 13318 | ENSP00000341885 | RPS2 | 1.193E-05 | -0.258 | - | - |
| 13319 | ENSP00000319673 | GPR89A | 1.919E-05 | -0.258 | - | - |
| 13320 | ENSP00000431814 | RP11-467M13.1 | 1.019E-05 | -0.258 | - | - |
| 13321 | ENSP00000253083 | HIP1R | 1.251E-05 | -0.258 | - | - |
| 13322 | ENSP00000437073 | MYO18A | 3.804E-05 | -0.258 | - | - |
| 13323 | ENSP00000297477 | TMEM184A | 2.962E-05 | -0.258 | - | - |
| 13324 | ENSP00000339030 | ZFP91 | 1.748E-05 | -0.258 | - | - |
| 13325 | ENSP00000386359 | PPP1R1C | 1.118E-05 | -0.258 | - | - |
| 13326 | ENSP00000429726 | FAM159A | 5.663E-07 | -0.258 | - | - |
| 13327 | ENSP00000435325 | RP11-1212A22.2 | 1.018E-05 | -0.259 | - | - |
| 13328 | ENSP00000379217 | UBFD1 | 1.549E-05 | -0.259 | - | - |
| 13329 | ENSP00000351832 | RAB3GAP2 | 2.639E-05 | -0.259 | - | - |
| 13330 | ENSP00000314776 | MBLAC2 | 1.336E-05 | -0.259 | - | - |
| 13331 | ENSP00000440847 | ZNF185 | 2.776E-05 | -0.259 | - | - |
| 13332 | ENSP00000322545 | ZNF543 | 1.847E-05 | -0.259 | - | - |
| 13333 | ENSP00000326240 | MYNN | 1.833E-05 | -0.259 | - | - |
| 13334 | ENSP00000253812 | PCDHGA3 | 2.709E-05 | -0.259 | - | - |
| 13335 | ENSP00000296144 | LRRC2 | 9.954E-06 | -0.259 | - | - |
| 13336 | ENSP00000337320 | ZNF664 | 1.586E-05 | -0.259 | - | - |
| 13337 | ENSP00000254235 | ADCY7 | 1.834E-05 | -0.259 | - | - |
| 13338 | ENSP00000369014 | NDNF | 1.464E-05 | -0.259 | - | - |
| 13339 | ENSP00000321711 | DIS3L | 1.375E-05 | -0.259 | - | - |
| 13340 | ENSP00000308845 | MRPS12 | 1.413E-05 | -0.259 | - | - |
| 13341 | ENSP00000261173 | ATP2B1 | 7.153E-06 | -0.259 | - | - |
| 13342 | ENSP00000373637 | SMYD3 | 9.666E-06 | -0.259 | - | - |
| 13343 | ENSP00000367721 | NUP160 | 2.385E-05 | -0.259 | - | - |
| 13344 | ENSP00000399863 | ZNF839 | 1.655E-07 | -0.259 | - | - |
| 13345 | ENSP00000259400 | STX17 | 9.349E-06 | -0.259 | - | - |
| 13346 | ENSP00000294753 | ZNF496 | 1.515E-05 | -0.259 | - | - |
| 13347 | ENSP00000296122 | PPP1CB | 2.227E-05 | -0.259 | - | - |
| 13348 | ENSP00000310521 | SEC22A | 8.439E-06 | -0.259 | - | - |
| 13349 | ENSP00000367104 | BFSP1 | 1.509E-05 | -0.259 | - | - |
| 13350 | ENSP00000322439 | TUFM | 1.719E-05 | -0.259 | - | - |
| 13351 | ENSP00000382951 | DEFB130 | 3.345E-06 | -0.259 | - | - |
| 13352 | ENSP00000433343 | FAM118B | 1.045E-05 | -0.259 | - | - |
| 13353 | ENSP00000425257 | - | 4.285E-06 | -0.259 | - | - |
| 13354 | ENSP00000445508 | CNOT4 | 2.182E-05 | -0.259 | - | - |
| 13355 | ENSP00000302319 | PRY | 3.362E-05 | -0.259 | - | - |
| 13356 | ENSP00000346017 | CALCB | 1.896E-05 | -0.259 | - | - |
| 13357 | ENSP00000305725 | CHST11 | 2.384E-05 | -0.259 | - | - |
| 13358 | ENSP00000312277 | ZNF507 | 1.409E-05 | -0.259 | - | - |
| 13359 | ENSP00000338887 | LRRC8D | 1.000E-05 | -0.259 | - | - |
| 13360 | ENSP00000356853 | UCK2 | 2.538E-05 | -0.259 | - | - |
| 13361 | ENSP00000357067 | KCNJ9 | 1.990E-05 | -0.259 | - | - |
| 13362 | ENSP00000250615 | AANAT | 1.447E-05 | -0.260 | - | - |
| 13363 | ENSP00000386357 | LRRTM4 | 1.505E-05 | -0.260 | - | - |
| 13364 | ENSP00000358932 | ABHD16B | 3.624E-06 | -0.260 | - | - |
| 13365 | ENSP00000269243 | MYH10 | 1.356E-05 | -0.260 | - | - |
| 13366 | ENSP00000383563 | ZG16 | 1.210E-05 | -0.260 | - | - |
| 13367 | ENSP00000276066 | RAB41 | 7.606E-06 | -0.260 | - | - |
| 13368 | ENSP00000278198 | LRRC4C | 8.978E-06 | -0.260 | - | - |
| 13369 | ENSP00000370194 | GMDS | 1.651E-05 | -0.260 | - | - |
| 13370 | ENSP00000359958 | ZRANB2 | 1.977E-05 | -0.260 | - | - |
| 13371 | ENSP00000262105 | MCM4 | 8.638E-06 | -0.260 | - | - |
| 13372 | ENSP00000351380 | TMA16 | 6.903E-06 | -0.260 | - | - |
| 13373 | ENSP00000384869 | CLHC1 | 2.294E-05 | -0.260 | - | - |
| 13374 | ENSP00000300107 | CLPX | 7.534E-06 | -0.260 | - | - |
| 13375 | ENSP00000338093 | TARSL2 | 7.091E-06 | -0.260 | - | - |
| 13376 | ENSP00000306524 | LRRC8E | 1.206E-05 | -0.260 | - | - |
| 13377 | ENSP00000279387 | PPP4C | 1.026E-05 | -0.260 | - | - |
| 13378 | ENSP00000304226 | HIPK3 | 1.330E-05 | -0.260 | - | - |
| 13379 | ENSP00000229812 | STK38 | 8.497E-06 | -0.260 | - | - |
| 13380 | ENSP00000353249 | AGBL5 | 1.771E-05 | -0.260 | - | - |
| 13381 | ENSP00000360549 | SLC1A7 | 2.206E-05 | -0.260 | - | - |
| 13382 | ENSP00000421853 | RANBP3L | 1.576E-05 | -0.260 | - | - |
| 13383 | ENSP00000462116 | SAP30BP | 1.008E-05 | -0.260 | - | - |
| 13384 | ENSP00000356912 | C1orf111 | 1.588E-06 | -0.260 | - | - |
| 13385 | ENSP00000254193 | SNRPA1 | 9.359E-06 | -0.260 | - | - |
| 13386 | ENSP00000356652 | CACYBP | 1.698E-05 | -0.260 | - | - |
| 13387 | ENSP00000306382 | MYO1B | 1.109E-05 | -0.260 | - | - |
| 13388 | ENSP00000333926 | ABCC5 | 1.724E-05 | -0.260 | - | - |
| 13389 | ENSP00000417451 | RBMXL3 | 8.182E-07 | -0.260 | - | - |
| 13390 | ENSP00000373539 | STAB2 | 1.742E-05 | -0.260 | - | - |
| 13391 | ENSP00000386609 | WDR81 | 1.375E-05 | -0.260 | - | - |
| 13392 | ENSP00000438863 | TMEM206 | 2.461E-06 | -0.260 | - | - |
| 13393 | ENSP00000399718 | MBIP | 1.755E-05 | -0.260 | - | - |
| 13394 | ENSP00000303766 | HTRA3 | 1.963E-05 | -0.260 | - | - |
| 13395 | ENSP00000346768 | UGT1A9 | 1.764E-05 | -0.260 | - | - |
| 13396 | ENSP00000365441 | CACNA1F | 1.006E-05 | -0.260 | - | - |
| 13397 | ENSP00000338217 | ZNF532 | 1.597E-05 | -0.260 | - | - |
| 13398 | ENSP00000301318 | ZFP28 | 1.249E-05 | -0.260 | - | - |
| 13399 | ENSP00000365877 | SUV39H1 | 1.957E-05 | -0.260 | - | - |
| 13400 | ENSP00000297265 | CHMP4C | 6.274E-06 | -0.260 | - | - |
| 13401 | ENSP00000415106 | KBTBD4 | 9.037E-06 | -0.260 | - | - |
| 13402 | ENSP00000284957 | RABGEF1 | 1.010E-05 | -0.260 | - | - |
| 13403 | ENSP00000379203 | ATP6V1C1 | 2.263E-05 | -0.260 | - | - |
| 13404 | ENSP00000359333 | FAM69A | 8.489E-06 | -0.260 | - | - |
| 13405 | ENSP00000302310 | ZNF396 | 1.526E-05 | -0.260 | - | - |
| 13406 | ENSP00000371936 | SEMA5A | 2.644E-05 | -0.260 | - | - |
| 13407 | ENSP00000300811 | ZNF428 | 2.350E-05 | -0.260 | - | - |
| 13408 | ENSP00000379156 | MPRIP | 1.736E-05 | -0.260 | - | - |
| 13409 | ENSP00000265563 | PRKAR2A | 1.609E-05 | -0.261 | - | - |
| 13410 | ENSP00000398350 | C11orf68 | 3.351E-06 | -0.261 | - | - |
| 13411 | ENSP00000385939 | ZNF12 | 1.560E-05 | -0.261 | - | - |
| 13412 | ENSP00000345491 | PCDHB13 | 1.033E-05 | -0.261 | - | - |
| 13413 | ENSP00000390407 | TMEM213 | 6.172E-06 | -0.261 | - | - |
| 13414 | ENSP00000262854 | HUWE1 | 1.144E-05 | -0.261 | - | - |
| 13415 | ENSP00000338562 | STX3 | 1.128E-05 | -0.261 | - | - |
| 13416 | ENSP00000262519 | SETD1A | 1.372E-05 | -0.261 | - | - |
| 13417 | ENSP00000295542 | DCST1 | 2.596E-05 | -0.261 | - | - |
| 13418 | ENSP00000344742 | STAMBP | 1.753E-05 | -0.261 | - | - |
| 13419 | ENSP00000333591 | SLC35D3 | 2.189E-05 | -0.261 | - | - |
| 13420 | ENSP00000227524 | PRPF19 | 7.298E-06 | -0.261 | - | - |
| 13421 | ENSP00000252590 | PLVAP | 2.385E-05 | -0.261 | - | - |
| 13422 | ENSP00000380037 | FITM2 | 1.179E-05 | -0.261 | - | - |
| 13423 | ENSP00000262891 | MARK4 | 1.424E-05 | -0.261 | - | - |
| 13424 | ENSP00000261292 | LIPG | 1.779E-05 | -0.261 | - | - |
| 13425 | ENSP00000296343 | KIAA0226 | 2.061E-05 | -0.261 | - | - |
| 13426 | ENSP00000235307 | C1orf21 | 1.078E-05 | -0.261 | - | - |
| 13427 | ENSP00000356379 | ASPM | 1.401E-05 | -0.261 | - | - |
| 13428 | ENSP00000289371 | EIF5B | 7.738E-06 | -0.261 | - | - |
| 13429 | ENSP00000358421 | HSD3B1 | 2.927E-05 | -0.261 | - | - |
| 13430 | ENSP00000441410 | ZNF135 | 2.981E-05 | -0.261 | - | - |
| 13431 | ENSP00000346534 | SCN8A | 2.330E-05 | -0.261 | - | - |
| 13432 | ENSP00000296127 | ZDHHC3 | 2.837E-05 | -0.261 | - | - |
| 13433 | ENSP00000259407 | BAAT | 1.835E-05 | -0.261 | - | - |
| 13434 | ENSP00000356634 | PAPPA2 | 1.916E-05 | -0.261 | - | - |
| 13435 | ENSP00000305777 | CSNK1G1 | 1.242E-05 | -0.261 | - | - |
| 13436 | ENSP00000389087 | NAGK | 1.086E-05 | -0.261 | - | - |
| 13437 | ENSP00000384109 | CUL4B | 1.763E-05 | -0.261 | - | - |
| 13438 | ENSP00000300119 | MYO1A | 1.168E-05 | -0.261 | - | - |
| 13439 | ENSP00000361790 | ZFP69 | 1.046E-05 | -0.261 | - | - |
| 13440 | ENSP00000312054 | C7orf10 | 2.387E-05 | -0.261 | - | - |
| 13441 | ENSP00000439467 | PDCD2 | 2.792E-05 | -0.261 | - | - |
| 13442 | ENSP00000387462 | ZBTB10 | 1.140E-05 | -0.261 | - | - |
| 13443 | ENSP00000305449 | CNNM3 | 1.075E-05 | -0.261 | - | - |
| 13444 | ENSP00000372295 | GJB2 | 2.436E-05 | -0.262 | - | - |
| 13445 | ENSP00000405700 | AL953854.2 | 3.729E-06 | -0.262 | - | - |
| 13446 | ENSP00000467556 | GPATCH8 | 3.905E-06 | -0.262 | - | - |
| 13447 | ENSP00000387282 | AGFG1 | 1.956E-05 | -0.262 | - | - |
| 13448 | ENSP00000386621 | PTGES3L-AARSD1 | 1.084E-05 | -0.262 | - | - |
| 13449 | ENSP00000433597 | PKD1P1 | 1.019E-05 | -0.262 | - | - |
| 13450 | ENSP00000360217 | RHAG | 1.554E-05 | -0.262 | - | - |
| 13451 | ENSP00000378181 | BRD7 | 1.516E-05 | -0.262 | - | - |
| 13452 | ENSP00000261531 | SNW1 | 1.393E-05 | -0.262 | - | - |
| 13453 | ENSP00000361867 | SEMG1 | 1.382E-05 | -0.262 | - | - |
| 13454 | ENSP00000341944 | LRRC66 | 1.356E-05 | -0.262 | - | - |
| 13455 | ENSP00000349496 | ADPRH | 1.562E-05 | -0.262 | - | - |
| 13456 | ENSP00000472802 | ZNF446 | 9.633E-06 | -0.262 | - | - |
| 13457 | ENSP00000453973 | EPDR1 | 5.728E-06 | -0.262 | - | - |
| 13458 | ENSP00000367398 | NPHP4 | 2.443E-05 | -0.262 | - | - |
| 13459 | ENSP00000234040 | PASK | 1.253E-05 | -0.262 | - | - |
| 13460 | ENSP00000324827 | AKAP17A | 1.294E-05 | -0.262 | - | - |
| 13461 | ENSP00000332116 | PDE4B | 1.546E-05 | -0.262 | - | - |
| 13462 | ENSP00000358810 | STRIP1 | 2.290E-05 | -0.262 | - | - |
| 13463 | ENSP00000334685 | ZNF568 | 1.354E-05 | -0.262 | - | - |
| 13464 | ENSP00000366156 | SRM | 5.488E-06 | -0.262 | - | - |
| 13465 | ENSP00000417601 | FBXL2 | 2.724E-05 | -0.262 | - | - |
| 13466 | ENSP00000335677 | FOXK2 | 1.008E-05 | -0.262 | - | - |
| 13467 | ENSP00000387654 | PCYOX1 | 2.776E-05 | -0.262 | - | - |
| 13468 | ENSP00000274766 | KAAG1 | 1.779E-05 | -0.262 | - | - |
| 13469 | ENSP00000388169 | ARPC4 | 1.461E-05 | -0.262 | - | - |
| 13470 | ENSP00000296591 | EDIL3 | 2.649E-05 | -0.262 | - | - |
| 13471 | ENSP00000367756 | UNC13B | 2.402E-05 | -0.262 | - | - |
| 13472 | ENSP00000318165 | OS9 | 2.003E-05 | -0.262 | - | - |
| 13473 | ENSP00000360991 | CYP4B1 | 1.911E-05 | -0.262 | - | - |
| 13474 | ENSP00000448598 | C17orf49 | 9.328E-06 | -0.262 | - | - |
| 13475 | ENSP00000430853 | 61E3.4 | 1.019E-05 | -0.262 | - | - |
| 13476 | ENSP00000288666 | RPS4Y2 | 8.278E-06 | -0.262 | - | - |
| 13477 | ENSP00000336661 | LRRC71 | 1.867E-05 | -0.262 | - | - |
| 13478 | ENSP00000296273 | RFC4 | 1.364E-05 | -0.262 | - | - |
| 13479 | ENSP00000296786 | UBLCP1 | 1.129E-05 | -0.262 | - | - |
| 13480 | ENSP00000377570 | KRT34 | 1.283E-05 | -0.263 | - | - |
| 13481 | ENSP00000310701 | TEAD2 | 2.174E-05 | -0.263 | - | - |
| 13482 | ENSP00000383587 | PNRC2 | 1.460E-05 | -0.263 | - | - |
| 13483 | ENSP00000386895 | FAM124B | 1.760E-05 | -0.263 | - | - |
| 13484 | ENSP00000354597 | DENND4B | 2.955E-05 | -0.263 | - | - |
| 13485 | ENSP00000261601 | USP14 | 1.326E-05 | -0.263 | - | - |
| 13486 | ENSP00000421981 | PAQR3 | 8.799E-06 | -0.263 | - | - |
| 13487 | ENSP00000311430 | RPL4 | 3.981E-05 | -0.263 | - | - |
| 13488 | ENSP00000263629 | MTIF2 | 1.058E-05 | -0.263 | - | - |
| 13489 | ENSP00000266659 | GLIPR1 | 2.161E-05 | -0.263 | - | - |
| 13490 | ENSP00000243189 | C1orf63 | 3.839E-05 | -0.263 | - | - |
| 13491 | ENSP00000254260 | RHPN2 | 6.453E-06 | -0.263 | - | - |
| 13492 | ENSP00000332805 | KRTAP8-1 | 1.448E-05 | -0.263 | - | - |
| 13493 | ENSP00000302657 | ABHD15 | 2.117E-05 | -0.263 | - | - |
| 13494 | ENSP00000425133 | SMG7 | 8.485E-06 | -0.263 | - | - |
| 13495 | ENSP00000438262 | TJP2 | 2.774E-05 | -0.263 | - | - |
| 13496 | ENSP00000281589 | PABPC3 | 1.138E-05 | -0.263 | - | - |
| 13497 | ENSP00000309161 | ZNF471 | 1.230E-05 | -0.263 | - | - |
| 13498 | ENSP00000331901 | EEF1G | 2.308E-05 | -0.263 | - | - |
| 13499 | ENSP00000432412 | ATG13 | 1.455E-05 | -0.263 | - | - |
| 13500 | ENSP00000344860 | IGFL3 | 7.868E-06 | -0.263 | - | - |
| 13501 | ENSP00000445677 | CLTCL1 | 2.603E-05 | -0.263 | - | - |
| 13502 | ENSP00000456743 | KLHL36 | 2.126E-06 | -0.263 | - | - |
| 13503 | ENSP00000371512 | SGCZ | 1.285E-05 | -0.263 | - | - |
| 13504 | ENSP00000261556 | TMEM260 | 3.575E-05 | -0.263 | - | - |
| 13505 | ENSP00000332681 | LRP2BP | 1.441E-05 | -0.263 | - | - |
| 13506 | ENSP00000317992 | NOC2L | 1.018E-05 | -0.263 | - | - |
| 13507 | ENSP00000417038 | PHACTR2 | 6.322E-06 | -0.263 | - | - |
| 13508 | ENSP00000323913 | TRIM55 | 1.050E-05 | -0.263 | - | - |
| 13509 | ENSP00000261973 | RBM25 | 1.430E-05 | -0.263 | - | - |
| 13510 | ENSP00000386951 | MED26 | 2.197E-05 | -0.263 | - | - |
| 13511 | ENSP00000381290 | CHTF8 | 3.648E-06 | -0.263 | - | - |
| 13512 | ENSP00000316898 | C12orf54 | 6.908E-06 | -0.263 | - | - |
| 13513 | ENSP00000259750 | TTBK1 | 1.167E-05 | -0.263 | - | - |
| 13514 | ENSP00000310126 | LINGO2 | 1.303E-05 | -0.263 | - | - |
| 13515 | ENSP00000264276 | ALS2 | 1.524E-05 | -0.263 | - | - |
| 13516 | ENSP00000296877 | LEAP2 | 2.793E-05 | -0.263 | - | - |
| 13517 | ENSP00000381167 | DYNC2H1 | 1.881E-05 | -0.263 | - | - |
| 13518 | ENSP00000320532 | WFDC9 | 3.784E-06 | -0.264 | - | - |
| 13519 | ENSP00000316605 | CNGB3 | 2.265E-05 | -0.264 | - | - |
| 13520 | ENSP00000238497 | VPS4B | 1.761E-05 | -0.264 | - | - |
| 13521 | ENSP00000432029 | RP11-719K4.2 | 1.019E-05 | -0.264 | - | - |
| 13522 | ENSP00000328484 | HIST1H2AJ | 1.636E-05 | -0.264 | - | - |
| 13523 | ENSP00000420075 | EFCC1 | 3.015E-06 | -0.264 | - | - |
| 13524 | ENSP00000259845 | PSORS1C2 | 5.440E-05 | -0.264 | - | - |
| 13525 | ENSP00000394394 | ANAPC7 | 1.373E-05 | -0.264 | - | - |
| 13526 | ENSP00000216297 | SUPT16H | 1.171E-05 | -0.264 | - | - |
| 13527 | ENSP00000351284 | RAD52 | 2.006E-05 | -0.264 | - | - |
| 13528 | ENSP00000348573 | AKAP9 | 1.524E-05 | -0.264 | - | - |
| 13529 | ENSP00000429065 | INTS9 | 1.579E-05 | -0.264 | - | - |
| 13530 | ENSP00000345055 | KCNQ5 | 2.121E-05 | -0.264 | - | - |
| 13531 | ENSP00000310661 | PDE7B | 9.140E-06 | -0.264 | - | - |
| 13532 | ENSP00000334430 | FAM9C | 1.464E-05 | -0.264 | - | - |
| 13533 | ENSP00000377971 | ANKLE1 | 1.237E-05 | -0.264 | - | - |
| 13534 | ENSP00000242827 | EBPL | 7.040E-06 | -0.264 | - | - |
| 13535 | ENSP00000357075 | TAGLN2 | 1.608E-05 | -0.264 | - | - |
| 13536 | ENSP00000337501 | NRIP2 | 1.017E-05 | -0.264 | - | - |
| 13537 | ENSP00000366953 | DCAF10 | 3.378E-06 | -0.264 | - | - |
| 13538 | ENSP00000326200 | ZBTB11 | 1.199E-05 | -0.264 | - | - |
| 13539 | ENSP00000302530 | BUB1 | 1.739E-05 | -0.264 | - | - |
| 13540 | ENSP00000314709 | ZHX2 | 2.581E-05 | -0.264 | - | - |
| 13541 | ENSP00000323816 | AHRR | 2.311E-05 | -0.264 | - | - |
| 13542 | ENSP00000369654 | HBD | 3.058E-05 | -0.264 | - | - |
| 13543 | ENSP00000318373 | ZNF513 | 1.423E-05 | -0.265 | - | - |
| 13544 | ENSP00000415030 | TMEM171 | 5.265E-06 | -0.265 | - | - |
| 13545 | ENSP00000427329 | FOXR2 | 1.641E-05 | -0.265 | - | - |
| 13546 | ENSP00000343633 | FOXN2 | 1.177E-05 | -0.265 | - | - |
| 13547 | ENSP00000363640 | PHF1 | 1.313E-05 | -0.265 | - | - |
| 13548 | ENSP00000298852 | PSMC3 | 1.551E-05 | -0.265 | - | - |
| 13549 | ENSP00000380318 | MPST | 2.054E-05 | -0.265 | - | - |
| 13550 | ENSP00000295488 | HELQ | 6.172E-06 | -0.265 | - | - |
| 13551 | ENSP00000259988 | FGFBP1 | 2.254E-05 | -0.265 | - | - |
| 13552 | ENSP00000357904 | LYSMD1 | 1.484E-05 | -0.265 | - | - |
| 13553 | ENSP00000409936 | MYO19 | 2.798E-05 | -0.265 | - | - |
| 13554 | ENSP00000434516 | TXNRD1 | 2.954E-05 | -0.265 | - | - |
| 13555 | ENSP00000273286 | LRTM1 | 9.172E-06 | -0.265 | - | - |
| 13556 | ENSP00000320757 | TMEM150B | 2.810E-05 | -0.265 | - | - |
| 13557 | ENSP00000386764 | ZC3H6 | 1.971E-05 | -0.265 | - | - |
| 13558 | ENSP00000392568 | DEFB130 | 3.345E-06 | -0.265 | - | - |
| 13559 | ENSP00000465517 | C18orf21 | 3.839E-06 | -0.265 | - | - |
| 13560 | ENSP00000311679 | ZNF483 | 1.062E-05 | -0.265 | - | - |
| 13561 | ENSP00000305647 | VAMP5 | 8.595E-06 | -0.265 | - | - |
| 13562 | ENSP00000168977 | NMRK2 | 7.582E-06 | -0.265 | - | - |
| 13563 | ENSP00000350673 | FAM3B | 1.403E-05 | -0.265 | - | - |
| 13564 | ENSP00000386331 | MYO7A | 1.484E-05 | -0.265 | - | - |
| 13565 | ENSP00000216639 | VRK1 | 9.682E-06 | -0.265 | - | - |
| 13566 | ENSP00000340019 | HSPD1 | 4.013E-05 | -0.265 | - | - |
| 13567 | ENSP00000390354 | HS3ST6 | 2.027E-05 | -0.265 | - | - |
| 13568 | ENSP00000295822 | EIF5A2 | 1.041E-05 | -0.265 | - | - |
| 13569 | ENSP00000393393 | RPL35A | 1.417E-05 | -0.265 | - | - |
| 13570 | ENSP00000282908 | STK32B | 1.266E-05 | -0.265 | - | - |
| 13571 | ENSP00000386657 | FAM183B | 7.065E-06 | -0.266 | - | - |
| 13572 | ENSP00000278816 | CSRP2BP | 1.001E-05 | -0.266 | - | - |
| 13573 | ENSP00000229264 | GNB3 | 1.021E-05 | -0.266 | - | - |
| 13574 | ENSP00000273398 | ATP6V1A | 1.275E-05 | -0.266 | - | - |
| 13575 | ENSP00000456333 | TMEM210 | 6.121E-07 | -0.266 | - | - |
| 13576 | ENSP00000285039 | MYO5B | 1.429E-05 | -0.266 | - | - |
| 13577 | ENSP00000349204 | CRB3 | 2.255E-05 | -0.266 | - | - |
| 13578 | ENSP00000367439 | RPP38 | 8.593E-06 | -0.266 | - | - |
| 13579 | ENSP00000325355 | CASKIN2 | 1.152E-05 | -0.266 | - | - |
| 13580 | ENSP00000282878 | RAB3C | 9.799E-06 | -0.266 | - | - |
| 13581 | ENSP00000411439 | PATE4 | 1.406E-06 | -0.266 | - | - |
| 13582 | ENSP00000337122 | ZNF354A | 1.259E-05 | -0.266 | - | - |
| 13583 | ENSP00000368572 | SAT1 | 2.439E-05 | -0.266 | - | - |
| 13584 | ENSP00000354982 | MT-CO3 | 2.269E-05 | -0.266 | - | - |
| 13585 | ENSP00000317123 | SNRNP200 | 9.804E-06 | -0.266 | - | - |
| 13586 | ENSP00000353422 | VPS13A | 1.404E-05 | -0.266 | - | - |
| 13587 | ENSP00000386796 | SCN7A | 4.298E-06 | -0.266 | - | - |
| 13588 | ENSP00000308022 | TADA2B | 6.006E-06 | -0.266 | - | - |
| 13589 | ENSP00000386452 | C17orf104 | 5.912E-07 | -0.266 | - | - |
| 13590 | ENSP00000406164 | GMNC | 3.955E-06 | -0.266 | - | - |
| 13591 | ENSP00000252744 | ZSWIM6 | 8.750E-06 | -0.266 | - | - |
| 13592 | ENSP00000308339 | SEC16B | 2.139E-05 | -0.266 | - | - |
| 13593 | ENSP00000359198 | PPDPF | 9.162E-06 | -0.267 | - | - |
| 13594 | ENSP00000373454 | SRRM3 | 2.033E-05 | -0.267 | - | - |
| 13595 | ENSP00000323183 | ZBTB2 | 1.621E-05 | -0.267 | - | - |
| 13596 | ENSP00000262719 | PHLPP1 | 1.175E-05 | -0.267 | - | - |
| 13597 | ENSP00000265773 | SMARCA2 | 1.534E-05 | -0.267 | - | - |
| 13598 | ENSP00000330460 | DDX59 | 4.855E-06 | -0.267 | - | - |
| 13599 | ENSP00000371243 | TEX33 | 1.152E-05 | -0.267 | - | - |
| 13600 | ENSP00000254090 | FMO5 | 1.719E-05 | -0.267 | - | - |
| 13601 | ENSP00000264932 | SDHA | 1.160E-05 | -0.267 | - | - |
| 13602 | ENSP00000351430 | GYPE | 1.454E-05 | -0.267 | - | - |
| 13603 | ENSP00000267812 | MFAP1 | 3.121E-05 | -0.267 | - | - |
| 13604 | ENSP00000395535 | MECP2 | 3.074E-05 | -0.267 | - | - |
| 13605 | ENSP00000376258 | RNF34 | 1.866E-05 | -0.267 | - | - |
| 13606 | ENSP00000368761 | ZNF827 | 1.364E-05 | -0.267 | - | - |
| 13607 | ENSP00000344364 | LRRC29 | 7.857E-06 | -0.267 | - | - |
| 13608 | ENSP00000366628 | GPR157 | 6.707E-06 | -0.267 | - | - |
| 13609 | ENSP00000267890 | TTBK2 | 1.189E-05 | -0.267 | - | - |
| 13610 | ENSP00000285814 | MKI67IP | 7.436E-06 | -0.267 | - | - |
| 13611 | ENSP00000264346 | HERC6 | 1.706E-05 | -0.267 | - | - |
| 13612 | ENSP00000314543 | MAGEB18 | 1.761E-05 | -0.267 | - | - |
| 13613 | ENSP00000262283 | OC90 | 2.710E-05 | -0.267 | - | - |
| 13614 | ENSP00000286749 | SLC28A1 | 2.189E-05 | -0.267 | - | - |
| 13615 | ENSP00000284856 | TMSB4Y | 2.330E-05 | -0.267 | - | - |
| 13616 | ENSP00000360998 | ANKRD22 | 2.839E-05 | -0.267 | - | - |
| 13617 | ENSP00000334285 | FAM170A | 1.903E-05 | -0.267 | - | - |
| 13618 | ENSP00000395220 | SYTL5 | 9.792E-06 | -0.267 | - | - |
| 13619 | ENSP00000287437 | NSMCE2 | 4.095E-05 | -0.267 | - | - |
| 13620 | ENSP00000361446 | POLR3A | 2.841E-05 | -0.267 | - | - |
| 13621 | ENSP00000305702 | KCTD19 | 5.206E-06 | -0.267 | - | - |
| 13622 | ENSP00000362392 | C9orf117 | 2.137E-06 | -0.267 | - | - |
| 13623 | ENSP00000317376 | MRPS11 | 6.066E-06 | -0.267 | - | - |
| 13624 | ENSP00000358865 | INA | 1.236E-05 | -0.267 | - | - |
| 13625 | ENSP00000222256 | RAB3A | 1.442E-05 | -0.267 | - | - |
| 13626 | ENSP00000324527 | MYO1D | 1.289E-05 | -0.267 | - | - |
| 13627 | ENSP00000414558 | ABHD4 | 3.567E-05 | -0.267 | - | - |
| 13628 | ENSP00000381086 | CXorf36 | 2.421E-05 | -0.267 | - | - |
| 13629 | ENSP00000430548 | PTDSS1 | 6.433E-06 | -0.267 | - | - |
| 13630 | ENSP00000264414 | CUL3 | 1.400E-05 | -0.267 | - | - |
| 13631 | ENSP00000317170 | TIGD2 | 2.127E-05 | -0.268 | - | - |
| 13632 | ENSP00000366314 | TRPM3 | 1.917E-05 | -0.268 | - | - |
| 13633 | ENSP00000251296 | IGSF21 | 4.336E-05 | -0.268 | - | - |
| 13634 | ENSP00000220244 | KIAA1199 | 2.422E-05 | -0.268 | - | - |
| 13635 | ENSP00000349297 | ACADVL | 2.623E-05 | -0.268 | - | - |
| 13636 | ENSP00000466051 | ZNF404 | 9.675E-06 | -0.268 | - | - |
| 13637 | ENSP00000313088 | NETO1 | 2.211E-05 | -0.268 | - | - |
| 13638 | ENSP00000274289 | PLK2 | 1.418E-05 | -0.268 | - | - |
| 13639 | ENSP00000331465 | ZNF84 | 1.402E-05 | -0.268 | - | - |
| 13640 | ENSP00000012443 | PPP5C | 7.558E-06 | -0.268 | - | - |
| 13641 | ENSP00000403910 | ATP5SL | 2.622E-05 | -0.268 | - | - |
| 13642 | ENSP00000384259 | ATG4B | 8.474E-06 | -0.268 | - | - |
| 13643 | ENSP00000330631 | RTN4RL1 | 9.833E-06 | -0.268 | - | - |
| 13644 | ENSP00000417970 | FAM120B | 1.057E-05 | -0.268 | - | - |
| 13645 | ENSP00000292586 | C5orf45 | 2.701E-05 | -0.268 | - | - |
| 13646 | ENSP00000251337 | GNAT2 | 1.154E-05 | -0.268 | - | - |
| 13647 | ENSP00000379866 | COL4A4 | 2.025E-05 | -0.268 | - | - |
| 13648 | ENSP00000455442 | NUPR1L | 1.178E-06 | -0.268 | - | - |
| 13649 | ENSP00000354723 | H2BFWT | 1.789E-05 | -0.268 | - | - |
| 13650 | ENSP00000302805 | DDI1 | 1.171E-05 | -0.268 | - | - |
| 13651 | ENSP00000053469 | GUCA1A | 1.564E-05 | -0.268 | - | - |
| 13652 | ENSP00000396192 | C1QTNF9B-AS1 | 8.010E-06 | -0.268 | - | - |
| 13653 | ENSP00000414208 | POM121C | 6.868E-06 | -0.268 | - | - |
| 13654 | ENSP00000470555 | KLK13 | 1.499E-05 | -0.268 | - | - |
| 13655 | ENSP00000264606 | HDAC4 | 1.442E-05 | -0.268 | - | - |
| 13656 | ENSP00000316809 | PITPNA | 9.936E-06 | -0.268 | - | - |
| 13657 | ENSP00000232564 | GNB4 | 9.224E-06 | -0.268 | - | - |
| 13658 | ENSP00000472703 | AL035252.1 | 2.680E-07 | -0.268 | - | - |
| 13659 | ENSP00000324172 | ATP2B2 | 9.065E-06 | -0.268 | - | - |
| 13660 | ENSP00000265174 | PAPSS1 | 1.686E-05 | -0.268 | - | - |
| 13661 | ENSP00000384965 | C22orf15 | 8.336E-07 | -0.268 | - | - |
| 13662 | ENSP00000344233 | LRRC43 | 8.619E-06 | -0.268 | - | - |
| 13663 | ENSP00000273353 | MYH15 | 1.139E-05 | -0.268 | - | - |
| 13664 | ENSP00000367910 | FANCG | 2.192E-05 | -0.268 | - | - |
| 13665 | ENSP00000334675 | ERCC6L | 1.855E-05 | -0.268 | - | - |
| 13666 | ENSP00000465213 | POP4 | 8.273E-06 | -0.268 | - | - |
| 13667 | ENSP00000246337 | UROD | 1.517E-05 | -0.268 | - | - |
| 13668 | ENSP00000359174 | SLC35A3 | 2.440E-05 | -0.269 | - | - |
| 13669 | ENSP00000377655 | SENP7 | 1.801E-05 | -0.269 | - | - |
| 13670 | ENSP00000333664 | ACAA1 | 2.223E-05 | -0.269 | - | - |
| 13671 | ENSP00000340514 | ZNF14 | 1.509E-05 | -0.269 | - | - |
| 13672 | ENSP00000253159 | ZNF236 | 1.321E-05 | -0.269 | - | - |
| 13673 | ENSP00000269025 | LRRC46 | 1.181E-05 | -0.269 | - | - |
| 13674 | ENSP00000428518 | GFRA2 | 9.397E-06 | -0.269 | - | - |
| 13675 | ENSP00000276014 | CCNB3 | 1.381E-05 | -0.269 | - | - |
| 13676 | ENSP00000394183 | CDKN2AIPNL | 1.380E-05 | -0.269 | - | - |
| 13677 | ENSP00000440485 | PSMD9 | 2.182E-05 | -0.269 | - | - |
| 13678 | ENSP00000352668 | RBM12 | 9.944E-06 | -0.269 | - | - |
| 13679 | ENSP00000428209 | UBE2V2 | 2.581E-05 | -0.269 | - | - |
| 13680 | ENSP00000240139 | PPP3CC | 8.602E-06 | -0.269 | - | - |
| 13681 | ENSP00000311766 | ATAD3B | 9.996E-06 | -0.269 | - | - |
| 13682 | ENSP00000391085 | GOLGA6D | 7.782E-06 | -0.269 | - | - |
| 13683 | ENSP00000420588 | TFAM | 1.961E-05 | -0.269 | - | - |
| 13684 | ENSP00000473092 | MUC8 | 7.847E-06 | -0.269 | - | - |
| 13685 | ENSP00000411825 | PDCD6IP | 2.400E-05 | -0.269 | - | - |
| 13686 | ENSP00000295087 | ARL5A | 1.111E-05 | -0.269 | - | - |
| 13687 | ENSP00000355899 | RRP15 | 4.904E-06 | -0.269 | - | - |
| 13688 | ENSP00000299300 | CCT2 | 1.990E-05 | -0.269 | - | - |
| 13689 | ENSP00000259335 | KIAA0368 | 7.807E-06 | -0.269 | - | - |
| 13690 | ENSP00000405738 | ESRP1 | 7.218E-06 | -0.269 | - | - |
| 13691 | ENSP00000352270 | CMTM5 | 2.806E-05 | -0.269 | - | - |
| 13692 | ENSP00000443459 | BCAT1 | 2.359E-05 | -0.269 | - | - |
| 13693 | ENSP00000237264 | TBPL1 | 9.380E-06 | -0.269 | - | - |
| 13694 | ENSP00000325377 | WDR33 | 1.766E-05 | -0.269 | - | - |
| 13695 | ENSP00000344460 | CBS | 4.979E-05 | -0.270 | - | - |
| 13696 | ENSP00000311505 | IQCB1 | 1.200E-05 | -0.270 | - | - |
| 13697 | ENSP00000363746 | WDR46 | 1.861E-05 | -0.270 | - | - |
| 13698 | ENSP00000353151 | HOXA4 | 1.973E-05 | -0.270 | - | - |
| 13699 | ENSP00000386759 | SETD2 | 2.803E-05 | -0.270 | - | - |
| 13700 | ENSP00000323811 | HACL1 | 8.853E-06 | -0.270 | - | - |
| 13701 | ENSP00000363944 | LAS1L | 4.650E-06 | -0.270 | - | - |
| 13702 | ENSP00000434442 | ARFGAP2 | 2.239E-05 | -0.270 | - | - |
| 13703 | ENSP00000345195 | UBQLN2 | 1.302E-05 | -0.270 | - | - |
| 13704 | ENSP00000343392 | XRCC3 | 2.375E-05 | -0.270 | - | - |
| 13705 | ENSP00000304642 | FAM134B | 1.068E-05 | -0.270 | - | - |
| 13706 | ENSP00000396732 | PRDM7 | 1.081E-05 | -0.270 | - | - |
| 13707 | ENSP00000301042 | ZNF641 | 2.029E-05 | -0.270 | - | - |
| 13708 | ENSP00000357838 | OAT | 2.715E-05 | -0.270 | - | - |
| 13709 | ENSP00000406145 | CTXN2 | 4.437E-06 | -0.270 | - | - |
| 13710 | ENSP00000371205 | C2orf48 | 7.860E-06 | -0.270 | - | - |
| 13711 | ENSP00000348258 | HIST1H4L | 1.704E-05 | -0.270 | - | - |
| 13712 | ENSP00000369579 | ANKDD1A | 1.116E-05 | -0.270 | - | - |
| 13713 | ENSP00000367623 | HSPA14 | 2.619E-05 | -0.270 | - | - |
| 13714 | ENSP00000232461 | GNAT1 | 1.053E-05 | -0.270 | - | - |
| 13715 | ENSP00000260983 | HECW2 | 2.035E-05 | -0.270 | - | - |
| 13716 | ENSP00000396995 | PAPD5 | 1.613E-05 | -0.270 | - | - |
| 13717 | ENSP00000439985 | MTRNR2L7 | 5.339E-06 | -0.270 | - | - |
| 13718 | ENSP00000406861 | MUC6 | 1.997E-05 | -0.270 | - | - |
| 13719 | ENSP00000258091 | CCT7 | 1.287E-05 | -0.270 | - | - |
| 13720 | ENSP00000357204 | CRABP2 | 2.167E-05 | -0.270 | - | - |
| 13721 | ENSP00000387739 | AMOTL1 | 1.588E-05 | -0.270 | - | - |
| 13722 | ENSP00000405574 | TBL1XR1 | 2.647E-05 | -0.270 | - | - |
| 13723 | ENSP00000285208 | RAB6B | 7.049E-06 | -0.270 | - | - |
| 13724 | ENSP00000355720 | JMJD4 | 4.908E-06 | -0.270 | - | - |
| 13725 | ENSP00000350364 | NEGR1 | 8.837E-06 | -0.270 | - | - |
| 13726 | ENSP00000255120 | NASP | 1.216E-05 | -0.271 | - | - |
| 13727 | ENSP00000264350 | HERC5 | 2.073E-05 | -0.271 | - | - |
| 13728 | ENSP00000330247 | NT5DC4 | 6.186E-06 | -0.271 | - | - |
| 13729 | ENSP00000320147 | EZH2 | 3.096E-05 | -0.271 | - | - |
| 13730 | ENSP00000315931 | AHCYL2 | 2.077E-05 | -0.271 | - | - |
| 13731 | ENSP00000309823 | FOXP4 | 9.563E-06 | -0.271 | - | - |
| 13732 | ENSP00000430031 | TXNRD3 | 1.551E-05 | -0.271 | - | - |
| 13733 | ENSP00000347883 | PLEKHA7 | 2.079E-05 | -0.271 | - | - |
| 13734 | ENSP00000450399 | DNM1L | 4.433E-05 | -0.271 | - | - |
| 13735 | ENSP00000262415 | DHX8 | 8.211E-06 | -0.271 | - | - |
| 13736 | ENSP00000432870 | CABYR | 1.019E-05 | -0.271 | - | - |
| 13737 | ENSP00000310878 | KLF14 | 2.192E-05 | -0.271 | - | - |
| 13738 | ENSP00000359334 | GABRA3 | 1.701E-05 | -0.271 | - | - |
| 13739 | ENSP00000348168 | GTF2E2 | 8.221E-06 | -0.271 | - | - |
| 13740 | ENSP00000359698 | UBTD1 | 1.446E-05 | -0.271 | - | - |
| 13741 | ENSP00000428343 | RGS21 | 1.448E-05 | -0.271 | - | - |
| 13742 | ENSP00000357442 | PYGO2 | 9.069E-06 | -0.271 | - | - |
| 13743 | ENSP00000371318 | TMEM74B | 1.397E-05 | -0.271 | - | - |
| 13744 | ENSP00000417175 | VAPB | 2.580E-05 | -0.271 | - | - |
| 13745 | ENSP00000377771 | SIPA1 | 2.318E-05 | -0.271 | - | - |
| 13746 | ENSP00000461295 | RP11-343C2.3 | 4.091E-06 | -0.271 | - | - |
| 13747 | ENSP00000331944 | AEN | 1.528E-05 | -0.271 | - | - |
| 13748 | ENSP00000430897 | CCDC71L | 1.774E-05 | -0.271 | - | - |
| 13749 | ENSP00000331385 | KIAA0825 | 1.756E-05 | -0.271 | - | - |
| 13750 | ENSP00000285667 | HSPA13 | 1.450E-05 | -0.271 | - | - |
| 13751 | ENSP00000278715 | HMBS | 1.122E-05 | -0.271 | - | - |
| 13752 | ENSP00000375639 | TFPT | 1.608E-05 | -0.271 | - | - |
| 13753 | ENSP00000340546 | KRTAP4-5 | 4.699E-06 | -0.271 | - | - |
| 13754 | ENSP00000263245 | ARFGAP3 | 1.488E-05 | -0.271 | - | - |
| 13755 | ENSP00000247138 | SLC35A2 | 1.834E-05 | -0.271 | - | - |
| 13756 | ENSP00000360860 | IFIT5 | 1.353E-05 | -0.271 | - | - |
| 13757 | ENSP00000378307 | PBRM1 | 1.390E-05 | -0.271 | - | - |
| 13758 | ENSP00000362762 | BAI2 | 4.952E-06 | -0.271 | - | - |
| 13759 | ENSP00000301067 | MLL2 | 1.488E-05 | -0.271 | - | - |
| 13760 | ENSP00000298746 | TRUB1 | 6.638E-06 | -0.271 | - | - |
| 13761 | ENSP00000355198 | MAGEB3 | 1.150E-05 | -0.272 | - | - |
| 13762 | ENSP00000355325 | PSMB5 | 2.176E-05 | -0.272 | - | - |
| 13763 | ENSP00000045083 | FAM65C | 4.470E-05 | -0.272 | - | - |
| 13764 | ENSP00000358617 | PHTF1 | 1.732E-05 | -0.272 | - | - |
| 13765 | ENSP00000377411 | NDUFA4L2 | 3.042E-06 | -0.272 | - | - |
| 13766 | ENSP00000383178 | DIAPH3 | 2.830E-05 | -0.272 | - | - |
| 13767 | ENSP00000277554 | NACC2 | 1.534E-05 | -0.272 | - | - |
| 13768 | ENSP00000363149 | SPDEF | 1.841E-05 | -0.272 | - | - |
| 13769 | ENSP00000354532 | PNP | 1.833E-05 | -0.272 | - | - |
| 13770 | ENSP00000323720 | MED14 | 1.387E-05 | -0.272 | - | - |
| 13771 | ENSP00000299601 | LEO1 | 7.856E-06 | -0.272 | - | - |
| 13772 | ENSP00000369411 | RFC3 | 2.620E-05 | -0.272 | - | - |
| 13773 | ENSP00000411904 | CPNE4 | 2.648E-05 | -0.272 | - | - |
| 13774 | ENSP00000304004 | FOXA3 | 1.573E-05 | -0.272 | - | - |
| 13775 | ENSP00000334072 | TMEM212 | 5.998E-06 | -0.272 | - | - |
| 13776 | ENSP00000216267 | BRD1 | 8.379E-06 | -0.272 | - | - |
| 13777 | ENSP00000220659 | BRF2 | 5.412E-06 | -0.272 | - | - |
| 13778 | ENSP00000307155 | RBMY1F | 1.537E-05 | -0.272 | - | - |
| 13779 | ENSP00000262646 | RAB2A | 1.215E-05 | -0.272 | - | - |
| 13780 | ENSP00000412365 | CDKL2 | 1.490E-05 | -0.272 | - | - |
| 13781 | ENSP00000307188 | ASL | 1.946E-05 | -0.272 | - | - |
| 13782 | ENSP00000439493 | HIST1H3C | 2.274E-05 | -0.272 | - | - |
| 13783 | ENSP00000336606 | RAD54B | 1.033E-05 | -0.272 | - | - |
| 13784 | ENSP00000337255 | KCNE1 | 1.036E-05 | -0.272 | - | - |
| 13785 | ENSP00000387851 | AC104809.3 | 1.878E-06 | -0.272 | - | - |
| 13786 | ENSP00000228347 | POLR3B | 5.193E-06 | -0.272 | - | - |
| 13787 | ENSP00000404151 | CEP170B | 1.444E-06 | -0.272 | - | - |
| 13788 | ENSP00000354201 | UBE2Z | 3.227E-05 | -0.272 | - | - |
| 13789 | ENSP00000384996 | TBC1D10A | 7.993E-06 | -0.272 | - | - |
| 13790 | ENSP00000428459 | TMEM200B | 7.388E-06 | -0.272 | - | - |
| 13791 | ENSP00000371308 | CENPJ | 1.352E-05 | -0.272 | - | - |
| 13792 | ENSP00000384744 | DERL3 | 9.121E-06 | -0.272 | - | - |
| 13793 | ENSP00000284425 | ABCA6 | 1.969E-05 | -0.272 | - | - |
| 13794 | ENSP00000295057 | LRRTM1 | 9.921E-06 | -0.272 | - | - |
| 13795 | ENSP00000368124 | CAMK1D | 1.866E-05 | -0.272 | - | - |
| 13796 | ENSP00000396163 | CLEC2A | 1.739E-05 | -0.273 | - | - |
| 13797 | ENSP00000351967 | C9orf169 | 3.186E-06 | -0.273 | - | - |
| 13798 | ENSP00000364166 | TMEM225 | 5.661E-06 | -0.273 | - | - |
| 13799 | ENSP00000440045 | ATP2A2 | 3.296E-05 | -0.273 | - | - |
| 13800 | ENSP00000352442 | HIST1H2BM | 1.722E-05 | -0.273 | - | - |
| 13801 | ENSP00000335084 | PPP1CC | 2.319E-05 | -0.273 | - | - |
| 13802 | ENSP00000293892 | MSLNL | 5.111E-06 | -0.273 | - | - |
| 13803 | ENSP00000382561 | C8orf22 | 2.211E-06 | -0.273 | - | - |
| 13804 | ENSP00000357674 | SNAPIN | 9.700E-06 | -0.273 | - | - |
| 13805 | ENSP00000435310 | WDR65 | 3.738E-06 | -0.273 | - | - |
| 13806 | ENSP00000355739 | ADCK3 | 7.736E-06 | -0.273 | - | - |
| 13807 | ENSP00000367075 | KLHL1 | 9.408E-06 | -0.273 | - | - |
| 13808 | ENSP00000263116 | RAB36 | 7.275E-06 | -0.273 | - | - |
| 13809 | ENSP00000341625 | CYB5A | 1.866E-05 | -0.273 | - | - |
| 13810 | ENSP00000366395 | SYVN1 | 2.207E-05 | -0.273 | - | - |
| 13811 | ENSP00000274711 | LRRTM2 | 9.730E-06 | -0.273 | - | - |
| 13812 | ENSP00000291576 | PWP2 | 6.470E-06 | -0.273 | - | - |
| 13813 | ENSP00000378890 | GCH1 | 2.832E-05 | -0.273 | - | - |
| 13814 | ENSP00000242275 | SLC25A51 | 1.587E-05 | -0.273 | - | - |
| 13815 | ENSP00000381132 | C12orf66 | 1.168E-05 | -0.273 | - | - |
| 13816 | ENSP00000379030 | TRIM16L | 5.945E-06 | -0.273 | - | - |
| 13817 | ENSP00000379282 | ABHD16A | 2.076E-05 | -0.273 | - | - |
| 13818 | ENSP00000315569 | DIS3L2 | 9.844E-06 | -0.273 | - | - |
| 13819 | ENSP00000303712 | RBMY1A1 | 2.931E-05 | -0.273 | - | - |
| 13820 | ENSP00000345359 | PODXL2 | 2.374E-05 | -0.274 | - | - |
| 13821 | ENSP00000409952 | VPS52 | 9.588E-06 | -0.274 | - | - |
| 13822 | ENSP00000216271 | HDAC10 | 7.979E-06 | -0.274 | - | - |
| 13823 | ENSP00000346634 | THRAP3 | 1.077E-05 | -0.274 | - | - |
| 13824 | ENSP00000298532 | SNAPC4 | 9.121E-06 | -0.274 | - | - |
| 13825 | ENSP00000357555 | RPS27 | 1.769E-05 | -0.274 | - | - |
| 13826 | ENSP00000338160 | - | 2.801E-05 | -0.274 | - | - |
| 13827 | ENSP00000347168 | HIST1H4J | 1.640E-05 | -0.274 | - | - |
| 13828 | ENSP00000270221 | EMP3 | 2.770E-05 | -0.274 | - | - |
| 13829 | ENSP00000239462 | TNN | 1.644E-05 | -0.274 | - | - |
| 13830 | ENSP00000349823 | SMARCAL1 | 1.300E-05 | -0.274 | - | - |
| 13831 | ENSP00000368959 | REEP5 | 1.944E-05 | -0.274 | - | - |
| 13832 | ENSP00000360365 | MCTS1 | 1.635E-05 | -0.274 | - | - |
| 13833 | ENSP00000406579 | RP11-124D2.6 | 3.355E-06 | -0.274 | - | - |
| 13834 | ENSP00000254442 | WDR7 | 2.452E-05 | -0.274 | - | - |
| 13835 | ENSP00000331581 | C11orf87 | 3.500E-05 | -0.274 | - | - |
| 13836 | ENSP00000310572 | PSMC5 | 1.480E-05 | -0.274 | - | - |
| 13837 | ENSP00000314615 | ARFGAP1 | 1.457E-05 | -0.274 | - | - |
| 13838 | ENSP00000460850 | CLUAP1 | 6.094E-06 | -0.274 | - | - |
| 13839 | ENSP00000317334 | TCP1 | 2.524E-05 | -0.274 | - | - |
| 13840 | ENSP00000357917 | MLLT11 | 9.538E-06 | -0.274 | - | - |
| 13841 | ENSP00000367220 | ACTR2 | 2.406E-05 | -0.274 | - | - |
| 13842 | ENSP00000326804 | CUL1 | 3.170E-05 | -0.274 | - | - |
| 13843 | ENSP00000306010 | ARF4 | 1.270E-05 | -0.274 | - | - |
| 13844 | ENSP00000380675 | WEE2 | 1.989E-05 | -0.274 | - | - |
| 13845 | ENSP00000323148 | ZKSCAN1 | 1.709E-05 | -0.274 | - | - |
| 13846 | ENSP00000417470 | ZNF786 | 1.328E-05 | -0.274 | - | - |
| 13847 | ENSP00000322396 | RRS1 | 1.597E-05 | -0.274 | - | - |
| 13848 | ENSP00000377369 | C14orf166B | 2.436E-06 | -0.274 | - | - |
| 13849 | ENSP00000374359 | ZXDC | 1.716E-05 | -0.274 | - | - |
| 13850 | ENSP00000405573 | GPI | 2.473E-05 | -0.274 | - | - |
| 13851 | ENSP00000162023 | MEF2BNB-MEF2B | 9.995E-06 | -0.274 | - | - |
| 13852 | ENSP00000304643 | CALML6 | 9.759E-06 | -0.274 | - | - |
| 13853 | ENSP00000329200 | STAC3 | 3.340E-06 | -0.274 | - | - |
| 13854 | ENSP00000350934 | RABEP2 | 2.139E-05 | -0.274 | - | - |
| 13855 | ENSP00000331643 | PGBD2 | 2.316E-05 | -0.275 | - | - |
| 13856 | ENSP00000264677 | SERPINI2 | 2.166E-05 | -0.275 | - | - |
| 13857 | ENSP00000330601 | HHIPL1 | 2.121E-05 | -0.275 | - | - |
| 13858 | ENSP00000464872 | ZNF519 | 9.588E-06 | -0.275 | - | - |
| 13859 | ENSP00000375147 | KRTAP16-1 | 8.428E-06 | -0.275 | - | - |
| 13860 | ENSP00000385636 | OBSL1 | 1.267E-05 | -0.275 | - | - |
| 13861 | ENSP00000346600 | C11orf48 | 2.247E-05 | -0.275 | - | - |
| 13862 | ENSP00000350159 | HIST1H4K | 1.710E-05 | -0.275 | - | - |
| 13863 | ENSP00000299140 | SPATA19 | 2.198E-05 | -0.275 | - | - |
| 13864 | ENSP00000261636 | ARL1 | 9.159E-06 | -0.275 | - | - |
| 13865 | ENSP00000427690 | GYPB | 1.167E-05 | -0.275 | - | - |
| 13866 | ENSP00000363418 | MTFR1L | 5.468E-06 | -0.275 | - | - |
| 13867 | ENSP00000300190 | MS4A5 | 5.344E-06 | -0.275 | - | - |
| 13868 | ENSP00000314348 | DDX10 | 8.413E-06 | -0.275 | - | - |
| 13869 | ENSP00000386306 | SCN9A | 1.550E-05 | -0.275 | - | - |
| 13870 | ENSP00000353415 | PRKAR1B | 3.232E-05 | -0.275 | - | - |
| 13871 | ENSP00000273173 | SLC22A14 | 2.311E-05 | -0.275 | - | - |
| 13872 | ENSP00000268206 | EFTUD1 | 6.049E-06 | -0.275 | - | - |
| 13873 | ENSP00000459753 | AC027763.2 | 1.399E-06 | -0.275 | - | - |
| 13874 | ENSP00000324105 | ENO3 | 2.484E-05 | -0.275 | - | - |
| 13875 | ENSP00000337450 | CYP3A7 | 1.881E-05 | -0.275 | - | - |
| 13876 | ENSP00000350011 | C7orf50 | 1.228E-05 | -0.275 | - | - |
| 13877 | ENSP00000348455 | UVRAG | 1.685E-05 | -0.275 | - | - |
| 13878 | ENSP00000455258 | RP11-58C22.1 | 3.194E-06 | -0.275 | - | - |
| 13879 | ENSP00000423563 | H2AFY | 1.381E-05 | -0.275 | - | - |
| 13880 | ENSP00000415840 | RP11-295K3.1 | 6.696E-06 | -0.275 | - | - |
| 13881 | ENSP00000339795 | RPL7 | 7.262E-06 | -0.276 | - | - |
| 13882 | ENSP00000361512 | PRPS1 | 2.300E-05 | -0.276 | - | - |
| 13883 | ENSP00000295101 | KCNJ3 | 2.151E-05 | -0.276 | - | - |
| 13884 | ENSP00000371940 | FOXD4 | 9.888E-06 | -0.276 | - | - |
| 13885 | ENSP00000377954 | LARS | 2.373E-05 | -0.276 | - | - |
| 13886 | ENSP00000367319 | FBXO47 | 3.860E-06 | -0.276 | - | - |
| 13887 | ENSP00000282050 | ATP5A1 | 1.109E-05 | -0.276 | - | - |
| 13888 | ENSP00000301488 | CDK2AP2 | 3.036E-05 | -0.276 | - | - |
| 13889 | ENSP00000338127 | TESK1 | 1.224E-05 | -0.276 | - | - |
| 13890 | ENSP00000319716 | ZNF101 | 1.557E-05 | -0.276 | - | - |
| 13891 | ENSP00000356886 | C1orf110 | 1.437E-05 | -0.276 | - | - |
| 13892 | ENSP00000435634 | C1orf94 | 5.446E-06 | -0.276 | - | - |
| 13893 | ENSP00000432688 | PIGY | 4.989E-06 | -0.276 | - | - |
| 13894 | ENSP00000325868 | ODF3 | 5.283E-06 | -0.276 | - | - |
| 13895 | ENSP00000352834 | MYO1C | 1.355E-05 | -0.276 | - | - |
| 13896 | ENSP00000467396 | RP11-886H22.1 | 5.872E-06 | -0.276 | - | - |
| 13897 | ENSP00000295688 | CCT3 | 1.779E-05 | -0.276 | - | - |
| 13898 | ENSP00000378306 | PPP3CB | 2.278E-05 | -0.276 | - | - |
| 13899 | ENSP00000313983 | WHSC1L1 | 9.019E-06 | -0.276 | - | - |
| 13900 | ENSP00000381005 | KRTAP12-3 | 2.420E-06 | -0.276 | - | - |
| 13901 | ENSP00000352314 | KLHL14 | 1.766E-05 | -0.276 | - | - |
| 13902 | ENSP00000289820 | NPM2 | 2.064E-05 | -0.276 | - | - |
| 13903 | ENSP00000360660 | NPDC1 | 1.827E-05 | -0.276 | - | - |
| 13904 | ENSP00000349708 | ZMYM6 | 1.308E-05 | -0.276 | - | - |
| 13905 | ENSP00000353920 | ADARB1 | 1.215E-05 | -0.276 | - | - |
| 13906 | ENSP00000370430 | PNPLA4 | 1.738E-05 | -0.276 | - | - |
| 13907 | ENSP00000433457 | ZBTB44 | 9.133E-06 | -0.276 | - | - |
| 13908 | ENSP00000400717 | GNA13 | 2.119E-05 | -0.276 | - | - |
| 13909 | ENSP00000236166 | FMO6P | 2.653E-05 | -0.276 | - | - |
| 13910 | ENSP00000367888 | LRP4 | 1.225E-05 | -0.276 | - | - |
| 13911 | ENSP00000388996 | AP1M1 | 2.672E-05 | -0.277 | - | - |
| 13912 | ENSP00000403987 | C15orf40 | 3.915E-06 | -0.277 | - | - |
| 13913 | ENSP00000215376 | C19orf26 | 2.838E-05 | -0.277 | - | - |
| 13914 | ENSP00000363055 | ZWINT | 8.536E-06 | -0.277 | - | - |
| 13915 | ENSP00000392395 | CHFR | 1.701E-05 | -0.277 | - | - |
| 13916 | ENSP00000361345 | FAM78A | 1.388E-05 | -0.277 | - | - |
| 13917 | ENSP00000357307 | SYT11 | 2.493E-05 | -0.277 | - | - |
| 13918 | ENSP00000268043 | PIF1 | 1.456E-05 | -0.277 | - | - |
| 13919 | ENSP00000334375 | LRCH3 | 1.140E-05 | -0.277 | - | - |
| 13920 | ENSP00000423422 | C5orf17 | 7.157E-06 | -0.277 | - | - |
| 13921 | ENSP00000365570 | PRAF2 | 1.974E-05 | -0.277 | - | - |
| 13922 | ENSP00000397050 | PPM1N | 1.925E-05 | -0.277 | - | - |
| 13923 | ENSP00000354960 | COLGALT2 | 1.418E-05 | -0.277 | - | - |
| 13924 | ENSP00000419249 | RIIAD1 | 6.782E-07 | -0.277 | - | - |
| 13925 | ENSP00000295290 | PACRGL | 3.140E-05 | -0.277 | - | - |
| 13926 | ENSP00000417492 | FSD1L | 7.098E-06 | -0.277 | - | - |
| 13927 | ENSP00000412272 | - | 2.034E-06 | -0.277 | - | - |
| 13928 | ENSP00000350708 | RAD23B | 2.993E-05 | -0.277 | - | - |
| 13929 | ENSP00000384703 | DDX11 | 9.851E-06 | -0.277 | - | - |
| 13930 | ENSP00000263468 | KIAA1377 | 2.427E-05 | -0.277 | - | - |
| 13931 | ENSP00000346283 | HRCT1 | 8.957E-06 | -0.277 | - | - |
| 13932 | ENSP00000417587 | PEG10 | 2.225E-05 | -0.277 | - | - |
| 13933 | ENSP00000456645 | ALG9 | 4.587E-06 | -0.277 | - | - |
| 13934 | ENSP00000349824 | REPS2 | 7.820E-06 | -0.277 | - | - |
| 13935 | ENSP00000470652 | SLC35E1 | 5.866E-06 | -0.277 | - | - |
| 13936 | ENSP00000424328 | MRPS30 | 2.108E-05 | -0.277 | - | - |
| 13937 | ENSP00000380880 | PQLC1 | 2.311E-05 | -0.277 | - | - |
| 13938 | ENSP00000389182 | RPP30 | 8.269E-06 | -0.277 | - | - |
| 13939 | ENSP00000392896 | HSBP1 | 1.778E-05 | -0.277 | - | - |
| 13940 | ENSP00000295966 | C3orf67 | 2.935E-05 | -0.277 | - | - |
| 13941 | ENSP00000357669 | NPR1 | 1.160E-05 | -0.278 | - | - |
| 13942 | ENSP00000316909 | SLC17A8 | 1.190E-05 | -0.278 | - | - |
| 13943 | ENSP00000350509 | AIG1 | 1.268E-05 | -0.278 | - | - |
| 13944 | ENSP00000466433 | - | 3.160E-06 | -0.278 | - | - |
| 13945 | ENSP00000408632 | CDH13 | 2.710E-05 | -0.278 | - | - |
| 13946 | ENSP00000421915 | ZNF672 | 1.161E-05 | -0.278 | - | - |
| 13947 | ENSP00000345957 | RPS21 | 1.876E-05 | -0.278 | - | - |
| 13948 | ENSP00000362702 | NEK6 | 2.424E-05 | -0.278 | - | - |
| 13949 | ENSP00000347498 | ZFYVE19 | 1.078E-05 | -0.278 | - | - |
| 13950 | ENSP00000305682 | MRPL39 | 5.080E-06 | -0.278 | - | - |
| 13951 | ENSP00000260382 | LRRC49 | 9.560E-06 | -0.278 | - | - |
| 13952 | ENSP00000313877 | TSFM | 1.200E-05 | -0.278 | - | - |
| 13953 | ENSP00000383093 | RFPL3S | 8.036E-06 | -0.278 | - | - |
| 13954 | ENSP00000341139 | C3orf62 | 7.270E-07 | -0.278 | - | - |
| 13955 | ENSP00000340887 | C5orf47 | 5.849E-06 | -0.278 | - | - |
| 13956 | ENSP00000359233 | ABCD3 | 1.611E-05 | -0.278 | - | - |
| 13957 | ENSP00000275364 | GNA12 | 9.670E-06 | -0.278 | - | - |
| 13958 | ENSP00000357791 | HRNR | 1.250E-05 | -0.278 | - | - |
| 13959 | ENSP00000406201 | ZNF160 | 1.323E-05 | -0.278 | - | - |
| 13960 | ENSP00000257570 | OASL | 1.293E-05 | -0.278 | - | - |
| 13961 | ENSP00000265728 | DBF4 | 9.781E-06 | -0.278 | - | - |
| 13962 | ENSP00000387286 | RAB1A | 1.569E-05 | -0.278 | - | - |
| 13963 | ENSP00000270112 | HUNK | 9.472E-06 | -0.278 | - | - |
| 13964 | ENSP00000362718 | MTCH1 | 2.408E-05 | -0.278 | - | - |
| 13965 | ENSP00000365505 | PLP2 | 1.886E-05 | -0.278 | - | - |
| 13966 | ENSP00000370883 | NOMO2 | 1.358E-05 | -0.278 | - | - |
| 13967 | ENSP00000296736 | TIGD6 | 8.013E-06 | -0.278 | - | - |
| 13968 | ENSP00000261890 | RAB11A | 1.157E-05 | -0.278 | - | - |
| 13969 | ENSP00000324450 | - | 1.508E-05 | -0.278 | - | - |
| 13970 | ENSP00000356814 | DCAF6 | 1.778E-05 | -0.278 | - | - |
| 13971 | ENSP00000384683 | RNASE9 | 1.226E-06 | -0.278 | - | - |
| 13972 | ENSP00000384774 | HK1 | 1.653E-05 | -0.279 | - | - |
| 13973 | ENSP00000443070 | - | 3.330E-07 | -0.279 | - | - |
| 13974 | ENSP00000380024 | ING4 | 1.312E-05 | -0.279 | - | - |
| 13975 | ENSP00000342974 | ZNF791 | 1.378E-05 | -0.279 | - | - |
| 13976 | ENSP00000346810 | PLAGL1 | 1.303E-05 | -0.279 | - | - |
| 13977 | ENSP00000379712 | IQSEC2 | 1.764E-05 | -0.279 | - | - |
| 13978 | ENSP00000335397 | RTN4RL2 | 1.174E-05 | -0.279 | - | - |
| 13979 | ENSP00000393667 | FAM126B | 1.299E-05 | -0.279 | - | - |
| 13980 | ENSP00000318687 | HSPH1 | 2.150E-05 | -0.279 | - | - |
| 13981 | ENSP00000355533 | RYR2 | 2.453E-05 | -0.279 | - | - |
| 13982 | ENSP00000347821 | MORC4 | 1.064E-05 | -0.279 | - | - |
| 13983 | ENSP00000429633 | MYBL1 | 3.041E-05 | -0.279 | - | - |
| 13984 | ENSP00000365624 | GRIPAP1 | 1.367E-05 | -0.279 | - | - |
| 13985 | ENSP00000387347 | IQCA1 | 2.428E-05 | -0.279 | - | - |
| 13986 | ENSP00000256969 | C12orf39 | 1.900E-05 | -0.279 | - | - |
| 13987 | ENSP00000364694 | ASPN | 1.525E-05 | -0.279 | - | - |
| 13988 | ENSP00000355689 | GUK1 | 1.711E-05 | -0.279 | - | - |
| 13989 | ENSP00000369252 | LRIT3 | 1.457E-05 | -0.279 | - | - |
| 13990 | ENSP00000271715 | POGZ | 1.014E-05 | -0.279 | - | - |
| 13991 | ENSP00000400101 | SLC25A13 | 8.265E-06 | -0.279 | - | - |
| 13992 | ENSP00000344831 | SLC25A40 | 1.143E-05 | -0.279 | - | - |
| 13993 | ENSP00000375151 | KRTAP4-3 | 2.038E-06 | -0.279 | - | - |
| 13994 | ENSP00000298048 | MELK | 1.386E-05 | -0.279 | - | - |
| 13995 | ENSP00000308149 | AMZ1 | 2.479E-05 | -0.279 | - | - |
| 13996 | ENSP00000376103 | C19orf12 | 4.482E-06 | -0.279 | - | - |
| 13997 | ENSP00000336701 | RAD51C | 2.351E-05 | -0.279 | - | - |
| 13998 | ENSP00000346335 | NRDE2 | 2.272E-05 | -0.279 | - | - |
| 13999 | ENSP00000341083 | NPR2 | 1.549E-05 | -0.279 | - | - |
| 14000 | ENSP00000368605 | ACOT9 | 6.054E-06 | -0.279 | - | - |
| 14001 | ENSP00000417291 | REPIN1 | 1.797E-05 | -0.280 | - | - |
| 14002 | ENSP00000329654 | WBSCR17 | 2.900E-05 | -0.280 | - | - |
| 14003 | ENSP00000264705 | CAD | 1.579E-05 | -0.280 | - | - |
| 14004 | ENSP00000415070 | ZNF177 | 1.138E-05 | -0.280 | - | - |
| 14005 | ENSP00000369003 | TRPC4 | 1.638E-05 | -0.280 | - | - |
| 14006 | ENSP00000255304 | USPL1 | 1.161E-05 | -0.280 | - | - |
| 14007 | ENSP00000301466 | SOAT2 | 8.751E-06 | -0.280 | - | - |
| 14008 | ENSP00000285928 | LRGUK | 1.299E-05 | -0.280 | - | - |
| 14009 | ENSP00000352427 | RGR | 9.206E-06 | -0.280 | - | - |
| 14010 | ENSP00000348054 | COMMD6 | 1.950E-05 | -0.280 | - | - |
| 14011 | ENSP00000403400 | CCPG1 | 1.372E-05 | -0.280 | - | - |
| 14012 | ENSP00000382274 | NOMO3 | 1.607E-05 | -0.280 | - | - |
| 14013 | ENSP00000457780 | AC073043.2 | 5.295E-06 | -0.280 | - | - |
| 14014 | ENSP00000374037 | VPS9D1 | 4.965E-06 | -0.280 | - | - |
| 14015 | ENSP00000360532 | CDC5L | 1.745E-05 | -0.280 | - | - |
| 14016 | ENSP00000314311 | LARP7 | 1.288E-05 | -0.280 | - | - |
| 14017 | ENSP00000355077 | NSMCE1 | 8.159E-06 | -0.280 | - | - |
| 14018 | ENSP00000387907 | ANKUB1 | 8.448E-06 | -0.281 | - | - |
| 14019 | ENSP00000391200 | ZNF692 | 1.854E-05 | -0.281 | - | - |
| 14020 | ENSP00000358417 | PHGDH | 1.595E-05 | -0.281 | - | - |
| 14021 | ENSP00000341677 | TPD52L3 | 1.703E-05 | -0.281 | - | - |
| 14022 | ENSP00000348554 | CDC16 | 2.694E-05 | -0.281 | - | - |
| 14023 | ENSP00000345270 | MGAT3 | 1.816E-05 | -0.281 | - | - |
| 14024 | ENSP00000384169 | FBLN2 | 1.826E-05 | -0.281 | - | - |
| 14025 | ENSP00000317468 | CHMP6 | 5.928E-06 | -0.281 | - | - |
| 14026 | ENSP00000360170 | ANGPTL3 | 2.013E-05 | -0.281 | - | - |
| 14027 | ENSP00000418823 | FTO | 2.687E-05 | -0.281 | - | - |
| 14028 | ENSP00000417128 | DEFB121 | 2.640E-06 | -0.281 | - | - |
| 14029 | ENSP00000422338 | FAM173B | 3.935E-06 | -0.281 | - | - |
| 14030 | ENSP00000470257 | hsa-mir-150 | 1.650E-06 | -0.281 | - | - |
| 14031 | ENSP00000334681 | DEFB107A | 3.349E-06 | -0.281 | - | - |
| 14032 | ENSP00000255189 | DMGDH | 5.135E-06 | -0.281 | - | - |
| 14033 | ENSP00000417085 | SLC15A2 | 2.245E-05 | -0.281 | - | - |
| 14034 | ENSP00000190165 | DMRT3 | 1.500E-05 | -0.281 | - | - |
| 14035 | ENSP00000414055 | NRBP2 | 2.329E-05 | -0.281 | - | - |
| 14036 | ENSP00000473172 | RUVBL2 | 3.169E-05 | -0.281 | - | - |
| 14037 | ENSP00000370227 | AREGB | 3.812E-06 | -0.281 | - | - |
| 14038 | ENSP00000265074 | NPR3 | 7.636E-06 | -0.281 | - | - |
| 14039 | ENSP00000273221 | IQSEC1 | 1.619E-05 | -0.281 | - | - |
| 14040 | ENSP00000415836 | ZNF276 | 9.376E-06 | -0.281 | - | - |
| 14041 | ENSP00000332615 | FAM169B | 8.853E-06 | -0.281 | - | - |
| 14042 | ENSP00000418348 | CMC1 | 1.943E-05 | -0.281 | - | - |
| 14043 | ENSP00000319796 | SERINC4 | 1.719E-05 | -0.281 | - | - |
| 14044 | ENSP00000359025 | STXBP3 | 2.168E-05 | -0.281 | - | - |
| 14045 | ENSP00000380869 | RAB2B | 1.567E-05 | -0.281 | - | - |
| 14046 | ENSP00000452571 | GATSL2 | 2.405E-06 | -0.281 | - | - |
| 14047 | ENSP00000305603 | FUT3 | 1.910E-05 | -0.281 | - | - |
| 14048 | ENSP00000364486 | FBP2 | 1.449E-05 | -0.281 | - | - |
| 14049 | ENSP00000242317 | DNAI1 | 1.670E-05 | -0.281 | - | - |
| 14050 | ENSP00000411012 | NSMAF | 1.846E-05 | -0.282 | - | - |
| 14051 | ENSP00000329794 | TCEAL7 | 3.060E-06 | -0.282 | - | - |
| 14052 | ENSP00000320303 | DNAJC28 | 6.890E-06 | -0.282 | - | - |
| 14053 | ENSP00000331681 | DGCR6 | 9.520E-06 | -0.282 | - | - |
| 14054 | ENSP00000351137 | XAB2 | 1.338E-05 | -0.282 | - | - |
| 14055 | ENSP00000310189 | MOB1B | 1.370E-05 | -0.282 | - | - |
| 14056 | ENSP00000292644 | PSMC2 | 1.430E-05 | -0.282 | - | - |
| 14057 | ENSP00000419599 | DPH3 | 6.958E-06 | -0.282 | - | - |
| 14058 | ENSP00000382271 | CEP152 | 1.925E-05 | -0.282 | - | - |
| 14059 | ENSP00000335612 | SPATA21 | 1.360E-05 | -0.282 | - | - |
| 14060 | ENSP00000352447 | C1orf116 | 1.662E-05 | -0.282 | - | - |
| 14061 | ENSP00000385481 | METTL21A | 7.015E-06 | -0.282 | - | - |
| 14062 | ENSP00000265717 | PRKAR2B | 1.234E-05 | -0.282 | - | - |
| 14063 | ENSP00000342381 | VRK2 | 1.612E-05 | -0.282 | - | - |
| 14064 | ENSP00000355013 | CTR9 | 1.177E-05 | -0.282 | - | - |
| 14065 | ENSP00000401502 | SIGLEC6 | 8.582E-06 | -0.282 | - | - |
| 14066 | ENSP00000377384 | PSMC3IP | 1.313E-05 | -0.282 | - | - |
| 14067 | ENSP00000290431 | PKD2L2 | 2.005E-05 | -0.282 | - | - |
| 14068 | ENSP00000310042 | ZNF622 | 1.753E-05 | -0.282 | - | - |
| 14069 | ENSP00000221498 | DKKL1 | 2.251E-05 | -0.282 | - | - |
| 14070 | ENSP00000394415 | PTGES3L | 1.004E-05 | -0.282 | - | - |
| 14071 | ENSP00000368801 | WBP4 | 1.307E-05 | -0.282 | - | - |
| 14072 | ENSP00000397566 | GTF2IRD1 | 1.324E-05 | -0.282 | - | - |
| 14073 | ENSP00000436496 | RP11-481A20.11 | 3.333E-06 | -0.282 | - | - |
| 14074 | ENSP00000387361 | SBNO1 | 9.259E-06 | -0.282 | - | - |
| 14075 | ENSP00000358153 | HIST2H4B | 2.003E-05 | -0.283 | - | - |
| 14076 | ENSP00000366203 | C1orf127 | 8.123E-06 | -0.283 | - | - |
| 14077 | ENSP00000270235 | NTN5 | 2.696E-05 | -0.283 | - | - |
| 14078 | ENSP00000410452 | STMN1 | 1.868E-05 | -0.283 | - | - |
| 14079 | ENSP00000282141 | CRYGC | 2.484E-05 | -0.283 | - | - |
| 14080 | ENSP00000352572 | PCNT | 2.221E-05 | -0.283 | - | - |
| 14081 | ENSP00000333667 | SHMT2 | 1.456E-05 | -0.283 | - | - |
| 14082 | ENSP00000315635 | TMEM25 | 1.630E-05 | -0.283 | - | - |
| 14083 | ENSP00000357149 | CD1E | 1.203E-05 | -0.283 | - | - |
| 14084 | ENSP00000345772 | TEAD3 | 1.607E-05 | -0.283 | - | - |
| 14085 | ENSP00000424198 | KLHL2 | 2.043E-05 | -0.283 | - | - |
| 14086 | ENSP00000388606 | GOLGA6L10 | 6.497E-06 | -0.283 | - | - |
| 14087 | ENSP00000439228 | MTRNR2L1 | 6.639E-06 | -0.283 | - | - |
| 14088 | ENSP00000229570 | SMIM8 | 5.480E-05 | -0.283 | - | - |
| 14089 | ENSP00000353013 | CLEC14A | 1.299E-05 | -0.283 | - | - |
| 14090 | ENSP00000346987 | ANAPC13 | 1.324E-05 | -0.283 | - | - |
| 14091 | ENSP00000411584 | MSANTD1 | 3.394E-06 | -0.283 | - | - |
| 14092 | ENSP00000420321 | ROBO1 | 2.442E-05 | -0.283 | - | - |
| 14093 | ENSP00000220940 | GML | 2.541E-05 | -0.283 | - | - |
| 14094 | ENSP00000348821 | MYOM1 | 1.853E-05 | -0.283 | - | - |
| 14095 | ENSP00000335500 | C7orf57 | 6.854E-06 | -0.283 | - | - |
| 14096 | ENSP00000315152 | METAP1D | 1.896E-05 | -0.283 | - | - |
| 14097 | ENSP00000326424 | NADSYN1 | 5.272E-06 | -0.284 | - | - |
| 14098 | ENSP00000386992 | IPO11 | 1.675E-05 | -0.284 | - | - |
| 14099 | ENSP00000402389 | C1QTNF5 | 6.275E-06 | -0.284 | - | - |
| 14100 | ENSP00000360317 | CYP2C8 | 1.628E-05 | -0.284 | - | - |
| 14101 | ENSP00000388275 | AGBL3 | 1.836E-05 | -0.284 | - | - |
| 14102 | ENSP00000361413 | FIBCD1 | 1.969E-05 | -0.284 | - | - |
| 14103 | ENSP00000450832 | SIPA1L1 | 2.506E-05 | -0.284 | - | - |
| 14104 | ENSP00000470123 | ZSCAN18 | 1.382E-05 | -0.284 | - | - |
| 14105 | ENSP00000360329 | TTC4 | 9.813E-06 | -0.284 | - | - |
| 14106 | ENSP00000311579 | TNKS | 1.326E-05 | -0.284 | - | - |
| 14107 | ENSP00000422185 | SORCS2 | 8.162E-06 | -0.284 | - | - |
| 14108 | ENSP00000429148 | ZNF674 | 1.301E-05 | -0.284 | - | - |
| 14109 | ENSP00000423333 | USP51 | 5.193E-06 | -0.284 | - | - |
| 14110 | ENSP00000371722 | NRSN2 | 1.683E-06 | -0.284 | - | - |
| 14111 | ENSP00000279028 | OCSTAMP | 3.227E-05 | -0.284 | - | - |
| 14112 | ENSP00000359682 | BRS3 | 1.719E-05 | -0.284 | - | - |
| 14113 | ENSP00000367991 | 8-Sep | 2.104E-05 | -0.284 | - | - |
| 14114 | ENSP00000380308 | DYNC1I2 | 2.076E-05 | -0.284 | - | - |
| 14115 | ENSP00000393860 | PLEKHA2 | 1.540E-05 | -0.284 | - | - |
| 14116 | ENSP00000346576 | SYTL2 | 2.279E-05 | -0.284 | - | - |
| 14117 | ENSP00000064780 | RELT | 2.614E-05 | -0.284 | - | - |
| 14118 | ENSP00000402343 | ZNF469 | 1.169E-05 | -0.284 | - | - |
| 14119 | ENSP00000464100 | RP11-51F16.8 | 2.303E-06 | -0.284 | - | - |
| 14120 | ENSP00000377007 | CENPN | 2.154E-05 | -0.284 | - | - |
| 14121 | ENSP00000370744 | ITPR2 | 1.777E-05 | -0.284 | - | - |
| 14122 | ENSP00000356632 | ABRACL | 1.280E-05 | -0.284 | - | - |
| 14123 | ENSP00000403980 | AC002365.1 | 2.514E-06 | -0.284 | - | - |
| 14124 | ENSP00000418511 | C21orf62 | 2.521E-06 | -0.284 | - | - |
| 14125 | ENSP00000374323 | EPHA6 | 2.125E-05 | -0.284 | - | - |
| 14126 | ENSP00000392057 | MTUS2 | 1.094E-05 | -0.284 | - | - |
| 14127 | ENSP00000337862 | OVOL1 | 1.729E-05 | -0.285 | - | - |
| 14128 | ENSP00000319052 | CETN1 | 1.209E-05 | -0.285 | - | - |
| 14129 | ENSP00000373278 | ZCWPW2 | 5.045E-06 | -0.285 | - | - |
| 14130 | ENSP00000328487 | CCDC87 | 1.124E-05 | -0.285 | - | - |
| 14131 | ENSP00000398617 | FAM71E2 | 4.938E-06 | -0.285 | - | - |
| 14132 | ENSP00000358714 | PRSS35 | 4.322E-06 | -0.285 | - | - |
| 14133 | ENSP00000353741 | ETF1 | 1.040E-05 | -0.285 | - | - |
| 14134 | ENSP00000470526 | MGC4771 | 2.546E-06 | -0.285 | - | - |
| 14135 | ENSP00000347742 | PDZD3 | 1.104E-05 | -0.285 | - | - |
| 14136 | ENSP00000357072 | SLAMF9 | 1.137E-05 | -0.285 | - | - |
| 14137 | ENSP00000310880 | MAL | 2.214E-05 | -0.285 | - | - |
| 14138 | ENSP00000311695 | CCDC85B | 2.103E-05 | -0.285 | - | - |
| 14139 | ENSP00000324464 | CSNK1D | 1.704E-05 | -0.285 | - | - |
| 14140 | ENSP00000386105 | POLD2 | 1.838E-05 | -0.285 | - | - |
| 14141 | ENSP00000363411 | GNG10 | 1.350E-05 | -0.285 | - | - |
| 14142 | ENSP00000344470 | LRRC39 | 9.190E-06 | -0.285 | - | - |
| 14143 | ENSP00000472832 | AC090427.1 | 3.930E-07 | -0.285 | - | - |
| 14144 | ENSP00000256925 | CABLES1 | 1.133E-05 | -0.285 | - | - |
| 14145 | ENSP00000352614 | ZSWIM5 | 9.770E-06 | -0.285 | - | - |
| 14146 | ENSP00000286049 | XAGE2B | 5.028E-06 | -0.285 | - | - |
| 14147 | ENSP00000352941 | KCTD18 | 8.774E-06 | -0.285 | - | - |
| 14148 | ENSP00000404375 | RPL36A | 9.803E-06 | -0.285 | - | - |
| 14149 | ENSP00000354461 | FAM109A | 1.633E-05 | -0.285 | - | - |
| 14150 | ENSP00000324651 | TMEM108 | 2.291E-05 | -0.285 | - | - |
| 14151 | ENSP00000354490 | ATP1A2 | 2.280E-05 | -0.285 | - | - |
| 14152 | ENSP00000366299 | SLC25A39 | 1.551E-05 | -0.285 | - | - |
| 14153 | ENSP00000347140 | ASGR2 | 5.977E-06 | -0.285 | - | - |
| 14154 | ENSP00000384363 | GDPD3 | 1.916E-05 | -0.286 | - | - |
| 14155 | ENSP00000368226 | GK | 2.406E-05 | -0.286 | - | - |
| 14156 | ENSP00000320488 | NAP1L5 | 8.732E-06 | -0.286 | - | - |
| 14157 | ENSP00000257637 | TMEM243 | 2.747E-05 | -0.286 | - | - |
| 14158 | ENSP00000378201 | ZBTB1 | 1.695E-05 | -0.286 | - | - |
| 14159 | ENSP00000405218 | ZNF347 | 1.164E-05 | -0.286 | - | - |
| 14160 | ENSP00000357311 | CENPW | 1.783E-05 | -0.286 | - | - |
| 14161 | ENSP00000467286 | ZNF763 | 8.704E-06 | -0.286 | - | - |
| 14162 | ENSP00000376871 | RABL2A | 2.274E-05 | -0.286 | - | - |
| 14163 | ENSP00000293441 | SHANK1 | 9.225E-06 | -0.286 | - | - |
| 14164 | ENSP00000358708 | SNAP91 | 1.580E-05 | -0.286 | - | - |
| 14165 | ENSP00000268057 | BBS4 | 1.016E-05 | -0.286 | - | - |
| 14166 | ENSP00000329614 | YBEY | 1.777E-05 | -0.286 | - | - |
| 14167 | ENSP00000265294 | GABRP | 1.971E-05 | -0.286 | - | - |
| 14168 | ENSP00000342026 | PRDX6 | 2.311E-05 | -0.286 | - | - |
| 14169 | ENSP00000381795 | CCDC33 | 7.158E-06 | -0.286 | - | - |
| 14170 | ENSP00000300784 | FN3K | 2.148E-05 | -0.286 | - | - |
| 14171 | ENSP00000340672 | ARMCX3 | 3.233E-06 | -0.286 | - | - |
| 14172 | ENSP00000446872 | GATC | 1.771E-06 | -0.286 | - | - |
| 14173 | ENSP00000456010 | RP11-831H9.16 | 3.080E-06 | -0.286 | - | - |
| 14174 | ENSP00000377040 | AASS | 1.156E-05 | -0.286 | - | - |
| 14175 | ENSP00000295092 | FAM84A | 2.252E-05 | -0.287 | - | - |
| 14176 | ENSP00000360593 | SELRC1 | 1.214E-05 | -0.287 | - | - |
| 14177 | ENSP00000404438 | SOHLH1 | 6.446E-06 | -0.287 | - | - |
| 14178 | ENSP00000333012 | SS18L1 | 1.379E-05 | -0.287 | - | - |
| 14179 | ENSP00000359740 | ZNF451 | 1.293E-05 | -0.287 | - | - |
| 14180 | ENSP00000367705 | BCOR | 5.795E-06 | -0.287 | - | - |
| 14181 | ENSP00000350767 | HIST4H4 | 1.721E-05 | -0.287 | - | - |
| 14182 | ENSP00000420227 | - | 5.716E-06 | -0.287 | - | - |
| 14183 | ENSP00000254908 | PCBD2 | 1.923E-05 | -0.287 | - | - |
| 14184 | ENSP00000345656 | VAPA | 2.292E-05 | -0.287 | - | - |
| 14185 | ENSP00000365776 | TMC4 | 3.406E-06 | -0.287 | - | - |
| 14186 | ENSP00000363573 | SRSF10 | 9.763E-06 | -0.287 | - | - |
| 14187 | ENSP00000303248 | RXFP1 | 1.165E-05 | -0.287 | - | - |
| 14188 | ENSP00000440385 | AL138764.1 | 7.986E-08 | -0.287 | - | - |
| 14189 | ENSP00000384400 | CHKB | 1.806E-05 | -0.287 | - | - |
| 14190 | ENSP00000354340 | UBA3 | 2.853E-05 | -0.287 | - | - |
| 14191 | ENSP00000331500 | TEX19 | 1.403E-05 | -0.287 | - | - |
| 14192 | ENSP00000331127 | BOLA2 | 1.180E-05 | -0.287 | - | - |
| 14193 | ENSP00000450909 | SLIRP | 9.820E-06 | -0.287 | - | - |
| 14194 | ENSP00000251809 | SPAG1 | 7.893E-06 | -0.287 | - | - |
| 14195 | ENSP00000315203 | CREG2 | 2.341E-05 | -0.287 | - | - |
| 14196 | ENSP00000363463 | TMEM57 | 1.921E-05 | -0.287 | - | - |
| 14197 | ENSP00000361707 | TCEAL1 | 1.150E-05 | -0.287 | - | - |
| 14198 | ENSP00000322802 | TSPYL5 | 1.464E-05 | -0.287 | - | - |
| 14199 | ENSP00000362763 | ZBTB6 | 1.473E-05 | -0.287 | - | - |
| 14200 | ENSP00000379032 | EXT2 | 1.920E-05 | -0.287 | - | - |
| 14201 | ENSP00000411097 | ZNF275 | 1.025E-05 | -0.287 | - | - |
| 14202 | ENSP00000281722 | RBM46 | 2.955E-05 | -0.287 | - | - |
| 14203 | ENSP00000356355 | NEK7 | 1.988E-05 | -0.288 | - | - |
| 14204 | ENSP00000377793 | PRC1 | 1.698E-05 | -0.288 | - | - |
| 14205 | ENSP00000264644 | RAPGEFL1 | 2.475E-05 | -0.288 | - | - |
| 14206 | ENSP00000316030 | COL19A1 | 2.118E-05 | -0.288 | - | - |
| 14207 | ENSP00000373730 | H2AFJ | 1.272E-05 | -0.288 | - | - |
| 14208 | ENSP00000346251 | BPIFA1 | 1.134E-05 | -0.288 | - | - |
| 14209 | ENSP00000355437 | TRIM58 | 8.451E-06 | -0.288 | - | - |
| 14210 | ENSP00000362777 | ATOH7 | 2.529E-05 | -0.288 | - | - |
| 14211 | ENSP00000359329 | GABRQ | 2.015E-05 | -0.288 | - | - |
| 14212 | ENSP00000367792 | MORN1 | 1.683E-05 | -0.288 | - | - |
| 14213 | ENSP00000346080 | RPL22L1 | 1.528E-05 | -0.288 | - | - |
| 14214 | ENSP00000363506 | UQCC | 2.014E-05 | -0.288 | - | - |
| 14215 | ENSP00000304111 | ABCG4 | 1.623E-05 | -0.288 | - | - |
| 14216 | ENSP00000449241 | AGAP2 | 1.856E-05 | -0.288 | - | - |
| 14217 | ENSP00000303178 | CDY1B | 1.208E-05 | -0.288 | - | - |
| 14218 | ENSP00000314949 | POLR2A | 1.237E-05 | -0.288 | - | - |
| 14219 | ENSP00000402608 | CPS1 | 2.294E-05 | -0.288 | - | - |
| 14220 | ENSP00000239374 | CCDC170 | 1.811E-05 | -0.288 | - | - |
| 14221 | ENSP00000346693 | ELOVL2 | 2.171E-05 | -0.288 | - | - |
| 14222 | ENSP00000385892 | PLEKHB2 | 1.462E-05 | -0.288 | - | - |
| 14223 | ENSP00000360621 | ORC1 | 1.860E-05 | -0.288 | - | - |
| 14224 | ENSP00000319897 | FAM214B | 6.225E-06 | -0.288 | - | - |
| 14225 | ENSP00000342510 | CEP97 | 1.423E-05 | -0.288 | - | - |
| 14226 | ENSP00000379176 | CDKL1 | 1.621E-05 | -0.288 | - | - |
| 14227 | ENSP00000298943 | C1QL3 | 1.502E-05 | -0.288 | - | - |
| 14228 | ENSP00000402527 | SENP6 | 2.234E-05 | -0.289 | - | - |
| 14229 | ENSP00000335481 | EMID1 | 9.202E-06 | -0.289 | - | - |
| 14230 | ENSP00000363794 | HSD17B8 | 9.760E-06 | -0.289 | - | - |
| 14231 | ENSP00000456827 | UBE2F | 1.213E-05 | -0.289 | - | - |
| 14232 | ENSP00000439856 | CHERP | 1.974E-05 | -0.289 | - | - |
| 14233 | ENSP00000361592 | ERMAP | 8.193E-06 | -0.289 | - | - |
| 14234 | ENSP00000359778 | ELTD1 | 2.063E-05 | -0.289 | - | - |
| 14235 | ENSP00000392549 | TTLL3 | 8.129E-06 | -0.289 | - | - |
| 14236 | ENSP00000362296 | POU3F4 | 1.427E-05 | -0.289 | - | - |
| 14237 | ENSP00000300145 | XRCC6BP1 | 1.022E-05 | -0.289 | - | - |
| 14238 | ENSP00000374574 | DDX42 | 1.296E-05 | -0.289 | - | - |
| 14239 | ENSP00000361219 | GTF3C4 | 9.146E-06 | -0.289 | - | - |
| 14240 | ENSP00000361036 | LHFPL1 | 1.398E-05 | -0.289 | - | - |
| 14241 | ENSP00000345808 | ARHGAP12 | 1.745E-05 | -0.289 | - | - |
| 14242 | ENSP00000311837 | SNTG2 | 1.223E-05 | -0.289 | - | - |
| 14243 | ENSP00000295759 | GLB1L | 1.722E-05 | -0.289 | - | - |
| 14244 | ENSP00000350698 | DDX47 | 1.516E-05 | -0.289 | - | - |
| 14245 | ENSP00000333024 | PHF7 | 1.174E-05 | -0.289 | - | - |
| 14246 | ENSP00000355250 | KIAA0753 | 5.672E-06 | -0.289 | - | - |
| 14247 | ENSP00000377944 | UTP14A | 5.817E-06 | -0.289 | - | - |
| 14248 | ENSP00000251507 | RABGAP1L | 1.311E-05 | -0.289 | - | - |
| 14249 | ENSP00000322832 | ROGDI | 2.973E-05 | -0.289 | - | - |
| 14250 | ENSP00000358576 | DCLRE1B | 1.909E-05 | -0.289 | - | - |
| 14251 | ENSP00000324857 | SEMA6D | 2.010E-05 | -0.289 | - | - |
| 14252 | ENSP00000356263 | C6orf211 | 1.302E-05 | -0.289 | - | - |
| 14253 | ENSP00000308450 | CDC20 | 1.514E-05 | -0.289 | - | - |
| 14254 | ENSP00000350102 | TOR4A | 1.745E-05 | -0.289 | - | - |
| 14255 | ENSP00000261669 | CAB39L | 1.946E-05 | -0.289 | - | - |
| 14256 | ENSP00000392270 | STRAP | 1.906E-05 | -0.289 | - | - |
| 14257 | ENSP00000297459 | TMEM74 | 2.626E-05 | -0.290 | - | - |
| 14258 | ENSP00000341412 | C11orf34 | 2.520E-05 | -0.290 | - | - |
| 14259 | ENSP00000348565 | UBA5 | 2.055E-05 | -0.290 | - | - |
| 14260 | ENSP00000332674 | LRRC8B | 1.002E-05 | -0.290 | - | - |
| 14261 | ENSP00000354841 | ABHD14B | 2.261E-05 | -0.290 | - | - |
| 14262 | ENSP00000316012 | SPAG11A | 4.297E-06 | -0.290 | - | - |
| 14263 | ENSP00000360017 | ZNF280C | 1.539E-05 | -0.290 | - | - |
| 14264 | ENSP00000410007 | FBXO46 | 1.375E-05 | -0.290 | - | - |
| 14265 | ENSP00000345144 | RCBTB2 | 1.557E-05 | -0.290 | - | - |
| 14266 | ENSP00000361502 | HYI | 1.295E-05 | -0.290 | - | - |
| 14267 | ENSP00000354927 | MAP3K3 | 2.523E-05 | -0.290 | - | - |
| 14268 | ENSP00000388220 | SGK110 | 3.971E-06 | -0.290 | - | - |
| 14269 | ENSP00000365426 | GGACT | 1.297E-05 | -0.290 | - | - |
| 14270 | ENSP00000325655 | CHCHD5 | 1.295E-06 | -0.290 | - | - |
| 14271 | ENSP00000351642 | BCAS4 | 8.817E-06 | -0.290 | - | - |
| 14272 | ENSP00000346045 | RPS17 | 1.129E-05 | -0.290 | - | - |
| 14273 | ENSP00000432487 | SAAL1 | 8.555E-06 | -0.290 | - | - |
| 14274 | ENSP00000276326 | FBXO25 | 1.499E-04 | -0.290 | - | - |
| 14275 | ENSP00000280772 | ANK3 | 1.159E-05 | -0.290 | - | - |
| 14276 | ENSP00000355133 | NOS1AP | 1.467E-05 | -0.290 | - | - |
| 14277 | ENSP00000466950 | TAF15 | 7.366E-06 | -0.290 | - | - |
| 14278 | ENSP00000382390 | NEURL4 | 8.319E-06 | -0.291 | - | - |
| 14279 | ENSP00000339027 | RPLP0 | 1.148E-05 | -0.291 | - | - |
| 14280 | ENSP00000368109 | ACCSL | 4.988E-06 | -0.291 | - | - |
| 14281 | ENSP00000432768 | CKAP5 | 1.308E-05 | -0.291 | - | - |
| 14282 | ENSP00000448323 | BRF1 | 1.576E-05 | -0.291 | - | - |
| 14283 | ENSP00000265709 | ANK1 | 1.107E-05 | -0.291 | - | - |
| 14284 | ENSP00000364249 | PLA2G5 | 1.991E-05 | -0.291 | - | - |
| 14285 | ENSP00000339830 | TMEM220 | 2.319E-05 | -0.291 | - | - |
| 14286 | ENSP00000303437 | TSNARE1 | 7.858E-06 | -0.291 | - | - |
| 14287 | ENSP00000350965 | TMBIM4 | 9.970E-06 | -0.291 | - | - |
| 14288 | ENSP00000349568 | WDR96 | 2.663E-06 | -0.291 | - | - |
| 14289 | ENSP00000356170 | ETNK2 | 1.968E-05 | -0.291 | - | - |
| 14290 | ENSP00000168216 | HSD17B10 | 6.257E-06 | -0.291 | - | - |
| 14291 | ENSP00000333534 | CYP2F1 | 1.676E-05 | -0.291 | - | - |
| 14292 | ENSP00000354568 | BRDT | 2.229E-05 | -0.291 | - | - |
| 14293 | ENSP00000360406 | CACNA1B | 2.194E-05 | -0.291 | - | - |
| 14294 | ENSP00000368237 | ATL2 | 1.289E-05 | -0.291 | - | - |
| 14295 | ENSP00000262138 | CACNG4 | 1.742E-05 | -0.291 | - | - |
| 14296 | ENSP00000276410 | CHRNA6 | 2.049E-05 | -0.291 | - | - |
| 14297 | ENSP00000373998 | CCDC64B | 1.111E-05 | -0.291 | - | - |
| 14298 | ENSP00000330838 | ZNF775 | 1.269E-05 | -0.291 | - | - |
| 14299 | ENSP00000278222 | SAA4 | 2.611E-05 | -0.291 | - | - |
| 14300 | ENSP00000435210 | BCLAF1 | 1.428E-05 | -0.291 | - | - |
| 14301 | ENSP00000396620 | NFYC | 1.173E-05 | -0.292 | - | - |
| 14302 | ENSP00000368767 | ASPH | 1.849E-05 | -0.292 | - | - |
| 14303 | ENSP00000371393 | PGM2 | 1.354E-05 | -0.292 | - | - |
| 14304 | ENSP00000406970 | LAX1 | 1.581E-05 | -0.292 | - | - |
| 14305 | ENSP00000457689 | POTEB | 2.010E-05 | -0.292 | - | - |
| 14306 | ENSP00000355877 | 1-Mar | 2.126E-05 | -0.292 | - | - |
| 14307 | ENSP00000448403 | - | 7.639E-06 | -0.292 | - | - |
| 14308 | ENSP00000361418 | IPO13 | 1.594E-05 | -0.292 | - | - |
| 14309 | ENSP00000355930 | SLC22A1 | 2.123E-05 | -0.292 | - | - |
| 14310 | ENSP00000353847 | WWTR1 | 1.737E-05 | -0.292 | - | - |
| 14311 | ENSP00000372067 | PRMT8 | 2.298E-05 | -0.292 | - | - |
| 14312 | ENSP00000438042 | SAMD8 | 1.373E-05 | -0.292 | - | - |
| 14313 | ENSP00000339916 | LIMK2 | 1.514E-05 | -0.292 | - | - |
| 14314 | ENSP00000418593 | SNX21 | 4.274E-06 | -0.292 | - | - |
| 14315 | ENSP00000185206 | CLIC5 | 2.077E-05 | -0.292 | - | - |
| 14316 | ENSP00000354575 | LSR | 2.022E-05 | -0.292 | - | - |
| 14317 | ENSP00000365773 | PSAT1 | 1.382E-05 | -0.292 | - | - |
| 14318 | ENSP00000011292 | CPA1 | 2.145E-05 | -0.292 | - | - |
| 14319 | ENSP00000333873 | CIB1 | 1.083E-05 | -0.292 | - | - |
| 14320 | ENSP00000355966 | NEK2 | 2.406E-05 | -0.292 | - | - |
| 14321 | ENSP00000332592 | SPAG16 | 7.289E-06 | -0.292 | - | - |
| 14322 | ENSP00000356121 | LEMD1 | 1.629E-05 | -0.292 | - | - |
| 14323 | ENSP00000252071 | ACTR3C | 7.109E-06 | -0.292 | - | - |
| 14324 | ENSP00000160382 | ACTL6B | 6.030E-06 | -0.292 | - | - |
| 14325 | ENSP00000364037 | TEX10 | 1.107E-05 | -0.292 | - | - |
| 14326 | ENSP00000361097 | MMRN2 | 5.043E-06 | -0.292 | - | - |
| 14327 | ENSP00000354028 | CYB561 | 1.490E-05 | -0.292 | - | - |
| 14328 | ENSP00000361486 | PCIF1 | 4.955E-06 | -0.292 | - | - |
| 14329 | ENSP00000364501 | CD300LD | 3.846E-06 | -0.292 | - | - |
| 14330 | ENSP00000467676 | RPS15 | 7.708E-06 | -0.292 | - | - |
| 14331 | ENSP00000363654 | PALM2-AKAP2 | 1.198E-05 | -0.292 | - | - |
| 14332 | ENSP00000434480 | ACER3 | 1.066E-05 | -0.293 | - | - |
| 14333 | ENSP00000282026 | ARL11 | 9.681E-06 | -0.293 | - | - |
| 14334 | ENSP00000356574 | QSOX1 | 1.649E-05 | -0.293 | - | - |
| 14335 | ENSP00000420927 | PDK2 | 2.170E-05 | -0.293 | - | - |
| 14336 | ENSP00000370395 | SGTB | 1.657E-05 | -0.293 | - | - |
| 14337 | ENSP00000388241 | KIF26A | 2.681E-05 | -0.293 | - | - |
| 14338 | ENSP00000330381 | WDR5B | 8.315E-06 | -0.293 | - | - |
| 14339 | ENSP00000357586 | FAM26D | 6.319E-06 | -0.293 | - | - |
| 14340 | ENSP00000371328 | KLRC3 | 2.057E-05 | -0.293 | - | - |
| 14341 | ENSP00000368244 | CHGB | 1.665E-05 | -0.293 | - | - |
| 14342 | ENSP00000454268 | KIAA1024L | 7.059E-07 | -0.293 | - | - |
| 14343 | ENSP00000400882 | CAPNS2 | 2.480E-05 | -0.293 | - | - |
| 14344 | ENSP00000315379 | PRPF3 | 1.040E-05 | -0.293 | - | - |
| 14345 | ENSP00000369038 | EEF1E1 | 2.427E-05 | -0.293 | - | - |
| 14346 | ENSP00000452885 | FAM63B | 1.028E-05 | -0.293 | - | - |
| 14347 | ENSP00000293636 | CELA1 | 1.897E-05 | -0.293 | - | - |
| 14348 | ENSP00000417496 | FAM19A1 | 1.548E-06 | -0.293 | - | - |
| 14349 | ENSP00000346916 | TRIM6-TRIM34 | 1.703E-05 | -0.293 | - | - |
| 14350 | ENSP00000368245 | CXorf21 | 7.634E-06 | -0.294 | - | - |
| 14351 | ENSP00000463517 | EGFL8 | 4.080E-06 | -0.294 | - | - |
| 14352 | ENSP00000352976 | AMZ2 | 7.309E-06 | -0.294 | - | - |
| 14353 | ENSP00000319323 | STOML1 | 7.019E-06 | -0.294 | - | - |
| 14354 | ENSP00000357651 | TUBE1 | 1.896E-05 | -0.294 | - | - |
| 14355 | ENSP00000366582 | FAM196B | 2.993E-06 | -0.294 | - | - |
| 14356 | ENSP00000368391 | TPD52 | 1.136E-05 | -0.294 | - | - |
| 14357 | ENSP00000347978 | ERCC5 | 1.893E-05 | -0.294 | - | - |
| 14358 | ENSP00000346931 | C6orf120 | 2.147E-05 | -0.294 | - | - |
| 14359 | ENSP00000354525 | MRPL24 | 5.563E-06 | -0.294 | - | - |
| 14360 | ENSP00000372689 | STAG1 | 1.365E-05 | -0.294 | - | - |
| 14361 | ENSP00000404439 | 61E3.4 | 1.071E-05 | -0.294 | - | - |
| 14362 | ENSP00000447879 | ZBED6 | 5.580E-06 | -0.294 | - | - |
| 14363 | ENSP00000286808 | CLDN17 | 2.140E-05 | -0.294 | - | - |
| 14364 | ENSP00000355090 | ARHGAP11A | 1.213E-05 | -0.294 | - | - |
| 14365 | ENSP00000353717 | CCDC144A | 1.642E-05 | -0.294 | - | - |
| 14366 | ENSP00000306864 | VASN | 1.417E-05 | -0.294 | - | - |
| 14367 | ENSP00000384690 | MMD2 | 2.244E-05 | -0.294 | - | - |
| 14368 | ENSP00000296805 | GFM2 | 5.813E-06 | -0.294 | - | - |
| 14369 | ENSP00000367172 | GKN1 | 1.355E-05 | -0.294 | - | - |
| 14370 | ENSP00000372202 | N6AMT2 | 5.134E-06 | -0.294 | - | - |
| 14371 | ENSP00000305260 | GNB2 | 1.064E-05 | -0.294 | - | - |
| 14372 | ENSP00000357189 | HDGF | 1.416E-05 | -0.295 | - | - |
| 14373 | ENSP00000443140 | CHURC1-FNTB | 3.868E-06 | -0.295 | - | - |
| 14374 | ENSP00000397552 | ACTL6A | 1.911E-05 | -0.295 | - | - |
| 14375 | ENSP00000387362 | UBE4A | 1.618E-05 | -0.295 | - | - |
| 14376 | ENSP00000358575 | C6orf165 | 7.541E-06 | -0.295 | - | - |
| 14377 | ENSP00000347443 | RASSF5 | 1.470E-05 | -0.295 | - | - |
| 14378 | ENSP00000364632 | BPIFB4 | 4.789E-06 | -0.295 | - | - |
| 14379 | ENSP00000353401 | CUX1 | 2.074E-05 | -0.295 | - | - |
| 14380 | ENSP00000258200 | FBXL8 | 1.246E-05 | -0.295 | - | - |
| 14381 | ENSP00000385036 | TCL1A | 1.206E-05 | -0.295 | - | - |
| 14382 | ENSP00000282507 | UGT3A2 | 8.465E-06 | -0.295 | - | - |
| 14383 | ENSP00000364405 | ZNF367 | 1.861E-05 | -0.295 | - | - |
| 14384 | ENSP00000356256 | TIMM17A | 1.748E-05 | -0.295 | - | - |
| 14385 | ENSP00000361650 | CRIP3 | 1.089E-05 | -0.295 | - | - |
| 14386 | ENSP00000363734 | PFDN6 | 6.762E-06 | -0.295 | - | - |
| 14387 | ENSP00000409318 | ZNF230 | 1.083E-05 | -0.295 | - | - |
| 14388 | ENSP00000371152 | ASAH1 | 1.652E-05 | -0.295 | - | - |
| 14389 | ENSP00000326981 | IMP3 | 4.925E-06 | -0.295 | - | - |
| 14390 | ENSP00000368199 | PGPEP1L | 3.002E-06 | -0.295 | - | - |
| 14391 | ENSP00000379154 | RASGEF1A | 1.276E-05 | -0.295 | - | - |
| 14392 | ENSP00000456566 | RNF151 | 3.444E-06 | -0.295 | - | - |
| 14393 | ENSP00000389770 | FXYD3 | 4.568E-06 | -0.295 | - | - |
| 14394 | ENSP00000286719 | PPEF2 | 9.355E-06 | -0.296 | - | - |
| 14395 | ENSP00000345445 | SAMM50 | 7.042E-06 | -0.296 | - | - |
| 14396 | ENSP00000269586 | ARL5C | 1.094E-05 | -0.296 | - | - |
| 14397 | ENSP00000356104 | PM20D1 | 1.833E-05 | -0.296 | - | - |
| 14398 | ENSP00000363980 | TMEM246 | 3.798E-06 | -0.296 | - | - |
| 14399 | ENSP00000363021 | RPA2 | 1.333E-05 | -0.296 | - | - |
| 14400 | ENSP00000331556 | CYLC1 | 1.743E-05 | -0.296 | - | - |
| 14401 | ENSP00000427820 | C8orf34 | 3.378E-06 | -0.296 | - | - |
| 14402 | ENSP00000346012 | RPL36AL | 1.302E-05 | -0.296 | - | - |
| 14403 | ENSP00000377380 | ARHGAP9 | 2.019E-05 | -0.296 | - | - |
| 14404 | ENSP00000356972 | NDUFS2 | 7.739E-06 | -0.296 | - | - |
| 14405 | ENSP00000298198 | PGM2L1 | 1.454E-05 | -0.296 | - | - |
| 14406 | ENSP00000391402 | CISD3 | 8.801E-06 | -0.296 | - | - |
| 14407 | ENSP00000398163 | TSPY3 | 2.255E-05 | -0.296 | - | - |
| 14408 | ENSP00000393316 | TMX1 | 8.051E-06 | -0.296 | - | - |
| 14409 | ENSP00000445920 | MMAB | 1.672E-05 | -0.296 | - | - |
| 14410 | ENSP00000295240 | BBS5 | 1.595E-05 | -0.296 | - | - |
| 14411 | ENSP00000373873 | UNKL | 1.550E-05 | -0.296 | - | - |
| 14412 | ENSP00000357229 | RHBG | 1.176E-05 | -0.296 | - | - |
| 14413 | ENSP00000379363 | ZNF501 | 2.215E-05 | -0.296 | - | - |
| 14414 | ENSP00000418529 | NSFL1C | 9.453E-06 | -0.297 | - | - |
| 14415 | ENSP00000334197 | KRTAP10-11 | 2.311E-05 | -0.297 | - | - |
| 14416 | ENSP00000379865 | ADHFE1 | 1.976E-05 | -0.297 | - | - |
| 14417 | ENSP00000358812 | PDCD11 | 2.302E-05 | -0.297 | - | - |
| 14418 | ENSP00000370741 | THUMPD1 | 1.672E-05 | -0.297 | - | - |
| 14419 | ENSP00000452037 | DNAL1 | 6.868E-06 | -0.297 | - | - |
| 14420 | ENSP00000419539 | CTAGE4 | 6.055E-06 | -0.297 | - | - |
| 14421 | ENSP00000414920 | SPATA24 | 7.223E-06 | -0.297 | - | - |
| 14422 | ENSP00000358059 | RTN4IP1 | 1.462E-05 | -0.297 | - | - |
| 14423 | ENSP00000340839 | ZC3H12B | 2.231E-05 | -0.297 | - | - |
| 14424 | ENSP00000367595 | TPRG1L | 6.163E-06 | -0.297 | - | - |
| 14425 | ENSP00000416097 | GOLGA2 | 1.750E-05 | -0.297 | - | - |
| 14426 | ENSP00000350540 | - | 1.386E-05 | -0.297 | - | - |
| 14427 | ENSP00000289013 | RHPN1 | 6.737E-06 | -0.297 | - | - |
| 14428 | ENSP00000412189 | PGAM4 | 1.535E-05 | -0.297 | - | - |
| 14429 | ENSP00000334805 | H1FNT | 2.228E-05 | -0.297 | - | - |
| 14430 | ENSP00000359393 | HMGB3 | 1.578E-05 | -0.297 | - | - |
| 14431 | ENSP00000364840 | VWA7 | 7.019E-06 | -0.297 | - | - |
| 14432 | ENSP00000361138 | TMEM164 | 3.584E-06 | -0.297 | - | - |
| 14433 | ENSP00000403067 | FRMD5 | 1.060E-05 | -0.297 | - | - |
| 14434 | ENSP00000381577 | USP34 | 1.248E-05 | -0.297 | - | - |
| 14435 | ENSP00000362285 | SLC29A3 | 1.664E-05 | -0.297 | - | - |
| 14436 | ENSP00000262103 | EFCAB1 | 1.394E-05 | -0.297 | - | - |
| 14437 | ENSP00000370984 | COMMD8 | 1.758E-05 | -0.297 | - | - |
| 14438 | ENSP00000469432 | PKD1L2 | 1.523E-06 | -0.297 | - | - |
| 14439 | ENSP00000412178 | DGKZ | 1.523E-05 | -0.297 | - | - |
| 14440 | ENSP00000334560 | KRTAP6-2 | 9.978E-06 | -0.297 | - | - |
| 14441 | ENSP00000256001 | ACTR3B | 6.976E-06 | -0.297 | - | - |
| 14442 | ENSP00000294829 | FAM71A | 2.306E-05 | -0.297 | - | - |
| 14443 | ENSP00000461413 | ZNF286B | 1.069E-05 | -0.298 | - | - |
| 14444 | ENSP00000368349 | POLA1 | 2.170E-05 | -0.298 | - | - |
| 14445 | ENSP00000343488 | - | 1.860E-05 | -0.298 | - | - |
| 14446 | ENSP00000311684 | SPEG | 1.220E-05 | -0.298 | - | - |
| 14447 | ENSP00000358640 | SLC16A1 | 7.083E-06 | -0.298 | - | - |
| 14448 | ENSP00000355902 | GPATCH2 | 1.496E-05 | -0.298 | - | - |
| 14449 | ENSP00000419027 | PCCB | 2.450E-05 | -0.298 | - | - |
| 14450 | ENSP00000362329 | PPA1 | 2.664E-05 | -0.298 | - | - |
| 14451 | ENSP00000274432 | SPATA9 | 2.809E-05 | -0.298 | - | - |
| 14452 | ENSP00000286809 | CLDN8 | 1.986E-05 | -0.298 | - | - |
| 14453 | ENSP00000259883 | PGBD1 | 2.779E-05 | -0.298 | - | - |
| 14454 | ENSP00000384288 | MYCBP2 | 2.355E-05 | -0.298 | - | - |
| 14455 | ENSP00000381844 | ATL3 | 1.230E-05 | -0.298 | - | - |
| 14456 | ENSP00000358857 | EMD | 3.270E-06 | -0.298 | - | - |
| 14457 | ENSP00000455298 | GOLGA8S | 4.809E-06 | -0.298 | - | - |
| 14458 | ENSP00000274897 | MLIP | 8.906E-06 | -0.298 | - | - |
| 14459 | ENSP00000442339 | SAP25 | 5.448E-06 | -0.298 | - | - |
| 14460 | ENSP00000414922 | USP41 | 2.789E-05 | -0.298 | - | - |
| 14461 | ENSP00000245923 | RTN2 | 1.789E-05 | -0.298 | - | - |
| 14462 | ENSP00000340683 | ZNF823 | 1.608E-05 | -0.298 | - | - |
| 14463 | ENSP00000367631 | ANKEF1 | 1.926E-05 | -0.298 | - | - |
| 14464 | ENSP00000386845 | ZNF385B | 1.419E-05 | -0.298 | - | - |
| 14465 | ENSP00000355511 | CHML | 1.619E-05 | -0.298 | - | - |
| 14466 | ENSP00000373347 | SRGAP3 | 1.628E-05 | -0.298 | - | - |
| 14467 | ENSP00000339280 | CCDC30 | 1.717E-05 | -0.298 | - | - |
| 14468 | ENSP00000334985 | KRTAP19-5 | 6.610E-06 | -0.298 | - | - |
| 14469 | ENSP00000345044 | SBK2 | 1.776E-05 | -0.298 | - | - |
| 14470 | ENSP00000367164 | PRR20A | 6.444E-06 | -0.298 | - | - |
| 14471 | ENSP00000404132 | C8orf59 | 4.032E-06 | -0.299 | - | - |
| 14472 | ENSP00000370047 | BNC2 | 7.846E-06 | -0.299 | - | - |
| 14473 | ENSP00000329638 | ZNF804B | 4.452E-06 | -0.299 | - | - |
| 14474 | ENSP00000425446 | RBM4 | 1.065E-05 | -0.299 | - | - |
| 14475 | ENSP00000356811 | HBS1L | 1.869E-05 | -0.299 | - | - |
| 14476 | ENSP00000353586 | ZNF280B | 1.817E-05 | -0.299 | - | - |
| 14477 | ENSP00000306459 | B4GALT6 | 1.924E-05 | -0.299 | - | - |
| 14478 | ENSP00000327786 | IZUMO1 | 1.713E-05 | -0.299 | - | - |
| 14479 | ENSP00000471635 | ARHGEF18 | 1.602E-06 | -0.299 | - | - |
| 14480 | ENSP00000350386 | CHM | 1.541E-05 | -0.299 | - | - |
| 14481 | ENSP00000328412 | PDE6G | 1.839E-05 | -0.299 | - | - |
| 14482 | ENSP00000221797 | LGALS13 | 2.643E-05 | -0.299 | - | - |
| 14483 | ENSP00000356437 | SASH1 | 2.266E-05 | -0.299 | - | - |
| 14484 | ENSP00000376899 | PTGFRN | 1.473E-05 | -0.299 | - | - |
| 14485 | ENSP00000348283 | WWP2 | 2.580E-05 | -0.299 | - | - |
| 14486 | ENSP00000317743 | HARBI1 | 1.088E-05 | -0.299 | - | - |
| 14487 | ENSP00000361158 | TESK2 | 1.195E-05 | -0.299 | - | - |
| 14488 | ENSP00000392718 | POTEI | 2.960E-05 | -0.299 | - | - |
| 14489 | ENSP00000377566 | ELMO3 | 1.465E-05 | -0.299 | - | - |
| 14490 | ENSP00000375268 | FAM127C | 3.897E-06 | -0.299 | - | - |
| 14491 | ENSP00000374447 | LRCH1 | 2.299E-05 | -0.299 | - | - |
| 14492 | ENSP00000375599 | - | 8.068E-06 | -0.299 | - | - |
| 14493 | ENSP00000376440 | RAD9B | 6.582E-06 | -0.300 | - | - |
| 14494 | ENSP00000257894 | FAM186B | 3.569E-05 | -0.300 | - | - |
| 14495 | ENSP00000395219 | IGFL2 | 1.052E-05 | -0.300 | - | - |
| 14496 | ENSP00000457168 | SZT2 | 1.852E-05 | -0.300 | - | - |
| 14497 | ENSP00000420616 | TFDP2 | 1.716E-05 | -0.300 | - | - |
| 14498 | ENSP00000279168 | GPHA2 | 2.192E-05 | -0.300 | - | - |
| 14499 | ENSP00000306497 | KCNJ4 | 1.687E-05 | -0.300 | - | - |
| 14500 | ENSP00000349078 | FDPS | 1.809E-05 | -0.300 | - | - |
| 14501 | ENSP00000392709 | NDUFS1 | 1.495E-05 | -0.300 | - | - |
| 14502 | ENSP00000393762 | SESN1 | 1.608E-05 | -0.300 | - | - |
| 14503 | ENSP00000370373 | FKBP1B | 2.075E-05 | -0.300 | - | - |
| 14504 | ENSP00000384725 | PRDM6 | 1.378E-05 | -0.300 | - | - |
| 14505 | ENSP00000231420 | AGXT2 | 1.385E-05 | -0.300 | - | - |
| 14506 | ENSP00000384597 | GTF2A1L | 4.409E-06 | -0.300 | - | - |
| 14507 | ENSP00000333833 | CMTM4 | 2.070E-05 | -0.300 | - | - |
| 14508 | ENSP00000380256 | CCNF | 2.211E-05 | -0.300 | - | - |
| 14509 | ENSP00000361662 | GUCA2B | 5.225E-06 | -0.300 | - | - |
| 14510 | ENSP00000369774 | ZNF763 | 2.250E-05 | -0.300 | - | - |
| 14511 | ENSP00000345502 | PDE4D | 1.526E-05 | -0.301 | - | - |
| 14512 | ENSP00000254963 | HSPA12B | 1.213E-05 | -0.301 | - | - |
| 14513 | ENSP00000400500 | THAP5 | 9.461E-06 | -0.301 | - | - |
| 14514 | ENSP00000296096 | TCF23 | 1.668E-05 | -0.301 | - | - |
| 14515 | ENSP00000426691 | GOLGA8K | 5.801E-06 | -0.301 | - | - |
| 14516 | ENSP00000356247 | LGR6 | 1.001E-05 | -0.301 | - | - |
| 14517 | ENSP00000361929 | CERCAM | 1.393E-05 | -0.301 | - | - |
| 14518 | ENSP00000423820 | ZFP62 | 1.747E-05 | -0.301 | - | - |
| 14519 | ENSP00000359049 | HENMT1 | 1.823E-05 | -0.301 | - | - |
| 14520 | ENSP00000335503 | KRTAP20-1 | 2.156E-06 | -0.301 | - | - |
| 14521 | ENSP00000395772 | GNL3 | 1.237E-05 | -0.301 | - | - |
| 14522 | ENSP00000428426 | TCEA1 | 3.197E-05 | -0.301 | - | - |
| 14523 | ENSP00000360515 | SUPT3H | 1.280E-05 | -0.301 | - | - |
| 14524 | ENSP00000353074 | HIST1H2BF | 2.073E-05 | -0.301 | - | - |
| 14525 | ENSP00000243286 | TCEAL3 | 3.707E-05 | -0.301 | - | - |
| 14526 | ENSP00000393556 | FLJ20373 | 5.508E-06 | -0.301 | - | - |
| 14527 | ENSP00000329141 | ZNF37A | 1.274E-05 | -0.301 | - | - |
| 14528 | ENSP00000385879 | KBTBD12 | 6.990E-06 | -0.301 | - | - |
| 14529 | ENSP00000290663 | MED8 | 7.847E-06 | -0.301 | - | - |
| 14530 | ENSP00000319341 | LSM10 | 1.278E-05 | -0.301 | - | - |
| 14531 | ENSP00000351155 | ATL1 | 2.100E-05 | -0.301 | - | - |
| 14532 | ENSP00000359392 | KHDC3L | 1.398E-05 | -0.301 | - | - |
| 14533 | ENSP00000348924 | HIST1H2BE | 1.797E-05 | -0.301 | - | - |
| 14534 | ENSP00000370521 | AIPL1 | 1.675E-05 | -0.302 | - | - |
| 14535 | ENSP00000372160 | DOK6 | 2.280E-05 | -0.302 | - | - |
| 14536 | ENSP00000356465 | RAB32 | 1.800E-05 | -0.302 | - | - |
| 14537 | ENSP00000369045 | SPINK4 | 2.307E-05 | -0.302 | - | - |
| 14538 | ENSP00000462986 | PABPC4L | 8.118E-06 | -0.302 | - | - |
| 14539 | ENSP00000346067 | RPSA | 2.188E-05 | -0.302 | - | - |
| 14540 | ENSP00000366974 | HIST1H4F | 2.058E-05 | -0.302 | - | - |
| 14541 | ENSP00000337146 | ENOX2 | 1.722E-05 | -0.302 | - | - |
| 14542 | ENSP00000333887 | KRTAP22-1 | 2.282E-06 | -0.302 | - | - |
| 14543 | ENSP00000366581 | HIST1H4B | 2.015E-05 | -0.302 | - | - |
| 14544 | ENSP00000381473 | HIGD1C | 9.717E-06 | -0.302 | - | - |
| 14545 | ENSP00000343242 | C7orf29 | 1.631E-05 | -0.302 | - | - |
| 14546 | ENSP00000335306 | DPPA4 | 1.657E-05 | -0.302 | - | - |
| 14547 | ENSP00000244576 | ZNF391 | 2.119E-05 | -0.302 | - | - |
| 14548 | ENSP00000246895 | STATH | 1.481E-05 | -0.302 | - | - |
| 14549 | ENSP00000369424 | RBBP7 | 2.599E-05 | -0.302 | - | - |
| 14550 | ENSP00000349877 | ATP13A1 | 1.788E-05 | -0.302 | - | - |
| 14551 | ENSP00000382883 | MAGEB17 | 6.127E-06 | -0.302 | - | - |
| 14552 | ENSP00000297767 | PRSS37 | 1.068E-05 | -0.302 | - | - |
| 14553 | ENSP00000346635 | ERO1LB | 9.003E-06 | -0.302 | - | - |
| 14554 | ENSP00000282077 | PDK1 | 3.234E-05 | -0.302 | - | - |
| 14555 | ENSP00000382328 | N4BP2L2 | 3.952E-06 | -0.302 | - | - |
| 14556 | ENSP00000369335 | FBXO18 | 1.725E-05 | -0.302 | - | - |
| 14557 | ENSP00000375397 | RNF208 | 2.476E-06 | -0.302 | - | - |
| 14558 | ENSP00000368797 | KBTBD7 | 8.386E-06 | -0.302 | - | - |
| 14559 | ENSP00000375091 | TAS2R19 | 4.049E-06 | -0.302 | - | - |
| 14560 | ENSP00000424048 | TRIM39-RPP21 | 1.431E-05 | -0.302 | - | - |
| 14561 | ENSP00000352995 | ARHGEF18 | 1.745E-05 | -0.302 | - | - |
| 14562 | ENSP00000300105 | CACNG2 | 1.830E-05 | -0.302 | - | - |
| 14563 | ENSP00000306330 | YWHAG | 1.824E-05 | -0.302 | - | - |
| 14564 | ENSP00000390941 | ATCAY | 4.870E-06 | -0.302 | - | - |
| 14565 | ENSP00000393987 | FAM19A2 | 1.401E-05 | -0.302 | - | - |
| 14566 | ENSP00000384832 | FAM104A | 2.542E-06 | -0.303 | - | - |
| 14567 | ENSP00000363308 | WDR31 | 2.003E-05 | -0.303 | - | - |
| 14568 | ENSP00000369129 | DSP | 1.790E-05 | -0.303 | - | - |
| 14569 | ENSP00000349588 | ANK2 | 1.418E-05 | -0.303 | - | - |
| 14570 | ENSP00000370611 | MIER3 | 2.573E-05 | -0.303 | - | - |
| 14571 | ENSP00000276211 | ARHGAP36 | 1.636E-05 | -0.303 | - | - |
| 14572 | ENSP00000435550 | ZC2HC1C | 8.019E-07 | -0.303 | - | - |
| 14573 | ENSP00000427941 | ATP6V0E1 | 1.179E-05 | -0.303 | - | - |
| 14574 | ENSP00000286744 | ADAMTSL3 | 9.708E-06 | -0.303 | - | - |
| 14575 | ENSP00000339867 | CLCN1 | 1.867E-05 | -0.303 | - | - |
| 14576 | ENSP00000385491 | GTF2IRD2 | 8.300E-06 | -0.303 | - | - |
| 14577 | ENSP00000244728 | COL21A1 | 2.336E-05 | -0.303 | - | - |
| 14578 | ENSP00000362122 | TNMD | 1.463E-05 | -0.303 | - | - |
| 14579 | ENSP00000360244 | C1orf87 | 1.080E-05 | -0.303 | - | - |
| 14580 | ENSP00000468046 | TCEB3CL2 | 3.803E-06 | -0.303 | - | - |
| 14581 | ENSP00000383594 | TMEM242 | 2.695E-06 | -0.303 | - | - |
| 14582 | ENSP00000345771 | PFKM | 1.797E-05 | -0.303 | - | - |
| 14583 | ENSP00000354812 | C9orf114 | 1.132E-05 | -0.303 | - | - |
| 14584 | ENSP00000389466 | TGM7 | 5.554E-06 | -0.303 | - | - |
| 14585 | ENSP00000402537 | MTMR10 | 1.387E-05 | -0.303 | - | - |
| 14586 | ENSP00000367034 | HIST1H4C | 1.686E-05 | -0.303 | - | - |
| 14587 | ENSP00000358549 | RARS2 | 1.749E-05 | -0.303 | - | - |
| 14588 | ENSP00000391490 | AGR2 | 1.475E-05 | -0.304 | - | - |
| 14589 | ENSP00000307852 | NUDT18 | 1.309E-05 | -0.304 | - | - |
| 14590 | ENSP00000370557 | MIS12 | 1.490E-05 | -0.304 | - | - |
| 14591 | ENSP00000356541 | SF3B5 | 1.274E-05 | -0.304 | - | - |
| 14592 | ENSP00000377796 | METTL7B | 1.068E-05 | -0.304 | - | - |
| 14593 | ENSP00000363412 | DNAJC25-GNG10 | 4.403E-06 | -0.304 | - | - |
| 14594 | ENSP00000366434 | UIMC1 | 1.253E-05 | -0.304 | - | - |
| 14595 | ENSP00000371577 | MTMR12 | 1.858E-05 | -0.304 | - | - |
| 14596 | ENSP00000342162 | TMEM221 | 5.430E-06 | -0.304 | - | - |
| 14597 | ENSP00000376293 | OSBPL6 | 6.316E-06 | -0.304 | - | - |
| 14598 | ENSP00000157600 | LMCD1 | 2.365E-05 | -0.304 | - | - |
| 14599 | ENSP00000430955 | RNF145 | 7.971E-06 | -0.304 | - | - |
| 14600 | ENSP00000370808 | SLC25A6 | 1.734E-05 | -0.304 | - | - |
| 14601 | ENSP00000382767 | SEL1L3 | 7.846E-06 | -0.304 | - | - |
| 14602 | ENSP00000378505 | CLK3 | 1.664E-05 | -0.304 | - | - |
| 14603 | ENSP00000326759 | LRIG3 | 8.321E-06 | -0.304 | - | - |
| 14604 | ENSP00000284885 | TMPRSS15 | 1.858E-05 | -0.304 | - | - |
| 14605 | ENSP00000183605 | CLDN18 | 2.155E-05 | -0.304 | - | - |
| 14606 | ENSP00000431049 | GSTK1 | 1.364E-05 | -0.304 | - | - |
| 14607 | ENSP00000390783 | SCFD1 | 1.075E-05 | -0.304 | - | - |
| 14608 | ENSP00000260283 | ARHGAP20 | 2.189E-05 | -0.304 | - | - |
| 14609 | ENSP00000357218 | APOA1BP | 7.845E-06 | -0.304 | - | - |
| 14610 | ENSP00000385158 | FAM161A | 1.581E-05 | -0.304 | - | - |
| 14611 | ENSP00000272371 | OTOF | 1.564E-05 | -0.304 | - | - |
| 14612 | ENSP00000328397 | VMO1 | 2.256E-05 | -0.304 | - | - |
| 14613 | ENSP00000351926 | AP2A1 | 1.946E-05 | -0.304 | - | - |
| 14614 | ENSP00000363360 | INIP | 1.362E-05 | -0.304 | - | - |
| 14615 | ENSP00000458075 | BOLA2B | 1.665E-05 | -0.304 | - | - |
| 14616 | ENSP00000469716 | AC090574.1 | 1.525E-06 | -0.304 | - | - |
| 14617 | ENSP00000333374 | CCDC60 | 2.059E-05 | -0.304 | - | - |
| 14618 | ENSP00000362674 | HDAC8 | 1.817E-05 | -0.304 | - | - |
| 14619 | ENSP00000369292 | SAMD9 | 1.151E-05 | -0.304 | - | - |
| 14620 | ENSP00000364207 | TBC1D2 | 1.094E-05 | -0.304 | - | - |
| 14621 | ENSP00000373331 | RPUSD3 | 1.084E-05 | -0.305 | - | - |
| 14622 | ENSP00000335567 | KRTAP19-4 | 2.066E-06 | -0.305 | - | - |
| 14623 | ENSP00000348300 | BTG4 | 8.162E-06 | -0.305 | - | - |
| 14624 | ENSP00000367638 | ARHGEF39 | 9.243E-06 | -0.305 | - | - |
| 14625 | ENSP00000462795 | FOXD1 | 1.459E-05 | -0.305 | - | - |
| 14626 | ENSP00000357701 | S100A3 | 9.861E-06 | -0.305 | - | - |
| 14627 | ENSP00000418668 | MURC | 3.134E-06 | -0.305 | - | - |
| 14628 | ENSP00000361658 | NUP188 | 1.169E-05 | -0.305 | - | - |
| 14629 | ENSP00000428612 | SLC10A5 | 3.829E-06 | -0.305 | - | - |
| 14630 | ENSP00000349732 | C3orf18 | 1.558E-05 | -0.305 | - | - |
| 14631 | ENSP00000337746 | ISYNA1 | 7.394E-06 | -0.305 | - | - |
| 14632 | ENSP00000454407 | GOLGA6L2 | 6.505E-06 | -0.305 | - | - |
| 14633 | ENSP00000308926 | PPM1J | 1.631E-05 | -0.305 | - | - |
| 14634 | ENSP00000357920 | DDO | 1.582E-05 | -0.305 | - | - |
| 14635 | ENSP00000330267 | C15orf32 | 2.539E-05 | -0.305 | - | - |
| 14636 | ENSP00000298386 | RXFP2 | 9.206E-06 | -0.305 | - | - |
| 14637 | ENSP00000346643 | HKDC1 | 1.560E-05 | -0.305 | - | - |
| 14638 | ENSP00000344822 | S100A13 | 1.495E-05 | -0.305 | - | - |
| 14639 | ENSP00000339086 | MKL2 | 1.835E-05 | -0.305 | - | - |
| 14640 | ENSP00000362744 | RPS4X | 1.827E-05 | -0.305 | - | - |
| 14641 | ENSP00000251566 | UGT2A3 | 1.085E-05 | -0.305 | - | - |
| 14642 | ENSP00000356015 | ACAT2 | 1.356E-05 | -0.305 | - | - |
| 14643 | ENSP00000278060 | PAOX | 2.100E-05 | -0.305 | - | - |
| 14644 | ENSP00000379364 | MOB1A | 2.335E-05 | -0.305 | - | - |
| 14645 | ENSP00000367841 | DYNLT3 | 8.628E-06 | -0.305 | - | - |
| 14646 | ENSP00000427772 | WWC1 | 1.905E-05 | -0.306 | - | - |
| 14647 | ENSP00000431254 | IGLL5 | 1.373E-05 | -0.306 | - | - |
| 14648 | ENSP00000256362 | VRTN | 5.574E-06 | -0.306 | - | - |
| 14649 | ENSP00000312706 | CCDC65 | 1.288E-05 | -0.306 | - | - |
| 14650 | ENSP00000405963 | RASGRF1 | 1.479E-05 | -0.306 | - | - |
| 14651 | ENSP00000366452 | SNX15 | 1.338E-05 | -0.306 | - | - |
| 14652 | ENSP00000265993 | TCTN3 | 2.147E-05 | -0.306 | - | - |
| 14653 | ENSP00000328347 | FAM110C | 1.685E-05 | -0.306 | - | - |
| 14654 | ENSP00000302676 | GPR22 | 1.921E-05 | -0.306 | - | - |
| 14655 | ENSP00000263160 | SLC17A6 | 1.746E-05 | -0.306 | - | - |
| 14656 | ENSP00000343493 | C17orf99 | 1.280E-05 | -0.306 | - | - |
| 14657 | ENSP00000263238 | ACTR3 | 7.852E-06 | -0.306 | - | - |
| 14658 | ENSP00000223167 | MYL10 | 1.544E-05 | -0.306 | - | - |
| 14659 | ENSP00000290332 | CLIC6 | 1.877E-05 | -0.306 | - | - |
| 14660 | ENSP00000433681 | NCAPD3 | 9.517E-06 | -0.307 | - | - |
| 14661 | ENSP00000281416 | MFSD6 | 2.003E-05 | -0.307 | - | - |
| 14662 | ENSP00000305302 | SLC6A19 | 1.880E-05 | -0.307 | - | - |
| 14663 | ENSP00000263707 | TFCP2L1 | 2.000E-05 | -0.307 | - | - |
| 14664 | ENSP00000410759 | ATXN7L1 | 1.598E-05 | -0.307 | - | - |
| 14665 | ENSP00000343799 | RNF222 | 1.716E-05 | -0.307 | - | - |
| 14666 | ENSP00000284142 | ASB17 | 1.940E-05 | -0.307 | - | - |
| 14667 | ENSP00000347038 | PLSCR4 | 2.014E-05 | -0.307 | - | - |
| 14668 | ENSP00000415273 | AL353608.1 | 1.858E-06 | -0.307 | - | - |
| 14669 | ENSP00000364813 | LSM2 | 1.109E-05 | -0.307 | - | - |
| 14670 | ENSP00000346598 | RPSAP58 | 1.050E-05 | -0.307 | - | - |
| 14671 | ENSP00000328570 | GLRX5 | 1.378E-05 | -0.307 | - | - |
| 14672 | ENSP00000296582 | TMEM184C | 6.309E-06 | -0.307 | - | - |
| 14673 | ENSP00000361926 | CNPY3 | 1.967E-05 | -0.307 | - | - |
| 14674 | ENSP00000355265 | MT-ATP8 | 4.752E-06 | -0.307 | - | - |
| 14675 | ENSP00000403712 | SENP3 | 1.803E-05 | -0.307 | - | - |
| 14676 | ENSP00000340220 | C7orf26 | 2.053E-05 | -0.308 | - | - |
| 14677 | ENSP00000360376 | FAM209B | 1.036E-05 | -0.308 | - | - |
| 14678 | ENSP00000265800 | EPB49 | 1.743E-05 | -0.308 | - | - |
| 14679 | ENSP00000378289 | SYNPO2L | 2.362E-06 | -0.308 | - | - |
| 14680 | ENSP00000381628 | PCDHA12 | 6.361E-06 | -0.308 | - | - |
| 14681 | ENSP00000362758 | ZBTB26 | 1.164E-05 | -0.308 | - | - |
| 14682 | ENSP00000360191 | APCDD1L | 1.467E-05 | -0.308 | - | - |
| 14683 | ENSP00000378072 | PNPLA1 | 1.826E-05 | -0.308 | - | - |
| 14684 | ENSP00000402020 | C17orf58 | 1.874E-05 | -0.308 | - | - |
| 14685 | ENSP00000362287 | AGO3 | 1.952E-05 | -0.308 | - | - |
| 14686 | ENSP00000455585 | ZNF853 | 9.121E-06 | -0.308 | - | - |
| 14687 | ENSP00000381008 | MUC16 | 9.923E-06 | -0.308 | - | - |
| 14688 | ENSP00000324960 | CABP4 | 9.950E-06 | -0.308 | - | - |
| 14689 | ENSP00000356848 | TADA1 | 5.976E-06 | -0.308 | - | - |
| 14690 | ENSP00000322628 | ARL4D | 1.117E-05 | -0.308 | - | - |
| 14691 | ENSP00000362261 | TRAPPC3 | 6.937E-06 | -0.308 | - | - |
| 14692 | ENSP00000310856 | VN1R4 | 1.815E-05 | -0.308 | - | - |
| 14693 | ENSP00000221455 | CLASRP | 1.837E-05 | -0.308 | - | - |
| 14694 | ENSP00000266560 | RBP5 | 2.171E-05 | -0.308 | - | - |
| 14695 | ENSP00000377401 | SLC35B2 | 1.760E-05 | -0.308 | - | - |
| 14696 | ENSP00000265707 | ADAM18 | 8.721E-06 | -0.308 | - | - |
| 14697 | ENSP00000344242 | LRRC17 | 1.513E-05 | -0.308 | - | - |
| 14698 | ENSP00000367330 | C12orf73 | 7.878E-06 | -0.309 | - | - |
| 14699 | ENSP00000305906 | UTF1 | 2.121E-05 | -0.309 | - | - |
| 14700 | ENSP00000346453 | IPO4 | 1.225E-05 | -0.309 | - | - |
| 14701 | ENSP00000403310 | SNRNP35 | 2.080E-05 | -0.309 | - | - |
| 14702 | ENSP00000451145 | SOX7 | 1.443E-05 | -0.309 | - | - |
| 14703 | ENSP00000363284 | POLE3 | 9.500E-06 | -0.309 | - | - |
| 14704 | ENSP00000359417 | MTMR1 | 1.782E-05 | -0.309 | - | - |
| 14705 | ENSP00000369400 | NHS | 2.074E-05 | -0.309 | - | - |
| 14706 | ENSP00000442521 | BEX2 | 2.914E-06 | -0.309 | - | - |
| 14707 | ENSP00000357883 | PIP5K1A | 1.889E-05 | -0.309 | - | - |
| 14708 | ENSP00000454748 | LRRC9 | 7.128E-06 | -0.309 | - | - |
| 14709 | ENSP00000356115 | MFSD4 | 2.503E-05 | -0.309 | - | - |
| 14710 | ENSP00000368688 | LYRM7 | 1.401E-05 | -0.309 | - | - |
| 14711 | ENSP00000473005 | PIH1 | 2.064E-06 | -0.309 | - | - |
| 14712 | ENSP00000295757 | HDAC11 | 1.032E-05 | -0.309 | - | - |
| 14713 | ENSP00000355880 | 2-Mar | 2.274E-05 | -0.309 | - | - |
| 14714 | ENSP00000348489 | FBRS | 1.550E-05 | -0.309 | - | - |
| 14715 | ENSP00000402505 | CPAMD8 | 8.884E-06 | -0.309 | - | - |
| 14716 | ENSP00000406541 | C11orf21 | 1.301E-06 | -0.310 | - | - |
| 14717 | ENSP00000318770 | AQP11 | 1.979E-05 | -0.310 | - | - |
| 14718 | ENSP00000367086 | ACOT7 | 7.528E-06 | -0.310 | - | - |
| 14719 | ENSP00000428205 | FBXO32 | 2.961E-05 | -0.310 | - | - |
| 14720 | ENSP00000360938 | SARDH | 1.043E-05 | -0.310 | - | - |
| 14721 | ENSP00000409423 | FAM47E | 6.719E-06 | -0.310 | - | - |
| 14722 | ENSP00000374280 | RTF1 | 1.103E-05 | -0.310 | - | - |
| 14723 | ENSP00000352926 | TEAD4 | 2.178E-05 | -0.310 | - | - |
| 14724 | ENSP00000367208 | SUGT1 | 2.176E-05 | -0.310 | - | - |
| 14725 | ENSP00000384900 | ERLIN1 | 1.083E-05 | -0.310 | - | - |
| 14726 | ENSP00000379401 | ODAM | 1.769E-05 | -0.310 | - | - |
| 14727 | ENSP00000354888 | CCDC152 | 1.454E-05 | -0.310 | - | - |
| 14728 | ENSP00000332613 | OAF | 1.700E-05 | -0.310 | - | - |
| 14729 | ENSP00000285116 | SKA1 | 6.096E-06 | -0.310 | - | - |
| 14730 | ENSP00000337722 | ARL6 | 1.219E-05 | -0.310 | - | - |
| 14731 | ENSP00000416040 | KIAA1549 | 5.068E-06 | -0.310 | - | - |
| 14732 | ENSP00000356744 | SCYL3 | 1.422E-05 | -0.310 | - | - |
| 14733 | ENSP00000351206 | TXLNB | 1.295E-05 | -0.310 | - | - |
| 14734 | ENSP00000380661 | CEP250 | 2.034E-05 | -0.310 | - | - |
| 14735 | ENSP00000400513 | FASTKD1 | 7.179E-06 | -0.310 | - | - |
| 14736 | ENSP00000359869 | USP26 | 1.629E-05 | -0.311 | - | - |
| 14737 | ENSP00000265431 | CALB1 | 1.207E-05 | -0.311 | - | - |
| 14738 | ENSP00000438285 | CCDC177 | 1.269E-06 | -0.311 | - | - |
| 14739 | ENSP00000257515 | FAM189A2 | 1.539E-05 | -0.311 | - | - |
| 14740 | ENSP00000351947 | SMARCAD1 | 9.541E-06 | -0.311 | - | - |
| 14741 | ENSP00000408206 | - | 4.694E-06 | -0.311 | - | - |
| 14742 | ENSP00000469958 | ZNF626 | 1.521E-05 | -0.311 | - | - |
| 14743 | ENSP00000364126 | APEX2 | 2.376E-05 | -0.311 | - | - |
| 14744 | ENSP00000371711 | DMRT1 | 1.069E-05 | -0.311 | - | - |
| 14745 | ENSP00000265007 | SOX30 | 2.069E-05 | -0.311 | - | - |
| 14746 | ENSP00000344551 | MORN2 | 2.213E-05 | -0.311 | - | - |
| 14747 | ENSP00000328737 | LYPD4 | 1.838E-05 | -0.311 | - | - |
| 14748 | ENSP00000347549 | CPA5 | 8.649E-06 | -0.311 | - | - |
| 14749 | ENSP00000307288 | MCM7 | 1.193E-05 | -0.311 | - | - |
| 14750 | ENSP00000471505 | CKS1B | 1.056E-05 | -0.311 | - | - |
| 14751 | ENSP00000366977 | PLEKHG5 | 1.827E-05 | -0.311 | - | - |
| 14752 | ENSP00000364145 | PFKFB1 | 7.431E-06 | -0.311 | - | - |
| 14753 | ENSP00000420357 | RWDD1 | 6.312E-06 | -0.311 | - | - |
| 14754 | ENSP00000433919 | CLNS1A | 2.076E-05 | -0.311 | - | - |
| 14755 | ENSP00000440509 | TIFAB | 2.720E-06 | -0.311 | - | - |
| 14756 | ENSP00000364864 | KIF3B | 1.961E-05 | -0.311 | - | - |
| 14757 | ENSP00000356156 | PPP1R15B | 1.324E-05 | -0.311 | - | - |
| 14758 | ENSP00000345964 | SORCS1 | 1.263E-05 | -0.311 | - | - |
| 14759 | ENSP00000395328 | TMEM100 | 4.650E-06 | -0.311 | - | - |
| 14760 | ENSP00000376506 | PDCD10 | 1.548E-05 | -0.311 | - | - |
| 14761 | ENSP00000374484 | TMEM87A | 2.120E-05 | -0.311 | - | - |
| 14762 | ENSP00000280326 | CCT5 | 1.432E-05 | -0.312 | - | - |
| 14763 | ENSP00000301391 | CYB5D2 | 1.731E-05 | -0.312 | - | - |
| 14764 | ENSP00000362688 | EIF3I | 1.273E-05 | -0.312 | - | - |
| 14765 | ENSP00000376403 | GPRC5C | 1.762E-05 | -0.312 | - | - |
| 14766 | ENSP00000349259 | SPTBN1 | 2.332E-05 | -0.312 | - | - |
| 14767 | ENSP00000400895 | OTOGL | 1.468E-05 | -0.312 | - | - |
| 14768 | ENSP00000325638 | PRM3 | 8.699E-06 | -0.312 | - | - |
| 14769 | ENSP00000324175 | MAMSTR | 1.151E-05 | -0.312 | - | - |
| 14770 | ENSP00000354957 | SMC5 | 1.452E-05 | -0.312 | - | - |
| 14771 | ENSP00000330442 | PLB1 | 1.949E-05 | -0.312 | - | - |
| 14772 | ENSP00000349490 | MFNG | 1.936E-05 | -0.312 | - | - |
| 14773 | ENSP00000247992 | PLA2G2C | 2.696E-05 | -0.312 | - | - |
| 14774 | ENSP00000354762 | COA3 | 5.754E-06 | -0.312 | - | - |
| 14775 | ENSP00000370826 | HR | 1.322E-05 | -0.312 | - | - |
| 14776 | ENSP00000396304 | CLDN25 | 5.811E-06 | -0.312 | - | - |
| 14777 | ENSP00000264382 | SI | 1.617E-05 | -0.312 | - | - |
| 14778 | ENSP00000364464 | C9orf3 | 1.879E-05 | -0.312 | - | - |
| 14779 | ENSP00000242505 | FAM149B1 | 1.174E-05 | -0.312 | - | - |
| 14780 | ENSP00000402855 | TREML1 | 1.259E-05 | -0.312 | - | - |
| 14781 | ENSP00000413234 | AP2A2 | 2.321E-05 | -0.312 | - | - |
| 14782 | ENSP00000316224 | ALS2CR8 | 1.413E-05 | -0.312 | - | - |
| 14783 | ENSP00000364404 | DCAF17 | 7.263E-06 | -0.312 | - | - |
| 14784 | ENSP00000351520 | ENTPD4 | 1.656E-05 | -0.313 | - | - |
| 14785 | ENSP00000345917 | LYAR | 8.591E-06 | -0.313 | - | - |
| 14786 | ENSP00000340271 | TAF3 | 1.312E-05 | -0.313 | - | - |
| 14787 | ENSP00000472066 | PRED60 | 1.716E-06 | -0.313 | - | - |
| 14788 | ENSP00000354045 | RRBP1 | 2.219E-05 | -0.313 | - | - |
| 14789 | ENSP00000379228 | HECW1 | 2.479E-05 | -0.313 | - | - |
| 14790 | ENSP00000298545 | AK8 | 1.579E-05 | -0.313 | - | - |
| 14791 | ENSP00000398089 | ZNF821 | 1.827E-06 | -0.313 | - | - |
| 14792 | ENSP00000295588 | POGLUT1 | 1.673E-05 | -0.313 | - | - |
| 14793 | ENSP00000338862 | BRIX1 | 8.345E-06 | -0.313 | - | - |
| 14794 | ENSP00000364295 | PQLC2 | 5.459E-06 | -0.313 | - | - |
| 14795 | ENSP00000304697 | UBB | 2.144E-05 | -0.313 | - | - |
| 14796 | ENSP00000313506 | ARHGAP28 | 2.104E-05 | -0.313 | - | - |
| 14797 | ENSP00000353210 | ARL9 | 1.157E-05 | -0.313 | - | - |
| 14798 | ENSP00000331514 | ACTG1 | 1.979E-05 | -0.313 | - | - |
| 14799 | ENSP00000472867 | ZIK1 | 8.564E-06 | -0.313 | - | - |
| 14800 | ENSP00000258229 | PCNXL2 | 3.176E-05 | -0.313 | - | - |
| 14801 | ENSP00000005180 | CCL26 | 2.229E-05 | -0.313 | - | - |
| 14802 | ENSP00000377778 | MIEN1 | 1.866E-05 | -0.313 | - | - |
| 14803 | ENSP00000411532 | TOP2A | 3.798E-05 | -0.313 | - | - |
| 14804 | ENSP00000229554 | RSPH4A | 1.712E-05 | -0.313 | - | - |
| 14805 | ENSP00000332488 | SPPL2C | 1.737E-05 | -0.313 | - | - |
| 14806 | ENSP00000323421 | SMC1A | 1.364E-05 | -0.313 | - | - |
| 14807 | ENSP00000360112 | RAVER2 | 1.515E-05 | -0.313 | - | - |
| 14808 | ENSP00000370658 | CCDC91 | 9.358E-06 | -0.313 | - | - |
| 14809 | ENSP00000454670 | TMBIM4 | 1.312E-05 | -0.314 | - | - |
| 14810 | ENSP00000317289 | FAM220A | 9.562E-06 | -0.314 | - | - |
| 14811 | ENSP00000417303 | KLF8 | 1.036E-05 | -0.314 | - | - |
| 14812 | ENSP00000381631 | C2orf73 | 6.243E-06 | -0.314 | - | - |
| 14813 | ENSP00000263331 | POLR1B | 6.317E-06 | -0.314 | - | - |
| 14814 | ENSP00000378720 | KTN1 | 1.222E-05 | -0.314 | - | - |
| 14815 | ENSP00000348815 | HYLS1 | 9.988E-06 | -0.314 | - | - |
| 14816 | ENSP00000440896 | PLD5 | 5.332E-06 | -0.314 | - | - |
| 14817 | ENSP00000365585 | FAM182B | 5.762E-06 | -0.314 | - | - |
| 14818 | ENSP00000343995 | TEKT3 | 2.069E-05 | -0.314 | - | - |
| 14819 | ENSP00000347271 | RPS10 | 9.286E-06 | -0.314 | - | - |
| 14820 | ENSP00000332528 | OTOP2 | 1.510E-05 | -0.314 | - | - |
| 14821 | ENSP00000373244 | CSPG5 | 1.201E-05 | -0.314 | - | - |
| 14822 | ENSP00000301831 | ULK4 | 1.635E-05 | -0.314 | - | - |
| 14823 | ENSP00000345412 | CPSF7 | 9.440E-06 | -0.314 | - | - |
| 14824 | ENSP00000421169 | BEND4 | 5.360E-06 | -0.314 | - | - |
| 14825 | ENSP00000386292 | TMEM194B | 1.408E-05 | -0.314 | - | - |
| 14826 | ENSP00000347117 | SEMA4A | 1.971E-05 | -0.314 | - | - |
| 14827 | ENSP00000177648 | ALPK1 | 1.310E-05 | -0.314 | - | - |
| 14828 | ENSP00000387230 | UPP2 | 1.318E-05 | -0.314 | - | - |
| 14829 | ENSP00000350024 | SLC29A2 | 1.418E-05 | -0.314 | - | - |
| 14830 | ENSP00000321108 | SPOCK2 | 1.629E-05 | -0.314 | - | - |
| 14831 | ENSP00000358400 | MDN1 | 6.496E-06 | -0.315 | - | - |
| 14832 | ENSP00000378400 | LIMA1 | 9.381E-06 | -0.315 | - | - |
| 14833 | ENSP00000362353 | GLP1R | 2.004E-05 | -0.315 | - | - |
| 14834 | ENSP00000353622 | SIN3A | 2.205E-05 | -0.315 | - | - |
| 14835 | ENSP00000367346 | NDUFAF5 | 5.521E-06 | -0.315 | - | - |
| 14836 | ENSP00000301246 | C19orf33 | 1.048E-05 | -0.315 | - | - |
| 14837 | ENSP00000369372 | BEND2 | 2.065E-06 | -0.315 | - | - |
| 14838 | ENSP00000363533 | MDH1B | 1.921E-05 | -0.315 | - | - |
| 14839 | ENSP00000376384 | GPR123 | 1.130E-05 | -0.315 | - | - |
| 14840 | ENSP00000366396 | XRN2 | 2.659E-05 | -0.315 | - | - |
| 14841 | ENSP00000355045 | FAM179B | 7.178E-06 | -0.315 | - | - |
| 14842 | ENSP00000364403 | UBR4 | 6.984E-06 | -0.315 | - | - |
| 14843 | ENSP00000348657 | NCAPG2 | 1.409E-05 | -0.315 | - | - |
| 14844 | ENSP00000357907 | CDK19 | 1.661E-05 | -0.315 | - | - |
| 14845 | ENSP00000368152 | ALKBH6 | 2.543E-06 | -0.315 | - | - |
| 14846 | ENSP00000348752 | ORAI2 | 1.781E-05 | -0.315 | - | - |
| 14847 | ENSP00000419740 | NSUN4 | 9.084E-06 | -0.315 | - | - |
| 14848 | ENSP00000274606 | NHP2 | 5.474E-06 | -0.315 | - | - |
| 14849 | ENSP00000350876 | DNM3 | 1.672E-05 | -0.315 | - | - |
| 14850 | ENSP00000411645 | TMPRSS7 | 7.718E-06 | -0.315 | - | - |
| 14851 | ENSP00000352498 | - | 1.876E-05 | -0.315 | - | - |
| 14852 | ENSP00000331470 | DBX2 | 1.780E-05 | -0.315 | - | - |
| 14853 | ENSP00000239450 | PCDHB12 | 1.697E-05 | -0.315 | - | - |
| 14854 | ENSP00000365782 | CEP78 | 2.106E-05 | -0.315 | - | - |
| 14855 | ENSP00000332369 | ALAS2 | 1.885E-05 | -0.315 | - | - |
| 14856 | ENSP00000399627 | LCN6 | 5.485E-08 | -0.315 | - | - |
| 14857 | ENSP00000405577 | CLMP | 1.126E-05 | -0.315 | - | - |
| 14858 | ENSP00000364635 | PADI2 | 1.324E-05 | -0.316 | - | - |
| 14859 | ENSP00000301057 | TP53I13 | 9.234E-06 | -0.316 | - | - |
| 14860 | ENSP00000312770 | C2orf69 | 1.880E-05 | -0.316 | - | - |
| 14861 | ENSP00000306754 | NEUROD2 | 2.012E-05 | -0.316 | - | - |
| 14862 | ENSP00000266712 | TMTC3 | 6.349E-06 | -0.316 | - | - |
| 14863 | ENSP00000348911 | AADACL2 | 1.225E-05 | -0.316 | - | - |
| 14864 | ENSP00000380597 | EEF1E1-MUTED | 2.241E-05 | -0.316 | - | - |
| 14865 | ENSP00000365172 | KDELC1 | 1.710E-05 | -0.316 | - | - |
| 14866 | ENSP00000359022 | ELOVL3 | 1.005E-05 | -0.316 | - | - |
| 14867 | ENSP00000373979 | TRAPPC9 | 1.747E-05 | -0.316 | - | - |
| 14868 | ENSP00000416662 | ZNF713 | 1.054E-05 | -0.316 | - | - |
| 14869 | ENSP00000228515 | CSRNP2 | 1.638E-05 | -0.316 | - | - |
| 14870 | ENSP00000429276 | SNTG1 | 1.368E-05 | -0.316 | - | - |
| 14871 | ENSP00000364188 | DDOST | 1.612E-05 | -0.316 | - | - |
| 14872 | ENSP00000360645 | MAN1B1 | 1.459E-05 | -0.316 | - | - |
| 14873 | ENSP00000364363 | SMIM5 | 2.889E-06 | -0.316 | - | - |
| 14874 | ENSP00000409932 | TCEB3CL | 5.630E-06 | -0.316 | - | - |
| 14875 | ENSP00000347195 | TMEM110 | 5.990E-06 | -0.316 | - | - |
| 14876 | ENSP00000364687 | EHMT2 | 2.845E-05 | -0.316 | - | - |
| 14877 | ENSP00000446479 | FICD | 1.636E-05 | -0.316 | - | - |
| 14878 | ENSP00000299415 | AC007431.1 | 4.172E-06 | -0.316 | - | - |
| 14879 | ENSP00000355162 | TIGD4 | 1.709E-05 | -0.316 | - | - |
| 14880 | ENSP00000341044 | ACACB | 1.337E-05 | -0.317 | - | - |
| 14881 | ENSP00000408910 | DCTN2 | 2.584E-05 | -0.317 | - | - |
| 14882 | ENSP00000458130 | GOLGA8G | 5.216E-06 | -0.317 | - | - |
| 14883 | ENSP00000369907 | FAM154A | 3.368E-06 | -0.317 | - | - |
| 14884 | ENSP00000363118 | ASAH2B | 1.185E-05 | -0.317 | - | - |
| 14885 | ENSP00000448219 | TAS2R38 | 4.638E-06 | -0.317 | - | - |
| 14886 | ENSP00000462754 | SLC25A52 | 1.266E-06 | -0.317 | - | - |
| 14887 | ENSP00000328674 | CLDN6 | 1.456E-05 | -0.317 | - | - |
| 14888 | ENSP00000367432 | AJAP1 | 1.563E-05 | -0.317 | - | - |
| 14889 | ENSP00000239614 | MSANTD2 | 3.076E-05 | -0.317 | - | - |
| 14890 | ENSP00000361642 | PPCS | 1.305E-05 | -0.317 | - | - |
| 14891 | ENSP00000341128 | CLUL1 | 1.064E-05 | -0.317 | - | - |
| 14892 | ENSP00000436318 | CCS | 1.088E-05 | -0.317 | - | - |
| 14893 | ENSP00000360822 | KCNT1 | 1.101E-05 | -0.317 | - | - |
| 14894 | ENSP00000366995 | FRMPD1 | 3.425E-06 | -0.317 | - | - |
| 14895 | ENSP00000397900 | MRO | 1.169E-05 | -0.317 | - | - |
| 14896 | ENSP00000361935 | HPCAL4 | 2.314E-05 | -0.317 | - | - |
| 14897 | ENSP00000378508 | DNAJC22 | 1.035E-05 | -0.317 | - | - |
| 14898 | ENSP00000332565 | SULT4A1 | 1.906E-05 | -0.317 | - | - |
| 14899 | ENSP00000321927 | FBXL13 | 7.393E-06 | -0.317 | - | - |
| 14900 | ENSP00000438144 | MDH1 | 2.530E-05 | -0.317 | - | - |
| 14901 | ENSP00000346037 | RPLP1 | 9.556E-06 | -0.317 | - | - |
| 14902 | ENSP00000386770 | DNAH10 | 1.867E-05 | -0.317 | - | - |
| 14903 | ENSP00000358242 | PNISR | 1.900E-05 | -0.317 | - | - |
| 14904 | ENSP00000364721 | MAPRE1 | 1.840E-05 | -0.317 | - | - |
| 14905 | ENSP00000308575 | FAM46D | 7.843E-06 | -0.317 | - | - |
| 14906 | ENSP00000311390 | C16orf91 | 1.227E-05 | -0.317 | - | - |
| 14907 | ENSP00000368464 | KIF24 | 7.597E-06 | -0.317 | - | - |
| 14908 | ENSP00000354945 | - | 1.904E-05 | -0.317 | - | - |
| 14909 | ENSP00000406046 | POLD1 | 2.618E-05 | -0.317 | - | - |
| 14910 | ENSP00000291560 | HSF2BP | 2.137E-05 | -0.317 | - | - |
| 14911 | ENSP00000258776 | C7orf69 | 1.996E-05 | -0.317 | - | - |
| 14912 | ENSP00000357475 | ATP8B2 | 1.823E-05 | -0.317 | - | - |
| 14913 | ENSP00000444823 | HIST1H3F | 2.250E-05 | -0.318 | - | - |
| 14914 | ENSP00000338613 | CRYGN | 1.823E-05 | -0.318 | - | - |
| 14915 | ENSP00000363128 | 1-Dec | 8.187E-06 | -0.318 | - | - |
| 14916 | ENSP00000330148 | GRID1 | 1.845E-05 | -0.318 | - | - |
| 14917 | ENSP00000400815 | C16orf62 | 3.662E-06 | -0.318 | - | - |
| 14918 | ENSP00000366050 | C9orf40 | 9.374E-06 | -0.318 | - | - |
| 14919 | ENSP00000391266 | RP1-241P17.4 | 1.622E-05 | -0.318 | - | - |
| 14920 | ENSP00000305533 | THAP9 | 1.709E-05 | -0.318 | - | - |
| 14921 | ENSP00000419471 | RAD51B | 3.665E-05 | -0.318 | - | - |
| 14922 | ENSP00000345464 | NHLRC1 | 1.918E-05 | -0.318 | - | - |
| 14923 | ENSP00000409667 | SYNJ1 | 3.217E-05 | -0.318 | - | - |
| 14924 | ENSP00000334437 | NUDT17 | 1.110E-05 | -0.318 | - | - |
| 14925 | ENSP00000379108 | MTHFD2L | 2.230E-05 | -0.318 | - | - |
| 14926 | ENSP00000363559 | EIF6 | 1.736E-05 | -0.318 | - | - |
| 14927 | ENSP00000370414 | ATP5EP2 | 2.604E-06 | -0.318 | - | - |
| 14928 | ENSP00000358742 | SFR1 | 6.393E-06 | -0.318 | - | - |
| 14929 | ENSP00000377769 | ARL13B | 2.117E-05 | -0.318 | - | - |
| 14930 | ENSP00000397773 | TMEM50B | 8.120E-06 | -0.318 | - | - |
| 14931 | ENSP00000352980 | HIST1H4A | 2.060E-05 | -0.318 | - | - |
| 14932 | ENSP00000380824 | 10-Sep | 1.737E-05 | -0.318 | - | - |
| 14933 | ENSP00000399903 | NBEAL1 | 8.103E-06 | -0.318 | - | - |
| 14934 | ENSP00000336861 | IQCH | 3.402E-06 | -0.318 | - | - |
| 14935 | ENSP00000384917 | SVEP1 | 1.852E-05 | -0.318 | - | - |
| 14936 | ENSP00000392466 | LDB1 | 1.733E-05 | -0.319 | - | - |
| 14937 | ENSP00000242109 | KIAA0087 | 8.454E-06 | -0.319 | - | - |
| 14938 | ENSP00000451369 | RP11-371E8.4 | 6.467E-06 | -0.319 | - | - |
| 14939 | ENSP00000330188 | RSRC2 | 1.489E-05 | -0.319 | - | - |
| 14940 | ENSP00000433378 | PCDHA6 | 6.824E-06 | -0.319 | - | - |
| 14941 | ENSP00000338293 | ROMO1 | 7.123E-06 | -0.319 | - | - |
| 14942 | ENSP00000378132 | IRX5 | 2.150E-05 | -0.319 | - | - |
| 14943 | ENSP00000315017 | MRPL1 | 8.476E-06 | -0.319 | - | - |
| 14944 | ENSP00000422375 | NUDT16 | 9.705E-06 | -0.319 | - | - |
| 14945 | ENSP00000310515 | PRM1 | 1.003E-05 | -0.319 | - | - |
| 14946 | ENSP00000366221 | CASZ1 | 1.403E-05 | -0.319 | - | - |
| 14947 | ENSP00000360918 | CH25H | 2.032E-05 | -0.319 | - | - |
| 14948 | ENSP00000354720 | SMC3 | 1.928E-05 | -0.319 | - | - |
| 14949 | ENSP00000328939 | UTY | 1.442E-05 | -0.319 | - | - |
| 14950 | ENSP00000469582 | ZNF729 | 9.503E-06 | -0.319 | - | - |
| 14951 | ENSP00000355014 | PJA1 | 1.956E-05 | -0.319 | - | - |
| 14952 | ENSP00000302441 | NANP | 2.036E-05 | -0.319 | - | - |
| 14953 | ENSP00000308057 | SNCB | 1.681E-05 | -0.319 | - | - |
| 14954 | ENSP00000373752 | RNF180 | 7.482E-06 | -0.319 | - | - |
| 14955 | ENSP00000361465 | POLR1C | 2.168E-05 | -0.319 | - | - |
| 14956 | ENSP00000384801 | PCNXL4 | 8.220E-06 | -0.319 | - | - |
| 14957 | ENSP00000371535 | SEPSECS | 1.132E-05 | -0.319 | - | - |
| 14958 | ENSP00000371110 | GCAT | 1.880E-05 | -0.319 | - | - |
| 14959 | ENSP00000295890 | COX18 | 4.074E-06 | -0.320 | - | - |
| 14960 | ENSP00000442159 | MTRNR2L10 | 3.308E-06 | -0.320 | - | - |
| 14961 | ENSP00000312735 | POLR2B | 9.070E-06 | -0.320 | - | - |
| 14962 | ENSP00000306335 | RFX2 | 1.363E-05 | -0.320 | - | - |
| 14963 | ENSP00000469305 | AC093323.1 | 1.933E-06 | -0.320 | - | - |
| 14964 | ENSP00000365569 | FLOT1 | 9.562E-06 | -0.320 | - | - |
| 14965 | ENSP00000382770 | KCNU1 | 1.649E-05 | -0.320 | - | - |
| 14966 | ENSP00000216068 | DNAL4 | 1.341E-05 | -0.320 | - | - |
| 14967 | ENSP00000382670 | GRXCR1 | 7.949E-06 | -0.320 | - | - |
| 14968 | ENSP00000359211 | DPYD | 3.377E-05 | -0.320 | - | - |
| 14969 | ENSP00000370517 | PFKP | 2.114E-05 | -0.320 | - | - |
| 14970 | ENSP00000323847 | ARL14 | 1.210E-05 | -0.320 | - | - |
| 14971 | ENSP00000451542 | SOHLH2 | 3.660E-06 | -0.320 | - | - |
| 14972 | ENSP00000355587 | NTPCR | 1.784E-05 | -0.320 | - | - |
| 14973 | ENSP00000375108 | KRTAP19-1 | 9.649E-07 | -0.320 | - | - |
| 14974 | ENSP00000385903 | NAA60 | 1.194E-05 | -0.320 | - | - |
| 14975 | ENSP00000431603 | POU2F2 | 1.787E-05 | -0.320 | - | - |
| 14976 | ENSP00000366513 | CLSTN1 | 1.582E-05 | -0.320 | - | - |
| 14977 | ENSP00000365063 | ABHD13 | 4.116E-06 | -0.320 | - | - |
| 14978 | ENSP00000357796 | TCHHL1 | 5.054E-06 | -0.321 | - | - |
| 14979 | ENSP00000402414 | TRIM48 | 5.951E-06 | -0.321 | - | - |
| 14980 | ENSP00000260386 | ITPKA | 1.405E-05 | -0.321 | - | - |
| 14981 | ENSP00000456927 | GOLGA8M | 5.237E-06 | -0.321 | - | - |
| 14982 | ENSP00000367797 | SKI | 1.344E-05 | -0.321 | - | - |
| 14983 | ENSP00000321386 | FBXO39 | 1.297E-05 | -0.321 | - | - |
| 14984 | ENSP00000344095 | ZNF683 | 1.419E-05 | -0.321 | - | - |
| 14985 | ENSP00000393453 | SMYD1 | 1.085E-05 | -0.321 | - | - |
| 14986 | ENSP00000297737 | ZMAT4 | 1.027E-05 | -0.321 | - | - |
| 14987 | ENSP00000367229 | CEP68 | 6.685E-06 | -0.321 | - | - |
| 14988 | ENSP00000368646 | PRDX4 | 2.214E-05 | -0.321 | - | - |
| 14989 | ENSP00000281772 | KANSL1L | 1.723E-05 | -0.321 | - | - |
| 14990 | ENSP00000334910 | PDE2A | 8.411E-06 | -0.321 | - | - |
| 14991 | ENSP00000321976 | NKPD1 | 1.822E-05 | -0.321 | - | - |
| 14992 | ENSP00000380795 | - | 1.828E-05 | -0.321 | - | - |
| 14993 | ENSP00000451476 | AKAP2 | 1.262E-05 | -0.321 | - | - |
| 14994 | ENSP00000450560 | MTHFD1 | 3.343E-05 | -0.322 | - | - |
| 14995 | ENSP00000410447 | IKZF2 | 1.438E-05 | -0.322 | - | - |
| 14996 | ENSP00000390153 | C6orf203 | 5.692E-06 | -0.322 | - | - |
| 14997 | ENSP00000404179 | DOCK4 | 1.576E-05 | -0.322 | - | - |
| 14998 | ENSP00000375076 | KRTAP4-4 | 1.689E-06 | -0.322 | - | - |
| 14999 | ENSP00000458062 | ZNF286A | 1.009E-05 | -0.322 | - | - |
| 15000 | ENSP00000407978 | NKX1-1 | 1.021E-05 | -0.322 | - | - |
| 15001 | ENSP00000406084 | WDR20 | 1.265E-05 | -0.322 | - | - |
| 15002 | ENSP00000405455 | TKT | 2.326E-05 | -0.322 | - | - |
| 15003 | ENSP00000362153 | GNL2 | 7.756E-06 | -0.322 | - | - |
| 15004 | ENSP00000216029 | CBY1 | 1.988E-05 | -0.322 | - | - |
| 15005 | ENSP00000221204 | - | 2.604E-05 | -0.322 | - | - |
| 15006 | ENSP00000460823 | TOM1L1 | 2.024E-05 | -0.322 | - | - |
| 15007 | ENSP00000432614 | NDUFC2-KCTD14 | 5.473E-06 | -0.322 | - | - |
| 15008 | ENSP00000299432 | MSS51 | 9.806E-06 | -0.322 | - | - |
| 15009 | ENSP00000421172 | AWAT2 | 7.214E-06 | -0.322 | - | - |
| 15010 | ENSP00000262430 | MLYCD | 1.769E-05 | -0.322 | - | - |
| 15011 | ENSP00000261892 | SLC24A1 | 1.078E-05 | -0.322 | - | - |
| 15012 | ENSP00000295569 | FAM19A4 | 2.217E-05 | -0.323 | - | - |
| 15013 | ENSP00000322087 | NHLH2 | 1.320E-05 | -0.323 | - | - |
| 15014 | ENSP00000381488 | KRTAP9-1 | 2.000E-06 | -0.323 | - | - |
| 15015 | ENSP00000389275 | PKD1P1 | 1.256E-05 | -0.323 | - | - |
| 15016 | ENSP00000368264 | MAGEB1 | 9.686E-06 | -0.323 | - | - |
| 15017 | ENSP00000326693 | SLC25A42 | 1.914E-05 | -0.323 | - | - |
| 15018 | ENSP00000386439 | SYNC | 6.404E-06 | -0.323 | - | - |
| 15019 | ENSP00000412436 | PRH1 | 9.674E-07 | -0.323 | - | - |
| 15020 | ENSP00000380544 | OGFOD2 | 1.177E-05 | -0.323 | - | - |
| 15021 | ENSP00000387547 | AC016745.1 | 2.185E-06 | -0.323 | - | - |
| 15022 | ENSP00000352173 | PLAC1 | 1.107E-05 | -0.323 | - | - |
| 15023 | ENSP00000373610 | OTOA | 1.394E-05 | -0.323 | - | - |
| 15024 | ENSP00000334834 | KRTAP13-4 | 1.897E-06 | -0.323 | - | - |
| 15025 | ENSP00000360782 | PMPCA | 1.783E-05 | -0.323 | - | - |
| 15026 | ENSP00000454746 | RP11-1035H13.3 | 8.464E-06 | -0.323 | - | - |
| 15027 | ENSP00000472922 | DKFZP547L112 | 7.759E-07 | -0.323 | - | - |
| 15028 | ENSP00000382476 | MROH2B | 6.947E-06 | -0.323 | - | - |
| 15029 | ENSP00000371715 | ZG16B | 6.191E-06 | -0.323 | - | - |
| 15030 | ENSP00000358695 | CYB5R4 | 1.479E-05 | -0.323 | - | - |
| 15031 | ENSP00000399563 | GPR89C | 8.610E-06 | -0.323 | - | - |
| 15032 | ENSP00000324553 | GPR156 | 1.824E-05 | -0.323 | - | - |
| 15033 | ENSP00000311165 | AQP1 | 1.784E-05 | -0.323 | - | - |
| 15034 | ENSP00000369374 | SKOR1 | 1.580E-05 | -0.323 | - | - |
| 15035 | ENSP00000424176 | EPPIN-WFDC6 | 6.721E-06 | -0.323 | - | - |
| 15036 | ENSP00000469533 | CTC-490E21.12 | 8.888E-06 | -0.323 | - | - |
| 15037 | ENSP00000359729 | SLC9A6 | 1.375E-05 | -0.324 | - | - |
| 15038 | ENSP00000374268 | GXYLT2 | 6.289E-06 | -0.324 | - | - |
| 15039 | ENSP00000349154 | KIAA1841 | 6.676E-06 | -0.324 | - | - |
| 15040 | ENSP00000453153 | BLID | 6.105E-06 | -0.324 | - | - |
| 15041 | ENSP00000371514 | KCNV2 | 1.914E-05 | -0.324 | - | - |
| 15042 | ENSP00000366586 | SLC39A12 | 1.627E-05 | -0.324 | - | - |
| 15043 | ENSP00000333142 | PLA2G6 | 1.735E-05 | -0.324 | - | - |
| 15044 | ENSP00000415822 | ATXN1L | 1.629E-05 | -0.324 | - | - |
| 15045 | ENSP00000359600 | ODF2L | 7.170E-06 | -0.324 | - | - |
| 15046 | ENSP00000454322 | GOLGA8F | 5.240E-06 | -0.324 | - | - |
| 15047 | ENSP00000361298 | KIF2C | 1.715E-05 | -0.324 | - | - |
| 15048 | ENSP00000359680 | HOGA1 | 3.693E-06 | -0.324 | - | - |
| 15049 | ENSP00000352268 | AADACL3 | 4.492E-06 | -0.324 | - | - |
| 15050 | ENSP00000328422 | PP13439 | 4.397E-06 | -0.324 | - | - |
| 15051 | ENSP00000373340 | BRPF1 | 1.616E-05 | -0.324 | - | - |
| 15052 | ENSP00000447488 | LIN7A | 2.014E-05 | -0.324 | - | - |
| 15053 | ENSP00000379047 | CCDC51 | 5.638E-06 | -0.324 | - | - |
| 15054 | ENSP00000370129 | AKR1C2 | 1.653E-05 | -0.324 | - | - |
| 15055 | ENSP00000363616 | EDEM2 | 9.507E-06 | -0.324 | - | - |
| 15056 | ENSP00000301532 | OR5I1 | 1.845E-05 | -0.324 | - | - |
| 15057 | ENSP00000348751 | BTN3A2 | 2.582E-05 | -0.324 | - | - |
| 15058 | ENSP00000347744 | FAM49A | 9.533E-06 | -0.324 | - | - |
| 15059 | ENSP00000297109 | SAP30L | 1.823E-05 | -0.324 | - | - |
| 15060 | ENSP00000434849 | LRRC24 | 6.846E-06 | -0.325 | - | - |
| 15061 | ENSP00000354499 | MT-CO1 | 2.230E-05 | -0.325 | - | - |
| 15062 | ENSP00000327599 | CLEC4G | 1.597E-05 | -0.325 | - | - |
| 15063 | ENSP00000420610 | C6orf201 | 1.875E-06 | -0.325 | - | - |
| 15064 | ENSP00000317671 | C12orf40 | 6.451E-06 | -0.325 | - | - |
| 15065 | ENSP00000430241 | EBF2 | 1.721E-05 | -0.325 | - | - |
| 15066 | ENSP00000424424 | SLC4A9 | 1.182E-05 | -0.325 | - | - |
| 15067 | ENSP00000264728 | TMEM40 | 2.334E-05 | -0.325 | - | - |
| 15068 | ENSP00000364782 | NEU1 | 2.866E-06 | -0.325 | - | - |
| 15069 | ENSP00000309126 | NSUN5 | 9.825E-06 | -0.325 | - | - |
| 15070 | ENSP00000400168 | ATP5J2-PTCD1 | 4.809E-06 | -0.325 | - | - |
| 15071 | ENSP00000369257 | RPGRIP1L | 1.655E-05 | -0.325 | - | - |
| 15072 | ENSP00000356951 | C1orf192 | 6.688E-06 | -0.325 | - | - |
| 15073 | ENSP00000356846 | MAEL | 1.489E-05 | -0.325 | - | - |
| 15074 | ENSP00000406052 | TDRD5 | 1.491E-05 | -0.325 | - | - |
| 15075 | ENSP00000368144 | PRR5L | 7.510E-06 | -0.325 | - | - |
| 15076 | ENSP00000383892 | TFIP11 | 1.479E-05 | -0.325 | - | - |
| 15077 | ENSP00000395232 | MTFR2 | 1.382E-05 | -0.325 | - | - |
| 15078 | ENSP00000386717 | RPL31 | 1.248E-05 | -0.325 | - | - |
| 15079 | ENSP00000427123 | RAI14 | 1.146E-05 | -0.325 | - | - |
| 15080 | ENSP00000374443 | LYST | 1.777E-05 | -0.325 | - | - |
| 15081 | ENSP00000387512 | AC139426.2 | 4.883E-06 | -0.325 | - | - |
| 15082 | ENSP00000219320 | SLC38A7 | 2.067E-05 | -0.325 | - | - |
| 15083 | ENSP00000396976 | BAHD1 | 7.740E-06 | -0.325 | - | - |
| 15084 | ENSP00000355428 | SH3BP5L | 8.347E-06 | -0.326 | - | - |
| 15085 | ENSP00000358861 | LCA5 | 1.267E-05 | -0.326 | - | - |
| 15086 | ENSP00000331479 | CYB5D1 | 3.707E-06 | -0.326 | - | - |
| 15087 | ENSP00000216468 | TMED8 | 1.690E-05 | -0.326 | - | - |
| 15088 | ENSP00000368869 | PRR16 | 8.587E-06 | -0.326 | - | - |
| 15089 | ENSP00000410858 | NHLRC4 | 4.530E-06 | -0.326 | - | - |
| 15090 | ENSP00000375107 | KRTAP19-6 | 1.791E-06 | -0.326 | - | - |
| 15091 | ENSP00000342056 | CS | 1.629E-05 | -0.326 | - | - |
| 15092 | ENSP00000355938 | FLVCR1 | 1.588E-05 | -0.326 | - | - |
| 15093 | ENSP00000306275 | KCNK3 | 8.826E-06 | -0.326 | - | - |
| 15094 | ENSP00000441000 | VARS2 | 1.193E-05 | -0.326 | - | - |
| 15095 | ENSP00000335223 | LCE4A | 1.648E-05 | -0.326 | - | - |
| 15096 | ENSP00000295201 | TEKT4 | 1.583E-05 | -0.326 | - | - |
| 15097 | ENSP00000305875 | C20orf196 | 7.258E-06 | -0.326 | - | - |
| 15098 | ENSP00000334836 | BMP2K | 9.224E-06 | -0.326 | - | - |
| 15099 | ENSP00000353512 | PARP9 | 1.950E-05 | -0.327 | - | - |
| 15100 | ENSP00000360163 | SMARCA1 | 2.123E-05 | -0.327 | - | - |
| 15101 | ENSP00000331915 | FAM101B | 1.976E-05 | -0.327 | - | - |
| 15102 | ENSP00000371546 | DEPDC5 | 1.371E-05 | -0.327 | - | - |
| 15103 | ENSP00000412215 | C4orf45 | 5.353E-06 | -0.327 | - | - |
| 15104 | ENSP00000376246 | MB21D2 | 5.161E-06 | -0.327 | - | - |
| 15105 | ENSP00000449784 | HECTD4 | 1.184E-05 | -0.327 | - | - |
| 15106 | ENSP00000451112 | NPC2 | 1.874E-05 | -0.327 | - | - |
| 15107 | ENSP00000364764 | LAYN | 4.432E-06 | -0.327 | - | - |
| 15108 | ENSP00000362456 | PBDC1 | 5.166E-06 | -0.327 | - | - |
| 15109 | ENSP00000343279 | FAM83G | 1.827E-05 | -0.327 | - | - |
| 15110 | ENSP00000370288 | SELO | 1.572E-05 | -0.327 | - | - |
| 15111 | ENSP00000355572 | COA6 | 4.664E-06 | -0.327 | - | - |
| 15112 | ENSP00000378430 | - | 5.590E-06 | -0.327 | - | - |
| 15113 | ENSP00000368856 | NFX1 | 1.970E-05 | -0.327 | - | - |
| 15114 | ENSP00000347602 | ARID4A | 1.356E-05 | -0.327 | - | - |
| 15115 | ENSP00000254323 | ZSWIM4 | 7.800E-06 | -0.327 | - | - |
| 15116 | ENSP00000318674 | FBXL16 | 1.988E-05 | -0.327 | - | - |
| 15117 | ENSP00000254286 | ACTR10 | 6.550E-06 | -0.327 | - | - |
| 15118 | ENSP00000248572 | GNGT1 | 1.339E-05 | -0.327 | - | - |
| 15119 | ENSP00000371661 | LCORL | 9.715E-06 | -0.327 | - | - |
| 15120 | ENSP00000357073 | IGSF9 | 1.497E-05 | -0.328 | - | - |
| 15121 | ENSP00000296776 | TMEM174 | 6.696E-06 | -0.328 | - | - |
| 15122 | ENSP00000387307 | RAB6C | 6.064E-06 | -0.328 | - | - |
| 15123 | ENSP00000348762 | LSS | 2.127E-05 | -0.328 | - | - |
| 15124 | ENSP00000313070 | PPIP5K2 | 1.798E-05 | -0.328 | - | - |
| 15125 | ENSP00000358635 | SYNCRIP | 9.663E-06 | -0.328 | - | - |
| 15126 | ENSP00000417289 | CTAGE8 | 7.059E-06 | -0.328 | - | - |
| 15127 | ENSP00000338276 | CRISP1 | 1.857E-05 | -0.328 | - | - |
| 15128 | ENSP00000381937 | C4orf29 | 6.210E-06 | -0.328 | - | - |
| 15129 | ENSP00000366942 | SLC36A3 | 1.379E-05 | -0.328 | - | - |
| 15130 | ENSP00000363478 | TMEM50A | 1.127E-05 | -0.328 | - | - |
| 15131 | ENSP00000384604 | FAM150B | 1.007E-05 | -0.328 | - | - |
| 15132 | ENSP00000470539 | PET100 | 2.954E-06 | -0.328 | - | - |
| 15133 | ENSP00000346918 | DHRS4-AS1 | 8.508E-06 | -0.328 | - | - |
| 15134 | ENSP00000398615 | GOLGA6L6 | 6.490E-06 | -0.328 | - | - |
| 15135 | ENSP00000356828 | RCSD1 | 6.787E-06 | -0.328 | - | - |
| 15136 | ENSP00000359305 | TMED5 | 1.434E-05 | -0.328 | - | - |
| 15137 | ENSP00000405252 | AKAP7 | 1.167E-05 | -0.328 | - | - |
| 15138 | ENSP00000358232 | PNLIPRP3 | 1.375E-05 | -0.328 | - | - |
| 15139 | ENSP00000368305 | GPCPD1 | 1.132E-05 | -0.328 | - | - |
| 15140 | ENSP00000466829 | RP11-318A15.7 | 5.109E-06 | -0.329 | - | - |
| 15141 | ENSP00000337224 | LRAT | 2.055E-05 | -0.329 | - | - |
| 15142 | ENSP00000362513 | UGT1A5 | 8.284E-06 | -0.329 | - | - |
| 15143 | ENSP00000422644 | SEPW1 | 9.124E-06 | -0.329 | - | - |
| 15144 | ENSP00000323872 | P2RY11 | 2.243E-05 | -0.329 | - | - |
| 15145 | ENSP00000355155 | GRIN3A | 1.828E-05 | -0.329 | - | - |
| 15146 | ENSP00000382239 | RBM12B | 9.494E-06 | -0.329 | - | - |
| 15147 | ENSP00000378857 | WDR6 | 1.305E-05 | -0.329 | - | - |
| 15148 | ENSP00000307129 | C8orf74 | 1.537E-05 | -0.329 | - | - |
| 15149 | ENSP00000362937 | RCC1 | 1.205E-05 | -0.329 | - | - |
| 15150 | ENSP00000386456 | AAK1 | 7.792E-06 | -0.329 | - | - |
| 15151 | ENSP00000399637 | GOLGA8I | 5.459E-06 | -0.329 | - | - |
| 15152 | ENSP00000350734 | ST7L | 1.294E-05 | -0.329 | - | - |
| 15153 | ENSP00000439182 | MYO1H | 9.455E-06 | -0.329 | - | - |
| 15154 | ENSP00000381377 | C15orf38-AP3S2 | 6.381E-06 | -0.329 | - | - |
| 15155 | ENSP00000386049 | MBD5 | 8.725E-06 | -0.329 | - | - |
| 15156 | ENSP00000364324 | FAM120C | 6.927E-06 | -0.329 | - | - |
| 15157 | ENSP00000356096 | FAM72A | 9.609E-06 | -0.329 | - | - |
| 15158 | ENSP00000306817 | RBKS | 6.491E-06 | -0.330 | - | - |
| 15159 | ENSP00000341108 | HMX2 | 1.900E-05 | -0.330 | - | - |
| 15160 | ENSP00000301332 | KIFC2 | 1.007E-05 | -0.330 | - | - |
| 15161 | ENSP00000351492 | UBL5 | 1.014E-05 | -0.330 | - | - |
| 15162 | ENSP00000417580 | IAH1 | 1.723E-06 | -0.330 | - | - |
| 15163 | ENSP00000419000 | LSAMP | 1.336E-05 | -0.330 | - | - |
| 15164 | ENSP00000321584 | IMPDH2 | 1.734E-05 | -0.330 | - | - |
| 15165 | ENSP00000378165 | ZNF207 | 1.278E-05 | -0.330 | - | - |
| 15166 | ENSP00000376417 | ARMC2 | 1.656E-05 | -0.330 | - | - |
| 15167 | ENSP00000393953 | PNPT1 | 1.317E-05 | -0.330 | - | - |
| 15168 | ENSP00000343348 | VMAC | 1.563E-05 | -0.330 | - | - |
| 15169 | ENSP00000377496 | ACD | 1.206E-05 | -0.330 | - | - |
| 15170 | ENSP00000347586 | CCDC69 | 1.518E-05 | -0.330 | - | - |
| 15171 | ENSP00000252032 | C20orf194 | 6.967E-06 | -0.330 | - | - |
| 15172 | ENSP00000364293 | TSR2 | 1.051E-05 | -0.330 | - | - |
| 15173 | ENSP00000388658 | SLC25A12 | 6.606E-06 | -0.330 | - | - |
| 15174 | ENSP00000360076 | SGIP1 | 1.966E-05 | -0.330 | - | - |
| 15175 | ENSP00000418693 | RWDD2B | 7.490E-06 | -0.330 | - | - |
| 15176 | ENSP00000354643 | DIO1 | 1.212E-05 | -0.330 | - | - |
| 15177 | ENSP00000290823 | GPR114 | 1.398E-05 | -0.330 | - | - |
| 15178 | ENSP00000332162 | C1orf64 | 1.140E-05 | -0.330 | - | - |
| 15179 | ENSP00000419944 | PXT1 | 2.888E-06 | -0.330 | - | - |
| 15180 | ENSP00000420821 | RPL41 | 1.359E-05 | -0.330 | - | - |
| 15181 | ENSP00000357863 | GTF3C6 | 1.991E-05 | -0.330 | - | - |
| 15182 | ENSP00000306900 | PRND | 1.061E-05 | -0.330 | - | - |
| 15183 | ENSP00000288048 | C1orf158 | 2.375E-05 | -0.330 | - | - |
| 15184 | ENSP00000371811 | MICU2 | 5.841E-06 | -0.330 | - | - |
| 15185 | ENSP00000378138 | TMEM98 | 8.182E-06 | -0.330 | - | - |
| 15186 | ENSP00000454445 | RP11-463D19.2 | 1.698E-05 | -0.331 | - | - |
| 15187 | ENSP00000356476 | TRMT1L | 6.598E-06 | -0.331 | - | - |
| 15188 | ENSP00000463483 | KRT222 | 9.448E-06 | -0.331 | - | - |
| 15189 | ENSP00000331784 | C7orf13 | 6.237E-06 | -0.331 | - | - |
| 15190 | ENSP00000430846 | KCNB2 | 1.911E-05 | -0.331 | - | - |
| 15191 | ENSP00000366144 | SOX21 | 1.226E-05 | -0.331 | - | - |
| 15192 | ENSP00000362566 | HPCA | 1.639E-05 | -0.331 | - | - |
| 15193 | ENSP00000332171 | DMTF1 | 1.449E-05 | -0.331 | - | - |
| 15194 | ENSP00000278360 | PAMR1 | 1.700E-05 | -0.331 | - | - |
| 15195 | ENSP00000275461 | FERD3L | 2.154E-05 | -0.331 | - | - |
| 15196 | ENSP00000362993 | BICC1 | 7.412E-06 | -0.331 | - | - |
| 15197 | ENSP00000349250 | ARL4A | 1.330E-05 | -0.331 | - | - |
| 15198 | ENSP00000373713 | SACM1L | 2.355E-05 | -0.331 | - | - |
| 15199 | ENSP00000340982 | SPAG8 | 1.168E-05 | -0.331 | - | - |
| 15200 | ENSP00000414712 | MAP7 | 6.544E-06 | -0.331 | - | - |
| 15201 | ENSP00000387185 | ATHL1 | 1.960E-05 | -0.331 | - | - |
| 15202 | ENSP00000334592 | CYP2R1 | 7.145E-06 | -0.331 | - | - |
| 15203 | ENSP00000359660 | AVPI1 | 8.355E-06 | -0.331 | - | - |
| 15204 | ENSP00000429734 | 61E3.4 | 9.857E-06 | -0.331 | - | - |
| 15205 | ENSP00000411004 | XG | 5.826E-06 | -0.331 | - | - |
| 15206 | ENSP00000316674 | ACTL9 | 1.176E-05 | -0.331 | - | - |
| 15207 | ENSP00000454697 | BLOC1S5-TXNDC5 | 1.706E-06 | -0.332 | - | - |
| 15208 | ENSP00000343900 | CPSF4L | 9.964E-06 | -0.332 | - | - |
| 15209 | ENSP00000370607 | TECRL | 1.788E-05 | -0.332 | - | - |
| 15210 | ENSP00000472810 | AC090673.2 | 1.230E-06 | -0.332 | - | - |
| 15211 | ENSP00000385025 | SMCR8 | 3.960E-06 | -0.332 | - | - |
| 15212 | ENSP00000356286 | TNNT2 | 1.867E-05 | -0.332 | - | - |
| 15213 | ENSP00000354688 | FAM45A | 4.311E-06 | -0.332 | - | - |
| 15214 | ENSP00000309945 | SPNS1 | 1.541E-05 | -0.332 | - | - |
| 15215 | ENSP00000363727 | STARD8 | 1.616E-05 | -0.332 | - | - |
| 15216 | ENSP00000354227 | C11orf80 | 1.653E-05 | -0.332 | - | - |
| 15217 | ENSP00000379946 | GLTSCR1 | 9.782E-06 | -0.332 | - | - |
| 15218 | ENSP00000457031 | CHKB-CPT1B | 3.192E-06 | -0.332 | - | - |
| 15219 | ENSP00000415564 | TCEAL4 | 4.584E-06 | -0.332 | - | - |
| 15220 | ENSP00000411672 | ATP6V0E2 | 9.242E-06 | -0.332 | - | - |
| 15221 | ENSP00000360312 | BSND | 1.382E-05 | -0.332 | - | - |
| 15222 | ENSP00000358062 | AIM1 | 1.669E-05 | -0.332 | - | - |
| 15223 | ENSP00000333627 | NKAIN3 | 1.275E-05 | -0.332 | - | - |
| 15224 | ENSP00000383145 | SFI1 | 2.440E-05 | -0.332 | - | - |
| 15225 | ENSP00000398106 | ZBED5 | 5.164E-06 | -0.332 | - | - |
| 15226 | ENSP00000395281 | C9orf116 | 4.988E-06 | -0.332 | - | - |
| 15227 | ENSP00000361699 | CTPS1 | 1.778E-05 | -0.332 | - | - |
| 15228 | ENSP00000341549 | KLHL33 | 1.862E-05 | -0.332 | - | - |
| 15229 | ENSP00000410818 | MAD2L1BP | 3.624E-06 | -0.332 | - | - |
| 15230 | ENSP00000354255 | CPZ | 1.663E-05 | -0.333 | - | - |
| 15231 | ENSP00000267838 | LYSMD2 | 1.429E-05 | -0.333 | - | - |
| 15232 | ENSP00000378409 | C10orf54 | 4.183E-06 | -0.333 | - | - |
| 15233 | ENSP00000427428 | FAM218A | 1.356E-06 | -0.333 | - | - |
| 15234 | ENSP00000359454 | HFM1 | 1.130E-05 | -0.333 | - | - |
| 15235 | ENSP00000378414 | SMARCD1 | 1.781E-05 | -0.333 | - | - |
| 15236 | ENSP00000348128 | SVIL | 1.472E-05 | -0.333 | - | - |
| 15237 | ENSP00000355493 | ADSS | 1.415E-05 | -0.333 | - | - |
| 15238 | ENSP00000371051 | ZZEF1 | 1.932E-05 | -0.333 | - | - |
| 15239 | ENSP00000373304 | CCDC174 | 4.198E-06 | -0.333 | - | - |
| 15240 | ENSP00000364609 | PADI3 | 1.127E-05 | -0.333 | - | - |
| 15241 | ENSP00000355205 | INO80 | 1.205E-05 | -0.333 | - | - |
| 15242 | ENSP00000404220 | CCDC155 | 7.386E-06 | -0.333 | - | - |
| 15243 | ENSP00000378328 | ANKRD13B | 6.227E-06 | -0.333 | - | - |
| 15244 | ENSP00000366487 | ARL5B | 1.521E-05 | -0.333 | - | - |
| 15245 | ENSP00000225235 | TBC1D12 | 1.894E-05 | -0.333 | - | - |
| 15246 | ENSP00000384330 | MYH8 | 1.716E-05 | -0.333 | - | - |
| 15247 | ENSP00000330098 | FBXL6 | 1.004E-05 | -0.333 | - | - |
| 15248 | ENSP00000424381 | LMF2 | 7.401E-06 | -0.333 | - | - |
| 15249 | ENSP00000361646 | ZMYND12 | 1.031E-05 | -0.333 | - | - |
| 15250 | ENSP00000260191 | HTR3B | 2.451E-05 | -0.333 | - | - |
| 15251 | ENSP00000434045 | GPR68 | 2.234E-05 | -0.334 | - | - |
| 15252 | ENSP00000387262 | IMMT | 1.569E-05 | -0.334 | - | - |
| 15253 | ENSP00000387187 | SERF2 | 9.823E-06 | -0.334 | - | - |
| 15254 | ENSP00000338607 | THEM6 | 2.120E-05 | -0.334 | - | - |
| 15255 | ENSP00000354588 | TEAD1 | 1.979E-05 | -0.334 | - | - |
| 15256 | ENSP00000323275 | DYX1C1 | 8.850E-06 | -0.334 | - | - |
| 15257 | ENSP00000363270 | RUFY4 | 4.663E-06 | -0.334 | - | - |
| 15258 | ENSP00000372088 | RAD51 | 5.337E-05 | -0.334 | - | - |
| 15259 | ENSP00000370479 | VCX3A | 5.101E-06 | -0.334 | - | - |
| 15260 | ENSP00000378917 | ATXN2L | 9.160E-06 | -0.334 | - | - |
| 15261 | ENSP00000440273 | SH2B2 | 8.495E-06 | -0.334 | - | - |
| 15262 | ENSP00000431245 | GUCY1A2 | 7.529E-06 | -0.334 | - | - |
| 15263 | ENSP00000466298 | STX10 | 4.762E-06 | -0.334 | - | - |
| 15264 | ENSP00000355185 | DCLRE1A | 1.767E-05 | -0.335 | - | - |
| 15265 | ENSP00000333018 | TTC32 | 8.022E-06 | -0.335 | - | - |
| 15266 | ENSP00000402584 | MDGA1 | 1.701E-05 | -0.335 | - | - |
| 15267 | ENSP00000290378 | ACTC1 | 1.135E-05 | -0.335 | - | - |
| 15268 | ENSP00000358262 | CHD1L | 1.850E-05 | -0.335 | - | - |
| 15269 | ENSP00000418744 | SH3GLB1 | 1.124E-05 | -0.335 | - | - |
| 15270 | ENSP00000319623 | IGDCC4 | 1.677E-05 | -0.335 | - | - |
| 15271 | ENSP00000337477 | FAM60A | 1.058E-05 | -0.335 | - | - |
| 15272 | ENSP00000404658 | EZH1 | 1.351E-05 | -0.335 | - | - |
| 15273 | ENSP00000348380 | CYP20A1 | 7.044E-06 | -0.335 | - | - |
| 15274 | ENSP00000416892 | EIF3L | 9.538E-06 | -0.335 | - | - |
| 15275 | ENSP00000364371 | HIATL2 | 2.552E-06 | -0.335 | - | - |
| 15276 | ENSP00000384222 | WWC2 | 9.548E-06 | -0.335 | - | - |
| 15277 | ENSP00000417696 | ZNF525 | 1.275E-05 | -0.335 | - | - |
| 15278 | ENSP00000402644 | TRIM16 | 2.413E-06 | -0.335 | - | - |
| 15279 | ENSP00000369081 | TXNDC5 | 2.064E-05 | -0.335 | - | - |
| 15280 | ENSP00000291691 | C21orf58 | 6.056E-06 | -0.335 | - | - |
| 15281 | ENSP00000373918 | GARS | 1.030E-05 | -0.335 | - | - |
| 15282 | ENSP00000307951 | HIGD2B | 1.499E-05 | -0.335 | - | - |
| 15283 | ENSP00000265969 | KCNC1 | 1.900E-05 | -0.335 | - | - |
| 15284 | ENSP00000261070 | COX17 | 8.870E-06 | -0.335 | - | - |
| 15285 | ENSP00000398290 | NDUFA3 | 3.296E-06 | -0.335 | - | - |
| 15286 | ENSP00000379834 | SLC2A14 | 1.777E-05 | -0.335 | - | - |
| 15287 | ENSP00000302400 | SMR3B | 7.379E-06 | -0.335 | - | - |
| 15288 | ENSP00000436897 | SMIM3 | 1.118E-05 | -0.336 | - | - |
| 15289 | ENSP00000346537 | SMOC2 | 1.663E-05 | -0.336 | - | - |
| 15290 | ENSP00000439660 | HIST1H3G | 2.215E-05 | -0.336 | - | - |
| 15291 | ENSP00000462046 | PIRT | 3.307E-06 | -0.336 | - | - |
| 15292 | ENSP00000433712 | SPRN | 1.511E-05 | -0.336 | - | - |
| 15293 | ENSP00000365534 | THNSL1 | 7.509E-06 | -0.336 | - | - |
| 15294 | ENSP00000355632 | GALNT2 | 1.127E-05 | -0.336 | - | - |
| 15295 | ENSP00000366342 | - | 1.008E-05 | -0.336 | - | - |
| 15296 | ENSP00000367049 | MZT1 | 1.167E-05 | -0.336 | - | - |
| 15297 | ENSP00000380942 | ARHGEF12 | 2.370E-05 | -0.336 | - | - |
| 15298 | ENSP00000417569 | STIM2 | 1.139E-05 | -0.336 | - | - |
| 15299 | ENSP00000365757 | PIP4K2A | 1.910E-05 | -0.336 | - | - |
| 15300 | ENSP00000363832 | AOX1 | 2.430E-05 | -0.336 | - | - |
| 15301 | ENSP00000335447 | HEATR4 | 1.935E-05 | -0.336 | - | - |
| 15302 | ENSP00000358474 | BRCC3 | 1.114E-05 | -0.336 | - | - |
| 15303 | ENSP00000366939 | MGME1 | 1.325E-05 | -0.336 | - | - |
| 15304 | ENSP00000274278 | UGT3A1 | 7.495E-06 | -0.336 | - | - |
| 15305 | ENSP00000397759 | BSDC1 | 3.194E-06 | -0.336 | - | - |
| 15306 | ENSP00000304032 | STARD5 | 6.898E-06 | -0.336 | - | - |
| 15307 | ENSP00000386141 | XKR9 | 8.044E-06 | -0.336 | - | - |
| 15308 | ENSP00000380378 | PAFAH1B1 | 2.678E-05 | -0.336 | - | - |
| 15309 | ENSP00000379430 | NDUFAF6 | 7.158E-06 | -0.336 | - | - |
| 15310 | ENSP00000361900 | TOMM34 | 1.220E-05 | -0.336 | - | - |
| 15311 | ENSP00000358233 | GPR89B | 1.160E-05 | -0.336 | - | - |
| 15312 | ENSP00000457601 | TMSB15B | 1.031E-05 | -0.336 | - | - |
| 15313 | ENSP00000384496 | PPP1R12B | 1.872E-05 | -0.336 | - | - |
| 15314 | ENSP00000362018 | CENPI | 7.899E-06 | -0.337 | - | - |
| 15315 | ENSP00000381476 | TMPRSS12 | 8.090E-06 | -0.337 | - | - |
| 15316 | ENSP00000362442 | ARHGAP40 | 1.407E-05 | -0.337 | - | - |
| 15317 | ENSP00000375479 | KRTAP10-2 | 1.818E-06 | -0.337 | - | - |
| 15318 | ENSP00000364728 | XAGE1D | 2.823E-06 | -0.337 | - | - |
| 15319 | ENSP00000367353 | C12orf42 | 5.556E-06 | -0.337 | - | - |
| 15320 | ENSP00000265068 | KIAA1257 | 1.219E-05 | -0.337 | - | - |
| 15321 | ENSP00000359370 | SEC31B | 1.743E-05 | -0.337 | - | - |
| 15322 | ENSP00000350854 | VPS13D | 1.510E-05 | -0.337 | - | - |
| 15323 | ENSP00000312789 | DYRK1B | 1.212E-05 | -0.337 | - | - |
| 15324 | ENSP00000419038 | GFM1 | 1.559E-05 | -0.337 | - | - |
| 15325 | ENSP00000370800 | MBD3L2 | 8.384E-06 | -0.337 | - | - |
| 15326 | ENSP00000267484 | RTN1 | 1.333E-05 | -0.337 | - | - |
| 15327 | ENSP00000389787 | PLEKHF1 | 1.146E-05 | -0.337 | - | - |
| 15328 | ENSP00000444617 | NHSL2 | 8.064E-06 | -0.337 | - | - |
| 15329 | ENSP00000323217 | GOLGA8R | 9.048E-06 | -0.337 | - | - |
| 15330 | ENSP00000345892 | NDE1 | 1.219E-05 | -0.337 | - | - |
| 15331 | ENSP00000318119 | C9orf139 | 8.035E-06 | -0.337 | - | - |
| 15332 | ENSP00000006658 | SPATA20 | 2.563E-05 | -0.337 | - | - |
| 15333 | ENSP00000327168 | PRR7 | 5.132E-06 | -0.337 | - | - |
| 15334 | ENSP00000453502 | ST20 | 6.021E-06 | -0.337 | - | - |
| 15335 | ENSP00000362900 | SRSF4 | 8.954E-06 | -0.337 | - | - |
| 15336 | ENSP00000472698 | L34079.2 | 4.324E-06 | -0.337 | - | - |
| 15337 | ENSP00000427108 | CDKN2AIP | 5.615E-06 | -0.337 | - | - |
| 15338 | ENSP00000420127 | RAB9A | 2.098E-05 | -0.338 | - | - |
| 15339 | ENSP00000381270 | C6orf170 | 3.211E-06 | -0.338 | - | - |
| 15340 | ENSP00000338967 | ARHGAP6 | 1.648E-05 | -0.338 | - | - |
| 15341 | ENSP00000418194 | PARP14 | 1.510E-05 | -0.338 | - | - |
| 15342 | ENSP00000361640 | SH3GLB2 | 1.272E-05 | -0.338 | - | - |
| 15343 | ENSP00000351346 | VPS13B | 1.300E-05 | -0.338 | - | - |
| 15344 | ENSP00000364092 | ASIP | 1.552E-05 | -0.338 | - | - |
| 15345 | ENSP00000329995 | FAM186A | 7.328E-06 | -0.338 | - | - |
| 15346 | ENSP00000367752 | NRSN1 | 2.869E-06 | -0.338 | - | - |
| 15347 | ENSP00000299866 | TVP23A | 7.952E-06 | -0.338 | - | - |
| 15348 | ENSP00000253362 | BPIFA2 | 1.600E-05 | -0.338 | - | - |
| 15349 | ENSP00000361095 | TMEM69 | 9.535E-06 | -0.338 | - | - |
| 15350 | ENSP00000354689 | SLC39A13 | 8.052E-06 | -0.338 | - | - |
| 15351 | ENSP00000457628 | NUB1 | 6.103E-06 | -0.338 | - | - |
| 15352 | ENSP00000361177 | LRIT1 | 1.385E-05 | -0.338 | - | - |
| 15353 | ENSP00000341819 | VWC2 | 1.828E-05 | -0.338 | - | - |
| 15354 | ENSP00000353643 | MORF4L2 | 1.180E-05 | -0.338 | - | - |
| 15355 | ENSP00000455075 | AC114783.1 | 1.021E-06 | -0.338 | - | - |
| 15356 | ENSP00000361731 | TBC1D13 | 9.157E-06 | -0.338 | - | - |
| 15357 | ENSP00000337209 | CCDC68 | 5.007E-06 | -0.339 | - | - |
| 15358 | ENSP00000385450 | MAGI1 | 1.816E-05 | -0.339 | - | - |
| 15359 | ENSP00000350358 | PRAMEF12 | 1.288E-05 | -0.339 | - | - |
| 15360 | ENSP00000378624 | HERC4 | 1.922E-05 | -0.339 | - | - |
| 15361 | ENSP00000406367 | GPR124 | 1.155E-05 | -0.339 | - | - |
| 15362 | ENSP00000277491 | AL354898.1 | 2.036E-05 | -0.339 | - | - |
| 15363 | ENSP00000472465 | SARS2 | 9.051E-06 | -0.339 | - | - |
| 15364 | ENSP00000215912 | PIK3IP1 | 1.996E-05 | -0.339 | - | - |
| 15365 | ENSP00000383392 | NCAM2 | 9.922E-06 | -0.339 | - | - |
| 15366 | ENSP00000377148 | AP1G1 | 1.965E-05 | -0.339 | - | - |
| 15367 | ENSP00000364526 | KIAA1462 | 1.045E-05 | -0.339 | - | - |
| 15368 | ENSP00000406327 | COX20 | 5.847E-06 | -0.339 | - | - |
| 15369 | ENSP00000375691 | JOSD2 | 4.005E-06 | -0.339 | - | - |
| 15370 | ENSP00000348381 | ADD3 | 1.563E-05 | -0.339 | - | - |
| 15371 | ENSP00000408860 | CYP21A2 | 7.868E-06 | -0.339 | - | - |
| 15372 | ENSP00000429896 | FAM178B | 1.428E-06 | -0.339 | - | - |
| 15373 | ENSP00000360327 | PARS2 | 3.893E-06 | -0.339 | - | - |
| 15374 | ENSP00000358146 | BOLA1 | 1.158E-05 | -0.339 | - | - |
| 15375 | ENSP00000419235 | FAM115A | 6.648E-06 | -0.339 | - | - |
| 15376 | ENSP00000413572 | NLE1 | 2.616E-05 | -0.339 | - | - |
| 15377 | ENSP00000354622 | RUNDC1 | 5.487E-06 | -0.339 | - | - |
| 15378 | ENSP00000360762 | ANKRD1 | 1.374E-05 | -0.339 | - | - |
| 15379 | ENSP00000364603 | BPIFA3 | 4.817E-06 | -0.339 | - | - |
| 15380 | ENSP00000354991 | ALPK2 | 1.001E-05 | -0.339 | - | - |
| 15381 | ENSP00000368025 | WDR87 | 4.680E-06 | -0.339 | - | - |
| 15382 | ENSP00000446058 | SHD | 1.017E-05 | -0.339 | - | - |
| 15383 | ENSP00000254336 | C19orf57 | 1.186E-05 | -0.339 | - | - |
| 15384 | ENSP00000463533 | KCNH6 | 1.378E-05 | -0.339 | - | - |
| 15385 | ENSP00000361110 | GBGT1 | 1.154E-05 | -0.339 | - | - |
| 15386 | ENSP00000348959 | MAN1A2 | 1.391E-05 | -0.339 | - | - |
| 15387 | ENSP00000300022 | YPEL4 | 1.775E-05 | -0.339 | - | - |
| 15388 | ENSP00000360116 | UBE2U | 1.767E-05 | -0.340 | - | - |
| 15389 | ENSP00000382177 | MYO5A | 1.979E-05 | -0.340 | - | - |
| 15390 | ENSP00000352222 | CTPS2 | 1.442E-05 | -0.340 | - | - |
| 15391 | ENSP00000289672 | PKD1L1 | 2.262E-05 | -0.340 | - | - |
| 15392 | ENSP00000421258 | ZNF154 | 1.287E-05 | -0.340 | - | - |
| 15393 | ENSP00000380505 | NEB | 1.773E-05 | -0.340 | - | - |
| 15394 | ENSP00000328017 | KRBA2 | 4.011E-06 | -0.340 | - | - |
| 15395 | ENSP00000264659 | SRCIN1 | 2.223E-05 | -0.340 | - | - |
| 15396 | ENSP00000360855 | SLC16A12 | 9.011E-06 | -0.340 | - | - |
| 15397 | ENSP00000356940 | MOXD1 | 1.336E-05 | -0.340 | - | - |
| 15398 | ENSP00000334767 | CCDC84 | 9.735E-06 | -0.340 | - | - |
| 15399 | ENSP00000368920 | NHLRC3 | 1.011E-05 | -0.340 | - | - |
| 15400 | ENSP00000421586 | GOLGA6L4 | 7.939E-06 | -0.340 | - | - |
| 15401 | ENSP00000389014 | C5orf42 | 2.196E-06 | -0.340 | - | - |
| 15402 | ENSP00000299320 | C16orf71 | 2.390E-05 | -0.340 | - | - |
| 15403 | ENSP00000307006 | IRX2 | 2.007E-05 | -0.340 | - | - |
| 15404 | ENSP00000291481 | HAPLN4 | 1.491E-05 | -0.340 | - | - |
| 15405 | ENSP00000348064 | ART3 | 1.173E-05 | -0.340 | - | - |
| 15406 | ENSP00000447650 | RP11-644F5.10 | 1.812E-06 | -0.340 | - | - |
| 15407 | ENSP00000360381 | CDCP2 | 4.681E-06 | -0.340 | - | - |
| 15408 | ENSP00000333737 | C1QTNF9 | 1.001E-05 | -0.340 | - | - |
| 15409 | ENSP00000330375 | EPGN | 1.251E-05 | -0.340 | - | - |
| 15410 | ENSP00000318912 | CA13 | 9.930E-06 | -0.340 | - | - |
| 15411 | ENSP00000360366 | ACOT11 | 1.122E-05 | -0.340 | - | - |
| 15412 | ENSP00000260447 | GCHFR | 2.632E-05 | -0.341 | - | - |
| 15413 | ENSP00000248089 | HCFC1R1 | 1.816E-05 | -0.341 | - | - |
| 15414 | ENSP00000446121 | LIMS1 | 7.515E-06 | -0.341 | - | - |
| 15415 | ENSP00000379754 | CCDC125 | 5.239E-06 | -0.341 | - | - |
| 15416 | ENSP00000301917 | GPR25 | 1.460E-05 | -0.341 | - | - |
| 15417 | ENSP00000420863 | SERINC5 | 1.492E-05 | -0.341 | - | - |
| 15418 | ENSP00000391432 | RNPC3 | 8.691E-06 | -0.341 | - | - |
| 15419 | ENSP00000362555 | RNF19B | 1.487E-05 | -0.341 | - | - |
| 15420 | ENSP00000417706 | DCUN1D2 | 8.090E-06 | -0.341 | - | - |
| 15421 | ENSP00000425658 | ANKRD50 | 6.773E-06 | -0.341 | - | - |
| 15422 | ENSP00000442057 | CINP | 8.956E-06 | -0.341 | - | - |
| 15423 | ENSP00000429064 | MTSS1 | 1.383E-05 | -0.341 | - | - |
| 15424 | ENSP00000452521 | NAA30 | 1.106E-05 | -0.341 | - | - |
| 15425 | ENSP00000411514 | ZNF385C | 4.312E-06 | -0.341 | - | - |
| 15426 | ENSP00000375087 | C8orf44 | 1.261E-05 | -0.341 | - | - |
| 15427 | ENSP00000359583 | HPSE2 | 1.527E-05 | -0.341 | - | - |
| 15428 | ENSP00000326340 | ATG16L2 | 1.183E-05 | -0.341 | - | - |
| 15429 | ENSP00000301258 | PSCA | 1.614E-05 | -0.341 | - | - |
| 15430 | ENSP00000317564 | EID2B | 1.592E-05 | -0.341 | - | - |
| 15431 | ENSP00000381250 | APOF | 7.116E-06 | -0.341 | - | - |
| 15432 | ENSP00000383840 | MMACHC | 6.695E-06 | -0.341 | - | - |
| 15433 | ENSP00000423014 | OCIAD2 | 4.972E-06 | -0.341 | - | - |
| 15434 | ENSP00000404923 | TECPR1 | 1.019E-05 | -0.342 | - | - |
| 15435 | ENSP00000211413 | PRRT1 | 7.825E-06 | -0.342 | - | - |
| 15436 | ENSP00000403925 | TBCEL | 4.883E-06 | -0.342 | - | - |
| 15437 | ENSP00000441024 | UBAP1 | 1.237E-05 | -0.342 | - | - |
| 15438 | ENSP00000442778 | TRAPPC2P1 | 1.692E-05 | -0.342 | - | - |
| 15439 | ENSP00000289228 | ACTR1B | 8.850E-06 | -0.342 | - | - |
| 15440 | ENSP00000298032 | ARMC3 | 1.983E-05 | -0.342 | - | - |
| 15441 | ENSP00000419370 | MALSU1 | 5.028E-06 | -0.342 | - | - |
| 15442 | ENSP00000326708 | OVCH1 | 1.487E-05 | -0.342 | - | - |
| 15443 | ENSP00000386532 | ASB18 | 1.680E-05 | -0.342 | - | - |
| 15444 | ENSP00000362387 | TSPAN15 | 8.434E-06 | -0.342 | - | - |
| 15445 | ENSP00000239451 | SLC25A2 | 1.604E-05 | -0.342 | - | - |
| 15446 | ENSP00000369346 | ARSK | 8.345E-06 | -0.342 | - | - |
| 15447 | ENSP00000367848 | GABRD | 1.529E-05 | -0.342 | - | - |
| 15448 | ENSP00000367697 | ATP6AP2 | 6.411E-06 | -0.342 | - | - |
| 15449 | ENSP00000361275 | PLK3 | 1.497E-05 | -0.342 | - | - |
| 15450 | ENSP00000348593 | ZFYVE27 | 1.065E-05 | -0.342 | - | - |
| 15451 | ENSP00000355444 | GCSAML | 3.331E-06 | -0.342 | - | - |
| 15452 | ENSP00000299492 | PPFIBP2 | 1.495E-05 | -0.342 | - | - |
| 15453 | ENSP00000391564 | RIMBP3 | 6.025E-06 | -0.342 | - | - |
| 15454 | ENSP00000312272 | YPEL2 | 1.471E-05 | -0.343 | - | - |
| 15455 | ENSP00000371973 | SAP18 | 1.272E-05 | -0.343 | - | - |
| 15456 | ENSP00000246000 | - | 2.659E-05 | -0.343 | - | - |
| 15457 | ENSP00000327509 | STAC2 | 3.347E-06 | -0.343 | - | - |
| 15458 | ENSP00000343290 | C9orf171 | 9.238E-06 | -0.343 | - | - |
| 15459 | ENSP00000298569 | KIAA1191 | 2.221E-05 | -0.343 | - | - |
| 15460 | ENSP00000358896 | AS3MT | 5.831E-06 | -0.343 | - | - |
| 15461 | ENSP00000406027 | EPM2AIP1 | 7.532E-06 | -0.343 | - | - |
| 15462 | ENSP00000360519 | RBP4 | 6.139E-06 | -0.343 | - | - |
| 15463 | ENSP00000369611 | SPZ1 | 7.945E-06 | -0.343 | - | - |
| 15464 | ENSP00000332900 | GPR97 | 2.045E-05 | -0.343 | - | - |
| 15465 | ENSP00000359035 | RTEL1 | 1.477E-05 | -0.343 | - | - |
| 15466 | ENSP00000378295 | INCENP | 1.013E-05 | -0.343 | - | - |
| 15467 | ENSP00000317445 | PSAPL1 | 1.382E-05 | -0.343 | - | - |
| 15468 | ENSP00000415634 | - | 1.888E-05 | -0.343 | - | - |
| 15469 | ENSP00000404705 | NAALADL2 | 1.177E-05 | -0.343 | - | - |
| 15470 | ENSP00000342709 | PRR19 | 4.793E-06 | -0.343 | - | - |
| 15471 | ENSP00000373640 | AGPHD1 | 2.876E-06 | -0.343 | - | - |
| 15472 | ENSP00000379201 | METTL21D | 1.840E-06 | -0.343 | - | - |
| 15473 | ENSP00000305631 | NXNL1 | 9.285E-06 | -0.343 | - | - |
| 15474 | ENSP00000385000 | KIF2A | 1.777E-05 | -0.343 | - | - |
| 15475 | ENSP00000370215 | NOXRED1 | 9.072E-06 | -0.343 | - | - |
| 15476 | ENSP00000332313 | PIGW | 8.184E-06 | -0.343 | - | - |
| 15477 | ENSP00000377686 | MED24 | 1.158E-05 | -0.344 | - | - |
| 15478 | ENSP00000442436 | URI1 | 7.602E-06 | -0.344 | - | - |
| 15479 | ENSP00000319590 | PRR18 | 1.492E-05 | -0.344 | - | - |
| 15480 | ENSP00000356982 | UFC1 | 8.421E-06 | -0.344 | - | - |
| 15481 | ENSP00000293274 | LYZL6 | 2.131E-05 | -0.344 | - | - |
| 15482 | ENSP00000386134 | NGDN | 6.950E-06 | -0.344 | - | - |
| 15483 | ENSP00000367894 | PRRG1 | 5.814E-06 | -0.344 | - | - |
| 15484 | ENSP00000372192 | TMEM211 | 5.018E-07 | -0.344 | - | - |
| 15485 | ENSP00000356231 | RABIF | 8.223E-06 | -0.344 | - | - |
| 15486 | ENSP00000360985 | ARFGEF2 | 1.550E-05 | -0.344 | - | - |
| 15487 | ENSP00000346997 | SUV39H2 | 1.100E-05 | -0.344 | - | - |
| 15488 | ENSP00000349142 | ATP5C1 | 1.955E-05 | -0.344 | - | - |
| 15489 | ENSP00000374218 | ESYT3 | 1.594E-05 | -0.344 | - | - |
| 15490 | ENSP00000409799 | RPP21 | 5.669E-06 | -0.344 | - | - |
| 15491 | ENSP00000256367 | TTC9 | 1.472E-05 | -0.344 | - | - |
| 15492 | ENSP00000370224 | PARM1 | 2.020E-05 | -0.344 | - | - |
| 15493 | ENSP00000367228 | - | 9.368E-06 | -0.344 | - | - |
| 15494 | ENSP00000384004 | POU6F2 | 1.342E-05 | -0.344 | - | - |
| 15495 | ENSP00000380380 | C9orf38 | 2.381E-06 | -0.344 | - | - |
| 15496 | ENSP00000363745 | HNRNPR | 9.126E-06 | -0.344 | - | - |
| 15497 | ENSP00000272367 | CLEC4F | 1.517E-05 | -0.344 | - | - |
| 15498 | ENSP00000288025 | TMED6 | 2.034E-05 | -0.344 | - | - |
| 15499 | ENSP00000291536 | RSPH1 | 1.350E-05 | -0.344 | - | - |
| 15500 | ENSP00000363603 | FUCA1 | 8.946E-06 | -0.344 | - | - |
| 15501 | ENSP00000417914 | ASB11 | 1.875E-05 | -0.344 | - | - |
| 15502 | ENSP00000438125 | TPCN1 | 9.454E-06 | -0.344 | - | - |
| 15503 | ENSP00000353670 | SPOCD1 | 1.396E-05 | -0.344 | - | - |
| 15504 | ENSP00000360437 | FAM210B | 8.337E-06 | -0.344 | - | - |
| 15505 | ENSP00000216799 | EMC9 | 1.585E-05 | -0.344 | - | - |
| 15506 | ENSP00000367276 | CKAP2 | 1.001E-05 | -0.344 | - | - |
| 15507 | ENSP00000374387 | TRMT44 | 8.033E-06 | -0.345 | - | - |
| 15508 | ENSP00000381657 | DOT1L | 1.118E-05 | -0.345 | - | - |
| 15509 | ENSP00000357008 | ITLN2 | 1.547E-05 | -0.345 | - | - |
| 15510 | ENSP00000381109 | ZCWPW1 | 9.203E-06 | -0.345 | - | - |
| 15511 | ENSP00000266546 | CLSTN3 | 1.223E-05 | -0.345 | - | - |
| 15512 | ENSP00000370568 | KIAA1644 | 5.897E-06 | -0.345 | - | - |
| 15513 | ENSP00000333547 | RAB11B | 1.029E-05 | -0.345 | - | - |
| 15514 | ENSP00000445905 | EVI5L | 3.474E-06 | -0.345 | - | - |
| 15515 | ENSP00000357998 | SEC63 | 1.673E-05 | -0.345 | - | - |
| 15516 | ENSP00000364298 | FKBPL | 8.079E-06 | -0.345 | - | - |
| 15517 | ENSP00000359234 | PDZD7 | 1.894E-05 | -0.345 | - | - |
| 15518 | ENSP00000402595 | TRIM34 | 1.154E-05 | -0.345 | - | - |
| 15519 | ENSP00000367139 | SPINK9 | 5.265E-06 | -0.345 | - | - |
| 15520 | ENSP00000358853 | SH3BGRL2 | 1.931E-05 | -0.345 | - | - |
| 15521 | ENSP00000431116 | AC133919.6 | 1.990E-06 | -0.345 | - | - |
| 15522 | ENSP00000363388 | SUSD1 | 3.048E-06 | -0.345 | - | - |
| 15523 | ENSP00000383216 | KRTAP10-12 | 1.603E-06 | -0.346 | - | - |
| 15524 | ENSP00000355302 | KRTAP1-5 | 1.727E-05 | -0.346 | - | - |
| 15525 | ENSP00000405210 | SPANXN4 | 3.696E-06 | -0.346 | - | - |
| 15526 | ENSP00000335616 | C9orf47 | 9.935E-06 | -0.346 | - | - |
| 15527 | ENSP00000359982 | ANKRD13C | 1.225E-05 | -0.346 | - | - |
| 15528 | ENSP00000351207 | KIAA1671 | 1.231E-05 | -0.346 | - | - |
| 15529 | ENSP00000360811 | LHX3 | 1.478E-05 | -0.346 | - | - |
| 15530 | ENSP00000360874 | IFIT1B | 9.382E-06 | -0.346 | - | - |
| 15531 | ENSP00000342118 | HHIPL2 | 1.100E-05 | -0.346 | - | - |
| 15532 | ENSP00000405290 | NPAS1 | 1.496E-05 | -0.346 | - | - |
| 15533 | ENSP00000366070 | RILPL1 | 5.875E-06 | -0.346 | - | - |
| 15534 | ENSP00000367746 | CLLU1 | 5.772E-06 | -0.346 | - | - |
| 15535 | ENSP00000328410 | MACC1 | 8.278E-06 | -0.346 | - | - |
| 15536 | ENSP00000383023 | MYL5 | 2.323E-05 | -0.346 | - | - |
| 15537 | ENSP00000359799 | DNAJB4 | 1.555E-05 | -0.346 | - | - |
| 15538 | ENSP00000378408 | GPT | 1.517E-05 | -0.346 | - | - |
| 15539 | ENSP00000345659 | CA7 | 1.808E-05 | -0.346 | - | - |
| 15540 | ENSP00000433282 | SLC22A18AS | 2.878E-06 | -0.346 | - | - |
| 15541 | ENSP00000335623 | VSIG10L | 1.488E-05 | -0.346 | - | - |
| 15542 | ENSP00000370781 | CDC20B | 1.473E-05 | -0.346 | - | - |
| 15543 | ENSP00000354193 | ABP1 | 1.188E-05 | -0.346 | - | - |
| 15544 | ENSP00000361965 | ADA | 1.271E-05 | -0.346 | - | - |
| 15545 | ENSP00000287748 | LYZL4 | 1.432E-05 | -0.346 | - | - |
| 15546 | ENSP00000464265 | UBBP4 | 4.938E-05 | -0.346 | - | - |
| 15547 | ENSP00000367770 | SLC35B4 | 6.300E-06 | -0.347 | - | - |
| 15548 | ENSP00000381102 | TMEM55B | 5.654E-06 | -0.347 | - | - |
| 15549 | ENSP00000406144 | TTC39A | 1.706E-06 | -0.347 | - | - |
| 15550 | ENSP00000356536 | TEDDM1 | 6.298E-06 | -0.347 | - | - |
| 15551 | ENSP00000304987 | BFSP2 | 1.840E-05 | -0.347 | - | - |
| 15552 | ENSP00000382101 | RP11-166B2.1 | 1.158E-05 | -0.347 | - | - |
| 15553 | ENSP00000443772 | RP11-87C12.2 | 6.021E-06 | -0.347 | - | - |
| 15554 | ENSP00000355518 | FH | 1.419E-05 | -0.347 | - | - |
| 15555 | ENSP00000362550 | FTSJD2 | 7.378E-06 | -0.347 | - | - |
| 15556 | ENSP00000355920 | SLC22A2 | 1.518E-05 | -0.347 | - | - |
| 15557 | ENSP00000347454 | ANO1 | 1.268E-05 | -0.347 | - | - |
| 15558 | ENSP00000343164 | MATN4 | 1.110E-05 | -0.347 | - | - |
| 15559 | ENSP00000449770 | PA2G4 | 1.990E-05 | -0.347 | - | - |
| 15560 | ENSP00000378965 | SNTB1 | 1.158E-05 | -0.347 | - | - |
| 15561 | ENSP00000368699 | ISG15 | 4.802E-05 | -0.347 | - | - |
| 15562 | ENSP00000363023 | DLGAP4 | 6.247E-06 | -0.347 | - | - |
| 15563 | ENSP00000307297 | CHST14 | 1.653E-05 | -0.347 | - | - |
| 15564 | ENSP00000293756 | IP6K3 | 2.521E-05 | -0.347 | - | - |
| 15565 | ENSP00000345895 | NUP50 | 1.229E-05 | -0.347 | - | - |
| 15566 | ENSP00000467041 | RP11-322E11.6 | 2.967E-06 | -0.347 | - | - |
| 15567 | ENSP00000366528 | TMEM252 | 3.460E-06 | -0.347 | - | - |
| 15568 | ENSP00000375073 | EID2 | 3.821E-06 | -0.347 | - | - |
| 15569 | ENSP00000393912 | COPG2 | 1.642E-05 | -0.348 | - | - |
| 15570 | ENSP00000393154 | FBXO15 | 1.464E-05 | -0.348 | - | - |
| 15571 | ENSP00000368565 | DNAJC2 | 9.364E-06 | -0.348 | - | - |
| 15572 | ENSP00000347635 | KRTAP13-1 | 7.637E-06 | -0.348 | - | - |
| 15573 | ENSP00000432163 | KBTBD3 | 3.696E-06 | -0.348 | - | - |
| 15574 | ENSP00000326570 | FKRP | 1.691E-05 | -0.348 | - | - |
| 15575 | ENSP00000349595 | ATP2A1 | 2.335E-05 | -0.348 | - | - |
| 15576 | ENSP00000263433 | PPP1R12C | 1.522E-05 | -0.348 | - | - |
| 15577 | ENSP00000374565 | FAM168B | 1.638E-05 | -0.348 | - | - |
| 15578 | ENSP00000333900 | OIT3 | 4.742E-06 | -0.348 | - | - |
| 15579 | ENSP00000424595 | PRPS1L1 | 2.377E-05 | -0.348 | - | - |
| 15580 | ENSP00000337478 | ARL17A | 1.074E-05 | -0.348 | - | - |
| 15581 | ENSP00000321309 | VCX2 | 4.447E-06 | -0.348 | - | - |
| 15582 | ENSP00000393631 | ADM5 | 7.021E-06 | -0.348 | - | - |
| 15583 | ENSP00000354883 | UBL7 | 5.980E-06 | -0.348 | - | - |
| 15584 | ENSP00000408730 | CCDC124 | 2.113E-06 | -0.348 | - | - |
| 15585 | ENSP00000381030 | STK32A | 2.310E-05 | -0.348 | - | - |
| 15586 | ENSP00000415032 | GTF2H5 | 7.035E-06 | -0.348 | - | - |
| 15587 | ENSP00000360323 | TTC22 | 6.566E-06 | -0.348 | - | - |
| 15588 | ENSP00000329312 | IGLL1 | 1.406E-05 | -0.348 | - | - |
| 15589 | ENSP00000182290 | TSPAN32 | 1.989E-05 | -0.348 | - | - |
| 15590 | ENSP00000370737 | GLDC | 2.145E-05 | -0.349 | - | - |
| 15591 | ENSP00000359115 | C20orf195 | 5.040E-06 | -0.349 | - | - |
| 15592 | ENSP00000392859 | GMPR2 | 1.629E-05 | -0.349 | - | - |
| 15593 | ENSP00000402634 | ELOVL7 | 1.520E-05 | -0.349 | - | - |
| 15594 | ENSP00000275200 | TAAR8 | 1.507E-05 | -0.349 | - | - |
| 15595 | ENSP00000359375 | FATE1 | 1.493E-05 | -0.349 | - | - |
| 15596 | ENSP00000358529 | TSPAN2 | 1.663E-05 | -0.349 | - | - |
| 15597 | ENSP00000351664 | MICAL1 | 1.692E-05 | -0.349 | - | - |
| 15598 | ENSP00000431987 | TRIM64C | 4.882E-06 | -0.349 | - | - |
| 15599 | ENSP00000296498 | PRSS12 | 1.913E-05 | -0.349 | - | - |
| 15600 | ENSP00000370254 | AKR1C1 | 1.618E-05 | -0.349 | - | - |
| 15601 | ENSP00000414068 | RP4-559A3.7 | 1.010E-05 | -0.349 | - | - |
| 15602 | ENSP00000348852 | FAM168A | 4.716E-06 | -0.349 | - | - |
| 15603 | ENSP00000378364 | SLC11A2 | 2.430E-05 | -0.349 | - | - |
| 15604 | ENSP00000356202 | MTRF1L | 8.746E-06 | -0.349 | - | - |
| 15605 | ENSP00000265334 | CDKL3 | 1.471E-05 | -0.349 | - | - |
| 15606 | ENSP00000450436 | ALDH6A1 | 1.708E-05 | -0.349 | - | - |
| 15607 | ENSP00000365944 | FBXO6 | 1.385E-05 | -0.349 | - | - |
| 15608 | ENSP00000363071 | DES | 1.282E-05 | -0.350 | - | - |
| 15609 | ENSP00000369862 | COL4A3BP | 1.540E-05 | -0.350 | - | - |
| 15610 | ENSP00000371054 | INSL6 | 5.506E-06 | -0.350 | - | - |
| 15611 | ENSP00000451235 | DCAF8 | 1.084E-05 | -0.350 | - | - |
| 15612 | ENSP00000333593 | NXPH4 | 1.511E-05 | -0.350 | - | - |
| 15613 | ENSP00000361726 | WFDC10A | 4.907E-06 | -0.350 | - | - |
| 15614 | ENSP00000324196 | AATK | 1.735E-05 | -0.350 | - | - |
| 15615 | ENSP00000318415 | RBMXL1 | 1.973E-05 | -0.350 | - | - |
| 15616 | ENSP00000418070 | PVRL3 | 1.619E-05 | -0.350 | - | - |
| 15617 | ENSP00000331302 | TCEB3B | 9.930E-06 | -0.350 | - | - |
| 15618 | ENSP00000317027 | GCNT4 | 8.270E-06 | -0.350 | - | - |
| 15619 | ENSP00000470383 | CTC-273B12.7 | 5.645E-06 | -0.350 | - | - |
| 15620 | ENSP00000378323 | PPP3CA | 3.447E-05 | -0.350 | - | - |
| 15621 | ENSP00000417214 | PARP15 | 1.343E-05 | -0.350 | - | - |
| 15622 | ENSP00000360992 | STAMBPL1 | 9.618E-06 | -0.350 | - | - |
| 15623 | ENSP00000431376 | TATDN3 | 6.584E-06 | -0.350 | - | - |
| 15624 | ENSP00000361032 | SURF2 | 7.135E-06 | -0.350 | - | - |
| 15625 | ENSP00000346901 | FMO1 | 1.648E-05 | -0.350 | - | - |
| 15626 | ENSP00000365530 | STXBP4 | 8.603E-06 | -0.350 | - | - |
| 15627 | ENSP00000360988 | LUZP4 | 2.537E-06 | -0.350 | - | - |
| 15628 | ENSP00000351937 | C17orf80 | 5.399E-06 | -0.350 | - | - |
| 15629 | ENSP00000330596 | C22orf29 | 8.428E-06 | -0.350 | - | - |
| 15630 | ENSP00000343140 | XIRP1 | 4.854E-06 | -0.350 | - | - |
| 15631 | ENSP00000371689 | CDH12 | 5.461E-06 | -0.350 | - | - |
| 15632 | ENSP00000384040 | CCDC85A | 4.606E-06 | -0.350 | - | - |
| 15633 | ENSP00000295682 | KRTCAP2 | 4.153E-06 | -0.350 | - | - |
| 15634 | ENSP00000357644 | GATAD2B | 5.852E-06 | -0.351 | - | - |
| 15635 | ENSP00000377577 | CCDC176 | 9.878E-07 | -0.351 | - | - |
| 15636 | ENSP00000323068 | TMEM168 | 1.671E-05 | -0.351 | - | - |
| 15637 | ENSP00000350352 | TRMT1 | 9.460E-06 | -0.351 | - | - |
| 15638 | ENSP00000417378 | ATP6V1F | 1.566E-05 | -0.351 | - | - |
| 15639 | ENSP00000361990 | UBR2 | 9.911E-06 | -0.351 | - | - |
| 15640 | ENSP00000434236 | - | 1.351E-05 | -0.351 | - | - |
| 15641 | ENSP00000355593 | DISC1 | 1.266E-05 | -0.351 | - | - |
| 15642 | ENSP00000407487 | UNC45A | 7.479E-06 | -0.351 | - | - |
| 15643 | ENSP00000367197 | SCGN | 1.666E-05 | -0.351 | - | - |
| 15644 | ENSP00000250156 | MRM1 | 1.996E-05 | -0.351 | - | - |
| 15645 | ENSP00000413697 | TOM1 | 1.739E-05 | -0.351 | - | - |
| 15646 | ENSP00000355374 | OSGIN1 | 1.327E-05 | -0.351 | - | - |
| 15647 | ENSP00000359645 | RBMX | 1.141E-05 | -0.351 | - | - |
| 15648 | ENSP00000320180 | GHRHR | 1.704E-05 | -0.351 | - | - |
| 15649 | ENSP00000357692 | S100A16 | 5.243E-06 | -0.351 | - | - |
| 15650 | ENSP00000359219 | KAZALD1 | 1.568E-05 | -0.351 | - | - |
| 15651 | ENSP00000360727 | RABL6 | 8.630E-06 | -0.351 | - | - |
| 15652 | ENSP00000355064 | SHISA4 | 3.542E-06 | -0.351 | - | - |
| 15653 | ENSP00000394487 | FGD4 | 1.105E-05 | -0.351 | - | - |
| 15654 | ENSP00000351933 | KLHL9 | 1.386E-05 | -0.351 | - | - |
| 15655 | ENSP00000408405 | KCTD1 | 7.409E-06 | -0.351 | - | - |
| 15656 | ENSP00000353769 | SCAF1 | 7.861E-06 | -0.351 | - | - |
| 15657 | ENSP00000329860 | CCDC172 | 4.012E-06 | -0.351 | - | - |
| 15658 | ENSP00000367189 | C11orf83 | 3.392E-06 | -0.351 | - | - |
| 15659 | ENSP00000354387 | NMRK1 | 6.455E-06 | -0.351 | - | - |
| 15660 | ENSP00000424261 | PTTG2 | 5.487E-06 | -0.351 | - | - |
| 15661 | ENSP00000414302 | SRSF12 | 3.164E-06 | -0.352 | - | - |
| 15662 | ENSP00000305151 | PWWP2A | 2.170E-05 | -0.352 | - | - |
| 15663 | ENSP00000447547 | MRPL42 | 2.618E-06 | -0.352 | - | - |
| 15664 | ENSP00000229583 | ADTRP | 1.451E-05 | -0.352 | - | - |
| 15665 | ENSP00000334708 | TMEM150A | 1.217E-05 | -0.352 | - | - |
| 15666 | ENSP00000381565 | DIAPH1 | 1.823E-05 | -0.352 | - | - |
| 15667 | ENSP00000360133 | ITGB3BP | 5.371E-06 | -0.352 | - | - |
| 15668 | ENSP00000349351 | BICD2 | 8.134E-06 | -0.352 | - | - |
| 15669 | ENSP00000307636 | MORN4 | 1.981E-05 | -0.352 | - | - |
| 15670 | ENSP00000327718 | MYADML2 | 4.476E-06 | -0.352 | - | - |
| 15671 | ENSP00000300873 | GNG8 | 1.999E-05 | -0.352 | - | - |
| 15672 | ENSP00000413520 | HID1 | 6.298E-06 | -0.352 | - | - |
| 15673 | ENSP00000332258 | DGAT1 | 2.022E-05 | -0.352 | - | - |
| 15674 | ENSP00000262916 | KCNQ4 | 1.640E-05 | -0.352 | - | - |
| 15675 | ENSP00000330426 | C1QTNF8 | 1.049E-05 | -0.352 | - | - |
| 15676 | ENSP00000311712 | RTP1 | 4.382E-06 | -0.352 | - | - |
| 15677 | ENSP00000280245 | CCDC83 | 3.368E-05 | -0.352 | - | - |
| 15678 | ENSP00000381148 | CTDSP2 | 1.685E-05 | -0.352 | - | - |
| 15679 | ENSP00000455469 | GJE1 | 6.759E-06 | -0.352 | - | - |
| 15680 | ENSP00000473036 | PNMAL2 | 2.504E-05 | -0.353 | - | - |
| 15681 | ENSP00000387100 | TPRN | 7.689E-06 | -0.353 | - | - |
| 15682 | ENSP00000365401 | CCDC22 | 1.096E-05 | -0.353 | - | - |
| 15683 | ENSP00000358211 | HSPA12A | 2.269E-05 | -0.353 | - | - |
| 15684 | ENSP00000365477 | CCDC93 | 1.354E-05 | -0.353 | - | - |
| 15685 | ENSP00000419542 | LRRC55 | 7.296E-06 | -0.353 | - | - |
| 15686 | ENSP00000353910 | FUT8 | 1.083E-05 | -0.353 | - | - |
| 15687 | ENSP00000350036 | SMC1B | 1.163E-05 | -0.353 | - | - |
| 15688 | ENSP00000253410 | HIGD1B | 1.363E-05 | -0.353 | - | - |
| 15689 | ENSP00000425634 | HARS | 1.463E-05 | -0.353 | - | - |
| 15690 | ENSP00000278520 | CCDC82 | 7.059E-06 | -0.353 | - | - |
| 15691 | ENSP00000420174 | ERVFRD-1 | 3.358E-06 | -0.353 | - | - |
| 15692 | ENSP00000352785 | DSG4 | 1.426E-05 | -0.353 | - | - |
| 15693 | ENSP00000319336 | FAM216B | 2.304E-06 | -0.353 | - | - |
| 15694 | ENSP00000355752 | C1orf95 | 8.477E-06 | -0.353 | - | - |
| 15695 | ENSP00000377558 | KRT35 | 8.675E-06 | -0.353 | - | - |
| 15696 | ENSP00000367911 | SYCE1L | 1.735E-06 | -0.353 | - | - |
| 15697 | ENSP00000370938 | CDK8 | 1.761E-05 | -0.353 | - | - |
| 15698 | ENSP00000356905 | VNN1 | 1.477E-05 | -0.353 | - | - |
| 15699 | ENSP00000373363 | GOLM1 | 8.274E-06 | -0.353 | - | - |
| 15700 | ENSP00000373674 | NT5M | 1.324E-05 | -0.353 | - | - |
| 15701 | ENSP00000215061 | OCEL1 | 1.357E-05 | -0.353 | - | - |
| 15702 | ENSP00000297873 | WBSCR27 | 6.619E-06 | -0.354 | - | - |
| 15703 | ENSP00000329292 | GKN2 | 1.274E-05 | -0.354 | - | - |
| 15704 | ENSP00000297107 | GALNT10 | 6.048E-06 | -0.354 | - | - |
| 15705 | ENSP00000347486 | MORN3 | 1.557E-05 | -0.354 | - | - |
| 15706 | ENSP00000471312 | AC011530.4 | 3.029E-06 | -0.354 | - | - |
| 15707 | ENSP00000456832 | NDUFB8 | 6.473E-06 | -0.354 | - | - |
| 15708 | ENSP00000264771 | TMEM175 | 8.612E-06 | -0.354 | - | - |
| 15709 | ENSP00000347538 | SYT17 | 1.132E-05 | -0.354 | - | - |
| 15710 | ENSP00000307722 | NSG2 | 1.787E-05 | -0.354 | - | - |
| 15711 | ENSP00000454374 | RP11-468E2.2 | 2.408E-06 | -0.354 | - | - |
| 15712 | ENSP00000314992 | RRH | 1.613E-05 | -0.354 | - | - |
| 15713 | ENSP00000311436 | ABRA | 1.299E-05 | -0.354 | - | - |
| 15714 | ENSP00000350249 | POT1 | 1.892E-05 | -0.354 | - | - |
| 15715 | ENSP00000368790 | MTRF1 | 8.480E-06 | -0.354 | - | - |
| 15716 | ENSP00000383456 | C9orf152 | 5.527E-06 | -0.354 | - | - |
| 15717 | ENSP00000370748 | IDI1 | 1.090E-05 | -0.354 | - | - |
| 15718 | ENSP00000303092 | CACNG5 | 1.919E-05 | -0.354 | - | - |
| 15719 | ENSP00000454565 | C17orf103 | 6.431E-06 | -0.354 | - | - |
| 15720 | ENSP00000376188 | CRLF1 | 1.458E-05 | -0.354 | - | - |
| 15721 | ENSP00000321724 | INSL3 | 1.070E-05 | -0.355 | - | - |
| 15722 | ENSP00000380779 | TCTN1 | 4.582E-06 | -0.355 | - | - |
| 15723 | ENSP00000369268 | CNGA4 | 1.407E-05 | -0.355 | - | - |
| 15724 | ENSP00000341360 | COBLL1 | 6.266E-06 | -0.355 | - | - |
| 15725 | ENSP00000284881 | C21orf91 | 1.974E-05 | -0.355 | - | - |
| 15726 | ENSP00000353375 | MAP4 | 1.111E-05 | -0.355 | - | - |
| 15727 | ENSP00000332404 | TMIGD1 | 6.338E-06 | -0.355 | - | - |
| 15728 | ENSP00000347626 | NUDCD3 | 8.558E-06 | -0.355 | - | - |
| 15729 | ENSP00000379951 | LEPREL2 | 2.843E-06 | -0.355 | - | - |
| 15730 | ENSP00000365908 | PRUNE2 | 1.088E-05 | -0.355 | - | - |
| 15731 | ENSP00000343318 | B3GALT5 | 8.513E-06 | -0.355 | - | - |
| 15732 | ENSP00000380336 | SMU1 | 1.383E-05 | -0.355 | - | - |
| 15733 | ENSP00000403438 | TMEM150C | 3.734E-06 | -0.355 | - | - |
| 15734 | ENSP00000360031 | RRP12 | 5.979E-06 | -0.355 | - | - |
| 15735 | ENSP00000363338 | UBXN11 | 2.117E-05 | -0.355 | - | - |
| 15736 | ENSP00000370949 | NFXL1 | 6.244E-06 | -0.355 | - | - |
| 15737 | ENSP00000341021 | NANOS2 | 1.784E-05 | -0.355 | - | - |
| 15738 | ENSP00000396527 | AC068533.7 | 5.179E-06 | -0.355 | - | - |
| 15739 | ENSP00000365388 | PDSS1 | 1.343E-05 | -0.355 | - | - |
| 15740 | ENSP00000431748 | PCDHA2 | 4.002E-06 | -0.355 | - | - |
| 15741 | ENSP00000278949 | MPZL3 | 1.214E-05 | -0.355 | - | - |
| 15742 | ENSP00000313890 | RBM15B | 1.712E-05 | -0.355 | - | - |
| 15743 | ENSP00000440190 | TDRD3 | 7.857E-06 | -0.356 | - | - |
| 15744 | ENSP00000357219 | TTC24 | 7.587E-06 | -0.356 | - | - |
| 15745 | ENSP00000408342 | PRAM1 | 1.468E-05 | -0.356 | - | - |
| 15746 | ENSP00000362546 | TTI1 | 9.100E-06 | -0.356 | - | - |
| 15747 | ENSP00000386772 | GPATCH11 | 7.621E-06 | -0.356 | - | - |
| 15748 | ENSP00000425955 | USP17L5 | 6.097E-06 | -0.356 | - | - |
| 15749 | ENSP00000422473 | TMEM33 | 1.363E-05 | -0.356 | - | - |
| 15750 | ENSP00000416829 | OARD1 | 9.247E-06 | -0.356 | - | - |
| 15751 | ENSP00000468098 | - | 1.981E-06 | -0.356 | - | - |
| 15752 | ENSP00000376309 | HNRNPA3 | 2.324E-05 | -0.356 | - | - |
| 15753 | ENSP00000330051 | MFSD6L | 1.940E-05 | -0.356 | - | - |
| 15754 | ENSP00000380635 | ARL16 | 1.148E-05 | -0.356 | - | - |
| 15755 | ENSP00000340434 | LDOC1L | 1.670E-05 | -0.356 | - | - |
| 15756 | ENSP00000358265 | KLHL32 | 1.076E-05 | -0.356 | - | - |
| 15757 | ENSP00000408108 | WDR26 | 5.380E-06 | -0.356 | - | - |
| 15758 | ENSP00000384474 | HYPK | 1.172E-05 | -0.356 | - | - |
| 15759 | ENSP00000380531 | MRPS34 | 3.610E-06 | -0.356 | - | - |
| 15760 | ENSP00000320886 | MLXIPL | 1.686E-05 | -0.356 | - | - |
| 15761 | ENSP00000368459 | NUFIP1 | 7.800E-06 | -0.357 | - | - |
| 15762 | ENSP00000415434 | USP40 | 1.592E-05 | -0.357 | - | - |
| 15763 | ENSP00000360690 | LONRF3 | 2.155E-05 | -0.357 | - | - |
| 15764 | ENSP00000382485 | PROL1 | 7.384E-06 | -0.357 | - | - |
| 15765 | ENSP00000463645 | MYO15B | 9.628E-06 | -0.357 | - | - |
| 15766 | ENSP00000381220 | HDDC2 | 3.061E-06 | -0.357 | - | - |
| 15767 | ENSP00000346027 | RPL21 | 1.268E-05 | -0.357 | - | - |
| 15768 | ENSP00000352219 | BCS1L | 1.417E-05 | -0.357 | - | - |
| 15769 | ENSP00000359691 | LGSN | 1.179E-05 | -0.357 | - | - |
| 15770 | ENSP00000381184 | TMEM231 | 4.347E-06 | -0.357 | - | - |
| 15771 | ENSP00000366936 | SHB | 1.636E-05 | -0.357 | - | - |
| 15772 | ENSP00000365255 | ANKRD26 | 8.808E-06 | -0.357 | - | - |
| 15773 | ENSP00000258704 | SPDYE1 | 1.426E-05 | -0.357 | - | - |
| 15774 | ENSP00000424077 | USP17L23 | 5.964E-06 | -0.357 | - | - |
| 15775 | ENSP00000375428 | KRTAP3-3 | 1.310E-06 | -0.357 | - | - |
| 15776 | ENSP00000325775 | GJC3 | 1.266E-05 | -0.357 | - | - |
| 15777 | ENSP00000421364 | ECSCR | 5.628E-06 | -0.357 | - | - |
| 15778 | ENSP00000455908 | SOGA3 | 3.456E-06 | -0.357 | - | - |
| 15779 | ENSP00000457016 | INO80E | 1.425E-05 | -0.357 | - | - |
| 15780 | ENSP00000327832 | ATAT1 | 1.327E-05 | -0.357 | - | - |
| 15781 | ENSP00000431822 | FAU | 5.078E-05 | -0.357 | - | - |
| 15782 | ENSP00000367794 | SRPX | 6.416E-06 | -0.358 | - | - |
| 15783 | ENSP00000355385 | R3HDM4 | 6.072E-06 | -0.358 | - | - |
| 15784 | ENSP00000364665 | SSX2B | 9.699E-06 | -0.358 | - | - |
| 15785 | ENSP00000361084 | MRPL14 | 3.409E-06 | -0.358 | - | - |
| 15786 | ENSP00000322460 | SCN4B | 1.793E-05 | -0.358 | - | - |
| 15787 | ENSP00000351259 | FAM21B | 4.367E-06 | -0.358 | - | - |
| 15788 | ENSP00000366673 | CLN5 | 1.279E-05 | -0.358 | - | - |
| 15789 | ENSP00000331086 | ODF4 | 7.224E-06 | -0.358 | - | - |
| 15790 | ENSP00000355560 | TBCE | 1.027E-05 | -0.358 | - | - |
| 15791 | ENSP00000386878 | WIPF3 | 1.536E-05 | -0.358 | - | - |
| 15792 | ENSP00000379026 | FBXW10 | 6.177E-06 | -0.358 | - | - |
| 15793 | ENSP00000361336 | CDH22 | 1.281E-05 | -0.358 | - | - |
| 15794 | ENSP00000365152 | MYLK2 | 1.206E-05 | -0.358 | - | - |
| 15795 | ENSP00000356992 | ARHGAP30 | 1.629E-05 | -0.358 | - | - |
| 15796 | ENSP00000451484 | RP5-1021I20.4 | 4.256E-06 | -0.358 | - | - |
| 15797 | ENSP00000329647 | ACTL10 | 8.641E-06 | -0.358 | - | - |
| 15798 | ENSP00000333212 | CYP2U1 | 1.132E-05 | -0.358 | - | - |
| 15799 | ENSP00000359034 | FNDC7 | 3.578E-06 | -0.358 | - | - |
| 15800 | ENSP00000300406 | GNGT2 | 1.801E-05 | -0.358 | - | - |
| 15801 | ENSP00000470240 | CTD-2192J16.22 | 4.098E-06 | -0.358 | - | - |
| 15802 | ENSP00000461388 | OVCA2 | 7.083E-06 | -0.358 | - | - |
| 15803 | ENSP00000369921 | ADAMTSL1 | 1.970E-05 | -0.358 | - | - |
| 15804 | ENSP00000303928 | KIAA0232 | 7.170E-06 | -0.359 | - | - |
| 15805 | ENSP00000345156 | RPL14 | 1.295E-05 | -0.359 | - | - |
| 15806 | ENSP00000264661 | KCNH4 | 2.093E-05 | -0.359 | - | - |
| 15807 | ENSP00000337757 | PIH1D3 | 1.696E-05 | -0.359 | - | - |
| 15808 | ENSP00000368887 | PAK1IP1 | 6.357E-06 | -0.359 | - | - |
| 15809 | ENSP00000354739 | RPL12 | 1.064E-05 | -0.359 | - | - |
| 15810 | ENSP00000414624 | PRPSAP1 | 1.953E-05 | -0.359 | - | - |
| 15811 | ENSP00000361042 | SURF1 | 6.399E-06 | -0.359 | - | - |
| 15812 | ENSP00000386741 | CHN1 | 1.669E-05 | -0.359 | - | - |
| 15813 | ENSP00000296387 | CLDN19 | 1.221E-05 | -0.359 | - | - |
| 15814 | ENSP00000347161 | TSGA10 | 1.063E-05 | -0.359 | - | - |
| 15815 | ENSP00000452959 | ZMYND11 | 1.165E-05 | -0.359 | - | - |
| 15816 | ENSP00000261854 | SPPL2A | 1.907E-05 | -0.359 | - | - |
| 15817 | ENSP00000370526 | ARSE | 1.413E-05 | -0.359 | - | - |
| 15818 | ENSP00000364742 | FBXO42 | 7.399E-06 | -0.359 | - | - |
| 15819 | ENSP00000366620 | H6PD | 2.100E-05 | -0.359 | - | - |
| 15820 | ENSP00000392553 | PPAPDC1B | 1.154E-05 | -0.359 | - | - |
| 15821 | ENSP00000349325 | ATP10A | 1.565E-05 | -0.359 | - | - |
| 15822 | ENSP00000253807 | PCDHAC1 | 2.333E-05 | -0.359 | - | - |
| 15823 | ENSP00000423503 | USP17L18 | 6.062E-06 | -0.360 | - | - |
| 15824 | ENSP00000428780 | CLDN23 | 3.448E-06 | -0.360 | - | - |
| 15825 | ENSP00000451812 | LIN52 | 1.424E-05 | -0.360 | - | - |
| 15826 | ENSP00000464383 | RP11-599B13.6 | 1.868E-05 | -0.360 | - | - |
| 15827 | ENSP00000386738 | KCMF1 | 4.504E-06 | -0.360 | - | - |
| 15828 | ENSP00000355890 | EPRS | 2.803E-05 | -0.360 | - | - |
| 15829 | ENSP00000415026 | PRRT4 | 7.648E-06 | -0.360 | - | - |
| 15830 | ENSP00000260502 | BCAR3 | 1.641E-05 | -0.360 | - | - |
| 15831 | ENSP00000362186 | PCDH11X | 2.086E-05 | -0.360 | - | - |
| 15832 | ENSP00000360689 | TNKS2 | 1.571E-05 | -0.360 | - | - |
| 15833 | ENSP00000295440 | NPPC | 1.616E-05 | -0.360 | - | - |
| 15834 | ENSP00000386226 | RBM34 | 9.425E-06 | -0.360 | - | - |
| 15835 | ENSP00000420716 | C1orf228 | 3.201E-06 | -0.360 | - | - |
| 15836 | ENSP00000378326 | ZP3 | 9.941E-06 | -0.360 | - | - |
| 15837 | ENSP00000371085 | BAIAP2L2 | 3.328E-06 | -0.360 | - | - |
| 15838 | ENSP00000383599 | WDR47 | 1.648E-05 | -0.360 | - | - |
| 15839 | ENSP00000419975 | PEX5L | 9.880E-06 | -0.360 | - | - |
| 15840 | ENSP00000392995 | RNF103-CHMP3 | 1.487E-05 | -0.360 | - | - |
| 15841 | ENSP00000455068 | METRN | 2.783E-06 | -0.360 | - | - |
| 15842 | ENSP00000375057 | ARL4C | 1.446E-05 | -0.360 | - | - |
| 15843 | ENSP00000351379 | PFN3 | 1.496E-05 | -0.360 | - | - |
| 15844 | ENSP00000462333 | ENGASE | 2.717E-06 | -0.360 | - | - |
| 15845 | ENSP00000391668 | MTMR11 | 1.273E-05 | -0.360 | - | - |
| 15846 | ENSP00000262765 | QRICH2 | 1.422E-05 | -0.360 | - | - |
| 15847 | ENSP00000362330 | CHD6 | 1.663E-05 | -0.360 | - | - |
| 15848 | ENSP00000456609 | STRA6 | 5.236E-06 | -0.360 | - | - |
| 15849 | ENSP00000261584 | PALB2 | 1.475E-05 | -0.360 | - | - |
| 15850 | ENSP00000411084 | ZNF799 | 9.420E-06 | -0.360 | - | - |
| 15851 | ENSP00000316237 | CCDC102B | 1.863E-05 | -0.361 | - | - |
| 15852 | ENSP00000379434 | KLHL28 | 8.247E-06 | -0.361 | - | - |
| 15853 | ENSP00000233826 | KCNJ13 | 1.980E-05 | -0.361 | - | - |
| 15854 | ENSP00000299721 | CPLX4 | 1.696E-05 | -0.361 | - | - |
| 15855 | ENSP00000373509 | C11orf31 | 7.073E-06 | -0.361 | - | - |
| 15856 | ENSP00000312099 | CRYGS | 1.485E-05 | -0.361 | - | - |
| 15857 | ENSP00000411418 | RAB3GAP1 | 1.612E-05 | -0.361 | - | - |
| 15858 | ENSP00000414786 | TMEM64 | 3.679E-06 | -0.361 | - | - |
| 15859 | ENSP00000324920 | C14orf39 | 1.893E-05 | -0.361 | - | - |
| 15860 | ENSP00000261862 | THSD4 | 1.878E-05 | -0.361 | - | - |
| 15861 | ENSP00000367815 | DNAAF1 | 8.761E-06 | -0.361 | - | - |
| 15862 | ENSP00000370196 | SBF1 | 8.559E-06 | -0.361 | - | - |
| 15863 | ENSP00000348429 | ACSL5 | 1.547E-05 | -0.361 | - | - |
| 15864 | ENSP00000434797 | RNF170 | 2.197E-05 | -0.361 | - | - |
| 15865 | ENSP00000360777 | INPP5E | 8.861E-06 | -0.361 | - | - |
| 15866 | ENSP00000390722 | SLC25A17 | 4.045E-06 | -0.361 | - | - |
| 15867 | ENSP00000356405 | CDC73 | 9.595E-06 | -0.361 | - | - |
| 15868 | ENSP00000462972 | FDXR | 2.090E-05 | -0.361 | - | - |
| 15869 | ENSP00000307741 | CMTM8 | 1.701E-05 | -0.361 | - | - |
| 15870 | ENSP00000370671 | KLHL42 | 1.206E-05 | -0.361 | - | - |
| 15871 | ENSP00000418741 | CNTNAP4 | 1.400E-05 | -0.362 | - | - |
| 15872 | ENSP00000357775 | UROS | 6.924E-06 | -0.362 | - | - |
| 15873 | ENSP00000360021 | GNG12 | 1.117E-05 | -0.362 | - | - |
| 15874 | ENSP00000382178 | MDGA2 | 6.740E-06 | -0.362 | - | - |
| 15875 | ENSP00000335392 | SPATA12 | 8.183E-06 | -0.362 | - | - |
| 15876 | ENSP00000389465 | RPL17-C18orf32 | 4.255E-06 | -0.362 | - | - |
| 15877 | ENSP00000388299 | TRIM49C | 5.293E-06 | -0.362 | - | - |
| 15878 | ENSP00000355211 | KIAA1704 | 1.273E-05 | -0.362 | - | - |
| 15879 | ENSP00000316670 | DHRS9 | 1.355E-05 | -0.362 | - | - |
| 15880 | ENSP00000222275 | UPK1A | 1.399E-05 | -0.362 | - | - |
| 15881 | ENSP00000379242 | CDRT1 | 3.314E-06 | -0.362 | - | - |
| 15882 | ENSP00000370200 | SNAPC3 | 6.407E-06 | -0.362 | - | - |
| 15883 | ENSP00000369131 | SIN3B | 1.295E-05 | -0.362 | - | - |
| 15884 | ENSP00000464890 | POU5F2 | 1.353E-05 | -0.362 | - | - |
| 15885 | ENSP00000299578 | C16orf46 | 5.504E-06 | -0.362 | - | - |
| 15886 | ENSP00000378958 | RABL2B | 1.634E-05 | -0.362 | - | - |
| 15887 | ENSP00000427264 | USP17L20 | 6.062E-06 | -0.362 | - | - |
| 15888 | ENSP00000360773 | SLC29A1 | 1.369E-05 | -0.362 | - | - |
| 15889 | ENSP00000348704 | KPNA5 | 1.375E-05 | -0.363 | - | - |
| 15890 | ENSP00000362135 | YRDC | 1.497E-05 | -0.363 | - | - |
| 15891 | ENSP00000324122 | PRPF31 | 1.081E-05 | -0.363 | - | - |
| 15892 | ENSP00000362187 | FAM102A | 1.587E-05 | -0.363 | - | - |
| 15893 | ENSP00000355800 | TMEM63A | 6.230E-06 | -0.363 | - | - |
| 15894 | ENSP00000367323 | KCNAB2 | 1.747E-05 | -0.363 | - | - |
| 15895 | ENSP00000356925 | FCRLB | 1.344E-05 | -0.363 | - | - |
| 15896 | ENSP00000367766 | RPGR | 1.227E-05 | -0.363 | - | - |
| 15897 | ENSP00000431162 | EID1 | 3.671E-06 | -0.363 | - | - |
| 15898 | ENSP00000305899 | SUV420H1 | 1.539E-05 | -0.363 | - | - |
| 15899 | ENSP00000402038 | MTO1 | 7.937E-06 | -0.363 | - | - |
| 15900 | ENSP00000359529 | SPANXN2 | 3.869E-06 | -0.363 | - | - |
| 15901 | ENSP00000431482 | NEDD8-MDP1 | 1.072E-05 | -0.363 | - | - |
| 15902 | ENSP00000429865 | TVP23C-CDRT4 | 3.826E-06 | -0.363 | - | - |
| 15903 | ENSP00000425582 | USP17L19 | 6.062E-06 | -0.363 | - | - |
| 15904 | ENSP00000345667 | ILDR1 | 1.302E-05 | -0.363 | - | - |
| 15905 | ENSP00000320346 | ELL3 | 1.506E-05 | -0.363 | - | - |
| 15906 | ENSP00000336842 | ACTR8 | 8.712E-06 | -0.363 | - | - |
| 15907 | ENSP00000422216 | USP17L21 | 6.069E-06 | -0.363 | - | - |
| 15908 | ENSP00000333037 | ACTRT3 | 1.131E-05 | -0.363 | - | - |
| 15909 | ENSP00000417132 | BAP1 | 1.364E-05 | -0.363 | - | - |
| 15910 | ENSP00000370589 | NOP56 | 9.361E-06 | -0.363 | - | - |
| 15911 | ENSP00000345420 | ASCL4 | 1.106E-05 | -0.364 | - | - |
| 15912 | ENSP00000313318 | SLC35C1 | 1.673E-05 | -0.364 | - | - |
| 15913 | ENSP00000356298 | TMEM9 | 1.629E-05 | -0.364 | - | - |
| 15914 | ENSP00000357106 | CADM3 | 1.375E-05 | -0.364 | - | - |
| 15915 | ENSP00000362386 | TTC16 | 7.838E-06 | -0.364 | - | - |
| 15916 | ENSP00000419449 | EIF1 | 1.693E-05 | -0.364 | - | - |
| 15917 | ENSP00000422621 | USP17L17 | 6.069E-06 | -0.364 | - | - |
| 15918 | ENSP00000357461 | CHRNB2 | 1.775E-05 | -0.364 | - | - |
| 15919 | ENSP00000368165 | SDHAF1 | 6.240E-06 | -0.364 | - | - |
| 15920 | ENSP00000363349 | SNX30 | 1.369E-05 | -0.364 | - | - |
| 15921 | ENSP00000403760 | USP17L10 | 6.147E-06 | -0.364 | - | - |
| 15922 | ENSP00000349364 | IFFO1 | 1.243E-05 | -0.364 | - | - |
| 15923 | ENSP00000360195 | KANK4 | 1.203E-05 | -0.364 | - | - |
| 15924 | ENSP00000420176 | POLE4 | 1.562E-05 | -0.364 | - | - |
| 15925 | ENSP00000354468 | CYB5R3 | 8.390E-06 | -0.364 | - | - |
| 15926 | ENSP00000272521 | TMEM177 | 5.613E-06 | -0.364 | - | - |
| 15927 | ENSP00000362469 | SLC2A8 | 1.138E-05 | -0.364 | - | - |
| 15928 | ENSP00000454303 | AP5B1 | 8.782E-06 | -0.364 | - | - |
| 15929 | ENSP00000402935 | TBC1D5 | 2.140E-05 | -0.364 | - | - |
| 15930 | ENSP00000337133 | SERPINA9 | 1.676E-05 | -0.364 | - | - |
| 15931 | ENSP00000262487 | ISM1 | 1.566E-05 | -0.364 | - | - |
| 15932 | ENSP00000457258 | OGFOD1 | 8.577E-06 | -0.364 | - | - |
| 15933 | ENSP00000344729 | FAM166A | 4.638E-06 | -0.364 | - | - |
| 15934 | ENSP00000388723 | KBTBD13 | 6.476E-06 | -0.364 | - | - |
| 15935 | ENSP00000298298 | C10orf25 | 1.471E-05 | -0.365 | - | - |
| 15936 | ENSP00000357547 | NUP210L | 1.335E-05 | -0.365 | - | - |
| 15937 | ENSP00000414390 | C11orf70 | 1.479E-05 | -0.365 | - | - |
| 15938 | ENSP00000303148 | TMEM37 | 9.127E-06 | -0.365 | - | - |
| 15939 | ENSP00000353072 | ATP2A3 | 2.065E-05 | -0.365 | - | - |
| 15940 | ENSP00000394869 | EIF2B4 | 4.984E-06 | -0.365 | - | - |
| 15941 | ENSP00000348010 | PRPF39 | 8.264E-06 | -0.365 | - | - |
| 15942 | ENSP00000455329 | CRIP1 | 1.048E-05 | -0.365 | - | - |
| 15943 | ENSP00000355541 | HEATR1 | 7.056E-06 | -0.365 | - | - |
| 15944 | ENSP00000355932 | VASH2 | 5.540E-06 | -0.365 | - | - |
| 15945 | ENSP00000403302 | ANKRD36C | 1.733E-05 | -0.365 | - | - |
| 15946 | ENSP00000345633 | ZCCHC13 | 1.464E-05 | -0.365 | - | - |
| 15947 | ENSP00000370718 | ASMTL | 2.193E-05 | -0.365 | - | - |
| 15948 | ENSP00000368720 | PLEKHN1 | 4.556E-06 | -0.365 | - | - |
| 15949 | ENSP00000472249 | GMFG | 1.027E-05 | -0.365 | - | - |
| 15950 | ENSP00000351813 | 5-Mar | 1.071E-05 | -0.365 | - | - |
| 15951 | ENSP00000355492 | C1orf101 | 4.806E-06 | -0.365 | - | - |
| 15952 | ENSP00000420132 | PLSCR2 | 8.089E-06 | -0.365 | - | - |
| 15953 | ENSP00000366984 | KRT40 | 5.352E-06 | -0.366 | - | - |
| 15954 | ENSP00000253792 | ACLY | 6.611E-06 | -0.366 | - | - |
| 15955 | ENSP00000356113 | SLC45A3 | 9.825E-06 | -0.366 | - | - |
| 15956 | ENSP00000357341 | NCOA7 | 6.048E-06 | -0.366 | - | - |
| 15957 | ENSP00000353677 | SLC17A2 | 7.529E-06 | -0.366 | - | - |
| 15958 | ENSP00000360753 | LCN8 | 7.819E-06 | -0.366 | - | - |
| 15959 | ENSP00000356807 | TIPRL | 1.554E-05 | -0.366 | - | - |
| 15960 | ENSP00000354856 | CLK2 | 9.477E-06 | -0.366 | - | - |
| 15961 | ENSP00000454926 | C16orf52 | 2.764E-06 | -0.366 | - | - |
| 15962 | ENSP00000240079 | CCDC53 | 1.268E-05 | -0.366 | - | - |
| 15963 | ENSP00000405932 | PROX2 | 1.118E-05 | -0.366 | - | - |
| 15964 | ENSP00000345773 | BEND7 | 5.254E-06 | -0.366 | - | - |
| 15965 | ENSP00000423115 | USP17L22 | 6.062E-06 | -0.366 | - | - |
| 15966 | ENSP00000368981 | SLC35B3 | 4.289E-06 | -0.366 | - | - |
| 15967 | ENSP00000370105 | GTF2H2C | 1.079E-05 | -0.367 | - | - |
| 15968 | ENSP00000356001 | MRPL18 | 9.524E-06 | -0.367 | - | - |
| 15969 | ENSP00000315357 | ARL6IP6 | 5.106E-06 | -0.367 | - | - |
| 15970 | ENSP00000310149 | CYP2W1 | 1.084E-05 | -0.367 | - | - |
| 15971 | ENSP00000361254 | FAM213A | 8.936E-06 | -0.367 | - | - |
| 15972 | ENSP00000461518 | TRPV3 | 9.212E-06 | -0.367 | - | - |
| 15973 | ENSP00000382157 | C14orf182 | 1.662E-06 | -0.367 | - | - |
| 15974 | ENSP00000346890 | NRD1 | 1.224E-05 | -0.367 | - | - |
| 15975 | ENSP00000328336 | CEND1 | 1.220E-05 | -0.367 | - | - |
| 15976 | ENSP00000310547 | ADCK5 | 8.282E-06 | -0.367 | - | - |
| 15977 | ENSP00000348205 | ATP5G1 | 7.807E-06 | -0.367 | - | - |
| 15978 | ENSP00000376307 | C14orf80 | 5.350E-06 | -0.367 | - | - |
| 15979 | ENSP00000370736 | TMEM165 | 1.026E-05 | -0.367 | - | - |
| 15980 | ENSP00000450995 | PTCD1 | 4.773E-06 | -0.367 | - | - |
| 15981 | ENSP00000453793 | MEIS2 | 2.137E-05 | -0.367 | - | - |
| 15982 | ENSP00000427336 | DIO3 | 6.166E-06 | -0.367 | - | - |
| 15983 | ENSP00000355721 | SNAP47 | 9.783E-06 | -0.367 | - | - |
| 15984 | ENSP00000384084 | DTNB | 7.991E-06 | -0.367 | - | - |
| 15985 | ENSP00000470609 | NDUFA7 | 1.112E-05 | -0.367 | - | - |
| 15986 | ENSP00000362932 | RAB42 | 1.448E-05 | -0.367 | - | - |
| 15987 | ENSP00000413001 | AGBL1 | 4.994E-06 | -0.367 | - | - |
| 15988 | ENSP00000469468 | LSM4 | 1.151E-05 | -0.367 | - | - |
| 15989 | ENSP00000363313 | PRPF4 | 1.098E-05 | -0.367 | - | - |
| 15990 | ENSP00000369438 | KRTAP10-10 | 1.149E-06 | -0.367 | - | - |
| 15991 | ENSP00000406751 | CRY2 | 1.783E-05 | -0.367 | - | - |
| 15992 | ENSP00000345767 | ANKRD6 | 1.652E-05 | -0.368 | - | - |
| 15993 | ENSP00000311760 | TTC9B | 6.631E-06 | -0.368 | - | - |
| 15994 | ENSP00000355354 | SOX15 | 1.482E-05 | -0.368 | - | - |
| 15995 | ENSP00000379086 | ADM2 | 1.629E-05 | -0.368 | - | - |
| 15996 | ENSP00000355827 | FBXO28 | 8.244E-06 | -0.368 | - | - |
| 15997 | ENSP00000380702 | MYCBP | 1.063E-05 | -0.368 | - | - |
| 15998 | ENSP00000454591 | FAM188B2 | 2.570E-06 | -0.368 | - | - |
| 15999 | ENSP00000413049 | SLC25A48 | 5.538E-06 | -0.368 | - | - |
| 16000 | ENSP00000355840 | C1orf65 | 3.475E-06 | -0.368 | - | - |
| 16001 | ENSP00000350789 | SFMBT1 | 1.548E-05 | -0.368 | - | - |
| 16002 | ENSP00000357292 | UBQLN4 | 1.782E-05 | -0.368 | - | - |
| 16003 | ENSP00000360299 | GPR110 | 1.139E-05 | -0.368 | - | - |
| 16004 | ENSP00000375475 | KRTAP12-1 | 2.280E-06 | -0.368 | - | - |
| 16005 | ENSP00000423897 | KLHL5 | 7.987E-06 | -0.368 | - | - |
| 16006 | ENSP00000455307 | CHD9 | 1.729E-05 | -0.368 | - | - |
| 16007 | ENSP00000352510 | TECPR2 | 7.154E-06 | -0.368 | - | - |
| 16008 | ENSP00000377192 | G6PD | 9.433E-06 | -0.368 | - | - |
| 16009 | ENSP00000386443 | TGOLN2 | 1.502E-05 | -0.368 | - | - |
| 16010 | ENSP00000358798 | CALHM3 | 1.027E-05 | -0.368 | - | - |
| 16011 | ENSP00000284776 | SORBS2 | 1.284E-05 | -0.368 | - | - |
| 16012 | ENSP00000358105 | APH1A | 1.243E-05 | -0.368 | - | - |
| 16013 | ENSP00000339917 | TAF9B | 1.323E-05 | -0.368 | - | - |
| 16014 | ENSP00000359859 | GTPBP5 | 1.141E-05 | -0.368 | - | - |
| 16015 | ENSP00000334922 | KRTAP9-4 | 2.916E-06 | -0.369 | - | - |
| 16016 | ENSP00000372445 | METTL17 | 4.444E-06 | -0.369 | - | - |
| 16017 | ENSP00000355961 | INTS7 | 1.294E-05 | -0.369 | - | - |
| 16018 | ENSP00000380692 | JHDM1D | 1.634E-05 | -0.369 | - | - |
| 16019 | ENSP00000365413 | SLC28A3 | 1.131E-05 | -0.369 | - | - |
| 16020 | ENSP00000398502 | FAM86A | 5.789E-06 | -0.369 | - | - |
| 16021 | ENSP00000443824 | ZNRF3 | 1.884E-05 | -0.369 | - | - |
| 16022 | ENSP00000347778 | SLC26A8 | 6.764E-06 | -0.369 | - | - |
| 16023 | ENSP00000227918 | SCGB2A2 | 1.643E-05 | -0.369 | - | - |
| 16024 | ENSP00000357748 | BCCIP | 5.188E-06 | -0.369 | - | - |
| 16025 | ENSP00000349493 | GUCA2A | 6.868E-06 | -0.369 | - | - |
| 16026 | ENSP00000373810 | NOP16 | 4.097E-06 | -0.369 | - | - |
| 16027 | ENSP00000406026 | SH2D5 | 8.330E-06 | -0.369 | - | - |
| 16028 | ENSP00000426174 | CCDC169 | 1.101E-05 | -0.369 | - | - |
| 16029 | ENSP00000429168 | SLC25A30 | 4.302E-06 | -0.369 | - | - |
| 16030 | ENSP00000358424 | HSD3B2 | 1.234E-05 | -0.369 | - | - |
| 16031 | ENSP00000424400 | SPATS1 | 5.007E-06 | -0.369 | - | - |
| 16032 | ENSP00000347872 | VAT1 | 7.753E-06 | -0.369 | - | - |
| 16033 | ENSP00000385021 | FANCL | 1.336E-05 | -0.369 | - | - |
| 16034 | ENSP00000350869 | ZNF346 | 8.586E-06 | -0.369 | - | - |
| 16035 | ENSP00000409231 | TRAPPC13 | 1.560E-06 | -0.369 | - | - |
| 16036 | ENSP00000353010 | ATG2B | 8.081E-06 | -0.369 | - | - |
| 16037 | ENSP00000365200 | TEX30 | 4.250E-06 | -0.369 | - | - |
| 16038 | ENSP00000357602 | LRRC27 | 1.285E-05 | -0.370 | - | - |
| 16039 | ENSP00000361616 | COMTD1 | 1.055E-05 | -0.370 | - | - |
| 16040 | ENSP00000335255 | ANGPTL5 | 1.251E-05 | -0.370 | - | - |
| 16041 | ENSP00000400587 | AC005008.2 | 4.755E-06 | -0.370 | - | - |
| 16042 | ENSP00000453399 | TMEM185B | 6.790E-06 | -0.370 | - | - |
| 16043 | ENSP00000281146 | C4orf33 | 1.343E-05 | -0.370 | - | - |
| 16044 | ENSP00000440704 | SYTL1 | 1.204E-05 | -0.370 | - | - |
| 16045 | ENSP00000366083 | SSX4 | 4.436E-06 | -0.370 | - | - |
| 16046 | ENSP00000357226 | C1orf61 | 3.660E-06 | -0.370 | - | - |
| 16047 | ENSP00000410621 | USP17L15 | 6.167E-06 | -0.370 | - | - |
| 16048 | ENSP00000406955 | ZBTB38 | 1.184E-05 | -0.370 | - | - |
| 16049 | ENSP00000377640 | RPL24 | 1.325E-05 | -0.370 | - | - |
| 16050 | ENSP00000414019 | GDPD2 | 1.016E-05 | -0.370 | - | - |
| 16051 | ENSP00000362841 | NKAIN1 | 1.479E-05 | -0.370 | - | - |
| 16052 | ENSP00000366672 | DTD1 | 6.339E-06 | -0.370 | - | - |
| 16053 | ENSP00000359225 | CNN3 | 1.167E-05 | -0.370 | - | - |
| 16054 | ENSP00000369736 | TBCA | 8.556E-06 | -0.370 | - | - |
| 16055 | ENSP00000354876 | MT-CO2 | 1.559E-05 | -0.370 | - | - |
| 16056 | ENSP00000414721 | WBP1L | 4.199E-06 | -0.370 | - | - |
| 16057 | ENSP00000363638 | LYPLA2 | 9.097E-06 | -0.370 | - | - |
| 16058 | ENSP00000361813 | BEX1 | 3.530E-06 | -0.370 | - | - |
| 16059 | ENSP00000354724 | PCGF3 | 1.370E-05 | -0.370 | - | - |
| 16060 | ENSP00000367203 | KDM6A | 2.729E-05 | -0.370 | - | - |
| 16061 | ENSP00000379691 | PDXDC1 | 1.300E-05 | -0.370 | - | - |
| 16062 | ENSP00000380969 | THOC5 | 8.065E-06 | -0.370 | - | - |
| 16063 | ENSP00000414144 | TMEM236 | 1.444E-06 | -0.370 | - | - |
| 16064 | ENSP00000386104 | CPE | 1.563E-05 | -0.370 | - | - |
| 16065 | ENSP00000280082 | MIA2 | 7.537E-06 | -0.370 | - | - |
| 16066 | ENSP00000456594 | TMEM178B | 2.250E-06 | -0.370 | - | - |
| 16067 | ENSP00000370022 | EBF4 | 1.660E-05 | -0.371 | - | - |
| 16068 | ENSP00000362021 | TMEM35 | 8.772E-06 | -0.371 | - | - |
| 16069 | ENSP00000328149 | SPATA8 | 1.188E-05 | -0.371 | - | - |
| 16070 | ENSP00000332062 | SNX20 | 1.048E-05 | -0.371 | - | - |
| 16071 | ENSP00000293303 | KLHL10 | 5.233E-06 | -0.371 | - | - |
| 16072 | ENSP00000275954 | TMEM47 | 1.956E-05 | -0.371 | - | - |
| 16073 | ENSP00000359594 | CLCA4 | 1.802E-05 | -0.371 | - | - |
| 16074 | ENSP00000354313 | GRAMD4 | 1.068E-05 | -0.371 | - | - |
| 16075 | ENSP00000386531 | SMPD4 | 8.052E-06 | -0.371 | - | - |
| 16076 | ENSP00000269967 | CCDC97 | 9.260E-06 | -0.371 | - | - |
| 16077 | ENSP00000382269 | HECTD1 | 1.375E-05 | -0.371 | - | - |
| 16078 | ENSP00000338988 | SLMO1 | 1.099E-05 | -0.371 | - | - |
| 16079 | ENSP00000366608 | CBWD7 | 3.848E-06 | -0.371 | - | - |
| 16080 | ENSP00000342169 | TEX9 | 8.817E-06 | -0.371 | - | - |
| 16081 | ENSP00000439056 | HAUS5 | 1.203E-05 | -0.371 | - | - |
| 16082 | ENSP00000443007 | C12orf36 | 2.300E-06 | -0.371 | - | - |
| 16083 | ENSP00000273905 | SLC10A6 | 1.485E-05 | -0.371 | - | - |
| 16084 | ENSP00000278505 | ENDOD1 | 8.264E-06 | -0.371 | - | - |
| 16085 | ENSP00000366557 | CRNKL1 | 7.601E-06 | -0.371 | - | - |
| 16086 | ENSP00000265720 | DUS4L | 1.635E-05 | -0.371 | - | - |
| 16087 | ENSP00000351325 | PATE2 | 4.215E-06 | -0.371 | - | - |
| 16088 | ENSP00000366988 | SNX5 | 1.398E-05 | -0.371 | - | - |
| 16089 | ENSP00000379802 | AMPD3 | 1.200E-05 | -0.371 | - | - |
| 16090 | ENSP00000366662 | CA6 | 1.035E-05 | -0.371 | - | - |
| 16091 | ENSP00000424040 | TAS2R50 | 7.375E-06 | -0.371 | - | - |
| 16092 | ENSP00000449428 | TWF1 | 7.711E-06 | -0.371 | - | - |
| 16093 | ENSP00000470381 | ZNF468 | 1.018E-05 | -0.371 | - | - |
| 16094 | ENSP00000447731 | TMEM116 | 5.339E-06 | -0.371 | - | - |
| 16095 | ENSP00000369614 | CNTROB | 7.669E-06 | -0.372 | - | - |
| 16096 | ENSP00000368965 | HGSNAT | 7.930E-06 | -0.372 | - | - |
| 16097 | ENSP00000400880 | USP17L11 | 6.328E-06 | -0.372 | - | - |
| 16098 | ENSP00000285402 | ODF1 | 7.432E-06 | -0.372 | - | - |
| 16099 | ENSP00000360296 | PPAP2B | 1.673E-05 | -0.372 | - | - |
| 16100 | ENSP00000355315 | RPL39 | 5.603E-06 | -0.372 | - | - |
| 16101 | ENSP00000381553 | CCDC11 | 7.098E-06 | -0.372 | - | - |
| 16102 | ENSP00000434676 | USP50 | 1.272E-05 | -0.372 | - | - |
| 16103 | ENSP00000355264 | FAM111A | 5.804E-06 | -0.372 | - | - |
| 16104 | ENSP00000380460 | PLAA | 1.110E-05 | -0.372 | - | - |
| 16105 | ENSP00000347408 | AP3M1 | 1.181E-05 | -0.372 | - | - |
| 16106 | ENSP00000372005 | DNAJC19 | 4.747E-06 | -0.372 | - | - |
| 16107 | ENSP00000417235 | MED12L | 1.279E-05 | -0.372 | - | - |
| 16108 | ENSP00000349781 | TBC1D8B | 1.176E-05 | -0.372 | - | - |
| 16109 | ENSP00000340474 | EXD3 | 8.946E-06 | -0.372 | - | - |
| 16110 | ENSP00000339292 | CLDN14 | 1.321E-05 | -0.372 | - | - |
| 16111 | ENSP00000300575 | C16orf92 | 1.082E-05 | -0.372 | - | - |
| 16112 | ENSP00000439287 | RIMS4 | 2.178E-06 | -0.372 | - | - |
| 16113 | ENSP00000411409 | ZNF598 | 1.656E-05 | -0.372 | - | - |
| 16114 | ENSP00000424474 | SAMD14 | 4.087E-06 | -0.372 | - | - |
| 16115 | ENSP00000328270 | KRTAP4-6 | 2.497E-06 | -0.372 | - | - |
| 16116 | ENSP00000392094 | EFTUD2 | 2.120E-05 | -0.372 | - | - |
| 16117 | ENSP00000412394 | MAML2 | 7.985E-06 | -0.372 | - | - |
| 16118 | ENSP00000355607 | GNPAT | 1.398E-05 | -0.373 | - | - |
| 16119 | ENSP00000342278 | OAS2 | 2.308E-05 | -0.373 | - | - |
| 16120 | ENSP00000226460 | SMR3A | 2.715E-05 | -0.373 | - | - |
| 16121 | ENSP00000406933 | NT5DC2 | 5.373E-06 | -0.373 | - | - |
| 16122 | ENSP00000374408 | MROH2A | 5.077E-06 | -0.373 | - | - |
| 16123 | ENSP00000468007 | LUZP6 | 3.529E-06 | -0.373 | - | - |
| 16124 | ENSP00000390759 | USP17L13 | 6.338E-06 | -0.373 | - | - |
| 16125 | ENSP00000471799 | AL049747.1 | 2.793E-06 | -0.373 | - | - |
| 16126 | ENSP00000261643 | COX10 | 7.850E-06 | -0.373 | - | - |
| 16127 | ENSP00000361599 | CCDC23 | 5.487E-06 | -0.373 | - | - |
| 16128 | ENSP00000463094 | PIEZO2 | 8.739E-06 | -0.373 | - | - |
| 16129 | ENSP00000362525 | UGT1A7 | 9.506E-06 | -0.373 | - | - |
| 16130 | ENSP00000278853 | ZP1 | 8.499E-06 | -0.373 | - | - |
| 16131 | ENSP00000376337 | BTBD6 | 1.246E-05 | -0.373 | - | - |
| 16132 | ENSP00000384144 | HYOU1 | 2.060E-05 | -0.373 | - | - |
| 16133 | ENSP00000357737 | SPRR2G | 1.001E-05 | -0.373 | - | - |
| 16134 | ENSP00000357922 | C1orf56 | 1.750E-05 | -0.373 | - | - |
| 16135 | ENSP00000338556 | RSPH10B | 1.337E-05 | -0.373 | - | - |
| 16136 | ENSP00000328326 | XKR4 | 1.439E-05 | -0.373 | - | - |
| 16137 | ENSP00000393066 | SLC4A10 | 1.086E-05 | -0.373 | - | - |
| 16138 | ENSP00000420477 | NACAD | 1.721E-05 | -0.373 | - | - |
| 16139 | ENSP00000360644 | DPM1 | 1.844E-05 | -0.373 | - | - |
| 16140 | ENSP00000280236 | FSIP1 | 2.006E-05 | -0.374 | - | - |
| 16141 | ENSP00000367059 | ESPN | 1.553E-05 | -0.374 | - | - |
| 16142 | ENSP00000438526 | KLHL35 | 6.709E-06 | -0.374 | - | - |
| 16143 | ENSP00000377083 | ALDH1L1 | 1.131E-05 | -0.374 | - | - |
| 16144 | ENSP00000266031 | HYAL1 | 1.802E-05 | -0.374 | - | - |
| 16145 | ENSP00000361602 | ZNF503 | 7.691E-06 | -0.374 | - | - |
| 16146 | ENSP00000456021 | - | 1.401E-06 | -0.374 | - | - |
| 16147 | ENSP00000325144 | C15orf53 | 1.348E-05 | -0.374 | - | - |
| 16148 | ENSP00000470478 | CTD-3088G3.8 | 3.345E-06 | -0.374 | - | - |
| 16149 | ENSP00000352522 | ATP6V1H | 9.549E-06 | -0.374 | - | - |
| 16150 | ENSP00000334300 | ACTL7A | 8.891E-06 | -0.374 | - | - |
| 16151 | ENSP00000452085 | EPPIN | 9.007E-06 | -0.374 | - | - |
| 16152 | ENSP00000396722 | NTM | 1.661E-05 | -0.374 | - | - |
| 16153 | ENSP00000434593 | C8orf82 | 5.831E-07 | -0.374 | - | - |
| 16154 | ENSP00000258662 | NUDT15 | 1.854E-05 | -0.374 | - | - |
| 16155 | ENSP00000395742 | JAM3 | 1.165E-05 | -0.374 | - | - |
| 16156 | ENSP00000418707 | SLAIN1 | 2.906E-06 | -0.374 | - | - |
| 16157 | ENSP00000423391 | RFPL4B | 5.014E-06 | -0.374 | - | - |
| 16158 | ENSP00000380996 | KIAA1211L | 4.833E-06 | -0.374 | - | - |
| 16159 | ENSP00000359050 | C10orf76 | 7.752E-06 | -0.374 | - | - |
| 16160 | ENSP00000380251 | NUDT19 | 5.074E-06 | -0.374 | - | - |
| 16161 | ENSP00000392879 | LL0XNC01-19D8.1 | 6.654E-06 | -0.375 | - | - |
| 16162 | ENSP00000253934 | TMEM204 | 1.987E-05 | -0.375 | - | - |
| 16163 | ENSP00000307208 | BPTF | 1.511E-05 | -0.375 | - | - |
| 16164 | ENSP00000380109 | NFKBID | 1.368E-05 | -0.375 | - | - |
| 16165 | ENSP00000379888 | RPS8 | 1.086E-05 | -0.375 | - | - |
| 16166 | ENSP00000320017 | PHYHIP | 1.770E-05 | -0.375 | - | - |
| 16167 | ENSP00000369604 | FBXO16 | 5.941E-06 | -0.375 | - | - |
| 16168 | ENSP00000346536 | KRTAP23-1 | 2.959E-06 | -0.375 | - | - |
| 16169 | ENSP00000355195 | ASB12 | 8.991E-06 | -0.375 | - | - |
| 16170 | ENSP00000436049 | EIF3M | 8.965E-06 | -0.375 | - | - |
| 16171 | ENSP00000371847 | DEFB125 | 3.789E-06 | -0.375 | - | - |
| 16172 | ENSP00000428982 | CCNC | 1.682E-05 | -0.375 | - | - |
| 16173 | ENSP00000424417 | PLRG1 | 7.361E-06 | -0.375 | - | - |
| 16174 | ENSP00000296721 | AFAP1L1 | 9.571E-06 | -0.375 | - | - |
| 16175 | ENSP00000370381 | SLC12A1 | 1.946E-05 | -0.375 | - | - |
| 16176 | ENSP00000321179 | RBM44 | 1.242E-05 | -0.375 | - | - |
| 16177 | ENSP00000397441 | COG6 | 1.061E-05 | -0.375 | - | - |
| 16178 | ENSP00000367715 | DCDC2 | 8.513E-06 | -0.375 | - | - |
| 16179 | ENSP00000401197 | USP19 | 8.682E-06 | -0.375 | - | - |
| 16180 | ENSP00000378529 | FZR1 | 1.671E-05 | -0.375 | - | - |
| 16181 | ENSP00000359170 | DPCD | 4.479E-06 | -0.376 | - | - |
| 16182 | ENSP00000441823 | SALL3 | 1.460E-05 | -0.376 | - | - |
| 16183 | ENSP00000426528 | AMER2 | 5.936E-06 | -0.376 | - | - |
| 16184 | ENSP00000395574 | TCEB3 | 7.499E-06 | -0.376 | - | - |
| 16185 | ENSP00000472696 | AD000671.6 | 6.288E-06 | -0.376 | - | - |
| 16186 | ENSP00000360968 | CYP4X1 | 6.736E-06 | -0.376 | - | - |
| 16187 | ENSP00000302951 | STRA13 | 7.445E-06 | -0.376 | - | - |
| 16188 | ENSP00000373846 | LTN1 | 1.342E-05 | -0.376 | - | - |
| 16189 | ENSP00000443988 | SUDS3 | 7.015E-06 | -0.376 | - | - |
| 16190 | ENSP00000356224 | SYNE1 | 1.535E-05 | -0.376 | - | - |
| 16191 | ENSP00000465932 | PRCD | 7.797E-06 | -0.376 | - | - |
| 16192 | ENSP00000349856 | PRRC2B | 6.180E-06 | -0.376 | - | - |
| 16193 | ENSP00000445768 | KIAA1147 | 4.794E-06 | -0.376 | - | - |
| 16194 | ENSP00000429214 | HMGB4 | 9.153E-06 | -0.376 | - | - |
| 16195 | ENSP00000377385 | PPAN-P2RY11 | 1.442E-05 | -0.376 | - | - |
| 16196 | ENSP00000375236 | KRTAP4-7 | 1.924E-06 | -0.376 | - | - |
| 16197 | ENSP00000410216 | POC5 | 4.505E-06 | -0.376 | - | - |
| 16198 | ENSP00000371833 | PLA2G4F | 1.753E-05 | -0.376 | - | - |
| 16199 | ENSP00000355419 | PCDH11Y | 1.336E-05 | -0.376 | - | - |
| 16200 | ENSP00000368758 | CTXN3 | 2.375E-06 | -0.376 | - | - |
| 16201 | ENSP00000413493 | CPSF3L | 1.219E-05 | -0.376 | - | - |
| 16202 | ENSP00000306918 | PCDHGC4 | 2.134E-05 | -0.376 | - | - |
| 16203 | ENSP00000384081 | EFR3B | 1.126E-05 | -0.376 | - | - |
| 16204 | ENSP00000416583 | DERA | 9.988E-06 | -0.376 | - | - |
| 16205 | ENSP00000348849 | RPS26 | 9.519E-06 | -0.376 | - | - |
| 16206 | ENSP00000417764 | ALG2 | 2.505E-05 | -0.377 | - | - |
| 16207 | ENSP00000414851 | ELP2 | 1.255E-05 | -0.377 | - | - |
| 16208 | ENSP00000429367 | FAM92A1 | 3.601E-06 | -0.377 | - | - |
| 16209 | ENSP00000363394 | SLC30A2 | 1.265E-05 | -0.377 | - | - |
| 16210 | ENSP00000380427 | ARPC4-TTLL3 | 1.283E-05 | -0.377 | - | - |
| 16211 | ENSP00000472710 | CTD-2192J16.24 | 4.578E-06 | -0.377 | - | - |
| 16212 | ENSP00000334198 | CACNA1H | 1.574E-05 | -0.377 | - | - |
| 16213 | ENSP00000362121 | IFT52 | 1.003E-05 | -0.377 | - | - |
| 16214 | ENSP00000354730 | CHAMP1 | 5.097E-06 | -0.377 | - | - |
| 16215 | ENSP00000354830 | DDIT4L | 1.396E-05 | -0.377 | - | - |
| 16216 | ENSP00000430338 | INTS8 | 6.866E-06 | -0.377 | - | - |
| 16217 | ENSP00000365812 | EP400NL | 1.134E-05 | -0.377 | - | - |
| 16218 | ENSP00000364407 | METTL8 | 8.105E-06 | -0.377 | - | - |
| 16219 | ENSP00000330878 | KPNA7 | 1.278E-05 | -0.377 | - | - |
| 16220 | ENSP00000325954 | CDHR3 | 1.407E-05 | -0.377 | - | - |
| 16221 | ENSP00000357835 | LHPP | 1.096E-05 | -0.377 | - | - |
| 16222 | ENSP00000296088 | SNRK | 1.965E-05 | -0.377 | - | - |
| 16223 | ENSP00000246896 | HTN1 | 1.916E-05 | -0.378 | - | - |
| 16224 | ENSP00000354039 | UBAP2 | 1.372E-05 | -0.378 | - | - |
| 16225 | ENSP00000315945 | CD163L1 | 9.051E-06 | -0.378 | - | - |
| 16226 | ENSP00000362549 | UGT1A8 | 8.361E-06 | -0.378 | - | - |
| 16227 | ENSP00000418721 | PURG | 1.405E-05 | -0.378 | - | - |
| 16228 | ENSP00000356047 | PFKFB2 | 1.034E-05 | -0.378 | - | - |
| 16229 | ENSP00000295622 | C3orf30 | 4.330E-06 | -0.378 | - | - |
| 16230 | ENSP00000389443 | USP17L12 | 6.303E-06 | -0.378 | - | - |
| 16231 | ENSP00000355228 | HMGN2 | 8.506E-06 | -0.378 | - | - |
| 16232 | ENSP00000299752 | CDH16 | 1.319E-05 | -0.378 | - | - |
| 16233 | ENSP00000354152 | MTHFSD | 9.893E-06 | -0.378 | - | - |
| 16234 | ENSP00000390590 | DDN | 6.603E-06 | -0.378 | - | - |
| 16235 | ENSP00000366565 | VPS28 | 1.161E-05 | -0.378 | - | - |
| 16236 | ENSP00000386398 | ANKRD30BL | 1.161E-05 | -0.378 | - | - |
| 16237 | ENSP00000368389 | NGRN | 9.094E-06 | -0.378 | - | - |
| 16238 | ENSP00000299698 | A2ML1 | 1.360E-05 | -0.378 | - | - |
| 16239 | ENSP00000254337 | DCAF15 | 1.083E-05 | -0.378 | - | - |
| 16240 | ENSP00000367227 | TMEM8B | 6.359E-06 | -0.378 | - | - |
| 16241 | ENSP00000363513 | NCMAP | 8.211E-06 | -0.378 | - | - |
| 16242 | ENSP00000358953 | CUEDC2 | 9.796E-06 | -0.378 | - | - |
| 16243 | ENSP00000341171 | MTSS1L | 1.523E-05 | -0.378 | - | - |
| 16244 | ENSP00000272462 | MALL | 1.768E-05 | -0.378 | - | - |
| 16245 | ENSP00000373669 | C17orf59 | 4.686E-06 | -0.378 | - | - |
| 16246 | ENSP00000360492 | NDUFA1 | 1.268E-05 | -0.378 | - | - |
| 16247 | ENSP00000378897 | ASAH2 | 8.233E-06 | -0.378 | - | - |
| 16248 | ENSP00000312129 | KCNG4 | 1.723E-05 | -0.378 | - | - |
| 16249 | ENSP00000410186 | AK9 | 1.391E-05 | -0.378 | - | - |
| 16250 | ENSP00000358142 | SV2A | 1.074E-05 | -0.378 | - | - |
| 16251 | ENSP00000378288 | MYLK3 | 1.490E-05 | -0.378 | - | - |
| 16252 | ENSP00000458238 | PI4KA | 1.917E-05 | -0.378 | - | - |
| 16253 | ENSP00000360065 | WDR78 | 9.438E-06 | -0.378 | - | - |
| 16254 | ENSP00000401878 | KBTBD8 | 6.251E-06 | -0.378 | - | - |
| 16255 | ENSP00000261486 | EPB41L4A | 9.576E-06 | -0.379 | - | - |
| 16256 | ENSP00000349174 | SLC39A8 | 1.872E-05 | -0.379 | - | - |
| 16257 | ENSP00000376333 | LLGL2 | 1.123E-05 | -0.379 | - | - |
| 16258 | ENSP00000352011 | CACNA1G | 1.275E-05 | -0.379 | - | - |
| 16259 | ENSP00000359988 | SRSF11 | 6.423E-06 | -0.379 | - | - |
| 16260 | ENSP00000347924 | SFXN4 | 4.934E-06 | -0.379 | - | - |
| 16261 | ENSP00000408295 | RFC5 | 1.434E-05 | -0.379 | - | - |
| 16262 | ENSP00000380888 | MED13 | 1.139E-05 | -0.379 | - | - |
| 16263 | ENSP00000397415 | RLN3 | 5.421E-06 | -0.379 | - | - |
| 16264 | ENSP00000392028 | CHD7 | 2.347E-05 | -0.379 | - | - |
| 16265 | ENSP00000415436 | KANSL2 | 1.018E-05 | -0.379 | - | - |
| 16266 | ENSP00000390158 | HERC1 | 1.562E-05 | -0.379 | - | - |
| 16267 | ENSP00000367407 | NMT2 | 9.066E-06 | -0.379 | - | - |
| 16268 | ENSP00000413405 | PIGZ | 1.242E-05 | -0.379 | - | - |
| 16269 | ENSP00000355645 | ACTA1 | 2.762E-05 | -0.379 | - | - |
| 16270 | ENSP00000369198 | GPR64 | 1.886E-05 | -0.379 | - | - |
| 16271 | ENSP00000404381 | DNAJB14 | 1.147E-05 | -0.379 | - | - |
| 16272 | ENSP00000368612 | VWA8 | 1.080E-05 | -0.379 | - | - |
| 16273 | ENSP00000332723 | COLEC10 | 1.367E-05 | -0.379 | - | - |
| 16274 | ENSP00000416033 | PROB1 | 2.793E-06 | -0.379 | - | - |
| 16275 | ENSP00000363891 | SLC38A10 | 4.358E-06 | -0.379 | - | - |
| 16276 | ENSP00000352706 | HIBCH | 9.014E-06 | -0.379 | - | - |
| 16277 | ENSP00000366179 | DNAJC1 | 8.922E-06 | -0.380 | - | - |
| 16278 | ENSP00000379901 | TMEM106B | 5.150E-06 | -0.380 | - | - |
| 16279 | ENSP00000398930 | SGCE | 1.252E-05 | -0.380 | - | - |
| 16280 | ENSP00000379290 | BCAS1 | 3.740E-06 | -0.380 | - | - |
| 16281 | ENSP00000405726 | CDC45 | 1.632E-05 | -0.380 | - | - |
| 16282 | ENSP00000453436 | RP11-467N20.5 | 4.695E-06 | -0.380 | - | - |
| 16283 | ENSP00000368858 | TMEM14B | 4.862E-06 | -0.380 | - | - |
| 16284 | ENSP00000368814 | CMTM1 | 1.682E-05 | -0.380 | - | - |
| 16285 | ENSP00000343665 | DOC2B | 1.147E-05 | -0.380 | - | - |
| 16286 | ENSP00000352175 | SNX11 | 6.339E-06 | -0.380 | - | - |
| 16287 | ENSP00000261592 | NOL4 | 2.300E-05 | -0.380 | - | - |
| 16288 | ENSP00000361473 | KDM4A | 1.286E-05 | -0.380 | - | - |
| 16289 | ENSP00000358753 | PIFO | 8.567E-06 | -0.380 | - | - |
| 16290 | ENSP00000345974 | ENOSF1 | 9.209E-06 | -0.380 | - | - |
| 16291 | ENSP00000424768 | MSANTD3-TMEFF1 | 8.954E-07 | -0.380 | - | - |
| 16292 | ENSP00000386711 | JAKMIP1 | 7.729E-06 | -0.380 | - | - |
| 16293 | ENSP00000357511 | C1orf189 | 6.552E-06 | -0.380 | - | - |
| 16294 | ENSP00000420443 | CNIH4 | 7.193E-06 | -0.381 | - | - |
| 16295 | ENSP00000339971 | MROH8 | 9.024E-06 | -0.381 | - | - |
| 16296 | ENSP00000436337 | EPPK1 | 1.206E-05 | -0.381 | - | - |
| 16297 | ENSP00000217026 | MYBL2 | 9.260E-06 | -0.381 | - | - |
| 16298 | ENSP00000351596 | TTC37 | 1.043E-05 | -0.381 | - | - |
| 16299 | ENSP00000304250 | CDK5R2 | 1.977E-05 | -0.381 | - | - |
| 16300 | ENSP00000369500 | SYAP1 | 3.538E-06 | -0.381 | - | - |
| 16301 | ENSP00000307096 | RNASE4 | 5.536E-06 | -0.381 | - | - |
| 16302 | ENSP00000276127 | CPXCR1 | 2.031E-05 | -0.381 | - | - |
| 16303 | ENSP00000407323 | EBNA1BP2 | 1.599E-05 | -0.381 | - | - |
| 16304 | ENSP00000373354 | SETMAR | 1.235E-05 | -0.381 | - | - |
| 16305 | ENSP00000442291 | IQCC | 1.905E-06 | -0.381 | - | - |
| 16306 | ENSP00000456200 | IST1 | 5.772E-06 | -0.381 | - | - |
| 16307 | ENSP00000346240 | FBXO2 | 1.408E-05 | -0.381 | - | - |
| 16308 | ENSP00000333234 | DEFB108B | 1.613E-05 | -0.381 | - | - |
| 16309 | ENSP00000358878 | AMIGO1 | 1.288E-05 | -0.381 | - | - |
| 16310 | ENSP00000357057 | CASQ1 | 1.855E-05 | -0.381 | - | - |
| 16311 | ENSP00000426296 | PCP4L1 | 2.871E-06 | -0.381 | - | - |
| 16312 | ENSP00000350976 | RTP2 | 1.022E-05 | -0.382 | - | - |
| 16313 | ENSP00000391751 | ARL17B | 1.516E-05 | -0.382 | - | - |
| 16314 | ENSP00000351416 | ANKRD17 | 1.399E-05 | -0.382 | - | - |
| 16315 | ENSP00000304078 | FAM83B | 1.228E-05 | -0.382 | - | - |
| 16316 | ENSP00000361064 | MINPP1 | 9.501E-06 | -0.382 | - | - |
| 16317 | ENSP00000299732 | IQCD | 4.814E-06 | -0.382 | - | - |
| 16318 | ENSP00000407193 | ARSG | 7.931E-06 | -0.382 | - | - |
| 16319 | ENSP00000353518 | ANKS1A | 1.276E-05 | -0.382 | - | - |
| 16320 | ENSP00000359151 | DBT | 1.577E-05 | -0.382 | - | - |
| 16321 | ENSP00000387535 | IRGQ | 6.685E-06 | -0.382 | - | - |
| 16322 | ENSP00000347301 | BEGAIN | 5.756E-06 | -0.382 | - | - |
| 16323 | ENSP00000431605 | ATP6V0B | 5.283E-06 | -0.383 | - | - |
| 16324 | ENSP00000377865 | RPL23 | 1.372E-05 | -0.383 | - | - |
| 16325 | ENSP00000377958 | CCT4 | 2.361E-05 | -0.383 | - | - |
| 16326 | ENSP00000304676 | ATOH8 | 1.905E-05 | -0.383 | - | - |
| 16327 | ENSP00000312942 | SEZ6 | 5.180E-06 | -0.383 | - | - |
| 16328 | ENSP00000360129 | EFCAB7 | 1.550E-05 | -0.383 | - | - |
| 16329 | ENSP00000354560 | KIFAP3 | 1.736E-05 | -0.383 | - | - |
| 16330 | ENSP00000394071 | GDI1 | 8.017E-06 | -0.383 | - | - |
| 16331 | ENSP00000299339 | CLDN10 | 1.440E-05 | -0.383 | - | - |
| 16332 | ENSP00000354848 | KIAA1279 | 8.440E-06 | -0.383 | - | - |
| 16333 | ENSP00000358159 | ASCC3 | 1.122E-05 | -0.383 | - | - |
| 16334 | ENSP00000298815 | ARHGAP42 | 1.532E-05 | -0.383 | - | - |
| 16335 | ENSP00000329565 | SPDYE3 | 8.927E-06 | -0.383 | - | - |
| 16336 | ENSP00000184183 | ROPN1 | 1.842E-05 | -0.383 | - | - |
| 16337 | ENSP00000370546 | ARSD | 6.036E-06 | -0.384 | - | - |
| 16338 | ENSP00000362936 | TRNAU1AP | 1.141E-05 | -0.384 | - | - |
| 16339 | ENSP00000360247 | CYP2J2 | 1.434E-05 | -0.384 | - | - |
| 16340 | ENSP00000377914 | CIAPIN1 | 7.488E-06 | -0.384 | - | - |
| 16341 | ENSP00000343087 | CCDC157 | 1.010E-05 | -0.384 | - | - |
| 16342 | ENSP00000354884 | RASSF9 | 1.106E-05 | -0.384 | - | - |
| 16343 | ENSP00000366170 | CST9 | 4.160E-06 | -0.384 | - | - |
| 16344 | ENSP00000386450 | C7orf34 | 5.337E-07 | -0.384 | - | - |
| 16345 | ENSP00000380321 | TULP3 | 1.186E-05 | -0.384 | - | - |
| 16346 | ENSP00000280190 | WDR17 | 2.034E-05 | -0.384 | - | - |
| 16347 | ENSP00000367882 | LANCL3 | 2.970E-06 | -0.384 | - | - |
| 16348 | ENSP00000310303 | TOPAZ1 | 1.466E-05 | -0.384 | - | - |
| 16349 | ENSP00000352163 | LYPD2 | 1.459E-05 | -0.384 | - | - |
| 16350 | ENSP00000357762 | KPRP | 1.128E-05 | -0.384 | - | - |
| 16351 | ENSP00000246646 | KRT38 | 9.173E-06 | -0.384 | - | - |
| 16352 | ENSP00000355614 | FAM89A | 1.492E-05 | -0.384 | - | - |
| 16353 | ENSP00000415556 | DCAF11 | 5.765E-06 | -0.384 | - | - |
| 16354 | ENSP00000352182 | FAM86C1 | 7.052E-06 | -0.384 | - | - |
| 16355 | ENSP00000253262 | NUTMF | 1.896E-05 | -0.385 | - | - |
| 16356 | ENSP00000439189 | POTEE | 3.354E-05 | -0.385 | - | - |
| 16357 | ENSP00000313172 | BVES | 1.834E-05 | -0.385 | - | - |
| 16358 | ENSP00000448182 | TBC1D15 | 9.941E-06 | -0.385 | - | - |
| 16359 | ENSP00000357048 | COPA | 1.423E-05 | -0.385 | - | - |
| 16360 | ENSP00000383295 | NBEA | 6.645E-06 | -0.385 | - | - |
| 16361 | ENSP00000376767 | C11orf57 | 2.948E-06 | -0.385 | - | - |
| 16362 | ENSP00000404078 | COX6A1P2 | 4.772E-06 | -0.385 | - | - |
| 16363 | ENSP00000386133 | POM121L12 | 1.943E-06 | -0.385 | - | - |
| 16364 | ENSP00000352547 | GPAT2 | 7.771E-06 | -0.385 | - | - |
| 16365 | ENSP00000407436 | AGAP10 | 5.715E-06 | -0.385 | - | - |
| 16366 | ENSP00000363590 | CSGALNACT2 | 9.482E-06 | -0.385 | - | - |
| 16367 | ENSP00000335660 | KRTAP19-2 | 2.344E-06 | -0.385 | - | - |
| 16368 | ENSP00000306328 | SLC25A33 | 1.773E-05 | -0.385 | - | - |
| 16369 | ENSP00000356094 | AVPR1B | 1.420E-05 | -0.385 | - | - |
| 16370 | ENSP00000393539 | ARHGAP23 | 1.338E-05 | -0.385 | - | - |
| 16371 | ENSP00000398688 | VWA5B2 | 8.813E-06 | -0.385 | - | - |
| 16372 | ENSP00000392066 | DIP2A | 1.363E-05 | -0.385 | - | - |
| 16373 | ENSP00000351214 | TOMM7 | 4.536E-06 | -0.386 | - | - |
| 16374 | ENSP00000289921 | FAM160B2 | 8.139E-06 | -0.386 | - | - |
| 16375 | ENSP00000366387 | MAMDC2 | 6.228E-06 | -0.386 | - | - |
| 16376 | ENSP00000363642 | BMS1 | 9.323E-06 | -0.386 | - | - |
| 16377 | ENSP00000430000 | ATXN7L3B | 2.595E-05 | -0.386 | - | - |
| 16378 | ENSP00000363753 | FAM206A | 4.665E-06 | -0.386 | - | - |
| 16379 | ENSP00000347792 | SYNJ2 | 1.433E-05 | -0.386 | - | - |
| 16380 | ENSP00000304822 | CSN3 | 1.520E-05 | -0.386 | - | - |
| 16381 | ENSP00000368716 | NAA16 | 7.450E-06 | -0.386 | - | - |
| 16382 | ENSP00000367029 | POLR1E | 9.373E-06 | -0.386 | - | - |
| 16383 | ENSP00000357453 | MAN1A1 | 1.531E-05 | -0.386 | - | - |
| 16384 | ENSP00000417763 | NAA10 | 8.326E-06 | -0.386 | - | - |
| 16385 | ENSP00000460885 | CORO7-PAM16 | 3.445E-06 | -0.386 | - | - |
| 16386 | ENSP00000264703 | FNDC4 | 1.848E-05 | -0.386 | - | - |
| 16387 | ENSP00000273158 | SLC25A38 | 1.646E-05 | -0.386 | - | - |
| 16388 | ENSP00000363250 | GPN2 | 5.320E-06 | -0.386 | - | - |
| 16389 | ENSP00000361993 | TIMM8A | 9.680E-06 | -0.386 | - | - |
| 16390 | ENSP00000281471 | AMN1 | 1.885E-05 | -0.386 | - | - |
| 16391 | ENSP00000363985 | MTMR8 | 1.175E-05 | -0.386 | - | - |
| 16392 | ENSP00000384876 | TRIM66 | 6.302E-06 | -0.386 | - | - |
| 16393 | ENSP00000455507 | OBSCN | 2.767E-05 | -0.386 | - | - |
| 16394 | ENSP00000441949 | TAS2R14 | 6.549E-06 | -0.386 | - | - |
| 16395 | ENSP00000352962 | LPPR3 | 1.187E-05 | -0.386 | - | - |
| 16396 | ENSP00000287196 | PARP6 | 1.612E-05 | -0.387 | - | - |
| 16397 | ENSP00000385814 | RPAIN | 3.265E-06 | -0.387 | - | - |
| 16398 | ENSP00000438204 | TOMM5 | 5.570E-06 | -0.387 | - | - |
| 16399 | ENSP00000394510 | PRR12 | 6.699E-06 | -0.387 | - | - |
| 16400 | ENSP00000372390 | SLC7A4 | 5.513E-06 | -0.387 | - | - |
| 16401 | ENSP00000361894 | GNMT | 1.074E-05 | -0.387 | - | - |
| 16402 | ENSP00000371201 | NSUN7 | 8.228E-06 | -0.387 | - | - |
| 16403 | ENSP00000310841 | FBXO31 | 8.613E-06 | -0.387 | - | - |
| 16404 | ENSP00000395086 | TRIM51 | 7.278E-06 | -0.387 | - | - |
| 16405 | ENSP00000309521 | LIME1 | 1.028E-05 | -0.387 | - | - |
| 16406 | ENSP00000359307 | MAGEA2 | 7.175E-06 | -0.387 | - | - |
| 16407 | ENSP00000366418 | LZIC | 6.913E-06 | -0.387 | - | - |
| 16408 | ENSP00000335156 | GPR144 | 1.546E-05 | -0.387 | - | - |
| 16409 | ENSP00000311899 | DAW1 | 1.057E-05 | -0.387 | - | - |
| 16410 | ENSP00000340017 | DOC2A | 7.249E-06 | -0.387 | - | - |
| 16411 | ENSP00000376504 | VWA5A | 2.002E-05 | -0.387 | - | - |
| 16412 | ENSP00000360472 | LGI1 | 8.253E-06 | -0.387 | - | - |
| 16413 | ENSP00000298999 | R3HCC1L | 1.345E-05 | -0.387 | - | - |
| 16414 | ENSP00000371101 | NOL10 | 1.070E-05 | -0.387 | - | - |
| 16415 | ENSP00000318409 | TMEM176B | 8.485E-06 | -0.387 | - | - |
| 16416 | ENSP00000323191 | SAGE1 | 9.608E-06 | -0.387 | - | - |
| 16417 | ENSP00000392936 | RFPL4A | 1.052E-05 | -0.388 | - | - |
| 16418 | ENSP00000366970 | GPR20 | 1.585E-05 | -0.388 | - | - |
| 16419 | ENSP00000379034 | KLHDC7B | 8.689E-06 | -0.388 | - | - |
| 16420 | ENSP00000333490 | PRHOXNB | 1.309E-05 | -0.388 | - | - |
| 16421 | ENSP00000446264 | TMEM75 | 9.962E-07 | -0.388 | - | - |
| 16422 | ENSP00000386881 | DYSF | 1.165E-05 | -0.388 | - | - |
| 16423 | ENSP00000362601 | CHIC1 | 1.448E-05 | -0.388 | - | - |
| 16424 | ENSP00000299295 | CHODL | 1.700E-05 | -0.388 | - | - |
| 16425 | ENSP00000355299 | TSEN15 | 3.086E-06 | -0.388 | - | - |
| 16426 | ENSP00000297404 | KCNV1 | 1.651E-05 | -0.388 | - | - |
| 16427 | ENSP00000361331 | ERI3 | 1.069E-05 | -0.388 | - | - |
| 16428 | ENSP00000376304 | TMEM121 | 4.478E-06 | -0.388 | - | - |
| 16429 | ENSP00000363575 | - | 7.052E-06 | -0.388 | - | - |
| 16430 | ENSP00000452007 | ISCA2 | 4.675E-06 | -0.388 | - | - |
| 16431 | ENSP00000366677 | CBWD6 | 4.128E-06 | -0.388 | - | - |
| 16432 | ENSP00000378152 | AKTIP | 1.111E-05 | -0.388 | - | - |
| 16433 | ENSP00000301190 | ANKRD33 | 7.213E-06 | -0.388 | - | - |
| 16434 | ENSP00000409315 | RBM18 | 1.743E-06 | -0.388 | - | - |
| 16435 | ENSP00000371193 | CPPED1 | 7.704E-06 | -0.388 | - | - |
| 16436 | ENSP00000395259 | SCAND3 | 1.043E-05 | -0.389 | - | - |
| 16437 | ENSP00000427130 | ANKRD13D | 3.680E-06 | -0.389 | - | - |
| 16438 | ENSP00000358945 | OPN1MW2 | 1.334E-05 | -0.389 | - | - |
| 16439 | ENSP00000335382 | DEFB128 | 4.700E-06 | -0.389 | - | - |
| 16440 | ENSP00000385995 | THADA | 1.504E-05 | -0.389 | - | - |
| 16441 | ENSP00000321038 | TMEM86B | 9.031E-06 | -0.389 | - | - |
| 16442 | ENSP00000354778 | CNTNAP2 | 1.470E-05 | -0.389 | - | - |
| 16443 | ENSP00000354733 | SBNO2 | 1.083E-05 | -0.389 | - | - |
| 16444 | ENSP00000349957 | ANAPC11 | 1.417E-05 | -0.389 | - | - |
| 16445 | ENSP00000361536 | ELOVL1 | 1.027E-05 | -0.389 | - | - |
| 16446 | ENSP00000369341 | RFESD | 3.613E-06 | -0.389 | - | - |
| 16447 | ENSP00000396452 | HDHD1 | 1.249E-05 | -0.389 | - | - |
| 16448 | ENSP00000421280 | MYO10 | 1.483E-05 | -0.389 | - | - |
| 16449 | ENSP00000362041 | DNAJC9 | 7.065E-06 | -0.389 | - | - |
| 16450 | ENSP00000430497 | ZFHX4 | 6.209E-06 | -0.390 | - | - |
| 16451 | ENSP00000347719 | TBCD | 7.689E-06 | -0.390 | - | - |
| 16452 | ENSP00000381003 | TTC28 | 2.319E-05 | -0.390 | - | - |
| 16453 | ENSP00000350078 | ARID5A | 1.079E-05 | -0.390 | - | - |
| 16454 | ENSP00000467494 | GIPR | 1.455E-05 | -0.390 | - | - |
| 16455 | ENSP00000361124 | GNG5P2 | 3.035E-06 | -0.390 | - | - |
| 16456 | ENSP00000375267 | FAM127B | 3.786E-06 | -0.390 | - | - |
| 16457 | ENSP00000341282 | SYCE1 | 9.900E-06 | -0.390 | - | - |
| 16458 | ENSP00000386908 | MEGF11 | 1.160E-05 | -0.390 | - | - |
| 16459 | ENSP00000404063 | GRIK4 | 8.932E-06 | -0.390 | - | - |
| 16460 | ENSP00000281924 | TMEM163 | 1.493E-05 | -0.390 | - | - |
| 16461 | ENSP00000357297 | RNF146 | 7.537E-06 | -0.390 | - | - |
| 16462 | ENSP00000421489 | TIGD5 | 5.508E-06 | -0.390 | - | - |
| 16463 | ENSP00000406546 | SLC23A3 | 2.957E-06 | -0.390 | - | - |
| 16464 | ENSP00000263196 | DGCR2 | 1.627E-05 | -0.390 | - | - |
| 16465 | ENSP00000394546 | NHSL1 | 3.233E-06 | -0.390 | - | - |
| 16466 | ENSP00000393379 | KIF5C | 1.517E-05 | -0.390 | - | - |
| 16467 | ENSP00000312753 | FAM109B | 1.307E-05 | -0.390 | - | - |
| 16468 | ENSP00000358060 | TARS2 | 8.649E-06 | -0.390 | - | - |
| 16469 | ENSP00000345193 | SHANK2 | 1.230E-05 | -0.390 | - | - |
| 16470 | ENSP00000295226 | CCDC140 | 1.826E-05 | -0.390 | - | - |
| 16471 | ENSP00000231484 | PCDH12 | 2.107E-05 | -0.390 | - | - |
| 16472 | ENSP00000324292 | FBF1 | 1.154E-05 | -0.390 | - | - |
| 16473 | ENSP00000415332 | SESTD1 | 1.833E-05 | -0.390 | - | - |
| 16474 | ENSP00000285013 | SLFN13 | 9.625E-06 | -0.391 | - | - |
| 16475 | ENSP00000374562 | COX16 | 5.852E-06 | -0.391 | - | - |
| 16476 | ENSP00000311218 | C1orf100 | 7.828E-06 | -0.391 | - | - |
| 16477 | ENSP00000404676 | PGGT1B | 6.099E-06 | -0.391 | - | - |
| 16478 | ENSP00000362095 | SRPX2 | 1.189E-05 | -0.391 | - | - |
| 16479 | ENSP00000270824 | EVA1B | 1.312E-05 | -0.391 | - | - |
| 16480 | ENSP00000462945 | MSL1 | 6.896E-06 | -0.391 | - | - |
| 16481 | ENSP00000361338 | PPAPDC3 | 7.194E-06 | -0.391 | - | - |
| 16482 | ENSP00000342868 | SCXB | 1.289E-05 | -0.391 | - | - |
| 16483 | ENSP00000263266 | FAM83E | 1.293E-05 | -0.391 | - | - |
| 16484 | ENSP00000375069 | COL23A1 | 1.511E-05 | -0.391 | - | - |
| 16485 | ENSP00000415178 | RP4-539M6.19 | 5.032E-06 | -0.391 | - | - |
| 16486 | ENSP00000295728 | CRYBA2 | 1.741E-05 | -0.391 | - | - |
| 16487 | ENSP00000362532 | RPRD1B | 6.383E-06 | -0.391 | - | - |
| 16488 | ENSP00000438788 | CUL7 | 9.114E-06 | -0.391 | - | - |
| 16489 | ENSP00000302579 | SCEL | 1.399E-05 | -0.391 | - | - |
| 16490 | ENSP00000393183 | VPRBP | 8.471E-06 | -0.391 | - | - |
| 16491 | ENSP00000418661 | PTRH1 | 5.327E-06 | -0.391 | - | - |
| 16492 | ENSP00000328150 | KCNJ12 | 1.255E-05 | -0.391 | - | - |
| 16493 | ENSP00000333519 | SLC24A3 | 1.647E-05 | -0.391 | - | - |
| 16494 | ENSP00000403802 | FBXO10 | 1.300E-05 | -0.391 | - | - |
| 16495 | ENSP00000355388 | MPHOSPH8 | 1.808E-05 | -0.392 | - | - |
| 16496 | ENSP00000264784 | SLC2A9 | 1.105E-05 | -0.392 | - | - |
| 16497 | ENSP00000297354 | SBSPON | 1.050E-05 | -0.392 | - | - |
| 16498 | ENSP00000385638 | HADH | 2.622E-05 | -0.392 | - | - |
| 16499 | ENSP00000353165 | TPK1 | 1.602E-05 | -0.392 | - | - |
| 16500 | ENSP00000430271 | ETV3L | 6.723E-06 | -0.392 | - | - |
| 16501 | ENSP00000378292 | KRT80 | 3.868E-06 | -0.392 | - | - |
| 16502 | ENSP00000376553 | TMEM119 | 9.049E-06 | -0.392 | - | - |
| 16503 | ENSP00000357360 | MTX1 | 5.492E-06 | -0.392 | - | - |
| 16504 | ENSP00000382863 | CHD8 | 1.723E-05 | -0.392 | - | - |
| 16505 | ENSP00000354001 | KRTAP12-2 | 2.333E-06 | -0.392 | - | - |
| 16506 | ENSP00000356991 | PVRL4 | 9.869E-06 | -0.392 | - | - |
| 16507 | ENSP00000357844 | SLC16A10 | 1.098E-05 | -0.393 | - | - |
| 16508 | ENSP00000361628 | CXorf57 | 5.840E-06 | -0.393 | - | - |
| 16509 | ENSP00000346022 | RPL9 | 5.507E-06 | -0.393 | - | - |
| 16510 | ENSP00000347931 | PCNXL3 | 1.177E-05 | -0.393 | - | - |
| 16511 | ENSP00000446880 | BAZ2A | 9.877E-06 | -0.393 | - | - |
| 16512 | ENSP00000419088 | FAM162A | 4.274E-06 | -0.393 | - | - |
| 16513 | ENSP00000458021 | PMF1 | 4.056E-06 | -0.393 | - | - |
| 16514 | ENSP00000366240 | ABHD17B | 4.075E-06 | -0.393 | - | - |
| 16515 | ENSP00000377015 | PICALM | 1.209E-05 | -0.393 | - | - |
| 16516 | ENSP00000452454 | GTF2A1 | 1.231E-05 | -0.393 | - | - |
| 16517 | ENSP00000362987 | PHYHIPL | 1.855E-06 | -0.393 | - | - |
| 16518 | ENSP00000361518 | LRRC73 | 5.294E-06 | -0.393 | - | - |
| 16519 | ENSP00000347802 | ANKRD35 | 1.295E-05 | -0.393 | - | - |
| 16520 | ENSP00000352216 | SLC2A10 | 1.171E-05 | -0.393 | - | - |
| 16521 | ENSP00000430025 | LYRM2 | 4.558E-06 | -0.393 | - | - |
| 16522 | ENSP00000374183 | CAMSAP1 | 1.024E-05 | -0.393 | - | - |
| 16523 | ENSP00000356563 | PEX3 | 6.009E-06 | -0.393 | - | - |
| 16524 | ENSP00000343507 | TMEM198 | 1.243E-05 | -0.393 | - | - |
| 16525 | ENSP00000384302 | WDR43 | 6.134E-06 | -0.394 | - | - |
| 16526 | ENSP00000360561 | ENTPD8 | 1.348E-05 | -0.394 | - | - |
| 16527 | ENSP00000348314 | SPEF2 | 1.421E-05 | -0.394 | - | - |
| 16528 | ENSP00000372857 | NEK11 | 5.849E-06 | -0.394 | - | - |
| 16529 | ENSP00000376204 | ASH1L | 1.476E-05 | -0.394 | - | - |
| 16530 | ENSP00000454657 | KIAA0391 | 4.996E-06 | -0.394 | - | - |
| 16531 | ENSP00000378782 | QPRT | 1.351E-05 | -0.394 | - | - |
| 16532 | ENSP00000417980 | EHMT1 | 2.203E-05 | -0.394 | - | - |
| 16533 | ENSP00000361151 | CEL | 1.144E-05 | -0.394 | - | - |
| 16534 | ENSP00000365837 | PUS1 | 7.137E-06 | -0.394 | - | - |
| 16535 | ENSP00000378605 | DNAJC30 | 1.240E-05 | -0.394 | - | - |
| 16536 | ENSP00000450731 | VTI1B | 7.952E-06 | -0.394 | - | - |
| 16537 | ENSP00000269081 | ABCA10 | 1.949E-05 | -0.394 | - | - |
| 16538 | ENSP00000411217 | - | 2.154E-06 | -0.394 | - | - |
| 16539 | ENSP00000354501 | ZNF277 | 1.202E-05 | -0.394 | - | - |
| 16540 | ENSP00000308606 | AGR3 | 1.478E-05 | -0.394 | - | - |
| 16541 | ENSP00000370936 | E2F6 | 1.152E-05 | -0.394 | - | - |
| 16542 | ENSP00000344285 | BSX | 9.265E-06 | -0.395 | - | - |
| 16543 | ENSP00000371483 | SLC34A2 | 1.941E-05 | -0.395 | - | - |
| 16544 | ENSP00000375082 | BORA | 1.052E-05 | -0.395 | - | - |
| 16545 | ENSP00000451131 | TTC6 | 8.526E-06 | -0.395 | - | - |
| 16546 | ENSP00000365588 | MDC1 | 1.514E-05 | -0.395 | - | - |
| 16547 | ENSP00000292357 | PEAR1 | 1.136E-05 | -0.395 | - | - |
| 16548 | ENSP00000387888 | UGT2A2 | 9.638E-06 | -0.395 | - | - |
| 16549 | ENSP00000329926 | CALHM1 | 1.167E-05 | -0.395 | - | - |
| 16550 | ENSP00000370394 | RUFY3 | 5.855E-06 | -0.395 | - | - |
| 16551 | ENSP00000458290 | METTL4 | 4.866E-06 | -0.395 | - | - |
| 16552 | ENSP00000378736 | CRCP | 1.095E-05 | -0.395 | - | - |
| 16553 | ENSP00000429430 | TENM2 | 1.174E-05 | -0.395 | - | - |
| 16554 | ENSP00000430128 | RALYL | 1.039E-05 | -0.395 | - | - |
| 16555 | ENSP00000334657 | TRIM46 | 1.729E-05 | -0.395 | - | - |
| 16556 | ENSP00000345575 | DNAJB12 | 6.309E-06 | -0.395 | - | - |
| 16557 | ENSP00000351075 | SUSD2 | 8.455E-06 | -0.395 | - | - |
| 16558 | ENSP00000363621 | GALE | 1.062E-05 | -0.395 | - | - |
| 16559 | ENSP00000359121 | OLFM3 | 1.609E-05 | -0.395 | - | - |
| 16560 | ENSP00000209668 | ADH1A | 1.436E-05 | -0.395 | - | - |
| 16561 | ENSP00000382610 | ANKRD18A | 1.150E-05 | -0.395 | - | - |
| 16562 | ENSP00000308292 | FAM170B | 1.788E-05 | -0.395 | - | - |
| 16563 | ENSP00000363942 | TP53INP2 | 1.594E-05 | -0.395 | - | - |
| 16564 | ENSP00000339613 | POFUT2 | 1.068E-05 | -0.396 | - | - |
| 16565 | ENSP00000371802 | DAZ3 | 8.969E-06 | -0.396 | - | - |
| 16566 | ENSP00000376946 | BBS10 | 1.866E-05 | -0.396 | - | - |
| 16567 | ENSP00000263084 | OVCA2 | 1.153E-05 | -0.396 | - | - |
| 16568 | ENSP00000382379 | ANKRD28 | 1.378E-05 | -0.396 | - | - |
| 16569 | ENSP00000433415 | NT5C1B-RDH14 | 3.646E-06 | -0.396 | - | - |
| 16570 | ENSP00000394107 | AP000304.12 | 1.174E-05 | -0.396 | - | - |
| 16571 | ENSP00000278865 | MS4A3 | 1.452E-05 | -0.396 | - | - |
| 16572 | ENSP00000363229 | SLC18A3 | 1.517E-05 | -0.396 | - | - |
| 16573 | ENSP00000311184 | WFIKKN2 | 1.030E-05 | -0.396 | - | - |
| 16574 | ENSP00000201647 | EPS8L1 | 2.056E-05 | -0.396 | - | - |
| 16575 | ENSP00000274400 | GTF2H2 | 1.495E-05 | -0.396 | - | - |
| 16576 | ENSP00000286732 | C15orf26 | 1.014E-05 | -0.396 | - | - |
| 16577 | ENSP00000352284 | TMEM139 | 1.015E-05 | -0.396 | - | - |
| 16578 | ENSP00000379003 | NUPR1 | 1.275E-05 | -0.396 | - | - |
| 16579 | ENSP00000404029 | SUPT5H | 1.704E-05 | -0.396 | - | - |
| 16580 | ENSP00000350415 | GJA9 | 1.045E-05 | -0.396 | - | - |
| 16581 | ENSP00000364475 | FBP1 | 1.597E-05 | -0.396 | - | - |
| 16582 | ENSP00000397598 | KY | 5.154E-06 | -0.396 | - | - |
| 16583 | ENSP00000432845 | MEX3A | 7.259E-06 | -0.397 | - | - |
| 16584 | ENSP00000385899 | SDK1 | 1.075E-05 | -0.397 | - | - |
| 16585 | ENSP00000365725 | ABHD12 | 9.329E-06 | -0.397 | - | - |
| 16586 | ENSP00000368030 | ATAD3A | 9.557E-06 | -0.397 | - | - |
| 16587 | ENSP00000465845 | CTB-102L5.4 | 3.213E-06 | -0.397 | - | - |
| 16588 | ENSP00000358042 | QRSL1 | 1.197E-05 | -0.397 | - | - |
| 16589 | ENSP00000315013 | CRAT | 1.424E-05 | -0.397 | - | - |
| 16590 | ENSP00000379709 | ARHGAP21 | 1.377E-05 | -0.397 | - | - |
| 16591 | ENSP00000374212 | EP400 | 1.545E-05 | -0.397 | - | - |
| 16592 | ENSP00000355192 | CACNA1S | 1.308E-05 | -0.397 | - | - |
| 16593 | ENSP00000370522 | ARSH | 9.731E-06 | -0.397 | - | - |
| 16594 | ENSP00000380982 | KIAA0020 | 4.554E-06 | -0.397 | - | - |
| 16595 | ENSP00000368273 | MAGEB2 | 8.169E-06 | -0.397 | - | - |
| 16596 | ENSP00000350377 | MCTP2 | 5.880E-06 | -0.397 | - | - |
| 16597 | ENSP00000339251 | KRTDAP | 1.302E-05 | -0.397 | - | - |
| 16598 | ENSP00000312021 | FUT1 | 1.714E-05 | -0.397 | - | - |
| 16599 | ENSP00000346954 | TMEM205 | 4.078E-06 | -0.397 | - | - |
| 16600 | ENSP00000361052 | POMGNT1 | 8.473E-06 | -0.398 | - | - |
| 16601 | ENSP00000420333 | ARMC8 | 4.561E-06 | -0.398 | - | - |
| 16602 | ENSP00000362560 | VSTM2L | 2.904E-06 | -0.398 | - | - |
| 16603 | ENSP00000359557 | LDOC1 | 9.336E-06 | -0.398 | - | - |
| 16604 | ENSP00000396615 | GLB1L3 | 1.029E-05 | -0.398 | - | - |
| 16605 | ENSP00000230012 | HDGFL1 | 2.125E-05 | -0.398 | - | - |
| 16606 | ENSP00000456318 | RP11-234B24.6 | 2.495E-06 | -0.398 | - | - |
| 16607 | ENSP00000381666 | GXYLT1 | 7.029E-06 | -0.398 | - | - |
| 16608 | ENSP00000367024 | NAA40 | 3.823E-06 | -0.398 | - | - |
| 16609 | ENSP00000426103 | MIB2 | 1.659E-05 | -0.398 | - | - |
| 16610 | ENSP00000351981 | SGMS2 | 1.091E-05 | -0.398 | - | - |
| 16611 | ENSP00000349205 | PIK3R4 | 1.158E-05 | -0.398 | - | - |
| 16612 | ENSP00000405482 | SVOPL | 5.779E-06 | -0.398 | - | - |
| 16613 | ENSP00000457071 | MPV17L | 2.434E-06 | -0.399 | - | - |
| 16614 | ENSP00000417556 | RP11-101E3.5 | 1.167E-06 | -0.399 | - | - |
| 16615 | ENSP00000366030 | C9orf41 | 6.258E-06 | -0.399 | - | - |
| 16616 | ENSP00000355651 | RAB4A | 1.498E-05 | -0.399 | - | - |
| 16617 | ENSP00000321929 | NME9 | 1.657E-05 | -0.399 | - | - |
| 16618 | ENSP00000396915 | SCN1B | 4.239E-06 | -0.399 | - | - |
| 16619 | ENSP00000355927 | RPS6KC1 | 9.973E-06 | -0.399 | - | - |
| 16620 | ENSP00000258456 | GPR45 | 2.467E-05 | -0.399 | - | - |
| 16621 | ENSP00000324701 | MAB21L2 | 8.889E-06 | -0.399 | - | - |
| 16622 | ENSP00000377486 | MRPL19 | 4.118E-06 | -0.399 | - | - |
| 16623 | ENSP00000217299 | XKR7 | 2.444E-05 | -0.399 | - | - |
| 16624 | ENSP00000357906 | TNFAIP8L2 | 6.710E-06 | -0.399 | - | - |
| 16625 | ENSP00000294117 | GNG3 | 1.791E-05 | -0.399 | - | - |
| 16626 | ENSP00000416558 | FBXO24 | 4.399E-06 | -0.399 | - | - |
| 16627 | ENSP00000382104 | DOPEY2 | 1.320E-05 | -0.399 | - | - |
| 16628 | ENSP00000438205 | MANSC1 | 8.542E-06 | -0.399 | - | - |
| 16629 | ENSP00000362677 | WDR38 | 1.003E-05 | -0.399 | - | - |
| 16630 | ENSP00000362273 | ADPRHL2 | 7.481E-06 | -0.399 | - | - |
| 16631 | ENSP00000381821 | YPEL3 | 8.347E-06 | -0.399 | - | - |
| 16632 | ENSP00000363522 | OR13A1 | 4.083E-06 | -0.399 | - | - |
| 16633 | ENSP00000254072 | KRTAP9-8 | 3.762E-06 | -0.399 | - | - |
| 16634 | ENSP00000270077 | PSG9 | 1.488E-05 | -0.399 | - | - |
| 16635 | ENSP00000457512 | RNF31 | 1.869E-06 | -0.399 | - | - |
| 16636 | ENSP00000386156 | WIBG | 5.252E-06 | -0.399 | - | - |
| 16637 | ENSP00000356290 | MTHFD1L | 1.690E-05 | -0.399 | - | - |
| 16638 | ENSP00000385215 | KIAA1009 | 8.429E-06 | -0.399 | - | - |
| 16639 | ENSP00000346802 | PCDHB11 | 1.014E-05 | -0.400 | - | - |
| 16640 | ENSP00000373860 | ASPDH | 4.897E-06 | -0.400 | - | - |
| 16641 | ENSP00000381982 | FER1L6 | 1.023E-05 | -0.400 | - | - |
| 16642 | ENSP00000430075 | AMPD1 | 1.191E-05 | -0.400 | - | - |
| 16643 | ENSP00000376443 | ABCA5 | 6.572E-06 | -0.400 | - | - |
| 16644 | ENSP00000382120 | AIFM3 | 1.718E-05 | -0.400 | - | - |
| 16645 | ENSP00000218197 | SLC25A14 | 2.071E-05 | -0.400 | - | - |
| 16646 | ENSP00000358909 | SFXN2 | 7.017E-06 | -0.400 | - | - |
| 16647 | ENSP00000362656 | GOLGA1 | 6.510E-06 | -0.400 | - | - |
| 16648 | ENSP00000426906 | RP11-514O12.4 | 4.825E-06 | -0.400 | - | - |
| 16649 | ENSP00000298542 | FRMD7 | 1.516E-05 | -0.400 | - | - |
| 16650 | ENSP00000287152 | KIF6 | 1.011E-05 | -0.400 | - | - |
| 16651 | ENSP00000376827 | MKS1 | 7.491E-06 | -0.400 | - | - |
| 16652 | ENSP00000242248 | POLM | 1.498E-05 | -0.401 | - | - |
| 16653 | ENSP00000289407 | TSPAN33 | 1.592E-05 | -0.401 | - | - |
| 16654 | ENSP00000355387 | SCAMP5 | 6.973E-06 | -0.401 | - | - |
| 16655 | ENSP00000423067 | WDR36 | 1.548E-05 | -0.401 | - | - |
| 16656 | ENSP00000419279 | TTC26 | 1.809E-06 | -0.401 | - | - |
| 16657 | ENSP00000357556 | VENTX | 1.465E-05 | -0.401 | - | - |
| 16658 | ENSP00000353462 | CASS4 | 1.391E-05 | -0.401 | - | - |
| 16659 | ENSP00000387593 | TANC2 | 1.189E-05 | -0.401 | - | - |
| 16660 | ENSP00000319166 | CDH7 | 1.633E-05 | -0.401 | - | - |
| 16661 | ENSP00000244625 | TBCC | 1.727E-05 | -0.401 | - | - |
| 16662 | ENSP00000454021 | TGIF2-C20orf24 | 1.322E-05 | -0.401 | - | - |
| 16663 | ENSP00000472409 | ARL14EPL | 7.447E-06 | -0.401 | - | - |
| 16664 | ENSP00000223208 | CEP41 | 8.087E-06 | -0.401 | - | - |
| 16665 | ENSP00000468743 | CTD-3116E22.4 | 1.647E-06 | -0.401 | - | - |
| 16666 | ENSP00000351536 | QSOX2 | 1.079E-05 | -0.401 | - | - |
| 16667 | ENSP00000328992 | PAXBP1 | 8.794E-06 | -0.401 | - | - |
| 16668 | ENSP00000362249 | AK1 | 1.389E-05 | -0.401 | - | - |
| 16669 | ENSP00000449960 | CALCOCO1 | 5.133E-06 | -0.401 | - | - |
| 16670 | ENSP00000470389 | CTD-2287O16.3 | 7.447E-06 | -0.401 | - | - |
| 16671 | ENSP00000426978 | NXF5 | 8.732E-06 | -0.401 | - | - |
| 16672 | ENSP00000354109 | RGS22 | 5.023E-06 | -0.401 | - | - |
| 16673 | ENSP00000432931 | FAM86B1 | 7.383E-06 | -0.401 | - | - |
| 16674 | ENSP00000315070 | GJD4 | 1.319E-05 | -0.401 | - | - |
| 16675 | ENSP00000350704 | C1orf109 | 7.307E-06 | -0.401 | - | - |
| 16676 | ENSP00000389160 | NDUFA11 | 3.378E-06 | -0.402 | - | - |
| 16677 | ENSP00000345152 | TMEM120B | 1.583E-05 | -0.402 | - | - |
| 16678 | ENSP00000356071 | SERAC1 | 1.434E-05 | -0.402 | - | - |
| 16679 | ENSP00000240615 | TAS2R8 | 1.815E-05 | -0.402 | - | - |
| 16680 | ENSP00000438284 | PDE10A | 1.197E-05 | -0.402 | - | - |
| 16681 | ENSP00000367459 | KIAA0319 | 4.810E-06 | -0.402 | - | - |
| 16682 | ENSP00000365920 | GCNT1 | 7.274E-06 | -0.402 | - | - |
| 16683 | ENSP00000359423 | MTM1 | 1.521E-05 | -0.402 | - | - |
| 16684 | ENSP00000457386 | SMIM1 | 1.410E-05 | -0.402 | - | - |
| 16685 | ENSP00000452549 | YJEFN3 | 8.038E-06 | -0.402 | - | - |
| 16686 | ENSP00000219833 | SLC6A2 | 1.441E-05 | -0.402 | - | - |
| 16687 | ENSP00000416126 | PRAP1 | 6.200E-06 | -0.402 | - | - |
| 16688 | ENSP00000424363 | BBS5 | 4.863E-06 | -0.402 | - | - |
| 16689 | ENSP00000419195 | CAMKV | 1.392E-05 | -0.403 | - | - |
| 16690 | ENSP00000323686 | C15orf54 | 5.514E-06 | -0.403 | - | - |
| 16691 | ENSP00000441032 | GRAMD1A | 3.874E-06 | -0.403 | - | - |
| 16692 | ENSP00000363386 | FAM110D | 6.414E-06 | -0.403 | - | - |
| 16693 | ENSP00000371594 | GNG7 | 1.255E-05 | -0.403 | - | - |
| 16694 | ENSP00000434995 | CLP1 | 8.096E-06 | -0.403 | - | - |
| 16695 | ENSP00000264444 | MXD1 | 1.784E-05 | -0.403 | - | - |
| 16696 | ENSP00000400099 | FAM65A | 5.601E-06 | -0.403 | - | - |
| 16697 | ENSP00000365714 | TTYH1 | 9.931E-06 | -0.403 | - | - |
| 16698 | ENSP00000347324 | MSRB3 | 1.151E-05 | -0.403 | - | - |
| 16699 | ENSP00000224756 | CCSER2 | 1.305E-05 | -0.403 | - | - |
| 16700 | ENSP00000392760 | ARHGAP11B | 1.259E-05 | -0.403 | - | - |
| 16701 | ENSP00000401513 | STRC | 1.206E-05 | -0.403 | - | - |
| 16702 | ENSP00000369716 | CHD3 | 1.862E-05 | -0.403 | - | - |
| 16703 | ENSP00000354972 | NAA35 | 5.384E-06 | -0.403 | - | - |
| 16704 | ENSP00000433821 | RPS3 | 1.126E-05 | -0.403 | - | - |
| 16705 | ENSP00000384369 | METTL15 | 8.221E-06 | -0.403 | - | - |
| 16706 | ENSP00000368062 | ATAD3C | 4.209E-06 | -0.403 | - | - |
| 16707 | ENSP00000378756 | SLC16A9 | 5.281E-06 | -0.403 | - | - |
| 16708 | ENSP00000354660 | MAGEC2 | 1.430E-05 | -0.403 | - | - |
| 16709 | ENSP00000362948 | MED18 | 7.095E-06 | -0.403 | - | - |
| 16710 | ENSP00000334547 | TAL2 | 1.538E-05 | -0.403 | - | - |
| 16711 | ENSP00000354608 | PPT2 | 6.943E-06 | -0.403 | - | - |
| 16712 | ENSP00000346809 | PGAP1 | 1.117E-05 | -0.403 | - | - |
| 16713 | ENSP00000370023 | HADHA | 2.045E-05 | -0.403 | - | - |
| 16714 | ENSP00000362991 | XKR8 | 7.670E-06 | -0.403 | - | - |
| 16715 | ENSP00000354871 | PPEF1 | 1.525E-05 | -0.403 | - | - |
| 16716 | ENSP00000349525 | AHSA2 | 9.161E-06 | -0.404 | - | - |
| 16717 | ENSP00000259983 | C6orf52 | 1.416E-05 | -0.404 | - | - |
| 16718 | ENSP00000448888 | - | 3.671E-06 | -0.404 | - | - |
| 16719 | ENSP00000299155 | AMN | 1.501E-05 | -0.404 | - | - |
| 16720 | ENSP00000360485 | AKAP14 | 4.763E-06 | -0.404 | - | - |
| 16721 | ENSP00000317232 | FKBP10 | 1.361E-05 | -0.404 | - | - |
| 16722 | ENSP00000449598 | MYRFL | 2.858E-06 | -0.404 | - | - |
| 16723 | ENSP00000409075 | CYB5RL | 3.895E-06 | -0.404 | - | - |
| 16724 | ENSP00000290607 | STARD9 | 8.532E-06 | -0.404 | - | - |
| 16725 | ENSP00000426159 | C6orf223 | 4.978E-06 | -0.404 | - | - |
| 16726 | ENSP00000422496 | TTC39B | 8.392E-06 | -0.404 | - | - |
| 16727 | ENSP00000327234 | TMCO5A | 1.226E-05 | -0.404 | - | - |
| 16728 | ENSP00000373808 | FAM169A | 6.424E-06 | -0.405 | - | - |
| 16729 | ENSP00000360371 | SSBP3 | 1.106E-05 | -0.405 | - | - |
| 16730 | ENSP00000380157 | MOCS2 | 1.304E-05 | -0.405 | - | - |
| 16731 | ENSP00000413445 | KIAA1324L | 1.139E-05 | -0.405 | - | - |
| 16732 | ENSP00000336887 | SLC39A9 | 9.198E-06 | -0.405 | - | - |
| 16733 | ENSP00000428845 | VDAC3 | 8.316E-06 | -0.405 | - | - |
| 16734 | ENSP00000444565 | TMUB2 | 8.064E-06 | -0.405 | - | - |
| 16735 | ENSP00000379680 | NAGA | 9.121E-06 | -0.405 | - | - |
| 16736 | ENSP00000311291 | SLCO2A1 | 1.457E-05 | -0.405 | - | - |
| 16737 | ENSP00000268919 | KIF2B | 1.329E-05 | -0.405 | - | - |
| 16738 | ENSP00000397394 | C19orf25 | 5.894E-06 | -0.405 | - | - |
| 16739 | ENSP00000412673 | GABRR1 | 1.743E-05 | -0.405 | - | - |
| 16740 | ENSP00000356389 | GINM1 | 9.998E-06 | -0.405 | - | - |
| 16741 | ENSP00000369513 | ATP6AP1L | 2.132E-06 | -0.405 | - | - |
| 16742 | ENSP00000216471 | SAMD15 | 7.214E-06 | -0.405 | - | - |
| 16743 | ENSP00000358931 | TKTL1 | 1.716E-05 | -0.405 | - | - |
| 16744 | ENSP00000259963 | FAM8A1 | 2.035E-05 | -0.405 | - | - |
| 16745 | ENSP00000402698 | TMEM30C | 5.315E-06 | -0.405 | - | - |
| 16746 | ENSP00000363714 | TMEM245 | 5.695E-06 | -0.405 | - | - |
| 16747 | ENSP00000320634 | SLCO3A1 | 1.320E-05 | -0.405 | - | - |
| 16748 | ENSP00000333441 | TREX2 | 9.941E-06 | -0.405 | - | - |
| 16749 | ENSP00000467958 | AC006538.4 | 1.330E-05 | -0.405 | - | - |
| 16750 | ENSP00000265753 | EIF4H | 1.102E-05 | -0.405 | - | - |
| 16751 | ENSP00000361087 | SNCG | 1.512E-05 | -0.405 | - | - |
| 16752 | ENSP00000378733 | LBH | 5.018E-06 | -0.405 | - | - |
| 16753 | ENSP00000438349 | TRMT112 | 1.124E-05 | -0.406 | - | - |
| 16754 | ENSP00000418082 | RPL37A | 1.108E-05 | -0.406 | - | - |
| 16755 | ENSP00000387617 | SMCO2 | 1.598E-06 | -0.406 | - | - |
| 16756 | ENSP00000377265 | TFAP2B | 1.726E-05 | -0.406 | - | - |
| 16757 | ENSP00000295992 | PCOLCE2 | 1.331E-05 | -0.406 | - | - |
| 16758 | ENSP00000450337 | PPFIA2 | 4.341E-06 | -0.406 | - | - |
| 16759 | ENSP00000327557 | PAPL | 1.550E-05 | -0.406 | - | - |
| 16760 | ENSP00000425556 | PALLD | 1.032E-05 | -0.406 | - | - |
| 16761 | ENSP00000319126 | DEFB112 | 4.252E-06 | -0.406 | - | - |
| 16762 | ENSP00000401867 | MXD3 | 6.443E-06 | -0.406 | - | - |
| 16763 | ENSP00000280191 | SPATA4 | 1.653E-05 | -0.406 | - | - |
| 16764 | ENSP00000367125 | GPR153 | 7.749E-06 | -0.406 | - | - |
| 16765 | ENSP00000281081 | NUBPL | 1.530E-05 | -0.406 | - | - |
| 16766 | ENSP00000344041 | SYNGR4 | 1.322E-05 | -0.406 | - | - |
| 16767 | ENSP00000267594 | FAM181A | 2.101E-05 | -0.406 | - | - |
| 16768 | ENSP00000455510 | SIGLEC5 | 1.035E-05 | -0.406 | - | - |
| 16769 | ENSP00000357251 | SOGA3 | 1.149E-05 | -0.406 | - | - |
| 16770 | ENSP00000261369 | SNX24 | 1.272E-05 | -0.406 | - | - |
| 16771 | ENSP00000397239 | KDM4E | 1.017E-05 | -0.406 | - | - |
| 16772 | ENSP00000431876 | USP35 | 7.043E-06 | -0.406 | - | - |
| 16773 | ENSP00000216211 | UPK3A | 1.286E-05 | -0.406 | - | - |
| 16774 | ENSP00000365840 | ENTPD6 | 4.374E-06 | -0.407 | - | - |
| 16775 | ENSP00000297784 | TMC1 | 8.010E-06 | -0.407 | - | - |
| 16776 | ENSP00000357588 | FAM26E | 6.028E-06 | -0.407 | - | - |
| 16777 | ENSP00000260126 | SLCO5A1 | 1.732E-05 | -0.407 | - | - |
| 16778 | ENSP00000381542 | KRTAP5-10 | 2.042E-06 | -0.407 | - | - |
| 16779 | ENSP00000441858 | DMXL2 | 1.512E-05 | -0.407 | - | - |
| 16780 | ENSP00000359297 | NSDHL | 7.814E-06 | -0.407 | - | - |
| 16781 | ENSP00000363557 | MYOM3 | 1.678E-05 | -0.407 | - | - |
| 16782 | ENSP00000435289 | PAFAH1B2 | 8.080E-06 | -0.407 | - | - |
| 16783 | ENSP00000353373 | CUEDC1 | 5.010E-06 | -0.407 | - | - |
| 16784 | ENSP00000306564 | TMEM42 | 5.010E-06 | -0.407 | - | - |
| 16785 | ENSP00000333711 | LL0XNC01-19D8.1 | 9.646E-06 | -0.407 | - | - |
| 16786 | ENSP00000336783 | TNRC6C | 1.083E-05 | -0.407 | - | - |
| 16787 | ENSP00000360541 | CPT2 | 1.155E-05 | -0.407 | - | - |
| 16788 | ENSP00000366460 | PLXDC2 | 1.039E-05 | -0.407 | - | - |
| 16789 | ENSP00000405674 | NOL7 | 2.189E-06 | -0.407 | - | - |
| 16790 | ENSP00000387046 | PPM1M | 6.953E-06 | -0.408 | - | - |
| 16791 | ENSP00000255078 | IGHMBP2 | 1.269E-05 | -0.408 | - | - |
| 16792 | ENSP00000263284 | CCDC61 | 5.808E-06 | -0.408 | - | - |
| 16793 | ENSP00000360583 | ZYG11A | 2.526E-06 | -0.408 | - | - |
| 16794 | ENSP00000459356 | - | 2.768E-06 | -0.408 | - | - |
| 16795 | ENSP00000391942 | NECAP2 | 7.200E-06 | -0.408 | - | - |
| 16796 | ENSP00000232519 | C3orf14 | 9.572E-06 | -0.408 | - | - |
| 16797 | ENSP00000452252 | MIA2 | 1.505E-06 | -0.408 | - | - |
| 16798 | ENSP00000373734 | DENND5B | 4.827E-06 | -0.408 | - | - |
| 16799 | ENSP00000344403 | FSIP2 | 7.206E-06 | -0.408 | - | - |
| 16800 | ENSP00000362029 | CIZ1 | 6.151E-06 | -0.408 | - | - |
| 16801 | ENSP00000341030 | CSN2 | 1.384E-05 | -0.408 | - | - |
| 16802 | ENSP00000461269 | SCIMP | 5.956E-06 | -0.408 | - | - |
| 16803 | ENSP00000406012 | IFT140 | 5.662E-06 | -0.408 | - | - |
| 16804 | ENSP00000410396 | CMSS1 | 6.432E-06 | -0.408 | - | - |
| 16805 | ENSP00000265012 | GCNT2 | 1.404E-05 | -0.408 | - | - |
| 16806 | ENSP00000357480 | NUS1 | 7.055E-06 | -0.408 | - | - |
| 16807 | ENSP00000361835 | NXF2B | 1.280E-05 | -0.408 | - | - |
| 16808 | ENSP00000359782 | BEND6 | 3.041E-06 | -0.408 | - | - |
| 16809 | ENSP00000423660 | SH3TC2 | 5.627E-06 | -0.408 | - | - |
| 16810 | ENSP00000355955 | NENF | 1.105E-05 | -0.408 | - | - |
| 16811 | ENSP00000443191 | KLHL13 | 3.945E-06 | -0.408 | - | - |
| 16812 | ENSP00000335060 | HPDL | 1.482E-05 | -0.409 | - | - |
| 16813 | ENSP00000416706 | ACTBL2 | 2.481E-05 | -0.409 | - | - |
| 16814 | ENSP00000375476 | KRTAP12-4 | 2.082E-06 | -0.409 | - | - |
| 16815 | ENSP00000360035 | PPP1R3D | 7.628E-06 | -0.409 | - | - |
| 16816 | ENSP00000343248 | SBK1 | 1.391E-05 | -0.409 | - | - |
| 16817 | ENSP00000361914 | OXCT2 | 1.808E-05 | -0.409 | - | - |
| 16818 | ENSP00000427401 | SREK1IP1 | 6.038E-06 | -0.409 | - | - |
| 16819 | ENSP00000440594 | VNN3 | 1.028E-05 | -0.409 | - | - |
| 16820 | ENSP00000387209 | CEP19 | 2.342E-06 | -0.409 | - | - |
| 16821 | ENSP00000358554 | BCAS2 | 6.390E-06 | -0.409 | - | - |
| 16822 | ENSP00000349874 | NUDT13 | 1.214E-05 | -0.409 | - | - |
| 16823 | ENSP00000373370 | FLG2 | 6.767E-06 | -0.409 | - | - |
| 16824 | ENSP00000350353 | METTL20 | 8.629E-06 | -0.409 | - | - |
| 16825 | ENSP00000342576 | FAM221A | 4.171E-06 | -0.409 | - | - |
| 16826 | ENSP00000434655 | PCDHA8 | 5.239E-06 | -0.409 | - | - |
| 16827 | ENSP00000301891 | SLC22A11 | 1.508E-05 | -0.410 | - | - |
| 16828 | ENSP00000361025 | C9orf96 | 5.639E-06 | -0.410 | - | - |
| 16829 | ENSP00000351669 | MANEA | 8.190E-06 | -0.410 | - | - |
| 16830 | ENSP00000363216 | OGDHL | 1.159E-05 | -0.410 | - | - |
| 16831 | ENSP00000352097 | SPTSSB | 9.686E-06 | -0.410 | - | - |
| 16832 | ENSP00000256246 | TEX15 | 1.778E-05 | -0.410 | - | - |
| 16833 | ENSP00000363003 | PHF19 | 1.443E-05 | -0.410 | - | - |
| 16834 | ENSP00000445859 | MPHOSPH9 | 6.777E-06 | -0.410 | - | - |
| 16835 | ENSP00000298622 | JAKMIP3 | 1.681E-05 | -0.410 | - | - |
| 16836 | ENSP00000269209 | GAREM | 1.195E-05 | -0.410 | - | - |
| 16837 | ENSP00000346667 | WNK3 | 1.571E-05 | -0.410 | - | - |
| 16838 | ENSP00000338034 | SPRYD4 | 1.462E-05 | -0.410 | - | - |
| 16839 | ENSP00000369518 | ATRAID | 1.033E-05 | -0.410 | - | - |
| 16840 | ENSP00000304419 | OR2B2 | 1.633E-05 | -0.410 | - | - |
| 16841 | ENSP00000411471 | ABCF3 | 4.475E-06 | -0.411 | - | - |
| 16842 | ENSP00000362332 | TFAP2E | 9.378E-06 | -0.411 | - | - |
| 16843 | ENSP00000358571 | HIPK1 | 1.520E-05 | -0.411 | - | - |
| 16844 | ENSP00000344648 | SLC37A1 | 1.124E-05 | -0.411 | - | - |
| 16845 | ENSP00000328118 | MCF2L2 | 1.239E-05 | -0.411 | - | - |
| 16846 | ENSP00000354432 | ANKRD30A | 8.927E-06 | -0.411 | - | - |
| 16847 | ENSP00000419879 | CDS2 | 5.300E-06 | -0.411 | - | - |
| 16848 | ENSP00000252318 | GALNT8 | 8.003E-06 | -0.411 | - | - |
| 16849 | ENSP00000340979 | RNF175 | 1.160E-05 | -0.411 | - | - |
| 16850 | ENSP00000421686 | SPDYE2 | 5.083E-06 | -0.411 | - | - |
| 16851 | ENSP00000387641 | CAND2 | 5.143E-06 | -0.411 | - | - |
| 16852 | ENSP00000372210 | BET1L | 4.924E-06 | -0.411 | - | - |
| 16853 | ENSP00000361603 | C1orf50 | 4.198E-06 | -0.411 | - | - |
| 16854 | ENSP00000413632 | MCMDC2 | 8.086E-06 | -0.411 | - | - |
| 16855 | ENSP00000223862 | RLN1 | 2.025E-05 | -0.411 | - | - |
| 16856 | ENSP00000348471 | SH3YL1 | 7.213E-06 | -0.411 | - | - |
| 16857 | ENSP00000339484 | ZFAND4 | 1.894E-05 | -0.411 | - | - |
| 16858 | ENSP00000356134 | TFB1M | 7.064E-06 | -0.411 | - | - |
| 16859 | ENSP00000369099 | ADPRM | 7.901E-06 | -0.411 | - | - |
| 16860 | ENSP00000317331 | SSR4 | 9.967E-06 | -0.411 | - | - |
| 16861 | ENSP00000354891 | FAM163A | 8.871E-06 | -0.411 | - | - |
| 16862 | ENSP00000364580 | GRTP1 | 1.107E-05 | -0.411 | - | - |
| 16863 | ENSP00000418734 | MYSM1 | 1.130E-05 | -0.411 | - | - |
| 16864 | ENSP00000450710 | PPAN | 1.982E-05 | -0.411 | - | - |
| 16865 | ENSP00000333183 | MBD3L3 | 8.467E-06 | -0.412 | - | - |
| 16866 | ENSP00000368468 | MCUR1 | 6.513E-06 | -0.412 | - | - |
| 16867 | ENSP00000414237 | INTS2 | 3.025E-06 | -0.412 | - | - |
| 16868 | ENSP00000007735 | KRT33A | 1.789E-05 | -0.412 | - | - |
| 16869 | ENSP00000398789 | SNX13 | 4.819E-06 | -0.412 | - | - |
| 16870 | ENSP00000457868 | RTEL1-TNFRSF6B | 1.515E-05 | -0.412 | - | - |
| 16871 | ENSP00000386239 | TONSL | 9.095E-06 | -0.412 | - | - |
| 16872 | ENSP00000381489 | KRTAP4-1 | 2.020E-06 | -0.412 | - | - |
| 16873 | ENSP00000251691 | KIAA1244 | 8.670E-06 | -0.412 | - | - |
| 16874 | ENSP00000370419 | TTC38 | 4.572E-06 | -0.412 | - | - |
| 16875 | ENSP00000295083 | PQLC3 | 9.664E-06 | -0.413 | - | - |
| 16876 | ENSP00000349543 | PEX2 | 8.624E-06 | -0.413 | - | - |
| 16877 | ENSP00000231887 | EHHADH | 7.773E-06 | -0.413 | - | - |
| 16878 | ENSP00000368528 | APOO | 4.102E-06 | -0.413 | - | - |
| 16879 | ENSP00000354968 | C1orf191 | 4.114E-06 | -0.413 | - | - |
| 16880 | ENSP00000370930 | MRPL23 | 8.242E-06 | -0.413 | - | - |
| 16881 | ENSP00000464814 | COASY | 1.196E-05 | -0.413 | - | - |
| 16882 | ENSP00000365486 | DEFB123 | 1.104E-05 | -0.413 | - | - |
| 16883 | ENSP00000381693 | ZSWIM8 | 6.504E-06 | -0.413 | - | - |
| 16884 | ENSP00000398410 | KDM8 | 8.404E-06 | -0.413 | - | - |
| 16885 | ENSP00000311257 | STOX2 | 1.096E-05 | -0.413 | - | - |
| 16886 | ENSP00000337854 | HEXDC | 8.022E-06 | -0.413 | - | - |
| 16887 | ENSP00000382323 | OTOG | 5.535E-06 | -0.413 | - | - |
| 16888 | ENSP00000413064 | NAT8L | 2.581E-06 | -0.413 | - | - |
| 16889 | ENSP00000452237 | KRT86 | 4.460E-06 | -0.413 | - | - |
| 16890 | ENSP00000352734 | RNF217 | 8.258E-06 | -0.413 | - | - |
| 16891 | ENSP00000384416 | CCDC129 | 3.680E-06 | -0.413 | - | - |
| 16892 | ENSP00000305138 | FAM195A | 5.615E-06 | -0.413 | - | - |
| 16893 | ENSP00000302276 | HEXIM2 | 1.758E-05 | -0.413 | - | - |
| 16894 | ENSP00000391524 | DENND6B | 3.821E-06 | -0.413 | - | - |
| 16895 | ENSP00000349887 | ASNA1 | 5.368E-06 | -0.413 | - | - |
| 16896 | ENSP00000330384 | OR6A2 | 1.451E-05 | -0.413 | - | - |
| 16897 | ENSP00000381654 | HMGCLL1 | 6.409E-06 | -0.413 | - | - |
| 16898 | ENSP00000365567 | TM9SF2 | 1.195E-05 | -0.413 | - | - |
| 16899 | ENSP00000237275 | ZC2HC1B | 1.760E-05 | -0.413 | - | - |
| 16900 | ENSP00000355559 | B3GALNT2 | 1.140E-05 | -0.414 | - | - |
| 16901 | ENSP00000360245 | - | 2.455E-06 | -0.414 | - | - |
| 16902 | ENSP00000347188 | KCTD6 | 9.154E-06 | -0.414 | - | - |
| 16903 | ENSP00000358272 | NDUFAF4 | 5.578E-06 | -0.414 | - | - |
| 16904 | ENSP00000386487 | C14orf180 | 2.815E-06 | -0.414 | - | - |
| 16905 | ENSP00000282641 | A1CF | 9.535E-06 | -0.414 | - | - |
| 16906 | ENSP00000378635 | DRP2 | 5.762E-06 | -0.414 | - | - |
| 16907 | ENSP00000305596 | FAM71B | 7.627E-06 | -0.414 | - | - |
| 16908 | ENSP00000348645 | PXDNL | 1.370E-05 | -0.414 | - | - |
| 16909 | ENSP00000368678 | AGRN | 1.396E-05 | -0.414 | - | - |
| 16910 | ENSP00000352601 | LRP10 | 5.307E-06 | -0.414 | - | - |
| 16911 | ENSP00000418802 | NBPF11 | 3.534E-06 | -0.414 | - | - |
| 16912 | ENSP00000434364 | SLC6A5 | 1.153E-05 | -0.414 | - | - |
| 16913 | ENSP00000410925 | GPR42 | 6.634E-06 | -0.414 | - | - |
| 16914 | ENSP00000228820 | PARP11 | 2.066E-05 | -0.414 | - | - |
| 16915 | ENSP00000363232 | KIF12 | 1.199E-05 | -0.414 | - | - |
| 16916 | ENSP00000358999 | TMEM187 | 9.585E-06 | -0.414 | - | - |
| 16917 | ENSP00000352904 | NT5C1B | 8.988E-06 | -0.414 | - | - |
| 16918 | ENSP00000311657 | GRAMD2 | 6.147E-06 | -0.414 | - | - |
| 16919 | ENSP00000398803 | TSTA3 | 9.324E-06 | -0.414 | - | - |
| 16920 | ENSP00000275767 | TMEM140 | 1.760E-05 | -0.414 | - | - |
| 16921 | ENSP00000207457 | TEKT2 | 1.438E-05 | -0.414 | - | - |
| 16922 | ENSP00000356218 | CYB5R1 | 7.959E-06 | -0.415 | - | - |
| 16923 | ENSP00000320025 | CACNB2 | 1.400E-05 | -0.415 | - | - |
| 16924 | ENSP00000424151 | KCTD16 | 3.512E-06 | -0.415 | - | - |
| 16925 | ENSP00000373884 | RYR3 | 1.141E-05 | -0.415 | - | - |
| 16926 | ENSP00000389948 | NT5C3B | 2.417E-06 | -0.415 | - | - |
| 16927 | ENSP00000385142 | NRXN1 | 1.209E-05 | -0.415 | - | - |
| 16928 | ENSP00000386029 | GABRR2 | 1.690E-05 | -0.415 | - | - |
| 16929 | ENSP00000350050 | TMEM229B | 4.925E-06 | -0.415 | - | - |
| 16930 | ENSP00000307617 | GGNBP2 | 1.253E-05 | -0.415 | - | - |
| 16931 | ENSP00000377078 | AAMDC | 7.085E-06 | -0.415 | - | - |
| 16932 | ENSP00000331435 | MBD3L5 | 8.504E-06 | -0.415 | - | - |
| 16933 | ENSP00000351737 | TMEM126B | 4.549E-06 | -0.415 | - | - |
| 16934 | ENSP00000359224 | ALG14 | 4.042E-06 | -0.415 | - | - |
| 16935 | ENSP00000355022 | C16orf59 | 6.385E-06 | -0.415 | - | - |
| 16936 | ENSP00000335325 | CCDC73 | 1.327E-05 | -0.415 | - | - |
| 16937 | ENSP00000409016 | TBRG1 | 8.802E-06 | -0.415 | - | - |
| 16938 | ENSP00000417935 | AKR1CL1 | 2.836E-06 | -0.416 | - | - |
| 16939 | ENSP00000328977 | RPH3AL | 1.202E-05 | -0.416 | - | - |
| 16940 | ENSP00000294664 | WDR63 | 8.740E-06 | -0.416 | - | - |
| 16941 | ENSP00000343331 | LIPI | 9.239E-06 | -0.416 | - | - |
| 16942 | ENSP00000351345 | FAM150A | 5.180E-06 | -0.416 | - | - |
| 16943 | ENSP00000351856 | POTEC | 9.022E-06 | -0.416 | - | - |
| 16944 | ENSP00000334289 | C9orf85 | 2.833E-06 | -0.416 | - | - |
| 16945 | ENSP00000371037 | CATSPERD | 5.463E-06 | -0.416 | - | - |
| 16946 | ENSP00000345294 | TTLL13 | 1.198E-05 | -0.416 | - | - |
| 16947 | ENSP00000301599 | TMEM88 | 1.992E-05 | -0.416 | - | - |
| 16948 | ENSP00000229330 | HCFC2 | 8.443E-06 | -0.416 | - | - |
| 16949 | ENSP00000381968 | SPTLC3 | 1.232E-05 | -0.416 | - | - |
| 16950 | ENSP00000250366 | KLK9 | 1.682E-05 | -0.416 | - | - |
| 16951 | ENSP00000370555 | GYG2 | 1.108E-05 | -0.416 | - | - |
| 16952 | ENSP00000367658 | ACTRT2 | 1.627E-05 | -0.416 | - | - |
| 16953 | ENSP00000273980 | TBCK | 1.111E-05 | -0.416 | - | - |
| 16954 | ENSP00000383698 | SLC2A7 | 1.379E-05 | -0.416 | - | - |
| 16955 | ENSP00000372576 | CCDC178 | 4.766E-06 | -0.416 | - | - |
| 16956 | ENSP00000456881 | TMX2-CTNND1 | 1.874E-06 | -0.417 | - | - |
| 16957 | ENSP00000355028 | DZIP3 | 8.471E-06 | -0.417 | - | - |
| 16958 | ENSP00000367934 | UQCRQ | 8.847E-06 | -0.417 | - | - |
| 16959 | ENSP00000256404 | PEBP4 | 1.554E-05 | -0.417 | - | - |
| 16960 | ENSP00000300714 | ENTHD2 | 1.328E-05 | -0.417 | - | - |
| 16961 | ENSP00000241041 | PEX16 | 8.937E-06 | -0.417 | - | - |
| 16962 | ENSP00000354543 | TSC22D2 | 1.336E-05 | -0.417 | - | - |
| 16963 | ENSP00000330190 | POU6F1 | 1.545E-05 | -0.417 | - | - |
| 16964 | ENSP00000358312 | PEX11B | 8.955E-06 | -0.417 | - | - |
| 16965 | ENSP00000351740 | FAM114A1 | 5.237E-06 | -0.417 | - | - |
| 16966 | ENSP00000365462 | PCCA | 1.096E-05 | -0.417 | - | - |
| 16967 | ENSP00000310146 | AP000769.1 | 1.505E-05 | -0.417 | - | - |
| 16968 | ENSP00000308873 | CCDC74B | 1.329E-05 | -0.417 | - | - |
| 16969 | ENSP00000354580 | MRPL21 | 5.827E-06 | -0.417 | - | - |
| 16970 | ENSP00000363211 | TIMM23B | 3.040E-06 | -0.417 | - | - |
| 16971 | ENSP00000391167 | DENND4A | 1.397E-05 | -0.417 | - | - |
| 16972 | ENSP00000436557 | PCDHA5 | 3.803E-06 | -0.417 | - | - |
| 16973 | ENSP00000359939 | EXOSC1 | 8.187E-06 | -0.417 | - | - |
| 16974 | ENSP00000362395 | ITM2A | 7.725E-06 | -0.418 | - | - |
| 16975 | ENSP00000355672 | IBA57 | 3.748E-06 | -0.418 | - | - |
| 16976 | ENSP00000368799 | KBTBD6 | 3.613E-06 | -0.418 | - | - |
| 16977 | ENSP00000362682 | OLFML2A | 3.790E-06 | -0.418 | - | - |
| 16978 | ENSP00000386807 | C4orf48 | 6.859E-06 | -0.418 | - | - |
| 16979 | ENSP00000366656 | BX255923.1 | 3.759E-06 | -0.418 | - | - |
| 16980 | ENSP00000327386 | PRSS57 | 1.326E-05 | -0.418 | - | - |
| 16981 | ENSP00000414202 | PPP6R1 | 1.001E-05 | -0.418 | - | - |
| 16982 | ENSP00000387694 | SLC12A5 | 1.350E-05 | -0.418 | - | - |
| 16983 | ENSP00000369739 | BPHL | 2.551E-06 | -0.418 | - | - |
| 16984 | ENSP00000323926 | TRPM8 | 9.029E-06 | -0.418 | - | - |
| 16985 | ENSP00000396068 | UBR3 | 5.512E-06 | -0.418 | - | - |
| 16986 | ENSP00000230381 | PRPH2 | 1.433E-05 | -0.418 | - | - |
| 16987 | ENSP00000405708 | CCDC39 | 7.052E-06 | -0.418 | - | - |
| 16988 | ENSP00000345702 | NFYA | 1.453E-05 | -0.418 | - | - |
| 16989 | ENSP00000454786 | RP11-152F13.10 | 7.894E-06 | -0.419 | - | - |
| 16990 | ENSP00000450924 | NKX1-2 | 6.588E-06 | -0.419 | - | - |
| 16991 | ENSP00000293599 | AQP5 | 1.283E-05 | -0.419 | - | - |
| 16992 | ENSP00000364398 | HABP4 | 1.037E-05 | -0.419 | - | - |
| 16993 | ENSP00000384198 | INO80D | 8.991E-06 | -0.419 | - | - |
| 16994 | ENSP00000389934 | EXOC5 | 1.026E-05 | -0.419 | - | - |
| 16995 | ENSP00000436005 | TRAPPC4 | 6.762E-06 | -0.419 | - | - |
| 16996 | ENSP00000363041 | CISD1 | 7.279E-06 | -0.419 | - | - |
| 16997 | ENSP00000245551 | MIF4GD | 1.124E-05 | -0.419 | - | - |
| 16998 | ENSP00000371040 | RLN2 | 5.565E-06 | -0.419 | - | - |
| 16999 | ENSP00000239125 | C10orf95 | 8.495E-06 | -0.419 | - | - |
| 17000 | ENSP00000337240 | MFSD11 | 5.190E-06 | -0.419 | - | - |
| 17001 | ENSP00000398105 | CHAC1 | 6.310E-06 | -0.420 | - | - |
| 17002 | ENSP00000356214 | MYCT1 | 2.631E-06 | -0.420 | - | - |
| 17003 | ENSP00000291672 | SPATC1L | 8.787E-06 | -0.420 | - | - |
| 17004 | ENSP00000414109 | HJURP | 5.593E-06 | -0.420 | - | - |
| 17005 | ENSP00000380793 | ALG3 | 1.118E-05 | -0.420 | - | - |
| 17006 | ENSP00000225899 | KRT32 | 8.389E-06 | -0.420 | - | - |
| 17007 | ENSP00000352463 | TCF20 | 1.221E-05 | -0.420 | - | - |
| 17008 | ENSP00000320207 | GOLGA6L1 | 3.483E-06 | -0.420 | - | - |
| 17009 | ENSP00000367486 | MEIG1 | 9.605E-06 | -0.420 | - | - |
| 17010 | ENSP00000365630 | RASEF | 1.137E-05 | -0.420 | - | - |
| 17011 | ENSP00000391447 | ASXL2 | 7.714E-06 | -0.420 | - | - |
| 17012 | ENSP00000350409 | UNC50 | 8.903E-06 | -0.420 | - | - |
| 17013 | ENSP00000290295 | HOXB13 | 1.585E-05 | -0.420 | - | - |
| 17014 | ENSP00000300618 | C17orf78 | 1.121E-05 | -0.420 | - | - |
| 17015 | ENSP00000373277 | RBMS3 | 9.014E-06 | -0.420 | - | - |
| 17016 | ENSP00000412064 | PRR13 | 5.044E-06 | -0.421 | - | - |
| 17017 | ENSP00000302629 | CHSY3 | 1.426E-05 | -0.421 | - | - |
| 17018 | ENSP00000303518 | ANKRD49 | 8.840E-06 | -0.421 | - | - |
| 17019 | ENSP00000363709 | FRRS1L | 1.202E-05 | -0.421 | - | - |
| 17020 | ENSP00000373413 | UFSP1 | 4.033E-06 | -0.421 | - | - |
| 17021 | ENSP00000316130 | SYNE4 | 1.536E-05 | -0.421 | - | - |
| 17022 | ENSP00000366826 | ANKRD20A2 | 6.658E-06 | -0.421 | - | - |
| 17023 | ENSP00000364627 | NXPE4 | 2.647E-06 | -0.421 | - | - |
| 17024 | ENSP00000377747 | CHD2 | 1.367E-05 | -0.421 | - | - |
| 17025 | ENSP00000369251 | MAB21L1 | 7.974E-06 | -0.421 | - | - |
| 17026 | ENSP00000398391 | CC2D2A | 4.533E-06 | -0.421 | - | - |
| 17027 | ENSP00000328923 | KANK3 | 8.197E-06 | -0.421 | - | - |
| 17028 | ENSP00000253354 | BPIFB1 | 1.821E-05 | -0.421 | - | - |
| 17029 | ENSP00000342609 | ACER2 | 1.193E-05 | -0.421 | - | - |
| 17030 | ENSP00000358019 | NSMCE4A | 6.932E-06 | -0.421 | - | - |
| 17031 | ENSP00000328079 | WDR53 | 1.075E-05 | -0.422 | - | - |
| 17032 | ENSP00000359286 | PNMA3 | 7.758E-06 | -0.422 | - | - |
| 17033 | ENSP00000378920 | SLC26A6 | 1.368E-05 | -0.422 | - | - |
| 17034 | ENSP00000223273 | YAE1D1 | 1.728E-05 | -0.422 | - | - |
| 17035 | ENSP00000264819 | MIER2 | 9.252E-06 | -0.422 | - | - |
| 17036 | ENSP00000432695 | KIAA1456 | 4.562E-06 | -0.422 | - | - |
| 17037 | ENSP00000369134 | PDHA1 | 1.114E-05 | -0.422 | - | - |
| 17038 | ENSP00000378166 | TMCO6 | 3.781E-06 | -0.422 | - | - |
| 17039 | ENSP00000417330 | KRTAP5-7 | 3.100E-06 | -0.422 | - | - |
| 17040 | ENSP00000349677 | SPOCK3 | 1.124E-05 | -0.422 | - | - |
| 17041 | ENSP00000340088 | THEG | 1.276E-05 | -0.422 | - | - |
| 17042 | ENSP00000249079 | C22orf23 | 1.258E-05 | -0.422 | - | - |
| 17043 | ENSP00000334714 | IGFN1 | 1.078E-05 | -0.422 | - | - |
| 17044 | ENSP00000274710 | PSD2 | 9.066E-06 | -0.422 | - | - |
| 17045 | ENSP00000343656 | RAPGEF5 | 1.001E-05 | -0.422 | - | - |
| 17046 | ENSP00000311648 | UGT8 | 1.039E-05 | -0.423 | - | - |
| 17047 | ENSP00000230256 | UNC93A | 1.674E-05 | -0.423 | - | - |
| 17048 | ENSP00000438685 | PCDHB17 | 2.879E-06 | -0.423 | - | - |
| 17049 | ENSP00000364150 | GALNT12 | 9.593E-06 | -0.423 | - | - |
| 17050 | ENSP00000417050 | TM4SF2 | 3.195E-06 | -0.423 | - | - |
| 17051 | ENSP00000386385 | PXMP4 | 6.647E-06 | -0.423 | - | - |
| 17052 | ENSP00000369080 | SPEF1 | 5.905E-06 | -0.423 | - | - |
| 17053 | ENSP00000358956 | IRAK1BP1 | 1.039E-05 | -0.423 | - | - |
| 17054 | ENSP00000369785 | PIR | 8.869E-06 | -0.423 | - | - |
| 17055 | ENSP00000389543 | TSRM | 1.299E-06 | -0.423 | - | - |
| 17056 | ENSP00000389998 | TMEM67 | 7.358E-06 | -0.423 | - | - |
| 17057 | ENSP00000445077 | EIF2S3L | 4.425E-06 | -0.423 | - | - |
| 17058 | ENSP00000359772 | CT45A2 | 2.896E-06 | -0.423 | - | - |
| 17059 | ENSP00000334044 | UBL4B | 1.788E-05 | -0.423 | - | - |
| 17060 | ENSP00000264968 | MGAT4A | 1.499E-05 | -0.423 | - | - |
| 17061 | ENSP00000418193 | SPRR2F | 1.171E-05 | -0.423 | - | - |
| 17062 | ENSP00000229771 | TULP1 | 1.639E-05 | -0.424 | - | - |
| 17063 | ENSP00000371870 | DAZ2 | 8.732E-06 | -0.424 | - | - |
| 17064 | ENSP00000323047 | MTG1 | 5.627E-06 | -0.424 | - | - |
| 17065 | ENSP00000324551 | SSUH2 | 8.337E-06 | -0.424 | - | - |
| 17066 | ENSP00000269878 | CIB3 | 1.308E-05 | -0.424 | - | - |
| 17067 | ENSP00000371169 | RCL1 | 6.349E-06 | -0.424 | - | - |
| 17068 | ENSP00000419851 | GMPS | 2.966E-05 | -0.424 | - | - |
| 17069 | ENSP00000270238 | LMTK3 | 1.626E-05 | -0.424 | - | - |
| 17070 | ENSP00000432481 | NAALAD2 | 6.514E-06 | -0.424 | - | - |
| 17071 | ENSP00000345229 | DNER | 1.244E-05 | -0.424 | - | - |
| 17072 | ENSP00000382133 | DNA2 | 8.825E-06 | -0.424 | - | - |
| 17073 | ENSP00000452762 | ETFA | 1.224E-05 | -0.424 | - | - |
| 17074 | ENSP00000342991 | CLYBL | 8.890E-06 | -0.424 | - | - |
| 17075 | ENSP00000377807 | FAM122A | 7.355E-06 | -0.424 | - | - |
| 17076 | ENSP00000319817 | OPA3 | 1.129E-05 | -0.424 | - | - |
| 17077 | ENSP00000301031 | C16orf55 | 5.434E-06 | -0.425 | - | - |
| 17078 | ENSP00000447537 | BLOC1S1 | 6.210E-06 | -0.425 | - | - |
| 17079 | ENSP00000353742 | KRTAP26-1 | 4.551E-06 | -0.425 | - | - |
| 17080 | ENSP00000376957 | DENND2C | 1.618E-05 | -0.425 | - | - |
| 17081 | ENSP00000394051 | NOSTRIN | 6.610E-06 | -0.425 | - | - |
| 17082 | ENSP00000354080 | SCUBE1 | 1.228E-05 | -0.425 | - | - |
| 17083 | ENSP00000417602 | MRPL47 | 3.854E-06 | -0.425 | - | - |
| 17084 | ENSP00000393854 | CENPBD1 | 2.764E-06 | -0.425 | - | - |
| 17085 | ENSP00000410157 | ABTB2 | 4.992E-06 | -0.425 | - | - |
| 17086 | ENSP00000455643 | ST20-MTHFS | 5.632E-06 | -0.425 | - | - |
| 17087 | ENSP00000465894 | MPPE1 | 2.487E-06 | -0.425 | - | - |
| 17088 | ENSP00000029410 | B4GALT7 | 7.761E-06 | -0.425 | - | - |
| 17089 | ENSP00000297267 | FNDC1 | 1.432E-05 | -0.425 | - | - |
| 17090 | ENSP00000301159 | LIN37 | 1.419E-05 | -0.425 | - | - |
| 17091 | ENSP00000359098 | COX7A2 | 3.176E-06 | -0.425 | - | - |
| 17092 | ENSP00000345689 | RAB5C | 1.094E-05 | -0.425 | - | - |
| 17093 | ENSP00000362268 | APOOL | 5.163E-06 | -0.425 | - | - |
| 17094 | ENSP00000442518 | SHANK3 | 1.901E-05 | -0.425 | - | - |
| 17095 | ENSP00000297623 | C9orf24 | 6.408E-06 | -0.425 | - | - |
| 17096 | ENSP00000398277 | GCNT6 | 3.897E-06 | -0.426 | - | - |
| 17097 | ENSP00000362094 | GTSF1L | 1.048E-05 | -0.426 | - | - |
| 17098 | ENSP00000389625 | OR2M3 | 1.010E-05 | -0.426 | - | - |
| 17099 | ENSP00000435342 | RTL1 | 3.930E-06 | -0.426 | - | - |
| 17100 | ENSP00000313384 | OR1E1 | 5.603E-06 | -0.426 | - | - |
| 17101 | ENSP00000347930 | TMEM30B | 7.986E-06 | -0.426 | - | - |
| 17102 | ENSP00000280358 | TEX12 | 9.186E-06 | -0.426 | - | - |
| 17103 | ENSP00000386918 | KDELR3 | 9.244E-06 | -0.426 | - | - |
| 17104 | ENSP00000329165 | KRT36 | 1.295E-05 | -0.426 | - | - |
| 17105 | ENSP00000319388 | MAMDC4 | 7.406E-06 | -0.426 | - | - |
| 17106 | ENSP00000387683 | UGT2B11 | 4.513E-06 | -0.426 | - | - |
| 17107 | ENSP00000393583 | AZI1 | 9.300E-06 | -0.426 | - | - |
| 17108 | ENSP00000282226 | FAM151B | 9.763E-06 | -0.426 | - | - |
| 17109 | ENSP00000382507 | UBXN2B | 8.280E-06 | -0.426 | - | - |
| 17110 | ENSP00000376799 | C11orf65 | 4.569E-06 | -0.426 | - | - |
| 17111 | ENSP00000360803 | DNLZ | 8.277E-06 | -0.427 | - | - |
| 17112 | ENSP00000295156 | VSNL1 | 1.311E-05 | -0.427 | - | - |
| 17113 | ENSP00000268533 | NUDT7 | 9.883E-06 | -0.427 | - | - |
| 17114 | ENSP00000405620 | DHX30 | 6.605E-06 | -0.427 | - | - |
| 17115 | ENSP00000384164 | KIF16B | 8.986E-06 | -0.427 | - | - |
| 17116 | ENSP00000342924 | MCPH1 | 1.172E-05 | -0.427 | - | - |
| 17117 | ENSP00000454454 | RP11-512M8.5 | 5.366E-06 | -0.427 | - | - |
| 17118 | ENSP00000354204 | TOMM20L | 5.277E-06 | -0.427 | - | - |
| 17119 | ENSP00000424126 | MCTP1 | 7.889E-06 | -0.427 | - | - |
| 17120 | ENSP00000362085 | C9orf16 | 3.963E-06 | -0.427 | - | - |
| 17121 | ENSP00000342445 | CLDN4 | 1.263E-06 | -0.427 | - | - |
| 17122 | ENSP00000242819 | CCDC70 | 9.452E-06 | -0.427 | - | - |
| 17123 | ENSP00000365116 | CTRC | 1.471E-05 | -0.427 | - | - |
| 17124 | ENSP00000394770 | CMYA5 | 7.391E-06 | -0.427 | - | - |
| 17125 | ENSP00000301698 | PRR25 | 4.552E-06 | -0.427 | - | - |
| 17126 | ENSP00000341539 | TCAIM | 1.160E-05 | -0.427 | - | - |
| 17127 | ENSP00000216133 | CBX7 | 1.480E-05 | -0.428 | - | - |
| 17128 | ENSP00000345774 | ALDH3A2 | 1.340E-05 | -0.428 | - | - |
| 17129 | ENSP00000272321 | WDPCP | 1.380E-05 | -0.428 | - | - |
| 17130 | ENSP00000360498 | MRPL41 | 6.523E-06 | -0.428 | - | - |
| 17131 | ENSP00000349291 | TBC1D9B | 4.781E-06 | -0.428 | - | - |
| 17132 | ENSP00000257663 | TMEM60 | 1.304E-05 | -0.428 | - | - |
| 17133 | ENSP00000406209 | EPN1 | 7.465E-06 | -0.428 | - | - |
| 17134 | ENSP00000426638 | ETFDH | 7.424E-06 | -0.428 | - | - |
| 17135 | ENSP00000221538 | RSPH6A | 1.026E-05 | -0.428 | - | - |
| 17136 | ENSP00000355121 | ITPRIPL1 | 4.099E-06 | -0.428 | - | - |
| 17137 | ENSP00000374390 | GPR149 | 9.105E-06 | -0.428 | - | - |
| 17138 | ENSP00000375413 | AC011841.1 | 4.013E-06 | -0.428 | - | - |
| 17139 | ENSP00000358064 | RPRD2 | 9.035E-06 | -0.428 | - | - |
| 17140 | ENSP00000370531 | ACOT6 | 1.321E-05 | -0.428 | - | - |
| 17141 | ENSP00000369141 | APTX | 1.035E-05 | -0.428 | - | - |
| 17142 | ENSP00000416168 | TRANK1 | 6.512E-06 | -0.428 | - | - |
| 17143 | ENSP00000358854 | TAF5 | 9.215E-06 | -0.428 | - | - |
| 17144 | ENSP00000327133 | SLC35A4 | 5.211E-06 | -0.428 | - | - |
| 17145 | ENSP00000398177 | KCTD7 | 9.394E-06 | -0.428 | - | - |
| 17146 | ENSP00000386190 | C2orf16 | 4.476E-06 | -0.428 | - | - |
| 17147 | ENSP00000312284 | SPESP1 | 1.507E-05 | -0.428 | - | - |
| 17148 | ENSP00000313243 | HSF5 | 1.358E-05 | -0.429 | - | - |
| 17149 | ENSP00000364320 | MRTO4 | 7.878E-06 | -0.429 | - | - |
| 17150 | ENSP00000370258 | EIF3CL | 5.479E-06 | -0.429 | - | - |
| 17151 | ENSP00000367151 | PCDH17 | 1.166E-05 | -0.429 | - | - |
| 17152 | ENSP00000356432 | FAM5C | 8.063E-06 | -0.429 | - | - |
| 17153 | ENSP00000330746 | KRTAP20-2 | 1.244E-05 | -0.429 | - | - |
| 17154 | ENSP00000349270 | HBM | 5.628E-06 | -0.429 | - | - |
| 17155 | ENSP00000410403 | RAB34 | 1.354E-05 | -0.429 | - | - |
| 17156 | ENSP00000366876 | PHF13 | 9.195E-06 | -0.429 | - | - |
| 17157 | ENSP00000457656 | CYB5RL | 4.310E-06 | -0.429 | - | - |
| 17158 | ENSP00000456272 | TRIM59 | 8.105E-06 | -0.429 | - | - |
| 17159 | ENSP00000372059 | IFITM5 | 1.088E-05 | -0.429 | - | - |
| 17160 | ENSP00000377696 | NIT2 | 5.480E-06 | -0.429 | - | - |
| 17161 | ENSP00000378161 | ACYP2 | 6.161E-06 | -0.429 | - | - |
| 17162 | ENSP00000362687 | DDX50 | 6.169E-06 | -0.429 | - | - |
| 17163 | ENSP00000377303 | RENBP | 1.048E-05 | -0.430 | - | - |
| 17164 | ENSP00000234392 | VAX2 | 2.056E-05 | -0.430 | - | - |
| 17165 | ENSP00000368732 | - | 3.716E-06 | -0.430 | - | - |
| 17166 | ENSP00000356272 | RMND1 | 5.305E-06 | -0.430 | - | - |
| 17167 | ENSP00000358120 | PLEKHO1 | 6.450E-06 | -0.430 | - | - |
| 17168 | ENSP00000393559 | PACS2 | 6.873E-06 | -0.430 | - | - |
| 17169 | ENSP00000296444 | SHISA5 | 1.096E-05 | -0.430 | - | - |
| 17170 | ENSP00000318429 | CCDC114 | 7.720E-06 | -0.430 | - | - |
| 17171 | ENSP00000431459 | FAM89B | 5.690E-06 | -0.430 | - | - |
| 17172 | ENSP00000378451 | PHF15 | 1.044E-05 | -0.430 | - | - |
| 17173 | ENSP00000363392 | SNX12 | 6.137E-06 | -0.430 | - | - |
| 17174 | ENSP00000331298 | UBALD2 | 1.056E-05 | -0.430 | - | - |
| 17175 | ENSP00000326445 | SHISA3 | 6.033E-06 | -0.430 | - | - |
| 17176 | ENSP00000350310 | ATP2B4 | 9.047E-06 | -0.430 | - | - |
| 17177 | ENSP00000388431 | TMEM251 | 8.281E-06 | -0.431 | - | - |
| 17178 | ENSP00000359928 | TNNI3K | 1.265E-05 | -0.431 | - | - |
| 17179 | ENSP00000354053 | GLT8D2 | 7.286E-06 | -0.431 | - | - |
| 17180 | ENSP00000355551 | GPR137B | 5.654E-06 | -0.431 | - | - |
| 17181 | ENSP00000386126 | UMODL1 | 4.733E-06 | -0.431 | - | - |
| 17182 | ENSP00000311181 | - | 6.493E-06 | -0.431 | - | - |
| 17183 | ENSP00000417654 | SLC35A5 | 3.173E-06 | -0.431 | - | - |
| 17184 | ENSP00000377549 | FABP6 | 1.430E-05 | -0.431 | - | - |
| 17185 | ENSP00000319170 | DHFRL1 | 8.422E-06 | -0.431 | - | - |
| 17186 | ENSP00000420714 | MRPS14 | 7.267E-06 | -0.431 | - | - |
| 17187 | ENSP00000359520 | ENTPD7 | 5.182E-06 | -0.431 | - | - |
| 17188 | ENSP00000386121 | BTBD9 | 5.762E-06 | -0.431 | - | - |
| 17189 | ENSP00000362616 | NAP1L2 | 1.102E-05 | -0.431 | - | - |
| 17190 | ENSP00000386538 | TBC1D10B | 5.005E-06 | -0.431 | - | - |
| 17191 | ENSP00000295055 | CAPN13 | 1.436E-05 | -0.431 | - | - |
| 17192 | ENSP00000313886 | TCEANC | 7.166E-06 | -0.431 | - | - |
| 17193 | ENSP00000278947 | SCN2B | 1.518E-05 | -0.431 | - | - |
| 17194 | ENSP00000406157 | PAPSS2 | 2.984E-05 | -0.431 | - | - |
| 17195 | ENSP00000367923 | SUCLA2 | 9.847E-06 | -0.431 | - | - |
| 17196 | ENSP00000382518 | SRL | 1.249E-05 | -0.432 | - | - |
| 17197 | ENSP00000357336 | RUSC1 | 9.304E-06 | -0.432 | - | - |
| 17198 | ENSP00000393557 | SLC4A4 | 1.790E-05 | -0.432 | - | - |
| 17199 | ENSP00000329137 | FAM132A | 8.596E-06 | -0.432 | - | - |
| 17200 | ENSP00000363329 | SLC31A1 | 1.359E-05 | -0.432 | - | - |
| 17201 | ENSP00000271835 | CRNN | 9.258E-06 | -0.432 | - | - |
| 17202 | ENSP00000359464 | OGFRL1 | 6.628E-06 | -0.432 | - | - |
| 17203 | ENSP00000323096 | AMIGO3 | 5.384E-06 | -0.432 | - | - |
| 17204 | ENSP00000346265 | SYT5 | 1.411E-05 | -0.432 | - | - |
| 17205 | ENSP00000290079 | TMEM141 | 4.374E-06 | -0.432 | - | - |
| 17206 | ENSP00000348757 | CCDC151 | 4.177E-06 | -0.432 | - | - |
| 17207 | ENSP00000379568 | ACBD5 | 2.770E-06 | -0.432 | - | - |
| 17208 | ENSP00000424870 | SRP19 | 3.232E-06 | -0.432 | - | - |
| 17209 | ENSP00000386264 | TMEM237 | 5.043E-06 | -0.432 | - | - |
| 17210 | ENSP00000390111 | PLSCR5 | 5.118E-06 | -0.432 | - | - |
| 17211 | ENSP00000343890 | GLTPD1 | 8.608E-06 | -0.432 | - | - |
| 17212 | ENSP00000346063 | RPL7L1 | 8.363E-06 | -0.432 | - | - |
| 17213 | ENSP00000372112 | FAHD1 | 5.301E-06 | -0.432 | - | - |
| 17214 | ENSP00000360214 | OPALIN | 7.655E-06 | -0.432 | - | - |
| 17215 | ENSP00000382058 | POLR3G | 4.402E-06 | -0.432 | - | - |
| 17216 | ENSP00000311827 | MSL2 | 1.031E-05 | -0.433 | - | - |
| 17217 | ENSP00000421871 | C5orf20 | 2.191E-06 | -0.433 | - | - |
| 17218 | ENSP00000416250 | EIF2B1 | 9.291E-06 | -0.433 | - | - |
| 17219 | ENSP00000365625 | DHX16 | 1.097E-05 | -0.433 | - | - |
| 17220 | ENSP00000401018 | GINS3 | 3.728E-06 | -0.433 | - | - |
| 17221 | ENSP00000309786 | FAM21D | 5.368E-06 | -0.433 | - | - |
| 17222 | ENSP00000382204 | JMJD1C | 7.489E-06 | -0.433 | - | - |
| 17223 | ENSP00000350331 | ASB13 | 8.027E-06 | -0.433 | - | - |
| 17224 | ENSP00000379531 | TMC5 | 6.330E-06 | -0.433 | - | - |
| 17225 | ENSP00000383719 | UBE2J2 | 6.644E-06 | -0.433 | - | - |
| 17226 | ENSP00000363939 | PPP3R2 | 1.994E-05 | -0.433 | - | - |
| 17227 | ENSP00000350651 | ERI2 | 8.759E-06 | -0.433 | - | - |
| 17228 | ENSP00000354583 | MFAP3L | 5.906E-06 | -0.433 | - | - |
| 17229 | ENSP00000456850 | THEGL | 3.388E-06 | -0.434 | - | - |
| 17230 | ENSP00000280734 | TMEM86A | 1.155E-05 | -0.434 | - | - |
| 17231 | ENSP00000387365 | PWP1 | 1.303E-05 | -0.434 | - | - |
| 17232 | ENSP00000285871 | CCDC146 | 5.024E-06 | -0.434 | - | - |
| 17233 | ENSP00000346001 | RPL3 | 6.068E-06 | -0.434 | - | - |
| 17234 | ENSP00000355958 | DTL | 8.024E-06 | -0.434 | - | - |
| 17235 | ENSP00000343686 | BTBD8 | 8.094E-06 | -0.434 | - | - |
| 17236 | ENSP00000318085 | HINFP | 1.478E-05 | -0.434 | - | - |
| 17237 | ENSP00000362869 | MORN5 | 1.080E-05 | -0.434 | - | - |
| 17238 | ENSP00000244669 | APOBEC2 | 1.654E-05 | -0.434 | - | - |
| 17239 | ENSP00000463576 | FAM230A | 9.070E-06 | -0.434 | - | - |
| 17240 | ENSP00000371786 | ONECUT3 | 6.522E-06 | -0.434 | - | - |
| 17241 | ENSP00000261448 | CASQ2 | 1.765E-05 | -0.434 | - | - |
| 17242 | ENSP00000360483 | TMEM48 | 9.218E-06 | -0.434 | - | - |
| 17243 | ENSP00000354571 | RNF121 | 5.272E-06 | -0.434 | - | - |
| 17244 | ENSP00000416050 | TM2D2 | 7.022E-06 | -0.434 | - | - |
| 17245 | ENSP00000427802 | NACA2 | 6.498E-06 | -0.434 | - | - |
| 17246 | ENSP00000395650 | KANK2 | 8.916E-06 | -0.434 | - | - |
| 17247 | ENSP00000363988 | ALDOB | 1.129E-05 | -0.434 | - | - |
| 17248 | ENSP00000361998 | TAF7L | 7.058E-06 | -0.435 | - | - |
| 17249 | ENSP00000417656 | COX7B | 7.511E-06 | -0.435 | - | - |
| 17250 | ENSP00000342197 | BTNL8 | 7.892E-06 | -0.435 | - | - |
| 17251 | ENSP00000332771 | CLCNKA | 1.007E-05 | -0.435 | - | - |
| 17252 | ENSP00000450895 | FPGT-TNNI3K | 1.193E-05 | -0.435 | - | - |
| 17253 | ENSP00000369407 | SLC25A35 | 3.090E-06 | -0.435 | - | - |
| 17254 | ENSP00000457544 | RP11-77K12.7 | 1.190E-06 | -0.435 | - | - |
| 17255 | ENSP00000385616 | SUN2 | 6.087E-06 | -0.435 | - | - |
| 17256 | ENSP00000455434 | RD3L | 3.441E-06 | -0.435 | - | - |
| 17257 | ENSP00000359376 | SLC17A9 | 7.664E-06 | -0.435 | - | - |
| 17258 | ENSP00000366006 | UBIAD1 | 5.508E-06 | -0.435 | - | - |
| 17259 | ENSP00000361392 | CCDC24 | 7.886E-06 | -0.435 | - | - |
| 17260 | ENSP00000369689 | CALML5 | 1.827E-05 | -0.435 | - | - |
| 17261 | ENSP00000308312 | DNAI2 | 7.137E-06 | -0.435 | - | - |
| 17262 | ENSP00000329318 | CECR6 | 1.446E-05 | -0.435 | - | - |
| 17263 | ENSP00000354086 | MRPL37 | 3.901E-06 | -0.435 | - | - |
| 17264 | ENSP00000381594 | PCDHGB7 | 4.600E-06 | -0.435 | - | - |
| 17265 | ENSP00000386348 | SAPCD2 | 3.982E-06 | -0.435 | - | - |
| 17266 | ENSP00000347999 | ZC3H7A | 4.468E-06 | -0.436 | - | - |
| 17267 | ENSP00000301272 | TMIGD2 | 1.230E-05 | -0.436 | - | - |
| 17268 | ENSP00000411822 | ISY1-RAB43 | 3.735E-06 | -0.436 | - | - |
| 17269 | ENSP00000407401 | PEX5 | 1.030E-05 | -0.436 | - | - |
| 17270 | ENSP00000370057 | FRMPD4 | 5.241E-06 | -0.436 | - | - |
| 17271 | ENSP00000396622 | MROH7 | 6.868E-06 | -0.436 | - | - |
| 17272 | ENSP00000407761 | ACOXL | 9.562E-06 | -0.436 | - | - |
| 17273 | ENSP00000371036 | SIRPD | 4.659E-06 | -0.436 | - | - |
| 17274 | ENSP00000369784 | TUBAL3 | 1.102E-05 | -0.436 | - | - |
| 17275 | ENSP00000374423 | ARHGAP8 | 8.672E-06 | -0.437 | - | - |
| 17276 | ENSP00000365830 | SOWAHC | 1.279E-05 | -0.437 | - | - |
| 17277 | ENSP00000312457 | BAALC | 1.339E-05 | -0.437 | - | - |
| 17278 | ENSP00000309792 | TMEM234 | 4.507E-06 | -0.437 | - | - |
| 17279 | ENSP00000361005 | ATPAF1 | 6.691E-06 | -0.437 | - | - |
| 17280 | ENSP00000363614 | HMGCL | 9.587E-06 | -0.437 | - | - |
| 17281 | ENSP00000440915 | KIAA0391 | 2.971E-06 | -0.437 | - | - |
| 17282 | ENSP00000352992 | ART5 | 1.107E-05 | -0.437 | - | - |
| 17283 | ENSP00000156084 | OTUD5 | 2.622E-05 | -0.437 | - | - |
| 17284 | ENSP00000352265 | TPGS1 | 4.177E-06 | -0.437 | - | - |
| 17285 | ENSP00000384182 | SCFD2 | 3.961E-06 | -0.437 | - | - |
| 17286 | ENSP00000427900 | KIF13B | 1.351E-05 | -0.437 | - | - |
| 17287 | ENSP00000359396 | DPPA5 | 9.423E-06 | -0.437 | - | - |
| 17288 | ENSP00000363400 | PAFAH2 | 1.309E-05 | -0.437 | - | - |
| 17289 | ENSP00000318846 | ASCL3 | 9.966E-06 | -0.438 | - | - |
| 17290 | ENSP00000372199 | URB1 | 4.088E-06 | -0.438 | - | - |
| 17291 | ENSP00000359521 | SLITRK2 | 1.032E-05 | -0.438 | - | - |
| 17292 | ENSP00000348129 | WDR88 | 8.984E-06 | -0.438 | - | - |
| 17293 | ENSP00000364271 | DFNB59 | 9.453E-06 | -0.438 | - | - |
| 17294 | ENSP00000395249 | FAM134A | 5.678E-06 | -0.438 | - | - |
| 17295 | ENSP00000469517 | CTB-167G5.5 | 4.365E-06 | -0.438 | - | - |
| 17296 | ENSP00000376705 | ANO4 | 6.380E-06 | -0.438 | - | - |
| 17297 | ENSP00000225550 | KRT37 | 8.612E-06 | -0.438 | - | - |
| 17298 | ENSP00000369446 | SMAGP | 5.538E-06 | -0.438 | - | - |
| 17299 | ENSP00000260988 | CRYGB | 1.379E-05 | -0.438 | - | - |
| 17300 | ENSP00000312624 | TCAP | 1.505E-05 | -0.438 | - | - |
| 17301 | ENSP00000322486 | OTOS | 1.222E-05 | -0.438 | - | - |
| 17302 | ENSP00000403852 | POLR2F | 1.442E-05 | -0.438 | - | - |
| 17303 | ENSP00000289359 | MITD1 | 1.054E-05 | -0.438 | - | - |
| 17304 | ENSP00000323663 | DBF4B | 6.844E-06 | -0.438 | - | - |
| 17305 | ENSP00000379008 | SLC5A10 | 4.676E-06 | -0.438 | - | - |
| 17306 | ENSP00000253680 | HSH2D | 1.953E-05 | -0.439 | - | - |
| 17307 | ENSP00000368439 | PCYT1B | 9.177E-06 | -0.439 | - | - |
| 17308 | ENSP00000426359 | MCIDAS | 9.028E-06 | -0.439 | - | - |
| 17309 | ENSP00000342143 | PDZK1 | 1.041E-05 | -0.439 | - | - |
| 17310 | ENSP00000300658 | PGAP3 | 8.456E-06 | -0.439 | - | - |
| 17311 | ENSP00000437464 | FBXL17 | 1.106E-05 | -0.439 | - | - |
| 17312 | ENSP00000368174 | MCM8 | 9.629E-06 | -0.439 | - | - |
| 17313 | ENSP00000371587 | KCNIP4 | 1.504E-05 | -0.439 | - | - |
| 17314 | ENSP00000402825 | FAM197Y1 | 1.521E-05 | -0.439 | - | - |
| 17315 | ENSP00000344866 | FBXL5 | 6.148E-06 | -0.439 | - | - |
| 17316 | ENSP00000258180 | KIAA0513 | 1.639E-05 | -0.439 | - | - |
| 17317 | ENSP00000209929 | FMO2 | 1.768E-05 | -0.439 | - | - |
| 17318 | ENSP00000338260 | APOL4 | 9.819E-06 | -0.439 | - | - |
| 17319 | ENSP00000379496 | PDE1C | 1.175E-05 | -0.439 | - | - |
| 17320 | ENSP00000362507 | CCDC167 | 7.621E-06 | -0.439 | - | - |
| 17321 | ENSP00000414321 | RPS24 | 7.242E-06 | -0.439 | - | - |
| 17322 | ENSP00000381634 | SLC38A1 | 5.326E-06 | -0.439 | - | - |
| 17323 | ENSP00000396704 | TOP2B | 2.759E-05 | -0.439 | - | - |
| 17324 | ENSP00000320649 | CCDC89 | 1.189E-05 | -0.439 | - | - |
| 17325 | ENSP00000412733 | OSBPL9 | 6.901E-06 | -0.439 | - | - |
| 17326 | ENSP00000371272 | PLIN5 | 5.391E-06 | -0.439 | - | - |
| 17327 | ENSP00000354574 | SLC9B2 | 9.900E-06 | -0.439 | - | - |
| 17328 | ENSP00000472847 | SARS2 | 1.624E-05 | -0.439 | - | - |
| 17329 | ENSP00000358921 | ACTR1A | 1.599E-05 | -0.440 | - | - |
| 17330 | ENSP00000381425 | MPP3 | 1.697E-05 | -0.440 | - | - |
| 17331 | ENSP00000373964 | SMTNL2 | 1.508E-05 | -0.440 | - | - |
| 17332 | ENSP00000428288 | PCDHGB4 | 7.584E-06 | -0.440 | - | - |
| 17333 | ENSP00000392617 | SMARCD2 | 8.804E-06 | -0.440 | - | - |
| 17334 | ENSP00000333064 | C22orf39 | 6.344E-06 | -0.440 | - | - |
| 17335 | ENSP00000361982 | TRUB2 | 5.213E-06 | -0.440 | - | - |
| 17336 | ENSP00000418648 | UBN2 | 4.559E-06 | -0.440 | - | - |
| 17337 | ENSP00000367343 | GBA2 | 1.452E-05 | -0.440 | - | - |
| 17338 | ENSP00000307911 | CHST2 | 1.545E-05 | -0.440 | - | - |
| 17339 | ENSP00000445340 | SLC5A8 | 1.699E-05 | -0.440 | - | - |
| 17340 | ENSP00000267383 | CDH24 | 1.468E-05 | -0.440 | - | - |
| 17341 | ENSP00000324403 | PHF21B | 2.421E-06 | -0.440 | - | - |
| 17342 | ENSP00000263174 | PALMD | 1.010E-05 | -0.440 | - | - |
| 17343 | ENSP00000378167 | CCDC66 | 6.826E-06 | -0.441 | - | - |
| 17344 | ENSP00000336524 | CEP63 | 1.096E-05 | -0.441 | - | - |
| 17345 | ENSP00000385695 | SEC14L6 | 3.770E-06 | -0.441 | - | - |
| 17346 | ENSP00000427366 | USP17L26 | 6.338E-06 | -0.441 | - | - |
| 17347 | ENSP00000352282 | KLHDC1 | 4.931E-06 | -0.441 | - | - |
| 17348 | ENSP00000368818 | TOM1L2 | 6.796E-06 | -0.441 | - | - |
| 17349 | ENSP00000322339 | VN1R1 | 9.194E-06 | -0.442 | - | - |
| 17350 | ENSP00000295150 | FAM228A | 1.537E-05 | -0.442 | - | - |
| 17351 | ENSP00000448012 | CEP290 | 8.590E-06 | -0.442 | - | - |
| 17352 | ENSP00000349560 | RAB4B | 1.165E-05 | -0.442 | - | - |
| 17353 | ENSP00000359759 | CT45A5 | 3.487E-06 | -0.442 | - | - |
| 17354 | ENSP00000248306 | METTL25 | 1.077E-05 | -0.442 | - | - |
| 17355 | ENSP00000307264 | C11orf24 | 1.427E-05 | -0.442 | - | - |
| 17356 | ENSP00000355577 | SLC35F3 | 9.227E-06 | -0.442 | - | - |
| 17357 | ENSP00000423777 | USP17L28 | 6.338E-06 | -0.442 | - | - |
| 17358 | ENSP00000310978 | AGXT2L2 | 9.594E-06 | -0.442 | - | - |
| 17359 | ENSP00000403954 | TENM1 | 9.777E-06 | -0.442 | - | - |
| 17360 | ENSP00000369802 | - | 4.844E-06 | -0.442 | - | - |
| 17361 | ENSP00000423211 | USP17L27 | 6.338E-06 | -0.442 | - | - |
| 17362 | ENSP00000228567 | SYT10 | 1.956E-05 | -0.442 | - | - |
| 17363 | ENSP00000319651 | DMRTA1 | 1.108E-05 | -0.442 | - | - |
| 17364 | ENSP00000420040 | KCNIP2 | 1.676E-05 | -0.442 | - | - |
| 17365 | ENSP00000346691 | TBC1D3 | 8.381E-06 | -0.442 | - | - |
| 17366 | ENSP00000263655 | CNRIP1 | 8.253E-06 | -0.442 | - | - |
| 17367 | ENSP00000385545 | KIF26B | 9.095E-06 | -0.442 | - | - |
| 17368 | ENSP00000422097 | USP17L25 | 6.342E-06 | -0.442 | - | - |
| 17369 | ENSP00000348915 | CBWD1 | 5.268E-06 | -0.442 | - | - |
| 17370 | ENSP00000378359 | ADH6 | 1.203E-05 | -0.443 | - | - |
| 17371 | ENSP00000361281 | BEST4 | 1.118E-05 | -0.443 | - | - |
| 17372 | ENSP00000362863 | REEP3 | 4.350E-06 | -0.443 | - | - |
| 17373 | ENSP00000341942 | ATP13A5 | 1.262E-05 | -0.443 | - | - |
| 17374 | ENSP00000356319 | KIF14 | 7.863E-06 | -0.443 | - | - |
| 17375 | ENSP00000355177 | RUSC2 | 7.557E-06 | -0.443 | - | - |
| 17376 | ENSP00000422969 | USP17L29 | 6.342E-06 | -0.443 | - | - |
| 17377 | ENSP00000339182 | ATP13A4 | 1.221E-05 | -0.443 | - | - |
| 17378 | ENSP00000422887 | USP17L30 | 6.342E-06 | -0.443 | - | - |
| 17379 | ENSP00000303042 | AFAP1L2 | 5.824E-06 | -0.443 | - | - |
| 17380 | ENSP00000364737 | CENPP | 5.849E-06 | -0.443 | - | - |
| 17381 | ENSP00000425375 | USP17L24 | 6.330E-06 | -0.443 | - | - |
| 17382 | ENSP00000334134 | RGS9BP | 1.233E-05 | -0.443 | - | - |
| 17383 | ENSP00000354877 | ULK2 | 1.434E-05 | -0.444 | - | - |
| 17384 | ENSP00000272641 | NXPH2 | 1.213E-05 | -0.444 | - | - |
| 17385 | ENSP00000436500 | GRAMD1B | 2.841E-06 | -0.444 | - | - |
| 17386 | ENSP00000291495 | CILP2 | 1.610E-05 | -0.444 | - | - |
| 17387 | ENSP00000365256 | CLCN5 | 8.477E-06 | -0.444 | - | - |
| 17388 | ENSP00000300181 | TSC22D4 | 1.078E-05 | -0.444 | - | - |
| 17389 | ENSP00000349891 | ANKRD20A4 | 9.263E-06 | -0.444 | - | - |
| 17390 | ENSP00000274473 | MEGF10 | 8.950E-06 | -0.444 | - | - |
| 17391 | ENSP00000258930 | CIB2 | 1.333E-05 | -0.444 | - | - |
| 17392 | ENSP00000446654 | RP11-176H8.1 | 2.501E-06 | -0.444 | - | - |
| 17393 | ENSP00000401328 | PRSS48 | 6.028E-06 | -0.444 | - | - |
| 17394 | ENSP00000233545 | MPV17 | 1.409E-05 | -0.444 | - | - |
| 17395 | ENSP00000366121 | ARHGAP27 | 1.170E-05 | -0.444 | - | - |
| 17396 | ENSP00000354721 | SLC26A1 | 6.233E-06 | -0.445 | - | - |
| 17397 | ENSP00000262074 | TTC23 | 1.881E-05 | -0.445 | - | - |
| 17398 | ENSP00000425786 | C4orf22 | 3.879E-06 | -0.445 | - | - |
| 17399 | ENSP00000366390 | SPDYC | 5.770E-06 | -0.445 | - | - |
| 17400 | ENSP00000275988 | SPIN2B | 5.271E-06 | -0.445 | - | - |
| 17401 | ENSP00000361932 | FUT11 | 6.732E-06 | -0.445 | - | - |
| 17402 | ENSP00000355500 | CEP170 | 8.675E-06 | -0.445 | - | - |
| 17403 | ENSP00000321360 | C14orf177 | 1.476E-05 | -0.446 | - | - |
| 17404 | ENSP00000433433 | MAGEL2 | 5.223E-06 | -0.446 | - | - |
| 17405 | ENSP00000381049 | NSG1 | 8.813E-06 | -0.446 | - | - |
| 17406 | ENSP00000331849 | MRPL54 | 8.195E-06 | -0.446 | - | - |
| 17407 | ENSP00000260453 | MNS1 | 1.943E-05 | -0.446 | - | - |
| 17408 | ENSP00000323435 | SNX22 | 7.231E-06 | -0.446 | - | - |
| 17409 | ENSP00000297581 | DCSTAMP | 1.629E-05 | -0.446 | - | - |
| 17410 | ENSP00000345216 | USP54 | 6.336E-06 | -0.447 | - | - |
| 17411 | ENSP00000368931 | CCDC112 | 3.514E-06 | -0.447 | - | - |
| 17412 | ENSP00000462701 | CDH8 | 7.399E-06 | -0.447 | - | - |
| 17413 | ENSP00000466399 | RAD51D | 2.820E-05 | -0.447 | - | - |
| 17414 | ENSP00000291823 | HIPK4 | 9.360E-06 | -0.447 | - | - |
| 17415 | ENSP00000305334 | FSTL5 | 1.005E-05 | -0.447 | - | - |
| 17416 | ENSP00000348635 | AMICA1 | 1.154E-05 | -0.447 | - | - |
| 17417 | ENSP00000339802 | ANKRD34B | 7.161E-06 | -0.447 | - | - |
| 17418 | ENSP00000364412 | HSD17B3 | 5.773E-06 | -0.447 | - | - |
| 17419 | ENSP00000360828 | DDX27 | 7.353E-06 | -0.447 | - | - |
| 17420 | ENSP00000363345 | SLC46A2 | 2.385E-06 | -0.448 | - | - |
| 17421 | ENSP00000370223 | IDH3B | 6.856E-06 | -0.448 | - | - |
| 17422 | ENSP00000465075 | SPC24 | 5.859E-06 | -0.448 | - | - |
| 17423 | ENSP00000448035 | NACA | 6.655E-06 | -0.448 | - | - |
| 17424 | ENSP00000351446 | WDR5 | 1.210E-05 | -0.448 | - | - |
| 17425 | ENSP00000320416 | VPS37D | 3.806E-06 | -0.448 | - | - |
| 17426 | ENSP00000377523 | DQX1 | 8.402E-06 | -0.448 | - | - |
| 17427 | ENSP00000309595 | C10orf2 | 1.317E-05 | -0.448 | - | - |
| 17428 | ENSP00000357943 | ANXA9 | 8.932E-06 | -0.448 | - | - |
| 17429 | ENSP00000413937 | INPP5K | 8.539E-06 | -0.448 | - | - |
| 17430 | ENSP00000370964 | SLCO1C1 | 8.951E-06 | -0.448 | - | - |
| 17431 | ENSP00000361206 | MRPS18A | 4.798E-06 | -0.448 | - | - |
| 17432 | ENSP00000361057 | SURF4 | 8.269E-06 | -0.448 | - | - |
| 17433 | ENSP00000386227 | MCC | 6.676E-06 | -0.448 | - | - |
| 17434 | ENSP00000358016 | SCML4 | 1.310E-05 | -0.448 | - | - |
| 17435 | ENSP00000435061 | FNTA | 3.657E-06 | -0.449 | - | - |
| 17436 | ENSP00000233331 | INO80B | 9.292E-06 | -0.449 | - | - |
| 17437 | ENSP00000358414 | HMGCS2 | 1.299E-05 | -0.449 | - | - |
| 17438 | ENSP00000380354 | SPATA22 | 2.796E-06 | -0.449 | - | - |
| 17439 | ENSP00000363482 | FAM21C | 3.765E-06 | -0.449 | - | - |
| 17440 | ENSP00000364591 | TSPYL2 | 1.870E-05 | -0.449 | - | - |
| 17441 | ENSP00000296484 | POC1A | 1.455E-05 | -0.449 | - | - |
| 17442 | ENSP00000347834 | FBXL3 | 7.491E-06 | -0.449 | - | - |
| 17443 | ENSP00000218056 | WDR13 | 1.779E-05 | -0.449 | - | - |
| 17444 | ENSP00000367137 | SLC17A4 | 6.715E-06 | -0.449 | - | - |
| 17445 | ENSP00000361083 | ADIRF | 1.018E-05 | -0.449 | - | - |
| 17446 | ENSP00000216268 | ZBED4 | 9.181E-06 | -0.450 | - | - |
| 17447 | ENSP00000385916 | RP11-145E5.5 | 4.398E-06 | -0.450 | - | - |
| 17448 | ENSP00000368516 | LGR4 | 1.358E-05 | -0.450 | - | - |
| 17449 | ENSP00000331625 | C3orf35 | 6.571E-06 | -0.450 | - | - |
| 17450 | ENSP00000315465 | DSCAML1 | 1.487E-05 | -0.450 | - | - |
| 17451 | ENSP00000462023 | RPL17 | 6.900E-06 | -0.450 | - | - |
| 17452 | ENSP00000387841 | - | 4.239E-06 | -0.450 | - | - |
| 17453 | ENSP00000430706 | EIF5AL1 | 1.274E-05 | -0.450 | - | - |
| 17454 | ENSP00000409256 | EFCAB4A | 3.716E-06 | -0.450 | - | - |
| 17455 | ENSP00000404011 | RP11-13B9.3 | 1.616E-06 | -0.450 | - | - |
| 17456 | ENSP00000258436 | MFSD9 | 1.345E-05 | -0.450 | - | - |
| 17457 | ENSP00000358451 | GDAP2 | 5.669E-06 | -0.450 | - | - |
| 17458 | ENSP00000310998 | NAGPA | 1.426E-05 | -0.450 | - | - |
| 17459 | ENSP00000246891 | CSN1S1 | 1.480E-05 | -0.450 | - | - |
| 17460 | ENSP00000392262 | PPP5D1 | 1.288E-05 | -0.450 | - | - |
| 17461 | ENSP00000358563 | DKC1 | 8.814E-06 | -0.451 | - | - |
| 17462 | ENSP00000399013 | CNTNAP5 | 1.436E-05 | -0.451 | - | - |
| 17463 | ENSP00000428864 | CES5A | 7.178E-06 | -0.451 | - | - |
| 17464 | ENSP00000323075 | GAPT | 1.315E-05 | -0.451 | - | - |
| 17465 | ENSP00000417583 | ST6GALNAC5 | 1.541E-05 | -0.451 | - | - |
| 17466 | ENSP00000361475 | NCS1 | 1.401E-05 | -0.451 | - | - |
| 17467 | ENSP00000336729 | GALNT16 | 4.248E-06 | -0.451 | - | - |
| 17468 | ENSP00000355471 | TFB2M | 7.100E-06 | -0.451 | - | - |
| 17469 | ENSP00000348778 | RAB19 | 6.667E-06 | -0.451 | - | - |
| 17470 | ENSP00000296978 | TMEM200A | 1.423E-05 | -0.452 | - | - |
| 17471 | ENSP00000350475 | SLC4A5 | 1.169E-05 | -0.452 | - | - |
| 17472 | ENSP00000338164 | TMEM217 | 1.050E-05 | -0.452 | - | - |
| 17473 | ENSP00000420659 | KLHL29 | 1.934E-06 | -0.452 | - | - |
| 17474 | ENSP00000354950 | PCDH15 | 1.444E-05 | -0.452 | - | - |
| 17475 | ENSP00000378145 | CCDC109B | 4.915E-06 | -0.452 | - | - |
| 17476 | ENSP00000378588 | TIMM9 | 5.751E-06 | -0.452 | - | - |
| 17477 | ENSP00000408132 | GOLGA6B | 6.967E-06 | -0.452 | - | - |
| 17478 | ENSP00000347548 | RNF220 | 6.100E-06 | -0.452 | - | - |
| 17479 | ENSP00000359019 | PITX3 | 1.469E-05 | -0.452 | - | - |
| 17480 | ENSP00000436585 | ECHDC1 | 6.474E-06 | -0.452 | - | - |
| 17481 | ENSP00000363452 | MAN1C1 | 1.089E-05 | -0.452 | - | - |
| 17482 | ENSP00000264228 | SRD5A3 | 1.198E-05 | -0.452 | - | - |
| 17483 | ENSP00000417116 | LBX2 | 6.400E-06 | -0.452 | - | - |
| 17484 | ENSP00000334998 | MT1B | 6.851E-06 | -0.452 | - | - |
| 17485 | ENSP00000361557 | RBM41 | 6.249E-06 | -0.452 | - | - |
| 17486 | ENSP00000371236 | GART | 3.092E-05 | -0.452 | - | - |
| 17487 | ENSP00000241312 | CSMD2 | 1.571E-05 | -0.453 | - | - |
| 17488 | ENSP00000242315 | KIAA1045 | 9.468E-06 | -0.453 | - | - |
| 17489 | ENSP00000253110 | C19orf66 | 4.800E-06 | -0.453 | - | - |
| 17490 | ENSP00000466420 | UQCR11 | 5.793E-06 | -0.453 | - | - |
| 17491 | ENSP00000465204 | CNTD1 | 2.466E-06 | -0.453 | - | - |
| 17492 | ENSP00000222212 | CACNG7 | 1.444E-05 | -0.453 | - | - |
| 17493 | ENSP00000369465 | TXLNG | 6.097E-06 | -0.453 | - | - |
| 17494 | ENSP00000360054 | PHACTR3 | 1.135E-05 | -0.453 | - | - |
| 17495 | ENSP00000405424 | OXR1 | 5.283E-06 | -0.453 | - | - |
| 17496 | ENSP00000399547 | ANKRD63 | 6.474E-06 | -0.453 | - | - |
| 17497 | ENSP00000303486 | BAHCC1 | 1.719E-05 | -0.453 | - | - |
| 17498 | ENSP00000363384 | ZNF593 | 4.063E-06 | -0.453 | - | - |
| 17499 | ENSP00000312439 | RNF26 | 8.484E-06 | -0.453 | - | - |
| 17500 | ENSP00000355512 | OPN3 | 1.471E-05 | -0.453 | - | - |
| 17501 | ENSP00000227618 | ANAPC15 | 8.206E-06 | -0.454 | - | - |
| 17502 | ENSP00000341914 | PTCHD4 | 1.453E-05 | -0.454 | - | - |
| 17503 | ENSP00000239444 | PCDHB8 | 1.995E-05 | -0.454 | - | - |
| 17504 | ENSP00000385028 | SLC22A23 | 6.671E-06 | -0.454 | - | - |
| 17505 | ENSP00000401191 | WBSCR22 | 9.832E-06 | -0.454 | - | - |
| 17506 | ENSP00000358307 | NHLRC2 | 7.077E-06 | -0.454 | - | - |
| 17507 | ENSP00000403946 | MBTD1 | 9.669E-06 | -0.454 | - | - |
| 17508 | ENSP00000348496 | SUN5 | 6.218E-06 | -0.454 | - | - |
| 17509 | ENSP00000355893 | SLC30A10 | 1.494E-05 | -0.455 | - | - |
| 17510 | ENSP00000357823 | MRPL9 | 3.964E-06 | -0.455 | - | - |
| 17511 | ENSP00000363036 | FBXW2 | 1.164E-05 | -0.455 | - | - |
| 17512 | ENSP00000380646 | TMEM80 | 7.365E-06 | -0.455 | - | - |
| 17513 | ENSP00000272438 | TEX261 | 7.987E-06 | -0.455 | - | - |
| 17514 | ENSP00000370077 | CCDC171 | 3.397E-06 | -0.455 | - | - |
| 17515 | ENSP00000365938 | UGGT2 | 9.026E-06 | -0.455 | - | - |
| 17516 | ENSP00000434724 | CCDC90B | 4.859E-06 | -0.455 | - | - |
| 17517 | ENSP00000363799 | ACTL7B | 1.248E-05 | -0.455 | - | - |
| 17518 | ENSP00000354966 | SLC9A8 | 1.425E-05 | -0.455 | - | - |
| 17519 | ENSP00000440053 | - | 6.068E-06 | -0.455 | - | - |
| 17520 | ENSP00000331466 | TMEM95 | 9.001E-06 | -0.455 | - | - |
| 17521 | ENSP00000323246 | E2F7 | 9.792E-06 | -0.455 | - | - |
| 17522 | ENSP00000468690 | RP11-618P17.4 | 1.205E-05 | -0.455 | - | - |
| 17523 | ENSP00000344855 | FBXL14 | 8.182E-06 | -0.455 | - | - |
| 17524 | ENSP00000373261 | MOBP | 3.806E-06 | -0.455 | - | - |
| 17525 | ENSP00000337572 | DGKH | 1.013E-05 | -0.455 | - | - |
| 17526 | ENSP00000331474 | SYNDIG1L | 8.297E-06 | -0.455 | - | - |
| 17527 | ENSP00000399356 | CT62 | 3.507E-06 | -0.456 | - | - |
| 17528 | ENSP00000296785 | ANKRA2 | 1.119E-05 | -0.456 | - | - |
| 17529 | ENSP00000330808 | OTOL1 | 1.017E-05 | -0.456 | - | - |
| 17530 | ENSP00000435300 | PCDHA4 | 6.203E-06 | -0.456 | - | - |
| 17531 | ENSP00000353791 | SLC6A9 | 6.145E-06 | -0.456 | - | - |
| 17532 | ENSP00000357739 | SPRR2E | 6.740E-06 | -0.456 | - | - |
| 17533 | ENSP00000252729 | CACNG6 | 9.600E-06 | -0.456 | - | - |
| 17534 | ENSP00000356785 | NME7 | 7.953E-06 | -0.456 | - | - |
| 17535 | ENSP00000424707 | TMEM167A | 3.493E-06 | -0.456 | - | - |
| 17536 | ENSP00000417161 | GPR143 | 1.334E-05 | -0.456 | - | - |
| 17537 | ENSP00000362704 | CCDC28B | 5.287E-06 | -0.456 | - | - |
| 17538 | ENSP00000419786 | RGAG1 | 5.707E-06 | -0.456 | - | - |
| 17539 | ENSP00000298892 | CAPRIN2 | 9.085E-06 | -0.456 | - | - |
| 17540 | ENSP00000406577 | CKMT1A | 6.772E-06 | -0.456 | - | - |
| 17541 | ENSP00000438970 | FLJ22184 | 3.244E-06 | -0.457 | - | - |
| 17542 | ENSP00000280020 | KIAA1328 | 1.776E-05 | -0.457 | - | - |
| 17543 | ENSP00000384264 | CNGA1 | 1.211E-05 | -0.457 | - | - |
| 17544 | ENSP00000354722 | EIF1AY | 9.932E-06 | -0.457 | - | - |
| 17545 | ENSP00000363162 | ATP6V1G1 | 5.030E-06 | -0.457 | - | - |
| 17546 | ENSP00000412060 | GREB1L | 2.917E-06 | -0.457 | - | - |
| 17547 | ENSP00000335094 | TMEM17 | 2.692E-06 | -0.457 | - | - |
| 17548 | ENSP00000320813 | DEFB104A | 1.248E-05 | -0.457 | - | - |
| 17549 | ENSP00000356579 | CEP350 | 7.723E-06 | -0.458 | - | - |
| 17550 | ENSP00000364035 | FAAH2 | 6.856E-06 | -0.458 | - | - |
| 17551 | ENSP00000301204 | TMEM145 | 1.068E-05 | -0.458 | - | - |
| 17552 | ENSP00000346130 | SVIP | 6.475E-06 | -0.458 | - | - |
| 17553 | ENSP00000348897 | GABRA2 | 8.938E-06 | -0.458 | - | - |
| 17554 | ENSP00000329199 | MAGEA6 | 6.496E-06 | -0.459 | - | - |
| 17555 | ENSP00000359607 | PYROXD2 | 1.353E-05 | -0.459 | - | - |
| 17556 | ENSP00000388137 | MPPED1 | 1.591E-06 | -0.459 | - | - |
| 17557 | ENSP00000300182 | MS4A6E | 1.070E-05 | -0.459 | - | - |
| 17558 | ENSP00000424243 | EYS | 2.385E-05 | -0.459 | - | - |
| 17559 | ENSP00000419199 | ASB14 | 7.648E-06 | -0.459 | - | - |
| 17560 | ENSP00000443130 | RP11-286N22.8 | 3.064E-06 | -0.459 | - | - |
| 17561 | ENSP00000351147 | DCAF4 | 5.554E-06 | -0.459 | - | - |
| 17562 | ENSP00000382237 | TMEM26 | 1.049E-05 | -0.459 | - | - |
| 17563 | ENSP00000440374 | MCF2L | 8.183E-06 | -0.459 | - | - |
| 17564 | ENSP00000357564 | RAB13 | 9.990E-06 | -0.459 | - | - |
| 17565 | ENSP00000261226 | TMCC3 | 1.270E-05 | -0.459 | - | - |
| 17566 | ENSP00000422533 | LPHN3 | 1.767E-05 | -0.459 | - | - |
| 17567 | ENSP00000220676 | RP1 | 1.683E-05 | -0.459 | - | - |
| 17568 | ENSP00000360473 | PFDN4 | 6.157E-06 | -0.459 | - | - |
| 17569 | ENSP00000371517 | AMACR | 9.437E-06 | -0.459 | - | - |
| 17570 | ENSP00000265191 | NME5 | 7.254E-06 | -0.459 | - | - |
| 17571 | ENSP00000329880 | PHACTR1 | 1.260E-05 | -0.459 | - | - |
| 17572 | ENSP00000271064 | TINAGL1 | 1.349E-05 | -0.460 | - | - |
| 17573 | ENSP00000253401 | ARHGEF9 | 1.225E-05 | -0.460 | - | - |
| 17574 | ENSP00000347839 | RAB11FIP2 | 6.404E-06 | -0.460 | - | - |
| 17575 | ENSP00000363582 | GGNBP1 | 4.405E-06 | -0.460 | - | - |
| 17576 | ENSP00000244527 | SLC17A1 | 1.072E-05 | -0.460 | - | - |
| 17577 | ENSP00000377430 | DPEP2 | 3.537E-06 | -0.460 | - | - |
| 17578 | ENSP00000308770 | SULT1B1 | 1.706E-05 | -0.460 | - | - |
| 17579 | ENSP00000349320 | CACNA2D1 | 1.348E-05 | -0.460 | - | - |
| 17580 | ENSP00000393832 | PPP1R3G | 6.591E-06 | -0.460 | - | - |
| 17581 | ENSP00000390621 | TDRD12 | 5.482E-06 | -0.460 | - | - |
| 17582 | ENSP00000317385 | GLIPR1L2 | 1.243E-05 | -0.460 | - | - |
| 17583 | ENSP00000457733 | SLC25A24 | 5.972E-06 | -0.460 | - | - |
| 17584 | ENSP00000365694 | PPP1R10 | 5.999E-06 | -0.460 | - | - |
| 17585 | ENSP00000371929 | HTR3D | 8.127E-06 | -0.461 | - | - |
| 17586 | ENSP00000444052 | CES4A | 4.965E-06 | -0.461 | - | - |
| 17587 | ENSP00000381970 | GIN1 | 5.170E-06 | -0.461 | - | - |
| 17588 | ENSP00000354536 | ASTN1 | 8.859E-06 | -0.461 | - | - |
| 17589 | ENSP00000372193 | CLCN7 | 1.190E-05 | -0.461 | - | - |
| 17590 | ENSP00000395983 | SLC12A4 | 1.050E-05 | -0.461 | - | - |
| 17591 | ENSP00000364620 | PADI1 | 1.019E-05 | -0.461 | - | - |
| 17592 | ENSP00000365601 | IDNK | 2.902E-06 | -0.461 | - | - |
| 17593 | ENSP00000218008 | ATP1B4 | 1.352E-05 | -0.461 | - | - |
| 17594 | ENSP00000294309 | TPCN2 | 1.249E-05 | -0.461 | - | - |
| 17595 | ENSP00000355086 | DARS2 | 3.966E-06 | -0.461 | - | - |
| 17596 | ENSP00000403117 | MFSD1 | 6.274E-06 | -0.461 | - | - |
| 17597 | ENSP00000341987 | OR51I2 | 1.636E-05 | -0.461 | - | - |
| 17598 | ENSP00000366800 | DNAJC11 | 4.638E-06 | -0.462 | - | - |
| 17599 | ENSP00000416095 | INE1 | 3.332E-06 | -0.462 | - | - |
| 17600 | ENSP00000365251 | AGTPBP1 | 6.182E-06 | -0.462 | - | - |
| 17601 | ENSP00000347206 | GPAA1 | 5.756E-06 | -0.462 | - | - |
| 17602 | ENSP00000416673 | PATL2 | 4.654E-06 | -0.462 | - | - |
| 17603 | ENSP00000306888 | FAM151A | 1.260E-05 | -0.462 | - | - |
| 17604 | ENSP00000360530 | NSMF | 9.329E-06 | -0.462 | - | - |
| 17605 | ENSP00000376570 | GALNT13 | 1.153E-05 | -0.462 | - | - |
| 17606 | ENSP00000353695 | GSTCD | 9.041E-06 | -0.462 | - | - |
| 17607 | ENSP00000297468 | GPR146 | 1.351E-05 | -0.462 | - | - |
| 17608 | ENSP00000387233 | ANKRD44 | 8.517E-06 | -0.462 | - | - |
| 17609 | ENSP00000413575 | CROT | 9.288E-06 | -0.462 | - | - |
| 17610 | ENSP00000366645 | SCP2D1 | 3.768E-06 | -0.462 | - | - |
| 17611 | ENSP00000357591 | BET3L | 2.392E-06 | -0.462 | - | - |
| 17612 | ENSP00000309681 | CCDC106 | 9.372E-06 | -0.462 | - | - |
| 17613 | ENSP00000387176 | POTEJ | 2.361E-05 | -0.462 | - | - |
| 17614 | ENSP00000334540 | GPR141 | 1.330E-05 | -0.462 | - | - |
| 17615 | ENSP00000221347 | FCGBP | 1.248E-05 | -0.462 | - | - |
| 17616 | ENSP00000366886 | KLHL21 | 4.936E-06 | -0.463 | - | - |
| 17617 | ENSP00000342518 | SLC35F4 | 9.045E-06 | -0.463 | - | - |
| 17618 | ENSP00000379090 | PPP6R2 | 6.901E-06 | -0.463 | - | - |
| 17619 | ENSP00000264833 | OLFM2 | 1.524E-05 | -0.463 | - | - |
| 17620 | ENSP00000329553 | IMMP2L | 9.527E-06 | -0.463 | - | - |
| 17621 | ENSP00000264387 | C2orf83 | 1.594E-05 | -0.463 | - | - |
| 17622 | ENSP00000364555 | ACTL8 | 1.201E-05 | -0.463 | - | - |
| 17623 | ENSP00000330031 | PIWIL3 | 8.966E-06 | -0.463 | - | - |
| 17624 | ENSP00000337475 | ZDHHC4 | 1.112E-05 | -0.463 | - | - |
| 17625 | ENSP00000276646 | SYBU | 1.221E-05 | -0.463 | - | - |
| 17626 | ENSP00000286544 | FAM161B | 9.804E-06 | -0.463 | - | - |
| 17627 | ENSP00000322649 | SLC25A41 | 7.777E-06 | -0.463 | - | - |
| 17628 | ENSP00000354665 | MT-ND6 | 7.279E-06 | -0.463 | - | - |
| 17629 | ENSP00000338532 | FAM53B | 6.122E-06 | -0.463 | - | - |
| 17630 | ENSP00000359848 | PIGK | 6.491E-06 | -0.463 | - | - |
| 17631 | ENSP00000429301 | PGRMC2 | 4.420E-06 | -0.464 | - | - |
| 17632 | ENSP00000339260 | SLC6A13 | 1.375E-05 | -0.464 | - | - |
| 17633 | ENSP00000362639 | RABEPK | 5.014E-06 | -0.464 | - | - |
| 17634 | ENSP00000242249 | RAMP3 | 2.232E-05 | -0.464 | - | - |
| 17635 | ENSP00000463847 | RPL26 | 3.960E-06 | -0.464 | - | - |
| 17636 | ENSP00000354107 | SLC6A6 | 7.813E-06 | -0.464 | - | - |
| 17637 | ENSP00000355735 | C6orf70 | 6.853E-06 | -0.464 | - | - |
| 17638 | ENSP00000386181 | TTC30B | 4.368E-06 | -0.464 | - | - |
| 17639 | ENSP00000335247 | STARD10 | 1.382E-05 | -0.464 | - | - |
| 17640 | ENSP00000357882 | PSTK | 5.985E-06 | -0.464 | - | - |
| 17641 | ENSP00000345580 | SLC25A10 | 5.453E-06 | -0.464 | - | - |
| 17642 | ENSP00000358888 | SYPL2 | 8.076E-06 | -0.465 | - | - |
| 17643 | ENSP00000335261 | TMLHE | 1.019E-05 | -0.465 | - | - |
| 17644 | ENSP00000237353 | PMFBP1 | 8.596E-06 | -0.465 | - | - |
| 17645 | ENSP00000310891 | CSGALNACT1 | 7.107E-06 | -0.465 | - | - |
| 17646 | ENSP00000328940 | SLX1B | 1.280E-05 | -0.465 | - | - |
| 17647 | ENSP00000359665 | PI4K2A | 9.592E-06 | -0.465 | - | - |
| 17648 | ENSP00000410862 | RUNDC3A | 8.098E-06 | -0.465 | - | - |
| 17649 | ENSP00000417401 | HELZ2 | 1.501E-05 | -0.465 | - | - |
| 17650 | ENSP00000355499 | SDCCAG8 | 6.333E-06 | -0.465 | - | - |
| 17651 | ENSP00000382777 | KRTAP13-2 | 3.257E-06 | -0.465 | - | - |
| 17652 | ENSP00000365492 | DEFB119 | 4.011E-06 | -0.465 | - | - |
| 17653 | ENSP00000343657 | MCCC2 | 4.939E-06 | -0.465 | - | - |
| 17654 | ENSP00000352167 | SLC25A29 | 6.099E-06 | -0.465 | - | - |
| 17655 | ENSP00000462747 | RP11-343C2.7 | 2.348E-06 | -0.465 | - | - |
| 17656 | ENSP00000442046 | TMTC1 | 1.141E-05 | -0.465 | - | - |
| 17657 | ENSP00000338783 | CHST5 | 8.359E-06 | -0.465 | - | - |
| 17658 | ENSP00000351875 | ANKRD30B | 8.570E-06 | -0.465 | - | - |
| 17659 | ENSP00000443900 | LYRM4 | 6.418E-06 | -0.465 | - | - |
| 17660 | ENSP00000310593 | MAP9 | 1.372E-05 | -0.466 | - | - |
| 17661 | ENSP00000321962 | FIBIN | 1.528E-05 | -0.466 | - | - |
| 17662 | ENSP00000318716 | FAM9B | 7.762E-06 | -0.466 | - | - |
| 17663 | ENSP00000255087 | MTL5 | 1.067E-05 | -0.466 | - | - |
| 17664 | ENSP00000369618 | FASTKD5 | 3.371E-06 | -0.466 | - | - |
| 17665 | ENSP00000356470 | SWT1 | 6.719E-06 | -0.466 | - | - |
| 17666 | ENSP00000360398 | SLC25A27 | 8.027E-06 | -0.466 | - | - |
| 17667 | ENSP00000366249 | UBD | 2.506E-05 | -0.466 | - | - |
| 17668 | ENSP00000468267 | RP11-146D12.2 | 5.784E-06 | -0.466 | - | - |
| 17669 | ENSP00000358515 | SLC22A15 | 9.927E-06 | -0.466 | - | - |
| 17670 | ENSP00000321242 | PRNT | 1.050E-05 | -0.467 | - | - |
| 17671 | ENSP00000354518 | ZNF830 | 4.536E-06 | -0.467 | - | - |
| 17672 | ENSP00000400471 | TMEM106C | 3.938E-06 | -0.467 | - | - |
| 17673 | ENSP00000354293 | PCDHB16 | 5.927E-06 | -0.467 | - | - |
| 17674 | ENSP00000355109 | SFMBT2 | 8.452E-06 | -0.467 | - | - |
| 17675 | ENSP00000389009 | CARNS1 | 1.059E-05 | -0.467 | - | - |
| 17676 | ENSP00000252804 | PXDN | 1.542E-05 | -0.467 | - | - |
| 17677 | ENSP00000361667 | DOLK | 5.191E-06 | -0.467 | - | - |
| 17678 | ENSP00000346827 | ALDH9A1 | 1.198E-05 | -0.467 | - | - |
| 17679 | ENSP00000312834 | MLXIP | 1.085E-05 | -0.468 | - | - |
| 17680 | ENSP00000355384 | SLC26A11 | 6.645E-06 | -0.468 | - | - |
| 17681 | ENSP00000265529 | KIF9 | 1.146E-05 | -0.468 | - | - |
| 17682 | ENSP00000270642 | IGLON5 | 9.119E-06 | -0.468 | - | - |
| 17683 | ENSP00000387252 | CCDC103 | 2.669E-06 | -0.468 | - | - |
| 17684 | ENSP00000374566 | KIF19 | 1.044E-05 | -0.468 | - | - |
| 17685 | ENSP00000416658 | NME6 | 6.010E-06 | -0.469 | - | - |
| 17686 | ENSP00000335292 | SLC51B | 8.336E-06 | -0.469 | - | - |
| 17687 | ENSP00000332879 | FLRT2 | 1.726E-05 | -0.469 | - | - |
| 17688 | ENSP00000362171 | NAP1L3 | 1.103E-05 | -0.469 | - | - |
| 17689 | ENSP00000464146 | OAZ1 | 6.515E-06 | -0.469 | - | - |
| 17690 | ENSP00000419194 | MSMP | 2.551E-06 | -0.469 | - | - |
| 17691 | ENSP00000322273 | OLFML3 | 1.066E-05 | -0.469 | - | - |
| 17692 | ENSP00000365025 | CSNK2B | 1.022E-05 | -0.469 | - | - |
| 17693 | ENSP00000417061 | C1orf222 | 4.053E-06 | -0.469 | - | - |
| 17694 | ENSP00000358532 | RBM20 | 5.893E-06 | -0.470 | - | - |
| 17695 | ENSP00000324901 | IQCK | 1.123E-05 | -0.470 | - | - |
| 17696 | ENSP00000311122 | TMEM52 | 1.011E-05 | -0.470 | - | - |
| 17697 | ENSP00000344125 | GTDC2 | 8.555E-06 | -0.470 | - | - |
| 17698 | ENSP00000276077 | GPR174 | 8.865E-06 | -0.470 | - | - |
| 17699 | ENSP00000311687 | NIPAL4 | 6.385E-06 | -0.470 | - | - |
| 17700 | ENSP00000289957 | CHRNB3 | 1.115E-05 | -0.470 | - | - |
| 17701 | ENSP00000327889 | INTS5 | 6.256E-06 | -0.470 | - | - |
| 17702 | ENSP00000349124 | PPP4R2 | 5.553E-06 | -0.470 | - | - |
| 17703 | ENSP00000296059 | TM4SF18 | 5.177E-06 | -0.470 | - | - |
| 17704 | ENSP00000458307 | GGT6 | 1.480E-05 | -0.470 | - | - |
| 17705 | ENSP00000359127 | DPH5 | 4.223E-06 | -0.470 | - | - |
| 17706 | ENSP00000384193 | CADM2 | 5.238E-06 | -0.470 | - | - |
| 17707 | ENSP00000305964 | COX7B2 | 1.042E-05 | -0.470 | - | - |
| 17708 | ENSP00000303584 | N6AMT1 | 1.284E-05 | -0.470 | - | - |
| 17709 | ENSP00000353650 | FABP12 | 3.657E-06 | -0.471 | - | - |
| 17710 | ENSP00000314129 | TFE3 | 1.485E-05 | -0.471 | - | - |
| 17711 | ENSP00000417658 | PTCHD3 | 9.200E-06 | -0.471 | - | - |
| 17712 | ENSP00000362208 | MRPS15 | 5.957E-06 | -0.471 | - | - |
| 17713 | ENSP00000389175 | GLYCTK | 7.044E-06 | -0.471 | - | - |
| 17714 | ENSP00000278550 | TENM4 | 1.312E-05 | -0.471 | - | - |
| 17715 | ENSP00000346627 | TYW5 | 1.050E-05 | -0.471 | - | - |
| 17716 | ENSP00000377086 | LONRF2 | 1.125E-05 | -0.471 | - | - |
| 17717 | ENSP00000262113 | MYOM2 | 1.648E-05 | -0.471 | - | - |
| 17718 | ENSP00000274721 | GFRA3 | 1.324E-05 | -0.471 | - | - |
| 17719 | ENSP00000367564 | C19orf54 | 3.947E-06 | -0.471 | - | - |
| 17720 | ENSP00000331691 | C12orf60 | 1.085E-05 | -0.471 | - | - |
| 17721 | ENSP00000335196 | GLDN | 1.247E-05 | -0.471 | - | - |
| 17722 | ENSP00000348982 | MEGF6 | 1.168E-05 | -0.472 | - | - |
| 17723 | ENSP00000406674 | MOGAT1 | 9.020E-06 | -0.472 | - | - |
| 17724 | ENSP00000257312 | DZIP1 | 1.387E-05 | -0.472 | - | - |
| 17725 | ENSP00000275884 | DENND2A | 1.072E-05 | -0.472 | - | - |
| 17726 | ENSP00000449629 | ANKS1B | 9.389E-06 | -0.472 | - | - |
| 17727 | ENSP00000410943 | TSPYL4 | 1.567E-05 | -0.472 | - | - |
| 17728 | ENSP00000281142 | SCLT1 | 7.954E-06 | -0.472 | - | - |
| 17729 | ENSP00000436767 | MTG1 | 5.068E-06 | -0.472 | - | - |
| 17730 | ENSP00000274368 | CRHBP | 1.521E-05 | -0.472 | - | - |
| 17731 | ENSP00000322617 | HTR3C | 9.848E-06 | -0.472 | - | - |
| 17732 | ENSP00000344996 | PALM3 | 1.169E-05 | -0.473 | - | - |
| 17733 | ENSP00000324419 | SYT9 | 1.452E-05 | -0.473 | - | - |
| 17734 | ENSP00000403636 | SYNGAP1 | 1.077E-05 | -0.473 | - | - |
| 17735 | ENSP00000311364 | MUCL1 | 6.944E-06 | -0.473 | - | - |
| 17736 | ENSP00000434614 | CDC27 | 1.582E-05 | -0.473 | - | - |
| 17737 | ENSP00000254901 | REEP2 | 1.134E-05 | -0.473 | - | - |
| 17738 | ENSP00000282020 | GRID2 | 1.166E-05 | -0.473 | - | - |
| 17739 | ENSP00000369461 | ECI2 | 7.821E-06 | -0.473 | - | - |
| 17740 | ENSP00000368876 | FAM179A | 5.138E-06 | -0.474 | - | - |
| 17741 | ENSP00000369176 | NDUFB6 | 5.845E-06 | -0.474 | - | - |
| 17742 | ENSP00000307004 | APLF | 1.175E-05 | -0.474 | - | - |
| 17743 | ENSP00000252597 | USHBP1 | 8.051E-06 | -0.474 | - | - |
| 17744 | ENSP00000419503 | PTPLAD2 | 7.787E-06 | -0.474 | - | - |
| 17745 | ENSP00000307369 | SHE | 1.442E-05 | -0.474 | - | - |
| 17746 | ENSP00000296046 | CPA3 | 1.505E-05 | -0.474 | - | - |
| 17747 | ENSP00000245812 | ALKBH7 | 1.261E-05 | -0.474 | - | - |
| 17748 | ENSP00000433427 | ARL15 | 4.978E-06 | -0.474 | - | - |
| 17749 | ENSP00000376465 | GALNT3 | 1.081E-05 | -0.474 | - | - |
| 17750 | ENSP00000306324 | PWWP2B | 7.148E-06 | -0.474 | - | - |
| 17751 | ENSP00000339155 | CRISP2 | 1.504E-05 | -0.474 | - | - |
| 17752 | ENSP00000461785 | RAB40B | 7.673E-06 | -0.475 | - | - |
| 17753 | ENSP00000339692 | RASSF4 | 1.271E-05 | -0.475 | - | - |
| 17754 | ENSP00000399982 | TAF6 | 7.275E-06 | -0.475 | - | - |
| 17755 | ENSP00000365167 | FHAD1 | 3.705E-06 | -0.475 | - | - |
| 17756 | ENSP00000421380 | SNF8 | 7.835E-06 | -0.475 | - | - |
| 17757 | ENSP00000325674 | EXOC3L1 | 1.607E-05 | -0.475 | - | - |
| 17758 | ENSP00000310657 | NDST2 | 1.277E-05 | -0.475 | - | - |
| 17759 | ENSP00000320234 | MFSD7 | 1.401E-05 | -0.475 | - | - |
| 17760 | ENSP00000355167 | HORMAD1 | 1.188E-05 | -0.476 | - | - |
| 17761 | ENSP00000442050 | PCYT2 | 5.412E-06 | -0.476 | - | - |
| 17762 | ENSP00000418532 | UGT1A3 | 6.235E-06 | -0.476 | - | - |
| 17763 | ENSP00000355467 | SCCPDH | 8.701E-06 | -0.476 | - | - |
| 17764 | ENSP00000364567 | ADPRHL1 | 5.114E-06 | -0.476 | - | - |
| 17765 | ENSP00000405600 | SLC4A2 | 9.517E-06 | -0.476 | - | - |
| 17766 | ENSP00000286688 | C8orf37 | 1.536E-05 | -0.476 | - | - |
| 17767 | ENSP00000375909 | SPHKAP | 6.936E-06 | -0.476 | - | - |
| 17768 | ENSP00000333744 | GPR19 | 8.694E-06 | -0.476 | - | - |
| 17769 | ENSP00000363645 | AWAT1 | 3.936E-06 | -0.477 | - | - |
| 17770 | ENSP00000329295 | NXPH3 | 6.091E-06 | -0.477 | - | - |
| 17771 | ENSP00000250823 | VCY1B | 4.759E-06 | -0.477 | - | - |
| 17772 | ENSP00000374036 | RPH3A | 8.947E-06 | -0.477 | - | - |
| 17773 | ENSP00000294889 | C1orf115 | 2.668E-06 | -0.477 | - | - |
| 17774 | ENSP00000364515 | BTBD17 | 3.219E-06 | -0.477 | - | - |
| 17775 | ENSP00000263265 | PLEKHA4 | 1.239E-05 | -0.477 | - | - |
| 17776 | ENSP00000239316 | INSL4 | 1.687E-05 | -0.477 | - | - |
| 17777 | ENSP00000300226 | MS4A8 | 1.193E-05 | -0.477 | - | - |
| 17778 | ENSP00000164640 | PDZD4 | 2.361E-05 | -0.477 | - | - |
| 17779 | ENSP00000322097 | TPRXL | 6.282E-06 | -0.478 | - | - |
| 17780 | ENSP00000434359 | PUF60 | 8.679E-06 | -0.478 | - | - |
| 17781 | ENSP00000362058 | NDUFS5 | 4.702E-06 | -0.478 | - | - |
| 17782 | ENSP00000374399 | TRMT61A | 5.768E-06 | -0.478 | - | - |
| 17783 | ENSP00000454770 | ANKRD66 | 5.897E-06 | -0.478 | - | - |
| 17784 | ENSP00000468969 | WDR83OS | 4.748E-06 | -0.478 | - | - |
| 17785 | ENSP00000373586 | C2orf54 | 1.467E-06 | -0.478 | - | - |
| 17786 | ENSP00000240364 | FAM117A | 1.015E-05 | -0.478 | - | - |
| 17787 | ENSP00000359942 | KLHL31 | 8.293E-06 | -0.478 | - | - |
| 17788 | ENSP00000384610 | PNPLA7 | 1.615E-05 | -0.478 | - | - |
| 17789 | ENSP00000287474 | FRRS1 | 6.282E-06 | -0.478 | - | - |
| 17790 | ENSP00000295898 | C4orf36 | 1.356E-05 | -0.478 | - | - |
| 17791 | ENSP00000338330 | PAQR6 | 1.152E-05 | -0.478 | - | - |
| 17792 | ENSP00000308591 | GAL3ST3 | 1.094E-05 | -0.478 | - | - |
| 17793 | ENSP00000352288 | PLXNB2 | 8.823E-06 | -0.478 | - | - |
| 17794 | ENSP00000328800 | WBP2NL | 1.051E-05 | -0.478 | - | - |
| 17795 | ENSP00000363398 | EXTL1 | 1.150E-05 | -0.478 | - | - |
| 17796 | ENSP00000335522 | WDR86 | 1.008E-05 | -0.478 | - | - |
| 17797 | ENSP00000298715 | - | 1.086E-05 | -0.478 | - | - |
| 17798 | ENSP00000455755 | RP11-12J10.3 | 1.003E-06 | -0.478 | - | - |
| 17799 | ENSP00000309036 | TSPAN17 | 1.203E-05 | -0.478 | - | - |
| 17800 | ENSP00000358762 | UBE3D | 4.411E-06 | -0.479 | - | - |
| 17801 | ENSP00000354498 | PHLDB1 | 6.149E-06 | -0.479 | - | - |
| 17802 | ENSP00000321617 | ANKZF1 | 7.371E-06 | -0.479 | - | - |
| 17803 | ENSP00000264233 | POLQ | 5.223E-06 | -0.479 | - | - |
| 17804 | ENSP00000365007 | DNAJC16 | 5.424E-06 | -0.479 | - | - |
| 17805 | ENSP00000340776 | CCDC108 | 6.957E-06 | -0.479 | - | - |
| 17806 | ENSP00000288065 | ARMC12 | 1.080E-05 | -0.479 | - | - |
| 17807 | ENSP00000295237 | XIRP2 | 1.540E-05 | -0.479 | - | - |
| 17808 | ENSP00000258034 | TAAR5 | 1.528E-05 | -0.479 | - | - |
| 17809 | ENSP00000287387 | WDYHV1 | 7.731E-06 | -0.480 | - | - |
| 17810 | ENSP00000396040 | PCED1B | 7.206E-06 | -0.480 | - | - |
| 17811 | ENSP00000386971 | POTEG | 7.286E-06 | -0.480 | - | - |
| 17812 | ENSP00000384967 | VSTM2A | 4.095E-06 | -0.480 | - | - |
| 17813 | ENSP00000377878 | ATP5G2 | 6.259E-06 | -0.480 | - | - |
| 17814 | ENSP00000341782 | GDAP1L1 | 8.040E-06 | -0.480 | - | - |
| 17815 | ENSP00000288710 | DRC1 | 3.749E-06 | -0.480 | - | - |
| 17816 | ENSP00000248879 | DGCR6L | 1.100E-05 | -0.480 | - | - |
| 17817 | ENSP00000423630 | FBXL7 | 5.159E-06 | -0.480 | - | - |
| 17818 | ENSP00000260403 | TMEM62 | 6.510E-06 | -0.480 | - | - |
| 17819 | ENSP00000307801 | ZPLD1 | 7.870E-06 | -0.480 | - | - |
| 17820 | ENSP00000394168 | SLCO1B7 | 7.430E-06 | -0.480 | - | - |
| 17821 | ENSP00000350881 | GRAMD1C | 3.767E-06 | -0.480 | - | - |
| 17822 | ENSP00000359596 | CLCA2 | 1.061E-05 | -0.481 | - | - |
| 17823 | ENSP00000359904 | TYW3 | 5.184E-06 | -0.481 | - | - |
| 17824 | ENSP00000262301 | LMF1 | 1.526E-05 | -0.481 | - | - |
| 17825 | ENSP00000376548 | GALNTL5 | 2.057E-06 | -0.481 | - | - |
| 17826 | ENSP00000357812 | TDRKH | 5.022E-06 | -0.481 | - | - |
| 17827 | ENSP00000452776 | SLC12A6 | 1.021E-05 | -0.481 | - | - |
| 17828 | ENSP00000355261 | SMG5 | 4.447E-06 | -0.481 | - | - |
| 17829 | ENSP00000275198 | TAAR6 | 1.631E-05 | -0.482 | - | - |
| 17830 | ENSP00000253381 | DEFB118 | 9.657E-06 | -0.482 | - | - |
| 17831 | ENSP00000468367 | RAB5C | 1.363E-05 | -0.482 | - | - |
| 17832 | ENSP00000333300 | HSD17B13 | 1.304E-05 | -0.482 | - | - |
| 17833 | ENSP00000328698 | C10orf107 | 1.216E-05 | -0.482 | - | - |
| 17834 | ENSP00000318982 | EPSTI1 | 1.474E-05 | -0.482 | - | - |
| 17835 | ENSP00000235532 | OSCP1 | 1.389E-05 | -0.482 | - | - |
| 17836 | ENSP00000310031 | CDRT4 | 8.216E-06 | -0.482 | - | - |
| 17837 | ENSP00000361681 | SLC25A53 | 4.389E-06 | -0.482 | - | - |
| 17838 | ENSP00000257626 | GSAP | 1.305E-05 | -0.483 | - | - |
| 17839 | ENSP00000337022 | TNNI1 | 1.362E-05 | -0.483 | - | - |
| 17840 | ENSP00000218230 | PCSK1N | 1.514E-05 | -0.483 | - | - |
| 17841 | ENSP00000423049 | C5orf27 | 9.075E-06 | -0.483 | - | - |
| 17842 | ENSP00000362873 | NDUFA8 | 4.022E-06 | -0.483 | - | - |
| 17843 | ENSP00000442464 | NCEH1 | 6.307E-06 | -0.483 | - | - |
| 17844 | ENSP00000454699 | DDX19B | 7.401E-06 | -0.483 | - | - |
| 17845 | ENSP00000346572 | 61E3.4 | 1.363E-05 | -0.483 | - | - |
| 17846 | ENSP00000406909 | PHOSPHO1 | 2.856E-06 | -0.483 | - | - |
| 17847 | ENSP00000366827 | ST8SIA6 | 5.254E-06 | -0.483 | - | - |
| 17848 | ENSP00000282185 | ATG10 | 1.700E-05 | -0.484 | - | - |
| 17849 | ENSP00000300079 | GLYATL1 | 1.222E-05 | -0.484 | - | - |
| 17850 | ENSP00000420840 | PCDHA1 | 4.928E-06 | -0.484 | - | - |
| 17851 | ENSP00000396673 | FTSJ3 | 6.634E-06 | -0.484 | - | - |
| 17852 | ENSP00000369855 | ASB9 | 8.838E-06 | -0.484 | - | - |
| 17853 | ENSP00000267260 | SRRM4 | 8.970E-06 | -0.484 | - | - |
| 17854 | ENSP00000382086 | EVPLL | 9.717E-06 | -0.484 | - | - |
| 17855 | ENSP00000451628 | - | 8.069E-06 | -0.484 | - | - |
| 17856 | ENSP00000366477 | C10orf112 | 6.752E-06 | -0.484 | - | - |
| 17857 | ENSP00000423822 | GPX8 | 8.674E-06 | -0.484 | - | - |
| 17858 | ENSP00000360165 | ACTRT1 | 1.118E-05 | -0.485 | - | - |
| 17859 | ENSP00000187762 | TMEM38A | 1.595E-05 | -0.485 | - | - |
| 17860 | ENSP00000329008 | S100A7A | 1.178E-05 | -0.485 | - | - |
| 17861 | ENSP00000376390 | RAB37 | 4.116E-06 | -0.485 | - | - |
| 17862 | ENSP00000418428 | CHCHD10 | 9.341E-06 | -0.485 | - | - |
| 17863 | ENSP00000263062 | EPC1 | 1.583E-05 | -0.485 | - | - |
| 17864 | ENSP00000260276 | C11orf1 | 1.120E-05 | -0.485 | - | - |
| 17865 | ENSP00000339621 | LCN6 | 1.190E-05 | -0.485 | - | - |
| 17866 | ENSP00000402219 | VSIG1 | 7.694E-06 | -0.485 | - | - |
| 17867 | ENSP00000296529 | TMEM144 | 1.082E-05 | -0.485 | - | - |
| 17868 | ENSP00000352021 | NOL12 | 9.600E-06 | -0.485 | - | - |
| 17869 | ENSP00000297866 | CXorf22 | 4.725E-06 | -0.486 | - | - |
| 17870 | ENSP00000399668 | TSPY4 | 1.550E-05 | -0.486 | - | - |
| 17871 | ENSP00000229729 | SLC44A4 | 1.158E-05 | -0.486 | - | - |
| 17872 | ENSP00000377344 | DDX60 | 8.925E-06 | -0.486 | - | - |
| 17873 | ENSP00000328352 | KCTD11 | 1.041E-05 | -0.486 | - | - |
| 17874 | ENSP00000304108 | FAM171B | 6.987E-06 | -0.486 | - | - |
| 17875 | ENSP00000332809 | C2orf71 | 6.859E-06 | -0.486 | - | - |
| 17876 | ENSP00000370761 | LYRM5 | 3.215E-06 | -0.486 | - | - |
| 17877 | ENSP00000301825 | ENTPD3 | 1.296E-05 | -0.486 | - | - |
| 17878 | ENSP00000353270 | ERGIC2 | 7.019E-06 | -0.486 | - | - |
| 17879 | ENSP00000298223 | FOLR2 | 7.368E-06 | -0.486 | - | - |
| 17880 | ENSP00000358358 | GJA10 | 3.587E-06 | -0.486 | - | - |
| 17881 | ENSP00000361076 | RPL7A | 9.148E-06 | -0.486 | - | - |
| 17882 | ENSP00000356518 | SHCBP1L | 7.209E-06 | -0.487 | - | - |
| 17883 | ENSP00000383939 | SETD5 | 7.247E-06 | -0.487 | - | - |
| 17884 | ENSP00000356587 | NPHS2 | 6.583E-06 | -0.487 | - | - |
| 17885 | ENSP00000375387 | AL356585.1 | 4.562E-06 | -0.487 | - | - |
| 17886 | ENSP00000359131 | EXTL2 | 6.089E-06 | -0.487 | - | - |
| 17887 | ENSP00000380679 | CCDC40 | 5.840E-06 | -0.487 | - | - |
| 17888 | ENSP00000249330 | VGF | 1.642E-05 | -0.488 | - | - |
| 17889 | ENSP00000326846 | C14orf28 | 8.730E-06 | -0.488 | - | - |
| 17890 | ENSP00000394700 | SYT16 | 1.246E-05 | -0.488 | - | - |
| 17891 | ENSP00000244296 | PSG1 | 5.870E-06 | -0.488 | - | - |
| 17892 | ENSP00000321455 | MICU3 | 1.332E-05 | -0.488 | - | - |
| 17893 | ENSP00000368207 | FTHL17 | 5.545E-06 | -0.488 | - | - |
| 17894 | ENSP00000356076 | DYRK3 | 1.420E-05 | -0.488 | - | - |
| 17895 | ENSP00000365637 | KIAA1217 | 5.803E-06 | -0.488 | - | - |
| 17896 | ENSP00000371451 | DNAJC21 | 4.404E-06 | -0.488 | - | - |
| 17897 | ENSP00000381824 | SAC3D1 | 1.054E-05 | -0.489 | - | - |
| 17898 | ENSP00000273695 | TM4SF19 | 7.884E-06 | -0.489 | - | - |
| 17899 | ENSP00000308750 | CBX2 | 1.090E-05 | -0.489 | - | - |
| 17900 | ENSP00000359505 | C6orf57 | 5.472E-06 | -0.489 | - | - |
| 17901 | ENSP00000360817 | ZNFX1 | 9.875E-06 | -0.489 | - | - |
| 17902 | ENSP00000269385 | CBX8 | 1.083E-05 | -0.489 | - | - |
| 17903 | ENSP00000322530 | PGBD5 | 1.155E-05 | -0.489 | - | - |
| 17904 | ENSP00000202677 | RALGAPA2 | 4.848E-06 | -0.489 | - | - |
| 17905 | ENSP00000357807 | THEM5 | 5.885E-06 | -0.489 | - | - |
| 17906 | ENSP00000357754 | SMCP | 6.100E-06 | -0.489 | - | - |
| 17907 | ENSP00000295297 | C1QTNF7 | 9.401E-06 | -0.489 | - | - |
| 17908 | ENSP00000315731 | METRNL | 9.268E-06 | -0.490 | - | - |
| 17909 | ENSP00000348132 | STK31 | 7.196E-06 | -0.490 | - | - |
| 17910 | ENSP00000358886 | ATXN7L2 | 8.101E-06 | -0.490 | - | - |
| 17911 | ENSP00000404464 | CCBE1 | 8.665E-06 | -0.490 | - | - |
| 17912 | ENSP00000383143 | SLITRK6 | 1.056E-05 | -0.490 | - | - |
| 17913 | ENSP00000360268 | ALDH18A1 | 9.907E-06 | -0.490 | - | - |
| 17914 | ENSP00000328677 | C2CD4C | 7.206E-06 | -0.490 | - | - |
| 17915 | ENSP00000331867 | TAS1R1 | 1.451E-05 | -0.491 | - | - |
| 17916 | ENSP00000382717 | WDR19 | 5.138E-06 | -0.491 | - | - |
| 17917 | ENSP00000263765 | PRDM11 | 7.797E-06 | -0.491 | - | - |
| 17918 | ENSP00000353078 | NRAP | 1.208E-05 | -0.491 | - | - |
| 17919 | ENSP00000351480 | SPECC1L | 1.157E-05 | -0.491 | - | - |
| 17920 | ENSP00000290374 | GJD2 | 9.243E-06 | -0.491 | - | - |
| 17921 | ENSP00000417901 | GPHN | 1.094E-05 | -0.492 | - | - |
| 17922 | ENSP00000420976 | DLEU7 | 6.400E-06 | -0.492 | - | - |
| 17923 | ENSP00000221573 | SNAPC2 | 1.539E-05 | -0.492 | - | - |
| 17924 | ENSP00000219689 | USP31 | 6.989E-06 | -0.492 | - | - |
| 17925 | ENSP00000264428 | GLRB | 1.224E-05 | -0.492 | - | - |
| 17926 | ENSP00000231136 | PCDHB6 | 1.620E-05 | -0.493 | - | - |
| 17927 | ENSP00000395653 | SLC46A1 | 7.676E-06 | -0.493 | - | - |
| 17928 | ENSP00000334153 | RFTN1 | 4.247E-06 | -0.493 | - | - |
| 17929 | ENSP00000397259 | ATXN7L3 | 4.580E-06 | -0.493 | - | - |
| 17930 | ENSP00000317836 | PRR15 | 1.165E-05 | -0.493 | - | - |
| 17931 | ENSP00000413074 | ADAL | 8.990E-06 | -0.493 | - | - |
| 17932 | ENSP00000311447 | ASPHD1 | 7.576E-06 | -0.493 | - | - |
| 17933 | ENSP00000342356 | OSTN | 8.522E-06 | -0.493 | - | - |
| 17934 | ENSP00000289166 | FAM46B | 1.018E-05 | -0.493 | - | - |
| 17935 | ENSP00000280481 | FREM2 | 1.197E-05 | -0.493 | - | - |
| 17936 | ENSP00000295461 | NIPAL1 | 6.383E-06 | -0.493 | - | - |
| 17937 | ENSP00000361636 | TNNC2 | 1.480E-05 | -0.493 | - | - |
| 17938 | ENSP00000411197 | TBC1D9 | 6.332E-06 | -0.494 | - | - |
| 17939 | ENSP00000167588 | KRT20 | 1.523E-05 | -0.494 | - | - |
| 17940 | ENSP00000269080 | ABCA8 | 1.669E-05 | -0.494 | - | - |
| 17941 | ENSP00000261234 | TMEM5 | 5.686E-06 | -0.494 | - | - |
| 17942 | ENSP00000295694 | TMEM79 | 9.152E-06 | -0.494 | - | - |
| 17943 | ENSP00000309285 | CCDC96 | 2.992E-06 | -0.494 | - | - |
| 17944 | ENSP00000431040 | MRPL22 | 3.297E-06 | -0.494 | - | - |
| 17945 | ENSP00000251646 | KRT33B | 1.267E-05 | -0.494 | - | - |
| 17946 | ENSP00000243578 | B9D2 | 9.255E-06 | -0.494 | - | - |
| 17947 | ENSP00000221554 | CCDC130 | 1.163E-05 | -0.495 | - | - |
| 17948 | ENSP00000349970 | ERGIC3 | 6.651E-06 | -0.495 | - | - |
| 17949 | ENSP00000311833 | SLC37A2 | 9.714E-06 | -0.495 | - | - |
| 17950 | ENSP00000263177 | LPPR5 | 1.248E-05 | -0.496 | - | - |
| 17951 | ENSP00000329158 | CLRN1 | 3.962E-06 | -0.496 | - | - |
| 17952 | ENSP00000324510 | BAIAP3 | 8.129E-06 | -0.496 | - | - |
| 17953 | ENSP00000431840 | SLC24A4 | 7.594E-06 | -0.496 | - | - |
| 17954 | ENSP00000158009 | FNDC8 | 9.565E-06 | -0.496 | - | - |
| 17955 | ENSP00000347277 | MRPL52 | 2.212E-06 | -0.496 | - | - |
| 17956 | ENSP00000365176 | TMEM51 | 1.019E-05 | -0.496 | - | - |
| 17957 | ENSP00000384700 | PAPOLB | 7.790E-06 | -0.496 | - | - |
| 17958 | ENSP00000323034 | FAM83A | 5.168E-06 | -0.496 | - | - |
| 17959 | ENSP00000243314 | MAGEA9B | 2.005E-05 | -0.496 | - | - |
| 17960 | ENSP00000377051 | ANKRD42 | 9.103E-06 | -0.496 | - | - |
| 17961 | ENSP00000381782 | SLC25A45 | 3.302E-06 | -0.497 | - | - |
| 17962 | ENSP00000367950 | SHROOM1 | 7.579E-06 | -0.497 | - | - |
| 17963 | ENSP00000454783 | CORO2B | 1.115E-05 | -0.497 | - | - |
| 17964 | ENSP00000389289 | AKR1B15 | 4.531E-06 | -0.497 | - | - |
| 17965 | ENSP00000320520 | FAM211B | 3.394E-06 | -0.497 | - | - |
| 17966 | ENSP00000219301 | PRSS54 | 1.333E-05 | -0.497 | - | - |
| 17967 | ENSP00000329735 | FAM212A | 6.805E-06 | -0.497 | - | - |
| 17968 | ENSP00000373684 | STK38L | 1.439E-05 | -0.497 | - | - |
| 17969 | ENSP00000310969 | STON1 | 4.530E-06 | -0.497 | - | - |
| 17970 | ENSP00000279024 | KIAA1755 | 7.717E-06 | -0.497 | - | - |
| 17971 | ENSP00000300113 | CHP2 | 8.375E-06 | -0.497 | - | - |
| 17972 | ENSP00000300591 | LOXHD1 | 8.183E-06 | -0.497 | - | - |
| 17973 | ENSP00000373566 | CCDC158 | 4.890E-06 | -0.497 | - | - |
| 17974 | ENSP00000385727 | MAST4 | 8.623E-06 | -0.498 | - | - |
| 17975 | ENSP00000414066 | ACN9 | 4.763E-06 | -0.498 | - | - |
| 17976 | ENSP00000321627 | ANKFN1 | 9.931E-06 | -0.498 | - | - |
| 17977 | ENSP00000247706 | ABHD8 | 9.252E-06 | -0.498 | - | - |
| 17978 | ENSP00000368320 | MAGEB6 | 1.357E-05 | -0.498 | - | - |
| 17979 | ENSP00000366475 | ATG2A | 5.190E-06 | -0.498 | - | - |
| 17980 | ENSP00000308354 | TIGD3 | 4.614E-06 | -0.498 | - | - |
| 17981 | ENSP00000295367 | SPRR3 | 1.233E-05 | -0.499 | - | - |
| 17982 | ENSP00000295452 | GABRG1 | 1.278E-05 | -0.499 | - | - |
| 17983 | ENSP00000417229 | EIF2A | 6.536E-06 | -0.499 | - | - |
| 17984 | ENSP00000384290 | WDR60 | 1.102E-05 | -0.499 | - | - |
| 17985 | ENSP00000359788 | DDX26B | 6.302E-06 | -0.499 | - | - |
| 17986 | ENSP00000442068 | CDCA3 | 2.997E-06 | -0.500 | - | - |
| 17987 | ENSP00000223026 | HYAL4 | 1.505E-05 | -0.500 | - | - |
| 17988 | ENSP00000262095 | - | 1.133E-05 | -0.500 | - | - |
| 17989 | ENSP00000349989 | CXorf24 | 8.150E-06 | -0.500 | - | - |
| 17990 | ENSP00000421307 | RP11-455G16.1 | 2.270E-06 | -0.500 | - | - |
| 17991 | ENSP00000334940 | GGN | 1.153E-05 | -0.500 | - | - |
| 17992 | ENSP00000339820 | TSPAN18 | 1.360E-05 | -0.500 | - | - |
| 17993 | ENSP00000391950 | ANKRD36 | 8.089E-06 | -0.500 | - | - |
| 17994 | ENSP00000455547 | TBC1D24 | 7.237E-06 | -0.500 | - | - |
| 17995 | ENSP00000297290 | BRI3 | 9.601E-06 | -0.500 | - | - |
| 17996 | ENSP00000376349 | NUDT14 | 7.935E-06 | -0.500 | - | - |
| 17997 | ENSP00000318158 | MRPS24 | 3.514E-06 | -0.500 | - | - |
| 17998 | ENSP00000013222 | INMT | 1.456E-05 | -0.500 | - | - |
| 17999 | ENSP00000349456 | CLCC1 | 4.397E-06 | -0.501 | - | - |
| 18000 | ENSP00000379339 | RPS29 | 7.903E-06 | -0.501 | - | - |
| 18001 | ENSP00000466834 | RAD51D | 1.646E-05 | -0.501 | - | - |
| 18002 | ENSP00000335024 | SERPINA11 | 1.294E-05 | -0.501 | - | - |
| 18003 | ENSP00000379326 | SLC5A12 | 1.163E-05 | -0.502 | - | - |
| 18004 | ENSP00000367462 | OLAH | 4.392E-06 | -0.502 | - | - |
| 18005 | ENSP00000447001 | RPL18 | 5.784E-06 | -0.502 | - | - |
| 18006 | ENSP00000456434 | SDHD | 6.332E-06 | -0.502 | - | - |
| 18007 | ENSP00000381340 | GGT5 | 1.138E-05 | -0.502 | - | - |
| 18008 | ENSP00000294484 | PTCHD2 | 8.977E-06 | -0.502 | - | - |
| 18009 | ENSP00000350491 | PRIM1 | 5.592E-06 | -0.502 | - | - |
| 18010 | ENSP00000332208 | RFX6 | 8.921E-06 | -0.502 | - | - |
| 18011 | ENSP00000345868 | GJB4 | 6.716E-06 | -0.503 | - | - |
| 18012 | ENSP00000347689 | PDE4C | 1.004E-05 | -0.503 | - | - |
| 18013 | ENSP00000447000 | RP11-762I7.5 | 5.244E-06 | -0.503 | - | - |
| 18014 | ENSP00000409267 | AC006156.1 | 1.525E-05 | -0.503 | - | - |
| 18015 | ENSP00000356123 | KLHDC8A | 8.583E-06 | -0.503 | - | - |
| 18016 | ENSP00000369943 | GCOM1 | 5.697E-06 | -0.503 | - | - |
| 18017 | ENSP00000296882 | GJB7 | 7.615E-06 | -0.503 | - | - |
| 18018 | ENSP00000462799 | TBC1D29 | 3.788E-06 | -0.503 | - | - |
| 18019 | ENSP00000330433 | TM2D3 | 9.631E-06 | -0.503 | - | - |
| 18020 | ENSP00000284224 | CHST9 | 6.932E-06 | -0.503 | - | - |
| 18021 | ENSP00000303779 | EME2 | 8.035E-06 | -0.503 | - | - |
| 18022 | ENSP00000423541 | SV2C | 7.183E-06 | -0.503 | - | - |
| 18023 | ENSP00000371553 | NANOGNB | 6.785E-06 | -0.503 | - | - |
| 18024 | ENSP00000314560 | MAP6D1 | 8.335E-06 | -0.503 | - | - |
| 18025 | ENSP00000304846 | OR2AT4 | 1.009E-06 | -0.503 | - | - |
| 18026 | ENSP00000366623 | FAM203B | 3.080E-06 | -0.504 | - | - |
| 18027 | ENSP00000353114 | AHNAK2 | 5.189E-06 | -0.504 | - | - |
| 18028 | ENSP00000454836 | TBL3 | 6.726E-06 | -0.504 | - | - |
| 18029 | ENSP00000290438 | GOLGA6A | 1.163E-05 | -0.504 | - | - |
| 18030 | ENSP00000361337 | PLAC9 | 7.638E-06 | -0.504 | - | - |
| 18031 | ENSP00000355968 | SLC30A1 | 7.171E-06 | -0.504 | - | - |
| 18032 | ENSP00000346173 | ETFB | 6.857E-06 | -0.504 | - | - |
| 18033 | ENSP00000397879 | VPS8 | 4.012E-06 | -0.504 | - | - |
| 18034 | ENSP00000399235 | FOLR3 | 3.857E-06 | -0.504 | - | - |
| 18035 | ENSP00000325239 | MYLPF | 1.251E-05 | -0.504 | - | - |
| 18036 | ENSP00000384582 | GPR98 | 1.085E-05 | -0.504 | - | - |
| 18037 | ENSP00000430236 | ALG11 | 9.559E-06 | -0.504 | - | - |
| 18038 | ENSP00000383611 | CCNL2 | 8.216E-06 | -0.504 | - | - |
| 18039 | ENSP00000010132 | DYRK4 | 7.649E-06 | -0.505 | - | - |
| 18040 | ENSP00000358126 | VPS45 | 5.303E-06 | -0.505 | - | - |
| 18041 | ENSP00000235628 | NT5C1A | 1.233E-05 | -0.505 | - | - |
| 18042 | ENSP00000274849 | ABT1 | 1.132E-05 | -0.505 | - | - |
| 18043 | ENSP00000394008 | GABARAPL3 | 9.735E-06 | -0.505 | - | - |
| 18044 | ENSP00000301419 | C19orf48 | 9.673E-06 | -0.505 | - | - |
| 18045 | ENSP00000370192 | SLC46A3 | 1.055E-05 | -0.505 | - | - |
| 18046 | ENSP00000363634 | TXNDC8 | 8.301E-06 | -0.505 | - | - |
| 18047 | ENSP00000334594 | SLC10A7 | 6.072E-06 | -0.505 | - | - |
| 18048 | ENSP00000299045 | TCP11L2 | 7.477E-06 | -0.505 | - | - |
| 18049 | ENSP00000417176 | RNF183 | 2.215E-06 | -0.505 | - | - |
| 18050 | ENSP00000403396 | FAM126A | 2.271E-06 | -0.505 | - | - |
| 18051 | ENSP00000382492 | RADIL | 5.834E-06 | -0.505 | - | - |
| 18052 | ENSP00000397644 | RFX7 | 3.580E-06 | -0.505 | - | - |
| 18053 | ENSP00000438949 | CACNB4 | 6.874E-06 | -0.506 | - | - |
| 18054 | ENSP00000309673 | OR52W1 | 1.007E-06 | -0.506 | - | - |
| 18055 | ENSP00000252939 | CALY | 1.215E-05 | -0.506 | - | - |
| 18056 | ENSP00000357474 | CEP85L | 4.338E-06 | -0.506 | - | - |
| 18057 | ENSP00000361923 | CHCHD1 | 3.001E-06 | -0.506 | - | - |
| 18058 | ENSP00000374219 | ALKBH8 | 7.365E-06 | -0.506 | - | - |
| 18059 | ENSP00000277309 | OR1K1 | 9.883E-07 | -0.506 | - | - |
| 18060 | ENSP00000384115 | MEI1 | 8.529E-06 | -0.507 | - | - |
| 18061 | ENSP00000336693 | ABHD12B | 6.038E-06 | -0.507 | - | - |
| 18062 | ENSP00000362159 | SLC25A25 | 8.119E-06 | -0.507 | - | - |
| 18063 | ENSP00000278243 | PGAP2 | 1.215E-05 | -0.507 | - | - |
| 18064 | ENSP00000280527 | CRIM1 | 1.373E-05 | -0.507 | - | - |
| 18065 | ENSP00000298231 | PHOX2A | 1.210E-05 | -0.507 | - | - |
| 18066 | ENSP00000303246 | ACOT12 | 9.285E-06 | -0.508 | - | - |
| 18067 | ENSP00000286955 | FUT6 | 1.192E-05 | -0.508 | - | - |
| 18068 | ENSP00000378812 | CHCHD2 | 3.761E-06 | -0.508 | - | - |
| 18069 | ENSP00000265299 | FAM188B | 9.123E-06 | -0.508 | - | - |
| 18070 | ENSP00000312368 | SNX31 | 1.284E-05 | -0.508 | - | - |
| 18071 | ENSP00000400113 | ZDHHC13 | 1.040E-05 | -0.508 | - | - |
| 18072 | ENSP00000363899 | NIPSNAP3A | 4.656E-06 | -0.508 | - | - |
| 18073 | ENSP00000473200 | RP11-295P9.3 | 2.808E-06 | -0.509 | - | - |
| 18074 | ENSP00000344140 | PPP1R32 | 7.011E-06 | -0.509 | - | - |
| 18075 | ENSP00000350961 | C9orf123 | 3.598E-06 | -0.509 | - | - |
| 18076 | ENSP00000449535 | ACSS3 | 6.576E-06 | -0.509 | - | - |
| 18077 | ENSP00000264231 | POPDC2 | 8.879E-06 | -0.509 | - | - |
| 18078 | ENSP00000357575 | TTC40 | 5.710E-06 | -0.509 | - | - |
| 18079 | ENSP00000460871 | RSL1D1 | 6.875E-06 | -0.509 | - | - |
| 18080 | ENSP00000359688 | RPF1 | 5.928E-06 | -0.510 | - | - |
| 18081 | ENSP00000361232 | DDX31 | 4.394E-06 | -0.510 | - | - |
| 18082 | ENSP00000449334 | RP11-345J4.3 | 2.630E-06 | -0.510 | - | - |
| 18083 | ENSP00000319531 | GCSH | 9.390E-06 | -0.510 | - | - |
| 18084 | ENSP00000281087 | ATP6V1G3 | 5.257E-06 | -0.510 | - | - |
| 18085 | ENSP00000366690 | SPATC1 | 1.705E-06 | -0.510 | - | - |
| 18086 | ENSP00000449223 | RP11-347C12.3 | 2.630E-06 | -0.510 | - | - |
| 18087 | ENSP00000332325 | ZNF703 | 7.467E-06 | -0.510 | - | - |
| 18088 | ENSP00000417464 | RRP1 | 8.422E-06 | -0.511 | - | - |
| 18089 | ENSP00000377840 | CACNB1 | 4.979E-06 | -0.511 | - | - |
| 18090 | ENSP00000359371 | BHLHE23 | 1.263E-05 | -0.511 | - | - |
| 18091 | ENSP00000335675 | PRELID2 | 7.318E-06 | -0.511 | - | - |
| 18092 | ENSP00000274545 | GABRA6 | 1.194E-05 | -0.511 | - | - |
| 18093 | ENSP00000337446 | STARD3 | 1.149E-05 | -0.511 | - | - |
| 18094 | ENSP00000381247 | EFCAB9 | 1.126E-05 | -0.511 | - | - |
| 18095 | ENSP00000223145 | GLCCI1 | 1.284E-05 | -0.511 | - | - |
| 18096 | ENSP00000409195 | TBC1D3H | 7.268E-06 | -0.512 | - | - |
| 18097 | ENSP00000370262 | FREM1 | 7.692E-06 | -0.512 | - | - |
| 18098 | ENSP00000401177 | NOL8 | 3.946E-06 | -0.512 | - | - |
| 18099 | ENSP00000339067 | FGFBP3 | 6.336E-06 | -0.512 | - | - |
| 18100 | ENSP00000323087 | WSCD1 | 5.210E-06 | -0.512 | - | - |
| 18101 | ENSP00000328563 | OR4D9 | 1.162E-06 | -0.512 | - | - |
| 18102 | ENSP00000245121 | KATNAL2 | 7.525E-06 | -0.513 | - | - |
| 18103 | ENSP00000344489 | RNF133 | 9.449E-06 | -0.513 | - | - |
| 18104 | ENSP00000346466 | WDR91 | 6.720E-06 | -0.513 | - | - |
| 18105 | ENSP00000354772 | MVB12B | 4.873E-06 | -0.513 | - | - |
| 18106 | ENSP00000334308 | SYNE3 | 1.083E-05 | -0.513 | - | - |
| 18107 | ENSP00000354519 | INPP5F | 5.875E-06 | -0.513 | - | - |
| 18108 | ENSP00000369399 | SLC4A11 | 9.293E-06 | -0.514 | - | - |
| 18109 | ENSP00000285600 | OR4K1 | 7.926E-06 | -0.514 | - | - |
| 18110 | ENSP00000433157 | BBIP1 | 2.644E-06 | -0.514 | - | - |
| 18111 | ENSP00000332511 | OLFML1 | 1.065E-05 | -0.515 | - | - |
| 18112 | ENSP00000271751 | KCNH1 | 1.306E-05 | -0.515 | - | - |
| 18113 | ENSP00000303740 | B3GALT1 | 1.360E-05 | -0.515 | - | - |
| 18114 | ENSP00000371734 | KANK1 | 8.084E-06 | -0.515 | - | - |
| 18115 | ENSP00000261381 | XYLT1 | 9.901E-06 | -0.515 | - | - |
| 18116 | ENSP00000217901 | IDH3G | 1.034E-05 | -0.515 | - | - |
| 18117 | ENSP00000442563 | POLG2 | 6.585E-06 | -0.515 | - | - |
| 18118 | ENSP00000270620 | FAM71E1 | 6.462E-06 | -0.515 | - | - |
| 18119 | ENSP00000243878 | ENKD1 | 1.143E-05 | -0.515 | - | - |
| 18120 | ENSP00000251303 | SLX1A | 7.302E-06 | -0.516 | - | - |
| 18121 | ENSP00000333845 | C11orf88 | 9.107E-06 | -0.516 | - | - |
| 18122 | ENSP00000302851 | SLC23A1 | 9.564E-06 | -0.516 | - | - |
| 18123 | ENSP00000438455 | TIMM8B | 6.187E-06 | -0.516 | - | - |
| 18124 | ENSP00000323424 | ISG20L2 | 6.996E-06 | -0.517 | - | - |
| 18125 | ENSP00000456615 | RP11-697E2.6 | 9.669E-07 | -0.517 | - | - |
| 18126 | ENSP00000362206 | KLHL4 | 9.153E-06 | -0.517 | - | - |
| 18127 | ENSP00000318258 | CSRNP3 | 9.634E-06 | -0.517 | - | - |
| 18128 | ENSP00000380488 | GALNT9 | 2.543E-06 | -0.517 | - | - |
| 18129 | ENSP00000318395 | C5orf64 | 6.417E-06 | -0.517 | - | - |
| 18130 | ENSP00000260843 | GPR87 | 1.219E-05 | -0.517 | - | - |
| 18131 | ENSP00000381781 | TBC1D3B | 7.499E-06 | -0.518 | - | - |
| 18132 | ENSP00000330240 | CCDC34 | 6.991E-06 | -0.518 | - | - |
| 18133 | ENSP00000318502 | TGIF2LY | 1.461E-05 | -0.518 | - | - |
| 18134 | ENSP00000371548 | LGI2 | 4.206E-06 | -0.518 | - | - |
| 18135 | ENSP00000367476 | CEP104 | 5.816E-06 | -0.518 | - | - |
| 18136 | ENSP00000348460 | NPAS3 | 1.403E-05 | -0.518 | - | - |
| 18137 | ENSP00000361776 | BEX4 | 3.430E-06 | -0.518 | - | - |
| 18138 | ENSP00000383505 | KLHDC7A | 5.880E-06 | -0.518 | - | - |
| 18139 | ENSP00000215882 | SLC25A1 | 1.179E-05 | -0.518 | - | - |
| 18140 | ENSP00000347716 | DPF1 | 8.555E-06 | -0.519 | - | - |
| 18141 | ENSP00000301924 | TRIM35 | 8.510E-06 | -0.519 | - | - |
| 18142 | ENSP00000262442 | DNAH9 | 1.037E-05 | -0.519 | - | - |
| 18143 | ENSP00000334564 | POLR3C | 5.863E-06 | -0.519 | - | - |
| 18144 | ENSP00000332788 | ANO9 | 6.419E-06 | -0.519 | - | - |
| 18145 | ENSP00000217320 | TLDC2 | 6.867E-06 | -0.519 | - | - |
| 18146 | ENSP00000402338 | RPF2 | 8.259E-06 | -0.519 | - | - |
| 18147 | ENSP00000387303 | TDRD9 | 9.640E-06 | -0.520 | - | - |
| 18148 | ENSP00000375907 | PID1 | 8.649E-06 | -0.520 | - | - |
| 18149 | ENSP00000351138 | FAM73B | 3.563E-06 | -0.520 | - | - |
| 18150 | ENSP00000365040 | LY6G5B | 8.615E-06 | -0.520 | - | - |
| 18151 | ENSP00000219299 | CCDC113 | 4.683E-06 | -0.520 | - | - |
| 18152 | ENSP00000342889 | POLR2K | 7.775E-06 | -0.520 | - | - |
| 18153 | ENSP00000223864 | PLGRKT | 1.138E-05 | -0.520 | - | - |
| 18154 | ENSP00000253699 | ZFYVE20 | 7.643E-06 | -0.520 | - | - |
| 18155 | ENSP00000388320 | CTU2 | 4.154E-06 | -0.520 | - | - |
| 18156 | ENSP00000347003 | OR5K4 | 1.266E-06 | -0.520 | - | - |
| 18157 | ENSP00000423313 | GALNTL6 | 5.266E-06 | -0.520 | - | - |
| 18158 | ENSP00000357706 | S100A5 | 3.198E-06 | -0.521 | - | - |
| 18159 | ENSP00000258457 | C2orf49 | 1.011E-05 | -0.521 | - | - |
| 18160 | ENSP00000364005 | - | 6.322E-06 | -0.521 | - | - |
| 18161 | ENSP00000268281 | PRSS36 | 1.332E-05 | -0.521 | - | - |
| 18162 | ENSP00000435352 | B3GNT6 | 2.979E-06 | -0.521 | - | - |
| 18163 | ENSP00000362836 | KCTD20 | 2.680E-06 | -0.521 | - | - |
| 18164 | ENSP00000301457 | NDUFA7 | 1.007E-05 | -0.522 | - | - |
| 18165 | ENSP00000382349 | ACSM4 | 6.062E-06 | -0.522 | - | - |
| 18166 | ENSP00000267406 | CBLN3 | 8.438E-06 | -0.522 | - | - |
| 18167 | ENSP00000366629 | KM-PA-2 | 5.581E-06 | -0.522 | - | - |
| 18168 | ENSP00000310770 | GLIPR1L1 | 1.122E-05 | -0.522 | - | - |
| 18169 | ENSP00000363139 | CD164L2 | 7.908E-06 | -0.523 | - | - |
| 18170 | ENSP00000361306 | ATG4A | 6.719E-06 | -0.523 | - | - |
| 18171 | ENSP00000402297 | LACTBL1 | 3.373E-06 | -0.523 | - | - |
| 18172 | ENSP00000362255 | TSPO2 | 4.546E-06 | -0.523 | - | - |
| 18173 | ENSP00000428765 | LETM2 | 3.140E-06 | -0.523 | - | - |
| 18174 | ENSP00000332886 | FREM3 | 6.771E-06 | -0.523 | - | - |
| 18175 | ENSP00000347915 | TTC30A | 3.262E-06 | -0.523 | - | - |
| 18176 | ENSP00000347345 | POLR3H | 6.422E-06 | -0.524 | - | - |
| 18177 | ENSP00000373404 | MRPL2 | 4.587E-06 | -0.524 | - | - |
| 18178 | ENSP00000269298 | SAT2 | 9.869E-06 | -0.524 | - | - |
| 18179 | ENSP00000339750 | GTDC1 | 7.363E-06 | -0.524 | - | - |
| 18180 | ENSP00000326538 | CLEC18C | 1.139E-05 | -0.524 | - | - |
| 18181 | ENSP00000368402 | KCTD4 | 6.390E-06 | -0.524 | - | - |
| 18182 | ENSP00000309538 | DAPL1 | 1.253E-05 | -0.525 | - | - |
| 18183 | ENSP00000333638 | SLC52A2 | 5.195E-06 | -0.525 | - | - |
| 18184 | ENSP00000307275 | SCAMP3 | 6.965E-06 | -0.525 | - | - |
| 18185 | ENSP00000360642 | CC2D1B | 4.097E-06 | -0.525 | - | - |
| 18186 | ENSP00000298050 | CCDC67 | 6.508E-06 | -0.525 | - | - |
| 18187 | ENSP00000286428 | VBP1 | 1.244E-05 | -0.526 | - | - |
| 18188 | ENSP00000442770 | GPN3 | 3.484E-06 | -0.526 | - | - |
| 18189 | ENSP00000330269 | RESP18 | 8.362E-06 | -0.526 | - | - |
| 18190 | ENSP00000271139 | MOB3C | 1.275E-05 | -0.526 | - | - |
| 18191 | ENSP00000404079 | DNAJB5 | 8.645E-06 | -0.526 | - | - |
| 18192 | ENSP00000357892 | FAM24B | 6.702E-06 | -0.526 | - | - |
| 18193 | ENSP00000400325 | AP000350.10 | 4.300E-06 | -0.527 | - | - |
| 18194 | ENSP00000264601 | RAB17 | 8.920E-06 | -0.527 | - | - |
| 18195 | ENSP00000435777 | RPS13 | 7.880E-06 | -0.527 | - | - |
| 18196 | ENSP00000358512 | MAB21L3 | 7.215E-06 | -0.527 | - | - |
| 18197 | ENSP00000441269 | SLCO1B3 | 1.157E-05 | -0.527 | - | - |
| 18198 | ENSP00000346560 | FILIP1L | 9.038E-06 | -0.527 | - | - |
| 18199 | ENSP00000362894 | RTKN2 | 6.733E-06 | -0.527 | - | - |
| 18200 | ENSP00000361092 | SURF6 | 5.384E-06 | -0.528 | - | - |
| 18201 | ENSP00000344241 | ZMAT5 | 5.957E-06 | -0.528 | - | - |
| 18202 | ENSP00000357682 | FANK1 | 7.532E-06 | -0.528 | - | - |
| 18203 | ENSP00000262794 | MOV10L1 | 7.498E-06 | -0.528 | - | - |
| 18204 | ENSP00000360412 | NOC3L | 5.522E-06 | -0.528 | - | - |
| 18205 | ENSP00000264079 | MCOLN1 | 1.252E-05 | -0.529 | - | - |
| 18206 | ENSP00000393241 | RPS18 | 4.931E-06 | -0.529 | - | - |
| 18207 | ENSP00000393161 | TMEM151B | 2.755E-06 | -0.529 | - | - |
| 18208 | ENSP00000344219 | SUSD4 | 6.110E-06 | -0.529 | - | - |
| 18209 | ENSP00000361173 | KCNE1L | 9.708E-06 | -0.530 | - | - |
| 18210 | ENSP00000420954 | FAM156B | 3.038E-06 | -0.530 | - | - |
| 18211 | ENSP00000419395 | CDNF | 6.283E-06 | -0.530 | - | - |
| 18212 | ENSP00000296218 | DNALI1 | 6.499E-06 | -0.530 | - | - |
| 18213 | ENSP00000327465 | MYT1 | 1.274E-05 | -0.530 | - | - |
| 18214 | ENSP00000359000 | GBF1 | 8.858E-06 | -0.531 | - | - |
| 18215 | ENSP00000417052 | EBP | 4.496E-06 | -0.531 | - | - |
| 18216 | ENSP00000376794 | SLC35G2 | 3.742E-06 | -0.531 | - | - |
| 18217 | ENSP00000348813 | TBC1D7 | 5.222E-06 | -0.531 | - | - |
| 18218 | ENSP00000320794 | KIAA2018 | 7.565E-06 | -0.531 | - | - |
| 18219 | ENSP00000292114 | TMEM199 | 1.215E-05 | -0.531 | - | - |
| 18220 | ENSP00000344579 | FSCB | 6.562E-06 | -0.531 | - | - |
| 18221 | ENSP00000365080 | FAM155A | 4.403E-06 | -0.532 | - | - |
| 18222 | ENSP00000335511 | HTR3E | 7.970E-06 | -0.532 | - | - |
| 18223 | ENSP00000362650 | SCAI | 8.136E-06 | -0.532 | - | - |
| 18224 | ENSP00000378441 | C6orf1 | 5.089E-06 | -0.532 | - | - |
| 18225 | ENSP00000416289 | WDR27 | 2.829E-06 | -0.532 | - | - |
| 18226 | ENSP00000245615 | MBOAT7 | 1.049E-05 | -0.532 | - | - |
| 18227 | ENSP00000255266 | PDE6A | 9.230E-06 | -0.533 | - | - |
| 18228 | ENSP00000388207 | RNF148 | 3.962E-06 | -0.533 | - | - |
| 18229 | ENSP00000312700 | B3GNT8 | 1.066E-05 | -0.533 | - | - |
| 18230 | ENSP00000450040 | ASPG | 9.383E-06 | -0.533 | - | - |
| 18231 | ENSP00000272452 | SULT1C4 | 8.750E-06 | -0.534 | - | - |
| 18232 | ENSP00000466248 | PLK5 | 1.443E-05 | -0.534 | - | - |
| 18233 | ENSP00000299290 | TBATA | 1.148E-05 | -0.534 | - | - |
| 18234 | ENSP00000334996 | CCIN | 9.215E-06 | -0.534 | - | - |
| 18235 | ENSP00000405969 | ACBD4 | 4.202E-06 | -0.534 | - | - |
| 18236 | ENSP00000308870 | CLDN15 | 8.278E-06 | -0.534 | - | - |
| 18237 | ENSP00000343636 | GFRAL | 7.243E-06 | -0.534 | - | - |
| 18238 | ENSP00000418379 | TAF1L | 1.155E-05 | -0.534 | - | - |
| 18239 | ENSP00000334219 | MEGF8 | 5.996E-06 | -0.535 | - | - |
| 18240 | ENSP00000355273 | ST6GAL2 | 9.166E-06 | -0.535 | - | - |
| 18241 | ENSP00000273962 | TRMT10A | 5.063E-06 | -0.535 | - | - |
| 18242 | ENSP00000305924 | PPP4R4 | 5.630E-06 | -0.535 | - | - |
| 18243 | ENSP00000319601 | OR4N2 | 7.975E-06 | -0.535 | - | - |
| 18244 | ENSP00000369445 | KRTAP10-7 | 4.976E-06 | -0.535 | - | - |
| 18245 | ENSP00000219660 | AQP8 | 1.223E-05 | -0.535 | - | - |
| 18246 | ENSP00000307640 | TTC36 | 4.327E-06 | -0.535 | - | - |
| 18247 | ENSP00000315988 | DPY19L2 | 1.183E-05 | -0.536 | - | - |
| 18248 | ENSP00000419101 | CACNA2D3 | 6.751E-06 | -0.536 | - | - |
| 18249 | ENSP00000374353 | HAGHL | 8.877E-06 | -0.536 | - | - |
| 18250 | ENSP00000332772 | YIPF7 | 5.012E-06 | -0.536 | - | - |
| 18251 | ENSP00000234800 | SLC35E2B | 7.210E-06 | -0.536 | - | - |
| 18252 | ENSP00000454833 | AC025263.3 | 4.240E-06 | -0.536 | - | - |
| 18253 | ENSP00000313740 | SLC38A5 | 9.961E-06 | -0.537 | - | - |
| 18254 | ENSP00000272433 | SFXN5 | 9.488E-06 | -0.537 | - | - |
| 18255 | ENSP00000262376 | UBN1 | 6.406E-06 | -0.537 | - | - |
| 18256 | ENSP00000378160 | RPL34 | 8.502E-06 | -0.537 | - | - |
| 18257 | ENSP00000349902 | FAM115C | 7.477E-06 | -0.537 | - | - |
| 18258 | ENSP00000382064 | DTNA | 1.375E-05 | -0.537 | - | - |
| 18259 | ENSP00000248701 | SPINK2 | 5.652E-06 | -0.538 | - | - |
| 18260 | ENSP00000406293 | TCEA3 | 7.905E-06 | -0.538 | - | - |
| 18261 | ENSP00000357412 | LENEP | 4.999E-06 | -0.538 | - | - |
| 18262 | ENSP00000309504 | SLC26A7 | 1.117E-05 | -0.538 | - | - |
| 18263 | ENSP00000216487 | RIN3 | 7.330E-06 | -0.538 | - | - |
| 18264 | ENSP00000350676 | PHF8 | 9.750E-06 | -0.538 | - | - |
| 18265 | ENSP00000435466 | KIRREL3 | 9.586E-06 | -0.538 | - | - |
| 18266 | ENSP00000261530 | GPATCH2L | 1.474E-05 | -0.538 | - | - |
| 18267 | ENSP00000381856 | TOMM6 | 2.797E-06 | -0.539 | - | - |
| 18268 | ENSP00000252085 | PCDHGA12 | 1.301E-05 | -0.539 | - | - |
| 18269 | ENSP00000341051 | CLEC18B | 7.201E-06 | -0.539 | - | - |
| 18270 | ENSP00000324343 | UPB1 | 9.373E-06 | -0.539 | - | - |
| 18271 | ENSP00000017003 | XYLT2 | 8.290E-06 | -0.539 | - | - |
| 18272 | ENSP00000370849 | ITPRIPL2 | 2.382E-06 | -0.539 | - | - |
| 18273 | ENSP00000334042 | TAC4 | 6.912E-06 | -0.539 | - | - |
| 18274 | ENSP00000416453 | RP11-287D1.3 | 6.256E-06 | -0.540 | - | - |
| 18275 | ENSP00000284110 | HS3ST3A1 | 1.278E-05 | -0.540 | - | - |
| 18276 | ENSP00000366927 | ALDH1B1 | 1.175E-05 | -0.540 | - | - |
| 18277 | ENSP00000365243 | COX4I2 | 6.046E-06 | -0.540 | - | - |
| 18278 | ENSP00000299564 | CDYL2 | 9.683E-06 | -0.540 | - | - |
| 18279 | ENSP00000341765 | SPATA16 | 4.930E-06 | -0.541 | - | - |
| 18280 | ENSP00000311313 | CST6 | 9.270E-06 | -0.541 | - | - |
| 18281 | ENSP00000217254 | SLC52A3 | 1.413E-05 | -0.541 | - | - |
| 18282 | ENSP00000342535 | NLGN4Y | 6.723E-06 | -0.542 | - | - |
| 18283 | ENSP00000332448 | ADAT3 | 5.621E-06 | -0.542 | - | - |
| 18284 | ENSP00000380250 | SLC17A3 | 3.951E-06 | -0.542 | - | - |
| 18285 | ENSP00000351695 | PNLIPRP1 | 9.569E-06 | -0.542 | - | - |
| 18286 | ENSP00000419974 | CLASP2 | 8.580E-06 | -0.543 | - | - |
| 18287 | ENSP00000364013 | TMEFF1 | 3.940E-06 | -0.543 | - | - |
| 18288 | ENSP00000363298 | BSPRY | 5.659E-06 | -0.543 | - | - |
| 18289 | ENSP00000364520 | TAS1R2 | 1.242E-05 | -0.543 | - | - |
| 18290 | ENSP00000368318 | PUSL1 | 5.412E-06 | -0.543 | - | - |
| 18291 | ENSP00000348648 | JPH4 | 9.371E-06 | -0.543 | - | - |
| 18292 | ENSP00000215885 | PLA2G3 | 1.311E-05 | -0.543 | - | - |
| 18293 | ENSP00000324775 | TMIE | 7.744E-06 | -0.544 | - | - |
| 18294 | ENSP00000362814 | SERINC2 | 9.648E-06 | -0.544 | - | - |
| 18295 | ENSP00000354213 | HS3ST3B1 | 7.622E-06 | -0.544 | - | - |
| 18296 | ENSP00000292894 | THAP8 | 3.813E-06 | -0.544 | - | - |
| 18297 | ENSP00000365473 | MUC21 | 6.068E-06 | -0.544 | - | - |
| 18298 | ENSP00000269142 | TAF4B | 1.030E-05 | -0.544 | - | - |
| 18299 | ENSP00000318684 | VSIG2 | 8.670E-06 | -0.544 | - | - |
| 18300 | ENSP00000444293 | KDM5D | 1.801E-05 | -0.544 | - | - |
| 18301 | ENSP00000297977 | HDX | 1.349E-05 | -0.545 | - | - |
| 18302 | ENSP00000374482 | MRGPRE | 5.445E-06 | -0.545 | - | - |
| 18303 | ENSP00000383155 | SELM | 2.867E-06 | -0.546 | - | - |
| 18304 | ENSP00000429275 | TMEM125 | 4.471E-06 | -0.546 | - | - |
| 18305 | ENSP00000352820 | WDSUB1 | 6.661E-06 | -0.546 | - | - |
| 18306 | ENSP00000391742 | ZMYND15 | 4.127E-06 | -0.546 | - | - |
| 18307 | ENSP00000362684 | FAM167B | 7.412E-06 | -0.546 | - | - |
| 18308 | ENSP00000312189 | CPT1B | 9.790E-06 | -0.546 | - | - |
| 18309 | ENSP00000352849 | SIRPB2 | 9.593E-06 | -0.546 | - | - |
| 18310 | ENSP00000453785 | SNX1 | 6.832E-06 | -0.546 | - | - |
| 18311 | ENSP00000233615 | WBP1 | 1.207E-05 | -0.546 | - | - |
| 18312 | ENSP00000429473 | HS6ST2 | 5.828E-06 | -0.546 | - | - |
| 18313 | ENSP00000329803 | ZAR1 | 9.123E-06 | -0.547 | - | - |
| 18314 | ENSP00000395323 | KCNIP1 | 1.320E-05 | -0.547 | - | - |
| 18315 | ENSP00000428417 | RIMS1 | 1.068E-05 | -0.547 | - | - |
| 18316 | ENSP00000435619 | EID3 | 5.900E-06 | -0.547 | - | - |
| 18317 | ENSP00000469315 | KLK15 | 1.301E-05 | -0.547 | - | - |
| 18318 | ENSP00000379310 | CASC1 | 6.383E-06 | -0.547 | - | - |
| 18319 | ENSP00000288861 | CIB4 | 9.367E-06 | -0.548 | - | - |
| 18320 | ENSP00000381206 | RDH16 | 3.432E-06 | -0.548 | - | - |
| 18321 | ENSP00000302968 | CDY1 | 1.153E-05 | -0.548 | - | - |
| 18322 | ENSP00000330278 | PLEKHH1 | 7.050E-06 | -0.548 | - | - |
| 18323 | ENSP00000377616 | KRT222 | 5.902E-06 | -0.549 | - | - |
| 18324 | ENSP00000319179 | C1orf172 | 9.023E-06 | -0.549 | - | - |
| 18325 | ENSP00000307726 | OR2C1 | 9.388E-06 | -0.549 | - | - |
| 18326 | ENSP00000216416 | CNIH | 9.105E-06 | -0.549 | - | - |
| 18327 | ENSP00000440272 | RP13-512J5.1 | 2.643E-06 | -0.549 | - | - |
| 18328 | ENSP00000182096 | CRYBG3 | 7.630E-06 | -0.549 | - | - |
| 18329 | ENSP00000320217 | SLC26A10 | 1.132E-05 | -0.550 | - | - |
| 18330 | ENSP00000330264 | TMPRSS9 | 8.872E-06 | -0.550 | - | - |
| 18331 | ENSP00000237642 | FAM47E-STBD1 | 1.004E-05 | -0.550 | - | - |
| 18332 | ENSP00000446743 | CNPY2 | 1.021E-05 | -0.551 | - | - |
| 18333 | ENSP00000193391 | IMPG2 | 1.637E-05 | -0.551 | - | - |
| 18334 | ENSP00000453089 | RP11-468E2.6 | 8.606E-06 | -0.551 | - | - |
| 18335 | ENSP00000380605 | LYPD1 | 6.598E-06 | -0.551 | - | - |
| 18336 | ENSP00000230323 | TFEB | 8.106E-06 | -0.551 | - | - |
| 18337 | ENSP00000334854 | ZACN | 5.322E-06 | -0.552 | - | - |
| 18338 | ENSP00000429553 | NKX6-3 | 7.374E-06 | -0.552 | - | - |
| 18339 | ENSP00000362026 | TRMT2B | 5.299E-06 | -0.552 | - | - |
| 18340 | ENSP00000447852 | POC1B-GALNT4 | 1.157E-06 | -0.552 | - | - |
| 18341 | ENSP00000373272 | TRIM71 | 6.887E-06 | -0.552 | - | - |
| 18342 | ENSP00000240333 | SLC35B1 | 7.291E-06 | -0.552 | - | - |
| 18343 | ENSP00000314196 | MBOAT4 | 9.048E-06 | -0.552 | - | - |
| 18344 | ENSP00000317595 | TPPP2 | 3.802E-06 | -0.552 | - | - |
| 18345 | ENSP00000289388 | C2orf62 | 6.611E-06 | -0.552 | - | - |
| 18346 | ENSP00000292907 | COX7A1 | 9.719E-06 | -0.553 | - | - |
| 18347 | ENSP00000282611 | CATSPER3 | 8.798E-06 | -0.553 | - | - |
| 18348 | ENSP00000427888 | HS3ST5 | 5.386E-06 | -0.553 | - | - |
| 18349 | ENSP00000368295 | REXO1L1 | 8.329E-06 | -0.553 | - | - |
| 18350 | ENSP00000431184 | C6orf141 | 4.407E-06 | -0.553 | - | - |
| 18351 | ENSP00000361238 | RSPH9 | 4.074E-06 | -0.553 | - | - |
| 18352 | ENSP00000294794 | OLFML2B | 7.953E-06 | -0.553 | - | - |
| 18353 | ENSP00000384396 | FAM58A | 8.202E-06 | -0.553 | - | - |
| 18354 | ENSP00000257776 | MRAP2 | 8.820E-06 | -0.554 | - | - |
| 18355 | ENSP00000345295 | SH2D4B | 5.330E-06 | -0.554 | - | - |
| 18356 | ENSP00000255681 | MACROD1 | 6.386E-06 | -0.554 | - | - |
| 18357 | ENSP00000259711 | KIF13A | 1.014E-05 | -0.554 | - | - |
| 18358 | ENSP00000372316 | POLN | 8.482E-06 | -0.554 | - | - |
| 18359 | ENSP00000318641 | INTS3 | 6.384E-06 | -0.554 | - | - |
| 18360 | ENSP00000330634 | PKD1P1 | 1.158E-05 | -0.554 | - | - |
| 18361 | ENSP00000376268 | SEC14L1 | 3.998E-06 | -0.555 | - | - |
| 18362 | ENSP00000419704 | TIMM10B | 2.682E-06 | -0.555 | - | - |
| 18363 | ENSP00000280571 | RILPL2 | 8.334E-06 | -0.555 | - | - |
| 18364 | ENSP00000276480 | ST18 | 1.022E-05 | -0.555 | - | - |
| 18365 | ENSP00000396103 | MYT1L | 7.859E-06 | -0.555 | - | - |
| 18366 | ENSP00000441802 | PCDH7 | 4.717E-06 | -0.555 | - | - |
| 18367 | ENSP00000335038 | VSTM2B | 7.474E-06 | -0.556 | - | - |
| 18368 | ENSP00000382508 | CDIP1 | 2.696E-06 | -0.556 | - | - |
| 18369 | ENSP00000265304 | SSBP1 | 9.552E-06 | -0.556 | - | - |
| 18370 | ENSP00000286482 | MAGEA8 | 7.426E-06 | -0.556 | - | - |
| 18371 | ENSP00000266254 | TTLL1 | 5.586E-06 | -0.556 | - | - |
| 18372 | ENSP00000373176 | GTPBP8 | 3.151E-06 | -0.556 | - | - |
| 18373 | ENSP00000350552 | RFX4 | 7.236E-06 | -0.556 | - | - |
| 18374 | ENSP00000362136 | MANEAL | 7.152E-06 | -0.557 | - | - |
| 18375 | ENSP00000341682 | SLC26A9 | 1.200E-05 | -0.557 | - | - |
| 18376 | ENSP00000300179 | NYAP1 | 9.171E-06 | -0.558 | - | - |
| 18377 | ENSP00000324323 | TCTEX1D2 | 4.926E-06 | -0.558 | - | - |
| 18378 | ENSP00000252675 | FUT5 | 1.090E-05 | -0.558 | - | - |
| 18379 | ENSP00000380690 | CCDC64 | 4.608E-06 | -0.558 | - | - |
| 18380 | ENSP00000290390 | C2orf81 | 4.740E-06 | -0.559 | - | - |
| 18381 | ENSP00000420939 | SEPP1 | 6.929E-06 | -0.559 | - | - |
| 18382 | ENSP00000272203 | PLEKHA6 | 7.073E-06 | -0.559 | - | - |
| 18383 | ENSP00000358948 | TEX28P1 | 5.127E-06 | -0.560 | - | - |
| 18384 | ENSP00000258888 | ALPK3 | 1.415E-05 | -0.560 | - | - |
| 18385 | ENSP00000355353 | SLC34A3 | 7.703E-06 | -0.560 | - | - |
| 18386 | ENSP00000302166 | KCNK9 | 1.224E-05 | -0.561 | - | - |
| 18387 | ENSP00000359204 | LPPR4 | 4.880E-06 | -0.561 | - | - |
| 18388 | ENSP00000302144 | TMEM154 | 3.750E-06 | -0.561 | - | - |
| 18389 | ENSP00000401536 | RFX8 | 5.077E-06 | -0.561 | - | - |
| 18390 | ENSP00000363624 | CUTA | 3.959E-06 | -0.561 | - | - |
| 18391 | ENSP00000359300 | CETN2 | 1.664E-05 | -0.561 | - | - |
| 18392 | ENSP00000335575 | OR1J2 | 1.710E-06 | -0.562 | - | - |
| 18393 | ENSP00000355480 | EFCAB2 | 1.115E-05 | -0.562 | - | - |
| 18394 | ENSP00000367173 | RNF207 | 3.594E-06 | -0.562 | - | - |
| 18395 | ENSP00000347646 | SOX12 | 1.084E-05 | -0.563 | - | - |
| 18396 | ENSP00000340467 | LRRC16B | 6.622E-06 | -0.563 | - | - |
| 18397 | ENSP00000405165 | LIMS3 | 4.313E-06 | -0.563 | - | - |
| 18398 | ENSP00000468633 | SEC11C | 7.242E-06 | -0.563 | - | - |
| 18399 | ENSP00000307831 | GPR113 | 8.202E-06 | -0.563 | - | - |
| 18400 | ENSP00000362617 | NAP1L6 | 1.117E-05 | -0.563 | - | - |
| 18401 | ENSP00000300051 | LDHD | 4.203E-06 | -0.564 | - | - |
| 18402 | ENSP00000362071 | JPH2 | 8.535E-06 | -0.564 | - | - |
| 18403 | ENSP00000227065 | FAM149A | 1.357E-05 | -0.565 | - | - |
| 18404 | ENSP00000387123 | ALDH7A1 | 9.327E-06 | -0.565 | - | - |
| 18405 | ENSP00000369863 | FAM57B | 3.113E-06 | -0.565 | - | - |
| 18406 | ENSP00000470318 | CTD-3148I10.9 | 8.668E-06 | -0.565 | - | - |
| 18407 | ENSP00000307126 | DEGS2 | 1.273E-05 | -0.565 | - | - |
| 18408 | ENSP00000329943 | LRRC36 | 3.322E-06 | -0.565 | - | - |
| 18409 | ENSP00000363807 | SLC39A7 | 8.539E-06 | -0.565 | - | - |
| 18410 | ENSP00000353868 | PCED1A | 6.811E-06 | -0.566 | - | - |
| 18411 | ENSP00000334851 | RGS7BP | 5.640E-06 | -0.566 | - | - |
| 18412 | ENSP00000322181 | DPM2 | 5.740E-06 | -0.566 | - | - |
| 18413 | ENSP00000198801 | MOGAT2 | 1.021E-05 | -0.566 | - | - |
| 18414 | ENSP00000429931 | EIF3H | 7.356E-06 | -0.566 | - | - |
| 18415 | ENSP00000321343 | ST8SIA5 | 7.141E-06 | -0.566 | - | - |
| 18416 | ENSP00000370126 | SERF1B | 3.461E-06 | -0.567 | - | - |
| 18417 | ENSP00000355900 | SPATA17 | 5.967E-06 | -0.567 | - | - |
| 18418 | ENSP00000384887 | CEACAM19 | 3.550E-06 | -0.567 | - | - |
| 18419 | ENSP00000264037 | TECTA | 8.963E-06 | -0.568 | - | - |
| 18420 | ENSP00000005284 | CACNG3 | 1.248E-05 | -0.568 | - | - |
| 18421 | ENSP00000286063 | PDE11A | 6.374E-06 | -0.568 | - | - |
| 18422 | ENSP00000361454 | NCBP2L | 7.042E-06 | -0.568 | - | - |
| 18423 | ENSP00000322300 | TMTC2 | 1.183E-05 | -0.568 | - | - |
| 18424 | ENSP00000294435 | RBP7 | 8.302E-06 | -0.568 | - | - |
| 18425 | ENSP00000274565 | SPINK7 | 8.980E-06 | -0.569 | - | - |
| 18426 | ENSP00000321810 | TRIT1 | 8.492E-06 | -0.569 | - | - |
| 18427 | ENSP00000457718 | COG8 | 2.344E-06 | -0.569 | - | - |
| 18428 | ENSP00000433967 | TM9SF1 | 6.190E-06 | -0.569 | - | - |
| 18429 | ENSP00000305424 | OR1F1 | 9.211E-06 | -0.570 | - | - |
| 18430 | ENSP00000444777 | REXO1L10P | 4.535E-06 | -0.570 | - | - |
| 18431 | ENSP00000343234 | GAL3ST1 | 5.593E-06 | -0.570 | - | - |
| 18432 | ENSP00000379804 | OSBPL10 | 4.846E-06 | -0.571 | - | - |
| 18433 | ENSP00000359361 | DDX43 | 9.414E-06 | -0.571 | - | - |
| 18434 | ENSP00000324834 | MUC3A | 6.145E-06 | -0.571 | - | - |
| 18435 | ENSP00000358430 | TECTB | 9.806E-06 | -0.571 | - | - |
| 18436 | ENSP00000450353 | RP11-162P23.2 | 8.716E-06 | -0.571 | - | - |
| 18437 | ENSP00000222481 | CPA2 | 1.182E-05 | -0.571 | - | - |
| 18438 | ENSP00000363894 | NIPSNAP3B | 5.179E-06 | -0.571 | - | - |
| 18439 | ENSP00000340369 | RINL | 5.846E-06 | -0.571 | - | - |
| 18440 | ENSP00000359935 | FPGT | 4.314E-06 | -0.571 | - | - |
| 18441 | ENSP00000353129 | KCTD8 | 8.839E-06 | -0.571 | - | - |
| 18442 | ENSP00000254853 | SLC52A1 | 8.123E-06 | -0.572 | - | - |
| 18443 | ENSP00000262946 | UQCR11 | 6.669E-06 | -0.572 | - | - |
| 18444 | ENSP00000327533 | LYG2 | 7.976E-06 | -0.572 | - | - |
| 18445 | ENSP00000353557 | SLC35F1 | 2.795E-06 | -0.573 | - | - |
| 18446 | ENSP00000262915 | ST3GAL3 | 1.176E-05 | -0.573 | - | - |
| 18447 | ENSP00000399753 | TEX13A | 8.101E-06 | -0.573 | - | - |
| 18448 | ENSP00000218104 | ABCD1 | 6.647E-06 | -0.573 | - | - |
| 18449 | ENSP00000344260 | GALNT15 | 7.743E-06 | -0.573 | - | - |
| 18450 | ENSP00000319062 | BBS12 | 9.212E-06 | -0.574 | - | - |
| 18451 | ENSP00000312587 | COQ10A | 9.624E-06 | -0.574 | - | - |
| 18452 | ENSP00000241274 | SLITRK3 | 6.505E-06 | -0.574 | - | - |
| 18453 | ENSP00000357540 | FUOM | 5.987E-06 | -0.574 | - | - |
| 18454 | ENSP00000409382 | EFCAB4B | 3.091E-06 | -0.574 | - | - |
| 18455 | ENSP00000302177 | MBOAT2 | 6.303E-06 | -0.575 | - | - |
| 18456 | ENSP00000273037 | TAMM41 | 5.422E-06 | -0.575 | - | - |
| 18457 | ENSP00000363391 | SPAG4 | 1.110E-05 | -0.575 | - | - |
| 18458 | ENSP00000364550 | KDM5C | 1.337E-05 | -0.575 | - | - |
| 18459 | ENSP00000262305 | RAB11FIP3 | 8.427E-06 | -0.575 | - | - |
| 18460 | ENSP00000313816 | CCDC111 | 3.968E-06 | -0.576 | - | - |
| 18461 | ENSP00000295190 | SLC16A14 | 8.930E-06 | -0.576 | - | - |
| 18462 | ENSP00000318182 | FAM156A | 7.263E-06 | -0.576 | - | - |
| 18463 | ENSP00000365103 | DAOA | 8.226E-06 | -0.577 | - | - |
| 18464 | ENSP00000365395 | AADACL4 | 6.089E-06 | -0.577 | - | - |
| 18465 | ENSP00000410111 | TBC1D26 | 4.467E-06 | -0.577 | - | - |
| 18466 | ENSP00000349336 | FAM163B | 2.590E-06 | -0.578 | - | - |
| 18467 | ENSP00000323455 | TPRX1 | 8.936E-06 | -0.578 | - | - |
| 18468 | ENSP00000321731 | ANKRD24 | 7.855E-06 | -0.578 | - | - |
| 18469 | ENSP00000319799 | H1FOO | 1.011E-05 | -0.578 | - | - |
| 18470 | ENSP00000261275 | FAM189A1 | 7.675E-06 | -0.579 | - | - |
| 18471 | ENSP00000376227 | TMEM44 | 5.009E-06 | -0.579 | - | - |
| 18472 | ENSP00000417748 | DDI2 | 8.210E-06 | -0.579 | - | - |
| 18473 | ENSP00000362125 | PCDH19 | 7.053E-06 | -0.579 | - | - |
| 18474 | ENSP00000254528 | EMILIN2 | 5.759E-06 | -0.579 | - | - |
| 18475 | ENSP00000260723 | BTBD16 | 7.393E-06 | -0.579 | - | - |
| 18476 | ENSP00000303515 | DUS1L | 7.347E-06 | -0.579 | - | - |
| 18477 | ENSP00000307164 | PATE1 | 8.672E-06 | -0.579 | - | - |
| 18478 | ENSP00000259782 | TINAG | 1.244E-05 | -0.579 | - | - |
| 18479 | ENSP00000358951 | OPN1MW | 9.303E-06 | -0.579 | - | - |
| 18480 | ENSP00000391227 | MGAT5B | 3.930E-06 | -0.580 | - | - |
| 18481 | ENSP00000332340 | DDX28 | 5.072E-06 | -0.580 | - | - |
| 18482 | ENSP00000232892 | AADAC | 6.841E-06 | -0.580 | - | - |
| 18483 | ENSP00000345014 | TBC1D3G | 1.017E-05 | -0.580 | - | - |
| 18484 | ENSP00000354961 | MT-ND4 | 8.753E-06 | -0.581 | - | - |
| 18485 | ENSP00000358107 | CA14 | 5.376E-06 | -0.581 | - | - |
| 18486 | ENSP00000454500 | COG8 | 3.293E-06 | -0.581 | - | - |
| 18487 | ENSP00000338349 | NRXN3 | 1.150E-05 | -0.581 | - | - |
| 18488 | ENSP00000265459 | NRXN2 | 1.055E-05 | -0.582 | - | - |
| 18489 | ENSP00000419081 | CALML4 | 1.522E-05 | -0.582 | - | - |
| 18490 | ENSP00000246070 | LAMP5 | 1.407E-05 | -0.582 | - | - |
| 18491 | ENSP00000257632 | UPK3B | 6.012E-06 | -0.582 | - | - |
| 18492 | ENSP00000359581 | HS2ST1 | 9.283E-06 | -0.583 | - | - |
| 18493 | ENSP00000347358 | MAGEA11 | 9.296E-06 | -0.583 | - | - |
| 18494 | ENSP00000350718 | TMCC2 | 6.205E-06 | -0.583 | - | - |
| 18495 | ENSP00000258955 | RSAD1 | 9.550E-06 | -0.583 | - | - |
| 18496 | ENSP00000255039 | HAPLN2 | 7.639E-06 | -0.583 | - | - |
| 18497 | ENSP00000410603 | FAM131B | 3.053E-06 | -0.585 | - | - |
| 18498 | ENSP00000267426 | FITM1 | 6.704E-06 | -0.585 | - | - |
| 18499 | ENSP00000319486 | TSPAN16 | 6.808E-06 | -0.585 | - | - |
| 18500 | ENSP00000290871 | TEPP | 8.983E-06 | -0.585 | - | - |
| 18501 | ENSP00000020926 | SYT13 | 8.596E-06 | -0.585 | - | - |
| 18502 | ENSP00000382646 | FAM166B | 3.187E-06 | -0.586 | - | - |
| 18503 | ENSP00000302260 | C8orf46 | 5.129E-06 | -0.586 | - | - |
| 18504 | ENSP00000348775 | ACOX3 | 6.151E-06 | -0.586 | - | - |
| 18505 | ENSP00000341206 | CHST4 | 8.727E-06 | -0.587 | - | - |
| 18506 | ENSP00000389039 | SYT14 | 3.447E-06 | -0.587 | - | - |
| 18507 | ENSP00000353142 | GAL3ST4 | 3.287E-06 | -0.589 | - | - |
| 18508 | ENSP00000302836 | HOXC9 | 1.029E-05 | -0.590 | - | - |
| 18509 | ENSP00000455047 | SQRDL | 1.057E-05 | -0.590 | - | - |
| 18510 | ENSP00000369146 | MT1M | 9.001E-06 | -0.590 | - | - |
| 18511 | ENSP00000331938 | SLC9B1P1 | 1.210E-05 | -0.590 | - | - |
| 18512 | ENSP00000372319 | CDY2B | 1.539E-05 | -0.590 | - | - |
| 18513 | ENSP00000369135 | SLCO6A1 | 3.923E-06 | -0.591 | - | - |
| 18514 | ENSP00000265498 | MGST2 | 1.434E-05 | -0.592 | - | - |
| 18515 | ENSP00000344040 | OR2S2 | 1.369E-05 | -0.592 | - | - |
| 18516 | ENSP00000469872 | RAB4B-EGLN2 | 7.858E-06 | -0.592 | - | - |
| 18517 | ENSP00000341237 | AC008132.1 | 1.130E-05 | -0.593 | - | - |
| 18518 | ENSP00000281961 | TMEM178A | 1.024E-05 | -0.593 | - | - |
| 18519 | ENSP00000342711 | LY6H | 6.244E-06 | -0.593 | - | - |
| 18520 | ENSP00000417919 | TSPYL6 | 1.456E-05 | -0.593 | - | - |
| 18521 | ENSP00000346990 | SCARA5 | 8.355E-06 | -0.594 | - | - |
| 18522 | ENSP00000287766 | SLC6A1 | 1.014E-05 | -0.595 | - | - |
| 18523 | ENSP00000347152 | ATRNL1 | 6.147E-06 | -0.595 | - | - |
| 18524 | ENSP00000350914 | BDH1 | 3.796E-06 | -0.596 | - | - |
| 18525 | ENSP00000236709 | A4GNT | 1.206E-05 | -0.596 | - | - |
| 18526 | ENSP00000342434 | BAZ1B | 8.032E-06 | -0.596 | - | - |
| 18527 | ENSP00000379377 | GCNT3 | 7.530E-06 | -0.596 | - | - |
| 18528 | ENSP00000349577 | PRODH | 1.286E-05 | -0.596 | - | - |
| 18529 | ENSP00000307093 | MAP6 | 8.678E-06 | -0.596 | - | - |
| 18530 | ENSP00000358559 | SYT6 | 5.900E-06 | -0.596 | - | - |
| 18531 | ENSP00000273390 | MAATS1 | 6.377E-06 | -0.596 | - | - |
| 18532 | ENSP00000358316 | PLEKHS1 | 5.221E-06 | -0.597 | - | - |
| 18533 | ENSP00000222033 | ZNRF4 | 8.548E-06 | -0.597 | - | - |
| 18534 | ENSP00000325562 | MINOS1 | 4.772E-06 | -0.597 | - | - |
| 18535 | ENSP00000262717 | CDH20 | 1.063E-05 | -0.597 | - | - |
| 18536 | ENSP00000237201 | SPACA1 | 1.078E-05 | -0.597 | - | - |
| 18537 | ENSP00000385576 | CEACAM16 | 5.808E-06 | -0.597 | - | - |
| 18538 | ENSP00000269197 | ASXL3 | 1.110E-05 | -0.597 | - | - |
| 18539 | ENSP00000338964 | GGT7 | 9.323E-06 | -0.597 | - | - |
| 18540 | ENSP00000405038 | REXO1L11P | 3.990E-06 | -0.597 | - | - |
| 18541 | ENSP00000320965 | B4GALT3 | 6.150E-06 | -0.598 | - | - |
| 18542 | ENSP00000296142 | RTP3 | 6.405E-06 | -0.598 | - | - |
| 18543 | ENSP00000350162 | SYCP2 | 6.174E-06 | -0.599 | - | - |
| 18544 | ENSP00000361895 | MFSD2A | 5.831E-06 | -0.599 | - | - |
| 18545 | ENSP00000225428 | CWC25 | 1.401E-05 | -0.599 | - | - |
| 18546 | ENSP00000429216 | TRNP1 | 4.049E-06 | -0.599 | - | - |
| 18547 | ENSP00000384593 | GAREML | 4.082E-06 | -0.600 | - | - |
| 18548 | ENSP00000251645 | KRT31 | 7.171E-06 | -0.600 | - | - |
| 18549 | ENSP00000361952 | KCNK15 | 7.538E-06 | -0.600 | - | - |
| 18550 | ENSP00000322218 | PITPNM2 | 7.474E-06 | -0.600 | - | - |
| 18551 | ENSP00000292079 | FXYD2 | 9.281E-06 | -0.600 | - | - |
| 18552 | ENSP00000312774 | SPACA4 | 7.406E-06 | -0.600 | - | - |
| 18553 | ENSP00000359045 | ARHGAP4 | 6.185E-06 | -0.601 | - | - |
| 18554 | ENSP00000361010 | REXO4 | 5.578E-06 | -0.601 | - | - |
| 18555 | ENSP00000303468 | ZCCHC4 | 6.060E-06 | -0.601 | - | - |
| 18556 | ENSP00000368591 | TTLL10 | 8.246E-06 | -0.601 | - | - |
| 18557 | ENSP00000264249 | CHST10 | 7.520E-06 | -0.602 | - | - |
| 18558 | ENSP00000300688 | ATP5L | 7.806E-06 | -0.602 | - | - |
| 18559 | ENSP00000228936 | ART4 | 1.068E-05 | -0.602 | - | - |
| 18560 | ENSP00000344961 | PLEKHG7 | 7.666E-06 | -0.603 | - | - |
| 18561 | ENSP00000384391 | KCTD17 | 9.162E-06 | -0.603 | - | - |
| 18562 | ENSP00000355837 | CAPN8 | 1.264E-05 | -0.604 | - | - |
| 18563 | ENSP00000273936 | CABS1 | 7.768E-06 | -0.604 | - | - |
| 18564 | ENSP00000310585 | PCP2 | 1.037E-05 | -0.604 | - | - |
| 18565 | ENSP00000356404 | B3GALT2 | 4.646E-06 | -0.604 | - | - |
| 18566 | ENSP00000356729 | FMO3 | 9.009E-06 | -0.605 | - | - |
| 18567 | ENSP00000265840 | ELMOD1 | 6.935E-06 | -0.605 | - | - |
| 18568 | ENSP00000280056 | DHRS12 | 6.111E-06 | -0.606 | - | - |
| 18569 | ENSP00000360967 | SLC6A14 | 4.073E-06 | -0.606 | - | - |
| 18570 | ENSP00000360270 | OMA1 | 7.097E-06 | -0.607 | - | - |
| 18571 | ENSP00000265340 | PITX1 | 1.073E-05 | -0.607 | - | - |
| 18572 | ENSP00000331600 | GPR173 | 1.086E-05 | -0.608 | - | - |
| 18573 | ENSP00000303028 | TM4SF20 | 5.498E-06 | -0.608 | - | - |
| 18574 | ENSP00000359579 | HS2ST1 | 9.271E-06 | -0.609 | - | - |
| 18575 | ENSP00000361311 | TMEM53 | 4.185E-06 | -0.609 | - | - |
| 18576 | ENSP00000410732 | GABRG2 | 8.231E-06 | -0.610 | - | - |
| 18577 | ENSP00000301305 | SLC39A4 | 8.235E-06 | -0.610 | - | - |
| 18578 | ENSP00000269945 | DMRTC2 | 8.376E-06 | -0.610 | - | - |
| 18579 | ENSP00000302297 | LGI3 | 5.978E-06 | -0.610 | - | - |
| 18580 | ENSP00000321804 | PAQR4 | 5.463E-06 | -0.611 | - | - |
| 18581 | ENSP00000261058 | CCDC54 | 7.106E-06 | -0.611 | - | - |
| 18582 | ENSP00000270458 | CACNG8 | 1.161E-05 | -0.612 | - | - |
| 18583 | ENSP00000274361 | ANKRD31 | 7.831E-06 | -0.612 | - | - |
| 18584 | ENSP00000312399 | CCDC63 | 9.697E-06 | -0.613 | - | - |
| 18585 | ENSP00000336733 | ST6GALNAC4 | 5.309E-06 | -0.613 | - | - |
| 18586 | ENSP00000358902 | SAMD10 | 2.622E-06 | -0.613 | - | - |
| 18587 | ENSP00000350266 | DENND2D | 9.400E-06 | -0.613 | - | - |
| 18588 | ENSP00000350833 | MPPED2 | 5.000E-06 | -0.613 | - | - |
| 18589 | ENSP00000258613 | THSD1 | 8.211E-06 | -0.613 | - | - |
| 18590 | ENSP00000390011 | CTU1 | 6.070E-06 | -0.614 | - | - |
| 18591 | ENSP00000221730 | EPHX3 | 6.404E-06 | -0.614 | - | - |
| 18592 | ENSP00000351068 | KCNMB2 | 3.300E-06 | -0.615 | - | - |
| 18593 | ENSP00000344937 | DPY19L3 | 7.841E-06 | -0.615 | - | - |
| 18594 | ENSP00000341564 | PAQR9 | 5.892E-06 | -0.615 | - | - |
| 18595 | ENSP00000318974 | FAM133A | 8.542E-06 | -0.615 | - | - |
| 18596 | ENSP00000240361 | TEX14 | 9.873E-06 | -0.616 | - | - |
| 18597 | ENSP00000371434 | RFX3 | 9.020E-06 | -0.616 | - | - |
| 18598 | ENSP00000317214 | CAPN6 | 9.614E-06 | -0.616 | - | - |
| 18599 | ENSP00000055335 | PPP1R3F | 1.058E-05 | -0.617 | - | - |
| 18600 | ENSP00000359683 | PI4K2A | 9.761E-06 | -0.617 | - | - |
| 18601 | ENSP00000274787 | HIGD2A | 6.391E-06 | -0.618 | - | - |
| 18602 | ENSP00000358207 | VAX1 | 6.630E-06 | -0.618 | - | - |
| 18603 | ENSP00000319318 | STAG3 | 6.945E-06 | -0.620 | - | - |
| 18604 | ENSP00000353295 | CBWD3 | 6.636E-06 | -0.620 | - | - |
| 18605 | ENSP00000386272 | SLC38A11 | 2.537E-06 | -0.621 | - | - |
| 18606 | ENSP00000084798 | CA11 | 6.514E-06 | -0.621 | - | - |
| 18607 | ENSP00000355736 | TCTE3 | 6.104E-06 | -0.622 | - | - |
| 18608 | ENSP00000255152 | ZSWIM3 | 9.784E-06 | -0.622 | - | - |
| 18609 | ENSP00000362131 | RPA4 | 7.455E-06 | -0.622 | - | - |
| 18610 | ENSP00000240662 | KCNJ8 | 1.080E-05 | -0.622 | - | - |
| 18611 | ENSP00000280758 | BTBD11 | 9.294E-06 | -0.622 | - | - |
| 18612 | ENSP00000256324 | C14orf159 | 7.736E-06 | -0.623 | - | - |
| 18613 | ENSP00000358957 | TEX28P2 | 4.020E-06 | -0.623 | - | - |
| 18614 | ENSP00000417548 | DNAJC24 | 7.211E-06 | -0.624 | - | - |
| 18615 | ENSP00000317000 | TM6SF1 | 5.742E-06 | -0.624 | - | - |
| 18616 | ENSP00000265104 | DNAH5 | 9.086E-06 | -0.624 | - | - |
| 18617 | ENSP00000006275 | TRAPPC6A | 5.051E-06 | -0.626 | - | - |
| 18618 | ENSP00000287957 | GATAD1 | 6.495E-06 | -0.626 | - | - |
| 18619 | ENSP00000156109 | GPKOW | 4.288E-06 | -0.626 | - | - |
| 18620 | ENSP00000357840 | KIAA1919 | 6.315E-06 | -0.627 | - | - |
| 18621 | ENSP00000368808 | SMPX | 6.519E-06 | -0.627 | - | - |
| 18622 | ENSP00000379353 | ST8SIA1 | 4.544E-06 | -0.628 | - | - |
| 18623 | ENSP00000377043 | TTLL6 | 4.952E-06 | -0.629 | - | - |
| 18624 | ENSP00000291539 | PDE9A | 6.803E-06 | -0.630 | - | - |
| 18625 | ENSP00000323071 | ACOT4 | 5.308E-06 | -0.631 | - | - |
| 18626 | ENSP00000269503 | CBLN2 | 9.251E-06 | -0.631 | - | - |
| 18627 | ENSP00000338627 | SLC6A16 | 3.448E-06 | -0.632 | - | - |
| 18628 | ENSP00000421076 | ATP5L2 | 2.717E-06 | -0.632 | - | - |
| 18629 | ENSP00000226021 | CACNG1 | 1.257E-05 | -0.632 | - | - |
| 18630 | ENSP00000334113 | DHRSX | 5.401E-06 | -0.633 | - | - |
| 18631 | ENSP00000293502 | SDR9C7 | 4.538E-06 | -0.633 | - | - |
| 18632 | ENSP00000364965 | SECISBP2 | 4.335E-06 | -0.634 | - | - |
| 18633 | ENSP00000310490 | SLC16A11 | 8.504E-06 | -0.636 | - | - |
| 18634 | ENSP00000362381 | TOR2A | 6.009E-06 | -0.636 | - | - |
| 18635 | ENSP00000321346 | TTLL11 | 7.534E-06 | -0.636 | - | - |
| 18636 | ENSP00000329467 | OR4M2 | 6.523E-06 | -0.638 | - | - |
| 18637 | ENSP00000413228 | C3orf55 | 3.614E-06 | -0.639 | - | - |
| 18638 | ENSP00000316173 | B3GNT5 | 5.435E-06 | -0.639 | - | - |
| 18639 | ENSP00000266395 | PDE6H | 8.986E-06 | -0.639 | - | - |
| 18640 | ENSP00000261917 | HCN4 | 1.113E-05 | -0.639 | - | - |
| 18641 | ENSP00000260637 | SULT6B1 | 1.226E-05 | -0.640 | - | - |
| 18642 | ENSP00000392252 | TTLL8 | 4.818E-06 | -0.640 | - | - |
| 18643 | ENSP00000333292 | SPNS2 | 6.454E-06 | -0.640 | - | - |
| 18644 | ENSP00000259241 | HS6ST1 | 1.111E-05 | -0.640 | - | - |
| 18645 | ENSP00000268251 | ABAT | 6.772E-06 | -0.642 | - | - |
| 18646 | ENSP00000281182 | ACAD8 | 3.535E-06 | -0.642 | - | - |
| 18647 | ENSP00000352782 | PHOSPHO2 | 3.319E-06 | -0.644 | - | - |
| 18648 | ENSP00000329214 | ST6GALNAC3 | 4.995E-06 | -0.645 | - | - |
| 18649 | ENSP00000360167 | DCAF12L1 | 2.211E-06 | -0.645 | - | - |
| 18650 | ENSP00000420449 | RP11-1220K2.2 | 3.887E-06 | -0.646 | - | - |
| 18651 | ENSP00000293695 | SYCE2 | 5.617E-06 | -0.646 | - | - |
| 18652 | ENSP00000306678 | ANKK1 | 1.102E-05 | -0.646 | - | - |
| 18653 | ENSP00000415398 | - | 8.656E-06 | -0.647 | - | - |
| 18654 | ENSP00000309463 | LIPT2 | 7.158E-06 | -0.647 | - | - |
| 18655 | ENSP00000266980 | SLC39A5 | 5.801E-06 | -0.647 | - | - |
| 18656 | ENSP00000332124 | MRC1L1 | 8.088E-06 | -0.647 | - | - |
| 18657 | ENSP00000372169 | CACNA2D4 | 7.539E-06 | -0.647 | - | - |
| 18658 | ENSP00000299308 | TMEM132B | 8.539E-06 | -0.649 | - | - |
| 18659 | ENSP00000378464 | CPLX3 | 2.826E-06 | -0.649 | - | - |
| 18660 | ENSP00000332818 | SV2B | 8.098E-06 | -0.649 | - | - |
| 18661 | ENSP00000155858 | TRPM5 | 9.587E-06 | -0.649 | - | - |
| 18662 | ENSP00000386167 | OR2A25 | 5.623E-06 | -0.650 | - | - |
| 18663 | ENSP00000466795 | - | 2.262E-06 | -0.650 | - | - |
| 18664 | ENSP00000354963 | RASGEF1C | 6.933E-06 | -0.651 | - | - |
| 18665 | ENSP00000324557 | OR2M7 | 1.207E-05 | -0.651 | - | - |
| 18666 | ENSP00000347721 | SPNS3 | 6.296E-06 | -0.651 | - | - |
| 18667 | ENSP00000323549 | SLC6A18 | 4.014E-06 | -0.651 | - | - |
| 18668 | ENSP00000250838 | CDY2A | 7.859E-06 | -0.652 | - | - |
| 18669 | ENSP00000364831 | CLCNKB | 9.852E-06 | -0.653 | - | - |
| 18670 | ENSP00000261615 | DPEP1 | 9.744E-06 | -0.654 | - | - |
| 18671 | ENSP00000385026 | CHRNA2 | 1.057E-05 | -0.654 | - | - |
| 18672 | ENSP00000264990 | ACAD11 | 9.782E-06 | -0.654 | - | - |
| 18673 | ENSP00000239587 | TTLL2 | 1.100E-05 | -0.655 | - | - |
| 18674 | ENSP00000245957 | C20orf26 | 4.510E-06 | -0.659 | - | - |
| 18675 | ENSP00000450554 | RP11-1012A1.4 | 2.290E-06 | -0.659 | - | - |
| 18676 | ENSP00000281474 | BICD1 | 6.792E-06 | -0.663 | - | - |
| 18677 | ENSP00000386389 | KLHL30 | 3.729E-06 | -0.663 | - | - |
| 18678 | ENSP00000287590 | B3GNT7 | 5.883E-06 | -0.663 | - | - |
| 18679 | ENSP00000160373 | CTTNBP2 | 1.020E-05 | -0.663 | - | - |
| 18680 | ENSP00000345436 | CASKIN1 | 9.226E-06 | -0.664 | - | - |
| 18681 | ENSP00000225609 | PIGL | 9.400E-06 | -0.664 | - | - |
| 18682 | ENSP00000355692 | MRPL55 | 3.402E-06 | -0.664 | - | - |
| 18683 | ENSP00000244333 | LYPD3 | 5.633E-06 | -0.665 | - | - |
| 18684 | ENSP00000419751 | GGTLC2 | 7.206E-06 | -0.665 | - | - |
| 18685 | ENSP00000163416 | GOLGA5 | 6.524E-06 | -0.666 | - | - |
| 18686 | ENSP00000282146 | KCNK13 | 9.368E-06 | -0.666 | - | - |
| 18687 | ENSP00000298912 | CLMN | 1.075E-05 | -0.667 | - | - |
| 18688 | ENSP00000316794 | ZCCHC5 | 4.634E-06 | -0.667 | - | - |
| 18689 | ENSP00000360577 | ECHDC2 | 4.192E-06 | -0.667 | - | - |
| 18690 | ENSP00000220478 | SCG3 | 1.165E-05 | -0.667 | - | - |
| 18691 | ENSP00000350022 | OR8D2 | 1.035E-06 | -0.668 | - | - |
| 18692 | ENSP00000225410 | ZNHIT3 | 8.077E-06 | -0.668 | - | - |
| 18693 | ENSP00000268989 | SGSM2 | 5.851E-06 | -0.668 | - | - |
| 18694 | ENSP00000217043 | R3HDML | 1.025E-05 | -0.670 | - | - |
| 18695 | ENSP00000282670 | TCTEX1D1 | 9.178E-06 | -0.670 | - | - |
| 18696 | ENSP00000209718 | KRT23 | 7.945E-06 | -0.670 | - | - |
| 18697 | ENSP00000353655 | MAN2A2 | 6.366E-06 | -0.672 | - | - |
| 18698 | ENSP00000206423 | CCDC80 | 8.765E-06 | -0.672 | - | - |
| 18699 | ENSP00000252482 | EXOC3L2 | 1.024E-05 | -0.672 | - | - |
| 18700 | ENSP00000303151 | OR1B1 | 8.037E-06 | -0.672 | - | - |
| 18701 | ENSP00000296422 | SLC9B1 | 9.841E-06 | -0.672 | - | - |
| 18702 | ENSP00000199936 | HSD17B2 | 8.509E-06 | -0.673 | - | - |
| 18703 | ENSP00000262622 | CHST8 | 1.025E-05 | -0.674 | - | - |
| 18704 | ENSP00000328327 | ASB7 | 9.139E-06 | -0.674 | - | - |
| 18705 | ENSP00000472299 | AC013449.1 | 2.627E-06 | -0.675 | - | - |
| 18706 | ENSP00000352144 | B4GALT4 | 5.550E-06 | -0.675 | - | - |
| 18707 | ENSP00000035307 | CHPF2 | 7.452E-06 | -0.675 | - | - |
| 18708 | ENSP00000360502 | PDE6C | 1.382E-05 | -0.676 | - | - |
| 18709 | ENSP00000355206 | MT-ND3 | 7.840E-06 | -0.677 | - | - |
| 18710 | ENSP00000319370 | KCNMB3 | 5.485E-06 | -0.678 | - | - |
| 18711 | ENSP00000333751 | PLCXD3 | 7.730E-06 | -0.679 | - | - |
| 18712 | ENSP00000243103 | HOXC12 | 6.056E-06 | -0.679 | - | - |
| 18713 | ENSP00000396586 | SLC22A24 | 3.270E-06 | -0.680 | - | - |
| 18714 | ENSP00000218516 | GLA | 8.596E-06 | -0.681 | - | - |
| 18715 | ENSP00000258324 | PIGC | 1.098E-05 | -0.681 | - | - |
| 18716 | ENSP00000310696 | B4GALT2 | 5.348E-06 | -0.682 | - | - |
| 18717 | ENSP00000320431 | ST8SIA3 | 4.986E-06 | -0.682 | - | - |
| 18718 | ENSP00000326259 | OR52H1 | 1.185E-05 | -0.682 | - | - |
| 18719 | ENSP00000318445 | ST3GAL1 | 5.650E-06 | -0.684 | - | - |
| 18720 | ENSP00000357915 | SLC22A16 | 3.789E-06 | -0.684 | - | - |
| 18721 | ENSP00000222125 | CAPS | 8.603E-06 | -0.684 | - | - |
| 18722 | ENSP00000441093 | FDX1L | 3.978E-06 | -0.685 | - | - |
| 18723 | ENSP00000265440 | TFEC | 8.718E-06 | -0.687 | - | - |
| 18724 | ENSP00000316955 | OR7A5 | 3.337E-06 | -0.688 | - | - |
| 18725 | ENSP00000328983 | CHST6 | 5.022E-06 | -0.689 | - | - |
| 18726 | ENSP00000466514 | CTB-54O9.9 | 3.508E-06 | -0.690 | - | - |
| 18727 | ENSP00000377717 | ST3GAL6 | 4.252E-06 | -0.691 | - | - |
| 18728 | ENSP00000254037 | ZCCHC9 | 5.142E-06 | -0.691 | - | - |
| 18729 | ENSP00000260953 | METTL5 | 7.201E-06 | -0.692 | - | - |
| 18730 | ENSP00000301175 | PRODH2 | 7.736E-06 | -0.692 | - | - |
| 18731 | ENSP00000232744 | ABTB1 | 9.281E-06 | -0.692 | - | - |
| 18732 | ENSP00000234827 | TCEANC2 | 4.830E-06 | -0.692 | - | - |
| 18733 | ENSP00000293860 | POLR3K | 6.134E-06 | -0.693 | - | - |
| 18734 | ENSP00000329990 | BX072566.1 | 1.015E-05 | -0.693 | - | - |
| 18735 | ENSP00000317357 | OR13D1 | 1.208E-05 | -0.694 | - | - |
| 18736 | ENSP00000304235 | OR1L6 | 2.104E-06 | -0.695 | - | - |
| 18737 | ENSP00000297325 | SUN3 | 5.136E-06 | -0.697 | - | - |
| 18738 | ENSP00000352717 | OR13G1 | 5.991E-06 | -0.698 | - | - |
| 18739 | ENSP00000315835 | GALNT11 | 6.418E-06 | -0.699 | - | - |
| 18740 | ENSP00000291839 | ST6GALNAC6 | 8.417E-06 | -0.699 | - | - |
| 18741 | ENSP00000359629 | CRTAC1 | 5.480E-06 | -0.701 | - | - |
| 18742 | ENSP00000308334 | ATP5S | 6.856E-06 | -0.705 | - | - |
| 18743 | ENSP00000339973 | TBC1D28 | 6.846E-06 | -0.705 | - | - |
| 18744 | ENSP00000243913 | GCNT7 | 7.660E-06 | -0.706 | - | - |
| 18745 | ENSP00000381036 | ADGB | 9.174E-06 | -0.707 | - | - |
| 18746 | ENSP00000383212 | SGSM1 | 4.247E-06 | -0.707 | - | - |
| 18747 | ENSP00000450281 | GATC | 2.792E-06 | -0.710 | - | - |
| 18748 | ENSP00000470059 | ECH1 | 4.026E-06 | -0.711 | - | - |
| 18749 | ENSP00000366549 | ST3GAL5 | 2.952E-06 | -0.713 | - | - |
| 18750 | ENSP00000230053 | B3GAT2 | 8.386E-06 | -0.716 | - | - |
| 18751 | ENSP00000250699 | CHRNA10 | 9.574E-06 | -0.717 | - | - |
| 18752 | ENSP00000299333 | SCN3B | 7.058E-06 | -0.717 | - | - |
| 18753 | ENSP00000305613 | CPLX1 | 7.289E-06 | -0.718 | - | - |
| 18754 | ENSP00000381758 | CAPN11 | 9.853E-06 | -0.719 | - | - |
| 18755 | ENSP00000301050 | CACNB3 | 9.389E-06 | -0.719 | - | - |
| 18756 | ENSP00000251108 | CDADC1 | 6.244E-06 | -0.719 | - | - |
| 18757 | ENSP00000385632 | PDE7A | 9.127E-06 | -0.719 | - | - |
| 18758 | ENSP00000334181 | KDM4D | 6.835E-06 | -0.720 | - | - |
| 18759 | ENSP00000362354 | LPIN3 | 7.113E-06 | -0.720 | - | - |
| 18760 | ENSP00000352544 | CPLX2 | 4.577E-06 | -0.722 | - | - |
| 18761 | ENSP00000288988 | GALNT14 | 8.965E-06 | -0.723 | - | - |
| 18762 | ENSP00000272252 | GALM | 5.934E-06 | -0.725 | - | - |
| 18763 | ENSP00000267731 | GOLGA8B | 6.941E-06 | -0.725 | - | - |
| 18764 | ENSP00000345477 | ST3GAL2 | 6.689E-06 | -0.726 | - | - |
| 18765 | ENSP00000242592 | ACADS | 6.594E-06 | -0.727 | - | - |
| 18766 | ENSP00000323302 | POC1B | 7.597E-06 | -0.727 | - | - |
| 18767 | ENSP00000283871 | HGD | 7.093E-06 | -0.728 | - | - |
| 18768 | ENSP00000330389 | PTRHD1 | 3.689E-06 | -0.728 | - | - |
| 18769 | ENSP00000287490 | COX6A2 | 5.610E-06 | -0.730 | - | - |
| 18770 | ENSP00000319654 | OR4M1 | 9.434E-06 | -0.730 | - | - |
| 18771 | ENSP00000252602 | MRPL34 | 4.697E-06 | -0.733 | - | - |
| 18772 | ENSP00000273598 | NICN1 | 5.095E-06 | -0.733 | - | - |
| 18773 | ENSP00000327611 | KCNK12 | 7.871E-06 | -0.735 | - | - |
| 18774 | ENSP00000355429 | OR2T5 | 1.067E-05 | -0.738 | - | - |
| 18775 | ENSP00000387523 | OR7E24 | 3.344E-06 | -0.739 | - | - |
| 18776 | ENSP00000450694 | - | 4.886E-06 | -0.742 | - | - |
| 18777 | ENSP00000369409 | EXOC3L4 | 5.053E-06 | -0.743 | - | - |
| 18778 | ENSP00000254101 | PRKAB2 | 9.627E-06 | -0.743 | - | - |
| 18779 | ENSP00000302801 | TRMT61B | 4.983E-06 | -0.744 | - | - |
| 18780 | ENSP00000266086 | SLC5A4 | 9.237E-06 | -0.746 | - | - |
| 18781 | ENSP00000421315 | AQP1 | 7.168E-06 | -0.746 | - | - |
| 18782 | ENSP00000312329 | STAG3L3 | 6.976E-06 | -0.748 | - | - |
| 18783 | ENSP00000327569 | SLC22A10 | 6.310E-06 | -0.749 | - | - |
| 18784 | ENSP00000263208 | HIRA | 9.306E-06 | -0.751 | - | - |
| 18785 | ENSP00000261483 | MAN2A1 | 6.138E-06 | -0.753 | - | - |
| 18786 | ENSP00000303252 | ZNF804A | 7.949E-06 | -0.754 | - | - |
| 18787 | ENSP00000271971 | CAPN9 | 8.729E-06 | -0.755 | - | - |
| 18788 | ENSP00000299989 | - | 7.084E-06 | -0.756 | - | - |
| 18789 | ENSP00000355435 | OR2L2 | 2.505E-06 | -0.757 | - | - |
| 18790 | ENSP00000346401 | KIF25 | 8.156E-06 | -0.758 | - | - |
| 18791 | ENSP00000253122 | SLC6A8 | 6.126E-06 | -0.762 | - | - |
| 18792 | ENSP00000297994 | TRMT10B | 5.086E-06 | -0.763 | - | - |
| 18793 | ENSP00000377311 | FDX1L | 4.204E-06 | -0.766 | - | - |
| 18794 | ENSP00000348772 | PITPNM1 | 7.609E-06 | -0.767 | - | - |
| 18795 | ENSP00000314103 | ANKRD34A | 3.987E-06 | -0.769 | - | - |
| 18796 | ENSP00000264020 | IFT46 | 4.498E-06 | -0.771 | - | - |
| 18797 | ENSP00000365895 | HS6ST3 | 6.844E-06 | -0.772 | - | - |
| 18798 | ENSP00000280350 | PIH1D2 | 6.835E-06 | -0.775 | - | - |
| 18799 | ENSP00000236495 | SLC5A9 | 7.760E-06 | -0.777 | - | - |
| 18800 | ENSP00000278559 | CAPN5 | 8.586E-06 | -0.778 | - | - |
| 18801 | ENSP00000306095 | OR10H2 | 2.524E-06 | -0.779 | - | - |
| 18802 | ENSP00000329256 | TBC1D3F | 7.343E-06 | -0.779 | - | - |
| 18803 | ENSP00000319636 | B3GNT4 | 4.374E-06 | -0.785 | - | - |
| 18804 | ENSP00000320247 | AQP6 | 5.389E-06 | -0.786 | - | - |
| 18805 | ENSP00000332068 | OR5P3 | 1.071E-06 | -0.787 | - | - |
| 18806 | ENSP00000415635 | GGTLC3 | 4.430E-06 | -0.790 | - | - |
| 18807 | ENSP00000350836 | OR2L13 | 1.550E-06 | -0.792 | - | - |
| 18808 | ENSP00000334115 | OR10T2 | 8.300E-06 | -0.793 | - | - |
| 18809 | ENSP00000386959 | CAPS2 | 9.540E-06 | -0.794 | - | - |
| 18810 | ENSP00000431914 | OR10S1 | 3.520E-06 | -0.795 | - | - |
| 18811 | ENSP00000401966 | OR1M1 | 5.052E-07 | -0.796 | - | - |
| 18812 | ENSP00000331636 | CAPN12 | 6.638E-06 | -0.800 | - | - |
| 18813 | ENSP00000376633 | OR2F1 | 1.769E-06 | -0.801 | - | - |
| 18814 | ENSP00000401514 | DNAH1 | 4.753E-06 | -0.802 | - | - |
| 18815 | ENSP00000294725 | KCNT2 | 5.207E-06 | -0.804 | - | - |
| 18816 | ENSP00000321874 | B3GNT3 | 4.980E-06 | -0.804 | - | - |
| 18817 | ENSP00000341479 | KCNJ14 | 5.285E-06 | -0.808 | - | - |
| 18818 | ENSP00000261383 | DNAH3 | 7.496E-06 | -0.810 | - | - |
| 18819 | ENSP00000296097 | DNAJC5G | 7.962E-06 | -0.810 | - | - |
| 18820 | ENSP00000465737 | RP11-729L2.2 | 3.779E-06 | -0.810 | - | - |
| 18821 | ENSP00000456953 | POTEB | 5.249E-06 | -0.818 | - | - |
| 18822 | ENSP00000324769 | OR4C6 | 3.642E-06 | -0.818 | - | - |
| 18823 | ENSP00000225276 | ST6GALNAC2 | 6.059E-06 | -0.819 | - | - |
| 18824 | ENSP00000328277 | B4GALNT4 | 4.804E-06 | -0.820 | - | - |
| 18825 | ENSP00000188312 | ACTR6 | 5.306E-06 | -0.823 | - | - |
| 18826 | ENSP00000380153 | OR51E2 | 2.606E-06 | -0.823 | - | - |
| 18827 | ENSP00000344101 | OR6N2 | 2.217E-06 | -0.827 | - | - |
| 18828 | ENSP00000298642 | OR4K2 | 6.913E-07 | -0.829 | - | - |
| 18829 | ENSP00000319511 | OR4K5 | 5.886E-07 | -0.830 | - | - |
| 18830 | ENSP00000307443 | SLC22A25 | 3.935E-06 | -0.832 | - | - |
| 18831 | ENSP00000322435 | OR6B3 | 4.919E-06 | -0.837 | - | - |
| 18832 | ENSP00000216027 | HSCB | 5.685E-06 | -0.841 | - | - |
| 18833 | ENSP00000386502 | ANKRD61 | 8.986E-06 | -0.842 | - | - |
| 18834 | ENSP00000232766 | KLHL18 | 6.399E-06 | -0.846 | - | - |
| 18835 | ENSP00000326948 | FTSJ1 | 4.434E-06 | -0.848 | - | - |
| 18836 | ENSP00000367219 | OR13J1 | 1.886E-06 | -0.850 | - | - |
| 18837 | ENSP00000396747 | ANKRD60 | 8.988E-06 | -0.856 | - | - |
| 18838 | ENSP00000276570 | DNAJC5B | 6.757E-06 | -0.857 | - | - |
| 18839 | ENSP00000279178 | SLC22A9 | 4.380E-06 | -0.861 | - | - |
| 18840 | ENSP00000258111 | KCNMB4 | 6.654E-06 | -0.863 | - | - |
| 18841 | ENSP00000263080 | ASPA | 5.557E-06 | -0.864 | - | - |
| 18842 | ENSP00000266383 | B4GALNT3 | 5.750E-06 | -0.869 | - | - |
| 18843 | ENSP00000428619 | UTP14C | 4.116E-06 | -0.871 | - | - |
| 18844 | ENSP00000254616 | TIMM10B | 3.401E-06 | -0.876 | - | - |
| 18845 | ENSP00000261377 | AC004381.6 | 5.340E-06 | -0.877 | - | - |
| 18846 | ENSP00000445688 | ANKRD65 | 9.074E-06 | -0.883 | - | - |
| 18847 | ENSP00000471417 | ANKDD1B | 9.141E-06 | -0.894 | - | - |
| 18848 | ENSP00000333684 | OR52A1 | 9.276E-06 | -0.897 | - | - |
| 18849 | ENSP00000327585 | OR1D2 | 3.378E-06 | -0.899 | - | - |
| 18850 | ENSP00000312554 | DNAH12 | 6.418E-06 | -0.902 | - | - |
| 18851 | ENSP00000365451 | OR4D1 | 3.079E-06 | -0.904 | - | - |
| 18852 | ENSP00000321419 | OR4C3 | 1.585E-06 | -0.911 | - | - |
| 18853 | ENSP00000324251 | OR10K2 | 8.807E-06 | -0.915 | - | - |
| 18854 | ENSP00000278765 | GGTLC1 | 7.619E-06 | -0.916 | - | - |
| 18855 | ENSP00000380155 | OR51E1 | 3.574E-06 | -0.917 | - | - |
| 18856 | ENSP00000373193 | OR5K1 | 5.193E-06 | -0.918 | - | - |
| 18857 | ENSP00000333310 | SULT1C3 | 5.391E-06 | -0.933 | - | - |
| 18858 | ENSP00000303096 | OR5A1 | 5.447E-07 | -0.943 | - | - |
| 18859 | ENSP00000313803 | OR3A1 | 1.270E-06 | -0.943 | - | - |
| 18860 | ENSP00000300778 | OR51Q1 | 1.784E-06 | -0.947 | - | - |
| 18861 | ENSP00000386138 | OR1C1 | 8.684E-06 | -0.951 | - | - |
| 18862 | ENSP00000444054 | OR52E8 | 3.296E-06 | -0.952 | - | - |
| 18863 | ENSP00000328215 | OR56A4 | 1.043E-06 | -0.955 | - | - |
| 18864 | ENSP00000331823 | OR5P2 | 6.426E-06 | -0.967 | - | - |
| 18865 | ENSP00000353988 | OR10A3 | 8.099E-06 | -0.976 | - | - |
| 18866 | ENSP00000257549 | SDS | 4.893E-06 | -0.976 | - | - |
| 18867 | ENSP00000300127 | OR4D6 | 3.234E-06 | -0.983 | - | - |
| 18868 | ENSP00000435416 | OR5M1 | 1.051E-06 | -0.984 | - | - |
| 18869 | ENSP00000371377 | OR1A2 | 4.632E-06 | -1.000 | - | - |
| 18870 | ENSP00000334456 | OR5D14 | 4.347E-06 | -1.003 | - | - |
| 18871 | ENSP00000322784 | OR5AK2 | 4.987E-06 | -1.011 | - | - |
| 18872 | ENSP00000368831 | SNX2 | 9.330E-05 | -1.021 | - | - |
| 18873 | ENSP00000445383 | OR10G2 | 5.946E-07 | -1.038 | - | - |
| 18874 | ENSP00000308764 | OR52I2 | 1.084E-06 | -1.041 | - | - |
| 18875 | ENSP00000331545 | OR1G1 | 3.006E-06 | -1.044 | - | - |
| 18876 | ENSP00000386195 | OR4E2 | 6.127E-07 | -1.048 | - | - |
| 18877 | ENSP00000304188 | OR8U1 | 2.419E-06 | -1.049 | - | - |
| 18878 | ENSP00000332500 | OR4N4 | 7.113E-06 | -1.049 | - | - |
| 18879 | ENSP00000363911 | OR13C5 | 6.724E-06 | -1.054 | - | - |
| 18880 | ENSP00000302437 | OR10G3 | 6.916E-07 | -1.060 | - | - |
| 18881 | ENSP00000350474 | OR8D1 | 3.579E-06 | -1.063 | - | - |
| 18882 | ENSP00000436004 | OR5M10 | 5.708E-07 | -1.065 | - | - |
| 18883 | ENSP00000328402 | OR6C76 | 1.495E-06 | -1.107 | - | - |
| 18884 | ENSP00000259357 | OR1J1 | 4.980E-06 | -1.124 | - | - |
| 18885 | ENSP00000317482 | OR4F4 | 4.248E-06 | -1.125 | - | - |
| 18886 | ENSP00000386160 | OR52B4 | 1.490E-06 | -1.145 | - | - |
| 18887 | ENSP00000313936 | OR2AE1 | 1.281E-06 | -1.147 | - | - |
| 18888 | ENSP00000330049 | OR4Q3 | 4.961E-06 | -1.149 | - | - |
| 18889 | ENSP00000302863 | OR1L3 | 1.015E-06 | -1.149 | - | - |
| 18890 | ENSP00000333184 | OR4F15 | 4.874E-06 | -1.165 | - | - |
| 18891 | ENSP00000325682 | OR2B11 | 3.607E-06 | -1.166 | - | - |
| 18892 | ENSP00000307751 | OR4X2 | 4.889E-06 | -1.168 | - | - |
| 18893 | ENSP00000393889 | OR5K2 | 3.657E-06 | -1.170 | - | - |
| 18894 | ENSP00000345563 | OR7D2 | 5.035E-06 | -1.180 | - | - |
| 18895 | ENSP00000310773 | OR1L1 | 1.327E-06 | -1.182 | - | - |
| 18896 | ENSP00000310337 | OR4S2 | 3.668E-06 | -1.195 | - | - |
| 18897 | ENSP00000334393 | OR4F5 | 4.005E-06 | -1.204 | - | - |
| 18898 | ENSP00000373196 | OR5H6 | 1.237E-06 | -1.207 | - | - |
| 18899 | ENSP00000305207 | OR1A1 | 2.506E-06 | -1.215 | - | - |
| 18900 | ENSP00000432011 | OR52B2 | 4.577E-07 | -1.216 | - | - |
| 18901 | ENSP00000386209 | OR2A2 | 8.949E-07 | -1.222 | - | - |
| 18902 | ENSP00000341581 | OR52B6 | 5.503E-07 | -1.227 | - | - |
| 18903 | ENSP00000326232 | OR52D1 | 6.871E-07 | -1.227 | - | - |
| 18904 | ENSP00000367649 | OR5D16 | 4.857E-07 | -1.229 | - | - |
| 18905 | ENSP00000441354 | OR4D2 | 1.331E-06 | -1.230 | - | - |
| 18906 | ENSP00000305640 | OR6F1 | 1.739E-06 | -1.237 | - | - |
| 18907 | ENSP00000340748 | OR13H1 | 1.509E-06 | -1.239 | - | - |
| 18908 | ENSP00000334441 | OR10J5 | 5.625E-07 | -1.249 | - | - |
| 18909 | ENSP00000322546 | OR51G1 | 3.916E-06 | -1.250 | - | - |
| 18910 | ENSP00000355441 | OR14A2 | 4.046E-07 | -1.251 | - | - |
| 18911 | ENSP00000244623 | OR2B6 | 2.159E-06 | -1.254 | - | - |
| 18912 | ENSP00000350248 | OR14A16 | 4.570E-07 | -1.254 | - | - |
| 18913 | ENSP00000412752 | OR4A47 | 1.433E-06 | -1.255 | - | - |
| 18914 | ENSP00000352710 | OR2M2 | 4.028E-06 | -1.258 | - | - |
| 18915 | ENSP00000318878 | OR4F21 | 4.036E-06 | -1.259 | - | - |
| 18916 | ENSP00000355432 | OR2M5 | 4.027E-06 | -1.259 | - | - |
| 18917 | ENSP00000355430 | OR2T1 | 3.076E-06 | -1.265 | - | - |
| 18918 | ENSP00000326349 | OR2G2 | 1.999E-06 | -1.266 | - | - |
| 18919 | ENSP00000331789 | OR10J3 | 3.519E-06 | -1.266 | - | - |
| 18920 | ENSP00000313110 | OR6S1 | 3.475E-06 | -1.273 | - | - |
| 18921 | ENSP00000341291 | OR2G6 | 1.029E-06 | -1.278 | - | - |
| 18922 | ENSP00000252835 | OR11H1 | 4.459E-06 | -1.288 | - | - |
| 18923 | ENSP00000303111 | OR5AP2 | 1.161E-06 | -1.289 | - | - |
| 18924 | ENSP00000302639 | OR5AR1 | 1.161E-06 | -1.291 | - | - |
| 18925 | ENSP00000349719 | OR2L8 | 1.160E-06 | -1.294 | - | - |
| 18926 | ENSP00000369731 | OR51A4 | 2.921E-06 | -1.295 | - | - |
| 18927 | ENSP00000324913 | OR4C16 | 1.039E-06 | -1.307 | - | - |
| 18928 | ENSP00000347418 | OR5H2 | 1.113E-06 | -1.307 | - | - |
| 18929 | ENSP00000310632 | OR8S1 | 8.690E-07 | -1.308 | - | - |
| 18930 | ENSP00000301529 | OR8J3 | 3.318E-06 | -1.317 | - | - |
| 18931 | ENSP00000355443 | OR2C3 | 1.673E-06 | -1.320 | - | - |
| 18932 | ENSP00000357134 | OR10R2 | 2.011E-06 | -1.325 | - | - |
| 18933 | ENSP00000367664 | OR4A5 | 1.451E-06 | -1.335 | - | - |
| 18934 | ENSP00000368987 | OR6C75 | 2.078E-06 | -1.345 | - | - |
| 18935 | ENSP00000325381 | OR8D4 | 2.141E-06 | -1.346 | - | - |
| 18936 | ENSP00000315047 | OR4F17 | 1.131E-06 | -1.348 | - | - |
| 18937 | ENSP00000328144 | OR7A17 | 1.020E-06 | -1.348 | - | - |
| 18938 | ENSP00000312403 | OR2Y1 | 3.198E-06 | -1.351 | - | - |
| 18939 | ENSP00000308595 | OR5R1 | 1.799E-06 | -1.354 | - | - |
| 18940 | ENSP00000367650 | OR5L2 | 8.360E-07 | -1.361 | - | - |
| 18941 | ENSP00000342697 | OR2AG2 | 5.799E-07 | -1.367 | - | - |
| 18942 | ENSP00000386174 | OR2A12 | 9.606E-07 | -1.384 | - | - |
| 18943 | ENSP00000310488 | OR7D4 | 2.959E-06 | -1.385 | - | - |
| 18944 | ENSP00000343521 | OR1J4 | 1.902E-06 | -1.390 | - | - |
| 18945 | ENSP00000322866 | OR52N5 | 2.109E-06 | -1.392 | - | - |
| 18946 | ENSP00000321196 | OR56B4 | 2.237E-06 | -1.399 | - | - |
| 18947 | ENSP00000335596 | OR10H1 | 7.634E-07 | -1.400 | - | - |
| 18948 | ENSP00000330338 | OR52L1 | 1.884E-06 | -1.404 | - | - |
| 18949 | ENSP00000386151 | OR6B1 | 7.928E-07 | -1.411 | - | - |
| 18950 | ENSP00000333196 | OR51M1 | 4.969E-07 | -1.413 | - | - |
| 18951 | ENSP00000327540 | OR51B2 | 9.961E-07 | -1.416 | - | - |
| 18952 | ENSP00000342448 | OR5W2 | 4.735E-07 | -1.420 | - | - |
| 18953 | ENSP00000319546 | OR2A4 | 1.740E-06 | -1.421 | - | - |
| 18954 | ENSP00000310704 | OR10H5 | 6.036E-07 | -1.424 | - | - |
| 18955 | ENSP00000321729 | OR51V1 | 6.715E-07 | -1.428 | - | - |
| 18956 | ENSP00000314324 | OR10Q1 | 5.726E-07 | -1.437 | - | - |
| 18957 | ENSP00000324583 | OR2T12 | 1.449E-06 | -1.439 | - | - |
| 18958 | ENSP00000325065 | OR4A15 | 8.490E-07 | -1.442 | - | - |
| 18959 | ENSP00000386222 | OR2F2 | 2.624E-06 | -1.442 | - | - |
| 18960 | ENSP00000320302 | OR5AN1 | 4.466E-07 | -1.442 | - | - |
| 18961 | ENSP00000324534 | OR14C36 | 1.553E-06 | -1.445 | - | - |
| 18962 | ENSP00000355431 | OR2T4 | 9.395E-07 | -1.446 | - | - |
| 18963 | ENSP00000303834 | OR5A2 | 5.782E-07 | -1.448 | - | - |
| 18964 | ENSP00000284287 | OR8A1 | 7.697E-07 | -1.448 | - | - |
| 18965 | ENSP00000352604 | OR2T3 | 1.269E-06 | -1.449 | - | - |
| 18966 | ENSP00000347965 | OR2T6 | 8.996E-07 | -1.452 | - | - |
| 18967 | ENSP00000334452 | OR13F1 | 1.257E-06 | -1.455 | - | - |
| 18968 | ENSP00000328934 | OR2T11 | 4.216E-07 | -1.455 | - | - |
| 18969 | ENSP00000329056 | OR4C46 | 1.193E-06 | -1.456 | - | - |
| 18970 | ENSP00000304077 | OR4K15 | 4.791E-07 | -1.457 | - | - |
| 18971 | ENSP00000369728 | OR52J3 | 1.243E-06 | -1.458 | - | - |
| 18972 | ENSP00000300773 | OR51B5 | 6.866E-07 | -1.461 | - | - |
| 18973 | ENSP00000323354 | OR5M8 | 4.594E-07 | -1.463 | - | - |
| 18974 | ENSP00000322724 | OR51H1P | 4.089E-07 | -1.466 | - | - |
| 18975 | ENSP00000348033 | OR11L1 | 5.218E-07 | -1.468 | - | - |
| 18976 | ENSP00000278409 | OR5F1 | 1.042E-06 | -1.469 | - | - |
| 18977 | ENSP00000323224 | OR52N4 | 1.061E-06 | -1.469 | - | - |
| 18978 | ENSP00000305469 | OR1S2 | 9.415E-07 | -1.470 | - | - |
| 18979 | ENSP00000353516 | OR2W3 | 1.118E-06 | -1.470 | - | - |
| 18980 | ENSP00000323982 | OR8H2 | 4.745E-07 | -1.470 | - | - |
| 18981 | ENSP00000248058 | OR7A10 | 1.033E-06 | -1.471 | - | - |
| 18982 | ENSP00000368989 | OR6C3 | 5.403E-07 | -1.472 | - | - |
| 18983 | ENSP00000307159 | OR8B12 | 4.497E-07 | -1.472 | - | - |
| 18984 | ENSP00000444134 | OR7G1 | 7.138E-07 | -1.476 | - | - |
| 18985 | ENSP00000306657 | OR5B12 | 4.923E-07 | -1.477 | - | - |
| 18986 | ENSP00000329153 | OR6C70 | 6.430E-07 | -1.478 | - | - |
| 18987 | ENSP00000291231 | OR3A3 | 6.378E-07 | -1.478 | - | - |
| 18988 | ENSP00000248384 | OR1E2 | 1.253E-06 | -1.478 | - | - |
| 18989 | ENSP00000330904 | OR2T34 | 4.175E-07 | -1.478 | - | - |
| 18990 | ENSP00000327525 | OR4F6 | 4.195E-07 | -1.478 | - | - |
| 18991 | ENSP00000307598 | OR9K2 | 6.833E-07 | -1.479 | - | - |
| 18992 | ENSP00000322823 | OR52N1 | 4.220E-07 | -1.479 | - | - |
| 18993 | ENSP00000438815 | OR13C2 | 3.882E-07 | -1.480 | - | - |
| 18994 | ENSP00000307130 | OR10H3 | 9.970E-07 | -1.480 | - | - |
| 18995 | ENSP00000326225 | OR2T8 | 4.204E-07 | -1.481 | - | - |
| 18996 | ENSP00000259466 | OR1L4 | 5.064E-07 | -1.481 | - | - |
| 18997 | ENSP00000334418 | OR4C12 | 6.645E-07 | -1.481 | - | - |
| 18998 | ENSP00000321338 | OR4C5 | 6.590E-07 | -1.481 | - | - |
| 18999 | ENSP00000307447 | OR2AG1 | 6.045E-07 | -1.482 | - | - |
| 19000 | ENSP00000325128 | OR4A16 | 1.171E-06 | -1.482 | - | - |
| 19001 | ENSP00000308270 | OR5B3 | 8.215E-07 | -1.482 | - | - |
| 19002 | ENSP00000378516 | OR10W1 | 4.851E-07 | -1.482 | - | - |
| 19003 | ENSP00000323928 | OR8H3 | 4.218E-07 | -1.483 | - | - |
| 19004 | ENSP00000353537 | OR5B21 | 4.833E-07 | -1.483 | - | - |
| 19005 | ENSP00000321447 | OR4S1 | 4.227E-07 | -1.483 | - | - |
| 19006 | ENSP00000322939 | OR56B1 | 4.219E-07 | -1.483 | - | - |
| 19007 | ENSP00000322593 | OR51G2 | 4.226E-07 | -1.483 | - | - |
| 19008 | ENSP00000401706 | OR5H14 | 3.354E-07 | -1.484 | - | - |
| 19009 | ENSP00000311688 | OR1S1 | 6.398E-07 | -1.484 | - | - |
| 19010 | ENSP00000437629 | OR6J1 | 5.022E-07 | -1.484 | - | - |
| 19011 | ENSP00000368986 | OR6C65 | 3.972E-07 | -1.484 | - | - |
| 19012 | ENSP00000321506 | OR4X1 | 5.963E-07 | -1.484 | - | - |
| 19013 | ENSP00000302422 | OR52K1 | 4.344E-07 | -1.484 | - | - |
| 19014 | ENSP00000322156 | OR51L1 | 4.036E-07 | -1.485 | - | - |
| 19015 | ENSP00000324369 | OR2T35 | 3.852E-07 | -1.485 | - | - |
| 19016 | ENSP00000331774 | OR2T29 | 5.767E-07 | -1.485 | - | - |
| 19017 | ENSP00000386208 | OR2A5 | 3.910E-07 | -1.486 | - | - |
| 19018 | ENSP00000312208 | OR5M3 | 4.314E-07 | -1.486 | - | - |
| 19019 | ENSP00000323423 | OR2AP1 | 4.219E-07 | -1.486 | - | - |
| 19020 | ENSP00000329210 | OR2T10 | 7.503E-07 | -1.486 | - | - |
| 19021 | ENSP00000386137 | OR2A14 | 3.918E-07 | -1.486 | - | - |
| 19022 | ENSP00000248072 | OR7C2 | 1.088E-06 | -1.486 | - | - |
| 19023 | ENSP00000332473 | OR51J1 | 3.651E-07 | -1.486 | - | - |
| 19024 | ENSP00000248073 | OR7C1 | 1.031E-06 | -1.486 | - | - |
| 19025 | ENSP00000319071 | OR11H6 | 4.320E-07 | -1.486 | - | - |
| 19026 | ENSP00000330280 | OR8B8 | 5.636E-07 | -1.486 | - | - |
| 19027 | ENSP00000409094 | OR52I1 | 4.086E-07 | -1.486 | - | - |
| 19028 | ENSP00000334934 | OR9Q1 | 4.134E-07 | -1.486 | - | - |
| 19029 | ENSP00000362792 | OR1N2 | 4.080E-07 | -1.487 | - | - |
| 19030 | ENSP00000319197 | OR4K17 | 4.243E-07 | -1.487 | - | - |
| 19031 | ENSP00000369738 | OR51T1 | 4.015E-07 | -1.487 | - | - |
| 19032 | ENSP00000319217 | OR4L1 | 4.243E-07 | -1.487 | - | - |
| 19033 | ENSP00000318834 | OR10H4 | 4.244E-07 | -1.487 | - | - |
| 19034 | ENSP00000308714 | OR9Q2 | 4.318E-07 | -1.487 | - | - |
| 19035 | ENSP00000386180 | OR3A2 | 3.934E-07 | -1.487 | - | - |
| 19036 | ENSP00000369559 | OR51I1 | 3.971E-07 | -1.487 | - | - |
| 19037 | ENSP00000335529 | OR5L1 | 4.134E-07 | -1.487 | - | - |
| 19038 | ENSP00000312470 | OR10A6 | 4.454E-07 | -1.488 | - | - |
| 19039 | ENSP00000307734 | OR6Q1 | 4.318E-07 | -1.488 | - | - |
| 19040 | ENSP00000302199 | OR10V1 | 5.151E-07 | -1.488 | - | - |
| 19041 | ENSP00000303864 | OR8I2 | 4.341E-07 | -1.488 | - | - |
| 19042 | ENSP00000306974 | OR1N1 | 4.346E-07 | -1.488 | - | - |
| 19043 | ENSP00000209540 | OR1I1 | 5.085E-07 | -1.488 | - | - |
| 19044 | ENSP00000362784 | OR5C1 | 4.080E-07 | -1.488 | - | - |
| 19045 | ENSP00000448811 | OR6C68 | 3.882E-07 | -1.488 | - | - |
| 19046 | ENSP00000311038 | OR6M1 | 4.314E-07 | -1.488 | - | - |
| 19047 | ENSP00000373195 | OR5H15 | 3.404E-07 | -1.488 | - | - |
| 19048 | ENSP00000348368 | OR52R1 | 4.104E-07 | -1.489 | - | - |
| 19049 | ENSP00000259362 | OR13C9 | 5.082E-07 | -1.489 | - | - |
| 19050 | ENSP00000283225 | OR14K1 | 3.693E-07 | -1.489 | - | - |
| 19051 | ENSP00000348449 | OR8B4 | 4.104E-07 | -1.489 | - | - |
| 19052 | ENSP00000377799 | OR6C4 | 3.931E-07 | -1.489 | - | - |
| 19053 | ENSP00000311477 | OR10AG1 | 4.314E-07 | -1.489 | - | - |
| 19054 | ENSP00000350222 | OR51D1 | 4.104E-07 | -1.489 | - | - |
| 19055 | ENSP00000342836 | OR6C74 | 4.115E-07 | -1.489 | - | - |
| 19056 | ENSP00000335025 | OR5D18 | 4.134E-07 | -1.490 | - | - |
| 19057 | ENSP00000323606 | OR6C2 | 4.253E-07 | -1.490 | - | - |
| 19058 | ENSP00000329689 | OR10G7 | 9.272E-07 | -1.490 | - | - |
| 19059 | ENSP00000346611 | OR8B3 | 4.795E-07 | -1.490 | - | - |
| 19060 | ENSP00000279791 | OR5M9 | 4.366E-07 | -1.491 | - | - |
| 19061 | ENSP00000342008 | OR2T27 | 4.115E-07 | -1.491 | - | - |
| 19062 | ENSP00000349930 | OR11G2 | 4.187E-07 | -1.491 | - | - |
| 19063 | ENSP00000308689 | OR10AD1 | 4.318E-07 | -1.491 | - | - |
| 19064 | ENSP00000347428 | OR2L5 | 4.104E-07 | -1.491 | - | - |
| 19065 | ENSP00000404102 | OR2V1 | 4.636E-07 | -1.491 | - | - |
| 19066 | ENSP00000389072 | OR10G8 | 3.908E-07 | -1.491 | - | - |
| 19067 | ENSP00000302057 | OR5AU1 | 6.900E-07 | -1.491 | - | - |
| 19068 | ENSP00000306607 | OR1L8 | 4.323E-07 | -1.491 | - | - |
| 19069 | ENSP00000352305 | OR51A7 | 4.094E-07 | -1.491 | - | - |
| 19070 | ENSP00000318997 | OR11H4 | 4.320E-07 | -1.491 | - | - |
| 19071 | ENSP00000320077 | OR4D11 | 4.262E-07 | -1.492 | - | - |
| 19072 | ENSP00000324831 | OR4P4 | 7.232E-07 | -1.492 | - | - |
| 19073 | ENSP00000432417 | OR5M11 | 3.976E-07 | -1.492 | - | - |
| 19074 | ENSP00000346575 | OR5H1 | 4.105E-07 | -1.492 | - | - |
| 19075 | ENSP00000318956 | OR52K2 | 4.243E-07 | -1.492 | - | - |
| 19076 | ENSP00000302606 | OR9I1 | 4.343E-07 | -1.492 | - | - |
| 19077 | ENSP00000357126 | OR6K6 | 4.081E-07 | -1.492 | - | - |
| 19078 | ENSP00000345163 | OR51F1 | 5.244E-07 | -1.493 | - | - |
| 19079 | ENSP00000343062 | OR2T2 | 4.115E-07 | -1.493 | - | - |
| 19080 | ENSP00000331572 | OR56A3 | 4.392E-07 | -1.493 | - | - |
| 19081 | ENSP00000328878 | OR52E6 | 4.192E-07 | -1.494 | - | - |
| 19082 | ENSP00000334068 | OR13C8 | 4.882E-07 | -1.494 | - | - |
| 19083 | ENSP00000303862 | OR10A2 | 4.479E-07 | -1.494 | - | - |
| 19084 | ENSP00000322088 | OR52E2 | 8.989E-07 | -1.494 | - | - |
| 19085 | ENSP00000320560 | OR2D3 | 4.476E-07 | -1.494 | - | - |
| 19086 | ENSP00000351211 | OR6C6 | 4.509E-07 | -1.494 | - | - |
| 19087 | ENSP00000335535 | OR6N1 | 4.134E-07 | -1.494 | - | - |
| 19088 | ENSP00000364152 | OR8B2 | 4.094E-07 | -1.495 | - | - |
| 19089 | ENSP00000321426 | OR52E4 | 4.818E-07 | -1.495 | - | - |
| 19090 | ENSP00000289451 | OR10K1 | 7.281E-07 | -1.495 | - | - |
| 19091 | ENSP00000324958 | OR4C15 | 8.244E-07 | -1.496 | - | - |
| 19092 | ENSP00000353343 | OR52M1 | 4.305E-07 | -1.496 | - | - |
| 19093 | ENSP00000321246 | OR56A1 | 4.441E-07 | -1.496 | - | - |
| 19094 | ENSP00000353044 | OR2L3 | 4.213E-07 | -1.496 | - | - |
| 19095 | ENSP00000326718 | OR10A7 | 4.342E-07 | -1.496 | - | - |
| 19096 | ENSP00000306688 | OR2M4 | 7.867E-07 | -1.497 | - | - |
| 19097 | ENSP00000306651 | OR4C11 | 6.367E-07 | -1.497 | - | - |
| 19098 | ENSP00000420502 | OR2A7 | 4.349E-07 | -1.498 | - | - |
| 19099 | ENSP00000349945 | OR5B17 | 7.048E-07 | -1.498 | - | - |
| 19100 | ENSP00000373194 | OR5K3 | 4.021E-07 | -1.498 | - | - |
| 19101 | ENSP00000303469 | OR52A5 | 4.714E-07 | -1.498 | - | - |
| 19102 | ENSP00000355436 | OR2AK2 | 4.702E-07 | -1.499 | - | - |
| 19103 | ENSP00000368990 | OR6C1 | 5.209E-07 | -1.499 | - | - |
| 19104 | ENSP00000299454 | OR10A5 | 4.500E-07 | -1.499 | - | - |
| 19105 | ENSP00000325078 | OR2AJ1 | 4.042E-07 | -1.500 | - | - |
| 19106 | ENSP00000310788 | OR5J2 | 5.484E-07 | -1.500 | - | - |
| 19107 | ENSP00000305970 | OR4D5 | 4.354E-07 | -1.501 | - | - |
| 19108 | ENSP00000326301 | OR2G3 | 4.558E-07 | -1.501 | - | - |
| 19109 | ENSP00000311605 | OR4B1 | 4.592E-07 | -1.503 | - | - |
| 19110 | ENSP00000297913 | OR1Q1 | 5.642E-07 | -1.507 | - | - |
| 19111 | ENSP00000332185 | OR2V2 | 4.981E-07 | -1.507 | - | - |
| 19112 | ENSP00000308082 | OR10P1 | 1.015E-06 | -1.507 | - | - |
| 19113 | ENSP00000364164 | OR10G9 | 4.539E-07 | -1.508 | - | - |
| 19114 | ENSP00000305011 | OR4K14 | 8.354E-07 | -1.509 | - | - |
| 19115 | ENSP00000299459 | OR2D2 | 4.426E-07 | -1.509 | - | - |
| 19116 | ENSP00000303822 | OR7G2 | 1.613E-06 | -1.512 | - | - |
| 19117 | ENSP00000324687 | OR2T33 | 5.789E-07 | -1.513 | - | - |
| 19118 | ENSP00000332110 | OR4N5 | 4.841E-07 | -1.513 | - | - |
| 19119 | ENSP00000322754 | OR51S1 | 5.543E-07 | -1.515 | - | - |
| 19120 | ENSP00000325076 | OR10G4 | 4.709E-07 | -1.515 | - | - |
| 19121 | ENSP00000369157 | OR10A4 | 5.382E-07 | -1.517 | - | - |
| 19122 | ENSP00000324111 | OR5AS1 | 4.903E-07 | -1.517 | - | - |
| 19123 | ENSP00000334721 | OR6P1 | 4.248E-07 | -1.519 | - | - |
| 19124 | ENSP00000357127 | OR6K3 | 9.195E-07 | -1.521 | - | - |
| 19125 | ENSP00000323952 | OR51F2 | 7.360E-07 | -1.523 | - | - |
| 19126 | ENSP00000436424 | OR4D10 | 5.529E-07 | -1.523 | - | - |
| 19127 | ENSP00000354800 | OR5D13 | 7.049E-07 | -1.526 | - | - |
| 19128 | ENSP00000352626 | OR6K2 | 6.130E-07 | -1.527 | - | - |
| 19129 | ENSP00000277216 | OR13C4 | 8.987E-07 | -1.529 | - | - |
| 19130 | ENSP00000303076 | OR5B2 | 4.815E-07 | -1.530 | - | - |
| 19131 | ENSP00000369568 | OR51B6 | 5.976E-07 | -1.534 | - | - |
| 19132 | ENSP00000363913 | OR13C3 | 7.379E-07 | -1.535 | - | - |
| 19133 | ENSP00000305055 | OR2K2 | 8.752E-07 | -1.544 | - | - |
| 19134 | ENSP00000304807 | OR6Y1 | 6.883E-07 | -1.575 | - | - |
